# MAP2K1 is a potential therapeutic target in erlotinib resistant head and neck squamous cell carcinoma

**DOI:** 10.1038/s41598-019-55208-5

**Published:** 2019-12-11

**Authors:** Ankit P. Jain, Krishna Patel, Sneha Pinto, Aneesha Radhakrishnan, Vishalakshi Nanjappa, Manish Kumar, Remya Raja, Arun H. Patil, Anjali Kumari, Malini Manoharan, Coral Karunakaran, Saktivel Murugan, T. S. Keshava Prasad, Xiaofei Chang, Premendu Prakash Mathur, Prashant Kumar, Ravi Gupta, Rohit Gupta, Arati Khanna-Gupta, David Sidransky, Aditi Chatterjee, Harsha Gowda

**Affiliations:** 1 Institute of Bioinformatics, International Technology Park, Bangalore, 560066 India; 2 School of Biotechnology, Kalinga Institute of Industrial Technology, Odisha, 751024 India; 3 School of Biotechnology, Amrita Vishwa Vidyapeetham, Kollam, 690525 India; 4 Manipal Academy of Higher Education (MAHE), Manipal, 576104 Karnataka India; 5 Center for Systems Biology and Molecular Medicine, Yenepoya Research Centre, Yenepoya (Deemed to be University), Mangalore, 575018 India; 6 Medgenome Labs Pvt. Ltd., Bangalore, 560099 India; 7 Department of Otolaryngology—Head and Neck Surgery, Johns Hopkins University School of Medicine, Baltimore, 21231 MD USA; 8 Dept. of Biochemistry & Molecular Biology, School of Life Sciences, Pondicherry University, Pondicherry, 605014 India; 9 QIMR Berghofer Medical Research Institute, 300 Herston Road, Brisbane, QLD, 4006 Australia

**Keywords:** Cancer, Head and neck cancer

## Abstract

Epidermal growth factor receptor (EGFR) targeted therapies have shown limited efficacy in head and neck squamous cell carcinoma (HNSCC) patients despite its overexpression. Identifying molecular mechanisms associated with acquired resistance to EGFR-TKIs such as erlotinib remains an unmet need and a therapeutic challenge. In this study, we employed an integrated multi-omics approach to delineate mechanisms associated with acquired resistance to erlotinib by carrying out whole exome sequencing, quantitative proteomic and phosphoproteomic profiling. We observed amplification of several genes including *AXL* kinase and transcription factor *YAP1* resulting in protein overexpression. We also observed expression of constitutively active mutant MAP2K1 (p.K57E) in erlotinib resistant SCC-R cells. An integrated analysis of genomic, proteomic and phosphoproteomic data revealed alterations in MAPK pathway and its downstream targets in SCC-R cells. We demonstrate that erlotinib-resistant cells are sensitive to MAPK pathway inhibition. This study revealed multiple genetic, proteomic and phosphoproteomic alterations associated with erlotinib resistant SCC-R cells. Our data indicates that therapeutic targeting of MAPK pathway is an effective strategy for treating erlotinib-resistant HNSCC tumors.

## Introduction

Head and neck squamous cell carcinoma (HNSCC) is one of the leading cause of cancer-related deaths with a dismal 5-year survival rate^1^. Epidermal growth factor receptor (EGFR) is overexpressed in most epithelial malignancies including HNSCC^2^. Molecular target-based therapies against EGFR activity using small molecule EGFR-tyrosine kinase inhibitors (TKI) such as erlotinib or monoclonal antibodies against EGFR (Cetuximab) are under evaluation as potential therapeutic options. However, clinical trials with erlotinib have shown modest response rates of less than 15% in most cases and up to 25% in select cases of HNSCC^3,4^. Also, EGFR-TKIs have shown minimal improvement in overall or progression-free survival of patients. Median overall survival response to erlotinib in EGFR overexpressing HNSCC patients was reported to be 6 months^5^. This dismal performance of erlotinib in clinical trials is attributed to development of drug resistance. Though T790M gatekeeper mutations in EGFR and KRAS mutations define resistance in a majority of non-small cell lung carcinoma (NSCLC) and colorectal cancer patients respectively, these mutations contribute minimally to resistance in HNSCC patients^6^. Activation of related tyrosine kinases, “bypass” signaling mechanisms and mutations in downstream effectors are known as mediators of acquired resistance in HNSCC and other cancers^7,8^. However, full spectrum of molecular alterations associated with such resistance mechanisms remains unknown. Hence, exploring these mechanisms is essential to identify new therapeutic strategies that effectively target resistant tumors in HNSCC patients.

Genome sequencing can identify somatic mutations and copy number alterations associated with drug resistant tumors. However, a systematic understanding of the functional consequences of these genetic alterations and the resulting deregulation of cellular signaling networks is of biological importance. Delineating downstream oncogenic signaling pathways that are activated in resistant cells is essential for identifying therapeutic targets. Mass spectrometry-based proteomics and phosphoproteomics are valuable approaches to study signaling pathways^9^. An integrated genomic, proteomic and phosphoproteomic approach can provide a broad framework to identify molecular alterations that drive altered signaling networks in erlotinib resistant HNSCC and enable identification of alternate therapeutic targets.

In this study, we used an isogenic pair of erlotinib-sensitive and resistant HNSCC cell line UMSCC1^10^. We employed whole exome sequencing approach to identify genomic alterations in erlotinib resistant cell line. In addition, proteomic and phosphoproteomic approaches were employed to characterize activated signaling pathways in resistant cells. Through this integrated multi-omics approach, we identified several molecular alterations in MAPK pathway downstream to EGFR that might govern erlotinib resistance in HNSCC by bypassing EGFR-mediated signaling.

## Results

### Erlotinib-resistant cells show epithelial to mesenchymal transition phenotype

**Figure 1:**
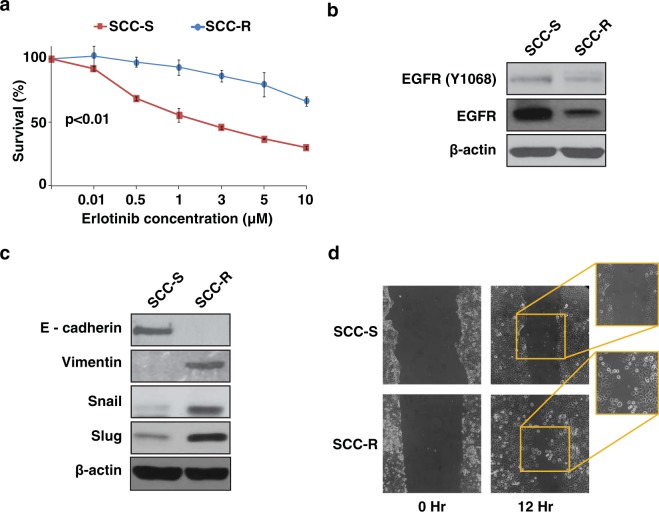
Erlotinib-resistant SCC-R cells show epithelial-to-mesenchymal transition phenotype: (**a**) Erlotinib-sensitive (SCC-S) and resistant (SCC-R) cells were treated with erlotinib and metabolic activity was evaluated using MTT assay to determine percentage of viable cells. Values were set at 100% for untreated controls. Western blot analysis of (**b**) EGFR and pEGFR (Y1068) (**c**) proteins associated with EMT in SCC-S and SCC-R cells. *β*-actin served as loading control. (**d**) Migration phenotype of SCC-S and SCC-R cells as seen at 0 hr and 12 hr post-scratch wounding.

Erlotinib-resistant (SCC-R) cells generated from HNSCC cell line UMSCC1 were used to investigate genetic and proteomic alterations associated with erlotinib resistance^10,11^. SCC-R cells showed an IC50 value ten times that of parental erlotinib-sensitive (SCC-S) (IC50 = 1 µM) cells. SCC-R cells showed decrease in expression as well as activation of EGFR (Tyr1068) (Fig. 1a,b). Previous studies have also reported association of Epithelial-mesenchymal transition (EMT) phenotype with erlotinib resistance^12–15^. We evaluated the expression of EMT associated markers including E-cadherin, vimentin, snail and slug in SCC-R cells. We observed loss of epithelial marker E-cadherin and elevated expression of mesenchymal markers - vimentin, snail and slug in SCC-R cells (Fig. 1c). SCC-R cells also showed scattered migration pattern associated with EMT phenotype as opposed to sheet migration pattern displayed by erlotinib sensitive cells (Supplementary Fig. S1A,B, Fig. 1d). In concordance with the literature our results indicate that erlotinib resistant cells demonstrate an EMT phenotype. We employed a multi-omics approach including genomics, proteomics and phosphoproteomics to understand the molecular underpinnings of erlotinib resistance in SCC-R cells.

### Whole exome sequencing analysis

**Figure 2:**
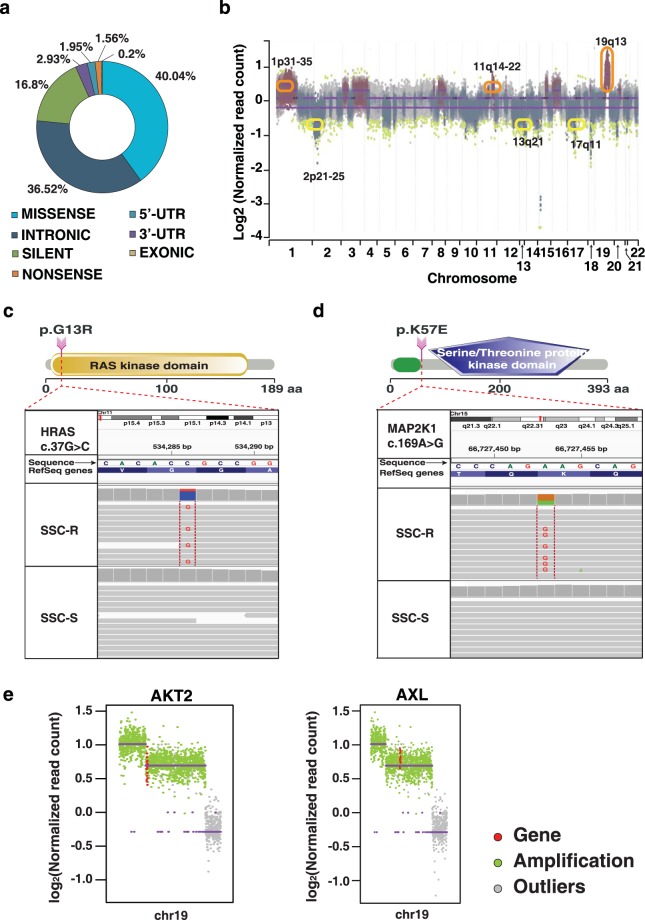
Genomic alterations observed in SCC-R cells: (**a**) Summary of SNVs observed in SCC-R cells. (**b**) CNAs identified using OncoCNV in SCC-R cells. Each dot corresponds to an amplicon. (Color code – green dots: outliers; grey dots: unchanged amplicons; plum color surroundings: 1-level gain; all purple dots in red circles represent copy number amplifications >1-level gain while yellow circles represent copy number loss in SCC-R cells). Single nucleotide variant in SCC-R cells resulting in (**c**) *p.G13R* in *HRAS* in SCC-R cells. (**d**) *p.K57E* in gene *MAP2K1*. (**e**) Copy number profile of *AKT2* and *AXL* in SCC-R cells. Each dot corresponds to an amplicon. (Color code – red dots: gene amplicon, green dots: other amplicons; grey dots: outliers).

Whole exome sequencing of SCC-S and SCC-R cell lines was performed using Illumina HiSeq. 2500 (2 × 100 bps). 115 million reads were generated for SCC-S and 116 million reads for SCC-R cells at a depth of ~100X. Target coverage of ~99% and ~99.5% alignment was achieved for SCC-S and SCC-R, respectively (Supplementary Table S1). Variant calling using Strelka led to identification of 512 SNVs in SCC-R cells compared to SCC-S cells. SNVs with at least 10X coverage and >10% allele frequency were considered for further analysis. A total of 418 SNVs corresponding to 378 genes qualified the criteria. Among the 418 variants, we observed 180 non-synonymous SNVs belonging to 174 protein coding genes including kinases such as *HRAS* and *MAP2K1* (Fig. 2a,c,d) Pan-cancer expression of *HRAS* and *MAP2K1* mutations from TCGA is represented in Supplementary Figs. S2 and S3.

In addition to SNVs in kinases associated with EGFR pathway we observed SNVs in transcription factor *ZEB2* (p.W97L) and cell adhesion molecule RGMA (p.V363I) that are predicted to be deleterious by SIFT, CONDEL and LRT algorithms. We also identified several SNVs that are present either in the close vicinity or directly modified at post-translational modification site and are predicted to be deleterious to protein function. For example, we identified SNV in *LCP2* gene (p.D31N) encoding SH2 domain-containing leukocyte protein. This SNV lies close to *K30*, an ubiquitination site that targets *LCP2* for proteasomal degradation. Similarly, we also identified a SNV in *CKS1B* gene (p.H56Q) adjacent to a known phosphorylation site *Y57*. A complete list of SNVs identified in SCC-R cells is available in Supplementary Table S2.

In SCC-R cells, CNAs affecting 1,369 genes were identified (Copy number loss ≤ 1 copy, copy number gain ≥ 3 copies, p-value ≤ 1 × 10^−6^) using OncoCNV kit (Fig. 2b). For instance, we observed 4-fold amplification of genes such as *AKT2* and *AXL* shown in Fig. 2e. In addition, large copy number changes (amplifications) were identified on chromosome1 (p31-p35 region) and chromosome 19 (q13) affecting 375 and 276 genes, respectively. Amplification of chromosome 11q22 region encompassing two gene clusters with nine matrix metalloproteinase (MMP) genes (MMP1, 3, 7, 8, 10, 12, 13, 20, and 27), and two baculoviral IAP repeat-containing protein (BIRC) genes (BIRC2 and BIRC3) was also observed in SCC-R cells. A complete list of CNAs identified in SCC-R cells is provided in Supplementary Table S3.

### Proteomic and phosphoproteomic alterations in erlotinib resistant cells

**Figure 3:**
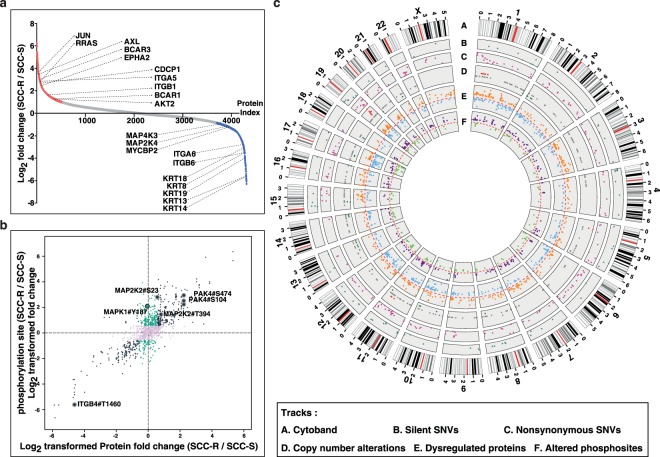
Proteomic and phosphoproteomic alterations in SCC-R cells: (**a**) Distribution of log2 transformed protein fold changes comparing the expression levels in SCC-R cells over SCC-S cells. (Red dots = overexpressed by ≥2 fold, Blue dots = downregulated by ≥2 fold) (**b**) Scatter plot of log2 transformed phosphosite ratios with total protein expression ratios (black dots depict dysregulation of total protein and phosphosite by ≥2 fold, cyan dots depict dysregulation of phosphosite by ≥2 fold at phosphopeptide level only) (**c**) Circos plot representing genomic and proteomic alterations in SCC-R cells compared to SCC-S cells. Chromosome ideograms are shown around the outer ring (*Track A*) and are oriented pter–qter in a clockwise direction with centromeres indicated in red. Other tracks contain alterations (from outside to inside): *Track B***-** silent SNVs (cyan dots), *Track C***-** non-synonymous SNVs (magenta dots), *Track D***-** copy number alterations (red dots = CNA gain, grey dots = CNA neutral, and dark green dots = CNA loss), *Track E*- dysregulated proteins (orange dots = overexpressed, light blue dots = downregulated, fold change ≥2), *Track F*- altered phosphosites (purple dots = hyperphosphorylated, green dots = hypophosphorylated, fold change ≥2).

SILAC-based quantitative proteomic analysis of SCC-R and SCC-S cells resulted in identification of 5,426 proteins of which 532 proteins were overexpressed and 521 were downregulated by ≥2 fold in SCC-R cells (Fig. 3a). We observed more than 2 fold overexpression of receptor tyrosine kinases such as AXL kinase and EPHA2 in SCC-R cells. In addition, we also observed overexpression of integral structural proteins such as integrin β1 (ITGB1) and integrin α5 (ITGA5) and their interactors such as proline-rich AKT1 substrate 1 (AKT1S1) in SCC-R cells. We observed downregulation of a number of proteins from the keratin family including KRT8 and KRT18 that are known epithelial markers. Epithelial differentiation-specific keratins K13, K14 were also found to be downregulated in SCC-R cells. A complete list of identified proteins is provided in Supplementary Table S4.

TiO_2_-based quantitative phosphoproteome strategy was employed to identify aberrant signalling events. Both SCC-S and SCC-R cells were serum starved for a period of 12 hours prior to TiO_2_-based phosphopeptide enrichment to study basal signalling events without interference from growth factors in serum. Enriched phosphopeptides were analyzed in duplicates (R^2^ = 0.89). Phosphoproteomics lead to identification of 7,633 phosphopeptides corresponding to 2,394 proteins. 246 phosphosites belonging to 188 proteins were hyperphosphorylated and 146 phosphosites belonging to 122 proteins were hypophosphorylated in SCC-R cells (≥2 fold) in both replicates (Fig. 3b, Supplementary Table S5).

### Integrative analysis of genomic, proteomic and phosphoproteomic datasets

**Figure 4:**
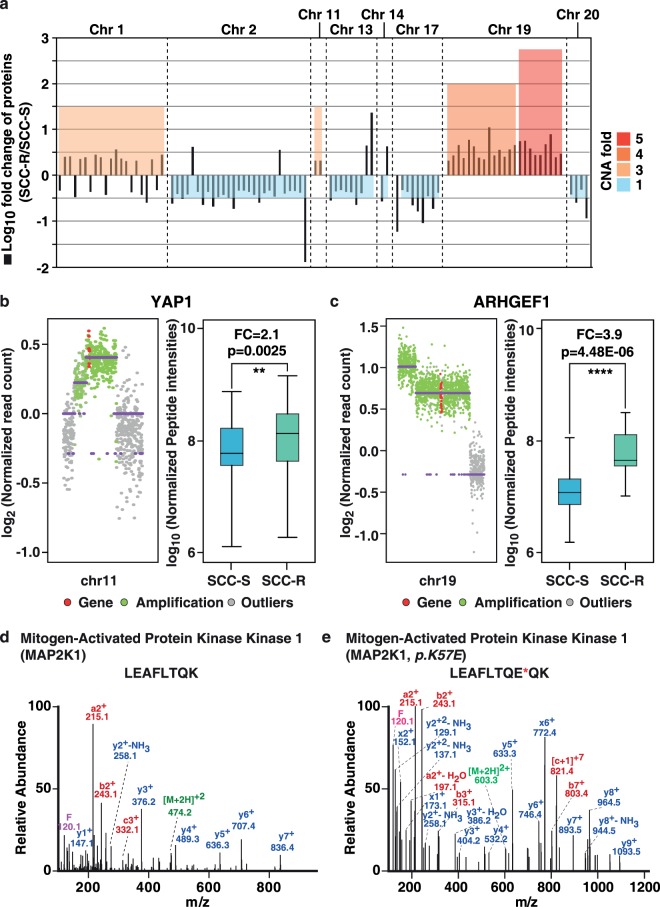
Genomic and proteomic alterations in SCC-R cells: (**a**) Copy number alterations (CNAs) of genes and their protein expression in SCC-R cells compared to SCC-S cells. Amplification and protein overexpression of (**b**) YAP1 and (**c**) AHRGEF1. MS/MS spectra of (**d**) wild-type peptide of MAP2K1 (**e**) mutant peptide representing *p.K57E* point mutation in *MAP2K1* gene.

By integrating genomic, proteomic and phosphoproteomic datasets, we observed overexpression of proteins encoded by genomic regions chr1 (p35–36) and chr19 (q13) that showed gene amplification in SCC-R cells (Fig. 3c and 4a). For instance, we observed amplification and corresponding overexpression of *YAP1* (chr11) and *AHRGEF1* (chr19) in SCC-R cells (Fig. 4b,c). We also observe hyperphosphorylation of some of the proteins from these regions. Similarly, in genomic regions with copy number loss such as chr2 (p25) and chr17 (q11), a decrease in the expression of proteins in these regions was observed in SCC-R cells. These results indicate that CNAs impact cellular protein expression levels and may alter cellular signaling mechanisms. We also identified proteins encoded by mutant alleles by searching unassigned spectra from proteomics dataset against customized database where amino acid variations were incorporated into human protein database. This second-pass search led to identification of variant peptide with *p.K57E* mutation in *MAP2K1* in SCC-R cells (Fig. 4d,e). MAP2K1 *p.K57* mutation is frequently observed in acquired resistance following EGFR blockade therapies^16–18^. Thus, targeting MAP2K1 in tumors has been proposed as a potential therapeutic strategy to overcome acquired resistance in colorectal cancer^19^.

### Targeting MAPK pathway in erlotinib resistant cells

**Figure 5:**
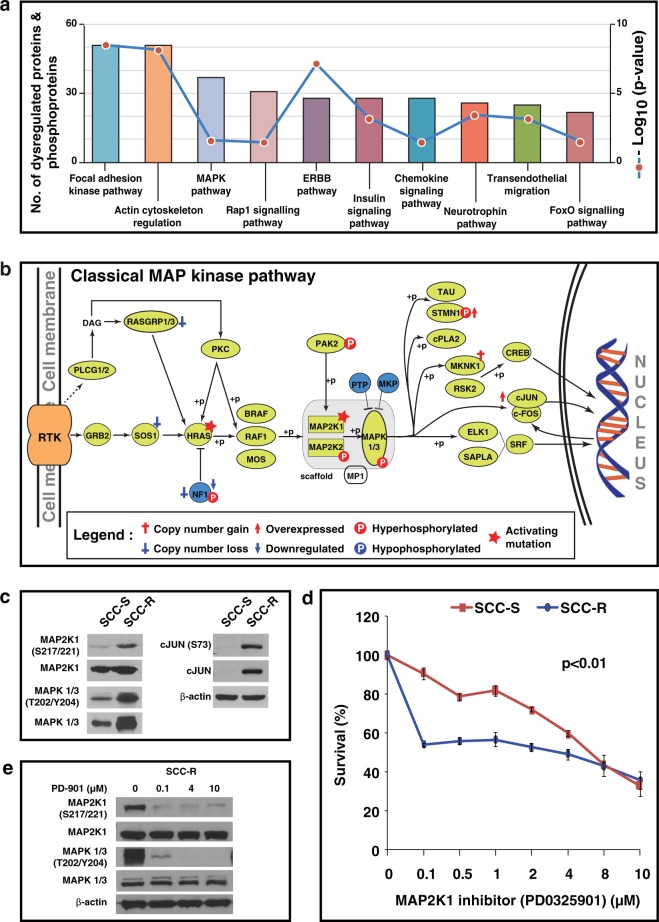
Activation of Map kinase pathway in erlotinib-resistant cells: (**a**) Significantly affected signalling pathways in SCC-R cells. (**b**) Schematic representation of canonical MAPK pathway showing genetic, proteomic and phosphoproteomic alterations observed in SCC-R cells. (**c**) Western blot validation of MAPK pathway intermediates (phospho-MAP2K1 (S217/S221), MAP2K1, phospho-MAPK1/3 (T202/204) and MAPK1/3, c-JUN and phospho-c-JUN (S73). β-actin served as loading control (**d**) Survival curve for erlotinib-sensitive (SCC-S) and resistant (SCC-R) cells treated with various concentration of MAP2K1 inhibitor (PD0325901). Metabolic activity was evaluated using MTT assay to determine percentage of viable cells. Values were set at 100% for untreated controls. (**e**) Western blot showing effect of different concentration of PD-0325901 on MAPK pathway intermediates (phospho-MAP2K1 (S217/S221), MAP2K1, phospho-MAPK1/3 (T202/204) and MAPK1/3 in SCC-R cells. β-actin served as loading control.

Pathway enrichment analysis of dysregulated proteins (fold change ≥ 2) and aberrantly phosphorylated proteins (fold change ≥ 2) was performed using DAVID and KEGG-pathway database. Figure 5a shows top 10 signalling pathways and processes enriched in SCC-R cells. We observed enrichment of focal adhesion kinase pathway and actin cytoskeleton regulation pathway in SCC-R cells. Overexpression of proteins including vimentin (VIM) and zyxin (ZYX) that are associated with cytoskeleton reorganisation and cell motility corroborates these observations.

Several mechanisms of intrinsic and acquired resistance to EGFR-TKIs are known to converge on Ras/Raf/Mek/Erk pathway^20^. We observed molecular alterations in several receptor tyrosine kinase pathway intermediates (Fig. 5b). We observed overexpression and activation of MAP2K1 and its downstream targets MAPK1/3 and cJun in SCC-R cells (Fig. 5c). As MAP2K1 activation is frequently observed in acquired resistance to EGFR inhibitors, we examined the effects of MAPK pathway inhibition on SCC-R cells using a second generation highly selective MAP2K1 inhibitor, PD-0325901. PD-0325901 was effective in inhibiting the growth of erlotinib resistant SCC-R cells at a concentration 40 times lower than IC50 of SCC-S cells (IC50 = 4 µM) (Fig. 5d). Treatment with PD-0325901 resulted in decrease in phosphorylation of MAP2K1 and its downstream target MAPK1/3 (Fig. 5e).

Further, data from TCGA showed that a subset of HNSCC patients show alterations in genes related to MAPK pathway (Supplementary Fig. S4). MAPK pathway inhibition can be an effective strategy as a second line treatment following acquired resistance to EGFR inhibitor therapy. MAP2K1 K57 mutation can be used as a marker to select patients who are likely to benefit from this therapy. A combination therapy using EGFR and MAPK inhibitors might be efficacious and may delay the onset of resistance.

## Discussion

Targeted therapies have emerged as promising cancer treatment modality due to remarkable efficacy in treating “oncogene-addicted” cancers. However, EGFR-targeted therapies have shown suboptimal performance in clinical trials for EGFR overexpressing HNSCC tumours. Acquired resistance remains a significant contributor to poor therapeutic response^3–5^. In the present study, we used an isogenic pair of erlotinib-sensitive (SCC-S) and resistant (SCC-R) HNSCC cell line to characterize molecular alterations associated with drug resistance. Erlotinib resistant SCC-R cells showed decrease in expression and activation of EGFR. Acquisition of erlotinib resistance was associated with an EMT phenotype with altered cell migration pattern, loss of E-cadherin and gain of mesenchymal markers such as snail, slug and vimentin. Overexpression of EMT markers such as snail is known to drive erlotinib resistance in human oral epithelial cells^21^. Although different mechanisms contributing to erlotinib-resistance are identified, a comprehensive genomic, proteomic and phosphoproteomic profile of resistant cells has not yet been investigated.

Whole exome sequencing revealed genetic alterations in SCC-R cells. In contrast to non-small cell lung cancer, EGFR tyrosine kinase domain mutations were not observed in SCC-R cells^22,23^. However, we identified SNVs in multiple genes downstream of EGFR. A non-synonymous mutation *p.G13R* was identified in *HRAS*, a ras-kinase downstream of EGFR in SCC-R cells. This substitution in the GTPase domain of *HRAS* that results in increased kinase activity is a known oncogenic driver mutation in different cancer types^24^. Constitutively active *HRAS*-mutants (*p.G12D* and *p.G12V*) are known to confer resistance to erlotinib and other *EGFR*-targeted therapies in HNSCC cell lines^25,26^. Pan-cancer data from TCGA and cBioPortal show that 5–15% of HNSCC patients have mutations in *HRAS*^27,28^. Activating mutations at *p.G12* and *p.G13* in *HRAS* are observed with relatively high frequency in HNSCC. A novel substitution *in MAP2K1* (*p.K57E)*, which alters a ubiquitination site was found in SCC-R cells. MAP2K1 is downstream of EGFR and HRAS. Although *MAP2K1* mutations have been observed at a lower frequency, they have been identified in several cancers including lung adenocarcinoma, melanoma and gastric cancer^29–31^. Similar to lung cancer, *MAP2K1* mutations are observed in about 1% of HNSCC cases. In lung adenocarcinoma, mutations in amino acid residues 53–67 in exon 2 account for about 86% of all *MAP2K1* mutations^29^. Pan-cancer data from cBioPortal showed high frequency of substitution at *p.K57*. Although functional consequences of loss of this ubiquitination site are not well characterized, this (*p.K57E)* mutation in the coil-coil hinge region of MAP2K1 is predicted to be deleterious by SIFT, CONDEL and LRT. Pre-clinical data has shown that substitution of *p.K57* leads to constitutive activation of MAP2K1 and downstream RAS/MAPK pathway^32^. *p.K57N* mutation in *MAP2K1* is also reported to be associated with gefitinib resistance in lung adenocarcinoma^33^. This data indicates activating mutations in *HRAS (p.G13R)* and *MAP2K1* (*p.K57E*) could drive resistance to EGFR-TKIs in subsets of HNSCC patients.

In addition to SNVs in kinases associated with EGFR-MAPK signaling axis, we identified SNVs in several other proteins such as transcription factor *ZEB2 p.W97L* and cell adhesion molecule *RGMA p.V363I* that are predicted to be deleterious to protein function. ZEB2 is known to regulate epithelial to mesenchymal transition (EMT) and drive proliferation and differentiation^34^. Downregulation of RGMA is reported in colorectal and breast cancer and has been associated with cancer progression and poor prognosis^35,36^. We also identified SNVs that were in close proximity to post-translational modification sites. For example, *p.D31N* variant in LCP2 gene encoding SH2 domain-containing leukocyte protein lies close to p.K30, an ubiquitination site that targets it for proteasomal degradation. Loss of this site is reported to enhance activation of MAPK1 and JNK^37^. Similarly, p.H56Q substitution in CKS1B gene is close to Y57 phosphorylation site. CKS1B is known to drive cell proliferation through MAPK pathway^38,39^. Although these genes are known to play essential roles in oncogenesis, functional consequences of these variants and their role in driving resistance to erlotinib in HNSCC is unexplored.

In addition to SNVs, we also observed a large number of CNAs affecting several genes on multiple chromosomes in SCC-R cells. We observed copy number gain in Chr1p35-36 and Chr19q13, which are reported in other cancers^40,41^. We observed copy number gain in *MKNK1*, a downstream target of MAPK1. Pharmacologically induced degradation of MKNK1 is known to impair cell migration and promote cell death in breast cancer cells^42^. We also observed copy number gain in *AKT2* and *AXL*. AKT2 overexpression is known to confer resistance to EGFR targeted therapies in NSCLC and pancreatic cancer cell lines^43–45^. Similarly, overexpression of AXL is known to drive survival and proliferative signals and is reported to be associated with erlotinib resistance in head and neck cancer^46^. We observed focal amplification and 2 fold protein overexpression of YAP1 which is a reported oncogenic target of 11q22 amplification in multiple cancer subtypes including HNSCC^47,48^ and shown to promote resistance to erlotinib in NSCLC^49^. In addition, we observed copy number gain and a corresponding 3.9 fold overexpression of ARHGEF1, which is known to regulate epithelial cell plasticity and contribute to EMT in breast cancer cell lines^50^. We observed protein downregulation of NF1 and copy number loss in SCC-R cells. Loss of NF1 has been associated with hyperactivation of RAS kinase and MAP2K1 dependence in melanoma^51^. Reduced expression of NF1 is also associated with erlotinib resistance in NSCLC^52^.

Pathway analysis of dysregulated proteins and phosphoproteins revealed that cellular processes such as cytoskeleton reorganization and ECM-receptor interaction were altered in SCC-R cells. There is a known association between genetic alteration such as activating HRAS mutation or overexpression of kinases such as AXL and expression of mesenchymal markers such as vimentin^12,53^. Increased expression of integrin α5 and β1 during EMT is shown to be involved in increased cell-matrix interaction and promoting migration. Along with overexpression of integrin α5 and β1, we also observed an increase in zyxin (ZYX) that is known to regulate motility in lung cancer via integrin α5β1 pathway^54,55^. Epithelial differentiation-specific keratins K13 and K14 were downregulated in SCC-R cells. siRNA mediated knockdown of vimentin is known to restore expression of these keratins^56^. Focal adhesion kinase pathway is also known to mediate erlotinib resistance in lung cancer^57,58^. Overall, we observed altered expression of proteins related with focal adhesion kinase and cytoskeleton reorganisation along with changes in migration phenotype and epithelial-to-mesenchymal transition of erlotinib-resistant SCC-R cells. These results demonstrate genetic and proteomic alterations rewire signalling networks that drive both cellular transformation processes like EMT and acquired drug resistance.

Apart from enrichment of focal adhesion and cellular cytoskeleton reorganization we saw enrichment of MAPK pathway downstream of EGFR in SCC-R cells. We observed hyperphosphorylation of kinases in classical MAPK pathway including dual specificity mitogen-activated protein kinase kinase 2 (MAP2K2) at Ser23/Thr394, MAPK1 at Tyr187 and proteins such as NF1 at Ser1140. Phosphorylation at Thr394 is known to increase activity of MAP2K2 and subsequent activation of downstream MAPK1^59^. We also observed hyperphosphorylation of PAK family of kinases such as p21 activated protein kinase 2 (PAK2) (Ser141/Ser197) and (PAK4) (Ser104/Ser474). Hyper-activation of PAK is associated with advanced tumor grade and poor survival in multiple cancers^60^. PAK2 is also shown to activate MAP2K1 independent of RAF activity and drive proliferative signals through MAPK1 pathway^61^. Concomitantly, hyperphosphorylation of downstream proteins such as stathmin isoform a (STMN1) at Ser25/Ser38 was also observed in erlotinib-resistant SCC-R cells. Phosphorylation of STMN1 at Ser25 and Ser38 results in activation of these proteins and cell cycle regulation and cytoskeleton reorganization^62^. Although hyperphosphorylation of MAP2K2 at Ser23 and NF1 at Ser1140 was also observed, the functional consequences of these sites are not known. Presence of known activating mutations in key kinases as well as elevated protein expression and phosphorylation levels demonstrates activation of MAPK pathway in erlotinib resistant cells.

Several mechanisms of acquired and adaptive resistance are known to converge on MAPK pathway in NSCLC^20^. We observed that erlotinib-resistant SCC-R cells were highly sensitive to MAP2K1/2 inhibitor (PD-0325901) compared to SCC-S cells. High efficacy of PD-0325901 towards SCC-R cells could be due to increased binding affinity of the drug to mutant MAP2K1. It has been reported that PD-0325901 has as increased potency towards activated MAP2K1^63^. Treatment with PD-0325901 resulted in decreased levels of pMAP2K1 and pMAPK1/3 in SCC-R cells indicating inhibition of MAPK pathway in these cells. Together, our results indicate that in erlotinib resistant SCC-R cells, activation of Ras/Raf/Mek/Erk signaling axis is an important survival mechanism. Genes related to MAPK pathway are altered at a frequency of 4–15% across HNSCC cohorts while mRNA overexpression of MAPK pathway genes is seen in around 30% of HNSCC patients. These results from TCGA data indicate that MAPK pathway might be a potential driver in a subset of HNSCC patients and targeting MAPK pathway might be a useful strategy in a subset of HNSCC patients.

In summary, our study demonstrates a framework to understand molecular alterations associated with erlotinib resistance in HNSCC. We demonstrate that multiple alterations are associated with erlotinib resistance in HNSCC. Activation of Ras/Raf/Mek/Erk pathway downstream of EGFR may drive survival and proliferation in erlotinib resistant HNSCC. Our findings support further investigation of MAP2K1 inhibitors in pre-clinical and clinical studies as a therapeutic strategy to treat HNSCC.

## Materials and Methods

### Cell culture and SILAC labelling

Head and Neck Squamous Cell Carcinoma (HNSCC) cell line UMSCC1 was used as a model to study erlotinib resistance^10^. UMSCC1 cell line sensitive to erlotinib (SCC-S) and an isogenic cell line resistant to erlotinib (SCC-R) were used in the study. Both SCC-S and SCC-R cell lines were maintained in DMEM-high glucose medium containing 10% foetal bovine serum (GIBCO^TM^, ThermoFisher Scientific) and 1% penicillin/streptomycin at 37 °C in a humidified 5% CO_2_ atmosphere. For Stable isotope labeling with amino acids in cell culture (SILAC) based proteomics experiments, SCC-S cells were adapted to DMEM-SILAC media supplemented with L-lysine-2HCL (^13^C_6_, ^15^N_2_, 98% isotopic purity) and L–arginine-HCL (^13^C_6_, 98% isotopic purity). SCC-R cells were maintained in regular media^64^.

### Cell viability and drug sensitivity assay

Cells were plated at a density of 3000/well in 96-well plates. The following day, cells were treated with indicated concentrations of erlotinib or MAP2K1/3 inhibitor (PD-0325901) for 72hrs. MTT (3-(4,5-dimethylthiazol-2yl)-2,5-diphenyl tetrazolium bromide) assay was performed as described before^65–67^. Absorbance was measured at 570 nm and 650 nm. All experiments were carried out in triplicates.

### Western blotting

Whole cell extracts of SCC-S and SCC-R cells were prepared using modified Radioimmunoprecipitation assay lysis buffer (Merck Millipore, Billerica, MA, USA) containing protease inhibitors (Roche, Indianapolis, IN, USA) and phosphatase inhibitors (Thermo Scientific, Bremen, Germany). Western blot analysis was performed as previously described using 30 µg protein lysates^68^. Nitrocellulose membranes were hybridized with primary antibodies and developed using Luminol reagent (Santa Cruz Biotechnology, Dallas, TX,) as per the manufacturer’s instructions.

Anti-E-cadherin, anti-Vimentin, anti-Snail, anti-Slug, anti-EGFR, anti-phospho EGFR (Y1068), anti-MAP2K1, anti-phospho-MAP2K1 (S217/221), anti-MAPK1/3, anti-phospho-MAPK1/3 (T202,T204), anti-cJun, and anti-phospho-c-JUN (S73) antibodies were all obtained from Cell Signaling Technology (Cell Signaling Technology, Beverly, MA). The beta-actin antibody was obtained from Sigma (St. Louis, MO).

### Cell migration assays

Cell migration assays were performed as described previously^69^. Briefly, 1.5 × 10^6^ cells were seeded and allowed to grow until they formed a monolayer. A uniform sized wound was introduced with a 200 µl tip. The wound photomicrographs were taken at 0, 8 and 12 hours under the microscope at 20X magnification. All experiments were performed in triplicate. Wound assay data was quantified using the ImageJ software *v*1.52a^70^.

### Bioinformatic analysis

Whole exome sequencing, SILAC-based quantitative proteomics, phosphoproteomics and data analysis were performed as described in Supplementary methods. Enrichment analysis of subcellular localization of differentially expressed proteins and biological processes they are involved in was obtained from Human Protein Reference Database (HPRD; http://www.hprd.org)^71^. Pathway analysis was performed using DAVID version 6.8 using KEGG pathway database as background^72–74^. Volcano plot was generated using R and circos diagram was plotted using BioCircos^75^. Stastical analysis were done using GraphPad Prism ^TM^ software version 6.1. Non-parametric paired two-tailed Student’s t-test was used for p-value calculation.

## Supplementary information

Supplementary information

## Data Availability

Mass spectrometry data has been deposited to ProteomeXchange Consortium via the PRIDE^76^ partner repository with the dataset identifiers PXD007785 and PXD007768. Sequence data has been submitted at the Sequence Read Archive (SRA) which is hosted by the National Centre for Biotechnology Information (NCBI) under the accession number SRP117857.
