# Supplementary material for: MAP2K1 is a potential therapeutic target in erlotinib resistant head and neck squamous cell carcinoma
Source: Sci Rep. 2019 Dec 11;9:18793. doi: 10.1038/s41598-019-55208-5 (PMC6906491; doi:10.1038/s41598-019-55208-5)
Supplement: Supplementary file 1 — Supplementary information [file 41598_2019_55208_MOESM1_ESM.pdf]

# MAP2K1 is a potential therapeutic target in erlotinib resistant head and neck squamous cell carcinoma

Ankit P. Jain[1,2#], Krishna Patel[1,3#], Sneha Pinto[1], Aneesha Radhakrishnan[1], Vishalakshi Nanjappa[1], Manish Kumar[1], Remya Raja[1,4], Arun H. Patil[1,2,5], Anjali Kumari[6], Malini Manoharan[6], Coral Karunakaran[6], Saktivel Murugan[6], T. S. Keshava Prasad[1,5], Xiaofei Chang[7], Premendu P. Mathur[2,8], Prashant Kumar[1], Ravi Gupta[6], Rohit Gupta[6], Arati Khanna-Gupta[6], David Sidransky[7], Aditi Chatterjee[1,4*]and Harsha Gowda[1,2,4,9*]

*[1] Institute of Bioinformatics, International Technology Park, Bangalore, 560066 India*
*[2] School of Biotechnology, Kalinga Institute of Industrial Technology, Odisha-751024, India*
*[3] School of Biotechnology, Amrita Vishwa Vidyapeetham, Kollam-690 525, India*
*[4] Manipal Academy of Higher Education (MAHE), Manipal 576104, Karnataka, India*
*[5] Center for Systems Biology and Molecular Medicine, Yenepoya, Mangalore 575018, India*
*[6] Medgenome Labs Pvt. Ltd., Bangalore 560099, India*
*[7] Department of Otolaryngology—Head and Neck Surgery; Johns Hopkins University School of Medicine; Baltimore 21231, MD, USA*
*[8] Dept. of Biochemistry & Molecular Biology, School of Life Sciences, Pondicherry University, Pondicherry 605014, India*
*[9] QIMR Berghofer Medical Research Institute, 300 Herston Road, Brisbane, QLD, 4006, Australia*

**[*]Correspondence details**

Aditi Chatterjee, Ph.D.
Institute of Bioinformatics,
International Tech Park,
Bangalore-560 066, India
E-mail: aditi@ibioinformatics.org

Harsha Gowda, Ph.D.
Institute of Bioinformatics,
International Tech Park,
Bangalore-560 066, India
E-mail: harsha@ibioinformatics.org

[#]Authors have contributed equally

**Supplementary Methods**

## 1. Whole exome sequence analysis

For whole exome sequencing (WXS), cells were grown to 75% confluence and washed thrice with ice-cold phosphate-buffered saline (PBS) and harvested in PBS. Genomic DNA was extracted and exome capture and library preparation was done using Agilent SureSelectXT Human All Exon V5 kit as per manufacturer's instruction. Paired end sequencing was carried out on Illumina HiSeq 2500 platform with read length of 100 bps. Raw files obtained in Fastq format were assessed for Phred quality score using fastqc tool [https://www.bioinformatics.babraham.ac.uk/projects/fastqc/]. Bases comprising exogenous sequences such as adaptors or with Phred score ≤ 20 were trimmed using fastq-mcf program. Clean reads were further aligned against human reference genome hg19 (GRCh37) using Burrows-Wheeler Aligner (BWA, version 0.7.12) [1]. Binary alignment maps (BAM) files were pre-processed using MarkDuplicates of Picard tools, IndelRealigner and BaseRecalibrator of GATK tool suite (the Genome Analysis Toolkit, Broad Institute). Pre-processed BAM files were further subjected to somatic single nucleotide variant (SNVs) identification using Strelka [2] with default parameters and annotated using *in-house* resources VariMAT and OncoMD databases. Functional consequences of variants were annotated using SIFT [3], Polyphen-2 [4] and LRT [5]. Copy number alterations (CNAs) were identified using OncoCNV toolkit [6]. BAM files were processed with default parameter setting to correct for library size, GC content and amplicon length. Erlotinib sensitive SCC-S cells were used as a baseline against which SCC-R was normalized. Copy numbers ≤ 1.5 were considered copy number loss and copy numbers ≥ 3 were considered copy number gain with p-value threshold of 0.0000001. Results were visualized using Integrative Genomics Viewer (IGV) [7].

## 2. Peptide fractionation and phosphopeptide enrichment

SCC-S and SCC-R cell lines were grown in DMEM-SILAC (K8R6) and DMEM media, respectively and were maintained in serum-free media for 12 hours prior to harvesting. Cells were lysed in urea lysis buffer (20mM 4-(2-hydroxyethyl)-1-piperazineethanesulfonic acid (HEPES) buffer pH 8.0, 9M urea, 1mM sodium orthovanadate, 2.5mM sodium pyrophosphate, 1mM phosphoglycerophosphate). Protein concentration was determined using Bicinchoninic acid (BCA assay and 2mg equivalent lysate from SCC-S and SCC-R cells were pooled. Pooled

lysate was reduced with 10mM dithiothreitol at 60$^{o}$C for 30mins and alkylated using 20mM iodoacetamide for 15mins at room temperature in the dark. Concentration of urea was brought to 2M by diluting with HEPES buffer and digested overnight at 37$^{o}$C using sequencing grade trypsin (Promega) at 1:50 enzyme-to-substrate ratio. Protein digests were desalted using Sep-Pak $C_{18}$ column (with 40% acetonitrile (ACN) with 0.1% Tri-fluoro acetic acid (TFA) as the final elution buffer), lyophilized and reconstituted in 7mM Triethylammonium bicarbonate (TEABC) buffer (pH 9) method. Peptides were fractionated using basic pH reverse phase chromatography (bRPLC) on XBridge BEH $C_{18}$ Column (Waters, UK). Fractionation was carried out using a linear gradient from 5% to 40% solvent B using the following solvent system (solvent A (7mM TEABC, pH 9) and solvent B (90% ACN, pH 9). Fractions were concatenated and pooled into 24 fractions. One-tenth of the sample was vacuum-dried prior to LC-MS/MS and used for total proteome analysis. Remaining sample was further pooled and reduced to 12 fractions. These 12 fractions were lyophilized and reconstituted in 80% ACN, 1% TFA, and 3% 2, 5-dihydroxybenzoic acid (DHB) for $TiO_2$-based phosphopeptide enrichment. Briefly, TiO2 beads were activated with DHB solution (80% ACN, 1% TFA, and 3% 2, 5-dihydroxybenzoic acid (DHB)) for 4 hours at room temperature. Each fraction resuspended in DHB solution was incubated with pre-activated TiO2 beads (1mg). Phosphopeptide-bound TiO2 beads were washed three times with DHB solution and twice with 40% ACN. Peptides were eluted three times with 40µl of 2% ammonia into 10µl of 20% TFA. The peptides were lyophilized and reconstituted in 0.1% formic acid prior to LC-MS/MS analysis.

**3. Mass spectrometry analysis**

The enriched phosphopeptides as well as 24 fractions from total protein lysate were analysed on Orbitrap Fusion Tribrid mass spectrometer (Thermo Electron, Bremen, Germany) interfaced with Easy-nLC II nanoflow liquid chromatography system (Thermo Scientific, Odense, Denmark). Peptide samples were loaded onto trap columns packed (75µm × 2cm) with Magic C18 AQ 5µm particle (Michrom Bioresources, Inc., Auburn, CA) at a flow rate of 4µL/min. Peptides were separated on an analytical column (75µm × 12cm) at a flow rate of 300nl/min using a step gradient of 5–30 % solvent B (0.1% formic acid in 95% ACN) for first 75min and 30–40% solvent B from 75-90 min. The total run time was set to 120min and the mass spectrometer was operated in data-dependent acquisition mode. Precursor MS scan (m/z 350–1800) and MS/MS

(m/z 350–1800) was acquired with a mass resolution of 120K and 30K at 200 m/z, respectively. Automatic gain control (AGC) target was set to 500K for MS and 50K for MS/MS and maximum ion injection time was set to 60ms for MS and 200ms for MS/MS scan events, respectively. In each duty cycle, 10 most intense peaks were selected for MS/MS fragmentation using higher-energy collision dissociation (HCD) mode at 32% normalized collision energy and isolation width was set to 2.0 m/z. Dynamic exclusion was set for 45 seconds with a 10 ppm mass window. The lock-mass option was enabled using polysiloxane ion (m/z= 445.12002) from ambient air for internal calibration.

**4. Proteomic and phosphoproteomic data analysis**

The tandem mass spectrometry data were searched using MASCOT (v 2.2) and Sequest-HT search algorithms against a Human RefSeq database (RefSeq 65) supplemented with frequently observed contaminants through the Proteome Discoverer platform Version 1.4.1.14 (Thermo Fisher Scientific, Bremen, Germany). For both algorithms, search parameters included a maximum of 2 missed cleavages; carbamidomethylation at cysteine as a fixed modification, oxidation of methionine, and SILAC labels $^{13}C_{6,}$ $^{15}N_2$-lysine (K8); $^{13}C_6$-arginine as variable modifications. The MS mass error tolerance was set at 10ppm and MS/MS error tolerance at 0.05Da. The data was also searched against a decoy database and PSMs above 1% false discovery rate (FDR) score threshold were considered for further analysis. Precursor ion quantifier node with a mass precision of 3ppm was used for peptide and protein quantification. Phosphorylation at serine, threonine and tyrosine was added as dynamic modification in both SequestHT and MASCOT node for analysis of phosphoproteome data. The probability of phosphorylation was calculated by the PhosphoRS node (Version 3.0) in Proteome Discoverer. Peptides with ≥75% phosphosite probability were considered for downstream analysis. High-confidence PTMs were profiled using post-translational modification – profiling (PTM-Pro) tool [PRIDE: 30106353] with a PhosphoRS site probability greater than 75% [8].

Unassigned spectra from the first pass search with Human RefSeq 65 database along with contaminants were further searched against a database of tryptic single amino acid variant (SAAV) peptides comprising of somatic non-synonymous SNVs identified using WXS. Search and quantitation parameters were same as stated earlier except phosphorylation was not used as a dynamic modification.

## References


1. Li, H. and R. Durbin, *Fast and accurate short read alignment with Burrows-Wheeler transform.* Bioinformatics, 2009. **25**(14): p. 1754-60.
2. Saunders, C.T., et al., *Strelka: accurate somatic small-variant calling from sequenced tumor-normal sample pairs.* Bioinformatics, 2012. **28**(14): p. 1811-7.
3. Kumar, P., S. Henikoff, and P.C. Ng, *Predicting the effects of coding non-synonymous variants on protein function using the SIFT algorithm.* Nat Protoc, 2009. **4**(7): p. 1073-81.
4. Adzhubei, I.A., et al., *A method and server for predicting damaging missense mutations.* Nat Methods, 2010. **7**(4): p. 248-9.
5. Chun, S. and J.C. Fay, *Identification of deleterious mutations within three human genomes.* Genome Res, 2009. **19**(9): p. 1553-61.
6. Boeva, V., et al., *Multi-factor data normalization enables the detection of copy number aberrations in amplicon sequencing data.* Bioinformatics, 2014. **30**(24): p. 3443-50.
7. Robinson, J.T., et al., *Integrative genomics viewer.* Nat Biotechnol, 2011. **29**(1): p. 24-6.
8. Patil, A.H., et al., *Dissecting Candida Pathobiology: Post-Translational Modifications on the Candida tropicalis Proteome.* OMICS, 2018. **22**(8): p. 544-552.

## Supplementary fig. S1

**a**

0 Hr

8 Hr

12 Hr

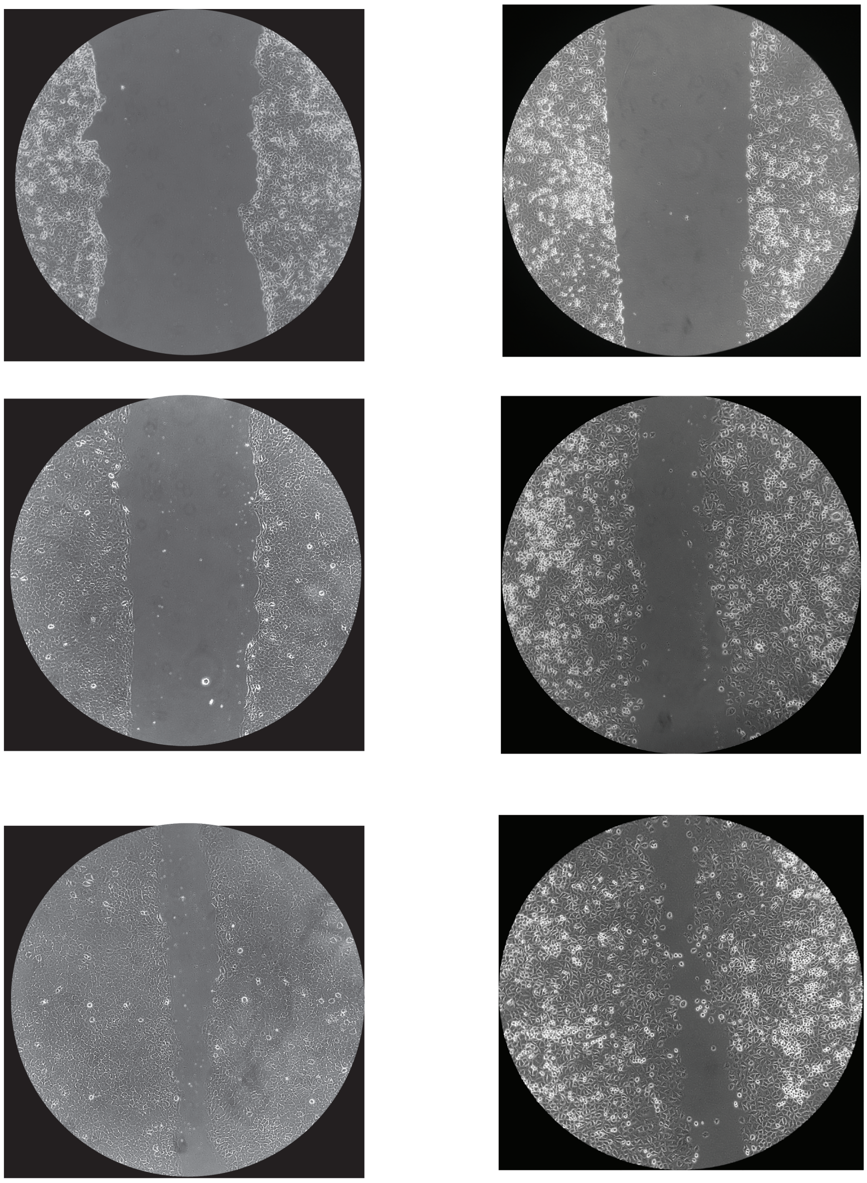

**b**

SCC-S SCC-R

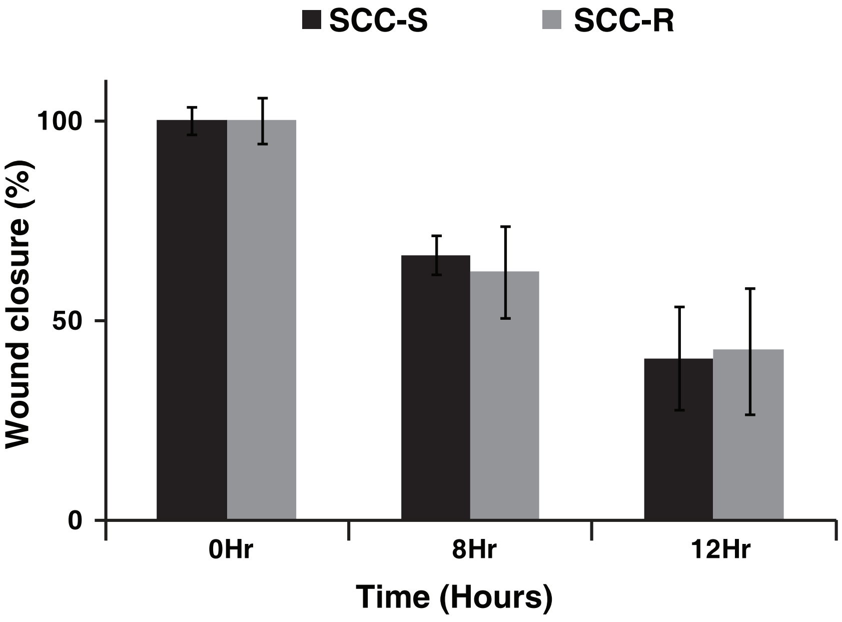

# Supplementary fig. S2

**a**

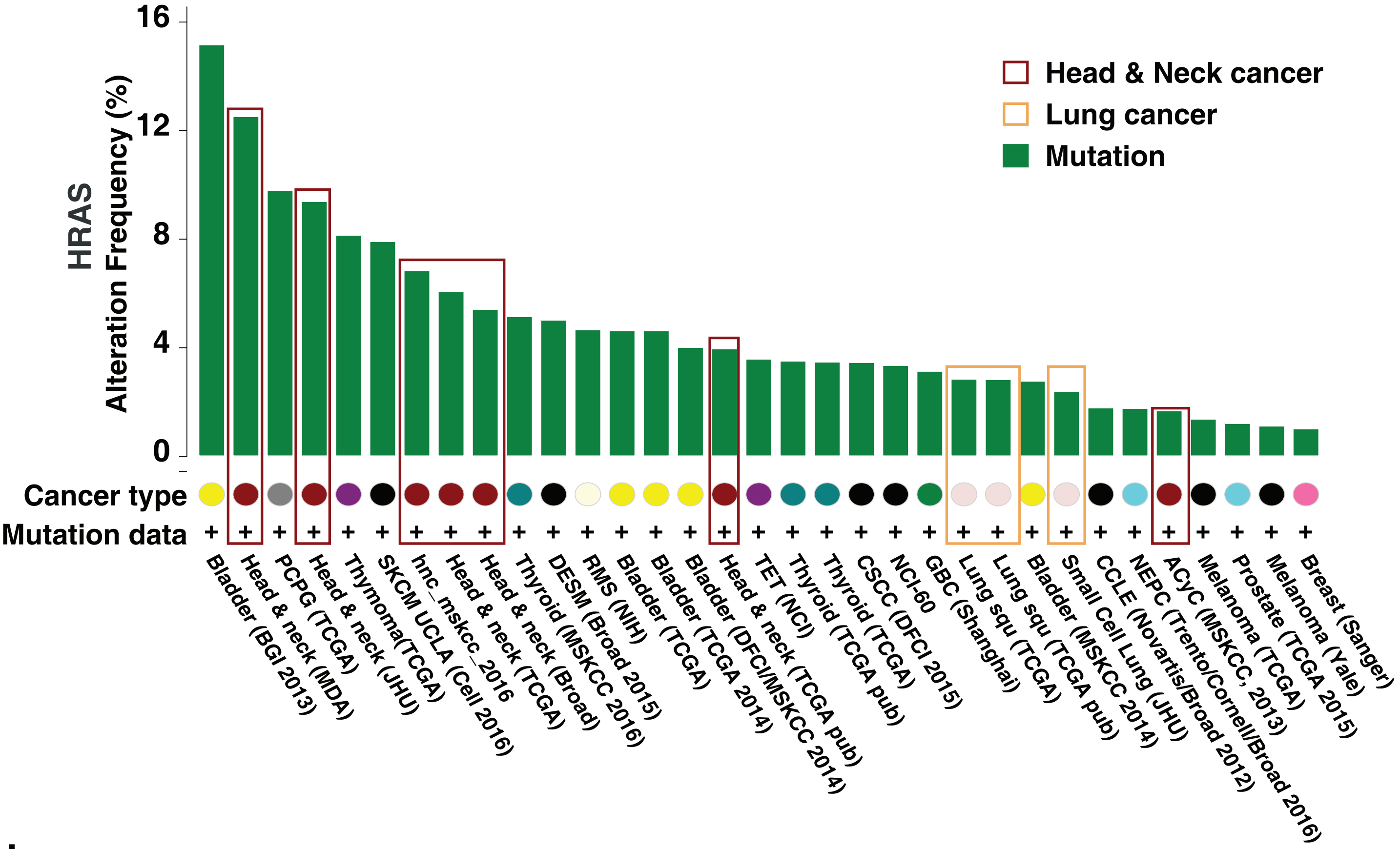


**b**

| 44 mutations<br>AA change : G12A/C/D/N/R/S/V | |
|---|---|
| **Cancer Type** | **Count** |
| Head and Neck Squamous Cell Carcinoma | 22 |
| Mixed Cancer Types | 11 |
| Bladder Urothelial Cancer | 6 |
| Non-Small Cell Lung Cancer | 1 |
| Tymoma | 1 |
| Adenoid Cystic Carcinoma | 3 |
| Invasive Breast Carcinoma | 4 |

| 55 mutations<br>AA change : G13C/D/I/N/R/S/V/dup | |
|---|---|
| **Cancer Type** | **Count** |
| Mixed Cancer Types | 15 |
| Head and Neck Squamous Cell Carcinoma | 14 |
| Bladder Urothelial Cancer | 5 |
| Cutaneous Melanoma | 4 |
| Tymoma | 4 |
| Lung Squamous Cell Carcinoma | 3 |
| Non-Small Cell Lung Cancer | 2 |
| Miscellaneous Neuroepithelial Tumor | 2 |
| Thyroid | 2 |
| Cutaneous Squamous Cell Carcinoma | 1 |

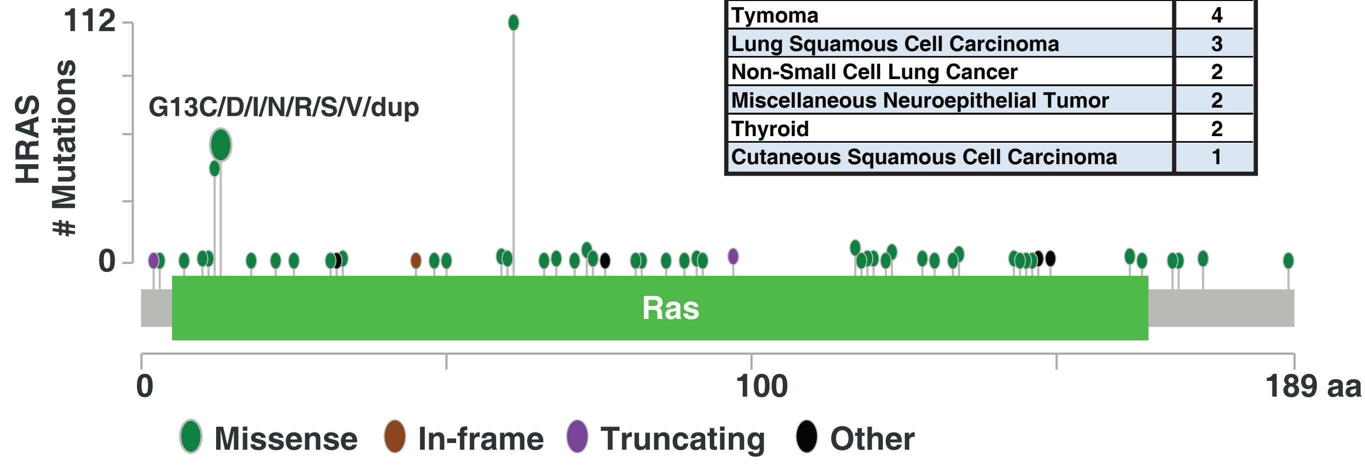

# Supplementary fig. S3

**a**

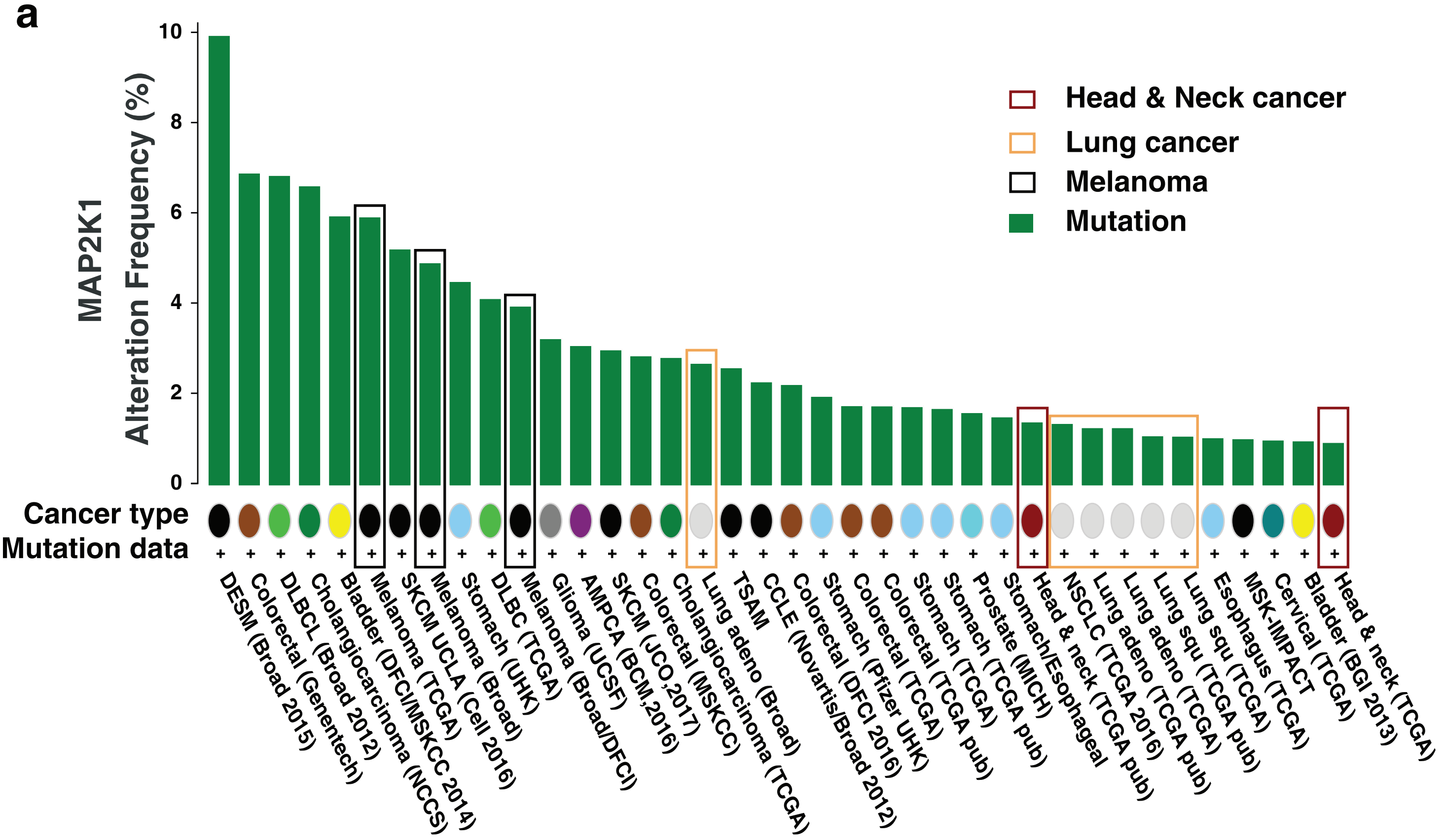


**b**

32 mutations
AA change : K57E/N/T

| Cancer Type | Count |
|---|---|
| Mixed Cancer Types | 16 |
| Colorectal Adenocarcinoma | 3 |
| Lung Adenocarcinoma | 2 |
| Non-Small Cell Lung Cancer | 2 |
| Cutaneous Melanoma | 2 |
| Ampullary Carcinoma | 2 |
| Prostrate Carcinoma | 2 |
| Diffuse Large B-Cell Lymphoma | 1 |
| Head and Neck Squamous Cell Carcinoma | 1 |
| Stomach Adenocarcinoma | 1 |

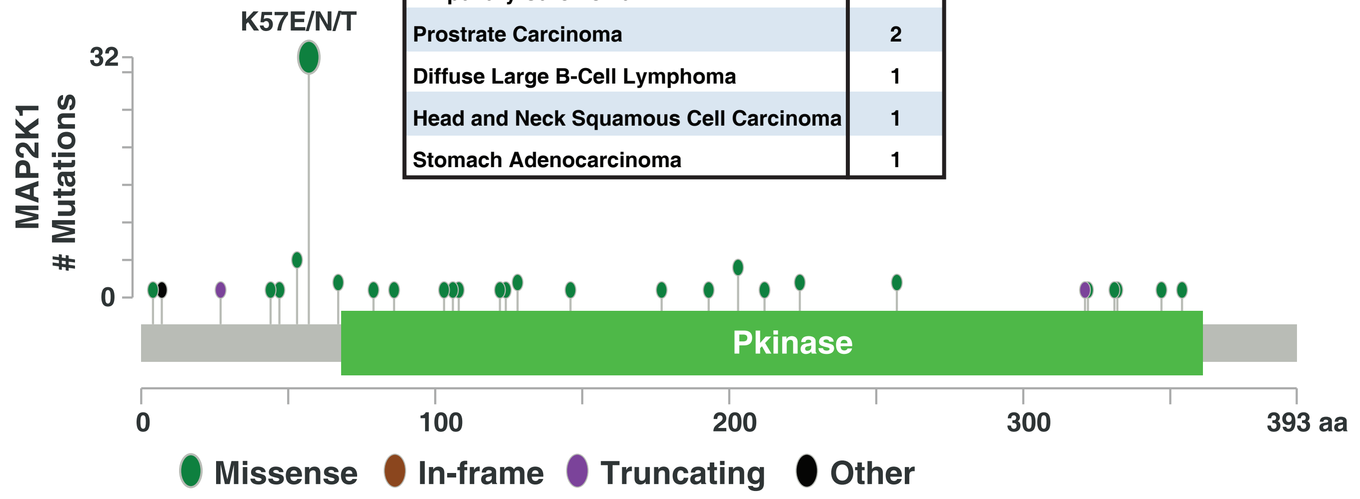

## Supplementary fig. S4

**a**

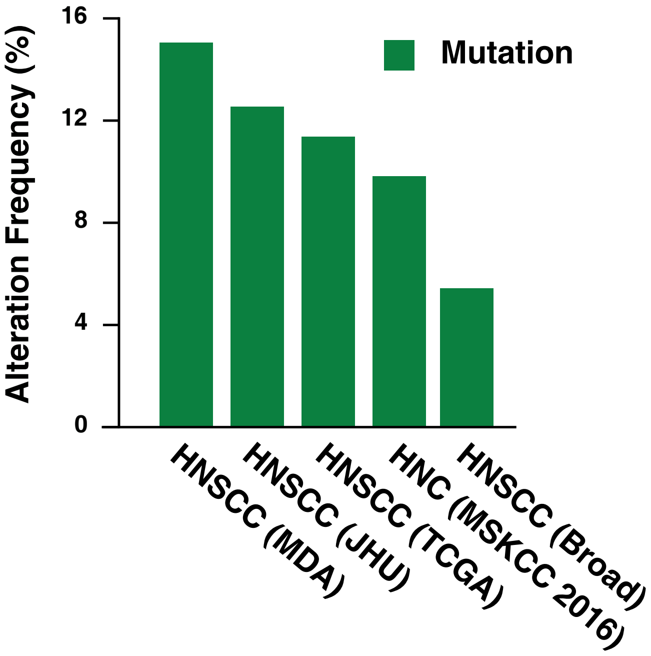


**b**

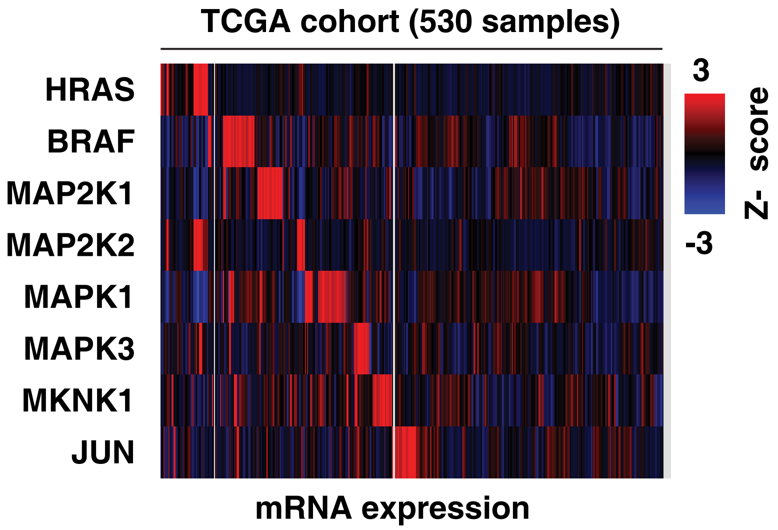

**Jain *et al.* , 2019. MAP2K1 is a potential therapeutic target in erlotinib resistant head and neck squamous cell carcinoma**

**Supplementary Table S1: Summary of exome sequencing analysis of SCC-R and SCC-S cells**

| **Cell line** | | **SSC-S** | **SSC-R** |
|---|---|---|---|
| **Fastq metrics** | **Total reads** | 115562852 | 116202990 |
| | **Average read length** | 100 | 100 |
| | **Average base quality** | 39.19 | 38.97 |
| **Trimming metrics** | **Total reads** | 115562006 | 116202460 |
| | **Average read length** | 99.9 | 99.9 |
| | **Average base quality** | 39.03 | 38.82 |
| **Alignment metrics** | **Alignment percentage** | 99.57% | 99.87% |
| **Target region coverage statistics** | **1x- 10x** | 0.9 | 0.7 |
| | **11x – 30x** | 5.7 | 5.0 |
| | **31x – 50x** | 10.4 | 10.0 |
| | **51x – 100x** | 29.7 | 30.1 |
| | **>100x** | 53.0 | 53.9 |
| | **On-target region average depth** | 124.6 | 127.8 |
| **Alterations** | **somatic SNVs (allele frequency ≥ 10%)** | - | 418 |
| | **somatic non-synonymous SNVs (allele frequency ≥ 10%)** | - | 180 |
| | **Genes affected by CNAs [$\log_2$(fold change) < 1 OR ≥ 3]** | - | 1369 |

Jain *et al.* , 2019. MAP2K1 is a potential therapeutic target in erlotinib resistant head and neck squamous cell carcinoma
**Supplementary Table S2: List of single nucleotide variants identified in SCC-R cells.**

| Map Location | Genomic position | Reference allele | Alternate allele | Mutation type | Gene symbol | Protein | Nucleotide change | Description | OncoMD | COSMIC | dbSNP | ExAC | 1000G | SIFT | CONDEL | LRT | VEP | FATHMM | METASVM | METALR | PhastCons7 way | DP in SCC-S | Reference read in SCC-S | Alternative read in SCC-S | Alternate allele frequency in SCC-S (%) | DP in SCC-R | Reference read in SCC-R | Alternate read in SCC-R | Alternate allele frequency in SCC-R (%) |
|---|---|---|---|---|---|---|---|---|---|---|---|---|---|---|---|---|---|---|---|---|---|---|---|---|---|---|---|---|---|
| Chr1_p12 | 118579447 | C | A | NONSENSE | **SPAG17** | sperm associated antigen 17 | c.3379G>T | SPAG17:NM_206996:exon24:c.G3379T:p.E1127X | NA | NA | NA | NA | NA | NA | NA | NA | HIGH | NA | NA | NA | 0.998 | 98 | 97 | 0 | 0 | 142 | 106 | 36 | 25.4 |
| Chr1_p22.2 | 89225947 | G | T | MISSENSE | **PKN2** | protein kinase N2 | c.392G>T | PKN2:NM_006256:exon3:c.G392T:p.C131F | NA | NA | NA | NA | NA | T | N | U | MODERATE | T | T | T | 0.999 | 51 | 51 | 0 | 0 | 105 | 86 | 18 | 17.1 |
| Chr1_p31.1 | 70644562 | G | T | MISSENSE | **LRRC40** | leucine rich repeat containing 40 | c.776C>A | LRRC40:NM_017768:exon6:c.C776A:p.P259Q | chr1*70644562*70644562*G*T*1*1*Stomach_(gastric)_cancer | NA | NA | NA | NA | D | D | D | MODERATE | T | T | T | 0.998 | 33 | 33 | 0 | 0 | 96 | 85 | 11 | 11.5 |
| Chr1_p31.1 | 74929160 | A | G | MISSENSE | **FPGT-TNNI3K** | FPGT-TNNI3K readthrough | c.2350A>G,c.2047A>G,c.2350A>G | TNNI3K:NM_015978:exon21:c.A2047G:p.R683G,FPGT-TNNI3K:NM_001112808:exon23:c.A2389G:p.R797G,FPGT-TNNI3K:NM_001199327:exon23:c.A2389G:p.R797G | NA | NA | NA | NA | NA | D | D | NA | MODERATE | NA | NA | NA | 0.998 | 141 | 141 | 0 | 0 | 228 | 179 | 47 | 20.6 |
| Chr1_p31.1 | 78194335 | G | A | SILENT | **USP33** | ubiquitin specific peptidase 33 | c.873C>T,c.873C>T,c.780C>T | USP33:NM_201624:exon10:c.C780T:p.V260V,USP33:NM_015017:exon11:c.C873T:p.V291V,USP33:NM_201626:exon11:c.C873T:p.V291V | NA | NA | NA | NA | NA | NA | NA | NA | LOW | NA | NA | NA | NA | 221 | 220 | 0 | 0 | 338 | 254 | 83 | 24.6 |
| Chr1_p31.3 | 64125368 | C | T | 3UTR | **PGM1** | phosphoglucomutase 1 | c.*22C>T,c.*22C>T,c.*22C>T | NM_001172818.1 | NA | NA | rs8294 | 1.99E-01 | 1.85E-01 | NA | NA | NA | MODIFIER | NA | NA | NA | NA | 104 | 104 | 0 | 0 | 131 | 125 | 6 | 4.6 |
| Chr1_p31.3 | 67098652 | G | C | INTRONIC | **SGIP1** | SH3 domain GRB2 like endophilin interacting protein 1 | c.75-101G>C | NM_032291.2 | NA | NA | NA | NA | NA | NA | NA | NA | MODIFIER | NA | NA | NA | NA | 74 | 73 | 0 | 0 | 97 | 56 | 41 | 42.3 |
| Chr1_p32.2 | 57399106 | A | C | MISSENSE | **C8B** | complement C8 beta chain | c.1268T>G,c.1298T>G,c.1454T>G | C8B:NM_000066:exon10:c.T1454G:p.V485G,C8B:NM_001278543:exon11:c.T1298G:p.V433G,C8B:NM_001278544:exon11:c.T1268G:p.V423G | NA | NA | NA | NA | NA | D | D | N | MODERATE | D | D | D | 0.179 | 141 | 141 | 0 | 0 | 166 | 124 | 42 | 25.3 |
| Chr1_p32.3 | 55139637 | A | G | INTRONIC | **MROH7** | maestro heat like repeat family member 7 | c.1801-52A>G | NM_001039464.2 | NA | NA | NA | NA | NA | NA | NA | NA | MODIFIER | NA | NA | NA | NA | 45 | 44 | 0 | 0 | 62 | 47 | 15 | 24.2 |
| Chr1_p33 | 48244142 | C | A | MISSENSE | **TRABD2B** | TraB domain containing 2B | c.1063G>T | TRABD2B:NM_001194986:exon5:c.G1063T:p.G355W | NA | NA | NA | NA | NA | D | D | NA | MODERATE | NA | NA | NA | NA | 83 | 83 | 0 | 0 | 140 | 130 | 9 | 6.4 |
| Chr1_p34.1 | 44477358 | G | T | INTRONIC | **SLC6A9** | solute carrier family 6 member 9 | c.88-804C>A,c.31-804C>A | SLC6A9:NM_201649:exon2:c.C124A:p.P42T | NA | NA | NA | NA | NA | NA | NA | NA | MODIFIER | NA | NA | NA | NA | 95 | 95 | 0 | 0 | 126 | 86 | 40 | 31.8 |
| Chr1_p34.1 | 44477358 | G | T | MISSENSE | **SLC6A9** | solute carrier family 6 member 9 | c.124C>A | SLC6A9:NM_201649:exon2:c.C124A:p.P42T | NA | NA | NA | NA | NA | T_lc | N | N | MODERATE | T | T | T | 0.001 | 95 | 95 | 0 | 0 | 126 | 86 | 40 | 31.8 |
| Chr1_p34.2 | 43772563 | G | A | SILENT | **TIE1** | tyrosine kinase with immunoglobulin like and EGF like domains 1 | c.537G>A | TIE1:NM_001253357:exon4:c.G402A:p.L134L,TIE1:NM_005424:exon4:c.G537A:p.L179L | NA | NA | NA | NA | NA | NA | NA | NA | LOW | NA | NA | NA | NA | 117 | 117 | 0 | 0 | 202 | 146 | 54 | 26.7 |
| Chr1_p34.3 | 35885198 | C | T | MISSENSE | **ZMYM4** | zinc finger MYM-type containing 4 | c.4567C>T | ZMYM4:NM_005095:exon30:c.C4567T:p.R1523C | NA | NA | NA | NA | NA | D | D | D | MODERATE | T | T | T | 0.999 | 88 | 88 | 0 | 0 | 202 | 124 | 75 | 37.1 |
| Chr1_p35.2 | 32092833 | C | T | 3UTR | **HCRTR1** | hypocretin receptor 1 | c.*252C>T | NM_001525.2 | NA | NA | NA | NA | NA | NA | NA | NA | MODIFIER | NA | NA | NA | NA | 170 | 170 | 0 | 0 | 260 | 165 | 93 | 35.8 |
| Chr1_p35.2 | 32204468 | G | T | MISSENSE | **BAI2** | adhesion G protein-coupled receptor B2 | c.2566C>A | ADGRB2:NM_001294335:exon16:c.C2566A:p.P856T,ADGRB2:NM_001294336:exon16:c.C2566A:p.P856T | NA | NA | rs763244451 | 8.26E-06 | NA | D | D | N | MODERATE | T | T | T | 0.969 | 115 | 115 | 0 | 0 | 156 | 120 | 35 | 22.4 |
| Chr1_p36.11 | 26110143 | G | A | INTRONIC | **MAN1C1** | mannosidase alpha class 1C member 1 | c.1767-11G>A | NM_020379.2 | NA | NA | rs3767877 | 6.34E-01 | 6.42E-01 | NA | NA | NA | MODIFIER | NA | NA | NA | NA | 117 | 116 | 0 | 0 | 161 | 155 | 6 | 3.7 |
| Chr1_p36.11 | 26110162 | A | C | MISSENSE | **MAN1C1** | mannosidase alpha class 1C member 1 | c.1775A>C | MAN1C1:NM_020379:exon12:c.A1775C:p.Y592S | NA | NA | NA | NA | NA | D | D | D | MODERATE | D | D | D | 0.999 | 120 | 120 | 0 | 0 | 168 | 92 | 76 | 45.2 |
| Chr1_p36.12 | 22838274 | G | T | MISSENSE | **ZBTB40** | zinc finger and BTB domain containing 40 | c.2108G>T,c.2108G>T | ZBTB40:NM_014870:exon11:c.G2108T:p.S703I,ZBTB40:NM_001083621:exon12:c.G2108T:p.S703I | NA | NA | NA | NA | NA | T | N | N | MODERATE | T | T | T | 0.005 | 77 | 77 | 0 | 0 | 107 | 50 | 56 | 52.3 |
| Chr1_p36.32 | 3328114 | C | A | NONSENSE | **PRDM16** | PR/SET domain 16 | c.1353C>A,c.1353C>A | PRDM16:NM_022114:exon9:c.C1353A:p.Y451X,PRDM16:NM_199454:exon9:c.C1353A:p.Y451X | NA | NA | NA | NA | NA | NA | NA | NA | HIGH | NA | NA | NA | 0.999 | 178 | 178 | 0 | 0 | 210 | 101 | 109 | 51.9 |
| Chr1_p36.32 | 3432058 | C | G | MISSENSE | **MEGF6** | multiple EGF like domains 6 | c.638G>C | MEGF6:NM_001409:exon6:c.G638C:p.C213S | NA | NA | NA | NA | NA | D | D | D | MODERATE | D | D | D | 0.991 | 55 | 55 | 0 | 0 | 91 | 27 | 64 | 70.3 |
| Chr1_p36.33 | 1688035 | C | G | MISSENSE | **NADK** | NAD kinase | c.841G>C,c.406G>C,c.310G>C,c.406G>C | NADK:NM_001198995:exon3:c.G310C:p.V104L,NADK:NM_001198993:exon5:c.G406C:p.V136L,NADK:NM_023018:exon5:c.G406C:p.V136L,NADK:NM_001198994:exon7:c.G841C:p.V281L | chr1*1688035*1688035*C*T*1*1*Breast_cancer | chr1*1688035*1688035*COSM1472952*C*T*1*breast*1\|chr1*1688035*1688035*COSM1472951*C*T*1*breast*1 | NA | NA | NA | D | D | D | MODERATE | T | T | T | 0.998 | 71 | 71 | 0 | 0 | 86 | 32 | 54 | 62.8 |
| Chr1_q21.2 | 150256778 | T | G | INTRONIC | **C1orf51** | circadian associated repressor of transcription | c.443-58T>G | NM_144697.2 | NA | NA | NA | NA | NA | NA | NA | NA | MODIFIER | NA | NA | NA | NA | 43 | 43 | 0 | 0 | 54 | 37 | 17 | 31.5 |
| Chr1_q21.3 | 154950571 | T | G | MISSENSE | **CKS1B** | CDC28 protein kinase regulatory subunit 1B | c.168T>G | CKS1B:NM_001826:exon2:c.T168G:p.H56Q | NA | NA | NA | NA | NA | D | D | D | MODERATE | 0 | T | T | 0.999 | 134 | 134 | 0 | 0 | 130 | 95 | 35 | 26.9 |
| Chr1_q22 | 155264177 | C | A | INTRONIC-SS-ACR | **PKLR** | pyruvate kinase L/R | c.966-1G>T,c.873-1G>T | NM_181871.3 | NA | NA | NA | NA | NA | NA | NA | NA | HIGH | NA | NA | NA | NA | 163 | 162 | 0 | 0 | 126 | 86 | 40 | 31.8 |
| Chr1_q22 | 156265447 | T | C | 5UTR | **C1orf85** | glycosylated lysosomal membrane protein | c.-11A>G | NM_001256609.1,NM_144580.2,NM_001256604.1 | NA | NA | NA | NA | NA | NA | NA | NA | MODIFIER | NA | NA | NA | NA | 32 | 32 | 0 | 0 | 31 | 18 | 13 | 41.9 |
| Chr1_q23.1 | 158450382 | C | T | MISSENSE | **OR10R2** | olfactory receptor family 10 subfamily R member 2 | c.715C>T | OR10R2:NM_001004472:exon1:c.C715T:p.L239F | NA | NA | rs1418843 | 4.41E-01 | 4.35E-01 | D | N | NA | MODERATE | NA | NA | NA | NA | 224 | 223 | 0 | 0 | 220 | 212 | 8 | 3.6 |
| Chr1_q23.1 | 158735723 | G | T | SILENT | **OR6N1** | olfactory receptor family 6 subfamily N member 1 | c.750C>A | OR6N1:NM_001005185:exon1:c.C750A:p.I250I | NA | NA | NA | NA | NA | NA | NA | NA | LOW | NA | NA | NA | 1 | 120 | 118 | 0 | 0 | 92 | 63 | 28 | 30.4 |
| Chr1_q23.2 | 160263124 | C | T | INTRONIC | **COPA** | coatomer protein complex subunit alpha | c.2754+52G>A | NM_004371.3,NM_001098398.1 | NA | NA | rs1317944 | NA | 6.69E-01 | NA | NA | NA | MODIFIER | NA | NA | NA | NA | 197 | 195 | 1 | 0.51 | 208 | 200 | 8 | 3.9 |
| Chr1_q23.3 | 161132402 | C | G | INTRONIC-SS-ACR-PRX | **USP21** | ubiquitin specific peptidase 21 | c.782-3C>G,c.782-3C>G | NM_001014443.2 | NA | NA | NA | NA | NA | NA | NA | NA | LOW | NA | NA | NA | NA | 140 | 140 | 0 | 0 | 141 | 129 | 12 | 8.5 |
| Chr1_q23.3 | 162372431 | A | G | INTRONIC | **SH2D1B** | SH2 domain containing 1B | c.198+98T>C | NM_053282.4 | NA | NA | rs556684664 | NA | NA | NA | NA | NA | MODIFIER | NA | NA | NA | NA | 207 | 206 | 0 | 0 | 204 | 134 | 68 | 33.3 |
| Chr1_q25.3 | 180601342 | A | T | MISSENSE | **XPR1** | xenotropic and polytropic retrovirus receptor 1 | c.5A>T,c.5A>T | XPR1:NM_001135669:exon1:c.A5T:p.K2M,XPR1:NM_004736:exon1:c.A5T:p.K2M | NA | NA | NA | NA | NA | D | D | D | MODERATE | T | D | D | 1 | 151 | 150 | 0 | 0 | 116 | 89 | 26 | 22.4 |
| Chr1_q25.3 | 184801065 | T | C | SILENT | **FAM129A** | family with sequence similarity 129 member A | c.633A>G | FAM129A:NM_052966:exon6:c.A633G:p.Q211Q | chr1*184801065*184801065*A*G*1*1*Colorectal_cancer | chr1*184801065*184801065*COSM1337073*T*C*1*large_intestine*1 | rs487675 | 2.97E-01 | 2.84E-01 | NA | NA | NA | LOW | NA | NA | NA | 1 | 239 | 238 | 0 | 0 | 239 | 228 | 7 | 2.9 |
| Chr1_q41 | 220154768 | C | T | SILENT | **EPRS** | glutamyl-prolyl-tRNA synthetase | c.3405G>A | EPRS:NM_004446:exon24:c.G3405A:p.Q1135Q | NA | NA | rs1061160 | 3.46E-01 | 3.54E-01 | NA | NA | NA | LOW | NA | NA | NA | 0.998 | 198 | 198 | 0 | 0 | 192 | 186 | 6 | 3.1 |
| Chr1_q41 | 222817762 | G | C | INTRONIC | **MIA3** | MIA family member 3, ER export factor | c.3478-1134G>C | NM_198551.2 | NA | NA | NA | NA | NA | NA | NA | NA | MODIFIER | NA | NA | NA | NA | 141 | 141 | 0 | 0 | 216 | 174 | 41 | 19.0 |
| Chr1_q42.13 | 227172935 | C | G | INTRONIC | **ADCK3** | coenzyme Q8A | c.1573-20C>G | NM_020247.4 | NA | NA | rs2297416 | 3.63E-01 | 2.65E-01 | NA | NA | NA | MODIFIER | NA | NA | NA | NA | 60 | 60 | 0 | 0 | 59 | 53 | 6 | 10.2 |
| Chr1_q42.13 | 228113046 | C | T | SILENT | **WNT9A** | Wnt family member 9A | c.270G>A | WNT9A:NM_003395:exon2:c.G270A:p.A90A | NA | NA | rs8192629 | 1.07E-01 | 8.27E-02 | NA | NA | NA | LOW | NA | NA | NA | NA | 110 | 110 | 0 | 0 | 98 | 93 | 5 | 5.1 |
| Chr1_q42.2 | 230807384 | C | A | SILENT-SS-PRX | **COG2** | component of oligomeric golgi complex 2 | c.897C>A | COG2:NM_001145036:exon8:c.C897A:p.S299S,COG2:NM_007357:exon8:c.C897A:p.S299S | NA | NA | NA | NA | NA | NA | NA | NA | LOW | NA | NA | NA | NA | 122 | 121 | 0 | 0 | 96 | 59 | 37 | 38.5 |
| Chr1_q43 | 237755076 | A | G | MISSENSE | **RYR2** | ryanodine receptor 2 | c.4198A>G | RYR2:NM_001035:exon32:c.A4198G:p.S1400G | NA | NA | rs56229512 | 1.82E-02 | 7.19E-03 | NA | NA | N | MODERATE | D | T | D | 0.993 | 249 | 247 | 1 | 0.4 | 199 | 191 | 7 | 3.5 |
| Chr1_q43 | 240256558 | G | T | MISSENSE | **FMN2** | formin 2 | c.1149G>T | FMN2:NM_001305424:exon1:c.G1149T:p.E383D,FMN2:NM_020066:exon1:c.G1149T:p.E383D | NA | NA | NA | NA | NA | NA | NA | NA | MODERATE | NA | NA | NA | NA | 110 | 110 | 0 | 0 | 118 | 81 | 36 | 30.5 |
| Chr1_q43 | 241683024 | G | A | 5UTR | **FH** | fumarate hydratase | c.-2C>T | NM_000143.3 | NA | NA | NA | NA | NA | NA | NA | NA | MODIFIER | NA | NA | NA | NA | 22 | 21 | 1 | 4.55 | 17 | 10 | 7 | 41.2 |
| Chr1_q43 | 243328738 | C | A | NONSENSE | **CEP170** | centrosomal protein 170 | c.2230G>T,c.2230G>T,c.2524G>T | CEP170:NM_001042404:exon12:c.G2230T:p.E744X,CEP170:NM_001042405:exon12:c.G2230T:p.E744X,CEP170:NM_014812:exon13:c.G2524T:p.E842X | NA | NA | NA | NA | NA | NA | NA | NA | HIGH | NA | NA | NA | 0.987 | 98 | 97 | 0 | 0 | 98 | 81 | 14 | 14.3 |
| Chr1_q43\|1_q23.1 | 240975213 | T | C | INTRONIC-SS-DNR-PRX | **RGS7** | regulator of G protein signaling 7 | c.1082+5A>G,c.1082+5A>G,c.1082+5A>G,c.923+5A>G | NM_001282773.1 | NA | chr1*240975213*240975213*COSM146839*T*C*1*stomach*1 | rs2275742 | 5.06E-01 | 4.05E-01 | NA | NA | NA | LOW | NA | NA | NA | NA | 127 | 127 | 0 | 0 | 126 | 118 | 7 | 5.6 |
| Chr1_q44 | 246727600 | C | A | INTRONIC | **TFB2M** | transcription factor B2, mitochondrial | c.402+48G>T | NM_022366.2 | NA | NA | NA | NA | NA | NA | NA | NA | MODIFIER | NA | NA | NA | NA | 42 | 42 | 0 | 0 | 43 | 26 | 17 | 39.5 |
| Chr1_q44 | 247769511 | C | A | SILENT | **OR2G3** | olfactory receptor family 2 subfamily G member 3 | c.624C>A | OR2G3:NM_001001914:exon1:c.C624A:p.V208V | NA | NA | NA | NA | NA | NA | NA | NA | LOW | NA | NA | NA | NA | 299 | 296 | 0 | 0 | 236 | 225 | 10 | 4.2 |
| Chr2_p11.2 | 85554397 | C | T | MISSENSE | **TGOLN2** | trans-golgi network protein 2 | c.458G>A | TGOLN2:NM_001206841:exon2:c.G458A:p.R153K,TGOLN2:NM_001206844:exon2:c.G458A:p.R153K,TGOLN2:NM_006464:exon2:c.G458A:p.R153K | NA | NA | rs201124506 | 1.66E-05 | NA | T | N | NA | MODERATE | NA | NA | NA | NA | 739 | 720 | 10 | 1.35 | 598 | 571 | 19 | 3.2 |
| Chr2_p16.1 | 55561679 | C | A | MISSENSE | **CCDC88A** | coiled-coil domain containing 88A | c.2278G>T,c.2278G>T | CCDC88A:NM_001135597:exon15:c.G2278T:p.G760C,CCDC88A:NM_001254943:exon15:c.G2278T:p.G760C,CCDC88A:NM_018084:exon15:c.G2278T:p.G760C | NA | NA | NA | NA | NA | D | D | U | MODERATE | T | T | T | 0.998 | 80 | 80 | 0 | 0 | 102 | 63 | 39 | 38.2 |
| Chr2_p16.1 | 55874511 | C | G | MISSENSE | **PNPT1** | polyribonucleotide nucleotidyltransferase 1 | c.1573G>C | PNPT1:NM_033109:exon19:c.G1573C:p.E525Q | NA | NA | NA | NA | NA | T | N | D | MODERATE | T | T | T | 1 | 72 | 72 | 0 | 0 | 76 | 64 | 11 | 14.5 |
| Chr2_p16.3 | 49190342 | G | C | MISSENSE | **FSHR** | follicle stimulating hormone receptor | c.1540C>G,c.1618C>G | FSHR:NM_181446:exon9:c.C1540G:p.L514V,FSHR:NM_000145:exon10:c.C1618G:p.L540V | chr2*49190342*49190342*C*A*1*1*Lung_cancer | chr2*49190342*49190342*COSM394499*G*T*1*lung*1 | NA | NA | NA | T | N | N | MODERATE | T | T | T | 0.998 | 253 | 253 | 0 | 0 | 156 | 144 | 9 | 5.8 |

**Supplementary Table S2: List of single nucleotide variants identified in SCC-R cells.**

| Map Location | Genomic position | Reference allele | Alternate allele | Mutation type | Gene symbol | Protein | Nucleotide change | Description | OncoMD | COSMIC | dbSNP | ExAC | 1000G | SIFT | CONDEL | LRT | VEP | FATHMM | METASVM | METALR | PhastCons7 way | DP in SCC-S | Reference read in SCC-S | Alternative read in SCC-S | Alternate allele frequency in SCC-S (%) | DP in SCC-R | Reference read in SCC-R | Alternate read in SCC-R | Alternate allele frequency in SCC-R (%) |
|---|---|---|---|---|---|---|---|---|---|---|---|---|---|---|---|---|---|---|---|---|---|---|---|---|---|---|---|---|---|
| Chr2_p16.3 | 50539812 | G | T | INTRONIC | **NRXN1** | neurexin 1 | c.3485-75704C>A,c.3365-75704C>A,c.259+34017C>A | NM_138735.2 | NA | NA | NA | NA | NA | NA | NA | NA | MODIFIER | NA | NA | NA | NA | 142 | 141 | 0 | 0 | 137 | 126 | 11 | 8.0 |
| Chr2_p16.3 | 51254713 | C | A | SILENT | **NRXN1** | neurexin 1 | c.699G>T,c.699G>T | NRXN1:NM_001135659:exon2:c.G699T:p.V233V,NRXN1:NM_004801:exon2:c.G699T:p.V233V | NA | NA | NA | NA | NA | NA | NA | NA | LOW | NA | NA | NA | NA | 159 | 159 | 0 | 0 | 112 | 64 | 48 | 42.9 |
| Chr2_p16.3 | 51254714 | A | T | MISSENSE | **NRXN1** | neurexin 1 | c.698T>A,c.698T>A | NRXN1:NM_001135659:exon2:c.T698A:p.V233E,NRXN1:NM_004801:exon2:c.T698A:p.V233E | NA | NA | NA | NA | NA | D | N | U | MODERATE | T | D | D | 0.995 | 159 | 159 | 0 | 0 | 113 | 64 | 49 | 43.4 |
| Chr2_p22.3 | 32379538 | C | G | MISSENSE | **SPAST** | spastin | c.1824C>G,c.1728C>G | SPAST:NM_199436:exon16:c.C1728G:p.N576K,SPAST:NM_014946:exon17:c.C1824G:p.N608K | NA | NA | NA | NA | NA | D | D | D | MODERATE | D | D | D | 1 | 171 | 171 | 0 | 0 | 123 | 105 | 18 | 14.6 |
| Chr2_p25.3 | 1440162 | G | T | INTRONIC-SS-DNR-PRX | **TPO** | thyroid peroxidase | c.482+6G>T,c.482+6G>T,c.482+6G>T,c.482+6G>T,c.482+6G>T | NM_000547.5 | NA | NA | rs776798689 | 8.26E-06 | NA | NA | NA | NA | LOW | NA | NA | NA | NA | 202 | 202 | 0 | 0 | 94 | 62 | 31 | 33.0 |
| Chr2_q12.1 | 102955414 | A | G | MISSENSE | **IL1RL1** | interleukin 1 receptor like 1 | c.179A>G,c.179A>G,c.179A>G | IL1RL1:NM_003856:exon3:c.A179G:p.Q60R,IL1RL1:NM_016232:exon3:c.A179G:p.Q60R | NA | NA | rs765932268 | 8.24E-06 | NA | T | N | N | MODERATE | T | T | T | 0.015 | 216 | 216 | 0 | 0 | 250 | 176 | 73 | 29.2 |
| Chr2_q12.1 | 103121852 | A | T | MISSENSE | **SLC9A4** | solute carrier family 9 member A4 | c.1120A>T | SLC9A4:NM_001011552:exon4:c.A1120T:p.T374S | NA | NA | NA | NA | NA | D | D | D | MODERATE | T | T | T | 0.927 | 206 | 205 | 0 | 0 | 180 | 119 | 61 | 33.9 |
| Chr2_q14.1 | 113780355 | C | A | INTRONIC-SS-ACR | **IL36B** | interleukin 36 beta | c.392-1G>T | NM_014438.4 | NA | NA | rs763458820 | 8.27E-06 | NA | NA | NA | NA | HIGH | NA | NA | NA | NA | 130 | 130 | 0 | 0 | 129 | 110 | 18 | 14.0 |
| Chr2_q21.2 | 133554175 | G | A | INTRONIC | **NCKAP5** | NCK associated protein 5 | c.909+26C>T,c.909+26C>T | NM_207363.2 | NA | NA | NA | NA | NA | NA | NA | NA | MODIFIER | NA | NA | NA | NA | 255 | 255 | 0 | 0 | 286 | 205 | 80 | 28.0 |
| Chr2_q21.3 | 136261864 | T | C | INTRONIC | **ZRANB3** | zinc finger RANBP2-type containing 3 | c.161+35A>G,c.161+35A>G | NM_032143.2 | NA | NA | NA | NA | NA | NA | NA | NA | MODIFIER | NA | NA | NA | NA | 59 | 59 | 0 | 0 | 94 | 69 | 24 | 25.5 |
| Chr2_q22.1 | 138758469 | C | G | INTRONIC | **HNMT** | histamine N-methyltransferase | c.191-19C>G | NM_006895.2 | NA | NA | NA | NA | NA | NA | NA | NA | MODIFIER | NA | NA | NA | NA | 130 | 130 | 0 | 0 | 126 | 91 | 32 | 25.4 |
| Chr2_q22.1-_q22.2 | 141459897 | A | G | INTRONIC | **LRP1B** | LDL receptor related protein 1B | c.6151-36T>C | NM_018557.2 | NA | NA | NA | NA | NA | NA | NA | NA | MODIFIER | NA | NA | NA | NA | 177 | 177 | 0 | 0 | 253 | 177 | 72 | 28.5 |
| Chr2_q22.2 | 143718298 | C | T | MISSENSE | **KYNU** | kynureninase | c.688C>T,c.688C>T,c.688C>T | KYNU:NM_001032998:exon8:c.C688T:p.H230Y,KYNU:NM_003937:exon8:c.C688T:p.H230Y,KYNU:NM_001199241:exon9:c.C688T:p.H230Y | NA | NA | NA | NA | NA | T | N | N | MODERATE | D | T | T | 0.987 | 216 | 216 | 0 | 0 | 270 | 225 | 44 | 16.3 |
| Chr2_q22.3 | 145187377 | C | A | MISSENSE | **ZEB2** | zinc finger E-box binding homeobox 2 | c.290G>T,c.290G>T | ZEB2:NM_001171653:exon3:c.G290T:p.W97L,ZEB2:NM_014795:exon3:c.G290T:p.W97L | chr2*145187377*145187377*G*A*1*1*Endometrial_carcinoma | chr2*145187377*145187377*COSM1007175*C*T*1*endometrium*1 | NA | NA | NA | D | D | D | MODERATE | D | D | D | 0.975 | 117 | 117 | 0 | 0 | 134 | 102 | 31 | 23.1 |
| Chr2_q24.3 | 166915099 | T | C | MISSENSE | **SCN1A** | sodium voltage-gated channel alpha subunit 1 | c.364A>G,c.364A>G | SCN1A:NM_001165963:exon2:c.A364G:p.I122V,SCN1A:NM_001165964:exon2:c.A364G:p.I122V,SCN1A:NM_006920:exon2:c.A364G:p.I122V,SCN1A:NM_001202435:exon4:c.A364G:p.I122V | NA | NA | NA | NA | NA | T | N | D | MODERATE | D | D | D | 1 | 96 | 96 | 0 | 0 | 104 | 67 | 37 | 35.6 |
| Chr2_q31.1 | 170082963 | G | T | MISSENSE | **LRP2** | LDL receptor related protein 2 | c.5363C>A | LRP2:NM_004525:exon32:c.C5363A:p.A1788D | NA | NA | NA | NA | NA | NA | NA | N | MODERATE | D | T | T | 0.948 | 127 | 127 | 0 | 0 | 113 | 83 | 30 | 26.6 |
| Chr2_q31.1 | 176812439 | T | C | INTRONIC | **KIAA1715** | lunapark, ER junction formation factor | c.494-19A>G | NM_030650.1 | NA | NA | NA | NA | NA | NA | NA | NA | MODIFIER | NA | NA | NA | NA | 24 | 23 | 0 | 0 | 13 | 8 | 5 | 38.5 |
| Chr2_q31.1 | 177036756 | G | T | MISSENSE | **HOXD3** | homeobox D3 | c.1053G>T | HOXD3:NM_006898:exon3:c.G1053T:p.L351F | NA | NA | NA | NA | NA | D | D | D | MODERATE | D | D | D | 1 | 270 | 269 | 0 | 0 | 274 | 180 | 90 | 32.9 |
| Chr2_q31.2 | 179590175 | G | T | MISSENSE | **TTN** | titin | c.17024C>A,c.20756C>A | TTN:NM_133378:exon68:c.C17024A:p.P5675Q,TTN:NM_001256850:exon69:c.C19805A:p.P6602Q,TTN:NM_001267550:exon71:c.C20756A:p.P6919Q | NA | NA | NA | NA | NA | NA | NA | NA | MODERATE | NA | NA | NA | NA | 369 | 366 | 1 | 0.27 | 338 | 226 | 111 | 32.8 |
| Chr2_q31.2 | 179590175 | G | T | INTRONIC | **TTN** | titin | c.13657+12634C>A,c.13858+12634C>A,c.13282+12634C>A | TTN:NM_133378:exon68:c.C17024A:p.P5675Q,TTN:NM_001256850:exon69:c.C19805A:p.P6602Q,TTN:NM_001267550:exon71:c.C20756A:p.P6919Q | NA | NA | NA | NA | NA | NA | NA | NA | MODIFIER | NA | NA | NA | NA | 369 | 366 | 1 | 0.27 | 338 | 226 | 111 | 32.8 |
| Chr2_q32.2 | 189859857 | G | T | INTRONIC | **COL3A1** | collagen type III alpha 1 chain | c.1509+32G>T | NM_000090.3 | NA | NA | NA | NA | NA | NA | NA | NA | MODIFIER | NA | NA | NA | NA | 64 | 63 | 0 | 0 | 59 | 34 | 25 | 42.4 |
| Chr2_q32.3 | 196663997 | T | C | INTRONIC | **DNAH7** | dynein axonemal heavy chain 7 | c.10353+23A>G | NM_018897.2 | NA | NA | rs7567285 | 5.67E-01 | 4.59E-01 | NA | NA | NA | MODIFIER | NA | NA | NA | NA | 67 | 66 | 0 | 0 | 77 | 71 | 5 | 6.5 |
| Chr2_q33.1 | 198621289 | C | A | INTRONIC-SS-ACR-PRX | **BOLL** | boule homolog, RNA binding protein | c.601-9G>T,c.274-9G>T,c.637-9G>T,c.667-9G>T | NM_001284361.1 | NA | NA | NA | NA | NA | NA | NA | NA | MODIFIER | NA | NA | NA | NA | 51 | 50 | 0 | 0 | 71 | 50 | 21 | 29.6 |
| Chr2_q35 | 219204758 | A | G | MISSENSE | **PNKD** | paroxysmal nonkinesigenic dyskinesia | c.287A>G,c.359A>G | PNKD:NM_022572:exon3:c.A287G:p.K96R,PNKD:NM_015488:exon4:c.A359G:p.K120R | NA | NA | NA | NA | NA | T | N | D | MODERATE | D | D | D | 1 | 118 | 117 | 0 | 0 | 111 | 65 | 45 | 40.5 |
| Chr2_q36.1 | 223158894 | C | G | MISSENSE | **PAX3** | paired box 3 | c.578G>C,c.578G>C,c.578G>C,c.578G>C,c.578G>C | PAX3:NM_000438:exon4:c.G578C:p.S193T,PAX3:NM_001127366:exon4:c.G575C:p.S192T,PAX3:NM_013942:exon4:c.G578C:p.S193T,PAX3:NM_181457:exon4:c.G578C:p.S193T,PAX3:NM_181458:exon4:c.G578C:p.S193T,PAX3:NM_181459:exon4:c.G578C:p.S193T,PAX3:NM_181460:exon4:c.G578C:p.S193T,PAX3:NM_181461:exon4:c.G578C:p.S193T | NA | NA | NA | NA | NA | T | N | N | MODERATE | D | D | D | 0.99 | 165 | 165 | 0 | 0 | 99 | 68 | 30 | 30.3 |
| Chr2_q37.1 | 231738263 | C | T | SILENT | **ITM2C** | integral membrane protein 2C | c.252C>T,c.252C>T | ITM2C:NM_001012516:exon2:c.C252T:p.F84F,ITM2C:NM_001287240:exon2:c.C66T:p.F22F,ITM2C:NM_030926:exon2:c.C252T:p.F84F,ITM2C:NM_001287241:exon3:c.C252T:p.F84F | NA | NA | rs2289234 | 5.19E-01 | 5.10E-01 | NA | NA | NA | LOW | NA | NA | NA | 0.941 | 100 | 98 | 1 | 1 | 99 | 93 | 6 | 6.1 |
| Chr2_q37.1 | 231738263 | C | T | INTRONIC | **ITM2C** | integral membrane protein 2C | c.121-2072C>T | ITM2C:NM_001012516:exon2:c.C252T:p.F84F,ITM2C:NM_001287240:exon2:c.C66T:p.F22F,ITM2C:NM_030926:exon2:c.C252T:p.F84F,ITM2C:NM_001287241:exon3:c.C252T:p.F84F | NA | NA | rs2289234 | 5.19E-01 | 5.10E-01 | NA | NA | NA | MODIFIER | NA | NA | NA | NA | 100 | 98 | 1 | 1 | 99 | 93 | 6 | 6.1 |
| Chr2_q37.1 | 231738269 | G | A | INTRONIC | **ITM2C** | integral membrane protein 2C | c.121-2066G>A | ITM2C:NM_001012516:exon2:c.G258A:p.A86A,ITM2C:NM_001287240:exon2:c.G72A:p.A24A,ITM2C:NM_030926:exon2:c.G258A:p.A86A,ITM2C:NM_001287241:exon3:c.G258A:p.A86A | NA | NA | rs2289233 | 5.19E-01 | 5.09E-01 | NA | NA | NA | MODIFIER | NA | NA | NA | NA | 96 | 94 | 1 | 1.04 | 95 | 89 | 6 | 6.3 |
| Chr2_q37.1 | 231738269 | G | A | SILENT | **ITM2C** | integral membrane protein 2C | c.258G>A,c.258G>A | ITM2C:NM_001012516:exon2:c.G258A:p.A86A,ITM2C:NM_001287240:exon2:c.G72A:p.A24A,ITM2C:NM_030926:exon2:c.G258A:p.A86A,ITM2C:NM_001287241:exon3:c.G258A:p.A86A | NA | NA | rs2289233 | 5.19E-01 | 5.09E-01 | NA | NA | NA | LOW | NA | NA | NA | NA | 96 | 94 | 1 | 1.04 | 95 | 89 | 6 | 6.3 |
| Chr2_q37.1 | 233347919 | C | G | INTRONIC | **ECEL1** | endothelin converting enzyme like 1 | c.1507-30G>C | NM_004826.2 | NA | NA | rs6750085 | 4.32E-01 | 3.14E-01 | NA | NA | NA | MODIFIER | NA | NA | NA | NA | 107 | 106 | 1 | 0.93 | 101 | 95 | 6 | 5.9 |
| Chr2_q37.3 | 238303795 | C | A | INTRONIC | **COL6A3** | collagen type VI alpha 3 chain | c.91+1575G>T,c.91+1575G>T,c.91+1575G>T,c.91+1575G>T | NM_057165.4 | NA | NA | NA | NA | NA | NA | NA | NA | MODIFIER | NA | NA | NA | NA | 113 | 111 | 0 | 0 | 117 | 87 | 30 | 25.6 |
| Chr2_q37.3 | 238303795 | C | A | MISSENSE | **COL6A3** | collagen type VI alpha 3 chain | c.144G>T | COL6A3:NM_004369:exon3:c.G144T:p.W48C | NA | NA | NA | NA | NA | NA | NA | U | MODERATE | T | D | D | 0.998 | 113 | 111 | 0 | 0 | 117 | 87 | 30 | 25.6 |
| Chr2_q37.3 | 241405728 | C | G | 3UTR | **GPC1** | glypican 1 | c.*21C>G | NM_002081.2 | NA | NA | NA | NA | NA | NA | NA | NA | MODIFIER | NA | NA | NA | NA | 85 | 84 | 0 | 0 | 74 | 54 | 19 | 25.7 |
| Chr2_q37.3 | 241465125 | C | T | MISSENSE | **ANKMY1** | ankyrin repeat and MYND domain containing 1 | c.622G>A,c.1312G>A,c.1045G>A,c.622G>A,c.622G>A | ANKMY1:NM_001282780:exon5:c.G622A:p.E208K,ANKMY1:NM_001282781:exon5:c.G622A:p.E208K,ANKMY1:NM_017844:exon5:c.G622A:p.E208K,ANKMY1:NM_016552:exon6:c.G1045A:p.E349K,ANKMY1:NM_001282771:exon7:c.G1312A:p.E438K | NA | NA | NA | NA | NA | D | N | D | MODERATE | T | T | T | 0.147 | 151 | 151 | 0 | 0 | 146 | 108 | 38 | 26.0 |
| Chr2_q37.3 | 241711892 | C | A | INTRONIC | **KIF1A** | kinesin family member 1A | c.1207+95G>T,c.1180+639G>T | NM_004321.6 | NA | NA | NA | NA | NA | NA | NA | NA | MODIFIER | NA | NA | NA | NA | 50 | 50 | 0 | 0 | 52 | 43 | 9 | 17.3 |
| Chr3_p12.3 | 75714783 | G | T | MISSENSE | **FRG2C** | FSHD region gene 2 family member C | c.440G>T | FRG2C:NM_001124759:exon4:c.G440T:p.R147M | NA | NA | NA | NA | NA | D | D | U | MODERATE | T | T | T | 0.047 | 508 | 506 | 0 | 0 | 430 | 357 | 71 | 16.5 |
| Chr3_p14.1 | 69151152 | C | A | SILENT | **ARL6IP5** | ADP ribosylation factor like GTPase 6 interacting protein 5 | c.339C>A | ARL6IP5:NM_006407:exon2:c.C339A:p.I113I | NA | NA | rs10489 | 8.58E-02 | 1.65E-01 | NA | NA | NA | LOW | NA | NA | NA | 0.993 | 69 | 69 | 0 | 0 | 113 | 106 | 7 | 6.2 |
| Chr3_p21.2 | 51895977 | T | G | INTRONIC | **IQCF2** | IQ motif containing F2 | c.111+11T>G | NM_203424.1 | NA | NA | NA | NA | NA | NA | NA | NA | MODIFIER | NA | NA | NA | NA | 45 | 45 | 0 | 0 | 46 | 41 | 5 | 10.9 |
| Chr3_p21.2 | 51972235 | G | A | INTRONIC | **RRP9** | ribosomal RNA processing 9, U3 small nucleolar RNA binding protein | c.171-15C>T | NM_004704.4 | NA | NA | NA | NA | NA | NA | NA | NA | MODIFIER | NA | NA | NA | NA | 46 | 46 | 0 | 0 | 54 | 28 | 26 | 48.2 |
| Chr3_p21.31 | 45875809 | G | C | INTRONIC | **LZTFL1** | leucine zipper transcription factor like 1 | c.273-19C>G,c.312-19C>G,c.324-19C>G | NM_020347.3 | NA | NA | NA | NA | NA | NA | NA | NA | MODIFIER | NA | NA | NA | NA | 37 | 37 | 0 | 0 | 46 | 18 | 28 | 60.9 |
| Chr3_p21.31 | 47299061 | G | T | INTRONIC | **KIF9** | kinesin family member 9 | c.982-11C>A,c.982-11C>A | NM_001134878.1,NM_182902.3 | NA | NA | NA | NA | NA | NA | NA | NA | MODIFIER | NA | NA | NA | NA | 36 | 36 | 0 | 0 | 82 | 65 | 17 | 20.7 |
| Chr3_p21.31 | 48659002 | G | T | MISSENSE | **TMEM89** | transmembrane protein 89 | c.188C>A | TMEM89:NM_001008269:exon1:c.C188A:p.A63E | NA | NA | NA | NA | NA | T_lc | N | N | MODERATE | T | T | T | 0.019 | 52 | 51 | 1 | 1.92 | 44 | 40 | 4 | 9.1 |
| Chr3_p22.1 | 40231947 | C | T | MISSENSE | **MYRIP** | myosin VIIA and Rab interacting protein | c.1658C>T,c.1658C>T,c.1391C>T,c.1097C>T | MYRIP:NM_001284426:exon8:c.C1097T:p.T366I,MYRIP:NM_001284425:exon9:c.C1391T:p.T464I,MYRIP:NM_001284423:exon10:c.C1658T:p.T553I,MYRIP:NM_001284424:exon10:c.C1658T:p.T553I,MYRIP:NM_015460:exon10:c.C1658T:p.T553I | NA | NA | NA | NA | NA | T | N | N | MODERATE | T | T | T | 0.002 | 92 | 92 | 0 | 0 | 108 | 101 | 7 | 6.5 |
| Chr3_p22.1 | 40553086 | G | A | INTRONIC | **ZNF620** | zinc finger protein 620 | c.-77-4265G>A | ZNF620:NM_175888:exon3:c.G150A:p.L50L | NA | NA | NA | NA | NA | NA | NA | NA | MODIFIER | NA | NA | NA | NA | 159 | 159 | 0 | 0 | 202 | 159 | 42 | 20.8 |
| Chr3_p22.1 | 40553086 | G | A | SILENT-SS-PRX | **ZNF620** | zinc finger protein 620 | c.150G>A | ZNF620:NM_175888:exon3:c.G150A:p.L50L | NA | NA | NA | NA | NA | NA | NA | NA | LOW | NA | NA | NA | NA | 159 | 159 | 0 | 0 | 202 | 159 | 42 | 20.8 |
| Chr3_p22.1 | 42916322 | G | A | SILENT | **CYP8B1** | cytochrome P450 family 8 subfamily B member 1 | c.987C>T | CYP8B1:NM_004391:exon1:c.C987T:p.F329F | NA | NA | NA | NA | NA | NA | NA | NA | LOW | NA | NA | NA | 0.002 | 118 | 117 | 0 | 0 | 142 | 119 | 23 | 16.2 |
| Chr3_p22.2 | 38420664 | A | G | INTRONIC | **XYLB** | xylulokinase | c.1195-73A>G | NM_005108.3 | NA | NA | NA | NA | NA | NA | NA | NA | MODIFIER | NA | NA | NA | NA | 147 | 147 | 0 | 0 | 202 | 149 | 52 | 25.7 |
| Chr3_p22.2 | 39227903 | C | A | MISSENSE | **XIRP1** | xin actin binding repeat containing 1 | c.3034G>T,c.3034G>T | XIRP1:NM_001198621:exon2:c.G3034T:p.A1012S,XIRP1:NM_194293:exon2:c.G3034T:p.A1012S | NA | NA | NA | NA | NA | D | N | N | MODERATE | T | T | T | 0.011 | 130 | 130 | 0 | 0 | 168 | 140 | 27 | 16.1 |
| Chr3_p25.3 | 9691988 | G | T | INTRONIC | **MTMR14** | myotubularin related protein 14 | c.159+562G>T,c.159+562G>T,c.159+562G>T | NM_001077525.2 | NA | NA | NA | NA | NA | NA | NA | NA | MODIFIER | NA | NA | NA | NA | 174 | 171 | 2 | 1.15 | 195 | 100 | 93 | 47.7 |

**Jain *et al.* , 2019. MAP2K1 is a potential therapeutic target in erlotinib resistant head and neck squamous cell carcinoma**
**Supplementary Table S2: List of single nucleotide variants identified in SCC-R cells.**

| Map Location | Genomic position | Reference allele | Alternate allele | Mutation type | Gene symbol | Protein | Nucleotide change | Description | OncoMD | COSMIC | dbSNP | ExAC | 1000G | SIFT | CONDEL | LRT | VEP | FATHMM | METASVM | METALR | PhastCons7 way | DP in SCC-S | Reference read in SCC-S | Alternative read in SCC-S | Alternate allele frequency in SCC-S (%) | DP in SCC-R | Reference read in SCC-R | Alternate read in SCC-R | Alternate allele frequency in SCC-R (%) |
|---|---|---|---|---|---|---|---|---|---|---|---|---|---|---|---|---|---|---|---|---|---|---|---|---|---|---|---|---|---|
| Chr3_p26.1 | 4732856 | T | C | MISSENSE | **ITPR1** | inositol 1,4,5-trisphosphate receptor type 1 | c.3830T>C,c.3785T>C,c.3812T>C | ITPR1:NM_001168272:exon31:c.T3812C:p.I1271T,ITPR1:NM_002222:exon31:c.T3785C:p.I1262T,ITPR1:NM_001099952:exon32:c.T3830C:p.I1277T | NA | NA | NA | NA | NA | D | D | D | MODERATE | D | D | D | 1 | 179 | 179 | 0 | 0 | 172 | 141 | 31 | 18.0 |
| Chr3_p26.3 | 407640 | A | C | SILENT | **CHL1** | cell adhesion molecule L1 like | c.1593A>C | CHL1:NM_001253388:exon13:c.A1593C:p.T531T,CHL1:NM_001253387:exon14:c.A1545C:p.T515T,CHL1:NM_006614:exon15:c.A1593C:p.T531T | NA | NA | NA | NA | NA | NA | NA | NA | LOW | NA | NA | NA | NA | 45 | 45 | 0 | 0 | 47 | 25 | 22 | 46.8 |
| Chr3_q11.2 | 97661211 | C | T | SILENT | **CRYBG3** | crystallin beta-gamma domain containing 3 | c.2904C>T | CRYBG3:NM_153605:exon21:c.C8748T:p.F2916F | NA | NA | NA | NA | NA | NA | NA | NA | LOW | NA | NA | NA | 0.845 | 153 | 150 | 0 | 0 | 234 | 186 | 47 | 20.1 |
| Chr3_q11.2 | 97661211 | C | T | 3UTR | **MINA** | ribosomal oxygenase 2 | c.*2817G>A | CRYBG3:NM_153605:exon21:c.C8748T:p.F2916F | NA | NA | NA | NA | NA | NA | NA | NA | MODIFIER | NA | NA | NA | NA | 153 | 150 | 0 | 0 | 234 | 186 | 47 | 20.1 |
| Chr3_q12.2 | 100548526 | C | A | INTRONIC | **ABI3BP** | ABI family member 3 binding protein | c.1597+16690G>T | NM_015429.3 | NA | NA | NA | NA | NA | NA | NA | NA | MODIFIER | NA | NA | NA | NA | 240 | 239 | 0 | 0 | 292 | 236 | 56 | 19.2 |
| Chr3_q12.2 | 100557116 | G | C | INTRONIC | **ABI3BP** | ABI family member 3 binding protein | c.1597+8100C>G | NM_015429.3 | NA | NA | NA | NA | NA | NA | NA | NA | MODIFIER | NA | NA | NA | NA | 267 | 267 | 0 | 0 | 326 | 269 | 57 | 17.5 |
| Chr3_q13.13 | 109027122 | G | C | MISSENSE | **DPPA2** | developmental pluripotency associated 2 | c.415C>G | DPPA2:NM_138815:exon6:c.C415G:p.Q139E | NA | NA | NA | NA | NA | T | N | N | MODERATE | T | T | T | 0.001 | 163 | 163 | 0 | 0 | 222 | 209 | 13 | 5.9 |
| Chr3_q21.1-_q21.2 | 124377377 | G | A | INTRONIC | **KALRN** | kalirin RhoGEF kinase | c.1029+13G>A,c.6120+13G>A | NM_001024660.3 | NA | NA | NA | NA | NA | NA | NA | NA | MODIFIER | NA | NA | NA | NA | 60 | 60 | 0 | 0 | 99 | 81 | 18 | 18.2 |
| Chr3_q21.3 | 126218889 | C | A | INTRONIC | **UROC1** | urocanate hydratase 1 | c.1423+11G>T,c.1243+11G>T | NM_144639.2 | NA | NA | NA | NA | NA | NA | NA | NA | MODIFIER | NA | NA | NA | NA | 84 | 84 | 0 | 0 | 112 | 79 | 33 | 29.5 |
| Chr3_q22.1 | 130189863 | T | C | INTRONIC | **COL6A5** | collagen type VI alpha 5 chain | c.7574+52T>C,c.7574+52T>C | NM_001278298.1 | chr3*130189863*130189863*T*C*1*1*Breast_cancer | NA | NA | NA | NA | NA | NA | NA | MODIFIER | NA | NA | NA | NA | 91 | 91 | 0 | 0 | 84 | 67 | 16 | 19.1 |
| Chr3_q22.1 | 130190590 | C | G | MISSENSE | **COL6A5** | collagen type VI alpha 5 chain | c.7639C>G | COL6A5:NM_001278298:exon40:c.C7639G:p.Q2547E | NA | NA | NA | NA | NA | D_lc | N | NA | MODERATE | NA | NA | NA | NA | 110 | 109 | 0 | 0 | 127 | 91 | 36 | 28.4 |
| Chr3_q22.1 | 130190590 | C | G | INTRONIC | **COL6A5** | collagen type VI alpha 5 chain | c.7574+779C>G | COL6A5:NM_001278298:exon40:c.C7639G:p.Q2547E | NA | NA | NA | NA | NA | NA | NA | NA | MODIFIER | NA | NA | NA | NA | 110 | 109 | 0 | 0 | 127 | 91 | 36 | 28.4 |
| Chr3_q22.1 | 130300696 | T | G | MISSENSE | **COL6A6** | collagen type VI alpha 6 chain | c.3839T>G | COL6A6:NM_001102608:exon8:c.T3839G:p.L1280R | NA | NA | NA | NA | NA | D | D | NA | MODERATE | NA | NA | NA | NA | 131 | 131 | 0 | 0 | 175 | 133 | 41 | 23.4 |
| Chr3_q22.1 | 131274443 | T | C | INTRONIC | **CPNE4** | copine 4 | c.1062-48A>G | NM_130808.1 | NA | NA | rs771283890 | 4.05E-05 | NA | NA | NA | NA | MODIFIER | NA | NA | NA | NA | 62 | 62 | 0 | 0 | 76 | 53 | 20 | 26.3 |
| Chr3_q23 | 139079940 | G | T | MISSENSE | **COPB2** | coatomer protein complex subunit beta 2 | c.2193C>A | COPB2:NM_004766:exon17:c.C2193A:p.S731R | NA | NA | NA | NA | NA | D | D | D | MODERATE | T | T | D | 1 | 55 | 55 | 0 | 0 | 97 | 83 | 14 | 14.4 |
| Chr3_q25.33 | 160167498 | G | C | INTRONIC-SS-DNR-PRX | **TRIM59** | tripartite motif containing 59 | c.-74+7C>G | NM_173084.2 | NA | NA | NA | NA | NA | NA | NA | NA | LOW | NA | NA | NA | NA | 131 | 130 | 0 | 0 | 194 | 165 | 29 | 15.0 |
| Chr3_q26.2 | 169801776 | T | A | MISSENSE | **GPR160** | G protein-coupled receptor 160 | c.16T>A | GPR160:NM_014373:exon4:c.T16A:p.S6T | NA | NA | NA | NA | NA | T | N | N | MODERATE | 0 | T | T | 0.532 | 52 | 50 | 0 | 0 | 95 | 89 | 6 | 6.3 |
| Chr3_q27.1 | 184100313 | C | A | MISSENSE | **CHRD** | chordin | c.836C>A | CHRD:NM_001304472:exon7:c.C836A:p.A279D,CHRD:NM_003741:exon7:c.C836A:p.A279D | NA | NA | NA | NA | NA | T | D | D | MODERATE | T | T | T | 0.186 | 144 | 143 | 0 | 0 | 201 | 150 | 51 | 25.4 |
| Chr3_q28 | 191000015 | C | A | 5UTR | **UTS2B** | urotensin 2B | c.-37G>T | NM_198152.3 | NA | NA | NA | NA | NA | NA | NA | NA | MODIFIER | NA | NA | NA | NA | 83 | 83 | 0 | 0 | 120 | 88 | 31 | 25.8 |
| Chr3_q28-_q29 | 191986428 | C | A | INTRONIC | **FGF12** | fibroblast growth factor 12 | c.228+66722G>T,c.414+66722G>T | NM_021032.4 | NA | NA | NA | NA | NA | NA | NA | NA | MODIFIER | NA | NA | NA | NA | 79 | 79 | 0 | 0 | 119 | 113 | 6 | 5.0 |
| Chr3_q28-_q29 | 191986429 | A | G | INTRONIC | **FGF12** | fibroblast growth factor 12 | c.228+66721T>C,c.414+66721T>C | NM_021032.4 | NA | NA | NA | NA | NA | NA | NA | NA | MODIFIER | NA | NA | NA | NA | 78 | 78 | 0 | 0 | 119 | 112 | 6 | 5.0 |
| Chr3_q29 | 193032013 | T | C | INTRONIC | **ATP13A5** | ATPase 13A5 | c.2159-31A>G | NM_198505.2 | NA | NA | NA | NA | NA | NA | NA | NA | MODIFIER | NA | NA | NA | NA | 99 | 98 | 0 | 0 | 110 | 91 | 19 | 17.3 |
| Chr3_q29 | 193069024 | C | A | INTRONIC | **ATP13A5** | ATPase 13A5 | c.607-34G>T | NM_198505.2 | NA | NA | NA | NA | NA | NA | NA | NA | MODIFIER | NA | NA | NA | NA | 197 | 194 | 1 | 0.51 | 225 | 212 | 10 | 4.4 |
| Chr3_q29 | 193376711 | C | T | SILENT | **OPA1** | OPA1, mitochondrial dynamin like GTPase | c.2205C>T,c.2259C>T,c.2313C>T,c.2256C>T,c.2202C>T,c.2367C>T | OPA1:NM_130831:exon21:c.C2094T:p.S698S,OPA1:NM_015560:exon22:c.C2202T:p.S734S,OPA1:NM_130832:exon22:c.C2148T:p.S716S,OPA1:NM_130833:exon22:c.C2205T:p.S735S,OPA1:NM_130834:exon23:c.C2256T:p.S752S,OPA1:NM_130835:exon23:c.C2259T:p.S753S,OPA1:NM_130836:exon23:c.C2313T:p.S771S,OPA1:NM_130837:exon24:c.C2367T:p.S789S | NA | NA | NA | NA | NA | NA | NA | NA | LOW | NA | NA | NA | NA | 198 | 198 | 0 | 0 | 269 | 203 | 65 | 24.2 |
| Chr4_p12-_p11 | 48172218 | C | T | INTRONIC | **TEC** | tec protein tyrosine kinase | c.454+47G>A | NM_003215.2 | NA | NA | rs2271174 | 4.22E-01 | 5.21E-01 | NA | NA | NA | MODIFIER | NA | NA | NA | NA | 23 | 23 | 0 | 0 | 53 | 43 | 10 | 18.9 |
| Chr4_p12-_p11 | 48172266 | G | A | SILENT-SS-PRX | **TEC** | tec protein tyrosine kinase | c.453C>T | TEC:NM_003215:exon5:c.C453T:p.S151S | NA | NA | rs2271173 | 4.23E-01 | 5.21E-01 | NA | NA | NA | LOW | NA | NA | NA | 0.995 | 36 | 36 | 0 | 0 | 57 | 46 | 11 | 19.3 |
| Chr4_p13 | 44177046 | T | C | MISSENSE | **KCTD8** | potassium channel tetramerization domain containing 8 | c.1183A>G | KCTD8:NM_198353:exon2:c.A1183G:p.K395E | NA | NA | NA | NA | NA | D | D | D | MODERATE | T | T | T | 0.994 | 240 | 236 | 1 | 0.42 | 190 | 124 | 66 | 34.7 |
| Chr4_p15.2 | 22415026 | G | A | INTRONIC | **GPR125** | adhesion G protein-coupled receptor A3 | c.2024-13C>T | NM_145290.3 | NA | NA | rs9790838 | 2.47E-02 | 2.60E-02 | NA | NA | NA | MODIFIER | NA | NA | NA | NA | 45 | 44 | 1 | 2.22 | 67 | 50 | 17 | 25.4 |
| Chr4_p15.2 | 25677688 | C | A | INTRONIC | **SLC34A2** | solute carrier family 34 member 2 | c.1456-69C>A,c.1459-69C>A | NM_006424.2,NM_001177998.1 | NA | NA | NA | NA | NA | NA | NA | NA | MODIFIER | NA | NA | NA | NA | 109 | 109 | 0 | 0 | 131 | 102 | 29 | 22.1 |
| Chr4_p15.2 | 25930071 | G | C | INTRONIC | **SMIM20** | small integral membrane protein 20 | c.166+80G>C | NM_001145432.1 | NA | NA | rs2309292 | NA | 1.76E-01 | NA | NA | NA | MODIFIER | NA | NA | NA | NA | 63 | 60 | 2 | 3.17 | 103 | 69 | 29 | 28.2 |
| Chr4_p15.31 | 20525361 | C | A | INTRONIC | **SLIT2** | slit guidance ligand 2 | c.1131-22C>A | NM_004787.1 | NA | NA | NA | NA | NA | NA | NA | NA | MODIFIER | NA | NA | NA | NA | 64 | 64 | 0 | 0 | 91 | 68 | 23 | 25.3 |
| Chr4_p15.32 | 15018905 | A | G | INTRONIC-SS-DNR-PRX | **CPEB2** | cytoplasmic polyadenylation element binding protein 2 | c.2125+3A>G,c.733+3A>G,c.2125+3A>G,c.2044+3A>G | NM_001177381.1,NM_001177383.1 | NA | NA | NA | NA | NA | NA | NA | NA | LOW | NA | NA | NA | NA | 32 | 32 | 0 | 0 | 47 | 30 | 15 | 31.9 |
| Chr4_p15.33 | 13583838 | C | T | INTRONIC | **BOD1L1** | biorientation of chromosomes in cell division 1 like 1 | c.8592+23G>A | NM_148894.2 | NA | NA | rs17806907 | 1.52E-01 | 1.40E-01 | NA | NA | NA | MODIFIER | NA | NA | NA | NA | 44 | 44 | 0 | 0 | 73 | 53 | 20 | 27.4 |
| Chr4_p15.33 | 13615174 | G | A | MISSENSE | **BOD1L1** | biorientation of chromosomes in cell division 1 like 1 | c.1286C>T | BOD1L1:NM_148894:exon5:c.C1286T:p.T429M | NA | NA | rs2035820 | 1.45E-01 | 1.37E-01 | NA | NA | N | MODERATE | T | T | T | 0.963 | 147 | 142 | 4 | 2.72 | 232 | 155 | 77 | 33.2 |
| Chr4_p16.1 | 6292915 | A | G | INTRONIC-SS-ACR-PRX | **WFS1** | wolframin ER transmembrane glycoprotein | c.461-9A>G | NM_001145853.1,NM_006005.3 | NA | NA | rs10010131 | 6.55E-01 | 7.29E-01 | NA | NA | NA | MODIFIER | NA | NA | NA | NA | 97 | 94 | 3 | 3.09 | 111 | 76 | 34 | 30.6 |
| Chr4_p16.1 | 6325086 | G | A | SILENT | **PPP2R2C** | protein phosphatase 2 regulatory subunit Bgamma | c.1236C>T,c.1266C>T,c.1266C>T,c.1287C>T | PPP2R2C:NM_020416:exon9:c.C1287T:p.I429I,PPP2R2C:NM_181876:exon9:c.C1287T:p.I429I,PPP2R2C:NM_001206994:exon10:c.C1266T:p.I422I,PPP2R2C:NM_001206995:exon10:c.C1266T:p.I422I,PPP2R2C:NM_001206996:exon10:c.C1236T:p.I412I | NA | NA | rs3796403 | 6.32E-01 | 6.54E-01 | NA | NA | NA | LOW | NA | NA | NA | 0.993 | 80 | 77 | 3 | 3.75 | 91 | 58 | 33 | 36.3 |
| Chr4_p16.1 | 7726847 | A | G | INTRONIC | **SORCS2** | sortilin related VPS10 domain containing receptor 2 | c.2612-34A>G | NM_020777.2 | NA | NA | rs2285777 | 3.78E-01 | 3.93E-01 | NA | NA | NA | MODIFIER | NA | NA | NA | NA | 160 | 156 | 4 | 2.5 | 180 | 126 | 53 | 29.4 |
| Chr4_p16.1 | 7783163 | G | A | INTRONIC | **AFAP1** | actin filament associated protein 1 | c.1531-2560C>T | AFAP1:NM_001134647:exon13:c.C1722T:p.S574S | NA | NA | rs28542374 | 6.27E-01 | 6.25E-01 | NA | NA | NA | MODIFIER | NA | NA | NA | NA | 51 | 49 | 2 | 3.92 | 87 | 53 | 34 | 39.1 |
| Chr4_p16.1 | 7783163 | G | A | SILENT | **AFAP1** | actin filament associated protein 1 | c.1722C>T | AFAP1:NM_001134647:exon13:c.C1722T:p.S574S | NA | NA | rs28542374 | 6.27E-01 | 6.25E-01 | NA | NA | NA | LOW | NA | NA | NA | NA | 51 | 49 | 2 | 3.92 | 87 | 53 | 34 | 39.1 |
| Chr4_p16.1 | 8582742 | C | T | SILENT | **GPR78** | G protein-coupled receptor 78 | c.33C>T | GPR78:NM_080819:exon1:c.C33T:p.L11L | NA | NA | rs17844776 | 2.95E-01 | 1.39E-01 | NA | NA | NA | LOW | NA | NA | NA | NA | 37 | 37 | 0 | 0 | 47 | 34 | 12 | 25.5 |
| Chr4_p16.1 | 10027643 | A | G | INTRONIC | **SLC2A9** | solute carrier family 2 member 9 | c.-40-13T>C | NM_001001290.1 | NA | NA | rs6449237 | NA | 2.67E-01 | NA | NA | NA | MODIFIER | NA | NA | NA | NA | 48 | 47 | 1 | 2.08 | 63 | 46 | 17 | 27.0 |
| Chr4_p16.1 | 10094783 | T | C | INTRONIC | **WDR1** | WD repeat domain 1 | c.559-4418A>G,c.139-4418A>G | NM_005112.4 | NA | NA | rs3822245 | NA | 5.82E-01 | NA | NA | NA | MODIFIER | NA | NA | NA | NA | 84 | 81 | 3 | 3.57 | 115 | 77 | 38 | 33.0 |
| Chr4_p16.2 | 5747072 | C | T | INTRONIC-SS-DNR-PRX | **EVC** | EvC ciliary complex subunit 1 | c.939+4C>T | NM_153717.2 | NA | NA | rs2286343 | 3.73E-01 | 4.09E-01 | NA | NA | NA | LOW | NA | NA | NA | NA | 71 | 69 | 1 | 1.41 | 96 | 63 | 33 | 34.4 |
| Chr4_p16.2 | 5838513 | C | G | SILENT | **CRMP1** | collapsin response mediator protein 1 | c.1083G>C,c.1431G>C,c.1089G>C | CRMP1:NM_001014809:exon10:c.G1431C:p.T477T,CRMP1:NM_001288661:exon10:c.G1083C:p.T361T,CRMP1:NM_001288662:exon10:c.G1071C:p.T357T,CRMP1:NM_001313:exon10:c.G1089C:p.T363T | chr4*5838513*5838513*G*C*1*1*Breast_cancer | chr4*5838513*5838513*COSM447986*C*G*1*breast*1 | rs12331 | 4.79E-01 | 5.89E-01 | NA | NA | NA | LOW | NA | NA | NA | NA | 114 | 113 | 1 | 0.88 | 178 | 131 | 47 | 26.4 |
| Chr4_p16.3 | 1165131 | C | T | MISSENSE | **SPON2** | spondin 2 | c.364G>A,c.364G>A | SPON2:NM_012445:exon3:c.G364A:p.A122T,SPON2:NM_001128325:exon4:c.G364A:p.A122T,SPON2:NM_001199021:exon5:c.G364A:p.A122T | NA | NA | rs193114328 | 1.23E-02 | 4.59E-03 | T | N | N | MODERATE | T | T | T | 0.385 | 37 | 36 | 1 | 2.7 | 55 | 34 | 21 | 38.2 |
| Chr4_p16.3 | 2513615 | G | C | INTRONIC | **RNF4** | ring finger protein 4 | c.205-68G>C,c.205-68G>C,c.205-68G>C | NM_001185009.2 | NA | NA | rs1770823 | NA | 2.83E-01 | NA | NA | NA | MODIFIER | NA | NA | NA | NA | 56 | 55 | 1 | 1.79 | 68 | 49 | 19 | 27.9 |
| Chr4_p16.3 | 2746307 | A | C | INTRONIC | **TNIP2** | TNFAIP3 interacting protein 2 | c.907-74T>G,c.586-74T>G | NM_001161527.1 | chr4*2746307*2746307*T*G*1*1*Leukemia | NA | rs11728346 | NA | 8.05E-01 | NA | NA | NA | MODIFIER | NA | NA | NA | NA | 91 | 88 | 3 | 3.3 | 150 | 109 | 39 | 26.0 |
| Chr4_p16.3 | 2927788 | C | G | MISSENSE | **ADD1** | adducin 1 | c.1903C>G,c.1810C>G,c.1903C>G,c.1903C>G | ADD1:NM_001119:exon14:c.C1810G:p.Q604E,ADD1:NM_014189:exon14:c.C1903G:p.Q635E,ADD1:NM_014190:exon14:c.C1810G:p.Q604E,ADD1:NM_176801:exon14:c.C1903G:p.Q635E,ADD1:NM_001286645:exon16:c.C1903G:p.Q635E | NA | NA | rs201305164 | 5.81E-05 | NA | T | N | N | MODERATE | T | T | T | 0.791 | 89 | 87 | 2 | 2.25 | 109 | 68 | 41 | 37.6 |
| Chr4_p16.3 | 3162056 | C | T | SILENT | **HTT** | huntingtin | c.3801C>T | HTT:NM_002111:exon29:c.C3801T:p.L1267L | NA | chr4*3162056*3162056*COSM447790*C*T*1*breast*1 | rs363099 | 2.59E-01 | 2.14E-01 | NA | NA | NA | LOW | NA | NA | NA | NA | 67 | 64 | 3 | 4.48 | 115 | 69 | 46 | 40.0 |
| Chr4_p16.3 | 3445745 | C | T | INTRONIC | **HGFAC** | HGF activator | c.476-21C>T | NM_001528.2 | NA | NA | rs4690097 | 7.18E-01 | 7.32E-01 | NA | NA | NA | MODIFIER | NA | NA | NA | NA | 40 | 40 | 0 | 0 | 58 | 42 | 15 | 25.9 |
| Chr4_p16.3 | 4199634 | A | T | MISSENSE | **OTOP1** | otopetrin 1 | c.927T>A | OTOP1:NM_177998:exon5:c.T927A:p.D309E | NA | NA | rs2916414 | 6.84E-01 | 6.62E-01 | T | N | N | MODERATE | T | T | T | 0.021 | 73 | 71 | 2 | 2.74 | 98 | 60 | 37 | 37.8 |

**Supplementary Table S2: List of single nucleotide variants identified in SCC-R cells.**

| Map Location | Genomic position | Reference allele | Alternate allele | Mutation type | Gene symbol | Protein | Nucleotide change | Description | OncoMD | COSMIC | dbSNP | ExAC | 1000G | SIFT | CONDEL | LRT | VEP | FATHMM | METASVM | METALR | PhastCons7 way | DP in SCC-S | Reference read in SCC-S | Alternative read in SCC-S | Alternate allele frequency in SCC-S (%) | DP in SCC-R | Reference read in SCC-R | Alternate read in SCC-R | Alternate allele frequency in SCC-R (%) |
|---|---|---|---|---|---|---|---|---|---|---|---|---|---|---|---|---|---|---|---|---|---|---|---|---|---|---|---|---|---|
| Chr4_q23 | 100261739 | A | T | SILENT | **ADH1C** | alcohol dehydrogenase 1C (class I), gamma polypeptide | c.957T>A | NM_000669.3 | NA | NA | NA | NA | NA | NA | NA | NA | LOW | NA | NA | NA | NA | 35 | 34 | 0 | 0 | 49 | 29 | 19 | 38.8 |
| Chr4_q26 | 115791999 | C | A | MISSENSE | **NDST4** | N-deacetylase and N-sulfotransferase 4 | c.1644G>T | NDST4:NM_022569:exon7:c.G1644T:p.L548F | NA | NA | NA | NA | NA | D | D | D | MODERATE | T | T | T | 0.998 | 96 | 96 | 0 | 0 | 111 | 59 | 52 | 46.9 |
| Chr4_q27 | 122059720 | C | T | INTRONIC | **TNIP3** | TNFAIP3 interacting protein 3 | c.1116+3251G>A,c.946+59G>A,c.1095+3251G>A | NM_001128843.2 | NA | NA | NA | NA | NA | NA | NA | NA | MODIFIER | NA | NA | NA | NA | 99 | 98 | 0 | 0 | 92 | 45 | 47 | 51.1 |
| Chr4_q31.3 | 154553890 | C | A | MISSENSE | **KIAA0922** | transmembrane 131 like | c.4225C>A,c.4228C>A | KIAA0922:NM_001131007:exon32:c.C4228A:p.L1410M,KIAA0922:NM_015196:exon32:c.C4225A:p.L1409M | NA | NA | NA | NA | NA | T | D | N | MODERATE | T | T | T | 0.852 | 104 | 104 | 0 | 0 | 119 | 111 | 7 | 5.9 |
| Chr4_q34.1 | 175429854 | A | T | SILENT | **HPGD** | 15-hydroxyprostaglandin dehydrogenase | c.414T>A,c.414T>A | HPGD:NM_001256307:exon3:c.T51A:p.S17S,HPGD:NM_000860:exon4:c.T414A:p.S138S,HPGD:NM_001145816:exon4:c.T414A:p.S138S,HPGD:NM_001256301:exon4:c.T51A:p.S17S,HPGD:NM_001256305:exon4:c.T414A:p.S138S | NA | NA | NA | NA | NA | NA | NA | NA | LOW | NA | NA | NA | NA | 28 | 28 | 0 | 0 | 33 | 20 | 13 | 39.4 |
| Chr4_q34.1 | 175429854 | A | T | INTRONIC | **HPGD** | 15-hydroxyprostaglandin dehydrogenase | c.218-13079T>A | HPGD:NM_001256307:exon3:c.T51A:p.S17S,HPGD:NM_000860:exon4:c.T414A:p.S138S,HPGD:NM_001145816:exon4:c.T414A:p.S138S,HPGD:NM_001256301:exon4:c.T51A:p.S17S,HPGD:NM_001256305:exon4:c.T414A:p.S138S | NA | NA | NA | NA | NA | NA | NA | NA | MODIFIER | NA | NA | NA | NA | 28 | 28 | 0 | 0 | 33 | 20 | 13 | 39.4 |
| Chr4_q34.1 | 175709988 | T | A | NONSENSE | **GLRA3** | glycine receptor alpha 3 | c.178A>T,c.178A>T | GLRA3:NM_001042543:exon2:c.A178T:p.R60X,GLRA3:NM_006529:exon2:c.A178T:p.R60X | NA | NA | NA | NA | NA | NA | NA | NA | HIGH | NA | NA | NA | 1 | 93 | 93 | 0 | 0 | 113 | 63 | 48 | 42.5 |
| Chr4_q35.1 | 184190278 | C | G | MISSENSE | **WWC2** | WW and C2 domain containing 2 | c.2362C>G | WWC2:NM_024949:exon15:c.C2362G:p.L788V | NA | NA | NA | NA | NA | T | N | N | MODERATE | T | T | T | 0.781 | 158 | 158 | 0 | 0 | 172 | 88 | 83 | 48.3 |
| Chr4_q35.1 | 185940952 | C | G | MISSENSE | **HELT** | helt bHLH transcription factor | c.439C>G | HELT:NM_001300781:exon3:c.C184G:p.L62V,HELT:NM_001300782:exon3:c.C184G:p.L62V | NA | NA | rs1078461 | 1.10E-01 | 1.12E-01 | D | D | D | MODERATE | D | T | T | 0.996 | 48 | 48 | 0 | 0 | 61 | 56 | 5 | 8.2 |
| Chr4_q35.1 | 186347055 | G | A | 5UTR | **UFSP2** | UFM1 specific peptidase 2 | c.-33C>T | NM_018359.3 | NA | NA | rs2289720 | 6.92E-01 | 5.70E-01 | NA | NA | NA | MODIFIER | NA | NA | NA | NA | 98 | 98 | 0 | 0 | 150 | 143 | 7 | 4.7 |
| Chr5_p12 | 44809582 | C | A | MISSENSE | **MRPS30** | mitochondrial ribosomal protein S30 | c.518C>A | MRPS30:NM_016640:exon1:c.C518A:p.S173Y | chr5*44809582*44809582*C*A*1*1*Endometrial_cancer | chr5*44809582*44809582*COSM1437697*C*A*2*endometrium,large_intestine*1,1\|chr5*44809582*44809582*COSM1068464*C*A*2*endometrium,large_intestine*1,1 | NA | NA | NA | T | N | N | MODERATE | T | T | T | 0.024 | 239 | 239 | 0 | 0 | 273 | 227 | 45 | 16.5 |
| Chr5_p13.2 | 33954436 | A | C | INTRONIC | **SLC45A2** | solute carrier family 45 member 2 | c.1032+30T>G,c.1032+30T>G | NM_016180.3 | NA | NA | NA | NA | NA | NA | NA | NA | MODIFIER | NA | NA | NA | NA | 174 | 174 | 0 | 0 | 188 | 136 | 52 | 27.7 |
| Chr5_p14.3 | 19571687 | C | G | INTRONIC-SS-DNR | **CDH18** | cadherin 18 | c.1253+1G>C | NM_004934.3,NM_001167667.1 | NA | NA | NA | NA | NA | NA | NA | NA | HIGH | NA | NA | NA | NA | 123 | 122 | 0 | 0 | 147 | 103 | 44 | 29.9 |
| Chr5_p15.2 | 11346504 | A | C | MISSENSE | **CTNND2** | catenin delta 2 | c.1608T>G | CTNND2:NM_001288716:exon6:c.T597G:p.D199E,CTNND2:NM_001288715:exon8:c.T1335G:p.D445E,CTNND2:NM_001288717:exon8:c.T309G:p.D103E,CTNND2:NM_001332:exon9:c.T1608G:p.D536E | NA | NA | NA | NA | NA | T | D | D | MODERATE | T | T | T | 0.998 | 380 | 378 | 0 | 0 | 428 | 318 | 110 | 25.7 |
| Chr5_p15.2 | 13752304 | A | G | MISSENSE | **DNAH5** | dynein axonemal heavy chain 5 | c.10967T>C | DNAH5:NM_001369:exon64:c.T10967C:p.L3656P | NA | NA | NA | NA | NA | NA | NA | D | MODERATE | T | T | T | 0.955 | 196 | 196 | 0 | 0 | 194 | 156 | 36 | 18.6 |
| Chr5_p15.31 | 7706890 | C | T | SILENT | **ADCY2** | adenylate cyclase 2 | c.1143C>T | ADCY2:NM_020546:exon8:c.C1143T:p.N381N | chr5*7706890*7706890*C*T*5*3*Renal_cancer | chr5*7706890*7706890*COSM3366163*C*T*2*kidney*2\|chr5*7706890*7706890*COSM3366164*C*T*2*kidney*2 | NA | NA | NA | NA | NA | NA | LOW | NA | NA | NA | 1 | 176 | 175 | 0 | 0 | 188 | 142 | 45 | 23.9 |
| Chr5_p15.31 | 7743740 | A | G | INTRONIC | **ADCY2** | adenylate cyclase 2 | c.1872-41A>G | NM_020546.2 | NA | NA | NA | NA | NA | NA | NA | NA | MODIFIER | NA | NA | NA | NA | 310 | 310 | 0 | 0 | 252 | 243 | 8 | 3.2 |
| Chr5_p15.32 | 5146204 | C | A | INTRONIC | **ADAMTS16** | ADAM metallopeptidase with thrombospondin type 1 motif 16 | c.176-39C>A | NM_139056.2 | NA | NA | NA | NA | NA | NA | NA | NA | MODIFIER | NA | NA | NA | NA | 130 | 129 | 0 | 0 | 121 | 92 | 26 | 21.5 |
| Chr5_p15.33 | 864721 | C | G | INTRONIC | **BRD9** | bromodomain containing 9 | c.1694-38G>C,c.1535-38G>C | NM_001009877.2 | NA | NA | NA | NA | NA | NA | NA | NA | MODIFIER | NA | NA | NA | NA | 212 | 212 | 0 | 0 | 196 | 160 | 36 | 18.4 |
| Chr5_p15.33 | 1470900 | G | A | INTRONIC | **LPCAT1** | lysophosphatidylcholine acyltransferase 1 | c.1278+41C>T | NM_024830.3 | NA | NA | rs2277005 | 3.64E-01 | 2.58E-01 | NA | NA | NA | MODIFIER | NA | NA | NA | NA | 183 | 182 | 0 | 0 | 191 | 182 | 8 | 4.2 |
| Chr5_q14.1 | 79855185 | T | C | SILENT | **ANKRD34B** | ankyrin repeat domain 34B | c.654A>G | ANKRD34B:NM_001004441:exon5:c.A654G:p.G218G | NA | NA | NA | NA | NA | NA | NA | NA | LOW | NA | NA | NA | NA | 140 | 140 | 0 | 0 | 116 | 57 | 59 | 50.9 |
| Chr5_q14.3 | 89988598 | G | A | SILENT | **GPR98** | adhesion G protein-coupled receptor V1 | c.7128G>A | ADGRV1:NM_032119:exon32:c.G7128A:p.R2376R | NA | NA | NA | NA | NA | NA | NA | NA | LOW | NA | NA | NA | 0.187 | 144 | 144 | 0 | 0 | 102 | 53 | 48 | 47.1 |
| Chr5_q15 | 93856021 | T | C | MISSENSE | **KIAA0825** | KIAA0825 | c.902A>G,c.902A>G | KIAA0825:NM_001145678:exon5:c.A902G:p.E301G,KIAA0825:NM_173665:exon5:c.A902G:p.E301G | NA | NA | NA | NA | NA | D | D | N | MODERATE | D | T | D | 0.999 | 173 | 173 | 0 | 0 | 181 | 105 | 76 | 42.0 |
| Chr5_q23.2 | 121779262 | C | T | INTRONIC | **SNCAIP** | synuclein alpha interacting protein | c.1423-996C>T,c.325-996C>T | NM_001242935.1 | NA | NA | rs2737094 | 1.12E-01 | 9.42E-02 | NA | NA | NA | MODIFIER | NA | NA | NA | NA | 84 | 82 | 0 | 0 | 50 | 46 | 4 | 8.0 |
| Chr5_q31.2 | 136974786 | G | T | MISSENSE | **KLHL3** | kelch like family member 3 | c.829C>A,c.1075C>A,c.979C>A | KLHL3:NM_001257195:exon8:c.C829A:p.L277M,KLHL3:NM_001257194:exon10:c.C979A:p.L327M,KLHL3:NM_017415:exon10:c.C1075A:p.L359M | NA | NA | NA | 8.45E-06 | NA | D | D | D | MODERATE | T | D | D | 0.919 | 224 | 224 | 0 | 0 | 150 | 141 | 7 | 4.7 |
| Chr5_q32 | 145522560 | T | C | SILENT | **LARS** | leucyl-tRNA synthetase | c.1965A>G | LARS:NM_020117:exon20:c.A1965G:p.L655L | NA | NA | NA | NA | NA | NA | NA | NA | LOW | NA | NA | NA | 0.998 | 188 | 188 | 0 | 0 | 201 | 76 | 125 | 62.2 |
| Chr5_q32 | 149512366 | G | T | SILENT | **PDGFRB** | platelet derived growth factor receptor beta | c.1074C>A | PDGFRB:NM_002609:exon7:c.C1074A:p.G358G | NA | NA | NA | NA | NA | NA | NA | NA | LOW | NA | NA | NA | NA | 58 | 57 | 0 | 0 | 59 | 40 | 19 | 32.2 |
| Chr5_q32 | 149677760 | G | T | MISSENSE | **ARSI** | arylsulfatase family member I | c.727C>A | ARSI:NM_001012301:exon2:c.C727A:p.Q243K | NA | NA | NA | NA | NA | D | D | D | MODERATE | D | D | D | 0.998 | 195 | 194 | 0 | 0 | 208 | 79 | 128 | 61.5 |
| Chr5_q35.1 | 169533606 | T | C | INTRONIC | **FOXI1** | forkhead box I1 | c.574+71T>C | NM_012188.4,NM_144769.2 | NA | NA | rs2277945 | NA | 2.61E-01 | NA | NA | NA | MODIFIER | NA | NA | NA | NA | 97 | 95 | 0 | 0 | 73 | 65 | 7 | 9.6 |
| Chr5_q35.1 | 169720364 | C | T | MISSENSE | **LCP2** | lymphocyte cytosolic protein 2 | c.91G>A | LCP2:NM_005565:exon2:c.G91A:p.D31N | NA | NA | NA | NA | NA | D | D | D | MODERATE | D | D | D | 0.992 | 162 | 162 | 0 | 0 | 121 | 86 | 33 | 27.3 |
| Chr5_q35.1 | 171766241 | G | T | MISSENSE | **SH3PXD2B** | SH3 and PX domains 2B | c.1868C>A | SH3PXD2B:NM_001017995:exon13:c.C1868A:p.P623Q | NA | NA | NA | NA | NA | T | N | N | MODERATE | T | T | T | 0.389 | 221 | 221 | 0 | 0 | 186 | 92 | 93 | 50.0 |
| Chr5_q35.1 | 172097020 | C | T | SILENT | **NEURL1B** | neuralized E3 ubiquitin protein ligase 1B | c.264C>T | NEURL1B:NM_001142651:exon2:c.C264T:p.A88A | NA | NA | rs748791908 | 0.00E+00 | NA | NA | NA | NA | LOW | NA | NA | NA | NA | 249 | 249 | 0 | 0 | 211 | 174 | 36 | 17.1 |
| Chr5_q35.3 | 180052963 | G | T | MISSENSE | **FLT4** | fms related tyrosine kinase 4 | c.1327C>A,c.1327C>A | FLT4:NM_002020:exon10:c.C1327A:p.L443I,FLT4:NM_182925:exon10:c.C1327A:p.L443I | NA | NA | NA | NA | NA | D | D | NA | MODERATE | NA | NA | NA | NA | 81 | 81 | 0 | 0 | 64 | 52 | 12 | 18.8 |
| Chr6_p12.1 | 55659160 | A | G | MISSENSE | **BMP5** | bone morphogenetic protein 5 | c.749T>C | BMP5:NM_021073:exon3:c.T749C:p.F250S | NA | NA | NA | NA | NA | D | D | D | MODERATE | T | D | D | 1 | 217 | 216 | 0 | 0 | 234 | 224 | 10 | 4.3 |
| Chr6_p12.1 | 56879143 | A | G | INTRONIC | **BEND6** | BEN domain containing 6 | c.299-788A>G | NM_152731.2 | NA | NA | NA | NA | NA | NA | NA | NA | MODIFIER | NA | NA | NA | NA | 70 | 70 | 0 | 0 | 73 | 47 | 26 | 35.6 |
| Chr6_p12.3 | 49754094 | G | T | SILENT | **PGK2** | phosphoglycerate kinase 2 | c.807C>A | PGK2:NM_138733:exon1:c.C807A:p.I269I | NA | NA | NA | NA | NA | NA | NA | NA | LOW | NA | NA | NA | 0.935 | 308 | 307 | 0 | 0 | 291 | 278 | 10 | 3.4 |
| Chr6_p21.1 | 43227323 | G | T | MISSENSE | **TTBK1** | tau tubulin kinase 1 | c.1303G>T | TTBK1:NM_032538:exon12:c.G1303T:p.V435L | NA | NA | NA | NA | NA | NA | NA | D | MODERATE | T | T | T | 0.994 | 74 | 74 | 0 | 0 | 68 | 56 | 10 | 14.7 |
| Chr6_p21.2 | 39033567 | G | A | MISSENSE | **GLP1R** | glucagon like peptide 1 receptor | c.364G>A | GLP1R:NM_002062:exon4:c.G364A:p.D122N | NA | NA | NA | NA | NA | T | N | N | MODERATE | T | T | T | 0.973 | 165 | 165 | 0 | 0 | 123 | 99 | 23 | 18.7 |
| Chr6_p21.31 | 35565040 | A | G | MISSENSE | **FKBP5** | FK506 binding protein 5 | c.650T>C,c.650T>C,c.650T>C,c.650T>C | FKBP5:NM_001145776:exon6:c.T650C:p.L217S,FKBP5:NM_001145777:exon6:c.T650C:p.L217S,FKBP5:NM_004117:exon6:c.T650C:p.L217S,FKBP5:NM_001145775:exon7:c.T650C:p.L217S | NA | NA | NA | NA | NA | D | D | D | MODERATE | D | D | D | 0.997 | 266 | 266 | 0 | 0 | 312 | 197 | 115 | 36.9 |
| Chr6_p21.33 | 30995226 | C | G | MISSENSE | **MUC22** | mucin 22 | c.2018C>G | MUC22:NM_001198815:exon3:c.C2018G:p.S673C | NA | NA | NA | NA | NA | NA | NA | NA | MODERATE | NA | NA | NA | NA | 773 | 769 | 0 | 0 | 692 | 453 | 231 | 33.4 |
| Chr6_p21.33 | 31732218 | G | T | MISSENSE | **SAPCD1** | suppressor APC domain containing 1 | c.447G>T | SAPCD1:NM_001039651:exon5:c.G447T:p.L149F | NA | NA | NA | NA | NA | T | N | NA | MODERATE | NA | NA | NA | NA | 54 | 54 | 0 | 0 | 62 | 42 | 20 | 32.3 |
| Chr6_p22.1 | 27421058 | T | G | INTRONIC | **ZNF184** | zinc finger protein 184 | c.299-19A>C | NM_007149.2 | NA | NA | NA | NA | NA | NA | NA | NA | MODIFIER | NA | NA | NA | NA | 68 | 68 | 0 | 0 | 68 | 44 | 22 | 32.4 |
| Chr6_p22.2 | 25426848 | A | G | INTRONIC | **LRRC16A** | capping protein regulator and myosin 1 linker 1 | c.249+60A>G | NM_001173977.1,NM_017640.5 | NA | NA | NA | NA | NA | NA | NA | NA | MODIFIER | NA | NA | NA | NA | 141 | 141 | 0 | 0 | 121 | 91 | 30 | 24.8 |
| Chr6_p22.2 | 26234468 | A | C | 3UTR | **HIST1H1D** | histone cluster 1 H1 family member d | c.*28T>G | NM_005320.2 | NA | NA | NA | NA | NA | NA | NA | NA | MODIFIER | NA | NA | NA | NA | 32 | 32 | 0 | 0 | 57 | 45 | 11 | 19.3 |
| Chr6_p22.3 | 16145083 | C | A | INTRONIC | **MYLIP** | myosin regulatory light chain interacting protein | c.828-45C>A | NM_013262.3 | NA | NA | NA | NA | NA | NA | NA | NA | MODIFIER | NA | NA | NA | NA | 176 | 175 | 0 | 0 | 136 | 89 | 46 | 33.8 |
| Chr6_p22.3 | 17772230 | T | G | MISSENSE | **KIF13A** | kinesin family member 13A | c.4346A>C,c.4385A>C,c.4385A>C,c.4346A>C | KIF13A:NM_001105567:exon36:c.A4346C:p.Q1449P,KIF13A:NM_001105568:exon36:c.A4346C:p.Q1449P,KIF13A:NM_001105566:exon37:c.A4385C:p.Q1462P,KIF13A:NM_022113:exon37:c.A4385C:p.Q1462P | chr6*17772230*17772230*T*C*1*1*Brain_cancer | NA | NA | NA | NA | D | D | N | MODERATE | T | T | T | 1 | 137 | 137 | 0 | 0 | 146 | 83 | 61 | 41.8 |
| Chr6_p23 | 13615619 | G | T | MISSENSE | **NOL7** | nucleolar protein 7 | c.29G>T | NOL7:NM_016167:exon1:c.G29T:p.R10L | NA | NA | NA | NA | NA | T_lc | N | N | MODERATE | T | T | T | 0.003 | 48 | 48 | 0 | 0 | 41 | 28 | 13 | 31.7 |
| Chr6_q12 | 64421166 | C | G | MISSENSE | **PHF3** | PHD finger protein 3 | c.3905C>G | PHF3:NM_015153:exon14:c.C3905G:p.A1302G,PHF3:NM_001290259:exon16:c.C3641G:p.A1214G | NA | NA | NA | NA | NA | T | D | N | MODERATE | T | T | T | 1 | 142 | 140 | 0 | 0 | 165 | 112 | 52 | 31.5 |
| Chr6_q15 | 90432711 | T | A | MISSENSE | **MDN1** | midasin AAA ATPase 1 | c.5930A>T | MDN1:NM_014611:exon40:c.A5930T:p.Y1977F | NA | NA | NA | NA | NA | NA | NA | D | MODERATE | T | T | T | 0.997 | 87 | 86 | 0 | 0 | 93 | 68 | 25 | 26.9 |
| Chr6_q15 | 90440507 | T | C | MISSENSE | **MDN1** | midasin AAA ATPase 1 | c.5078A>G | MDN1:NM_014611:exon35:c.A5078G:p.K1693R | NA | NA | NA | NA | NA | NA | NA | N | MODERATE | T | T | T | 0.196 | 63 | 61 | 0 | 0 | 54 | 33 | 20 | 37.0 |
| Chr6_q15 | 90481432 | G | T | INTRONIC | **MDN1** | midasin AAA ATPase 1 | c.2084-92C>A | NM_014611.1 | NA | NA | NA | NA | NA | NA | NA | NA | MODIFIER | NA | NA | NA | NA | 78 | 78 | 0 | 0 | 74 | 48 | 25 | 33.8 |

| Map Location | Genomic position | Reference allele | Alternate allele | Mutation type | Gene symbol | Protein | Nucleotide change | Description | OncoMD | COSMIC | dbSNP | ExAC | 1000G | SIFT | CONDEL | LRT | VEP | FATHMM | METASVM | METALR | PhastCons7 way | DP in SCC-S | Reference read in SCC-S | Alternative read in SCC-S | Alternate allele frequency in SCC-S (%) | DP in SCC-R | Reference read in SCC-R | Alternate read in SCC-R | Alternate allele frequency in SCC-R (%) |
|---|---|---|---|---|---|---|---|---|---|---|---|---|---|---|---|---|---|---|---|---|---|---|---|---|---|---|---|---|---|
| Chr6_q16.2 | 100390871 | C | G | MISSENSE | **MCHR2** | melanin concentrating hormone receptor 2 | c.541G>C,c.541G>C | MCHR2:NM_001040179:exon4:c.G541C:p.V181L,MCHR2:NM_032503:exon4:c.G541C:p.V181L | chr6*100390871*100390871*C*A*2*2*Renal_cancer | chr6*100390871*100390871*COSM483197*C*A*1*kidney*1 | NA | NA | NA | T | N | N | MODERATE | T | T | T | 0.932 | 262 | 260 | 0 | 0 | 242 | 162 | 78 | 32.2 |
| Chr6_q21 | 106987278 | G | T | SILENT-SS-PRX | **AIM1** | crystallin beta-gamma domain containing 1 | c.3495G>T | AIM1:NM_001624:exon7:c.G3495T:p.T1165T | NA | NA | NA | NA | NA | NA | NA | NA | LOW | NA | NA | NA | NA | 201 | 200 | 0 | 0 | 164 | 104 | 59 | 36.0 |
| Chr6_q22.1 | 116836895 | C | G | MISSENSE | **FAM26E** | calcium homeostasis modulator family member 5 | c.673C>G | FAM26E:NM_153711:exon2:c.C673G:p.Q225E | NA | NA | NA | NA | NA | T | N | N | MODERATE | T | T | T | 0.996 | 321 | 321 | 0 | 0 | 295 | 209 | 83 | 28.1 |
| Chr6_q22.1 | 116836895 | C | G | INTRONIC | **TRAPPC3L** | trafficking protein particle complex 3 like | c.241-15066G>C | FAM26E:NM_153711:exon2:c.C673G:p.Q225E | NA | NA | NA | NA | NA | NA | NA | NA | MODIFIER | NA | NA | NA | NA | 321 | 321 | 0 | 0 | 295 | 209 | 83 | 28.1 |
| Chr6_q22.33 | 129635830 | T | C | MISSENSE | **LAMA2** | laminin subunit alpha 2 | c.3442T>C | LAMA2:NM_000426:exon24:c.T3442C:p.C1148R,LAMA2:NM_001079823:exon24:c.T3442C:p.C1148R | NA | NA | NA | NA | NA | D | D | D | MODERATE | T | D | D | 0.999 | 40 | 40 | 0 | 0 | 42 | 32 | 10 | 23.8 |
| Chr6_q24.2 | 144179139 | G | C | MISSENSE | **LTV1** | LTV1 ribosome biogenesis factor | c.790G>C | LTV1:NM_032860:exon6:c.G790C:p.E264Q | chr6*144179139*144179139*G*C*1*1*Bladder_cancer | chr6*144179139*144179139*COSM1311621*G*C*1*urinary_tract*1 | rs753858250 | 8.26E-06 | NA | D | D | D | MODERATE | 0 | D | D | 1 | 91 | 90 | 0 | 0 | 91 | 56 | 34 | 37.4 |
| Chr6_q24.3 | 146870925 | C | A | INTRONIC | **RAB32** | RAB32, member RAS oncogene family | c.528+48C>A | NM_006834.3 | NA | NA | NA | NA | NA | NA | NA | NA | MODIFIER | NA | NA | NA | NA | 51 | 50 | 0 | 0 | 51 | 33 | 16 | 31.4 |
| Chr6_q25.1 | 151687330 | C | T | MISSENSE | **ZBTB2** | zinc finger and BTB domain containing 2 | c.871G>A | ZBTB2:NM_020861:exon3:c.G871A:p.V291I | NA | NA | NA | 3.29E-05 | NA | T | N | N | MODERATE | T | T | T | 0.019 | 196 | 196 | 0 | 0 | 161 | 96 | 64 | 39.8 |
| Chr6_q25.3 | 158342537 | T | G | INTRONIC | **SNX9** | sorting nexin 9 | c.950-26T>G | NM_016224.4 | NA | NA | NA | NA | NA | NA | NA | NA | MODIFIER | NA | NA | NA | NA | 133 | 133 | 0 | 0 | 110 | 63 | 47 | 42.7 |
| Chr6_q27 | 166576012 | C | A | MISSENSE | **T** | T brachyury transcription factor | c.827G>T | T:NM_003181:exon7:c.G827T:p.C276F | NA | NA | NA | NA | NA | D | D | D | MODERATE | D | D | D | 0.997 | 101 | 101 | 0 | 0 | 64 | 46 | 18 | 28.1 |
| Chr6_q27 | 166576012 | C | A | INTRONIC | **T** | T brachyury transcription factor | c.731-1558G>T | T:NM_003181:exon7:c.G827T:p.C276F | NA | NA | NA | NA | NA | NA | NA | NA | MODIFIER | NA | NA | NA | NA | 101 | 101 | 0 | 0 | 64 | 46 | 18 | 28.1 |
| Chr6_q27 | 167352493 | C | A | MISSENSE | **RNASET2** | ribonuclease T2 | c.336G>T | RNASET2:NM_003730:exon6:c.G336T:p.K112N | NA | NA | NA | NA | NA | T | N | D | MODERATE | T | T | T | 0.998 | 65 | 64 | 0 | 0 | 43 | 38 | 4 | 9.3 |
| Chr7_p12.3 | 47988008 | A | G | SILENT | **PKD1L1** | polycystin 1 like 1, transient receptor potential channel interacting | c.30T>C | PKD1L1:NM_138295:exon1:c.T30C:p.S10S | NA | NA | NA | NA | NA | NA | NA | NA | LOW | NA | NA | NA | NA | 196 | 194 | 0 | 0 | 256 | 215 | 41 | 16.0 |
| Chr7_p14.2-_p14.1 | 37172719 | G | T | INTRONIC | **ELMO1** | engulfment and cell motility 1 | c.1191+16C>A, c.1191+16C>A | NM_014800.10,NM_001206480.1 | NA | NA | NA | NA | NA | NA | NA | NA | MODIFIER | NA | NA | NA | NA | 131 | 131 | 0 | 0 | 155 | 120 | 34 | 21.9 |
| Chr7_p15.2 | 26232907 | C | G | MISSENSE | **HNRNPA2B1** | heterogeneous nuclear ribonucleoprotein A2/B1 | c.964G>C,c.928G>C | HNRNPA2B1:NM_002137:exon9:c.G928C:p.G310R,HNRNPA2B1:NM_031243:exon10:c.G964C:p.G322R | NA | chr7*26232907*26232908*COSM1635261*CA*C*1*liver*1\|chr7*26232907*26232908*COSM1635260*CA*C*1*liver*1 | NA | NA | NA | T | NA | D | MODERATE | D | D | D | 1 | 242 | 242 | 0 | 0 | 298 | 237 | 60 | 20.1 |
| Chr7_p22.2 | 4266969 | T | G | INTRONIC | **SDK1** | sidekick cell adhesion molecule 1 | c.5828-5918T>G | NM_152744.3 | NA | NA | NA | NA | NA | NA | NA | NA | MODIFIER | NA | NA | NA | NA | 45 | 45 | 0 | 0 | 67 | 55 | 11 | 16.4 |
| Chr7_q11.21 | 5360009 | T | A | MISSENSE | **TNRC18** | trinucleotide repeat containing 18 pseudogene 2 | c.6685A>T | TNRC18:NM_001080495:exon24:c.A6685T:p.R2229W | NA | NA | NA | NA | NA | NA | NA | NA | MODERATE | NA | NA | NA | NA | 106 | 105 | 0 | 0 | 92 | 73 | 18 | 19.6 |
| Chr7_q11.22 | 70242117 | T | C | MISSENSE | **AUTS2** | AUTS2, activator of transcription and developmental regulator | c.1961T>C,c.1889T>C | AUTS2:NM_001127231:exon13:c.T1889C:p.V630A,AUTS2:NM_015570:exon14:c.T1961C:p.V654A | NA | NA | NA | NA | NA | D | D | D | MODERATE | T | T | T | 0.998 | 62 | 62 | 0 | 0 | 70 | 64 | 6 | 8.6 |
| Chr7_q11.23 | 75174347 | G | A | INTRONIC | **HIP1** | huntingtin interacting protein 1 | c.2507+39C>T, c.2660+39C>T | NM_005338.6 | NA | NA | NA | NA | NA | NA | NA | NA | MODIFIER | NA | NA | NA | NA | 48 | 48 | 0 | 0 | 41 | 26 | 14 | 34.2 |
| Chr7_q11.23 | 75184815 | A | G | MISSENSE | **HIP1** | huntingtin interacting protein 1 | c.1868T>C,c.1868T>C | HIP1:NM_001243198:exon19:c.T1868C:p.L623P,HIP1:NM_005338:exon19:c.T1868C:p.L623P | NA | NA | NA | NA | NA | D | N | N | MODERATE | T | T | T | 0.002 | 63 | 62 | 0 | 0 | 76 | 65 | 10 | 13.2 |
| Chr7_q21.11 | 84727204 | C | A | NONSENSE | **SEMA3D** | semaphorin 3D | c.229G>T | SEMA3D:NM_152754:exon2:c.G229T:p.E77X | NA | NA | NA | NA | NA | NA | NA | NA | HIGH | NA | NA | NA | 1 | 90 | 90 | 0 | 0 | 131 | 113 | 17 | 13.0 |
| Chr7_q21.12 | 87792315 | A | G | INTRONIC | **ADAM22** | ADAM metallopeptidase domain 22 | c.1908-12A>G,c.1908-12A>G,c.1908-12A>G | NM_021723.3,NM_016351.4 | NA | NA | NA | NA | NA | NA | NA | NA | MODIFIER | NA | NA | NA | NA | 93 | 91 | 0 | 0 | 123 | 81 | 37 | 30.1 |
| Chr7_q21.3 | 96116877 | T | G | INTRONIC | **C7orf76** | SEM1, 26S proteasome complex subunit | c.13-1148A>C | NM_001201450.1 | NA | NA | NA | NA | NA | NA | NA | NA | MODIFIER | NA | NA | NA | NA | 126 | 126 | 0 | 0 | 157 | 123 | 32 | 20.4 |
| Chr7_q22.1 | 100007170 | G | T | INTRONIC-SS-ACR-PRX | **ZCWPW1** | zinc finger CW-type and PWWP domain containing 1 | c.755-3C>A,c.752-3C>A | NM_017984.4 | NA | NA | NA | NA | NA | NA | NA | NA | LOW | NA | NA | NA | NA | 111 | 110 | 0 | 0 | 124 | 104 | 20 | 16.1 |
| Chr7_q22.1 | 102112963 | C | A | SILENT | **LRWD1** | leucine rich repeats and WD repeat domain containing 1 | c.1597C>A | LRWD1:NM_152892:exon13:c.C1597A:p.R533R | NA | NA | NA | NA | NA | NA | NA | NA | LOW | NA | NA | NA | 0.009 | 88 | 87 | 0 | 0 | 87 | 60 | 24 | 27.6 |
| Chr7_q32.3 | 131878859 | C | A | NONSENSE | **PLXNA4** | plexin A4 | c.2818G>T | PLXNA4:NM_020911:exon14:c.G2818T:p.E940X | NA | NA | NA | NA | NA | NA | NA | NA | HIGH | NA | NA | NA | 0.998 | 84 | 84 | 0 | 0 | 110 | 75 | 34 | 30.9 |
| Chr7_q33 | 133261336 | C | A | INTRONIC | **EXOC4** | exocyst complex component 4 | c.1418-53462C>A | NM_021807.3 | NA | NA | NA | NA | NA | NA | NA | NA | MODIFIER | NA | NA | NA | NA | 183 | 183 | 0 | 0 | 244 | 180 | 63 | 25.8 |
| Chr7_q34-_q35 | 143088526 | T | C | 3UTR | **EPHA1** | EPH receptor A1 | c.*24A>G | NM_005232.4 | NA | NA | rs1131885 | 2.80E-01 | 1.64E-01 | NA | NA | NA | MODIFIER | NA | NA | NA | NA | 91 | 91 | 0 | 0 | 111 | 105 | 6 | 5.4 |
| Chr7_q35 | 143633177 | G | T | SILENT | **OR2F2** | olfactory receptor family 2 subfamily F member 2 | c.852G>T | OR2F2:NM_001004685:exon1:c.G852T:p.L284L | NA | NA | NA | NA | NA | NA | NA | NA | LOW | NA | NA | NA | NA | 192 | 191 | 0 | 0 | 197 | 175 | 21 | 10.7 |
| Chr7_q36.1 | 150734282 | A | G | INTRONIC | **ABCB8** | ATP binding cassette subfamily B member 8 | c.1268+546A>G,c.1217+546A>G,c.1217+546A>G,c.953+546A>G | NM_001282293.1 | NA | NA | rs552496198 | 8.09E-05 | NA | NA | NA | NA | MODIFIER | NA | NA | NA | NA | 76 | 76 | 0 | 0 | 61 | 43 | 18 | 29.5 |
| Chr7_q36.1 | 150771209 | G | A | SILENT | **SLC4A2** | solute carrier family 4 member 2 | c.2619G>A,c.2577G>A,c.2619G>A,c.2592G>A | SLC4A2:NM_001199693:exon16:c.G2592A:p.G864G,SLC4A2:NM_001199694:exon16:c.G2577A:p.G859G,SLC4A2:NM_001199692:exon17:c.G2619A:p.G873G,SLC4A2:NM_003040:exon17:c.G2619A:p.G873G | NA | NA | NA | NA | NA | NA | NA | NA | LOW | NA | NA | NA | NA | 36 | 35 | 1 | 2.78 | 43 | 29 | 14 | 32.6 |
| Chr7_q36.2 | 154593091 | T | A | MISSENSE | **DPP6** | dipeptidyl peptidase like 6 | c.1134T>A | DPP6:NM_001290252:exon11:c.T1005A:p.D335E,DPP6:NM_001039350:exon13:c.T1134A:p.D378E,DPP6:NM_001936:exon13:c.T1140A:p.D380E,DPP6:NM_130797:exon13:c.T1326A:p.D442E | NA | NA | NA | NA | NA | D | D | D | MODERATE | T | T | T | 1 | 75 | 75 | 0 | 0 | 57 | 46 | 11 | 19.3 |
| Chr8_p11.21 | 41122835 | G | C | MISSENSE | **SFRP1** | secreted frizzled related protein 1 | c.796C>G | SFRP1:NM_003012:exon3:c.C796G:p.H266D | NA | NA | NA | NA | NA | T | N | N | MODERATE | T | T | T | 0.994 | 48 | 47 | 0 | 0 | 41 | 15 | 25 | 61.0 |
| Chr8_p11.22 | 39142297 | T | G | INTRONIC | **ADAM32** | ADAM metallopeptidase domain 32 | c.2358-15T>G | NM_145004.5 | NA | NA | NA | NA | NA | NA | NA | NA | MODIFIER | NA | NA | NA | NA | 126 | 124 | 0 | 0 | 141 | 92 | 48 | 34.0 |
| Chr8_p21.2 | 24261526 | A | G | MISSENSE | **ADAMDEC1** | ADAM like decysin 1 | c.1331A>G,c.1094A>G,c.1094A>G | ADAMDEC1:NM_001145272:exon12:c.A1094G:p.N365S,ADAMDEC1:NM_014479:exon13:c.A1331G:p.N444S,ADAMDEC1:NM_001145271:exon14:c.A1094G:p.N365S | chr8*24261526*24261526*A*G*1*1*Brain_cancer | NA | rs3765124 | 3.94E-01 | 2.95E-01 | T | N | N | MODERATE | T | T | T | 0.987 | 233 | 231 | 0 | 0 | 164 | 157 | 6 | 3.7 |
| Chr8_p21.2 | 26721784 | G | A | MISSENSE | **ADRA1A** | adrenoceptor alpha 1A | c.703C>T,c.703C>T,c.703C>T, c.703C>T | ADRA1A:NM_000680:exon1:c.C703T:p.R235C,ADRA1A:NM_033302:exon1:c.C703T:p.R235C,ADRA1A:NM_033303:exon1:c.C703T:p.R235C,ADRA1A:NM_033304:exon1:c.C703T:p.R235C | NA | NA | NA | NA | NA | D | D | D | MODERATE | T | D | T | 0.995 | 111 | 111 | 0 | 0 | 90 | 32 | 56 | 62.2 |
| Chr8_p21.2 | 26722380 | A | G | MISSENSE | **ADRA1A** | adrenoceptor alpha 1A | c.107T>C,c.107T>C,c.107T>C, c.107T>C | ADRA1A:NM_000680:exon1:c.T107C:p.L36P,ADRA1A:NM_033302:exon1:c.T107C:p.L36P,ADRA1A:NM_033303:exon1:c.T107C:p.L36P,ADRA1A:NM_033304:exon1:c.T107C:p.L36P | NA | NA | NA | NA | NA | D | D | D | MODERATE | T | T | T | 0.999 | 222 | 220 | 1 | 0.45 | 250 | 237 | 12 | 4.8 |
| Chr8_p23.1 | 11643459 | C | T | INTRONIC | **NEIL2** | nei like DNA glycosylase 2 | c.506-13C>T,c.689-13C>T,c.341-13C>T,c.689-13C>T | NM_145043.2 | NA | NA | rs8191663 | 2.31E-01 | 2.60E-01 | NA | NA | NA | MODIFIER | NA | NA | NA | NA | 49 | 49 | 0 | 0 | 38 | 34 | 4 | 10.5 |
| Chr8_p23.2 | 3257055 | A | G | INTRONIC-SS-ACR-PRX | **CSMD1** | CUB and Sushi multiple domains 1 | c.2267-4T>C | NM_033225.5 | NA | NA | NA | NA | NA | NA | NA | NA | LOW | NA | NA | NA | NA | 24 | 24 | 0 | 0 | 42 | 32 | 10 | 23.8 |
| Chr8_q12.3 | 65517304 | C | G | MISSENSE | **CYP7B1** | cytochrome P450 family 7 subfamily B member 1 | c.1168G>C | CYP7B1:NM_004820:exon5:c.G1168C:p.G390R | NA | NA | NA | NA | NA | D | D | D | MODERATE | T | D | D | 0.146 | 143 | 143 | 0 | 0 | 142 | 117 | 25 | 17.6 |
| Chr8_q13.2 | 69103960 | A | T | SILENT | **PREX2** | phosphatidylinositol-3,4,5-trisphosphate dependent Rac exchange factor 2 | c.4350A>T | PREX2:NM_024870:exon36:c.A4350T:p.A1450A | NA | NA | NA | NA | NA | NA | NA | NA | LOW | NA | NA | NA | NA | 94 | 94 | 0 | 0 | 126 | 94 | 31 | 24.6 |
| Chr8_q13.3 | 70617332 | G | A | MISSENSE | **SLCO5A1** | solute carrier organic anion transporter family member 5A1 | c.1391C>T,c.1556C>T,c.1556C>T | SLCO5A1:NM_001146009:exon4:c.C1391T:p.S464L,SLCO5A1:NM_001146008:exon5:c.C1556T:p.S519L,SLCO5A1:NM_030958:exon6:c.C1556T:p.S519L | NA | NA | NA | NA | NA | T | N | D | MODERATE | T | T | T | 0.998 | 149 | 149 | 0 | 0 | 218 | 188 | 28 | 12.8 |
| Chr8_q13.3 | 71075761 | G | T | SILENT | **NCOA2** | nuclear receptor coactivator 2 | c.771C>A | NCOA2:NM_006540:exon8:c.C771A:p.P257P | chr8*71075761*71075761*G*T*1*1*Lung_cancer | chr8*71075761*71075761*COSM750799*G*T*1*lung*1 | NA | NA | NA | NA | NA | NA | LOW | NA | NA | NA | NA | 213 | 213 | 0 | 0 | 308 | 245 | 61 | 19.8 |
| Chr8_q21.13 | 80577117 | G | T | 3UTR | **STMN2** | stathmin 2 | c.*8G>T | NM_007029.3,NM_001199214.1 | NA | NA | NA | NA | NA | NA | NA | NA | MODIFIER | NA | NA | NA | NA | 87 | 86 | 0 | 0 | 96 | 78 | 18 | 18.8 |
| Chr8_q21.13 | 81399384 | C | G | INTRONIC | **ZBTB10** | zinc finger and BTB domain containing 10 | c.96+1270C>G | ZBTB10:NM_001105539:exon1:c.C339G:p.P113P,ZBTB10:NM_023929:exon1:c.C339G:p.P113P | NA | NA | NA | NA | NA | NA | NA | NA | MODIFIER | NA | NA | NA | NA | 208 | 208 | 0 | 0 | 321 | 254 | 67 | 20.9 |
| Chr8_q21.13 | 81399384 | C | G | SILENT | **ZBTB10** | zinc finger and BTB domain containing 10 | c.339C>G,c.339C>G | ZBTB10:NM_001105539:exon1:c.C339G:p.P113P,ZBTB10:NM_023929:exon1:c.C339G:p.P113P | NA | NA | NA | NA | NA | NA | NA | NA | LOW | NA | NA | NA | NA | 208 | 208 | 0 | 0 | 321 | 254 | 67 | 20.9 |

Jain *et al.* , 2019. MAP2K1 is a potential therapeutic target in erlotinib resistant head and neck squamous cell carcinoma
Supplementary Table S2: List of single nucleotide variants identified in SCC-R cells.

| Map Location | Genomic position | Reference allele | Alternate allele | Mutation type | Gene symbol | Protein | Nucleotide change | Description | OncoMD | COSMIC | dbSNP | ExAC | 1000G | SIFT | CONDEL | LRT | VEP | FATHMM | METASVM | METALR | PhastCons7 way | DP in SCC-S | Reference read in SCC-S | Alternative read in SCC-S | Alternate allele frequency in SCC-S (%) | DP in SCC-R | Reference read in SCC-R | Alternate read in SCC-R | Alternate allele frequency in SCC-R (%) |
|---|---|---|---|---|---|---|---|---|---|---|---|---|---|---|---|---|---|---|---|---|---|---|---|---|---|---|---|---|---|
| Chr8_q22.1 | 95550445 | C | A | INTRONIC | **KIAA1429** | vir like m6A methyltransferase associated | c.266+43G>T,c.266+43G>T | NM_183009.2 | NA | NA | NA | NA | NA | NA | NA | NA | MODIFIER | NA | NA | NA | NA | 117 | 117 | 0 | 0 | 159 | 124 | 34 | 21.4 |
| Chr8_q22.1 | 95952484 | G | A | INTRONIC | **TP53INP1** | tumor protein p53 inducible nuclear protein 1 | c.113-36C>T,c.113-36C>T | NM_001135733.1 | NA | NA | NA | NA | NA | NA | NA | NA | MODIFIER | NA | NA | NA | NA | 77 | 77 | 0 | 0 | 86 | 68 | 18 | 20.9 |
| Chr8_q22.3 | 102705157 | A | C | INTRONIC | **NCALD** | neurocalcin delta | c.379-33T>G,c.379-33T>G,c.379-33T>G,c.379-33T>G | NM_001040630.1,NM_001040627.1,NM_001040629.1,NM_001040628.1 | NA | NA | NA | NA | NA | NA | NA | NA | MODIFIER | NA | NA | NA | NA | 142 | 141 | 0 | 0 | 207 | 199 | 8 | 3.9 |
| Chr8_q23.1 | 107694827 | T | C | INTRONIC | **OXR1** | oxidation resistance 1 | c.307-600T>C,c.283-600T>C,c.304-600T>C,c.304-600T>C | NM_001198533.1 | NA | NA | NA | NA | NA | NA | NA | NA | MODIFIER | NA | NA | NA | NA | 170 | 168 | 0 | 0 | 216 | 161 | 55 | 25.5 |
| Chr8_q23.1-_q23.2 | 110424552 | C | A | MISSENSE | **PKHD1L1** | PKHD1 like 1 | c.2144C>A | PKHD1L1:NM_177531:exon20:c.C2144A:p.S715Y | NA | NA | NA | NA | NA | D | D | D | MODERATE | D | D | D | 1 | 208 | 208 | 0 | 0 | 312 | 222 | 88 | 28.2 |
| Chr8_q23.1-_q23.2 | 110534936 | T | C | INTRONIC | **PKHD1L1** | PKHD1 like 1 | c.12184-37T>C | NM_177531.4 | NA | NA | NA | NA | NA | NA | NA | NA | MODIFIER | NA | NA | NA | NA | 46 | 46 | 0 | 0 | 87 | 59 | 27 | 31.0 |
| Chr8_q23.3 | 113293554 | G | A | SILENT | **CSMD3** | CUB and Sushi multiple domains 3 | c.8850C>T,c.9357C>T,c.9237C>T | CSMD3:NM_052900:exon57:c.C8850T:p.T2950T,CSMD3:NM_198123:exon59:c.C9357T:p.T3119T,CSMD3:NM_198124:exon60:c.C9237T:p.T3079T | chr8*113293554*113293554*G*A*1*1*Lung_cancer | chr8*113293554*113293554*COSM603873*G*A*1*lung*1\|chr8*113293554*113293554*COSM1144786*G*A*1*lung*1 | NA | NA | NA | NA | NA | NA | LOW | NA | NA | NA | NA | 133 | 133 | 0 | 0 | 205 | 153 | 52 | 25.4 |
| Chr8_q24.12 | 121267464 | T | C | INTRONIC | **COL14A1** | collagen type XIV alpha 1 chain | c.2753-15T>C | NM_021110.2 | NA | NA | NA | NA | NA | NA | NA | NA | MODIFIER | NA | NA | NA | NA | 189 | 189 | 0 | 0 | 162 | 115 | 45 | 27.8 |
| Chr8_q24.12 | 121530214 | A | G | SILENT | **MTBP** | MDM2 binding protein | c.2370A>G | MTBP:NM_022045:exon19:c.A2370G:p.P790P | NA | NA | rs776624602 | 2.48E-05 | NA | NA | NA | NA | LOW | NA | NA | NA | NA | 225 | 224 | 0 | 0 | 338 | 253 | 84 | 24.9 |
| Chr8_q24.13 | 125057986 | T | A | INTRONIC | **FER1L6** | fer-1 like family member 6 | c.2590-22T>A | NM_001039112.2 | NA | NA | NA | NA | NA | NA | NA | NA | MODIFIER | NA | NA | NA | NA | 381 | 378 | 0 | 0 | 274 | 217 | 56 | 20.4 |
| Chr8_q24.22 | 134297038 | G | T | INTRONIC | **NDRG1** | N-myc downstream regulated 1 | c.-18-466C>A,c.-18-466C>A,c.-155-466C>A,c.-100+12339C>A | NM_001258432.1 | NA | NA | NA | NA | NA | NA | NA | NA | MODIFIER | NA | NA | NA | NA | 112 | 112 | 0 | 0 | 114 | 103 | 9 | 7.9 |
| Chr8_q24.3 | 142202847 | G | A | INTRONIC | **DENND3** | DENN domain containing 3 | c.3396+85G>A | NM_014957.2 | NA | NA | rs28446884 | 8.02E-02 | 1.19E-01 | NA | NA | NA | MODIFIER | NA | NA | NA | NA | 106 | 106 | 0 | 0 | 144 | 135 | 7 | 4.9 |
| Chr8_q24.3 | 142202856 | C | T | INTRONIC | **DENND3** | DENN domain containing 3 | c.3396+94C>T | NM_014957.2 | NA | NA | rs6990186 | 4.60E-01 | 4.69E-01 | NA | NA | NA | MODIFIER | NA | NA | NA | NA | 105 | 105 | 0 | 0 | 143 | 136 | 6 | 4.2 |
| Chr8_q24.3 | 142487521 | T | G | MISSENSE | **MROH5** | maestro heat like repeat family member 5 | c.1427A>C | NM_207414.2 | NA | NA | NA | NA | NA | T | N | NA | MODERATE | NA | NA | NA | NA | 52 | 52 | 0 | 0 | 61 | 47 | 14 | 23.0 |
| Chr8_q24.3 | 143425720 | T | C | MISSENSE | **TSNARE1** | t-SNARE domain containing 1 | c.352A>G | TSNARE1:NM_145003:exon4:c.A352G:p.T118A | chr8*143425720*143425720*T*G*1*1*Renal_cancer | NA | rs10100935 | 4.48E-01 | 5.86E-01 | T_lc | N | N | MODERATE | T | T | T | 0.019 | 149 | 149 | 0 | 0 | 174 | 165 | 8 | 4.6 |
| Chr8_q24.3 | 144357213 | G | C | INTRONIC | **GLI4** | GLI family zinc finger 4 | c.223+241G>C | NM_138465.3 | NA | NA | rs3817675 | 5.79E-01 | 4.76E-01 | NA | NA | NA | MODIFIER | NA | NA | NA | NA | 176 | 176 | 0 | 0 | 228 | 221 | 7 | 3.1 |
| Chr8_q24.3 | 145003777 | G | A | INTRONIC | **PLEC** | plectin | c.3334+37C>T, c.2923+37C>T, c.2935+37C>T, c.2827+37C>T, c.2923+37C>T, c.2881+37C>T, c.3004+37C>T, c.2857+37C>T | NM_201379.1 | NA | NA | rs11136335 | 3.35E-01 | 2.25E-01 | NA | NA | NA | MODIFIER | NA | NA | NA | NA | 187 | 185 | 0 | 0 | 195 | 185 | 9 | 4.6 |
| Chr9_p13.3 | 34493153 | G | T | INTRONIC | **DNAI1** | dynein axonemal intermediate chain 1 | c.682-39G>T | NM_012144.3,NM_001281428.1 | NA | NA | NA | NA | NA | NA | NA | NA | MODIFIER | NA | NA | NA | NA | 59 | 59 | 0 | 0 | 61 | 44 | 17 | 27.9 |
| Chr9_p13.3 | 36217485 | G | T | SILENT | **GNE** | glucosamine (UDP-N-acetyl)-2-epimerase/N-acetylmannosamine kinase | c.2046C>A,c.2031C>A,c.1716C>A,c.2139C>A | GNE:NM_001190384:exon10:c.C1716A:p.V572V,GNE:NM_001190383:exon11:c.C1824A:p.V608V,GNE:NM_001190388:exon11:c.C2031A:p.V677V,GNE:NM_001128227:exon12:c.C2139A:p.V713V,GNE:NM_005476:exon12:c.C2046A:p.V682V | NA | NA | NA | NA | NA | NA | NA | NA | LOW | NA | NA | NA | NA | 128 | 128 | 0 | 0 | 160 | 126 | 34 | 21.3 |
| Chr9_p24.1 | 5534989 | C | T | SILENT | **PDCD1LG2** | programmed cell death 1 ligand 2 | c.300C>T | PDCD1LG2:NM_025239:exon3:c.C300T:p.Y100Y | NA | NA | rs747508512 | 8.28E-06 | NA | NA | NA | NA | LOW | NA | NA | NA | 0.555 | 108 | 108 | 0 | 0 | 101 | 69 | 30 | 29.7 |
| Chr9_p24.3 | 990309 | G | A | SILENT | **DMRT3** | doublesex and mab-3 related transcription factor 3 | c.723G>A | DMRT3:NM_021240:exon2:c.G723A:p.L241L | NA | NA | rs139342042 | 1.16E-04 | 2.00E-04 | NA | NA | NA | LOW | NA | NA | NA | NA | 214 | 213 | 0 | 0 | 177 | 110 | 66 | 37.3 |
| Chr9_q21.32 | 86357590 | G | A | INTRONIC | **GKAP1** | G kinase anchoring protein 1 | c.752-75C>T,c.905-75C>T | NM_025211.3 | NA | NA | rs775941020 | NA | NA | NA | NA | NA | MODIFIER | NA | NA | NA | NA | 46 | 46 | 0 | 0 | 58 | 40 | 17 | 29.3 |
| Chr9_q22.31 | 95051629 | A | T | MISSENSE | **IARS** | isoleucyl-tRNA synthetase | c.73T>A,c.73T>A | IARS:NM_002161:exon2:c.T73A:p.F25I,IARS:NM_013417:exon2:c.T73A:p.F25I | NA | NA | NA | NA | NA | T | N | N | MODERATE | T | T | T | 0.745 | 56 | 56 | 0 | 0 | 65 | 48 | 17 | 26.2 |
| Chr9_q22.33 | 100126338 | C | T | MISSENSE | **CCDC180** | coiled-coil domain containing 180 | c.4040C>T | CCDC180:NM_020893:exon29:c.C4040T:p.T1347I | NA | NA | NA | NA | NA | D | D | N | MODERATE | T | T | T | 0.034 | 85 | 85 | 0 | 0 | 72 | 66 | 6 | 8.3 |
| Chr9_q22.33 | 101817547 | G | C | MISSENSE | **COL15A1** | collagen type XV alpha 1 chain | c.3085G>C | COL15A1:NM_001855:exon34:c.G3085C:p.E1029Q | NA | NA | NA | NA | NA | D | D | N | MODERATE | D | D | D | 0.974 | 103 | 103 | 0 | 0 | 84 | 51 | 31 | 36.9 |
| Chr9_q31.1 | 102888696 | G | T | MISSENSE | **INVS** | inversin | c.138G>T,c.138G>T | INVS:NM_014425:exon3:c.G138T:p.Q46H,INVS:NM_183245:exon3:c.G138T:p.Q46H | NA | NA | NA | NA | NA | T | N | D | MODERATE | T | T | T | 0.992 | 137 | 137 | 0 | 0 | 190 | 129 | 61 | 32.1 |
| Chr9_q31.1 | 104500132 | T | G | MISSENSE | **GRIN3A** | glutamate ionotropic receptor NMDA type subunit 3A | c.130A>C | GRIN3A:NM_133445:exon1:c.A130C:p.I44L | NA | NA | NA | NA | NA | D_lc | D | N | MODERATE | T | T | T | 1 | 68 | 68 | 0 | 0 | 63 | 36 | 26 | 41.3 |
| Chr9_q31.2 | 109688903 | C | A | MISSENSE | **ZNF462** | zinc finger protein 462 | c.2710C>A | ZNF462:NM_021224:exon3:c.C2710A:p.H904N | NA | NA | NA | NA | NA | NA | NA | D | MODERATE | T | T | T | 0.999 | 449 | 447 | 1 | 0.22 | 399 | 383 | 12 | 3.0 |
| Chr9_q32 | 116972013 | G | T | INTRONIC-SS-DNR-PRX | **COL27A1** | collagen type XXVII alpha 1 chain | c.2322+5G>T | NM_032888.2 | NA | NA | NA | NA | NA | NA | NA | NA | LOW | NA | NA | NA | NA | 152 | 151 | 0 | 0 | 118 | 96 | 22 | 18.6 |
| Chr9_q32 | 117046095 | A | G | INTRONIC | **COL27A1** | collagen type XXVII alpha 1 chain | c.3933+53A>G | NM_032888.2 | NA | NA | NA | NA | NA | NA | NA | NA | MODIFIER | NA | NA | NA | NA | 180 | 173 | 0 | 0 | 170 | 122 | 40 | 23.5 |
| Chr9_q32 | 117064405 | C | G | INTRONIC | **COL27A1** | collagen type XXVII alpha 1 chain | c.4872+17C>G | NM_032888.2 | NA | NA | rs10817586 | 3.29E-01 | 2.16E-01 | NA | NA | NA | MODIFIER | NA | NA | NA | NA | 62 | 62 | 0 | 0 | 76 | 71 | 5 | 6.6 |
| Chr9_q32 | 117086226 | A | G | INTRONIC | **ORM1** | orosomucoid 1 | c.258-72A>G | NM_000607.2 | NA | NA | rs1111796 | NA | 7.31E-01 | NA | NA | NA | MODIFIER | NA | NA | NA | NA | 296 | 296 | 0 | 0 | 321 | 308 | 11 | 3.4 |
| Chr9_q33.1 | 119144785 | G | C | INTRONIC | **PAPPA** | pappalysin 1 | c.4776+13G>C | NM_002581.3 | NA | NA | NA | NA | NA | NA | NA | NA | MODIFIER | NA | NA | NA | NA | 112 | 110 | 0 | 0 | 113 | 95 | 18 | 15.9 |
| Chr9_q33.3 | 126201736 | G | T | INTRONIC | **DENND1A** | DENN domain containing 1A | c.1488+903C>A,c.1488+903C>A | NM_024820.2 | NA | NA | NA | NA | NA | NA | NA | NA | MODIFIER | NA | NA | NA | NA | 172 | 171 | 0 | 0 | 187 | 161 | 26 | 13.9 |
| Chr9_q34.13 | 134346355 | G | T | MISSENSE | **PRRC2B** | proline rich coiled-coil 2B | c.2092G>T | PRRC2B:NM_013318:exon13:c.G2092T:p.A698S | chr9*134346355*134346355*G*A*1*1*Lung_cancer | chr9*134346355*134346355*COSM1195378*G*A*1*lung*1\|chr9*134346355*134346355*COSM1195379*G*A*1*lung*1 | NA | NA | NA | T | N | U | MODERATE | T | T | T | 0.989 | 245 | 244 | 0 | 0 | 247 | 236 | 10 | 4.1 |
| Chr9_q34.13 | 134366008 | G | T | INTRONIC | **PRRC2B** | proline rich coiled-coil 2B | c.6226-804G>T | NM_013318.3 | NA | NA | NA | NA | NA | NA | NA | NA | MODIFIER | NA | NA | NA | NA | 231 | 229 | 0 | 0 | 251 | 242 | 8 | 3.2 |
| Chr9_q34.3 | 139741354 | C | T | MISSENSE | **C9orf172** | apical junction component 1 homolog | c.2488C>T | C9orf172:NM_001080482:exon1:c.C2488T:p.P830S | NA | NA | NA | NA | NA | T | N | NA | MODERATE | NA | NA | NA | NA | 134 | 134 | 0 | 0 | 135 | 101 | 34 | 25.2 |
| Chr9_q34.3 | 140079522 | G | A | SILENT | **ANAPC2** | anaphase promoting complex subunit 2 | c.891C>T | ANAPC2:NM_013366:exon4:c.C891T:p.V297V | NA | NA | rs11549106 | 2.99E-01 | 1.85E-01 | NA | NA | NA | LOW | NA | NA | NA | NA | 262 | 262 | 0 | 0 | 253 | 244 | 8 | 3.2 |
| Chr10_p11.1 | 38344488 | G | T | MISSENSE | **ZNF33A** | zinc finger protein 33A | c.1454G>T,c.1436G>T | ZNF33A:NM_001278170:exon4:c.G1454T:p.S485I,ZNF33A:NM_001278177:exon5:c.G1499T:p.S500I,ZNF33A:NM_001278178:exon5:c.G1100T:p.S367I,ZNF33A:NM_006954:exon5:c.G1436T:p.S479I,ZNF33A:NM_006974:exon5:c.G1433T:p.S478I,ZNF33A:NM_001278171:exon6:c.G1100T:p.S367I,ZNF33A:NM_001278173:exon6:c.G1490T:p.S497I,ZNF33A:NM_001278174:exon6:c.G1100T:p.S367I,ZNF33A:NM_001278175:exon6:c.G1202T:p.S401I,ZNF33A:NM_001278176:exon6:c.G1307T:p.S436I,ZNF33A:NM_001278179:exon6:c.G1100T:p.S367I | NA | NA | NA | NA | NA | T | N | N | MODERATE | T | T | T | 0.142 | 97 | 97 | 0 | 0 | 130 | 123 | 6 | 4.6 |
| Chr10_p15.2 | 3199658 | T | C | MISSENSE | **PITRM1** | pitrilysin metallopeptidase 1 | c.1316A>G,c.1220A>G | PITRM1:NM_001242309:exon11:c.A1220G:p.Q407R,PITRM1:NM_001242307:exon12:c.A1316G:p.Q439R,PITRM1:NM_014889:exon12:c.A1316G:p.Q439R | NA | NA | NA | NA | NA | D | D | D | MODERATE | T | T | T | 0.986 | 34 | 34 | 0 | 0 | 55 | 21 | 32 | 58.2 |
| Chr10_q11.23 | 52596072 | T | C | SILENT-SS-PRX | **A1CF** | APOBEC1 complementation factor | c.366A>G,c.390A>G,c.366A>G,c.345A>G | A1CF:NM_014576:exon6:c.A366G:p.R122R,A1CF:NM_138932:exon6:c.A366G:p.R122R,A1CF:NM_138933:exon6:c.A390G:p.R130R,A1CF:NM_001198818:exon7:c.A366G:p.R122R,A1CF:NM_001198820:exon7:c.A390G:p.R130R,A1CF:NM_001198819:exon8:c.A390G:p.R130R | NA | NA | NA | NA | NA | NA | NA | NA | LOW | NA | NA | NA | 0.763 | 224 | 223 | 0 | 0 | 173 | 113 | 54 | 31.2 |
| Chr10_q22.2 | 75535164 | C | T | INTRONIC | **FUT11** | fucosyltransferase 11 | c.1335-135C>T,c.1335-13C>T | NM_001284194.1 | NA | NA | NA | NA | NA | NA | NA | NA | MODIFIER | NA | NA | NA | NA | 271 | 271 | 0 | 0 | 181 | 131 | 50 | 27.6 |
| Chr10_q23.1 | 82403785 | C | T | MISSENSE | **SH2D4B** | SH2 domain containing 4B | c.1022C>T,c.878C>T | SH2D4B:NM_001145719:exon7:c.C878T:p.S293L,SH2D4B:NM_207372:exon7:c.C1022T:p.S341L | NA | NA | NA | NA | NA | D_lc | N | NA | MODERATE | NA | NA | NA | NA | 230 | 228 | 1 | 0.43 | 157 | 121 | 36 | 22.9 |
| Chr10_q23.1 | 85904644 | T | C | MISSENSE | **GHITM** | growth hormone inducible transmembrane protein | c.355T>C | GHITM:NM_014394:exon5:c.T355C:p.Y119H | NA | NA | NA | NA | NA | D | D | D | MODERATE | 0 | T | T | 1 | 254 | 252 | 1 | 0.39 | 165 | 115 | 50 | 30.3 |

**Supplementary Table S2: List of single nucleotide variants identified in SCC-R cells.**

| Map Location | Genomic position | Reference allele | Alternate allele | Mutation type | Gene symbol | Protein | Nucleotide change | Description | OncoMD | COSMIC | dbSNP | ExAC | 1000G | SIFT | CONDEL | LRT | VEP | FATHMM | METASVM | METALR | PhastCons7 way | DP in SCC-S | Reference read in SCC-S | Alternative read in SCC-S | Alternate allele frequency in SCC-S (%) | DP in SCC-R | Reference read in SCC-R | Alternate read in SCC-R | Alternate allele frequency in SCC-R (%) |
|---|---|---|---|---|---|---|---|---|---|---|---|---|---|---|---|---|---|---|---|---|---|---|---|---|---|---|---|---|---|
| Chr10_q24.1 | 99225846 | C | T | MISSENSE | **MMS19** | MMS19 homolog, cytosolic iron-sulfur assembly component | c.1576G>A | MMS19:NM_001289403:exon16:c.G1447A:p.V483I,MMS19:NM_001289404:exon16:c.G1099A:p.V367I,MMS19:NM_022362:exon17:c.G1576A:p.V526I,MMS19:NM_001289405:exon18:c.G1576A:p.V526I | NA | NA | rs17112809 | 2.70E-02 | 4.29E-02 | T | N | N | MODERATE | T | T | T | 0.4 | 141 | 141 | 0 | 0 | 114 | 70 | 43 | 37.7 |
| Chr10_q24.33 | 105362278 | G | C | MISSENSE | **SH3PXD2A** | SH3 and PX domains 2A | c.2613C>G | SH3PXD2A:NM_014631:exon14:c.C2613G:p.N871K | NA | NA | NA | NA | NA | T | N | N | MODERATE | T | T | T | 0.198 | 304 | 304 | 0 | 0 | 160 | 108 | 51 | 31.9 |
| Chr10_q25.2 | 112266771 | C | T | SILENT | **DUSP5** | dual specificity phosphatase 5 | c.607C>T | DUSP5:NM_004419:exon3:c.C607T:p.L203L | chr10*112266771*112266771*C*T*1*1*Stomach_(gastric)_cancer | chr10*112266771*112266771*COSM147043*C*T*1*stomach*1 | rs1889567 | 2.89E-01 | 2.84E-01 | NA | NA | NA | LOW | NA | NA | NA | 0.999 | 225 | 225 | 0 | 0 | 162 | 155 | 7 | 4.3 |
| Chr10_q25.2 | 114192990 | G | A | INTRONIC-SS-DNR-PRX | **ZDHHC6** | zinc finger DHHC-type containing 6 | c.945+8C>T | NM_022494.1 | NA | NA | NA | NA | NA | NA | NA | NA | LOW | NA | NA | NA | NA | 41 | 40 | 0 | 0 | 47 | 35 | 10 | 21.3 |
| Chr10_q26.12 | 122611118 | G | A | INTRONIC | **WDR11** | WD repeat domain 11 | c.86+100G>A | NM_018117.11 | NA | NA | NA | NA | NA | NA | NA | NA | MODIFIER | NA | NA | NA | NA | 165 | 164 | 0 | 0 | 96 | 72 | 24 | 25.0 |
| Chr10_q26.13 | 124793872 | C | A | MISSENSE-SS-PRX | **ACADSB** | acyl-CoA dehydrogenase short/branched chain | c.43C>A | ACADSB:NM_001609:exon2:c.C43A:p.L15I | NA | NA | NA | NA | NA | T | D | N | MODERATE | D | D | D | 0.205 | 143 | 143 | 0 | 0 | 89 | 62 | 27 | 30.3 |
| Chr10_q26.2 | 127753438 | C | T | MISSENSE | **ADAM12** | ADAM metallopeptidase domain 12 | c.1555G>A,c.1555G>A | ADAM12:NM_001288973:exon14:c.G1546A:p.G516S,ADAM12:NM_001288974:exon14:c.G1546A:p.G516S,ADAM12:NM_001288975:exon14:c.G1546A:p.G516S,ADAM12:NM_003474:exon14:c.G1555A:p.G519S,ADAM12:NM_021641:exon14:c.G1555A:p.G519S | NA | NA | rs145695439 | 3.39E-04 | 2.00E-04 | T | D | D | MODERATE | T | T | T | 0.98 | 122 | 122 | 0 | 0 | 49 | 36 | 13 | 26.5 |
| Chr10_q26.3 | 133058649 | G | A | SILENT | **TCERG1L** | transcription elongation regulator 1 like | c.729C>T | TCERG1L:NM_174937:exon4:c.C729T:p.A243A | chr10*133058649*133058649*C*A*2*1*Liver_cancer | chr10*133058649*133058649*COSM3665587*G*T*2*liver*2 | rs149055623 | 3.25E-03 | 9.98E-04 | NA | NA | NA | LOW | NA | NA | NA | NA | 51 | 51 | 0 | 0 | 47 | 39 | 8 | 17.0 |
| Chr10_q26.3 | 134705979 | C | T | INTRONIC | **TTC40** | cilia and flagella associated protein 46 | c.3220-58G>A | NM_001200049.2 | NA | NA | NA | NA | NA | NA | NA | NA | MODIFIER | NA | NA | NA | NA | 279 | 279 | 0 | 0 | 221 | 143 | 77 | 34.8 |
| Chr11_p11.2 | 47435015 | C | A | MISSENSE | **SLC39A13** | solute carrier family 39 member 13 | c.602C>A,c.602C>A | SLC39A13:NM_001128225:exon5:c.C602A:p.P201Q,SLC39A13:NM_152264:exon5:c.C602A:p.P201Q | NA | NA | NA | NA | NA | T | N | N | MODERATE | T | T | T | 0.999 | 130 | 130 | 0 | 0 | 151 | 112 | 39 | 25.8 |
| Chr11_p11.2 | 47800531 | G | T | 3UTR | **NUP160** | nucleoporin 160 | c.*126C>A | NM_015231.1 | NA | NA | NA | NA | NA | NA | NA | NA | MODIFIER | NA | NA | NA | NA | 53 | 53 | 0 | 0 | 67 | 40 | 27 | 40.3 |
| Chr11_p15.1 | 17416678 | G | T | INTRONIC | **ABCC8** | ATP binding cassette subfamily C member 8 | c.4414+41C>A | NM_000352.3,NM_001287174.1 | NA | NA | NA | NA | NA | NA | NA | NA | MODIFIER | NA | NA | NA | NA | 94 | 93 | 0 | 0 | 85 | 77 | 7 | 8.2 |
| Chr11_p15.4 | 3752755 | A | G | SILENT | **NUP98** | nucleoporin 98 | c.1596T>C,c.1647T>C,c.1596T>C | NUP98:NM_005387:exon14:c.T1647C:p.H549H,NUP98:NM_016320:exon14:c.T1596C:p.H532H,NUP98:NM_139131:exon14:c.T1596C:p.H532H,NUP98:NM_139132:exon14:c.T1596C:p.H532H | NA | NA | NA | NA | NA | NA | NA | NA | LOW | NA | NA | NA | 1 | 31 | 31 | 0 | 0 | 36 | 28 | 8 | 22.2 |
| Chr11_p15.5 | 281164 | C | T | MISSENSE | **NLRP6** | NLR family pyrin domain containing 6 | c.1430C>T | NLRP6:NM_001276700:exon4:c.C1430T:p.T477M,NLRP6:NM_138329:exon4:c.C1430T:p.T477M | NA | NA | NA | NA | NA | T | N | N | MODERATE | T | T | T | 0.187 | 203 | 202 | 0 | 0 | 235 | 164 | 68 | 28.9 |
| Chr11_p15.5 | 534286 | C | G | MISSENSE | **HRAS** | HRas proto-oncogene, GTPase | c.37G>C,c.37G>C | HRAS:NM_001130442:exon2:c.G37C:p.G13R,HRAS:NM_005343:exon2:c.G37C:p.G13R,HRAS:NM_176795:exon2:c.G37C:p.G13R | chr11*534286*534286*G*T*6*5*Thyroid_cancer\|chr11*534286*534286*G*C*64*27*Head_and_neck_cancer\|chr11*534286*534286*G*A*11*8*Skin_cancer\|chr11*534286*534286*C*T*1*1*Renal_cancer\|chr11*534286*534286*C*G*17*10*Head_and_neck_cancer\|chr11*534286*534286*C*A*1*1*Lung_cancer | chr11*534286*534286*COSM99938*C*G*8*urinary_tract,upper_aerodigestive_tract,lung*4,2,2\|chr11*534286*534286*COSM487*C*T*17*lung,skin,soft_tissue,upper_aerodigestive_tract,bone*7,2,5,2,1\|chr11*534286*534286*COSM486*C*G*231*salivary_gland,urinary_tract,upper_aerodigestive_tract,thyroid,skin,soft_tissue,adrenal_gland,lung,prostate,central_nervous_system*3,10,8,15,148,3,2,2,3,37\|chr11*534286*534286*COSM689078*C*A*1*lung*1\|chr11*534286*534286*COSM488*C*A*11*lung,thyroid,upper_aerodigestive_tract,urinary_tract*5,2,1,3 | NA | NA | NA | D | D | D | MODERATE | T | D | D | 0.984 | 151 | 151 | 0 | 0 | 152 | 121 | 31 | 20.4 |
| Chr11_p15.5 | 637442 | C | T | SILENT | **DRD4** | dopamine receptor D4 | c.138C>T | DRD4:NM_000797:exon1:c.C138T:p.G46G | NA | NA | NA | NA | NA | NA | NA | NA | LOW | NA | NA | NA | NA | 79 | 79 | 0 | 0 | 63 | 47 | 16 | 25.4 |
| Chr11_p15.5 | 1605808 | C | T | SILENT | **KRTAP5-1** | keratin associated protein 5-1 | c.672G>A | KRTAP5-1:NM_001005922:exon1:c.G672A:p.A224A | NA | NA | rs200453575 | 4.94E-05 | 2.00E-04 | NA | NA | NA | LOW | NA | NA | NA | NA | 178 | 178 | 0 | 0 | 169 | 117 | 49 | 29.0 |
| Chr11_q12.1 | 56019875 | T | C | MISSENSE | **OR5T3** | olfactory receptor family 5 subfamily T member 3 | c.200T>C | OR5T3:NM_001004747:exon1:c.T200C:p.F67S | NA | NA | NA | NA | NA | D | N | D | MODERATE | T | T | T | 0.004 | 144 | 141 | 0 | 0 | 147 | 117 | 28 | 19.1 |
| Chr11_q12.1 | 58170214 | G | A | SILENT | **OR5B3** | olfactory receptor family 5 subfamily B member 3 | c.669C>T | OR5B3:NM_001005469:exon1:c.C669T:p.I223I | NA | NA | rs780387507 | 3.30E-05 | NA | NA | NA | NA | LOW | NA | NA | NA | 0.955 | 204 | 204 | 0 | 0 | 198 | 173 | 25 | 12.6 |
| Chr11_q12.1 | 58170808 | G | T | SILENT | **OR5B3** | olfactory receptor family 5 subfamily B member 3 | c.75C>A | OR5B3:NM_001005469:exon1:c.C75A:p.L25L | NA | NA | NA | NA | NA | NA | NA | NA | LOW | NA | NA | NA | NA | 419 | 418 | 0 | 0 | 417 | 372 | 45 | 10.8 |
| Chr11_q12.1 | 58891683 | C | A | MISSENSE | **FAM111B** | family with sequence similarity 111 member B | c.23C>A,c.113C>A | FAM111B:NM_001142704:exon2:c.C23A:p.T8K,FAM111B:NM_001142703:exon3:c.C23A:p.T8K,FAM111B:NM_198947:exon4:c.C113A:p.T38K | NA | NA | NA | NA | NA | D | N | NA | MODERATE | NA | NA | NA | NA | 138 | 138 | 0 | 0 | 139 | 108 | 30 | 21.6 |
| Chr11_q12.2 | 60050011 | C | T | INTRONIC | **MS4A4A** | membrane spanning 4-domains A4A | c.41+1822C>T,c.-162-56C>T,c.41+1822C>T | NM_148975.2 | NA | NA | NA | NA | NA | NA | NA | NA | MODIFIER | NA | NA | NA | NA | 156 | 156 | 0 | 0 | 130 | 87 | 43 | 33.1 |
| Chr11_q12.2 | 60183220 | C | A | NONSENSE | **MS4A14** | membrane spanning 4-domains A14 | c.728C>A,c.878C>A,c.779C>A | MS4A14:NM_001079692:exon4:c.C728A:p.S243X,MS4A14:NM_001261827:exon5:c.C827A:p.S276X,MS4A14:NM_032597:exon5:c.C779A:p.S260X,MS4A14:NM_001261828:exon6:c.C878A:p.S293X | NA | NA | NA | NA | NA | NA | NA | NA | HIGH | NA | NA | NA | 0.129 | 280 | 280 | 0 | 0 | 259 | 172 | 86 | 33.2 |
| Chr11_q12.2 | 60296964 | A | G | INTRONIC | **MS4A13** | membrane spanning 4-domains A13 | c.402+31A>G,c.282+31A>G,c.225+31A>G | NM_001278320.1 | NA | NA | NA | NA | NA | NA | NA | NA | MODIFIER | NA | NA | NA | NA | 35 | 35 | 0 | 0 | 29 | 19 | 10 | 34.5 |
| Chr11_q12.2 | 60610065 | G | A | SILENT | **CCDC86** | coiled-coil domain containing 86 | c.468G>A | CCDC86:NM_024098:exon1:c.G468A:p.P156P | chr11*60610065*60610065*G*A*1*1*Breast_cancer | chr11*60610065*60610065*COSM429370*G*A*1*breast*1 | rs2074420 | 2.69E-01 | 3.41E-01 | NA | NA | NA | LOW | NA | NA | NA | NA | 156 | 154 | 0 | 0 | 187 | 178 | 7 | 3.7 |
| Chr11_q12.2 | 61523220 | C | T | INTRONIC | **MYRF** | myelin regulatory factor | c.46+2958C>T,c.19+306C>T | NM_013279.2 | NA | NA | NA | NA | NA | NA | NA | NA | MODIFIER | NA | NA | NA | NA | 130 | 130 | 0 | 0 | 114 | 82 | 24 | 21.1 |
| Chr11_q12.2 | 61533138 | A | G | MISSENSE | **MYRF** | myelin regulatory factor | c.88A>G,c.61A>G | MYRF:NM_001127392:exon2:c.A88G:p.T30A,MYRF:NM_013279:exon2:c.A61G:p.T21A | NA | NA | NA | NA | NA | D_lc | D | D | MODERATE | T | T | T | 0.993 | 41 | 41 | 0 | 0 | 44 | 33 | 11 | 25.0 |
| Chr11_q12.2 | 61547586 | C | T | INTRONIC | **MYRF** | myelin regulatory factor | c.2337-108C>T,c.2310-108C>T | NM_013279.2 | NA | NA | NA | NA | NA | NA | NA | NA | MODIFIER | NA | NA | NA | NA | 78 | 78 | 0 | 0 | 54 | 44 | 9 | 16.7 |
| Chr11_q12.3 | 62370155 | G | A | INTRONIC-SS-ACR-PRX | **EML3** | echinoderm microtubule associated protein like 3 | c.2488-5C>T | NM_153265.2 | chr11*62370155*62370155*C*T*1*1*Breast_cancer | chr11*62370155*62370155*COSM429448*G*A*1*breast*1 | rs71490394 | 3.34E-01 | 1.64E-01 | NA | NA | NA | LOW | NA | NA | NA | NA | 304 | 304 | 0 | 0 | 299 | 287 | 12 | 4.0 |
| Chr11_q12.3 | 62458765 | C | T | MISSENSE | **BSCL2** | BSCL2, seipin lipid droplet biogenesis associated | c.992G>A,c.800G>A | BSCL2:NM_001122955:exon7:c.G992A:p.R331H,BSCL2:NM_032667:exon7:c.G800A:p.R267H | NA | NA | rs201229787 | 6.59E-05 | 2.00E-04 | T | N | U | MODERATE | D | T | D | 0.999 | 155 | 154 | 0 | 0 | 156 | 149 | 7 | 4.5 |
| Chr11_q12.3 | 62458765 | C | T | INTRONIC | **BSCL2** | BSCL2, seipin lipid droplet biogenesis associated | c.672-152G>A | BSCL2:NM_001122955:exon7:c.G992A:p.R331H,BSCL2:NM_032667:exon7:c.G800A:p.R267H | NA | NA | rs201229787 | 6.59E-05 | 2.00E-04 | NA | NA | NA | MODIFIER | NA | NA | NA | NA | 155 | 154 | 0 | 0 | 156 | 149 | 7 | 4.5 |
| Chr11_q13.1 | 64684548 | C | A | MISSENSE | **ATG2A** | autophagy related 2A | c.60G>T | ATG2A:NM_015104:exon1:c.G60T:p.L20F | NA | NA | NA | NA | NA | D | D | D | MODERATE | D | D | D | 0.997 | 239 | 238 | 0 | 0 | 219 | 186 | 30 | 13.7 |
| Chr11_q13.2 | 66059532 | C | T | SILENT | **TMEM151A** | transmembrane protein 151A | c.48C>T | TMEM151A:NM_153266:exon1:c.C48T:p.I16I | NA | NA | NA | NA | NA | NA | NA | NA | LOW | NA | NA | NA | 0.246 | 29 | 28 | 0 | 0 | 39 | 32 | 7 | 18.0 |
| Chr11_q13.2 | 66083112 | C | A | MISSENSE | **CD248** | CD248 molecule | c.1387G>T | CD248:NM_020404:exon1:c.G1387T:p.A463S | NA | NA | NA | NA | NA | D_lc | N | N | MODERATE | D | T | T | 0.508 | 246 | 245 | 0 | 0 | 244 | 209 | 34 | 13.9 |
| Chr11_q13.2 | 66083720 | T | A | MISSENSE | **CD248** | CD248 molecule | c.779A>T | CD248:NM_020404:exon1:c.A779T:p.E260V | NA | NA | NA | NA | NA | D | D | N | MODERATE | D | D | D | 0.992 | 281 | 280 | 0 | 0 | 317 | 262 | 55 | 17.4 |
| Chr11_q13.2 | 66276837 | A | G | 3UTR | **DPP3** | dipeptidyl peptidase 3 | c.*115A>G | NM_130443.3,NM_005700.4,NM_001256670.1 | NA | NA | NA | NA | NA | NA | NA | NA | MODIFIER | NA | NA | NA | NA | 167 | 167 | 0 | 0 | 213 | 180 | 32 | 15.0 |
| Chr11_q13.4 | 73998387 | G | A | INTRONIC | **P4HA3** | prolyl 4-hydroxylase subunit alpha 3 | c.770-951C>T | NM_182904.3,NM_001288748.1 | NA | NA | NA | NA | NA | NA | NA | NA | MODIFIER | NA | NA | NA | NA | 92 | 92 | 0 | 0 | 95 | 59 | 35 | 36.8 |
| Chr11_q13.5 | 75299093 | G | T | MISSENSE | **MAP6** | microtubule associated protein 6 | c.1453C>A | MAP6:NM_033063:exon4:c.C1453A:p.Q485K | NA | NA | NA | NA | NA | D_lc | N | N | MODERATE | T | T | T | 0.005 | 208 | 207 | 0 | 0 | 225 | 194 | 30 | 13.3 |
| Chr11_q14.1 | 78601000 | A | G | MISSENSE | **TENM4** | teneurin transmembrane protein 4 | c.914T>C | TENM4:NM_001098816:exon9:c.T914C:p.L305P | NA | NA | NA | NA | NA | D_lc | D | D | MODERATE | T | T | T | 0.995 | 135 | 134 | 0 | 0 | 105 | 86 | 18 | 17.1 |
| Chr11_q14.3 | 89222185 | T | C | INTRONIC | **NOX4** | NADPH oxidase 4 | c.81+1441A>G,c.81+1441A>G | NM_001143837.1 | NA | NA | NA | NA | NA | NA | NA | NA | MODIFIER | NA | NA | NA | NA | 263 | 262 | 0 | 0 | 361 | 330 | 29 | 8.0 |
| Chr11_q14.3 | 92507251 | A | T | MISSENSE | **FAT3** | FAT atypical cadherin 3 | c.4240A>T | FAT3:NM_001008781:exon6:c.A4240T:p.T1414S | NA | NA | NA | NA | NA | NA | NA | NA | MODERATE | NA | NA | NA | NA | 333 | 333 | 0 | 0 | 304 | 235 | 69 | 22.7 |
| Chr11_q22.2 | 102592423 | A | G | MISSENSE | **MMP8** | matrix metallopeptidase 8 | c.418T>C | MMP8:NM_002424:exon3:c.T418C:p.W140R,MMP8:NM_001304441:exon4:c.T349C:p.W117R,MMP8:NM_001304442:exon4:c.T349C:p.W117R | NA | NA | NA | NA | NA | D | D | D | MODERATE | T | D | T | 0.299 | 709 | 706 | 0 | 0 | 900 | 852 | 45 | 5.0 |
| Chr11_q22.3 | 108068038 | G | C | SILENT | **NPAT** | nuclear protein, coactivator of histone transcription | c.147C>G | NPAT:NM_002519:exon2:c.C147G:p.A49A | NA | NA | NA | NA | NA | NA | NA | NA | LOW | NA | NA | NA | NA | 41 | 41 | 0 | 0 | 38 | 22 | 15 | 39.5 |

Jain *et al.* , 2019. MAP2K1 is a potential therapeutic target in erlotinib resistant head and neck squamous cell carcinoma
**Supplementary Table S2: List of single nucleotide variants identified in SCC-R cells.**

| Map Location | Genomic position | Reference allele | Alternate allele | Mutation type | Gene symbol | Protein | Nucleotide change | Description | OncoMD | COSMIC | dbSNP | ExAC | 1000G | SIFT | CONDEL | LRT | VEP | FATHMM | METASVM | METALR | PhastCons7 way | DP in SCC-S | Reference read in SCC-S | Alternative read in SCC-S | Alternate allele frequency in SCC-S (%) | DP in SCC-R | Reference read in SCC-R | Alternate read in SCC-R | Alternate allele frequency in SCC-R (%) |
|---|---|---|---|---|---|---|---|---|---|---|---|---|---|---|---|---|---|---|---|---|---|---|---|---|---|---|---|---|---|
| Chr11_q23.2 | 113320820 | A | T | INTRONIC | **DRD2** | dopamine receptor D2 | c.-32+24978T>A,c.-32+24978T>A | NM_016574.3 | NA | NA | NA | NA | NA | NA | NA | NA | MODIFIER | NA | NA | NA | NA | 28 | 28 | 0 | 0 | 19 | 9 | 10 | 52.6 |
| Chr11_q23.2 | 114027143 | A | G | SILENT | **ZBTB16** | zinc finger and BTB domain containing 16 | c.1353A>G,c.1353A>G | ZBTB16:NM_001018011:exon3:c.A1353G:p.L451L,ZBTB16:NM_006006:exon3:c.A1353G:p.L451L | NA | NA | NA | NA | NA | NA | NA | NA | LOW | NA | NA | NA | 1 | 103 | 103 | 0 | 0 | 93 | 55 | 38 | 40.9 |
| Chr11_q23.3 | 117266312 | C | G | MISSENSE | **CEP164** | centrosomal protein 164 | c.2963C>G | CEP164:NM_001271933:exon23:c.C2972G:p.T991S,CEP164:NM_014956:exon24:c.C2963G:p.T988S | NA | NA | rs2305830 | 2.80E-01 | 2.78E-01 | T | N | N | MODERATE | T | T | T | 0.988 | 27 | 27 | 0 | 0 | 38 | 33 | 5 | 13.2 |
| Chr11_q24.1 | 122775040 | C | A | MISSENSE | **C11orf63** | junctional cadherin complex regulator | c.752C>A,c.752C>A | C11orf63:NM_024806:exon3:c.C752A:p.S251Y,C11orf63:NM_199124:exon3:c.C752A:p.S251Y | NA | NA | NA | NA | NA | D | D | NA | MODERATE | NA | NA | NA | NA | 73 | 72 | 0 | 0 | 101 | 57 | 41 | 40.6 |
| Chr11_q24.2 | 124539306 | G | A | MISSENSE | **SIAE** | sialic acid acetylesterase | c.74C>T | SIAE:NM_170601:exon2:c.C179T:p.T60I,SIAE:NM_001199922:exon4:c.C74T:p.T25I | NA | NA | NA | NA | NA | T | N | N | MODERATE | D | T | D | 0.926 | 86 | 86 | 0 | 0 | 112 | 74 | 36 | 32.1 |
| Chr11_q24.2 | 125467089 | C | T | INTRONIC | **STT3A** | STT3A, catalytic subunit of the oligosaccharyltransferase complex | c.149+75C>T | NM_001278503.1,NM_152713.4,NM_001278504.1 | NA | NA | NA | NA | NA | NA | NA | NA | MODIFIER | NA | NA | NA | NA | 120 | 119 | 0 | 0 | 105 | 51 | 54 | 51.4 |
| Chr11_q24.2 | 126310299 | T | C | INTRONIC | **KIRREL3** | kirre like nephrin family adhesion molecule 3 | c.1353+45A>G, c.1353+45A>G | NM_001161707.1 | NA | NA | rs3212313 | 3.07E-01 | 3.57E-01 | NA | NA | NA | MODIFIER | NA | NA | NA | NA | 194 | 193 | 0 | 0 | 176 | 165 | 10 | 5.7 |
| Chr11_q24.3 | 130343306 | C | T | MISSENSE | **ADAMTS15** | ADAM metallopeptidase with thrombospondin type 1 motif 15 | c.2443C>T | ADAMTS15:NM_139055:exon8:c.C2443T:p.P815S | chr11*130343306*130343306*C*T*1*1*Lung_cancer | NA | rs147148311 | 3.27E-03 | 1.20E-03 | T | N | NA | MODERATE | NA | NA | NA | NA | 133 | 133 | 0 | 0 | 131 | 124 | 7 | 5.3 |
| Chr11_q25 | 133796906 | G | T | MISSENSE | **IGSF9B** | immunoglobulin superfamily member 9B | c.1712C>A | IGSF9B:NM_001277285:exon13:c.C1712A:p.T571N | NA | NA | NA | NA | NA | T | N | NA | MODERATE | NA | NA | NA | NA | 23 | 23 | 0 | 0 | 33 | 17 | 16 | 48.5 |
| Chr12_p11.22 | 29598253 | G | T | SILENT | **OVCH1** | ovochymase 1 | c.2839C>A | OVCH1:NM_183378:exon23:c.C2839A:p.R947R | NA | NA | NA | NA | NA | NA | NA | NA | LOW | NA | NA | NA | NA | 246 | 246 | 0 | 0 | 236 | 188 | 48 | 20.3 |
| Chr12_p11.23 | 27655054 | A | G | SILENT-STOPCODON | **SMCO2** | single-pass membrane protein with coiled-coil domains 2 | c.1032A>G | SMCO2:NM_001145010:exon9:c.A1032G:p.X344X | NA | NA | NA | NA | NA | NA | NA | NA | LOW | NA | NA | NA | NA | 115 | 115 | 0 | 0 | 144 | 113 | 31 | 21.5 |
| Chr12_p12.1 | 22627668 | G | A | INTRONIC | **C2CD5** | C2 calcium dependent domain containing 5 | c.2082+17C>T, c.2043+17C>T, c.2043+17C>T, c.2043+17C>T, c.2049+17C>T, c.2016+17C>T | NM_001286177.1 | NA | NA | NA | NA | NA | NA | NA | NA | MODIFIER | NA | NA | NA | NA | 205 | 203 | 0 | 0 | 231 | 186 | 44 | 19.1 |
| Chr12_p12.3 | 14813880 | G | T | INTRONIC | **GUCY2C** | guanylate cyclase 2C | c.1364+35C>A | NM_004963.3 | NA | NA | NA | NA | NA | NA | NA | NA | MODIFIER | NA | NA | NA | NA | 142 | 141 | 0 | 0 | 131 | 108 | 23 | 17.6 |
| Chr12_p12.3 | 16053861 | C | T | INTRONIC | **STRAP** | serine/threonine kinase receptor associated protein | c.926-20C>T | NM_007178.3 | NA | NA | NA | NA | NA | NA | NA | NA | MODIFIER | NA | NA | NA | NA | 38 | 38 | 0 | 0 | 54 | 37 | 17 | 31.5 |
| Chr12_p13.31 | 6883798 | C | A | MISSENSE | **LAG3** | lymphocyte activating 3 | c.549C>A | LAG3:NM_002286:exon4:c.C549A:p.D183E | NA | NA | NA | NA | NA | T | N | N | MODERATE | T | T | T | 0.103 | 253 | 253 | 0 | 0 | 236 | 176 | 60 | 25.4 |
| Chr12_p13.31 | 6883860 | G | T | MISSENSE | **LAG3** | lymphocyte activating 3 | c.611G>T | LAG3:NM_002286:exon4:c.G611T:p.R204L | NA | NA | NA | NA | NA | D | D | N | MODERATE | T | T | T | 0.694 | 291 | 290 | 1 | 0.34 | 240 | 180 | 60 | 25.0 |
| Chr12_p13.31 | 6946612 | G | T | MISSENSE | **LEPREL2** | prolyl 3-hydroxylase 3 | c.1031G>T | NM_014262.3 | NA | NA | NA | NA | NA | T | N | N | MODERATE | 0 | T | T | 0.763 | 120 | 120 | 0 | 0 | 105 | 91 | 14 | 13.3 |
| Chr12_p13.33 | 2659078 | G | T | INTRONIC | **CACNA1C** | calcium voltage-gated channel subunit alpha1 C | c.1391-31G>T,c.1391-31G>T,c.1391-31G>T,c.1391-31G>T,c.1391-31G>T,c.1391-31G>T,c.1391-31G>T,c.1391-31G>T,c.1391-31G>T,c.1391-31G>T,c.1391-31G>T,c.1391-31G>T,c.1391-31G>T,c.1391-31G>T,c.1391-31G>T,c.1391-31G>T,c.1391-31G>T,c.1391-31G>T | NM_001129839.1 | NA | NA | NA | NA | NA | NA | NA | NA | MODIFIER | NA | NA | NA | NA | 482 | 482 | 0 | 0 | 389 | 299 | 88 | 22.6 |
| Chr12_p13.33 | 2676972 | C | T | INTRONIC | **CACNA1C** | calcium voltage-gated channel subunit alpha1 C | c.1895+12C>T, c.1895+12C>T, c.1895+12C>T, c.1895+12C>T, c.1895+12C>T, c.1895+12C>T, c.1895+12C>T, c.1895+12C>T, c.1895+12C>T, c.1895+12C>T, c.1895+12C>T, c.1895+12C>T, c.1895+12C>T, c.1895+12C>T, c.1895+12C>T, c.1895+12C>T, c.1895+12C>T, c.1895+12C>T | NM_001129839.1 | NA | NA | NA | NA | NA | NA | NA | NA | MODIFIER | NA | NA | NA | NA | 159 | 158 | 1 | 0.63 | 127 | 108 | 18 | 14.2 |
| Chr12_p13.33 | 2908447 | C | A | INTRONIC | **FKBP4** | FK506 binding protein 4 | c.671+37C>A | NM_002014.3 | NA | NA | NA | NA | NA | NA | NA | NA | MODIFIER | NA | NA | NA | NA | 117 | 117 | 0 | 0 | 129 | 111 | 17 | 13.2 |
| Chr12_q13.12 | 50399177 | T | C | INTRONIC-SS-ACR | **RACGAP1** | Rac GTPase activating protein 1 | c.289-2A>G,c.289-2A>G,c.289-2A>G | NM_013277.3 | NA | NA | NA | NA | NA | NA | NA | NA | HIGH | NA | NA | NA | NA | 115 | 115 | 0 | 0 | 94 | 74 | 20 | 21.3 |
| Chr12_q13.13 | 52369107 | G | A | 5UTR | **ACVR1B** | activin A receptor type 1B | c.-7G>A | ACVR1B:NM_004302:exon2:c.G150A:p.G50G,ACVR1B:NM_020328:exon2:c.G150A:p.G50G | NA | NA | NA | NA | NA | NA | NA | NA | MODIFIER | NA | NA | NA | NA | 145 | 145 | 0 | 0 | 120 | 79 | 40 | 33.3 |
| Chr12_q13.13 | 52369107 | G | A | SILENT | **ACVR1B** | activin A receptor type 1B | c.150G>A | ACVR1B:NM_004302:exon2:c.G150A:p.G50G,ACVR1B:NM_020328:exon2:c.G150A:p.G50G | NA | NA | NA | NA | NA | NA | NA | NA | LOW | NA | NA | NA | NA | 145 | 145 | 0 | 0 | 120 | 79 | 40 | 33.3 |
| Chr12_q13.13 | 52986187 | T | C | MISSENSE | **KRT72** | keratin 72 | c.791A>G,c.791A>G,c.791A>G | KRT72:NM_001146225:exon4:c.A791G:p.Y264C,KRT72:NM_001146226:exon4:c.A791G:p.Y264C,KRT72:NM_080747:exon4:c.A791G:p.Y264C | chr12*52986187*52986187*T*C*1*1*Brain_cancer | NA | rs12833456 | 3.87E-01 | 3.73E-01 | D | D | N | MODERATE | D | T | T | 0.233 | 113 | 111 | 0 | 0 | 101 | 95 | 6 | 5.9 |
| Chr12_q13.2 | 55361698 | A | T | INTRONIC | **TESPA1** | thymocyte expressed, positive selection associated 1 | c.207-22T>A,c.207-22T>A,c.-208-22T>A | NM_014796.2,NM_001261844.1 | NA | NA | NA | NA | NA | NA | NA | NA | MODIFIER | NA | NA | NA | NA | 89 | 89 | 0 | 0 | 84 | 63 | 21 | 25.0 |
| Chr12_q13.3 | 56845089 | A | G | MISSENSE | **MIP** | major intrinsic protein of lens fiber | c.767T>C | MIP:NM_012064:exon4:c.T767C:p.V256A | NA | NA | NA | NA | NA | T | N | D | MODERATE | D | T | D | 0.99 | 28 | 28 | 0 | 0 | 30 | 20 | 10 | 33.3 |
| Chr12_q13.3 | 56997395 | C | A | MISSENSE | **BAZ2A** | bromodomain adjacent to zinc finger domain 2A | c.3134G>T | BAZ2A:NM_001300905:exon17:c.G3128T:p.R1043L,BAZ2A:NM_013449:exon17:c.G3134T:p.R1045L | NA | NA | NA | NA | NA | T | D | D | MODERATE | T | T | T | 0.997 | 199 | 199 | 0 | 0 | 174 | 116 | 55 | 31.6 |
| Chr12_q21.31 | 80626849 | T | G | MISSENSE | **OTOGL** | otogelin like | c.762T>G | OTOGL:NM_173591:exon8:c.T762G:p.I254M | NA | NA | NA | NA | NA | T | N | NA | MODERATE | NA | NA | NA | NA | 131 | 131 | 0 | 0 | 133 | 92 | 41 | 30.8 |
| Chr12_q23.1 | 100042151 | C | A | INTRONIC | **ANKS1B** | ankyrin repeat and sterile alpha motif domain containing 1B | c.1272+6694G>T | FAM71C:NM_153364:exon1:c.C199A:p.R67R | NA | NA | NA | NA | NA | NA | NA | NA | MODIFIER | NA | NA | NA | NA | 271 | 270 | 0 | 0 | 159 | 106 | 53 | 33.3 |
| Chr12_q23.1 | 100042151 | C | A | SILENT | **FAM71C** | family with sequence similarity 71 member C | c.199C>A | FAM71C:NM_153364:exon1:c.C199A:p.R67R | NA | NA | NA | NA | NA | NA | NA | NA | LOW | NA | NA | NA | 0.955 | 271 | 270 | 0 | 0 | 159 | 106 | 53 | 33.3 |
| Chr12_q23.2 | 101776874 | G | A | MISSENSE | **UTP20** | UTP20, small subunit processome component | c.7712G>A | UTP20:NM_014503:exon59:c.G7712A:p.C2571Y | NA | NA | NA | NA | NA | T_lc | N | N | MODERATE | T | T | T | 0.004 | 59 | 59 | 0 | 0 | 49 | 33 | 16 | 32.7 |
| Chr12_q24.11 | 109186580 | G | T | MISSENSE | **SSH1** | slingshot protein phosphatase 1 | c.1408C>A,c.1375C>A,c.1375C>A | SSH1:NM_001161331:exon13:c.C1408A:p.R470S,SSH1:NM_001161330:exon14:c.C1375A:p.R459S,SSH1:NM_018984:exon14:c.C1375A:p.R459S | NA | NA | NA | NA | NA | D | N | N | MODERATE | T | T | T | 0.851 | 57 | 57 | 0 | 0 | 49 | 32 | 17 | 34.7 |
| Chr12_q24.22 | 117672445 | C | T | 3UTR | **NOS1** | nitric oxide synthase 1 | c.*1992G>A | NOS1:NM_001204213:exon20:c.G2152A:p.E718K,NOS1:NM_001204214:exon20:c.G2152A:p.E718K,NOS1:NM_000620:exon21:c.G3160A:p.E1054K,NOS1:NM_001204218:exon22:c.G3262A:p.E1088K | NA | NA | NA | NA | NA | NA | NA | NA | MODIFIER | NA | NA | NA | NA | 85 | 85 | 0 | 0 | 70 | 42 | 27 | 38.6 |
| Chr12_q24.22 | 117672445 | C | T | MISSENSE | **NOS1** | nitric oxide synthase 1 | c.3160G>A | NOS1:NM_001204213:exon20:c.G2152A:p.E718K,NOS1:NM_001204214:exon20:c.G2152A:p.E718K,NOS1:NM_000620:exon21:c.G3160A:p.E1054K,NOS1:NM_001204218:exon22:c.G3262A:p.E1088K | NA | NA | NA | NA | NA | T | N | D | MODERATE | T | T | T | 0.988 | 85 | 85 | 0 | 0 | 70 | 42 | 27 | 38.6 |
| Chr12_q24.31 | 122255803 | G | T | SILENT | **SETD1B** | SET domain containing 1B | c.3282G>T | SETD1B:NM_015048:exon10:c.G3282T:p.S1094S | NA | NA | NA | NA | NA | NA | NA | NA | LOW | NA | NA | NA | NA | 341 | 340 | 0 | 0 | 274 | 195 | 79 | 28.8 |
| Chr12_q24.31 | 124218528 | G | C | MISSENSE | **ATP6V0A2** | ATPase H+ transporting V0 subunit a2 | c.704G>C | ATP6V0A2:NM_012463:exon7:c.G704C:p.G235A | NA | NA | NA | NA | NA | T | D | D | MODERATE | D | D | D | 1 | 72 | 72 | 0 | 0 | 54 | 35 | 18 | 33.3 |

Supplementary Table S2: List of single nucleotide variants identified in SCC-R cells.

| Map Location | Genomic position | Reference allele | Alternate allele | Mutation type | Gene symbol | Protein | Nucleotide change | Description | OncoMD | COSMIC | dbSNP | ExAC | 1000G | SIFT | CONDEL | LRT | VEP | FATHMM | METASVM | METALR | PhastCons7 way | DP in SCC-S | Reference read in SCC-S | Alternative read in SCC-S | Alternate allele frequency in SCC-S (%) | DP in SCC-R | Reference read in SCC-R | Alternate read in SCC-R | Alternate allele frequency in SCC-R (%) |
|---|---|---|---|---|---|---|---|---|---|---|---|---|---|---|---|---|---|---|---|---|---|---|---|---|---|---|---|---|---|
| Chr12_q24.33 | 132393785 | A | T | INTRONIC-SS-DNR-PRX | **ULK1** | unc-51 like autophagy activating kinase 1 | c.666+3A>T | NM_003565.2 | NA | NA | NA | NA | NA | NA | NA | NA | LOW | NA | NA | NA | NA | 154 | 153 | 0 | 0 | 104 | 67 | 36 | 34.6 |
| Chr13_q12.12\|13_q12.1-_q12.3 | 25281375 | G | A | INTRONIC | **ATP12A** | ATPase H+/K+ transporting non-gastric alpha2 subunit | c.2357-42G>A | NM_001185085.1,NM_001676.5 | NA | NA | NA | NA | NA | NA | NA | NA | MODIFIER | NA | NA | NA | NA | 150 | 149 | 0 | 0 | 105 | 63 | 40 | 38.1 |
| Chr13_q12.2 | 28624321 | T | C | MISSENSE | **FLT3** | fms related tyrosine kinase 3 | c.653A>G | FLT3:NM_004119:exon6:c.A653G:p.E218G | NA | NA | NA | NA | NA | T | N | N | MODERATE | T | T | T | 0.979 | 130 | 130 | 0 | 0 | 126 | 110 | 14 | 11.1 |
| Chr13_q12.3 | 29284901 | T | G | MISSENSE | **SLC46A3** | solute carrier family 46 member 3 | c.1140A>C | SLC46A3:NM_001135919:exon4:c.A1140C:p.E380D,SLC46A3:NM_181785:exon4:c.A1140C:p.E380D | NA | NA | NA | NA | NA | D | D | D | MODERATE | T | T | T | 0.89 | 212 | 212 | 0 | 0 | 158 | 141 | 15 | 9.5 |
| Chr13_q12.3 | 29284903 | C | T | MISSENSE | **SLC46A3** | solute carrier family 46 member 3 | c.1138G>A | SLC46A3:NM_001135919:exon4:c.G1138A:p.E380K,SLC46A3:NM_181785:exon4:c.G1138A:p.E380K | chr13*29284903*29284903*C*T*1*1*Renal_cancer | NA | rs781609491 | 8.25E-06 | NA | D | D | D | MODERATE | T | T | T | 0.85 | 215 | 215 | 0 | 0 | 161 | 143 | 16 | 9.9 |
| Chr13_q13.1 | 32905055 | G | A | INTRONIC-SS-ACR | **BRCA2** | BRCA2, DNA repair associated | c.682-1G>A | NM_000059.3 | NA | NA | NA | NA | NA | NA | NA | NA | HIGH | NA | NA | NA | NA | 43 | 43 | 0 | 0 | 31 | 25 | 6 | 19.4 |
| Chr13_q13.3 | 35926440 | T | C | SILENT | **NBEA** | neurobeachin | c.6159T>C | NBEA:NM_015678:exon38:c.T6159C:p.A2053A | NA | NA | NA | NA | NA | NA | NA | NA | LOW | NA | NA | NA | NA | 56 | 55 | 0 | 0 | 39 | 22 | 17 | 43.6 |
| Chr13_q14.11 | 40230379 | G | T | INTRONIC | **COG6** | component of oligomeric golgi complex 6 | c.153+363G>T, c.153+363G>T | NM_020751.2 | NA | NA | NA | NA | NA | NA | NA | NA | MODIFIER | NA | NA | NA | NA | 50 | 50 | 0 | 0 | 52 | 37 | 15 | 28.9 |
| Chr13_q14.11 | 41533039 | A | C | SILENT | **ELF1** | E74 like ETS transcription factor 1 | c.186T>G,c.186T>G | ELF1:NM_001145353:exon2:c.T186G:p.T62T,ELF1:NM_172373:exon3:c.T186G:p.T62T | NA | NA | NA | NA | NA | NA | NA | NA | LOW | NA | NA | NA | NA | 201 | 200 | 0 | 0 | 178 | 159 | 18 | 10.1 |
| Chr13_q14.3 | 53602926 | C | T | 5UTR | **OLFM4** | olfactomedin 4 | c.-46C>T | NM_006418.4 | NA | NA | rs756519933 | 2.54E-05 | NA | NA | NA | NA | MODIFIER | NA | NA | NA | NA | 77 | 77 | 0 | 0 | 61 | 27 | 34 | 55.7 |
| Chr13_q22.2 | 76334888 | C | G | 5UTR | **LMO7** | LIM domain 7 | c.-669C>G | NM_015842.2 | NA | NA | NA | NA | NA | NA | NA | NA | MODIFIER | NA | NA | NA | NA | 170 | 170 | 0 | 0 | 99 | 71 | 26 | 26.3 |
| Chr13_q22.2 | 76334888 | C | G | INTRONIC | **LMO7** | LIM domain 7 | c.367-180C>G | NM_005358.5 | NA | NA | NA | NA | NA | NA | NA | NA | MODIFIER | NA | NA | NA | NA | 170 | 170 | 0 | 0 | 99 | 71 | 26 | 26.3 |
| Chr13_q22.3 | 77631245 | A | G | INTRONIC | **MYCBP2** | MYC binding protein 2, E3 ubiquitin protein ligase | c.13330-17T>C | NM_015057.4 | NA | NA | NA | NA | NA | NA | NA | NA | MODIFIER | NA | NA | NA | NA | 116 | 116 | 0 | 0 | 85 | 50 | 33 | 38.8 |
| Chr13_q22.3 | 77632418 | G | T | MISSENSE | **MYCBP2** | MYC binding protein 2, E3 ubiquitin protein ligase | c.13264C>A | MYCBP2:NM_015057:exon78:c.C13264A:p.Q4422K | NA | NA | NA | NA | NA | NA | NA | D | MODERATE | T | T | T | 0.991 | 132 | 131 | 0 | 0 | 108 | 66 | 42 | 38.9 |
| Chr13_q22.3 | 77760036 | C | A | MISSENSE | **MYCBP2** | MYC binding protein 2, E3 ubiquitin protein ligase | c.4414G>T | MYCBP2:NM_015057:exon31:c.G4414T:p.V1472F | NA | NA | NA | NA | NA | NA | NA | D | MODERATE | T | T | T | 1 | 161 | 160 | 0 | 0 | 105 | 55 | 50 | 47.6 |
| Chr13_q32.3 | 99575568 | G | A | SILENT | **DOCK9** | dedicator of cytokinesis 9 | c.474C>T,c.474C>T | DOCK9:NM_001130049:exon5:c.C477T:p.V159V,DOCK9:NM_001130050:exon5:c.C474T:p.V158V | chr13*99575568*99575568*C*T*1*1*Colorectal_cancer | chr13*99575568*99575568*COSM1368203*G*A*1*large_intestine*1\|chr13*99575568*99575568*COSM1368205*G*A*1*large_intestine*1\|chr13*99575568*99575568*COSM1368204*G*A*1*large_intestine*1 | rs12428661 | 1.81E-01 | 1.68E-01 | NA | NA | NA | LOW | NA | NA | NA | NA | 212 | 212 | 0 | 0 | 128 | 122 | 6 | 4.7 |
| Chr14_q11.2 | 23873106 | C | G | INTRONIC | **MYH6** | myosin heavy chain 6 | c.736-119G>C | NM_002471.3 | NA | NA | NA | NA | NA | NA | NA | NA | MODIFIER | NA | NA | NA | NA | 42 | 42 | 0 | 0 | 35 | 19 | 16 | 45.7 |
| Chr14_q12 | 24634337 | C | T | INTRONIC | **IRF9** | interferon regulatory factor 9 | c.991+173C>T | NM_006084.4 | NA | NA | NA | NA | NA | NA | NA | NA | MODIFIER | NA | NA | NA | NA | 248 | 244 | 1 | 0.4 | 241 | 160 | 80 | 33.2 |
| Chr14_q24.1 | 68040453 | G | T | INTRONIC | **PLEKHH1** | pleckstrin homology, MyTH4 and FERM domain containing H1 | c.1840-65G>T | NM_020715.2 | NA | NA | NA | NA | NA | NA | NA | NA | MODIFIER | NA | NA | NA | NA | 68 | 68 | 0 | 0 | 60 | 40 | 20 | 33.3 |
| Chr14_q32.2 | 96794794 | C | A | MISSENSE | **ATG2B** | autophagy related 2B | c.2053G>T | ATG2B:NM_018036:exon14:c.G2053T:p.D685Y | NA | NA | NA | NA | NA | D | D | U | MODERATE | T | T | T | 1 | 155 | 155 | 0 | 0 | 98 | 73 | 24 | 24.5 |
| Chr14_q32.31 | 102695693 | T | C | MISSENSE | **MOK** | MOK protein kinase | c.1103A>G,c.1193A>G | MOK:NM_001272011:exon11:c.A1103G:p.Q368R,MOK:NM_014226:exon12:c.A1193G:p.Q398R | chr14*102695693*102695693*T*C*1*1*Colorectal_cancer | chr14*102695693*102695693*COSM147846*T*C*1*stomach*1 | rs2236493 | 2.36E-01 | 2.99E-01 | T | N | N | MODERATE | T | T | T | 0.646 | 124 | 124 | 0 | 0 | 96 | 90 | 6 | 6.3 |
| Chr15_q11.2 | 22369277 | T | C | SILENT | **OR4M2** | olfactory receptor family 4 subfamily M member 2 | c.702T>C | OR4M2:NM_001004719:exon1:c.T702C:p.N234N | NA | NA | NA | NA | NA | NA | NA | NA | LOW | NA | NA | NA | 0.154 | 431 | 430 | 1 | 0.23 | 377 | 364 | 11 | 2.9 |
| Chr15_q12 | 25940144 | G | T | SILENT | **ATP10A** | ATPase phospholipid transporting 10A (putative) | c.2910C>A | ATP10A:NM_024490:exon14:c.C2910A:p.P970P | NA | NA | NA | NA | NA | NA | NA | NA | LOW | NA | NA | NA | NA | 108 | 108 | 0 | 0 | 111 | 91 | 20 | 18.0 |
| Chr15_q12 | 26107875 | G | C | SILENT | **ATP10A** | ATPase phospholipid transporting 10A (putative) | c.369C>G | ATP10A:NM_024490:exon1:c.C369G:p.A123A | NA | NA | NA | NA | NA | NA | NA | NA | LOW | NA | NA | NA | NA | 95 | 95 | 0 | 0 | 97 | 60 | 37 | 38.1 |
| Chr15_q12 | 27122776 | T | C | INTRONIC | **GABRA5** | gamma-aminobutyric acid type A receptor alpha5 subunit | c.87-3217T>C,c.87-3217T>C | NM_001165037.1 | NA | NA | NA | NA | NA | NA | NA | NA | MODIFIER | NA | NA | NA | NA | 160 | 159 | 0 | 0 | 146 | 56 | 90 | 61.6 |
| Chr15_q15.1 | 40293410 | C | T | SILENT | **EIF2AK4** | eukaryotic translation initiation factor 2 alpha kinase 4 | c.3144C>T | EIF2AK4:NM_001013703:exon21:c.C3144T:p.D1048D | NA | NA | NA | NA | NA | NA | NA | NA | LOW | NA | NA | NA | 1 | 45 | 45 | 0 | 0 | 37 | 25 | 12 | 32.4 |
| Chr15_q15.3 | 43821169 | C | T | MISSENSE | **MAP1A** | microtubule associated protein 1A | c.7498C>T | MAP1A:NM_002373:exon4:c.C7498T:p.P2500S | NA | NA | NA | NA | NA | NA | NA | NA | MODERATE | NA | NA | NA | NA | 221 | 221 | 0 | 0 | 186 | 124 | 61 | 32.8 |
| Chr15_q21.1 | 45695562 | G | A | MISSENSE | **SPATA5L1** | spermatogenesis associated 5 like 1 | c.935G>A | SPATA5L1:NM_024063:exon1:c.G935A:p.R312Q | NA | NA | NA | NA | NA | D | D | D | MODERATE | D | D | D | 0.891 | 189 | 189 | 0 | 0 | 188 | 75 | 111 | 59.0 |
| Chr15_q22.31 | 66727453 | A | G | MISSENSE | **MAP2K1** | mitogen-activated protein kinase kinase 1 | c.169A>G | MAP2K1:NM_002755:exon2:c.A169G:p.K57E | chr15*66727409*66727456*TTGATGAGCAGCAGCGAAAGCGCCTTGAGGCCTTTCTTACCCAGAAGC*del*1*1*Leukemia\|chr15*66727453*66727453*A*G*1*1*Leukemia | NA | NA | NA | NA | D | D | D | MODERATE | D | D | D | 1 | 62 | 62 | 0 | 0 | 85 | 33 | 52 | 61.2 |
| Chr15_q23 | 69347763 | C | A | MISSENSE | **NOX5** | NADPH oxidase 5 | c.2089C>A,c.1984C>A | NOX5:NM_001184779:exon15:c.C2005A:p.L669M,NOX5:NM_001184780:exon15:c.C1984A:p.L662M,NOX5:NM_024505:exon15:c.C2089A:p.L697M | NA | NA | NA | NA | NA | T | D | D | MODERATE | D | D | D | 0.997 | 100 | 99 | 0 | 0 | 141 | 123 | 17 | 12.1 |
| Chr15_q24.1 | 73879887 | T | C | SILENT | **NPTN** | neuroplastin | c.684A>G,c.336A>G | NPTN:NM_001161364:exon3:c.A336G:p.A112A,NPTN:NM_017455:exon3:c.A336G:p.A112A,NPTN:NM_001161363:exon4:c.A684G:p.A228A,NPTN:NM_012428:exon4:c.A684G:p.A228A | NA | NA | NA | NA | NA | NA | NA | NA | LOW | NA | NA | NA | NA | 37 | 37 | 0 | 0 | 55 | 40 | 15 | 27.3 |
| Chr15_q24.1 | 74000839 | G | A | INTRONIC | **CD276** | CD276 molecule | c.1504+25G>A, c.850+25G>A | NM_025240.2 | NA | NA | rs2291011 | 4.26E-01 | 3.23E-01 | NA | NA | NA | MODIFIER | NA | NA | NA | NA | 93 | 92 | 0 | 0 | 80 | 75 | 5 | 6.3 |
| Chr15_q24.1 | 75116737 | G | A | MISSENSE | **LMAN1L** | lectin, mannose binding 1 like | c.1369G>A | LMAN1L:NM_021819:exon13:c.G1369A:p.A457T | NA | NA | NA | NA | NA | T | N | N | MODERATE | T | T | T | 0.043 | 188 | 188 | 0 | 0 | 178 | 127 | 50 | 28.1 |
| Chr15_q25.1 | 80215818 | G | T | 5UTR | **ST20-MTHFS** | ST20-MTHFS readthrough | c.-36C>A | NM_001199760.1 | NA | NA | NA | NA | NA | NA | NA | NA | MODIFIER | NA | NA | NA | NA | 117 | 115 | 1 | 0.85 | 120 | 82 | 37 | 30.8 |
| Chr15_q25.3 | 85327326 | G | T | MISSENSE | **ZNF592** | zinc finger protein 592 | c.1420G>T | ZNF592:NM_014630:exon4:c.G1420T:p.A474S | NA | NA | NA | NA | NA | T | N | N | MODERATE | T | T | T | 0.018 | 267 | 267 | 0 | 0 | 248 | 177 | 71 | 28.6 |
| Chr15_q25.3 | 85658656 | T | A | INTRONIC | **PDE8A** | phosphodiesterase 8A | c.1351-14T>A,c.1213-14T>A,c.1135-14T>A | NM_001243137.1 | NA | NA | NA | NA | NA | NA | NA | NA | MODIFIER | NA | NA | NA | NA | 84 | 83 | 0 | 0 | 82 | 60 | 22 | 26.8 |
| Chr15_q26.1 | 90270498 | G | T | NONSENSE | **WDR93** | WD repeat domain 93 | c.991G>T,c.991G>T | WDR93:NM_001284395:exon9:c.G991T:p.E331X,WDR93:NM_020212:exon9:c.G991T:p.E331X | NA | NA | NA | NA | NA | NA | NA | NA | HIGH | NA | NA | NA | 0.051 | 73 | 73 | 0 | 0 | 84 | 26 | 58 | 69.1 |
| Chr15_q26.1 | 93588446 | C | T | MISSENSE | **RGMA** | repulsive guidance molecule family member a | c.1159G>A,c.1135G>A,c.1087G>A,c.1087G>A, c.1087G>A | RGMA:NM_001166286:exon3:c.G1087A:p.V363I,RGMA:NM_001166283:exon4:c.G1159A:p.V387I,RGMA:NM_001166287:exon4:c.G1087A:p.V363I,RGMA:NM_001166288:exon4:c.G1087A:p.V363I,RGMA:NM_001166289:exon4:c.G1087A:p.V363I,RGMA:NM_020211:exon4:c.G1135A:p.V379I | NA | NA | NA | NA | NA | D | D | D | MODERATE | D | D | D | 0.999 | 216 | 215 | 1 | 0.46 | 145 | 49 | 94 | 64.8 |
| Chr16_p11.2 | 30134345 | C | A | INTRONIC | **MAPK3** | mitogen-activated protein kinase 3 | c.170+16G>T,c.170+16G>T,c.170+16G>T | NM_002746.2 | NA | NA | NA | NA | NA | NA | NA | NA | MODIFIER | NA | NA | NA | NA | 37 | 35 | 0 | 0 | 49 | 32 | 12 | 24.5 |
| Chr16_p11.2 | 31477560 | G | A | MISSENSE | **ARMC5** | armadillo repeat containing 5 | c.2158G>A,c.2443G>A | ARMC5:NM_001105247:exon6:c.G2158A:p.V720I,ARMC5:NM_001301820:exon7:c.G2254A:p.V752I,ARMC5:NM_001288767:exon8:c.G2443A:p.V815I | NA | NA | NA | NA | NA | T | N | N | MODERATE | T | T | T | 0.003 | 154 | 154 | 0 | 0 | 202 | 141 | 58 | 28.7 |
| Chr16_p11.2 | 31477560 | G | A | 3UTR | **ARMC5** | armadillo repeat containing 5 | c.*1038G>A | ARMC5:NM_001105247:exon6:c.G2158A:p.V720I,ARMC5:NM_001301820:exon7:c.G2254A:p.V752I,ARMC5:NM_001288767:exon8:c.G2443A:p.V815I | NA | NA | NA | NA | NA | NA | NA | NA | MODIFIER | NA | NA | NA | NA | 154 | 154 | 0 | 0 | 202 | 141 | 58 | 28.7 |
| Chr16_p12.1 | 27476086 | G | A | SILENT | **GTF3C1** | general transcription factor IIIC subunit 1 | c.5427C>T,c.5427C>T | GTF3C1:NM_001286242:exon34:c.C5427T:p.A1809A,GTF3C1:NM_001520:exon34:c.C5427T:p.A1809A | NA | NA | NA | NA | NA | NA | NA | NA | LOW | NA | NA | NA | NA | 45 | 45 | 0 | 0 | 71 | 59 | 12 | 16.9 |
| Chr16_p12.3 | 20330996 | G | A | MISSENSE | **GP2** | glycoprotein 2 | c.512C>T,c.962C>T,c.521C>T | GP2:NM_001007242:exon5:c.C512T:p.A171V,GP2:NM_001007241:exon6:c.C521T:p.A174V,GP2:NM_001502:exon6:c.C953T:p.A318V,GP2:NM_001007240:exon7:c.C962T:p.A321V | NA | NA | NA | NA | NA | D | D | NA | MODERATE | NA | NA | NA | NA | 106 | 106 | 0 | 0 | 113 | 75 | 36 | 31.9 |
| Chr16_p12.3 | 20360368 | G | T | MISSENSE | **UMOD** | uromodulin | c.354C>A,c.402C>A | UMOD:NM_001008389:exon3:c.C255A:p.N85K,UMOD:NM_003361:exon3:c.C255A:p.N85K,UMOD:NM_001278614:exon4:c.C354A:p.N118K | NA | NA | NA | NA | NA | D | D | D | MODERATE | D | D | D | 0.104 | 100 | 100 | 0 | 0 | 135 | 77 | 57 | 42.2 |
| Chr16_p12.3 | 20792256 | A | C | INTRONIC | **ERI2** | ERI1 exoribonuclease family member 2 | c.783-40A>C,c.783-40A>C,c.895-200T>G | NM_080663.2 | NA | NA | NA | NA | NA | NA | NA | NA | MODIFIER | NA | NA | NA | NA | 109 | 109 | 0 | 0 | 191 | 127 | 62 | 32.5 |

**Supplementary Table S2: List of single nucleotide variants identified in SCC-R cells.**

| Map Location | Genomic position | Reference allele | Alternate allele | Mutation type | Gene symbol | Protein | Nucleotide change | Description | OncoMD | COSMIC | dbSNP | ExAC | 1000G | SIFT | CONDEL | LRT | VEP | FATHMM | METASVM | METALR | PhastCons7 way | DP in SCC-S | Reference read in SCC-S | Alternative read in SCC-S | Alternate allele frequency in SCC-S (%) | DP in SCC-R | Reference read in SCC-R | Alternate read in SCC-R | Alternate allele frequency in SCC-R (%) |
|---|---|---|---|---|---|---|---|---|---|---|---|---|---|---|---|---|---|---|---|---|---|---|---|---|---|---|---|---|---|
| Chr16_p12.3 | 20802020 | T | C | INTRONIC-SS-DNR-PRX | **ACSM3** | acyl-CoA synthetase medium chain family member 3 | c.1326+10T>C | NM_005622.3 | NA | NA | NA | NA | NA | NA | NA | NA | MODIFIER | NA | NA | NA | NA | 134 | 134 | 0 | 0 | 198 | 146 | 51 | 25.8 |
| Chr16_p12.3 | 20802020 | T | C | INTRONIC | **ERI2** | ERI1 exoribonuclease family member 2 | c.815+152A>G | NM_080663.2 | NA | NA | NA | NA | NA | NA | NA | NA | MODIFIER | NA | NA | NA | NA | 134 | 134 | 0 | 0 | 198 | 146 | 51 | 25.8 |
| Chr16_p13.11 | 15702225 | C | T | MISSENSE | **KIAA0430** | meiosis regulator and mRNA stability factor 1 | c.4105G>A | KIAA0430:NM_001184998:exon21:c.G4105A:p.G1369S,KIAA0430:NM_001184999:exon21:c.G4096A:p.G1366S,KIAA0430:NM_014647:exon21:c.G4105A:p.G1369S | NA | NA | NA | NA | NA | D | D | D | MODERATE | 0 | T | T | 1 | 37 | 37 | 0 | 0 | 75 | 49 | 24 | 32.0 |
| Chr16_p13.3 | 1501750 | G | A | INTRONIC | **CLCN7** | chloride voltage-gated channel 7 | c.1282-33C>T,c.1354-33C>T | NM_001287.5 | NA | NA | rs753288093 | 1.08E-05 | NA | NA | NA | NA | MODIFIER | NA | NA | NA | NA | 78 | 78 | 0 | 0 | 80 | 54 | 26 | 32.5 |
| Chr16_p13.3 | 2239185 | G | T | INTRONIC | **CASKIN1** | CASK interacting protein 1 | c.487-27C>A | NM_020764.3 | NA | NA | NA | NA | NA | NA | NA | NA | MODIFIER | NA | NA | NA | NA | 59 | 59 | 0 | 0 | 84 | 61 | 21 | 25.0 |
| Chr16_q21 | 58324904 | G | C | MISSENSE | **PRSS54** | protease, serine 54 | c.222C>G | PRSS54:NM_001080492:exon4:c.C222G:p.S74R,PRSS54:NM_001305173:exon4:c.C222G:p.S74R | NA | NA | NA | NA | NA | D | D | D | MODERATE | T | T | T | 0.989 | 167 | 165 | 1 | 0.6 | 139 | 102 | 36 | 25.9 |
| Chr16_q22.2 | 71167116 | G | T | INTRONIC | **HYDIN** | HYDIN, axonemal central pair apparatus protein | c.1044-3390C>A,c.1095-3390C>A,c.1125-3390C>A,c.1044-3390C>A | NM_017558.4 | chr16*71167116*71167116*G*T*1*1*Stomach_(gastric)_cancer | NA | NA | NA | NA | NA | NA | NA | MODIFIER | NA | NA | NA | NA | 228 | 226 | 2 | 0.88 | 228 | 192 | 35 | 15.4 |
| Chr16_q23.3-_q24.1 | 84099277 | A | G | INTRONIC | **MBTPS1** | membrane bound transcription factor peptidase, site 1 | c.2431+18T>C | NM_003791.2 | NA | NA | NA | NA | NA | NA | NA | NA | MODIFIER | NA | NA | NA | NA | 76 | 76 | 0 | 0 | 101 | 75 | 25 | 24.8 |
| Chr16_q24.2 | 88495621 | G | A | SILENT | **ZNF469** | zinc finger protein 469 | c.1743G>A | ZNF469:NM_001127464:exon1:c.G1743A:p.P581P | chr16*88495621*88495621*G*A*1*1*Renal_cancer | NA | rs753212011 | 7.53E-05 | NA | NA | NA | NA | LOW | NA | NA | NA | NA | 281 | 281 | 0 | 0 | 193 | 128 | 64 | 33.2 |
| Chr16_q24.2 | 88709737 | C | T | 3UTR | **CYBA** | cytochrome b-245 alpha chain | c.*24G>A | NM_000101.3 | NA | NA | rs1049255 | 5.60E-01 | 4.69E-01 | NA | NA | NA | MODIFIER | NA | NA | NA | NA | 86 | 86 | 0 | 0 | 64 | 57 | 6 | 9.4 |
| Chr17_p11.2 | 18167210 | G | A | MISSENSE | **MIEF2** | mitochondrial elongation factor 2 | c.530G>A,c.497G>A | MIEF2:NM_139162:exon4:c.G497A:p.S166N,MIEF2:NM_148886:exon4:c.G530A:p.S177N | NA | NA | NA | NA | NA | T | N | N | MODERATE | T | T | T | 0.98 | 225 | 225 | 0 | 0 | 126 | 84 | 42 | 33.3 |
| Chr17_p11.2 | 21202140 | G | T | INTRONIC | **MAP2K3** | mitogen-activated protein kinase kinase 3 | c.30-50G>T,c.117-50G>T | NM_145109.2 | NA | NA | NA | NA | NA | NA | NA | NA | MODIFIER | NA | NA | NA | NA | 294 | 293 | 0 | 0 | 184 | 149 | 33 | 17.9 |
| Chr17_p11.2 | 21438546 | T | C | 3UTR | **C17orf51** | chromosome 17 open reading frame 51 | c.*15A>G | NM_001113434.3 | NA | NA | NA | NA | NA | NA | NA | NA | MODIFIER | NA | NA | NA | NA | 35 | 35 | 0 | 0 | 43 | 25 | 18 | 41.9 |
| Chr17_p13.1 | 7340587 | C | T | MISSENSE | **TMEM102** | transmembrane protein 102 | c.1289C>T | TMEM102:NM_178518:exon3:c.C1289T:p.A430V | NA | NA | NA | NA | NA | T | D | D | MODERATE | T | T | T | 0.99 | 104 | 103 | 0 | 0 | 103 | 85 | 18 | 17.5 |
| Chr17_p13.1 | 7352146 | C | T | INTRONIC | **CHRNB1** | cholinergic receptor nicotinic beta 1 subunit | c.820+39C>T | NM_000747.2 | NA | NA | NA | NA | NA | NA | NA | NA | MODIFIER | NA | NA | NA | NA | 116 | 116 | 0 | 0 | 101 | 73 | 28 | 27.7 |
| Chr17_p13.1 | 7722365 | C | T | MISSENSE | **DNAH2** | dynein axonemal heavy chain 2 | c.10799C>T | DNAH2:NM_020877:exon71:c.C10799T:p.T3600I | chr17*7722365*7722365*C*T*1*1*Brain_cancer | NA | rs7213894 | 6.06E-01 | 6.11E-01 | NA | NA | N | MODERATE | T | T | T | 0.996 | 72 | 72 | 0 | 0 | 62 | 54 | 7 | 11.3 |
| Chr17_p13.1 | 9538734 | C | A | MISSENSE | **WDR16** | cilia and flagella associated protein 52 | c.1333C>A,c.1129C>A | CFAP52:NM_001080556:exon10:c.C1129A:p.Q377K,CFAP52:NM_145054:exon11:c.C1333A:p.Q445K | NA | NA | NA | NA | NA | T | N | N | MODERATE | T | T | T | 0.995 | 36 | 36 | 0 | 0 | 28 | 18 | 10 | 35.7 |
| Chr17_p13.1 | 10399847 | T | C | MISSENSE | **MYH1** | myosin heavy chain 1 | c.4676A>G | MYH1:NM_005963:exon34:c.A4676G:p.E1559G | NA | NA | NA | NA | NA | D | D | U | MODERATE | D | D | D | 0.999 | 404 | 402 | 0 | 0 | 311 | 220 | 90 | 28.9 |
| Chr17_p13.3 | 3300936 | C | A | MISSENSE | **OR1E1** | olfactory receptor family 1 subfamily E member 1 | c.769G>T | OR1E1:NM_003553:exon1:c.G769T:p.G257C | NA | NA | NA | NA | NA | D | D | D | MODERATE | T | T | T | 0.192 | 185 | 185 | 0 | 0 | 149 | 113 | 35 | 23.5 |
| Chr17_q11.2 | 30611885 | A | G | INTRONIC | **RHBDL3** | rhomboid like 3 | c.294+49A>G | NM_138328.2 | NA | NA | rs60163475 | 1.97E-01 | 1.88E-01 | NA | NA | NA | MODIFIER | NA | NA | NA | NA | 72 | 72 | 0 | 0 | 36 | 32 | 4 | 11.1 |
| Chr17_q21.1 | 38320197 | G | T | MISSENSE | **CASC3** | cancer susceptibility 3 | c.1249G>T | CASC3:NM_007359:exon7:c.G1249T:p.A417S | NA | NA | NA | NA | NA | T_lc | N | D | MODERATE | 0 | T | T | 0.998 | 130 | 129 | 0 | 0 | 117 | 80 | 37 | 31.6 |
| Chr17_q21.2 | 39884419 | C | T | INTRONIC | **HAP1** | huntingtin associated protein 1 | c.1200+34G>A, c.1224+34G>A | NM_001079870.1,NM_001079871.1 | NA | NA | rs4796692 | 7.98E-01 | 8.10E-01 | NA | NA | NA | MODIFIER | NA | NA | NA | NA | 112 | 111 | 0 | 0 | 133 | 127 | 6 | 4.5 |
| Chr17_q21.2 | 39884583 | G | A | MISSENSE-SS-PRX | **HAP1** | huntingtin associated protein 1 | c.1070C>T,c.1094C>T | HAP1:NM_001079870:exon7:c.C1094T:p.S365L,HAP1:NM_001079871:exon7:c.C1070T:p.S357L,HAP1:NM_177977:exon7:c.C1070T:p.S357L | chr17*39884583*39884583*G*A*1*1*Brain_cancer | NA | rs4796693 | 7.76E-01 | 7.73E-01 | D | N | N | MODERATE | T | T | T | 0.816 | 68 | 68 | 0 | 0 | 70 | 65 | 5 | 7.1 |
| Chr17_q21.2 | 40030016 | T | C | INTRONIC | **ACLY** | ATP citrate lyase | c.2612+48A>G, c.2642+48A>G | NM_001096.2 | NA | NA | NA | NA | NA | NA | NA | NA | MODIFIER | NA | NA | NA | NA | 25 | 25 | 0 | 0 | 17 | 8 | 9 | 52.9 |
| Chr17_q21.31 | 41935100 | G | A | INTRONIC | **CD300LG** | CD300 molecule like family member g | c.885+576G>A, c.784-58G>A,c.886-58G>A | NM_001168322.1 | NA | NA | NA | NA | NA | NA | NA | NA | MODIFIER | NA | NA | NA | NA | 67 | 67 | 0 | 0 | 63 | 29 | 34 | 54.0 |
| Chr17_q21.31 | 41935100 | G | A | 3UTR | **CD300LG** | CD300 molecule like family member g | c.*197G>A | NM_001168324.1 | NA | NA | NA | NA | NA | NA | NA | NA | MODIFIER | NA | NA | NA | NA | 67 | 67 | 0 | 0 | 63 | 29 | 34 | 54.0 |
| Chr17_q21.33 | 48072069 | A | G | SILENT | **DLX3** | distal-less homeobox 3 | c.294T>C | DLX3:NM_005220:exon1:c.T294C:p.Y98Y | NA | NA | NA | NA | NA | NA | NA | NA | LOW | NA | NA | NA | 0.997 | 208 | 205 | 2 | 0.96 | 208 | 181 | 27 | 13.0 |
| Chr17_q22 | 55163976 | C | G | INTRONIC | **AKAP1** | A-kinase anchoring protein 1 | c.-25+78C>G,c.-25+1217C>G | NM_003488.3 | NA | NA | NA | NA | NA | NA | NA | NA | MODIFIER | NA | NA | NA | NA | 107 | 107 | 0 | 0 | 94 | 76 | 18 | 19.2 |
| Chr17_q22 | 56232879 | G | T | MISSENSE | **OR4D1** | olfactory receptor family 4 subfamily D member 1 | c.365G>T | OR4D1:NM_012374:exon1:c.G365T:p.R122L | NA | chr17*56232879*56232879*COSM1679943*G*A*1*ovary*1 | NA | NA | NA | D | D | D | MODERATE | T | D | D | 0.888 | 234 | 232 | 0 | 0 | 242 | 190 | 48 | 19.8 |
| Chr17_q23.1 | 57642949 | T | A | 5UTR | **DHX40** | DEAH-box helicase 40 | c.-84T>A | NM_024612.4 | NA | NA | NA | NA | NA | NA | NA | NA | MODIFIER | NA | NA | NA | NA | 171 | 170 | 0 | 0 | 197 | 145 | 51 | 25.9 |
| Chr17_q25.1 | 72588513 | A | G | MISSENSE | **C17orf77** | chromosome 17 open reading frame 77 | c.328A>G | C17orf77:NM_001302809:exon3:c.A328G:p.R110G,C17orf77:NM_152460:exon3:c.A328G:p.R110G | NA | NA | NA | NA | NA | T_lc | N | NA | MODERATE | NA | NA | NA | NA | 183 | 183 | 0 | 0 | 171 | 151 | 17 | 9.9 |
| Chr17_q25.3 | 76799880 | T | C | SILENT | **USP36** | ubiquitin specific peptidase 36 | c.2397A>G | USP36:NM_025090:exon16:c.A2397G:p.P799P | NA | NA | NA | NA | NA | NA | NA | NA | LOW | NA | NA | NA | NA | 69 | 68 | 0 | 0 | 72 | 56 | 15 | 20.8 |
| Chr18_p11.23 | 7231253 | C | G | MISSENSE | **LRRC30** | leucine rich repeat containing 30 | c.117C>G | LRRC30:NM_001105581:exon1:c.C117G:p.D39E | NA | NA | NA | NA | NA | T | N | N | MODERATE | T | T | T | 0.976 | 105 | 105 | 0 | 0 | 102 | 66 | 35 | 34.3 |
| Chr18_p11.32-_p11.31 | 2891348 | A | G | MISSENSE | **EMILIN2** | elastin microfibril interfacer 2 | c.1223A>G | EMILIN2:NM_032048:exon4:c.A1223G:p.Q408R | NA | NA | NA | NA | NA | T | N | N | MODERATE | T | T | T | 0.895 | 191 | 191 | 0 | 0 | 213 | 153 | 60 | 28.2 |
| Chr19_p12 | 24270163 | C | A | INTRONIC | **ZNF254** | zinc finger protein 254 | c.30+24C>A,c.30+24C>A,c.-2-38893C>A | NM_001278663.1,NM_001278678.1 | NA | NA | NA | NA | NA | NA | NA | NA | MODIFIER | NA | NA | NA | NA | 137 | 137 | 0 | 0 | 159 | 86 | 71 | 44.7 |
| Chr19_p13.11 | 16265217 | C | T | SILENT | **HSH2D** | hematopoietic SH2 domain containing | c.390C>T | HSH2D:NM_001291274:exon7:c.C219T:p.P73P,HSH2D:NM_032855:exon7:c.C390T:p.P130P | NA | NA | rs376371671 | NA | NA | NA | NA | NA | LOW | NA | NA | NA | NA | 31 | 31 | 0 | 0 | 26 | 19 | 7 | 26.9 |
| Chr19_p13.11 | 17444287 | G | A | SILENT | **ANO8** | anoctamin 8 | c.294C>T | ANO8:NM_020959:exon3:c.C294T:p.V98V | NA | NA | NA | NA | NA | NA | NA | NA | LOW | NA | NA | NA | NA | 334 | 333 | 0 | 0 | 276 | 208 | 66 | 23.9 |
| Chr19_p13.11 | 18234058 | A | T | MISSENSE | **MAST3** | microtubule associated serine/threonine kinase 3 | c.344A>T | MAST3:NM_015016:exon6:c.A344T:p.Q115L | NA | NA | NA | NA | NA | D | D | NA | MODERATE | NA | NA | NA | NA | 121 | 121 | 0 | 0 | 99 | 58 | 40 | 40.4 |
| Chr19_p13.12 | 15654851 | G | C | INTRONIC-SS-DNR-PRX | **CYP4F22** | cytochrome P450 family 4 subfamily F member 22 | c.1006+3G>C | NM_173483.3 | NA | NA | NA | NA | NA | NA | NA | NA | LOW | NA | NA | NA | NA | 58 | 58 | 0 | 0 | 70 | 43 | 27 | 38.6 |
| Chr19_p13.13 | 12921049 | C | A | INTRONIC | **RNASEH2A** | ribonuclease H2 subunit A | c.549+27C>A | NM_006397.2 | NA | NA | rs754951723 | 8.24E-06 | NA | NA | NA | NA | MODIFIER | NA | NA | NA | NA | 163 | 163 | 0 | 0 | 167 | 98 | 68 | 40.7 |
| Chr19_p13.2 | 7143061 | C | G | MISSENSE | **INSR** | insulin receptor | c.2308G>C,c.2272G>C | INSR:NM_001079817:exon11:c.G2272C:p.V758L,INSR:NM_000208:exon12:c.G2308C:p.V770L | NA | NA | NA | NA | NA | T | N | U | MODERATE | T | T | T | 0.142 | 85 | 85 | 0 | 0 | 101 | 62 | 38 | 37.6 |
| Chr19_p13.2 | 7621483 | C | G | INTRONIC | **PNPLA6** | patatin like phospholipase domain containing 6 | c.3096+28C>G, c.3210+28C>G, c.3015+28C>G, c.3096+28C>G, c.3240+28C>G | NM_001166111.1 | NA | NA | NA | NA | NA | NA | NA | NA | MODIFIER | NA | NA | NA | NA | 87 | 87 | 0 | 0 | 101 | 87 | 13 | 12.9 |
| Chr19_p13.2 | 7694643 | C | A | 5UTR | **PET100** | PET100 homolog | c.-77C>A | NM_001171155.1 | NA | NA | NA | NA | NA | NA | NA | NA | MODIFIER | NA | NA | NA | NA | 239 | 239 | 0 | 0 | 193 | 154 | 38 | 19.7 |
| Chr19_p13.2 | 9027653 | G | T | INTRONIC | **MUC16** | mucin 16, cell surface associated | c.36569-78C>A | NM_024690.2 | NA | NA | NA | NA | NA | NA | NA | NA | MODIFIER | NA | NA | NA | NA | 100 | 92 | 1 | 1 | 74 | 68 | 5 | 6.8 |
| Chr19_p13.2 | 10940887 | C | T | SILENT | **DNM2** | dynamin 2 | c.2364C>T,c.2376C>T,c.2376C>T,c.2364C>T | DNM2:NM_001005362:exon19:c.C2364T:p.P788P,DNM2:NM_004945:exon19:c.C2364T:p.P788P,DNM2:NM_001005360:exon20:c.C2376T:p.P792P,DNM2:NM_001005361:exon20:c.C2376T:p.P792P,DNM2:NM_001190716:exon20:c.C2376T:p.P792P | NA | chr19*10940887*10940889*COSM1717674*CCT*C*1*haematopoietic_and_lymphoid_tissue*1\|chr19*10940887*10940889*COSM1717673*CCT*C*1*haematopoietic_and_lymphoid_tissue*1 | NA | 8.31E-06 | NA | NA | NA | NA | LOW | NA | NA | NA | NA | 132 | 132 | 0 | 0 | 134 | 82 | 49 | 36.6 |
| Chr19_p13.3 | 3118955 | G | T | SILENT | **GNA11** | G protein subunit alpha 11 | c.639G>T | GNA11:NM_002067:exon5:c.G639T:p.R213R | NA | NA | NA | NA | NA | NA | NA | NA | LOW | NA | NA | NA | NA | 178 | 178 | 0 | 0 | 148 | 92 | 56 | 37.8 |
| Chr19_q13.11 | 32873802 | G | T | MISSENSE | **ZNF507** | zinc finger protein 507 | c.2675G>T | ZNF507:NM_014910:exon6:c.G2675T:p.S892I,ZNF507:NM_001136156:exon7:c.G2675T:p.S892I | NA | NA | NA | NA | NA | T | N | N | MODERATE | T | T | T | 0.005 | 94 | 93 | 1 | 1.06 | 143 | 93 | 43 | 30.1 |
| Chr19_q13.12 | 37854608 | C | T | SILENT | **HKR1** | HKR1, GLI-Kruppel zinc finger family member | c.1911C>T | HKR1:NM_181786:exon6:c.C1911T:p.C637C | NA | NA | NA | NA | NA | NA | NA | NA | LOW | NA | NA | NA | NA | 148 | 147 | 0 | 0 | 337 | 293 | 42 | 12.5 |
| Chr19_q13.2 | 38702685 | G | T | 3UTR | **DPF1** | double PHD fingers 1 | c.*18C>A,c.*18C>A,c.*18C>A | NM_004647.2 | NA | NA | NA | NA | NA | NA | NA | NA | MODIFIER | NA | NA | NA | NA | 50 | 50 | 0 | 0 | 162 | 135 | 25 | 15.4 |
| Chr19_q13.2 | 40520048 | C | G | MISSENSE | **ZNF546** | zinc finger protein 546 | c.871C>G | ZNF546:NM_001297763:exon7:c.C793G:p.H265D,ZNF546:NM_178544:exon7:c.C871G:p.H291D | NA | NA | NA | NA | NA | D | D | NA | MODERATE | NA | NA | NA | NA | 134 | 133 | 0 | 0 | 421 | 361 | 57 | 13.5 |

**Jain *et al.* , 2019. MAP2K1 is a potential therapeutic target in erlotinib resistant head and neck squamous cell carcinoma**
**Supplementary Table S2: List of single nucleotide variants identified in SCC-R cells.**

| Map Location | Genomic position | Reference allele | Alternate allele | Mutation type | Gene symbol | Protein | Nucleotide change | Description | OncoMD | COSMIC | dbSNP | ExAC | 1000G | SIFT | CONDEL | LRT | VEP | FATHMM | METASVM | METALR | PhastCons7 way | DP in SCC-S | Reference read in SCC-S | Alternative read in SCC-S | Alternate allele frequency in SCC-S (%) | DP in SCC-R | Reference read in SCC-R | Alternate read in SCC-R | Alternate allele frequency in SCC-R (%) |
|---|---|---|---|---|---|---|---|---|---|---|---|---|---|---|---|---|---|---|---|---|---|---|---|---|---|---|---|---|---|
| Chr19_q13.2 | 41770456 | G | T | INTRONIC | **HNRNPUL1** | heterogeneous nuclear ribonucleoprotein U like 1 | c.-6+1969G>T | HNRNPUL1:NM_007040:exon1:c.G48T:p.Q16H | NA | NA | NA | NA | NA | NA | NA | NA | MODIFIER | NA | NA | NA | NA | 49 | 49 | 0 | 0 | 103 | 81 | 22 | 21.4 |
| Chr19_q13.2 | 41770456 | G | T | MISSENSE | **HNRNPUL1** | heterogeneous nuclear ribonucleoprotein U like 1 | c.48G>T | HNRNPUL1:NM_007040:exon1:c.G48T:p.Q16H | NA | NA | NA | NA | NA | T | NA | D | MODERATE | T | T | T | 1 | 49 | 49 | 0 | 0 | 103 | 81 | 22 | 21.4 |
| Chr19_q13.31 | 44791768 | C | A | MISSENSE | **ZNF235** | zinc finger protein 235 | c.1820G>T | ZNF235:NM_004234:exon5:c.G1820T:p.R607L | NA | NA | NA | NA | NA | T | N | N | MODERATE | T | T | T | 0.032 | 193 | 191 | 0 | 0 | 410 | 342 | 61 | 14.9 |
| Chr19_q13.33 | 49136808 | C | A | MISSENSE | **DBP** | D-box binding PAR bZIP transcription factor | c.655G>T | DBP:NM_001352:exon3:c.G655T:p.G219C | NA | NA | NA | NA | NA | D | D | U | MODERATE | 0 | D | T | 0.996 | 117 | 116 | 1 | 0.85 | 122 | 62 | 60 | 49.2 |
| Chr19_q13.33 | 49247786 | G | A | SILENT | **IZUMO1** | izumo sperm-egg fusion 1 | c.321C>T | IZUMO1:NM_182575:exon4:c.C321T:p.F107F | NA | NA | rs8108468 | 4.77E-01 | 5.04E-01 | NA | NA | NA | LOW | NA | NA | NA | 0.002 | 71 | 71 | 0 | 0 | 56 | 52 | 4 | 7.1 |
| Chr19_q13.42 | 54759361 | T | C | MISSENSE | **LILRB5** | leukocyte immunoglobulin like receptor B5 | c.440A>G,c.740A>G | LILRB5:NM_001081443:exon4:c.A440G:p.D147G,LILRB5:NM_001081442:exon5:c.A740G:p.D247G,LILRB5:NM_006840:exon5:c.A740G:p.D247G | chr19*54759361*54759361*T*C*1*1*Brain_cancer | chr19*54759361*54759361*COSM1396263*T*C*1*large_intestine*1\|chr19*54759361*54759361*COSM1396264*T*C*1*large_intestine*1 | rs12975366 | 3.44E-01 | 2.71E-01 | D | D | N | MODERATE | T | T | T | 0.021 | 193 | 193 | 0 | 0 | 188 | 181 | 7 | 3.7 |
| Chr19_q13.42 | 54966158 | C | A | MISSENSE | **LENG8** | leukocyte receptor cluster member 8 | c.708C>A | LENG8:NM_052925:exon7:c.C708A:p.F236L | NA | NA | NA | NA | NA | D | D | D | MODERATE | T | T | T | 0.998 | 166 | 166 | 0 | 0 | 142 | 74 | 67 | 47.2 |
| Chr19_q13.42 | 55329901 | A | G | MISSENSE | **KIR3DL1** | killer cell immunoglobulin like receptor, three Ig domains and long cytoplasmic tail 1 | c.202A>G | KIR3DL1:NM_013289:exon3:c.A202G:p.I68V | chr19*55329901*55329901*A*G*1*1*Brain_cancer | NA | rs45556431 | 3.23E-01 | 5.90E-01 | T | N | NA | MODERATE | NA | NA | NA | NA | 150 | 150 | 0 | 0 | 139 | 133 | 6 | 4.3 |
| Chr19_q13.43 | 56671069 | C | A | MISSENSE | **ZNF444** | zinc finger protein 444 | c.483C>A | ZNF444:NM_001253792:exon5:c.C480A:p.S160R,ZNF444:NM_018337:exon5:c.C483A:p.S161R | NA | NA | NA | NA | NA | T | N | NA | MODERATE | NA | NA | NA | NA | 65 | 65 | 0 | 0 | 65 | 34 | 31 | 47.7 |
| Chr20_p11.23 | 17943369 | C | T | INTRONIC | **SNX5** | sorting nexin 5 | c.51+5649G>A, c.51+5649G>A | NM_152227.1 | NA | NA | NA | NA | NA | NA | NA | NA | MODIFIER | NA | NA | NA | NA | 94 | 93 | 1 | 1.06 | 98 | 68 | 28 | 28.6 |
| Chr20_p11.23 | 17943369 | C | T | EXONIC-NC | **SNORD17** | small nucleolar RNA, C/D box 17 | n.221G>A | NR_003045.1 | NA | NA | NA | NA | NA | NA | NA | NA | MODIFIER | NA | NA | NA | NA | 94 | 93 | 1 | 1.06 | 98 | 68 | 28 | 28.6 |
| Chr20_p11.23 | 18462203 | C | G | INTRONIC | **POLR3F** | RNA polymerase III subunit F | c.682-60C>G | NM_006466.2 | NA | NA | NA | NA | NA | NA | NA | NA | MODIFIER | NA | NA | NA | NA | 128 | 128 | 0 | 0 | 116 | 79 | 37 | 31.9 |
| Chr20_p12.2 | 9496136 | A | G | MISSENSE | **LAMP5** | lysosomal associated membrane protein family member 5 | c.101A>G,c.101A>G | LAMP5:NM_001199897:exon2:c.A101G:p.E34G,LAMP5:NM_012261:exon2:c.A101G:p.E34G | NA | NA | NA | NA | NA | D | D | D | MODERATE | T | T | T | 1 | 119 | 119 | 0 | 0 | 82 | 33 | 49 | 59.8 |
| Chr20_p13 | 2844712 | A | G | MISSENSE | **VPS16** | VPS16, CORVET/HOPS core subunit | c.1162A>G,c.1594A>G | VPS16:NM_080413:exon12:c.A1162G:p.T388A,VPS16:NM_022575:exon16:c.A1594G:p.T532A | NA | NA | NA | NA | NA | T | N | N | MODERATE | T | T | T | 0.967 | 160 | 158 | 0 | 0 | 116 | 64 | 52 | 44.8 |
| Chr20_q11.21 | 31291717 | G | A | INTRONIC | **COMMD7** | COMM domain containing 7 | c.526+108C>T | NM_001099339.1,NM_053041.2 | NA | NA | NA | NA | NA | NA | NA | NA | MODIFIER | NA | NA | NA | NA | 95 | 95 | 0 | 0 | 89 | 83 | 6 | 6.7 |
| Chr20_q13.12 | 42331457 | A | G | MISSENSE | **MYBL2** | MYB proto-oncogene like 2 | c.1279A>G,c.1207A>G | MYBL2:NM_001278610:exon7:c.A1207G:p.S403G,MYBL2:NM_002466:exon8:c.A1279G:p.S427G | chr20*42331457*42331457*A*G*4*2*Soft-Tissue_Sarcomas | NA | rs2070235 | 1.08E-01 | 1.57E-01 | T | N | N | MODERATE | T | T | T | 0.992 | 191 | 190 | 0 | 0 | 192 | 183 | 8 | 4.2 |
| Chr20_q13.12 | 42635384 | C | G | SILENT | **TOX2** | TOX high mobility group box family member 2 | c.237C>G,c.237C>G,c.390C>G,c.363C>G | TOX2:NM_001098796:exon3:c.C237G:p.S79S,TOX2:NM_001098797:exon3:c.C363G:p.S121S,TOX2:NM_001098798:exon3:c.C390G:p.S130S,TOX2:NM_032883:exon4:c.C237G:p.S79S | NA | NA | NA | NA | NA | NA | NA | NA | LOW | NA | NA | NA | NA | 140 | 140 | 0 | 0 | 127 | 104 | 23 | 18.1 |
| Chr20_q13.13 | 47251220 | C | A | MISSENSE | **PREX1** | phosphatidylinositol-3,4,5-trisphosphate dependent Rac exchange factor 1 | c.4261G>T | PREX1:NM_020820:exon33:c.G4261T:p.V1421L | NA | NA | NA | NA | NA | T | D | U | MODERATE | T | T | T | 0.973 | 80 | 80 | 0 | 0 | 78 | 61 | 17 | 21.8 |
| Chr20_q13.32 | 57036223 | T | C | MISSENSE | **APCDD1L** | APC down-regulated 1 like | c.1129A>G | APCDD1L:NM_153360:exon4:c.A1129G:p.S377G,APCDD1L:NM_001304787:exon5:c.A1162G:p.S388G | NA | NA | NA | NA | NA | T | N | D | MODERATE | T | T | T | 0.699 | 116 | 116 | 0 | 0 | 115 | 76 | 38 | 33.0 |
| Chr20_q13.33 | 62193445 | G | A | MISSENSE | **HELZ2** | helicase with zinc finger 2 | c.6509C>T,c.4802C>T | HELZ2:NM_033405:exon5:c.C4802T:p.T1601M,HELZ2:NM_001037335:exon11:c.C6509T:p.T2170M | chr20*62193445*62193445*G*A*7*2*Soft-Tissue_Sarcomas | NA | rs3810481 | 1.85E-01 | 2.07E-01 | D | N | D | MODERATE | D | T | T | 0.257 | 128 | 128 | 0 | 0 | 154 | 145 | 8 | 5.2 |
| Chr20_q13.33 | 62597694 | T | G | SILENT | **ZNF512B** | zinc finger protein 512B | c.834A>C | ZNF512B:NM_020713:exon5:c.A834C:p.V278V | chr20*62597694*62597694*A*C*1*1*Prostate_cancer | chr20*62597694*62597694*COSM1130578*T*G*1*prostate*1 | rs817329 | 5.35E-01 | 4.90E-01 | NA | NA | NA | LOW | NA | NA | NA | NA | 606 | 603 | 0 | 0 | 581 | 562 | 16 | 2.8 |
| Chr21_p11.2 | 10910334 | A | T | MISSENSE | **TPTE** | transmembrane phosphatase with tensin homology | c.1422T>A,c.1308T>A,c.1368T>A | TPTE:NM_001290224:exon17:c.T1008A:p.D336E,TPTE:NM_199260:exon20:c.T1308A:p.D436E,TPTE:NM_199259:exon21:c.T1368A:p.D456E,TPTE:NM_199261:exon22:c.T1422A:p.D474E | NA | NA | NA | NA | NA | T | N | NA | MODERATE | NA | NA | NA | NA | 465 | 463 | 0 | 0 | 508 | 454 | 49 | 9.7 |
| Chr21_q22.2 | 41725570 | A | T | SILENT | **DSCAM** | DS cell adhesion molecule | c.756T>A | DSCAM:NM_001271534:exon5:c.T756A:p.P252P,DSCAM:NM_001389:exon5:c.T756A:p.P252P | NA | NA | NA | NA | NA | NA | NA | NA | LOW | NA | NA | NA | NA | 65 | 65 | 0 | 0 | 56 | 39 | 17 | 30.4 |
| Chr21_q22.3 | 43826477 | G | A | INTRONIC-SS-DNR-PRX | **UBASH3A** | ubiquitin associated and SH3 domain containing A | c.167+7G>A,c.167+7G>A,c.167+7G>A | NM_001001895.2 | NA | NA | NA | NA | NA | NA | NA | NA | LOW | NA | NA | NA | NA | 48 | 48 | 0 | 0 | 61 | 27 | 33 | 54.1 |
| Chr21_q22.3 | 46309341 | T | C | MISSENSE | **ITGB2** | integrin subunit beta 2 | c.1727A>G,c.1727A>G | ITGB2:NM_000211:exon13:c.A1727G:p.E576G,ITGB2:NM_001127491:exon13:c.A1727G:p.E576G,ITGB2:NM_001303238:exon13:c.A1520G:p.E507G | NA | NA | NA | NA | NA | T | N | NA | MODERATE | NA | NA | NA | NA | 63 | 63 | 0 | 0 | 66 | 32 | 32 | 48.5 |
| Chr21_q22.3 | 47316278 | T | G | INTRONIC-SS-DNR | **PCBP3** | poly(rC) binding protein 3 | c.165+2T>G,c.165+2T>G | NM_001130141.1 | NA | NA | NA | NA | NA | NA | NA | NA | HIGH | NA | NA | NA | NA | 46 | 46 | 0 | 0 | 43 | 21 | 22 | 51.2 |
| Chr22_q11.21 | 20102510 | G | T | MISSENSE | **TRMT2A** | tRNA methyltransferase 2 homolog A | c.983C>A | TRMT2A:NM_001257994:exon5:c.C983A:p.A328D,TRMT2A:NM_022727:exon5:c.C983A:p.A328D,TRMT2A:NM_182984:exon6:c.C983A:p.A328D | NA | NA | NA | NA | NA | D | D | D | MODERATE | T | T | T | 0.998 | 118 | 116 | 0 | 0 | 132 | 126 | 6 | 4.6 |
| Chr22_q11.21 | 20103214 | A | G | MISSENSE | **TRMT2A** | tRNA methyltransferase 2 homolog A | c.689T>C | TRMT2A:NM_001257994:exon3:c.T689C:p.V230A,TRMT2A:NM_022727:exon3:c.T689C:p.V230A,TRMT2A:NM_182984:exon4:c.T689C:p.V230A | NA | NA | NA | NA | NA | D | N | D | MODERATE | T | T | T | 0.996 | 227 | 227 | 0 | 0 | 228 | 165 | 61 | 26.8 |
| Chr22_q11.23 | 25294135 | A | G | MISSENSE | **SGSM1** | small G protein signaling modulator 1 | c.2384A>G,c.2219A>G | SGSM1:NM_001098498:exon18:c.A2036G:p.D679G,SGSM1:NM_001098497:exon19:c.A2219G:p.D740G,SGSM1:NM_133454:exon19:c.A2201G:p.D734G,SGSM1:NM_001039948:exon20:c.A2384G:p.D795G | NA | NA | NA | NA | NA | T | N | U | MODERATE | T | T | T | 0.636 | 246 | 246 | 0 | 0 | 209 | 157 | 51 | 24.4 |
| Chr22_q11.23 | 25297900 | A | G | MISSENSE | **SGSM1** | small G protein signaling modulator 1 | c.2876A>G,c.2711A>G | SGSM1:NM_001098498:exon19:c.A2528G:p.E843G,SGSM1:NM_001098497:exon20:c.A2711G:p.E904G,SGSM1:NM_133454:exon20:c.A2693G:p.E898G,SGSM1:NM_001039948:exon21:c.A2876G:p.E959G | NA | NA | NA | NA | NA | D | D | D | MODERATE | T | T | T | 1 | 86 | 86 | 0 | 0 | 75 | 61 | 14 | 18.7 |
| Chr22_q12.1 | 26270359 | G | T | MISSENSE | **MYO18B** | myosin XVIIIB | c.4058G>T | MYO18B:NM_032608:exon23:c.G4058T:p.R1353L | NA | NA | NA | NA | NA | D | D | D | MODERATE | T | D | D | 0.996 | 97 | 97 | 0 | 0 | 86 | 77 | 9 | 10.5 |
| Chr22_q12.1 | 26423220 | A | T | MISSENSE | **MYO18B** | myosin XVIIIB | c.7280A>T | MYO18B:NM_032608:exon43:c.A7280T:p.K2427I | NA | NA | NA | NA | NA | D | D | N | MODERATE | D | T | T | 0.977 | 389 | 387 | 0 | 0 | 377 | 281 | 95 | 25.2 |
| Chr22_q12.3 | 32500724 | C | A | INTRONIC | **SLC5A1** | solute carrier family 5 member 1 | c.1285-49C>A,c.1666-49C>A | NM_000343.3 | NA | NA | NA | NA | NA | NA | NA | NA | MODIFIER | NA | NA | NA | NA | 83 | 83 | 0 | 0 | 99 | 81 | 17 | 17.2 |
| Chr22_q12.3 | 32810324 | A | G | MISSENSE | **BPIFC** | BPI fold containing family C | c.1490T>C | BPIFC:NM_174932:exon15:c.T1490C:p.I497T | NA | NA | NA | NA | NA | D_lc | N | N | MODERATE | T | T | T | 0 | 166 | 165 | 0 | 0 | 186 | 144 | 42 | 22.6 |
| Chr22_q13.2-_q13.31 | 44168839 | A | T | INTRONIC | **EFCAB6** | EF-hand calcium binding domain 6 | c.-105-7566T>A | EFCAB6:NM_022785:exon4:c.T284A:p.L95Q | NA | NA | NA | NA | NA | NA | NA | NA | MODIFIER | NA | NA | NA | NA | 129 | 128 | 0 | 0 | 116 | 56 | 58 | 50.0 |
| Chr22_q13.2-_q13.31 | 44168839 | A | T | MISSENSE | **EFCAB6** | EF-hand calcium binding domain 6 | c.284T>A | EFCAB6:NM_022785:exon4:c.T284A:p.L95Q | NA | NA | NA | NA | NA | D | D | N | MODERATE | T | T | T | 0.014 | 129 | 128 | 0 | 0 | 116 | 56 | 58 | 50.0 |
| Chr22_q13.31 | 45741543 | G | C | INTRONIC | **SMC1B** | structural maintenance of chromosomes 1B | c.3496-93C>G | NM_148674.3 | NA | NA | rs5765260 | NA | 3.01E-01 | NA | NA | NA | MODIFIER | NA | NA | NA | NA | 46 | 45 | 0 | 0 | 61 | 55 | 5 | 8.2 |
| ChrX_p11.21 | 54956074 | C | A | MISSENSE | **TRO** | trophinin | c.1726C>A,c.1510C>A,c.2917C>A | TRO:NM_001271183:exon10:c.C1510A:p.L504I,TRO:NM_001271184:exon11:c.C1726A:p.L576I,TRO:NM_001039705:exon12:c.C2917A:p.L973I | NA | NA | NA | NA | NA | T | N | NA | MODERATE | NA | NA | NA | NA | 137 | 136 | 0 | 0 | 126 | 53 | 72 | 57.1 |
| ChrX_p11.21 | 54956074 | C | A | INTRONIC | **TRO** | trophinin | c.1986+931C>A,c.1986+931C>A,c.795+931C>A | TRO:NM_001271183:exon10:c.C1510A:p.L504I,TRO:NM_001271184:exon11:c.C1726A:p.L576I,TRO:NM_001039705:exon12:c.C2917A:p.L973I | NA | NA | NA | NA | NA | NA | NA | NA | MODIFIER | NA | NA | NA | NA | 137 | 136 | 0 | 0 | 126 | 53 | 72 | 57.1 |
| ChrX_p11.22 | 50053625 | C | T | INTRONIC | **CCNB3** | cyclin B3 | c.204+21738C>T | CCNB3:NM_033031:exon5:c.C2456T:p.P819L | NA | NA | NA | NA | NA | NA | NA | NA | MODIFIER | NA | NA | NA | NA | 108 | 107 | 0 | 0 | 140 | 66 | 70 | 50.0 |
| ChrX_p11.22 | 50053625 | C | T | MISSENSE | **CCNB3** | cyclin B3 | c.2456C>T | CCNB3:NM_033031:exon5:c.C2456T:p.P819L | NA | NA | NA | NA | NA | T | N | N | MODERATE | T | T | T | 0.002 | 108 | 107 | 0 | 0 | 140 | 66 | 70 | 50.0 |
| ChrX_p11.23 | 47920229 | C | A | MISSENSE | **ZNF630** | zinc finger protein 630 | c.111G>T,c.69G>T | ZNF630:NM_001037735:exon3:c.G111T:p.M37I,ZNF630:NM_001190255:exon3:c.G69T:p.M23I,ZNF630:NM_001282201:exon3:c.G111T:p.M37I | NA | NA | NA | NA | NA | T | N | NA | MODERATE | NA | NA | NA | NA | 120 | 119 | 0 | 0 | 79 | 48 | 31 | 39.2 |
| ChrX_p22.2 | 10085674 | T | C | SILENT | **WWC3** | WWC family member 3 | c.1575T>C | WWC3:NM_015691:exon11:c.T1575C:p.D525D | NA | NA | rs6530368 | 9.30E-01 | 9.59E-01 | NA | NA | NA | LOW | NA | NA | NA | 0.025 | 68 | 68 | 0 | 0 | 64 | 58 | 5 | 7.8 |
| ChrX_q21.2 | 86067835 | C | A | INTRONIC | **DACH2** | dachshund family transcription factor 2 | c.1241-24C>A,c.1202-24C>A,c.740-24C>A | NM_001139515.1 | NA | NA | NA | NA | NA | NA | NA | NA | MODIFIER | NA | NA | NA | NA | 82 | 81 | 0 | 0 | 71 | 66 | 5 | 7.0 |
| ChrX_q21.31 | 91132551 | G | C | MISSENSE | **PCDH11X** | protocadherin 11 X-linked | c.1312G>C,c.1312G>C,c.1312G>C,c.1312G>C,c.1312G>C,c.1312G>C,c.1312G>C | PCDH11X:NM_001168360:exon2:c.G1312C:p.A438P,PCDH11X:NM_001168361:exon2:c.G1312C:p.A438P,PCDH11X:NM_001168362:exon2:c.G1312C:p.A438P,PCDH11X:NM_001168363:exon2:c.G1312C:p.A438P,PCDH11X:NM_032968:exon2:c.G1312C:p.A438P,PCDH11X:NM_032969:exon2:c.G1312C:p.A438P | NA | NA | NA | NA | NA | D | D | D | MODERATE | T | D | D | 0.969 | 187 | 187 | 0 | 0 | 211 | 108 | 102 | 48.3 |
| ChrX_q22.2 | 103268020 | C | T | SILENT | **H2BFWT** | H2B histone family member W, testis specific | c.213G>A | H2BFWT:NM_001002916:exon1:c.G213A:p.R71R | NA | NA | NA | NA | NA | NA | NA | NA | LOW | NA | NA | NA | NA | 111 | 111 | 0 | 0 | 97 | 47 | 49 | 50.5 |
| ChrX_q23 | 115582737 | A | G | MISSENSE | **SLC6A14** | solute carrier family 6 member 14 | c.1061A>G | SLC6A14:NM_007231:exon8:c.A1061G:p.N354S | NA | NA | NA | NA | NA | D | D | NA | MODERATE | NA | NA | NA | 1 | 66 | 65 | 0 | 0 | 58 | 54 | 4 | 6.9 |

**Supplementary Table S2: List of single nucleotide variants identified in SCC-R cells.**

| Map Location | Genomic position | Reference allele | Alternate allele | Mutation type | Gene symbol | Protein | Nucleotide change | Description | OncoMD | COSMIC | dbSNP | ExAC | 1000G | SIFT | CONDEL | LRT | VEP | FATHMM | METASVM | METALR | PhastCons7 way | DP in SCC-S | Reference read in SCC-S | Alternative read in SCC-S | Alternate allele frequency in SCC-S (%) | DP in SCC-R | Reference read in SCC-R | Alternate read in SCC-R | Alternate allele frequency in SCC-R (%) |
|---|---|---|---|---|---|---|---|---|---|---|---|---|---|---|---|---|---|---|---|---|---|---|---|---|---|---|---|---|---|
| ChrX_q27.1 | 138671973 | A | G | INTRONIC | **MCF2** | MCF.2 cell line derived transforming sequence | c.2253+21T>C, c.2550+21T>C, c.2370+21T>C, c.2598+21T>C | NM_001171876.1 | NA | NA | NA | NA | NA | NA | NA | NA | MODIFIER | NA | NA | NA | NA | 19 | 19 | 0 | 0 | 41 | 16 | 25 | 61.0 |

Jain et al., 2019. MAP2K1 is a potential therapeutic target in erlotinib resistant head and neck squamous cell carcinoma

Supplementary Table S3. List of copy number alterations and affected genes in SCC-R cells

| Gene | Chromosome | Start co-ordinate | End co-ordinate | Copy number | P-value | Q-value |
|---|---|---|---|---|---|---|
| MED18 | chr1 | 28657152 | 28661530 | 3 | 0.0E+00 | 0.0E+00 |
| PHACTR4 | chr1 | 28696247 | 28823214 | 3 | 0.0E+00 | 0.0E+00 |
| RCC1 | chr1 | 28832512 | 28864573 | 3 | 0.0E+00 | 0.0E+00 |
| TRNAU1AP | chr1 | 28879593 | 28904236 | 3 | 0.0E+00 | 0.0E+00 |
| RAB42 | chr1 | 28918964 | 28920652 | 3 | 0.0E+00 | 0.0E+00 |
| TAF12 | chr1 | 28929979 | 28948596 | 3 | 0.0E+00 | 0.0E+00 |
| GMEB1 | chr1 | 28995190 | 29041277 | 3 | 0.0E+00 | 0.0E+00 |
| YTHDF2 | chr1 | 29063519 | 29095437 | 3 | 0.0E+00 | 0.0E+00 |
| OPRD1 | chr1 | 29138890 | 29189741 | 3 | 0.0E+00 | 0.0E+00 |
| EPB41 | chr1 | 29213543 | 29442310 | 3 | 0.0E+00 | 0.0E+00 |
| TMEM200B | chr1 | 29447397 | 29448391 | 3 | 0.0E+00 | 0.0E+00 |
| SRSF4 | chr1 | 29474513 | 29508255 | 3 | 0.0E+00 | 0.0E+00 |
| MECR | chr1 | 29520485 | 29557375 | 3 | 0.0E+00 | 0.0E+00 |
| PTPRU | chr1 | 29563148 | 29652184 | 3 | 0.0E+00 | 0.0E+00 |
| MATN1 | chr1 | 31186350 | 31196470 | 3 | 0.0E+00 | 0.0E+00 |
| LAPTM5 | chr1 | 31206330 | 31230625 | 3 | 0.0E+00 | 0.0E+00 |
| SDC3 | chr1 | 31346055 | 31381346 | 3 | 0.0E+00 | 0.0E+00 |
| PUM1 | chr1 | 31406058 | 31538626 | 3 | 0.0E+00 | 0.0E+00 |
| NKAIN1 | chr1 | 31654488 | 31712441 | 3 | 0.0E+00 | 0.0E+00 |
| SNRNP40 | chr1 | 31732478 | 31769690 | 3 | 0.0E+00 | 0.0E+00 |
| ZCCHC17 | chr1 | 31782921 | 31836956 | 3 | 0.0E+00 | 0.0E+00 |
| FABP3 | chr1 | 31838634 | 31845911 | 3 | 0.0E+00 | 0.0E+00 |
| SERINC2 | chr1 | 31882543 | 31907045 | 3 | 0.0E+00 | 0.0E+00 |
| AC114494 | chr1 | 31902215 | 31902425 | 3 | 0.0E+00 | 0.0E+00 |
| TINAGL1 | chr1 | 32042792 | 32052532 | 3 | 0.0E+00 | 0.0E+00 |
| HCRTR1 | chr1 | 32084819 | 32092989 | 3 | 0.0E+00 | 0.0E+00 |
| PEF1 | chr1 | 32096307 | 32110583 | 3 | 0.0E+00 | 0.0E+00 |
| COL16A1 | chr1 | 32118190 | 32167808 | 3 | 0.0E+00 | 0.0E+00 |
| BAI2 | chr1 | 32192950 | 32230504 | 3 | 0.0E+00 | 0.0E+00 |
| SPOCD1 | chr1 | 32256193 | 32280960 | 3 | 0.0E+00 | 0.0E+00 |
| PTP4A2 | chr1 | 32374525 | 32384575 | 3 | 0.0E+00 | 0.0E+00 |
| AL136115 | chr1 | 32379330 | 32380112 | 3 | 0.0E+00 | 0.0E+00 |
| KHDRBS1 | chr1 | 32479628 | 32508278 | 3 | 0.0E+00 | 0.0E+00 |
| TMEM39B | chr1 | 32538537 | 32568277 | 3 | 0.0E+00 | 0.0E+00 |
| KPNA6 | chr1 | 32573570 | 32636459 | 3 | 0.0E+00 | 0.0E+00 |
| TXLNA | chr1 | 32645868 | 32661400 | 3 | 0.0E+00 | 0.0E+00 |
| CCDC28B | chr1 | 32667534 | 32670984 | 3 | 0.0E+00 | 0.0E+00 |
| IQCC | chr1 | 32671297 | 32673672 | 3 | 0.0E+00 | 0.0E+00 |
| DCDC2B | chr1 | 32674718 | 32681494 | 3 | 0.0E+00 | 0.0E+00 |
| TMEM234 | chr1 | 32681942 | 32686869 | 3 | 0.0E+00 | 0.0E+00 |
| EIF3I | chr1 | 32687392 | 32696782 | 3 | 0.0E+00 | 0.0E+00 |
| FAM167B | chr1 | 32712956 | 32714285 | 3 | 0.0E+00 | 0.0E+00 |
| LCK | chr1 | 32739752 | 32751291 | 3 | 0.0E+00 | 0.0E+00 |
| HDAC1 | chr1 | 32757657 | 32798743 | 3 | 0.0E+00 | 0.0E+00 |
| MARCKSL1 | chr1 | 32800220 | 32801713 | 3 | 0.0E+00 | 0.0E+00 |
| FAM229A | chr1 | 32827055 | 32827662 | 3 | 0.0E+00 | 0.0E+00 |
| TSSK3 | chr1 | 32828305 | 32829934 | 3 | 0.0E+00 | 0.0E+00 |
| BSDC1 | chr1 | 32831883 | 32860094 | 3 | 0.0E+00 | 0.0E+00 |
| ZBTB8B | chr1 | 32936235 | 32951030 | 3 | 0.0E+00 | 0.0E+00 |

Supplementary Table S3. List of copy number alterations and affected genes in SCC-R cells

| Gene | Chromosome | Start co-ordinate | End co-ordinate | Copy number | P-value | Q-value |
|---|---|---|---|---|---|---|
| ZBTB8A | chr1 | 33058480 | 33060834 | 3 | 0.0E+00 | 0.0E+00 |
| ZBTB8OS | chr1 | 33065668 | 33116218 | 3 | 0.0E+00 | 0.0E+00 |
| RBBP4 | chr1 | 33116872 | 33150053 | 3 | 0.0E+00 | 0.0E+00 |
| SYNC | chr1 | 33160480 | 33168303 | 3 | 0.0E+00 | 0.0E+00 |
| KIAA1522 | chr1 | 33207583 | 33238552 | 3 | 0.0E+00 | 0.0E+00 |
| YARS | chr1 | 33241594 | 33282865 | 3 | 0.0E+00 | 0.0E+00 |
| S100PBP | chr1 | 33291646 | 33321662 | 3 | 0.0E+00 | 0.0E+00 |
| FNDC5 | chr1 | 33329303 | 33334000 | 3 | 0.0E+00 | 0.0E+00 |
| HPCA | chr1 | 33354548 | 33363942 | 3 | 0.0E+00 | 0.0E+00 |
| TMEM54 | chr1 | 33360299 | 33366875 | 3 | 0.0E+00 | 0.0E+00 |
| RNF19B | chr1 | 33402383 | 33430287 | 3 | 0.0E+00 | 0.0E+00 |
| AK2 | chr1 | 33476394 | 33502343 | 3 | 0.0E+00 | 0.0E+00 |
| TRIM62 | chr1 | 33611435 | 33647061 | 3 | 0.0E+00 | 0.0E+00 |
| ZNF362 | chr1 | 33736051 | 33764648 | 3 | 0.0E+00 | 0.0E+00 |
| A3GALT2 | chr1 | 33772390 | 33786762 | 3 | 0.0E+00 | 0.0E+00 |
| PHC2 | chr1 | 33790495 | 33841116 | 3 | 0.0E+00 | 0.0E+00 |
| ZSCAN20 | chr1 | 33944807 | 33961077 | 3 | 0.0E+00 | 0.0E+00 |
| HMGB4 | chr1 | 34329859 | 34330379 | 3 | 0.0E+00 | 0.0E+00 |
| C1orf94 | chr1 | 34643367 | 34684444 | 3 | 0.0E+00 | 0.0E+00 |
| GJB5 | chr1 | 35222904 | 35223792 | 3 | 0.0E+00 | 0.0E+00 |
| GJB3 | chr1 | 35250312 | 35251139 | 3 | 0.0E+00 | 0.0E+00 |
| GJA4 | chr1 | 35259805 | 35260871 | 3 | 0.0E+00 | 0.0E+00 |
| SMIM12 | chr1 | 35320645 | 35321549 | 3 | 0.0E+00 | 0.0E+00 |
| DLGAP3 | chr1 | 35331516 | 35370977 | 3 | 0.0E+00 | 0.0E+00 |
| ZMYM6NB | chr1 | 35447479 | 35450977 | 3 | 0.0E+00 | 0.0E+00 |
| ZMYM6 | chr1 | 35452708 | 35496297 | 3 | 0.0E+00 | 0.0E+00 |
| ZMYM1 | chr1 | 35559524 | 35580870 | 3 | 0.0E+00 | 0.0E+00 |
| SFPQ | chr1 | 35643502 | 35658738 | 3 | 0.0E+00 | 0.0E+00 |
| ZMYM4 | chr1 | 35734589 | 35885276 | 3 | 0.0E+00 | 0.0E+00 |
| KIAA0319L | chr1 | 35900545 | 36023463 | 3 | 0.0E+00 | 0.0E+00 |
| NCDN | chr1 | 36023646 | 36032586 | 3 | 0.0E+00 | 0.0E+00 |
| TFAP2E | chr1 | 36039149 | 36060280 | 3 | 0.0E+00 | 0.0E+00 |
| PSMB2 | chr1 | 36068793 | 36107128 | 3 | 0.0E+00 | 0.0E+00 |
| C1orf216 | chr1 | 36181190 | 36181874 | 3 | 0.0E+00 | 0.0E+00 |
| CLSPN | chr1 | 36186418 | 36235583 | 3 | 0.0E+00 | 0.0E+00 |
| AGO4 | chr1 | 36274069 | 36319273 | 3 | 0.0E+00 | 0.0E+00 |
| AGO1 | chr1 | 36348932 | 36385199 | 3 | 0.0E+00 | 0.0E+00 |
| AGO3 | chr1 | 36396971 | 36521371 | 3 | 0.0E+00 | 0.0E+00 |
| TEKT2 | chr1 | 36550577 | 36553811 | 3 | 0.0E+00 | 0.0E+00 |
| ADPRHL2 | chr1 | 36554562 | 36558998 | 3 | 0.0E+00 | 0.0E+00 |
| COL8A2 | chr1 | 36563169 | 36565889 | 3 | 0.0E+00 | 0.0E+00 |
| TRAPPC3 | chr1 | 36602734 | 36615001 | 3 | 0.0E+00 | 0.0E+00 |
| MAP7D1 | chr1 | 36621984 | 36646024 | 3 | 0.0E+00 | 0.0E+00 |
| THRAP3 | chr1 | 36748190 | 36769504 | 3 | 0.0E+00 | 0.0E+00 |
| SH3D21 | chr1 | 36771912 | 36788358 | 3 | 0.0E+00 | 0.0E+00 |
| EVA1B | chr1 | 36788505 | 36788709 | 3 | 0.0E+00 | 0.0E+00 |
| STK40 | chr1 | 36807422 | 36827028 | 3 | 0.0E+00 | 0.0E+00 |
| LSM10 | chr1 | 36859409 | 36859787 | 3 | 0.0E+00 | 0.0E+00 |
| OSCP1 | chr1 | 36881631 | 36916021 | 3 | 0.0E+00 | 0.0E+00 |

Supplementary Table S3. List of copy number alterations and affected genes in SCC-R cells

| Gene | Chromosome | Start co-ordinate | End co-ordinate | Copy number | P-value | Q-value |
|---|---|---|---|---|---|---|
| MRPS15 | chr1 | 36921336 | 36929888 | 3 | 0.0E+00 | 0.0E+00 |
| CSF3R | chr1 | 36931588 | 36945222 | 3 | 0.0E+00 | 0.0E+00 |
| GRIK3 | chr1 | 37267466 | 37499709 | 3 | 0.0E+00 | 0.0E+00 |
| ZC3H12A | chr1 | 37941141 | 37949131 | 3 | 0.0E+00 | 0.0E+00 |
| MEAF6 | chr1 | 37959327 | 37980351 | 3 | 0.0E+00 | 0.0E+00 |
| SNIP1 | chr1 | 38003307 | 38019880 | 3 | 0.0E+00 | 0.0E+00 |
| DNALI1 | chr1 | 38022523 | 38030686 | 3 | 0.0E+00 | 0.0E+00 |
| GNL2 | chr1 | 38032487 | 38061494 | 3 | 0.0E+00 | 0.0E+00 |
| RSPO1 | chr1 | 38078348 | 38095688 | 3 | 0.0E+00 | 0.0E+00 |
| C1orf109 | chr1 | 38148696 | 38156084 | 3 | 0.0E+00 | 0.0E+00 |
| CDCA8 | chr1 | 38158400 | 38174175 | 3 | 0.0E+00 | 0.0E+00 |
| EPHA10 | chr1 | 38181562 | 38230727 | 3 | 0.0E+00 | 0.0E+00 |
| MANEAL | chr1 | 38259876 | 38265827 | 3 | 0.0E+00 | 0.0E+00 |
| YRDC | chr1 | 38269589 | 38273854 | 3 | 0.0E+00 | 0.0E+00 |
| C1orf122 | chr1 | 38274073 | 38274913 | 3 | 0.0E+00 | 0.0E+00 |
| MTF1 | chr1 | 38280852 | 38323313 | 3 | 0.0E+00 | 0.0E+00 |
| AL929472 | chr1 | 38292206 | 38293957 | 3 | 0.0E+00 | 0.0E+00 |
| INPP5B | chr1 | 38327994 | 38412024 | 3 | 0.0E+00 | 0.0E+00 |
| SF3A3 | chr1 | 38423930 | 38455745 | 3 | 0.0E+00 | 0.0E+00 |
| FHL3 | chr1 | 38462865 | 38465078 | 3 | 0.0E+00 | 0.0E+00 |
| UTP11L | chr1 | 38478431 | 38489397 | 3 | 0.0E+00 | 0.0E+00 |
| POU3F1 | chr1 | 38511077 | 38512354 | 3 | 0.0E+00 | 0.0E+00 |
| RRAGC | chr1 | 39305255 | 39325330 | 3 | 0.0E+00 | 0.0E+00 |
| MYCBP | chr1 | 39330297 | 39347272 | 3 | 0.0E+00 | 0.0E+00 |
| RHBDL2 | chr1 | 39352215 | 39395222 | 3 | 0.0E+00 | 0.0E+00 |
| AKIRIN1 | chr1 | 39457019 | 39469820 | 3 | 0.0E+00 | 0.0E+00 |
| NDUFS5 | chr1 | 39494351 | 39500119 | 3 | 0.0E+00 | 0.0E+00 |
| MACF1 | chr1 | 39549803 | 39951506 | 3 | 0.0E+00 | 0.0E+00 |
| KIAA0754 | chr1 | 39875897 | 39880194 | 3 | 0.0E+00 | 0.0E+00 |
| BMP8A | chr1 | 39957618 | 39991547 | 3 | 0.0E+00 | 0.0E+00 |
| PABPC4 | chr1 | 40026720 | 40041603 | 3 | 0.0E+00 | 0.0E+00 |
| HEYL | chr1 | 40092114 | 40105360 | 3 | 0.0E+00 | 0.0E+00 |
| NT5C1A | chr1 | 40124834 | 40137685 | 3 | 0.0E+00 | 0.0E+00 |
| HPCAL4 | chr1 | 40148205 | 40150276 | 3 | 0.0E+00 | 0.0E+00 |
| PPIE | chr1 | 40204521 | 40229521 | 3 | 0.0E+00 | 0.0E+00 |
| BMP8B | chr1 | 40226180 | 40254230 | 3 | 0.0E+00 | 0.0E+00 |
| OXCT2 | chr1 | 40235358 | 40236912 | 3 | 0.0E+00 | 0.0E+00 |
| TRIT1 | chr1 | 40307429 | 40349194 | 3 | 0.0E+00 | 0.0E+00 |
| MYCL | chr1 | 40363038 | 40367594 | 3 | 0.0E+00 | 0.0E+00 |
| MFSD2A | chr1 | 40421006 | 40435250 | 3 | 0.0E+00 | 0.0E+00 |
| CAP1 | chr1 | 40525107 | 40537120 | 3 | 0.0E+00 | 0.0E+00 |
| PPT1 | chr1 | 40539683 | 40562890 | 3 | 0.0E+00 | 0.0E+00 |
| RLF | chr1 | 40627063 | 40706204 | 3 | 0.0E+00 | 0.0E+00 |
| TMCO2 | chr1 | 40713581 | 40717336 | 3 | 0.0E+00 | 0.0E+00 |
| ZMPSTE24 | chr1 | 40723961 | 40758361 | 3 | 0.0E+00 | 0.0E+00 |
| COL9A2 | chr1 | 40766909 | 40782905 | 3 | 0.0E+00 | 0.0E+00 |
| SMAP2 | chr1 | 40839791 | 40887791 | 3 | 0.0E+00 | 0.0E+00 |
| ZFP69B | chr1 | 40916652 | 40929278 | 3 | 0.0E+00 | 0.0E+00 |
| ZFP69 | chr1 | 40945052 | 40961767 | 3 | 0.0E+00 | 0.0E+00 |

**Supplementary Table S3. List of copy number alterations and affected genes in SCC-R cells**

| Gene | Chromosome | Start co-ordinate | End co-ordinate | Copy number | P-value | Q-value |
|---|---|---|---|---|---|---|
| EXO5 | chr1 | 40980224 | 40981367 | 3 | 0.0E+00 | 0.0E+00 |
| ZNF684 | chr1 | 40998695 | 41013104 | 3 | 0.0E+00 | 0.0E+00 |
| RIMS3 | chr1 | 41092176 | 41107559 | 3 | 0.0E+00 | 0.0E+00 |
| NFYC | chr1 | 41157359 | 41237260 | 3 | 0.0E+00 | 0.0E+00 |
| KCNQ4 | chr1 | 41249789 | 41304213 | 3 | 0.0E+00 | 0.0E+00 |
| CITED4 | chr1 | 41327241 | 41327792 | 3 | 0.0E+00 | 0.0E+00 |
| CTPS1 | chr1 | 41447548 | 41475938 | 3 | 0.0E+00 | 0.0E+00 |
| SLFNL1 | chr1 | 41481684 | 41486332 | 3 | 0.0E+00 | 0.0E+00 |
| SCMH1 | chr1 | 41493794 | 41651911 | 3 | 0.0E+00 | 0.0E+00 |
| FOXO6 | chr1 | 41827562 | 41848615 | 3 | 0.0E+00 | 0.0E+00 |
| EDN2 | chr1 | 41944963 | 41950358 | 3 | 0.0E+00 | 0.0E+00 |
| HIVEP3 | chr1 | 41976069 | 42050491 | 3 | 0.0E+00 | 0.0E+00 |
| GUCA2B | chr1 | 42619119 | 42621288 | 3 | 0.0E+00 | 0.0E+00 |
| GUCA2A | chr1 | 42628497 | 42630495 | 3 | 0.0E+00 | 0.0E+00 |
| FOXJ3 | chr1 | 42645316 | 42776827 | 3 | 0.0E+00 | 0.0E+00 |
| RIMKLA | chr1 | 42846596 | 42880696 | 3 | 0.0E+00 | 0.0E+00 |
| ZMYND12 | chr1 | 42896366 | 42921674 | 3 | 0.0E+00 | 0.0E+00 |
| PPCS | chr1 | 42922242 | 42925567 | 3 | 0.0E+00 | 0.0E+00 |
| CCDC30 | chr1 | 42948356 | 43119677 | 3 | 0.0E+00 | 0.0E+00 |
| PPIH | chr1 | 43124064 | 43133159 | 3 | 0.0E+00 | 0.0E+00 |
| YBX1 | chr1 | 43148283 | 43166708 | 3 | 0.0E+00 | 0.0E+00 |
| CLDN19 | chr1 | 43200640 | 43205705 | 3 | 0.0E+00 | 0.0E+00 |
| LEPRE1 | chr1 | 43212389 | 43232584 | 3 | 0.0E+00 | 0.0E+00 |
| C1orf50 | chr1 | 43233006 | 43263874 | 3 | 0.0E+00 | 0.0E+00 |
| CCDC23 | chr1 | 43273084 | 43282269 | 3 | 0.0E+00 | 0.0E+00 |
| ERMAP | chr1 | 43282783 | 43308919 | 3 | 0.0E+00 | 0.0E+00 |
| ZNF691 | chr1 | 43314921 | 43317614 | 3 | 0.0E+00 | 0.0E+00 |
| SLC2A1 | chr1 | 43392272 | 43424474 | 3 | 0.0E+00 | 0.0E+00 |
| FAM183A | chr1 | 43610840 | 43622032 | 3 | 0.0E+00 | 0.0E+00 |
| EBNA1BP2 | chr1 | 43630030 | 43697906 | 3 | 0.0E+00 | 0.0E+00 |
| WDR65 | chr1 | 43649231 | 43719803 | 3 | 0.0E+00 | 0.0E+00 |
| TMEM125 | chr1 | 43738366 | 43739089 | 3 | 0.0E+00 | 0.0E+00 |
| C1orf210 | chr1 | 43748436 | 43748988 | 3 | 0.0E+00 | 0.0E+00 |
| TIE1 | chr1 | 43766723 | 43788518 | 3 | 0.0E+00 | 0.0E+00 |
| MPL | chr1 | 43803513 | 43818513 | 3 | 0.0E+00 | 0.0E+00 |
| CDC20 | chr1 | 43824979 | 43828901 | 3 | 0.0E+00 | 0.0E+00 |
| ELOVL1 | chr1 | 43829646 | 43831308 | 3 | 0.0E+00 | 0.0E+00 |
| MED8 | chr1 | 43850035 | 43854168 | 3 | 0.0E+00 | 0.0E+00 |
| SZT2 | chr1 | 43855298 | 43918046 | 3 | 0.0E+00 | 0.0E+00 |
| HYI | chr1 | 43918566 | 43919450 | 3 | 0.0E+00 | 0.0E+00 |
| PTPRF | chr1 | 43998219 | 44087693 | 3 | 0.0E+00 | 0.0E+00 |
| KDM4A | chr1 | 44118810 | 44170051 | 3 | 0.0E+00 | 0.0E+00 |
| ST3GAL3 | chr1 | 44201912 | 44395875 | 3 | 0.0E+00 | 0.0E+00 |
| ARTN | chr1 | 44401228 | 44402411 | 3 | 0.0E+00 | 0.0E+00 |
| IPO13 | chr1 | 44413267 | 44433413 | 3 | 0.0E+00 | 0.0E+00 |
| DPH2 | chr1 | 44435850 | 44438198 | 3 | 0.0E+00 | 0.0E+00 |
| B4GALT2 | chr1 | 44445658 | 44456089 | 3 | 0.0E+00 | 0.0E+00 |
| CCDC24 | chr1 | 44457268 | 44461880 | 3 | 0.0E+00 | 0.0E+00 |
| SLC6A9 | chr1 | 44463211 | 44490046 | 3 | 0.0E+00 | 0.0E+00 |

Supplementary Table S3. List of copy number alterations and affected genes in SCC-R cells

| Gene | Chromosome | Start co-ordinate | End co-ordinate | Copy number | P-value | Q-value |
|---|---|---|---|---|---|---|
| KLF17 | chr1 | 44584497 | 44596435 | 3 | 0.0E+00 | 0.0E+00 |
| DMAP1 | chr1 | 44679435 | 44686379 | 3 | 0.0E+00 | 0.0E+00 |
| ERI3 | chr1 | 44687226 | 44820699 | 3 | 0.0E+00 | 0.0E+00 |
| RNF220 | chr1 | 44877769 | 45116568 | 3 | 0.0E+00 | 0.0E+00 |
| TMEM53 | chr1 | 45101997 | 45140105 | 3 | 0.0E+00 | 0.0E+00 |
| C1orf228 | chr1 | 45155309 | 45191066 | 3 | 0.0E+00 | 0.0E+00 |
| KIF2C | chr1 | 45205501 | 45232912 | 3 | 0.0E+00 | 0.0E+00 |
| RPS8 | chr1 | 45241135 | 45244250 | 3 | 0.0E+00 | 0.0E+00 |
| BEST4 | chr1 | 45249830 | 45253339 | 3 | 0.0E+00 | 0.0E+00 |
| PLK3 | chr1 | 45266044 | 45271329 | 3 | 0.0E+00 | 0.0E+00 |
| BTBD19 | chr1 | 45274465 | 45279451 | 3 | 0.0E+00 | 0.0E+00 |
| PTCH2 | chr1 | 45286011 | 45308618 | 3 | 0.0E+00 | 0.0E+00 |
| EIF2B3 | chr1 | 45316594 | 45446847 | 3 | 0.0E+00 | 0.0E+00 |
| HECTD3 | chr1 | 45469179 | 45476872 | 3 | 0.0E+00 | 0.0E+00 |
| UROD | chr1 | 45477897 | 45481237 | 3 | 0.0E+00 | 0.0E+00 |
| ZSWIM5 | chr1 | 45484170 | 45672035 | 3 | 0.0E+00 | 0.0E+00 |
| MUTYH | chr1 | 45794900 | 45803922 | 3 | 0.0E+00 | 0.0E+00 |
| TOE1 | chr1 | 45805444 | 45809419 | 3 | 0.0E+00 | 0.0E+00 |
| TESK2 | chr1 | 45810573 | 45923479 | 3 | 0.0E+00 | 0.0E+00 |
| CCDC163P | chr1 | 45960684 | 45965319 | 3 | 0.0E+00 | 0.0E+00 |
| MMACHC | chr1 | 45965903 | 45974940 | 3 | 0.0E+00 | 0.0E+00 |
| PRDX1 | chr1 | 45977000 | 45984824 | 3 | 0.0E+00 | 0.0E+00 |
| AKR1A1 | chr1 | 46027375 | 46035643 | 3 | 0.0E+00 | 0.0E+00 |
| NASP | chr1 | 46049805 | 46083750 | 3 | 0.0E+00 | 0.0E+00 |
| CCDC17 | chr1 | 46085944 | 46089559 | 3 | 0.0E+00 | 0.0E+00 |
| GPBP1L1 | chr1 | 46093886 | 46124852 | 3 | 0.0E+00 | 0.0E+00 |
| TMEM69 | chr1 | 46156776 | 46159607 | 3 | 0.0E+00 | 0.0E+00 |
| IPP | chr1 | 46160093 | 46212085 | 3 | 0.0E+00 | 0.0E+00 |
| MAST2 | chr1 | 46269685 | 46501776 | 3 | 0.0E+00 | 0.0E+00 |
| PIK3R3 | chr1 | 46509362 | 46642108 | 3 | 0.0E+00 | 0.0E+00 |
| TSPAN1 | chr1 | 46646743 | 46651286 | 3 | 0.0E+00 | 0.0E+00 |
| POMGNT1 | chr1 | 46654363 | 46663475 | 3 | 0.0E+00 | 0.0E+00 |
| LURAP1 | chr1 | 46669127 | 46685963 | 3 | 0.0E+00 | 0.0E+00 |
| RAD54L | chr1 | 46714126 | 46743915 | 3 | 0.0E+00 | 0.0E+00 |
| LRRC41 | chr1 | 46726893 | 46768965 | 3 | 0.0E+00 | 0.0E+00 |
| UQCRH | chr1 | 46769456 | 46782220 | 3 | 0.0E+00 | 0.0E+00 |
| NSUN4 | chr1 | 46806476 | 46827569 | 3 | 0.0E+00 | 0.0E+00 |
| FAAH | chr1 | 46860071 | 46879284 | 3 | 0.0E+00 | 0.0E+00 |
| DMBX1 | chr1 | 46972613 | 46978182 | 3 | 0.0E+00 | 0.0E+00 |
| KNCN | chr1 | 47012978 | 47016888 | 3 | 0.0E+00 | 0.0E+00 |
| MKNK1 | chr1 | 47024224 | 47060740 | 3 | 0.0E+00 | 0.0E+00 |
| MOB3C | chr1 | 47075277 | 47080738 | 3 | 0.0E+00 | 0.0E+00 |
| ATPAF1 | chr1 | 47099380 | 47133967 | 3 | 0.0E+00 | 0.0E+00 |
| EFCAB14 | chr1 | 47124289 | 47183829 | 3 | 0.0E+00 | 0.0E+00 |
| TEX38 | chr1 | 47135016 | 47139128 | 3 | 0.0E+00 | 0.0E+00 |
| CYP4B1 | chr1 | 47264771 | 47284447 | 3 | 0.0E+00 | 0.0E+00 |
| CYP4Z2P | chr1 | 47310148 | 47366098 | 3 | 0.0E+00 | 0.0E+00 |
| CYP4A11 | chr1 | 47395744 | 47407140 | 3 | 0.0E+00 | 0.0E+00 |
| CYP4X1 | chr1 | 47427205 | 47515871 | 3 | 0.0E+00 | 0.0E+00 |

Supplementary Table S3. List of copy number alterations and affected genes in SCC-R cells

| Gene | Chromosome | Start co-ordinate | End co-ordinate | Copy number | P-value | Q-value |
|---|---|---|---|---|---|---|
| CYP4Z1 | chr1 | 47533170 | 47583579 | 3 | 0.0E+00 | 0.0E+00 |
| CYP4A22 | chr1 | 47603154 | 47614511 | 3 | 0.0E+00 | 0.0E+00 |
| PDZK1IP1 | chr1 | 47649613 | 47655733 | 3 | 0.0E+00 | 0.0E+00 |
| TAL1 | chr1 | 47685028 | 47691601 | 3 | 0.0E+00 | 0.0E+00 |
| STIL | chr1 | 47716859 | 47776065 | 3 | 0.0E+00 | 0.0E+00 |
| CMPK1 | chr1 | 47799658 | 47842557 | 3 | 0.0E+00 | 0.0E+00 |
| FOXD2 | chr1 | 47903818 | 47905304 | 3 | 0.0E+00 | 0.0E+00 |
| TRABD2B | chr1 | 48231519 | 48462389 | 3 | 0.0E+00 | 0.0E+00 |
| SLC5A9 | chr1 | 48688333 | 48713255 | 3 | 0.0E+00 | 0.0E+00 |
| SPATA6 | chr1 | 48764373 | 48937697 | 3 | 0.0E+00 | 0.0E+00 |
| AGBL4 | chr1 | 48999710 | 50489519 | 3 | 0.0E+00 | 0.0E+00 |
| BEND5 | chr1 | 49193475 | 49242512 | 3 | 0.0E+00 | 0.0E+00 |
| AL645730 | chr1 | 50460784 | 50460911 | 3 | 0.0E+00 | 0.0E+00 |
| ELAVL4 | chr1 | 50513731 | 50666886 | 3 | 0.0E+00 | 0.0E+00 |
| DMRTA2 | chr1 | 50884391 | 50887220 | 3 | 0.0E+00 | 0.0E+00 |
| FAF1 | chr1 | 50907085 | 51425507 | 3 | 0.0E+00 | 0.0E+00 |
| CDKN2C | chr1 | 51436061 | 51439912 | 3 | 0.0E+00 | 0.0E+00 |
| C1orf185 | chr1 | 51567774 | 51613396 | 3 | 0.0E+00 | 0.0E+00 |
| RNF11 | chr1 | 51702447 | 51736905 | 3 | 0.0E+00 | 0.0E+00 |
| TTC39A | chr1 | 51753829 | 51810734 | 3 | 0.0E+00 | 0.0E+00 |
| EPS15 | chr1 | 51822350 | 51984978 | 3 | 0.0E+00 | 0.0E+00 |
| OSBPL9 | chr1 | 52082724 | 52253466 | 3 | 0.0E+00 | 0.0E+00 |
| NRD1 | chr1 | 52254905 | 52344351 | 3 | 0.0E+00 | 0.0E+00 |
| RAB3B | chr1 | 52385647 | 52442835 | 3 | 0.0E+00 | 0.0E+00 |
| TXNDC12 | chr1 | 52486536 | 52520763 | 3 | 0.0E+00 | 0.0E+00 |
| KTI12 | chr1 | 52498353 | 52499379 | 3 | 0.0E+00 | 0.0E+00 |
| BTF3L4 | chr1 | 52525538 | 52552366 | 3 | 0.0E+00 | 0.0E+00 |
| ZFYVE9 | chr1 | 52698810 | 52811937 | 3 | 0.0E+00 | 0.0E+00 |
| CC2D1B | chr1 | 52818753 | 52830418 | 3 | 0.0E+00 | 0.0E+00 |
| ORC1 | chr1 | 52838850 | 52867950 | 3 | 0.0E+00 | 0.0E+00 |
| PRPF38A | chr1 | 52870431 | 52882450 | 3 | 0.0E+00 | 0.0E+00 |
| ZCCHC11 | chr1 | 52889457 | 52991922 | 3 | 0.0E+00 | 0.0E+00 |
| GPX7 | chr1 | 53068063 | 53074097 | 3 | 0.0E+00 | 0.0E+00 |
| FAM159A | chr1 | 53099213 | 53122765 | 3 | 0.0E+00 | 0.0E+00 |
| SELRC1 | chr1 | 53153389 | 53164030 | 3 | 0.0E+00 | 0.0E+00 |
| ZYG11B | chr1 | 53192265 | 53287342 | 3 | 0.0E+00 | 0.0E+00 |
| ZYG11A | chr1 | 53308497 | 53358597 | 3 | 0.0E+00 | 0.0E+00 |
| ECHDC2 | chr1 | 53362067 | 53387293 | 3 | 0.0E+00 | 0.0E+00 |
| SCP2 | chr1 | 53393055 | 53516410 | 3 | 0.0E+00 | 0.0E+00 |
| PODN | chr1 | 53527824 | 53547763 | 3 | 0.0E+00 | 0.0E+00 |
| SLC1A7 | chr1 | 53553677 | 53608128 | 3 | 0.0E+00 | 0.0E+00 |
| CPT2 | chr1 | 53662647 | 53679263 | 3 | 0.0E+00 | 0.0E+00 |
| C1orf123 | chr1 | 53680317 | 53686294 | 3 | 0.0E+00 | 0.0E+00 |
| MAGOH | chr1 | 53692723 | 53704198 | 3 | 0.0E+00 | 0.0E+00 |
| LRP8 | chr1 | 53712558 | 53793490 | 3 | 0.0E+00 | 0.0E+00 |
| DMRTB1 | chr1 | 53925069 | 53932343 | 3 | 0.0E+00 | 0.0E+00 |
| GLIS1 | chr1 | 53972246 | 54060562 | 3 | 0.0E+00 | 0.0E+00 |
| NDC1 | chr1 | 54233511 | 54304051 | 3 | 0.0E+00 | 0.0E+00 |
| YIPF1 | chr1 | 54325696 | 54356280 | 3 | 0.0E+00 | 0.0E+00 |

Supplementary Table S3. List of copy number alterations and affected genes in SCC-R cells

| Gene | Chromosome | Start co-ordinate | End co-ordinate | Copy number | P-value | Q-value |
|---|---|---|---|---|---|---|
| DIO1 | chr1 | 54359912 | 54375697 | 3 | 0.0E+00 | 0.0E+00 |
| HSPB11 | chr1 | 54387329 | 54405775 | 3 | 0.0E+00 | 0.0E+00 |
| LRRC42 | chr1 | 54417728 | 54433757 | 3 | 0.0E+00 | 0.0E+00 |
| LDLRAD1 | chr1 | 54474579 | 54483958 | 3 | 0.0E+00 | 0.0E+00 |
| TMEM59 | chr1 | 54497815 | 54518909 | 3 | 0.0E+00 | 0.0E+00 |
| TCEANC2 | chr1 | 54519691 | 54562095 | 3 | 0.0E+00 | 0.0E+00 |
| AL161915 | chr1 | 54570102 | 54570568 | 3 | 0.0E+00 | 0.0E+00 |
| CDCP2 | chr1 | 54605176 | 54618603 | 3 | 0.0E+00 | 0.0E+00 |
| AL357673 | chr1 | 54636740 | 54637094 | 3 | 0.0E+00 | 0.0E+00 |
| CYB5RL | chr1 | 54640345 | 54661274 | 3 | 0.0E+00 | 0.0E+00 |
| MRPL37 | chr1 | 54649739 | 54691069 | 3 | 0.0E+00 | 0.0E+00 |
| SSBP3 | chr1 | 54692800 | 54871642 | 3 | 0.0E+00 | 0.0E+00 |
| C1orf191 | chr1 | 54703733 | 54704042 | 3 | 0.0E+00 | 0.0E+00 |
| ACOT11 | chr1 | 55013918 | 55100486 | 3 | 0.0E+00 | 0.0E+00 |
| FAM151A | chr1 | 55076040 | 55089150 | 3 | 0.0E+00 | 0.0E+00 |
| MROH7 | chr1 | 55118542 | 55175861 | 3 | 0.0E+00 | 0.0E+00 |
| TTC4 | chr1 | 55181512 | 55207217 | 3 | 0.0E+00 | 0.0E+00 |
| PARS2 | chr1 | 55223403 | 55224900 | 3 | 0.0E+00 | 0.0E+00 |
| TTC22 | chr1 | 55246879 | 55266853 | 3 | 0.0E+00 | 0.0E+00 |
| C1orf177 | chr1 | 55271787 | 55307574 | 3 | 0.0E+00 | 0.0E+00 |
| DHCR24 | chr1 | 55317919 | 55352825 | 3 | 0.0E+00 | 0.0E+00 |
| TMEM61 | chr1 | 55446695 | 55457806 | 3 | 0.0E+00 | 0.0E+00 |
| BSND | chr1 | 55464793 | 55474382 | 3 | 0.0E+00 | 0.0E+00 |
| PCSK9 | chr1 | 55505546 | 55529209 | 3 | 0.0E+00 | 0.0E+00 |
| USP24 | chr1 | 55534646 | 55680798 | 3 | 0.0E+00 | 0.0E+00 |
| OMA1 | chr1 | 58939542 | 59004969 | 3 | 0.0E+00 | 0.0E+00 |
| MYSM1 | chr1 | 59125694 | 59165761 | 3 | 0.0E+00 | 0.0E+00 |
| FGGY | chr1 | 59787159 | 60228367 | 3 | 0.0E+00 | 0.0E+00 |
| HOOK1 | chr1 | 60280680 | 60338666 | 3 | 0.0E+00 | 0.0E+00 |
| CYP2J2 | chr1 | 60359351 | 60392355 | 3 | 0.0E+00 | 0.0E+00 |
| C1orf87 | chr1 | 60456312 | 60538408 | 3 | 0.0E+00 | 0.0E+00 |
| NFIA | chr1 | 61330991 | 61921160 | 3 | 0.0E+00 | 0.0E+00 |
| TM2D1 | chr1 | 62149118 | 62190988 | 3 | 0.0E+00 | 0.0E+00 |
| INADL | chr1 | 62228622 | 62628718 | 3 | 0.0E+00 | 0.0E+00 |
| L1TD1 | chr1 | 62672228 | 62677065 | 3 | 0.0E+00 | 0.0E+00 |
| KANK4 | chr1 | 62703957 | 62747358 | 3 | 0.0E+00 | 0.0E+00 |
| USP1 | chr1 | 62905491 | 62916624 | 3 | 0.0E+00 | 0.0E+00 |
| DOCK7 | chr1 | 62920884 | 63153921 | 3 | 0.0E+00 | 0.0E+00 |
| ANGPTL3 | chr1 | 63063171 | 63070498 | 3 | 0.0E+00 | 0.0E+00 |
| ATG4C | chr1 | 63269372 | 63329877 | 3 | 0.0E+00 | 0.0E+00 |
| FOXD3 | chr1 | 63788687 | 63790131 | 3 | 0.0E+00 | 0.0E+00 |
| ALG6 | chr1 | 63836560 | 63902634 | 3 | 0.0E+00 | 0.0E+00 |
| ITGB3BP | chr1 | 63912409 | 64059381 | 3 | 0.0E+00 | 0.0E+00 |
| EFCAB7 | chr1 | 63991197 | 64038210 | 3 | 0.0E+00 | 0.0E+00 |
| DLEU2L | chr1 | 64014846 | 64015302 | 3 | 0.0E+00 | 0.0E+00 |
| PGM1 | chr1 | 64089111 | 64125462 | 3 | 0.0E+00 | 0.0E+00 |
| ROR1 | chr1 | 64239975 | 64644513 | 3 | 0.0E+00 | 0.0E+00 |
| UBE2U | chr1 | 64669613 | 64732952 | 3 | 0.0E+00 | 0.0E+00 |
| CACHD1 | chr1 | 64936484 | 65157256 | 3 | 0.0E+00 | 0.0E+00 |

Supplementary Table S3. List of copy number alterations and affected genes in SCC-R cells

| Gene | Chromosome | Start co-ordinate | End co-ordinate | Copy number | P-value | Q-value |
|---|---|---|---|---|---|---|
| RAVER2 | chr1 | 65210936 | 65296682 | 3 | 0.0E+00 | 0.0E+00 |
| JAK1 | chr1 | 65300181 | 65352071 | 3 | 0.0E+00 | 0.0E+00 |
| AK4 | chr1 | 65614185 | 65691760 | 3 | 0.0E+00 | 0.0E+00 |
| DNAJC6 | chr1 | 65720221 | 65878782 | 3 | 0.0E+00 | 0.0E+00 |
| LEPR | chr1 | 65886369 | 66102705 | 3 | 0.0E+00 | 0.0E+00 |
| LEPROT | chr1 | 65895593 | 65897590 | 3 | 0.0E+00 | 0.0E+00 |
| PDE4B | chr1 | 66378938 | 66838400 | 3 | 0.0E+00 | 0.0E+00 |
| SGIP1 | chr1 | 66999959 | 67208838 | 3 | 0.0E+00 | 0.0E+00 |
| AL139147 | chr1 | 67142650 | 67142828 | 3 | 0.0E+00 | 0.0E+00 |
| TCTEX1D1 | chr1 | 67220290 | 67243117 | 3 | 0.0E+00 | 0.0E+00 |
| INSL5 | chr1 | 67263626 | 67266889 | 3 | 0.0E+00 | 0.0E+00 |
| MIER1 | chr1 | 67390701 | 67452251 | 3 | 0.0E+00 | 0.0E+00 |
| SLC35D1 | chr1 | 67469956 | 67519690 | 3 | 0.0E+00 | 0.0E+00 |
| C1orf141 | chr1 | 67558730 | 67592970 | 3 | 0.0E+00 | 0.0E+00 |
| IL23R | chr1 | 67633755 | 67724895 | 3 | 0.0E+00 | 0.0E+00 |
| IL12RB2 | chr1 | 67785929 | 67861750 | 3 | 0.0E+00 | 0.0E+00 |
| SERBP1 | chr1 | 67878910 | 67896021 | 3 | 0.0E+00 | 0.0E+00 |
| GADD45A | chr1 | 68151146 | 68153416 | 3 | 0.0E+00 | 0.0E+00 |
| GNG12 | chr1 | 68171132 | 68173380 | 3 | 0.0E+00 | 0.0E+00 |
| DIRAS3 | chr1 | 68512218 | 68512966 | 3 | 0.0E+00 | 0.0E+00 |
| WLS | chr1 | 68564251 | 68698041 | 3 | 0.0E+00 | 0.0E+00 |
| RPE65 | chr1 | 68895425 | 68915706 | 3 | 0.0E+00 | 0.0E+00 |
| DEPDC1 | chr1 | 68942705 | 68962760 | 3 | 0.0E+00 | 0.0E+00 |
| LRRC7 | chr1 | 70034163 | 70587568 | 3 | 0.0E+00 | 0.0E+00 |
| LRRC40 | chr1 | 70611457 | 70671193 | 3 | 0.0E+00 | 0.0E+00 |
| SRSF11 | chr1 | 70687318 | 70716437 | 3 | 0.0E+00 | 0.0E+00 |
| ANKRD13C | chr1 | 70728314 | 70820044 | 3 | 0.0E+00 | 0.0E+00 |
| HHLA3 | chr1 | 70820648 | 70833337 | 3 | 0.0E+00 | 0.0E+00 |
| CTH | chr1 | 70877032 | 70904901 | 3 | 0.0E+00 | 0.0E+00 |
| PTGER3 | chr1 | 71318397 | 71513206 | 3 | 0.0E+00 | 0.0E+00 |
| ZRANB2 | chr1 | 71530708 | 71546694 | 3 | 0.0E+00 | 0.0E+00 |
| NEGR1 | chr1 | 71873105 | 72748140 | 3 | 0.0E+00 | 0.0E+00 |
| FAM110C | chr2 | 41459 | 46354 | 1 | 0.0E+00 | 0.0E+00 |
| SH3YL1 | chr2 | 218830 | 264036 | 1 | 0.0E+00 | 0.0E+00 |
| ACP1 | chr2 | 264939 | 277224 | 1 | 0.0E+00 | 0.0E+00 |
| FAM150B | chr2 | 280012 | 287797 | 1 | 0.0E+00 | 0.0E+00 |
| TMEM18 | chr2 | 669560 | 677479 | 1 | 0.0E+00 | 0.0E+00 |
| SNTG2 | chr2 | 946666 | 1371295 | 1 | 0.0E+00 | 0.0E+00 |
| TPO | chr2 | 1418142 | 1546409 | 1 | 0.0E+00 | 0.0E+00 |
| PXDN | chr2 | 1637926 | 1748191 | 1 | 0.0E+00 | 0.0E+00 |
| MYT1L | chr2 | 1794916 | 1983586 | 1 | 0.0E+00 | 0.0E+00 |
| TSSC1 | chr2 | 3193039 | 3381485 | 1 | 0.0E+00 | 0.0E+00 |
| TRAPPC12 | chr2 | 3391442 | 3488612 | 1 | 0.0E+00 | 0.0E+00 |
| ADI1 | chr2 | 3502750 | 3523351 | 1 | 0.0E+00 | 0.0E+00 |
| RNASEH1 | chr2 | 3595614 | 3605867 | 1 | 0.0E+00 | 0.0E+00 |
| RPS7 | chr2 | 3623274 | 3628598 | 1 | 0.0E+00 | 0.0E+00 |
| COLEC11 | chr2 | 3651175 | 3691709 | 1 | 0.0E+00 | 0.0E+00 |
| ALLC | chr2 | 3718694 | 3750096 | 1 | 0.0E+00 | 0.0E+00 |
| DCDC2C | chr2 | 3751386 | 3826585 | 1 | 0.0E+00 | 0.0E+00 |

Supplementary Table S3. List of copy number alterations and affected genes in SCC-R cells

| Gene | Chromosome | Start co-ordinate | End co-ordinate | Copy number | P-value | Q-value |
|---|---|---|---|---|---|---|
| SOX11 | chr2 | 5832864 | 5834182 | 1 | 0.0E+00 | 0.0E+00 |
| CMPK2 | chr2 | 6980627 | 7005797 | 1 | 0.0E+00 | 0.0E+00 |
| RSAD2 | chr2 | 7006031 | 7036109 | 1 | 0.0E+00 | 0.0E+00 |
| RNF144A | chr2 | 7076524 | 7208417 | 1 | 0.0E+00 | 0.0E+00 |
| AC007464 | chr2 | 8178651 | 8178822 | 1 | 0.0E+00 | 0.0E+00 |
| ID2 | chr2 | 8822295 | 8823055 | 1 | 0.0E+00 | 0.0E+00 |
| KIDINS220 | chr2 | 8866922 | 8967241 | 1 | 0.0E+00 | 0.0E+00 |
| MBOAT2 | chr2 | 8998719 | 9143823 | 1 | 0.0E+00 | 0.0E+00 |
| ASAP2 | chr2 | 9347193 | 9543527 | 1 | 0.0E+00 | 0.0E+00 |
| ITGB1BP1 | chr2 | 9546916 | 9563671 | 1 | 0.0E+00 | 0.0E+00 |
| CPSF3 | chr2 | 9563779 | 9613206 | 1 | 0.0E+00 | 0.0E+00 |
| IAH1 | chr2 | 9614685 | 9634920 | 1 | 0.0E+00 | 0.0E+00 |
| ADAM17 | chr2 | 9630294 | 9695809 | 1 | 0.0E+00 | 0.0E+00 |
| YWHAQ | chr2 | 9725439 | 9770622 | 1 | 0.0E+00 | 0.0E+00 |
| TAF1B | chr2 | 9983531 | 10074109 | 1 | 0.0E+00 | 0.0E+00 |
| GRHL1 | chr2 | 10085227 | 10140837 | 1 | 0.0E+00 | 0.0E+00 |
| KLF11 | chr2 | 10183816 | 10192594 | 1 | 0.0E+00 | 0.0E+00 |
| CYS1 | chr2 | 10198987 | 10220380 | 1 | 0.0E+00 | 0.0E+00 |
| AC104794 | chr2 | 10223934 | 10226327 | 1 | 0.0E+00 | 0.0E+00 |
| RRM2 | chr2 | 10262690 | 10269566 | 1 | 0.0E+00 | 0.0E+00 |
| C2orf48 | chr2 | 10281998 | 10350666 | 1 | 0.0E+00 | 0.0E+00 |
| HPCAL1 | chr2 | 10559894 | 10566940 | 1 | 0.0E+00 | 0.0E+00 |
| AC007249 | chr2 | 10594016 | 10595690 | 1 | 0.0E+00 | 0.0E+00 |
| NOL10 | chr2 | 10712213 | 10830051 | 1 | 0.0E+00 | 0.0E+00 |
| ATP6V1C2 | chr2 | 10862955 | 10925250 | 1 | 0.0E+00 | 0.0E+00 |
| PDIA6 | chr2 | 10927385 | 10977735 | 1 | 0.0E+00 | 0.0E+00 |
| AC092687 | chr2 | 10987580 | 10995172 | 1 | 0.0E+00 | 0.0E+00 |
| KCNF1 | chr2 | 11052612 | 11054091 | 1 | 0.0E+00 | 0.0E+00 |
| AC062028 | chr2 | 11245721 | 11269042 | 1 | 0.0E+00 | 0.0E+00 |
| C2orf50 | chr2 | 11273416 | 11284193 | 1 | 0.0E+00 | 0.0E+00 |
| PQLC3 | chr2 | 11295285 | 11318032 | 1 | 0.0E+00 | 0.0E+00 |
| ROCK2 | chr2 | 11323407 | 11484237 | 1 | 0.0E+00 | 0.0E+00 |
| E2F6 | chr2 | 11586660 | 11605960 | 1 | 0.0E+00 | 0.0E+00 |
| GREB1 | chr2 | 11680651 | 11780649 | 1 | 0.0E+00 | 0.0E+00 |
| NTSR2 | chr2 | 11798581 | 11810247 | 1 | 0.0E+00 | 0.0E+00 |
| LPIN1 | chr2 | 11817719 | 11964923 | 1 | 0.0E+00 | 0.0E+00 |
| AC096559 | chr2 | 12339231 | 12339428 | 1 | 0.0E+00 | 0.0E+00 |
| TRIB2 | chr2 | 12858404 | 12880921 | 1 | 0.0E+00 | 0.0E+00 |
| AC011897 | chr2 | 14774123 | 14789525 | 1 | 0.0E+00 | 0.0E+00 |
| NBAS | chr2 | 15307195 | 15701412 | 1 | 0.0E+00 | 0.0E+00 |
| DDX1 | chr2 | 15732002 | 15771057 | 1 | 0.0E+00 | 0.0E+00 |
| AC008271 | chr2 | 15840465 | 15884490 | 1 | 0.0E+00 | 0.0E+00 |
| MYCN | chr2 | 16080624 | 16086270 | 1 | 0.0E+00 | 0.0E+00 |
| FAM49A | chr2 | 16734166 | 16769503 | 1 | 0.0E+00 | 0.0E+00 |
| RAD51AP2 | chr2 | 17692091 | 17699656 | 1 | 0.0E+00 | 0.0E+00 |
| VSNL1 | chr2 | 17773308 | 17836708 | 1 | 0.0E+00 | 0.0E+00 |
| SMC6 | chr2 | 17846715 | 17927222 | 1 | 0.0E+00 | 0.0E+00 |
| GEN1 | chr2 | 17941169 | 17963224 | 1 | 0.0E+00 | 0.0E+00 |
| MSGN1 | chr2 | 17997785 | 17998448 | 1 | 0.0E+00 | 0.0E+00 |

Supplementary Table S3. List of copy number alterations and affected genes in SCC-R cells

| Gene | Chromosome | Start co-ordinate | End co-ordinate | Copy number | P-value | Q-value |
|---|---|---|---|---|---|---|
| KCNS3 | chr2 | 18112308 | 18113753 | 1 | 0.0E+00 | 0.0E+00 |
| RDH14 | chr2 | 18736460 | 18741791 | 1 | 0.0E+00 | 0.0E+00 |
| NT5C1B | chr2 | 18745010 | 18770812 | 1 | 0.0E+00 | 0.0E+00 |
| OSR1 | chr2 | 19552027 | 19553650 | 1 | 0.0E+00 | 0.0E+00 |
| TTC32 | chr2 | 20096754 | 20101663 | 1 | 0.0E+00 | 0.0E+00 |
| WDR35 | chr2 | 20113300 | 20189860 | 1 | 0.0E+00 | 0.0E+00 |
| MATN3 | chr2 | 20192777 | 20212311 | 1 | 0.0E+00 | 0.0E+00 |
| LAPTM4A | chr2 | 20232898 | 20251381 | 1 | 0.0E+00 | 0.0E+00 |
| AC098828 | chr2 | 20302499 | 20302620 | 1 | 0.0E+00 | 0.0E+00 |
| SDC1 | chr2 | 20401885 | 20424672 | 1 | 0.0E+00 | 0.0E+00 |
| PUM2 | chr2 | 20451307 | 20527140 | 1 | 0.0E+00 | 0.0E+00 |
| AC012065 | chr2 | 20699666 | 20700989 | 1 | 0.0E+00 | 0.0E+00 |
| HS1BP3 | chr2 | 20798017 | 20850868 | 1 | 0.0E+00 | 0.0E+00 |
| C2orf43 | chr2 | 20884547 | 21001249 | 1 | 0.0E+00 | 0.0E+00 |
| APOB | chr2 | 21224573 | 21266735 | 1 | 0.0E+00 | 0.0E+00 |
| TDRD15 | chr2 | 21360305 | 21366129 | 1 | 0.0E+00 | 0.0E+00 |
| AC011239 | chr2 | 23729935 | 23746933 | 1 | 0.0E+00 | 0.0E+00 |
| KLHL29 | chr2 | 23755352 | 23929604 | 1 | 0.0E+00 | 0.0E+00 |
| ATAD2B | chr2 | 23974789 | 24149641 | 1 | 0.0E+00 | 0.0E+00 |
| UBXN2A | chr2 | 24181160 | 24222724 | 1 | 0.0E+00 | 0.0E+00 |
| MFSD2B | chr2 | 24232912 | 24248442 | 1 | 3.6E-62 | 6.1E-60 |
| C2orf44 | chr2 | 24253771 | 24262406 | 1 | 3.6E-62 | 6.1E-60 |
| FKBP1B | chr2 | 24272671 | 24286041 | 1 | 3.6E-62 | 6.1E-60 |
| SF3B14 | chr2 | 24290536 | 24299134 | 1 | 3.6E-62 | 6.1E-60 |
| TP53I3 | chr2 | 24300430 | 24307206 | 1 | 3.6E-62 | 6.1E-60 |
| FAM228B | chr2 | 24303657 | 24392407 | 1 | 3.6E-62 | 6.1E-60 |
| PFN4 | chr2 | 24338424 | 24345485 | 1 | 3.6E-62 | 6.1E-60 |
| FAM228A | chr2 | 24398291 | 24423689 | 1 | 3.6E-62 | 6.1E-60 |
| ITSN2 | chr2 | 24426502 | 24551037 | 1 | 3.6E-62 | 6.1E-60 |
| NCOA1 | chr2 | 24881491 | 24991311 | 1 | 0.0E+00 | 0.0E+00 |
| PTRHD1 | chr2 | 25013209 | 25016402 | 1 | 0.0E+00 | 0.0E+00 |
| CENPO | chr2 | 25016724 | 25040675 | 1 | 0.0E+00 | 0.0E+00 |
| ADCY3 | chr2 | 25042749 | 25141916 | 1 | 0.0E+00 | 0.0E+00 |
| DNAJC27 | chr2 | 25170418 | 25194792 | 1 | 0.0E+00 | 0.0E+00 |
| EFR3B | chr2 | 25265081 | 25377237 | 1 | 0.0E+00 | 0.0E+00 |
| POMC | chr2 | 25383919 | 25387621 | 1 | 0.0E+00 | 0.0E+00 |
| DNMT3A | chr2 | 25457148 | 25551604 | 1 | 0.0E+00 | 0.0E+00 |
| DTNB | chr2 | 25602144 | 25875562 | 1 | 0.0E+00 | 0.0E+00 |
| ASXL2 | chr2 | 25964877 | 26101173 | 1 | 0.0E+00 | 0.0E+00 |
| KIF3C | chr2 | 26151812 | 26204790 | 1 | 0.0E+00 | 0.0E+00 |
| RAB10 | chr2 | 26257491 | 26357986 | 1 | 0.0E+00 | 0.0E+00 |
| GAREML | chr2 | 26396042 | 26411165 | 1 | 0.0E+00 | 0.0E+00 |
| HADHA | chr2 | 26414179 | 26467533 | 1 | 0.0E+00 | 0.0E+00 |
| HADHB | chr2 | 26477154 | 26512905 | 1 | 0.0E+00 | 0.0E+00 |
| GPR113 | chr2 | 26531901 | 26569161 | 1 | 0.0E+00 | 0.0E+00 |
| EPT1 | chr2 | 26587060 | 26612081 | 1 | 0.0E+00 | 0.0E+00 |
| DRC1 | chr2 | 26624832 | 26679413 | 1 | 0.0E+00 | 0.0E+00 |
| OTOF | chr2 | 26680922 | 26781443 | 1 | 0.0E+00 | 0.0E+00 |
| C2orf70 | chr2 | 26785463 | 26802390 | 1 | 0.0E+00 | 0.0E+00 |

**Supplementary Table S3. List of copy number alterations and affected genes in SCC-R cells**

| Gene | Chromosome | Start co-ordinate | End co-ordinate | Copy number | P-value | Q-value |
|---|---|---|---|---|---|---|
| CIB4 | chr2 | 26804083 | 26864222 | 1 | 0.0E+00 | 0.0E+00 |
| KCNK3 | chr2 | 26915794 | 26951446 | 1 | 0.0E+00 | 0.0E+00 |
| SLC35F6 | chr2 | 26987183 | 27001324 | 1 | 0.0E+00 | 0.0E+00 |
| CENPA | chr2 | 27009044 | 27016122 | 1 | 0.0E+00 | 0.0E+00 |
| DPYSL5 | chr2 | 27121312 | 27169876 | 1 | 0.0E+00 | 0.0E+00 |
| MAPRE3 | chr2 | 27245094 | 27249294 | 1 | 0.0E+00 | 0.0E+00 |
| TMEM214 | chr2 | 27255912 | 27263705 | 1 | 0.0E+00 | 0.0E+00 |
| AGBL5 | chr2 | 27275868 | 27293200 | 1 | 0.0E+00 | 0.0E+00 |
| OST4 | chr2 | 27294150 | 27294331 | 1 | 0.0E+00 | 0.0E+00 |
| EMILIN1 | chr2 | 27301932 | 27308892 | 1 | 0.0E+00 | 0.0E+00 |
| KHK | chr2 | 27310104 | 27323470 | 1 | 0.0E+00 | 0.0E+00 |
| CGREF1 | chr2 | 27324121 | 27327639 | 1 | 0.0E+00 | 0.0E+00 |
| ABHD1 | chr2 | 27346808 | 27353606 | 1 | 0.0E+00 | 0.0E+00 |
| PREB | chr2 | 27354225 | 27357267 | 1 | 0.0E+00 | 0.0E+00 |
| TCF23 | chr2 | 27372057 | 27375796 | 1 | 0.0E+00 | 0.0E+00 |
| SLC5A6 | chr2 | 27423292 | 27435352 | 1 | 0.0E+00 | 0.0E+00 |
| ATRAID | chr2 | 27435976 | 27439864 | 1 | 0.0E+00 | 0.0E+00 |
| CAD | chr2 | 27440413 | 27466363 | 1 | 0.0E+00 | 0.0E+00 |
| SLC30A3 | chr2 | 27477954 | 27498114 | 1 | 0.0E+00 | 0.0E+00 |
| DNAJC5G | chr2 | 27499551 | 27503140 | 1 | 0.0E+00 | 0.0E+00 |
| TRIM54 | chr2 | 27505598 | 27529349 | 1 | 0.0E+00 | 0.0E+00 |
| UCN | chr2 | 27530397 | 27530767 | 1 | 0.0E+00 | 0.0E+00 |
| MPV17 | chr2 | 27532754 | 27547408 | 1 | 0.0E+00 | 0.0E+00 |
| GTF3C2 | chr2 | 27548970 | 27579040 | 1 | 0.0E+00 | 0.0E+00 |
| EIF2B4 | chr2 | 27587279 | 27593205 | 1 | 0.0E+00 | 0.0E+00 |
| SNX17 | chr2 | 27593439 | 27599581 | 1 | 0.0E+00 | 0.0E+00 |
| ZNF513 | chr2 | 27600225 | 27603409 | 1 | 0.0E+00 | 0.0E+00 |
| PPM1G | chr2 | 27604428 | 27632281 | 1 | 0.0E+00 | 0.0E+00 |
| NRBP1 | chr2 | 27656187 | 27664706 | 1 | 0.0E+00 | 0.0E+00 |
| KRTCAP3 | chr2 | 27665273 | 27669323 | 1 | 0.0E+00 | 0.0E+00 |
| IFT172 | chr2 | 27667237 | 27712546 | 1 | 0.0E+00 | 0.0E+00 |
| FNDC4 | chr2 | 27715259 | 27717517 | 1 | 0.0E+00 | 0.0E+00 |
| GCKR | chr2 | 27719757 | 27746322 | 1 | 0.0E+00 | 0.0E+00 |
| AC109829 | chr2 | 27760249 | 27789998 | 1 | 0.0E+00 | 0.0E+00 |
| C2orf16 | chr2 | 27799456 | 27805436 | 1 | 0.0E+00 | 0.0E+00 |
| ZNF512 | chr2 | 27806497 | 27823729 | 1 | 0.0E+00 | 0.0E+00 |
| CCDC121 | chr2 | 27849836 | 27850820 | 1 | 0.0E+00 | 0.0E+00 |
| GPN1 | chr2 | 27851205 | 27873082 | 1 | 0.0E+00 | 0.0E+00 |
| SUPT7L | chr2 | 27876340 | 27887265 | 1 | 0.0E+00 | 0.0E+00 |
| SLC4A1AP | chr2 | 27887269 | 27917484 | 1 | 0.0E+00 | 0.0E+00 |
| AC074091 | chr2 | 27930392 | 27930999 | 1 | 0.0E+00 | 0.0E+00 |
| MRPL33 | chr2 | 27994580 | 28002463 | 1 | 0.0E+00 | 0.0E+00 |
| RBKS | chr2 | 28004503 | 28113236 | 1 | 0.0E+00 | 0.0E+00 |
| BRE | chr2 | 28117373 | 28561399 | 1 | 0.0E+00 | 0.0E+00 |
| FOSL2 | chr2 | 28615619 | 28635332 | 1 | 0.0E+00 | 0.0E+00 |
| PLB1 | chr2 | 28718849 | 28865877 | 1 | 0.0E+00 | 0.0E+00 |
| PPP1CB | chr2 | 28974608 | 29022219 | 1 | 0.0E+00 | 0.0E+00 |
| TRMT61B | chr2 | 29072716 | 29093098 | 1 | 0.0E+00 | 0.0E+00 |
| WDR43 | chr2 | 29117582 | 29169714 | 1 | 1.5E-150 | 3.9E-148 |

Supplementary Table S3. List of copy number alterations and affected genes in SCC-R cells

| Gene | Chromosome | Start co-ordinate | End co-ordinate | Copy number | P-value | Q-value |
|---|---|---|---|---|---|---|
| FAM179A | chr2 | 29217554 | 29275010 | 1 | 1.5E-150 | 3.9E-148 |
| C2orf71 | chr2 | 29287765 | 29297150 | 1 | 1.5E-150 | 3.9E-148 |
| CLIP4 | chr2 | 29344264 | 29412489 | 1 | 1.5E-150 | 3.9E-148 |
| ALK | chr2 | 29416104 | 30143489 | 1 | 1.5E-150 | 3.9E-148 |
| YPEL5 | chr2 | 30379577 | 30381717 | 1 | 1.5E-150 | 3.9E-148 |
| LBH | chr2 | 30454514 | 30546439 | 1 | 1.5E-150 | 3.9E-148 |
| LCLAT1 | chr2 | 30682383 | 30863459 | 1 | 1.5E-150 | 3.9E-148 |
| CAPN13 | chr2 | 30953565 | 31010246 | 1 | 1.5E-150 | 3.9E-148 |
| GALNT14 | chr2 | 31133796 | 31369850 | 1 | 1.5E-150 | 3.9E-148 |
| CAPN14 | chr2 | 31397506 | 31428357 | 1 | 1.5E-150 | 3.9E-148 |
| EHD3 | chr2 | 31457423 | 31489597 | 1 | 1.5E-150 | 3.9E-148 |
| XDH | chr2 | 31558688 | 31637602 | 1 | 1.5E-150 | 3.9E-148 |
| SRD5A2 | chr2 | 31751206 | 31805970 | 1 | 1.5E-150 | 3.9E-148 |
| DPY30 | chr2 | 32093517 | 32264489 | 1 | 0.0E+00 | 0.0E+00 |
| MEMO1 | chr2 | 32118221 | 32235295 | 1 | 0.0E+00 | 0.0E+00 |
| SPAST | chr2 | 32288840 | 32379611 | 1 | 0.0E+00 | 0.0E+00 |
| SLC30A6 | chr2 | 32390908 | 32445749 | 1 | 0.0E+00 | 0.0E+00 |
| NLRC4 | chr2 | 32449563 | 32481932 | 1 | 0.0E+00 | 0.0E+00 |
| YIPF4 | chr2 | 32503221 | 32532266 | 1 | 0.0E+00 | 0.0E+00 |
| BIRC6 | chr2 | 32582182 | 32843041 | 1 | 0.0E+00 | 0.0E+00 |
| TTC27 | chr2 | 32853332 | 33046065 | 1 | 0.0E+00 | 0.0E+00 |
| LTBP1 | chr2 | 33172389 | 33623634 | 1 | 0.0E+00 | 0.0E+00 |
| RASGRP3 | chr2 | 33740144 | 33787936 | 1 | 0.0E+00 | 0.0E+00 |
| FAM98A | chr2 | 33809833 | 33824390 | 1 | 0.0E+00 | 0.0E+00 |
| MYADML | chr2 | 33952113 | 33952850 | 1 | 0.0E+00 | 0.0E+00 |
| CRIM1 | chr2 | 36583467 | 36775887 | 1 | 0.0E+00 | 0.0E+00 |
| AC007401 | chr2 | 36758973 | 36818164 | 1 | 0.0E+00 | 0.0E+00 |
| FEZ2 | chr2 | 36780174 | 36825305 | 1 | 0.0E+00 | 0.0E+00 |
| VIT | chr2 | 36943395 | 37041546 | 1 | 0.0E+00 | 0.0E+00 |
| STRN | chr2 | 37076546 | 37193632 | 1 | 0.0E+00 | 0.0E+00 |
| HEATR5B | chr2 | 37195631 | 37310556 | 1 | 0.0E+00 | 0.0E+00 |
| GPATCH11 | chr2 | 37315391 | 37323420 | 1 | 0.0E+00 | 0.0E+00 |
| EIF2AK2 | chr2 | 37334377 | 37374949 | 1 | 0.0E+00 | 0.0E+00 |
| SULT6B1 | chr2 | 37395048 | 37415751 | 1 | 0.0E+00 | 0.0E+00 |
| AC007390 | chr2 | 37426802 | 37428288 | 1 | 0.0E+00 | 0.0E+00 |
| CEBPZ | chr2 | 37428795 | 37458884 | 1 | 0.0E+00 | 0.0E+00 |
| NDUFAF7 | chr2 | 37459195 | 37475508 | 1 | 0.0E+00 | 0.0E+00 |
| PRKD3 | chr2 | 37480338 | 37543698 | 1 | 0.0E+00 | 0.0E+00 |
| QPCT | chr2 | 37571857 | 37599942 | 1 | 0.0E+00 | 0.0E+00 |
| CDC42EP3 | chr2 | 37872927 | 37873659 | 1 | 0.0E+00 | 0.0E+00 |
| RMDN2 | chr2 | 38156391 | 38244850 | 1 | 0.0E+00 | 0.0E+00 |
| CYP1B1 | chr2 | 38294080 | 38302510 | 1 | 0.0E+00 | 0.0E+00 |
| ATL2 | chr2 | 38523084 | 38604469 | 1 | 0.0E+00 | 0.0E+00 |
| RPLP0P6 | chr2 | 38709055 | 38709390 | 1 | 0.0E+00 | 0.0E+00 |
| HNRNPLL | chr2 | 38791262 | 38830112 | 1 | 0.0E+00 | 0.0E+00 |
| GALM | chr2 | 38893290 | 38968418 | 1 | 0.0E+00 | 0.0E+00 |
| SRSF7 | chr2 | 38972211 | 38978476 | 1 | 0.0E+00 | 0.0E+00 |
| GEMIN6 | chr2 | 39006117 | 39009051 | 1 | 0.0E+00 | 0.0E+00 |
| DHX57 | chr2 | 39025134 | 39095577 | 1 | 0.0E+00 | 0.0E+00 |

Supplementary Table S3. List of copy number alterations and affected genes in SCC-R cells

| Gene | Chromosome | Start co-ordinate | End co-ordinate | Copy number | P-value | Q-value |
|---|---|---|---|---|---|---|
| MORN2 | chr2 | 39107728 | 39109668 | 1 | 0.0E+00 | 0.0E+00 |
| ARHGEF33 | chr2 | 39117008 | 39201014 | 1 | 0.0E+00 | 0.0E+00 |
| AC019171 | chr2 | 39186985 | 39187631 | 1 | 0.0E+00 | 0.0E+00 |
| SOS1 | chr2 | 39212938 | 39347584 | 1 | 0.0E+00 | 0.0E+00 |
| CDKL4 | chr2 | 39403114 | 39456743 | 1 | 0.0E+00 | 0.0E+00 |
| MAP4K3 | chr2 | 39477667 | 39664143 | 1 | 0.0E+00 | 0.0E+00 |
| TMEM178A | chr2 | 39893131 | 39944403 | 1 | 0.0E+00 | 0.0E+00 |
| THUMPD2 | chr2 | 39963869 | 40006400 | 1 | 0.0E+00 | 0.0E+00 |
| SLC8A1 | chr2 | 40342373 | 40657442 | 1 | 0.0E+00 | 0.0E+00 |
| C2orf91 | chr2 | 42165705 | 42181321 | 1 | 0.0E+00 | 0.0E+00 |
| PKDCC | chr2 | 42275358 | 42284867 | 1 | 0.0E+00 | 0.0E+00 |
| EML4 | chr2 | 42396715 | 42557374 | 1 | 0.0E+00 | 0.0E+00 |
| COX7A2L | chr2 | 42560900 | 42588617 | 1 | 0.0E+00 | 0.0E+00 |
| KCNG3 | chr2 | 42671125 | 42720609 | 1 | 0.0E+00 | 0.0E+00 |
| MTA3 | chr2 | 42795880 | 42980570 | 1 | 0.0E+00 | 0.0E+00 |
| OXER1 | chr2 | 42990064 | 42991343 | 1 | 0.0E+00 | 0.0E+00 |
| HAAO | chr2 | 42994588 | 43019696 | 1 | 0.0E+00 | 0.0E+00 |
| ZFP36L2 | chr2 | 43451455 | 43453511 | 1 | 0.0E+00 | 0.0E+00 |
| THADA | chr2 | 43454892 | 43819560 | 1 | 0.0E+00 | 0.0E+00 |
| PLEKHH2 | chr2 | 43871803 | 43992686 | 1 | 0.0E+00 | 0.0E+00 |
| DYNC2LI1 | chr2 | 44001218 | 44036954 | 1 | 0.0E+00 | 0.0E+00 |
| ABCG5 | chr2 | 44040208 | 44065885 | 1 | 0.0E+00 | 0.0E+00 |
| ABCG8 | chr2 | 44066149 | 44105057 | 1 | 0.0E+00 | 0.0E+00 |
| LRPPRC | chr2 | 44115724 | 44223161 | 1 | 0.0E+00 | 0.0E+00 |
| PPM1B | chr2 | 44428317 | 44471526 | 1 | 0.0E+00 | 0.0E+00 |
| SLC3A1 | chr2 | 44502680 | 44541112 | 1 | 0.0E+00 | 0.0E+00 |
| PREPL | chr2 | 44545177 | 44586899 | 1 | 0.0E+00 | 0.0E+00 |
| CAMKMT | chr2 | 44589136 | 44999374 | 1 | 0.0E+00 | 0.0E+00 |
| SIX3 | chr2 | 45169266 | 45171964 | 1 | 0.0E+00 | 0.0E+00 |
| SIX2 | chr2 | 45233264 | 45236299 | 1 | 0.0E+00 | 0.0E+00 |
| SRBD1 | chr2 | 45616393 | 45832653 | 1 | 0.0E+00 | 0.0E+00 |
| PRKCE | chr2 | 45879176 | 46412010 | 1 | 0.0E+00 | 0.0E+00 |
| EPAS1 | chr2 | 46524960 | 46611790 | 1 | 0.0E+00 | 0.0E+00 |
| TMEM247 | chr2 | 46706727 | 46711595 | 1 | 0.0E+00 | 0.0E+00 |
| ATP6V1E2 | chr2 | 46739131 | 46739840 | 1 | 0.0E+00 | 0.0E+00 |
| RHOQ | chr2 | 46770290 | 46803906 | 1 | 0.0E+00 | 0.0E+00 |
| PIGF | chr2 | 46815149 | 46842364 | 1 | 0.0E+00 | 0.0E+00 |
| CRIPT | chr2 | 46844328 | 46851417 | 1 | 0.0E+00 | 0.0E+00 |
| SOCS5 | chr2 | 46985617 | 46987290 | 1 | 0.0E+00 | 0.0E+00 |
| AC016722 | chr2 | 47043919 | 47085329 | 1 | 0.0E+00 | 0.0E+00 |
| MCFD2 | chr2 | 47132689 | 47168873 | 1 | 0.0E+00 | 0.0E+00 |
| TTC7A | chr2 | 47144404 | 47300999 | 1 | 0.0E+00 | 0.0E+00 |
| C2orf61 | chr2 | 47314145 | 47382421 | 1 | 0.0E+00 | 0.0E+00 |
| CALM2 | chr2 | 47348213 | 47403688 | 1 | 0.0E+00 | 0.0E+00 |
| AC073283 | chr2 | 47537597 | 47537991 | 1 | 0.0E+00 | 0.0E+00 |
| EPCAM | chr2 | 47596132 | 47613832 | 1 | 0.0E+00 | 0.0E+00 |
| MSH2 | chr2 | 47630296 | 47739643 | 1 | 0.0E+00 | 0.0E+00 |
| KCNK12 | chr2 | 47748069 | 47797405 | 1 | 0.0E+00 | 0.0E+00 |
| MSH6 | chr2 | 48010310 | 48034044 | 1 | 0.0E+00 | 0.0E+00 |

**Supplementary Table S3. List of copy number alterations and affected genes in SCC-R cells**

| Gene | Chromosome | Start co-ordinate | End co-ordinate | Copy number | P-value | Q-value |
|---|---|---|---|---|---|---|
| **FBXO11** | chr2 | 48016473 | 48132872 | **1** | 0.0E+00 | 0.0E+00 |
| **FOXN2** | chr2 | 48573349 | 48602560 | **1** | 0.0E+00 | 0.0E+00 |
| **PPP1R21** | chr2 | 48668056 | 48701996 | **1** | 0.0E+00 | 0.0E+00 |
| **DOCK2** | chr5 | 169422996 | 169509876 | **1** | 5.5E-20 | 4.4E-18 |
| **FOXI1** | chr5 | 169532960 | 169535626 | **1** | 5.5E-20 | 4.4E-18 |
| **CTC** | chr5 | 37646855 | 37690501 | **4** | 0.0E+00 | 0.0E+00 |
| **CTC** | chr5 | 39399819 | 39416982 | **5.5** | 0.0E+00 | 0.0E+00 |
| **FAM84B** | chr8 | 127568702 | 127569705 | **1** | 4.8E-13 | 2.9E-11 |
| **POU5F1B** | chr8 | 128428076 | 128429209 | **1** | 4.8E-13 | 2.9E-11 |
| **MYC** | chr8 | 128748799 | 128753230 | **1** | 4.8E-13 | 2.9E-11 |
| **PVT1** | chr8 | 128808124 | 129061523 | **1** | 4.8E-13 | 2.9E-11 |
| **TMEM75** | chr8 | 128959903 | 128960348 | **1** | 4.8E-13 | 2.9E-11 |
| **LINC00293** | chr8 | 47752735 | 47759716 | **3** | 3.1E-14 | 2.0E-12 |
| **SPIDR** | chr8 | 48173516 | 48648012 | **3** | 3.1E-14 | 2.0E-12 |
| **PARD3** | chr10 | 34573015 | 34759216 | **1** | 3.9E-19 | 3.0E-17 |
| **GFRA1** | chr10 | 117823875 | 118031563 | **1** | 9.7E-50 | 1.4E-47 |
| **CCDC172** | chr10 | 118084424 | 118138976 | **1** | 9.7E-50 | 1.4E-47 |
| **PNLIPRP3** | chr10 | 118187458 | 118236734 | **1** | 9.7E-50 | 1.4E-47 |
| **PNLIP** | chr10 | 118305570 | 118327342 | **1** | 9.7E-50 | 1.4E-47 |
| **PNLIPRP1** | chr10 | 118350551 | 118368638 | **1** | 9.7E-50 | 1.4E-47 |
| **PNLIPRP2** | chr10 | 118380329 | 118404734 | **1** | 9.7E-50 | 1.4E-47 |
| **C10orf82** | chr10 | 118423451 | 118427937 | **1** | 9.7E-50 | 1.4E-47 |
| **HSPA12A** | chr10 | 118434244 | 118502035 | **1** | 9.7E-50 | 1.4E-47 |
| **FGFR2** | chr10 | 123238996 | 123353405 | **1** | 3.1E-12 | 1.7E-10 |
| **SCN4B** | chr11 | 118007740 | 118023407 | **1** | 2.4E-03 | 4.1E-02 |
| **RAB38** | chr11 | 87846801 | 87908626 | **3** | 8.6E-70 | 1.6E-67 |
| **CTSC** | chr11 | 88027152 | 88070940 | **3** | 8.6E-70 | 1.6E-67 |
| **GRM5** | chr11 | 88241730 | 88781048 | **3** | 8.6E-70 | 1.6E-67 |
| **TYR** | chr11 | 88911175 | 89028523 | **3** | 8.6E-70 | 1.6E-67 |
| **NOX4** | chr11 | 89059897 | 89224428 | **3** | 8.6E-70 | 1.6E-67 |
| **FOLH1B** | chr11 | 89371686 | 89431812 | **3** | 8.6E-70 | 1.6E-67 |
| **TRIM77** | chr11 | 89443451 | 89451031 | **3** | 8.6E-70 | 1.6E-67 |
| **TRIM49** | chr11 | 89531479 | 89534127 | **3** | 8.6E-70 | 1.6E-67 |
| **TRIM64B** | chr11 | 89604797 | 89608204 | **3** | 8.6E-70 | 1.6E-67 |
| **TRIM49C** | chr11 | 89771889 | 89774505 | **3** | 8.6E-70 | 1.6E-67 |
| **NAALAD2** | chr11 | 89867911 | 89924936 | **3** | 8.6E-70 | 1.6E-67 |
| **CHORDC1** | chr11 | 89935668 | 89956035 | **3** | 8.6E-70 | 1.6E-67 |
| **DISC1FP1** | chr11 | 90288881 | 90602370 | **3** | 8.6E-70 | 1.6E-67 |
| **ANGPTL5** | chr11 | 101762021 | 101778736 | **3** | 5.2E-183 | 1.5E-180 |
| **KIAA1377** | chr11 | 101786033 | 101868471 | **3** | 5.2E-183 | 1.5E-180 |
| **C11orf70** | chr11 | 101918173 | 101953948 | **3** | 5.2E-183 | 1.5E-180 |
| **YAP1** | chr11 | 101981534 | 102100670 | **3** | 5.2E-183 | 1.5E-180 |
| **BIRC3** | chr11 | 102195239 | 102207852 | **3** | 5.2E-183 | 1.5E-180 |
| **BIRC2** | chr11 | 102219279 | 102248899 | **3** | 5.2E-183 | 1.5E-180 |
| **TMEM123** | chr11 | 102269506 | 102323363 | **3** | 5.2E-183 | 1.5E-180 |
| **MMP7** | chr11 | 102391378 | 102401497 | **3** | 5.2E-183 | 1.5E-180 |
| **MMP20** | chr11 | 102447951 | 102496080 | **3** | 5.2E-183 | 1.5E-180 |
| **MMP27** | chr11 | 102562511 | 102576534 | **3** | 5.2E-183 | 1.5E-180 |
| **MMP8** | chr11 | 102583997 | 102595624 | **3** | 5.2E-183 | 1.5E-180 |

**Supplementary Table S3. List of copy number alterations and affected genes in SCC-R cells**

| Gene | Chromosome | Start co-ordinate | End co-ordinate | Copy number | P-value | Q-value |
|---|---|---|---|---|---|---|
| AP000619 | chr11 | 102622777 | 102624252 | 3 | 5.2E-183 | 1.5E-180 |
| MMP10 | chr11 | 102641488 | 102651328 | 3 | 5.2E-183 | 1.5E-180 |
| MMP1 | chr11 | 102661082 | 102668221 | 3 | 5.2E-183 | 1.5E-180 |
| WTAPP1 | chr11 | 102665874 | 102668815 | 3 | 5.2E-183 | 1.5E-180 |
| MMP3 | chr11 | 102706754 | 102714279 | 3 | 5.2E-183 | 1.5E-180 |
| MMP12 | chr11 | 102733751 | 102745712 | 3 | 5.2E-183 | 1.5E-180 |
| MMP13 | chr11 | 102814823 | 102826377 | 3 | 5.2E-183 | 1.5E-180 |
| DCUN1D5 | chr11 | 102932998 | 102962606 | 3 | 5.2E-183 | 1.5E-180 |
| DYNC2H1 | chr11 | 102980278 | 103349996 | 3 | 5.2E-183 | 1.5E-180 |
| PDGFD | chr11 | 103780387 | 104034704 | 3 | 5.2E-183 | 1.5E-180 |
| DDI1 | chr11 | 103908357 | 103908787 | 3 | 5.2E-183 | 1.5E-180 |
| CASP12 | chr11 | 104757417 | 104769196 | 3 | 5.2E-183 | 1.5E-180 |
| CASP4 | chr11 | 104813538 | 104839304 | 3 | 5.2E-183 | 1.5E-180 |
| CASP5 | chr11 | 104866474 | 104893974 | 3 | 5.2E-183 | 1.5E-180 |
| CASP1 | chr11 | 104896142 | 104906002 | 3 | 5.2E-183 | 1.5E-180 |
| CARD16 | chr11 | 104912038 | 104916191 | 3 | 5.2E-183 | 1.5E-180 |
| CARD17 | chr11 | 104970003 | 104972271 | 3 | 5.2E-183 | 1.5E-180 |
| CARD18 | chr11 | 105009496 | 105010578 | 3 | 5.2E-183 | 1.5E-180 |
| GRIA4 | chr11 | 105481651 | 105850483 | 3 | 5.2E-183 | 1.5E-180 |
| KBTBD3 | chr11 | 105923549 | 105929832 | 3 | 5.2E-183 | 1.5E-180 |
| AASDHPPT | chr11 | 105948427 | 105967657 | 3 | 5.2E-183 | 1.5E-180 |
| GUCY1A2 | chr11 | 106558308 | 106888785 | 3 | 5.2E-183 | 1.5E-180 |
| IGF1 | chr12 | 102796154 | 102874250 | 1 | 1.1E-66 | 1.9E-64 |
| PAH | chr12 | 103232910 | 103352098 | 1 | 1.1E-66 | 1.9E-64 |
| ASCL1 | chr12 | 103352238 | 103352725 | 1 | 1.1E-66 | 1.9E-64 |
| C12orf42 | chr12 | 103667518 | 103872227 | 1 | 1.1E-66 | 1.9E-64 |
| STAB2 | chr12 | 103981216 | 104160073 | 1 | 1.1E-66 | 1.9E-64 |
| DIAPH3 | chr13 | 60240722 | 60737915 | 1 | 0.0E+00 | 0.0E+00 |
| TDRD3 | chr13 | 60971433 | 61141831 | 1 | 0.0E+00 | 0.0E+00 |
| PCDH20 | chr13 | 61985359 | 61989268 | 1 | 0.0E+00 | 0.0E+00 |
| PCDH9 | chr13 | 66878771 | 67802629 | 1 | 0.0E+00 | 0.0E+00 |
| KLHL1 | chr13 | 70275756 | 70681854 | 1 | 0.0E+00 | 0.0E+00 |
| DACH1 | chr13 | 72014681 | 72440953 | 1 | 0.0E+00 | 0.0E+00 |
| MZT1 | chr13 | 73284428 | 73301747 | 1 | 0.0E+00 | 0.0E+00 |
| BORA | chr13 | 73302019 | 73329436 | 1 | 0.0E+00 | 0.0E+00 |
| DIS3 | chr13 | 73333930 | 73356058 | 1 | 0.0E+00 | 0.0E+00 |
| PIBF1 | chr13 | 73357636 | 73590134 | 1 | 0.0E+00 | 0.0E+00 |
| KLF5 | chr13 | 73633441 | 73650015 | 1 | 0.0E+00 | 0.0E+00 |
| KLF12 | chr13 | 74269658 | 74569240 | 1 | 0.0E+00 | 0.0E+00 |
| AL355390 | chr13 | 74988746 | 74993030 | 1 | 0.0E+00 | 0.0E+00 |
| TBC1D4 | chr13 | 75860883 | 76055921 | 1 | 0.0E+00 | 0.0E+00 |
| COMMD6 | chr13 | 76100681 | 76111953 | 1 | 0.0E+00 | 0.0E+00 |
| UCHL3 | chr13 | 76123995 | 76180012 | 1 | 0.0E+00 | 0.0E+00 |
| LMO7 | chr13 | 76195841 | 76432176 | 1 | 0.0E+00 | 0.0E+00 |
| C13orf45 | chr13 | 76444982 | 76457367 | 1 | 0.0E+00 | 0.0E+00 |
| IRG1 | chr13 | 77522596 | 77532079 | 1 | 0.0E+00 | 0.0E+00 |
| CLN5 | chr13 | 77566000 | 77575160 | 1 | 0.0E+00 | 0.0E+00 |
| FBXL3 | chr13 | 77581262 | 77596037 | 1 | 0.0E+00 | 0.0E+00 |
| MYCBP2 | chr13 | 77619427 | 77900862 | 1 | 0.0E+00 | 0.0E+00 |

**Supplementary Table S3. List of copy number alterations and affected genes in SCC-R cells**

| Gene | Chromosome | Start co-ordinate | End co-ordinate | Copy number | P-value | Q-value |
|---|---|---|---|---|---|---|
| SCEL | chr13 | 78129909 | 78218459 | 1 | 0.0E+00 | 0.0E+00 |
| SLAIN1 | chr13 | 78272060 | 78337443 | 1 | 0.0E+00 | 0.0E+00 |
| EDNRB | chr13 | 78470586 | 78493722 | 1 | 0.0E+00 | 0.0E+00 |
| POU4F1 | chr13 | 79175547 | 79177522 | 1 | 0.0E+00 | 0.0E+00 |
| RNF219 | chr13 | 79189672 | 79233310 | 1 | 0.0E+00 | 0.0E+00 |
| RBM26 | chr13 | 79888995 | 79979955 | 1 | 0.0E+00 | 0.0E+00 |
| NDFIP2 | chr13 | 80055274 | 80125314 | 1 | 0.0E+00 | 0.0E+00 |
| SPRY2 | chr13 | 80910735 | 80911779 | 1 | 0.0E+00 | 0.0E+00 |
| SLITRK6 | chr13 | 86368088 | 86370673 | 1 | 0.0E+00 | 0.0E+00 |
| MIR4500HG | chr13 | 88270891 | 88271114 | 1 | 0.0E+00 | 0.0E+00 |
| MIR17HG | chr13 | 92002857 | 92003685 | 1 | 0.0E+00 | 0.0E+00 |
| GPC5 | chr13 | 92051296 | 93518734 | 1 | 0.0E+00 | 0.0E+00 |
| GPC6 | chr13 | 93879643 | 95055447 | 1 | 0.0E+00 | 0.0E+00 |
| DCT | chr13 | 95090769 | 95131566 | 1 | 0.0E+00 | 0.0E+00 |
| TGDS | chr13 | 95226973 | 95248414 | 1 | 0.0E+00 | 0.0E+00 |
| GPR180 | chr13 | 95254156 | 95279494 | 1 | 0.0E+00 | 0.0E+00 |
| SOX21 | chr13 | 95363485 | 95364376 | 1 | 0.0E+00 | 0.0E+00 |
| ABCC4 | chr13 | 95673757 | 95953573 | 1 | 0.0E+00 | 0.0E+00 |
| CLDN10 | chr13 | 96086148 | 96230348 | 1 | 0.0E+00 | 0.0E+00 |
| DZIP1 | chr13 | 96234366 | 96294213 | 1 | 0.0E+00 | 0.0E+00 |
| DNAJC3 | chr13 | 96329477 | 96443311 | 1 | 0.0E+00 | 0.0E+00 |
| UGGT2 | chr13 | 96453901 | 96705617 | 1 | 0.0E+00 | 0.0E+00 |
| HS6ST3 | chr13 | 96743122 | 97485473 | 1 | 0.0E+00 | 0.0E+00 |
| OXGR1 | chr13 | 97638995 | 97640039 | 1 | 0.0E+00 | 0.0E+00 |
| MBNL2 | chr13 | 97928455 | 98043763 | 1 | 0.0E+00 | 0.0E+00 |
| RAP2A | chr13 | 98086720 | 98116696 | 1 | 0.0E+00 | 0.0E+00 |
| IPO5 | chr13 | 98606073 | 98674195 | 1 | 0.0E+00 | 0.0E+00 |
| RNF113B | chr13 | 98828368 | 98829454 | 1 | 0.0E+00 | 0.0E+00 |
| FARP1 | chr13 | 98860752 | 99100676 | 1 | 0.0E+00 | 0.0E+00 |
| STK24 | chr13 | 99105427 | 99229109 | 1 | 0.0E+00 | 0.0E+00 |
| SLC15A1 | chr13 | 99336966 | 99404973 | 1 | 0.0E+00 | 0.0E+00 |
| DOCK9 | chr13 | 99446867 | 99738622 | 1 | 0.0E+00 | 0.0E+00 |
| UBAC2 | chr13 | 99853056 | 100037617 | 1 | 0.0E+00 | 0.0E+00 |
| GPR183 | chr13 | 99947306 | 99948444 | 1 | 0.0E+00 | 0.0E+00 |
| TM9SF2 | chr13 | 100153902 | 100215113 | 1 | 0.0E+00 | 0.0E+00 |
| CLYBL | chr13 | 100258855 | 100543742 | 1 | 0.0E+00 | 0.0E+00 |
| ZIC5 | chr13 | 100617645 | 100623997 | 1 | 0.0E+00 | 0.0E+00 |
| ZIC2 | chr13 | 100633954 | 100638366 | 1 | 0.0E+00 | 0.0E+00 |
| PCCA | chr13 | 100741270 | 101182488 | 1 | 0.0E+00 | 0.0E+00 |
| GGACT | chr13 | 101184382 | 101184839 | 1 | 0.0E+00 | 0.0E+00 |
| TMTC4 | chr13 | 101257179 | 101322670 | 1 | 0.0E+00 | 0.0E+00 |
| NALCN | chr13 | 101707581 | 102051584 | 1 | 0.0E+00 | 0.0E+00 |
| ITGBL1 | chr13 | 102105190 | 102375300 | 1 | 0.0E+00 | 0.0E+00 |
| FGF14 | chr13 | 102378938 | 102698473 | 1 | 0.0E+00 | 0.0E+00 |
| TPP2 | chr13 | 103249330 | 103330684 | 1 | 0.0E+00 | 0.0E+00 |
| METTL21C | chr13 | 103338415 | 103346945 | 1 | 0.0E+00 | 0.0E+00 |
| CCDC168 | chr13 | 103381765 | 103390016 | 1 | 0.0E+00 | 0.0E+00 |
| TEX30 | chr13 | 103418777 | 103422441 | 1 | 0.0E+00 | 0.0E+00 |
| KDELC1 | chr13 | 103436810 | 103451003 | 1 | 0.0E+00 | 0.0E+00 |

**Supplementary Table S3. List of copy number alterations and affected genes in SCC-R cells**

| Gene | Chromosome | Start co-ordinate | End co-ordinate | Copy number | P-value | Q-value |
|---|---|---|---|---|---|---|
| **BIVM** | chr13 | 103459628 | 103492239 | **1** | 0.0E+00 | 0.0E+00 |
| **ERCC5** | chr13 | 103498499 | 103528273 | **1** | 0.0E+00 | 0.0E+00 |
| **SLC10A2** | chr13 | 103698443 | 103718542 | **1** | 0.0E+00 | 0.0E+00 |
| **DAOA** | chr13 | 106118517 | 106142483 | **1** | 0.0E+00 | 0.0E+00 |
| **EFNB2** | chr13 | 107145433 | 107187313 | **1** | 0.0E+00 | 0.0E+00 |
| **ARGLU1** | chr13 | 107196282 | 107220320 | **1** | 0.0E+00 | 0.0E+00 |
| **FAM155A** | chr13 | 107822843 | 108518951 | **1** | 0.0E+00 | 0.0E+00 |
| **ABHD13** | chr13 | 108881523 | 108882563 | **1** | 0.0E+00 | 0.0E+00 |
| **TNFSF13B** | chr13 | 108922212 | 108959289 | **1** | 0.0E+00 | 0.0E+00 |
| **MYO16** | chr13 | 109282081 | 109859215 | **1** | 0.0E+00 | 0.0E+00 |
| **IRS2** | chr13 | 110408544 | 110438412 | **1** | 0.0E+00 | 0.0E+00 |
| **COL4A1** | chr13 | 110801458 | 110959378 | **1** | 0.0E+00 | 0.0E+00 |
| **COL4A2** | chr13 | 110960295 | 111164614 | **1** | 0.0E+00 | 0.0E+00 |
| **RAB20** | chr13 | 111175985 | 111213882 | **1** | 0.0E+00 | 0.0E+00 |
| **CARKD** | chr13 | 111267802 | 111291069 | **1** | 0.0E+00 | 0.0E+00 |
| **CARS2** | chr13 | 111293716 | 111358513 | **1** | 0.0E+00 | 0.0E+00 |
| **ING1** | chr13 | 111364932 | 111372284 | **1** | 0.0E+00 | 0.0E+00 |
| **LINC00346** | chr13 | 111521579 | 111522155 | **1** | 0.0E+00 | 0.0E+00 |
| **ANKRD10** | chr13 | 111532050 | 111567230 | **1** | 0.0E+00 | 0.0E+00 |
| **ARHGEF7** | chr13 | 111767872 | 111955442 | **1** | 0.0E+00 | 0.0E+00 |
| **TEX29** | chr13 | 111973147 | 111996553 | **1** | 0.0E+00 | 0.0E+00 |
| **SOX1** | chr13 | 112721938 | 112723184 | **1** | 0.0E+00 | 0.0E+00 |
| **SPACA7** | chr13 | 113030703 | 113088873 | **1** | 0.0E+00 | 0.0E+00 |
| **TUBGCP3** | chr13 | 113140218 | 113242330 | **1** | 0.0E+00 | 0.0E+00 |
| **C13orf35** | chr13 | 113333652 | 113337691 | **1** | 0.0E+00 | 0.0E+00 |
| **ATP11A** | chr13 | 113344647 | 113536392 | **1** | 0.0E+00 | 0.0E+00 |
| **MCF2L** | chr13 | 113556462 | 113751164 | **1** | 0.0E+00 | 0.0E+00 |
| **F7** | chr13 | 113760069 | 113773351 | **1** | 0.0E+00 | 0.0E+00 |
| **F10** | chr13 | 113777041 | 113803858 | **1** | 0.0E+00 | 0.0E+00 |
| **PROZ** | chr13 | 113812966 | 113826430 | **1** | 0.0E+00 | 0.0E+00 |
| **PCID2** | chr13 | 113832467 | 113863013 | **1** | 0.0E+00 | 0.0E+00 |
| **CUL4A** | chr13 | 113863970 | 113917950 | **1** | 0.0E+00 | 0.0E+00 |
| **LAMP1** | chr13 | 113951757 | 113976806 | **1** | 0.0E+00 | 0.0E+00 |
| **GRTP1** | chr13 | 113978803 | 114018281 | **1** | 0.0E+00 | 0.0E+00 |
| **ADPRHL1** | chr13 | 114077099 | 114107753 | **1** | 0.0E+00 | 0.0E+00 |
| **DCUN1D2** | chr13 | 114112234 | 114145064 | **1** | 0.0E+00 | 0.0E+00 |
| **TMCO3** | chr13 | 114149909 | 114204057 | **1** | 0.0E+00 | 0.0E+00 |
| **TFDP1** | chr13 | 114240134 | 114294465 | **1** | 0.0E+00 | 0.0E+00 |
| **ATP4B** | chr13 | 114303692 | 114312553 | **1** | 0.0E+00 | 0.0E+00 |
| **GRK1** | chr13 | 114321700 | 114438341 | **1** | 0.0E+00 | 0.0E+00 |
| **LINC00552** | chr13 | 114453493 | 114453617 | **1** | 0.0E+00 | 0.0E+00 |
| **TMEM255B** | chr13 | 114462200 | 114514953 | **1** | 0.0E+00 | 0.0E+00 |
| **GAS6** | chr13 | 114523864 | 114566850 | **1** | 0.0E+00 | 0.0E+00 |
| **LINC00452** | chr13 | 114618849 | 114624078 | **1** | 0.0E+00 | 0.0E+00 |
| **RASA3** | chr13 | 114748751 | 114897999 | **1** | 0.0E+00 | 0.0E+00 |
| **CDC16** | chr13 | 115000492 | 115037969 | **1** | 0.0E+00 | 0.0E+00 |
| **UPF3A** | chr13 | 115047123 | 115070390 | **1** | 0.0E+00 | 0.0E+00 |
| **UNC79** | chr14 | 93897282 | 94173252 | **1** | 2.7E-109 | 6.2E-107 |
| **PRIMA1** | chr14 | 94187781 | 94254067 | **1** | 2.7E-109 | 6.2E-107 |

**Supplementary Table S3. List of copy number alterations and affected genes in SCC-R cells**

| Gene | Chromosome | Start co-ordinate | End co-ordinate | Copy number | P-value | Q-value |
|---|---|---|---|---|---|---|
| FAM181A | chr14 | 94391563 | 94395510 | 1 | 2.7E-109 | 6.2E-107 |
| ASB2 | chr14 | 94400439 | 94430855 | 1 | 2.7E-109 | 6.2E-107 |
| LINC00521 | chr14 | 94464372 | 94475100 | 1 | 2.7E-109 | 6.2E-107 |
| OTUB2 | chr14 | 94492776 | 94512317 | 1 | 2.7E-109 | 6.2E-107 |
| DDX24 | chr14 | 94517582 | 94546146 | 1 | 2.7E-109 | 6.2E-107 |
| IFI27L1 | chr14 | 94554540 | 94569030 | 1 | 2.7E-109 | 6.2E-107 |
| IFI27 | chr14 | 94577229 | 94583015 | 1 | 2.7E-109 | 6.2E-107 |
| IFI27L2 | chr14 | 94594098 | 94596007 | 1 | 2.7E-109 | 6.2E-107 |
| PPP4R4 | chr14 | 94640778 | 94745069 | 1 | 2.7E-109 | 6.2E-107 |
| SERPINA10 | chr14 | 94750256 | 94759054 | 1 | 2.7E-109 | 6.2E-107 |
| SERPINA6 | chr14 | 94770751 | 94780997 | 1 | 2.7E-109 | 6.2E-107 |
| SERPINA1 | chr14 | 94844797 | 94849508 | 1 | 2.7E-109 | 6.2E-107 |
| SERPINA11 | chr14 | 94908891 | 94915161 | 1 | 2.7E-109 | 6.2E-107 |
| SERPINA9 | chr14 | 94929394 | 94942618 | 1 | 2.7E-109 | 6.2E-107 |
| SERPINA12 | chr14 | 94953611 | 94964761 | 1 | 2.7E-109 | 6.2E-107 |
| SERPINA4 | chr14 | 95028046 | 95035919 | 1 | 2.7E-109 | 6.2E-107 |
| SERPINA5 | chr14 | 95053752 | 95058607 | 1 | 2.7E-109 | 6.2E-107 |
| SERPINA3 | chr14 | 95078606 | 95090196 | 1 | 2.7E-109 | 6.2E-107 |
| SERPINA13P | chr14 | 95107118 | 95111421 | 1 | 2.7E-109 | 6.2E-107 |
| MIR381HG | chr14 | 101512212 | 101516008 | 1 | 1.2E-30 | 1.4E-28 |
| DIO3OS | chr14 | 102018759 | 102026756 | 1 | 1.2E-30 | 1.4E-28 |
| ASPG | chr14 | 104552105 | 104578994 | 1 | 3.8E-70 | 6.8E-68 |
| KIF26A | chr14 | 104605008 | 104647150 | 1 | 3.8E-70 | 6.8E-68 |
| C14orf144 | chr14 | 104710535 | 104710774 | 1 | 3.8E-70 | 6.8E-68 |
| TMEM179 | chr14 | 104979070 | 105071093 | 1 | 3.8E-70 | 6.8E-68 |
| C14orf180 | chr14 | 105052680 | 105055263 | 1 | 3.8E-70 | 6.8E-68 |
| INF2 | chr14 | 105167741 | 105185193 | 1 | 3.8E-70 | 6.8E-68 |
| SIVA1 | chr14 | 105219534 | 105225835 | 1 | 3.8E-70 | 6.8E-68 |
| AKT1 | chr14 | 105235895 | 105259046 | 1 | 3.8E-70 | 6.8E-68 |
| ZBTB42 | chr14 | 105267505 | 105268842 | 1 | 3.8E-70 | 6.8E-68 |
| CEP170B | chr14 | 105334780 | 105363110 | 1 | 3.8E-70 | 6.8E-68 |
| GPR132 | chr14 | 105517299 | 105523622 | 1 | 1.0E-119 | 2.4E-117 |
| JAG2 | chr14 | 105608611 | 105634777 | 1 | 1.0E-119 | 2.4E-117 |
| BRF1 | chr14 | 105675825 | 105752806 | 1 | 1.0E-119 | 2.4E-117 |
| BTBD6 | chr14 | 105715202 | 105716902 | 1 | 1.0E-119 | 2.4E-117 |
| PACS2 | chr14 | 105766747 | 105861108 | 1 | 1.0E-119 | 2.4E-117 |
| TEX22 | chr14 | 105865622 | 105878211 | 1 | 1.0E-119 | 2.4E-117 |
| MTA1 | chr14 | 105886301 | 105936492 | 1 | 1.0E-119 | 2.4E-117 |
| CRIP2 | chr14 | 105939591 | 105946033 | 1 | 1.0E-119 | 2.4E-117 |
| CRIP1 | chr14 | 105953550 | 105954807 | 1 | 1.0E-119 | 2.4E-117 |
| C14orf80 | chr14 | 105957291 | 105965265 | 1 | 1.0E-119 | 2.4E-117 |
| TMEM121 | chr14 | 105995186 | 105996125 | 1 | 1.0E-119 | 2.4E-117 |
| AL928742 | chr14 | 106064187 | 106066587 | 1 | 1.0E-119 | 2.4E-117 |
| AL122127 | chr14 | 106356888 | 106361507 | 1 | 1.0E-119 | 2.4E-117 |
| KIAA0125 | chr14 | 106385271 | 106388526 | 1 | 1.0E-119 | 2.4E-117 |
| ADAM6 | chr14 | 106436031 | 106437081 | 1 | 1.0E-119 | 2.4E-117 |
| GOLGA8A | chr15 | 34781178 | 34782733 | 0 | 1.1E-07 | 4.7E-06 |
| CSNK1A1P1 | chr15 | 37110488 | 37110609 | 1 | 4.4E-40 | 5.8E-38 |
| MEIS2 | chr15 | 37184344 | 37391773 | 1 | 4.4E-40 | 5.8E-38 |

Supplementary Table S3. List of copy number alterations and affected genes in SCC-R cells

| Gene | Chromosome | Start co-ordinate | End co-ordinate | Copy number | P-value | Q-value |
|---|---|---|---|---|---|---|
| TMCO5A | chr15 | 38228478 | 38258990 | 1 | 4.4E-40 | 5.8E-38 |
| SPRED1 | chr15 | 38545343 | 38643872 | 1 | 4.4E-40 | 5.8E-38 |
| FAM98B | chr15 | 38746353 | 38776798 | 1 | 4.4E-40 | 5.8E-38 |
| RASGRP1 | chr15 | 38782659 | 38857638 | 1 | 4.4E-40 | 5.8E-38 |
| PIEZO1 | chr16 | 88782039 | 88851423 | 1 | 2.6E-64 | 4.6E-62 |
| TBC1D3P5 | chr17 | 25752965 | 25753055 | 1 | 2.5E-289 | 7.5E-287 |
| KSR1 | chr17 | 25783644 | 25950486 | 1 | 2.5E-289 | 7.5E-287 |
| LGALS9 | chr17 | 25958195 | 25976008 | 1 | 2.5E-289 | 7.5E-287 |
| NOS2 | chr17 | 26084277 | 26125919 | 1 | 2.5E-289 | 7.5E-287 |
| LYRM9 | chr17 | 26206436 | 26219567 | 1 | 2.5E-289 | 7.5E-287 |
| NLK | chr17 | 26369827 | 26521744 | 1 | 2.5E-289 | 7.5E-287 |
| PPY2 | chr17 | 26575181 | 26575384 | 1 | 2.5E-289 | 7.5E-287 |
| KRT18P55 | chr17 | 26603967 | 26633299 | 1 | 2.5E-289 | 7.5E-287 |
| TMEM97 | chr17 | 26646185 | 26655789 | 1 | 2.5E-289 | 7.5E-287 |
| IFT20 | chr17 | 26656127 | 26659276 | 1 | 2.5E-289 | 7.5E-287 |
| TNFAIP1 | chr17 | 26666526 | 26671632 | 1 | 2.5E-289 | 7.5E-287 |
| POLDIP2 | chr17 | 26675064 | 26684435 | 1 | 2.5E-289 | 7.5E-287 |
| TMEM199 | chr17 | 26684753 | 26708870 | 1 | 2.5E-289 | 7.5E-287 |
| SARM1 | chr17 | 26691272 | 26727801 | 1 | 2.5E-289 | 7.5E-287 |
| SEBOX | chr17 | 26691773 | 26691948 | 1 | 2.5E-289 | 7.5E-287 |
| VTN | chr17 | 26694403 | 26697242 | 1 | 2.5E-289 | 7.5E-287 |
| SLC46A1 | chr17 | 26722866 | 26733062 | 1 | 2.5E-289 | 7.5E-287 |
| SLC13A2 | chr17 | 26800738 | 26824247 | 1 | 2.5E-289 | 7.5E-287 |
| FOXN1 | chr17 | 26850902 | 26864460 | 1 | 2.5E-289 | 7.5E-287 |
| UNC119 | chr17 | 26874267 | 26879582 | 1 | 2.5E-289 | 7.5E-287 |
| PIGS | chr17 | 26881255 | 26898579 | 1 | 2.5E-289 | 7.5E-287 |
| ALDOC | chr17 | 26900528 | 26902597 | 1 | 2.5E-289 | 7.5E-287 |
| SPAG5 | chr17 | 26906649 | 26944366 | 1 | 2.5E-289 | 7.5E-287 |
| SGK494 | chr17 | 26937504 | 26941244 | 1 | 2.5E-289 | 7.5E-287 |
| KIAA0100 | chr17 | 26942091 | 26972117 | 1 | 2.5E-289 | 7.5E-287 |
| SDF2 | chr17 | 26976054 | 26988973 | 1 | 2.5E-289 | 7.5E-287 |
| SUPT6H | chr17 | 27000345 | 27028605 | 1 | 2.5E-289 | 7.5E-287 |
| PROCA1 | chr17 | 27030540 | 27038756 | 1 | 2.5E-289 | 7.5E-287 |
| RAB34 | chr17 | 27041635 | 27045265 | 1 | 2.5E-289 | 7.5E-287 |
| RPL23A | chr17 | 27046866 | 27050878 | 1 | 2.5E-289 | 7.5E-287 |
| TLCD1 | chr17 | 27051526 | 27053858 | 1 | 2.5E-289 | 7.5E-287 |
| NEK8 | chr17 | 27055751 | 27069132 | 1 | 2.5E-289 | 7.5E-287 |
| TRAF4 | chr17 | 27071134 | 27076926 | 1 | 2.5E-289 | 7.5E-287 |
| FAM222B | chr17 | 27085206 | 27093686 | 1 | 2.5E-289 | 7.5E-287 |
| ERAL1 | chr17 | 27182077 | 27187594 | 1 | 2.5E-289 | 7.5E-287 |
| MIR4732 | chr17 | 27188338 | 27188696 | 1 | 2.5E-289 | 7.5E-287 |
| FLOT2 | chr17 | 27206265 | 27224656 | 1 | 2.5E-289 | 7.5E-287 |
| DHRS13 | chr17 | 27225408 | 27229980 | 1 | 2.5E-289 | 7.5E-287 |
| SEZ6 | chr17 | 27282683 | 27332920 | 1 | 2.5E-289 | 7.5E-287 |
| PIPOX | chr17 | 27370252 | 27383341 | 1 | 2.5E-289 | 7.5E-287 |
| TIAF1 | chr17 | 27400796 | 27418735 | 1 | 2.5E-289 | 7.5E-287 |
| MYO18A | chr17 | 27412559 | 27493926 | 1 | 2.5E-289 | 7.5E-287 |
| CRYBA1 | chr17 | 27573735 | 27581436 | 1 | 2.5E-289 | 7.5E-287 |
| NUFIP2 | chr17 | 27591428 | 27621083 | 1 | 2.5E-289 | 7.5E-287 |

Supplementary Table S3. List of copy number alterations and affected genes in SCC-R cells

| Gene | Chromosome | Start co-ordinate | End co-ordinate | Copy number | P-value | Q-value |
|---|---|---|---|---|---|---|
| TAOK1 | chr17 | 27717597 | 27870091 | 1 | 2.5E-289 | 7.5E-287 |
| ABHD15 | chr17 | 27889534 | 27890088 | 1 | 2.5E-289 | 7.5E-287 |
| TP53I13 | chr17 | 27893041 | 27899943 | 1 | 2.5E-289 | 7.5E-287 |
| GIT1 | chr17 | 27901539 | 27916428 | 1 | 2.5E-289 | 7.5E-287 |
| ANKRD13B | chr17 | 27920621 | 27940942 | 1 | 2.5E-289 | 7.5E-287 |
| CORO6 | chr17 | 27942776 | 27949938 | 1 | 2.5E-289 | 7.5E-287 |
| SSH2 | chr17 | 27957866 | 28257110 | 1 | 2.5E-289 | 7.5E-287 |
| EFCAB5 | chr17 | 28268761 | 28435087 | 1 | 1.2E-204 | 3.4E-202 |
| NSRP1 | chr17 | 28443823 | 28512722 | 1 | 1.2E-204 | 3.4E-202 |
| SLC6A4 | chr17 | 28525357 | 28549901 | 1 | 1.2E-204 | 3.4E-202 |
| BLMH | chr17 | 28576066 | 28618973 | 1 | 1.2E-204 | 3.4E-202 |
| TMIGD1 | chr17 | 28643631 | 28659235 | 1 | 1.2E-204 | 3.4E-202 |
| CPD | chr17 | 28706012 | 28791860 | 1 | 1.2E-204 | 3.4E-202 |
| GOSR1 | chr17 | 28804376 | 28849376 | 1 | 1.2E-204 | 3.4E-202 |
| TBC1D29 | chr17 | 28886681 | 28890425 | 1 | 1.2E-204 | 3.4E-202 |
| AC006050 | chr17 | 28900643 | 28903243 | 1 | 1.2E-204 | 3.4E-202 |
| AC005562 | chr17 | 28952043 | 28964364 | 1 | 1.2E-204 | 3.4E-202 |
| SUZ12P | chr17 | 29051332 | 29097016 | 1 | 1.2E-204 | 3.4E-202 |
| CRLF3 | chr17 | 29103310 | 29151672 | 1 | 1.2E-204 | 3.4E-202 |
| ATAD5 | chr17 | 29159355 | 29221974 | 1 | 1.2E-204 | 3.4E-202 |
| TEFM | chr17 | 29226160 | 29233323 | 1 | 1.2E-204 | 3.4E-202 |
| ADAP2 | chr17 | 29248956 | 29284953 | 1 | 1.2E-204 | 3.4E-202 |
| RNF135 | chr17 | 29298042 | 29326191 | 1 | 1.2E-204 | 3.4E-202 |
| NF1 | chr17 | 29422313 | 29709050 | 1 | 1.2E-204 | 3.4E-202 |
| EVI2B | chr17 | 29631245 | 29635078 | 1 | 1.2E-204 | 3.4E-202 |
| EVI2A | chr17 | 29647350 | 29647522 | 1 | 1.2E-204 | 3.4E-202 |
| RAB11FIP4 | chr17 | 29718912 | 29862007 | 1 | 1.2E-204 | 3.4E-202 |
| AC003101 | chr17 | 29898110 | 29902692 | 1 | 1.2E-204 | 3.4E-202 |
| COPRS | chr17 | 30179185 | 30186326 | 1 | 2.2E-96 | 4.7E-94 |
| UTP6 | chr17 | 30190434 | 30228665 | 1 | 2.2E-96 | 4.7E-94 |
| SUZ12 | chr17 | 30264328 | 30326040 | 1 | 2.2E-96 | 4.7E-94 |
| LRRC37B | chr17 | 30348022 | 30380388 | 1 | 2.2E-96 | 4.7E-94 |
| SH3GL1P1 | chr17 | 30368029 | 30368871 | 1 | 2.2E-96 | 4.7E-94 |
| AC090616 | chr17 | 30469239 | 30470163 | 1 | 2.2E-96 | 4.7E-94 |
| RHOT1 | chr17 | 30497948 | 30551778 | 1 | 2.2E-96 | 4.7E-94 |
| RHBDL3 | chr17 | 30593188 | 30648466 | 1 | 2.2E-96 | 4.7E-94 |
| C17orf75 | chr17 | 30658786 | 30669210 | 1 | 2.2E-96 | 4.7E-94 |
| ZNF207 | chr17 | 30688423 | 30700565 | 1 | 2.2E-96 | 4.7E-94 |
| PSMD11 | chr17 | 30771521 | 30807665 | 1 | 2.2E-96 | 4.7E-94 |
| CDK5R1 | chr17 | 30814643 | 30816359 | 1 | 2.2E-96 | 4.7E-94 |
| MYO1D | chr17 | 30821800 | 31203911 | 1 | 2.2E-96 | 4.7E-94 |
| TMEM98 | chr17 | 31258611 | 31267904 | 1 | 8.4E-17 | 5.6E-15 |
| SPACA3 | chr17 | 31318906 | 31324812 | 1 | 8.4E-17 | 5.6E-15 |
| ASIC2 | chr17 | 31340967 | 32483511 | 1 | 8.4E-17 | 5.6E-15 |
| RBBP8 | chr18 | 20513314 | 20606235 | 0.5 | 5.9E-81 | 1.2E-78 |
| CABLES1 | chr18 | 20715660 | 20837331 | 0.5 | 5.9E-81 | 1.2E-78 |
| TMEM241 | chr18 | 20877854 | 21017864 | 0.5 | 5.9E-81 | 1.2E-78 |
| RIOK3 | chr18 | 21033375 | 21061351 | 0.5 | 5.9E-81 | 1.2E-78 |
| C18orf8 | chr18 | 21083569 | 21110584 | 0.5 | 5.9E-81 | 1.2E-78 |

**Supplementary Table S3. List of copy number alterations and affected genes in SCC-R cells**

| Gene | Chromosome | Start co-ordinate | End co-ordinate | Copy number | P-value | Q-value |
|---|---|---|---|---|---|---|
| **NPC1** | chr18 | 21109079 | 21166437 | **0.5** | 5.9E-81 | 1.2E-78 |
| **ANKRD29** | chr18 | 21181188 | 21242767 | **0.5** | 5.9E-81 | 1.2E-78 |
| **LAMA3** | chr18 | 21269709 | 21534641 | **0.5** | 5.9E-81 | 1.2E-78 |
| **TTC39C** | chr18 | 21594874 | 21714204 | **0.5** | 5.6E-73 | 1.0E-70 |
| **CABYR** | chr18 | 21723098 | 21741531 | **1** | 6.7E-26 | 6.7E-24 |
| **OSBPL1A** | chr18 | 21743150 | 21819390 | **1** | 6.7E-26 | 6.7E-24 |
| **KIAA0355** | chr19 | 34791313 | 34843830 | **3** | 4.6E-29 | 5.0E-27 |
| **LSM14A** | chr19 | 34663465 | 34718241 | **3.5** | 4.2E-11 | 2.2E-09 |
| **GRAMD1A** | chr19 | 35486861 | 35517069 | **3.5** | 1.8E-61 | 3.1E-59 |
| **SCN1B** | chr19 | 35521632 | 35530615 | **3.5** | 1.8E-61 | 3.1E-59 |
| **HPN** | chr19 | 35532697 | 35557214 | **3.5** | 1.8E-61 | 3.1E-59 |
| **AC020907** | chr19 | 35597306 | 35597796 | **3.5** | 1.8E-61 | 3.1E-59 |
| **FXYD3** | chr19 | 35608176 | 35614384 | **3.5** | 1.8E-61 | 3.1E-59 |
| **LGI4** | chr19 | 35616066 | 35633457 | **3.5** | 1.8E-61 | 3.1E-59 |
| **FXYD1** | chr19 | 35630852 | 35633761 | **3.5** | 1.8E-61 | 3.1E-59 |
| **FXYD7** | chr19 | 35634203 | 35644850 | **3.5** | 1.8E-61 | 3.1E-59 |
| **FXYD5** | chr19 | 35646335 | 35660536 | **3.5** | 1.8E-61 | 3.1E-59 |
| **FAM187B** | chr19 | 35715725 | 35719532 | **3.5** | 1.8E-61 | 3.1E-59 |
| **LSR** | chr19 | 35739709 | 35758807 | **3.5** | 1.8E-61 | 3.1E-59 |
| **USF2** | chr19 | 35759985 | 35770161 | **3.5** | 1.8E-61 | 3.1E-59 |
| **HAMP** | chr19 | 35773400 | 35776023 | **3.5** | 1.8E-61 | 3.1E-59 |
| **MAG** | chr19 | 35783026 | 35804366 | **3.5** | 1.8E-61 | 3.1E-59 |
| **CD22** | chr19 | 35820056 | 35837599 | **4** | 0.0E+00 | 0.0E+00 |
| **FFAR1** | chr19 | 35842510 | 35843384 | **4** | 0.0E+00 | 0.0E+00 |
| **FFAR3** | chr19 | 35849760 | 35850833 | **4** | 0.0E+00 | 0.0E+00 |
| **GPR42** | chr19 | 35862229 | 35863302 | **4** | 0.0E+00 | 0.0E+00 |
| **AC002511** | chr19 | 35898671 | 35899550 | **4** | 0.0E+00 | 0.0E+00 |
| **FFAR2** | chr19 | 35940596 | 35941645 | **4** | 0.0E+00 | 0.0E+00 |
| **KRTDAP** | chr19 | 35978215 | 35981345 | **4** | 0.0E+00 | 0.0E+00 |
| **DMKN** | chr19 | 35989665 | 36004338 | **4** | 0.0E+00 | 0.0E+00 |
| **SBSN** | chr19 | 36014267 | 36019195 | **4** | 0.0E+00 | 0.0E+00 |
| **GAPDHS** | chr19 | 36024304 | 36036406 | **4** | 0.0E+00 | 0.0E+00 |
| **TMEM147** | chr19 | 36036648 | 36038428 | **4** | 0.0E+00 | 0.0E+00 |
| **ATP4A** | chr19 | 36041412 | 36054532 | **4** | 0.0E+00 | 0.0E+00 |
| **HAUS5** | chr19 | 36103702 | 36113832 | **4** | 0.0E+00 | 0.0E+00 |
| **RBM42** | chr19 | 36120068 | 36128441 | **4** | 0.0E+00 | 0.0E+00 |
| **ETV2** | chr19 | 36133316 | 36135814 | **4** | 0.0E+00 | 0.0E+00 |
| **COX6B1** | chr19 | 36139716 | 36149644 | **4** | 0.0E+00 | 0.0E+00 |
| **UPK1A** | chr19 | 36157686 | 36168972 | **4** | 0.0E+00 | 0.0E+00 |
| **ZBTB32** | chr19 | 36205469 | 36207701 | **4** | 0.0E+00 | 0.0E+00 |
| **WBP7** | chr19 | 36209013 | 36229501 | **4** | 0.0E+00 | 0.0E+00 |
| **IGFLR1** | chr19 | 36230126 | 36233691 | **4** | 0.0E+00 | 0.0E+00 |
| **U2AF1L4** | chr19 | 36234589 | 36235611 | **4** | 0.0E+00 | 0.0E+00 |
| **PSENEN** | chr19 | 36236022 | 36237765 | **4** | 0.0E+00 | 0.0E+00 |
| **LIN37** | chr19 | 36239558 | 36245425 | **4** | 0.0E+00 | 0.0E+00 |
| **HSPB6** | chr19 | 36246207 | 36248186 | **4** | 0.0E+00 | 0.0E+00 |
| **C19orf55** | chr19 | 36249067 | 36259502 | **4** | 0.0E+00 | 0.0E+00 |
| **ARHGAP33** | chr19 | 36266459 | 36279342 | **4** | 0.0E+00 | 0.0E+00 |
| **AC002398** | chr19 | 36279602 | 36279795 | **4** | 0.0E+00 | 0.0E+00 |

**Supplementary Table S3. List of copy number alterations and affected genes in SCC-R cells**

| Gene | Chromosome | Start co-ordinate | End co-ordinate | Copy number | P-value | Q-value |
|---|---|---|---|---|---|---|
| **PRODH2** | chr19 | 36290989 | 36304113 | **4** | 0.0E+00 | 0.0E+00 |
| **NPHS1** | chr19 | 36317403 | 36357495 | **4** | 0.0E+00 | 0.0E+00 |
| **KIRREL2** | chr19 | 36347953 | 36355692 | **4** | 0.0E+00 | 0.0E+00 |
| **APLP1** | chr19 | 36359009 | 36370319 | **4** | 0.0E+00 | 0.0E+00 |
| **NFKBID** | chr19 | 36379324 | 36393277 | **4** | 0.0E+00 | 0.0E+00 |
| **HCST** | chr19 | 36393406 | 36395200 | **4** | 0.0E+00 | 0.0E+00 |
| **TYROBP** | chr19 | 36395380 | 36399261 | **4** | 0.0E+00 | 0.0E+00 |
| **LRFN3** | chr19 | 36430374 | 36435873 | **4** | 0.0E+00 | 0.0E+00 |
| **SDHAF1** | chr19 | 36486142 | 36486556 | **4** | 0.0E+00 | 0.0E+00 |
| **SYNE4** | chr19 | 36494110 | 36499665 | **4** | 0.0E+00 | 0.0E+00 |
| **ALKBH6** | chr19 | 36500226 | 36505153 | **4** | 0.0E+00 | 0.0E+00 |
| **CLIP3** | chr19 | 36506946 | 36523298 | **4** | 0.0E+00 | 0.0E+00 |
| **THAP8** | chr19 | 36526277 | 36545146 | **4** | 0.0E+00 | 0.0E+00 |
| **WDR62** | chr19 | 36545882 | 36595888 | **4** | 0.0E+00 | 0.0E+00 |
| **OVOL3** | chr19 | 36602148 | 36604707 | **4** | 0.0E+00 | 0.0E+00 |
| **POLR2I** | chr19 | 36604866 | 36605721 | **4** | 0.0E+00 | 0.0E+00 |
| **TBCB** | chr19 | 36605160 | 36616672 | **4** | 0.0E+00 | 0.0E+00 |
| **CAPNS1** | chr19 | 36631883 | 36640735 | **4** | 0.0E+00 | 0.0E+00 |
| **COX7A1** | chr19 | 36641824 | 36643348 | **4** | 0.0E+00 | 0.0E+00 |
| **ZNF146** | chr19 | 36727286 | 36728199 | **4** | 0.0E+00 | 0.0E+00 |
| **ZFP14** | chr19 | 36831104 | 36858922 | **4** | 0.0E+00 | 0.0E+00 |
| **ZFP82** | chr19 | 36883725 | 36900764 | **4** | 0.0E+00 | 0.0E+00 |
| **ZNF566** | chr19 | 36939844 | 36980919 | **4** | 0.0E+00 | 0.0E+00 |
| **ZNF260** | chr19 | 37004879 | 37006030 | **4** | 0.0E+00 | 0.0E+00 |
| **ZNF529** | chr19 | 37037708 | 37068258 | **4** | 0.0E+00 | 0.0E+00 |
| **ZNF382** | chr19 | 37098474 | 37118463 | **4** | 0.0E+00 | 0.0E+00 |
| **ZNF461** | chr19 | 37128662 | 37155706 | **4** | 0.0E+00 | 0.0E+00 |
| **ZNF567** | chr19 | 37185612 | 37214269 | **4** | 0.0E+00 | 0.0E+00 |
| **ZNF850** | chr19 | 37238587 | 37253603 | **4** | 0.0E+00 | 0.0E+00 |
| **ZNF790** | chr19 | 37309355 | 37316667 | **4** | 0.0E+00 | 0.0E+00 |
| **ZNF345** | chr19 | 37367774 | 37369226 | **4** | 0.0E+00 | 0.0E+00 |
| **ZNF829** | chr19 | 37382331 | 37406876 | **4** | 0.0E+00 | 0.0E+00 |
| **ZNF568** | chr19 | 37413666 | 37488713 | **4** | 0.0E+00 | 0.0E+00 |
| **ZNF420** | chr19 | 37579886 | 37621198 | **4** | 0.0E+00 | 0.0E+00 |
| **ZNF585A** | chr19 | 37605948 | 37660925 | **4** | 0.0E+00 | 0.0E+00 |
| **ZNF585B** | chr19 | 37676150 | 37698123 | **4** | 0.0E+00 | 0.0E+00 |
| **ZNF383** | chr19 | 37721208 | 37734560 | **4** | 0.0E+00 | 0.0E+00 |
| **HKR1** | chr19 | 37825531 | 37854839 | **4** | 0.0E+00 | 0.0E+00 |
| **ZNF527** | chr19 | 37864958 | 37880741 | **4** | 0.0E+00 | 0.0E+00 |
| **ZNF569** | chr19 | 37903464 | 37945879 | **4** | 0.0E+00 | 0.0E+00 |
| **ZNF570** | chr19 | 37958704 | 37976135 | **4** | 0.0E+00 | 0.0E+00 |
| **ZNF793** | chr19 | 38014292 | 38033369 | **4** | 0.0E+00 | 0.0E+00 |
| **ZNF571** | chr19 | 38046934 | 38077725 | **4** | 0.0E+00 | 0.0E+00 |
| **ZNF540** | chr19 | 38089225 | 38104191 | **4** | 0.0E+00 | 0.0E+00 |
| **ZFP30** | chr19 | 38125832 | 38138805 | **4** | 0.0E+00 | 0.0E+00 |
| **ZNF781** | chr19 | 38160067 | 38161020 | **4** | 0.0E+00 | 0.0E+00 |
| **ZNF607** | chr19 | 38188924 | 38202671 | **4** | 0.0E+00 | 0.0E+00 |
| **ZNF573** | chr19 | 38229402 | 38264423 | **4** | 0.0E+00 | 0.0E+00 |
| **AC016582** | chr19 | 38314683 | 38315830 | **4** | 0.0E+00 | 0.0E+00 |

Supplementary Table S3. List of copy number alterations and affected genes in SCC-R cells

| Gene | Chromosome | Start co-ordinate | End co-ordinate | Copy number | P-value | Q-value |
|---|---|---|---|---|---|---|
| MAP3K10 | chr19 | 40697976 | 40721169 | 4 | 0.0E+00 | 0.0E+00 |
| TTC9B | chr19 | 40721964 | 40724262 | 4 | 0.0E+00 | 0.0E+00 |
| CNTD2 | chr19 | 40728921 | 40732567 | 4 | 0.0E+00 | 0.0E+00 |
| AKT2 | chr19 | 40739401 | 40788585 | 4 | 0.0E+00 | 0.0E+00 |
| C19orf47 | chr19 | 40827822 | 40847897 | 4 | 0.0E+00 | 0.0E+00 |
| PLD3 | chr19 | 40854333 | 40884204 | 4 | 0.0E+00 | 0.0E+00 |
| HIPK4 | chr19 | 40885541 | 40895747 | 4 | 0.0E+00 | 0.0E+00 |
| PRX | chr19 | 40899860 | 40913908 | 4 | 0.0E+00 | 0.0E+00 |
| SERTAD3 | chr19 | 40947356 | 40948006 | 4 | 0.0E+00 | 0.0E+00 |
| BLVRB | chr19 | 40953781 | 40971697 | 4 | 0.0E+00 | 0.0E+00 |
| SPTBN4 | chr19 | 40978462 | 41081440 | 4 | 0.0E+00 | 0.0E+00 |
| SHKBP1 | chr19 | 41082804 | 41097164 | 4 | 0.0E+00 | 0.0E+00 |
| LTBP4 | chr19 | 41098938 | 41135420 | 4 | 0.0E+00 | 0.0E+00 |
| NUMBL | chr19 | 41173370 | 41196454 | 4 | 0.0E+00 | 0.0E+00 |
| ADCK4 | chr19 | 41197942 | 41224147 | 4 | 0.0E+00 | 0.0E+00 |
| ITPKC | chr19 | 41223013 | 41245437 | 4 | 0.0E+00 | 0.0E+00 |
| C19orf54 | chr19 | 41247567 | 41255775 | 4 | 0.0E+00 | 0.0E+00 |
| SNRPA | chr19 | 41257189 | 41271072 | 4 | 0.0E+00 | 0.0E+00 |
| MIA | chr19 | 41281463 | 41283384 | 4 | 0.0E+00 | 0.0E+00 |
| RAB4B | chr19 | 41284244 | 41292833 | 4 | 0.0E+00 | 0.0E+00 |
| EGLN2 | chr19 | 41306445 | 41313784 | 4 | 0.0E+00 | 0.0E+00 |
| CYP2A6 | chr19 | 41349711 | 41356321 | 4 | 0.0E+00 | 0.0E+00 |
| CYP2A7 | chr19 | 41381608 | 41388142 | 4 | 0.0E+00 | 0.0E+00 |
| CYP2G1P | chr19 | 41397164 | 41406064 | 4 | 0.0E+00 | 0.0E+00 |
| CYP2B7P1 | chr19 | 41430134 | 41455188 | 4 | 0.0E+00 | 0.0E+00 |
| CYP2B6 | chr19 | 41497167 | 41522781 | 4 | 0.0E+00 | 0.0E+00 |
| CYP2A13 | chr19 | 41594355 | 41601895 | 4 | 0.0E+00 | 0.0E+00 |
| CYP2F1 | chr19 | 41622102 | 41634036 | 4 | 0.0E+00 | 0.0E+00 |
| CYP2S1 | chr19 | 41699214 | 41712743 | 4 | 0.0E+00 | 0.0E+00 |
| AXL | chr19 | 41725269 | 41765771 | 4 | 0.0E+00 | 0.0E+00 |
| HNRNPUL1 | chr19 | 41770423 | 41812487 | 4 | 0.0E+00 | 0.0E+00 |
| CCDC97 | chr19 | 41816194 | 41828622 | 4 | 0.0E+00 | 0.0E+00 |
| TGFB1 | chr19 | 41836949 | 41858902 | 4 | 0.0E+00 | 0.0E+00 |
| B9D2 | chr19 | 41860534 | 41869541 | 4 | 0.0E+00 | 0.0E+00 |
| TMEM91 | chr19 | 41888721 | 41889840 | 4 | 0.0E+00 | 0.0E+00 |
| EXOSC5 | chr19 | 41892467 | 41903236 | 4 | 0.0E+00 | 0.0E+00 |
| BCKDHA | chr19 | 41903728 | 41928867 | 4 | 0.0E+00 | 0.0E+00 |
| ATP5SL | chr19 | 41937018 | 41946347 | 4 | 0.0E+00 | 0.0E+00 |
| C19orf69 | chr19 | 41949118 | 41950189 | 4 | 0.0E+00 | 0.0E+00 |
| AC011526 | chr19 | 41962264 | 41962394 | 4 | 0.0E+00 | 0.0E+00 |
| CEACAM21 | chr19 | 42082591 | 42092834 | 4 | 0.0E+00 | 0.0E+00 |
| CEACAM4 | chr19 | 42125695 | 42133368 | 4 | 0.0E+00 | 0.0E+00 |
| CEACAM7 | chr19 | 42181339 | 42192142 | 4 | 0.0E+00 | 0.0E+00 |
| CEA | chr19 | 42212679 | 42213983 | 4 | 0.0E+00 | 0.0E+00 |
| CEACAM5 | chr19 | 42219047 | 42231218 | 4 | 0.0E+00 | 0.0E+00 |
| CEACAM6 | chr19 | 42259477 | 42270109 | 4 | 0.0E+00 | 0.0E+00 |
| CEACAM3 | chr19 | 42300660 | 42315280 | 4 | 0.0E+00 | 0.0E+00 |
| LYPD4 | chr19 | 42341257 | 42343454 | 4 | 0.0E+00 | 0.0E+00 |
| DMRTC2 | chr19 | 42351522 | 42355868 | 4 | 0.0E+00 | 0.0E+00 |

Supplementary Table S3. List of copy number alterations and affected genes in SCC-R cells

| Gene | Chromosome | Start co-ordinate | End co-ordinate | Copy number | P-value | Q-value |
|---|---|---|---|---|---|---|
| RPS19 | chr19 | 42364718 | 42375403 | 4 | 0.0E+00 | 0.0E+00 |
| ARHGEF1 | chr19 | 42387240 | 42433193 | 4 | 0.0E+00 | 0.0E+00 |
| RABAC1 | chr19 | 42461009 | 42463499 | 4 | 0.0E+00 | 0.0E+00 |
| GRIK5 | chr19 | 42503087 | 42570039 | 4 | 0.0E+00 | 0.0E+00 |
| ZNF574 | chr19 | 42572798 | 42585630 | 4 | 0.0E+00 | 0.0E+00 |
| POU2F2 | chr19 | 42595437 | 42637785 | 4 | 0.0E+00 | 0.0E+00 |
| DEDD2 | chr19 | 42703619 | 42721189 | 4 | 0.0E+00 | 0.0E+00 |
| ZNF526 | chr19 | 42728582 | 42730658 | 4 | 0.0E+00 | 0.0E+00 |
| GSK3A | chr19 | 42734899 | 42746530 | 4 | 0.0E+00 | 0.0E+00 |
| AC006486 | chr19 | 42746968 | 42747439 | 4 | 0.0E+00 | 0.0E+00 |
| ERF | chr19 | 42752635 | 42759183 | 4 | 0.0E+00 | 0.0E+00 |
| CIC | chr19 | 42788847 | 42799424 | 4 | 0.0E+00 | 0.0E+00 |
| PAFAH1B3 | chr19 | 42801280 | 42806466 | 4 | 0.0E+00 | 0.0E+00 |
| PRR19 | chr19 | 42813709 | 42814875 | 4 | 0.0E+00 | 0.0E+00 |
| TMEM145 | chr19 | 42817551 | 42829078 | 4 | 0.0E+00 | 0.0E+00 |
| MEGF8 | chr19 | 42830445 | 42880986 | 4 | 0.0E+00 | 0.0E+00 |
| CNFN | chr19 | 42891247 | 42893766 | 4 | 0.0E+00 | 0.0E+00 |
| LIPE | chr19 | 42905950 | 42931295 | 4 | 0.0E+00 | 0.0E+00 |
| CXCL17 | chr19 | 42932988 | 42947085 | 4 | 0.0E+00 | 0.0E+00 |
| CEACAM1 | chr19 | 43013245 | 43032676 | 4 | 0.0E+00 | 0.0E+00 |
| CEACAM8 | chr19 | 43087355 | 43098945 | 4 | 0.0E+00 | 0.0E+00 |
| PSG3 | chr19 | 43226124 | 43244601 | 4 | 0.0E+00 | 0.0E+00 |
| PSG8 | chr19 | 43257498 | 43269818 | 4 | 0.0E+00 | 0.0E+00 |
| PSG10P | chr19 | 43343436 | 43348708 | 4 | 0.0E+00 | 0.0E+00 |
| PSG1 | chr19 | 43372311 | 43376215 | 4 | 0.0E+00 | 0.0E+00 |
| PSG6 | chr19 | 43406497 | 43422011 | 4 | 0.0E+00 | 0.0E+00 |
| PSG11 | chr19 | 43514098 | 43530568 | 4 | 0.0E+00 | 0.0E+00 |
| PSG2 | chr19 | 43568697 | 43576101 | 4 | 0.0E+00 | 0.0E+00 |
| PSG5 | chr19 | 43674200 | 43690621 | 4 | 0.0E+00 | 0.0E+00 |
| PSG4 | chr19 | 43708022 | 43709011 | 4 | 0.0E+00 | 0.0E+00 |
| PSG9 | chr19 | 43757671 | 43772185 | 4 | 0.0E+00 | 0.0E+00 |
| CD177 | chr19 | 43857807 | 43866567 | 4 | 0.0E+00 | 0.0E+00 |
| TEX101 | chr19 | 43910609 | 43922617 | 4 | 0.0E+00 | 0.0E+00 |
| LYPD3 | chr19 | 43965503 | 43969728 | 4 | 0.0E+00 | 0.0E+00 |
| PHLDB3 | chr19 | 43979561 | 44008225 | 4 | 0.0E+00 | 0.0E+00 |
| ETHE1 | chr19 | 44010964 | 44031331 | 4 | 0.0E+00 | 0.0E+00 |
| ZNF575 | chr19 | 44035845 | 44039923 | 4 | 0.0E+00 | 0.0E+00 |
| XRCC1 | chr19 | 44047575 | 44079657 | 4 | 0.0E+00 | 0.0E+00 |
| PINLYP | chr19 | 44081299 | 44086183 | 4 | 0.0E+00 | 0.0E+00 |
| IRGQ | chr19 | 44096140 | 44099537 | 4 | 0.0E+00 | 0.0E+00 |
| ZNF576 | chr19 | 44101172 | 44103475 | 4 | 0.0E+00 | 0.0E+00 |
| ZNF428 | chr19 | 44111581 | 44112241 | 4 | 0.0E+00 | 0.0E+00 |
| SRRM5 | chr19 | 44116145 | 44118594 | 4 | 0.0E+00 | 0.0E+00 |
| PLAUR | chr19 | 44150491 | 44174288 | 4 | 0.0E+00 | 0.0E+00 |
| SMG9 | chr19 | 44235673 | 44254978 | 4 | 0.0E+00 | 0.0E+00 |
| KCNN4 | chr19 | 44271734 | 44284981 | 4 | 0.0E+00 | 0.0E+00 |
| LYPD5 | chr19 | 44301740 | 44319865 | 4 | 0.0E+00 | 0.0E+00 |
| ZNF283 | chr19 | 44335466 | 44352768 | 4 | 0.0E+00 | 0.0E+00 |
| ZNF404 | chr19 | 44376664 | 44388235 | 4 | 0.0E+00 | 0.0E+00 |

Supplementary Table S3. List of copy number alterations and affected genes in SCC-R cells

| Gene | Chromosome | Start co-ordinate | End co-ordinate | Copy number | P-value | Q-value |
|---|---|---|---|---|---|---|
| ZNF45 | chr19 | 44417506 | 44426459 | 4 | 0.0E+00 | 0.0E+00 |
| ZNF221 | chr19 | 44466783 | 44471585 | 4 | 0.0E+00 | 0.0E+00 |
| ZNF155 | chr19 | 44492540 | 44501549 | 4 | 0.0E+00 | 0.0E+00 |
| ZNF230 | chr19 | 44511144 | 44516698 | 4 | 0.0E+00 | 0.0E+00 |
| ZNF222 | chr19 | 44529492 | 44537287 | 4 | 0.0E+00 | 0.0E+00 |
| ZNF223 | chr19 | 44559294 | 44571628 | 4 | 0.0E+00 | 0.0E+00 |
| ZNF284 | chr19 | 44580454 | 44591311 | 4 | 0.0E+00 | 0.0E+00 |
| ZNF224 | chr19 | 44601088 | 44612470 | 4 | 0.0E+00 | 0.0E+00 |
| ZNF225 | chr19 | 44619868 | 44636799 | 4 | 0.0E+00 | 0.0E+00 |
| ZNF234 | chr19 | 44648724 | 44662360 | 4 | 0.0E+00 | 0.0E+00 |
| ZNF226 | chr19 | 44674079 | 44681812 | 4 | 0.0E+00 | 0.0E+00 |
| ZNF227 | chr19 | 44721632 | 44739853 | 4 | 0.0E+00 | 0.0E+00 |
| ZNF235 | chr19 | 44739858 | 44807606 | 4 | 0.0E+00 | 0.0E+00 |
| ZNF233 | chr19 | 44754376 | 44778847 | 4 | 0.0E+00 | 0.0E+00 |
| ZNF112 | chr19 | 44831599 | 44871351 | 4 | 0.0E+00 | 0.0E+00 |
| ZNF285 | chr19 | 44890643 | 44898752 | 4 | 0.0E+00 | 0.0E+00 |
| ZNF229 | chr19 | 44932465 | 44936466 | 4 | 0.0E+00 | 0.0E+00 |
| ZNF285B | chr19 | 44976369 | 44977366 | 4 | 0.0E+00 | 0.0E+00 |
| ZNF180 | chr19 | 44980587 | 45004314 | 4 | 0.0E+00 | 0.0E+00 |
| CEACAM20 | chr19 | 45010180 | 45033554 | 4 | 0.0E+00 | 0.0E+00 |
| WDR87 | chr19 | 38375538 | 38388909 | 4.5 | 0.0E+00 | 0.0E+00 |
| SIPA1L3 | chr19 | 38572236 | 38696957 | 5.5 | 0.0E+00 | 0.0E+00 |
| DPF1 | chr19 | 38702636 | 38720262 | 5.5 | 0.0E+00 | 0.0E+00 |
| PPP1R14A | chr19 | 38741859 | 38746972 | 5.5 | 0.0E+00 | 0.0E+00 |
| SPINT2 | chr19 | 38755523 | 38782722 | 5.5 | 0.0E+00 | 0.0E+00 |
| C19orf33 | chr19 | 38794885 | 38795660 | 5.5 | 0.0E+00 | 0.0E+00 |
| YIF1B | chr19 | 38796010 | 38806508 | 5.5 | 0.0E+00 | 0.0E+00 |
| KCNK6 | chr19 | 38810617 | 38818012 | 5.5 | 0.0E+00 | 0.0E+00 |
| CATSPERG | chr19 | 38826746 | 38861384 | 5.5 | 0.0E+00 | 0.0E+00 |
| PSMD8 | chr19 | 38865233 | 38874113 | 5.5 | 0.0E+00 | 0.0E+00 |
| GGN | chr19 | 38874967 | 38878745 | 5.5 | 0.0E+00 | 0.0E+00 |
| SPRED3 | chr19 | 38880868 | 38886788 | 5.5 | 0.0E+00 | 0.0E+00 |
| FAM98C | chr19 | 38893777 | 38899524 | 5.5 | 0.0E+00 | 0.0E+00 |
| RYR1 | chr19 | 38924435 | 39078137 | 5.5 | 0.0E+00 | 0.0E+00 |
| MAP4K1 | chr19 | 39078379 | 39108536 | 5.5 | 0.0E+00 | 0.0E+00 |
| EIF3K | chr19 | 39109810 | 39127666 | 5.5 | 0.0E+00 | 0.0E+00 |
| ACTN4 | chr19 | 39138383 | 39221843 | 5.5 | 0.0E+00 | 0.0E+00 |
| CAPN12 | chr19 | 39224298 | 39234825 | 5.5 | 0.0E+00 | 0.0E+00 |
| LGALS7 | chr19 | 39261616 | 39264234 | 5.5 | 0.0E+00 | 0.0E+00 |
| LGALS7B | chr19 | 39279764 | 39282382 | 5.5 | 0.0E+00 | 0.0E+00 |
| LGALS4 | chr19 | 39292356 | 39303583 | 5.5 | 0.0E+00 | 0.0E+00 |
| ECH1 | chr19 | 39306235 | 39322460 | 5.5 | 0.0E+00 | 0.0E+00 |
| HNRNPL | chr19 | 39327389 | 39340617 | 5.5 | 0.0E+00 | 0.0E+00 |
| RINL | chr19 | 39359699 | 39367499 | 5.5 | 0.0E+00 | 0.0E+00 |
| SIRT2 | chr19 | 39369756 | 39390333 | 5.5 | 0.0E+00 | 0.0E+00 |
| NFKBIB | chr19 | 39390608 | 39399582 | 5.5 | 0.0E+00 | 0.0E+00 |
| SARS2 | chr19 | 39421096 | 39421354 | 5.5 | 0.0E+00 | 0.0E+00 |
| MRPS12 | chr19 | 39421796 | 39423321 | 5.5 | 0.0E+00 | 0.0E+00 |
| FBXO17 | chr19 | 39433233 | 39443500 | 5.5 | 0.0E+00 | 0.0E+00 |

Supplementary Table S3. List of copy number alterations and affected genes in SCC-R cells

| Gene | Chromosome | Start co-ordinate | End co-ordinate | Copy number | P-value | Q-value |
|---|---|---|---|---|---|---|
| FBXO27 | chr19 | 39516068 | 39522871 | 5.5 | 0.0E+00 | 0.0E+00 |
| PAPL | chr19 | 39575926 | 39600767 | 5.5 | 0.0E+00 | 0.0E+00 |
| PAK4 | chr19 | 39660172 | 39669189 | 5.5 | 0.0E+00 | 0.0E+00 |
| NCCRP1 | chr19 | 39687625 | 39691479 | 5.5 | 0.0E+00 | 0.0E+00 |
| SYCN | chr19 | 39693588 | 39694859 | 5.5 | 0.0E+00 | 0.0E+00 |
| IFNL3 | chr19 | 39734241 | 39735745 | 5.5 | 0.0E+00 | 0.0E+00 |
| IFNL2 | chr19 | 39759180 | 39760672 | 5.5 | 0.0E+00 | 0.0E+00 |
| IFNL1 | chr19 | 39787097 | 39789182 | 5.5 | 0.0E+00 | 0.0E+00 |
| LRFN1 | chr19 | 39798270 | 39805920 | 5.5 | 0.0E+00 | 0.0E+00 |
| GMFG | chr19 | 39819005 | 39826755 | 5.5 | 0.0E+00 | 0.0E+00 |
| SAMD4B | chr19 | 39847580 | 39874401 | 5.5 | 0.0E+00 | 0.0E+00 |
| PAF1 | chr19 | 39876501 | 39881523 | 5.5 | 0.0E+00 | 0.0E+00 |
| MED29 | chr19 | 39882015 | 39888449 | 5.5 | 0.0E+00 | 0.0E+00 |
| ZFP36 | chr19 | 39897444 | 39899478 | 5.5 | 0.0E+00 | 0.0E+00 |
| PLEKHG2 | chr19 | 39904725 | 39916040 | 5.5 | 0.0E+00 | 0.0E+00 |
| RPS16 | chr19 | 39924023 | 39926495 | 5.5 | 0.0E+00 | 0.0E+00 |
| AC011500 | chr19 | 39930242 | 39932058 | 5.5 | 0.0E+00 | 0.0E+00 |
| SUPT5H | chr19 | 39936421 | 39967091 | 5.5 | 0.0E+00 | 0.0E+00 |
| TIMM50 | chr19 | 39971102 | 39980552 | 5.5 | 0.0E+00 | 0.0E+00 |
| DLL3 | chr19 | 39989553 | 39999056 | 5.5 | 0.0E+00 | 0.0E+00 |
| SELV | chr19 | 40005913 | 40009788 | 5.5 | 0.0E+00 | 0.0E+00 |
| EID2B | chr19 | 40022911 | 40023491 | 5.5 | 0.0E+00 | 0.0E+00 |
| EID2 | chr19 | 40029964 | 40030703 | 5.5 | 0.0E+00 | 0.0E+00 |
| LGALS13 | chr19 | 40093164 | 40097915 | 5.5 | 0.0E+00 | 0.0E+00 |
| LGALS16 | chr19 | 40146486 | 40151144 | 5.5 | 0.0E+00 | 0.0E+00 |
| LGALS17A | chr19 | 40169937 | 40174316 | 5.5 | 0.0E+00 | 0.0E+00 |
| LGALS14 | chr19 | 40195052 | 40199943 | 5.5 | 0.0E+00 | 0.0E+00 |
| CLC | chr19 | 40222086 | 40228714 | 5.5 | 0.0E+00 | 0.0E+00 |
| LEUTX | chr19 | 40267137 | 40276811 | 5.5 | 0.0E+00 | 0.0E+00 |
| DYRK1B | chr19 | 40316358 | 40322520 | 5.5 | 0.0E+00 | 0.0E+00 |
| FBL | chr19 | 40325170 | 40337017 | 5.5 | 0.0E+00 | 0.0E+00 |
| FCGBP | chr19 | 40354043 | 40440615 | 5.5 | 0.0E+00 | 0.0E+00 |
| PSMC4 | chr19 | 40477001 | 40487240 | 5.5 | 0.0E+00 | 0.0E+00 |
| ZNF546 | chr19 | 40504127 | 40521781 | 5.5 | 0.0E+00 | 0.0E+00 |
| ZNF780B | chr19 | 40535261 | 40556333 | 5.5 | 0.0E+00 | 0.0E+00 |
| AC005614 | chr19 | 40570423 | 40570544 | 5.5 | 0.0E+00 | 0.0E+00 |
| ZNF780A | chr19 | 40578662 | 40595340 | 5.5 | 0.0E+00 | 0.0E+00 |
| DEFB125 | chr20 | 68259 | 77076 | 1 | 2.5E-232 | 7.1E-230 |
| DEFB126 | chr20 | 123139 | 126320 | 1 | 2.5E-232 | 7.1E-230 |
| DEFB127 | chr20 | 138095 | 139687 | 1 | 2.5E-232 | 7.1E-230 |
| DEFB128 | chr20 | 168457 | 170364 | 1 | 2.5E-232 | 7.1E-230 |
| DEFB129 | chr20 | 207914 | 210463 | 1 | 2.5E-232 | 7.1E-230 |
| DEFB132 | chr20 | 238311 | 240037 | 1 | 2.5E-232 | 7.1E-230 |
| C20orf96 | chr20 | 251830 | 271344 | 1 | 2.5E-232 | 7.1E-230 |
| ZCCHC3 | chr20 | 278275 | 279507 | 1 | 2.5E-232 | 7.1E-230 |
| SOX12 | chr20 | 306616 | 307517 | 1 | 2.5E-232 | 7.1E-230 |
| NRSN2 | chr20 | 330301 | 334351 | 1 | 2.5E-232 | 7.1E-230 |
| TRIB3 | chr20 | 363164 | 377415 | 1 | 2.5E-232 | 7.1E-230 |
| RBCK1 | chr20 | 389302 | 411148 | 1 | 2.5E-232 | 7.1E-230 |

Supplementary Table S3. List of copy number alterations and affected genes in SCC-R cells

| Gene | Chromosome | Start co-ordinate | End co-ordinate | Copy number | P-value | Q-value |
|---|---|---|---|---|---|---|
| TBC1D20 | chr20 | 419168 | 443188 | 1 | 2.5E-232 | 7.1E-230 |
| CSNK2A1 | chr20 | 459241 | 489102 | 1 | 2.5E-232 | 7.1E-230 |
| TCF15 | chr20 | 585228 | 590876 | 1 | 2.5E-232 | 7.1E-230 |
| SRXN1 | chr20 | 633563 | 633766 | 1 | 2.5E-232 | 7.1E-230 |
| SCRT2 | chr20 | 644306 | 645139 | 1 | 2.5E-232 | 7.1E-230 |
| SLC52A3 | chr20 | 741675 | 746442 | 1 | 2.5E-232 | 7.1E-230 |
| ANGPT4 | chr20 | 853600 | 896901 | 1 | 2.5E-232 | 7.1E-230 |
| RSPO4 | chr20 | 941008 | 982841 | 1 | 2.5E-232 | 7.1E-230 |
| PSMF1 | chr20 | 1099428 | 1145745 | 1 | 2.5E-232 | 7.1E-230 |
| TMEM74B | chr20 | 1161485 | 1164502 | 1 | 2.5E-232 | 7.1E-230 |
| C20orf202 | chr20 | 1184065 | 1187831 | 1 | 2.5E-232 | 7.1E-230 |
| RAD21L1 | chr20 | 1209048 | 1235090 | 1 | 2.5E-232 | 7.1E-230 |
| SNPH | chr20 | 1247225 | 1286680 | 1 | 2.5E-232 | 7.1E-230 |
| SDCBP2 | chr20 | 1291067 | 1301119 | 1 | 2.5E-232 | 7.1E-230 |
| AL136531 | chr20 | 1316927 | 1317333 | 1 | 2.5E-232 | 7.1E-230 |
| FKBP1A | chr20 | 1350693 | 1373585 | 1 | 2.5E-232 | 7.1E-230 |
| NSFL1C | chr20 | 1424332 | 1447481 | 1 | 2.5E-232 | 7.1E-230 |
| SIRPB2 | chr20 | 1456735 | 1472018 | 1 | 2.5E-232 | 7.1E-230 |
| SIRPD | chr20 | 1514996 | 1518039 | 1 | 2.5E-232 | 7.1E-230 |
| SIRPB1 | chr20 | 1546711 | 1600604 | 1 | 2.5E-232 | 7.1E-230 |
| SIRPG | chr20 | 1610806 | 1638375 | 1 | 2.5E-232 | 7.1E-230 |
| SIRPA | chr20 | 1875990 | 1918244 | 1 | 2.5E-232 | 7.1E-230 |
| PDYN | chr20 | 1960917 | 1963805 | 1 | 2.5E-232 | 7.1E-230 |
| STK35 | chr20 | 2082525 | 2124571 | 1 | 2.5E-232 | 7.1E-230 |
| TGM3 | chr20 | 2276572 | 2321668 | 1 | 2.5E-232 | 7.1E-230 |
| TGM6 | chr20 | 2361564 | 2413363 | 1 | 2.5E-232 | 7.1E-230 |
| SNRPB | chr20 | 2442332 | 2451488 | 1 | 2.5E-232 | 7.1E-230 |
| ZNF343 | chr20 | 2463874 | 2474556 | 1 | 2.5E-232 | 7.1E-230 |
| TMC2 | chr20 | 2517202 | 2621963 | 1 | 2.5E-232 | 7.1E-230 |
| SNORD56 | chr20 | 2637138 | 2637390 | 1 | 2.5E-232 | 7.1E-230 |
| SNORD57 | chr20 | 2637392 | 2637732 | 1 | 2.5E-232 | 7.1E-230 |
| IDH3B | chr20 | 2639316 | 2644860 | 1 | 2.5E-232 | 7.1E-230 |
| EBF4 | chr20 | 2673557 | 2739638 | 1 | 2.5E-232 | 7.1E-230 |
| CPXM1 | chr20 | 2774890 | 2781176 | 1 | 2.5E-232 | 7.1E-230 |
| TMEM239 | chr20 | 2795823 | 2797695 | 1 | 2.5E-232 | 7.1E-230 |
| C20orf141 | chr20 | 2796188 | 2796477 | 1 | 2.5E-232 | 7.1E-230 |
| PCED1A | chr20 | 2816066 | 2820538 | 1 | 2.5E-232 | 7.1E-230 |
| VPS16 | chr20 | 2821361 | 2847167 | 1 | 2.5E-232 | 7.1E-230 |
| PTPRA | chr20 | 2903868 | 3018785 | 1 | 2.5E-232 | 7.1E-230 |
| GNRH2 | chr20 | 3025024 | 3026481 | 1 | 2.5E-232 | 7.1E-230 |
| MRPS26 | chr20 | 3026751 | 3028610 | 1 | 2.5E-232 | 7.1E-230 |
| OXT | chr20 | 3052198 | 3053140 | 1 | 2.5E-232 | 7.1E-230 |
| AVP | chr20 | 3063273 | 3065305 | 1 | 2.5E-232 | 7.1E-230 |
| UBOX5 | chr20 | 3090806 | 3104065 | 1 | 2.5E-232 | 7.1E-230 |
| FASTKD5 | chr20 | 3127410 | 3129764 | 1 | 2.5E-232 | 7.1E-230 |
| LZTS3 | chr20 | 3145039 | 3147764 | 1 | 2.5E-232 | 7.1E-230 |
| DDRGK1 | chr20 | 3171337 | 3185328 | 1 | 2.5E-232 | 7.1E-230 |
| ITPA | chr20 | 3190133 | 3204114 | 1 | 2.5E-232 | 7.1E-230 |
| SLC4A11 | chr20 | 3208382 | 3219847 | 1 | 2.5E-232 | 7.1E-230 |

Supplementary Table S3. List of copy number alterations and affected genes in SCC-R cells

| Gene | Chromosome | Start co-ordinate | End co-ordinate | Copy number | P-value | Q-value |
|---|---|---|---|---|---|---|
| C20orf194 | chr20 | 3233211 | 3388288 | 1 | 2.5E-232 | 7.1E-230 |
| ATRN | chr20 | 3451712 | 3627514 | 1 | 1.2E-215 | 3.4E-213 |
| GFRA4 | chr20 | 3640520 | 3644121 | 1 | 1.2E-215 | 3.4E-213 |
| ADAM33 | chr20 | 3649516 | 3662714 | 1 | 1.2E-215 | 3.4E-213 |
| SIGLEC1 | chr20 | 3669069 | 3687795 | 1 | 1.2E-215 | 3.4E-213 |
| HSPA12B | chr20 | 3719217 | 3732824 | 1 | 1.2E-215 | 3.4E-213 |
| C20orf27 | chr20 | 3734299 | 3740743 | 1 | 1.2E-215 | 3.4E-213 |
| SPEF1 | chr20 | 3758857 | 3761922 | 1 | 1.2E-215 | 3.4E-213 |
| CENPB | chr20 | 3765295 | 3767104 | 1 | 1.2E-215 | 3.4E-213 |
| CDC25B | chr20 | 3767721 | 3785542 | 1 | 1.2E-215 | 3.4E-213 |
| AP5S1 | chr20 | 3802809 | 3805004 | 1 | 1.2E-215 | 3.4E-213 |
| MAVS | chr20 | 3835241 | 3847715 | 1 | 1.2E-215 | 3.4E-213 |
| PANK2 | chr20 | 3869743 | 3904030 | 1 | 1.2E-215 | 3.4E-213 |
| RNF24 | chr20 | 3914705 | 3955001 | 1 | 1.2E-215 | 3.4E-213 |
| SMOX | chr20 | 4123790 | 4168132 | 1 | 1.2E-215 | 3.4E-213 |
| ADRA1D | chr20 | 4202161 | 4229651 | 1 | 1.2E-215 | 3.4E-213 |
| PRNP | chr20 | 4679815 | 4680815 | 1 | 1.2E-215 | 3.4E-213 |
| PRND | chr20 | 4705191 | 4705740 | 1 | 1.2E-215 | 3.4E-213 |
| PRNT | chr20 | 4713031 | 4713331 | 1 | 1.2E-215 | 3.4E-213 |
| RASSF2 | chr20 | 4764812 | 4781749 | 1 | 1.2E-215 | 3.4E-213 |
| SLC23A2 | chr20 | 4837633 | 4913276 | 1 | 1.2E-215 | 3.4E-213 |
| TMEM230 | chr20 | 5081529 | 5093597 | 1 | 1.2E-215 | 3.4E-213 |
| PCNA | chr20 | 5095948 | 5100376 | 1 | 1.2E-215 | 3.4E-213 |
| CDS2 | chr20 | 5107695 | 5170872 | 1 | 1.2E-215 | 3.4E-213 |
| PROKR2 | chr20 | 5282659 | 5297146 | 1 | 1.2E-215 | 3.4E-213 |
| LINC00654 | chr20 | 5482192 | 5482464 | 1 | 1.2E-215 | 3.4E-213 |
| GPCPD1 | chr20 | 5528297 | 5585156 | 1 | 1.2E-215 | 3.4E-213 |
| C20orf196 | chr20 | 5737452 | 5844118 | 1 | 1.2E-215 | 3.4E-213 |
| CHGB | chr20 | 5892221 | 5905812 | 1 | 1.2E-215 | 3.4E-213 |
| TRMT6 | chr20 | 5919174 | 5931030 | 1 | 1.2E-215 | 3.4E-213 |
| MCM8 | chr20 | 5932652 | 5975393 | 1 | 1.2E-215 | 3.4E-213 |
| CRLS1 | chr20 | 5986944 | 6017890 | 1 | 1.2E-215 | 3.4E-213 |
| LRRN4 | chr20 | 6021608 | 6033532 | 1 | 1.2E-215 | 3.4E-213 |
| FERMT1 | chr20 | 6056999 | 6100252 | 1 | 1.2E-215 | 3.4E-213 |
| BMP2 | chr20 | 6750739 | 6759756 | 1 | 1.2E-215 | 3.4E-213 |
| HAO1 | chr20 | 7864140 | 7921145 | 1 | 1.2E-215 | 3.4E-213 |
| TMX4 | chr20 | 7962855 | 8000214 | 1 | 1.2E-215 | 3.4E-213 |
| PLCB1 | chr20 | 8113285 | 8862527 | 1 | 1.2E-215 | 3.4E-213 |
| PLCB4 | chr20 | 9288420 | 9459710 | 1 | 1.2E-215 | 3.4E-213 |
| LAMP5 | chr20 | 9495488 | 9510460 | 1 | 1.1E-20 | 9.3E-19 |
| PAK7 | chr20 | 9520056 | 9624996 | 1 | 1.1E-20 | 9.3E-19 |
| GART | chr21 | 34876409 | 34915539 | 1 | 8.4E-38 | 1.1E-35 |
| SON | chr21 | 34918480 | 34948772 | 1 | 8.4E-38 | 1.1E-35 |
| AP000304 | chr21 | 34931822 | 35209268 | 1 | 8.4E-38 | 1.1E-35 |
| DONSON | chr21 | 34947936 | 34960991 | 1 | 8.4E-38 | 1.1E-35 |
| CRYZL1 | chr21 | 34962098 | 34975863 | 1 | 8.4E-38 | 1.1E-35 |

**Supplementary Table S4. List of all proteins identified in SCC-S and SCC-R cells using SequestHT and Mascot search algorithms and their gene ontology annotation**

| Gene Symbol | RefSeq_Accession | Entrez Gene ID | Description | Coverage (%) | Proteins | Unique Peptides | # Peptides | PSM | Area | SCC-R /SCC-S | SCC-R /SCC-S (Count) | SCC-R /SCC-S (Variability [%]) | # AAs | MW [kDa] | calc. pI | Biological Process | Localization | Molecular Function | Primary Localization |
|---|---|---|---|---|---|---|---|---|---|---|---|---|---|---|---|---|---|---|---|
| SERPINE1 | NP_000593.1 | 5054 | plasminogen activator inhibitor 1 precursor | 33.6 | 1 | 10 | 10 | 33 | 8.32E+08 | 74.48 | 1 | - | 402 | 45 | 7.2 | Protein metabolism | Extracellular | Protease inhibitor activity | Extracellular |
| ANPEP | NP_001141.2 | 290 | aminopeptidase N precursor | 56.6 | 1 | 47 | 47 | 218 | 3.18E+09 | 63.07 | 1 | - | 967 | 109.5 | 5.5 | Protein metabolism | Plasma membrane;Extracellular | Metallopeptidase activity | Plasma membrane |
| ISG15 | NP_005092.1 | 9636 | ubiquitin-like protein ISG15 precursor | 30.9 | 1 | 4 | 4 | 7 | 3.29E+08 | 43.19 | 1 | - | 165 | 17.9 | 7.4 | Immune response | Extracellular;Cytoplasm | Cytokine activity | Extracellular |
| HBB | NP_000509.1 | 3043 | hemoglobin subunit beta | 89.8 | 2 | 9 | 14 | 73 | 5.55E+08 | 40.95 | 1 | - | 147 | 16 | 7.3 | Transport | Extracellular | Transporter activity | Extracellular |
| AHNAK2 | NP_612429.2 | 113146 | protein AHNAK2 | 34.3 | 1 | 65 | 65 | 122 | 9.17E+08 | 39.92 | 6 | 22.8 | 5795 | 616.2 | 5.4 | Transport | Nucleus | Molecular function unknown | Nucleus |
| IFIT2 | NP_001538.4 | 3433 | interferon-induced protein with tetratricopeptide repeats 2 | 49.8 | 1 | 17 | 17 | 57 | 8.95E+08 | 36.75 | 2 | 71.8 | 472 | 54.6 | 6.8 | Cell growth and/or maintenance | Cytoplasm;Nucleus | Cytoskeletal protein binding | Cytoplasm |
| NT5E | NP_002517.1 | 4907 | 5'-nucleotidase isoform 1 preproprotein | 51.2 | 2 | 23 | 23 | 141 | 2.30E+09 | 35.98 | 19 | 33.3 | 574 | 63.3 | 7.0 | Regulation of nucleobase, nucleoside, nucleotide and nucleic acid metabolism | Plasma membrane | Hydrolase activity | Plasma membrane |
| MME | NP_000893.2 | 4311 | neprilysin | 42.8 | 1 | 23 | 23 | 56 | 9.15E+08 | 31.81 | 4 | 111.9 | 750 | 85.5 | 5.7 | Protein metabolism;Peptide metabolism | Plasma membrane;Cytoplasm;Endoplasmic reticulum;Golgi apparatus;Endosome;Extracellular matrix | Metallopeptidase activity | Plasma membrane |
| IFIT1 | NP_001539.3 | 3434 | interferon-induced protein with tetratricopeptide repeats 1 isoform 1 | 57.5 | 4 | 23 | 23 | 77 | 2.39E+09 | 29.45 | 13 | 66.3 | 478 | 55.3 | 7.2 | Biological_process unknown | Cytoplasm;Nucleolus | Nucleic acid binding;Protein binding | Cytoplasm |
| C11orf68 | NP_113638.2 | 83638 | UPF0696 protein C11orf68 isoform 2 | 24.7 | 2 | 7 | 7 | 13 | 1.25E+08 | 29.03 | 1 | - | 292 | 31.4 | 6.3 | Biological_process unknown | - | Molecular function unknown | - |
| GBP4 | NP_443173.2 | 115361 | guanylate-binding protein 4 | 27.3 | 2 | 10 | 10 | 14 | 9.64E+07 | 26.67 | 2 | 49.7 | 640 | 73.1 | 6.0 | Cell communication;Signal transduction | Cytoplasm | GTPase activator activity | Cytoplasm |
| SV2A | NP_055664.3 | 9900 | synaptic vesicle glycoprotein 2A isoform 1 | 2.4 | 1 | 1 | 1 | 1 | 1.02E+08 | 24.15 | 1 | - | 742 | 82.6 | 5.6 | Biological_process unknown | - | Neurotransmitter transporter activity | - |
| NR2C2 | NP_003289.2 | 7182 | nuclear receptor subfamily 2 group C member 2 isoform 1 | 4.2 | 1 | 1 | 1 | 1 | 6.78E+08 | 23.77 | 1 | - | 615 | 67.3 | 6.3 | Cell communication;Signal transduction | Nucleus | Ligand-dependent nuclear receptor activity | Nucleus |
| MX1 | NP_001138397.1 | 4599 | interferon-induced GTP-binding protein Mx1 isoform a | 81.0 | 2 | 53 | 57 | 286 | 4.05E+09 | 23.46 | 3 | 103.5 | 662 | 75.5 | 5.8 | Cell communication;Signal transduction | Cytoplasm;Endoplasmic reticulum;Nucleus | GTPase activity | Cytoplasm |
| RASA3 | NP_031394.2 | 22821 | ras GTPase-activating protein 3 | 8.6 | 1 | 3 | 3 | 5 | 9.60E+07 | 23.24 | 2 | 61.6 | 834 | 95.6 | 7.2 | Cell communication;Signal transduction | Plasma membrane;Cytoplasm | GTPase activator activity | Plasma membrane |
| PLSCR1 | NP_066928.1 | 5359 | phospholipid scramblase 1 | 18.9 | 1 | 4 | 4 | 9 | 3.42E+08 | 21.03 | 1 | - | 318 | 35 | 4.9 | Cell communication;Signal transduction | Plasma membrane | Protein binding | Plasma membrane |
| PML | NP_150241.2 | 5371 | protein PML isoform 1 | 33.2 | 7 | 2 | 17 | 26 | 4.32E+08 | 19.93 | 1 | - | 882 | 97.5 | 6.2 | Regulation of gene expression, epigenetic | Nucleus;Cytoplasm;Nucleolus;Centrosome;Nuclear matrix | Transcription regulator activity;Transporter activity | Nucleus;Cytoplasm |
| ME3 | NP_001014811.1 | 10873 | NADP-dependent malic enzyme, mitochondrial | 6.8 | 1 | 2 | 2 | 2 | 1.28E+08 | 19.13 | 1 | - | 604 | 67 | 8.0 | Metabolism;Energy pathways | Mitochondrion;Cytoplasm | Oxidoreductase activity | Mitochondrion |
| SH3KBP1 | NP_001171889.1 | 30011 | SH3 domain-containing kinase-binding protein 1 isoform c | 27.4 | 3 | 5 | 5 | 6 | 1.21E+08 | 18.84 | 2 | 19.4 | 427 | 46.6 | 8.8 | Cell communication;Signal transduction;Regulation of signal transduction;Cell adhesion | Cytoplasm;Nucleus;Endosome;Golgi apparatus;Cytoskeleton;Cell projection | Receptor signaling complex scaffold activity | Cytoplasm |
| LGALS1 | NP_002296.1 | 3956 | galectin-1 | 74.8 | 1 | 11 | 11 | 219 | 1.86E+10 | 18.67 | 23 | 26.7 | 135 | 14.7 | 5.5 | Immune response | Nucleus;Cytoplasm;Extracellular;Plasma membrane | Receptor binding | Extracellular |
| DDX58 | NP_055129.2 | 23586 | probable ATP-dependent RNA helicase DDX58 | 34.0 | 1 | 25 | 25 | 48 | 3.26E+08 | 18.55 | 9 | 116.2 | 925 | 106.5 | 6.4 | Regulation of nucleobase, nucleoside, nucleotide and nucleic acid metabolism | Cytoplasm | Molecular function unknown | Cytoplasm |
| MT2A | NP_005944.1 | 4502 | metallothionein-2 | 67.2 | 4 | 1 | 4 | 28 | 1.80E+09 | 16.84 | 5 | 13 | 61 | 6 | 7.8 | Biological_process unknown | Cytoplasm;Nucleus | Molecular function unknown | Nucleus |
| PTTG1IP | NP_004330.1 | 754 | pituitary tumor-transforming gene 1 protein-interacting protein isoform 1 precursor | 10.0 | 1 | 1 | 1 | 6 | 2.99E+08 | 16.57 | 1 | - | 180 | 20.3 | 8.8 | Transport | Nucleus;Cytoplasm | Transporter activity | Nucleus |
| SOD2 | NP_000627.2 | 6648 | superoxide dismutase [Mn], mitochondrial isoform A precursor | 62.6 | 2 | 11 | 11 | 108 | 4.36E+09 | 16.53 | 17 | 18.3 | 222 | 24.7 | 8.3 | Cell proliferation;Anti-apoptosis;Cell growth and/or maintenance | Mitochondrion | Superoxide dismutase activity | Mitochondrion |
| MYEF2 | NP_057216.2 | 50804 | myelin expression factor 2 | 26.0 | 1 | 8 | 8 | 12 | 5.25E+07 | 14.71 | 2 | 21.4 | 600 | 64.1 | 8.9 | Regulation of nucleobase, nucleoside, nucleotide and nucleic acid metabolism | Nucleus | Transcription regulator activity | Nucleus |
| HMGA2 | NP_003474.1 | 8091 | high mobility group protein HMGI-C isoform a | 41.3 | 2 | 5 | 5 | 18 | 2.02E+09 | 14.55 | 5 | 15.4 | 109 | 11.8 | 10.6 | Regulation of nucleobase, nucleoside, nucleotide and nucleic acid metabolism | Nucleus | Transcription factor activity | Nucleus |
| SMAD3 | NP_001138574.1 | 4088 | mothers against decapentaplegic homolog 3 isoform 2 | 11.9 | 8 | 2 | 2 | 3 | 1.77E+07 | 13.59 | 1 | - | 320 | 35.9 | 5.4 | Regulation of nucleobase, nucleoside, nucleotide and nucleic acid metabolism | Nucleus;Cytoplasm | Transcription regulator activity | Nucleus |
| S100A4 | NP_002952.1 | 6275 | protein S100-A4 | 19.8 | 1 | 2 | 2 | 5 | 4.90E+08 | 13.48 | 3 | 5.9 | 101 | 11.7 | 6.1 | Cell growth and/or maintenance | Cytoplasm;Nucleus;Nucleolus | Calcium ion binding | Cytoplasm |
| VIM | NP_003371.2 | 7431 | vimentin | 92.9 | 8 | 68 | 71 | 1875 | 2.29E+10 | 12.26 | 7 | 94.2 | 466 | 53.6 | 5.1 | Cell growth and/or maintenance | Intermediate filament;Nucleolus;Extracellular;Membrane fraction;Golgi apparatus;Endoplasmic | Structural constituent of cytoskeleton | Intermediate filament |
| JUN | NP_002219.1 | 3725 | transcription factor AP-1 | 25.1 | 1 | 4 | 4 | 6 | 6.85E+07 | 12.07 | 2 | 75.5 | 331 | 35.7 | 8.8 | Regulation of nucleobase, nucleoside, nucleotide and nucleic acid metabolism | Nucleus;Cytoplasm | Transcription factor activity | Nucleus |
| SAMD9L | NP_689916.2 | 219285 | sterile alpha motif domain-containing protein 9-like | 3.9 | 1 | 3 | 3 | 4 | 7.78E+07 | 12.01 | 2 | 1.4 | 1584 | 184.4 | 8.0 | Biological_process unknown | Nucleus | Molecular function unknown | Nucleus |
| MT1E | NP_783316.2 | 4493 | metallothionein-1E | 67.2 | 3 | 1 | 4 | 26 | 2.64E+09 | 11.75 | 3 | 5.6 | 61 | 6 | 8.0 | Transport | Nucleus;Cytoplasm | Transporter activity | Nucleus |
| USP13 | NP_003931.2 | 8975 | ubiquitin carboxyl-terminal hydrolase 13 | 10.0 | 1 | 4 | 4 | 4 | 5.74E+07 | 11.67 | 1 | - | 863 | 97.3 | 5.5 | Protein metabolism | - | Ubiquitin-specific protease activity | - |
| PKM | NP_001193725.1 | 5315 | pyruvate kinase PKM isoform c | 66.8 | 4 | 3 | 46 | 1652 | 2.34E+10 | 11.21 | 1 | - | 605 | 65.9 | 7.9 | Energy pathways;Metabolism | Cytoplasm;Perinuclear region;Extracellular;Cytosol | Catalytic activity;Kinase activity | Cytoplasm |
| AXL | NP_001265528.1 | 558 | tyrosine-protein kinase receptor UFO isoform 3 | 13.7 | 3 | 6 | 6 | 7 | 1.23E+08 | 11.16 | 1 | - | 626 | 69.2 | 5.5 | Cell communication;Signal transduction | Plasma membrane;Extracellular | Transmembrane receptor protein tyrosine kinase activity | Plasma membrane |
| ABCD1 | NP_000024.2 | 215 | ATP-binding cassette sub-family D member 1 | 1.9 | 1 | 1 | 1 | 1 | 4.50E+07 | 11.11 | 1 | - | 745 | 82.9 | 9.0 | Metabolism;Energy pathways | Peroxisome | ATPase activity | Peroxisome |
| CD99L2 | NP_001171737.1 | 83692 | CD99 antigen-like protein 2 isoform 4 precursor | 18.5 | 5 | 2 | 2 | 4 | 9.31E+07 | 11.08 | 1 | - | 189 | 20 | 5.0 | Biological_process unknown | Extracellular | Molecular function unknown | Extracellular |

Jain *et al.*, 2019. MAP2K1 is a potential therapeutic target in erlotinib resistant head and neck squamous cell carcinoma
Supplementary Table S4. List of all proteins identified in SCC-S and SCC-R cells using SequestHT and Mascot search algorithms and their gene ontology annotation

| Gene Symbol | RefSeq_Accession | Entrez Gene ID | Description | Coverage (%) | Proteins | Unique Peptides | # Peptides | PSM | Area | SCC-R /SCC-S | SCC-R /SCC-S (Count) | SCC-R /SCC-S (Variability [%]) | # AAs | MW [kDa] | calc. pI | Biological Process | Localization | Molecular Function | Primary Localization |
|---|---|---|---|---|---|---|---|---|---|---|---|---|---|---|---|---|---|---|---|
| HBA1 | NP_000549.1 | 3039 | hemoglobin subunit alpha | 88.7 | 1 | 10 | 10 | 80 | 5.75E+08 | 10.90 | 2 | 897 | 142 | 15.2 | 8.7 | Transport | - | Transporter activity | - |
| DHX58 | NP_077024.2 | 79132 | probable ATP-dependent RNA helicase DHX58 | 3.0 | 1 | 1 | 1 | 1 | 1.49E+07 | 10.51 | 1 | - | 678 | 76.6 | 7.4 | Regulation of nucleobase, nucleoside, nucleotide and nucleic acid metabolism | Cytoplasm | Nucleotide binding | Cytoplasm |
| SDCBP | NP_001007070.1 | 6386 | syntenin-1 isoform 3 | 67.3 | 3 | 13 | 13 | 45 | 6.65E+08 | 9.83 | 7 | 17.3 | 297 | 32.3 | 7.5 | Cell communication;Signal transduction | Nucleus;Synaptic vesicle;Plasma membrane;Cytosol;Microsome;Cell junction;Cytoplasm;Intracellular vesicle;Endoplasmic | Receptor signaling complex scaffold activity | Plasma membrane |
| CD97 | NP_001775.2 | 976 | CD97 antigen isoform 2 preproprotein | 21.0 | 5 | 9 | 9 | 15 | 1.21E+08 | 9.77 | 5 | 26.1 | 742 | 81.7 | 7.1 | Cell communication;Signal transduction | Plasma membrane | Receptor activity | Plasma membrane |
| TM7SF3 | NP_057635.1 | 51768 | transmembrane 7 superfamily member 3 precursor | 2.8 | 1 | 1 | 1 | 2 | 3.18E+07 | 9.76 | 1 | - | 570 | 64.1 | 7.0 | Biological_process unknown | Plasma membrane | Molecular function unknown | Plasma membrane |
| DPYSL2 | NP_001231533.1 | 1808 | dihydropyrimidinase-related protein 2 isoform 3 | 57.1 | 9 | 16 | 16 | 40 | 1.72E+09 | 9.70 | 8 | 18.2 | 536 | 58.1 | 6.2 | Cell communication;Signal transduction | Cytoplasm;Nucleus;Microtubule | Cytoskeletal protein binding | Cytoplasm |
| RAB32 | NP_006825.1 | 10981 | ras-related protein Rab-32 | 34.7 | 1 | 4 | 4 | 6 | 2.15E+08 | 9.50 | 2 | 71.8 | 225 | 25 | 6.5 | Cell communication;Signal transduction | Mitochondrion;Plasma membrane | GTPase activity | Mitochondrion |
| ARHGAP10 | NP_078881.3 | 79658 | rho GTPase-activating protein 10 | 2.5 | 1 | 1 | 1 | 1 | 9.68E+08 | 9.45 | 1 | - | 786 | 89.3 | 7.2 | Cell communication;Signal transduction | Cytoplasm | GTPase activator activity | Cytoplasm |
| SQSTM1 | NP_003891.1 | 8878 | sequestosome-1 isoform 1 | 83.4 | 2 | 22 | 22 | 92 | 2.18E+09 | 9.20 | 18 | 46.6 | 440 | 47.7 | 5.2 | Protein metabolism | Cytoplasm;Nucleus;Endosome | Ubiquitin-specific protease activity | Cytoplasm;Nucleus |
| CTSL | NP_666023.1 | 1514 | cathepsin L1 isoform 1 preproprotein | 29.7 | 2 | 4 | 4 | 6 | 5.76E+08 | 8.83 | 1 | - | 333 | 37.5 | 5.5 | Protein metabolism | Endosome;Lysosome | Cysteine-type peptidase activity | Endosome |
| PODXL | NP_005388.2 | 5420 | podocalyxin isoform 2 precursor | 30.2 | 2 | 9 | 9 | 23 | 1.65E+09 | 8.37 | 5 | 48.7 | 526 | 55.4 | 5.6 | Cell adhesion | Integral to membrane | Molecular function unknown | Integral to membrane |
| TGFB1I1 | NP_001035919.1 | 7041 | transforming growth factor beta-1-induced transcript 1 protein isoform 1 | 22.1 | 2 | 5 | 5 | 9 | 1.27E+08 | 8.22 | 2 | 72.1 | 461 | 49.8 | 7.0 | Cell communication;Signal transduction | Nucleus;Microtubule | Transcription regulator activity | Nucleus;Microtubule |
| BCAR3 | NP_001248338.1 | 8412 | breast cancer anti-estrogen resistance protein 3 isoform 1 | 33.2 | 2 | 14 | 14 | 14 | 2.22E+08 | 8.18 | 3 | 60.5 | 825 | 92.5 | 8.0 | Cell communication;Signal transduction | Nucleus | Guanyl-nucleotide exchange factor activity | Nucleus |
| ABR | NP_001269078.1 | 29 | active breakpoint cluster region-related protein isoform e | 17.9 | 5 | 5 | 6 | 6 | 5.79E+07 | 8.11 | 1 | - | 641 | 73.5 | 7.7 | Cell communication;Signal transduction | Cytoplasm | GTPase activator activity | Cytoplasm |
| ANXA6 | NP_001146.2 | 309 | annexin A6 isoform 1 | 46.4 | 2 | 24 | 24 | 61 | 7.56E+08 | 8.05 | 14 | 19.7 | 673 | 75.8 | 5.6 | Cell communication;Signal transduction | Endoplasmic reticulum;Plasma membrane;Cytoplasm;Mitochondrion | Calcium ion binding | Endoplasmic reticulum |
| CLN3 | NP_001273033.1 | 1201 | battenin isoform b | 3.4 | 2 | 1 | 1 | 1 | 3.31E+07 | 8.03 | 1 | - | 414 | 45.1 | 6.4 | Protein metabolism | Mitochondrion;Golgi apparatus;Cytoplasm;Endosome;Lysosome;Nucleus;Endoplasmic reticulum;Plasma membrane | Chaperone activity | Golgi apparatus |
| SAMD4B | NP_060498.2 | 55095 | protein Smaug homolog 2 | 17.9 | 1 | 7 | 7 | 9 | 6.55E+07 | 7.96 | 2 | 42.7 | 694 | 75.4 | 6.8 | Biological_process unknown | - | Molecular function unknown | - |
| SP100 | NP_001073860.1 | 6672 | nuclear autoantigen Sp-100 isoform 1 | 21.1 | 5 | 8 | 8 | 14 | 1.54E+08 | 7.87 | 2 | 185.7 | 885 | 101.5 | 7.4 | Regulation of nucleobase, nucleoside, nucleotide and nucleic acid metabolism | Nucleus;Cytoplasm;Nucleoplasm | Transcription regulator activity | Nucleus;Cytoplasm |
| EIF4A2 | NP_001958.2 | 1974 | eukaryotic initiation factor 4A-II | 57.7 | 1 | 10 | 21 | 194 | 5.23E+09 | 7.76 | 4 | 14.3 | 407 | 46.4 | 5.5 | Protein metabolism | Cytoplasm | Translation regulator activity | Cytoplasm |
| TFE3 | NP_006512.2 | 7030 | transcription factor E3 isoform 1 | 3.3 | 1 | 1 | 1 | 1 | 1.31E+08 | 7.51 | 1 | - | 575 | 61.5 | 5.6 | Regulation of nucleobase, nucleoside, nucleotide and nucleic acid metabolism | Nucleus;Cytoplasm | Transcription factor activity | Nucleus |
| FNDC3B | NP_001128567.1 | 64778 | fibronectin type III domain-containing protein 3B | 1.2 | 1 | 1 | 1 | 1 | 1.85E+06 | 7.48 | 1 | - | 1204 | 132.8 | 6.0 | Biological_process unknown | - | Molecular function unknown | - |
| C1orf85 | NP_001243534.1 | 112770 | lysosomal protein NCU-G1 isoform 3 | 14.4 | 5 | 2 | 2 | 2 | 1.04E+08 | 7.45 | 1 | - | 320 | 34.6 | 6.5 | Biological_process unknown | Integral to membrane | Molecular function unknown | Integral to membrane |
| ITGA5 | NP_002196.2 | 3678 | integrin alpha-5 precursor | 4.3 | 1 | 2 | 2 | 3 | 8.59E+07 | 7.30 | 1 | - | 1049 | 114.5 | 5.8 | Cell communication;Signal transduction | Plasma membrane | Receptor activity | Plasma membrane |
| HELZ2 | NP_001032412.2 | 85441 | helicase with zinc finger domain 2 isoform 1 | 7.2 | 2 | 11 | 11 | 13 | 9.64E+07 | 7.17 | 2 | 123.1 | 2649 | 294.5 | 7.5 | Biological_process unknown | Nucleus | Molecular function unknown | Nucleus |
| ELMO2 | NP_877496.1 | 63916 | engulfment and cell motility protein 2 | 6.5 | 1 | 3 | 3 | 3 | 1.30E+07 | 7.05 | 1 | - | 720 | 82.6 | 5.9 | Signal transduction;Cell growth and/or maintenance;Cell migration | Cytoplasm;Plasma membrane | Molecular function unknown | Cytoplasm |
| RRAS | NP_006261.1 | 6237 | ras-related protein R-Ras precursor | 14.2 | 1 | 1 | 3 | 8 | 9.14E+07 | 6.99 | 1 | - | 218 | 23.5 | 6.9 | Cell communication;Signal transduction | Plasma membrane;Endoplasmic reticulum | GTPase activity | Plasma membrane |
| CDKN1B | NP_004055.1 | 1027 | cyclin-dependent kinase inhibitor 1B | 20.7 | 1 | 2 | 2 | 2 | 1.11E+07 | 6.96 | 1 | - | 198 | 22.1 | 7.0 | Cell communication;Signal transduction | Nucleus;Cytoplasm;Apical membrane | Kinase regulator activity | Nucleus |
| TFPI | NP_001027452.1 | 7035 | tissue factor pathway inhibitor isoform b precursor | 8.4 | 2 | 1 | 1 | 2 | 2.07E+08 | 6.90 | 1 | - | 251 | 28.6 | 5.3 | Protein metabolism | Extracellular;Cytoplasm;Plasma membrane | Protease inhibitor activity | Extracellular |
| ICAM1 | NP_000192.2 | 3383 | intercellular adhesion molecule 1 precursor | 27.3 | 1 | 12 | 12 | 37 | 4.43E+08 | 6.88 | 11 | 67.6 | 532 | 57.8 | 8.0 | Cell communication;Signal transduction | Plasma membrane;Cytoplasm;Extracellular | Cell adhesion molecule activity | Plasma membrane |
| SEPT15 | NP_004252.2 | 9403 | 15 kDa selenoprotein isoform 1 precursor | 15.8 | 1 | 2 | 2 | 2 | 6.94E+07 | 6.73 | 1 | - | 165 | 18.1 | 5.0 | Protein folding | Endoplasmic reticulum | Protein binding | Endoplasmic reticulum |
| ENO2 | NP_001966.1 | 2026 | gamma-enolase | 60.1 | 1 | 12 | 15 | 267 | 4.44E+09 | 6.73 | 9 | 16.1 | 434 | 47.2 | 5.0 | Metabolism;Energy pathways | Cytoplasm | Catalytic activity | Cytoplasm |
| CEP170 | NP_001035864.1 | 9859 | centrosomal protein of 170 kDa isoform gamma | 20.8 | 4 | 17 | 17 | 22 | 2.87E+08 | 6.55 | 9 | 67.7 | 1460 | 161.3 | 7.0 | Biological_process unknown | Nucleus;Cytoplasm;Microtubule;Kinetochore | Molecular function unknown | Nucleus;Cytoplasm |
| IFIH1 | NP_071451.2 | 64135 | interferon-induced helicase C domain-containing protein 1 | 7.2 | 1 | 4 | 4 | 6 | 5.91E+07 | 6.55 | 1 | - | 1025 | 116.6 | 5.5 | Regulation of nucleobase, nucleoside, nucleotide and nucleic acid metabolism | Cytoplasm | ATPase activity | Cytoplasm |
| RBMS3 | NP_001003792.1 | 27303 | RNA-binding motif, single-stranded-interacting protein 3 isoform 3 | 11.0 | 5 | 1 | 2 | 3 | 3.10E+07 | 6.47 | 1 | - | 419 | 45.7 | 8.0 | Regulation of nucleobase, nucleoside, nucleotide and nucleic acid metabolism | Cytoplasm;Nucleus | RNA binding | Cytoplasm |
| PLOD3 | NP_001075.1 | 8985 | procollagen-lysine,2-oxoglutarate 5-dioxygenase 3 precursor | 26.7 | 1 | 13 | 13 | 22 | 4.52E+08 | 6.43 | 8 | 23.3 | 738 | 84.7 | 6.1 | Protein metabolism | Endoplasmic reticulum;Golgi apparatus | Catalytic activity | Endoplasmic reticulum |
| CDCP1 | NP_835488.1 | 64866 | CUB domain-containing protein 1 isoform 2 precursor | 10.5 | 2 | 2 | 2 | 4 | 1.06E+08 | 6.40 | 1 | - | 343 | 37.8 | 7.1 | Cell communication;Signal transduction;Cell adhesion;Cell-cell adhesion | Plasma membrane;Cytoplasm | Molecular function unknown;Cell adhesion molecule activity | Plasma membrane |
| FHL1 | NP_001161291.1 | 2273 | four and a half LIM domains protein 1 isoform 2 | 45.7 | 5 | 10 | 10 | 30 | 6.61E+08 | 6.37 | 5 | 3.8 | 280 | 31.9 | 8.3 | Cell growth and/or maintenance | Cytoplasm | Molecular function unknown | Cytoplasm |

| Gene Symbol | RefSeq_Accession | Entrez Gene ID | Description | Coverage (%) | Proteins | Unique Peptides | # Peptides | PSM | Area | SCC-R /SCC-S | SCC-R /SCC-S (Count) | SCC-R /SCC-S (Variability [%]) | # AAs | MW [kDa] | calc. pI | Biological Process | Localization | Molecular Function | Primary Localization |
|---|---|---|---|---|---|---|---|---|---|---|---|---|---|---|---|---|---|---|---|
| PLAU | NP_001138503.1 | 5328 | urokinase-type plasminogen activator isoform 2 | 8.9 | 2 | 2 | 2 | 5 | 1.67E+08 | 6.28 | 3 | 9.8 | 414 | 46.9 | 8.4 | Protein metabolism | Plasma membrane;Cytoplasm;Extracellular | Serine-type peptidase activity | Plasma membrane |
| PLD3 | NP_001026866.1 | 23646 | phospholipase D3 | 15.9 | 1 | 5 | 5 | 7 | 2.81E+08 | 5.98 | 2 | 23.5 | 490 | 54.7 | 6.5 | Metabolism;Energy pathways | Endoplasmic reticulum;Golgi apparatus | Phospholipase activity | Endoplasmic reticulum;Golgi apparatus |
| PKN1 | NP_002732.3 | 5585 | serine/threonine-protein kinase N1 isoform 2 | 6.4 | 2 | 3 | 3 | 3 | 1.70E+07 | 5.87 | 1 | - | 942 | 103.9 | 6.4 | Cell communication;Signal transduction | Cytoplasm;Nucleus | Protein serine/threonine kinase activity | Cytoplasm |
| LACTB | NP_116246.2 | 114294 | serine beta-lactamase-like protein LACTB, mitochondrial isoform a precursor | 6.8 | 2 | 2 | 2 | 3 | 3.60E+08 | 5.86 | 2 | 80.8 | 547 | 60.7 | 8.5 | Protein metabolism | Mitochondrion;Cytoplasm;Nucleus;Ribosome | Structural constituent of ribosome | Mitochondrion;Cytoplasm |
| GBP2 | NP_004111.2 | 2634 | interferon-induced guanylate-binding protein 2 | 32.3 | 1 | 8 | 11 | 19 | 2.18E+08 | 5.81 | 1 | - | 591 | 67.2 | 5.7 | Cell communication;Signal transduction | Cytoplasm | GTPase activity | Cytoplasm |
| PLOD2 | NP_000926.2 | 5352 | procollagen-lysine,2-oxoglutarate 5-dioxygenase 2 isoform 2 precursor | 2.7 | 2 | 2 | 2 | 2 | 1.12E+08 | 5.76 | 1 | - | 737 | 84.6 | 6.7 | Metabolism;Energy pathways | Endoplasmic reticulum | Catalytic activity | Endoplasmic reticulum |
| HLA-B | NP_005505.2 | 3106 | major histocompatibility complex, class I, B precursor | 49.7 | 3 | 7 | 15 | 109 | 4.31E+09 | 5.71 | 11 | 30.7 | 362 | 40.4 | 5.9 | Immune response | Plasma membrane;Extracellular;Cytoplasm | MHC class I receptor activity;MHC class II receptor activity | Plasma membrane |
| DCTN2 | NP_001248341.1 | 10540 | dynactin subunit 2 isoform 2 | 72.2 | 2 | 1 | 20 | 86 | 7.26E+08 | 5.69 | 1 | - | 403 | 44.4 | 5.2 | Cell growth and/or maintenance | Cytoplasm;Zymogen granule;Centrosome;Microtubule;Kinetochore | Motor activity | Cytoplasm |
| ACTN4 | NP_004915.2 | 81 | alpha-actinin-4 | 93.3 | 2 | 74 | 102 | 1095 | 1.24E+10 | 5.66 | 148 | 26.8 | 911 | 104.8 | 5.4 | Cell growth and/or maintenance | Cytoplasm;Nucleus;Plasma membrane;Mitochondrion | Structural constituent of cytoskeleton | Cytoplasm |
| RAP2C | NP_001258115.1 | 57826 | ras-related protein Rap-2c isoform 1 precursor | 37.2 | 3 | 3 | 6 | 10 | 1.49E+08 | 5.61 | 1 | - | 183 | 20.7 | 4.9 | Cell communication;Signal transduction | - | GTPase activity | - |
| SIPA1L3 | NP_055888.1 | 23094 | signal-induced proliferation-associated 1-like protein 3 | 0.9 | 1 | 1 | 1 | 1 | 2.09E+07 | 5.56 | 1 | - | 1781 | 194.5 | 8.3 | Biological_process unknown | - | Peptide binding | - |
| EIF4EBP1 | NP_004086.1 | 1978 | eukaryotic translation initiation factor 4E-binding protein 1 | 77.1 | 1 | 6 | 6 | 7 | 2.29E+08 | 5.54 | 2 | 132.5 | 118 | 12.6 | 5.5 | Protein metabolism | Cytoplasm;Nucleus | Translation regulator activity | Cytoplasm |
| MT1F | NP_005940.1 | 4494 | metallothionein-1F | 32.8 | 2 | 1 | 2 | 2 | 3.48E+07 | 5.54 | 1 | - | 61 | 6.1 | 7.8 | Transport | Cytoplasm;Nucleus | Transporter activity | Cytoplasm |
| EPHA2 | NP_004422.2 | 1969 | ephrin type-A receptor 2 precursor | 21.8 | 10 | 10 | 11 | 12 | 3.06E+08 | 5.49 | 4 | 44.6 | 976 | 108.2 | 6.2 | Cell communication;Signal transduction | Plasma membrane | Transmembrane receptor protein tyrosine kinase activity | Plasma membrane |
| DNMBP | NP_056036.1 | 23268 | dynamin-binding protein | 7.7 | 1 | 6 | 7 | 9 | 9.38E+07 | 5.47 | 4 | 50.2 | 1577 | 177.2 | 5.4 | Cell communication;Signal transduction | Golgi apparatus;Extracellular | Guanyl-nucleotide exchange factor activity | Extracellular |
| STAMBPL1 | NP_065850.1 | 57559 | AMSH-like protease | 2.5 | 1 | 1 | 1 | 1 | 1.12E+06 | 5.45 | 1 | - | 436 | 49.8 | 7.2 | Cell communication;Signal transduction | Nucleus;Cytoplasm | Receptor signaling complex scaffold activity | Nucleus |
| STAT1 | NP_009330.1 | 6772 | signal transducer and activator of transcription 1-alpha/beta isoform alpha | 54.5 | 2 | 35 | 35 | 179 | 3.79E+09 | 5.43 | 34 | 20.4 | 750 | 87.3 | 6.1 | Regulation of nucleobase, nucleoside, nucleotide and nucleic acid metabolism | Cytoplasm;Nucleus | Transcription factor activity | Cytoplasm |
| CYR61 | NP_001545.2 | 3491 | protein CYR61 precursor | 35.2 | 1 | 8 | 8 | 19 | 2.50E+08 | 5.39 | 4 | 42.8 | 381 | 42 | 8.2 | Cell communication;Signal transduction | Extracellular;Plasma membrane | Extracellular matrix structural constituent | Extracellular |
| OAS3 | NP_006178.2 | 4940 | 2'-5'-oligoadenylate synthase 3 | 41.5 | 1 | 31 | 31 | 81 | 5.38E+08 | 5.36 | 28 | 32.2 | 1087 | 121.1 | 8.4 | Immune response | Cytoplasm;Endoplasmic reticulum;Extracellular;Nucleus | Ligase activity | Cytoplasm |
| SH3BGRL | NP_003013.1 | 6451 | SH3 domain-binding glutamic acid-rich-like protein | 58.8 | 1 | 3 | 3 | 5 | 2.51E+07 | 5.34 | 1 | - | 114 | 12.8 | 5.3 | Cell communication;Signal transduction | - | Molecular function unknown | - |
| CTSB | NP_680092.1 | 1508 | cathepsin B preproprotein | 36.6 | 1 | 12 | 12 | 38 | 1.60E+09 | 5.32 | 7 | 24.4 | 339 | 37.8 | 6.3 | Protein metabolism;Apoptosis | Cytoplasm;Plasma membrane;Extracellular;Perinuclear region;Mitochondrion;Lysosome;Nuclear | Cysteine-type peptidase activity | Lysosome |
| HLA-A | NP_002107.3 | 3105 | HLA class I histocompatibility antigen, A-1 alpha chain precursor | 31.5 | 6 | 4 | 9 | 76 | 3.34E+09 | 5.32 | 7 | 41.2 | 365 | 40.8 | 6.0 | Immune response | Plasma membrane;Endoplasmic reticulum;Golgi apparatus;Extracellular;Nucleus | MHC class I receptor activity;MHC class II receptor activity | Plasma membrane |
| HLA-C | NP_002108.4 | 3107 | HLA class I histocompatibility antigen, Cw-1 alpha chain precursor | 41.5 | 4 | 1 | 14 | 60 | 1.16E+09 | 5.29 | 1 | - | 366 | 40.6 | 6.0 | Immune response | Plasma membrane | MHC class I receptor activity;MHC class II receptor activity | Plasma membrane |
| SFMBT1 | NP_057413.2 | 51460 | scm-like with four MBT domains protein 1 | 2.1 | 1 | 1 | 1 | 1 | 9.32E+07 | 5.25 | 1 | - | 866 | 98.1 | 6.2 | Regulation of gene expression, epigenetic | Nucleus | Transcription cofactor activity | Nucleus |
| C19orf66 | NP_060851.2 | 55337 | UPF0515 protein C19orf66 | 8.3 | 1 | 1 | 1 | 2 | 5.98E+07 | 5.12 | 1 | - | 291 | 33.1 | 7.3 | Biological_process unknown | - | Molecular function unknown | - |
| CDH13 | NP_001207418.1 | 1012 | cadherin-13 isoform 3 precursor | 3.4 | 3 | 1 | 1 | 1 | 3.20E+08 | 5.02 | 1 | - | 674 | 73.7 | 4.8 | Cell growth and/or maintenance | Plasma membrane;Extracellular | Cell adhesion molecule activity | Plasma membrane |
| RAB11FIP5 | NP_056285.1 | 26056 | rab11 family-interacting protein 5 | 8.0 | 1 | 2 | 3 | 3 | 4.34E+07 | 4.97 | 1 | - | 653 | 70.4 | 9.2 | Transport | Mitochondrion;Microsome;Endosome;Plasma membrane;Nucleus;Cytoplasm | Transporter activity | Mitochondrion |
| PARP9 | NP_001139578.1 | 83666 | poly [ADP-ribose] polymerase 9 isoform c | 17.6 | 3 | 6 | 6 | 11 | 2.34E+08 | 4.90 | 2 | 24.9 | 710 | 80.2 | 8.1 | Biological_process unknown | Nucleus | Molecular function unknown | Nucleus |
| PFN2 | NP_002619.1 | 5217 | profilin-2 isoform b | 74.3 | 2 | 7 | 7 | 34 | 3.26E+08 | 4.89 | 6 | 13.3 | 140 | 15.1 | 6.1 | Cell growth and/or maintenance | Cytoplasm | Structural molecule activity | Cytoplasm |
| SMYD5 | NP_006053.2 | 10322 | SET and MYND domain-containing protein 5 | 9.1 | 1 | 3 | 3 | 3 | 7.09E+07 | 4.86 | 2 | 1692.4 | 418 | 47.3 | 5.1 | Regulation of nucleobase, nucleoside, nucleotide and nucleic acid metabolism | - | Methyltransferase activity | - |
| S100A6 | NP_055439.1 | 6277 | protein S100-A6 | 58.9 | 1 | 13 | 13 | 208 | 9.71E+09 | 4.82 | 24 | 13.5 | 90 | 10.2 | 5.5 | Cell communication;Signal transduction | Cytoplasm;Nucleus | Calcium ion binding | Cytoplasm |
| PAK4 | NP_001014832.1 | 10298 | serine/threonine-protein kinase PAK 4 isoform 1 | 32.7 | 2 | 9 | 9 | 13 | 2.41E+08 | 4.81 | 2 | 45.4 | 591 | 64 | 9.7 | Cell growth and/or maintenance;Cell motility | Golgi membrane;Cytosol;Nucleus;Cytoplasm | Cytoskeletal protein binding | Golgi membrane;Nucleus;Cytoplasm |
| SCARB2 | NP_005497.1 | 950 | lysosome membrane protein 2 isoform 1 precursor | 23.4 | 2 | 7 | 7 | 15 | 1.75E+08 | 4.80 | 7 | 59.3 | 478 | 54.3 | 5.1 | Cell communication;Signal transduction | Plasma membrane | Receptor activity | Plasma membrane |
| UPP1 | NP_003355.1 | 7378 | uridine phosphorylase 1 isoform a | 53.2 | 2 | 15 | 15 | 28 | 1.58E+09 | 4.73 | 12 | 37.1 | 310 | 33.9 | 7.9 | Metabolism;Energy pathways;Pyrimidine salvage | Cytoplasm | Phosphorylase activity | Cytoplasm |
| KANK2 | NP_001129663.1 | 25959 | KN motif and ankyrin repeat domain-containing protein 2 isoform 2 | 5.5 | 2 | 3 | 3 | 4 | 1.28E+07 | 4.67 | 1 | - | 851 | 91.1 | 5.6 | Biological_process unknown | - | Structural molecule activity | - |
| TSC22D4 | NP_112197.1 | 81628 | TSC22 domain family protein 4 | 19.0 | 1 | 4 | 4 | 7 | 2.13E+08 | 4.65 | 3 | 103.5 | 395 | 41 | 7.2 | Regulation of nucleobase, nucleoside, nucleotide and nucleic acid metabolism | - | Transcription regulator activity | - |
| EPSTI1 | NP_150280.1 | 94240 | epithelial-stromal interaction protein 1 isoform 2 | 11.7 | 2 | 2 | 2 | 2 | 3.16E+07 | 4.63 | 1 | - | 307 | 35.4 | 9.9 | Biological_process unknown | - | Molecular function unknown | - |

| Gene Symbol | RefSeq_Accession | Entrez Gene ID | Description | Coverage (%) | Proteins | Unique Peptides | # Peptides | PSM | Area | SCC-R /SCC-S | SCC-R /SCC-S (Count) | SCC-R /SCC-S (Variability [%]) | # AAs | MW [kDa] | calc. pI | Biological Process | Localization | Molecular Function | Primary Localization |
|---|---|---|---|---|---|---|---|---|---|---|---|---|---|---|---|---|---|---|---|
| SAMHD1 | NP_056289.2 | 25939 | deoxynucleoside triphosphate triphosphohydrolase SAMHD1 | 52.4 | 1 | 26 | 26 | 70 | 5.60E+08 | 4.61 | 29 | 32.2 | 626 | 72.2 | 7.1 | Immune response | Nucleus;Cytoplasm | GTPase activity | Nucleus;Cytoplasm |
| UAP1 | NP_003106.3 | 6675 | UDP-N-acetylhexosamine pyrophosphorylase | 44.0 | 1 | 14 | 14 | 26 | 5.59E+08 | 4.59 | 11 | 63.4 | 505 | 57 | 6.4 | Metabolism;Energy pathways | Cytoplasm | Phosphorylase activity | Cytoplasm |
| DDX60L | NP_001012985.2 | 91351 | probable ATP-dependent RNA helicase DDX60-like isoform 1 | 10.1 | 2 | 9 | 9 | 12 | 2.56E+08 | 4.57 | 6 | 93.9 | 1706 | 197.5 | 8.3 | RNA metabolism | - | Helicase activity | - |
| NMI | NP_004679.2 | 9111 | N-myc-interactor | 31.9 | 1 | 7 | 7 | 11 | 2.09E+08 | 4.56 | 1 | - | 307 | 35 | 5.3 | Regulation of nucleobase, nucleoside, nucleotide and nucleic acid metabolism | Cytoplasm;Nucleus | Transcription factor activity | Cytoplasm |
| TBCB | NP_001272.2 | 1155 | tubulin-folding cofactor B | 58.2 | 1 | 13 | 13 | 38 | 4.42E+08 | 4.56 | 12 | 37.3 | 244 | 27.3 | 5.2 | Protein metabolism | Cytoplasm | Chaperone activity | Cytoplasm |
| KCNN4 | NP_002241.1 | 3783 | intermediate conductance calcium-activated potassium channel protein 4 | 8.4 | 1 | 2 | 2 | 2 | 2.00E+07 | 4.55 | 1 | - | 427 | 47.7 | 9.8 | Transport | Plasma membrane | Intracellular ligand-gated ion channel activity | Plasma membrane |
| ECE1 | NP_001106819.1 | 1889 | endothelin-converting enzyme 1 isoform 4 | 11.0 | 4 | 4 | 4 | 6 | 1.46E+08 | 4.54 | 1 | - | 754 | 85.5 | 5.8 | Protein metabolism | Plasma membrane;Golgi apparatus;Nucleus;Cytoplasm | Metallopeptidase activity | Plasma membrane |
| GGACT | NP_149101.1 | 87769 | gamma-glutamylaminecyclotransferase | 17.0 | 1 | 1 | 1 | 1 | 5.25E+07 | 4.46 | 1 | - | 153 | 17.3 | 6.9 | Biological_process unknown | - | Molecular function unknown | - |
| SDSL | NP_612441.1 | 113675 | serine dehydratase-like | 19.8 | 1 | 5 | 5 | 5 | 6.25E+07 | 4.45 | 3 | 171.9 | 329 | 34.7 | 6.9 | Biological_process unknown | - | Molecular function unknown | - |
| FAM3C | NP_055703.1 | 10447 | protein FAM3C precursor | 54.2 | 1 | 8 | 8 | 19 | 1.85E+08 | 4.45 | 4 | 50.5 | 227 | 24.7 | 8.3 | Cell communication;Signal transduction | Extracellular | Cytokine activity | Extracellular |
| PGPEP1 | NP_060182.1 | 54858 | pyroglutamyl-peptidase 1 | 7.7 | 1 | 1 | 1 | 1 | 3.01E+06 | 4.42 | 1 | - | 209 | 23.1 | 5.9 | Protein metabolism | Cytoplasm | Cysteine-type peptidase activity | Cytoplasm |
| LACTB2 | NP_057111.1 | 51110 | beta-lactamase-like protein 2 | 13.5 | 1 | 3 | 3 | 3 | 1.73E+07 | 4.36 | 1 | - | 288 | 32.8 | 6.8 | Biological_process unknown | - | Molecular function unknown | - |
| DDX60 | NP_060101.3 | 55601 | probable ATP-dependent RNA helicase DDX60 | 1.4 | 1 | 2 | 2 | 2 | 2.12E+07 | 4.36 | 2 | 85.8 | 1712 | 197.7 | 7.6 | Regulation of nucleobase, nucleoside, nucleotide and nucleic acid metabolism | - | DNA binding | - |
| BLVRB | NP_000704.1 | 645 | flavin reductase (NADPH) | 72.8 | 1 | 11 | 11 | 49 | 5.64E+08 | 4.32 | 11 | 23.9 | 206 | 22.1 | 7.7 | Metabolism;Energy pathways | Cytoplasm | Oxidoreductase activity | Cytoplasm |
| DST | NP_899236.1 | 667 | dystonin isoform 1 | 7.5 | 4 | 15 | 22 | 33 | 2.24E+09 | 4.29 | 6 | 39.5 | 5497 | 629.4 | 5.7 | Cell growth and/or maintenance | Nucleus;Plasma membrane;Extracellular;Cytoplasm | Cytoskeletal protein binding | Plasma membrane |
| CHKA | NP_997634.1 | 1119 | choline kinase alpha isoform b | 6.4 | 2 | 1 | 1 | 1 | 3.22E+07 | 4.27 | 1 | - | 439 | 50.1 | 6.7 | Metabolism;Energy pathways | - | Lipid kinase activity | - |
| CRIP2 | NP_001303.1 | 1397 | cysteine-rich protein 2 isoform 1 | 59.1 | 3 | 5 | 5 | 31 | 5.73E+08 | 4.27 | 11 | 19.2 | 208 | 22.5 | 8.7 | Regulation of nucleobase, nucleoside, nucleotide and nucleic acid metabolism | Cytoplasm;Nucleus | Receptor signaling complex scaffold activity | Nucleus |
| B2M | NP_004039.1 | 567 | beta-2-microglobulin precursor | 41.2 | 1 | 4 | 4 | 28 | 1.46E+09 | 4.26 | 8 | 12.6 | 119 | 13.7 | 6.5 | Immune response | Extracellular | MHC class I receptor activity;MHC class II receptor activity | Extracellular |
| RTN4 | NP_722550.1 | 57142 | reticulon-4 isoform B | 45.6 | 4 | 6 | 11 | 102 | 1.94E+09 | 4.26 | 12 | 56.8 | 373 | 40.3 | 4.8 | Regulation of cell growth | Endoplasmic reticulum membrane;Plasma membrane;Mitochondrion;Nucleus;Cytoplasm | Protein binding | Endoplasmic reticulum membrane;Nucleus;Cytoplasm |
| UBE2C | NP_008950.1 | 11065 | ubiquitin-conjugating enzyme E2 C isoform 1 | 24.6 | 5 | 2 | 2 | 2 | 4.37E+06 | 4.24 | 1 | - | 179 | 19.6 | 7.4 | Protein metabolism | Nucleus;Microtubule;Chromosome | Ubiquitin-specific protease activity | Nucleus |
| PPP1R18 | NP_597728.1 | 170954 | phostensin | 25.6 | 1 | 9 | 9 | 12 | 1.52E+08 | 4.22 | 3 | 16.2 | 613 | 67.9 | 5.4 | Biological_process unknown | Cell junction;Actin cytoskeleton | Molecular function unknown | Cell junction |
| HMGA1 | NP_665908.1 | 3159 | high mobility group protein HMG-I/HMG-Y isoform a | 38.3 | 1 | 4 | 6 | 87 | 5.01E+09 | 4.22 | 10 | 22.7 | 107 | 11.7 | 10.3 | Regulation of nucleobase, nucleoside, nucleotide and nucleic acid metabolism | Nucleus;Cytoplasm | DNA binding | Nucleus |
| CDV3 | NP_060018.1 | 55573 | protein CDV3 homolog isoform b | 56.2 | 6 | 6 | 6 | 51 | 8.86E+08 | 4.19 | 7 | 15.8 | 258 | 27.3 | 6.4 | Cell proliferation | Nucleus | Molecular function unknown | Nucleus |
| SEMA4B | NP_064595.2 | 10509 | semaphorin-4B precursor | 3.1 | 1 | 1 | 1 | 1 | 1.35E+08 | 4.18 | 1 | - | 837 | 92.7 | 7.1 | Biological_process unknown | Plasma membrane;Synapse;Cytoplasm | Molecular function unknown | Plasma membrane |
| HPCAL1 | NP_602293.1 | 3241 | hippocalcin-like protein 1 | 11.9 | 3 | 2 | 2 | 2 | 4.61E+07 | 4.17 | 1 | - | 193 | 22.3 | 5.4 | Cell communication;Signal transduction | Cytoplasm;Plasma membrane | Calcium ion binding | Cytoplasm |
| BIN3 | NP_061158.1 | 55909 | bridging integrator 3 | 19.8 | 1 | 3 | 3 | 4 | 7.17E+07 | 4.15 | 2 | 3.3 | 253 | 29.6 | 7.5 | Cell communication;Signal transduction | Mitochondrion;Golgi apparatus | Receptor signaling complex scaffold activity | Mitochondrion |
| ACAA2 | NP_006102.2 | 10449 | 3-ketoacyl-CoA thiolase, mitochondrial | 34.8 | 1 | 6 | 6 | 7 | 5.13E+07 | 4.15 | 2 | 38.9 | 397 | 41.9 | 8.1 | Metabolism;Energy pathways | Mitochondrion | Acyltransferase activity | Mitochondrion |
| LAP3 | NP_056991.2 | 51056 | cytosol aminopeptidase | 64.4 | 1 | 26 | 26 | 99 | 1.07E+09 | 4.07 | 28 | 39.9 | 519 | 56.1 | 7.9 | Protein metabolism | Cytoplasm | Aminopeptidase activity | Cytoplasm |
| PIP4K2B | NP_003550.1 | 8396 | phosphatidylinositol 5-phosphate 4-kinase type-2 beta | 16.4 | 2 | 4 | 4 | 6 | 6.37E+07 | 4.04 | 4 | 50.4 | 416 | 47.3 | 7.3 | Cell communication;Signal transduction | Plasma membrane;Nucleus;Cytosol;Endoplasmic reticulum | Lipid kinase activity | Plasma membrane |
| TRIOBP | NP_008963.3 | 11078 | TRIO and F-actin-binding protein isoform 1 | 18.3 | 3 | 9 | 9 | 16 | 9.22E+07 | 4.04 | 4 | 36.5 | 652 | 74 | 6.9 | Cell growth and/or maintenance | - | Cytoskeletal protein binding | - |
| TRIM22 | NP_001186502.1 | 10346 | E3 ubiquitin-protein ligase TRIM22 isoform 2 | 15.6 | 2 | 6 | 6 | 8 | 1.59E+08 | 4.02 | 3 | 4.8 | 494 | 56.4 | 7.7 | Regulation of nucleobase, nucleoside, nucleotide and nucleic acid metabolism | Cytoplasm | Transcription regulator activity | Cytoplasm |
| CISD1 | NP_060934.1 | 55847 | CDGSH iron-sulfur domain-containing protein 1 | 23.2 | 1 | 2 | 2 | 4 | 6.92E+07 | 4.02 | 2 | 10.8 | 108 | 12.2 | 9.1 | Biological_process unknown | - | Molecular function unknown | - |
| QKI | NP_996735.1 | 9444 | protein quaking isoform HQK-6 | 21.3 | 4 | 5 | 5 | 7 | 6.67E+07 | 3.99 | 2 | 21.6 | 319 | 35.1 | 7.4 | RNA metabolism | Cytoplasm | RNA binding | Cytoplasm |
| FOSL1 | NP_005429.1 | 8061 | fos-related antigen 1 | 31.7 | 1 | 4 | 4 | 8 | 1.51E+08 | 3.99 | 1 | - | 271 | 29.4 | 8.0 | Regulation of nucleobase, nucleoside, nucleotide and nucleic acid metabolism | Nucleus | Transcription factor activity | Nucleus |
| TOR4A | NP_060193.2 | 54863 | torsin-4A | 7.8 | 1 | 2 | 2 | 4 | 1.22E+08 | 3.99 | 2 | 44.4 | 423 | 46.9 | 9.9 | Metabolism;Energy pathways | Cytoplasm | ATPase activity | Cytoplasm |
| HAUS7 | NP_059988.3 | 55559 | HAUS augmin-like complex subunit 7 | 4.4 | 1 | 1 | 1 | 3 | 2.74E+07 | 3.97 | 1 | - | 368 | 40.8 | 4.8 | Biological_process unknown | Centrosome;Microtubule | Molecular function unknown | - |
| GBP1 | NP_002044.2 | 2633 | interferon-induced guanylate-binding protein 1 | 30.9 | 2 | 10 | 13 | 34 | 1.99E+08 | 3.95 | 7 | 13.9 | 592 | 67.9 | 6.3 | Cell communication;Signal transduction | Cytoplasm;Extracellular | GTPase activity | Cytoplasm |

Jain *et al.*, 2019. MAP2K1 is a potential therapeutic target in erlotinib resistant head and neck squamous cell carcinoma
Supplementary Table S4. List of all proteins identified in SCC-S and SCC-R cells using SequestHT and Mascot search algorithms and their gene ontology annotation

| Gene Symbol | RefSeq_Accession | Entrez Gene ID | Description | Coverage (%) | Proteins | Unique Peptides | # Peptides | PSM | Area | SCC-R /SCC-S | SCC-R /SCC-S (Count) | SCC-R /SCC-S (Variability [%]) | # AAs | MW [kDa] | calc. pI | Biological Process | Localization | Molecular Function | Primary Localization |
|---|---|---|---|---|---|---|---|---|---|---|---|---|---|---|---|---|---|---|---|
| TMSB4X | NP_066932.1 | 7114 | thymosin beta-4 | 93.2 | 1 | 9 | 9 | 49 | 4.85E+09 | 3.92 | 11 | 11 | 44 | 5 | 5.1 | Cell growth and/or maintenance | Cytoplasm | Cytoskeletal protein binding | Cytoplasm |
| HMGA1 | NP_665910.1 | 3159 | high mobility group protein HMG-I/HMG-Y isoform b | 31.3 | 1 | 1 | 3 | 11 | 3.69E+08 | 3.91 | 2 | 4.9 | 96 | 10.7 | 10.3 | Regulation of nucleobase, nucleoside, nucleotide and nucleic acid metabolism | Nucleus;Cytoplasm | DNA binding | Nucleus |
| LAG3 | NP_002277.4 | 3902 | lymphocyte activation gene 3 protein precursor | 1.1 | 1 | 1 | 1 | 1 | 1.82E+07 | 3.87 | 1 | - | 525 | 57.4 | 8.0 | Immune response | Plasma membrane | Molecular function unknown | Plasma membrane |
| ECH1 | NP_001389.2 | 1891 | delta(3,5)-Delta(2,4)-dienoyl-CoA isomerase, mitochondrial precursor | 54.3 | 1 | 12 | 12 | 52 | 5.87E+08 | 3.86 | 13 | 10 | 328 | 35.8 | 8.0 | Metabolism;Energy pathways | Peroxisome;Mitochondrion;Cytoplasm | Catalytic activity | Peroxisome |
| CD59 | NP_001120697.1 | 966 | CD59 glycoprotein preproprotein | 18.8 | 1 | 2 | 2 | 14 | 7.20E+08 | 3.86 | 4 | 63.4 | 128 | 14.2 | 6.5 | Immune response | Extracellular;Plasma membrane;Cytoplasm | Receptor activity | Plasma membrane |
| SP110 | NP_004501.3 | 3431 | sp110 nuclear body protein isoform b | 11.7 | 4 | 5 | 5 | 7 | 9.85E+06 | 3.84 | 3 | 13.3 | 549 | 61.8 | 9.6 | Regulation of gene expression, epigenetic | Nucleus;Nucleolus | Transcription regulator activity | Nucleus |
| LGALS3BP | NP_005558.1 | 3959 | galectin-3-binding protein precursor | 23.1 | 1 | 9 | 9 | 32 | 7.02E+08 | 3.84 | 13 | 71.2 | 585 | 65.3 | 5.3 | Immune response | Extracellular | Extracellular matrix structural constituent | Extracellular |
| RRAGA | NP_006561.1 | 10670 | ras-related GTP-binding protein A | 8.0 | 1 | 1 | 2 | 4 | 1.11E+08 | 3.83 | 1 | - | 313 | 36.5 | 7.7 | Cell communication;Signal transduction | Cytoplasm;Nucleus;Plasma membrane | Molecular function unknown | Cytoplasm |
| IGF2BP2 | NP_001007226.1 | 10644 | insulin-like growth factor 2 mRNA-binding protein 2 isoform b | 37.4 | 7 | 13 | 15 | 79 | 1.45E+09 | 3.80 | 20 | 13.2 | 556 | 61.8 | 8.3 | Regulation of nucleobase, nucleoside, nucleotide and nucleic acid metabolism;Regulation of translation | Cytoplasm;Cytoskeleton | RNA binding;Translation regulator activity | Cytoplasm |
| PARP10 | NP_116178.2 | 84875 | poly [ADP-ribose] polymerase 10 | 9.2 | 1 | 4 | 4 | 4 | 3.98E+06 | 3.76 | 1 | - | 1025 | 109.9 | 5.0 | Regulation of nucleobase, nucleoside, nucleotide and nucleic acid metabolism | - | Molecular function unknown | - |
| PTPN12 | NP_002826.3 | 5782 | tyrosine-protein phosphatase non-receptor type 12 isoform 1 | 17.3 | 3 | 8 | 8 | 12 | 1.65E+08 | 3.75 | 3 | 23.8 | 780 | 88.1 | 5.6 | Cell communication;Signal transduction | Cytoplasm;Nucleus | Protein tyrosine phosphatase activity | Cytoplasm |
| AFAP1 | NP_940997.1 | 60312 | actin filament-associated protein 1 isoform B | 2.5 | 2 | 1 | 1 | 1 | 1.51E+07 | 3.74 | 1 | - | 730 | 80.7 | 8.7 | Cell growth and/or maintenance | Cytoplasm;Plasma membrane | Receptor signaling complex scaffold activity;Cytoskeletal anchoring activity | Cytoplasm |
| TNS3 | NP_073585.8 | 64759 | tensin-3 | 26.6 | 5 | 18 | 18 | 30 | 6.69E+08 | 3.73 | 11 | 30.1 | 1445 | 155.2 | 6.8 | Cell communication;Signal transduction;Biological_process unknown | Integral to membrane | Receptor activity;Molecular function unknown | Integral to membrane |
| CC2D1B | NP_115825.1 | 200014 | coiled-coil and C2 domain-containing protein 1B | 3.9 | 1 | 2 | 2 | 4 | 1.11E+07 | 3.70 | 3 | 8.3 | 858 | 94.2 | 5.3 | Cell communication;Signal transduction | - | Molecular function unknown | - |
| C11orf83 | NP_001078841.1 | 790955 | UPF0723 protein C11orf83 precursor | 24.7 | 1 | 2 | 2 | 2 | 6.30E+06 | 3.70 | 1 | - | 93 | 10.1 | 9.4 | Biological_process unknown | - | Molecular function unknown | - |
| GMFB | NP_004115.1 | 2764 | glia maturation factor beta | 31.0 | 1 | 2 | 2 | 9 | 1.12E+08 | 3.68 | 3 | 51 | 142 | 16.7 | 5.3 | Cell differentiation;Cell proliferation | Cytoplasm;Plasma membrane | Growth factor activity | Cytoplasm |
| UBE2L6 | NP_004214.1 | 9246 | ubiquitin/ISG15-conjugating enzyme E2 L6 isoform 1 | 90.2 | 2 | 14 | 14 | 125 | 1.56E+09 | 3.68 | 24 | 29.2 | 153 | 17.8 | 7.9 | Protein metabolism | - | Protein binding | - |
| TLE1 | NP_005068.2 | 7088 | transducin-like enhancer protein 1 | 3.8 | 1 | 2 | 2 | 2 | 2.24E+07 | 3.68 | 1 | - | 770 | 83.1 | 7.2 | Regulation of nucleobase, nucleoside, nucleotide and nucleic acid metabolism | Nucleus | Transcription regulator activity | Nucleus |
| LAMP2 | NP_002285.1 | 3920 | lysosome-associated membrane glycoprotein 2 isoform A precursor | 5.1 | 3 | 2 | 2 | 3 | 1.16E+09 | 3.68 | 1 | - | 410 | 44.9 | 5.6 | Cell growth and/or maintenance | Extracellular;Endoplasmic reticulum;Endosome;Lysosome;Plasma membrane;Cytoplasmic vesicle;Golgi | Cell adhesion molecule activity | Lysosome |
| GANAB | NP_938149.2 | 23193 | neutral alpha-glucosidase AB isoform 3 precursor | 56.2 | 2 | 1 | 37 | 218 | 1.36E+09 | 3.67 | 2 | 61 | 966 | 109.4 | 6.2 | Carbohydrate metabolism | Endoplasmic reticulum;Centrosome | Hydrolase activity | Endoplasmic reticulum |
| UBE2E1 | NP_001189405.1 | 7324 | ubiquitin-conjugating enzyme E2 E1 isoform 3 | 16.3 | 2 | 2 | 2 | 5 | 2.92E+07 | 3.66 | 1 | - | 160 | 18 | 8.8 | Protein metabolism | Nucleus;Cytoplasm | Ubiquitin-specific protease activity | Nucleus |
| PAFAH1B3 | NP_001139411.1 | 5050 | platelet-activating factor acetylhydrolase IB subunit gamma | 62.8 | 1 | 8 | 8 | 31 | 1.57E+09 | 3.65 | 9 | 36.4 | 231 | 25.7 | 6.8 | Metabolism;Energy pathways | Cytoplasm | Acyltransferase activity | Cytoplasm |
| NDFIP1 | NP_085048.1 | 80762 | NEDD4 family-interacting protein 1 | 7.7 | 1 | 1 | 1 | 4 | 7.41E+07 | 3.65 | 2 | 49.7 | 221 | 24.9 | 4.7 | Cell communication;Signal transduction | Golgi apparatus | Receptor signaling complex scaffold activity | Golgi apparatus |
| FAM50A | NP_004690.1 | 9130 | protein FAM50A | 24.5 | 2 | 8 | 8 | 19 | 9.65E+08 | 3.63 | 7 | 17.2 | 339 | 40.2 | 6.8 | Regulation of nucleobase, nucleoside, nucleotide and nucleic acid metabolism | Nucleus | DNA binding | Nucleus |
| CDC42EP3 | NP_001257365.1 | 10602 | cdc42 effector protein 3 | 10.2 | 1 | 1 | 1 | 1 | 1.80E+08 | 3.62 | 1 | - | 254 | 27.7 | 5.8 | Cell communication;Signal transduction | Cytoplasm | GTPase activity | Cytoplasm |
| RPS27L | NP_057004.1 | 51065 | 40S ribosomal protein S27-like | 40.5 | 1 | 1 | 3 | 22 | 6.94E+08 | 3.62 | 1 | - | 84 | 9.5 | 9.5 | Biological_process unknown | Ribosome | Molecular function unknown | Ribosome |
| RPP25L | NP_680544.1 | 138716 | ribonuclease P protein subunit p25-like protein | 14.1 | 1 | 1 | 1 | 1 | 7.35E+07 | 3.61 | 1 | - | 163 | 17.6 | 10.3 | Biological_process unknown | - | Molecular function unknown | - |
| ARHGEF1 | NP_945328.1 | 9138 | rho guanine nucleotide exchange factor 1 isoform 3 | 36.1 | 3 | 19 | 19 | 46 | 2.89E+08 | 3.57 | 12 | 34.8 | 879 | 98.7 | 5.6 | Cell communication;Signal transduction | Plasma membrane;Cytoplasm | Guanyl-nucleotide exchange factor activity | Cytoplasm |
| CALU | NP_001210.1 | 813 | calumenin isoform a precursor | 59.7 | 5 | 17 | 17 | 71 | 1.49E+09 | 3.57 | 20 | 19.8 | 315 | 37.1 | 4.6 | Cell communication;Signal transduction | Endoplasmic reticulum;Golgi apparatus;Extracellular;Mitochondrion;Nucleus | Calcium ion binding | Endoplasmic reticulum |
| UACA | NP_060473.2 | 55075 | uveal autoantigen with coiled-coil domains and ankyrin repeats isoform 1 | 15.6 | 2 | 13 | 13 | 19 | 1.50E+08 | 3.54 | 9 | 36 | 1416 | 162.4 | 7.0 | Biological_process unknown | Nucleus;Cytoplasm | Molecular function unknown | Nucleus |
| CLCN7 | NP_001278.1 | 1186 | H(+)/Cl(-) exchange transporter 7 isoform a | 8.6 | 2 | 3 | 3 | 3 | 1.41E+08 | 3.51 | 2 | 28.5 | 805 | 88.6 | 8.4 | Transport | Plasma membrane;Endosome;Lysosome | Voltage-gated ion channel activity | Plasma membrane |
| NAMPT | NP_005737.1 | 10135 | nicotinamide phosphoribosyltransferase precursor | 64.2 | 1 | 24 | 24 | 108 | 8.11E+08 | 3.51 | 36 | 21.4 | 491 | 55.5 | 7.2 | Anti-apoptosis | Extracellular;Cytoplasm;Nucleus | Transferase activity | Extracellular |
| IGF2BP3 | NP_006538.2 | 10643 | insulin-like growth factor 2 mRNA-binding protein 3 | 19.3 | 1 | 5 | 7 | 38 | 1.02E+09 | 3.51 | 3 | 3.6 | 579 | 63.7 | 8.9 | Protein metabolism | Cytoplasm;Nucleus | Translation regulator activity | Cytoplasm |
| UNC93B1 | NP_112192.2 | 81622 | protein unc-93 homolog B1 | 7.7 | 1 | 3 | 3 | 7 | 1.95E+07 | 3.50 | 4 | 68.4 | 597 | 66.6 | 7.0 | Transport | Integral to membrane | Transporter activity | Integral to membrane |
| NCEH1 | NP_065843.3 | 57552 | neutral cholesterol ester hydrolase 1 isoform b | 28.2 | 3 | 8 | 8 | 28 | 1.75E+08 | 3.49 | 12 | 24.6 | 440 | 49 | 7.9 | Biological_process unknown | - | Molecular function unknown | - |
| PPP1R12C | NP_001258547.1 | 54776 | protein phosphatase 1 regulatory subunit 12C isoform b | 7.2 | 2 | 2 | 2 | 2 | 2.00E+08 | 3.48 | 1 | - | 780 | 84.6 | 5.6 | Metabolism;Energy pathways | Cytoplasm | Protein binding | Cytoplasm |
| DTX3L | NP_612144.1 | 151636 | E3 ubiquitin-protein ligase DTX3L | 22.2 | 1 | 9 | 9 | 16 | 1.97E+08 | 3.46 | 8 | 36.9 | 740 | 83.5 | 8.1 | Protein metabolism | Nucleus | Ubiquitin-specific protease activity | Nucleus |

| Gene Symbol | RefSeq_Accession | Entrez Gene ID | Description | Coverage (%) | Proteins | Unique Peptides | # Peptides | PSM | Area | SCC-R /SCC-S | SCC-R /SCC-S (Count) | SCC-R /SCC-S (Variability [%]) | # AAs | MW [kDa] | calc. pI | Biological Process | Localization | Molecular Function | Primary Localization |
|---|---|---|---|---|---|---|---|---|---|---|---|---|---|---|---|---|---|---|---|
| **TXNRD1** | NP_001248374.1 | 7296 | **thioredoxin reductase 1, cytoplasmic isoform 4** | 64.7 | 3 | 1 | 24 | 139 | 1.93E+09 | **3.46** | 4 | 3.5 | 549 | 60.2 | 6.7 | Metabolism;Energy pathways | Cytoplasm;Extracellular;Plasma membrane;Nucleus | Oxidoreductase activity | Cytoplasm |
| **SYPL1** | NP_874384.1 | 6856 | **synaptophysin-like protein 1 isoform b** | 21.2 | 2 | 3 | 3 | 9 | 5.80E+08 | **3.46** | 1 | - | 241 | 26.4 | 7.2 | Transport | Microsome;Plasma membrane;Cytoplasmic vesicle;Endoplasmic reticulum;Golgi vesicle;Cytoplasm;Golgi apparatus;Endosome | Transporter activity | Cytoplasmic vesicle |
| **CAPN2** | NP_001739.2 | 824 | **calpain-2 catalytic subunit isoform 1** | 63.7 | 2 | 34 | 34 | 131 | 1.12E+09 | **3.46** | 36 | 23.3 | 700 | 80 | 5.0 | Protein metabolism | Cytoplasm;Plasma membrane;Membrane fraction | Cysteine-type peptidase activity | Cytoplasm |
| **TMEM164** | NP_115603.2 | 84187 | **transmembrane protein 164 isoform b** | 6.1 | 1 | 1 | 1 | 1 | 1.95E+07 | **3.44** | 1 | - | 297 | 33.5 | 8.2 | Biological_process unknown | - | Molecular function unknown | - |
| **SYNJ2** | NP_001171559.1 | 8871 | **synaptojanin-2 isoform 2** | 4.1 | 2 | 3 | 3 | 3 | 3.35E+07 | **3.43** | 2 | 52.2 | 1259 | 139.2 | 7.5 | Metabolism;Energy pathways | Cytoplasm;Plasma membrane;Mitochondrion | Lipid phosphatase activity | Cytoplasm |
| **IFI35** | NP_005524.2 | 3430 | **interferon-induced 35 kDa protein** | 37.9 | 1 | 8 | 8 | 34 | 3.93E+08 | **3.43** | 9 | 40.7 | 288 | 31.8 | 6.1 | Immune response | Cytoplasm;Nucleus | Molecular function unknown | Cytoplasm |
| **MYL6B** | NP_001186558.1 | 140465 | **myosin light chain 6B** | 24.0 | 1 | 2 | 4 | 54 | 3.11E+09 | **3.42** | 1 | - | 208 | 22.7 | 5.7 | Cell growth and/or maintenance | Cytoplasm;Nucleus | Structural molecule activity | Cytoplasm |
| **MFI2** | NP_005920.2 | 4241 | **melanotransferrin isoform 1 precursor** | 6.0 | 2 | 3 | 3 | 6 | 1.59E+08 | **3.42** | 3 | 32.3 | 738 | 80.2 | 5.9 | Immune response | Plasma membrane;Extracellular | Molecular function unknown | Plasma membrane |
| **TRIM21** | NP_003132.2 | 6737 | **E3 ubiquitin-protein ligase TRIM21** | 11.0 | 1 | 4 | 4 | 7 | 3.56E+07 | **3.41** | 3 | 36 | 475 | 54.1 | 6.4 | Regulation of nucleobase, nucleoside, nucleotide and nucleic acid metabolism | Plasma membrane;Nucleus;Cytoplasm | Ribonucleoprotein | Plasma membrane |
| **HLA-C** | NP_001229971.1 | 3107 | **HLA class I histocompatibility antigen, Cw-1 alpha chain precursor** | 45.4 | 4 | 2 | 15 | 61 | 1.16E+09 | **3.41** | 1 | - | 366 | 40.7 | 5.9 | Immune response | Plasma membrane | MHC class I receptor activity;MHC class II receptor activity | Plasma membrane |
| **XYLT2** | NP_071450.2 | 64132 | **xylosyltransferase 2** | 1.5 | 1 | 1 | 1 | 1 | 1.43E+07 | **3.40** | 1 | - | 865 | 96.7 | 8.2 | Metabolism;Energy pathways | Extracellular;Endoplasmic reticulum;Golgi apparatus;Plasma membrane | Transferase activity | Extracellular |
| **CALCOCO1** | NP_001137154.1 | 57658 | **calcium-binding and coiled-coil domain-containing protein 1 isoform 2** | 4.1 | 2 | 2 | 2 | 2 | 3.31E+07 | **3.39** | 1 | - | 606 | 67.5 | 4.7 | Regulation of nucleobase, nucleoside, nucleotide and nucleic acid metabolism | Cytoplasm;Nucleus | Transcription regulator activity | Cytoplasm |
| **RAP2B** | NP_002877.2 | 5912 | **ras-related protein Rap-2b** | 50.3 | 2 | 5 | 8 | 21 | 2.75E+08 | **3.39** | 4 | 26.3 | 183 | 20.5 | 4.8 | Cell communication;Signal transduction | Plasma membrane | GTPase activity | Plasma membrane |
| **NECAP2** | NP_001138750.1 | 55707 | **adaptin ear-binding coat-associated protein 2 isoform 3** | 8.0 | 3 | 1 | 1 | 3 | 3.05E+08 | **3.38** | 2 | 20 | 237 | 25.3 | 6.2 | Biological_process unknown | - | Molecular function unknown | - |
| **TBC1D13** | NP_001273701.1 | 54662 | **TBC1 domain family member 13 isoform b** | 12.0 | 2 | 2 | 2 | 4 | 1.09E+08 | **3.37** | 3 | 6.3 | 275 | 32.2 | 5.6 | Cell communication;Signal transduction | - | GTPase activator activity | - |
| **FAM129B** | NP_001030611.1 | 64855 | **niban-like protein 1 isoform 2** | 57.0 | 2 | 26 | 26 | 120 | 1.56E+09 | **3.36** | 29 | 37.9 | 733 | 82.6 | 6.2 | Biological_process unknown | Nucleus | Molecular function unknown | Nucleus |
| **WHSC1** | NP_579877.1 | 7468 | **histone-lysine N-methyltransferase NSD2 isoform 1** | 5.2 | 3 | 4 | 4 | 5 | 1.82E+07 | **3.35** | 3 | 51.1 | 1365 | 152.2 | 8.7 | Regulation of gene expression, epigenetic | Nucleus;Cytoplasm;Nucleolus | DNA binding | Nucleus |
| **SLC4A2** | NP_001186623.1 | 6522 | **anion exchange protein 2 isoform 3** | 2.5 | 3 | 1 | 1 | 3 | 3.21E+08 | **3.34** | 2 | 7.6 | 1227 | 135.5 | 6.2 | Transport | Plasma membrane;Golgi apparatus;Nucleus;Cytoplasm | Auxiliary transport protein activity | Plasma membrane |
| **PARP14** | NP_060024.2 | 54625 | **poly [ADP-ribose] polymerase 14** | 5.4 | 1 | 6 | 6 | 10 | 7.88E+07 | **3.31** | 4 | 42 | 1801 | 202.7 | 7.2 | Biological_process unknown | - | Molecular function unknown | - |
| **CLIP2** | NP_115797.1 | 7461 | **CAP-Gly domain-containing linker protein 2 isoform 2** | 13.3 | 2 | 7 | 9 | 9 | 4.65E+07 | **3.30** | 1 | - | 1011 | 111.7 | 6.8 | Cell communication;Signal transduction | Cytoplasm | Structural constituent of cytoskeleton | Cytoplasm |
| **CTSZ** | NP_001327.2 | 1522 | **cathepsin Z preproprotein** | 13.9 | 1 | 3 | 3 | 3 | 9.38E+07 | **3.29** | 3 | 3.6 | 303 | 33.8 | 7.1 | Protein metabolism | Lysosome | Cysteine-type peptidase activity | Lysosome |
| **PDLIM7** | NP_005442.2 | 9260 | **PDZ and LIM domain protein 7 isoform 1** | 47.1 | 3 | 11 | 11 | 28 | 4.22E+08 | **3.28** | 10 | 52.6 | 457 | 49.8 | 8.4 | Cell communication;Signal transduction | Cytoskeleton;Nucleolus | Receptor signaling complex scaffold activity | Cytoskeleton |
| **C15orf38** | NP_872422.1 | 348110 | **arpin isoform 1** | 12.0 | 2 | 1 | 1 | 4 | 4.96E+07 | **3.27** | 1 | - | 226 | 24.9 | 5.8 | Biological_process unknown | - | Molecular function unknown | - |
| **TUBB3** | NP_006077.2 | 10381 | **tubulin beta-3 chain isoform 1** | 79.3 | 2 | 10 | 31 | 633 | 1.40E+10 | **3.26** | 7 | 18.5 | 450 | 50.4 | 4.9 | Cell growth and/or maintenance | Cytoplasm | Structural molecule activity | Cytoplasm |
| **SLC39A14** | NP_001128626.1 | 23516 | **zinc transporter ZIP14 isoform c precursor** | 10.0 | 3 | 2 | 2 | 3 | 4.06E+08 | **3.24** | 3 | 55.7 | 481 | 52.8 | 5.8 | Transport | Plasma membrane | Transporter activity | Plasma membrane |
| **SH3BGRL3** | NP_112576.1 | 83442 | **SH3 domain-binding glutamic acid-rich-like protein 3** | 66.7 | 1 | 3 | 3 | 20 | 1.91E+08 | **3.24** | 1 | - | 93 | 10.4 | 4.9 | Biological_process unknown | Cytoplasm;Nucleus;Plasma membrane | Molecular function unknown | Cytoplasm |
| **SDPR** | NP_004648.1 | 8436 | **serum deprivation-response protein** | 30.1 | 1 | 8 | 8 | 18 | 2.67E+08 | **3.22** | 5 | 11.5 | 425 | 47.1 | 5.2 | Cell growth and/or maintenance | Cytoplasm;Golgi apparatus;Plasma membrane | Serine-type peptidase activity | Cytoplasm |
| **NELFB** | NP_056271.2 | 25920 | **negative elongation factor B** | 3.3 | 1 | 1 | 1 | 4 | 2.46E+07 | **3.21** | 1 | - | 580 | 65.7 | 6.1 | Regulation of nucleobase, nucleoside, nucleotide and nucleic acid metabolism | Nucleus;Cytoplasm | Transcription regulator activity | Nucleus |
| **RNF213** | NP_001243000.2 | 57674 | **E3 ubiquitin-protein ligase RNF213 isoform 3** | 9.6 | 2 | 30 | 30 | 42 | 1.30E+08 | **3.20** | 18 | 36.9 | 5207 | 591 | 6.5 | Biological_process unknown | - | Molecular function unknown | - |
| **TMEM165** | NP_060945.2 | 55858 | **transmembrane protein 165 precursor** | 20.4 | 1 | 4 | 4 | 14 | 3.19E+08 | **3.18** | 3 | 16.3 | 324 | 34.9 | 7.0 | Biological_process unknown | - | Molecular function unknown | - |
| **RAI14** | NP_001138994.1 | 26064 | **ankycorbin isoform b** | 4.5 | 4 | 3 | 3 | 3 | 3.85E+07 | **3.18** | 3 | 3.9 | 951 | 106.8 | 6.0 | Biological_process unknown | Cytoplasm;Nucleus | Molecular function unknown | Cytoplasm |
| **CCDC82** | NP_079001.2 | 79780 | **coiled-coil domain-containing protein 82** | 13.1 | 1 | 4 | 4 | 4 | 5.71E+07 | **3.16** | 3 | 12.2 | 544 | 64 | 5.0 | Biological_process unknown | Cytoplasm | Molecular function unknown | Cytoplasm |
| **RHOC** | NP_001036144.1 | 389 | **rho-related GTP-binding protein RhoC precursor** | 61.1 | 1 | 2 | 8 | 41 | 1.20E+09 | **3.12** | 2 | 28.8 | 193 | 22 | 6.6 | Cell communication;Signal transduction | Cytoplasm;Plasma membrane | GTPase activity | Cytoplasm |
| **ITGB1** | NP_596867.1 | 3688 | **integrin beta-1 isoform 1A precursor** | 32.6 | 2 | 21 | 21 | 95 | 9.47E+08 | **3.12** | 32 | 24.2 | 798 | 88.4 | 5.4 | Cell communication;Immune cell migration | Plasma membrane;Cell surface | Receptor activity | Plasma membrane |
| **IRF2BP2** | NP_001070865.1 | 359948 | **interferon regulatory factor 2-binding protein 2 isoform B** | 46.2 | 2 | 10 | 10 | 20 | 2.51E+08 | **3.11** | 8 | 23.9 | 571 | 59.4 | 8.6 | Regulation of nucleobase, nucleoside, nucleotide and nucleic acid metabolism | Nucleus;Cytoplasm | Transcription regulator activity | Nucleus |
| **SH3GLB1** | NP_057093.1 | 51100 | **endophilin-B1 isoform 1** | 40.3 | 4 | 10 | 10 | 22 | 2.90E+08 | **3.11** | 8 | 37.9 | 365 | 40.8 | 6.0 | Metabolism;Energy pathways | Cytoplasm;Mitochondrion;Microsome | Acyltransferase activity | Cytoplasm |
| **FTH1** | NP_002023.2 | 2495 | **ferritin heavy chain** | 27.9 | 1 | 4 | 4 | 4 | 5.12E+07 | **3.10** | 1 | - | 183 | 21.2 | 5.6 | Transport | Cytoplasm | Storage protein | Cytoplasm |

| Gene Symbol | RefSeq_Accession | Entrez Gene ID | Description | Coverage (%) | Proteins | Unique Peptides | # Peptides | PSM | Area | SCC-R /SCC-S | SCC-R /SCC-S (Count) | SCC-R /SCC-S (Variability [%]) | # AAs | MW [kDa] | calc. pI | Biological Process | Localization | Molecular Function | Primary Localization |
|---|---|---|---|---|---|---|---|---|---|---|---|---|---|---|---|---|---|---|---|
| TNFRSF1A | NP_001056.1 | 7132 | tumor necrosis factor receptor superfamily member 1A precursor | 6.2 | 1 | 1 | 1 | 2 | 2.18E+07 | 3.10 | 1 | - | 455 | 50.5 | 6.6 | Cell communication;Signal transduction | Plasma membrane;Golgi apparatus;Endoplasmic reticulum | Receptor activity | Plasma membrane |
| COX17 | NP_005685.1 | 10063 | cytochrome c oxidase copper chaperone | 84.1 | 1 | 2 | 2 | 4 | 9.32E+07 | 3.10 | 3 | 17.1 | 63 | 6.9 | 7.2 | Metabolism;Energy pathways | Mitochondrion;Cytoplasm | Chaperone activity | Cytoplasm |
| VAT1 | NP_006364.2 | 10493 | synaptic vesicle membrane protein VAT-1 homolog | 58.0 | 1 | 14 | 14 | 55 | 9.70E+08 | 3.10 | 15 | 20.2 | 393 | 41.9 | 6.3 | Transport | Cytoplasmic vesicle | Transporter activity | Cytoplasmic vesicle |
| ERAP1 | NP_001035548.1 | 51752 | endoplasmic reticulum aminopeptidase 1 isoform b precursor | 7.6 | 2 | 4 | 4 | 10 | 4.66E+07 | 3.09 | 2 | 107.1 | 941 | 107.2 | 6.5 | Protein metabolism | Endoplasmic reticulum;Cytoplasm;Mitochondrion | Aminopeptidase activity | Endoplasmic reticulum |
| CPOX | NP_000088.3 | 1371 | oxygen-dependent coproporphyrinogen-III oxidase, mitochondrial precursor | 40.5 | 1 | 15 | 15 | 46 | 7.72E+08 | 3.09 | 16 | 10.4 | 454 | 50.1 | 8.3 | Metabolism;Energy pathways | Mitochondrion | Catalytic activity | Mitochondrion |
| ATG7 | NP_001138384.1 | 10533 | ubiquitin-like modifier-activating enzyme ATG7 isoform c | 24.9 | 3 | 11 | 11 | 23 | 1.77E+08 | 3.05 | 11 | 45.7 | 623 | 68.6 | 6.5 | Cell growth and/or maintenance | Cytoplasm | Molecular function unknown | Cytoplasm |
| CD47 | NP_942088.1 | 961 | leukocyte surface antigen CD47 isoform 2 precursor | 8.2 | 2 | 3 | 3 | 3 | 1.46E+08 | 3.04 | 2 | 41.6 | 305 | 33.2 | 7.9 | Immune response | Plasma membrane;Cell surface | Molecular function unknown | Plasma membrane;Cell surface |
| GBA | NP_001165282.1 | 2629 | glucosylceramidase isoform 2 | 32.3 | 3 | 10 | 10 | 18 | 2.33E+08 | 3.04 | 7 | 16.8 | 449 | 50.3 | 7.2 | Lipid metabolism | Lysosome;Endosome;Vesicle | Glucosidase activity | Lysosome |
| AKAP12 | NP_005091.2 | 9590 | A-kinase anchor protein 12 isoform 1 | 38.9 | 2 | 32 | 32 | 82 | 7.28E+08 | 3.04 | 32 | 29.4 | 1782 | 191.4 | 4.4 | Cell growth and/or maintenance | Plasma membrane;Cytoplasm;Nucleus | Cytoskeletal anchoring activity | Cytoplasm;Nucleus |
| VAC14 | NP_060522.3 | 55697 | protein VAC14 homolog | 15.5 | 1 | 8 | 8 | 18 | 1.05E+08 | 3.03 | 6 | 37.5 | 782 | 87.9 | 6.1 | Biological_process unknown | Microsome;Centrosome | Molecular function unknown | Microsome;Centrosome |
| DBN1 | NP_004386.2 | 1627 | drebrin isoform a | 34.4 | 2 | 13 | 13 | 33 | 3.64E+08 | 3.02 | 12 | 19.3 | 649 | 71.4 | 4.5 | Cell growth and/or maintenance | Cytoplasm | Cytoskeletal protein binding | Cytoplasm |
| DSCR3 | NP_006043.1 | 10311 | Down syndrome critical region protein 3 | 9.8 | 1 | 2 | 2 | 3 | 6.46E+06 | 3.01 | 1 | - | 297 | 33 | 7.7 | Biological_process unknown | - | Molecular function unknown | - |
| STMN1 | NP_981946.1 | 3925 | stathmin isoform a | 63.8 | 10 | 13 | 13 | 51 | 7.41E+08 | 3.01 | 18 | 15.4 | 149 | 17.3 | 6.0 | Cell growth and/or maintenance;Signal transduction | Cytoplasm | Structural molecule activity;Signal transducer activity | Cytoplasm |
| LPIN3 | NP_075047.1 | 64900 | phosphatidate phosphatase LPIN3 | 1.9 | 1 | 1 | 1 | 2 | 1.84E+07 | 3.00 | 2 | 21.4 | 851 | 93.6 | 5.5 | Biological_process unknown | Nucleus | Molecular function unknown | Nucleus |
| GGA2 | NP_055859.1 | 23062 | ADP-ribosylation factor-binding protein GGA2 | 18.6 | 1 | 4 | 4 | 7 | 1.61E+08 | 3.00 | 2 | 27.8 | 613 | 67.1 | 6.6 | Transport | Golgi apparatus | Transporter activity | Golgi apparatus |
| SLC2A6 | NP_001138571.1 | 11182 | solute carrier family 2, facilitated glucose transporter member 6 isoform 2 | 8.3 | 2 | 2 | 2 | 3 | 7.80E+07 | 2.98 | 1 | - | 445 | 48 | 9.0 | Transport | Plasma membrane | Auxiliary transport protein activity | Plasma membrane |
| PLOD1 | NP_000293.2 | 5351 | procollagen-lysine,2-oxoglutarate 5-dioxygenase 1 precursor | 10.3 | 1 | 5 | 5 | 8 | 1.31E+08 | 2.97 | 2 | 61.5 | 727 | 83.5 | 7.0 | Metabolism;Energy pathways | Endoplasmic reticulum | Catalytic activity | Endoplasmic reticulum |
| CALCOCO2 | NP_001248324.1 | 10241 | calcium-binding and coiled-coil domain-containing protein 2 isoform 5 | 2.7 | 5 | 1 | 1 | 1 | 1.04E+07 | 2.96 | 1 | - | 374 | 43.6 | 4.8 | Biological_process unknown | Nucleus;Cytoplasm | Molecular function unknown | Nucleus |
| TACC1 | NP_006274.2 | 6867 | transforming acidic coiled-coil-containing protein 1 isoform 1 | 5.3 | 2 | 2 | 2 | 3 | 3.98E+07 | 2.96 | 2 | 21.7 | 805 | 87.7 | 4.9 | Cell communication;Signal transduction | Nucleus;Centrosome;Nucleolus;Cytoplasm | Molecular function unknown | Centrosome |
| TIMM50 | NP_001001563.1 | 92609 | mitochondrial import inner membrane translocase subunit TIM50 | 20.6 | 1 | 8 | 8 | 24 | 3.92E+08 | 2.96 | 4 | 24.6 | 456 | 50.4 | 9.4 | Cell communication;Signal transduction | Mitochondrion | Protein tyrosine/serine/threonine phosphatase activity | Mitochondrion |
| GDI1 | NP_001484.1 | 2664 | rab GDP dissociation inhibitor alpha | 64.4 | 1 | 13 | 24 | 138 | 7.84E+08 | 2.96 | 14 | 38.7 | 447 | 50.6 | 5.1 | Cell communication;Signal transduction | Cytoplasm | GTPase activator activity | Cytoplasm |
| SQRDL | NP_067022.1 | 58472 | sulfide:quinone oxidoreductase, mitochondrial | 43.6 | 1 | 10 | 10 | 23 | 3.00E+08 | 2.95 | 9 | 18.1 | 450 | 49.9 | 9.1 | Metabolism;Energy pathways | Mitochondrion | Oxidoreductase activity | Mitochondrion |
| ANXA5 | NP_001145.1 | 308 | annexin A5 | 79.7 | 1 | 25 | 25 | 199 | 2.98E+09 | 2.95 | 50 | 20.8 | 320 | 35.9 | 5.1 | Cell communication;Signal transduction | Extracellular;Cytoplasm;Nucleus;Plasma membrane;Endoplasmic reticulum | Calcium ion binding | Cytoplasm |
| SRI | NP_001243820.1 | 6717 | sorcin isoform C | 65.1 | 4 | 7 | 7 | 26 | 5.18E+08 | 2.94 | 13 | 28.5 | 195 | 21.3 | 6.0 | Cell communication;Signal transduction | Cytoplasm;Plasma membrane | Calcium ion binding | Cytoplasm |
| NCOR2 | NP_001070729.2 | 9612 | nuclear receptor corepressor 2 isoform 2 | 1.7 | 3 | 3 | 3 | 3 | 2.95E+07 | 2.93 | 1 | - | 2458 | 267.9 | 7.8 | Regulation of nucleobase, nucleoside, nucleotide and nucleic acid metabolism | Nucleus;Cytoplasm | Transcription regulator activity | Nucleus |
| CDK6 | NP_001138778.1 | 1021 | cyclin-dependent kinase 6 | 4.6 | 1 | 1 | 1 | 1 | 3.45E+06 | 2.92 | 1 | - | 326 | 36.9 | 6.5 | Cell communication;Signal transduction | Nucleus;Cytoplasm | Protein binding | Nucleus |
| TSC1 | NP_001155899.1 | 7248 | hamartin isoform 4 | 5.0 | 3 | 3 | 3 | 3 | 1.01E+08 | 2.90 | 1 | - | 1113 | 123.9 | 6.2 | Regulation of cell growth | Cytoplasm;Centrosome | Protein binding | Cytoplasm |
| MSI2 | NP_733839.1 | 124540 | RNA-binding protein Musashi homolog 2 isoform b | 10.8 | 2 | 1 | 1 | 1 | 5.52E+07 | 2.90 | 1 | - | 251 | 28.4 | 8.5 | Regulation of nucleobase, nucleoside, nucleotide and nucleic acid metabolism | Cytoplasm | RNA binding | Cytoplasm |
| TGFBI | NP_000349.1 | 7045 | transforming growth factor-beta-induced protein ig-h3 precursor | 32.7 | 1 | 11 | 11 | 15 | 1.26E+08 | 2.90 | 6 | 15.9 | 683 | 74.6 | 7.7 | Cell communication;Signal transduction | Extracellular;Cytoplasm;Nucleus | Receptor binding | Extracellular |
| PPM1F | NP_055449.1 | 9647 | protein phosphatase 1F | 29.3 | 1 | 6 | 6 | 18 | 1.44E+08 | 2.90 | 6 | 22.9 | 454 | 49.8 | 5.1 | Cell communication;Signal transduction | Cytoplasm | Protein serine/threonine phosphatase activity | Cytoplasm |
| HIGD1A | NP_054775.2 | 25994 | HIG1 domain family member 1A, mitochondrial isoform b | 46.2 | 2 | 2 | 2 | 8 | 1.57E+08 | 2.89 | 2 | 51.9 | 93 | 10.1 | 9.8 | Biological_process unknown | - | Molecular function unknown | - |
| CRTC2 | NP_859066.1 | 200186 | CREB-regulated transcription coactivator 2 | 5.8 | 1 | 2 | 2 | 3 | 2.46E+07 | 2.89 | 1 | - | 693 | 73.3 | 7.1 | Regulation of nucleobase, nucleoside, nucleotide and nucleic acid metabolism | Nucleus;Cytoplasm | Transcription regulator activity | Nucleus |
| TMEM261 | NP_219500.1 | 90871 | transmembrane protein 261 | 33.9 | 1 | 1 | 1 | 1 | 5.56E+08 | 2.87 | 1 | - | 112 | 11.8 | 9.9 | Biological_process unknown | Integral to membrane | Molecular function unknown | Integral to membrane |
| YAE1D1 | NP_064577.1 | 57002 | yae1 domain-containing protein 1 isoform 1 | 12.4 | 1 | 1 | 1 | 1 | 5.70E+07 | 2.86 | 1 | - | 226 | 25.3 | 5.6 | Biological_process unknown | - | Molecular function unknown | - |
| GNB4 | NP_067642.1 | 59345 | guanine nucleotide-binding protein subunit beta-4 | 45.6 | 1 | 3 | 8 | 30 | 7.15E+08 | 2.85 | 1 | - | 340 | 37.5 | 6.0 | Cell communication;Signal transduction | Plasma membrane;Mitochondrion | GTPase activity | Plasma membrane |
| GNG12 | NP_061329.3 | 55970 | guanine nucleotide-binding protein G(I)/G(S)/G(O) subunit gamma-12 precursor | 70.8 | 1 | 5 | 5 | 13 | 6.19E+08 | 2.85 | 8 | 8.6 | 72 | 8 | 9.0 | Cell communication;Signal transduction | Cytoplasm | GTPase activity | Cytoplasm |
| KIAA1524 | NP_065941.2 | 57650 | protein CIP2A | 13.9 | 1 | 6 | 7 | 10 | 9.70E+07 | 2.85 | 6 | 31 | 905 | 102.1 | 6.2 | Biological_process unknown | Cytoplasm;Nucleus;Cytosol | Molecular function unknown | Cytoplasm |

| Gene Symbol | RefSeq_Accession | Entrez Gene ID | Description | Coverage (%) | Proteins | Unique Peptides | # Peptides | PSM | Area | SCC-R /SCC-S | SCC-R /SCC-S (Count) | SCC-R /SCC-S (Variability [%]) | # AAs | MW [kDa] | calc. pI | Biological Process | Localization | Molecular Function | Primary Localization |
|---|---|---|---|---|---|---|---|---|---|---|---|---|---|---|---|---|---|---|---|
| ANKRD28 | NP_056014.2 | 23243 | serine/threonine-protein phosphatase 6 regulatory ankyrin repeat subunit A isoform a | 25.3 | 2 | 13 | 13 | 16 | 2.09E+08 | 2.84 | 7 | 12.5 | 1053 | 112.9 | 6.3 | Biological_process unknown | - | Molecular function unknown | - |
| SLC30A7 | NP_598003.2 | 148867 | zinc transporter 7 | 6.7 | 1 | 2 | 2 | 7 | 5.45E+07 | 2.83 | 1 | - | 376 | 41.6 | 7.0 | Transport | Golgi apparatus;Cytoplasmic vesicle | Auxiliary transport protein activity | Golgi apparatus |
| WIPI2 | NP_001265228.1 | 26100 | WD repeat domain phosphoinositide-interacting protein 2 isoform f | 18.4 | 6 | 2 | 2 | 3 | 4.49E+07 | 2.83 | 2 | 89.1 | 310 | 33.5 | 5.5 | Cell death | - | Molecular function unknown | - |
| MACF1 | NP_036222.3 | 23499 | microtubule-actin cross-linking factor 1 | 17.0 | 1 | 53 | 53 | 73 | 5.07E+08 | 2.82 | 32 | 23.1 | 5430 | 620 | 5.4 | Cell communication;Signal transduction | Cytoplasm;Golgi apparatus;Microtubule;Mitochondrion;Nucleus | Cytoskeletal protein binding | Cytoplasm;Nucleus |
| OCRL | NP_000267.2 | 4952 | inositol polyphosphate 5-phosphatase OCRL-1 isoform a | 4.9 | 2 | 3 | 3 | 3 | 7.89E+07 | 2.82 | 3 | 19.8 | 901 | 104.1 | 6.6 | Metabolism;Energy pathways;Vesicle-mediated transport;Signal transduction;Cell-cell adhesion | Golgi apparatus;Cell projection;Lysosome;Early endosome;Clathrin-coated vesicle;Golgi membrane;Cytoplasm | Catalytic activity;Acid phosphatase activity | Golgi membrane |
| TMEM43 | NP_077310.1 | 79188 | transmembrane protein 43 | 41.0 | 1 | 10 | 10 | 39 | 3.67E+08 | 2.82 | 9 | 30.5 | 400 | 44.8 | 8.1 | Biological_process unknown | Integral to membrane | Molecular function unknown | Integral to membrane |
| QDPR | NP_000311.2 | 5860 | dihydropteridine reductase | 28.3 | 1 | 4 | 4 | 10 | 1.10E+08 | 2.81 | 3 | 14.7 | 244 | 25.8 | 7.4 | Metabolism;Energy pathways | Extracellular | Catalytic activity | Extracellular |
| CHCHD2 | NP_057223.1 | 51142 | coiled-coil-helix-coiled-coil-helix domain-containing protein 2, mitochondrial precursor | 33.8 | 1 | 3 | 3 | 31 | 7.42E+08 | 2.80 | 4 | 28.5 | 151 | 15.5 | 9.2 | Biological_process unknown | - | Molecular function unknown | - |
| TACC3 | NP_006333.1 | 10460 | transforming acidic coiled-coil-containing protein 3 | 7.8 | 1 | 4 | 4 | 6 | 1.41E+08 | 2.80 | 2 | 8.3 | 838 | 90.3 | 5.1 | Cell communication;Signal transduction | Nucleus;Cytoplasm;Centrosome;Microtubule | Molecular function unknown | Nucleus |
| EFHD2 | NP_077305.2 | 79180 | EF-hand domain-containing protein D2 | 55.0 | 1 | 10 | 10 | 25 | 3.93E+08 | 2.80 | 6 | 26.7 | 240 | 26.7 | 5.2 | Biological_process unknown | Nucleus;Cytoplasm | Molecular function unknown | Nucleus;Cytoplasm |
| DDAH2 | NP_039268.1 | 23564 | N(G),N(G)-dimethylarginine dimethylaminohydrolase 2 | 18.3 | 1 | 2 | 2 | 4 | 1.50E+08 | 2.79 | 2 | 50.9 | 285 | 29.6 | 6.0 | Metabolism;Energy pathways | Cytoplasm | Hydrolase activity | Cytoplasm |
| ASB6 | NP_001189332.1 | 140459 | ankyrin repeat and SOCS box protein 6 isoform 3 | 4.6 | 2 | 1 | 1 | 1 | 1.36E+08 | 2.79 | 1 | - | 392 | 44.1 | 6.0 | Cell communication;Signal transduction | Cytoplasm | Molecular function unknown | Cytoplasm |
| PRUNE2 | NP_056040.2 | 158471 | protein prune homolog 2 | 0.4 | 1 | 1 | 1 | 1 | 3.47E+07 | 2.79 | 1 | - | 3088 | 340.4 | 4.5 | Biological_process unknown | Nucleus;Cytoplasm | Molecular function unknown | Nucleus;Cytoplasm |
| CRTAP | NP_006362.1 | 10491 | cartilage-associated protein precursor | 3.7 | 1 | 1 | 1 | 1 | 1.22E+08 | 2.79 | 1 | - | 401 | 46.5 | 5.7 | Biological_process unknown | Cytoplasm;Plasma membrane | Molecular function unknown | Cytoplasm |
| PLA2G15 | NP_036452.1 | 23659 | group XV phospholipase A2 precursor | 4.1 | 1 | 1 | 1 | 1 | 2.96E+06 | 2.79 | 1 | - | 412 | 46.6 | 6.7 | Metabolism;Energy pathways | Extracellular;Lysosome | Phospholipase activity | Extracellular |
| PARP4 | NP_006428.2 | 143 | poly [ADP-ribose] polymerase 4 | 9.6 | 1 | 9 | 9 | 14 | 1.00E+08 | 2.79 | 4 | 4.3 | 1724 | 192.5 | 5.7 | Regulation of nucleobase, nucleoside, nucleotide and nucleic acid metabolism | Nucleus | Catalytic activity | Nucleus |
| WDYHV1 | NP_060494.1 | 55093 | protein N-terminal glutamine amidohydrolase isoform 1 | 8.8 | 1 | 1 | 1 | 1 | 2.52E+07 | 2.78 | 1 | - | 205 | 23.7 | 5.8 | Biological_process unknown | - | Molecular function unknown | - |
| SARS2 | NP_060297.1 | 54938 | serine--tRNA ligase, mitochondrial isoform b precursor | 37.8 | 2 | 11 | 11 | 21 | 1.02E+08 | 2.75 | 9 | 16.9 | 518 | 58.2 | 8.1 | Protein metabolism | Mitochondrion;Cytoplasm | Ligase activity | Mitochondrion |
| NFKBIB | NP_001230045.1 | 4793 | NF-kappa-B inhibitor beta isoform 2 | 23.7 | 2 | 4 | 4 | 8 | 2.34E+08 | 2.75 | 2 | 73.3 | 270 | 29.1 | 5.1 | Regulation of nucleobase, nucleoside, nucleotide and nucleic acid metabolism | Nucleus;Cytoplasm | Transcription regulator activity | Cytoplasm |
| IFIT5 | NP_036552.1 | 24138 | interferon-induced protein with tetratricopeptide repeats 5 | 30.5 | 1 | 9 | 9 | 13 | 1.39E+08 | 2.75 | 6 | 11 | 482 | 55.8 | 7.4 | Biological_process unknown | - | Molecular function unknown | - |
| HNRNPUL1 | NP_653333.1 | 11100 | heterogeneous nuclear ribonucleoprotein U-like protein 1 isoform d | 36.0 | 2 | 21 | 21 | 72 | 1.24E+09 | 2.74 | 28 | 25.9 | 756 | 84.7 | 8.8 | Regulation of nucleobase, nucleoside, nucleotide and nucleic acid metabolism;RNA metabolism;Regulation of gene expression, epigenetic | Cytoplasm;Nucleus | Ribonucleoprotein;RNA binding | Nucleus |
| AK6 | NP_057367.1 | 102157402 | adenylate kinase isoenzyme 6 isoform b | 9.3 | 1 | 2 | 2 | 3 | 3.12E+07 | 2.72 | 2 | 19 | 172 | 20 | 4.6 | - | - | - | - |
| VAMP4 | NP_001172056.1 | 8674 | vesicle-associated membrane protein 4 isoform 2 | 13.6 | 2 | 1 | 1 | 2 | 5.51E+07 | 2.72 | 1 | - | 140 | 16.2 | 6.6 | Protein transport | Golgi apparatus;Nucleus;Cytoplasm;Endosome | Auxiliary transport protein activity | Golgi apparatus |
| POLR2I | NP_006224.1 | 5438 | DNA-directed RNA polymerase II subunit RPB9 | 52.8 | 1 | 5 | 5 | 11 | 2.85E+08 | 2.72 | 5 | 30.9 | 125 | 14.5 | 5.1 | Regulation of nucleobase, nucleoside, nucleotide and nucleic acid metabolism | Nucleus | DNA-directed RNA polymerase activity | Nucleus |
| CD44 | NP_001189485.1 | 960 | CD44 antigen isoform 7 precursor | 27.9 | 7 | 8 | 8 | 52 | 1.10E+09 | 2.71 | 17 | 19.3 | 340 | 37.3 | 5.5 | Cell communication;Signal transduction | Plasma membrane;Cytoplasm;Nucleus | Receptor activity | Plasma membrane |
| KIAA1191 | NP_001073152.1 | 57179 | putative monooxygenase p33MONOX isoform b | 19.9 | 3 | 2 | 2 | 4 | 3.08E+07 | 2.71 | 2 | 102.5 | 286 | 31.3 | 9.4 | Biological_process unknown | - | Molecular function unknown | - |
| SLC9A6 | NP_001036002.1 | 10479 | sodium/hydrogen exchanger 6 isoform a precursor | 6.7 | 3 | 3 | 3 | 5 | 5.76E+07 | 2.71 | 2 | 141.6 | 701 | 77.9 | 6.4 | Transport | Mitochondrion;Plasma membrane | Transporter activity | Mitochondrion |
| PDCD5 | NP_004699.1 | 9141 | programmed cell death protein 5 | 56.0 | 1 | 6 | 6 | 26 | 8.65E+08 | 2.71 | 10 | 10.5 | 125 | 14.3 | 6.0 | Apoptosis | Nucleus;Cytoplasm | Molecular function unknown | Cytoplasm |
| FAM65A | NP_078795.2 | 79567 | protein FAM65A isoform 1 | 2.4 | 4 | 2 | 2 | 5 | 3.66E+07 | 2.70 | 3 | 33.1 | 1219 | 131.8 | 6.3 | Biological_process unknown | - | Molecular function unknown | - |
| STAT6 | NP_001171550.1 | 6778 | signal transducer and activator of transcription 6 isoform 1 | 6.7 | 2 | 4 | 4 | 7 | 8.91E+07 | 2.69 | 3 | 25.8 | 847 | 94.1 | 6.2 | Regulation of nucleobase, nucleoside, nucleotide and nucleic acid metabolism | Cytoplasm;Nucleus | Transcription factor activity | Cytoplasm |
| DTNBP1 | NP_898861.1 | 84062 | dysbindin isoform b | 4.3 | 3 | 1 | 1 | 1 | 2.47E+06 | 2.68 | 1 | - | 303 | 34.8 | 5.3 | Cell organization and biogenesis | Nucleus;Axon;Cytoplasm | Protein binding | Cytoplasm |
| OTUD6B | NP_057107.3 | 51633 | OTU domain-containing protein 6B isoform 1 | 15.5 | 2 | 2 | 2 | 4 | 1.06E+07 | 2.68 | 1 | - | 323 | 37.3 | 6.7 | Biological_process unknown | - | Molecular function unknown | - |
| AKAP8L | NP_001278407.1 | 26993 | A-kinase anchor protein 8-like isoform 2 | 9.7 | 2 | 2 | 2 | 4 | 5.92E+07 | 2.68 | 2 | 15.8 | 585 | 65.2 | 5.1 | Regulation of nucleobase, nucleoside, nucleotide and nucleic acid metabolism | Nucleus;Cytoplasm | DNA binding | Nucleus |
| CRTC1 | NP_056136.2 | 23373 | CREB-regulated transcription coactivator 1 isoform 1 | 6.5 | 2 | 2 | 2 | 3 | 4.46E+07 | 2.68 | 2 | 44.4 | 634 | 67.3 | 6.1 | Biological_process unknown;CAMP-mediated signaling | Cytoplasm;Nucleus | Molecular function unknown;Transcription cofactor activity | Cytoplasm;Nucleus |
| LZTFL1 | NP_001263308.1 | 54585 | leucine zipper transcription factor-like protein 1 isoform 3 | 14.5 | 3 | 3 | 3 | 3 | 3.07E+07 | 2.68 | 2 | 40.4 | 256 | 29.3 | 5.5 | Biological_process unknown | - | Molecular function unknown | - |
| TYMS | NP_001062.1 | 7298 | thymidylate synthase | 21.7 | 1 | 5 | 5 | 7 | 6.44E+07 | 2.66 | 1 | - | 313 | 35.7 | 7.0 | Metabolism;Energy pathways | Nucleus;Cytoplasm;Mitochondrion;Nucleolus | Ligase activity | Nucleus |
| GRN | NP_002078.1 | 2896 | granulins precursor | 13.5 | 1 | 4 | 4 | 5 | 6.28E+07 | 2.66 | 2 | 8.9 | 593 | 63.5 | 6.8 | Cell communication;Signal transduction | Extracellular;Cytoplasm | Growth factor activity | Extracellular |

| Gene Symbol | RefSeq_Accession | Entrez Gene ID | Description | Coverage (%) | Proteins | Unique Peptides | # Peptides | PSM | Area | SCC-R /SCC-S | SCC-R /SCC-S (Count) | SCC-R /SCC-S (Variability [%]) | # AAs | MW [kDa] | calc. pI | Biological Process | Localization | Molecular Function | Primary Localization |
|---|---|---|---|---|---|---|---|---|---|---|---|---|---|---|---|---|---|---|---|
| **UBE2E2** | NP_689866.1 | 7325 | **ubiquitin-conjugating enzyme E2 E2** | 20.4 | 2 | 2 | 2 | 2 | 1.57E+07 | **2.65** | 1 | - | 201 | 22.2 | 7.7 | Protein metabolism | - | Ubiquitin-specific protease activity | - |
| **SPEN** | NP_055816.2 | 23013 | **msx2-interacting protein** | 1.2 | 1 | 2 | 2 | 3 | 6.01E+07 | **2.65** | 1 | - | 3664 | 402 | 7.6 | Regulation of nucleobase, nucleoside, nucleotide and nucleic acid metabolism | Nucleus;Cytoplasm | Transcription regulator activity | Nucleus |
| **CNOT4** | NP_001008226.1 | 4850 | **CCR4-NOT transcription complex subunit 4 isoform b** | 2.3 | 6 | 1 | 1 | 1 | 2.04E+06 | **2.64** | 1 | - | 572 | 63 | 7.0 | Regulation of nucleobase, nucleoside, nucleotide and nucleic acid metabolism | Nucleus | Transcription regulator activity | Nucleus |
| **TMEM184B** | NP_001182001.1 | 25829 | **transmembrane protein 184B isoform b** | 10.6 | 2 | 1 | 1 | 1 | 4.92E+07 | **2.64** | 1 | - | 341 | 38.7 | 7.6 | Biological_process unknown | Cytoplasm | Molecular function unknown | Cytoplasm |
| **SDC4** | NP_002990.2 | 6385 | **syndecan-4 precursor** | 18.7 | 1 | 3 | 3 | 4 | 1.27E+08 | **2.64** | 2 | 43.8 | 198 | 21.6 | 4.5 | Cell communication;Signal transduction | Plasma membrane | Receptor activity | Plasma membrane |
| **GAMT** | NP_000147.1 | 2593 | **guanidinoacetate N-methyltransferase isoform a** | 23.3 | 2 | 2 | 2 | 4 | 8.24E+07 | **2.62** | 1 | - | 236 | 26.3 | 6.1 | Protein metabolism | - | Acyltransferase activity | - |
| **SPG20** | NP_055902.1 | 23111 | **spartin** | 19.5 | 1 | 7 | 7 | 12 | 1.72E+08 | **2.62** | 5 | 32.1 | 666 | 72.8 | 5.9 | Transport | Cytoplasm;Mitochondrion;Microtubule | Molecular function unknown | Cytoplasm |
| **DAP** | NP_004385.1 | 1611 | **death-associated protein 1 isoform 2** | 38.2 | 2 | 3 | 3 | 6 | 1.26E+08 | **2.61** | 1 | - | 102 | 11.2 | 9.3 | Apoptosis | Cytoplasm;Nucleus | Molecular function unknown | Cytoplasm |
| **CENPF** | NP_057427.3 | 1063 | **centromere protein F** | 1.6 | 1 | 3 | 3 | 4 | 3.74E+07 | **2.61** | 2 | 3.4 | 3114 | 357.3 | 5.1 | Cell growth and/or maintenance | Nucleus;Kinetochore;Cytoplasm | Transcription factor binding | Nucleus |
| **SERINC1** | NP_065806.1 | 57515 | **serine incorporator 1 precursor** | 6.6 | 1 | 2 | 2 | 5 | 6.89E+07 | **2.60** | 2 | 83.5 | 453 | 50.5 | 5.9 | Biological_process unknown | Plasma membrane;Nucleus;Cytoplasm | Protein binding | Plasma membrane |
| **NCDN** | NP_001014839.1 | 23154 | **neurochondrin isoform 1** | 23.1 | 1 | 1 | 10 | 21 | 1.34E+08 | **2.60** | 1 | - | 729 | 78.8 | 5.5 | Biological_process unknown | Cytoplasm | Molecular function unknown | Cytoplasm |
| **AIDA** | NP_073742.2 | 64853 | **axin interactor, dorsalization-associated protein** | 10.1 | 1 | 2 | 2 | 2 | 9.61E+06 | **2.59** | 1 | - | 306 | 35 | 6.6 | Biological_process unknown | - | Molecular function unknown | - |
| **ACOT7** | NP_863656.1 | 11332 | **cytosolic acyl coenzyme A thioester hydrolase isoform hBACHd** | 22.8 | 4 | 5 | 6 | 14 | 3.61E+08 | **2.59** | 5 | 13.3 | 329 | 36.5 | 7.8 | Lipid metabolism;Metabolism;Energy pathways | Cytoplasm;Nucleus;Mitochondrion;Cytosol | Hydrolase activity | Cytoplasm;Mitochondrion;Cytosol |
| **SCAF4** | NP_001138917.1 | 57466 | **splicing factor, arginine/serine-rich 15 isoform 3** | 10.0 | 3 | 5 | 5 | 5 | 1.20E+08 | **2.59** | 1 | - | 1125 | 123.6 | 9.6 | Regulation of nucleobase, nucleoside, nucleotide and nucleic acid metabolism | Nucleus;Cytoplasm | Transcription regulator activity | Nucleus;Cytoplasm |
| **AGTRAP** | NP_001035284.1 | 57085 | **type-1 angiotensin II receptor-associated protein isoform b** | 14.5 | 2 | 1 | 1 | 5 | 5.85E+08 | **2.59** | 1 | - | 152 | 16.7 | 6.3 | Biological_process unknown | Integral to membrane | Molecular function unknown | Integral to membrane |
| **INTS4** | NP_291025.3 | 92105 | **integrator complex subunit 4** | 3.8 | 1 | 2 | 2 | 3 | 2.60E+07 | **2.59** | 1 | - | 963 | 108.1 | 6.4 | Regulation of nucleobase, nucleoside, nucleotide and nucleic acid metabolism | - | Transcription regulator activity | - |
| **NNMT** | NP_006160.1 | 4837 | **nicotinamide N-methyltransferase** | 6.8 | 1 | 1 | 1 | 1 | 5.89E+07 | **2.58** | 1 | - | 264 | 29.6 | 5.7 | Metabolism;Energy pathways | Cytoplasm | Methyltransferase activity | Cytoplasm |
| **ACOT1** | NP_001032238.1 | 641371 | **acyl-coenzyme A thioesterase 1** | 48.7 | 4 | 11 | 11 | 16 | 2.89E+08 | **2.57** | 8 | 43.9 | 421 | 46.2 | 7.3 | Biological_process unknown | - | Molecular function unknown | - |
| **ZNF330** | NP_055302.1 | 27309 | **zinc finger protein 330 isoform 1** | 27.8 | 2 | 4 | 4 | 5 | 1.20E+07 | **2.57** | 1 | - | 320 | 36.2 | 6.2 | Biological_process unknown | Nucleolus;Nucleus;Cytoplasm | Molecular function unknown | Nucleolus |
| **DDAH1** | NP_036269.1 | 23576 | **N(G),N(G)-dimethylarginine dimethylaminohydrolase 1 isoform 1** | 51.6 | 2 | 8 | 8 | 13 | 3.29E+08 | **2.57** | 7 | 16.5 | 285 | 31.1 | 5.8 | Metabolism;Energy pathways | Cytoplasm;Integral to membrane | Hydrolase activity | Cytoplasm |
| **GYG1** | NP_001171649.1 | 2992 | **glycogenin-1 isoform 2** | 10.8 | 3 | 4 | 4 | 7 | 2.37E+08 | **2.56** | 4 | 20.6 | 333 | 37.5 | 5.4 | Metabolism;Energy pathways | - | Molecular function unknown | - |
| **VPS36** | NP_001269097.1 | 51028 | **vacuolar protein-sorting-associated protein 36 isoform 2** | 18.3 | 3 | 4 | 4 | 5 | 1.95E+07 | **2.55** | 1 | - | 377 | 42.7 | 7.5 | Biological_process unknown | - | Molecular function unknown | - |
| **N4BP1** | NP_694574.3 | 9683 | **NEDD4-binding protein 1** | 13.4 | 1 | 5 | 5 | 7 | 5.64E+07 | **2.55** | 1 | - | 896 | 100.3 | 5.4 | - | - | - | - |
| **BAD** | NP_116784.1 | 572 | **bcl2 antagonist of cell death** | 22.6 | 1 | 2 | 2 | 2 | 4.64E+07 | **2.54** | 2 | 64.7 | 168 | 18.4 | 7.2 | Cell cycle;Apoptosis | Mitochondrion;Cytoplasm | Peptide binding | Mitochondrion |
| **C19orf52** | NP_612367.1 | 90580 | **uncharacterized protein C19orf52** | 11.5 | 1 | 2 | 2 | 2 | 4.08E+07 | **2.54** | 1 | - | 260 | 29.2 | 8.1 | Biological_process unknown | - | Molecular function unknown | - |
| **CKAP4** | NP_006816.2 | 10970 | **cytoskeleton-associated protein 4** | 40.7 | 1 | 17 | 17 | 42 | 4.81E+08 | **2.54** | 12 | 8.1 | 602 | 66 | 5.9 | Cell growth and/or maintenance | Plasma membrane;Endoplasmic reticulum;Cytoplasm;Golgi apparatus;Cytoskeleton | Cytoskeletal protein binding | Plasma membrane |
| **DAB2IP** | NP_619723.1 | 153090 | **disabled homolog 2-interacting protein isoform 2** | 0.9 | 2 | 1 | 1 | 1 | 3.42E+07 | **2.53** | 1 | - | 1065 | 117.6 | 6.8 | Cell communication;Signal transduction | - | Molecular function unknown | - |
| **HMGB2** | NP_001124160.1 | 3148 | **high mobility group protein B2** | 32.1 | 1 | 11 | 11 | 53 | 1.49E+09 | **2.53** | 15 | 15.5 | 209 | 24 | 7.8 | Regulation of nucleobase, nucleoside, nucleotide and nucleic acid metabolism | Nucleus;Nucleolus;Cytoplasm;Mitochondrion | Transcription regulator activity | Nucleus |
| **GSTO1** | NP_004823.1 | 9446 | **glutathione S-transferase omega-1 isoform 1** | 46.9 | 3 | 12 | 12 | 88 | 1.91E+09 | **2.53** | 22 | 21.4 | 241 | 27.5 | 6.6 | Metabolism;Energy pathways | Nucleus;Cytoplasm;Plasma membrane | Glutathione transferase activity | Nucleus |
| **SMIM12** | NP_612437.3 | 113444 | **small integral membrane protein 12** | 30.4 | 1 | 2 | 2 | 2 | 1.33E+07 | **2.53** | 2 | 88.2 | 92 | 10.8 | 8.4 | Biological_process unknown | - | Molecular function unknown | - |
| **PHLDA1** | NP_031376.3 | 22822 | **pleckstrin homology-like domain family A member 1** | 2.2 | 1 | 1 | 1 | 4 | 1.67E+08 | **2.52** | 2 | 32.1 | 401 | 45 | 9.9 | Protein metabolism;Apoptosis | Cytoplasm;Peroxisome;Nucleolus | Transcription factor activity | Cytoplasm |
| **ABI2** | NP_005750.4 | 10152 | **abl interactor 2 isoform c** | 3.6 | 3 | 1 | 1 | 1 | 2.98E+07 | **2.52** | 1 | - | 475 | 52.4 | 6.0 | Cell communication;Signal transduction;Regulation of cell proliferation | Nucleus;Cytoplasm;Cytosol | Kinase regulator activity;Protein kinase binding | Cytoplasm |
| **CTSA** | NP_001121167.1 | 5476 | **lysosomal protective protein isoform b precursor** | 21.5 | 3 | 6 | 6 | 13 | 9.43E+07 | **2.52** | 2 | 15.2 | 480 | 54.4 | 6.6 | Protein metabolism | Lysosome;Endoplasmic reticulum;Golgi apparatus;Extracellular | Serine-type peptidase activity | Lysosome |
| **LRP1** | NP_002323.2 | 4035 | **prolow-density lipoprotein receptor-related protein 1 precursor** | 1.3 | 1 | 3 | 3 | 5 | 9.04E+07 | **2.52** | 3 | 24.9 | 4544 | 504.3 | 5.4 | Cell communication;Signal transduction | Plasma membrane;Endoplasmic reticulum;Golgi apparatus;Endosome;Extracellular;Nucleus | Receptor activity | Endoplasmic reticulum |
| **GSK3A** | NP_063937.2 | 2931 | **glycogen synthase kinase-3 alpha** | 15.1 | 1 | 4 | 5 | 7 | 3.12E+08 | **2.52** | 1 | - | 483 | 50.9 | 8.8 | Cell communication;Signal transduction | Cytoplasm;Centrosome | Protein serine/threonine kinase activity | Cytoplasm |
| **ANKHD1** | NP_060217.1 | 54882 | **ankyrin repeat and KH domain-containing protein 1 isoform 1** | 4.6 | 5 | 3 | 5 | 7 | 2.95E+07 | **2.51** | 2 | 12.6 | 2542 | 269.3 | 5.7 | Anti-apoptosis | Cytoplasm | Molecular function unknown | Cytoplasm |
| **CLIC1** | NP_001279.2 | 1192 | **chloride intracellular channel protein 1** | 88.0 | 5 | 19 | 20 | 158 | 6.14E+09 | **2.51** | 29 | 9.5 | 241 | 26.9 | 5.2 | Transport | Cytoplasm;Nucleus;Plasma membrane | Intracellular ligand-gated ion channel activity | Nucleus |

| Gene Symbol | RefSeq_Accession | Entrez Gene ID | Description | Coverage (%) | Proteins | Unique Peptides | # Peptides | PSM | Area | SCC-R /SCC-S | SCC-R /SCC-S (Count) | SCC-R /SCC-S (Variability [%]) | # AAs | MW [kDa] | calc. pI | Biological Process | Localization | Molecular Function | Primary Localization |
|---|---|---|---|---|---|---|---|---|---|---|---|---|---|---|---|---|---|---|---|
| FAM129A | NP_443198.1 | 116496 | protein Niban | 14.7 | 1 | 7 | 7 | 14 | 3.61E+08 | 2.51 | 7 | 18 | 928 | 103.1 | 4.8 | Biological_process unknown | - | Molecular function unknown | - |
| LEPRE1 | NP_071751.3 | 64175 | prolyl 3-hydroxylase 1 isoform 1 precursor | 18.9 | 3 | 8 | 8 | 15 | 1.16E+08 | 2.50 | 7 | 48.3 | 736 | 83.3 | 5.1 | Metabolism;Energy pathways | Cytoplasm;Nucleus;Integral to membrane | Catalytic activity | Cytoplasm |
| SUPT5H | NP_001124297.1 | 6829 | transcription elongation factor SPT5 isoform b | 25.1 | 2 | 16 | 16 | 28 | 1.77E+08 | 2.50 | 11 | 18.8 | 1083 | 120.4 | 5.1 | Regulation of nucleobase, nucleoside, nucleotide and nucleic acid metabolism | Nucleus | Transcription factor activity | Nucleus |
| ZC3HAV1L | NP_542391.2 | 92092 | zinc finger CCCH-type antiviral protein 1-like | 6.7 | 1 | 1 | 1 | 1 | 6.49E+07 | 2.49 | 1 | - | 300 | 32.9 | 8.1 | Biological_process unknown | - | Molecular function unknown | - |
| MRC2 | NP_006030.2 | 9902 | C-type mannose receptor 2 precursor | 2.0 | 1 | 2 | 2 | 2 | 9.35E+06 | 2.48 | 1 | - | 1479 | 166.6 | 5.8 | Biological_process unknown | Plasma membrane | Receptor activity | Plasma membrane |
| HAGH | NP_001035517.1 | 3029 | hydroxyacylglutathione hydrolase, mitochondrial isoform 2 | 41.9 | 3 | 5 | 5 | 7 | 1.22E+08 | 2.48 | 4 | 8.5 | 260 | 28.8 | 7.3 | Metabolism;Energy pathways | Cytoplasm;Mitochondrion | Hydrolase activity | Cytoplasm |
| OCIAD2 | NP_001014446.1 | 132299 | OCIA domain-containing protein 2 isoform 1 | 35.7 | 3 | 5 | 5 | 13 | 2.22E+08 | 2.48 | 7 | 6.8 | 154 | 16.9 | 9.0 | Biological_process unknown | - | Molecular function unknown | - |
| NPTN | NP_059429.1 | 27020 | neuroplastin isoform a precursor | 6.0 | 2 | 2 | 2 | 3 | 1.07E+08 | 2.47 | 2 | 23 | 282 | 31.3 | 6.9 | Immune response | Plasma membrane | Antigen binding | Plasma membrane |
| FHL3 | NP_001230807.1 | 2275 | four and a half LIM domains protein 3 isoform 2 | 27.3 | 2 | 2 | 2 | 2 | 1.02E+08 | 2.46 | 1 | - | 172 | 18.9 | 7.6 | Cell communication;Signal transduction | Nucleus;Cytoplasm;Plasma membrane;Mitochondrion | Transcription regulator activity | Nucleus |
| TSEN34 | NP_001269261.1 | 79042 | tRNA-splicing endonuclease subunit Sen34 isoform 1 | 4.2 | 2 | 1 | 1 | 3 | 4.82E+07 | 2.46 | 1 | - | 310 | 33.6 | 8.4 | Regulation of nucleobase, nucleoside, nucleotide and nucleic acid metabolism | - | Endonuclease activity | - |
| EHBP1L1 | NP_001092879.1 | 254102 | EH domain-binding protein 1-like protein 1 | 9.5 | 1 | 7 | 7 | 9 | 6.12E+07 | 2.46 | 4 | 18.1 | 1523 | 161.8 | 4.8 | Biological_process unknown | - | Molecular function unknown | - |
| GCC2 | NP_852118.1 | 9648 | GRIP and coiled-coil domain-containing protein 2 | 2.5 | 1 | 2 | 2 | 2 | 2.90E+07 | 2.46 | 1 | - | 1684 | 195.8 | 5.1 | Cell growth and/or maintenance | Golgi apparatus;Cytoplasm;Plasma membrane | Structural molecule activity | Golgi apparatus |
| WDR6 | NP_060501.3 | 11180 | WD repeat-containing protein 6 | 1.7 | 1 | 1 | 1 | 2 | 8.73E+06 | 2.46 | 1 | - | 1151 | 124.9 | 6.9 | Biological_process unknown | Plasma membrane | Molecular function unknown | Plasma membrane |
| EHD2 | NP_055416.2 | 30846 | EH domain-containing protein 2 | 35.2 | 1 | 12 | 12 | 35 | 3.34E+08 | 2.45 | 8 | 16.4 | 543 | 61.1 | 6.5 | Cell communication;Signal transduction | Nucleus;Cytoplasm;Plasma membrane | Molecular function unknown | Cytoplasm |
| BCAR1 | NP_001164190.1 | 9564 | breast cancer anti-estrogen resistance protein 1 isoform 7 | 20.9 | 9 | 9 | 9 | 16 | 2.02E+08 | 2.45 | 4 | 16.1 | 868 | 93.1 | 5.6 | Cell communication;Signal transduction | Cytoplasm;Plasma membrane;Endoplasmic reticulum;Nucleus | Receptor signaling complex scaffold activity | Cytoplasm |
| MAN1B1 | NP_057303.2 | 11253 | endoplasmic reticulum mannosyl-oligosaccharide 1,2-alpha-mannosidase | 8.7 | 1 | 3 | 3 | 3 | 3.19E+07 | 2.45 | 2 | 50.8 | 699 | 79.5 | 7.7 | Metabolism;Energy pathways | Endoplasmic reticulum | Hydrolase activity | Endoplasmic reticulum |
| ARL2 | NP_001186674.1 | 402 | ADP-ribosylation factor-like protein 2 isoform 2 | 11.5 | 2 | 1 | 1 | 3 | 1.24E+08 | 2.44 | 1 | - | 157 | 18 | 5.9 | Cell communication;Signal transduction | Cytoplasm | GTPase activity | Cytoplasm |
| CMSS1 | NP_001161396.1 | 84319 | protein CMSS1 isoform 2 | 6.1 | 2 | 1 | 1 | 1 | 5.70E+06 | 2.44 | 1 | - | 261 | 29.9 | 9.5 | Biological_process unknown | - | Molecular function unknown | - |
| PEPD | NP_000276.2 | 5184 | xaa-Pro dipeptidase isoform 1 | 46.7 | 3 | 15 | 15 | 36 | 2.76E+08 | 2.43 | 13 | 10.8 | 493 | 54.5 | 6.0 | Protein metabolism | Cytoplasm | Peptidase activity | Cytoplasm |
| DHRS1 | NP_612461.1 | 115817 | dehydrogenase/reductase SDR family member 1 | 8.6 | 1 | 1 | 1 | 2 | 2.63E+07 | 2.43 | 1 | - | 313 | 33.9 | 7.8 | Metabolism;Energy pathways | Endoplasmic reticulum | Catalytic activity | Endoplasmic reticulum |
| MTR | NP_001278868.1 | 4548 | methionine synthase isoform 2 | 2.9 | 3 | 3 | 3 | 4 | 3.99E+07 | 2.43 | 2 | 29.3 | 1214 | 134.7 | 5.5 | Metabolism;Energy pathways | Cytoplasm | Methyltransferase activity | Cytoplasm |
| CDKN2A | NP_478102.2 | 1029 | cyclin-dependent kinase inhibitor 2A isoform p14ARF | 14.4 | 1 | 1 | 1 | 1 | 5.37E+07 | 2.42 | 1 | - | 132 | 13.9 | 12.4 | Cell communication;Signal transduction | Nucleolus;Plasma membrane;Cytoplasm | Kinase regulator activity | Nucleolus |
| CTSD | NP_001900.1 | 1509 | cathepsin D preproprotein | 53.2 | 1 | 18 | 18 | 152 | 2.40E+09 | 2.42 | 37 | 26.6 | 412 | 44.5 | 6.5 | Protein metabolism | Lysosome;Extracellular;Golgi apparatus;Endoplasmic reticulum;Mitochondrion | Aspartic-type signal peptidase activity | Lysosome |
| TAP1 | NP_000584.2 | 6890 | antigen peptide transporter 1 isoform 1 | 25.7 | 1 | 10 | 10 | 29 | 8.29E+08 | 2.41 | 9 | 29.8 | 808 | 87.2 | 8.0 | Transport | Plasma membrane;Cytoplasm;Integral to membrane | Transporter activity | Plasma membrane |
| GNPDA1 | NP_005462.1 | 10007 | glucosamine-6-phosphate isomerase 1 | 47.4 | 1 | 7 | 10 | 33 | 6.44E+08 | 2.41 | 9 | 20 | 289 | 32.6 | 6.9 | Metabolism;Energy pathways | - | Deaminase activity | - |
| SHKBP1 | NP_612401.2 | 92799 | SH3KBP1-binding protein 1 | 8.1 | 1 | 2 | 3 | 3 | 6.02E+07 | 2.41 | 1 | - | 707 | 76.3 | 8.3 | Biological_process unknown | - | Molecular function unknown | - |
| NDUFA11 | NP_783313.1 | 126328 | NADH dehydrogenase [ubiquinone] 1 alpha subcomplex subunit 11 isoform 1 | 27.0 | 2 | 2 | 2 | 3 | 6.92E+07 | 2.40 | 1 | - | 141 | 14.8 | 8.7 | Metabolism;Energy pathways | Mitochondrion | Oxidoreductase activity | Mitochondrion |
| ITGA3 | NP_002195.1 | 3675 | integrin alpha-3 isoform a precursor | 16.5 | 2 | 14 | 14 | 37 | 8.28E+08 | 2.39 | 13 | 13.5 | 1051 | 116.5 | 6.8 | Cell communication;Signal transduction | Plasma membrane;Cytoplasm | Receptor activity | Plasma membrane |
| ATP2B1 | NP_001673.2 | 490 | plasma membrane calcium-transporting ATPase 1 isoform 1b | 21.4 | 8 | 15 | 15 | 26 | 2.11E+08 | 2.39 | 9 | 39.1 | 1220 | 134.6 | 5.9 | Transport | Plasma membrane;Mitochondrion;Nucleus;Cytoplasm | ATPase activity | Plasma membrane |
| ACAD8 | NP_055199.1 | 27034 | isobutyryl-CoA dehydrogenase, mitochondrial | 5.5 | 1 | 1 | 1 | 1 | 5.23E+07 | 2.39 | 1 | - | 415 | 45 | 7.9 | Metabolism;Energy pathways | Mitochondrion | Catalytic activity | Mitochondrion |
| RTN4 | NP_008939.1 | 57142 | reticulon-4 isoform C | 30.2 | 2 | 1 | 6 | 58 | 7.12E+08 | 2.38 | 1 | - | 199 | 22.4 | 9.3 | Regulation of cell growth | Endoplasmic reticulum membrane;Plasma membrane;Mitochondrion;Nucleus;Cytoplasm | Protein binding | Endoplasmic reticulum membrane;Nucleus;Cytoplasm |
| UBE2I | NP_919237.1 | 7329 | SUMO-conjugating enzyme UBC9 | 34.8 | 1 | 6 | 6 | 17 | 4.68E+08 | 2.38 | 8 | 19.2 | 158 | 18 | 8.7 | Protein metabolism | Nucleus;Cytoplasm;Nucleolus;Mitochondrion | Ubiquitin-specific protease activity;Protein binding | Nucleus |
| AKT2 | NP_001617.1 | 208 | RAC-beta serine/threonine-protein kinase isoform 1 | 18.9 | 9 | 4 | 6 | 12 | 7.01E+07 | 2.38 | 1 | - | 481 | 55.7 | 6.4 | Cell communication;Signal transduction | Cytoplasm;Plasma membrane;Nucleus | Protein serine/threonine kinase activity | Cytoplasm |
| EHD4 | NP_644670.1 | 30844 | EH domain-containing protein 4 | 40.3 | 1 | 16 | 17 | 36 | 3.88E+08 | 2.36 | 10 | 32.4 | 541 | 61.1 | 6.8 | Cell communication;Signal transduction | Extracellular;Cytoplasm | Calcium ion binding | Extracellular |
| HEBP1 | NP_057071.2 | 50865 | heme-binding protein 1 | 76.2 | 1 | 10 | 10 | 18 | 5.17E+08 | 2.35 | 10 | 50.3 | 189 | 21.1 | 5.8 | Biological_process unknown | Cytoplasm | Molecular function unknown | Cytoplasm |
| TPRG1L | NP_877429.2 | 127262 | tumor protein p63-regulated gene 1-like protein | 14.0 | 1 | 1 | 1 | 4 | 1.45E+08 | 2.35 | 1 | - | 272 | 30.2 | 7.3 | Biological_process unknown | - | Molecular function unknown | - |
| SCYL3 | NP_065156.5 | 57147 | protein-associating with the carboxyl-terminal domain of ezrin isoform 1 | 2.5 | 2 | 1 | 1 | 1 | 2.61E+07 | 2.35 | 1 | - | 688 | 76.7 | 4.9 | Biological_process unknown | Golgi apparatus;Plasma membrane | Molecular function unknown | Golgi apparatus |

| Gene Symbol | RefSeq_Accession | Entrez Gene ID | Description | Coverage (%) | Proteins | Unique Peptides | # Peptides | PSM | Area | SCC-R /SCC-S | SCC-R /SCC-S (Count) | SCC-R /SCC-S (Variability [%]) | # AAs | MW [kDa] | calc. pI | Biological Process | Localization | Molecular Function | Primary Localization |
|---|---|---|---|---|---|---|---|---|---|---|---|---|---|---|---|---|---|---|---|
| **FAM195B** | NP_997251.2 | 348262 | **protein FAM195B isoform 2** | 41.2 | 3 | 3 | 3 | 3 | 6.17E+07 | **2.35** | 3 | 22.7 | 97 | 10.9 | 9.4 | Biological_process unknown | - | Molecular function unknown | - |
| **TPM1** | NP_001018008.1 | 7168 | **tropomyosin alpha-1 chain isoform 6** | 42.5 | 1 | 2 | 15 | 195 | 8.07E+09 | **2.35** | 3 | 1.5 | 245 | 28.4 | 4.8 | Cell growth and/or maintenance | Perinuclear region;Cytoplasm;Actin cytoskeleton | Cytoskeletal protein binding | Actin cytoskeleton |
| **LIPA** | NP_001275908.1 | 3988 | **lysosomal acid lipase/cholesteryl ester hydrolase isoform 2** | 14.5 | 2 | 2 | 2 | 7 | 8.91E+07 | **2.34** | 2 | 75.4 | 283 | 32.5 | 7.8 | Metabolism;Energy pathways | Lysosome | Lipase activity | Lysosome |
| **C1orf123** | NP_060357.1 | 54987 | **UPF0587 protein C1orf123** | 25.0 | 1 | 2 | 2 | 6 | 3.56E+08 | **2.34** | 2 | 59.3 | 160 | 18 | 5.0 | Biological_process unknown | - | Molecular function unknown | - |
| **MAGED2** | NP_957516.1 | 10916 | **melanoma-associated antigen D2** | 50.5 | 6 | 19 | 20 | 47 | 3.23E+08 | **2.33** | 15 | 31 | 606 | 64.9 | 9.3 | Cell growth and/or maintenance | Nucleus;Cytoplasm | Molecular function unknown | Nucleus |
| **IFT27** | NP_001171172.1 | 11020 | **intraflagellar transport protein 27 homolog isoform 1** | 26.3 | 2 | 4 | 4 | 5 | 1.60E+08 | **2.33** | 2 | 10.9 | 186 | 20.5 | 5.4 | Cell communication;Signal transduction | - | GTPase activity | - |
| **BTF3L4** | NP_689478.1 | 91408 | **transcription factor BTF3 homolog 4 isoform 1** | 67.1 | 2 | 5 | 5 | 32 | 1.98E+09 | **2.32** | 6 | 16 | 158 | 17.3 | 6.4 | Biological_process unknown | - | Molecular function unknown | - |
| **MED28** | NP_079481.2 | 80306 | **mediator of RNA polymerase II transcription subunit 28** | 15.2 | 1 | 1 | 1 | 2 | 1.95E+07 | **2.32** | 1 | - | 178 | 19.5 | 5.6 | Regulation of nucleobase, nucleoside, nucleotide and nucleic acid metabolism | Nucleus;Cytoskeleton | Transcription regulator activity | Nucleus;Cytoskeleton |
| **OPTN** | NP_001008213.1 | 10133 | **optineurin** | 32.8 | 1 | 12 | 12 | 17 | 1.03E+08 | **2.31** | 7 | 21.8 | 577 | 65.9 | 5.2 | Regulation of nucleobase, nucleoside, nucleotide and nucleic acid metabolism | Perinuclear region;Cytoplasm;Nucleus | Transcription regulator activity | Nucleus;Cytoplasm |
| **FBLIM1** | NP_001019387.1 | 54751 | **filamin-binding LIM protein 1 isoform c** | 22.1 | 3 | 4 | 4 | 7 | 1.13E+08 | **2.30** | 3 | 23.3 | 276 | 30.7 | 8.0 | Regulation of cell shape | Actin cytoskeleton;Extracellular matrix | Cytoskeletal protein binding | Actin cytoskeleton |
| **SNX7** | NP_689424.2 | 51375 | **sorting nexin-7 isoform b** | 2.0 | 2 | 1 | 1 | 2 | 9.59E+07 | **2.30** | 1 | - | 396 | 45 | 4.7 | Biological_process unknown | - | Molecular function unknown | - |
| **BCL2L14** | NP_110393.1 | 79370 | **apoptosis facilitator Bcl-2-like protein 14 isoform 2** | 2.4 | 2 | 1 | 1 | 2 | 9.93E+07 | **2.30** | 1 | - | 252 | 28.1 | 5.7 | Apoptosis | Cytosol;Mitochondrion | Molecular function unknown | Cytosol |
| **TMEM14C** | NP_001158730.1 | 51522 | **transmembrane protein 14C** | 34.8 | 1 | 2 | 2 | 3 | 2.66E+07 | **2.30** | 1 | - | 112 | 11.6 | 9.9 | Biological_process unknown | Integral to membrane | Molecular function unknown | Integral to membrane |
| **SUPT4H1** | NP_003159.1 | 6827 | **transcription elongation factor SPT4** | 13.7 | 1 | 1 | 1 | 3 | 2.96E+08 | **2.29** | 2 | 2.4 | 117 | 13.2 | 8.1 | Regulation of nucleobase, nucleoside, nucleotide and nucleic acid metabolism | Nucleus | Transcription regulator activity | Nucleus |
| **MEPCE** | NP_062552.2 | 56257 | **7SK snRNA methylphosphate capping enzyme isoform A** | 11.8 | 2 | 4 | 4 | 5 | 1.16E+08 | **2.29** | 3 | 3 | 689 | 74.3 | 9.6 | Biological_process unknown | - | Molecular function unknown | - |
| **CUX1** | NP_001189475.1 | 1523 | **protein CASP isoform g** | 5.0 | 5 | 1 | 2 | 3 | 5.06E+07 | **2.29** | 1 | - | 639 | 72.8 | 5.5 | Regulation of nucleobase, nucleoside, nucleotide and nucleic acid metabolism | Nucleus;Golgi apparatus | Transcription regulator activity | Nucleus |
| **PNO1** | NP_064528.1 | 56902 | **RNA-binding protein PNO1** | 19.1 | 1 | 3 | 3 | 3 | 9.42E+07 | **2.29** | 2 | 371.5 | 252 | 27.9 | 9.7 | Biological_process unknown | - | Molecular function unknown | - |
| **MAP7D1** | NP_001273294.1 | 55700 | **MAP7 domain-containing protein 1 isoform 2** | 9.1 | 3 | 4 | 4 | 5 | 6.96E+07 | **2.29** | 1 | - | 803 | 88.7 | 10.1 | Biological_process unknown | - | Molecular function unknown | - |
| **NEDD4** | NP_006145.2 | 4734 | **E3 ubiquitin-protein ligase NEDD4 isoform 1** | 17.4 | 5 | 8 | 8 | 11 | 5.91E+07 | **2.29** | 3 | 45.2 | 900 | 104.2 | 6.0 | Protein metabolism | Cytoplasm;Plasma membrane;Nucleus | Ubiquitin-specific protease activity | Cytoplasm |
| **INPPL1** | NP_001558.3 | 3636 | **phosphatidylinositol 3,4,5-trisphosphate 5-phosphatase 2** | 9.0 | 1 | 7 | 7 | 8 | 2.31E+07 | **2.28** | 1 | - | 1258 | 138.5 | 6.5 | Cell communication;Signal transduction | Plasma membrane;Cytoplasm | Lipid phosphatase activity | Cytoplasm |
| **ZFAND1** | NP_001164268.1 | 79752 | **AN1-type zinc finger protein 1 isoform c** | 6.2 | 3 | 1 | 1 | 2 | 9.87E+07 | **2.28** | 1 | - | 227 | 26 | 8.5 | Biological_process unknown | - | Molecular function unknown | - |
| **AAMDC** | NP_078960.1 | 28971 | **mth938 domain-containing protein** | 32.0 | 1 | 2 | 2 | 4 | 1.46E+08 | **2.28** | 2 | 12.2 | 122 | 13.3 | 8.5 | Biological_process unknown | - | Molecular function unknown | - |
| **SAMD9** | NP_001180236.1 | 54809 | **sterile alpha motif domain-containing protein 9** | 13.5 | 1 | 12 | 12 | 17 | 2.08E+08 | **2.28** | 11 | 21.9 | 1589 | 184.2 | 7.8 | Biological_process unknown | - | Molecular function unknown | - |
| **FAM114A1** | NP_612398.2 | 92689 | **protein NOXP20** | 41.4 | 1 | 13 | 13 | 30 | 3.40E+08 | **2.28** | 11 | 46 | 563 | 60.7 | 4.7 | Biological_process unknown | Cytoplasm | Molecular function unknown | Cytoplasm |
| **CANT1** | NP_001153245.1 | 124583 | **soluble calcium-activated nucleotidase 1** | 6.0 | 1 | 1 | 1 | 1 | 1.41E+08 | **2.27** | 1 | - | 401 | 44.8 | 6.1 | Metabolism;Energy pathways | Extracellular;Plasma membrane;Endoplasmic reticulum;Golgi apparatus | Hydrolase activity | Endoplasmic reticulum |
| **TGOLN2** | NP_001193773.1 | 10618 | **trans-Golgi network integral membrane protein 2 isoform 4 precursor** | 21.9 | 4 | 5 | 5 | 8 | 4.23E+07 | **2.27** | 5 | 14.8 | 379 | 39.5 | 5.6 | Transport | Golgi apparatus;Plasma membrane;Endosome;Nucleus | Auxiliary transport protein activity | Golgi apparatus |
| **POFUT1** | NP_056167.1 | 23509 | **GDP-fucose protein O-fucosyltransferase 1 isoform 1 precursor** | 34.0 | 2 | 7 | 7 | 15 | 2.82E+08 | **2.26** | 5 | 28.3 | 388 | 43.9 | 8.5 | Metabolism | Golgi membrane | Transferase activity | Golgi membrane |
| **RHEB** | NP_005605.1 | 6009 | **GTP-binding protein Rheb** | 29.4 | 1 | 4 | 4 | 8 | 7.95E+07 | **2.26** | 2 | 5.8 | 184 | 20.5 | 5.9 | Cell communication;Signal transduction | Plasma membrane;Cytoplasmic vesicle | GTPase activity | Plasma membrane |
| **HIRA** | NP_003316.3 | 7290 | **protein HIRA** | 2.2 | 1 | 1 | 1 | 1 | 5.75E+07 | **2.25** | 1 | - | 1017 | 111.8 | 8.1 | Regulation of nucleobase, nucleoside, nucleotide and nucleic acid metabolism | Nucleus | Transcription regulator activity | Nucleus |
| **KDELC2** | NP_714916.3 | 143888 | **KDEL motif-containing protein 2 precursor** | 13.2 | 1 | 4 | 4 | 7 | 9.18E+07 | **2.25** | 5 | 17.1 | 507 | 58.5 | 8.2 | Biological_process unknown | Extracellular | Molecular function unknown | Extracellular |
| **CGGBP1** | NP_001008391.1 | 8545 | **CGG triplet repeat-binding protein 1** | 16.2 | 1 | 2 | 2 | 2 | 6.53E+07 | **2.25** | 2 | 62.6 | 167 | 18.8 | 9.0 | Regulation of nucleobase, nucleoside, nucleotide and nucleic acid metabolism | Nuclear matrix | DNA binding | Nuclear matrix |
| **AKR1B1** | NP_001619.1 | 231 | **aldose reductase** | 57.9 | 2 | 13 | 14 | 83 | 2.08E+09 | **2.24** | 14 | 13.4 | 316 | 35.8 | 7.0 | Metabolism;Energy pathways | Cytoplasm;Extracellular | Oxidoreductase activity | Cytoplasm |
| **MVP** | NP_059447.2 | 9961 | **major vault protein** | 50.7 | 1 | 35 | 35 | 144 | 1.03E+09 | **2.24** | 43 | 25.6 | 893 | 99.3 | 5.5 | Cell growth and/or maintenance | Cytoplasm;Nucleus | Nucleocytoplasmic transporter activity | Cytoplasm |
| **SNRPA** | NP_004587.1 | 6626 | **U1 small nuclear ribonucleoprotein A** | 18.8 | 1 | 3 | 5 | 23 | 6.05E+08 | **2.23** | 5 | 24.2 | 282 | 31.3 | 9.8 | Regulation of nucleobase, nucleoside, nucleotide and nucleic acid metabolism | Nucleus;Nucleolus | RNA binding | Nucleus |
| **CAP2** | NP_006357.1 | 10486 | **adenylyl cyclase-associated protein 2** | 17.4 | 1 | 4 | 5 | 12 | 3.74E+08 | **2.23** | 6 | 17.2 | 477 | 52.8 | 6.4 | Cell communication;Signal transduction | - | Molecular function unknown | - |
| **TGM2** | NP_004604.2 | 7052 | **protein-glutamine gamma-glutamyltransferase 2 isoform a** | 55.6 | 4 | 32 | 32 | 162 | 1.45E+09 | **2.23** | 42 | 27.7 | 687 | 77.3 | 5.2 | Metabolism;Energy pathways | Extracellular;Plasma membrane;Cytoplasm;Nucleus | Transaminase activity | Cytoplasm |
| **NCSTN** | NP_001277115.1 | 23385 | **nicastrin isoform 3 precursor** | 1.9 | 3 | 1 | 1 | 1 | 3.99E+07 | **2.22** | 1 | - | 571 | 63.3 | 5.8 | Cell communication;Signal transduction | Plasma membrane;Lysosome;Endoplasmic reticulum;Golgi apparatus | Protein binding | Plasma membrane |

| Gene Symbol | RefSeq_Accession | Entrez Gene ID | Description | Coverage (%) | Proteins | Unique Peptides | # Peptides | PSM | Area | SCC-R /SCC-S | SCC-R /SCC-S (Count) | SCC-R /SCC-S (Variability [%]) | # AAs | MW [kDa] | calc. pI | Biological Process | Localization | Molecular Function | Primary Localization |
|---|---|---|---|---|---|---|---|---|---|---|---|---|---|---|---|---|---|---|---|
| **NUDT16** | NP_689608.2 | 131870 | **U8 snoRNA-decapping enzyme isoform 2** | 20.0 | 3 | 2 | 2 | 5 | 5.67E+07 | 2.22 | 1 | - | 195 | 21.3 | 6.9 | Biological_process unknown | - | Molecular function unknown | - |
| **AGMAT** | NP_079034.3 | 79814 | **agmatinase, mitochondrial precursor** | 8.5 | 1 | 1 | 1 | 1 | 3.45E+07 | 2.22 | 1 | - | 352 | 37.6 | 7.6 | Metabolism;Energy pathways | Mitochondrion | Hydrolase activity | Mitochondrion |
| **SHC1** | NP_001123513.1 | 6464 | **SHC-transforming protein 1 isoform 4** | 27.1 | 5 | 7 | 7 | 10 | 8.28E+07 | 2.21 | 3 | 0.7 | 473 | 51.6 | 7.2 | Signal transduction;Cell communication | Plasma membrane;Cytoplasm;Endoplasmic reticulum membrane | Protein binding | Cytoplasm |
| **MIER1** | NP_001139585.1 | 57708 | **mesoderm induction early response protein 1 isoform i** | 10.0 | 9 | 2 | 2 | 2 | 6.98E+07 | 2.21 | 1 | - | 370 | 42.1 | 4.5 | Regulation of nucleobase, nucleoside, nucleotide and nucleic acid metabolism | Nucleus;Cytoplasm | Transcription regulator activity | Nucleus;Cytoplasm |
| **PON2** | NP_001018171.1 | 5445 | **serum paraoxonase/arylesterase 2 isoform 2** | 29.5 | 2 | 4 | 4 | 6 | 9.69E+07 | 2.21 | 1 | - | 342 | 38 | 5.7 | Metabolism;Energy pathways;Lipid metabolism | Plasma membrane | Hydrolase activity;Antioxidant activity | Plasma membrane |
| **STK10** | NP_005981.3 | 6793 | **serine/threonine-protein kinase 10** | 8.3 | 1 | 4 | 5 | 6 | 1.38E+08 | 2.21 | 2 | 8.3 | 968 | 112.1 | 7.0 | Cell communication;Signal transduction | - | Protein serine/threonine kinase activity | - |
| **IER3IP1** | NP_057181.1 | 51124 | **immediate early response 3-interacting protein 1 precursor** | 24.4 | 1 | 1 | 1 | 3 | 5.67E+07 | 2.20 | 1 | - | 82 | 9 | 8.2 | Biological_process unknown | Endoplasmic reticulum | Molecular function unknown | Endoplasmic reticulum |
| **ITPR3** | NP_002215.2 | 3710 | **inositol 1,4,5-trisphosphate receptor type 3** | 10.3 | 4 | 15 | 16 | 24 | 1.39E+08 | 2.20 | 8 | 18.5 | 2671 | 303.9 | 6.5 | Cell communication;Signal transduction | Endoplasmic reticulum;Plasma membrane;Integral to membrane | Intracellular ligand-gated ion channel activity | Plasma membrane |
| **ZYX** | NP_001010972.1 | 7791 | **zyxin** | 55.1 | 1 | 16 | 16 | 78 | 9.02E+08 | 2.20 | 20 | 18.8 | 572 | 61.2 | 6.7 | Signal transduction | Plasma membrane;Cytoplasm;Nucleus | Cell adhesion molecule activity | Plasma membrane;Cytoplasm |
| **KAT2A** | NP_066564.2 | 2648 | **histone acetyltransferase KAT2A** | 1.4 | 1 | 2 | 2 | 2 | 2.80E+08 | 2.20 | 2 | 435.8 | 837 | 93.9 | 9.0 | Regulation of nucleobase, nucleoside, nucleotide and nucleic acid metabolism | Nucleus | Transcription regulator activity | Nucleus |
| **PHLDB2** | NP_665696.1 | 90102 | **pleckstrin homology-like domain family B member 2 isoform c** | 5.0 | 3 | 3 | 3 | 5 | 3.97E+07 | 2.19 | 2 | 67.9 | 1210 | 136.8 | 7.5 | Cell communication;Signal transduction | Plasma membrane;Cytoplasmic vesicle;Cytoplasm | Cytoskeletal protein binding | Cytoplasm |
| **DFNA5** | NP_001120925.1 | 1687 | **non-syndromic hearing impairment protein 5 isoform a** | 23.4 | 2 | 7 | 7 | 9 | 1.12E+08 | 2.19 | 2 | 17.8 | 496 | 54.5 | 5.2 | Biological_process unknown | - | Molecular function unknown | - |
| **CMC1** | NP_872329.1 | 152100 | **COX assembly mitochondrial protein homolog** | 9.4 | 1 | 1 | 1 | 1 | 1.65E+08 | 2.18 | 1 | - | 106 | 12.5 | 8.6 | Biological_process unknown | - | Molecular function unknown | - |
| **LAMB1** | NP_002282.2 | 3912 | **laminin subunit beta-1 precursor** | 10.9 | 1 | 11 | 11 | 20 | 1.92E+08 | 2.18 | 6 | 41 | 1786 | 197.9 | 4.9 | Cell growth and/or maintenance | Extracellular | Extracellular matrix structural constituent | Extracellular |
| **APP** | NP_001129602.1 | 351 | **amyloid beta A4 protein isoform f precursor** | 16.4 | 10 | 6 | 6 | 12 | 8.73E+07 | 2.17 | 3 | 33.4 | 714 | 80.8 | 4.8 | Cell communication;Signal transduction | Nucleus;Integral to membrane;Cell surface;Vesicle;Endoplasmic reticulum;Golgi apparatus;Endosome;Cytoplasm;Extracellular;Plasma | Receptor activity | Cell surface;Plasma membrane |
| **TCEAL3** | NP_001006934.1 | 85012 | **transcription elongation factor A protein-like 3** | 20.0 | 3 | 2 | 2 | 5 | 6.68E+07 | 2.17 | 4 | 8 | 200 | 22.5 | 4.9 | Biological_process unknown | - | Molecular function unknown | - |
| **SRR** | NP_068766.1 | 63826 | **serine racemase** | 10.3 | 1 | 2 | 2 | 2 | 2.74E+07 | 2.17 | 2 | 62.9 | 340 | 36.5 | 6.5 | Metabolism;Energy pathways | Cytoplasm | Racemase and epimerase activity | Cytoplasm |
| **GNS** | NP_002067.1 | 2799 | **N-acetylglucosamine-6-sulfatase precursor** | 11.2 | 1 | 4 | 4 | 5 | 1.25E+08 | 2.17 | 2 | 21 | 552 | 62 | 8.3 | Metabolism;Energy pathways | Lysosome | Catalytic activity | Lysosome |
| **SRGAP2** | NP_001164108.1 | 23380 | **SLIT-ROBO Rho GTPase-activating protein 2 isoform c** | 3.6 | 4 | 2 | 3 | 4 | 4.56E+07 | 2.17 | 2 | 25.4 | 1070 | 120.7 | 6.7 | Cell communication;Signal transduction | Nucleus;Cytoplasm | GTPase activator activity | Nucleus;Cytoplasm |
| **APLP2** | NP_001135750.1 | 334 | **amyloid-like protein 2 isoform 4 precursor** | 9.8 | 5 | 3 | 3 | 3 | 2.01E+07 | 2.17 | 2 | 17 | 522 | 59.1 | 5.6 | Cell communication;Signal transduction | Plasma membrane | Protein binding | Plasma membrane |
| **SCRN1** | NP_055581.3 | 9805 | **secernin-1 isoform a** | 45.4 | 3 | 15 | 15 | 88 | 8.10E+08 | 2.16 | 12 | 34.7 | 414 | 46.4 | 4.8 | Immune response | Cytoplasm | Peptidase activity | Cytoplasm |
| **COX20** | NP_932342.1 | 116228 | **cytochrome c oxidase protein 20 homolog** | 22.9 | 1 | 3 | 3 | 4 | 3.94E+07 | 2.16 | 1 | - | 118 | 13.3 | 8.8 | Biological_process unknown | - | Molecular function unknown | - |
| **DPP9** | NP_631898.3 | 91039 | **dipeptidyl peptidase 9** | 12.0 | 1 | 7 | 7 | 12 | 1.68E+08 | 2.16 | 8 | 22.8 | 892 | 101.6 | 6.6 | Metabolism;Energy pathways | Cytoplasm | Aminopeptidase activity | Cytoplasm |
| **LRSAM1** | NP_001005373.1 | 90678 | **E3 ubiquitin-protein ligase LRSAM1 isoform 1** | 16.0 | 2 | 6 | 6 | 7 | 6.21E+07 | 2.16 | 3 | 16 | 723 | 83.5 | 5.9 | Cell communication;Signal transduction | Cytoplasm;Nucleus | Ubiquitin-specific protease activity | Cytoplasm |
| **NCK1** | NP_001177725.1 | 4690 | **cytoplasmic protein NCK1 isoform 2** | 10.5 | 2 | 2 | 2 | 4 | 6.04E+07 | 2.16 | 2 | 8.7 | 313 | 35.5 | 5.7 | Cell communication;Signal transduction | Cytoplasm;Nucleus;Plasma membrane | Receptor signaling complex scaffold activity | Cytoplasm |
| **VCPIP1** | NP_079330.2 | 80124 | **deubiquitinating protein VCIP135** | 9.9 | 1 | 7 | 7 | 7 | 3.36E+08 | 2.16 | 2 | 15.9 | 1222 | 134.2 | 7.2 | Protein metabolism | Golgi apparatus;Endoplasmic reticulum;Cytoplasm | Ubiquitin-specific protease activity | Cytoplasm |
| **SPAG9** | NP_001124000.1 | 9043 | **C-Jun-amino-terminal kinase-interacting protein 4 isoform 1** | 24.2 | 6 | 19 | 19 | 39 | 3.07E+08 | 2.16 | 17 | 25.7 | 1321 | 146.1 | 5.2 | Cell growth and/or maintenance | Cytoplasm;Plasma membrane;Nucleus | Molecular function unknown | Cytoplasm;Plasma membrane |
| **MRPL20** | NP_060441.2 | 55052 | **39S ribosomal protein L20, mitochondrial** | 7.4 | 1 | 1 | 1 | 1 | 1.63E+08 | 2.15 | 1 | - | 149 | 17.4 | 10.9 | Protein metabolism | Mitochondrion | Structural constituent of ribosome | Mitochondrion |
| **ATXN2L** | NP_009176.2 | 11273 | **ataxin-2-like protein isoform A** | 28.1 | 6 | 14 | 14 | 28 | 5.48E+08 | 2.15 | 10 | 40.5 | 1075 | 113.3 | 8.6 | Biological_process unknown | Nucleus;Cytosol;Cytoplasm | Molecular function unknown | Nucleus;Cytoplasm |
| **ZPR1** | NP_003895.1 | 8882 | **zinc finger protein ZPR1** | 17.7 | 1 | 3 | 3 | 4 | 8.58E+07 | 2.15 | 2 | 39.4 | 459 | 50.9 | 4.7 | Cell communication;Signal transduction | Cytoplasm;Nucleolus | Receptor signaling complex scaffold activity | Cytoplasm |
| **C19orf10** | NP_061980.1 | 56005 | **UPF0556 protein C19orf10 precursor** | 24.9 | 1 | 4 | 4 | 7 | 1.69E+08 | 2.15 | 3 | 76.6 | 173 | 18.8 | 6.7 | Cell communication;Signal transduction | - | Growth factor activity | - |
| **ACADM** | NP_000007.1 | 34 | **medium-chain specific acyl-CoA dehydrogenase, mitochondrial isoform a precursor** | 47.3 | 5 | 16 | 16 | 41 | 6.31E+08 | 2.15 | 14 | 17.4 | 421 | 46.6 | 8.4 | Metabolism;Energy pathways | Mitochondrion | Catalytic activity | Mitochondrion |
| **GLRX** | NP_001230587.1 | 2745 | **glutaredoxin-1** | 36.8 | 1 | 2 | 2 | 3 | 4.30E+07 | 2.14 | 2 | 27.4 | 106 | 11.8 | 8.1 | Metabolism;Energy pathways | Cytoplasm | Oxidoreductase activity | Cytoplasm |
| **CTU2** | NP_001012780.1 | 348180 | **cytoplasmic tRNA 2-thiolation protein 2 isoform 2** | 5.4 | 2 | 2 | 2 | 2 | 3.72E+07 | 2.14 | 2 | 28.6 | 485 | 52.4 | 6.9 | Biological_process unknown | Cytoplasm | Molecular function unknown | Cytoplasm |
| **TLN2** | NP_055874.2 | 83660 | **talin-2** | 14.2 | 1 | 14 | 21 | 43 | 6.12E+08 | 2.14 | 2 | 12663.8 | 2542 | 271.4 | 5.6 | Cell growth and/or maintenance | Plasma membrane;Nucleus | Cytoskeletal protein binding | Plasma membrane |
| **CDKN2B** | NP_004927.2 | 1030 | **cyclin-dependent kinase 4 inhibitor B isoform 1** | 8.7 | 3 | 1 | 1 | 1 | 5.53E+07 | 2.14 | 1 | - | 138 | 14.7 | 6.5 | Cell communication;Signal transduction | Cytoplasm | Kinase regulator activity | Cytoplasm |
| **MIEN1** | NP_115715.3 | 84299 | **migration and invasion enhancer 1** | 20.0 | 1 | 1 | 1 | 1 | 8.37E+07 | 2.13 | 1 | - | 115 | 12.4 | 4.4 | Biological_process unknown | - | Molecular function unknown | - |

| Gene Symbol | RefSeq_Accession | Entrez Gene ID | Description | Coverage (%) | Proteins | Unique Peptides | # Peptides | PSM | Area | SCC-R /SCC-S | SCC-R /SCC-S (Count) | SCC-R /SCC-S (Variability [%]) | # AAs | MW [kDa] | calc. pI | Biological Process | Localization | Molecular Function | Primary Localization |
|---|---|---|---|---|---|---|---|---|---|---|---|---|---|---|---|---|---|---|---|
| CCBL2 | NP_001008662.1 | 56267 | kynurenine--oxoglutarate transaminase 3 isoform 2 | 17.6 | 2 | 4 | 4 | 12 | 1.12E+08 | 2.13 | 3 | 63.7 | 420 | 47.7 | 6.8 | Metabolism;Energy pathways | - | Transaminase activity | - |
| ABCC1 | NP_004987.2 | 4363 | multidrug resistance-associated protein 1 | 4.6 | 1 | 5 | 5 | 7 | 7.91E+07 | 2.13 | 3 | 2.8 | 1531 | 171.5 | 7.1 | Transport | Plasma membrane;Lysosome;Cytoplasm | Transporter activity | Plasma membrane |
| FAM45A | NP_996892.1 | 404636 | protein FAM45A | 18.5 | 1 | 3 | 3 | 5 | 2.94E+07 | 2.13 | 3 | 411.2 | 357 | 40.5 | 6.6 | Biological_process unknown | - | Molecular function unknown | - |
| BASP1 | NP_006308.3 | 10409 | brain acid soluble protein 1 | 71.8 | 1 | 14 | 14 | 86 | 2.74E+09 | 2.12 | 25 | 11.5 | 227 | 22.7 | 4.6 | Regulation of nucleobase, nucleoside, nucleotide and nucleic acid metabolism | Nucleus;Plasma membrane;Cytoplasm | Transcription regulator activity | Nucleus |
| WBP2 | NP_036610.2 | 23558 | WW domain-binding protein 2 | 11.5 | 1 | 3 | 3 | 3 | 1.26E+07 | 2.12 | 2 | 4.1 | 261 | 28.1 | 5.9 | Cell communication;Signal transduction | - | Receptor binding | - |
| TMEM55B | NP_653169.2 | 90809 | type 1 phosphatidylinositol 4,5-bisphosphate 4-phosphatase isoform 2 | 15.5 | 3 | 3 | 3 | 6 | 8.67E+07 | 2.12 | 3 | 89.5 | 277 | 29.4 | 8.9 | Biological_process unknown | Integral to membrane;Apical membrane | Molecular function unknown | Integral to membrane |
| UBA5 | NP_938143.1 | 79876 | ubiquitin-like modifier-activating enzyme 5 isoform 2 | 25.6 | 2 | 4 | 4 | 9 | 2.08E+08 | 2.12 | 2 | 68.5 | 348 | 38.5 | 4.8 | Protein metabolism | Integral to membrane | Protein binding | Integral to membrane |
| ITFG3 | NP_114428.1 | 83986 | protein ITFG3 | 2.4 | 1 | 1 | 1 | 2 | 3.22E+07 | 2.11 | 1 | - | 552 | 59.6 | 6.3 | Biological_process unknown | - | Cell adhesion molecule activity | - |
| UBE2Z | NP_075567.2 | 65264 | ubiquitin-conjugating enzyme E2 Z | 17.0 | 1 | 5 | 5 | 12 | 1.26E+08 | 2.11 | 3 | 34.4 | 354 | 38.2 | 5.6 | Protein metabolism | - | Ubiquitin-specific protease activity | - |
| HSPB11 | NP_057210.2 | 51668 | intraflagellar transport protein 25 homolog | 36.1 | 1 | 4 | 4 | 11 | 1.83E+08 | 2.11 | 4 | 8.1 | 144 | 16.3 | 5.0 | Biological_process unknown | Extracellular | Molecular function unknown | Extracellular |
| UBP1 | NP_001121632.1 | 7342 | upstream-binding protein 1 isoform LBP-1a | 10.3 | 2 | 2 | 3 | 5 | 1.03E+08 | 2.11 | 2 | 3.3 | 504 | 56.4 | 6.3 | Regulation of nucleobase, nucleoside, nucleotide and nucleic acid metabolism | Nucleus | Transcription factor activity | Nucleus |
| CRYBG3 | NP_705833.3 | 131544 | beta/gamma crystallin domain-containing protein 3 | 2.8 | 1 | 4 | 4 | 4 | 5.29E+07 | 2.10 | 4 | 36.2 | 2970 | 330.4 | 5.2 | Biological_process unknown | - | Molecular function unknown | - |
| QSOX2 | NP_859052.3 | 169714 | sulfhydryl oxidase 2 precursor | 7.6 | 1 | 4 | 4 | 7 | 5.22E+07 | 2.10 | 5 | 9.6 | 698 | 77.5 | 7.7 | Apoptosis | Plasma membrane;Nucleus;Cytoplasm | Molecular function unknown | Plasma membrane |
| LIN37 | NP_061977.1 | 55957 | protein lin-37 homolog | 5.3 | 1 | 1 | 1 | 1 | 9.87E+06 | 2.10 | 1 | - | 246 | 28.4 | 8.7 | Biological_process unknown | - | Molecular function unknown | - |
| CORO7-PAM16 | NP_001188408.1 | 100529144 | CORO7-PAM16 protein | 5.6 | 5 | 3 | 3 | 10 | 1.18E+08 | 2.10 | 5 | 43.6 | 1048 | 114.1 | 6.3 | - | - | - | - |
| STK4 | NP_006273.1 | 6789 | serine/threonine-protein kinase 4 | 14.2 | 1 | 4 | 5 | 7 | 1.24E+08 | 2.09 | 4 | 27.8 | 487 | 55.6 | 5.1 | Cell communication;Signal transduction | Cytoplasm;Nucleus;Cytosol;Perinuclear region | Protein serine/threonine kinase activity | Cytoplasm |
| STX4 | NP_001259024.1 | 6810 | syntaxin-4 isoform 1 | 25.1 | 3 | 2 | 2 | 8 | 2.99E+08 | 2.09 | 4 | 4.8 | 219 | 25.2 | 7.0 | Cell communication;Signal transduction | Plasma membrane | Auxiliary transport protein activity | Plasma membrane |
| PPDPF | NP_077275.1 | 79144 | pancreatic progenitor cell differentiation and proliferation factor | 29.0 | 1 | 2 | 2 | 2 | 3.19E+07 | 2.08 | 1 | - | 114 | 11.8 | 7.4 | Biological_process unknown | - | Molecular function unknown | - |
| CMAS | NP_061156.1 | 55907 | N-acylneuraminate cytidylyltransferase | 30.7 | 1 | 9 | 9 | 22 | 2.04E+08 | 2.08 | 6 | 36.7 | 434 | 48.3 | 7.9 | Metabolism;Energy pathways | Nucleus;Cytoplasm | Ligase activity | Nucleus |
| CRYZ | NP_001123515.1 | 1429 | quinone oxidoreductase isoform b | 19.0 | 3 | 3 | 3 | 11 | 5.18E+07 | 2.08 | 3 | 0.4 | 295 | 31.5 | 8.5 | Metabolism;Energy pathways | Cytoplasm;Mitochondrion | Oxidoreductase activity | Cytoplasm |
| PPP1R9B | NP_115984.3 | 84687 | neurabin-2 | 16.9 | 1 | 7 | 7 | 11 | 1.33E+08 | 2.08 | 5 | 25.7 | 817 | 89.3 | 5.0 | Cell communication;Signal transduction | Plasma membrane;Cytoplasm;Nucleus | Phosphatase regulator activity | Cytoplasm |
| TBCC | NP_003183.1 | 6903 | tubulin-specific chaperone C | 9.0 | 1 | 3 | 3 | 5 | 1.19E+08 | 2.08 | 3 | 707.7 | 346 | 39.2 | 5.7 | Protein metabolism | Cytoplasm | Chaperone activity | Cytoplasm |
| TNFAIP2 | NP_006282.2 | 7127 | tumor necrosis factor alpha-induced protein 2 | 31.8 | 1 | 12 | 12 | 25 | 2.30E+08 | 2.08 | 10 | 25.4 | 654 | 72.6 | 6.5 | Biological_process unknown | Extracellular | Molecular function unknown | Extracellular |
| AASDHPPT | NP_056238.2 | 60496 | L-aminoadipate-semialdehyde dehydrogenase-phosphopantetheinyl transferase | 46.0 | 1 | 11 | 11 | 56 | 2.83E+08 | 2.08 | 16 | 19.1 | 309 | 35.8 | 6.8 | Metabolism;Energy pathways | Cytoplasm | Catalytic activity | Cytoplasm |
| LARP7 | NP_057732.2 | 51574 | la-related protein 7 isoform 1 | 6.7 | 2 | 2 | 2 | 4 | 1.17E+08 | 2.07 | 2 | 54.3 | 582 | 66.9 | 9.6 | Biological_process unknown | - | Molecular function unknown | - |
| YAP1 | NP_001123617.1 | 10413 | yorkie homolog isoform 1 | 65.7 | 9 | 20 | 20 | 76 | 9.89E+08 | 2.07 | 24 | 30.2 | 504 | 54.4 | 5.2 | Regulation of nucleobase, nucleoside, nucleotide and nucleic acid metabolism | Cytoplasm;Nucleus;Plasma membrane | Transcription regulator activity | Nucleus |
| DYNC1LI1 | NP_057225.2 | 51143 | cytoplasmic dynein 1 light intermediate chain 1 | 35.2 | 1 | 13 | 14 | 37 | 3.39E+08 | 2.07 | 9 | 9.6 | 523 | 56.5 | 6.4 | Cell growth and/or maintenance | Cytoplasm;Cytoskeleton;Endosome | Motor activity | Cytoskeleton |
| TAGLN2 | NP_001264152.1 | 8407 | transgelin-2 isoform b | 86.4 | 3 | 20 | 20 | 139 | 4.94E+09 | 2.07 | 40 | 16.1 | 199 | 22.4 | 8.3 | Biological_process unknown | - | Molecular function unknown | - |
| MST4 | NP_057626.2 | 51765 | serine/threonine-protein kinase MST4 isoform 1 | 31.3 | 3 | 7 | 9 | 34 | 9.57E+08 | 2.07 | 8 | 24.9 | 416 | 46.5 | 5.3 | Cell communication;Signal transduction | Cytoplasm;Nucleus;Golgi apparatus | Protein serine/threonine kinase activity | Golgi apparatus |
| MEN1 | NP_570711.1 | 4221 | menin isoform 2 | 4.8 | 2 | 1 | 1 | 1 | 2.67E+08 | 2.07 | 1 | - | 610 | 67.4 | 6.6 | Regulation of gene expression, epigenetic | Nucleus;Plasma membrane;Cytoplasm;Chromosome;Nuclear matrix | Transcription regulator activity | Nucleus |
| MLF2 | NP_005430.1 | 8079 | myeloid leukemia factor 2 | 18.2 | 1 | 2 | 2 | 6 | 1.08E+08 | 2.06 | 1 | - | 248 | 28.1 | 6.9 | Biological_process unknown | Nucleus | Molecular function unknown | Nucleus |
| TMSB10 | NP_066926.1 | 9168 | thymosin beta-10 | 81.8 | 1 | 5 | 5 | 176 | 1.36E+10 | 2.06 | 27 | 8.3 | 44 | 5 | 5.4 | Cell growth and/or maintenance | Nucleus;Cytoplasm | Cytoskeletal protein binding | Cytoplasm |
| MAT2B | NP_877725.1 | 27430 | methionine adenosyltransferase 2 subunit beta isoform 2 | 22.0 | 2 | 4 | 4 | 8 | 9.13E+07 | 2.06 | 2 | 65.1 | 323 | 36.4 | 6.7 | Metabolism | Cytoplasm | Catalytic activity | Cytoplasm |
| SLC26A11 | NP_001159820.1 | 284129 | sodium-independent sulfate anion transporter | 5.1 | 1 | 1 | 1 | 1 | 4.35E+07 | 2.06 | 1 | - | 606 | 65.3 | 7.3 | Transport | Plasma membrane | Transporter activity | Plasma membrane |
| TSPO | NP_001243459.1 | 706 | translocator protein | 29.0 | 1 | 2 | 2 | 5 | 3.32E+07 | 2.06 | 2 | 9 | 169 | 18.8 | 9.4 | Cell communication;Signal transduction | Mitochondrion | Receptor activity | Mitochondrion |
| TOLLIP | NP_061882.2 | 54472 | toll-interacting protein | 5.1 | 1 | 1 | 1 | 4 | 5.80E+08 | 2.06 | 2 | 9.4 | 274 | 30.3 | 6.0 | Cell communication;Signal transduction | Cytoplasm;Plasma membrane | Receptor signaling complex scaffold activity | Cytoplasm |
| TBC1D5 | NP_055559.1 | 9779 | TBC1 domain family member 5 isoform b | 16.2 | 2 | 7 | 7 | 11 | 1.42E+08 | 2.06 | 5 | 32.6 | 795 | 88.9 | 6.5 | Biological_process unknown | - | Molecular function unknown | - |

| Gene Symbol | RefSeq_Accession | Entrez Gene ID | Description | Coverage (%) | Proteins | Unique Peptides | # Peptides | PSM | Area | SCC-R /SCC-S | SCC-R /SCC-S (Count) | SCC-R /SCC-S (Variability [%]) | # AAs | MW [kDa] | calc. pI | Biological Process | Localization | Molecular Function | Primary Localization |
|---|---|---|---|---|---|---|---|---|---|---|---|---|---|---|---|---|---|---|---|
| LPCAT4 | NP_705841.2 | 254531 | lysophospholipid acyltransferase LPCAT4 | 6.1 | 1 | 1 | 1 | 1 | 4.95E+07 | 2.05 | 1 | - | 524 | 57.2 | 9.0 | Metabolism;Energy pathways | Endoplasmic reticulum;Perinuclear region | Acyltransferase activity | Endoplasmic reticulum |
| PAIP1 | NP_006442.2 | 10605 | polyadenylate-binding protein-interacting protein 1 isoform 1 | 26.3 | 2 | 2 | 6 | 18 | 2.24E+08 | 2.05 | 2 | 3.9 | 479 | 53.5 | 4.8 | Protein metabolism;Regulation of translation | Cytoplasm;Nucleus | Translation regulator activity;Protein binding | Cytoplasm |
| CTTN | NP_005222.2 | 2017 | src substrate cortactin isoform a | 47.1 | 3 | 29 | 29 | 118 | 2.63E+09 | 2.05 | 28 | 28.6 | 550 | 61.5 | 5.4 | Cell growth and/or maintenance | Cytoplasm;Cytoskeleton;Plasma membrane;Clathrin-coated vesicle | Cytoskeletal protein binding | Cytoplasm |
| TCOF1 | NP_001128716.1 | 6949 | treacle protein isoform e | 19.1 | 3 | 1 | 20 | 51 | 3.87E+08 | 2.05 | 2 | 1.2 | 1451 | 148.3 | 8.9 | Transcription;Cell migration;Ribosome biogenesis and assembly | Nucleoplasm;Nucleolus;Cytoplasm;Nucleus | Transcription regulator activity | Nucleolus |
| ATP6V1B2 | NP_001684.2 | 526 | V-type proton ATPase subunit B, brain isoform | 50.9 | 2 | 16 | 16 | 46 | 5.99E+08 | 2.05 | 18 | 26 | 511 | 56.5 | 5.8 | Transport | Endosome | Transporter activity | Endosome |
| SNX4 | NP_003785.1 | 8723 | sorting nexin-4 | 5.8 | 1 | 2 | 2 | 2 | 8.18E+07 | 2.05 | 2 | 35.9 | 450 | 51.9 | 6.0 | Transport | Integral to membrane;Cytoplasm;Endosome | Transporter activity | Integral to membrane |
| CPT1A | NP_001867.2 | 1374 | carnitine O-palmitoyltransferase 1, liver isoform isoform 1 | 13.5 | 2 | 7 | 7 | 19 | 1.22E+08 | 2.04 | 6 | 17.3 | 773 | 88.3 | 8.7 | Mitochondrial transport | Mitochondrial membrane;Mitochondrion | Palmitoyltransferase activity | Mitochondrial membrane |
| TRIP6 | NP_003293.2 | 7205 | thyroid receptor-interacting protein 6 | 38.0 | 1 | 9 | 9 | 25 | 9.23E+08 | 2.04 | 9 | 38.6 | 476 | 50.3 | 7.4 | Regulation of nucleobase, nucleoside, nucleotide and nucleic acid metabolism | Nucleus;Cytoplasm;Plasma membrane | Transcription regulator activity | Nucleus |
| CCNK | NP_001092872.1 | 8812 | cyclin-K | 2.8 | 1 | 1 | 1 | 1 | 1.88E+07 | 2.04 | 1 | - | 580 | 64.2 | 8.4 | Regulation of nucleobase, nucleoside, nucleotide and nucleic acid metabolism;Regulation of gene expression, epigenetic;Regulation of cell | Nucleus | Transcription regulator activity | Nucleus |
| RB1CC1 | NP_001077086.1 | 9821 | RB1-inducible coiled-coil protein 1 isoform 2 | 2.5 | 2 | 2 | 2 | 4 | 4.39E+07 | 2.04 | 1 | - | 1591 | 182.7 | 5.4 | Regulation of nucleobase, nucleoside, nucleotide and nucleic acid metabolism | Nucleus;Cytoplasm | Transcription factor activity | Nucleus |
| LRP8 | NP_059992.3 | 7804 | low-density lipoprotein receptor-related protein 8 isoform 3 precursor | 2.1 | 4 | 1 | 1 | 2 | 5.77E+07 | 2.04 | 2 | 28.3 | 700 | 77.8 | 5.0 | Cell communication;Signal transduction | Caveola;Soluble fraction | Lipoprotein receptor activity | Caveola |
| CTTNBP2NL | NP_061174.1 | 55917 | CTTNBP2 N-terminal-like protein | 12.5 | 1 | 4 | 4 | 6 | 1.30E+08 | 2.04 | 2 | 22.9 | 639 | 70.1 | 8.1 | Biological_process unknown | - | Molecular function unknown | - |
| CCBL1 | NP_001116144.1 | 883 | kynurenine--oxoglutarate transaminase 1 isoform b | 3.8 | 3 | 1 | 1 | 1 | 7.16E+06 | 2.03 | 1 | - | 372 | 42.5 | 7.2 | Metabolism;Energy pathways | Cytoplasm;Mitochondrion;Endoplasmic reticulum | Lyase activity | Cytoplasm |
| EIF1B | NP_005866.1 | 10289 | eukaryotic translation initiation factor 1b | 82.3 | 1 | 2 | 7 | 31 | 6.43E+08 | 2.03 | 2 | 26.8 | 113 | 12.8 | 7.4 | Protein metabolism | Nucleus | Translation regulator activity | Nucleus |
| EMC10 | NP_778233.4 | 284361 | ER membrane protein complex subunit 10 isoform 1 precursor | 6.7 | 2 | 1 | 1 | 1 | 2.23E+07 | 2.03 | 1 | - | 254 | 26.8 | 6.3 | Biological_process unknown | - | Molecular function unknown | - |
| RELA | NP_001230914.1 | 5970 | transcription factor p65 isoform 4 | 21.7 | 4 | 9 | 9 | 19 | 3.78E+08 | 2.02 | 9 | 48 | 448 | 49.5 | 7.4 | Regulation of nucleobase, nucleoside, nucleotide and nucleic acid metabolism | Cytoplasm;Nucleus | Transcription factor activity | Nucleus |
| PTPRE | NP_569119.1 | 5791 | receptor-type tyrosine-protein phosphatase epsilon isoform 2 | 7.2 | 2 | 2 | 2 | 2 | 8.53E+06 | 2.02 | 1 | - | 642 | 74.5 | 7.5 | Cell communication;Signal transduction | Plasma membrane;Cytoplasm;Nucleus | Receptor signaling protein tyrosine phosphatase activity | Plasma membrane |
| STXBP3 | NP_009200.2 | 6814 | syntaxin-binding protein 3 | 16.1 | 1 | 7 | 7 | 10 | 8.27E+07 | 2.02 | 4 | 17.4 | 592 | 67.7 | 7.8 | Transport | Cytoplasmic vesicle;Cytoplasm | Transporter activity | Cytoplasmic vesicle |
| CAMK2D | NP_742113.1 | 817 | calcium/calmodulin-dependent protein kinase type II subunit delta isoform 1 | 26.6 | 7 | 6 | 7 | 18 | 1.18E+08 | 2.02 | 2 | 39.8 | 478 | 54.1 | 7.3 | Regulation of cell growth | Cytoplasm;Nucleus | Protein serine/threonine kinase activity | Cytoplasm |
| ATP6V1E1 | NP_001687.1 | 529 | V-type proton ATPase subunit E 1 isoform a | 47.8 | 4 | 7 | 7 | 19 | 3.05E+08 | 2.02 | 5 | 41.6 | 226 | 26.1 | 8.0 | Transport | Plasma membrane;Endosome;Lysosome;Golgi apparatus;Endoplasmic reticulum;Cytoplasm | ATPase activity | Lysosome |
| SFXN3 | NP_112233.2 | 81855 | sideroflexin-3 | 20.3 | 1 | 3 | 5 | 21 | 1.91E+08 | 2.01 | 1 | - | 325 | 36 | 9.1 | Biological_process unknown | Integral to membrane | Molecular function unknown | Integral to membrane |
| TSC2 | NP_001070651.1 | 7249 | tuberin isoform 4 | 1.7 | 3 | 2 | 2 | 4 | 2.32E+07 | 2.01 | 2 | 32 | 1740 | 193.1 | 7.1 | Cell communication;Signal transduction | Plasma membrane;Cytoplasm;Golgi apparatus;Nucleus;Perinuclear region | GTPase activator activity | Nucleus |
| SNX6 | NP_689419.2 | 58533 | sorting nexin-6 isoform b | 26.3 | 2 | 11 | 11 | 41 | 5.65E+08 | 2.01 | 10 | 24.8 | 418 | 47.8 | 6.4 | Cell communication;Signal transduction | Cytoplasm;Nucleus;Golgi apparatus;Plasma membrane | Transporter activity | Cytoplasm |
| MGAT4B | NP_055090.1 | 11282 | alpha-1,3-mannosyl-glycoprotein 4-beta-N-acetylglucosaminyltransferase B isoform 1 precursor | 4.9 | 2 | 1 | 1 | 1 | 6.63E+07 | 2.01 | 1 | - | 548 | 63.2 | 7.9 | Glycoprotein metabolism | - | Catalase activity | - |
| CALD1 | NP_149131.1 | 800 | caldesmon isoform 5 | 45.5 | 4 | 3 | 21 | 52 | 4.21E+08 | 2.01 | 1 | - | 532 | 61.2 | 6.7 | Cell growth and/or maintenance | Cytoplasm;Plasma membrane;Nucleus | Cytoskeletal protein binding | Cytoplasm;Nucleus |
| RUFY1 | NP_079434.3 | 80230 | RUN and FYVE domain-containing protein 1 isoform a | 33.1 | 2 | 12 | 12 | 29 | 4.04E+08 | 2.01 | 13 | 37.7 | 708 | 79.8 | 5.7 | Cell communication;Signal transduction | Endosome;Plasma membrane | Ubiquitin-specific protease activity | Endosome |
| PLCG1 | NP_877963.1 | 5335 | 1-phosphatidylinositol 4,5-bisphosphate phosphodiesterase gamma-1 isoform b | 9.3 | 2 | 5 | 5 | 5 | 5.56E+07 | 2.00 | 2 | 15.5 | 1290 | 148.4 | 6.1 | Metabolism | Nucleus;Cytoplasm | Phospholipase activity | Cytoplasm |
| SNCA | NP_009292.1 | 6622 | alpha-synuclein isoform NACP112 | 54.5 | 3 | 6 | 6 | 6 | 2.99E+07 | 2.00 | 1 | - | 112 | 11.4 | 8.3 | Protein metabolism | Cytoplasm;Nucleus | Chaperone activity | Cytoplasm |
| AKT1S1 | NP_001265089.1 | 84335 | proline-rich AKT1 substrate 1 isoform b | 17.6 | 2 | 3 | 3 | 5 | 8.70E+07 | 2.00 | 3 | 80.7 | 256 | 27.4 | 4.8 | Biological_process unknown | Cytoplasm | Molecular function unknown | Cytoplasm |
| DYNLRB1 | NP_054902.1 | 83658 | dynein light chain roadblock-type 1 isoform a | 83.3 | 4 | 5 | 5 | 23 | 1.39E+09 | 2.00 | 4 | 24.3 | 96 | 10.9 | 7.3 | Biological_process unknown | - | Transporter activity;Molecular function unknown | - |
| IMPA1 | NP_005527.1 | 3612 | inositol monophosphatase 1 isoform 1 | 28.2 | 3 | 5 | 5 | 13 | 2.73E+08 | 2.00 | 3 | 3.6 | 277 | 30.2 | 5.3 | Metabolism;Energy pathways | Cytoplasm | Phosphoprotein phosphatase activity | Cytoplasm |
| PRKAB1 | NP_006244.2 | 5564 | 5'-AMP-activated protein kinase subunit beta-1 | 23.0 | 1 | 3 | 3 | 5 | 1.93E+07 | 1.98 | 3 | 50.3 | 270 | 30.4 | 6.4 | Cell communication;Signal transduction | Cytoplasm | Protein binding | Cytoplasm |
| ROCK1 | NP_005397.1 | 6093 | rho-associated protein kinase 1 | 6.5 | 1 | 5 | 6 | 7 | 1.06E+08 | 1.98 | 4 | 27.5 | 1354 | 158.1 | 5.9 | Cell communication;Signal transduction | Cytoplasm;Extracellular;Golgi apparatus | Protein serine/threonine kinase activity | Cytoplasm |
| TBC1D15 | NP_001139685.2 | 64786 | TBC1 domain family member 15 isoform 3 | 11.1 | 3 | 5 | 5 | 10 | 1.42E+08 | 1.98 | 4 | 8.9 | 674 | 77.3 | 5.4 | Cell communication;Signal transduction | - | GTPase activator activity | - |
| PHPT1 | NP_054891.2 | 29085 | 14 kDa phosphohistidine phosphatase isoform 3 | 58.4 | 4 | 5 | 5 | 31 | 4.29E+08 | 1.98 | 9 | 6.5 | 125 | 13.8 | 6.1 | Biological_process unknown | Cytoplasm | Phosphoprotein phosphatase activity | Cytoplasm |
| RWDD4 | NP_689895.2 | 201965 | RWD domain-containing protein 4 | 7.5 | 1 | 1 | 1 | 1 | 3.14E+06 | 1.98 | 1 | - | 188 | 21.2 | 5.3 | Biological_process unknown | - | Molecular function unknown | - |
| UTRN | NP_009055.2 | 7402 | utrophin | 2.7 | 1 | 4 | 4 | 5 | 9.47E+07 | 1.98 | 2 | 67.8 | 3433 | 394.2 | 5.3 | Cell growth and/or maintenance | Cytoplasm;Plasma membrane;Nucleus | Cytoskeletal anchoring activity | Cytoplasm |

| Gene Symbol | RefSeq_Accession | Entrez Gene ID | Description | Coverage (%) | Proteins | Unique Peptides | # Peptides | PSM | Area | SCC-R /SCC-S | SCC-R /SCC-S (Count) | SCC-R /SCC-S (Variability [%]) | # AAs | MW [kDa] | calc. pI | Biological Process | Localization | Molecular Function | Primary Localization |
|---|---|---|---|---|---|---|---|---|---|---|---|---|---|---|---|---|---|---|---|
| RXRA | NP_002948.1 | 6256 | retinoic acid receptor RXR-alpha isoform a | 9.3 | 3 | 2 | 2 | 2 | 1.67E+07 | 1.97 | 1 | - | 462 | 50.8 | 7.8 | Cell communication;Signal transduction | Nucleus | Ligand-dependent nuclear receptor activity | Nucleus |
| DR1 | NP_001929.1 | 1810 | protein Dr1 | 49.4 | 1 | 6 | 6 | 18 | 3.85E+08 | 1.97 | 7 | 37 | 176 | 19.4 | 4.8 | Regulation of nucleobase, nucleoside, nucleotide and nucleic acid metabolism | Nucleus | Transcription regulator activity | Nucleus |
| PNP | NP_000261.2 | 4860 | purine nucleoside phosphorylase | 72.7 | 1 | 17 | 17 | 54 | 4.97E+08 | 1.97 | 23 | 41.1 | 289 | 32.1 | 7.0 | Metabolism;Energy pathways | Cytoplasm | Phosphorylase activity | Cytoplasm |
| ZNFX1 | NP_066363.1 | 57169 | NFX1-type zinc finger-containing protein 1 | 1.8 | 1 | 2 | 2 | 3 | 5.58E+06 | 1.96 | 1 | - | 1918 | 220.1 | 7.3 | Regulation of nucleobase, nucleoside, nucleotide and nucleic acid metabolism | Nucleus | Transcription regulator activity | Nucleus |
| CASP2 | NP_116764.2 | 835 | caspase-2 isoform 1 preproprotein | 6.4 | 3 | 2 | 2 | 5 | 8.47E+07 | 1.96 | 2 | 184.1 | 452 | 50.7 | 6.8 | Apoptosis | Cytoplasm;Nucleus;Golgi apparatus;Mitochondrion | Cysteine-type peptidase activity | Cytoplasm |
| MFF | NP_001263991.1 | 56947 | mitochondrial fission factor isoform b | 22.7 | 7 | 3 | 3 | 5 | 1.35E+08 | 1.96 | 1 | - | 291 | 33 | 7.4 | Biological_process unknown | Integral to membrane;Mitochondrion | Molecular function unknown | Integral to membrane |
| P2RX4 | NP_001248327.1 | 5025 | P2X purinoceptor 4 isoform 4 | 6.2 | 3 | 1 | 1 | 1 | 2.53E+07 | 1.96 | 1 | - | 341 | 37.9 | 7.7 | Signal transduction | Plasma membrane | Extracellular ligand-gated ion channel activity | Plasma membrane |
| DNAJC13 | NP_056083.3 | 23317 | dnaJ homolog subfamily C member 13 | 14.0 | 1 | 20 | 20 | 40 | 2.63E+08 | 1.96 | 13 | 24.6 | 2243 | 254.3 | 6.7 | Protein metabolism;Biological_process unknown | Endosome | Chaperone activity;Molecular function unknown | Endosome |
| OXSR1 | NP_005100.1 | 9943 | serine/threonine-protein kinase OSR1 | 34.9 | 1 | 8 | 10 | 20 | 4.49E+08 | 1.96 | 6 | 15.3 | 527 | 58 | 6.4 | Cell communication;Signal transduction | Cytoplasm;Nucleus;Integral to membrane | Protein serine/threonine kinase activity | Cytoplasm |
| NAPG | NP_003817.1 | 8774 | gamma-soluble NSF attachment protein | 18.3 | 1 | 3 | 3 | 4 | 1.16E+08 | 1.96 | 4 | 14 | 312 | 34.7 | 5.4 | Transport | Cytoplasm;Golgi apparatus;Endoplasmic reticulum;Mitochondrion;Endosome | Auxiliary transport protein activity | Golgi apparatus |
| ANXA2 | NP_001002857.1 | 302 | annexin A2 isoform 2 | 78.2 | 2 | 42 | 42 | 552 | 1.42E+10 | 1.96 | 84 | 10.3 | 339 | 38.6 | 7.8 | Cell communication;Signal transduction | Cytoplasm;Nucleus;Plasma membrane;Endosome;Extracellular;Nucleolus;Apical membrane;Cytoskeleton | Calcium ion binding | Nucleus |
| ANXA4 | NP_001144.1 | 307 | annexin A4 | 56.1 | 1 | 17 | 17 | 55 | 7.20E+08 | 1.96 | 18 | 18.6 | 321 | 36.1 | 6.1 | Cell communication;Signal transduction | Nucleus;Cytoplasm;Golgi apparatus | Calcium ion binding | Nucleus |
| BIRC5 | NP_001012270.1 | 332 | baculoviral IAP repeat-containing protein 5 isoform 2 | 18.3 | 3 | 1 | 1 | 1 | 3.25E+07 | 1.95 | 1 | - | 137 | 15.6 | 7.7 | Cell communication;Signal transduction | Nucleus;Cytoskeleton;Centrosome;Cytoplasm;Chromosome;Microtubule;Kinetochore | Receptor signaling complex scaffold activity | Nucleus;Cytoplasm |
| GPX8 | NP_001008398.2 | 493869 | probable glutathione peroxidase 8 | 14.4 | 1 | 1 | 1 | 3 | 2.84E+08 | 1.95 | 1 | - | 209 | 23.9 | 9.4 | Biological_process unknown | Integral to membrane | Molecular function unknown | Integral to membrane |
| UTP20 | NP_055318.2 | 27340 | small subunit processome component 20 homolog | 8.5 | 1 | 15 | 15 | 15 | 1.31E+08 | 0.51 | 7 | 241.5 | 2785 | 318.2 | 7.4 | Cell communication;Signal transduction | Nucleolus | Molecular function unknown | Nucleolus |
| DCPS | NP_054745.1 | 28960 | m7GpppX diphosphatase | 21.7 | 1 | 4 | 4 | 7 | 7.36E+07 | 0.51 | 5 | 32.3 | 337 | 38.6 | 6.4 | Metabolism;Energy pathways | Nucleus;Cytoplasm | Hydrolase activity | Nucleus |
| STRBP | NP_001164608.1 | 55342 | spermatid perinuclear RNA-binding protein isoform 2 | 15.4 | 2 | 5 | 8 | 28 | 2.51E+08 | 0.51 | 4 | 8.5 | 658 | 71.9 | 8.7 | Regulation of nucleobase, nucleoside, nucleotide and nucleic acid metabolism | Nucleus | RNA binding | Nucleus |
| TSKS | NP_068379.1 | 60385 | testis-specific serine kinase substrate | 1.0 | 1 | 1 | 1 | 1 | 4.91E+07 | 0.51 | 1 | - | 592 | 65 | 6.0 | Protein metabolism | - | Protein serine/threonine kinase activity | - |
| PGM2 | NP_060760.2 | 55276 | phosphoglucomutase-2 | 40.9 | 1 | 18 | 18 | 42 | 6.39E+08 | 0.51 | 16 | 37.5 | 612 | 68.2 | 6.7 | Metabolism;Energy pathways | - | Catalytic activity | - |
| RRP12 | NP_001271266.1 | 23223 | RRP12-like protein isoform 3 | 29.8 | 3 | 23 | 23 | 54 | 2.30E+08 | 0.51 | 23 | 13.5 | 1197 | 132.6 | 8.7 | Biological_process unknown | Nucleolus;Nucleus | Molecular function unknown | Nucleolus |
| SLC25A3 | NP_998776.1 | 5250 | phosphate carrier protein, mitochondrial isoform b precursor | 32.4 | 2 | 11 | 11 | 73 | 6.39E+08 | 0.51 | 27 | 25.5 | 361 | 39.9 | 9.4 | Transport | Mitochondrial membrane | Transporter activity | Mitochondrial membrane |
| SMARCD2 | NP_001091896.1 | 6603 | SWI/SNF-related matrix-associated actin-dependent regulator of chromatin subfamily D member 2 | 15.4 | 3 | 2 | 3 | 3 | 5.97E+07 | 0.51 | 1 | - | 531 | 58.9 | 9.6 | Regulation of nucleobase, nucleoside, nucleotide and nucleic acid metabolism | Nucleus | DNA binding | Nucleus |
| MPRIP | NP_958431.2 | 23164 | myosin phosphatase Rho-interacting protein isoform 2 | 10.4 | 2 | 7 | 7 | 10 | 5.71E+07 | 0.51 | 5 | 20.4 | 1025 | 116.5 | 6.2 | Regulation of cell shape | Cytoplasm;Nucleus | Cytoskeletal protein binding | Cytoplasm |
| MRPL43 | NP_115488.2 | 84545 | 39S ribosomal protein L43, mitochondrial isoform a | 44.7 | 4 | 5 | 5 | 12 | 2.82E+08 | 0.51 | 3 | 86.6 | 159 | 17.8 | 9.9 | Protein metabolism | Mitochondrion | Structural constituent of ribosome | Mitochondrion |
| MVD | NP_002452.1 | 4597 | diphosphomevalonate decarboxylase | 39.8 | 1 | 8 | 8 | 17 | 4.70E+08 | 0.51 | 4 | 15.8 | 400 | 43.4 | 7.2 | Metabolism;Energy pathways | Cytoplasm;Peroxisome | Carboxy-lyase activity | Cytoplasm |
| MRPL18 | NP_054880.2 | 29074 | 39S ribosomal protein L18, mitochondrial | 13.3 | 1 | 2 | 2 | 3 | 7.95E+07 | 0.50 | 2 | 78.3 | 180 | 20.6 | 9.5 | Protein metabolism | Mitochondrion | Structural constituent of ribosome | Mitochondrion |
| SLC44A1 | NP_001273659.1 | 23446 | choline transporter-like protein 1 isoform b | 4.5 | 2 | 2 | 2 | 5 | 2.81E+07 | 0.50 | 2 | 54.1 | 652 | 73 | 8.7 | Transport | Integral to membrane | Molecular function unknown | Integral to membrane |
| LRRC59 | NP_060979.2 | 55379 | leucine-rich repeat-containing protein 59 | 50.8 | 1 | 15 | 15 | 90 | 1.89E+09 | 0.50 | 28 | 27.7 | 307 | 34.9 | 9.6 | Biological_process unknown | Integral to membrane;Membrane fraction | Molecular function unknown | Integral to membrane |
| GNAI1 | NP_001243343.1 | 2770 | guanine nucleotide-binding protein G(i) subunit alpha-1 isoform 2 | 34.4 | 3 | 4 | 6 | 25 | 6.18E+07 | 0.50 | 2 | 47.9 | 302 | 34.8 | 5.7 | Cell communication;Signal transduction | Plasma membrane;Nucleus;Cytoplasm | GTPase activity | Plasma membrane |
| DDX49 | NP_061943.2 | 54555 | probable ATP-dependent RNA helicase DDX49 | 10.1 | 1 | 3 | 3 | 3 | 2.85E+07 | 0.50 | 2 | 21.4 | 483 | 54.2 | 9.1 | Regulation of nucleobase, nucleoside, nucleotide and nucleic acid metabolism | Nucleus;Nucleolus | Helicase activity | Nucleus |
| CHD3 | NP_005843.2 | 1107 | chromodomain-helicase-DNA-binding protein 3 isoform 2 | 4.1 | 3 | 3 | 5 | 6 | 8.20E+07 | 0.50 | 3 | 4.4 | 1966 | 222.7 | 7.5 | Regulation of nucleobase, nucleoside, nucleotide and nucleic acid metabolism | Nucleus;Nucleolus;Mitochondrion;Cytoplasm | DNA binding | Nucleus |
| SRPR | NP_001171313.1 | 6734 | signal recognition particle receptor subunit alpha isoform 2 | 25.4 | 2 | 10 | 10 | 12 | 4.95E+07 | 0.50 | 3 | 62.7 | 610 | 66.5 | 9.0 | Protein metabolism | Endoplasmic reticulum | Molecular function unknown | Endoplasmic reticulum |
| AMDHD2 | NP_057028.2 | 51005 | putative N-acetylglucosamine-6-phosphate deacetylase isoform 1 | 7.5 | 2 | 2 | 2 | 2 | 1.42E+07 | 0.50 | 1 | - | 439 | 46.9 | 6.7 | Biological_process unknown | - | Molecular function unknown | - |
| NR3C1 | NP_001191194.1 | 2908 | glucocorticoid receptor isoform GR-P | 7.1 | 11 | 3 | 3 | 7 | 4.78E+07 | 0.50 | 2 | 90.3 | 676 | 73.7 | 6.1 | Regulation of nucleobase, nucleoside, nucleotide and nucleic acid metabolism | Nucleus;Cytoplasm;Mitochondrion;Plasma membrane | Ligand-dependent nuclear receptor activity | Nucleus |
| TFB2M | NP_071761.1 | 64216 | dimethyladenosine transferase 2, mitochondrial | 4.0 | 1 | 1 | 1 | 1 | 5.98E+07 | 0.50 | 1 | - | 396 | 45.3 | 9.2 | Regulation of nucleobase, nucleoside, nucleotide and nucleic acid metabolism | Mitochondrion | Transcription factor activity | Mitochondrion |
| DNAJB12 | NP_060096.3 | 54788 | dnaJ homolog subfamily B member 12 | 20.8 | 1 | 7 | 7 | 9 | 5.74E+07 | 0.50 | 3 | 17.1 | 409 | 45.5 | 9.4 | Biological_process unknown | - | Molecular function unknown | - |
| MRPS16 | NP_057149.1 | 51021 | 28S ribosomal protein S16, mitochondrial | 39.4 | 1 | 3 | 3 | 9 | 9.02E+07 | 0.50 | 3 | 25 | 137 | 15.3 | 9.5 | Protein metabolism | Mitochondrion | Structural constituent of ribosome | Mitochondrion |

Supplementary Table S4. List of all proteins identified in SCC-S and SCC-R cells using SequestHT and Mascot search algorithms and their gene ontology annotation

| Gene Symbol | RefSeq_Accession | Entrez Gene ID | Description | Coverage (%) | Proteins | Unique Peptides | # Peptides | PSM | Area | SCC-R /SCC-S | SCC-R /SCC-S (Count) | SCC-R /SCC-S (Variability [%]) | # AAs | MW [kDa] | calc. pI | Biological Process | Localization | Molecular Function | Primary Localization |
|---|---|---|---|---|---|---|---|---|---|---|---|---|---|---|---|---|---|---|---|
| CYB5A | NP_001905.1 | 1528 | cytochrome b5 isoform 2 | 21.4 | 3 | 1 | 1 | 1 | 4.04E+08 | 0.50 | 1 | - | 98 | 11.3 | 5.1 | Metabolism;Energy pathways | Mitochondrion | Oxidoreductase activity | Mitochondrion |
| DPM3 | NP_714963.1 | 54344 | dolichol-phosphate mannosyltransferase subunit 3 isoform 2 | 10.9 | 2 | 1 | 1 | 2 | 5.62E+08 | 0.50 | 1 | - | 92 | 10.1 | 5.9 | Metabolism | Endoplasmic reticulum;Plasma membrane;Golgi apparatus | Catalytic activity | Endoplasmic reticulum |
| NIF3L1 | NP_001135828.1 | 60491 | putative GTP cyclohydrolase 1 type 2 NIF3L1 isoform 3 | 20.4 | 3 | 3 | 3 | 3 | 3.33E+07 | 0.50 | 1 | - | 285 | 32.1 | 8.7 | Regulation of nucleobase, nucleoside, nucleotide and nucleic acid metabolism | Nucleus;Cytoplasm | Transcription regulator activity | Nucleus |
| DNPH1 | NP_006434.1 | 10591 | 2'-deoxynucleoside 5'-phosphate N-hydrolase 1 isoform 1 | 32.8 | 2 | 4 | 4 | 8 | 5.47E+08 | 0.49 | 5 | 13.6 | 174 | 19.1 | 5.1 | Biological_process unknown | Nucleus;Cytoplasm | Molecular function unknown | Nucleus |
| NSDHL | NP_057006.1 | 50814 | sterol-4-alpha-carboxylate 3-dehydrogenase, decarboxylating | 56.8 | 1 | 13 | 13 | 21 | 2.95E+08 | 0.49 | 10 | 39.6 | 373 | 41.9 | 8.1 | Metabolism;Energy pathways | Endoplasmic reticulum;Golgi apparatus;Mitochondrion | Catalytic activity | Endoplasmic reticulum |
| CAMSAP2 | NP_982284.1 | 23271 | calmodulin-regulated spectrin-associated protein 2 | 3.9 | 1 | 2 | 2 | 2 | 1.03E+08 | 0.49 | 1 | - | 1478 | 166.6 | 6.7 | Biological_process unknown | - | Molecular function unknown | - |
| CPNE1 | NP_001185792.1 | 8904 | copine-1 isoform c | 38.1 | 3 | 14 | 14 | 48 | 6.77E+08 | 0.49 | 13 | 10.5 | 536 | 58.9 | 5.8 | Vesicle-mediated transport | Cytoplasm | Transporter activity | Cytoplasm |
| MCCC2 | NP_071415.1 | 64087 | methylcrotonoyl-CoA carboxylase beta chain, mitochondrial | 21.9 | 1 | 9 | 9 | 15 | 2.26E+08 | 0.49 | 8 | 23.8 | 563 | 61.3 | 7.7 | Metabolism;Energy pathways | Mitochondrion | Ligase activity | Mitochondrion |
| RCOR1 | NP_055971.2 | 23186 | REST corepressor 1 | 11.1 | 5 | 3 | 3 | 3 | 1.55E+07 | 0.49 | 1 | - | 485 | 53.3 | 7.0 | Regulation of nucleobase, nucleoside, nucleotide and nucleic acid metabolism | Nucleus | Transcription regulator activity | Nucleus |
| TSR1 | NP_060598.3 | 55720 | pre-rRNA-processing protein TSR1 homolog | 18.4 | 1 | 9 | 9 | 19 | 1.54E+08 | 0.49 | 7 | 18.4 | 804 | 91.8 | 7.4 | Biological_process unknown | Nucleus | Molecular function unknown | Nucleus |
| GPD1L | NP_055956.1 | 23171 | glycerol-3-phosphate dehydrogenase 1-like protein | 28.2 | 1 | 6 | 6 | 10 | 1.13E+08 | 0.49 | 4 | 40.8 | 351 | 38.4 | 7.0 | Biological_process unknown | - | Molecular function unknown | - |
| RAB4A | NP_004569.2 | 5867 | ras-related protein Rab-4A isoform 1 | 11.5 | 14 | 1 | 2 | 11 | 4.33E+08 | 0.49 | 1 | - | 218 | 24.4 | 6.1 | Cell communication;Signal transduction | Endosome;Plasma membrane;Cytoplasm | GTPase activity | Endosome |
| HIST1H2BD | NP_619790.1 | 3017 | histone H2B type 1-D | 67.5 | 12 | 2 | 15 | 382 | 2.46E+10 | 0.49 | 5 | 20.3 | 126 | 13.9 | 10.3 | Chromosome organization and biogenesis (sensu Eukarya) | Nucleus;Nucleolus | DNA binding | Nucleus |
| SEPT6 | NP_665798.1 | 23157 | septin-6 isoform A | 34.2 | 4 | 4 | 11 | 22 | 4.34E+08 | 0.49 | 2 | 39.8 | 427 | 48.8 | 6.8 | Biological_process unknown | Cytoplasmic vesicle | Molecular function unknown | Cytoplasmic vesicle |
| CKAP5 | NP_055571.2 | 9793 | cytoskeleton-associated protein 5 isoform b | 16.7 | 2 | 22 | 22 | 40 | 1.79E+08 | 0.49 | 19 | 39.8 | 1972 | 218.4 | 8.1 | Mitosis | Centrosome;Microtubule;Mitochondrion | Cytoskeletal protein binding | Centrosome |
| MRPS9 | NP_872578.1 | 64965 | 28S ribosomal protein S9, mitochondrial | 22.5 | 1 | 6 | 6 | 10 | 8.15E+07 | 0.49 | 6 | 45 | 396 | 45.8 | 9.5 | Protein metabolism | Mitochondrion | Structural constituent of ribosome | Mitochondrion |
| PHKA1 | NP_001165907.1 | 5255 | phosphorylase b kinase regulatory subunit alpha, skeletal muscle isoform isoform 3 | 5.9 | 3 | 3 | 3 | 4 | 7.59E+07 | 0.49 | 2 | 4.1 | 1151 | 129.3 | 6.0 | Metabolism;Energy pathways | Endoplasmic reticulum;Plasma membrane | Phosphorylase activity | Endoplasmic reticulum |
| PWP2 | NP_005040.2 | 5822 | periodic tryptophan protein 2 homolog | 9.8 | 2 | 7 | 7 | 13 | 2.20E+08 | 0.49 | 7 | 7.7 | 919 | 102.4 | 6.2 | Cell communication;Signal transduction | Nucleolus;Ribosome | Signal transducer activity | Nucleolus |
| SLC9A3R1 | NP_004243.1 | 9368 | Na(+)/H(+) exchange regulatory cofactor NHE-RF1 | 35.5 | 1 | 8 | 8 | 21 | 4.10E+08 | 0.49 | 7 | 45.3 | 358 | 38.8 | 5.8 | Cell communication;Signal transduction | Cytoplasm;Membrane fraction | Receptor binding | Cytoplasm;Membrane fraction |
| TRMT6 | NP_057023.2 | 51605 | tRNA (adenine(58)-N(1))-methyltransferase non-catalytic subunit TRM6 isoform 1 | 23.1 | 2 | 6 | 6 | 8 | 1.08E+08 | 0.49 | 5 | 35 | 497 | 55.8 | 7.6 | Biological_process unknown | - | Molecular function unknown | - |
| RPL37A | NP_000989.1 | 6168 | 60S ribosomal protein L37a | 41.3 | 1 | 3 | 3 | 16 | 1.33E+08 | 0.49 | 5 | 9.3 | 92 | 10.3 | 10.4 | Protein metabolism | Ribosome | Structural constituent of ribosome | Ribosome |
| ABCB8 | NP_001269222.1 | 11194 | ATP-binding cassette sub-family B member 8, mitochondrial isoform d | 1.9 | 4 | 1 | 1 | 1 | 8.94E+07 | 0.49 | 1 | - | 630 | 68 | 8.5 | Transport | Mitochondrion | Transporter activity | Mitochondrion |
| CCZ1 | NP_056437.4 | 51622 | vacuolar fusion protein CCZ1 homolog | 11.6 | 1 | 3 | 3 | 5 | 6.94E+07 | 0.49 | 1 | - | 482 | 55.8 | 6.5 | Biological_process unknown | - | Molecular function unknown | - |
| DGKA | NP_958853.1 | 1606 | diacylglycerol kinase alpha | 4.2 | 1 | 2 | 2 | 3 | 6.55E+07 | 0.49 | 2 | 39.4 | 735 | 82.6 | 6.7 | Cell communication;Signal transduction | Cytoplasm;Nucleus;Plasma membrane;Cytoskeleton;Perinuclear region | Catalytic activity | Cytoplasm |
| DUS1L | NP_071439.3 | 64118 | tRNA-dihydrouridine(16/17) synthase [NAD(P)(+)]-like | 3.0 | 1 | 1 | 1 | 2 | 2.65E+06 | 0.49 | 1 | - | 473 | 53.2 | 8.3 | Regulation of nucleobase, nucleoside, nucleotide and nucleic acid metabolism | - | Molecular function unknown | - |
| PDLIM1 | NP_066272.1 | 9124 | PDZ and LIM domain protein 1 | 89.4 | 1 | 21 | 21 | 117 | 2.99E+09 | 0.49 | 35 | 16.8 | 329 | 36 | 7.0 | Biological_process unknown | Cytoplasm | Molecular function unknown | Cytoplasm |
| SLC25A11 | NP_001158889.1 | 8402 | mitochondrial 2-oxoglutarate/malate carrier protein isoform 2 | 39.3 | 3 | 8 | 8 | 26 | 6.67E+08 | 0.49 | 9 | 11.2 | 303 | 33 | 9.9 | Transport | Mitochondrion | Auxiliary transport protein activity | Mitochondrion |
| ALDH4A1 | NP_003739.2 | 8659 | delta-1-pyrroline-5-carboxylate dehydrogenase, mitochondrial isoform a precursor | 10.8 | 2 | 4 | 4 | 7 | 2.93E+07 | 0.48 | 1 | - | 563 | 61.7 | 8.1 | Metabolism;Energy pathways | Mitochondrion | Catalytic activity | Mitochondrion |
| COQ6 | NP_872282.1 | 51004 | ubiquinone biosynthesis monooxygenase COQ6 isoform a | 2.1 | 1 | 1 | 1 | 1 | 1.67E+07 | 0.48 | 1 | - | 468 | 50.8 | 7.3 | Metabolism | - | FAD binding | - |
| MTG1 | NP_612393.2 | 92170 | mitochondrial ribosome-associated GTPase 1 | 12.0 | 1 | 2 | 2 | 2 | 2.50E+07 | 0.48 | 1 | - | 334 | 37.2 | 9.5 | Biological_process unknown | - | Molecular function unknown | - |
| CEP55 | NP_060601.3 | 55165 | centrosomal protein of 55 kDa | 12.5 | 1 | 3 | 3 | 4 | 3.58E+07 | 0.48 | 2 | 14.5 | 464 | 54.1 | 7.0 | Biological_process unknown | - | Molecular function unknown | - |
| YTHDC2 | NP_073739.3 | 64848 | probable ATP-dependent RNA helicase YTHDC2 | 3.1 | 1 | 3 | 3 | 3 | 3.00E+07 | 0.48 | 3 | 7 | 1430 | 160.1 | 8.4 | Biological_process unknown | Nucleus | Molecular function unknown | Nucleus |
| CHAF1B | NP_005432.1 | 8208 | chromatin assembly factor 1 subunit B | 14.9 | 1 | 4 | 4 | 5 | 6.07E+07 | 0.48 | 4 | 6.6 | 559 | 61.5 | 7.5 | Regulation of nucleobase, nucleoside, nucleotide and nucleic acid metabolism | Nucleus | Chaperone activity | Nucleus |
| PPWD1 | NP_056157.1 | 23398 | peptidylprolyl isomerase domain and WD repeat-containing protein 1 isoform 1 | 15.0 | 4 | 6 | 6 | 9 | 2.45E+08 | 0.48 | 4 | 91.8 | 646 | 73.5 | 7.2 | Biological_process unknown | - | Molecular function unknown | - |
| WDR46 | NP_005443.3 | 9277 | WD repeat-containing protein 46 isoform 1 | 9.0 | 2 | 3 | 3 | 4 | 7.94E+07 | 0.48 | 1 | - | 610 | 68 | 9.7 | Cell communication;Signal transduction | Nucleolus | Molecular function unknown | Nucleolus |
| TBC1D2 | NP_060891.3 | 55357 | TBC1 domain family member 2A isoform 2 | 3.1 | 3 | 2 | 2 | 3 | 1.16E+08 | 0.48 | 2 | 55.3 | 917 | 104 | 6.5 | Cell communication;Signal transduction;Regulation of cell growth | - | GTPase activator activity | - |
| UNG | NP_550433.1 | 7374 | uracil-DNA glycosylase isoform UNG2 | 16.9 | 2 | 4 | 4 | 6 | 1.05E+08 | 0.48 | 3 | 15 | 313 | 34.6 | 9.3 | DNA repair | Nucleus;Mitochondrion;Nucleolus;Cytoplasm | DNA binding;DNA repair protein | Nucleus;Cytoplasm |

| Gene Symbol | RefSeq_Accession | Entrez Gene ID | Description | Coverage (%) | Proteins | Unique Peptides | # Peptides | PSM | Area | SCC-R /SCC-S | SCC-R /SCC-S (Count) | SCC-R /SCC-S (Variability [%]) | # AAs | MW [kDa] | calc. pI | Biological Process | Localization | Molecular Function | Primary Localization |
|---|---|---|---|---|---|---|---|---|---|---|---|---|---|---|---|---|---|---|---|
| **ALDH1L2** | NP_001029345.2 | 160428 | **mitochondrial 10-formyltetrahydrofolate dehydrogenase precursor** | 31.0 | 1 | 16 | 16 | 26 | 1.36E+08 | **0.48** | 12 | 16 | 923 | 101.7 | 6.5 | Biological_process unknown | - | Molecular function unknown | - |
| **RPL38** | NP_001030335.1 | 6169 | **60S ribosomal protein L38** | 54.3 | 1 | 6 | 6 | 26 | 1.72E+09 | **0.48** | 9 | 22.7 | 70 | 8.2 | 10.1 | Protein metabolism | Ribosome | Structural constituent of ribosome | Ribosome |
| **PNPLA6** | NP_001159584.1 | 10908 | **neuropathy target esterase isoform c** | 9.9 | 4 | 7 | 7 | 8 | 4.54E+07 | **0.48** | 3 | 25.3 | 1300 | 143.3 | 7.7 | Metabolism;Energy pathways | Endoplasmic reticulum;Mitochondrion;Microsome | Hydrolase activity | Endoplasmic reticulum |
| **SERBP1** | NP_001018078.1 | 26135 | **plasminogen activator inhibitor 1 RNA-binding protein isoform 2** | 50.0 | 4 | 16 | 16 | 75 | 2.54E+09 | **0.48** | 29 | 32.3 | 402 | 44.2 | 8.7 | Regulation of nucleobase, nucleoside, nucleotide and nucleic acid metabolism | Cytoplasm;Nucleus | RNA binding | Cytoplasm;Nucleus |
| **SERF2** | NP_001186807.1 | 10169 | **small EDRK-rich factor 2 isoform d** | 40.0 | 4 | 2 | 2 | 2 | 3.12E+07 | **0.48** | 2 | 60.3 | 45 | 5.2 | 10.1 | Regulation of nucleobase, nucleoside, nucleotide and nucleic acid metabolism | Nucleus;Nucleolus | Nucleic acid binding | Nucleus |
| **MRPS34** | NP_076425.1 | 65993 | **28S ribosomal protein S34, mitochondrial** | 34.4 | 1 | 6 | 6 | 12 | 7.22E+07 | **0.48** | 2 | 36.3 | 218 | 25.6 | 10.0 | Protein metabolism | Mitochondrion | Structural constituent of ribosome | Mitochondrion |
| **FLOT2** | NP_004466.2 | 2319 | **flotillin-2** | 39.0 | 1 | 11 | 11 | 24 | 3.21E+08 | **0.48** | 8 | 28.2 | 428 | 47 | 5.3 | Cell communication;Signal transduction | Plasma membrane;Cytoplasm | Cell adhesion molecule activity | Plasma membrane |
| **APEH** | NP_001631.3 | 327 | **acylamino-acid-releasing enzyme** | 12.8 | 1 | 6 | 6 | 8 | 7.71E+07 | **0.48** | 4 | 12 | 732 | 81.2 | 5.5 | Metabolism;Energy pathways | Cytoplasm | Hydrolase activity | Cytoplasm |
| **DHX37** | NP_116045.2 | 57647 | **probable ATP-dependent RNA helicase DHX37** | 2.0 | 1 | 2 | 2 | 3 | 2.60E+07 | **0.48** | 1 | - | 1157 | 129.5 | 8.1 | Regulation of nucleobase, nucleoside, nucleotide and nucleic acid metabolism | Nucleolus | Helicase activity | Nucleolus |
| **NOP2** | NP_001245237.1 | 4839 | **putative ribosomal RNA methyltransferase NOP2 isoform 2** | 32.6 | 4 | 19 | 19 | 37 | 6.86E+08 | **0.48** | 18 | 18.7 | 812 | 89.2 | 9.2 | Regulation of cell cycle;Cell proliferation | Nucleolus;Cytoplasm;Nucleus | Molecular function unknown | Nucleolus |
| **WDR75** | NP_115544.1 | 84128 | **WD repeat-containing protein 75** | 11.9 | 1 | 6 | 6 | 12 | 9.28E+07 | **0.47** | 6 | 28.5 | 830 | 94.4 | 6.0 | Cell communication | - | Molecular function unknown | - |
| **PRMT3** | NP_001138639.1 | 10196 | **protein arginine N-methyltransferase 3 isoform 3** | 10.6 | 3 | 4 | 4 | 10 | 9.35E+07 | **0.47** | 3 | 1.8 | 454 | 51.5 | 6.2 | Metabolism;Energy pathways | Cytoplasm;Nucleus;Ribosome | Methyltransferase activity | Cytoplasm |
| **CIRH1A** | NP_116219.2 | 84916 | **cirhin** | 7.9 | 1 | 4 | 4 | 6 | 2.60E+07 | **0.47** | 3 | 10.6 | 686 | 76.8 | 8.9 | Cell communication;Signal transduction | Mitochondrion;Nucleolus;Cytosol | Molecular function unknown | Mitochondrion |
| **CKMT1A** | NP_001015001.1 | 548596 | **creatine kinase U-type, mitochondrial precursor** | 37.7 | 2 | 7 | 8 | 14 | 3.15E+08 | **0.47** | 7 | 14.1 | 417 | 47 | 8.3 | Energy pathways | Mitochondrion | Transferase activity | Mitochondrion |
| **TBC1D4** | NP_001273588.1 | 9882 | **TBC1 domain family member 4 isoform 3** | 10.0 | 5 | 8 | 8 | 10 | 1.13E+08 | **0.47** | 5 | 21.3 | 1235 | 139.5 | 6.9 | Cell communication;Signal transduction | Nucleus;Cytoplasm | GTPase activator activity | Nucleus;Cytoplasm |
| **NFX1** | NP_002495.2 | 4799 | **transcriptional repressor NF-X1 isoform 1** | 1.8 | 1 | 1 | 1 | 2 | 1.82E+07 | **0.47** | 1 | - | 1120 | 124.3 | 8.2 | Regulation of nucleobase, nucleoside, nucleotide and nucleic acid metabolism;Regulation of gene expression, epigenetic | Nucleus;Cytoplasm | Transcription regulator activity;DNA binding | Nucleus |
| **URB1** | NP_055640.2 | 9875 | **nucleolar pre-ribosomal-associated protein 1** | 1.1 | 1 | 2 | 2 | 2 | 3.13E+07 | **0.47** | 1 | - | 2271 | 254.2 | 6.5 | Biological_process unknown | - | Protein binding | - |
| **CWF19L1** | NP_060764.3 | 55280 | **CWF19-like protein 1** | 13.8 | 1 | 5 | 5 | 6 | 3.94E+07 | **0.47** | 3 | 28.8 | 538 | 60.6 | 7.2 | Biological_process unknown | - | Molecular function unknown | - |
| **ELAC2** | NP_001159434.1 | 60528 | **zinc phosphodiesterase ELAC protein 2 isoform 3** | 36.6 | 3 | 19 | 19 | 38 | 3.39E+08 | **0.47** | 16 | 16 | 786 | 87.5 | 8.0 | Biological_process unknown | Nucleus;Cytoplasm | Molecular function unknown | Nucleus |
| **ABCF1** | NP_001081.1 | 23 | **ATP-binding cassette sub-family F member 1 isoform b** | 29.0 | 2 | 13 | 13 | 27 | 2.33E+08 | **0.47** | 14 | 26.5 | 807 | 91.6 | 7.7 | Protein metabolism | Ribosome;Nucleus;Cytoplasm | Translation regulator activity | Ribosome;Nucleus |
| **ANXA3** | NP_005130.1 | 306 | **annexin A3** | 66.3 | 1 | 20 | 20 | 118 | 2.33E+09 | **0.47** | 35 | 23.2 | 323 | 36.4 | 5.9 | Cell communication;Signal transduction | Cytoplasm | Calcium ion binding | Cytoplasm |
| **KIF1C** | NP_006603.2 | 10749 | **kinesin-like protein KIF1C** | 1.8 | 1 | 1 | 2 | 4 | 5.84E+07 | **0.47** | 1 | - | 1103 | 122.9 | 6.9 | Cell growth and/or maintenance | Golgi apparatus;Endoplasmic reticulum;Cytoplasm | Motor activity | Golgi apparatus |
| **MED6** | NP_005457.2 | 10001 | **mediator of RNA polymerase II transcription subunit 6 isoform 2** | 11.8 | 4 | 2 | 2 | 5 | 8.82E+07 | **0.47** | 3 | 1.1 | 246 | 28.4 | 8.6 | Regulation of nucleobase, nucleoside, nucleotide and nucleic acid metabolism | Nucleus | Transcription regulator activity | Nucleus |
| **MRPS35** | NP_068593.2 | 60488 | **28S ribosomal protein S35, mitochondrial isoform 1 precursor** | 13.0 | 2 | 3 | 3 | 17 | 2.52E+07 | **0.47** | 3 | 4.6 | 323 | 36.8 | 8.2 | Protein metabolism | Mitochondrion | Structural constituent of ribosome | Mitochondrion |
| **C5orf24** | NP_689622.2 | 134553 | **UPF0461 protein C5orf24** | 14.9 | 1 | 2 | 2 | 2 | 3.87E+06 | **0.47** | 1 | - | 188 | 20.1 | 9.9 | Biological_process unknown | - | Molecular function unknown | - |
| **HEATR5B** | NP_061897.1 | 54497 | **HEAT repeat-containing protein 5B** | 2.5 | 1 | 3 | 3 | 5 | 4.54E+07 | **0.47** | 2 | 3.8 | 2071 | 224.2 | 7.2 | Biological_process unknown | - | Molecular function unknown | - |
| **KIAA1522** | NP_001185901.1 | 57648 | **uncharacterized protein KIAA1522 isoform 2** | 8.4 | 2 | 5 | 5 | 6 | 2.74E+08 | **0.47** | 1 | - | 1035 | 107 | 9.7 | Biological_process unknown | - | Molecular function unknown | - |
| **LSS** | NP_001138909.1 | 4047 | **lanosterol synthase isoform 3** | 4.5 | 3 | 2 | 2 | 2 | 2.90E+07 | **0.47** | 1 | - | 652 | 74.2 | 6.5 | Metabolism;Energy pathways | Endoplasmic reticulum;Mitochondrion | Catalytic activity | Endoplasmic reticulum |
| **PYCR2** | NP_001258610.1 | 29920 | **pyrroline-5-carboxylate reductase 2 isoform 2** | 32.1 | 2 | 4 | 5 | 9 | 1.79E+08 | **0.47** | 2 | 26.7 | 246 | 25.9 | 9.3 | Biological_process unknown | Mitochondrion | Molecular function unknown | Mitochondrion |
| **RRP7A** | NP_056518.2 | 27341 | **ribosomal RNA-processing protein 7 homolog A** | 12.5 | 1 | 2 | 2 | 2 | 1.96E+07 | **0.47** | 2 | 93.7 | 280 | 32.3 | 9.6 | Regulation of nucleobase, nucleoside, nucleotide and nucleic acid metabolism | Nucleolus;Nucleus | RNA binding | Nucleolus |
| **ATXN10** | NP_037368.1 | 25814 | **ataxin-10 isoform 1** | 22.3 | 2 | 8 | 8 | 21 | 1.99E+08 | **0.47** | 6 | 24.7 | 475 | 53.5 | 5.3 | Biological_process unknown | - | Molecular function unknown | - |
| **HMGB3** | NP_005333.2 | 3149 | **high mobility group protein B3** | 48.0 | 1 | 10 | 10 | 26 | 6.33E+08 | **0.47** | 9 | 66.1 | 200 | 23 | 8.4 | Regulation of nucleobase, nucleoside, nucleotide and nucleic acid metabolism | Nucleus | DNA binding | Nucleus |
| **TARBP2** | NP_004169.3 | 6895 | **RISC-loading complex subunit TARBP2 isoform b** | 4.6 | 2 | 1 | 1 | 2 | 3.76E+06 | **0.47** | 1 | - | 345 | 36.9 | 7.4 | Regulation of nucleobase, nucleoside, nucleotide and nucleic acid metabolism | Cytoplasm;Nucleus | RNA binding | Cytoplasm |
| **C4orf32** | NP_689613.1 | 132720 | **uncharacterized protein C4orf32** | 37.1 | 1 | 3 | 3 | 3 | 2.44E+07 | **0.46** | 3 | 7 | 132 | 14.7 | 4.7 | Biological_process unknown | - | Molecular function unknown | - |
| **PCCB** | NP_000523.2 | 5096 | **propionyl-CoA carboxylase beta chain, mitochondrial isoform 1 precursor** | 11.0 | 2 | 4 | 4 | 8 | 1.11E+08 | **0.46** | 6 | 32.8 | 539 | 58.2 | 7.6 | Metabolism;Energy pathways | Mitochondrion | Ligase activity | Mitochondrion |
| **SPAST** | NP_955468.1 | 6683 | **spastin isoform 2** | 6.9 | 2 | 2 | 2 | 2 | 3.79E+07 | **0.46** | 1 | - | 584 | 63.6 | 9.7 | Protein metabolism | Nucleus;Centrosome;Cytoskeleton;Cytoplasm;Golgi apparatus | Cytoskeletal protein binding | Nucleus |
| **VARS** | NP_006286.1 | 7407 | **valine--tRNA ligase** | 40.6 | 1 | 30 | 30 | 70 | 7.65E+08 | **0.46** | 27 | 32.8 | 1264 | 140.4 | 7.6 | Metabolism;Energy pathways | - | Ligase activity | - |

| Gene Symbol | RefSeq_Accession | Entrez Gene ID | Description | Coverage (%) | Proteins | Unique Peptides | # Peptides | PSM | Area | SCC-R /SCC-S | SCC-R /SCC-S (Count) | SCC-R /SCC-S (Variability [%]) | # AAs | MW [kDa] | calc. pI | Biological Process | Localization | Molecular Function | Primary Localization |
|---|---|---|---|---|---|---|---|---|---|---|---|---|---|---|---|---|---|---|---|
| DAG1 | NP_001171107.1 | 1605 | dystroglycan preproprotein | 1.7 | 1 | 1 | 1 | 1 | 1.56E+07 | 0.46 | 1 | - | 895 | 97.5 | 8.6 | Cell communication;Signal transduction | Plasma membrane;Extracellular | Cell adhesion molecule activity | Plasma membrane |
| PHIP | NP_060404.3 | 55023 | PH-interacting protein | 3.8 | 1 | 4 | 4 | 4 | 1.95E+07 | 0.46 | 2 | 52.3 | 1821 | 206.5 | 8.9 | Cell communication;Signal transduction | - | Receptor binding | - |
| CRB2 | NP_775960.4 | 286204 | protein crumbs homolog 2 precursor | 1.9 | 1 | 1 | 1 | 3 | 4.05E+09 | 0.46 | 1 | - | 1285 | 134.2 | 5.6 | Cell growth and/or maintenance | Plasma membrane;Extracellular | Growth factor activity | Plasma membrane |
| MAN2B1 | NP_001166969.1 | 4125 | lysosomal alpha-mannosidase isoform 2 precursor | 8.5 | 2 | 5 | 5 | 6 | 2.35E+08 | 0.46 | 2 | 54 | 1010 | 113.5 | 7.3 | Metabolism;Energy pathways | Lysosome | Catalytic activity | Lysosome |
| MEMO1 | NP_057039.1 | 51072 | protein MEMO1 isoform 1 | 43.8 | 2 | 7 | 7 | 15 | 1.11E+08 | 0.46 | 8 | 11.8 | 297 | 33.7 | 7.1 | Biological_process unknown | - | Molecular function unknown | - |
| POLDIP3 | NP_835237.1 | 84271 | polymerase delta-interacting protein 3 isoform 2 | 41.6 | 3 | 10 | 10 | 20 | 1.72E+08 | 0.46 | 7 | 45.6 | 392 | 42.9 | 9.9 | Cell growth and/or maintenance | Nucleolus | RNA binding | Nucleolus |
| RETSAT | NP_060220.3 | 54884 | all-trans-retinol 13,14-reductase precursor | 9.7 | 1 | 4 | 4 | 5 | 5.80E+07 | 0.46 | 2 | 3.8 | 610 | 66.8 | 8.3 | Vitamin metabolism | Endoplasmic reticulum membrane;Microsome;Mitochondrion | Catalytic activity | Endoplasmic reticulum membrane |
| CTDSPL2 | NP_057480.2 | 51496 | CTD small phosphatase-like protein 2 | 6.9 | 1 | 1 | 2 | 2 | 3.10E+07 | 0.46 | 1 | - | 466 | 53 | 6.4 | Biological_process unknown | Nucleus | Molecular function unknown | Nucleus |
| KCTD3 | NP_057205.2 | 51133 | BTB/POZ domain-containing protein KCTD3 | 5.6 | 1 | 2 | 3 | 4 | 4.39E+07 | 0.46 | 2 | 42.7 | 815 | 88.9 | 7.0 | Transport | - | Ion channel activity | - |
| MDN1 | NP_055426.1 | 23195 | midasin | 1.2 | 1 | 5 | 5 | 5 | 4.81E+07 | 0.46 | 3 | 29.1 | 5596 | 632.4 | 5.7 | Regulation of nucleobase, nucleoside, nucleotide and nucleic acid metabolism | Nucleus;Nucleolus | Chaperone activity | Nucleus |
| NKRF | NP_001166959.1 | 55922 | NF-kappa-B-repressing factor isoform 2 | 11.9 | 2 | 4 | 4 | 4 | 2.06E+07 | 0.46 | 2 | 32.6 | 690 | 77.6 | 8.8 | Regulation of nucleobase, nucleoside, nucleotide and nucleic acid metabolism | Nucleus;Nucleolus | Transcription regulator activity | Nucleus |
| ZFP36L2 | NP_008818.3 | 678 | zinc finger protein 36, C3H1 type-like 2 | 4.1 | 1 | 1 | 1 | 1 | 3.23E+07 | 0.46 | 1 | - | 494 | 51 | 8.2 | Regulation of nucleobase, nucleoside, nucleotide and nucleic acid metabolism | Nucleus | Transcription factor activity | Nucleus |
| ACAT2 | NP_005882.2 | 39 | acetyl-CoA acetyltransferase, cytosolic | 62.7 | 1 | 14 | 14 | 63 | 5.04E+08 | 0.46 | 18 | 26 | 397 | 41.3 | 6.9 | Metabolism;Energy pathways | Cytoplasm;Endoplasmic reticulum | Acyltransferase activity | Cytoplasm |
| ALDH7A1 | NP_001188306.1 | 501 | alpha-aminoadipic semialdehyde dehydrogenase isoform 2 | 50.5 | 3 | 16 | 16 | 33 | 3.79E+08 | 0.46 | 14 | 12.1 | 511 | 55.3 | 6.9 | Metabolism;Energy pathways | - | Oxidoreductase activity | - |
| PFKM | NP_000280.1 | 5213 | 6-phosphofructokinase, muscle type isoform 2 | 23.9 | 2 | 10 | 11 | 20 | 2.75E+08 | 0.46 | 5 | 34.4 | 780 | 85.1 | 8.0 | Metabolism;Energy pathways | - | Catalytic activity | - |
| VASP | NP_003361.1 | 7408 | vasodilator-stimulated phosphoprotein | 17.4 | 1 | 5 | 5 | 8 | 1.27E+08 | 0.46 | 4 | 176.9 | 380 | 39.8 | 8.9 | Cytoskeleton organization and biogenesis | Cytoplasm;Focal adhesion;Extracellular matrix | Cytoskeletal protein binding | Cytoplasm |
| TRIM26 | NP_003440.1 | 7726 | tripartite motif-containing protein 26 | 7.6 | 1 | 2 | 2 | 2 | 2.21E+07 | 0.46 | 1 | - | 539 | 62.1 | 5.0 | Biological_process unknown | Nucleus | DNA binding | Nucleus |
| ATAD1 | NP_116199.2 | 84896 | ATPase family AAA domain-containing protein 1 | 15.2 | 1 | 4 | 4 | 10 | 1.10E+08 | 0.45 | 6 | 57.4 | 361 | 40.7 | 6.9 | Metabolism;Energy pathways | Mitochondrion;Nucleus | ATPase activity | Mitochondrion;Nucleus |
| MTA3 | NP_065795.1 | 57504 | metastasis-associated protein MTA3 isoform b | 10.5 | 2 | 1 | 4 | 5 | 9.83E+07 | 0.45 | 1 | - | 515 | 58.8 | 8.2 | Regulation of nucleobase, nucleoside, nucleotide and nucleic acid metabolism | Nucleus | Transcription regulator activity | Nucleus |
| CLUH | NP_056044.3 | 23277 | clustered mitochondria protein homolog | 30.6 | 3 | 25 | 25 | 53 | 8.79E+08 | 0.45 | 23 | 28.3 | 1309 | 146.6 | 6.1 | Protein metabolism | Cytoplasm | Translation regulator activity | Cytoplasm |
| SLTM | NP_001013865.1 | 79811 | SAFB-like transcription modulator isoform b | 13.1 | 2 | 9 | 9 | 16 | 9.04E+07 | 0.45 | 7 | 26.5 | 1016 | 115.3 | 8.2 | Regulation of nucleobase, nucleoside, nucleotide and nucleic acid metabolism | Nucleus | Nucleic acid binding | Nucleus |
| TMX2 | NP_001137484.1 | 51075 | thioredoxin-related transmembrane protein 2 isoform 2 | 26.7 | 2 | 6 | 6 | 15 | 7.37E+08 | 0.45 | 6 | 4.3 | 258 | 29.6 | 8.7 | Biological_process unknown | - | Molecular function unknown | - |
| DIDO1 | NP_001180298.1 | 11083 | death-inducer obliterator 1 isoform c | 8.4 | 3 | 10 | 10 | 13 | 1.09E+08 | 0.45 | 6 | 33.3 | 2240 | 243.7 | 7.9 | Apoptosis | Cytoplasm;Nucleus | Molecular function unknown | Nucleus;Cytoplasm |
| APC | NP_001120983.2 | 324 | adenomatous polyposis coli protein isoform a | 0.5 | 2 | 1 | 1 | 1 | 3.11E+07 | 0.45 | 1 | - | 2825 | 308.5 | 8.0 | Cell communication;Signal transduction | Cytoplasm;Nucleus;Plasma membrane | Cell adhesion molecule activity | Cytoplasm |
| SPTBN2 | NP_008877.1 | 6712 | spectrin beta chain, non-erythrocytic 2 | 13.6 | 3 | 14 | 18 | 25 | 2.21E+08 | 0.45 | 9 | 31.8 | 2390 | 271.1 | 6.1 | Cell growth and/or maintenance | Plasma membrane;Golgi apparatus;Cytoplasmic vesicle | Cytoskeletal protein binding | Golgi apparatus |
| BPTF | NP_004450.3 | 2186 | nucleosome-remodeling factor subunit BPTF isoform 2 | 2.6 | 2 | 4 | 4 | 4 | 2.26E+07 | 0.45 | 3 | 12.5 | 2903 | 322 | 6.4 | Regulation of nucleobase, nucleoside, nucleotide and nucleic acid metabolism | Nucleus;Cytoplasm;Perinuclear region | Transcription regulator activity | Nucleus |
| KTN1 | NP_001072989.1 | 3895 | kinectin isoform a | 49.7 | 4 | 56 | 56 | 156 | 1.36E+09 | 0.45 | 50 | 16.7 | 1357 | 156.2 | 5.6 | Cell growth and/or maintenance | Endoplasmic reticulum;Lysosome;Microtubule;Nucleus;Cytoplasm | Cytoskeletal anchoring activity | Endoplasmic reticulum;Nucleus;Cytoplasm |
| MAP2K4 | NP_003001.1 | 6416 | dual specificity mitogen-activated protein kinase kinase 4 isoform 1 | 11.3 | 2 | 3 | 3 | 6 | 8.20E+07 | 0.45 | 3 | 2.3 | 399 | 44.3 | 8.1 | Cell communication;Signal transduction | Cytoplasm;Nucleus;Cytosol;Plasma membrane | Protein threonine/tyrosine kinase activity | Cytoplasm;Plasma membrane |
| PTPRK | NP_002835.2 | 5796 | receptor-type tyrosine-protein phosphatase kappa isoform b precursor | 8.3 | 6 | 7 | 7 | 8 | 6.75E+07 | 0.45 | 4 | 11.4 | 1440 | 162.1 | 5.9 | Cell communication;Signal transduction | Plasma membrane;Extracellular | Receptor signaling protein tyrosine kinase activity | Plasma membrane |
| DIS3 | NP_001121698.1 | 22894 | exosome complex exonuclease RRP44 isoform b | 17.2 | 2 | 8 | 8 | 12 | 2.14E+08 | 0.45 | 6 | 26.1 | 928 | 105 | 7.2 | Regulation of nucleobase, nucleoside, nucleotide and nucleic acid metabolism | Nucleus;Nucleolus | RNA binding | Nucleus |
| GMDS | NP_001491.1 | 2762 | GDP-mannose 4,6 dehydratase isoform 1 | 14.0 | 2 | 2 | 2 | 5 | 1.68E+08 | 0.45 | 2 | 1.4 | 372 | 41.9 | 7.3 | Metabolism;Energy pathways | Cytoplasm | Hydro-lyase activity | Cytoplasm |
| TARSL2 | NP_689547.2 | 123283 | probable threonine--tRNA ligase 2, cytoplasmic | 6.0 | 1 | 1 | 3 | 9 | 3.28E+07 | 0.45 | 1 | - | 802 | 92.6 | 6.1 | Protein metabolism | - | Ligase activity | - |
| FDPS | NP_001129294.1 | 2224 | farnesyl pyrophosphate synthase isoform b | 39.4 | 3 | 11 | 11 | 35 | 1.69E+09 | 0.45 | 13 | 25.2 | 353 | 40.5 | 5.2 | Metabolism;Energy pathways | Peroxisome | Transferase activity | Peroxisome |
| MAPK10 | NP_002744.1 | 5602 | mitogen-activated protein kinase 10 isoform 1 | 6.4 | 9 | 1 | 2 | 2 | 5.25E+07 | 0.45 | 1 | - | 422 | 48.5 | 7.7 | Signal transduction | Vesicle;Cytoplasm;Nucleus | Protein serine/threonine kinase activity | Cytoplasm |
| TRMT61A | NP_689520.2 | 115708 | tRNA (adenine(58)-N(1))-methyltransferase catalytic subunit TRMT61A | 14.9 | 1 | 3 | 3 | 3 | 9.77E+07 | 0.45 | 2 | 9 | 289 | 31.4 | 7.4 | Regulation of nucleobase, nucleoside, nucleotide and nucleic acid metabolism | - | RNA methyltransferase activity | - |
| RPS6KA4 | NP_001006945.1 | 8986 | ribosomal protein S6 kinase alpha-4 isoform b | 6.7 | 2 | 2 | 2 | 2 | 5.59E+07 | 0.45 | 2 | 59.8 | 766 | 84.8 | 8.4 | Cell communication;Signal transduction;Regulation of nucleobase, nucleoside, nucleotide and nucleic acid metabolism | Nucleus | Protein serine/threonine kinase activity;Transcription regulator activity | Nucleus |
| GAN | NP_071324.1 | 8139 | gigaxonin | 2.9 | 1 | 1 | 1 | 1 | 6.56E+07 | 0.45 | 1 | - | 597 | 67.6 | 5.9 | Cell growth and/or maintenance | Cytoplasm;Nucleus | Cytoskeletal protein binding | Cytoplasm |

| Gene Symbol | RefSeq_Accession | Entrez Gene ID | Description | Coverage (%) | Proteins | Unique Peptides | # Peptides | PSM | Area | SCC-R /SCC-S | SCC-R /SCC-S (Count) | SCC-R /SCC-S (Variability [%]) | # AAs | MW [kDa] | calc. pI | Biological Process | Localization | Molecular Function | Primary Localization |
|---|---|---|---|---|---|---|---|---|---|---|---|---|---|---|---|---|---|---|---|
| TOR1AIP1 | NP_056417.2 | 26092 | torsin-1A-interacting protein 1 isoform 2 | 24.7 | 2 | 9 | 9 | 18 | 1.59E+08 | 0.45 | 9 | 22.1 | 583 | 66.2 | 8.2 | Biological_process unknown | Nucleus;Endoplasmic reticulum;Nuclear membrane | Molecular function unknown | Nucleus |
| TOX4 | NP_055643.1 | 9878 | TOX high mobility group box family member 4 | 9.5 | 7 | 3 | 3 | 3 | 5.69E+07 | 0.45 | 2 | 22.7 | 621 | 66.2 | 5.1 | Regulation of nucleobase, nucleoside, nucleotide and nucleic acid metabolism | Nucleus | DNA binding | Nucleus |
| ACLY | NP_942127.1 | 47 | ATP-citrate synthase isoform 2 | 50.2 | 2 | 46 | 46 | 164 | 1.77E+09 | 0.44 | 51 | 19.2 | 1091 | 119.7 | 7.3 | Metabolism;Energy pathways | Cytoplasm;Mitochondrion | ATPase activity | Cytoplasm |
| C1orf174 | NP_997239.2 | 339448 | UPF0688 protein C1orf174 | 5.8 | 1 | 1 | 1 | 2 | 1.55E+07 | 0.44 | 1 | - | 243 | 26 | 6.9 | Biological_process unknown | - | Molecular function unknown | - |
| TANGO6 | NP_078838.1 | 79613 | transport and Golgi organization protein 6 homolog | 1.4 | 1 | 1 | 1 | 1 | 4.58E+06 | 0.44 | 1 | - | 1094 | 120.7 | 6.1 | Biological_process unknown | - | Molecular function unknown | - |
| ASCC1 | NP_001185729.1 | 51008 | activating signal cointegrator 1 complex subunit 1 isoform b | 5.9 | 2 | 1 | 1 | 1 | 2.55E+07 | 0.44 | 1 | - | 357 | 41.2 | 5.5 | Regulation of nucleobase, nucleoside, nucleotide and nucleic acid metabolism | Nucleus | Transcription regulator activity | Nucleus |
| HADHA | NP_000173.2 | 3030 | trifunctional enzyme subunit alpha, mitochondrial precursor | 57.4 | 1 | 34 | 34 | 152 | 1.38E+09 | 0.44 | 50 | 28.1 | 763 | 82.9 | 9.0 | Metabolism;Energy pathways | Mitochondrion;Nucleus | Catalytic activity | Mitochondrion |
| KIF21A | NP_001166936.1 | 55605 | kinesin-like protein KIF21A isoform 4 | 0.9 | 4 | 1 | 1 | 1 | 5.88E+07 | 0.44 | 1 | - | 1621 | 180.6 | 6.3 | Cell growth and/or maintenance | Cytoplasm;Nucleus | Motor activity | Cytoplasm;Nucleus |
| TUBA4A | NP_001265481.1 | 7277 | tubulin alpha-4A chain isoform 2 | 61.7 | 3 | 4 | 25 | 1005 | 1.68E+10 | 0.44 | 4 | 16.7 | 433 | 48.3 | 5.0 | Cell growth and/or maintenance | Cytoplasm;Microtubule | Structural constituent of cytoskeleton | Cytoplasm |
| BIRC6 | NP_057336.3 | 57448 | baculoviral IAP repeat-containing protein 6 | 2.5 | 1 | 5 | 5 | 6 | 1.32E+08 | 0.44 | 2 | 17.8 | 4857 | 529.9 | 6.1 | Apoptosis | Cytoplasm;Nucleus | Receptor signaling complex scaffold activity | Cytoplasm |
| ELOVL5 | NP_068586.1 | 60481 | elongation of very long chain fatty acids protein 5 isoform 1 | 13.4 | 4 | 2 | 2 | 4 | 1.25E+08 | 0.44 | 1 | - | 299 | 35.3 | 9.4 | Biological_process unknown | Integral to membrane;Nucleus;Cytoplasm | Molecular function unknown | Integral to membrane |
| MTPAP | NP_060579.3 | 55149 | poly(A) RNA polymerase, mitochondrial precursor | 7.0 | 1 | 2 | 2 | 4 | 8.06E+07 | 0.44 | 1 | - | 582 | 66.1 | 9.0 | Regulation of nucleobase, nucleoside, nucleotide and nucleic acid metabolism | Mitochondrion;Nucleus | DNA-directed RNA polymerase activity | Mitochondrion |
| UTP11L | NP_057121.2 | 51118 | probable U3 small nucleolar RNA-associated protein 11 | 3.2 | 1 | 1 | 1 | 1 | 3.14E+07 | 0.44 | 1 | - | 253 | 30.4 | 10.2 | Regulation of nucleobase, nucleoside, nucleotide and nucleic acid metabolism | Nucleolus;Cytoplasm;Nucleus;Extracellular | RNA binding | Nucleolus |
| ATAD2 | NP_054828.2 | 29028 | ATPase family AAA domain-containing protein 2 | 17.0 | 1 | 14 | 14 | 23 | 1.64E+08 | 0.44 | 11 | 13.9 | 1390 | 158.5 | 6.3 | Metabolism;Energy pathways | Nucleus | ATPase activity | Nucleus |
| MYO18A | NP_976063.1 | 399687 | unconventional myosin-XVIIIa isoform b | 6.7 | 2 | 7 | 7 | 11 | 1.06E+08 | 0.44 | 4 | 19.3 | 2039 | 231.1 | 6.2 | Cell growth and/or maintenance | Endoplasmic reticulum;Golgi apparatus;Cytoplasm;Nucleus | Structural molecule activity | Endoplasmic reticulum;Nucleus |
| DHTKD1 | NP_061176.3 | 55526 | probable 2-oxoglutarate dehydrogenase E1 component DHKTD1, mitochondrial | 6.2 | 1 | 3 | 3 | 3 | 4.42E+07 | 0.43 | 1 | - | 919 | 103 | 6.9 | Metabolism;Energy pathways | - | Oxidoreductase activity | - |
| NOLC1 | NP_004732.2 | 9221 | nucleolar and coiled-body phosphoprotein 1 isoform 2 | 20.0 | 2 | 1 | 14 | 58 | 9.74E+08 | 0.43 | 2 | 6.7 | 699 | 73.6 | 9.5 | Regulation of nucleobase, nucleoside, nucleotide and nucleic acid metabolism | Nucleolus;Cytoplasm | Transcription factor activity | Nucleolus |
| UHRF1 | NP_001276980.1 | 29128 | E3 ubiquitin-protein ligase UHRF1 isoform 1 | 19.2 | 2 | 10 | 10 | 13 | 1.57E+08 | 0.43 | 4 | 23.3 | 793 | 89.8 | 7.6 | Regulation of nucleobase, nucleoside, nucleotide and nucleic acid metabolism | Nucleus;Mitochondrion | DNA binding | Nucleus |
| NOA1 | NP_115689.1 | 84273 | nitric oxide-associated protein 1 | 5.3 | 1 | 2 | 2 | 3 | 4.85E+07 | 0.43 | 2 | 26.4 | 698 | 78.4 | 8.7 | Biological_process unknown | - | Molecular function unknown | - |
| SDC1 | NP_001006947.1 | 6382 | syndecan-1 precursor | 15.5 | 1 | 3 | 3 | 21 | 4.35E+08 | 0.43 | 7 | 21.9 | 310 | 32.5 | 4.6 | Cell communication;Signal transduction;Cell surface receptor linked signal transduction;Cell adhesion | Plasma membrane | Receptor activity | Plasma membrane |
| ATP2A2 | NP_001672.1 | 488 | sarcoplasmic/endoplasmic reticulum calcium ATPase 2 isoform a | 42.1 | 11 | 41 | 41 | 129 | 1.19E+09 | 0.43 | 42 | 26.2 | 997 | 109.6 | 5.4 | Transport | Endoplasmic reticulum;Cytoplasm;Mitochondrion;Nucleus | Auxiliary transport protein activity | Endoplasmic reticulum;Nucleus;Cytoplasm |
| LMO7 | NP_005349.3 | 4008 | LIM domain only protein 7 isoform 1 | 16.7 | 2 | 15 | 15 | 18 | 7.21E+07 | 0.43 | 9 | 45.9 | 1349 | 153.6 | 7.8 | Regulation of nucleobase, nucleoside, nucleotide and nucleic acid metabolism | Nucleus;Cytoplasm | Transcription regulator activity | Nucleus |
| TECR | NP_612510.1 | 9524 | very-long-chain enoyl-CoA reductase | 21.8 | 1 | 9 | 9 | 28 | 3.46E+08 | 0.43 | 10 | 54.2 | 308 | 36 | 9.5 | Metabolism;Energy pathways | Endoplasmic reticulum;Mitochondrion | Catalytic activity | Endoplasmic reticulum |
| DNM1 | NP_001005336.1 | 1759 | dynamin-1 isoform 2 | 14.0 | 4 | 3 | 7 | 13 | 1.76E+08 | 0.43 | 1 | - | 851 | 96 | 7.0 | Cell communication;Signal transduction | Endocytic vesicle;Mitochondrion;Cytoplasmic vesicle;Cytoskeleton;Cell surface;Perinuclear region;Microtubule;Cytoplasm | GTPase activity | Mitochondrion |
| EXOSC10 | NP_002676.1 | 5394 | exosome component 10 isoform 2 | 7.9 | 2 | 3 | 3 | 4 | 3.60E+07 | 0.43 | 1 | - | 860 | 98 | 8.4 | Biological_process unknown | Nucleolus;Nucleoplasm | Protein serine/threonine kinase activity | Nucleolus |
| SEC63 | NP_009145.1 | 11231 | translocation protein SEC63 homolog | 16.1 | 1 | 8 | 8 | 12 | 7.72E+07 | 0.43 | 5 | 53 | 760 | 87.9 | 5.3 | Transport | Endoplasmic reticulum;Mitochondrion | Transporter activity | Endoplasmic reticulum |
| MYCBP2 | NP_055872.4 | 23077 | E3 ubiquitin-protein ligase MYCBP2 | 0.7 | 1 | 2 | 2 | 2 | 1.58E+07 | 0.43 | 1 | - | 4678 | 513.3 | 7.0 | Regulation of nucleobase, nucleoside, nucleotide and nucleic acid metabolism | Nucleus;Cytoplasm | Transcription regulator activity | Nucleus |
| IMPDH2 | NP_000875.2 | 3615 | inosine-5'-monophosphate dehydrogenase 2 | 56.4 | 1 | 20 | 21 | 99 | 1.53E+09 | 0.43 | 32 | 28.1 | 514 | 55.8 | 6.9 | Metabolism;Energy pathways | Cytosol;Cytoplasm | Catalytic activity | Cytosol;Cytoplasm |
| SMAD4 | NP_005350.1 | 4089 | mothers against decapentaplegic homolog 4 | 3.4 | 1 | 1 | 1 | 1 | 1.28E+07 | 0.43 | 1 | - | 552 | 60.4 | 7.0 | Regulation of nucleobase, nucleoside, nucleotide and nucleic acid metabolism | Nucleus;Cytoplasm | Transcription factor activity | Nucleus |
| TPMT | NP_000358.1 | 7172 | thiopurine S-methyltransferase | 6.1 | 1 | 1 | 1 | 1 | 9.46E+06 | 0.43 | 1 | - | 245 | 28.2 | 6.2 | Metabolism;Energy pathways | Cytoplasm | Methyltransferase activity | Cytoplasm |
| GSDMD | NP_079012.3 | 79792 | gasdermin-D | 20.7 | 1 | 5 | 5 | 7 | 1.23E+08 | 0.43 | 2 | 10.3 | 484 | 52.8 | 5.1 | Biological_process unknown | - | Molecular function unknown | - |
| NDC1 | NP_001162023.1 | 55706 | nucleoporin NDC1 isoform 2 | 6.6 | 2 | 3 | 3 | 3 | 3.99E+06 | 0.42 | 1 | - | 634 | 71.9 | 9.1 | Protein transport | Nuclear membrane;Endoplasmic reticulum | Structural constituent of nuclear pore | Nuclear membrane |
| RAB3D | NP_004274.1 | 9545 | ras-related protein Rab-3D | 15.5 | 15 | 2 | 3 | 12 | 3.18E+08 | 0.42 | 2 | 18 | 219 | 24.3 | 4.9 | Transport | Secretory granule;Cytoplasm;Plasma membrane;Zymogen granule | GTPase activity | Secretory granule |
| HEATR1 | NP_060542.4 | 55127 | HEAT repeat-containing protein 1 | 16.4 | 1 | 22 | 22 | 35 | 1.10E+08 | 0.42 | 18 | 41.3 | 2144 | 242.2 | 6.5 | Biological_process unknown | - | Molecular function unknown | - |
| UTP6 | NP_060898.2 | 55813 | U3 small nucleolar RNA-associated protein 6 homolog | 12.9 | 1 | 5 | 5 | 7 | 1.51E+08 | 0.42 | 3 | 19.9 | 597 | 70.1 | 7.3 | Biological_process unknown | Nucleolus | Molecular function unknown | Nucleolus |
| LAMA5 | NP_005551.3 | 3911 | laminin subunit alpha-5 precursor | 4.2 | 1 | 7 | 9 | 9 | 1.03E+08 | 0.42 | 5 | 51.4 | 3695 | 399.5 | 7.0 | Cell growth and/or maintenance | Extracellular;Plasma membrane;Cytoplasm | Cell adhesion molecule activity | Extracellular |
| WDR3 | NP_006775.1 | 10885 | WD repeat-containing protein 3 | 13.9 | 1 | 8 | 8 | 11 | 8.49E+07 | 0.42 | 4 | 24.2 | 943 | 106 | 6.6 | Biological_process unknown | Nucleus;Nucleolus | Molecular function unknown | Nucleus |

| Gene Symbol | RefSeq_Accession | Entrez Gene ID | Description | Coverage (%) | Proteins | Unique Peptides | # Peptides | PSM | Area | SCC-R /SCC-S | SCC-R /SCC-S (Count) | SCC-R /SCC-S (Variability [%]) | # AAs | MW [kDa] | calc. pI | Biological Process | Localization | Molecular Function | Primary Localization |
|---|---|---|---|---|---|---|---|---|---|---|---|---|---|---|---|---|---|---|---|
| GSTP1 | NP_000843.1 | 2950 | glutathione S-transferase P | 71.9 | 1 | 21 | 21 | 521 | 1.67E+10 | 0.42 | 59 | 24.1 | 210 | 23.3 | 5.6 | Metabolism;Energy pathways | Cytoplasm;Nucleus;Mitochondrion | Glutathione transferase activity | Cytoplasm |
| NARR | NP_001243210.1 | 100861437 | ras-related protein Rab-34, isoform NARR | 9.1 | 1 | 1 | 1 | 1 | 1.02E+08 | 0.42 | 1 | - | 198 | 21.1 | 12.2 | - | - | - | - |
| PDS5A | NP_001093869.1 | 23244 | sister chromatid cohesion protein PDS5 homolog A isoform 1 | 27.2 | 2 | 23 | 23 | 49 | 2.82E+08 | 0.42 | 15 | 51.1 | 1337 | 150.7 | 7.9 | Apoptosis | Nucleus;Mitochondrion | Molecular function unknown | Nucleus |
| TSTA3 | NP_003304.1 | 7264 | GDP-L-fucose synthase | 21.8 | 1 | 4 | 4 | 9 | 2.00E+08 | 0.42 | 5 | 11.3 | 321 | 35.9 | 6.6 | Metabolism;Energy pathways | Cytoplasm | Oxidoreductase activity | Cytoplasm |
| THADA | NP_071348.3 | 63892 | thyroid adenoma-associated protein isoform a | 3.0 | 1 | 3 | 3 | 3 | 2.10E+07 | 0.42 | 3 | 22.5 | 1953 | 219.5 | 6.1 | Biological_process unknown | - | Receptor binding | - |
| IGFBP7 | NP_001240764.1 | 3490 | insulin-like growth factor-binding protein 7 isoform 2 precursor | 11.8 | 2 | 2 | 2 | 3 | 1.91E+07 | 0.42 | 2 | 32.8 | 279 | 28.8 | 8.1 | Cell communication;Signal transduction | Extracellular;Plasma membrane;Cytoplasm | Cell adhesion molecule activity | Extracellular |
| MRM1 | NP_079140.2 | 79922 | rRNA methyltransferase 1, mitochondrial precursor | 6.5 | 1 | 2 | 2 | 2 | 3.28E+07 | 0.42 | 1 | - | 353 | 38.6 | 7.9 | Biological_process unknown | - | Methyltransferase activity | - |
| NOLC1 | NP_001271318.1 | 9221 | nucleolar and coiled-body phosphoprotein 1 isoform 3 | 20.1 | 1 | 1 | 14 | 49 | 9.74E+08 | 0.42 | 2 | 42.8 | 700 | 73.7 | 9.5 | Regulation of nucleobase, nucleoside, nucleotide and nucleic acid metabolism | Nucleolus;Cytoplasm | Transcription factor activity | Nucleolus |
| SRPRB | NP_067026.3 | 58477 | signal recognition particle receptor subunit beta | 54.6 | 1 | 11 | 11 | 23 | 2.85E+08 | 0.42 | 14 | 17.4 | 271 | 29.7 | 9.0 | Protein metabolism | Cytoplasm;Endoplasmic reticulum;Mitochondrion;Nucleus | GTPase activity | Endoplasmic reticulum |
| CDC73 | NP_078805.3 | 79577 | parafibromin | 19.2 | 1 | 9 | 9 | 18 | 1.54E+08 | 0.42 | 3 | 9.1 | 531 | 60.5 | 9.6 | Biological_process unknown | Nucleolus;Nucleus;Nuclear membrane | Molecular function unknown | Nucleolus;Nucleus |
| NIPBL | NP_056199.2 | 25836 | nipped-B-like protein isoform B | 2.5 | 2 | 4 | 4 | 6 | 1.34E+08 | 0.41 | 3 | 42.9 | 2697 | 304.2 | 7.8 | Regulation of nucleobase, nucleoside, nucleotide and nucleic acid metabolism | Nucleus;Nucleolus;Cytoplasm | DNA repair protein | Nucleus |
| PAPSS2 | NP_004661.2 | 9060 | bifunctional 3'-phosphoadenosine 5'-phosphosulfate synthase 2 isoform a | 33.9 | 2 | 10 | 11 | 20 | 1.35E+08 | 0.41 | 6 | 26.7 | 614 | 69.5 | 8.0 | Metabolism | Cytoplasm;Nucleus | Ligase activity | Cytoplasm |
| BTAF1 | NP_003963.1 | 9044 | TATA-binding protein-associated factor 172 | 4.2 | 1 | 4 | 4 | 4 | 8.14E+06 | 0.41 | 1 | - | 1849 | 206.8 | 6.5 | Regulation of nucleobase, nucleoside, nucleotide and nucleic acid metabolism | Nucleus | Transcription regulator activity | Nucleus |
| KIF23 | NP_004847.2 | 9493 | kinesin-like protein KIF23 isoform 2 | 8.6 | 3 | 4 | 4 | 5 | 2.77E+07 | 0.41 | 1 | - | 856 | 98 | 8.5 | Cell growth and/or maintenance | Nucleus;Cytoplasm;Microtubule;Chromosome | Motor activity | Nucleus |
| ZMIZ1 | NP_065071.1 | 57178 | zinc finger MIZ domain-containing protein 1 | 3.2 | 3 | 2 | 2 | 2 | 5.04E+07 | 0.41 | 2 | 97.5 | 1067 | 115.4 | 7.5 | Regulation of nucleobase, nucleoside, nucleotide and nucleic acid metabolism | Nucleus | Molecular function unknown | Nucleus |
| FAM208A | NP_056039.2 | 23272 | protein FAM208A isoform b | 0.7 | 2 | 1 | 1 | 1 | 2.29E+07 | 0.41 | 1 | - | 1233 | 139.9 | 5.6 | Cell communication;Signal transduction | Nucleus | Molecular function unknown | Nucleus |
| KPNA7 | NP_001139187.1 | 402569 | importin subunit alpha-8 | 2.1 | 1 | 1 | 1 | 1 | 4.98E+08 | 0.41 | 1 | - | 516 | 56.9 | 6.0 | Transport | - | Transporter activity | - |
| MRPL47 | NP_065142.2 | 57129 | 39S ribosomal protein L47, mitochondrial isoform a | 12.8 | 2 | 3 | 3 | 4 | 6.36E+07 | 0.41 | 1 | - | 250 | 29.4 | 10.4 | Protein metabolism | Mitochondrion | Structural constituent of ribosome | Mitochondrion |
| ARHGEF16 | NP_055263.2 | 27237 | rho guanine nucleotide exchange factor 16 | 2.5 | 1 | 1 | 1 | 2 | 8.17E+07 | 0.41 | 1 | - | 709 | 80.1 | 7.4 | Cell communication;Signal transduction | - | Guanyl-nucleotide exchange factor activity | - |
| C9orf114 | NP_057474.2 | 51490 | uncharacterized protein C9orf114 | 14.4 | 1 | 3 | 3 | 3 | 1.83E+07 | 0.41 | 1 | - | 376 | 42 | 7.4 | Biological_process unknown | Nucleolus | Molecular function unknown | Nucleolus |
| PCCA | NP_001171475.1 | 5095 | propionyl-CoA carboxylase alpha chain, mitochondrial isoform c precursor | 6.0 | 3 | 2 | 2 | 2 | 9.28E+07 | 0.41 | 1 | - | 681 | 75 | 7.4 | Metabolism;Energy pathways | Mitochondrion;Extracellular | Ligase activity | Mitochondrion |
| RNMTL1 | NP_060616.1 | 55178 | RNA methyltransferase-like protein 1 | 13.1 | 1 | 3 | 3 | 4 | 1.76E+07 | 0.41 | 3 | 26.3 | 420 | 47 | 8.7 | Regulation of nucleobase, nucleoside, nucleotide and nucleic acid metabolism | - | RNA methyltransferase activity | - |
| GCAT | NP_055106.1 | 23464 | 2-amino-3-ketobutyrate coenzyme A ligase, mitochondrial isoform 2 precursor | 20.1 | 2 | 4 | 4 | 9 | 6.62E+07 | 0.41 | 3 | 2 | 419 | 45.3 | 8.1 | Metabolism;Energy pathways | Mitochondrion | Acyltransferase activity | Mitochondrion |
| CDKAL1 | NP_060244.2 | 54901 | threonylcarbamoyladenosine tRNA methylthiotransferase | 6.6 | 1 | 2 | 2 | 3 | 8.13E+06 | 0.41 | 1 | - | 579 | 65.1 | 7.5 | Biological_process unknown | Integral to membrane | Molecular function unknown | Integral to membrane |
| STRN | NP_003153.2 | 6801 | striatin | 22.3 | 1 | 8 | 8 | 15 | 1.37E+08 | 0.41 | 4 | 8.1 | 780 | 86.1 | 5.3 | Cell communication;Signal transduction | Cytoplasm;Plasma membrane | Molecular function unknown | Cytoplasm |
| HSD17B12 | NP_057226.1 | 51144 | estradiol 17-beta-dehydrogenase 12 | 41.0 | 1 | 9 | 9 | 49 | 5.59E+08 | 0.40 | 11 | 26.9 | 312 | 34.3 | 9.3 | Metabolism;Energy pathways | Endoplasmic reticulum;Mitochondrion | Catalytic activity | Endoplasmic reticulum |
| SMARCA4 | NP_001122320.1 | 6597 | transcription activator BRG1 isoform F | 8.1 | 9 | 4 | 9 | 13 | 4.52E+07 | 0.40 | 2 | 1 | 1613 | 181.1 | 8.0 | Regulation of nucleobase, nucleoside, nucleotide and nucleic acid metabolism | Nucleus;Nucleolus;Kinetochore | Transcription factor activity | Nucleus |
| TMEM259 | NP_219488.1 | 91304 | membralin isoform 2 | 13.0 | 2 | 3 | 3 | 4 | 3.07E+07 | 0.40 | 1 | - | 408 | 46.4 | 5.6 | Biological_process unknown | Integral to membrane | Molecular function unknown | Integral to membrane |
| BAZ1B | NP_115784.1 | 9031 | tyrosine-protein kinase BAZ1B | 11.3 | 1 | 9 | 9 | 11 | 1.95E+08 | 0.40 | 6 | 30.9 | 1483 | 170.8 | 8.5 | Regulation of nucleobase, nucleoside, nucleotide and nucleic acid metabolism;Regulation of gene expression, epigenetic | Nucleus;Nucleolus;Cytoplasm | Transcription regulator activity | Nucleus;Cytoplasm |
| MRPL27 | NP_057588.1 | 51264 | 39S ribosomal protein L27, mitochondrial | 10.1 | 1 | 1 | 1 | 2 | 6.69E+07 | 0.40 | 1 | - | 148 | 16.1 | 10.4 | Protein metabolism | Mitochondrion | Structural constituent of ribosome | Mitochondrion |
| UBR3 | NP_742067.3 | 130507 | E3 ubiquitin-protein ligase UBR3 | 0.9 | 1 | 1 | 1 | 1 | 1.20E+07 | 0.40 | 1 | - | 1888 | 212.3 | 6.1 | Biological_process unknown | - | Molecular function unknown | - |
| EP400 | NP_056224.3 | 57634 | E1A-binding protein p400 | 2.0 | 1 | 4 | 4 | 4 | 2.13E+07 | 0.40 | 2 | 80.8 | 3123 | 339.6 | 9.2 | Regulation of nucleobase, nucleoside, nucleotide and nucleic acid metabolism | Nucleus | DNA binding | Nucleus |
| DHX57 | NP_945314.1 | 90957 | putative ATP-dependent RNA helicase DHX57 | 3.3 | 1 | 2 | 2 | 3 | 4.72E+07 | 0.40 | 2 | 7.6 | 1386 | 155.5 | 7.7 | Regulation of nucleobase, nucleoside, nucleotide and nucleic acid metabolism | Nucleus;Cytoplasm | Molecular function unknown | Nucleus;Cytoplasm |
| SFT2D2 | NP_955376.1 | 375035 | vesicle transport protein SFT2B | 6.3 | 1 | 1 | 1 | 2 | 8.60E+06 | 0.40 | 1 | - | 160 | 17.8 | 9.1 | Biological_process unknown | - | Molecular function unknown | - |
| CAPN7 | NP_055111.1 | 23473 | calpain-7 | 2.2 | 1 | 1 | 1 | 1 | 9.40E+06 | 0.40 | 1 | - | 813 | 92.6 | 7.7 | Protein metabolism | Nucleus | Cysteine-type peptidase activity | Nucleus |
| TRUB1 | NP_631908.1 | 142940 | probable tRNA pseudouridine synthase 1 | 14.3 | 1 | 3 | 3 | 5 | 3.73E+07 | 0.40 | 3 | 0.2 | 349 | 37.2 | 8.3 | Metabolism;Energy pathways | - | Lyase activity | - |
| ZHX3 | NP_055850.1 | 23051 | zinc fingers and homeoboxes protein 3 | 2.3 | 1 | 1 | 1 | 1 | 4.77E+07 | 0.40 | 1 | - | 956 | 104.6 | 6.1 | Regulation of nucleobase, nucleoside, nucleotide and nucleic acid metabolism | Nucleus;Cytoplasm | Transcription regulator activity | Nucleus |

| Gene Symbol | RefSeq_Accession | Entrez Gene ID | Description | Coverage (%) | Proteins | Unique Peptides | # Peptides | PSM | Area | SCC-R /SCC-S | SCC-R /SCC-S (Count) | SCC-R /SCC-S (Variability [%]) | # AAs | MW [kDa] | calc. pI | Biological Process | Localization | Molecular Function | Primary Localization |
|---|---|---|---|---|---|---|---|---|---|---|---|---|---|---|---|---|---|---|---|
| ARFGAP2 | NP_001229761.1 | 84364 | ADP-ribosylation factor GTPase-activating protein 2 isoform 2 | 22.9 | 2 | 5 | 5 | 11 | 5.76E+07 | 0.40 | 5 | 44.1 | 493 | 53.4 | 8.5 | Cell communication;Signal transduction | Nucleus;Cytoplasm | GTPase activator activity | Nucleus |
| CAMSAP3 | NP_065953.1 | 57662 | calmodulin-regulated spectrin-associated protein 3 isoform 2 | 5.2 | 2 | 2 | 2 | 3 | 2.54E+07 | 0.39 | 1 | - | 1249 | 134.7 | 8.4 | Biological_process unknown | - | Molecular function unknown | - |
| BTF3 | NP_001198.2 | 689 | transcription factor BTF3 isoform B | 75.9 | 1 | 1 | 9 | 90 | 1.36E+09 | 0.39 | 1 | - | 162 | 17.7 | 7.5 | Regulation of nucleobase, nucleoside, nucleotide and nucleic acid metabolism | Nucleus | Transcription factor activity | Nucleus |
| FER | NP_005237.2 | 2241 | tyrosine-protein kinase Fer | 4.0 | 1 | 2 | 2 | 3 | 6.52E+06 | 0.39 | 1 | - | 822 | 94.6 | 7.1 | Cell communication;Signal transduction | Cytoplasm;Nucleus | Protein-tyrosine kinase activity | Cytoplasm |
| LLGL1 | NP_004131.3 | 3996 | lethal(2) giant larvae protein homolog 1 | 8.9 | 1 | 5 | 5 | 7 | 8.27E+07 | 0.39 | 4 | 46.1 | 1064 | 115.3 | 6.3 | Cell growth and/or maintenance | Cytoplasm;Plasma membrane;Cell junction | Structural constituent of cytoskeleton | Cytoplasm;Plasma membrane;Cell junction |
| SUN2 | NP_001186509.1 | 25777 | SUN domain-containing protein 2 isoform b | 10.0 | 2 | 3 | 3 | 5 | 1.03E+08 | 0.39 | 3 | 62.6 | 717 | 80.3 | 6.7 | Cell organization and biogenesis | Integral to membrane;Nucleus;Cytoplasm | Molecular function unknown | Integral to membrane |
| MYBBP1A | NP_055335.2 | 10514 | myb-binding protein 1A isoform 2 | 27.3 | 2 | 28 | 28 | 75 | 3.45E+08 | 0.39 | 34 | 24.2 | 1328 | 148.8 | 9.3 | Regulation of nucleobase, nucleoside, nucleotide and nucleic acid metabolism | Nucleolus;Nucleus | Transcription regulator activity | Nucleolus |
| SSH3 | NP_060327.3 | 54961 | protein phosphatase Slingshot homolog 3 | 16.4 | 1 | 6 | 6 | 13 | 1.56E+08 | 0.39 | 6 | 35.4 | 659 | 73 | 5.3 | Cell growth and/or maintenance | Cytoplasm;Nucleus | Protein tyrosine/serine/threonine phosphatase activity | Cytoplasm |
| CKB | NP_001814.2 | 1152 | creatine kinase B-type | 17.3 | 1 | 2 | 2 | 2 | 7.95E+07 | 0.39 | 1 | - | 381 | 42.6 | 5.6 | Metabolism;Energy pathways | Cytoplasm | Catalytic activity | Cytoplasm |
| RNASEH1 | NP_001273763.1 | 246243 | ribonuclease H1 isoform 2 | 13.5 | 3 | 2 | 2 | 3 | 8.08E+06 | 0.39 | 1 | - | 260 | 29.2 | 8.6 | Regulation of nucleobase, nucleoside, nucleotide and nucleic acid metabolism | Nucleus;Mitochondrion | Ribonuclease activity | Nucleus |
| HIST1H1E | NP_005312.1 | 3008 | histone H1.4 | 30.1 | 3 | 3 | 11 | 140 | 8.04E+09 | 0.39 | 5 | 12.2 | 219 | 21.9 | 11.0 | Regulation of nucleobase, nucleoside, nucleotide and nucleic acid metabolism | Nucleus;Nucleolus | DNA binding | Nucleus |
| VPS16 | NP_536338.1 | 64601 | vacuolar protein sorting-associated protein 16 homolog isoform 3 | 6.5 | 2 | 3 | 3 | 5 | 1.06E+09 | 0.39 | 1 | - | 695 | 78.3 | 7.9 | Transport | Late endosome;Lysosome;Membrane fraction;Cytoplasm | Transporter activity | Late endosome |
| WDR36 | NP_644810.1 | 134430 | WD repeat-containing protein 36 | 12.8 | 1 | 6 | 6 | 10 | 1.21E+08 | 0.39 | 2 | 39.5 | 951 | 105.3 | 7.5 | Biological_process unknown | Nucleolus | Molecular function unknown | Nucleolus |
| GTF3C3 | NP_036218.1 | 9330 | general transcription factor 3C polypeptide 3 isoform 1 | 8.4 | 2 | 4 | 4 | 4 | 1.24E+07 | 0.39 | 3 | 18.5 | 886 | 101.2 | 5.1 | Regulation of nucleobase, nucleoside, nucleotide and nucleic acid metabolism | Nucleus | Transcription factor activity | Nucleus |
| MYO1E | NP_004989.2 | 4643 | unconventional myosin-Ie | 12.1 | 2 | 8 | 8 | 11 | 1.39E+08 | 0.39 | 5 | 42 | 1108 | 127 | 8.9 | Cell growth and/or maintenance | Cytoplasm | Motor activity | Cytoplasm |
| MLEC | NP_055545.1 | 9761 | malectin precursor | 14.7 | 1 | 3 | 3 | 5 | 2.06E+07 | 0.38 | 3 | 55.5 | 292 | 32.2 | 5.4 | Biological_process unknown | Integral to membrane | Molecular function unknown | Integral to membrane |
| ALDH18A1 | NP_002851.2 | 5832 | delta-1-pyrroline-5-carboxylate synthase isoform 1 | 38.2 | 2 | 23 | 23 | 58 | 6.70E+08 | 0.38 | 22 | 22.9 | 795 | 87.2 | 7.1 | Metabolism;Energy pathways;Amino acid and derivative metabolism | Mitochondrion | Ligase activity | Mitochondrion |
| CYP51A1 | NP_000777.1 | 1595 | lanosterol 14-alpha demethylase isoform 1 precursor | 20.0 | 2 | 6 | 6 | 7 | 1.62E+08 | 0.38 | 3 | 2.6 | 509 | 57.2 | 8.5 | Metabolism;Energy pathways | Endoplasmic reticulum | Molecular function unknown | Endoplasmic reticulum |
| ASAP2 | NP_001128663.1 | 8853 | arf-GAP with SH3 domain, ANK repeat and PH domain-containing protein 2 isoform b | 1.7 | 2 | 1 | 1 | 1 | 3.24E+07 | 0.38 | 1 | - | 961 | 106.9 | 6.5 | Cell communication;Signal transduction | Cytoplasm;Plasma membrane;Golgi apparatus | GTPase activator activity | Golgi apparatus;Cytoplasm |
| ATF2 | NP_001243023.1 | 1386 | cyclic AMP-dependent transcription factor ATF-2 isoform 5 | 7.2 | 3 | 1 | 1 | 2 | 2.01E+07 | 0.38 | 1 | - | 209 | 23 | 6.2 | Regulation of nucleobase, nucleoside, nucleotide and nucleic acid metabolism | Nucleus;Cytoplasm | Transcription factor activity | Nucleus |
| CAT | NP_001743.1 | 847 | catalase | 11.2 | 1 | 3 | 3 | 7 | 9.65E+07 | 0.38 | 3 | 6.7 | 527 | 59.7 | 7.4 | Metabolism;Energy pathways | Cytoplasm;Mitochondrion;Peroxisome | Oxidoreductase activity | Cytoplasm |
| PTBP3 | NP_001157260.1 | 9991 | polypyrimidine tract-binding protein 3 isoform 2 | 37.2 | 7 | 8 | 13 | 54 | 1.55E+09 | 0.38 | 6 | 4.8 | 524 | 56.8 | 9.2 | Regulation of nucleobase, nucleoside, nucleotide and nucleic acid metabolism | Nucleus | RNA binding | Nucleus |
| PTK7 | NP_002812.2 | 5754 | inactive tyrosine-protein kinase 7 isoform a precursor | 15.1 | 5 | 7 | 7 | 7 | 1.76E+08 | 0.38 | 3 | 42.3 | 1070 | 118.3 | 7.1 | Cell communication;Signal transduction | Plasma membrane | Transmembrane receptor protein tyrosine kinase activity | Plasma membrane |
| ALDH5A1 | NP_001071.1 | 7915 | succinate-semialdehyde dehydrogenase, mitochondrial isoform 2 precursor | 6.4 | 2 | 2 | 2 | 3 | 2.37E+07 | 0.38 | 1 | - | 535 | 57.2 | 8.3 | Metabolism;Energy pathways | Mitochondrion;Nucleus | Catalytic activity | Mitochondrion |
| C9orf142 | NP_899064.1 | 286257 | uncharacterized protein C9orf142 | 11.8 | 1 | 2 | 2 | 2 | 3.49E+07 | 0.38 | 2 | 4 | 204 | 21.6 | 5.5 | Biological_process unknown | Nucleus;Cytoplasm | Molecular function unknown | Nucleus;Cytoplasm |
| AGPAT5 | NP_060831.2 | 55326 | 1-acyl-sn-glycerol-3-phosphate acyltransferase epsilon | 3.3 | 1 | 1 | 1 | 1 | 1.63E+08 | 0.38 | 1 | - | 364 | 42 | 9.1 | Metabolism;Energy pathways | Integral to membrane | Transferase activity | Integral to membrane |
| RDH11 | NP_057110.3 | 51109 | retinol dehydrogenase 11 isoform 1 precursor | 25.8 | 2 | 7 | 7 | 23 | 2.37E+08 | 0.38 | 7 | 23.8 | 318 | 35.4 | 8.8 | Metabolism;Energy pathways | Endoplasmic reticulum | Catalytic activity | Endoplasmic reticulum |
| OAT | NP_000265.1 | 4942 | ornithine aminotransferase, mitochondrial isoform 1 precursor | 66.7 | 2 | 21 | 21 | 119 | 1.14E+09 | 0.38 | 27 | 27.4 | 439 | 48.5 | 7.0 | Metabolism;Energy pathways | Mitochondrion | Transaminase activity | Mitochondrion |
| ASCC3 | NP_006819.2 | 10973 | activating signal cointegrator 1 complex subunit 3 isoform a | 10.3 | 3 | 12 | 12 | 14 | 8.34E+07 | 0.38 | 5 | 22.6 | 2202 | 251.3 | 7.1 | Regulation of nucleobase, nucleoside, nucleotide and nucleic acid metabolism | Nucleus | Molecular function unknown | Nucleus |
| CAD | NP_004332.2 | 790 | CAD protein | 46.4 | 1 | 57 | 57 | 144 | 8.41E+08 | 0.38 | 45 | 25.9 | 2225 | 242.8 | 6.5 | Metabolism;Energy pathways | Cytoplasm;Nucleus | Ligase activity | Cytoplasm |
| LIG3 | NP_002302.2 | 3980 | DNA ligase 3 isoform beta precursor | 1.1 | 2 | 1 | 1 | 2 | 8.71E+07 | 0.38 | 1 | - | 949 | 106 | 9.2 | Regulation of nucleobase, nucleoside, nucleotide and nucleic acid metabolism | Nucleus;Mitochondrion | DNA ligase activity | Nucleus |
| PPIC | NP_000934.1 | 5480 | peptidyl-prolyl cis-trans isomerase C precursor | 10.9 | 1 | 2 | 2 | 6 | 2.06E+08 | 0.38 | 1 | - | 212 | 22.7 | 8.4 | Protein metabolism | - | Chaperone activity | - |
| SLFN13 | NP_653283.3 | 146857 | schlafen family member 13 | 1.1 | 2 | 1 | 1 | 1 | 4.47E+07 | 0.38 | 1 | - | 897 | 102 | 7.0 | Apoptosis | - | Growth factor activity | - |
| TTC27 | NP_001180438.1 | 55622 | tetratricopeptide repeat protein 27 isoform 2 | 11.1 | 2 | 5 | 5 | 9 | 8.24E+07 | 0.38 | 6 | 32.2 | 793 | 90.9 | 5.8 | Biological_process unknown | - | Molecular function unknown | - |
| CBLN4 | NP_542184.1 | 140689 | cerebellin-4 precursor | 3.5 | 1 | 1 | 1 | 1 | 4.82E+07 | 0.38 | 1 | - | 201 | 21.8 | 8.7 | Biological_process unknown | - | Molecular function unknown | - |
| EMB | NP_940851.1 | 133418 | embigin precursor | 4.0 | 1 | 1 | 1 | 1 | 2.58E+07 | 0.38 | 1 | - | 327 | 36.9 | 6.5 | Cell growth and/or maintenance | Plasma membrane | Cell adhesion molecule activity | Plasma membrane |
| FAM8A1 | NP_057339.1 | 51439 | protein FAM8A1 | 6.8 | 1 | 1 | 1 | 1 | 4.21E+07 | 0.38 | 1 | - | 413 | 44.1 | 7.2 | Biological_process unknown | Integral to membrane | Molecular function unknown | Integral to membrane |

| Gene Symbol | RefSeq_Accession | Entrez Gene ID | Description | Coverage (%) | Proteins | Unique Peptides | # Peptides | PSM | Area | SCC-R /SCC-S | SCC-R /SCC-S (Count) | SCC-R /SCC-S (Variability [%]) | # AAs | MW [kDa] | calc. pI | Biological Process | Localization | Molecular Function | Primary Localization |
|---|---|---|---|---|---|---|---|---|---|---|---|---|---|---|---|---|---|---|---|
| **PRIM1** | NP_000937.1 | 5557 | **DNA primase small subunit** | 5.7 | 1 | 2 | 2 | 3 | 1.89E+08 | **0.37** | 2 | 18.6 | 420 | 49.9 | 8.2 | Regulation of nucleobase, nucleoside, nucleotide and nucleic acid metabolism | Nucleus | DNA-directed RNA polymerase activity | Nucleus |
| **TSPAN1** | NP_005718.2 | 10103 | **tetraspanin-1** | 5.4 | 1 | 1 | 1 | 1 | 2.19E+08 | **0.37** | 1 | - | 241 | 26.3 | 5.3 | - | Plasma membrane;Lysosome;Endoplasmic reticulum;Cell surface | - | Plasma membrane;Cell surface |
| **AKAP13** | NP_001257475.1 | 11214 | **A-kinase anchor protein 13 isoform 4** | 1.2 | 3 | 1 | 1 | 1 | 6.13E+06 | **0.37** | 1 | - | 1434 | 161.7 | 7.0 | Cell communication;Signal transduction | Plasma membrane;Cytoplasm;Nucleus | Guanyl-nucleotide exchange factor activity | Plasma membrane |
| **C10orf118** | NP_060487.2 | 55088 | **uncharacterized protein C10orf118** | 1.8 | 1 | 1 | 1 | 2 | 1.82E+08 | **0.37** | 1 | - | 898 | 103.6 | 6.3 | Biological_process unknown | - | Molecular function unknown | - |
| **PBRM1** | NP_060783.3 | 55193 | **protein polybromo-1** | 3.4 | 1 | 4 | 4 | 4 | 1.08E+07 | **0.37** | 1 | - | 1582 | 181 | 6.9 | Regulation of development | Nucleus;Cytoplasm | Chromatin binding | Nucleus |
| **ACSL3** | NP_004448.2 | 2181 | **long-chain-fatty-acid--CoA ligase 3** | 47.8 | 1 | 21 | 24 | 73 | 1.03E+09 | **0.37** | 27 | 44.7 | 720 | 80.4 | 8.4 | Fatty acid metabolism | Mitochondrion;Peroxisome | CoA-ligase activity | Peroxisome |
| **DHCR24** | NP_055577.1 | 1718 | **delta(24)-sterol reductase precursor** | 21.1 | 1 | 11 | 11 | 21 | 6.57E+08 | **0.37** | 7 | 37.3 | 516 | 60.1 | 8.2 | Metabolism;Energy pathways | Plasma membrane;Endoplasmic reticulum | Catalytic activity | Plasma membrane |
| **VRK1** | NP_003375.1 | 7443 | **serine/threonine-protein kinase VRK1** | 10.6 | 1 | 3 | 3 | 6 | 1.37E+08 | **0.37** | 2 | 50.2 | 396 | 45.4 | 8.9 | Cell communication;Signal transduction | Nucleus;Nucleolus | Protein serine/threonine kinase activity | Nucleus |
| **ARL6IP5** | NP_006398.1 | 10550 | **PRA1 family protein 3** | 10.6 | 1 | 2 | 2 | 13 | 2.94E+08 | **0.37** | 4 | 29.5 | 188 | 21.6 | 9.8 | Cell communication;Signal transduction | Cytoplasm;Plasma membrane | Molecular function unknown | Cytoplasm |
| **HDAC6** | NP_006035.2 | 10013 | **histone deacetylase 6** | 6.8 | 1 | 3 | 3 | 6 | 5.56E+07 | **0.37** | 2 | 144.8 | 1215 | 131.3 | 5.3 | Regulation of nucleobase, nucleoside, nucleotide and nucleic acid metabolism | Nucleus;Cytoplasm | Deacetylase activity | Nucleus |
| **MB21D1** | NP_612450.2 | 115004 | **cyclic GMP-AMP synthase** | 2.1 | 1 | 1 | 1 | 1 | 3.12E+07 | **0.37** | 1 | - | 522 | 58.8 | 9.5 | Biological_process unknown | - | Molecular function unknown | - |
| **NPM3** | NP_008924.1 | 10360 | **nucleoplasmin-3** | 62.9 | 1 | 5 | 5 | 19 | 3.38E+08 | **0.37** | 8 | 20.7 | 178 | 19.3 | 4.6 | Protein metabolism | Nucleus;Cytoplasm;Nucleolus | Chaperone activity | Nucleus |
| **TKT** | NP_001055.1 | 7086 | **transketolase isoform 1** | 73.8 | 2 | 38 | 38 | 531 | 4.71E+09 | **0.37** | 92 | 21.8 | 623 | 67.8 | 7.7 | Metabolism;Energy pathways | Cytoplasm | Transferase activity, transferring aldehyde or ketonic groups | Cytoplasm |
| **F3** | NP_001984.1 | 2152 | **tissue factor isoform 1 precursor** | 32.9 | 2 | 8 | 8 | 30 | 7.27E+08 | **0.37** | 8 | 39.8 | 295 | 33 | 7.0 | Protein metabolism | Plasma membrane | Cofactor binding | Plasma membrane |
| **SHMT1** | NP_683718.1 | 6470 | **serine hydroxymethyltransferase, cytosolic isoform 2** | 58.3 | 3 | 17 | 18 | 65 | 1.10E+09 | **0.37** | 23 | 33.8 | 444 | 49 | 7.7 | Metabolism;Energy pathways | Cytoplasm;Mitochondrion | Methyltransferase activity;RNA binding | Cytoplasm |
| **RCC2** | NP_001129676.1 | 55920 | **protein RCC2** | 34.7 | 1 | 12 | 12 | 38 | 6.30E+08 | **0.36** | 17 | 31.5 | 522 | 56 | 8.8 | Cell growth and/or maintenance | Nucleus;Cytoplasm;Mitochondrion | Protein binding | Nucleus |
| **WIZ** | NP_067064.2 | 58525 | **protein Wiz** | 4.2 | 1 | 2 | 2 | 2 | 7.87E+07 | **0.36** | 2 | 1.2 | 794 | 85.3 | 9.1 | Regulation of nucleobase, nucleoside, nucleotide and nucleic acid metabolism | Nucleus | Transcription regulator activity | Nucleus |
| **ALDH3A2** | NP_000373.1 | 224 | **fatty aldehyde dehydrogenase isoform 2** | 47.8 | 6 | 15 | 16 | 32 | 5.81E+08 | **0.36** | 14 | 18.4 | 485 | 54.8 | 7.9 | Metabolism | Endoplasmic reticulum;Microsome | Catalytic activity | Endoplasmic reticulum |
| **FCF1** | NP_057046.1 | 51077 | **rRNA-processing protein FCF1 homolog** | 12.6 | 1 | 2 | 2 | 2 | 3.12E+07 | **0.36** | 1 | - | 198 | 23.4 | 9.7 | Biological_process unknown | Nucleolus | Ribonuclease activity | Nucleolus |
| **LIMA1** | NP_057441.1 | 51474 | **LIM domain and actin-binding protein 1 isoform 2** | 32.4 | 2 | 1 | 14 | 29 | 4.18E+08 | **0.36** | 1 | - | 759 | 85.2 | 6.8 | Cell growth and/or maintenance | Cytoplasm;Cytoskeleton;Nucleus | Structural constituent of cytoskeleton | Cytoplasm |
| **SETX** | NP_055861.3 | 23064 | **probable helicase senataxin** | 1.8 | 2 | 4 | 4 | 4 | 6.63E+07 | **0.36** | 2 | 284.9 | 2677 | 302.7 | 7.2 | Regulation of nucleobase, nucleoside, nucleotide and nucleic acid metabolism | Nucleus;Cytoplasm | Molecular function unknown | Nucleus;Cytoplasm |
| **CD14** | NP_001167575.1 | 929 | **monocyte differentiation antigen CD14 precursor** | 6.1 | 1 | 2 | 2 | 2 | 2.02E+07 | **0.36** | 2 | 61.5 | 375 | 40.1 | 6.2 | Immune response | Extracellular;Endoplasmic reticulum;Golgi apparatus;Lysosome;Endosome;Cell surface;Cytoplasm | Receptor activity | Cell surface |
| **METTL3** | NP_062826.2 | 56339 | **N6-adenosine-methyltransferase 70 kDa subunit** | 2.4 | 1 | 1 | 1 | 1 | 1.36E+06 | **0.36** | 1 | - | 580 | 64.4 | 6.4 | Regulation of nucleobase, nucleoside, nucleotide and nucleic acid metabolism | Nucleus | Methyltransferase activity | Nucleus |
| **IMPDH1** | NP_001136045.1 | 3614 | **inosine-5'-monophosphate dehydrogenase 1 isoform e** | 22.6 | 7 | 8 | 9 | 17 | 4.14E+08 | **0.36** | 5 | 12.3 | 514 | 55.4 | 6.9 | Metabolism;Energy pathways | Cytosol | Catalytic activity | Cytosol |
| **MARK2** | NP_001156769.1 | 2011 | **serine/threonine-protein kinase MARK2 isoform f** | 2.4 | 5 | 1 | 1 | 3 | 3.97E+07 | **0.36** | 1 | - | 709 | 79.4 | 9.6 | Cell communication;Signal transduction | Plasma membrane;Cytoplasm;Cytoskeleton;Nucleus | Protein serine/threonine kinase activity | Plasma membrane |
| **METTL15** | NP_001107000.1 | 196074 | **probable methyltransferase-like protein 15 isoform 1** | 10.8 | 2 | 2 | 2 | 3 | 6.83E+07 | **0.36** | 1 | - | 407 | 46.1 | 8.6 | Regulation of nucleobase, nucleoside, nucleotide and nucleic acid metabolism | - | Methyltransferase activity | - |
| **KAT7** | NP_001186087.1 | 11143 | **histone acetyltransferase KAT7 isoform 5** | 11.1 | 5 | 2 | 2 | 2 | 1.85E+07 | **0.36** | 2 | 220.5 | 442 | 51.4 | 8.9 | Regulation of nucleobase, nucleoside, nucleotide and nucleic acid metabolism;Transcription | Nucleus;Cytoplasm | Acyltransferase activity | Nucleus |
| **SMPD4** | NP_001164554.1 | 55627 | **sphingomyelin phosphodiesterase 4 isoform 3** | 7.2 | 3 | 4 | 4 | 4 | 4.45E+07 | **0.36** | 3 | 27.6 | 764 | 86.1 | 8.7 | Biological_process unknown | Endoplasmic reticulum;Golgi apparatus;Mitochondrion;Plasma membrane;Secretory vesicle | Molecular function unknown | Endoplasmic reticulum |
| **ADK** | NP_006712.2 | 132 | **adenosine kinase isoform b** | 58.0 | 4 | 14 | 14 | 45 | 3.95E+08 | **0.36** | 14 | 23.1 | 362 | 40.5 | 6.7 | Regulation of nucleobase, nucleoside, nucleotide and nucleic acid metabolism | Cytoplasm | Catalytic activity | Cytoplasm |
| **CTNND1** | NP_001078931.1 | 1500 | **catenin delta-1 isoform 1A** | 49.7 | 15 | 36 | 36 | 141 | 2.18E+09 | **0.36** | 38 | 36.4 | 933 | 104.1 | 7.0 | Cell communication;Signal transduction | Cytoplasm;Nucleus | Cell adhesion molecule activity | Cytoplasm;Nucleus |
| **LAMC2** | NP_061486.2 | 3918 | **laminin subunit gamma-2 isoform b precursor** | 9.4 | 2 | 5 | 5 | 8 | 1.64E+08 | **0.36** | 3 | 3.6 | 1111 | 121.5 | 6.4 | Cell growth and/or maintenance | Cytoplasm;Extracellular;Plasma membrane | Structural molecule activity | Cytoplasm |
| **LGMN** | NP_001008530.1 | 5641 | **legumain preproprotein** | 11.6 | 1 | 4 | 4 | 8 | 9.61E+07 | **0.36** | 5 | 30.9 | 433 | 49.4 | 6.6 | Protein metabolism | Lysosome;Mitochondrion | Cysteine-type peptidase activity | Lysosome |
| **RBM47** | NP_001092104.1 | 54502 | **RNA-binding protein 47 isoform a** | 19.2 | 2 | 5 | 5 | 6 | 3.59E+07 | **0.35** | 2 | 48.9 | 593 | 64.1 | 7.7 | Regulation of gene expression, epigenetic | - | RNA binding | - |
| **RIF1** | NP_001171136.1 | 55183 | **telomere-associated protein RIF1 isoform 2** | 6.3 | 2 | 10 | 10 | 18 | 1.30E+08 | **0.35** | 9 | 64.4 | 2446 | 271.5 | 5.4 | Regulation of nucleobase, nucleoside, nucleotide and nucleic acid metabolism | Nucleus | DNA repair protein | Nucleus |
| **UCK2** | NP_036606.2 | 7371 | **uridine-cytidine kinase 2** | 18.4 | 1 | 2 | 3 | 5 | 4.81E+07 | **0.35** | 1 | - | 261 | 29.3 | 6.7 | Regulation of nucleobase, nucleoside, nucleotide and nucleic acid metabolism | Cytoplasm | Catalytic activity | Cytoplasm |
| **VWA8** | NP_001009814.1 | 23078 | **von Willebrand factor A domain-containing protein 8 isoform b precursor** | 6.1 | 2 | 3 | 4 | 4 | 5.80E+07 | **0.35** | 2 | 8.3 | 1039 | 116.9 | 8.2 | Biological_process unknown | - | Molecular function unknown | - |
| **GTF3C1** | NP_001273171.1 | 2975 | **general transcription factor 3C polypeptide 1 isoform 2** | 7.2 | 2 | 7 | 7 | 10 | 1.03E+08 | **0.35** | 5 | 42.1 | 2084 | 236.1 | 7.5 | Regulation of nucleobase, nucleoside, nucleotide and nucleic acid metabolism | Nucleus | Transcription factor activity | Nucleus |

Supplementary Table S4. List of all proteins identified in SCC-S and SCC-R cells using SequestHT and Mascot search algorithms and their gene ontology annotation

| Gene Symbol | RefSeq_Accession | Entrez Gene ID | Description | Coverage (%) | Proteins | Unique Peptides | # Peptides | PSM | Area | SCC-R /SCC-S | SCC-R /SCC-S (Count) | SCC-R /SCC-S (Variability [%]) | # AAs | MW [kDa] | calc. pI | Biological Process | Localization | Molecular Function | Primary Localization |
|---|---|---|---|---|---|---|---|---|---|---|---|---|---|---|---|---|---|---|---|
| **SYNE2** | NP_055995.4 | 23224 | **nesprin-2 isoform 1** | 1.4 | 2 | 5 | 5 | 5 | 2.83E+07 | **0.35** | 2 | 5.9 | 6885 | 795.9 | 5.4 | Cell growth and/or maintenance | Sarcoplasmic reticulum;Plasma membrane;Nucleus;Nucleolus;Cytoplasm | Structural constituent of cytoskeleton | Nucleus;Cytoplasm |
| **ECD** | NP_001129225.1 | 11319 | **protein SGT1 isoform 3** | 6.7 | 3 | 3 | 3 | 8 | 3.02E+07 | **0.35** | 3 | 18 | 601 | 67.7 | 4.8 | Regulation of nucleobase, nucleoside, nucleotide and nucleic acid metabolism | - | Transcription regulator activity | - |
| **CCDC47** | NP_064583.2 | 57003 | **coiled-coil domain-containing protein 47 precursor** | 32.5 | 1 | 8 | 8 | 34 | 3.59E+08 | **0.35** | 9 | 36.5 | 483 | 55.8 | 4.9 | Biological_process unknown | Mitochondrion | Molecular function unknown | Mitochondrion |
| **CRLF3** | NP_057070.3 | 51379 | **cytokine receptor-like factor 3** | 21.7 | 1 | 6 | 6 | 12 | 1.82E+08 | **0.35** | 2 | 12 | 442 | 49.7 | 5.1 | Biological_process unknown | - | Molecular function unknown | - |
| **MICU2** | NP_689939.1 | 221154 | **calcium uptake protein 2, mitochondrial** | 3.9 | 1 | 1 | 1 | 1 | 4.57E+07 | **0.35** | 1 | - | 434 | 49.6 | 9.1 | Cell communication;Signal transduction | Cytoskeleton | Protein serine/threonine kinase activity | Cytoskeleton |
| **DDX52** | NP_008941.3 | 11056 | **probable ATP-dependent RNA helicase DDX52 isoform 1** | 21.0 | 2 | 7 | 7 | 12 | 2.01E+08 | **0.35** | 6 | 50.3 | 599 | 67.4 | 9.7 | Regulation of nucleobase, nucleoside, nucleotide and nucleic acid metabolism | Nucleolus;Nucleus | Helicase activity | Nucleolus |
| **DUS2** | NP_001258692.1 | 54920 | **tRNA-dihydrouridine(20) synthase [NAD(P)+]-like isoform 2** | 3.7 | 2 | 1 | 1 | 2 | 1.22E+08 | **0.34** | 1 | - | 458 | 51.4 | 7.3 | Regulation of translation | Nucleus;Endoplasmic reticulum | RNA binding;Catalytic activity | Nucleus |
| **GPATCH11** | NP_001265434.1 | 253635 | **G patch domain-containing protein 11 isoform 2** | 18.6 | 2 | 1 | 1 | 1 | 4.34E+07 | **0.34** | 1 | - | 156 | 18.6 | 5.0 | Regulation of nucleobase, nucleoside, nucleotide and nucleic acid metabolism | - | RNA binding | - |
| **S100A16** | NP_525127.1 | 140576 | **protein S100-A16** | 76.7 | 1 | 8 | 8 | 29 | 4.43E+08 | **0.34** | 7 | 3.4 | 103 | 11.8 | 6.8 | Cell communication;Signal transduction | Nucleolus | Calcium ion binding | Nucleolus |
| **ACP1** | NP_004291.1 | 52 | **low molecular weight phosphotyrosine protein phosphatase isoform c** | 72.2 | 2 | 4 | 9 | 15 | 4.25E+08 | **0.34** | 2 | 30.7 | 158 | 18 | 6.7 | Metabolism;Energy pathways | Cytoplasm | Acid phosphatase activity | Cytoplasm |
| **KIF1B** | NP_055889.2 | 23095 | **kinesin-like protein KIF1B isoform b** | 1.3 | 1 | 1 | 2 | 2 | 2.98E+07 | **0.34** | 1 | - | 1770 | 199.1 | 5.6 | Cell growth and/or maintenance | Cytoplasmic vesicle;Mitochondrion;Microtubule;Cytoplasm | Motor activity | Cytoplasmic vesicle |
| **MARK3** | NP_001122393.1 | 4140 | **MAP/microtubule affinity-regulating kinase 3 isoform e** | 7.4 | 10 | 3 | 3 | 4 | 7.25E+07 | **0.34** | 1 | - | 659 | 74.1 | 9.9 | Cell communication;Signal transduction | Cytoplasm | Protein serine/threonine kinase activity | Cytoplasm |
| **PCYOX1** | NP_057381.3 | 51449 | **prenylcysteine oxidase 1 precursor** | 24.8 | 1 | 6 | 6 | 12 | 2.54E+08 | **0.34** | 8 | 21 | 505 | 56.6 | 6.2 | Metabolism;Energy pathways | Lysosome | Catalytic activity | Lysosome |
| **TPM4** | NP_001138632.1 | 7171 | **tropomyosin alpha-4 chain isoform 1** | 60.6 | 1 | 2 | 27 | 270 | 8.25E+09 | **0.34** | 2 | 38.2 | 284 | 32.7 | 4.7 | Cell growth and/or maintenance | Cytoskeleton | Structural constituent of cytoskeleton | Cytoskeleton |
| **AGO1** | NP_036331.1 | 26523 | **protein argonaute-1** | 8.2 | 2 | 1 | 4 | 5 | 4.15E+07 | **0.34** | 1 | - | 857 | 97.2 | 9.2 | Protein metabolism | Cytoplasm | Translation regulator activity | Cytoplasm |
| **PTGFRN** | NP_065173.2 | 5738 | **prostaglandin F2 receptor negative regulator precursor** | 3.3 | 1 | 2 | 2 | 2 | 1.51E+08 | **0.34** | 1 | - | 879 | 98.5 | 6.6 | Cell communication;Signal transduction | Endoplasmic reticulum;Golgi apparatus;Endosome | Protein binding | Golgi apparatus |
| **C17orf49** | NP_777553.1 | 124944 | **chromatin complexes subunit BAP18 isoform 2** | 19.8 | 3 | 2 | 2 | 2 | 1.30E+08 | **0.34** | 2 | 38.6 | 172 | 17.9 | 7.3 | Biological_process unknown | Nucleus | Molecular function unknown | Nucleus |
| **PREB** | NP_037520.1 | 10113 | **prolactin regulatory element-binding protein** | 26.9 | 1 | 6 | 6 | 9 | 1.57E+08 | **0.34** | 3 | 21.9 | 417 | 45.4 | 7.9 | Regulation of nucleobase, nucleoside, nucleotide and nucleic acid metabolism | Endoplasmic reticulum | Transcription factor activity | Endoplasmic reticulum |
| **TFAM** | NP_001257711.1 | 7019 | **transcription factor A, mitochondrial isoform 2 precursor** | 30.8 | 2 | 5 | 5 | 21 | 5.08E+08 | **0.33** | 6 | 22.9 | 214 | 25.4 | 9.7 | Regulation of nucleobase, nucleoside, nucleotide and nucleic acid metabolism | Mitochondrion;Nucleus | Transcription factor activity | Mitochondrion |
| **DCAF13** | NP_056235.4 | 25879 | **DDB1- and CUL4-associated factor 13** | 5.0 | 1 | 2 | 2 | 8 | 5.23E+07 | **0.33** | 1 | - | 597 | 67.5 | 9.3 | Regulation of nucleobase, nucleoside, nucleotide and nucleic acid metabolism | Nucleolus | Molecular function unknown | Nucleolus |
| **MICALL1** | NP_203744.1 | 85377 | **MICAL-like protein 1** | 14.1 | 1 | 7 | 7 | 9 | 1.13E+08 | **0.33** | 3 | 32.7 | 863 | 93.4 | 7.3 | Cell communication;Signal transduction | - | Structural constituent of cytoskeleton | - |
| **CHAF1A** | NP_005474.2 | 10036 | **chromatin assembly factor 1 subunit A** | 13.9 | 1 | 6 | 6 | 8 | 5.68E+07 | **0.33** | 3 | 57.3 | 956 | 106.8 | 5.9 | Regulation of nucleobase, nucleoside, nucleotide and nucleic acid metabolism | Nucleus | Chaperone activity | Nucleus |
| **CUTC** | NP_057044.2 | 51076 | **copper homeostasis protein cutC homolog** | 12.5 | 1 | 2 | 2 | 2 | 4.06E+07 | **0.33** | 1 | - | 273 | 29.3 | 8.2 | Biological_process unknown | Nucleolus | Molecular function unknown | Nucleolus |
| **PRKDC** | NP_008835.5 | 5591 | **DNA-dependent protein kinase catalytic subunit isoform 1** | 37.0 | 2 | 121 | 121 | 360 | 1.63E+09 | **0.33** | 126 | 24.8 | 4128 | 468.8 | 7.1 | Cell communication;Signal transduction | Cytoplasm;Nucleus;Nucleolus;Cytosol;Microsome;Mitochondrion | Protein serine/threonine kinase activity;DNA repair protein | Nucleus |
| **SHMT2** | NP_001159831.1 | 6472 | **serine hydroxymethyltransferase, mitochondrial isoform 3** | 64.4 | 3 | 24 | 25 | 90 | 9.72E+08 | **0.33** | 29 | 29.4 | 483 | 53.4 | 8.2 | Metabolism;Energy pathways | Mitochondrion;Nucleolus | Methyltransferase activity | Mitochondrion |
| **PDS5B** | NP_055847.1 | 23047 | **sister chromatid cohesion protein PDS5 homolog B** | 16.5 | 1 | 15 | 15 | 25 | 2.09E+08 | **0.33** | 9 | 29.3 | 1447 | 164.6 | 8.5 | Regulation of nucleobase, nucleoside, nucleotide and nucleic acid metabolism | Nucleus | Transcription factor activity | Nucleus |
| **CCL26** | NP_006063.1 | 10344 | **C-C motif chemokine 26 precursor** | 6.4 | 1 | 1 | 1 | 1 | 1.60E+08 | **0.33** | 1 | - | 94 | 10.6 | 10.2 | Cell communication;Signal transduction | Plasma membrane;Extracellular | Chemokine activity | Extracellular |
| **HMGN3** | NP_004233.1 | 9324 | **high mobility group nucleosome-binding domain-containing protein 3 isoform HMGN3a** | 15.2 | 1 | 1 | 1 | 1 | 7.16E+06 | **0.33** | 1 | - | 99 | 10.7 | 9.7 | Regulation of nucleobase, nucleoside, nucleotide and nucleic acid metabolism | Nucleus | DNA binding | Nucleus |
| **ANKRD13A** | NP_149112.1 | 88455 | **ankyrin repeat domain-containing protein 13A** | 4.2 | 2 | 2 | 2 | 4 | 4.40E+07 | **0.32** | 2 | 102.1 | 590 | 67.6 | 5.0 | Biological_process unknown | - | Molecular function unknown | - |
| **ME1** | NP_002386.1 | 4199 | **NADP-dependent malic enzyme** | 37.9 | 1 | 12 | 12 | 27 | 2.50E+08 | **0.32** | 10 | 27.2 | 572 | 64.1 | 6.1 | Metabolism;Energy pathways | Cytoplasm | Carboxy-lyase activity | Cytoplasm |
| **AJUBA** | NP_001276026.1 | 84962 | **LIM domain-containing protein ajuba isoform 3** | 15.2 | 2 | 3 | 3 | 5 | 7.21E+07 | **0.32** | 2 | 13.8 | 369 | 37.8 | 8.5 | Cell communication;Signal transduction;Cell cycle;Signal complex formation | Centrosome;Plasma membrane;Nucleus;Cytoplasm | Protein binding | Centrosome |
| **ATE1** | NP_001275663.1 | 11101 | **arginyl-tRNA--protein transferase 1 isoform 3** | 5.2 | 5 | 1 | 1 | 5 | 1.13E+08 | **0.32** | 1 | - | 403 | 45.8 | 6.8 | Protein metabolism | Cytoplasm;Nucleus | Acyltransferase activity | Cytoplasm |
| **GTF3C2** | NP_001512.1 | 2976 | **general transcription factor 3C polypeptide 2** | 10.8 | 1 | 4 | 4 | 7 | 9.24E+07 | **0.32** | 4 | 3.8 | 911 | 100.6 | 7.3 | Regulation of nucleobase, nucleoside, nucleotide and nucleic acid metabolism | Nucleus;Cytoplasm | Transcription factor activity | Nucleus |
| **AHCTF1** | NP_056261.4 | 25909 | **protein ELYS** | 7.2 | 1 | 9 | 9 | 10 | 1.03E+08 | **0.32** | 6 | 41.9 | 2275 | 253.3 | 6.6 | Regulation of nucleobase, nucleoside, nucleotide and nucleic acid metabolism | Nucleus;Nuclear membrane | Transcription factor activity | Nucleus |
| **ZC3H11A** | NP_055642.3 | 9877 | **zinc finger CCCH domain-containing protein 11A** | 9.4 | 1 | 5 | 5 | 6 | 9.50E+06 | **0.32** | 1 | - | 810 | 89.1 | 8.4 | Regulation of nucleobase, nucleoside, nucleotide and nucleic acid metabolism | Nucleus;Cytosol | DNA binding | Nucleus |
| **DHCR7** | NP_001351.2 | 1717 | **7-dehydrocholesterol reductase** | 4.6 | 1 | 2 | 2 | 14 | 3.10E+08 | **0.32** | 4 | 8 | 475 | 54.5 | 8.7 | Metabolism;Energy pathways | Peroxisome;Endoplasmic reticulum | Catalytic activity | Peroxisome |
| **NAPRT1** | NP_001273758.1 | 93100 | **nicotinate phosphoribosyltransferase isoform 2** | 24.0 | 2 | 7 | 7 | 11 | 1.19E+08 | **0.32** | 4 | 46 | 525 | 56.1 | 5.7 | Regulation of nucleobase, nucleoside, nucleotide and nucleic acid metabolism | Cytoplasm | Catalytic activity | Cytoplasm |

| Gene Symbol | RefSeq_Accession | Entrez Gene ID | Description | Coverage (%) | Proteins | Unique Peptides | # Peptides | PSM | Area | SCC-R /SCC-S | SCC-R /SCC-S (Count) | SCC-R /SCC-S (Variability [%]) | # AAs | MW [kDa] | calc. pI | Biological Process | Localization | Molecular Function | Primary Localization |
|---|---|---|---|---|---|---|---|---|---|---|---|---|---|---|---|---|---|---|---|
| ZFAND5 | NP_001095891.1 | 7763 | AN1-type zinc finger protein 5 | 8.5 | 1 | 1 | 1 | 2 | 1.67E+07 | 0.32 | 1 | - | 213 | 23.1 | 8.5 | Regulation of nucleobase, nucleoside, nucleotide and nucleic acid metabolism | Nucleus;Cytoplasm | DNA binding | Cytoplasm |
| NAF1 | NP_001122403.1 | 92345 | H/ACA ribonucleoprotein complex non-core subunit NAF1 isoform b | 3.9 | 2 | 1 | 1 | 2 | 9.79E+06 | 0.32 | 1 | - | 389 | 41.9 | 4.7 | Biological_process unknown | Cytoplasm | Molecular function unknown | Cytoplasm |
| CUX1 | NP_853530.2 | 1523 | protein CASP isoform a | 4.0 | 2 | 2 | 3 | 4 | 1.61E+07 | 0.32 | 1 | - | 1505 | 164.1 | 5.9 | Regulation of nucleobase, nucleoside, nucleotide and nucleic acid metabolism | Nucleus;Golgi apparatus | Transcription regulator activity | Nucleus |
| SGPL1 | NP_003892.2 | 8879 | sphingosine-1-phosphate lyase 1 | 18.8 | 1 | 5 | 5 | 10 | 3.81E+08 | 0.32 | 5 | 12 | 568 | 63.5 | 9.2 | Metabolism;Energy pathways | Endoplasmic reticulum;Plasma membrane;Mitochondrion | Lyase activity | Endoplasmic reticulum |
| CASP7 | NP_001253987.1 | 840 | caspase-7 isoform f | 8.3 | 4 | 1 | 1 | 2 | 8.63E+07 | 0.32 | 2 | 19.5 | 278 | 31.6 | 7.6 | Apoptosis | Cytoplasm;Endoplasmic reticulum;Nucleus | Cysteine-type peptidase activity | Cytoplasm |
| KIF4A | NP_036442.3 | 24137 | chromosome-associated kinesin KIF4A | 7.7 | 2 | 6 | 6 | 8 | 1.90E+07 | 0.32 | 3 | 2.5 | 1232 | 139.8 | 6.3 | Regulation of nucleobase, nucleoside, nucleotide and nucleic acid metabolism | Nucleus;Cytoplasm;Microtubule;Mitochondrion;Chromosome | DNA binding | Nucleus |
| H2AFY | NP_004884.1 | 9555 | core histone macro-H2A.1 isoform 2 | 44.5 | 3 | 13 | 15 | 73 | 2.01E+09 | 0.31 | 18 | 16.9 | 371 | 39.5 | 9.8 | Regulation of nucleobase, nucleoside, nucleotide and nucleic acid metabolism | Centrosome;Nucleus;Chromosome | DNA binding | Centrosome;Nucleus |
| EBP | NP_006570.1 | 10682 | 3-beta-hydroxysteroid-Delta(8), Delta(7)-isomerase | 12.2 | 1 | 2 | 2 | 7 | 1.09E+08 | 0.31 | 4 | 15.2 | 230 | 26.3 | 7.9 | Metabolism;Energy pathways | Endoplasmic reticulum;Cytoplasm | Isomerase activity | Endoplasmic reticulum |
| PTPN6 | NP_002822.2 | 5777 | tyrosine-protein phosphatase non-receptor type 6 isoform 1 | 7.9 | 3 | 3 | 3 | 3 | 3.03E+07 | 0.31 | 2 | 8.7 | 595 | 67.5 | 7.8 | Cell communication;Signal transduction | Cytoplasm;Mitochondrion | Protein tyrosine phosphatase activity | Cytoplasm |
| FADS1 | NP_037534.3 | 3992 | fatty acid desaturase 1 | 2.2 | 1 | 1 | 1 | 1 | 3.57E+07 | 0.31 | 1 | - | 501 | 57.8 | 9.5 | Metabolism;Energy pathways | Endoplasmic reticulum | Oxidoreductase activity | Endoplasmic reticulum |
| PLS1 | NP_001138791.1 | 5357 | plastin-1 | 14.0 | 1 | 5 | 8 | 23 | 3.91E+08 | 0.31 | 4 | 9.7 | 629 | 70.2 | 5.4 | Cell growth and/or maintenance | Cytoplasm | Structural constituent of cytoskeleton | Cytoplasm |
| SPTBN1 | NP_842565.2 | 6711 | spectrin beta chain, non-erythrocytic 1 isoform 2 | 47.0 | 4 | 1 | 75 | 221 | 1.04E+09 | 0.31 | 1 | - | 2155 | 251.2 | 5.5 | Cell growth and/or maintenance | Cytoplasm;Plasma membrane;Cytoskeleton;Nucleus | Structural constituent of cytoskeleton | Cytoskeleton |
| KIDINS220 | NP_065789.1 | 57498 | kinase D-interacting substrate of 220 kDa | 2.9 | 1 | 3 | 3 | 3 | 1.68E+07 | 0.31 | 1 | - | 1771 | 196.4 | 6.6 | Cell growth and/or maintenance | Plasma membrane | Protein kinase binding | Plasma membrane |
| ESCO2 | NP_001017420.1 | 157570 | N-acetyltransferase ESCO2 | 2.2 | 1 | 2 | 2 | 5 | 4.80E+08 | 0.31 | 1 | - | 601 | 68.3 | 9.4 | Chromosome segregation | Nucleus | Structural constituent of chromatin | Nucleus |
| GALT | NP_000146.2 | 2592 | galactose-1-phosphate uridylyltransferase isoform 1 | 6.3 | 2 | 2 | 2 | 2 | 4.78E+07 | 0.30 | 1 | - | 379 | 43.3 | 7.0 | Energy pathways;Metabolism | - | Nucleotidyltransferase activity | - |
| SPTLC2 | NP_004854.1 | 9517 | serine palmitoyltransferase 2 | 15.7 | 1 | 5 | 5 | 11 | 1.64E+08 | 0.30 | 6 | 3.5 | 562 | 62.9 | 7.8 | Metabolism;Energy pathways | Endoplasmic reticulum;Mitochondrion | Acyltransferase activity | Endoplasmic reticulum |
| MBOAT7 | NP_001139528.1 | 79143 | lysophospholipid acyltransferase 7 isoform 2 | 9.0 | 3 | 3 | 3 | 6 | 1.06E+08 | 0.30 | 2 | 0.5 | 399 | 44.7 | 8.9 | Biological_process unknown | Plasma membrane;Integral to membrane | Receptor activity | Plasma membrane |
| TACSTD2 | NP_002344.2 | 4070 | tumor-associated calcium signal transducer 2 precursor | 30.3 | 1 | 8 | 8 | 50 | 1.03E+09 | 0.30 | 12 | 39.4 | 323 | 35.7 | 8.9 | Cell communication;Signal transduction | Plasma membrane;Cytoplasm | Receptor activity | Plasma membrane |
| EXD2 | NP_001180292.1 | 55218 | exonuclease 3'-5' domain-containing protein 2 isoform 1 | 6.0 | 2 | 2 | 2 | 2 | 3.91E+07 | 0.30 | 2 | 25.5 | 621 | 70.3 | 8.3 | Regulation of nucleobase, nucleoside, nucleotide and nucleic acid metabolism | - | Exonuclease activity | - |
| KIF14 | NP_055690.1 | 9928 | kinesin-like protein KIF14 | 1.1 | 1 | 1 | 1 | 1 | 4.57E+07 | 0.30 | 1 | - | 1648 | 186.4 | 7.9 | Cell growth and/or maintenance | Microtubule;Nucleus | Motor activity | Microtubule;Nucleus |
| ELMOD2 | NP_714913.1 | 255520 | ELMO domain-containing protein 2 | 6.8 | 1 | 1 | 1 | 1 | 8.75E+07 | 0.30 | 1 | - | 293 | 34.9 | 8.1 | Biological_process unknown | - | Molecular function unknown | - |
| MYL9 | NP_006088.2 | 10398 | myosin regulatory light polypeptide 9 isoform a | 56.4 | 2 | 3 | 9 | 68 | 1.27E+09 | 0.30 | 4 | 13.3 | 172 | 19.8 | 4.9 | Muscle contraction | - | Molecular function unknown | - |
| QARS | NP_005042.1 | 5859 | glutamine--tRNA ligase isoform a | 32.8 | 2 | 17 | 17 | 50 | 3.69E+08 | 0.30 | 16 | 16.5 | 775 | 87.7 | 7.2 | Metabolism;Energy pathways | Cytoplasm;Cytosol | Ligase activity | Cytoplasm |
| PIR | NP_001018119.1 | 8544 | pirin | 4.1 | 1 | 1 | 1 | 1 | 1.18E+07 | 0.30 | 1 | - | 290 | 32.1 | 6.9 | Regulation of nucleobase, nucleoside, nucleotide and nucleic acid metabolism | Nuclear matrix | Transcription regulator activity;Transcription cofactor activity | Nuclear matrix |
| ACSL1 | NP_001273640.1 | 2180 | long-chain-fatty-acid--CoA ligase 1 isoform c | 14.9 | 4 | 6 | 6 | 8 | 1.58E+08 | 0.29 | 2 | 24.6 | 664 | 74.2 | 7.3 | Metabolism;Energy pathways | Endoplasmic reticulum;Mitochondrion;Peroxisome | Ligase activity | Endoplasmic reticulum |
| PIK3C2A | NP_002636.2 | 5286 | phosphatidylinositol 4-phosphate 3-kinase C2 domain-containing subunit alpha | 5.5 | 1 | 5 | 5 | 6 | 4.76E+07 | 0.29 | 2 | 44.1 | 1686 | 190.6 | 8.0 | Cell communication;Signal transduction | Cytosol | Lipid kinase activity | Cytosol |
| CA2 | NP_000058.1 | 760 | carbonic anhydrase 2 | 19.2 | 1 | 3 | 3 | 7 | 5.81E+07 | 0.29 | 5 | 8.7 | 260 | 29.2 | 7.4 | Metabolism;Energy pathways | Cytoplasm;Plasma membrane;Golgi apparatus;Nucleus;Extracellular | Catalytic activity | Cytoplasm |
| GNG10 | NP_001017998.1 | 2790 | guanine nucleotide-binding protein G(I)/G(S)/G(O) subunit gamma-10 precursor | 52.9 | 2 | 2 | 2 | 3 | 9.09E+08 | 0.29 | 2 | 7.5 | 68 | 7.2 | 7.9 | Cell communication;Signal transduction | Plasma membrane | GTPase activity | Plasma membrane |
| GPR126 | NP_001027566.1 | 57211 | G-protein coupled receptor 126 isoform alpha 2 precursor | 1.1 | 4 | 1 | 1 | 1 | 3.29E+07 | 0.29 | 1 | - | 1193 | 133.6 | 7.8 | Cell communication | Plasma membrane | G-protein coupled receptor activity | Plasma membrane |
| MORF4L1 | NP_006782.1 | 10933 | mortality factor 4-like protein 1 isoform 1 | 4.0 | 2 | 1 | 1 | 1 | 1.26E+08 | 0.29 | 1 | - | 323 | 37.2 | 9.2 | Regulation of nucleobase, nucleoside, nucleotide and nucleic acid metabolism;Regulation of gene expression, epigenetic | Nucleus;Nucleolus | Transcription regulator activity | Nucleus |
| FDFT1 | NP_001274671.1 | 2222 | squalene synthase isoform 1 | 24.7 | 5 | 7 | 7 | 9 | 6.61E+07 | 0.29 | 2 | 45.4 | 417 | 48.1 | 6.5 | Metabolism;Energy pathways | Endoplasmic reticulum;Extracellular | Transferase activity | Endoplasmic reticulum |
| LIG4 | NP_996820.1 | 3981 | DNA ligase 4 | 5.5 | 1 | 2 | 2 | 2 | 1.65E+07 | 0.28 | 2 | 183 | 911 | 103.9 | 8.0 | Regulation of nucleobase, nucleoside, nucleotide and nucleic acid metabolism;DNA repair | Nucleus;Cytoplasm | Ligase activity;DNA binding | Nucleus |
| BAIAP2L1 | NP_061330.2 | 55971 | brain-specific angiogenesis inhibitor 1-associated protein 2-like protein 1 | 10.0 | 1 | 3 | 3 | 3 | 4.57E+07 | 0.28 | 2 | 6.8 | 511 | 56.8 | 8.7 | Biological_process unknown | - | Molecular function unknown | - |
| H3F3B | NP_005315.1 | 3021 | histone H3.3 | 66.9 | 2 | 3 | 14 | 237 | 9.51E+09 | 0.28 | 16 | 12.6 | 136 | 15.3 | 11.3 | Regulation of nucleobase, nucleoside, nucleotide and nucleic acid metabolism | Nucleus | DNA binding | Nucleus |
| DIAPH3 | NP_112194.2 | 81624 | protein diaphanous homolog 3 isoform b | 1.8 | 6 | 1 | 1 | 1 | 8.39E+06 | 0.28 | 1 | - | 849 | 98.1 | 6.1 | Biological_process unknown | - | Molecular function unknown | - |
| TOP3B | NP_003926.1 | 8940 | DNA topoisomerase 3-beta-1 | 2.2 | 1 | 1 | 1 | 1 | 2.67E+07 | 0.28 | 1 | - | 862 | 96.6 | 8.1 | Regulation of nucleobase, nucleoside, nucleotide and nucleic acid metabolism | Nucleus;Cytoplasm | DNA topoisomerase activity | Nucleus |
| TDRP | NP_778250.2 | 157695 | testis development-related protein isoform 1 | 6.5 | 2 | 1 | 1 | 1 | 7.43E+06 | 0.28 | 1 | - | 185 | 20.4 | 6.0 | Biological_process unknown | - | Molecular function unknown | - |

| Gene Symbol | RefSeq_Accession | Entrez Gene ID | Description | Coverage (%) | Proteins | Unique Peptides | # Peptides | PSM | Area | SCC-R /SCC-S | SCC-R /SCC-S (Count) | SCC-R /SCC-S (Variability [%]) | # AAs | MW [kDa] | calc. pI | Biological Process | Localization | Molecular Function | Primary Localization |
|---|---|---|---|---|---|---|---|---|---|---|---|---|---|---|---|---|---|---|---|
| FAM83H | NP_940890.3 | 286077 | protein FAM83H | 15.9 | 1 | 9 | 9 | 11 | 1.07E+08 | 0.28 | 4 | 8.6 | 1179 | 127.1 | 7.0 | Biological_process unknown | Nucleus | Molecular function unknown | Nucleus |
| QPRT | NP_055113.2 | 23475 | nicotinate-nucleotide pyrophosphorylase [carboxylating] precursor | 42.4 | 3 | 7 | 7 | 15 | 1.78E+08 | 0.28 | 6 | 23.8 | 297 | 30.8 | 6.2 | Metabolism;Energy pathways | - | Catalytic activity | - |
| HIST1H1C | NP_005310.1 | 3006 | histone H1.2 | 31.0 | 3 | 3 | 11 | 130 | 7.12E+09 | 0.27 | 6 | 3.1 | 213 | 21.4 | 10.9 | Regulation of nucleobase, nucleoside, nucleotide and nucleic acid metabolism | Nucleus;Nucleolus;Mitochondrion | DNA binding | Nucleus |
| SYNJ2BP | NP_060843.2 | 55333 | synaptojanin-2-binding protein | 19.3 | 4 | 2 | 2 | 3 | 1.71E+08 | 0.27 | 2 | 35.1 | 145 | 15.9 | 6.3 | Biological_process unknown;Mitochondrial transport | Cytoplasm;Mitochondrion | Protein domain specific binding | Mitochondrion |
| IDI1 | NP_004499.2 | 3422 | isopentenyl-diphosphate Delta-isomerase 1 | 26.4 | 1 | 5 | 5 | 8 | 1.30E+08 | 0.27 | 4 | 88.1 | 284 | 32.5 | 7.9 | Metabolism;Energy pathways | Peroxisome | Isomerase activity | Peroxisome |
| LOC102725117 | XP_006726519.1 | 102725117 | rho guanine nucleotide exchange factor 5-like | 0.9 | 2 | 1 | 1 | 1 | 3.06E+07 | 0.27 | 1 | - | 1493 | 165 | 5.7 | - | - | - | - |
| PHF2 | NP_005383.3 | 5253 | lysine-specific demethylase PHF2 | 3.6 | 1 | 2 | 2 | 2 | 7.10E+07 | 0.27 | 1 | - | 1096 | 120.7 | 9.2 | Regulation of nucleobase, nucleoside, nucleotide and nucleic acid metabolism | Nucleus;Cytoplasm | Transcription regulator activity | Nucleus |
| SMARCA5 | NP_003592.3 | 8467 | SWI/SNF-related matrix-associated actin-dependent regulator of chromatin subfamily A member 5 | 20.6 | 1 | 16 | 20 | 34 | 2.31E+08 | 0.27 | 16 | 46 | 1052 | 121.8 | 8.1 | Regulation of nucleobase, nucleoside, nucleotide and nucleic acid metabolism | Nucleus;Nucleolus | DNA binding | Nucleus |
| CEP170B | NP_055820.2 | 283638 | centrosomal protein of 170 kDa protein B isoform 2 | 2.3 | 2 | 2 | 2 | 2 | 4.52E+07 | 0.27 | 2 | 22.5 | 1519 | 163.5 | 7.0 | Biological_process unknown | - | Molecular function unknown | - |
| TAOK3 | NP_057365.3 | 51347 | serine/threonine-protein kinase TAO3 | 5.0 | 1 | 3 | 3 | 3 | 1.37E+07 | 0.27 | 1 | - | 898 | 105.3 | 7.3 | Cell communication;Signal transduction | - | Protein serine/threonine kinase activity | - |
| TONSL | NP_038460.4 | 4796 | tonsoku-like protein | 2.1 | 1 | 1 | 1 | 1 | 7.98E+07 | 0.27 | 1 | - | 1378 | 150.8 | 6.4 | Regulation of nucleobase, nucleoside, nucleotide and nucleic acid metabolism | - | Transcription regulator activity | - |
| PARP1 | NP_001609.2 | 142 | poly [ADP-ribose] polymerase 1 | 41.4 | 1 | 30 | 30 | 80 | 7.26E+08 | 0.27 | 33 | 26.9 | 1014 | 113 | 8.9 | Protein metabolism | Nucleus;Nucleolus;Cytoplasm | Catalytic activity | Nucleus |
| VGLL4 | NP_055482.2 | 9686 | transcription cofactor vestigial-like protein 4 isoform b | 13.5 | 6 | 2 | 2 | 2 | 4.49E+07 | 0.27 | 1 | - | 290 | 30.9 | 8.2 | Regulation of nucleobase, nucleoside, nucleotide and nucleic acid metabolism | Nucleus;Cytoplasm | Transcription regulator activity | Nucleus |
| LAMB2 | NP_002283.3 | 3913 | laminin subunit beta-2 precursor | 1.1 | 1 | 1 | 1 | 2 | 2.71E+07 | 0.26 | 1 | - | 1798 | 195.9 | 6.5 | Cell growth and/or maintenance | Extracellular | Extracellular matrix structural constituent | Extracellular |
| TOP1 | NP_003277.1 | 7150 | DNA topoisomerase 1 | 14.8 | 1 | 11 | 11 | 17 | 4.38E+08 | 0.26 | 13 | 21.4 | 765 | 90.7 | 9.3 | Regulation of nucleobase, nucleoside, nucleotide and nucleic acid metabolism | Nucleus;Cytoplasm;Nucleolus | DNA topoisomerase activity | Nucleus |
| FAM98A | NP_056290.3 | 25940 | protein FAM98A | 10.4 | 1 | 2 | 3 | 5 | 2.06E+06 | 0.26 | 1 | - | 518 | 55.2 | 9.0 | Biological_process unknown | - | Molecular function unknown | - |
| HOOK1 | NP_056972.1 | 51361 | protein Hook homolog 1 | 4.7 | 1 | 2 | 2 | 5 | 8.64E+07 | 0.25 | 2 | 27.5 | 728 | 84.6 | 5.2 | Cell growth and/or maintenance | Cytoplasm | Cytoskeletal protein binding | Cytoplasm |
| MMAB | NP_443077.1 | 326625 | cob(I)yrinic acid a,c-diamide adenosyltransferase, mitochondrial precursor | 42.8 | 1 | 5 | 5 | 10 | 1.21E+08 | 0.25 | 6 | 29.2 | 250 | 27.4 | 8.6 | Metabolism;Energy pathways | Mitochondrion | Catalytic activity | Mitochondrion |
| SBNO1 | NP_060653.3 | 55206 | protein strawberry notch homolog 1 isoform 2 | 6.2 | 2 | 4 | 4 | 6 | 4.95E+07 | 0.25 | 2 | 4.9 | 1392 | 154.1 | 7.9 | Biological_process unknown | - | Molecular function unknown | - |
| SLC27A4 | NP_005085.2 | 10999 | long-chain fatty acid transport protein 4 | 16.6 | 1 | 7 | 7 | 12 | 5.91E+07 | 0.25 | 1 | - | 643 | 72 | 8.5 | Transport | Plasma membrane | Auxiliary transport protein activity | Plasma membrane |
| PRNP | NP_001073592.1 | 5621 | major prion protein preproprotein | 4.7 | 1 | 1 | 1 | 6 | 2.13E+08 | 0.25 | 1 | - | 253 | 27.6 | 9.0 | Metabolism | Plasma membrane;Cytoplasm;Golgi apparatus;Nucleus;Extracellular;Endoplasmic reticulum | Molecular function unknown | Plasma membrane |
| PROSER2 | NP_694988.3 | 254427 | proline and serine-rich protein 2 | 9.4 | 1 | 3 | 3 | 4 | 8.77E+07 | 0.25 | 2 | 32.9 | 435 | 45.8 | 7.2 | Biological_process unknown | Nucleus | Molecular function unknown | Nucleus |
| ARHGEF35 | NP_001003702.2 | 445328 | rho guanine nucleotide exchange factor 35 | 5.0 | 1 | 1 | 1 | 1 | 5.74E+06 | 0.25 | 1 | - | 484 | 53.3 | 4.3 | Biological_process unknown | - | Molecular function unknown | - |
| MIB1 | NP_065825.1 | 57534 | E3 ubiquitin-protein ligase MIB1 | 1.5 | 1 | 1 | 1 | 2 | 7.75E+06 | 0.25 | 1 | - | 1006 | 110.1 | 6.9 | Protein metabolism | Vesicle | Ubiquitin-specific protease activity | Vesicle |
| BAIAP2 | NP_001138360.1 | 10458 | brain-specific angiogenesis inhibitor 1-associated protein 2 isoform 4 | 29.6 | 4 | 8 | 8 | 11 | 1.96E+08 | 0.25 | 7 | 168.2 | 534 | 59 | 9.0 | Cell communication;Signal transduction | Nucleus;Cytoplasm;Perinuclear region | Receptor signaling complex scaffold activity | Cytoplasm |
| TMPO | NP_001027454.1 | 7112 | thymopoietin isoform beta | 54.6 | 2 | 7 | 18 | 87 | 1.68E+09 | 0.24 | 2 | 5.2 | 454 | 50.6 | 9.4 | Nuclear organization and biogenesis;Regulation of cell cycle | Nucleus;Nuclear membrane;Mitochondrion;Cytoplasm | Molecular function unknown | Nucleus;Nuclear membrane;Mitochondrion;Cytoplasm |
| CSNK1A1 | NP_001883.4 | 1452 | casein kinase I isoform alpha isoform 2 | 16.9 | 5 | 4 | 4 | 8 | 1.04E+08 | 0.24 | 3 | 18.3 | 337 | 38.9 | 9.6 | Cell cycle | Nucleoplasm;Cytoskeleton;Synaptic vesicle;Cytoplasm;Nucleus;Nucleolus;Kinetochore;Centrosome;Microtubule | Protein serine/threonine kinase activity | Cytoplasm |
| MYO1B | NP_036355.2 | 4430 | unconventional myosin-Ib isoform 2 | 29.7 | 3 | 24 | 24 | 62 | 8.89E+08 | 0.24 | 21 | 31.2 | 1078 | 124.9 | 9.2 | Cell growth and/or maintenance | Plasma membrane;Nucleus;Cytoplasm | Motor activity | Plasma membrane |
| ADI1 | NP_060739.2 | 55256 | 1,2-dihydroxy-3-keto-5-methylthiopentene dioxygenase | 8.9 | 1 | 2 | 2 | 5 | 3.75E+08 | 0.24 | 3 | 11.3 | 179 | 21.5 | 5.7 | Cell growth and/or maintenance | Plasma membrane;Cytoplasm;Nucleus | Oxidoreductase activity | Plasma membrane |
| VPS54 | NP_001005739.1 | 51542 | vacuolar protein sorting-associated protein 54 isoform 2 | 2.7 | 2 | 1 | 1 | 1 | 6.37E+06 | 0.24 | 1 | - | 965 | 109.3 | 6.6 | Transport | Golgi apparatus | Transporter activity | Golgi apparatus |
| ACACA | NP_942135.1 | 31 | acetyl-CoA carboxylase 1 isoform 4 | 15.7 | 5 | 19 | 19 | 29 | 1.32E+08 | 0.24 | 9 | 20 | 2268 | 257.1 | 6.6 | Metabolism;Energy pathways | Cytoplasm;Nucleus | Ligase activity | Cytoplasm;Nucleus |
| HIST2H3C | NP_066403.2 | 126961 | histone H3.2 | 67.7 | 3 | 2 | 15 | 310 | 7.21E+09 | 0.24 | 30 | 12.1 | 136 | 15.4 | 11.3 | Regulation of nucleobase, nucleoside, nucleotide and nucleic acid metabolism | Nucleus | DNA binding | Nucleus |
| DNMT1 | NP_001370.1 | 1786 | DNA (cytosine-5)-methyltransferase 1 isoform b | 13.4 | 2 | 15 | 15 | 23 | 1.91E+08 | 0.24 | 6 | 21.4 | 1616 | 183 | 7.8 | Regulation of nucleobase, nucleoside, nucleotide and nucleic acid metabolism | Nucleus;Nucleolus;Mitochondrion | DNA-methyltransferase activity | Nucleus |
| FHL2 | NP_001441.4 | 2274 | four and a half LIM domains protein 2 | 57.7 | 1 | 10 | 10 | 25 | 4.96E+08 | 0.24 | 6 | 36.7 | 279 | 32.2 | 7.6 | Regulation of nucleobase, nucleoside, nucleotide and nucleic acid metabolism | Nucleus;Cytoskeleton;Cytoplasm;Mitochondrion | Protein binding | Nucleus |
| SLC3A2 | NP_001013269.1 | 6520 | 4F2 cell-surface antigen heavy chain isoform f | 49.5 | 2 | 1 | 22 | 170 | 5.33E+09 | 0.24 | 2 | 8.8 | 529 | 57.9 | 5.4 | Transport | Plasma membrane;Mitochondrion;Extracellular;Nucleus;Cytoplasm | Auxiliary transport protein activity | Plasma membrane;Nucleus;Cytoplasm |
| BRI3BP | NP_542193.3 | 140707 | BRI3-binding protein precursor | 14.3 | 1 | 2 | 2 | 3 | 2.34E+08 | 0.23 | 1 | - | 251 | 27.8 | 9.4 | Biological_process unknown | - | Molecular function unknown | - |
| GGCX | NP_001135741.1 | 2677 | vitamin K-dependent gamma-carboxylase isoform 2 | 2.3 | 2 | 1 | 1 | 1 | 5.67E+07 | 0.23 | 1 | - | 701 | 80.9 | 7.6 | Metabolism;Energy pathways | Endoplasmic reticulum | Ligase activity | Endoplasmic reticulum |

| Gene Symbol | RefSeq_Accession | Entrez Gene ID | Description | Coverage (%) | Proteins | Unique Peptides | # Peptides | PSM | Area | SCC-R /SCC-S | SCC-R /SCC-S (Count) | SCC-R /SCC-S (Variability [%]) | # AAs | MW [kDa] | calc. pI | Biological Process | Localization | Molecular Function | Primary Localization |
|---|---|---|---|---|---|---|---|---|---|---|---|---|---|---|---|---|---|---|---|
| **C1D** | NP_006324.1 | 10438 | **nuclear nucleic acid-binding protein C1D** | 22.7 | 1 | 1 | 1 | 1 | 6.47E+06 | **0.23** | 1 | - | 141 | 16 | 9.0 | Regulation of nucleobase, nucleoside, nucleotide and nucleic acid metabolism | Nucleus;Cytoplasm | Transcription regulator activity | Nucleus |
| **DDX21** | NP_004719.2 | 9188 | **nucleolar RNA helicase 2 isoform 1** | 48.5 | 2 | 34 | 36 | 155 | 8.99E+08 | **0.23** | 44 | 33.3 | 783 | 87.3 | 9.3 | Metabolism;Energy pathways | Nucleolus;Nucleus | ATPase activity | Nucleolus |
| **STOM** | NP_004090.4 | 2040 | **erythrocyte band 7 integral membrane protein isoform a** | 36.5 | 4 | 7 | 7 | 11 | 1.40E+08 | **0.23** | 5 | 45.2 | 288 | 31.7 | 7.9 | Cell communication;Signal transduction | Cytoplasm;Plasma membrane;Cytoplasmic vesicle;Nucleus | Channel regulator activity | Cytoplasmic vesicle |
| **DOCK9** | NP_001123522.1 | 23348 | **dedicator of cytokinesis protein 9 isoform d** | 5.8 | 4 | 3 | 3 | 3 | 3.45E+07 | **0.22** | 1 | - | 1253 | 142.6 | 7.7 | Cell communication;Signal transduction | Plasma membrane | Guanyl-nucleotide exchange factor activity | Plasma membrane |
| **TP53I3** | NP_671713.1 | 9540 | **quinone oxidoreductase PIG3 isoform 1** | 37.4 | 2 | 9 | 9 | 10 | 1.62E+08 | **0.22** | 2 | 14.3 | 332 | 35.5 | 7.2 | Cell communication;Signal transduction | Cytoplasm | Oxidoreductase activity | Cytoplasm |
| **HELLS** | NP_001276001.1 | 3070 | **lymphoid-specific helicase isoform 7** | 13.7 | 10 | 7 | 7 | 9 | 2.51E+08 | **0.22** | 3 | 76.4 | 708 | 81.6 | 7.8 | Regulation of nucleobase, nucleoside, nucleotide and nucleic acid metabolism | Nucleus | DNA binding | Nucleus |
| **AACS** | NP_076417.2 | 65985 | **acetoacetyl-CoA synthetase** | 4.5 | 1 | 2 | 2 | 3 | 7.54E+07 | **0.22** | 1 | - | 672 | 75.1 | 6.2 | Metabolism;Energy pathways | Cytoplasm | Ligase activity | Cytoplasm |
| **BLMH** | NP_000377.1 | 642 | **bleomycin hydrolase** | 23.3 | 1 | 6 | 6 | 9 | 1.43E+08 | **0.22** | 3 | 59.7 | 455 | 52.5 | 6.3 | Metabolism;Energy pathways | Cytoplasm;Nucleus;Endoplasmic reticulum;Ribosome | Hydrolase activity | Cytoplasm |
| **HIST1H3B** | NP_003528.1 | 8358 | **histone H3.1** | 67.7 | 3 | 2 | 15 | 342 | 1.46E+10 | **0.22** | 28 | 15.1 | 136 | 15.4 | 11.1 | Regulation of nucleobase, nucleoside, nucleotide and nucleic acid metabolism | Nucleus | DNA binding | Nucleus |
| **FGD4** | NP_640334.2 | 121512 | **FYVE, RhoGEF and PH domain-containing protein 4** | 2.2 | 1 | 1 | 1 | 1 | 7.79E+07 | **0.22** | 1 | - | 766 | 86.6 | 6.1 | Cell communication;Signal transduction | Cytoskeleton | Guanyl-nucleotide exchange factor activity | Cytoskeleton |
| **SULT1A3** | NP_808220.1 | 6818 | **sulfotransferase 1A3/1A4** | 20.0 | 1 | 3 | 3 | 6 | 1.27E+08 | **0.22** | 3 | 62.2 | 295 | 34.2 | 6.0 | Metabolism;Energy pathways | Cytoplasm | Sulfotransferase activity | Cytoplasm |
| **TPD52L1** | NP_001003397.1 | 7164 | **tumor protein D53 isoform 4** | 10.7 | 4 | 1 | 1 | 1 | 7.70E+07 | **0.22** | 1 | - | 131 | 14.7 | 5.6 | Regulation of cell proliferation;Signal transduction | - | Molecular function unknown | - |
| **CTCF** | NP_006556.1 | 10664 | **transcriptional repressor CTCF isoform 1** | 7.8 | 2 | 4 | 4 | 6 | 8.68E+06 | **0.21** | 1 | - | 727 | 82.7 | 7.0 | Regulation of nucleobase, nucleoside, nucleotide and nucleic acid metabolism | Nucleus | Transcription regulator activity | Nucleus |
| **EPS8L2** | NP_073609.2 | 64787 | **epidermal growth factor receptor kinase substrate 8-like protein 2** | 46.4 | 1 | 17 | 17 | 42 | 5.14E+08 | **0.21** | 12 | 43.2 | 715 | 80.6 | 6.8 | Cell communication;Signal transduction | - | Receptor signaling complex scaffold activity | - |
| **SIGIRR** | NP_068577.2 | 59307 | **single Ig IL-1-related receptor** | 2.9 | 1 | 1 | 1 | 1 | 4.26E+07 | **0.21** | 1 | - | 410 | 45.7 | 6.5 | Cell communication;Signal transduction | Plasma membrane | Molecular function unknown | Plasma membrane |
| **TMEM214** | NP_060197.4 | 54867 | **transmembrane protein 214 isoform 1** | 17.9 | 2 | 7 | 7 | 12 | 1.48E+08 | **0.21** | 1 | - | 689 | 77.1 | 9.1 | Biological_process unknown | - | Molecular function unknown | - |
| **SQLE** | NP_003120.2 | 6713 | **squalene monooxygenase** | 11.0 | 1 | 4 | 4 | 7 | 1.38E+08 | **0.21** | 3 | 74.2 | 574 | 63.9 | 8.6 | Metabolism;Energy pathways | Endoplasmic reticulum | Catalytic activity | Endoplasmic reticulum |
| **AARSD1** | NP_001248363.1 | 80755 | **alanyl-tRNA editing protein Aarsd1** | 3.6 | 3 | 1 | 1 | 2 | 1.21E+08 | **0.21** | 1 | - | 412 | 45.5 | 6.4 | Biological_process unknown | - | Molecular function unknown | - |
| **MINK1** | NP_056531.1 | 50488 | **misshapen-like kinase 1 isoform 1** | 7.6 | 12 | 3 | 6 | 7 | 1.08E+08 | **0.21** | 2 | 35.4 | 1295 | 145.7 | 7.4 | Signal transduction | Cytoplasm;Golgi apparatus;Nucleus | Protein serine/threonine kinase activity | Golgi apparatus;Nucleus;Cytoplasm |
| **MVB12A** | NP_612410.1 | 93343 | **multivesicular body subunit 12A** | 5.5 | 1 | 1 | 1 | 1 | 1.98E+06 | **0.21** | 1 | - | 273 | 28.8 | 8.9 | Biological_process unknown;Signal transduction | Nucleus;Actin cytoskeleton | Molecular function unknown | Nucleus |
| **KIAA1598** | NP_001120683.1 | 57698 | **shootin-1 isoform a** | 32.3 | 5 | 15 | 15 | 25 | 4.62E+08 | **0.20** | 10 | 34.7 | 631 | 71.6 | 5.3 | Biological_process unknown | - | Molecular function unknown | - |
| **RPS6KA5** | NP_872198.1 | 9252 | **ribosomal protein S6 kinase alpha-5 isoform b** | 1.8 | 2 | 1 | 1 | 1 | 5.87E+07 | **0.20** | 1 | - | 549 | 61.7 | 6.9 | Cytokine and chemokine mediated signaling pathway;Regulation of translation;Transcription | Nucleus;Cytoplasm | Protein serine/threonine kinase activity | Nucleus |
| **DSG2** | NP_001934.2 | 1829 | **desmoglein-2 preproprotein** | 20.8 | 1 | 11 | 11 | 15 | 2.97E+08 | **0.20** | 6 | 77.7 | 1118 | 122.2 | 5.2 | Cell growth and/or maintenance | Plasma membrane;Cytoskeleton;Nucleus;Cytoplasm | Cell adhesion molecule activity | Plasma membrane |
| **EVPL** | NP_001979.2 | 2125 | **envoplakin** | 11.3 | 2 | 13 | 13 | 15 | 7.57E+07 | **0.20** | 4 | 16.5 | 2033 | 231.5 | 7.0 | Cell growth and/or maintenance | Cytoplasm;Nucleus;Plasma membrane | Structural molecule activity | Plasma membrane |
| **DICER1** | NP_001182502.1 | 23405 | **endoribonuclease Dicer isoform 2** | 6.0 | 2 | 6 | 6 | 6 | 4.01E+07 | **0.20** | 1 | - | 1829 | 208.3 | 5.7 | Gene silencing | Cytoplasm;Endoplasmic reticulum | Ribonuclease activity | Cytoplasm |
| **MRRF** | NP_620132.1 | 92399 | **ribosome-recycling factor, mitochondrial isoform 1 precursor** | 45.4 | 3 | 7 | 7 | 11 | 2.05E+08 | **0.19** | 3 | 1.4 | 262 | 29.3 | 9.8 | Biological_process unknown | Mitochondrion | Molecular function unknown | Mitochondrion |
| **POLE** | NP_006222.2 | 5426 | **DNA polymerase epsilon catalytic subunit A** | 1.2 | 2 | 2 | 2 | 3 | 3.36E+07 | **0.19** | 1 | - | 2286 | 261.4 | 6.4 | Regulation of nucleobase, nucleoside, nucleotide and nucleic acid metabolism | Nucleus | DNA-directed DNA polymerase activity | Nucleus |
| **CASP10** | NP_001193453.1 | 843 | **caspase-10 isoform 6 preproprotein** | 3.7 | 3 | 1 | 1 | 1 | 2.38E+07 | **0.19** | 1 | - | 455 | 51.8 | 5.7 | Apoptosis | Cytoplasm;Nucleus | Cysteine-type peptidase activity | Cytoplasm |
| **NF1** | NP_001035957.1 | 4763 | **neurofibromin isoform 1** | 7.7 | 3 | 13 | 13 | 16 | 7.47E+07 | **0.19** | 4 | 28.5 | 2839 | 319.2 | 7.4 | Cell communication;Signal transduction | Cytoplasm | GTPase activator activity | Cytoplasm |
| **SPINT1** | NP_001027539.1 | 6692 | **kunitz-type protease inhibitor 1 isoform 2 precursor** | 9.9 | 2 | 3 | 3 | 3 | 7.60E+07 | **0.19** | 1 | - | 513 | 56.8 | 6.2 | Protein metabolism | Soluble fraction;Extracellular;Membrane fraction | Protease inhibitor activity | Membrane fraction |
| **PIGO** | NP_690577.2 | 84720 | **GPI ethanolamine phosphate transferase 3 isoform 2** | 7.1 | 2 | 2 | 2 | 2 | 1.78E+07 | **0.19** | 1 | - | 672 | 73.9 | 6.6 | Biological_process unknown | Endoplasmic reticulum | Molecular function unknown | Endoplasmic reticulum |
| **SLC4A1AP** | NP_060628.2 | 22950 | **kanadaptin** | 13.9 | 1 | 6 | 6 | 10 | 4.57E+07 | **0.19** | 4 | 49.8 | 796 | 88.8 | 5.2 | Cell communication;Signal transduction | Nucleus;Mitochondrion | Receptor signaling complex scaffold activity | Nucleus |
| **HIST1H4A** | NP_003529.1 | 8359 | **histone H4** | 65.1 | 1 | 21 | 21 | 568 | 2.34E+10 | **0.18** | 98 | 25.2 | 103 | 11.4 | 11.4 | Regulation of nucleobase, nucleoside, nucleotide and nucleic acid metabolism | Nucleus | DNA binding | Nucleus |
| **RBP7** | NP_443192.1 | 116362 | **retinoid-binding protein 7** | 13.4 | 1 | 1 | 1 | 2 | 8.84E+07 | **0.18** | 1 | - | 134 | 15.5 | 7.8 | Transport | Cytoplasm | Transporter activity | Cytoplasm |
| **ANKRD52** | NP_775866.2 | 283373 | **serine/threonine-protein phosphatase 6 regulatory ankyrin repeat subunit C** | 5.4 | 1 | 2 | 2 | 2 | 2.63E+08 | **0.18** | 1 | - | 1076 | 115 | 6.5 | Biological_process unknown | - | Protein binding | - |
| **SERPINB1** | NP_109591.1 | 1992 | **leukocyte elastase inhibitor** | 47.0 | 1 | 17 | 17 | 76 | 2.17E+09 | **0.18** | 18 | 34.8 | 379 | 42.7 | 6.3 | Protein metabolism | Cytoplasm | Protease inhibitor activity | Cytoplasm |
| **IDH2** | NP_001277043.1 | 3418 | **isocitrate dehydrogenase [NADP], mitochondrial isoform 3** | 22.1 | 3 | 5 | 6 | 10 | 6.20E+08 | **0.18** | 3 | 9.1 | 322 | 36.1 | 8.4 | Metabolism;Energy pathways | Mitochondrion | Catalytic activity | Mitochondrion |

| Gene Symbol | RefSeq_Accession | Entrez Gene ID | Description | Coverage (%) | Proteins | Unique Peptides | # Peptides | PSM | Area | SCC-R /SCC-S | SCC-R /SCC-S (Count) | SCC-R /SCC-S (Variability [%]) | # AAs | MW [kDa] | calc. pI | Biological Process | Localization | Molecular Function | Primary Localization |
|---|---|---|---|---|---|---|---|---|---|---|---|---|---|---|---|---|---|---|---|
| TOP2B | NP_001059.2 | 7155 | DNA topoisomerase 2-beta | 6.5 | 1 | 5 | 8 | 11 | 1.92E+08 | 0.18 | 1 | - | 1621 | 182.5 | 8.1 | Regulation of nucleobase, nucleoside, nucleotide and nucleic acid metabolism | Nucleus;Nucleolus;Kinetochore;Cytoplasm | DNA topoisomerase activity | Nucleus |
| TRAFD1 | NP_006691.1 | 10906 | TRAF-type zinc finger domain-containing protein 1 | 4.8 | 1 | 2 | 2 | 2 | 3.66E+07 | 0.18 | 1 | - | 582 | 64.8 | 5.3 | Regulation of nucleobase, nucleoside, nucleotide and nucleic acid metabolism | - | DNA binding | - |
| ACOX1 | NP_001171968.1 | 51 | peroxisomal acyl-coenzyme A oxidase 1 isoform c | 15.6 | 3 | 6 | 6 | 8 | 7.75E+07 | 0.18 | 3 | 18.1 | 622 | 70.1 | 7.6 | Metabolism;Energy pathways | Peroxisome;Mitochondrion;Nucleus | Catalytic activity | Peroxisome;Nucleus |
| LEMD3 | NP_001161086.1 | 23592 | inner nuclear membrane protein Man1 isoform 2 | 14.6 | 2 | 7 | 7 | 7 | 9.11E+07 | 0.18 | 3 | 3.7 | 910 | 99.8 | 7.8 | Immune response | Nucleus;Endoplasmic reticulum | Nucleotide binding | Nucleus |
| DCXR | NP_001182147.1 | 51181 | L-xylulose reductase isoform 2 | 27.7 | 2 | 5 | 5 | 9 | 2.97E+07 | 0.17 | 3 | 35.9 | 242 | 25.7 | 8.1 | Metabolism;Energy pathways | Integral to membrane;Mitochondrion | Oxidoreductase activity | Integral to membrane |
| TARS2 | NP_079426.2 | 80222 | threonine--tRNA ligase, mitochondrial isoform a | 8.2 | 3 | 3 | 3 | 3 | 2.31E+07 | 0.17 | 1 | - | 718 | 81 | 7.3 | Protein metabolism | - | Ligase activity | - |
| ALDH3A1 | NP_001128639.1 | 218 | aldehyde dehydrogenase, dimeric NADP-preferring | 20.5 | 5 | 5 | 6 | 11 | 2.11E+08 | 0.17 | 5 | 16 | 453 | 50.4 | 6.5 | Metabolism;Energy pathways | Cytoplasm;Nucleus | Catalytic activity | Cytoplasm |
| ERMP1 | NP_079172.2 | 79956 | endoplasmic reticulum metallopeptidase 1 | 4.9 | 1 | 3 | 3 | 4 | 8.85E+07 | 0.17 | 2 | 18.8 | 904 | 100.2 | 7.5 | Biological_process unknown | Integral to membrane | Molecular function unknown | Integral to membrane |
| CPD | NP_001295.2 | 1362 | carboxypeptidase D isoform 1 precursor | 3.1 | 2 | 2 | 2 | 3 | 6.15E+07 | 0.16 | 1 | - | 1380 | 152.8 | 6.1 | Protein metabolism | Plasma membrane;Endoplasmic reticulum;Golgi apparatus;Nucleus;Cytoplasm | Carboxypeptidase activity | Plasma membrane |
| GLUL | NP_001028216.1 | 2752 | glutamine synthetase | 11.5 | 1 | 3 | 3 | 3 | 2.19E+07 | 0.16 | 1 | - | 373 | 42 | 6.9 | Metabolism;Energy pathways | Cytosol | Transaminase activity | Cytosol |
| LYN | NP_001104567.1 | 4067 | tyrosine-protein kinase Lyn isoform B | 13.7 | 41 | 4 | 5 | 6 | 2.21E+08 | 0.16 | 1 | - | 491 | 56 | 6.5 | Cell communication;Signal transduction | Plasma membrane;Cytoplasm;Nucleus | Protein-tyrosine kinase activity | Plasma membrane |
| TMTC3 | NP_861448.2 | 160418 | transmembrane and TPR repeat-containing protein 3 | 4.4 | 1 | 3 | 3 | 5 | 5.97E+07 | 0.16 | 2 | 2.3 | 914 | 103.8 | 8.8 | Biological_process unknown | - | Molecular function unknown | - |
| HIP1R | NP_003950.1 | 9026 | huntingtin-interacting protein 1-related protein | 13.0 | 1 | 8 | 8 | 11 | 9.71E+07 | 0.16 | 4 | 15.5 | 1068 | 119.3 | 6.7 | Cell growth and/or maintenance | Cytoplasmic vesicle;Clathrin-coated vesicle | Cytoskeletal protein binding | Cytoplasmic vesicle |
| SNCG | NP_003078.2 | 6623 | gamma-synuclein | 73.2 | 1 | 8 | 8 | 18 | 3.78E+08 | 0.16 | 9 | 39.7 | 127 | 13.3 | 4.9 | Protein metabolism | Cytoplasm;Centrosome;Nucleus | Chaperone activity | Cytoplasm |
| TAGLN | NP_001001522.1 | 6876 | transgelin | 52.7 | 1 | 9 | 9 | 16 | 1.06E+09 | 0.16 | 7 | 54.1 | 201 | 22.6 | 8.8 | Muscle development | Cytoplasm | Cytoskeletal protein binding | Cytoplasm |
| CNN2 | NP_004359.1 | 1265 | calponin-2 isoform a | 47.6 | 2 | 9 | 9 | 33 | 5.67E+08 | 0.15 | 9 | 23.5 | 309 | 33.7 | 7.3 | Cell growth and/or maintenance | Cytoplasm;Nucleus;Mitochondrion | Cytoskeletal protein binding | Cytoplasm |
| LAMB3 | NP_001017402.1 | 3914 | laminin subunit beta-3 precursor | 9.7 | 2 | 8 | 8 | 11 | 7.74E+07 | 0.15 | 5 | 71.3 | 1172 | 129.5 | 7.2 | Cell growth and/or maintenance | Extracellular | Extracellular matrix structural constituent | Extracellular |
| ATP13A1 | NP_065143.2 | 57130 | probable cation-transporting ATPase 13A1 | 3.2 | 1 | 3 | 3 | 3 | 6.58E+07 | 0.15 | 3 | 54.8 | 1204 | 132.9 | 8.1 | Metabolism;Energy pathways | Integral to membrane;Nucleus | ATPase activity | Integral to membrane |
| MVK | NP_000422.1 | 4598 | mevalonate kinase | 32.6 | 1 | 7 | 7 | 9 | 3.83E+08 | 0.15 | 2 | 9.6 | 396 | 42.4 | 6.5 | Metabolism;Energy pathways | Cytoplasm;Peroxisome;Extracellular | Catalytic activity | Cytoplasm |
| PACSIN3 | NP_057307.2 | 29763 | protein kinase C and casein kinase substrate in neurons protein 3 | 32.8 | 1 | 10 | 10 | 12 | 1.78E+08 | 0.15 | 3 | 7.5 | 424 | 48.5 | 6.2 | Cell communication;Signal transduction | Cytoplasm;Nucleus | Receptor signaling complex scaffold activity | Cytoplasm |
| NSD1 | NP_758859.1 | 64324 | histone-lysine N-methyltransferase, H3 lysine-36 and H4 lysine-20 specific isoform a | 0.7 | 2 | 1 | 1 | 1 | 2.99E+07 | 0.15 | 1 | - | 2427 | 267.2 | 8.5 | Regulation of nucleobase, nucleoside, nucleotide and nucleic acid metabolism | Nucleus | Transcription factor activity | Nucleus |
| RALGPS2 | NP_001273176.1 | 55103 | ras-specific guanine nucleotide-releasing factor RalGPS2 isoform 2 | 5.0 | 2 | 2 | 2 | 5 | 2.82E+08 | 0.15 | 1 | - | 557 | 62.1 | 8.0 | Cell communication;Signal transduction | Cytoplasm;Nucleus;Plasma membrane | Guanyl-nucleotide exchange factor activity | Nucleus |
| YBX3 | NP_001138898.1 | 8531 | Y-box-binding protein 3 isoform b | 78.9 | 2 | 2 | 18 | 87 | 9.22E+08 | 0.15 | 2 | 54.7 | 303 | 31.9 | 9.7 | Regulation of nucleobase, nucleoside, nucleotide and nucleic acid metabolism | Nucleus;Cytoplasm | DNA binding | Nucleus |
| ITGA6 | NP_000201.2 | 3655 | integrin alpha-6 isoform b precursor | 24.2 | 2 | 18 | 18 | 41 | 3.25E+08 | 0.15 | 11 | 64.3 | 1073 | 119.4 | 6.8 | Cell communication;Signal transduction | Plasma membrane | Receptor activity | Plasma membrane |
| ZFC3H1 | NP_659419.3 | 196441 | zinc finger C3H1 domain-containing protein | 0.6 | 1 | 1 | 1 | 1 | 8.90E+06 | 0.15 | 1 | - | 1989 | 226.2 | 8.1 | Biological_process unknown | - | Molecular function unknown | - |
| ISYNA1 | NP_001240318.1 | 51477 | inositol-3-phosphate synthase 1 isoform 4 | 12.8 | 3 | 3 | 3 | 3 | 1.24E+08 | 0.14 | 1 | - | 430 | 47.1 | 5.4 | Metabolism;Energy pathways | - | Isomerase activity | - |
| ASCC2 | NP_001229835.1 | 84164 | activating signal cointegrator 1 complex subunit 2 isoform 2 | 5.1 | 2 | 2 | 2 | 4 | 3.70E+07 | 0.14 | 1 | - | 681 | 77.3 | 5.2 | Regulation of nucleobase, nucleoside, nucleotide and nucleic acid metabolism | Nucleus | Transcription regulator activity | Nucleus |
| HIST1H1D | NP_005311.1 | 3007 | histone H1.3 | 29.9 | 3 | 3 | 11 | 132 | 7.12E+09 | 0.14 | 5 | 20.6 | 221 | 22.3 | 11.0 | Regulation of nucleobase, nucleoside, nucleotide and nucleic acid metabolism | Nucleus;Nucleolus | DNA binding | Nucleus |
| FARS2 | NP_006558.1 | 10667 | phenylalanine--tRNA ligase, mitochondrial precursor | 2.4 | 1 | 1 | 1 | 1 | 2.38E+08 | 0.14 | 1 | - | 451 | 52.3 | 7.5 | Metabolism;Energy pathways | Mitochondrion | Ligase activity | Mitochondrion |
| PPP1R13L | NP_001135974.1 | 10848 | relA-associated inhibitor | 19.8 | 1 | 9 | 9 | 14 | 1.27E+08 | 0.13 | 4 | 24.6 | 828 | 89 | 6.8 | Apoptosis | Nucleus;Cytoplasm | Translation regulator activity | Nucleus;Cytoplasm |
| TOP2A | NP_001058.2 | 7153 | DNA topoisomerase 2-alpha | 16.1 | 1 | 14 | 17 | 25 | 2.22E+08 | 0.13 | 8 | 70.2 | 1531 | 174.3 | 8.7 | Regulation of nucleobase, nucleoside, nucleotide and nucleic acid metabolism | Nucleus;Nucleolus;Kinetochore | DNA topoisomerase activity | Nucleus |
| C18orf25 | NP_001008240.1 | 147339 | uncharacterized protein C18orf25 isoform b | 5.5 | 2 | 1 | 1 | 1 | 4.34E+07 | 0.13 | 1 | - | 343 | 37 | 5.1 | Biological_process unknown | Nucleus;Cytoplasm | Molecular function unknown | Nucleus;Cytoplasm |
| FASN | NP_004095.4 | 2194 | fatty acid synthase | 61.6 | 1 | 117 | 117 | 799 | 7.16E+09 | 0.13 | 149 | 30.3 | 2511 | 273.3 | 6.4 | Metabolism;Energy pathways | Cytoplasm;Cytosol;Mitochondrion;Nucleus | Catalytic activity | Cytoplasm |
| SH3BP1 | NP_061830.3 | 23616 | SH3 domain-binding protein 1 | 17.8 | 1 | 8 | 8 | 12 | 9.71E+07 | 0.13 | 3 | 28 | 701 | 75.7 | 6.8 | Cell communication;Signal transduction | - | GTPase activator activity | - |
| MYH14 | NP_079005.3 | 79784 | myosin-14 isoform 2 | 11.7 | 10 | 8 | 19 | 72 | 2.46E+09 | 0.12 | 1 | - | 1995 | 227.7 | 5.6 | Cell growth and/or maintenance | - | Structural molecule activity | - |
| SLC7A5 | NP_003477.4 | 8140 | large neutral amino acids transporter small subunit 1 | 6.7 | 1 | 3 | 3 | 8 | 1.19E+09 | 0.12 | 6 | 6.2 | 507 | 55 | 7.7 | Transport | Plasma membrane;Mitochondrion | Transporter activity | Plasma membrane |
| KRT18 | NP_954657.1 | 3875 | keratin, type I cytoskeletal 18 | 87.0 | 6 | 46 | 48 | 1055 | 1.21E+10 | 0.12 | 131 | 28.4 | 430 | 48 | 5.5 | Cell growth and/or maintenance | Cytoplasm;Nucleus;Nucleolus;Extracellular;Cytoskeleton | Structural constituent of cytoskeleton | Cytoplasm |

| Gene Symbol | RefSeq_Accession | Entrez Gene ID | Description | Coverage (%) | Proteins | Unique Peptides | # Peptides | PSM | Area | SCC-R /SCC-S | SCC-R /SCC-S (Count) | SCC-R /SCC-S (Variability [%]) | # AAs | MW [kDa] | calc. pI | Biological Process | Localization | Molecular Function | Primary Localization |
|---|---|---|---|---|---|---|---|---|---|---|---|---|---|---|---|---|---|---|---|
| **FERMT1** | NP_060141.3 | 55612 | **fermitin family homolog 1** | 5.6 | 1 | 3 | 3 | 5 | 3.23E+07 | **0.12** | 2 | 60.5 | 677 | 77.4 | 6.3 | Cell growth and/or maintenance | Cytoplasm | Structural molecule activity | Cytoplasm |
| **THBS1** | NP_003237.2 | 7057 | **thrombospondin-1 precursor** | 4.7 | 2 | 3 | 3 | 5 | 7.35E+07 | **0.12** | 3 | 5.3 | 1170 | 129.3 | 4.9 | Cell growth and/or maintenance | Extracellular | Extracellular matrix structural constituent | Extracellular |
| **RFC1** | NP_002904.3 | 5981 | **replication factor C subunit 1 isoform 1** | 10.1 | 2 | 8 | 8 | 12 | 5.34E+07 | **0.11** | 3 | 101.6 | 1147 | 128.1 | 9.4 | Regulation of nucleobase, nucleoside, nucleotide and nucleic acid metabolism | Nucleus;Nucleolus;Cytoplasm | DNA binding | Nucleus;Cytoplasm |
| **STAP2** | NP_001013863.1 | 55620 | **signal-transducing adaptor protein 2 isoform 2** | 12.7 | 2 | 2 | 2 | 3 | 2.26E+07 | **0.11** | 1 | - | 403 | 44.9 | 8.2 | Cell communication;Signal transduction;Regulation of signal transduction | Cytoplasm;Nucleus;Plasma membrane | Receptor signaling complex scaffold activity | Cytoplasm |
| **DHRS7B** | NP_056325.2 | 25979 | **dehydrogenase/reductase SDR family member 7B** | 17.2 | 1 | 4 | 4 | 8 | 5.54E+07 | **0.11** | 3 | 195.1 | 325 | 35.1 | 9.6 | Metabolism;Energy pathways | Cytosol | Oxidoreductase activity | Cytosol |
| **F11R** | NP_058642.1 | 50848 | **junctional adhesion molecule A precursor** | 27.1 | 1 | 5 | 5 | 16 | 3.28E+08 | **0.11** | 4 | 30.3 | 299 | 32.6 | 7.9 | Cell communication;Signal transduction | Cell junction;Cell surface | Cell adhesion molecule activity | Cell junction |
| **ESRP2** | NP_079215.2 | 80004 | **epithelial splicing regulatory protein 2** | 9.9 | 1 | 4 | 5 | 8 | 1.35E+08 | **0.10** | 1 | - | 717 | 77.3 | 6.8 | Regulation of nucleobase, nucleoside, nucleotide and nucleic acid metabolism | Nucleus | RNA binding | Nucleus |
| **AP1G2** | NP_001269404.1 | 8906 | **AP-1 complex subunit gamma-like 2 isoform 3** | 10.9 | 3 | 4 | 4 | 5 | 1.16E+08 | **0.10** | 3 | 37.5 | 713 | 79.1 | 6.9 | Transport | Perinuclear region;Cytosol;Golgi apparatus | Transporter activity | Cytosol |
| **KRT85** | NP_002274.1 | 3891 | **keratin, type II cuticular Hb5** | 5.1 | 4 | 2 | 4 | 34 | 5.45E+09 | **0.10** | 1 | - | 507 | 55.8 | 6.6 | Cell growth and/or maintenance | Cytoplasm | Structural constituent of cytoskeleton | Cytoplasm |
| **SPARC** | NP_003109.1 | 6678 | **SPARC precursor** | 12.2 | 1 | 2 | 2 | 2 | 2.83E+07 | **0.10** | 1 | - | 303 | 34.6 | 4.8 | Cell growth and/or maintenance | Extracellular;Cytoplasm | Extracellular matrix structural constituent | Extracellular |
| **HIST1H1B** | NP_005313.1 | 3009 | **histone H1.5** | 33.6 | 1 | 11 | 12 | 107 | 6.53E+09 | **0.09** | 22 | 53.9 | 226 | 22.6 | 10.9 | Regulation of nucleobase, nucleoside, nucleotide and nucleic acid metabolism | Nucleus;Nucleolus;Mitochondrion | DNA binding | Nucleus |
| **ZNF638** | NP_001239542.1 | 27332 | **zinc finger protein 638 isoform 2** | 7.4 | 2 | 8 | 8 | 11 | 1.09E+08 | **0.09** | 2 | 65.5 | 1957 | 218.1 | 6.4 | Regulation of nucleobase, nucleoside, nucleotide and nucleic acid metabolism | Nuclear matrix;Nucleoplasm;Cytoplasm;Nucleus | DNA binding | Nuclear matrix;Nucleus |
| **FAR1** | NP_115604.1 | 84188 | **fatty acyl-CoA reductase 1** | 11.3 | 1 | 5 | 5 | 6 | 1.76E+08 | **0.09** | 2 | 34.6 | 515 | 59.3 | 9.2 | Metabolism;Energy pathways | - | Catalytic activity | - |
| **GOSR1** | NP_004862.1 | 9527 | **Golgi SNAP receptor complex member 1 isoform 1** | 22.0 | 3 | 4 | 4 | 5 | 7.52E+07 | **0.09** | 1 | - | 250 | 28.6 | 9.4 | Transport | Golgi apparatus;Nucleus | Auxiliary transport protein activity | Golgi apparatus;Nucleus |
| **KRT8** | NP_001243222.1 | 3856 | **keratin, type II cytoskeletal 8 isoform 2** | 86.8 | 8 | 57 | 67 | 1509 | 2.88E+10 | **0.09** | 130 | 34.1 | 483 | 53.7 | 5.6 | Cell growth and/or maintenance | Cytoplasm;Nucleolus;Extracellular;Cytoskeleton;Nucleus | Structural molecule activity | Cytoplasm |
| **ITGB6** | NP_001269284.1 | 3694 | **integrin beta-6 isoform c precursor** | 2.6 | 3 | 1 | 1 | 1 | 1.76E+08 | **0.09** | 1 | - | 681 | 74.9 | 6.0 | Cell communication;Signal transduction | Plasma membrane | Receptor activity | Plasma membrane |
| **CTNNA1** | NP_001894.2 | 1495 | **catenin alpha-1 isoform 1** | 62.7 | 8 | 43 | 43 | 111 | 1.30E+09 | **0.09** | 29 | 34.9 | 906 | 100 | 6.3 | Cell growth and/or maintenance | Cytoplasm;Plasma membrane;Cell junction | Structural constituent of cytoskeleton | Cytoplasm |
| **AKR1B10** | NP_064695.3 | 57016 | **aldo-keto reductase family 1 member B10** | 3.2 | 1 | 1 | 1 | 1 | 3.60E+07 | **0.09** | 1 | - | 316 | 36 | 7.8 | Metabolism;Energy pathways | - | Catalytic activity | - |
| **KCTD12** | NP_612453.1 | 115207 | **BTB/POZ domain-containing protein KCTD12** | 32.0 | 1 | 9 | 9 | 17 | 1.87E+08 | **0.07** | 1 | - | 325 | 35.7 | 5.6 | Transport | - | Ion channel activity | - |
| **HMGCS1** | NP_002121.4 | 3157 | **hydroxymethylglutaryl-CoA synthase, cytoplasmic** | 19.8 | 1 | 5 | 5 | 6 | 8.78E+07 | **0.07** | 2 | 7.1 | 520 | 57.3 | 5.4 | Metabolism;Energy pathways | Cytoplasm | CoA-ligase activity | Cytoplasm |
| **MISP** | NP_775752.1 | 126353 | **mitotic interactor and substrate of PLK1** | 6.0 | 1 | 3 | 3 | 3 | 9.49E+07 | **0.07** | 1 | - | 679 | 75.3 | 6.8 | Biological_process unknown | Nucleus;Cytoplasm | Molecular function unknown | Nucleus;Cytoplasm |
| **FDXR** | NP_077728.2 | 2232 | **NADPH:adrenodoxin oxidoreductase, mitochondrial isoform 1 precursor** | 27.1 | 7 | 7 | 7 | 14 | 1.08E+08 | **0.06** | 3 | 25.7 | 491 | 53.8 | 8.4 | Metabolism;Energy pathways | Mitochondrion | Oxidoreductase activity | Mitochondrion |
| **KRT19** | NP_002267.2 | 3880 | **keratin, type I cytoskeletal 19** | 74.0 | 5 | 24 | 35 | 382 | 1.23E+10 | **0.06** | 32 | 27.3 | 400 | 44.1 | 5.1 | Cell growth and/or maintenance | Cytoplasm | Structural constituent of cytoskeleton | Cytoplasm |
| **KIAA0100** | NP_055495.2 | 9703 | **UPF0378 protein KIAA0100 precursor** | 0.7 | 1 | 1 | 1 | 1 | 2.57E+06 | **0.06** | 1 | - | 2235 | 253.5 | 7.1 | Biological_process unknown | - | Molecular function unknown | - |
| **S100A2** | NP_005969.1 | 6273 | **protein S100-A2** | 50.5 | 1 | 8 | 8 | 209 | 6.73E+09 | **0.06** | 14 | 34 | 97 | 11 | 4.8 | Cell communication;Signal transduction | Nucleus;Cytoplasm | Calcium ion binding | Nucleus |
| **EPB41L1** | NP_001245260.1 | 2036 | **band 4.1-like protein 1 isoform b** | 15.7 | 4 | 6 | 7 | 8 | 1.03E+08 | **0.06** | 1 | - | 779 | 87.6 | 5.9 | Cell growth and/or maintenance | Plasma membrane;Nucleus;Cytoplasm;Cytoskeleton | Cytoskeletal protein binding | Plasma membrane |
| **CGN** | NP_065821.1 | 57530 | **cingulin** | 10.6 | 1 | 5 | 6 | 6 | 1.05E+08 | **0.05** | 2 | 376.3 | 1203 | 137 | 5.5 | Cell growth and/or maintenance | Plasma membrane;Nucleus | Cell adhesion molecule activity | Nucleus |
| **ADIRF** | NP_006820.1 | 10974 | **adipogenesis regulatory factor** | 80.3 | 1 | 6 | 6 | 60 | 3.57E+09 | **0.05** | 3 | 46.5 | 76 | 7.8 | 5.3 | Biological_process unknown | - | Molecular function unknown | - |
| **CLIC3** | NP_004660.2 | 9022 | **chloride intracellular channel protein 3** | 47.0 | 1 | 8 | 8 | 12 | 3.13E+08 | **0.05** | 2 | 14.2 | 236 | 26.6 | 6.4 | Transport | Nucleus | Intracellular ligand-gated ion channel activity | Nucleus |
| **FAM213A** | NP_001230710.1 | 84293 | **redox-regulatory protein FAM213A isoform 2 precursor** | 22.5 | 3 | 5 | 5 | 7 | 2.49E+08 | **0.04** | 1 | - | 218 | 24.4 | 9.0 | Biological_process unknown | - | Molecular function unknown | - |
| **JUP** | NP_002221.1 | 3728 | **junction plakoglobin** | 56.8 | 1 | 22 | 25 | 65 | 7.77E+08 | **0.04** | 10 | 24.2 | 745 | 81.7 | 6.1 | Cell communication;Signal transduction | Cytoplasm;Plasma membrane;Extracellular;Cell junction;Nucleolus;Nucleus;Cytoskeleton | Cell adhesion molecule activity | Cytoplasm |
| **MAP7** | NP_001185546.1 | 9053 | **ensconsin isoform 7** | 8.1 | 8 | 3 | 3 | 3 | 4.44E+07 | **0.04** | 2 | 116.6 | 655 | 72.4 | 9.4 | Cell growth and/or maintenance | Cytoplasm;Nucleus;Microtubule | Cytoskeletal protein binding | Cytoplasm |
| **TRIM29** | NP_036233.2 | 23650 | **tripartite motif-containing protein 29** | 26.0 | 1 | 12 | 12 | 19 | 1.90E+08 | **0.04** | 1 | - | 588 | 65.8 | 7.2 | Regulation of nucleobase, nucleoside, nucleotide and nucleic acid metabolism | Cytoplasm | Transcription regulator activity | Cytoplasm |
| **BCAM** | NP_005572.2 | 4059 | **basal cell adhesion molecule isoform 1 precursor** | 38.4 | 2 | 16 | 16 | 36 | 6.29E+08 | **0.04** | 5 | 8.9 | 628 | 67.4 | 5.8 | Cell communication;Signal transduction;Cell-cell adhesion;Cell-matrix adhesion;Cell adhesion | Plasma membrane | Cell adhesion molecule activity | Plasma membrane |
| **MKI67** | NP_002408.3 | 4288 | **antigen KI-67 isoform 1** | 8.7 | 2 | 16 | 16 | 20 | 1.23E+08 | **0.04** | 5 | 20.9 | 3256 | 358.5 | 9.5 | Cell communication;Signal transduction | Nucleolus;Nucleus;Cytoplasm;Chromosome | Protein binding | Nucleolus |
| **ITGB4** | NP_001005731.1 | 3691 | **integrin beta-4 isoform 3 precursor** | 31.3 | 3 | 34 | 34 | 53 | 6.07E+08 | **0.04** | 7 | 9.9 | 1752 | 194.9 | 6.1 | Cell communication;Signal transduction;Cell migration;Cell adhesion;Wound healing | Integral to membrane;Nucleus | Cell adhesion molecule activity | Integral to membrane;Nucleus |
| **DSP** | NP_004406.2 | 1832 | **desmoplakin isoform I** | 44.0 | 2 | 101 | 101 | 259 | 1.45E+09 | **0.04** | 37 | 41.2 | 2871 | 331.6 | 6.8 | Cell growth and/or maintenance | Plasma membrane;Cytoplasm;Nucleus;Nucleolus;Extracellular;Cytoskeleton | Structural constituent of cytoskeleton | Cytoplasm |

| Gene Symbol | RefSeq_Accession | Entrez Gene ID | Description | Coverage (%) | Proteins | Unique Peptides | # Peptides | PSM | Area | SCC-R /SCC-S | SCC-R /SCC-S (Count) | SCC-R /SCC-S (Variability [%]) | # AAs | MW [kDa] | calc. pI | Biological Process | Localization | Molecular Function | Primary Localization |
|---|---|---|---|---|---|---|---|---|---|---|---|---|---|---|---|---|---|---|---|
| KRT7 | NP_005547.3 | 3855 | keratin, type II cytoskeletal 7 | 70.2 | 7 | 42 | 50 | 439 | 8.59E+09 | 0.04 | 53 | 51.9 | 469 | 51.4 | 5.5 | Cell growth and/or maintenance | Cytoplasm;Plasma membrane | Structural constituent of cytoskeleton | Cytoplasm;Plasma membrane |
| TRIM16 | NP_006461.3 | 10626 | tripartite motif-containing protein 16 | 23.8 | 2 | 9 | 9 | 14 | 2.41E+08 | 0.03 | 2 | 179.9 | 564 | 63.9 | 5.5 | Cell growth and/or maintenance | Cytoplasm;Nucleus | Structural constituent of cytoskeleton | Cytoplasm |
| PKP3 | NP_009114.1 | 11187 | plakophilin-3 | 36.8 | 1 | 18 | 18 | 32 | 3.13E+08 | 0.03 | 2 | 279.3 | 797 | 87 | 9.3 | Cell communication;Signal transduction | Plasma membrane;Nucleus | Cell adhesion molecule activity | Plasma membrane |
| SERPINB5 | NP_002630.2 | 5268 | serpin B5 | 55.7 | 1 | 15 | 15 | 32 | 8.89E+08 | 0.03 | 5 | 161.9 | 375 | 42.1 | 6.1 | Protein metabolism | Extracellular;Plasma membrane;Cytoplasm;Endoplasmic reticulum;Cytoplasmic vesicle | Protease inhibitor activity | Cytoplasm |
| ALDH1A3 | NP_000684.2 | 220 | aldehyde dehydrogenase family 1 member A3 | 52.9 | 4 | 21 | 21 | 60 | 9.26E+08 | 0.03 | 7 | 226.8 | 512 | 56.1 | 7.3 | Metabolism;Energy pathways | Cytoplasm | Catalytic activity | Cytoplasm |
| SFN | NP_006133.1 | 2810 | 14-3-3 protein sigma | 79.8 | 1 | 19 | 23 | 378 | 1.96E+10 | 0.03 | 21 | 31.4 | 248 | 27.8 | 4.7 | Cell communication;Signal transduction | Cytoplasm | Receptor signaling complex scaffold activity | Cytoplasm |
| ANXA8 | NP_001035173.1 | 653145 | annexin A8 isoform 2 | 64.5 | 4 | 5 | 23 | 123 | 3.17E+09 | 0.02 | 1 | - | 327 | 36.9 | 5.8 | Signal transduction | - | Calcium ion binding | - |
| PKP2 | NP_001005242.2 | 5318 | plakophilin-2 isoform 2a | 24.0 | 2 | 12 | 12 | 17 | 1.26E+08 | 0.02 | 1 | - | 837 | 92.7 | 9.3 | Cell adhesion | Integral to membrane;Plasma membrane;Desmosome;Nucleus | Molecular function unknown | Nucleus |
| CTNNB1 | NP_001091679.1 | 1499 | catenin beta-1 | 43.7 | 1 | 19 | 22 | 47 | 4.61E+08 | 0.02 | 2 | 3.3 | 781 | 85.4 | 5.9 | Cell communication;Signal transduction | Cytoplasm;Nucleus;Plasma membrane;Basolateral membrane;Perinuclear region;Cell junction;Cytoskeleton | Cell adhesion molecule activity | Plasma membrane;Nucleus;Cytoplasm |
| KRT14 | NP_000517.2 | 3861 | keratin, type I cytoskeletal 14 | 78.2 | 9 | 19 | 48 | 428 | 1.10E+10 | 0.02 | 5 | 22.3 | 472 | 51.6 | 5.2 | Cell growth and/or maintenance | Cytoplasm | Structural constituent of cytoskeleton | Cytoplasm |
| SCD | NP_005054.3 | 6319 | acyl-CoA desaturase | 23.7 | 1 | 9 | 9 | 21 | 6.34E+08 | 0.02 | 3 | 5.8 | 359 | 41.5 | 9.0 | Metabolism;Energy pathways | Endoplasmic reticulum;Nucleus;Mitochondrion | Oxidoreductase activity | Endoplasmic reticulum |
| EPPK1 | NP_112598.3 | 83481 | epiplakin | 47.7 | 1 | 53 | 59 | 144 | 1.02E+09 | 0.02 | 5 | 19.4 | 5088 | 555.3 | 5.6 | Cell growth and/or maintenance | Cytoplasm;Nucleus | Cytoskeletal protein binding | Cytoplasm |
| KRT13 | NP_705694.2 | 3860 | keratin, type I cytoskeletal 13 isoform a | 72.7 | 13 | 27 | 41 | 389 | 8.74E+09 | 0.02 | 6 | 21.3 | 458 | 49.5 | 5.0 | Cell growth and/or maintenance | Cytoplasm;Plasma membrane;Nucleus | Structural molecule activity | Cytoplasm;Nucleus |
| KRT17 | NP_000413.1 | 3872 | keratin, type I cytoskeletal 17 | 85.2 | 5 | 27 | 45 | 488 | 1.08E+10 | 0.02 | 8 | 34.7 | 432 | 48.1 | 5.0 | Cell growth and/or maintenance | Cytoplasm;Nucleolus;Extracellular;Nucleus | Structural constituent of cytoskeleton | Cytoplasm |
| GALNT3 | NP_004473.2 | 2591 | polypeptide N-acetylgalactosaminyltransferase 3 | 7.6 | 1 | 3 | 3 | 7 | 3.12E+08 | 0.02 | 2 | 40.5 | 633 | 72.6 | 8.0 | Protein metabolism | Golgi apparatus | Galactosyltransferase activity | Golgi apparatus |
| KRT5 | NP_000415.2 | 3852 | keratin, type II cytoskeletal 5 | 59.5 | 7 | 39 | 55 | 561 | 1.16E+10 | 0.02 | 9 | 51.5 | 590 | 62.3 | 7.7 | Cell growth and/or maintenance | Cytoplasm;Nucleus | Structural molecule activity | Cytoplasm |
| EPCAM | NP_002345.2 | 4072 | epithelial cell adhesion molecule precursor | 23.9 | 1 | 7 | 7 | 18 | 3.15E+08 | 0.01 | 1 | - | 314 | 34.9 | 7.5 | Cell communication;Signal transduction | Plasma membrane;Extracellular | Cell adhesion molecule activity | Plasma membrane |
| ITGA2 | NP_002194.2 | 3673 | integrin alpha-2 precursor | 13.2 | 1 | 10 | 10 | 24 | 1.88E+08 | 1.94 | 5 | 5.9 | 1181 | 129.2 | 5.3 | Cell communication;Signal transduction | Plasma membrane;Cytoplasm | Receptor activity | Plasma membrane |
| UROS | NP_000366.1 | 7390 | uroporphyrinogen-III synthase | 5.7 | 1 | 1 | 1 | 2 | 4.06E+07 | 1.94 | 1 | - | 265 | 28.6 | 5.5 | Metabolism;Energy pathways | Cytoplasm | Lyase activity | Cytoplasm |
| TRIP10 | NP_004231.1 | 9322 | cdc42-interacting protein 4 isoform 2 | 22.4 | 3 | 7 | 7 | 15 | 1.30E+08 | 1.94 | 4 | 19.9 | 545 | 62.6 | 5.3 | Cell growth and/or maintenance | Nucleus;Cytoplasm;Plasma membrane | Receptor signaling complex scaffold activity | Cytoplasm |
| HPRT1 | NP_000185.1 | 3251 | hypoxanthine-guanine phosphoribosyltransferase | 67.4 | 3 | 13 | 13 | 39 | 1.04E+09 | 1.94 | 11 | 19.7 | 218 | 24.6 | 6.7 | Metabolism;Energy pathways | Cytoplasm | Catalytic activity | Cytoplasm |
| TSC22D2 | NP_055594.1 | 9819 | TSC22 domain family protein 2 | 7.6 | 1 | 2 | 2 | 3 | 1.26E+08 | 1.93 | 1 | - | 780 | 79.2 | 5.0 | Signal transduction;Cell communication | - | Molecular function unknown | - |
| STAM2 | NP_005834.4 | 10254 | signal transducing adapter molecule 2 | 18.1 | 1 | 4 | 5 | 6 | 1.09E+08 | 1.93 | 3 | 8.7 | 525 | 58.1 | 5.1 | Cell communication;Signal transduction | Cytoplasm | Receptor signaling complex scaffold activity | Cytoplasm |
| TXNDC12 | NP_056997.1 | 51060 | thioredoxin domain-containing protein 12 precursor | 25.6 | 1 | 4 | 4 | 5 | 1.11E+08 | 1.93 | 3 | 16 | 172 | 19.2 | 5.4 | Protein metabolism | Golgi apparatus;Endoplasmic reticulum;Mitochondrion | Catalytic activity | Endoplasmic reticulum |
| THUMPD1 | NP_060206.2 | 55623 | THUMP domain-containing protein 1 | 21.0 | 1 | 6 | 6 | 13 | 8.53E+07 | 1.93 | 4 | 21.2 | 353 | 39.3 | 7.9 | Biological_process unknown | Cytoplasm | Molecular function unknown | Cytoplasm |
| NASP | NP_002473.2 | 4678 | nuclear autoantigenic sperm protein isoform 2 | 77.5 | 3 | 40 | 40 | 234 | 1.21E+09 | 1.93 | 47 | 17.9 | 788 | 85.2 | 4.3 | Cell communication;Signal transduction;Regulation of cell cycle | Nucleus;Cytoplasm;Golgi apparatus | Histone binding | Nucleus;Cytoplasm |
| NPEPL1 | NP_001191802.1 | 79716 | probable aminopeptidase NPEPL1 isoform 3 | 5.7 | 3 | 2 | 2 | 6 | 4.17E+07 | 1.93 | 1 | - | 475 | 50.5 | 6.5 | Protein metabolism | - | Aminopeptidase activity | - |
| TBL1XR1 | NP_078941.2 | 79718 | F-box-like/WD repeat-containing protein TBL1XR1 | 23.0 | 3 | 5 | 7 | 13 | 6.73E+07 | 1.92 | 4 | 54.4 | 514 | 55.6 | 5.6 | Regulation of nucleobase, nucleoside, nucleotide and nucleic acid metabolism | Nucleus | Transcription regulator activity | Nucleus |
| EHD1 | NP_001269373.1 | 10938 | EH domain-containing protein 1 isoform 1 | 52.4 | 2 | 19 | 22 | 80 | 7.73E+08 | 1.92 | 24 | 30.5 | 534 | 60.6 | 6.8 | Cell communication;Signal transduction | Plasma membrane;Endosome;Cytoplasm | Calcium ion binding | Plasma membrane |
| TAMM41 | NP_620162.1 | 132001 | mitochondrial translocator assembly and maintenance protein 41 homolog isoform b precursor | 18.0 | 2 | 3 | 3 | 3 | 6.96E+07 | 1.92 | 2 | 27.5 | 316 | 35.9 | 8.8 | Biological_process unknown | - | Molecular function unknown | - |
| GOLPH3L | NP_060648.2 | 55204 | Golgi phosphoprotein 3-like | 33.0 | 1 | 4 | 4 | 7 | 7.50E+07 | 1.92 | 2 | 17.2 | 285 | 32.7 | 5.8 | Biological_process unknown | - | Molecular function unknown | - |
| DCP1A | NP_001277136.1 | 55802 | mRNA-decapping enzyme 1A isoform e | 6.2 | 5 | 2 | 2 | 2 | 3.12E+07 | 1.92 | 2 | 13.7 | 423 | 45.3 | 6.5 | Regulation of nucleobase, nucleoside, nucleotide and nucleic acid metabolism | Nucleus;Cytoplasm | Transcription factor activity | Nucleus |
| PTK2B | NP_775267.1 | 2185 | protein-tyrosine kinase 2-beta isoform b | 2.3 | 2 | 1 | 1 | 1 | 5.09E+07 | 1.92 | 1 | - | 967 | 111.1 | 5.9 | Signal transduction | Cytoplasm;Nucleus;Nucleoplasm;Plasma membrane;Focal adhesion;Perinuclear region | Protein-tyrosine kinase activity | Cytoplasm |
| RNMT | NP_003790.1 | 8731 | mRNA cap guanine-N7 methyltransferase | 2.9 | 1 | 1 | 1 | 2 | 2.67E+07 | 1.92 | 2 | 57.1 | 476 | 54.8 | 6.6 | Regulation of nucleobase, nucleoside, nucleotide and nucleic acid metabolism | Cytoplasm;Nucleus | RNA methyltransferase activity | Cytoplasm |
| TP53BP2 | NP_005417.1 | 7159 | apoptosis-stimulating of p53 protein 2 isoform 2 | 1.6 | 2 | 1 | 1 | 2 | 2.71E+07 | 1.92 | 1 | - | 1005 | 111.4 | 6.0 | Cell communication;Signal transduction | Cytoplasm;Nucleus | Protein binding | Cytoplasm;Nucleus |
| PEX26 | NP_001186248.1 | 55670 | peroxisome assembly protein 26 isoform b | 4.3 | 2 | 1 | 1 | 1 | 1.14E+06 | 1.92 | 1 | - | 256 | 28.4 | 5.5 | Cell growth and/or maintenance | Peroxisome | Molecular function unknown | Peroxisome |
| SMARCD1 | NP_620710.2 | 6602 | SWI/SNF-related matrix-associated actin-dependent regulator of chromatin subfamily D member 1 isoform b | 13.3 | 4 | 3 | 4 | 5 | 2.23E+07 | 1.92 | 1 | - | 474 | 53.3 | 9.4 | Regulation of nucleobase, nucleoside, nucleotide and nucleic acid metabolism | Nucleus;Kinetochore | Transcription regulator activity | Nucleus |

Supplementary Table S4. List of all proteins identified in SCC-S and SCC-R cells using SequestHT and Mascot search algorithms and their gene ontology annotation

| Gene Symbol | RefSeq_Accession | Entrez Gene ID | Description | Coverage (%) | Proteins | Unique Peptides | # Peptides | PSM | Area | SCC-R /SCC-S | SCC-R /SCC-S (Count) | SCC-R /SCC-S (Variability [%]) | # AAs | MW [kDa] | calc. pI | Biological Process | Localization | Molecular Function | Primary Localization |
|---|---|---|---|---|---|---|---|---|---|---|---|---|---|---|---|---|---|---|---|
| AK2 | NP_037543.1 | 204 | adenylate kinase 2, mitochondrial isoform b | 81.5 | 3 | 21 | 21 | 136 | 2.07E+09 | 1.91 | 44 | 19.6 | 232 | 25.6 | 7.9 | Metabolism;Energy pathways | Mitochondrion;Cytoplasm | Catalytic activity | Mitochondrion |
| CTSC | NP_001805.3 | 1075 | dipeptidyl peptidase 1 isoform a preproprotein | 23.3 | 3 | 10 | 10 | 45 | 2.96E+08 | 1.91 | 11 | 26.6 | 463 | 51.8 | 7.0 | Protein metabolism | Lysosome;Mitochondrion | Cysteine-type peptidase activity | Lysosome |
| STX7 | NP_003560.2 | 8417 | syntaxin-7 | 26.1 | 1 | 4 | 4 | 19 | 2.65E+08 | 1.90 | 5 | 16.9 | 261 | 29.8 | 5.6 | Transport | Cytoplasm;Endosome;Lysosome;Extracellular | Protein binding | Endosome |
| MPST | NP_001013454.1 | 4357 | 3-mercaptopyruvate sulfurtransferase isoform 2 | 30.0 | 2 | 5 | 5 | 10 | 8.16E+08 | 1.90 | 5 | 12.3 | 297 | 33.2 | 6.6 | Metabolism;Energy pathways | Mitochondrion | Sulfotransferase activity | Mitochondrion |
| STAT2 | NP_938146.1 | 6773 | signal transducer and activator of transcription 2 isoform 2 | 6.5 | 2 | 3 | 3 | 6 | 3.08E+08 | 1.90 | 3 | 25.5 | 847 | 97.4 | 5.5 | Regulation of nucleobase, nucleoside, nucleotide and nucleic acid metabolism | Cytoplasm;Nucleus | Transcription factor activity | Cytoplasm |
| DST | NP_001714.1 | 667 | dystonin isoform 1e precursor | 6.6 | 1 | 3 | 10 | 17 | 2.19E+09 | 1.90 | 1 | - | 2649 | 306.6 | 6.3 | Cell growth and/or maintenance | Nucleus;Plasma membrane;Extracellular;Cytoplasm | Cytoskeletal protein binding | Plasma membrane |
| ALG2 | NP_149078.1 | 85365 | alpha-1,3/1,6-mannosyltransferase ALG2 | 7.9 | 1 | 1 | 1 | 1 | 9.77E+06 | 1.90 | 1 | - | 416 | 47.1 | 7.1 | Metabolism;Energy pathways | Endoplasmic reticulum | Mannosyltransferase activity | Endoplasmic reticulum |
| ATG3 | NP_001265641.1 | 64422 | ubiquitin-like-conjugating enzyme ATG3 isoform 2 | 51.8 | 2 | 9 | 9 | 22 | 4.30E+08 | 1.90 | 7 | 25.2 | 311 | 35.4 | 4.8 | Biological_process unknown | Cytoplasm | Molecular function unknown | Cytoplasm |
| DUSP3 | NP_004081.1 | 1845 | dual specificity protein phosphatase 3 | 29.7 | 1 | 4 | 4 | 6 | 6.26E+07 | 1.89 | 2 | 1.6 | 185 | 20.5 | 7.8 | Cell communication;Signal transduction | Nucleus;Cytoplasm | Protein tyrosine/serine/threonine phosphatase activity | Nucleus |
| PRKAR1A | NP_001265362.1 | 5573 | cAMP-dependent protein kinase type I-alpha regulatory subunit isoform a | 31.8 | 2 | 10 | 10 | 40 | 2.19E+08 | 1.89 | 14 | 31.8 | 381 | 43 | 5.4 | Cell communication;Signal transduction | Cytoplasm | Protein serine/threonine kinase activity | Cytoplasm |
| KATNAL1 | NP_001014402.1 | 84056 | katanin p60 ATPase-containing subunit A-like 1 | 3.7 | 1 | 1 | 1 | 1 | 2.13E+06 | 1.89 | 1 | - | 490 | 55.4 | 6.7 | Metabolism;Energy pathways | - | ATPase activity | - |
| OXCT1 | NP_000427.1 | 5019 | succinyl-CoA:3-ketoacid coenzyme A transferase 1, mitochondrial precursor | 24.6 | 2 | 10 | 10 | 28 | 2.97E+08 | 1.88 | 9 | 19.2 | 520 | 56.1 | 7.5 | Metabolism;Energy pathways | Mitochondrion | CoA-transferase activity | Mitochondrion |
| CFL2 | NP_619579.1 | 1073 | cofilin-2 isoform 1 | 64.5 | 2 | 6 | 12 | 209 | 5.77E+09 | 1.88 | 9 | 25.4 | 166 | 18.7 | 7.9 | Biological_process unknown;Cytoskeleton organization and biogenesis | Cytoplasm;Perinuclear region;Nucleus | Cytoskeletal protein binding | Cytoplasm |
| EIF6 | NP_001254739.1 | 3692 | eukaryotic translation initiation factor 6 isoform a | 58.0 | 2 | 8 | 8 | 49 | 1.46E+09 | 1.88 | 12 | 10.8 | 245 | 26.6 | 4.7 | Regulation of translation | Nucleolus;Nucleus;Cytoplasm;Cytoskeleton;Chromosome;Nuclear matrix;Desmosome;Ribosome | Translation regulator activity | Cytoplasm |
| FLNC | NP_001120959.1 | 2318 | filamin-C isoform b | 14.2 | 2 | 16 | 25 | 67 | 1.79E+09 | 1.87 | 14 | 13 | 2692 | 287.1 | 6.0 | Cell growth and/or maintenance | Cytoplasm | Cytoskeletal protein binding | Cytoplasm |
| ARL8B | NP_060654.1 | 55207 | ADP-ribosylation factor-like protein 8B | 43.6 | 1 | 3 | 7 | 26 | 6.54E+08 | 1.87 | 3 | 10.3 | 186 | 21.5 | 8.4 | Cell communication;Signal transduction | Cytoskeleton | GTPase activity | Cytoskeleton |
| ANGEL2 | NP_653168.2 | 90806 | protein angel homolog 2 | 3.3 | 1 | 1 | 1 | 2 | 2.81E+06 | 1.87 | 1 | - | 544 | 62.3 | 7.8 | Biological_process unknown | - | Molecular function unknown | - |
| TIA1 | NP_071505.2 | 7072 | nucleolysin TIA-1 isoform p40 isoform 2 | 10.6 | 1 | 2 | 3 | 9 | 8.62E+07 | 1.87 | 2 | 85.8 | 386 | 42.9 | 7.7 | Regulation of nucleobase, nucleoside, nucleotide and nucleic acid metabolism | Cytoplasm;Nucleus | RNA binding | Cytoplasm |
| CASP4 | NP_001216.1 | 837 | caspase-4 isoform alpha precursor | 57.3 | 6 | 13 | 13 | 70 | 6.57E+08 | 1.87 | 26 | 30.4 | 377 | 43.2 | 6.0 | Apoptosis | Cytoplasm;Endoplasmic reticulum;Mitochondrion | Cysteine-type peptidase activity | Cytoplasm |
| TCIRG1 | NP_006010.2 | 10312 | V-type proton ATPase 116 kDa subunit a isoform 3 isoform a | 10.5 | 2 | 6 | 6 | 9 | 5.10E+08 | 1.87 | 5 | 38.2 | 830 | 92.9 | 7.1 | Transport | Integral to membrane;Brush border;Cytosol | Transporter activity | Integral to membrane |
| COQ9 | NP_064708.1 | 57017 | ubiquinone biosynthesis protein COQ9, mitochondrial precursor | 13.5 | 1 | 3 | 3 | 6 | 1.50E+07 | 1.86 | 2 | 21.2 | 318 | 35.5 | 5.9 | Biological_process unknown | - | Molecular function unknown | - |
| NDUFAF2 | NP_777549.1 | 91942 | mimitin, mitochondrial | 24.9 | 1 | 3 | 3 | 4 | 5.63E+07 | 1.86 | 3 | 8.4 | 169 | 19.8 | 9.0 | Cell proliferation | Mitochondrion | Molecular function unknown | Mitochondrion |
| NIPSNAP3A | NP_056284.1 | 25934 | protein NipSnap homolog 3A | 9.3 | 1 | 1 | 1 | 1 | 1.27E+08 | 1.86 | 1 | - | 247 | 28.4 | 9.2 | Transport | Plasma membrane;Cytosol;Mitochondrion | Transporter activity | Plasma membrane |
| AMPD2 | NP_631895.1 | 271 | AMP deaminase 2 isoform 2 | 7.3 | 4 | 5 | 5 | 8 | 7.39E+07 | 1.86 | 4 | 19.4 | 798 | 92 | 6.3 | Metabolism;Energy pathways | Cytoplasm;Nucleus | Deaminase activity | Cytoplasm;Nucleus |
| TPM3 | NP_001265118.1 | 7170 | tropomyosin alpha-3 chain isoform 7 | 86.7 | 4 | 2 | 34 | 387 | 8.25E+09 | 1.86 | 1 | - | 248 | 28.9 | 4.8 | Cell growth and/or maintenance | Cytoplasm;Extracellular;Cytosol | Cytoskeletal protein binding | Cytoplasm |
| TWF2 | NP_009215.1 | 11344 | twinfilin-2 | 47.3 | 1 | 13 | 13 | 35 | 2.01E+08 | 1.86 | 9 | 19 | 349 | 39.5 | 6.8 | Cell communication;Signal transduction | Cytoplasm | Protein-tyrosine kinase activity | Cytoplasm |
| IFI16 | NP_005522.2 | 3428 | gamma-interferon-inducible protein 16 isoform 2 | 21.7 | 6 | 11 | 11 | 18 | 1.98E+08 | 1.86 | 10 | 36.6 | 729 | 82 | 9.3 | Regulation of cell growth | Nucleus;Nucleolus | Transcription regulator activity | Nucleus |
| CTBP1 | NP_001012632.1 | 1487 | C-terminal-binding protein 1 isoform 2 | 18.2 | 2 | 5 | 5 | 11 | 2.73E+08 | 1.86 | 5 | 21.1 | 429 | 46.4 | 6.7 | Regulation of nucleobase, nucleoside, nucleotide and nucleic acid metabolism | Nucleus;Cytoplasm | Transcription regulator activity | Nucleus |
| ZC3H7B | NP_060060.3 | 23264 | zinc finger CCCH domain-containing protein 7B | 5.5 | 1 | 2 | 2 | 2 | 2.22E+07 | 1.86 | 1 | - | 977 | 109.8 | 7.3 | Protein metabolism | Nucleus | Translation regulator activity | Nucleus |
| RIN1 | NP_004283.2 | 9610 | ras and Rab interactor 1 | 15.2 | 1 | 5 | 5 | 7 | 3.48E+07 | 1.86 | 3 | 37.5 | 783 | 84 | 8.0 | Cell communication;Signal transduction | Plasma membrane | Molecular function unknown | Plasma membrane |
| NUDCD3 | NP_056147.2 | 23386 | nudC domain-containing protein 3 | 28.5 | 1 | 4 | 4 | 8 | 2.55E+08 | 1.85 | 2 | 68.6 | 361 | 40.8 | 5.3 | Biological_process unknown | - | Molecular function unknown | - |
| PPT1 | NP_000301.1 | 5538 | palmitoyl-protein thioesterase 1 isoform 1 precursor | 33.7 | 2 | 6 | 6 | 16 | 4.20E+08 | 1.85 | 6 | 2.2 | 306 | 34.2 | 6.5 | Metabolism;Energy pathways | Lysosome | Hydrolase activity | Lysosome |
| ECHDC1 | NP_001099014.1 | 55862 | ethylmalonyl-CoA decarboxylase isoform 3 | 24.3 | 5 | 4 | 4 | 5 | 5.23E+07 | 1.85 | 3 | 17.1 | 226 | 24.8 | 7.1 | Biological_process unknown | - | Molecular function unknown | - |
| FAM134C | NP_835227.1 | 162427 | protein FAM134C | 6.4 | 1 | 2 | 2 | 3 | 5.71E+07 | 1.85 | 1 | - | 466 | 51.4 | 5.0 | Biological_process unknown | - | Molecular function unknown | - |
| FIS1 | NP_057152.2 | 51024 | mitochondrial fission 1 protein | 36.8 | 1 | 4 | 4 | 18 | 8.62E+07 | 1.85 | 9 | 15.3 | 152 | 16.9 | 8.8 | Apoptosis | Mitochondrion | Molecular function unknown | Mitochondrion |
| LRRC40 | NP_060238.3 | 55631 | leucine-rich repeat-containing protein 40 | 18.9 | 1 | 7 | 7 | 11 | 1.56E+08 | 1.85 | 6 | 19.1 | 602 | 68.2 | 6.4 | Biological_process unknown | - | Molecular function unknown | - |
| CCDC117 | NP_001271193.1 | 150275 | coiled-coil domain-containing protein 117 isoform c | 14.2 | 3 | 1 | 1 | 1 | 5.51E+06 | 1.85 | 1 | - | 204 | 22.3 | 5.2 | Biological_process unknown | - | Molecular function unknown | - |

Supplementary Table S4. List of all proteins identified in SCC-S and SCC-R cells using SequestHT and Mascot search algorithms and their gene ontology annotation

| Gene Symbol | RefSeq_Accession | Entrez Gene ID | Description | Coverage (%) | Proteins | Unique Peptides | # Peptides | PSM | Area | SCC-R /SCC-S | SCC-R /SCC-S (Count) | SCC-R /SCC-S (Variability [%]) | # AAs | MW [kDa] | calc. pI | Biological Process | Localization | Molecular Function | Primary Localization |
|---|---|---|---|---|---|---|---|---|---|---|---|---|---|---|---|---|---|---|---|
| **HDAC7** | NP_001091886.1 | 51564 | **histone deacetylase 7 isoform d** | 1.3 | 2 | 1 | 1 | 1 | 2.05E+07 | **1.85** | 1 | - | 954 | 102.8 | 7.3 | Regulation of nucleobase, nucleoside, nucleotide and nucleic acid metabolism;Regulation of gene expression, epigenetic | Nucleus;Cytoplasm;Mitochondrial intermembrane space | Deacetylase activity | Nucleus |
| **GBE1** | NP_000149.3 | 2632 | **1,4-alpha-glucan-branching enzyme** | 32.1 | 1 | 13 | 13 | 36 | 7.93E+08 | **1.85** | 14 | 22.3 | 702 | 80.4 | 6.3 | Metabolism;Energy pathways | - | Transferase activity | - |
| **TAP2** | NP_061313.2 | 6891 | **antigen peptide transporter 2 isoform 2** | 27.3 | 3 | 7 | 7 | 10 | 1.40E+08 | **1.85** | 3 | 18.2 | 653 | 72 | 7.8 | Transport | Cytoplasm;Endoplasmic reticulum;Integral to membrane | Transporter activity | Endoplasmic reticulum;Integral to membrane |
| **RAB38** | NP_071732.1 | 23682 | **ras-related protein Rab-38** | 23.2 | 1 | 4 | 4 | 5 | 8.65E+07 | **1.84** | 3 | 2.5 | 211 | 23.7 | 7.8 | Cell communication;Signal transduction | Cytoplasm;Endoplasmic reticulum;Golgi apparatus | GTPase activity | Cytoplasm |
| **CSTF2T** | NP_056050.1 | 23283 | **cleavage stimulation factor subunit 2 tau variant** | 24.7 | 1 | 5 | 7 | 13 | 2.31E+08 | **1.84** | 4 | 42.4 | 616 | 64.4 | 7.3 | Regulation of nucleobase, nucleoside, nucleotide and nucleic acid metabolism | Nucleus | RNA binding | Nucleus |
| **BCL2L1** | NP_001182.1 | 598 | **bcl-2-like protein 1 isoform 2** | 45.3 | 2 | 3 | 3 | 4 | 3.64E+07 | **1.84** | 2 | 26 | 170 | 18.9 | 5.1 | Apoptosis | Mitochondrial membrane;Nucleus;Cytosol;Cytoplasm | Receptor signaling complex scaffold activity | Mitochondrial membrane |
| **PEG10** | NP_001035242.1 | 23089 | **retrotransposon-derived protein PEG10 isoform 2** | 19.4 | 6 | 5 | 5 | 9 | 1.96E+08 | **1.84** | 6 | 28.3 | 325 | 36.9 | 6.2 | Apoptosis | Nucleus;Cytoplasm | Protein binding | Nucleus |
| **CTPS1** | NP_001896.2 | 1503 | **CTP synthase 1** | 30.3 | 2 | 13 | 13 | 34 | 4.94E+08 | **1.84** | 16 | 17.5 | 591 | 66.6 | 6.5 | Metabolism;Energy pathways | Cytoplasm | Ligase activity | Cytoplasm |
| **ZC3HAV1** | NP_064504.2 | 56829 | **zinc finger CCCH-type antiviral protein 1 isoform 1** | 24.9 | 2 | 12 | 12 | 31 | 4.47E+08 | **1.84** | 8 | 20.5 | 902 | 101.4 | 8.4 | Immune response | Cytoplasm;Nucleus | Defense/immunity protein activity | Cytoplasm;Nucleus |
| **DCAF8** | NP_056541.2 | 50717 | **DDB1- and CUL4-associated factor 8** | 8.9 | 1 | 3 | 3 | 3 | 5.68E+07 | **1.83** | 2 | 407.5 | 597 | 66.8 | 5.4 | Biological_process unknown | Nucleus;Cytoplasm | Molecular function unknown | Nucleus;Cytoplasm |
| **LGALS3** | NP_002297.2 | 3958 | **galectin-3 isoform 1** | 34.8 | 2 | 8 | 8 | 29 | 6.44E+08 | **1.83** | 12 | 11.8 | 250 | 26.1 | 8.6 | Regulation of nucleobase, nucleoside, nucleotide and nucleic acid metabolism | Cytoplasm;Nucleus;Extracellular | Transcription regulator activity | Cytoplasm |
| **PMS2** | NP_000526.1 | 5395 | **mismatch repair endonuclease PMS2 isoform a** | 3.3 | 1 | 2 | 2 | 2 | 1.03E+07 | **1.83** | 2 | 17 | 862 | 95.7 | 6.7 | Regulation of nucleobase, nucleoside, nucleotide and nucleic acid metabolism;DNA repair | Nucleus;Cytoplasm | DNA repair protein | Nucleus |
| **ERLEC1** | NP_001120869.1 | 27248 | **endoplasmic reticulum lectin 1 isoform 2 precursor** | 9.2 | 3 | 2 | 2 | 3 | 1.28E+08 | **1.83** | 2 | 25.5 | 457 | 51.9 | 6.1 | Biological_process unknown | - | Molecular function unknown | - |
| **BCAT1** | NP_005495.2 | 586 | **branched-chain-amino-acid aminotransferase, cytosolic isoform 1** | 22.0 | 5 | 6 | 6 | 14 | 3.82E+08 | **1.83** | 7 | 25.8 | 386 | 42.9 | 5.3 | Metabolism;Energy pathways | Cytoplasm;Mitochondrion | Transaminase activity | Cytoplasm |
| **ACADSB** | NP_001600.1 | 36 | **short/branched chain specific acyl-CoA dehydrogenase, mitochondrial precursor** | 11.6 | 1 | 2 | 2 | 3 | 2.17E+08 | **1.83** | 1 | - | 432 | 47.5 | 7.0 | Metabolism;Energy pathways | Mitochondrion | Catalytic activity | Mitochondrion |
| **CLCC1** | NP_001265132.1 | 23155 | **chloride channel CLIC-like protein 1 isoform 4 precursor** | 15.0 | 4 | 2 | 2 | 2 | 6.47E+06 | **1.83** | 1 | - | 366 | 39.8 | 5.7 | Transport | Endoplasmic reticulum;Golgi apparatus;Nucleus | Ion channel activity | Endoplasmic reticulum |
| **CDK5** | NP_001157882.1 | 1020 | **cyclin-dependent kinase 5 isoform 2** | 32.3 | 2 | 5 | 5 | 9 | 1.63E+08 | **1.82** | 4 | 38.5 | 260 | 29.5 | 6.4 | Cell communication;Signal transduction | Cytoplasm;Nucleus;Golgi apparatus | Protein serine/threonine kinase activity | Nucleus |
| **GPATCH8** | NP_001002909.1 | 23131 | **G patch domain-containing protein 8** | 2.8 | 1 | 2 | 2 | 4 | 1.81E+08 | **1.82** | 1 | - | 1502 | 164.1 | 8.7 | Biological_process unknown | Nucleus | Molecular function unknown | Nucleus |
| **RAD23A** | NP_001257291.1 | 5886 | **UV excision repair protein RAD23 homolog A isoform 2** | 46.7 | 1 | 1 | 4 | 13 | 2.26E+08 | **1.82** | 2 | 13.3 | 362 | 39.5 | 4.6 | DNA repair | Nucleus | DNA binding | Nucleus |
| **UBLCP1** | NP_659486.2 | 134510 | **ubiquitin-like domain-containing CTD phosphatase 1** | 10.1 | 1 | 2 | 2 | 2 | 2.95E+07 | **1.82** | 2 | 36.6 | 318 | 36.8 | 6.5 | RNA metabolism | Nucleus | Hydrolase activity | Nucleus |
| **RBBP4** | NP_001128727.1 | 5928 | **histone-binding protein RBBP4 isoform b** | 66.0 | 2 | 1 | 11 | 39 | 1.72E+09 | **1.82** | 1 | - | 424 | 47.6 | 4.9 | Regulation of nucleobase, nucleoside, nucleotide and nucleic acid metabolism | Nucleus;Nucleolus;Mitochondrion | Transcription regulator activity | Nucleus |
| **SLC16A3** | NP_001193879.1 | 9123 | **monocarboxylate transporter 4** | 10.8 | 1 | 3 | 3 | 4 | 7.00E+07 | **1.82** | 2 | 16 | 465 | 49.4 | 8.0 | Transport | Plasma membrane | Auxiliary transport protein activity | Plasma membrane |
| **TMEM192** | NP_001093859.1 | 201931 | **transmembrane protein 192** | 10.3 | 1 | 2 | 2 | 4 | 1.07E+08 | **1.82** | 2 | 26.6 | 271 | 30.9 | 8.0 | Biological_process unknown | Integral to membrane;Nucleus | Molecular function unknown | Integral to membrane |
| **GAR1** | NP_127460.1 | 54433 | **H/ACA ribonucleoprotein complex subunit 1** | 8.3 | 1 | 1 | 1 | 2 | 4.52E+07 | **1.82** | 1 | - | 217 | 22.3 | 10.9 | Regulation of nucleobase, nucleoside, nucleotide and nucleic acid metabolism | Nucleolus;Cytoplasm | Ribonucleoprotein | Nucleolus |
| **FKBP2** | NP_476433.1 | 2286 | **peptidyl-prolyl cis-trans isomerase FKBP2 precursor** | 38.7 | 1 | 4 | 4 | 10 | 2.11E+08 | **1.81** | 3 | 5.4 | 142 | 15.6 | 9.1 | Cell communication;Signal transduction | Endoplasmic reticulum lumen;Lysosome;Mitochondrial membrane;Membrane fraction | Isomerase activity | Endoplasmic reticulum lumen |
| **NAPA** | NP_003818.2 | 8775 | **alpha-soluble NSF attachment protein** | 50.2 | 1 | 9 | 9 | 21 | 1.52E+08 | **1.81** | 7 | 19.7 | 295 | 33.2 | 5.4 | Transport | Golgi apparatus;Cytoplasm;Lysosome | Receptor signaling complex scaffold activity | Golgi apparatus |
| **LUC7L** | NP_060502.1 | 55692 | **putative RNA-binding protein Luc7-like 1 isoform a** | 33.9 | 2 | 4 | 8 | 19 | 2.83E+08 | **1.81** | 3 | 42.4 | 325 | 38.4 | 10.0 | Biological_process unknown | Nucleus | Molecular function unknown | Nucleus |
| **MESDC2** | NP_055969.1 | 23184 | **LDLR chaperone MESD precursor** | 40.6 | 1 | 7 | 7 | 10 | 2.53E+08 | **1.81** | 4 | 15.7 | 234 | 26.1 | 7.8 | Cell growth and/or maintenance | Endoplasmic reticulum | Chaperone activity | Endoplasmic reticulum |
| **JMJD6** | NP_055982.2 | 23210 | **bifunctional arginine demethylase and lysyl-hydroxylase JMJD6 isoform 2** | 5.2 | 2 | 1 | 1 | 2 | 3.03E+07 | **1.81** | 1 | - | 403 | 46.4 | 8.7 | Cell communication;Signal transduction | Plasma membrane;Nucleus | Molecular function unknown | Plasma membrane |
| **CAPG** | NP_001243068.1 | 822 | **macrophage-capping protein isoform 1** | 54.6 | 2 | 12 | 12 | 45 | 4.97E+08 | **1.81** | 15 | 39.1 | 348 | 38.5 | 6.2 | Cell growth and/or maintenance | Cytoplasm;Nucleus | Cytoskeletal protein binding | Cytoplasm |
| **PLEC** | NP_958782.1 | 5339 | **plectin isoform 1** | 57.3 | 9 | 225 | 232 | 802 | 2.79E+09 | **1.80** | 273 | 25.7 | 4684 | 531.5 | 6.0 | Cytoskeletal anchoring | Cytoplasm;Nucleus;Cytoskeleton;Nucleolus;Plasma membrane;Mitochondrion | Cytoskeletal anchoring activity | Cytoskeleton;Nucleus;Cytoplasm |
| **CNPY3** | NP_006577.2 | 10695 | **protein canopy homolog 3 precursor** | 20.5 | 1 | 5 | 5 | 13 | 1.16E+08 | **1.80** | 6 | 12.1 | 278 | 30.7 | 5.5 | Biological_process unknown | - | Molecular function unknown | - |
| **NUDCD1** | NP_116258.2 | 84955 | **nudC domain-containing protein 1 isoform 1** | 37.7 | 2 | 12 | 12 | 25 | 4.65E+08 | **1.80** | 11 | 33.8 | 583 | 66.7 | 5.1 | Biological_process unknown | - | Molecular function unknown | - |
| **ARHGDIA** | NP_004300.1 | 396 | **rho GDP-dissociation inhibitor 1 isoform a** | 67.2 | 2 | 11 | 11 | 123 | 4.66E+09 | **1.80** | 20 | 29.8 | 204 | 23.2 | 5.1 | Cell growth and/or maintenance | Cytoplasm | Molecular function unknown | Cytoplasm |
| **WARS** | NP_004175.2 | 7453 | **tryptophan--tRNA ligase, cytoplasmic isoform a** | 70.7 | 2 | 25 | 25 | 156 | 2.58E+09 | **1.79** | 41 | 17.2 | 471 | 53.1 | 6.2 | Protein metabolism | Cytoplasm;Mitochondrion | Ligase activity | Cytoplasm |
| **NCDN** | NP_001014841.1 | 23154 | **neurochondrin isoform 2** | 23.0 | 1 | 1 | 10 | 21 | 1.79E+08 | **1.79** | 1 | - | 712 | 77.2 | 5.5 | Biological_process unknown | Cytoplasm | Molecular function unknown | Cytoplasm |
| **HEXIM1** | NP_006451.1 | 10614 | **protein HEXIM1** | 21.5 | 2 | 7 | 7 | 9 | 2.48E+08 | **1.79** | 5 | 59.3 | 359 | 40.6 | 4.9 | Regulation of nucleobase, nucleoside, nucleotide and nucleic acid metabolism | Nucleus;Cytoplasm | Transcription factor activity | Nucleus |

| Gene Symbol | RefSeq_Accession | Entrez Gene ID | Description | Coverage (%) | Proteins | Unique Peptides | # Peptides | PSM | Area | SCC-R /SCC-S | SCC-R /SCC-S (Count) | SCC-R /SCC-S (Variability [%]) | # AAs | MW [kDa] | calc. pI | Biological Process | Localization | Molecular Function | Primary Localization |
|---|---|---|---|---|---|---|---|---|---|---|---|---|---|---|---|---|---|---|---|
| **MSN** | NP_002435.1 | 4478 | **moesin** | 64.5 | 1 | 34 | 44 | 273 | 7.85E+09 | **1.79** | 65 | 18.8 | 577 | 67.8 | 6.4 | Cell growth and/or maintenance | Plasma membrane;Cytoplasm;Nucleus;Cytoskeleton;Mitochondrion | Structural constituent of cytoskeleton | Cytoplasm |
| **PNMA1** | NP_006020.4 | 9240 | **paraneoplastic antigen Ma1** | 11.9 | 1 | 2 | 2 | 2 | 3.45E+07 | **1.79** | 1 | - | 353 | 39.7 | 4.8 | Immune response | Cytoplasm;Nucleolus | Molecular function unknown | Nucleolus |
| **CYTH2** | NP_004219.3 | 9266 | **cytohesin-2 isoform 2** | 3.5 | 3 | 1 | 1 | 1 | 5.96E+07 | **1.78** | 1 | - | 399 | 46.5 | 5.5 | Signal transduction;Regulation of gene expression, epigenetic | Plasma membrane;Cytoplasm;Golgi apparatus;Cytosol;Nucleus;Lysosome | Guanyl-nucleotide exchange factor activity | Cytosol |
| **ARF6** | NP_001654.1 | 382 | **ADP-ribosylation factor 6** | 60.0 | 6 | 6 | 7 | 23 | 1.20E+09 | **1.78** | 8 | 6.8 | 175 | 20.1 | 9.0 | Transport | Cytosol;Endosome;Plasma membrane;Golgi apparatus;Cell projection | Transporter activity | Cytosol |
| **ADPGK** | NP_112574.3 | 83440 | **ADP-dependent glucokinase precursor** | 16.1 | 1 | 5 | 5 | 13 | 8.67E+07 | **1.78** | 4 | 24.4 | 496 | 53.9 | 6.2 | Metabolism;Energy pathways | - | Phosphorylase activity | - |
| **ATF1** | NP_005162.1 | 466 | **cyclic AMP-dependent transcription factor ATF-1** | 15.5 | 8 | 1 | 3 | 6 | 8.35E+07 | **1.78** | 1 | - | 271 | 29.2 | 8.4 | Regulation of nucleobase, nucleoside, nucleotide and nucleic acid metabolism | Nucleus | Transcription factor activity | Nucleus |
| **NDC80** | NP_006092.1 | 10403 | **kinetochore protein NDC80 homolog** | 7.5 | 1 | 3 | 3 | 5 | 6.27E+07 | **1.78** | 3 | 29.1 | 642 | 73.9 | 5.6 | Cell communication;Signal transduction;Chromosome segregation | Kinetochore;Nucleus;Microtubule | Chromatin binding | Nucleus |
| **ABHD10** | NP_060864.1 | 55347 | **mycophenolic acid acyl-glucuronide esterase, mitochondrial isoform 1 precursor** | 37.9 | 2 | 7 | 7 | 19 | 2.38E+08 | **1.78** | 6 | 28.9 | 306 | 33.9 | 8.6 | Metabolism | - | Hydrolase activity | - |
| **FLYWCH2** | NP_612448.1 | 114984 | **FLYWCH family member 2** | 10.0 | 3 | 1 | 1 | 2 | 7.13E+07 | **1.78** | 1 | - | 140 | 14.6 | 8.5 | Biological_process unknown | - | Molecular function unknown | - |
| **IRGQ** | NP_001007562.1 | 126298 | **immunity-related GTPase family Q protein** | 14.8 | 1 | 5 | 5 | 11 | 5.34E+07 | **1.78** | 3 | 2.7 | 623 | 62.7 | 4.9 | Biological_process unknown | - | Molecular function unknown | - |
| **LSM3** | NP_055278.1 | 27258 | **U6 snRNA-associated Sm-like protein LSm3** | 42.2 | 1 | 3 | 3 | 7 | 9.21E+08 | **1.78** | 3 | 42.5 | 102 | 11.8 | 4.7 | Regulation of nucleobase, nucleoside, nucleotide and nucleic acid metabolism | Cytoplasm;Nucleus | RNA binding | Cytoplasm |
| **HNRNPH1** | NP_001244222.1 | 3187 | **heterogeneous nuclear ribonucleoprotein H** | 48.8 | 2 | 9 | 18 | 116 | 2.28E+09 | **1.78** | 9 | 6 | 449 | 49.2 | 6.3 | Regulation of nucleobase, nucleoside, nucleotide and nucleic acid metabolism | Nucleus;Nucleolus;Cytoplasm | Ribonucleoprotein | Nucleus |
| **EIF1AD** | NP_001229414.1 | 84285 | **probable RNA-binding protein EIF1AD** | 24.9 | 1 | 3 | 3 | 6 | 1.63E+08 | **1.77** | 3 | 8.8 | 165 | 19 | 5.2 | Biological_process unknown | - | Molecular function unknown | - |
| **UBE2S** | NP_055316.2 | 27338 | **ubiquitin-conjugating enzyme E2 S** | 3.2 | 1 | 1 | 1 | 1 | 6.66E+07 | **1.77** | 1 | - | 222 | 23.8 | 8.4 | Protein metabolism | - | Ubiquitin-specific protease activity | - |
| **TMEM181** | NP_065874.1 | 57583 | **transmembrane protein 181** | 2.6 | 1 | 1 | 1 | 1 | 2.96E+07 | **1.77** | 1 | - | 612 | 69.3 | 8.9 | Biological_process unknown | - | Molecular function unknown | - |
| **SYNGR2** | NP_004701.1 | 9144 | **synaptogyrin-2** | 4.5 | 1 | 1 | 1 | 1 | 4.24E+05 | **1.77** | 1 | - | 224 | 24.8 | 4.9 | Transport | Plasma membrane | Molecular function unknown | Plasma membrane |
| **GARS** | NP_002038.2 | 2617 | **glycine--tRNA ligase precursor** | 58.6 | 1 | 33 | 33 | 217 | 1.63E+09 | **1.77** | 53 | 18.4 | 739 | 83.1 | 7.0 | Protein metabolism | Cytoplasm;Nucleus;Mitochondrion | Ligase activity | Cytoplasm |
| **HAUS1** | NP_612452.1 | 115106 | **HAUS augmin-like complex subunit 1** | 5.4 | 1 | 1 | 1 | 1 | 2.26E+07 | **1.77** | 1 | - | 278 | 31.8 | 5.5 | Cell growth and/or maintenance | Cytoskeleton;Centrosome;Plasma membrane;Microtubule | Molecular function unknown | Cytoskeleton |
| **THUMPD3** | NP_056268.2 | 25917 | **THUMP domain-containing protein 3** | 15.0 | 1 | 3 | 3 | 4 | 8.70E+07 | **1.77** | 2 | 155.2 | 507 | 57 | 6.4 | Biological_process unknown | - | Molecular function unknown | - |
| **CHORDC1** | NP_036256.2 | 26973 | **cysteine and histidine-rich domain-containing protein 1 isoform a** | 43.4 | 2 | 13 | 13 | 66 | 2.22E+09 | **1.77** | 27 | 22.2 | 332 | 37.5 | 7.9 | Biological_process unknown | - | Molecular function unknown | - |
| **LRPAP1** | NP_002328.1 | 4043 | **alpha-2-macroglobulin receptor-associated protein precursor** | 13.2 | 1 | 4 | 4 | 8 | 2.28E+08 | **1.77** | 5 | 19.7 | 357 | 41.4 | 8.8 | Protein metabolism | Endoplasmic reticulum;Plasma membrane;Cytoplasm;Extracellular;Golgi apparatus;Endosome;Nucleus | Chaperone activity | Endoplasmic reticulum |
| **PHYHIP** | NP_055574.3 | 9796 | **phytanoyl-CoA hydroxylase-interacting protein** | 3.0 | 1 | 1 | 1 | 1 | 1.76E+06 | **1.76** | 1 | - | 330 | 37.5 | 7.0 | Biological_process unknown | Cytoplasm | Molecular function unknown | Cytoplasm |
| **NUP210** | NP_079199.2 | 23225 | **nuclear pore membrane glycoprotein 210 precursor** | 11.0 | 1 | 12 | 12 | 26 | 1.87E+08 | **1.76** | 10 | 21.8 | 1887 | 205 | 6.8 | Transport | Nucleus;Mitochondrion;Nuclear membrane | Transporter activity | Nucleus |
| **XPOT** | NP_009166.2 | 11260 | **exportin-T** | 22.3 | 1 | 13 | 13 | 25 | 3.88E+08 | **1.76** | 14 | 34.2 | 962 | 109.9 | 5.4 | Transport | Nucleus;Cytoplasm | Transporter activity | Nucleus |
| **MOGS** | NP_001139630.1 | 7841 | **mannosyl-oligosaccharide glucosidase isoform 2** | 20.8 | 2 | 8 | 8 | 16 | 9.80E+07 | **1.76** | 8 | 43.7 | 731 | 80.7 | 7.1 | Metabolism;Energy pathways | Endoplasmic reticulum;Cytoplasm;Mitochondrion | Glucosidase activity | Endoplasmic reticulum |
| **WIPF2** | NP_573571.1 | 147179 | **WAS/WASL-interacting protein family member 2** | 3.0 | 1 | 1 | 1 | 1 | 8.44E+06 | **1.76** | 1 | - | 440 | 46.3 | 10.9 | Cell growth and/or maintenance | Cytoskeleton | Cytoskeletal protein binding | Cytoskeleton |
| **MED29** | NP_060062.1 | 55588 | **mediator of RNA polymerase II transcription subunit 29** | 14.0 | 1 | 2 | 2 | 4 | 4.25E+07 | **1.76** | 1 | - | 221 | 23.5 | 8.2 | Regulation of nucleobase, nucleoside, nucleotide and nucleic acid metabolism | Nucleus;Cytoplasm | Transcription regulator activity | Nucleus |
| **C6orf89** | NP_001273566.1 | 221477 | **bombesin receptor-activated protein C6orf89 isoform c** | 5.4 | 3 | 1 | 1 | 1 | 1.44E+07 | **1.75** | 1 | - | 241 | 27.8 | 6.6 | Biological_process unknown | - | Molecular function unknown | - |
| **UROD** | NP_000365.3 | 7389 | **uroporphyrinogen decarboxylase** | 34.3 | 1 | 8 | 8 | 28 | 7.56E+08 | **1.75** | 9 | 23.1 | 367 | 40.8 | 6.1 | Metabolism;Energy pathways | Cytoplasm | Carboxy-lyase activity | Cytoplasm |
| **ARGLU1** | NP_060481.3 | 55082 | **arginine and glutamate-rich protein 1** | 11.7 | 1 | 3 | 3 | 3 | 9.34E+07 | **1.75** | 3 | 13.4 | 273 | 33.2 | 10.4 | Biological_process unknown | - | Molecular function unknown | - |
| **CFL1** | NP_005498.1 | 1072 | **cofilin-1** | 77.7 | 1 | 16 | 22 | 802 | 1.59E+10 | **1.75** | 73 | 12.6 | 166 | 18.5 | 8.1 | Cell growth and/or maintenance | Cytoplasm;Nucleus;Plasma membrane;Extracellular;Microsome;Cytosol;Mitochondrion | Cytoskeletal protein binding | Cytoplasm |
| **TPM3** | NP_705935.1 | 7170 | **tropomyosin alpha-3 chain isoform 2** | 87.5 | 7 | 3 | 35 | 433 | 8.25E+09 | **1.75** | 3 | 20.6 | 248 | 29 | 4.8 | Cell growth and/or maintenance | Cytoplasm;Extracellular;Cytosol | Cytoskeletal protein binding | Cytoplasm |
| **CTDP1** | NP_001189433.1 | 9150 | **RNA polymerase II subunit A C-terminal domain phosphatase isoform 3** | 1.1 | 3 | 1 | 1 | 1 | 2.92E+07 | **1.75** | 1 | - | 842 | 92.3 | 5.1 | Transcription | Nucleus;Cytoplasm | Transcription factor binding | Nucleus;Cytoplasm |
| **NFKB2** | NP_001248332.1 | 4791 | **nuclear factor NF-kappa-B p100 subunit isoform b** | 20.4 | 2 | 9 | 9 | 19 | 1.26E+08 | **1.75** | 7 | 19.7 | 899 | 96.6 | 6.3 | Regulation of nucleobase, nucleoside, nucleotide and nucleic acid metabolism | Cytoplasm;Nucleus | Transcription factor activity | Cytoplasm |
| **POP1** | NP_001139333.1 | 10940 | **ribonucleases P/MRP protein subunit POP1** | 6.6 | 1 | 4 | 4 | 5 | 8.21E+07 | **1.75** | 1 | - | 1024 | 114.6 | 9.2 | Regulation of nucleobase, nucleoside, nucleotide and nucleic acid metabolism | Nucleus;Nucleolus;Ribosome;Cytoplasm | Ribonuclease activity | Nucleolus |
| **TRAF4** | NP_004286.2 | 9618 | **TNF receptor-associated factor 4** | 1.5 | 1 | 1 | 1 | 1 | 1.20E+08 | **1.74** | 1 | - | 470 | 53.5 | 8.2 | Cell communication;Signal transduction;Apoptosis;Regulation of cell proliferation | Cytoplasm;Nucleus | Receptor signaling complex scaffold activity | Cytoplasm |
| **AGRN** | NP_940978.2 | 375790 | **agrin precursor** | 7.1 | 1 | 7 | 7 | 10 | 3.97E+07 | **1.74** | 4 | 26.1 | 2045 | 214.7 | 6.4 | Cell growth and/or maintenance | Extracellular | Extracellular matrix structural constituent | Extracellular |

| Gene Symbol | RefSeq_Accession | Entrez Gene ID | Description | Coverage (%) | Proteins | Unique Peptides | # Peptides | PSM | Area | SCC-R /SCC-S | SCC-R /SCC-S (Count) | SCC-R /SCC-S (Variability [%]) | # AAs | MW [kDa] | calc. pI | Biological Process | Localization | Molecular Function | Primary Localization |
|---|---|---|---|---|---|---|---|---|---|---|---|---|---|---|---|---|---|---|---|
| C1orf50 | NP_077002.2 | 79078 | uncharacterized protein C1orf50 | 19.1 | 1 | 2 | 2 | 4 | 3.19E+07 | 1.74 | 2 | 15.8 | 199 | 21.9 | 5.9 | Biological_process unknown | - | Molecular function unknown | - |
| THOC6 | NP_077315.2 | 79228 | THO complex subunit 6 homolog isoform 1 | 19.1 | 2 | 4 | 4 | 5 | 7.27E+07 | 1.74 | 2 | 18.4 | 341 | 37.5 | 7.4 | Biological_process unknown | - | Molecular function unknown | - |
| CNOT11 | NP_060016.3 | 55571 | CCR4-NOT transcription complex subunit 11 | 11.0 | 1 | 3 | 3 | 5 | 5.38E+07 | 1.73 | 3 | 8.3 | 510 | 55.2 | 6.4 | Biological_process unknown | - | Molecular function unknown | - |
| FAM76B | NP_653265.3 | 143684 | protein FAM76B | 5.0 | 1 | 1 | 1 | 1 | 3.91E+06 | 1.73 | 1 | - | 339 | 38.7 | 9.3 | Biological_process unknown | - | Molecular function unknown | - |
| ANP32A | NP_006296.1 | 8125 | acidic leucine-rich nuclear phosphoprotein 32 family member A | 51.0 | 3 | 11 | 13 | 87 | 2.28E+09 | 1.73 | 18 | 10.8 | 249 | 28.6 | 4.1 | Immune response | Cytoplasm;Nucleus;Plasma membrane | MHC class I receptor activity;MHC class II receptor activity | Nucleus |
| CSTB | NP_000091.1 | 1476 | cystatin-B | 76.5 | 1 | 5 | 5 | 36 | 2.30E+09 | 1.73 | 11 | 17.6 | 98 | 11.1 | 7.6 | Protein metabolism | Nucleus;Cytoplasm | Protease inhibitor activity | Nucleus |
| SAE1 | NP_005491.1 | 10055 | SUMO-activating enzyme subunit 1 isoform a | 54.6 | 3 | 13 | 13 | 45 | 5.09E+08 | 1.73 | 14 | 4.6 | 346 | 38.4 | 5.3 | Metabolism;Energy pathways | Nucleus | Ligase activity | Nucleus |
| IFT22 | NP_001124292.1 | 64792 | rab-like protein 5 isoform b | 14.8 | 2 | 1 | 1 | 2 | 3.14E+07 | 1.73 | 1 | - | 155 | 17.5 | 5.5 | Biological_process unknown | - | GTPase activity | - |
| FADD | NP_003815.1 | 8772 | FAS-associated death domain protein | 45.7 | 1 | 4 | 4 | 16 | 1.61E+08 | 1.73 | 7 | 25.2 | 208 | 23.3 | 5.7 | Cell communication;Signal transduction | Cytoplasm;Plasma membrane;Early endosome | Receptor signaling complex scaffold activity | Cytoplasm |
| PAK2 | NP_002568.2 | 5062 | serine/threonine-protein kinase PAK 2 | 59.5 | 1 | 17 | 22 | 76 | 1.88E+09 | 1.73 | 28 | 28.2 | 524 | 58 | 6.0 | Cell communication;Signal transduction | Endoplasmic reticulum;Cytosol;Nucleus;Cytoplasm | Protein serine/threonine kinase activity | Endoplasmic reticulum |
| PDPK1 | NP_002604.1 | 5170 | 3-phosphoinositide-dependent protein kinase 1 isoform 1 | 11.3 | 7 | 4 | 4 | 5 | 7.40E+07 | 1.73 | 3 | 3.7 | 556 | 63.1 | 7.4 | Signal transduction | Plasma membrane;Cytoplasm;Nucleus | Protein serine/threonine kinase activity | Cytoplasm;Nucleus |
| TMEM127 | NP_001180233.1 | 55654 | transmembrane protein 127 | 7.6 | 1 | 1 | 1 | 1 | 2.58E+07 | 1.73 | 1 | - | 238 | 25.8 | 6.7 | Biological_process unknown | - | Molecular function unknown | - |
| NGFRAP1 | NP_996800.1 | 27018 | protein BEX3 isoform a | 25.7 | 1 | 1 | 1 | 1 | 2.55E+07 | 1.72 | 1 | - | 101 | 11.8 | 6.1 | Cell communication;Signal transduction;Apoptosis | Cytoplasm;Nucleus | Receptor signaling complex scaffold activity | Cytoplasm |
| CCDC127 | NP_660308.1 | 133957 | coiled-coil domain-containing protein 127 | 13.5 | 1 | 1 | 1 | 2 | 7.76E+07 | 1.72 | 1 | - | 260 | 30.8 | 9.2 | Biological_process unknown | - | Molecular function unknown | - |
| SNX27 | NP_112180.4 | 81609 | sorting nexin-27 | 11.6 | 1 | 3 | 3 | 4 | 7.84E+07 | 1.72 | 2 | 11.4 | 528 | 59.8 | 6.3 | Vesicle-mediated transport | Cytosol;Endosome;Microsome | Transporter activity | Cytosol |
| TLDC1 | NP_065998.3 | 57707 | TLD domain-containing protein 1 | 19.7 | 1 | 5 | 5 | 23 | 2.85E+08 | 1.72 | 3 | 3.4 | 456 | 51 | 6.2 | Biological_process unknown | - | Molecular function unknown;Protein binding | - |
| PDCD6 | NP_001254485.1 | 10016 | programmed cell death protein 6 isoform 2 | 58.7 | 5 | 6 | 6 | 19 | 2.37E+08 | 1.72 | 10 | 34.3 | 189 | 21.7 | 5.4 | Cell communication;Signal transduction | Cytoplasm;Nucleus;Nucleolus;Nucleoplasm;Perinuclear region | Calcium ion binding | Cytoplasm |
| RTN4IP1 | NP_116119.2 | 84816 | reticulon-4-interacting protein 1, mitochondrial precursor | 5.8 | 1 | 1 | 1 | 1 | 2.28E+06 | 1.72 | 1 | - | 396 | 43.6 | 9.1 | Metabolism;Energy pathways | Mitochondrion | Catalytic activity | Mitochondrion |
| VPS51 | NP_037397.2 | 738 | vacuolar protein sorting-associated protein 51 homolog | 11.1 | 1 | 6 | 6 | 9 | 8.71E+07 | 1.72 | 3 | 11.5 | 782 | 86 | 6.5 | Biological_process unknown | - | Molecular function unknown | - |
| RSU1 | NP_036557.1 | 6251 | ras suppressor protein 1 isoform 1 | 32.1 | 2 | 7 | 7 | 16 | 2.29E+08 | 1.72 | 6 | 18.7 | 277 | 31.5 | 8.7 | Cell adhesion;Signal transduction | - | Molecular function unknown | - |
| STXBP1 | NP_001027392.1 | 6812 | syntaxin-binding protein 1 isoform b | 20.9 | 2 | 7 | 7 | 7 | 7.55E+07 | 1.72 | 6 | 24.8 | 594 | 67.5 | 7.0 | Neurotransmitter transport | Cytoplasm;Plasma membrane;Nucleolus;Cytosol | Transporter activity | Plasma membrane;Cytosol |
| PIN1 | NP_006212.1 | 5300 | peptidyl-prolyl cis-trans isomerase NIMA-interacting 1 | 66.3 | 1 | 7 | 7 | 35 | 3.84E+08 | 1.72 | 7 | 14.9 | 163 | 18.2 | 8.8 | Metabolism;Energy pathways;Regulation of cell cycle | Nucleus;Cytoplasm | Isomerase activity | Nucleus |
| OGFR | NP_031372.2 | 11054 | opioid growth factor receptor | 19.2 | 1 | 7 | 7 | 15 | 1.57E+08 | 1.71 | 6 | 33.2 | 677 | 73.3 | 4.8 | Cell communication;Signal transduction | Nucleus;Cytoplasm | Ligand-dependent nuclear receptor activity | Nucleus;Cytoplasm |
| ZNF185 | NP_001171579.1 | 7739 | zinc finger protein 185 isoform 3 | 17.1 | 8 | 7 | 7 | 13 | 8.94E+07 | 1.71 | 2 | 16.2 | 690 | 73.6 | 7.0 | Regulation of nucleobase, nucleoside, nucleotide and nucleic acid metabolism | Nucleus | Transcription regulator activity | Nucleus |
| GALE | NP_000394.2 | 2582 | UDP-glucose 4-epimerase | 29.0 | 1 | 5 | 5 | 8 | 5.74E+07 | 1.71 | 1 | - | 348 | 38.3 | 6.7 | Metabolism | - | Racemase and epimerase activity | - |
| LSM14A | NP_056393.2 | 26065 | protein LSM14 homolog A isoform b | 15.3 | 2 | 4 | 4 | 5 | 7.70E+07 | 1.71 | 4 | 48.3 | 463 | 50.5 | 9.5 | Biological_process unknown | - | Molecular function unknown | - |
| SEPT10 | NP_653311.1 | 151011 | septin-10 isoform 1 | 42.7 | 2 | 12 | 13 | 19 | 3.53E+08 | 1.71 | 10 | 16.6 | 454 | 52.6 | 6.8 | Cell communication;Signal transduction | Cytoplasm;Nucleus | GTPase activity | Cytoplasm |
| ZC3H14 | NP_001153575.1 | 79882 | zinc finger CCCH domain-containing protein 14 isoform 5 | 35.0 | 4 | 1 | 15 | 28 | 1.20E+08 | 1.71 | 1 | - | 735 | 82.8 | 7.3 | Biological_process unknown | Nucleus | RNA binding | Nucleus |
| SORBS3 | NP_001018003.1 | 10174 | vinexin isoform 2 | 13.4 | 2 | 2 | 2 | 3 | 1.35E+08 | 1.71 | 2 | 14.7 | 329 | 36.7 | 8.7 | Cell growth and/or maintenance;Cytoskeleton organization and biogenesis | Focal adhesion;Cell projection;Cytoplasm;Nucleus;Plasma membrane | Cell adhesion molecule activity | Cytoplasm |
| TLN1 | NP_006280.3 | 7094 | talin-1 | 64.2 | 2 | 98 | 105 | 538 | 1.42E+09 | 1.71 | 152 | 24.8 | 2541 | 269.6 | 6.1 | Cell growth and/or maintenance | Extracellular;Plasma membrane;Cytoplasm;Nucleus | Cytoskeletal protein binding | Extracellular |
| AHNAK | NP_001611.1 | 79026 | neuroblast differentiation-associated protein AHNAK isoform 1 | 61.0 | 2 | 186 | 186 | 548 | 4.42E+09 | 1.71 | 210 | 20.9 | 5890 | 628.7 | 6.2 | Muscle contraction | Plasma membrane;Nucleus;Cytoskeleton | Protein binding | Nucleus |
| PYGB | NP_002853.2 | 5834 | glycogen phosphorylase, brain form | 40.8 | 3 | 20 | 25 | 70 | 1.99E+09 | 1.71 | 24 | 23.5 | 843 | 96.6 | 6.9 | Metabolism;Energy pathways | Cytoplasm;Nucleus | Phosphorylase activity | Cytoplasm |
| LMNA | NP_001269554.1 | 4000 | lamin isoform C | 78.5 | 2 | 2 | 63 | 416 | 7.82E+09 | 1.70 | 3 | 1.1 | 572 | 65.1 | 6.8 | Cell growth and/or maintenance | Nucleus;Cytoplasm;Nucleolus | Structural molecule activity | Nucleus;Cytoplasm |
| SMG1 | NP_055907.3 | 23049 | serine/threonine-protein kinase SMG1 | 0.6 | 1 | 1 | 1 | 1 | 9.07E+06 | 1.70 | 1 | - | 3661 | 410.2 | 6.5 | Regulation of nucleobase, nucleoside, nucleotide and nucleic acid metabolism | Nucleus;Cytoplasm | Molecular function unknown | Nucleus |
| VBP1 | NP_003363.1 | 7411 | prefoldin subunit 3 | 39.1 | 1 | 7 | 7 | 21 | 9.46E+07 | 1.70 | 12 | 23.6 | 197 | 22.6 | 7.1 | Protein metabolism | Cytoplasm;Nucleus | Chaperone activity | Cytoplasm |
| CHMP2B | NP_001231573.1 | 25978 | charged multivesicular body protein 2b isoform 2 | 5.8 | 2 | 1 | 1 | 1 | 2.87E+07 | 1.70 | 1 | - | 172 | 19.1 | 6.2 | Transport | - | Transporter activity | - |
| HINT2 | NP_115982.1 | 84681 | histidine triad nucleotide-binding protein 2, mitochondrial precursor | 57.7 | 1 | 5 | 5 | 20 | 4.24E+08 | 1.70 | 5 | 3.9 | 163 | 17.2 | 9.2 | Metabolism;Energy pathways | Mitochondrion | Hydrolase activity | Mitochondrion |

Jain *et al.*, 2019. MAP2K1 is a potential therapeutic target in erlotinib resistant head and neck squamous cell carcinoma
Supplementary Table S4. List of all proteins identified in SCC-S and SCC-R cells using SequestHT and Mascot search algorithms and their gene ontology annotation

| Gene Symbol | RefSeq_Accession | Entrez Gene ID | Description | Coverage (%) | Proteins | Unique Peptides | # Peptides | PSM | Area | SCC-R /SCC-S | SCC-R /SCC-S (Count) | SCC-R /SCC-S (Variability [%]) | # AAs | MW [kDa] | calc. pI | Biological Process | Localization | Molecular Function | Primary Localization |
|---|---|---|---|---|---|---|---|---|---|---|---|---|---|---|---|---|---|---|---|
| **CCM2** | NP_001161407.1 | 83605 | **malcavernin isoform 4** | 4.8 | 4 | 1 | 1 | 2 | 2.70E+07 | **1.70** | 1 | - | 353 | 39.4 | 6.0 | Biological_process unknown;Cell communication;Signal transduction | Cytoplasm | Molecular function unknown;Receptor signaling complex scaffold activity | Cytoplasm |
| **STAM** | NP_003464.1 | 8027 | **signal transducing adapter molecule 1** | 9.3 | 1 | 3 | 4 | 5 | 8.25E+07 | **1.70** | 2 | 39.1 | 540 | 59.1 | 4.8 | Cell communication;Signal transduction | Cytoplasm;Endosome;Mitochondrion;Nucleus | Receptor signaling complex scaffold activity | Cytoplasm |
| **UBFD1** | NP_061989.2 | 56061 | **ubiquitin domain-containing protein UBFD1** | 30.4 | 1 | 6 | 6 | 11 | 9.73E+07 | **1.70** | 3 | 8.5 | 309 | 33.4 | 5.8 | Protein metabolism | - | Molecular function unknown | - |
| **AMOTL2** | NP_001265614.1 | 51421 | **angiomotin-like protein 2 isoform 3** | 1.8 | 3 | 1 | 1 | 1 | 6.54E+07 | **1.70** | 1 | - | 777 | 85.4 | 7.2 | Biological_process unknown | - | Molecular function unknown | - |
| **CLTA** | NP_001824.1 | 1211 | **clathrin light chain A isoform a** | 32.1 | 6 | 8 | 8 | 27 | 1.28E+09 | **1.70** | 7 | 8.9 | 218 | 23.6 | 4.5 | Cell growth and/or maintenance | Cytoplasm;Plasma membrane;Golgi apparatus;Cell surface;Perinuclear region | Structural molecule activity | Cytoplasm |
| **UGDH** | NP_003350.1 | 7358 | **UDP-glucose 6-dehydrogenase isoform 1** | 47.2 | 3 | 13 | 13 | 36 | 4.61E+08 | **1.70** | 16 | 12.7 | 494 | 55 | 7.1 | Metabolism;Energy pathways | - | Catalytic activity | - |
| **RBM12B** | NP_976324.2 | 389677 | **RNA-binding protein 12B** | 1.4 | 1 | 1 | 1 | 2 | 6.35E+06 | **1.70** | 1 | - | 1001 | 118 | 6.8 | Regulation of nucleobase, nucleoside, nucleotide and nucleic acid metabolism | Nucleus;Cytoplasm | Molecular function unknown | Nucleus;Cytoplasm |
| **UQCC2** | NP_115716.1 | 84300 | **ubiquinol-cytochrome-c reductase complex assembly factor 2** | 42.9 | 1 | 3 | 3 | 4 | 5.99E+07 | **1.70** | 1 | - | 126 | 14.9 | 7.4 | Biological_process unknown | - | Molecular function unknown | - |
| **PCYT2** | NP_001243362.1 | 5833 | **ethanolamine-phosphate cytidylyltransferase isoform 5** | 11.3 | 6 | 3 | 3 | 3 | 2.53E+07 | **1.69** | 1 | - | 335 | 37.6 | 7.2 | Metabolism;Energy pathways | Cytoplasm;Endoplasmic reticulum | Nucleotidyltransferase activity | Cytoplasm |
| **APMAP** | NP_065392.1 | 57136 | **adipocyte plasma membrane-associated protein** | 30.8 | 1 | 8 | 8 | 32 | 7.55E+08 | **1.69** | 10 | 16.8 | 416 | 46.5 | 6.2 | Biological_process unknown | - | Molecular function unknown | - |
| **IMPAD1** | NP_060283.3 | 54928 | **inositol monophosphatase 3** | 12.0 | 1 | 2 | 2 | 3 | 2.42E+07 | **1.69** | 2 | 0.5 | 359 | 38.7 | 6.9 | Metabolism;Energy pathways | - | Catalytic activity | - |
| **IPO8** | NP_001177924.1 | 10526 | **importin-8 isoform 2** | 10.2 | 2 | 4 | 5 | 10 | 4.52E+07 | **1.69** | 4 | 40.1 | 832 | 96 | 5.0 | Transport | Nucleus | Transporter activity | Nucleus |
| **RGMB** | NP_001012779.2 | 285704 | **RGM domain family member B** | 1.3 | 1 | 1 | 1 | 1 | 1.35E+07 | **1.69** | 1 | - | 478 | 51.9 | 7.2 | Biological_process unknown | - | Molecular function unknown | - |
| **TBC1D8B** | NP_942582.1 | 54885 | **TBC1 domain family member 8B isoform b** | 1.1 | 2 | 1 | 1 | 5 | 3.69E+08 | **1.69** | 2 | 17.1 | 632 | 72.6 | 6.3 | Biological_process unknown | - | Molecular function unknown | - |
| **DRAP1** | NP_006433.2 | 10589 | **dr1-associated corepressor** | 11.2 | 1 | 3 | 3 | 7 | 1.16E+08 | **1.68** | 3 | 5.6 | 205 | 22.3 | 5.2 | Regulation of nucleobase, nucleoside, nucleotide and nucleic acid metabolism | Nucleus | Transcription regulator activity | Nucleus |
| **PIKFYVE** | NP_055855.2 | 200576 | **1-phosphatidylinositol 3-phosphate 5-kinase isoform 2** | 1.7 | 1 | 1 | 1 | 1 | 2.50E+07 | **1.68** | 1 | - | 2098 | 237 | 6.7 | Lipid metabolism;Transport | Cytoplasm;Plasma membrane;Nucleus | Lipid kinase activity | Cytoplasm |
| **HDGF** | NP_004485.1 | 3068 | **hepatoma-derived growth factor isoform a** | 69.6 | 3 | 14 | 14 | 99 | 5.60E+09 | **1.68** | 21 | 20.6 | 240 | 26.8 | 4.7 | Cell communication;Signal transduction | Nucleus;Extracellular;Cytoplasm | Growth factor activity | Nucleus |
| **RNF31** | NP_060469.4 | 55072 | **E3 ubiquitin-protein ligase RNF31** | 6.5 | 1 | 3 | 3 | 3 | 2.59E+07 | **1.68** | 3 | 6.3 | 1072 | 119.6 | 6.6 | Biological_process unknown | - | Molecular function unknown | - |
| **YTHDF2** | NP_057342.2 | 51441 | **YTH domain-containing family protein 2 isoform 1** | 18.1 | 2 | 6 | 8 | 17 | 3.52E+08 | **1.67** | 4 | 1.9 | 579 | 62.3 | 8.8 | Biological_process unknown | - | Molecular function unknown | - |
| **TAPBP** | NP_003181.3 | 6892 | **tapasin isoform 1 precursor** | 15.0 | 3 | 5 | 5 | 20 | 2.24E+08 | **1.67** | 6 | 10.5 | 448 | 47.5 | 7.0 | Protein folding | Endoplasmic reticulum;Golgi apparatus | Chaperone activity | Endoplasmic reticulum |
| **UBE2M** | NP_003960.1 | 9040 | **NEDD8-conjugating enzyme Ubc12** | 17.5 | 1 | 3 | 3 | 7 | 5.24E+07 | **1.67** | 1 | - | 183 | 20.9 | 7.7 | Protein metabolism | - | Ubiquitin-specific protease activity | - |
| **CYB5R3** | NP_015565.1 | 1727 | **NADH-cytochrome b5 reductase 3 isoform 2** | 74.8 | 3 | 12 | 12 | 39 | 7.49E+08 | **1.67** | 13 | 39.2 | 278 | 31.6 | 7.6 | Metabolism;Energy pathways | Endoplasmic reticulum;Mitochondrion;Plasma membrane;Cytoplasm | Catalytic activity | Cytoplasm |
| **M6PR** | NP_002346.1 | 4074 | **cation-dependent mannose-6-phosphate receptor isoform 1 precursor** | 19.9 | 2 | 4 | 4 | 19 | 4.32E+08 | **1.67** | 6 | 30.9 | 277 | 31 | 5.8 | Protein metabolism | Plasma membrane;Golgi apparatus;Endosome;Cytoplasmic vesicle | Receptor activity | Plasma membrane |
| **RBFOX2** | NP_001076048.1 | 23543 | **RNA binding protein fox-1 homolog 2 isoform 6** | 22.2 | 11 | 4 | 4 | 7 | 1.39E+08 | **1.67** | 3 | 7.1 | 450 | 47.2 | 6.6 | Regulation of nucleobase, nucleoside, nucleotide and nucleic acid metabolism | Nucleus | Transcription regulator activity | Nucleus |
| **RBPJ** | NP_976028.1 | 3516 | **recombining binding protein suppressor of hairless isoform 3** | 2.3 | 4 | 1 | 1 | 2 | 4.13E+07 | **1.67** | 1 | - | 485 | 54.1 | 8.0 | Regulation of nucleobase, nucleoside, nucleotide and nucleic acid metabolism | Nucleus;Nucleolus | Transcription factor activity;Transcription regulator activity | Nucleolus |
| **PNPT1** | NP_149100.2 | 87178 | **polyribonucleotide nucleotidyltransferase 1, mitochondrial precursor** | 30.9 | 1 | 18 | 18 | 39 | 3.40E+08 | **1.67** | 14 | 12 | 783 | 85.9 | 7.8 | Regulation of nucleobase, nucleoside, nucleotide and nucleic acid metabolism | Cytoplasm;Mitochondrion;Nucleus | Exonuclease activity | Mitochondrion |
| **PPM1A** | NP_808820.1 | 5494 | **protein phosphatase 1A isoform 2** | 20.1 | 3 | 3 | 4 | 5 | 4.91E+07 | **1.67** | 1 | - | 324 | 35.9 | 5.8 | Signal transduction | Cytoplasm;Nucleus | Protein serine/threonine phosphatase activity | Cytoplasm;Nucleus |
| **TMEM245** | NP_114401.2 | 23731 | **transmembrane protein 245** | 3.0 | 1 | 2 | 2 | 2 | 3.73E+07 | **1.67** | 1 | - | 879 | 97.3 | 8.9 | Biological_process unknown | - | Molecular function unknown | - |
| **LAMTOR4** | NP_001008396.1 | 389541 | **ragulator complex protein LAMTOR4** | 7.1 | 1 | 1 | 1 | 1 | 5.16E+07 | **1.67** | 1 | - | 99 | 10.7 | 6.5 | Biological_process unknown | - | Molecular function unknown | - |
| **YARS** | NP_003671.1 | 8565 | **tyrosine--tRNA ligase, cytoplasmic** | 65.5 | 1 | 30 | 30 | 120 | 2.21E+09 | **1.67** | 46 | 17.1 | 528 | 59.1 | 7.1 | Metabolism;Energy pathways | Extracellular;Cytoplasm | Ligase activity | Cytoplasm |
| **ATP6V0D1** | NP_004682.2 | 9114 | **V-type proton ATPase subunit d 1** | 22.2 | 1 | 5 | 5 | 6 | 8.30E+07 | **1.67** | 4 | 20.5 | 351 | 40.3 | 5.0 | Transport | - | ATPase activity | - |
| **AURKA** | NP_940838.1 | 6790 | **aurora kinase A** | 5.0 | 1 | 1 | 1 | 1 | 4.07E+07 | **1.66** | 1 | - | 403 | 45.8 | 9.4 | Cell communication;Signal transduction | Centrosome;Mitochondrion;Microtubule;Nucleus | Protein serine/threonine kinase activity | Centrosome |
| **HUS1** | NP_004498.1 | 3364 | **checkpoint protein HUS1** | 12.9 | 1 | 2 | 2 | 3 | 4.04E+07 | **1.66** | 2 | 32.6 | 280 | 31.7 | 6.8 | Cell communication;Signal transduction | Nucleus;Cytoplasm | Protein binding | Nucleus |
| **INTS8** | NP_060334.2 | 55656 | **integrator complex subunit 8** | 3.8 | 1 | 1 | 1 | 1 | 1.55E+07 | **1.66** | 1 | - | 995 | 113 | 7.1 | Biological_process unknown | - | Molecular function unknown | - |
| **GDAP1** | NP_001035808.1 | 54332 | **ganglioside-induced differentiation-associated protein 1 isoform b** | 18.3 | 2 | 2 | 2 | 2 | 5.29E+06 | **1.65** | 1 | - | 290 | 33.5 | 9.0 | Biological_process unknown | Plasma membrane | Molecular function unknown | Plasma membrane |
| **ZNF326** | NP_892021.1 | 284695 | **DBIRD complex subunit ZNF326 isoform 1** | 25.6 | 2 | 9 | 9 | 28 | 1.98E+09 | **1.65** | 9 | 4.6 | 582 | 65.6 | 5.2 | Regulation of nucleobase, nucleoside, nucleotide and nucleic acid metabolism | Nucleolus | DNA binding | Nucleolus |
| **SCAF1** | NP_067051.2 | 58506 | **splicing factor, arginine/serine-rich 19** | 3.6 | 1 | 2 | 2 | 4 | 8.46E+07 | **1.65** | 3 | 11.6 | 1312 | 139.2 | 9.3 | Regulation of nucleobase, nucleoside, nucleotide and nucleic acid metabolism | Nucleus;Cytoplasm | RNA binding | Nucleus |

| Gene Symbol | RefSeq_Accession | Entrez Gene ID | Description | Coverage (%) | Proteins | Unique Peptides | # Peptides | PSM | Area | SCC-R /SCC-S | SCC-R /SCC-S (Count) | SCC-R /SCC-S (Variability [%]) | # AAs | MW [kDa] | calc. pI | Biological Process | Localization | Molecular Function | Primary Localization |
|---|---|---|---|---|---|---|---|---|---|---|---|---|---|---|---|---|---|---|---|
| **EPB41L2** | NP_001422.1 | 2037 | **band 4.1-like protein 2 isoform a** | 23.1 | 7 | 3 | 16 | 38 | 6.04E+08 | **1.65** | 3 | 14.4 | 1005 | 112.5 | 5.4 | Cell communication;Signal transduction | Plasma membrane;Nucleus;Cytoplasm;Mitochondrion | Structural constituent of cytoskeleton | Plasma membrane |
| **PSAP** | NP_002769.1 | 5660 | **prosaposin isoform a preproprotein** | 39.3 | 3 | 14 | 14 | 32 | 2.34E+08 | **1.65** | 14 | 40.8 | 524 | 58.1 | 5.2 | Cell communication;Signal transduction | Lysosome;Extracellular;Plasma membrane | Lipid binding | Lysosome |
| **TUBB** | NP_821133.1 | 203068 | **tubulin beta chain** | 84.9 | 2 | 7 | 38 | 1571 | 1.51E+10 | **1.65** | 57 | 10.4 | 444 | 49.6 | 4.9 | Cell growth and/or maintenance | Cytoplasm;Plasma membrane;Kinetochore | Structural constituent of cytoskeleton | Cytoplasm;Plasma membrane |
| **DYRK4** | NP_003836.1 | 8798 | **dual specificity tyrosine-phosphorylation-regulated kinase 4 isoform 1** | 1.2 | 1 | 1 | 1 | 1 | 4.45E+08 | **1.65** | 1 | - | 520 | 59.6 | 8.9 | Cell communication;Signal transduction | - | Protein serine/threonine kinase activity | - |
| **HERC4** | NP_001265114.1 | 26091 | **probable E3 ubiquitin-protein ligase HERC4 isoform c** | 24.8 | 5 | 15 | 15 | 28 | 2.81E+08 | **1.65** | 8 | 40.2 | 979 | 109.5 | 6.5 | Protein metabolism | Cytoplasm | Ubiquitin-specific protease activity | Cytoplasm |
| **MAP4K4** | NP_004825.3 | 9448 | **mitogen-activated protein kinase kinase kinase kinase 4 isoform 1** | 10.2 | 13 | 3 | 6 | 8 | 9.52E+07 | **1.65** | 3 | 29.8 | 1165 | 133.3 | 7.7 | Signal transduction | Cytoplasm;Perinuclear region | Protein serine/threonine kinase activity | Cytoplasm |
| **THOC1** | NP_005122.2 | 9984 | **THO complex subunit 1** | 29.5 | 1 | 10 | 10 | 13 | 2.96E+08 | **1.65** | 6 | 31.1 | 657 | 75.6 | 5.0 | Regulation of nucleobase, nucleoside, nucleotide and nucleic acid metabolism | Nucleus | Transcription regulator activity | Nucleus |
| **AP3S1** | NP_001275.1 | 1176 | **AP-3 complex subunit sigma-1** | 21.8 | 1 | 3 | 3 | 7 | 4.79E+07 | **1.65** | 3 | 13.6 | 193 | 21.7 | 5.4 | Cell communication;Signal transduction | Cytoplasm;Golgi apparatus | Receptor signaling complex scaffold activity | Cytoplasm |
| **CTIF** | NP_055587.1 | 9811 | **CBP80/20-dependent translation initiation factor isoform 1** | 4.5 | 2 | 2 | 2 | 2 | 4.98E+06 | **1.64** | 1 | - | 598 | 67.5 | 6.5 | Protein metabolism | - | RNA binding | - |
| **SIVA1** | NP_068355.1 | 10572 | **apoptosis regulatory protein Siva isoform 2** | 11.8 | 2 | 1 | 1 | 1 | 3.60E+07 | **1.64** | 1 | - | 110 | 11.8 | 7.3 | Apoptosis | Cytoplasm;Mitochondrion | Receptor activity;Protein binding | Cytoplasm |
| **TRAPPC6B** | NP_803235.1 | 122553 | **trafficking protein particle complex subunit 6B isoform 2** | 14.6 | 2 | 1 | 1 | 4 | 1.83E+07 | **1.64** | 1 | - | 130 | 14.8 | 8.2 | Biological_process unknown | - | Molecular function unknown | - |
| **TRIO** | NP_009049.2 | 7204 | **triple functional domain protein** | 3.0 | 1 | 3 | 3 | 5 | 7.40E+07 | **1.64** | 3 | 29 | 3097 | 346.7 | 6.4 | Cell communication;Signal transduction | Cytoplasm;Nucleus | Guanyl-nucleotide exchange factor activity | Cytoplasm |
| **ASNS** | NP_001171546.1 | 440 | **asparagine synthetase [glutamine-hydrolyzing] isoform b** | 42.4 | 3 | 16 | 16 | 78 | 7.16E+08 | **1.64** | 20 | 22.9 | 540 | 62.1 | 7.1 | Protein metabolism | Cytoplasm;Cytosol | Ligase activity | Cytoplasm |
| **GNAI2** | NP_001269546.1 | 2771 | **guanine nucleotide-binding protein G(i) subunit alpha-2 isoform 3** | 45.9 | 7 | 9 | 11 | 43 | 3.00E+08 | **1.64** | 10 | 20.6 | 303 | 34.9 | 5.4 | Cell communication;Signal transduction | Plasma membrane;Cytoplasm;Endoplasmic reticulum;Cytosol | GTPase activity;G-protein-coupled receptor binding | Plasma membrane |
| **PNPO** | NP_060599.1 | 55163 | **pyridoxine-5'-phosphate oxidase** | 48.3 | 1 | 6 | 6 | 11 | 2.80E+08 | **1.64** | 6 | 16.2 | 261 | 30 | 7.1 | Metabolism;Energy pathways | Cytoplasm | Catalytic activity | Cytoplasm |
| **PARL** | NP_001032728.1 | 55486 | **presenilins-associated rhomboid-like protein, mitochondrial isoform 2 preproprotein** | 6.1 | 2 | 1 | 1 | 2 | 5.98E+07 | **1.64** | 2 | 71.8 | 329 | 36.6 | 9.9 | Protein metabolism | Mitochondrion | Metallopeptidase activity | Mitochondrion |
| **PYCRL** | NP_075566.2 | 65263 | **pyrroline-5-carboxylate reductase 3** | 23.1 | 1 | 4 | 4 | 6 | 8.78E+07 | **1.64** | 2 | 32.5 | 286 | 29.9 | 8.1 | Metabolism;Energy pathways | - | Oxidoreductase activity | - |
| **REXO2** | NP_056338.2 | 25996 | **oligoribonuclease, mitochondrial precursor** | 35.9 | 1 | 7 | 7 | 11 | 9.00E+07 | **1.64** | 4 | 56.7 | 237 | 26.8 | 6.9 | Regulation of nucleobase, nucleoside, nucleotide and nucleic acid metabolism | Mitochondrion | Ribonuclease activity | Mitochondrion |
| **PITPNA** | NP_006215.1 | 5306 | **phosphatidylinositol transfer protein alpha isoform** | 26.7 | 1 | 3 | 4 | 6 | 2.17E+08 | **1.63** | 3 | 5.9 | 270 | 31.8 | 6.6 | Transport | Cytoplasm | Transporter activity | Cytoplasm |
| **HMGCL** | NP_000182.2 | 3155 | **hydroxymethylglutaryl-CoA lyase, mitochondrial isoform 1 precursor** | 8.3 | 2 | 3 | 3 | 3 | 5.01E+07 | **1.63** | 1 | - | 325 | 34.3 | 8.5 | Metabolism;Energy pathways | Mitochondrion;Peroxisome;Endoplasmic reticulum;Extracellular | Lyase activity | Mitochondrion |
| **ARHGDIB** | NP_001166.3 | 397 | **rho GDP-dissociation inhibitor 2** | 63.7 | 1 | 11 | 11 | 53 | 2.96E+09 | **1.63** | 16 | 17.3 | 201 | 23 | 5.2 | Cell communication;Signal transduction | Cytoplasm;Mitochondrion | Receptor signaling complex scaffold activity | Cytoplasm |
| **CSNK2B** | NP_001311.3 | 1460 | **casein kinase II subunit beta isoform 1** | 36.7 | 2 | 4 | 4 | 15 | 1.65E+08 | **1.63** | 6 | 34.3 | 215 | 24.9 | 5.6 | Cell communication;Signal transduction | Nucleus;Cytoplasm | Protein serine/threonine kinase activity | - |
| **PCK2** | NP_004554.3 | 5106 | **phosphoenolpyruvate carboxykinase [GTP], mitochondrial isoform 1 precursor** | 24.8 | 3 | 10 | 10 | 17 | 1.32E+08 | **1.63** | 9 | 25.5 | 640 | 70.7 | 7.6 | Metabolism;Energy pathways | Mitochondrion;Cytosol | Catalytic activity | Mitochondrion |
| **USF2** | NP_997174.1 | 7392 | **upstream stimulatory factor 2 isoform 2** | 3.9 | 2 | 1 | 1 | 2 | 4.02E+07 | **1.63** | 1 | - | 279 | 30.6 | 5.2 | Regulation of nucleobase, nucleoside, nucleotide and nucleic acid metabolism | Nucleus | Transcription factor activity | Nucleus |
| **PPP5C** | NP_001191213.1 | 5536 | **serine/threonine-protein phosphatase 5 isoform 2** | 26.0 | 2 | 8 | 8 | 18 | 1.39E+08 | **1.63** | 7 | 41.6 | 477 | 54.4 | 6.3 | Cell communication;Signal transduction | Nucleus;Cytoplasm;Plasma membrane | Protein serine/threonine phosphatase activity | Nucleus |
| **ATPAF1** | NP_001243347.1 | 64756 | **ATP synthase mitochondrial F1 complex assembly factor 1 isoform 4** | 17.5 | 4 | 2 | 2 | 5 | 5.54E+07 | **1.63** | 1 | - | 177 | 20.3 | 5.1 | Protein metabolism | Mitochondrion | Chaperone activity | Mitochondrion |
| **CORO1B** | NP_001018080.1 | 57175 | **coronin-1B** | 51.3 | 1 | 17 | 17 | 129 | 1.87E+09 | **1.63** | 30 | 15 | 489 | 54.2 | 5.9 | Cell motility | - | Molecular function unknown | - |
| **USF1** | NP_996888.1 | 7391 | **upstream stimulatory factor 1 isoform 2** | 4.4 | 2 | 1 | 1 | 1 | 1.14E+07 | **1.63** | 1 | - | 251 | 27.4 | 6.3 | Regulation of nucleobase, nucleoside, nucleotide and nucleic acid metabolism | Nucleus | Transcription factor activity | Nucleus |
| **WASL** | NP_003932.3 | 8976 | **neural Wiskott-Aldrich syndrome protein** | 17.4 | 1 | 3 | 3 | 8 | 1.67E+08 | **1.62** | 3 | 20.8 | 505 | 54.8 | 7.9 | Cell growth and/or maintenance | Cytoplasm;Extracellular | Cytoskeletal protein binding | Cytoplasm |
| **XPO4** | NP_071904.4 | 64328 | **exportin-4** | 4.4 | 1 | 3 | 3 | 9 | 1.36E+07 | **1.62** | 3 | 82.8 | 1151 | 130.1 | 5.1 | Transport | Nucleus;Cytoplasm | Transporter activity | Nucleus |
| **CDK2** | NP_439892.2 | 1017 | **cyclin-dependent kinase 2 isoform 2** | 26.5 | 4 | 3 | 5 | 7 | 2.76E+08 | **1.62** | 1 | - | 264 | 30 | 9.1 | Regulation of nucleobase, nucleoside, nucleotide and nucleic acid metabolism | Nucleus;Cytoplasm;Nucleolus | Kinase activity | Nucleus |
| **H1FX** | NP_006017.1 | 8971 | **histone H1x** | 38.0 | 1 | 9 | 9 | 39 | 5.23E+08 | **1.62** | 13 | 17.7 | 213 | 22.5 | 10.8 | Regulation of nucleobase, nucleoside, nucleotide and nucleic acid metabolism | Nucleus;Nucleolus;Mitochondrion | DNA binding | Nucleus |
| **RBM42** | NP_077297.2 | 79171 | **RNA-binding protein 42** | 7.9 | 1 | 2 | 2 | 5 | 1.32E+08 | **1.62** | 2 | 40.7 | 480 | 50.4 | 9.6 | Biological_process unknown | - | Molecular function unknown | - |
| **SNX2** | NP_001265128.1 | 6643 | **sorting nexin-2 isoform 2** | 44.3 | 2 | 12 | 14 | 29 | 7.26E+08 | **1.62** | 11 | 16.9 | 402 | 46.1 | 7.2 | Transport | Endosome;Plasma membrane;Cytoplasm | Transporter activity | Endosome |
| **ARMC10** | NP_001154485.1 | 83787 | **armadillo repeat-containing protein 10 isoform f** | 7.1 | 6 | 1 | 1 | 1 | 2.54E+07 | **1.62** | 1 | - | 225 | 24.7 | 7.5 | Biological_process unknown | Endoplasmic reticulum | Molecular function unknown | Endoplasmic reticulum |
| **IGF2BP1** | NP_006537.3 | 10642 | **insulin-like growth factor 2 mRNA-binding protein 1 isoform 1** | 23.7 | 2 | 10 | 11 | 34 | 1.16E+09 | **1.62** | 8 | 19.6 | 577 | 63.4 | 9.2 | Regulation of nucleobase, nucleoside, nucleotide and nucleic acid metabolism | Nucleus;Cytoplasm | RNA binding | Cytoplasm |
| **PEA15** | NP_003759.1 | 8682 | **astrocytic phosphoprotein PEA-15** | 85.4 | 1 | 11 | 11 | 22 | 3.79E+08 | **1.62** | 9 | 42 | 130 | 15 | 5.0 | Cell communication;Signal transduction | Cytoplasm;Nucleus | Transporter activity | Cytoplasm |

| Gene Symbol | RefSeq_Accession | Entrez Gene ID | Description | Coverage (%) | Proteins | Unique Peptides | # Peptides | PSM | Area | SCC-R /SCC-S | SCC-R /SCC-S (Count) | SCC-R /SCC-S (Variability [%]) | # AAs | MW [kDa] | calc. pI | Biological Process | Localization | Molecular Function | Primary Localization |
|---|---|---|---|---|---|---|---|---|---|---|---|---|---|---|---|---|---|---|---|
| BCKDHA | NP_001158255.1 | 593 | 2-oxoisovalerate dehydrogenase subunit alpha, mitochondrial isoform 2 precursor | 17.3 | 2 | 4 | 4 | 7 | 6.52E+07 | 1.62 | 3 | 7.7 | 444 | 50.4 | 8.3 | Metabolism;Energy pathways | Mitochondrion | Catalytic activity | Mitochondrion |
| GALNT7 | NP_059119.2 | 51809 | N-acetylgalactosaminyltransferase 7 | 15.2 | 1 | 6 | 6 | 8 | 1.10E+08 | 1.62 | 4 | 8.2 | 657 | 75.3 | 7.1 | Metabolism;Energy pathways | Integral to membrane | Transferase activity | Integral to membrane |
| COLGALT1 | NP_078932.2 | 79709 | procollagen galactosyltransferase 1 precursor | 4.3 | 1 | 2 | 2 | 4 | 8.82E+07 | 1.62 | 2 | 63 | 622 | 71.6 | 7.3 | Biological_process unknown | - | Molecular function unknown | - |
| SLC38A9 | NP_775785.2 | 153129 | putative sodium-coupled neutral amino acid transporter 9 isoform 1 | 4.6 | 1 | 1 | 1 | 1 | 4.55E+07 | 1.62 | 1 | - | 561 | 63.7 | 7.7 | Transport | - | Molecular function unknown | - |
| HNRNPL | NP_001524.2 | 3191 | heterogeneous nuclear ribonucleoprotein L isoform a | 59.9 | 2 | 25 | 25 | 209 | 3.53E+09 | 1.61 | 41 | 28 | 589 | 64.1 | 8.2 | Regulation of nucleobase, nucleoside, nucleotide and nucleic acid metabolism | Nucleolus;Nucleus;Cytoplasm;Mitochondrion | RNA binding | Nucleus |
| NDUFV3 | NP_066553.3 | 4731 | NADH dehydrogenase [ubiquinone] flavoprotein 3, mitochondrial isoform a precursor | 8.9 | 1 | 3 | 3 | 3 | 3.10E+07 | 1.61 | 2 | 61.6 | 473 | 51 | 9.2 | Metabolism;Energy pathways | Mitochondrial membrane;Mitochondrion | Oxidoreductase activity | Mitochondrion |
| CCDC58 | NP_001017928.1 | 131076 | coiled-coil domain-containing protein 58 | 55.6 | 1 | 6 | 6 | 27 | 4.27E+08 | 1.61 | 10 | 29.7 | 144 | 16.6 | 7.8 | Biological_process unknown | - | Molecular function unknown | - |
| SNX18 | NP_001138899.1 | 112574 | sorting nexin-18 isoform c | 2.9 | 3 | 1 | 1 | 2 | 5.88E+07 | 1.61 | 1 | - | 591 | 64.7 | 5.7 | Transport | Golgi apparatus | Transporter activity | Golgi apparatus |
| BSDC1 | NP_060515.3 | 55108 | BSD domain-containing protein 1 isoform b | 12.1 | 4 | 4 | 4 | 8 | 8.30E+07 | 1.61 | 4 | 18 | 430 | 47.1 | 4.5 | Biological_process unknown | - | Molecular function unknown | - |
| FAH | NP_000128.1 | 2184 | fumarylacetoacetase | 18.4 | 1 | 5 | 5 | 12 | 1.75E+08 | 1.61 | 3 | 20.2 | 419 | 46.3 | 7.0 | Metabolism;Energy pathways | Cytoplasm;Extracellular | Hydrolase activity | Cytoplasm |
| EIF2AK2 | NP_001129123.1 | 5610 | interferon-induced, double-stranded RNA-activated protein kinase isoform a | 36.7 | 2 | 16 | 16 | 37 | 3.69E+08 | 1.61 | 18 | 16.1 | 551 | 62.1 | 8.4 | Cell communication;Signal transduction | Ribosome;Nucleus;Nucleolus | Protein serine/threonine kinase activity | Ribosome |
| EPS15 | NP_001972.1 | 2060 | epidermal growth factor receptor substrate 15 isoform A | 16.3 | 2 | 9 | 9 | 12 | 3.08E+08 | 1.61 | 6 | 5.7 | 896 | 98.6 | 4.6 | Cell communication;Signal transduction | Cytoplasmic vesicle;Plasma membrane;Endosome;Cytoplasm;Nucleus;Cytosol;Coated vesicle | Calcium ion binding | Plasma membrane |
| NDUFAF3 | NP_951032.1 | 25915 | NADH dehydrogenase [ubiquinone] 1 alpha subcomplex assembly factor 3 isoform a precursor | 26.1 | 2 | 3 | 3 | 5 | 4.94E+07 | 1.61 | 3 | 26 | 184 | 20.3 | 8.2 | Biological_process unknown | - | Molecular function unknown | - |
| EGLN1 | NP_071334.1 | 54583 | egl nine homolog 1 | 13.4 | 1 | 2 | 2 | 6 | 1.46E+08 | 1.60 | 2 | 16.7 | 426 | 46 | 8.5 | Protein metabolism | Cytoplasm | Ubiquitin-specific protease activity | Cytoplasm |
| NOL3 | NP_001263236.1 | 8996 | nucleolar protein 3 isoform MYP | 45.2 | 4 | 5 | 5 | 12 | 1.24E+08 | 1.60 | 4 | 17.3 | 208 | 22.6 | 4.2 | Cell communication;Signal transduction | Nucleolus;Nucleus;Cytoplasm;Mitochondrion | Protease inhibitor activity | Nucleolus |
| PNKD | NP_001070867.1 | 25953 | probable hydrolase PNKD isoform 3 precursor | 10.6 | 2 | 1 | 1 | 1 | 3.56E+07 | 1.60 | 1 | - | 142 | 15.4 | 10.6 | Muscle contraction | Plasma membrane;Cytoplasm;Nucleus;Mitochondrion | Cytoskeletal protein binding | Plasma membrane |
| PPP1R2 | NP_006232.1 | 5504 | protein phosphatase inhibitor 2 isoform 2 | 29.8 | 3 | 4 | 4 | 13 | 1.20E+08 | 1.60 | 6 | 9 | 205 | 23 | 4.7 | Cell communication;Signal transduction | Cytoplasm;Nucleus;Centrosome | Protein binding | Cytoplasm |
| ARL6 | NP_001265222.1 | 84100 | ADP-ribosylation factor-like protein 6 | 4.8 | 1 | 1 | 1 | 1 | 5.23E+07 | 1.60 | 1 | - | 186 | 21.1 | 8.5 | Transport | Nucleus;Cytoplasm;Membrane fraction | GTPase activity | Cytoplasm |
| NEK9 | NP_149107.4 | 91754 | serine/threonine-protein kinase Nek9 | 17.3 | 1 | 7 | 7 | 10 | 1.99E+08 | 1.60 | 5 | 6.3 | 979 | 107.1 | 5.7 | Cell growth and/or maintenance | Cytoplasm;Nucleus | Kinase activity | Cytoplasm |
| SPC25 | NP_065726.1 | 57405 | kinetochore protein Spc25 | 9.8 | 1 | 1 | 1 | 2 | 5.96E+07 | 1.60 | 1 | - | 224 | 26.1 | 8.0 | Apoptosis | Nucleus;Kinetochore | Protein binding | Nucleus |
| GOLGB1 | NP_004478.3 | 2804 | Golgin subfamily B member 1 isoform 2 | 18.0 | 4 | 33 | 33 | 54 | 3.18E+08 | 1.60 | 20 | 30.3 | 3259 | 375.8 | 5.0 | Cell growth and/or maintenance | Golgi apparatus;Extracellular;Mitochondrion | Structural molecule activity | Golgi apparatus |
| CITED2 | NP_001161860.1 | 10370 | cbp/p300-interacting transactivator 2 isoform 1 | 5.6 | 2 | 1 | 1 | 1 | 1.01E+07 | 1.60 | 1 | - | 270 | 28.5 | 7.0 | Regulation of nucleobase, nucleoside, nucleotide and nucleic acid metabolism | Nucleus;Cytoplasm | Transcription regulator activity | Nucleus |
| SLC38A1 | NP_109599.3 | 81539 | sodium-coupled neutral amino acid transporter 1 isoform 1 | 7.6 | 2 | 2 | 2 | 6 | 7.98E+07 | 1.60 | 3 | 14.2 | 487 | 54 | 7.0 | Transport | Plasma membrane | Auxiliary transport protein activity | Plasma membrane |
| RNF214 | NP_001265178.1 | 257160 | RING finger protein 214 isoform 2 | 12.0 | 2 | 3 | 3 | 4 | 6.11E+07 | 1.60 | 2 | 32 | 548 | 61.3 | 8.1 | Protein metabolism | - | Ubiquitin-specific protease activity | - |
| CD109 | NP_598000.2 | 135228 | CD109 antigen isoform 1 preproprotein | 18.8 | 3 | 17 | 17 | 37 | 1.28E+08 | 1.59 | 16 | 36.5 | 1445 | 161.6 | 5.9 | Immune response | Plasma membrane | Molecular function unknown | Plasma membrane |
| MAP2K1 | NP_002746.1 | 5604 | dual specificity mitogen-activated protein kinase kinase 1 | 37.2 | 1 | 5 | 11 | 36 | 4.92E+08 | 1.59 | 5 | 29.5 | 393 | 43.4 | 6.6 | Cell communication;Signal transduction | Cytoplasm | Protein threonine/tyrosine kinase activity | Cytoplasm |
| PKM | NP_002645.3 | 5315 | pyruvate kinase PKM isoform a | 85.3 | 5 | 8 | 51 | 2048 | 2.34E+10 | 1.59 | 41 | 24.4 | 531 | 57.9 | 7.8 | Energy pathways;Metabolism | Cytoplasm;Perinuclear region;Extracellular;Cytosol | Catalytic activity;Kinase activity | Cytoplasm |
| SPNS1 | NP_001135921.1 | 83985 | protein spinster homolog 1 isoform 4 | 10.6 | 3 | 2 | 2 | 3 | 1.14E+08 | 1.59 | 1 | - | 454 | 49 | 7.8 | Cell communication;Signal transduction | Mitochondrion;Cytoplasm | Receptor signaling complex scaffold activity | Mitochondrion |
| USP14 | NP_001032411.1 | 9097 | ubiquitin carboxyl-terminal hydrolase 14 isoform b | 54.9 | 2 | 23 | 23 | 55 | 5.71E+08 | 1.59 | 22 | 19.1 | 459 | 52.4 | 5.8 | Protein metabolism | Cytosol | Ubiquitin-specific protease activity | Cytosol |
| EEF1E1 | NP_004271.1 | 9521 | eukaryotic translation elongation factor 1 epsilon-1 isoform 1 | 31.6 | 2 | 4 | 4 | 22 | 2.44E+08 | 1.59 | 8 | 7.5 | 174 | 19.8 | 8.5 | Protein metabolism | Cytoplasm | Translation regulator activity | Cytoplasm |
| NUP62 | NP_001180286.1 | 23636 | nuclear pore glycoprotein p62 | 12.1 | 1 | 4 | 4 | 9 | 9.72E+07 | 1.59 | 3 | 31.5 | 522 | 53.2 | 5.3 | Transport | Nuclear membrane | Transporter activity | Nuclear membrane |
| PRMT9 | NP_612373.2 | 90826 | putative protein arginine N-methyltransferase 9 | 2.0 | 1 | 1 | 1 | 1 | 2.53E+07 | 1.59 | 1 | - | 845 | 94.4 | 5.3 | Biological_process unknown | - | Molecular function unknown | - |
| STAU2 | NP_001157855.1 | 27067 | double-stranded RNA-binding protein Staufen homolog 2 isoform d | 10.8 | 6 | 3 | 3 | 5 | 5.01E+07 | 1.59 | 3 | 18 | 398 | 43.3 | 9.4 | Regulation of nucleobase, nucleoside, nucleotide and nucleic acid metabolism | Ribosome;Cytoplasm;Nucleus | RNA binding | Ribosome |
| CDK4 | NP_000066.1 | 1019 | cyclin-dependent kinase 4 | 10.9 | 1 | 2 | 2 | 4 | 6.93E+06 | 1.59 | 1 | - | 303 | 33.7 | 7.0 | Cell communication;Signal transduction | Cytoplasm | Kinase activity | Cytoplasm |
| TOM1 | NP_005479.1 | 10043 | target of Myb protein 1 isoform 1 | 53.7 | 4 | 14 | 14 | 33 | 2.32E+08 | 1.59 | 9 | 33.9 | 492 | 53.8 | 4.7 | Cell communication;Signal transduction | Cytoplasm;Endosome | Transporter activity | Cytoplasm |
| ANKLE2 | NP_055929.1 | 23141 | ankyrin repeat and LEM domain-containing protein 2 | 4.7 | 1 | 2 | 2 | 4 | 1.03E+08 | 1.59 | 2 | 12.2 | 938 | 104 | 7.1 | Biological_process unknown | - | Molecular function unknown | - |
| EIF2B3 | NP_001248347.1 | 8891 | translation initiation factor eIF-2B subunit gamma isoform 3 | 36.7 | 3 | 9 | 9 | 19 | 3.01E+08 | 1.59 | 9 | 29.8 | 401 | 44.8 | 6.7 | Protein metabolism | Cytoplasm | Translation regulator activity | Cytoplasm |

| Gene Symbol | RefSeq_Accession | Entrez Gene ID | Description | Coverage (%) | Proteins | Unique Peptides | # Peptides | PSM | Area | SCC-R /SCC-S | SCC-R /SCC-S (Count) | SCC-R /SCC-S (Variability [%]) | # AAs | MW [kDa] | calc. pI | Biological Process | Localization | Molecular Function | Primary Localization |
|---|---|---|---|---|---|---|---|---|---|---|---|---|---|---|---|---|---|---|---|
| **MROH1** | NP_001275743.1 | 727957 | **maestro heat-like repeat-containing protein family member 1 isoform 3** | 3.4 | 3 | 3 | 3 | 4 | 2.51E+07 | **1.59** | 2 | 13.3 | 1632 | 180.2 | 6.9 | Biological_process unknown | - | Molecular function unknown | - |
| **UFM1** | NP_057701.1 | 51569 | **ubiquitin-fold modifier 1 isoform 1 precursor** | 58.8 | 4 | 2 | 2 | 24 | 3.07E+08 | **1.59** | 4 | 3.2 | 85 | 9.1 | 9.3 | Biological_process unknown | - | Molecular function unknown | - |
| **CASP8** | NP_001073593.1 | 841 | **caspase-8 isoform C precursor** | 7.5 | 5 | 2 | 2 | 2 | 2.38E+07 | **1.58** | 1 | - | 464 | 53.7 | 5.2 | Apoptosis | Mitochondrion;Nucleus;Nucleolus;Cytosol;Membrane fraction;Early endosome;Cytoplasm | Cysteine-type peptidase activity | Cytosol;Cytoplasm |
| **FERMT2** | NP_001128472.1 | 10979 | **fermitin family homolog 2 isoform 3** | 35.6 | 3 | 10 | 10 | 28 | 1.71E+08 | **1.58** | 11 | 42.1 | 633 | 72.4 | 7.5 | Cell growth and/or maintenance | Plasma membrane;Nucleus;Cytoplasm | Structural constituent of cytoskeleton | Plasma membrane;Nucleus;Cytoplasm |
| **UBQLN1** | NP_038466.2 | 29979 | **ubiquilin-1 isoform 1** | 56.7 | 3 | 12 | 15 | 57 | 8.16E+08 | **1.58** | 9 | 24.5 | 589 | 62.5 | 5.1 | Protein metabolism | Endoplasmic reticulum;Cytoplasm;Nucleus | Ubiquitin-specific protease activity | Cytoplasm |
| **ZC3H14** | NP_079100.2 | 79882 | **zinc finger CCCH domain-containing protein 14 isoform 1** | 35.1 | 2 | 1 | 15 | 27 | 1.17E+08 | **1.58** | 1 | - | 736 | 82.8 | 7.3 | Biological_process unknown | Nucleus | RNA binding | Nucleus |
| **C12orf75** | NP_001138671.1 | 387882 | **overexpressed in colon carcinoma 1 protein** | 34.9 | 1 | 1 | 1 | 1 | 1.99E+07 | **1.58** | 1 | - | 63 | 6.4 | 6.6 | Biological_process unknown | - | Molecular function unknown | - |
| **CNP** | NP_149124.3 | 1267 | **2',3'-cyclic-nucleotide 3'-phosphodiesterase** | 11.9 | 1 | 4 | 4 | 10 | 1.72E+08 | **1.58** | 3 | 0.6 | 421 | 47.5 | 9.1 | Metabolism;Energy pathways | Cytoplasm;Plasma membrane | Phosphoric diester hydrolase activity | Cytoplasm |
| **DCK** | NP_000779.1 | 1633 | **deoxycytidine kinase** | 21.5 | 1 | 4 | 4 | 5 | 7.58E+07 | **1.58** | 3 | 45.4 | 260 | 30.5 | 5.2 | Regulation of nucleobase, nucleoside, nucleotide and nucleic acid metabolism | Nucleus;Cytoplasm | Catalytic activity | Cytoplasm |
| **FLNB** | NP_001157791.1 | 2317 | **filamin-B isoform 4** | 54.4 | 4 | 96 | 106 | 491 | 4.66E+09 | **1.58** | 136 | 26.5 | 2578 | 275.5 | 5.8 | Cell growth and/or maintenance | Cytoplasm;Nucleus;Plasma membrane | Cytoskeletal protein binding | Cytoplasm |
| **CARD11** | NP_115791.3 | 84433 | **caspase recruitment domain-containing protein 11** | 1.5 | 1 | 1 | 1 | 1 | 2.53E+07 | **1.58** | 1 | - | 1154 | 133.2 | 6.1 | Apoptosis | Plasma membrane;Cytoplasm | Receptor signaling complex scaffold activity | Plasma membrane |
| **UBE2T** | NP_054895.1 | 29089 | **ubiquitin-conjugating enzyme E2 T** | 15.2 | 1 | 2 | 2 | 2 | 6.22E+06 | **1.58** | 2 | 8.2 | 197 | 22.5 | 8.0 | Protein metabolism | - | Ubiquitin-specific protease activity | - |
| **FOXK1** | NP_001032242.1 | 221937 | **forkhead box protein K1** | 31.1 | 1 | 9 | 9 | 15 | 7.02E+07 | **1.58** | 5 | 27.1 | 733 | 75.4 | 9.3 | Transcription | Nucleus;Cytoplasm | Transcription factor activity | Nucleus |
| **HEXA** | NP_000511.2 | 3073 | **beta-hexosaminidase subunit alpha preproprotein** | 10.6 | 1 | 4 | 4 | 5 | 8.30E+07 | **1.58** | 3 | 10.6 | 529 | 60.7 | 5.2 | Metabolism;Energy pathways | Lysosome | Hydrolase activity | Lysosome |
| **FAHD2A** | NP_057128.2 | 51011 | **fumarylacetoacetate hydrolase domain-containing protein 2A** | 43.0 | 2 | 6 | 6 | 12 | 1.51E+08 | **1.58** | 6 | 22.4 | 314 | 34.6 | 8.2 | Biological_process unknown | - | Molecular function unknown | - |
| **LAMP1** | NP_005552.3 | 3916 | **lysosome-associated membrane glycoprotein 1 precursor** | 11.3 | 1 | 5 | 5 | 39 | 1.41E+09 | **1.58** | 11 | 18.9 | 417 | 44.9 | 8.8 | Biological_process unknown | Lysosome;Plasma membrane;Late endosome;Golgi apparatus;Mitochondrion | Molecular function unknown | Lysosome |
| **LSM5** | NP_036454.1 | 23658 | **U6 snRNA-associated Sm-like protein LSm5 isoform a** | 56.0 | 2 | 5 | 5 | 18 | 4.66E+08 | **1.58** | 4 | 6.8 | 91 | 9.9 | 4.5 | Regulation of nucleobase, nucleoside, nucleotide and nucleic acid metabolism | Nucleus;Cytoplasm | RNA binding | Cytoplasm |
| **PCIF1** | NP_071387.1 | 63935 | **phosphorylated CTD-interacting factor 1** | 6.1 | 1 | 2 | 2 | 4 | 7.51E+07 | **1.58** | 3 | 20.7 | 704 | 80.6 | 7.4 | Regulation of nucleobase, nucleoside, nucleotide and nucleic acid metabolism | Nucleus | Transcription regulator activity | Nucleus |
| **ARHGEF10** | NP_055444.2 | 9639 | **rho guanine nucleotide exchange factor 10** | 5.5 | 1 | 3 | 3 | 5 | 4.27E+07 | **1.57** | 3 | 34.5 | 1344 | 148.8 | 5.6 | Cell communication;Signal transduction | Cytoplasm | Guanyl-nucleotide exchange factor activity | Cytoplasm |
| **MAGED1** | NP_008917.3 | 9500 | **melanoma-associated antigen D1 isoform b** | 2.3 | 2 | 1 | 2 | 2 | 9.90E+07 | **1.57** | 1 | - | 778 | 86.1 | 5.8 | Cell communication;Signal transduction;Apoptosis | Cytoplasm;Plasma membrane;Nucleus | Protein binding | Cytoplasm |
| **MRPL10** | NP_660298.2 | 124995 | **39S ribosomal protein L10, mitochondrial isoform a precursor** | 38.7 | 2 | 4 | 4 | 6 | 3.45E+07 | **1.57** | 2 | 16.1 | 261 | 29.3 | 9.6 | Protein metabolism | Mitochondrion | Structural constituent of ribosome | Mitochondrion |
| **TCEB1** | NP_001191793.1 | 6921 | **transcription elongation factor B polypeptide 1 isoform b** | 44.8 | 2 | 3 | 3 | 33 | 4.08E+08 | **1.57** | 10 | 24.7 | 96 | 10.8 | 5.4 | Regulation of nucleobase, nucleoside, nucleotide and nucleic acid metabolism | Nucleus | Transcription regulator activity | Nucleus |
| **ARPC1A** | NP_006400.2 | 10552 | **actin-related protein 2/3 complex subunit 1A isoform 1** | 25.7 | 2 | 5 | 6 | 27 | 3.88E+08 | **1.57** | 5 | 40.4 | 370 | 41.5 | 8.2 | Cell growth and/or maintenance | Cytoplasm;Nucleolus | Structural constituent of cytoskeleton | Cytoplasm |
| **LSM6** | NP_009011.1 | 11157 | **U6 snRNA-associated Sm-like protein LSm6** | 13.8 | 1 | 1 | 1 | 2 | 1.79E+08 | **1.57** | 1 | - | 80 | 9.1 | 9.6 | Regulation of nucleobase, nucleoside, nucleotide and nucleic acid metabolism | Cytoplasm;Nucleus | RNA binding | Cytoplasm |
| **LMNA** | NP_733821.1 | 4000 | **lamin isoform A** | 77.7 | 4 | 5 | 66 | 443 | 7.82E+09 | **1.57** | 8 | 15.8 | 664 | 74.1 | 7.0 | Cell growth and/or maintenance | Nucleus;Cytoplasm;Nucleolus | Structural molecule activity | Nucleus;Cytoplasm |
| **PGLS** | NP_036220.1 | 25796 | **6-phosphogluconolactonase** | 74.4 | 1 | 12 | 12 | 58 | 7.12E+08 | **1.57** | 15 | 24.7 | 258 | 27.5 | 6.1 | Metabolism;Energy pathways | - | Hydrolase activity | - |
| **SRRM2** | NP_057417.3 | 23524 | **serine/arginine repetitive matrix protein 2** | 14.4 | 1 | 24 | 24 | 77 | 4.70E+08 | **1.57** | 26 | 31 | 2752 | 299.4 | 12.1 | Regulation of nucleobase, nucleoside, nucleotide and nucleic acid metabolism | Nucleus;Cytoplasm | RNA binding | Nucleus |
| **YBX1** | NP_004550.2 | 4904 | **nuclease-sensitive element-binding protein 1** | 73.8 | 2 | 12 | 19 | 198 | 3.73E+09 | **1.57** | 26 | 21.7 | 324 | 35.9 | 9.9 | Regulation of nucleobase, nucleoside, nucleotide and nucleic acid metabolism | Cytoplasm;Nucleus | Transcription factor activity | Nucleus |
| **SNAP23** | NP_003816.2 | 8773 | **synaptosomal-associated protein 23 isoform SNAP23A** | 53.6 | 2 | 7 | 7 | 14 | 9.12E+07 | **1.56** | 4 | 34.6 | 211 | 23.3 | 5.0 | Transport | Plasma membrane;Cytoplasm;Cytoplasmic vesicle;Golgi apparatus;Secretory granule;Late endosome | Transporter activity | Plasma membrane |
| **OTULIN** | NP_612357.4 | 90268 | **ubiquitin thioesterase otulin** | 17.3 | 1 | 4 | 4 | 6 | 6.89E+07 | **1.56** | 1 | - | 352 | 40.2 | 5.5 | Biological_process unknown | - | Molecular function unknown | - |
| **PDAP1** | NP_055706.1 | 11333 | **28 kDa heat- and acid-stable phosphoprotein** | 40.9 | 1 | 8 | 8 | 21 | 4.07E+08 | **1.56** | 9 | 8.6 | 181 | 20.6 | 8.9 | Cell communication;Signal transduction | Extracellular | Growth factor binding | Extracellular |
| **COPS8** | NP_006701.1 | 10920 | **COP9 signalosome complex subunit 8 isoform 1** | 58.9 | 2 | 6 | 6 | 12 | 1.94E+08 | **1.56** | 4 | 15.7 | 209 | 23.2 | 5.4 | Regulation of signal transduction | Cytoplasm | Molecular function unknown | Cytoplasm |
| **FYCO1** | NP_078789.2 | 79443 | **FYVE and coiled-coil domain-containing protein 1** | 12.9 | 1 | 11 | 11 | 13 | 7.22E+07 | **1.56** | 5 | 48.1 | 1478 | 166.9 | 4.9 | Cell communication;Signal transduction | - | Molecular function unknown | - |
| **KIF15** | NP_064627.1 | 56992 | **kinesin-like protein KIF15** | 6.1 | 1 | 5 | 5 | 6 | 3.07E+07 | **1.56** | 4 | 3.8 | 1388 | 160.1 | 6.0 | Cell communication;Signal transduction | Centrosome | Protein binding;Protein domain specific binding | Centrosome |
| **MKRN2** | NP_001258636.1 | 23609 | **probable E3 ubiquitin-protein ligase makorin-2 isoform 2** | 9.7 | 2 | 2 | 2 | 3 | 7.41E+07 | **1.56** | 2 | 42.6 | 373 | 42 | 7.1 | Regulation of nucleobase, nucleoside, nucleotide and nucleic acid metabolism | - | Ribonucleoprotein | - |
| **NMD3** | NP_057022.2 | 51068 | **60S ribosomal export protein NMD3** | 17.1 | 1 | 4 | 4 | 10 | 2.78E+08 | **1.56** | 3 | 14.7 | 503 | 57.6 | 7.1 | Biological_process unknown | Nucleolus | Molecular function unknown | Nucleolus |
| **PYGO2** | NP_612157.1 | 90780 | **pygopus homolog 2** | 4.4 | 1 | 1 | 1 | 3 | 1.55E+07 | **1.56** | 2 | 0.7 | 406 | 41.2 | 7.3 | Regulation of nucleobase, nucleoside, nucleotide and nucleic acid metabolism | Nucleus | Transcription regulator activity | Nucleus |

| Gene Symbol | RefSeq_Accession | Entrez Gene ID | Description | Coverage (%) | Proteins | Unique Peptides | # Peptides | PSM | Area | SCC-R /SCC-S | SCC-R /SCC-S (Count) | SCC-R /SCC-S (Variability [%]) | # AAs | MW [kDa] | calc. pI | Biological Process | Localization | Molecular Function | Primary Localization |
|---|---|---|---|---|---|---|---|---|---|---|---|---|---|---|---|---|---|---|---|
| RSF1 | NP_057662.3 | 51773 | remodeling and spacing factor 1 | 11.5 | 1 | 9 | 9 | 14 | 6.55E+07 | 1.56 | 5 | 41 | 1441 | 163.7 | 5.0 | Regulation of nucleobase, nucleoside, nucleotide and nucleic acid metabolism | Nucleus;Cytoplasm | Transcription regulator activity | Nucleus |
| CUTA | NP_057005.1 | 51596 | protein CutA isoform 2 precursor | 55.8 | 3 | 6 | 6 | 28 | 4.82E+08 | 1.56 | 9 | 9.8 | 156 | 16.8 | 5.2 | Biological_process unknown | Plasma membrane | Molecular function unknown | Plasma membrane |
| LSM2 | NP_067000.1 | 57819 | U6 snRNA-associated Sm-like protein LSm2 | 60.0 | 1 | 3 | 3 | 15 | 1.49E+08 | 1.56 | 3 | 12.3 | 95 | 10.8 | 6.5 | Regulation of nucleobase, nucleoside, nucleotide and nucleic acid metabolism | Cytoplasm;Nucleus | RNA binding | Cytoplasm |
| BRD4 | NP_055114.1 | 23476 | bromodomain-containing protein 4 isoform short | 3.7 | 2 | 2 | 2 | 2 | 8.11E+07 | 1.56 | 1 | - | 722 | 80.4 | 8.6 | Cell communication;Signal transduction | Nucleus;Cytoplasm | Chromatin binding | Nucleus;Cytoplasm |
| NSF | NP_006169.2 | 4905 | vesicle-fusing ATPase | 26.5 | 1 | 15 | 15 | 30 | 4.00E+08 | 1.56 | 12 | 26.3 | 744 | 82.5 | 7.0 | Metabolism;Energy pathways | Cytoplasm;Golgi apparatus;Plasma membrane;Cytosol | ATPase activity | Cytoplasm |
| AP2A2 | NP_036437.1 | 161 | AP-2 complex subunit alpha-2 isoform 2 | 29.1 | 2 | 13 | 16 | 42 | 3.63E+08 | 1.56 | 10 | 16.8 | 939 | 103.9 | 7.0 | Transport | Plasma membrane | Transporter activity | Plasma membrane |
| PDPR | NP_060460.4 | 55066 | pyruvate dehydrogenase phosphatase regulatory subunit, mitochondrial precursor | 9.0 | 1 | 5 | 5 | 8 | 3.35E+07 | 1.55 | 4 | 20.6 | 879 | 99.3 | 6.4 | Cell communication;Signal transduction | Mitochondrion | Protein serine/threonine phosphatase activity | Mitochondrion |
| RAD23A | NP_005044.1 | 5886 | UV excision repair protein RAD23 homolog A isoform 1 | 46.8 | 2 | 1 | 4 | 12 | 2.23E+08 | 1.55 | 1 | - | 363 | 39.6 | 4.6 | DNA repair | Nucleus | DNA binding | Nucleus |
| HINT1 | NP_005331.1 | 3094 | histidine triad nucleotide-binding protein 1 | 88.1 | 1 | 9 | 9 | 109 | 5.11E+09 | 1.55 | 29 | 19.5 | 126 | 13.8 | 7.0 | Cell communication;Signal transduction | Cytoplasm;Nucleus | ATPase activity | Cytoplasm |
| EDC3 | NP_079359.2 | 80153 | enhancer of mRNA-decapping protein 3 | 7.3 | 1 | 2 | 2 | 5 | 1.38E+08 | 1.55 | 3 | 15.1 | 508 | 56 | 7.1 | Nucleobase, nucleoside, nucleotide and nucleic acid transport | Cytoplasm | RNA binding | Cytoplasm |
| ACO1 | NP_002188.1 | 48 | cytoplasmic aconitate hydratase | 30.3 | 1 | 16 | 16 | 47 | 4.22E+08 | 1.55 | 10 | 35.8 | 889 | 98.3 | 6.7 | Regulation of nucleobase, nucleoside, nucleotide and nucleic acid metabolism | Cytoplasm;Golgi membrane;Endoplasmic reticulum membrane | Isomerase activity | Cytoplasm |
| BRIX1 | NP_060791.3 | 55299 | ribosome biogenesis protein BRX1 homolog | 41.6 | 1 | 12 | 12 | 43 | 8.21E+08 | 1.55 | 14 | 32.8 | 353 | 41.4 | 9.9 | Biological_process unknown | Nucleolus;Extracellular;Ribosome;Nucleus | Molecular function unknown | Nucleolus |
| SEPT11 | NP_060713.1 | 55752 | septin-11 | 48.0 | 2 | 8 | 16 | 44 | 6.09E+08 | 1.55 | 6 | 7.7 | 429 | 49.4 | 6.8 | Cell cycle;Vesicle-mediated transport | Perinuclear region;Cytoplasm;Microtubule | GTPase activity | Perinuclear region |
| DOHH | NP_001138637.1 | 83475 | deoxyhypusine hydroxylase | 34.1 | 1 | 5 | 5 | 9 | 1.51E+08 | 1.55 | 2 | 79.5 | 302 | 32.9 | 4.8 | Protein modification | - | Oxidoreductase activity | - |
| FLNA | NP_001447.2 | 2316 | filamin-A isoform 1 | 61.8 | 2 | 111 | 120 | 724 | 4.00E+09 | 1.55 | 168 | 26.1 | 2639 | 279.8 | 6.1 | Cell growth and/or maintenance | Cytoplasm;Nucleus;Plasma membrane;Cell projection;Cytosol;Mitochondrion | Cytoskeletal anchoring activity | Cytoplasm |
| PPP1R12A | NP_002471.1 | 4659 | protein phosphatase 1 regulatory subunit 12A isoform a | 18.1 | 4 | 14 | 14 | 31 | 2.39E+08 | 1.55 | 13 | 37.8 | 1030 | 115.2 | 5.4 | Metabolism;Energy pathways | Cytoplasm;Plasma membrane | Phosphatase regulator activity | Cytoplasm |
| BLOC1S3 | NP_997715.1 | 388552 | biogenesis of lysosome-related organelles complex 1 subunit 3 | 9.4 | 1 | 1 | 1 | 2 | 5.49E+07 | 1.55 | 1 | - | 202 | 21.2 | 5.2 | Cell growth and/or maintenance | Cytoplasm | Molecular function unknown | Cytoplasm |
| FAM192A | NP_079222.1 | 80011 | protein FAM192A | 15.8 | 1 | 2 | 2 | 5 | 1.74E+08 | 1.55 | 3 | 13.6 | 254 | 28.9 | 5.5 | Biological_process unknown | Nucleus | Molecular function unknown | Nucleus |
| PFDN1 | NP_002613.2 | 5201 | prefoldin subunit 1 | 36.9 | 1 | 3 | 3 | 10 | 2.15E+08 | 1.55 | 3 | 6.8 | 122 | 14.2 | 6.8 | Protein metabolism | Cytoplasm | Chaperone activity | Cytoplasm |
| ARL8A | NP_620150.1 | 127829 | ADP-ribosylation factor-like protein 8A isoform 1 | 40.9 | 2 | 2 | 6 | 19 | 4.81E+08 | 1.55 | 1 | - | 186 | 21.4 | 7.8 | Cell communication;Signal transduction | - | GTPase activity | - |
| CENPK | NP_001253967.1 | 64105 | centromere protein K | 7.4 | 1 | 1 | 1 | 1 | 6.57E+06 | 1.55 | 1 | - | 269 | 31.6 | 4.9 | Biological_process unknown | Nucleus | Molecular function unknown | Nucleus |
| HNRNPC | NP_001070910.1 | 3183 | heterogeneous nuclear ribonucleoproteins C1/C2 isoform a | 67.3 | 5 | 2 | 22 | 173 | 5.14E+09 | 1.55 | 1 | - | 306 | 33.6 | 5.1 | Regulation of nucleobase, nucleoside, nucleotide and nucleic acid metabolism | Nucleus;Cytoplasm;Nucleolus;Mitochondrion | RNA binding | Nucleus |
| NUCKS1 | NP_073568.2 | 64710 | nuclear ubiquitous casein and cyclin-dependent kinase substrate 1 | 4.5 | 1 | 2 | 2 | 6 | 8.11E+07 | 1.54 | 3 | 3.4 | 243 | 27.3 | 5.1 | Regulation of nucleobase, nucleoside, nucleotide and nucleic acid metabolism | Nucleus;Cytoplasm | DNA binding | Nucleus |
| FMR1 | NP_001172010.1 | 2332 | fragile X mental retardation protein 1 isoform ISO12 | 16.5 | 5 | 4 | 5 | 8 | 8.16E+07 | 1.54 | 4 | 39.4 | 516 | 58.8 | 6.3 | Regulation of nucleobase, nucleoside, nucleotide and nucleic acid metabolism | Cytoplasm;Nucleus | RNA binding | Cytoplasm;Nucleus |
| CAPNS1 | NP_001740.1 | 826 | calpain small subunit 1 | 75.8 | 2 | 13 | 13 | 171 | 7.69E+08 | 1.54 | 36 | 32.1 | 268 | 28.3 | 5.2 | Protein folding | Cytoplasm;Plasma membrane;Nucleus | Peptide binding | Cytoplasm |
| BRD2 | NP_001278915.1 | 6046 | bromodomain-containing protein 2 isoform 4 | 10.9 | 4 | 4 | 4 | 9 | 1.52E+08 | 1.54 | 5 | 20.7 | 681 | 74.8 | 8.7 | Regulation of nucleobase, nucleoside, nucleotide and nucleic acid metabolism | Nucleus;Cytoplasm | Transcription regulator activity | Nucleus |
| CD151 | NP_620598.1 | 977 | CD151 antigen | 9.9 | 1 | 3 | 3 | 7 | 1.22E+08 | 1.54 | 3 | 1.3 | 253 | 28.3 | 7.5 | Cell communication;Cell motility | Plasma membrane;Late endosome;Perinuclear region;Early endosome | Receptor activity | Plasma membrane |
| HDDC2 | NP_057147.2 | 51020 | HD domain-containing protein 2 | 49.0 | 1 | 5 | 5 | 9 | 9.48E+07 | 1.54 | 4 | 7.5 | 204 | 23.4 | 5.5 | Biological_process unknown | - | Molecular function unknown | - |
| TJAP1 | NP_001139492.1 | 93643 | tight junction-associated protein 1 isoform b | 8.0 | 2 | 3 | 3 | 3 | 2.23E+07 | 1.54 | 2 | 30.9 | 547 | 60.7 | 5.9 | Cell growth and/or maintenance | Plasma membrane | Cell adhesion molecule activity | Plasma membrane |
| DYNLT3 | NP_006511.1 | 6990 | dynein light chain Tctex-type 3 | 23.3 | 1 | 2 | 2 | 3 | 5.93E+07 | 1.54 | 1 | - | 116 | 13.1 | 5.7 | Biological_process unknown | - | Molecular function unknown | - |
| NME7 | NP_932076.1 | 29922 | nucleoside diphosphate kinase 7 isoform b | 10.3 | 2 | 2 | 2 | 3 | 1.03E+08 | 1.54 | 2 | 8.7 | 340 | 38.2 | 6.8 | Metabolism;Energy pathways | - | Catalytic activity | - |
| PRKCSH | NP_001276032.1 | 5589 | glucosidase 2 subunit beta isoform 3 precursor | 55.7 | 3 | 24 | 24 | 167 | 1.88E+09 | 1.54 | 36 | 19.8 | 535 | 60.2 | 4.4 | Energy pathways;Metabolism | Endoplasmic reticulum | Glucosidase activity | Endoplasmic reticulum |
| ZMYM4 | NP_005086.2 | 9202 | zinc finger MYM-type protein 4 | 2.2 | 1 | 2 | 2 | 3 | 5.66E+07 | 1.54 | 1 | - | 1548 | 172.7 | 6.8 | Biological_process unknown | Nucleus | Molecular function unknown | Nucleus |
| CD81 | NP_004347.1 | 975 | CD81 antigen | 18.2 | 1 | 2 | 2 | 8 | 8.77E+08 | 1.54 | 4 | 28.8 | 236 | 25.8 | 5.3 | Metabolism;Energy pathways | Plasma membrane;Endosome | Catalytic activity | Plasma membrane |
| FAM195A | NP_612427.2 | 84331 | protein FAM195A | 6.9 | 1 | 1 | 1 | 1 | 2.19E+07 | 1.53 | 1 | - | 160 | 17.8 | 9.4 | Biological_process unknown | - | Molecular function unknown | - |
| PCYT1A | NP_005008.2 | 5130 | choline-phosphate cytidylyltransferase A | 38.4 | 4 | 9 | 9 | 20 | 1.86E+08 | 1.53 | 8 | 37.2 | 367 | 41.7 | 7.3 | Metabolism;Energy pathways | Nucleus;Endoplasmic reticulum;Golgi apparatus;Cytoplasm | Nucleotidyltransferase activity | Nucleus |
| TRIM56 | NP_112223.1 | 81844 | E3 ubiquitin-protein ligase TRIM56 | 13.9 | 1 | 6 | 6 | 10 | 2.70E+08 | 1.53 | 6 | 33.7 | 755 | 81.4 | 7.7 | Regulation of nucleobase, nucleoside, nucleotide and nucleic acid metabolism | Nucleus | Transcription regulator activity | Nucleus |

| Gene Symbol | RefSeq_Accession | Entrez Gene ID | Description | Coverage (%) | Proteins | Unique Peptides | # Peptides | PSM | Area | SCC-R /SCC-S | SCC-R /SCC-S (Count) | SCC-R /SCC-S (Variability [%]) | # AAs | MW [kDa] | calc. pI | Biological Process | Localization | Molecular Function | Primary Localization |
|---|---|---|---|---|---|---|---|---|---|---|---|---|---|---|---|---|---|---|---|
| **RAP1GDS1** | NP_066982.3 | 5910 | **rap1 GTPase-GDP dissociation stimulator 1 isoform 2** | 20.8 | 6 | 9 | 9 | 18 | 7.27E+08 | **1.53** | 7 | 46.4 | 607 | 66.3 | 5.3 | Cell communication;Signal transduction | - | Guanyl-nucleotide exchange factor activity | - |
| **RCN1** | NP_002892.1 | 5954 | **reticulocalbin-1 precursor** | 49.6 | 1 | 14 | 14 | 43 | 1.06E+09 | **1.53** | 17 | 33.1 | 331 | 38.9 | 5.0 | Cell communication;Signal transduction | Endoplasmic reticulum | Calcium ion binding | Endoplasmic reticulum |
| **PDE6D** | NP_001277947.1 | 5147 | **retinal rod rhodopsin-sensitive cGMP 3',5'-cyclic phosphodiesterase subunit delta isoform 2** | 33.3 | 2 | 2 | 2 | 4 | 1.81E+08 | **1.53** | 2 | 6.1 | 93 | 10.8 | 8.9 | Metabolism;Energy pathways | Cytoplasm;Cytoplasmic vesicle | Hydrolase activity | Cytoplasm |
| **MFN2** | NP_001121132.1 | 9927 | **mitofusin-2** | 2.3 | 1 | 1 | 1 | 1 | 5.82E+07 | **1.53** | 1 | - | 757 | 86.3 | 7.0 | Metabolism;Energy pathways | Mitochondrion;Endoplasmic reticulum;Cytoplasm;Mitochondrial membrane | Molecular function unknown | Mitochondrion |
| **CAP1** | NP_001099000.1 | 10487 | **adenylyl cyclase-associated protein 1** | 78.1 | 1 | 33 | 34 | 320 | 5.37E+09 | **1.53** | 88 | 19.2 | 475 | 51.6 | 8.0 | Cell growth and/or maintenance | Cytoplasm;Nucleus | Molecular function unknown | Cytoplasm |
| **KDM3A** | NP_001140160.1 | 55818 | **lysine-specific demethylase 3A** | 1.9 | 1 | 1 | 1 | 1 | 3.24E+07 | **1.53** | 1 | - | 1321 | 147.2 | 8.1 | Biological_process unknown | - | Molecular function unknown | - |
| **DLG1** | NP_001191315.1 | 1739 | **disks large homolog 1 isoform 3** | 9.4 | 10 | 5 | 5 | 8 | 1.14E+08 | **1.52** | 1 | - | 892 | 99.8 | 6.0 | Cell communication;Signal transduction | Plasma membrane;Cytoplasm;Nucleus;Membrane fraction;Cell junction | Cell adhesion molecule activity | Plasma membrane;Nucleus;Cytoplasm;Membrane fraction;Cell junction |
| **FAM103A1** | NP_113640.1 | 83640 | **RNMT-activating mini protein** | 23.7 | 1 | 1 | 1 | 1 | 3.93E+07 | **1.52** | 1 | - | 118 | 14.4 | 8.9 | Biological_process unknown | - | Molecular function unknown | - |
| **GPN1** | NP_001138519.1 | 11321 | **GPN-loop GTPase 1 isoform b** | 14.6 | 4 | 2 | 2 | 3 | 4.28E+07 | **1.52** | 2 | 25.6 | 362 | 40.9 | 5.0 | Cell communication;Signal transduction | Cytoplasm;Nucleus | GTPase activity | Cytoplasm |
| **DHPS** | NP_037538.1 | 1725 | **deoxyhypusine synthase isoform b** | 16.5 | 3 | 3 | 3 | 5 | 3.37E+07 | **1.52** | 1 | - | 322 | 35.9 | 5.1 | Metabolism;Energy pathways | - | Transferase activity | - |
| **PCNP** | NP_065090.1 | 57092 | **PEST proteolytic signal-containing nuclear protein** | 45.5 | 1 | 7 | 7 | 44 | 3.99E+08 | **1.52** | 17 | 21.8 | 178 | 18.9 | 7.5 | Protein metabolism | Nucleus | Ubiquitin-specific protease activity | Nucleus |
| **PLIN3** | NP_001157661.1 | 10226 | **perilipin-3 isoform 2** | 74.6 | 3 | 26 | 26 | 194 | 1.11E+09 | **1.52** | 47 | 23 | 433 | 46.9 | 5.4 | Transport | Extracellular;Endosome;Cytoplasm | Transporter activity | Cytoplasm |
| **COX6B1** | NP_001854.1 | 1340 | **cytochrome c oxidase subunit 6B1** | 73.3 | 1 | 8 | 8 | 27 | 3.48E+08 | **1.52** | 8 | 13.9 | 86 | 10.2 | 7.1 | Metabolism;Energy pathways | Mitochondrion | Oxidoreductase activity | Mitochondrion |
| **PHF6** | NP_115834.1 | 84295 | **PHD finger protein 6 isoform 1** | 22.5 | 2 | 5 | 5 | 11 | 3.58E+08 | **1.52** | 6 | 5.9 | 365 | 41.3 | 8.7 | Regulation of nucleobase, nucleoside, nucleotide and nucleic acid metabolism | Nucleolus;Nucleus;Cytoplasm | Transcription regulator activity | Nucleolus;Nucleus;Cytoplasm |
| **WDFY1** | NP_065881.1 | 57590 | **WD repeat and FYVE domain-containing protein 1** | 11.5 | 1 | 3 | 3 | 6 | 4.67E+07 | **1.52** | 2 | 16.5 | 410 | 46.3 | 7.3 | Biological_process unknown | Endosome | Molecular function unknown | Endosome |
| **CMPK1** | NP_057392.1 | 51727 | **UMP-CMP kinase isoform a** | 47.4 | 2 | 10 | 10 | 42 | 5.59E+08 | **1.52** | 15 | 21.1 | 228 | 25.8 | 8.0 | Regulation of nucleobase, nucleoside, nucleotide and nucleic acid metabolism | Cytoplasm;Nucleus | Catalytic activity | Cytoplasm |
| **ACAP2** | NP_036419.3 | 23527 | **arf-GAP with coiled-coil, ANK repeat and PH domain-containing protein 2** | 13.9 | 1 | 5 | 5 | 5 | 9.42E+07 | **1.52** | 3 | 25.7 | 778 | 88 | 6.8 | Cell communication;Signal transduction | Cytoplasm;Plasma membrane;Endosome | GTPase activator activity | Cytoplasm |
| **IQGAP1** | NP_003861.1 | 8826 | **ras GTPase-activating-like protein IQGAP1** | 61.7 | 6 | 80 | 80 | 419 | 2.77E+09 | **1.52** | 108 | 15.8 | 1657 | 189.1 | 6.5 | Cytoskeleton organization and biogenesis | Cytoplasm;Plasma membrane;Golgi apparatus;Mitochondrion | GTPase activator activity | Cytoplasm |
| **SEC22B** | NP_004883.3 | 9554 | **vesicle-trafficking protein SEC22b precursor** | 52.6 | 1 | 10 | 10 | 40 | 6.35E+08 | **1.52** | 11 | 12.1 | 215 | 24.7 | 8.5 | Cell communication;Signal transduction | Endoplasmic reticulum;Mitochondrion | Transporter activity | Endoplasmic reticulum |
| **ILK** | NP_001265371.1 | 3611 | **integrin-linked protein kinase isoform 3** | 19.2 | 3 | 5 | 5 | 10 | 1.87E+08 | **1.52** | 6 | 12.1 | 318 | 36.4 | 9.4 | Cell communication;Signal transduction | Cytoplasm;Nucleus;Microsome | Protein serine/threonine kinase activity | Cytoplasm |
| **ACAD9** | NP_054768.2 | 28976 | **acyl-CoA dehydrogenase family member 9, mitochondrial** | 23.8 | 1 | 9 | 9 | 29 | 3.09E+08 | **1.52** | 12 | 28.7 | 621 | 68.7 | 8.0 | Metabolism;Energy pathways | Mitochondrion | Catalytic activity | Mitochondrion |
| **LASP1** | NP_006139.1 | 3927 | **LIM and SH3 domain protein 1 isoform a** | 58.2 | 2 | 15 | 15 | 44 | 1.97E+09 | **1.51** | 17 | 28.2 | 261 | 29.7 | 7.1 | Signal transduction;Regulation of signal transduction;Cytoskeleton organization and biogenesis | Cytosol;Cytoskeleton;Focal adhesion | Cytoskeletal protein binding | Focal adhesion |
| **C11orf73** | NP_057485.2 | 51501 | **protein Hikeshi** | 14.7 | 1 | 2 | 2 | 6 | 2.35E+08 | **1.51** | 3 | 13 | 197 | 21.6 | 5.5 | Biological_process unknown | - | Molecular function unknown | - |
| **HACL1** | NP_001271342.1 | 26061 | **2-hydroxyacyl-CoA lyase 1 isoform b** | 18.7 | 4 | 6 | 6 | 11 | 7.29E+07 | **1.51** | 3 | 9.9 | 551 | 61 | 7.4 | Metabolism;Energy pathways | Peroxisome | Lyase activity | Peroxisome |
| **ZBED2** | NP_078784.2 | 79413 | **zinc finger BED domain-containing protein 2** | 3.2 | 1 | 1 | 1 | 2 | 1.54E+07 | **1.51** | 1 | - | 218 | 25.1 | 7.1 | Biological_process unknown | Nucleus | DNA binding | Nucleus |
| **MAP2K2** | NP_109587.1 | 5605 | **dual specificity mitogen-activated protein kinase kinase 2** | 47.3 | 1 | 10 | 16 | 48 | 6.86E+08 | **1.51** | 9 | 29.8 | 400 | 44.4 | 6.6 | Cell communication;Signal transduction | Cytoplasm;Nucleus | Protein threonine/tyrosine kinase activity | Cytoplasm |
| **PIGU** | NP_536724.1 | 128869 | **phosphatidylinositol glycan anchor biosynthesis class U protein precursor** | 3.9 | 1 | 1 | 1 | 1 | 5.00E+06 | **1.51** | 1 | - | 435 | 50 | 7.7 | Biological_process unknown | Plasma membrane;Endoplasmic reticulum | Hydrolase activity | Plasma membrane |
| **RBM12** | NP_001185767.1 | 10137 | **RNA-binding protein 12** | 9.1 | 1 | 4 | 4 | 9 | 1.42E+08 | **1.51** | 3 | 17.2 | 932 | 97.3 | 8.6 | Regulation of nucleobase, nucleoside, nucleotide and nucleic acid metabolism | Mitochondrion;Nucleus;Cytoplasm | RNA binding | Mitochondrion |
| **CPSF4** | NP_001075028.1 | 10898 | **cleavage and polyadenylation specificity factor subunit 4 isoform 2** | 12.3 | 2 | 2 | 2 | 3 | 2.29E+07 | **1.51** | 2 | 57.5 | 244 | 27.5 | 8.2 | Regulation of nucleobase, nucleoside, nucleotide and nucleic acid metabolism | Nucleus | RNA binding | Nucleus |
| **MTAP** | NP_002442.2 | 4507 | **S-methyl-5'-thioadenosine phosphorylase** | 49.5 | 1 | 7 | 7 | 18 | 1.84E+08 | **1.51** | 7 | 18.1 | 283 | 31.2 | 7.2 | Metabolism;Energy pathways | Cytoplasm | Phosphorylase activity | Cytoplasm |
| **TTC4** | NP_004614.3 | 7268 | **tetratricopeptide repeat protein 4 isoform 1** | 26.6 | 2 | 5 | 5 | 10 | 8.57E+07 | **1.51** | 3 | 7.8 | 387 | 44.7 | 5.6 | Biological_process unknown | - | Molecular function unknown | - |
| **C11orf58** | NP_055082.1 | 10944 | **small acidic protein** | 26.2 | 1 | 5 | 5 | 27 | 5.75E+08 | **1.51** | 8 | 9.8 | 183 | 20.3 | 4.7 | Biological_process unknown | - | Molecular function unknown | - |
| **KPNA4** | NP_002259.1 | 3840 | **importin subunit alpha-3** | 47.6 | 1 | 14 | 17 | 68 | 5.74E+08 | **1.51** | 19 | 16.9 | 521 | 57.9 | 5.0 | Transport | Cytoplasm;Nucleus;Nucleolus | Transporter activity | Cytoplasm |
| **RALB** | NP_002872.1 | 5899 | **ras-related protein Ral-B** | 31.1 | 1 | 1 | 4 | 8 | 2.41E+08 | **1.51** | 1 | - | 206 | 23.4 | 6.6 | Cell communication;Signal transduction | Cytoplasm | GTPase activity | Cytoplasm |
| **HOMER3** | NP_001139194.1 | 9454 | **homer protein homolog 3 isoform 1** | 13.0 | 3 | 2 | 2 | 3 | 6.42E+07 | **1.51** | 2 | 7.4 | 361 | 39.8 | 5.5 | Cell communication;Signal transduction | Plasma membrane;Perinuclear region;Cytoplasm;Endoplasmic reticulum;Extracellular;Nucleus | Receptor signaling complex scaffold activity | Cytoplasm |
| **MTMR14** | NP_071930.2 | 64419 | **myotubularin-related protein 14 isoform 3** | 11.0 | 3 | 3 | 3 | 4 | 5.15E+07 | **1.51** | 1 | - | 538 | 60 | 6.2 | Phosphoinositide-mediated signaling;Lipid metabolism;Endosome transport;Vesicle-mediated transport | Golgi apparatus;Cytoplasm;Nucleus;Plasma membrane | Lipid phosphatase activity | Cytoplasm |
| **PCLO** | NP_055325.2 | 27445 | **protein piccolo isoform 2** | 0.2 | 2 | 1 | 1 | 5 | 1.53E+08 | **1.50** | 3 | 4.4 | 4935 | 537.6 | 6.3 | Synaptic vesicle transport | - | Structural constituent of cytoskeleton | - |

| Gene Symbol | RefSeq_Accession | Entrez Gene ID | Description | Coverage (%) | Proteins | Unique Peptides | # Peptides | PSM | Area | SCC-R /SCC-S | SCC-R /SCC-S (Count) | SCC-R /SCC-S (Variability [%]) | # AAs | MW [kDa] | calc. pI | Biological Process | Localization | Molecular Function | Primary Localization |
|---|---|---|---|---|---|---|---|---|---|---|---|---|---|---|---|---|---|---|---|
| **CC2D1A** | NP_060191.3 | 54862 | **coiled-coil and C2 domain-containing protein 1A** | 4.7 | 1 | 2 | 2 | 3 | 6.09E+07 | **1.50** | 2 | 21.2 | 951 | 104 | 8.1 | Cell communication;Signal transduction | Cytoplasm | Transcription regulator activity | Cytoplasm |
| **VWA9** | NP_001193988.1 | 81556 | **von Willebrand factor A domain-containing protein 9 isoform 5** | 7.8 | 2 | 2 | 2 | 2 | 1.42E+07 | **1.50** | 1 | - | 461 | 50.7 | 5.1 | Biological_process unknown | - | Molecular function unknown | - |
| **NDUFV2** | NP_066552.2 | 4729 | **NADH dehydrogenase [ubiquinone] flavoprotein 2, mitochondrial precursor** | 42.2 | 1 | 8 | 8 | 15 | 2.71E+08 | **1.50** | 7 | 36.8 | 249 | 27.4 | 8.1 | Metabolism;Energy pathways | Mitochondrion | Oxidoreductase activity | Mitochondrion |
| **SENP8** | NP_660205.3 | 123228 | **sentrin-specific protease 8** | 19.3 | 1 | 2 | 2 | 2 | 2.21E+07 | **1.50** | 1 | - | 212 | 24.1 | 6.0 | Protein metabolism | Cytoplasm | Cysteine-type peptidase activity | Cytoplasm |
| **ZMYND8** | NP_001268710.1 | 23613 | **protein kinase C-binding protein 1 isoform o** | 8.3 | 18 | 4 | 4 | 5 | 3.39E+08 | **1.50** | 1 | - | 1088 | 120.8 | 6.3 | Regulation of nucleobase, nucleoside, nucleotide and nucleic acid metabolism | Nucleus;Cytoplasm | Transcription regulator activity | Nucleus;Cytoplasm |
| **KRR1** | NP_008974.5 | 11103 | **KRR1 small subunit processome component homolog** | 22.3 | 1 | 4 | 4 | 10 | 7.60E+07 | **1.50** | 1 | - | 381 | 43.6 | 9.8 | Regulation of nucleobase, nucleoside, nucleotide and nucleic acid metabolism | Nucleolus;Nucleus | DNA binding | Nucleolus |
| **PHRF1** | NP_001273512.1 | 57661 | **PHD and RING finger domain-containing protein 1 isoform 4** | 2.6 | 4 | 2 | 2 | 3 | 6.47E+07 | **1.50** | 2 | 5.5 | 1645 | 178.1 | 9.0 | Biological_process unknown | - | Molecular function unknown | - |
| **ALKBH4** | NP_060091.1 | 54784 | **alpha-ketoglutarate-dependent dioxygenase alkB homolog 4** | 9.3 | 1 | 1 | 1 | 1 | 6.87E+06 | **1.50** | 1 | - | 302 | 33.8 | 6.7 | Biological_process unknown | - | Molecular function unknown | - |
| **MTPN** | NP_665807.1 | 136319 | **myotrophin** | 79.7 | 1 | 7 | 7 | 65 | 1.52E+09 | **1.50** | 15 | 14 | 118 | 12.9 | 5.5 | Protein metabolism | Cytoplasm;Nucleus | Protein binding | Cytoplasm |
| **RMDN1** | NP_057117.2 | 51115 | **regulator of microtubule dynamics protein 1 isoform 1** | 17.8 | 3 | 4 | 4 | 7 | 1.01E+08 | **1.50** | 3 | 6.3 | 314 | 35.8 | 8.5 | Biological_process unknown | - | Molecular function unknown | - |
| **RBM3** | NP_006734.1 | 5935 | **putative RNA-binding protein 3** | 51.6 | 1 | 4 | 4 | 36 | 1.31E+09 | **1.49** | 10 | 12.9 | 157 | 17.2 | 8.9 | Regulation of nucleobase, nucleoside, nucleotide and nucleic acid metabolism | Nucleolus | RNA binding | Nucleolus |
| **NFKB1** | NP_001158884.1 | 4790 | **nuclear factor NF-kappa-B p105 subunit isoform 2** | 10.9 | 2 | 5 | 5 | 5 | 6.49E+07 | **1.49** | 3 | 29.5 | 968 | 105.3 | 5.4 | Regulation of nucleobase, nucleoside, nucleotide and nucleic acid metabolism | Mitochondrion;Cytoplasm;Nucleus | Transcription factor activity | Nucleus |
| **UBA2** | NP_005490.1 | 10054 | **SUMO-activating enzyme subunit 2** | 43.4 | 1 | 15 | 15 | 37 | 2.23E+08 | **1.49** | 12 | 33.2 | 640 | 71.2 | 5.3 | Protein metabolism | - | Ubiquitin-specific protease activity | - |
| **UQCC1** | NP_001171906.1 | 55245 | **ubiquinol-cytochrome-c reductase complex assembly factor 1 isoform c** | 4.8 | 3 | 1 | 1 | 1 | 7.11E+07 | **1.49** | 1 | - | 231 | 26.9 | 8.4 | Biological_process unknown | Cytoplasmic vesicle | Molecular function unknown | Cytoplasmic vesicle |
| **IFT74** | NP_079379.2 | 80173 | **intraflagellar transport protein 74 homolog isoform a** | 2.7 | 1 | 1 | 1 | 2 | 2.87E+07 | **1.49** | 1 | - | 600 | 69.2 | 6.0 | Cell growth and/or maintenance | Intracellular vesicle | Molecular function unknown | Intracellular vesicle |
| **ACO2** | NP_001089.1 | 50 | **aconitate hydratase, mitochondrial precursor** | 32.3 | 1 | 16 | 16 | 37 | 1.91E+08 | **1.49** | 16 | 29 | 780 | 85.4 | 7.6 | Metabolism;Energy pathways | Mitochondrion | Catalytic activity | Mitochondrion |
| **AGPAT6** | NP_848934.1 | 137964 | **glycerol-3-phosphate acyltransferase 6** | 2.6 | 1 | 1 | 1 | 1 | 3.03E+07 | **1.49** | 1 | - | 456 | 52 | 9.2 | Metabolism;Energy pathways | Integral to membrane | Acyltransferase activity | Integral to membrane |
| **GOLT1B** | NP_057156.1 | 51026 | **vesicle transport protein GOT1B** | 20.3 | 1 | 3 | 3 | 10 | 2.02E+08 | **1.49** | 5 | 2.3 | 138 | 15.4 | 10.4 | Cell communication;Signal transduction | - | Molecular function unknown | - |
| **LOC650157** | XP_006711976.1 | 650157 | **peptidyl-prolyl cis-trans isomerase A-like** | 5.9 | 2 | 1 | 1 | 9 | 1.94E+08 | **1.49** | 2 | 12.1 | 272 | 29.6 | 8.4 | - | - | - | - |
| **UBALD1** | NP_660296.1 | 124402 | **UBA-like domain-containing protein 1** | 11.3 | 1 | 1 | 1 | 1 | 4.20E+07 | **1.49** | 1 | - | 177 | 18.9 | 6.6 | Biological_process unknown | - | Molecular function unknown | - |
| **ARL6IP1** | NP_055976.1 | 23204 | **ADP-ribosylation factor-like protein 6-interacting protein 1** | 20.2 | 1 | 2 | 2 | 3 | 1.62E+07 | **1.49** | 1 | - | 203 | 23.3 | 9.3 | Transport | Cytoplasm;Plasma membrane | Auxiliary transport protein activity | Plasma membrane |
| **CAPZA2** | NP_006127.1 | 830 | **F-actin-capping protein subunit alpha-2** | 44.8 | 1 | 6 | 7 | 25 | 3.62E+08 | **1.49** | 5 | 23.9 | 286 | 32.9 | 5.9 | Cell growth and/or maintenance | Cytoskeleton | Structural constituent of cytoskeleton | Cytoskeleton |
| **TEX264** | NP_001230656.1 | 51368 | **testis-expressed sequence 264 protein isoform 2** | 5.9 | 2 | 1 | 1 | 1 | 5.93E+07 | **1.49** | 1 | - | 239 | 25.9 | 4.7 | Biological_process unknown | Extracellular | Molecular function unknown | Extracellular |
| **UGGT1** | NP_064505.1 | 56886 | **UDP-glucose:glycoprotein glucosyltransferase 1 precursor** | 34.9 | 1 | 29 | 29 | 77 | 3.16E+08 | **1.49** | 28 | 20.4 | 1555 | 177.1 | 5.6 | Metabolism;Energy pathways | Endoplasmic reticulum;Mitochondrion | Transferase activity | Endoplasmic reticulum |
| **RAB7A** | NP_004628.4 | 7879 | **ras-related protein Rab-7a** | 73.9 | 1 | 13 | 13 | 85 | 1.30E+09 | **1.48** | 18 | 23.9 | 207 | 23.5 | 6.7 | Cell communication;Signal transduction | Endosome;Lysosome;Mitochondrion | GTPase activity | Endosome |
| **AP1M1** | NP_115882.1 | 8907 | **AP-1 complex subunit mu-1 isoform 2** | 41.6 | 2 | 11 | 12 | 28 | 4.19E+08 | **1.48** | 9 | 14.3 | 423 | 48.6 | 7.3 | Cell communication;Signal transduction | Cytoplasm;Golgi apparatus;Endosome | Receptor signaling complex scaffold activity | Golgi apparatus |
| **C14orf142** | NP_115879.2 | 84520 | **uncharacterized protein C14orf142** | 62.0 | 1 | 3 | 3 | 3 | 6.28E+07 | **1.48** | 2 | 38.8 | 100 | 10.9 | 4.3 | Biological_process unknown | - | Molecular function unknown | - |
| **SNRPF** | NP_003086.1 | 6636 | **small nuclear ribonucleoprotein F** | 39.5 | 1 | 3 | 3 | 46 | 1.05E+09 | **1.48** | 4 | 29.1 | 86 | 9.7 | 4.7 | Regulation of nucleobase, nucleoside, nucleotide and nucleic acid metabolism | Nucleus;Cytoplasm | RNA binding | Nucleus |
| **THOC5** | NP_001002879.1 | 8563 | **THO complex subunit 5 homolog** | 15.4 | 1 | 6 | 6 | 9 | 9.26E+07 | **1.48** | 4 | 24.3 | 683 | 78.5 | 6.9 | Biological_process unknown | Nucleus;Cytoplasm | Molecular function unknown | Nucleus |
| **PPAT** | NP_002694.3 | 5471 | **amidophosphoribosyltransferase proprotein** | 24.0 | 1 | 8 | 8 | 12 | 1.34E+08 | **1.48** | 4 | 12.8 | 517 | 57.4 | 6.8 | Metabolism;Energy pathways | Nucleus | Catalytic activity | Nucleus |
| **SEC13** | NP_899195.1 | 6396 | **protein SEC13 homolog isoform 1** | 46.3 | 5 | 7 | 7 | 29 | 1.17E+09 | **1.48** | 5 | 24.9 | 322 | 35.5 | 5.5 | Transport | Endoplasmic reticulum;Nucleus;Cytoplasm;Nuclear membrane | Transporter activity | Endoplasmic reticulum |
| **EDC4** | NP_055144.3 | 23644 | **enhancer of mRNA-decapping protein 4** | 28.2 | 1 | 19 | 19 | 43 | 2.60E+08 | **1.47** | 17 | 27.7 | 1401 | 151.6 | 5.9 | Biological_process unknown | Nucleus;Cytoplasm | Molecular function unknown | Nucleus |
| **SLC25A10** | NP_036272.2 | 1468 | **mitochondrial dicarboxylate carrier isoform 2** | 18.5 | 3 | 3 | 3 | 4 | 3.60E+07 | **1.47** | 1 | - | 287 | 31.3 | 9.5 | Transport | Mitochondrion | Transporter activity | Mitochondrion |
| **OARD1** | NP_659500.1 | 221443 | **O-acetyl-ADP-ribose deacetylase 1** | 16.5 | 1 | 2 | 2 | 4 | 4.48E+07 | **1.47** | 2 | 25.9 | 152 | 17 | 8.3 | Regulation of nucleobase, nucleoside, nucleotide and nucleic acid metabolism | Nucleus | Molecular function unknown | Nucleus |
| **RPIA** | NP_653164.2 | 22934 | **ribose-5-phosphate isomerase** | 6.1 | 1 | 1 | 1 | 2 | 2.43E+08 | **1.47** | 1 | - | 311 | 33.2 | 8.5 | Metabolism;Energy pathways | Endoplasmic reticulum | Isomerase activity | Endoplasmic reticulum |
| **ATG5** | NP_001273040.1 | 9474 | **autophagy protein 5 isoform d** | 9.9 | 3 | 1 | 1 | 1 | 7.20E+07 | **1.47** | 1 | - | 91 | 10.9 | 5.0 | Apoptosis | Cytoskeleton | Structural constituent of cytoskeleton | Cytoskeleton |
| **PEF1** | NP_036524.1 | 553115 | **peflin** | 23.6 | 1 | 3 | 3 | 4 | 2.18E+07 | **1.47** | 2 | 17.3 | 284 | 30.4 | 6.5 | Cell communication;Signal transduction | Cytoplasm | Calcium ion binding | Cytoplasm |

| Gene Symbol | RefSeq_Accession | Entrez Gene ID | Description | Coverage (%) | Proteins | Unique Peptides | # Peptides | PSM | Area | SCC-R /SCC-S | SCC-R /SCC-S (Count) | SCC-R /SCC-S (Variability [%]) | # AAs | MW [kDa] | calc. pI | Biological Process | Localization | Molecular Function | Primary Localization |
|---|---|---|---|---|---|---|---|---|---|---|---|---|---|---|---|---|---|---|---|
| **PDP1** | NP_001155253.1 | 54704 | **pyruvate dehyrogenase phosphatase catalytic subunit 1 isoform 3** | 30.5 | 2 | 8 | 8 | 10 | 1.54E+08 | **1.47** | 4 | 5 | 537 | 61 | 6.7 | Cell communication;Signal transduction | Mitochondrion | Protein serine/threonine phosphatase activity | Mitochondrion |
| **RNF114** | NP_061153.1 | 55905 | **E3 ubiquitin-protein ligase RNF114** | 26.8 | 1 | 3 | 3 | 8 | 2.02E+08 | **1.47** | 4 | 14.5 | 228 | 25.7 | 7.3 | Protein metabolism | - | Ubiquitin-specific protease activity | - |
| **CCDC50** | NP_777568.1 | 152137 | **coiled-coil domain-containing protein 50 short isoform** | 20.6 | 2 | 4 | 4 | 10 | 1.02E+08 | **1.47** | 3 | 0.3 | 306 | 35.8 | 6.7 | Biological_process unknown | Nucleus;Cytoplasm | Molecular function unknown | Nucleus;Cytoplasm |
| **CRYZL1** | NP_665857.2 | 9946 | **quinone oxidoreductase-like protein 1** | 5.7 | 1 | 1 | 1 | 1 | 9.64E+07 | **1.47** | 1 | - | 349 | 38.7 | 5.8 | Metabolism;Energy pathways | - | Oxidoreductase activity | - |
| **MAPK14** | NP_620581.1 | 1432 | **mitogen-activated protein kinase 14 isoform 2** | 24.4 | 4 | 6 | 6 | 16 | 1.07E+08 | **1.47** | 7 | 25.1 | 360 | 41.3 | 5.8 | Cell communication;Signal transduction | Nucleus;Cytoplasm | Protein serine/threonine kinase activity | Cytoplasm |
| **ACBD6** | NP_115736.1 | 84320 | **acyl-CoA-binding domain-containing protein 6** | 4.3 | 1 | 1 | 1 | 2 | 3.76E+07 | **1.47** | 1 | - | 282 | 31.1 | 5.1 | Biological_process unknown | - | Molecular function unknown | - |
| **DVL1** | NP_004412.2 | 1855 | **segment polarity protein dishevelled homolog DVL-1** | 3.7 | 1 | 1 | 1 | 2 | 4.28E+07 | **1.47** | 1 | - | 670 | 72.8 | 7.4 | Cell communication;Signal transduction | Cytoplasm;Plasma membrane | Receptor signaling complex scaffold activity | Cytoplasm |
| **HAUS8** | NP_001011699.1 | 93323 | **HAUS augmin-like complex subunit 8 isoform b** | 14.7 | 2 | 3 | 3 | 9 | 8.24E+07 | **1.47** | 1 | - | 409 | 44.8 | 7.1 | Biological_process unknown;Immune response | - | Molecular function unknown | - |
| **TBC1D9B** | NP_055858.2 | 23061 | **TBC1 domain family member 9B isoform b** | 5.4 | 2 | 4 | 4 | 6 | 1.04E+08 | **1.47** | 3 | 7.9 | 1233 | 138.6 | 5.3 | Biological_process unknown | - | Molecular function unknown | - |
| **ANKFY1** | NP_057460.3 | 51479 | **ankyrin repeat and FYVE domain-containing protein 1 isoform 1** | 27.0 | 2 | 18 | 18 | 38 | 2.00E+08 | **1.47** | 13 | 24.1 | 1170 | 128.4 | 6.1 | Transport | Cytoplasm;Endosome | Transporter activity | Cytoplasm |
| **APIP** | NP_057041.2 | 51074 | **methylthioribulose-1-phosphate dehydratase** | 11.6 | 1 | 2 | 2 | 2 | 3.47E+07 | **1.47** | 1 | - | 242 | 27.1 | 7.1 | Apoptosis | Cytoplasm | Molecular function unknown | Cytoplasm |
| **MAPK1IP1L** | NP_653179.1 | 93487 | **MAPK-interacting and spindle-stabilizing protein-like** | 6.9 | 1 | 1 | 1 | 21 | 1.12E+09 | **1.47** | 2 | 1.6 | 245 | 24.3 | 5.6 | Biological_process unknown | Nucleus | Molecular function unknown | Nucleus |
| **NUCB2** | NP_005004.1 | 4925 | **nucleobindin-2 precursor** | 20.0 | 1 | 5 | 5 | 7 | 1.51E+08 | **1.47** | 1 | - | 420 | 50.2 | 5.1 | Cell communication;Signal transduction | Golgi apparatus;Cytoplasm;Plasma membrane;Extracellular;Nucleus | Calcium ion binding | Golgi apparatus;Nucleus |
| **DPH2** | NP_001375.2 | 1802 | **diphthamide biosynthesis protein 2 isoform a** | 24.1 | 2 | 6 | 6 | 13 | 7.22E+07 | **1.46** | 5 | 21.7 | 489 | 52 | 5.5 | Metabolism;Energy pathways | - | Catalytic activity | - |
| **TMX3** | NP_061895.3 | 54495 | **protein disulfide-isomerase TMX3 precursor** | 8.2 | 1 | 1 | 1 | 2 | 2.62E+08 | **1.46** | 1 | - | 454 | 51.8 | 4.9 | Metabolism;Energy pathways | Integral to membrane | Oxidoreductase activity | Integral to membrane |
| **ADPRHL2** | NP_060295.1 | 54936 | **poly(ADP-ribose) glycohydrolase ARH3** | 49.0 | 1 | 10 | 10 | 18 | 2.16E+08 | **1.46** | 8 | 35.5 | 363 | 38.9 | 5.1 | Metabolism;Energy pathways | - | Hydrolase activity | - |
| **C17orf75** | NP_071739.2 | 64149 | **protein Njmu-R1** | 9.6 | 1 | 2 | 2 | 2 | 1.88E+07 | **1.46** | 2 | 57.5 | 396 | 44.6 | 5.0 | Biological_process unknown | - | Molecular function unknown | - |
| **GDA** | NP_004284.1 | 9615 | **guanine deaminase isoform b** | 34.8 | 3 | 10 | 10 | 19 | 1.51E+08 | **1.46** | 12 | 39.6 | 454 | 51 | 5.7 | Metabolism;Energy pathways | Cytoplasm;Plasma membrane | Deaminase activity | Cytoplasm |
| **MIOS** | NP_061878.3 | 54468 | **WD repeat-containing protein mio** | 4.2 | 1 | 2 | 2 | 2 | 8.77E+07 | **1.46** | 1 | - | 875 | 98.5 | 6.7 | Cell communication;Signal transduction | - | Molecular function unknown | - |
| **TRNT1** | NP_886552.2 | 51095 | **CCA tRNA nucleotidyltransferase 1, mitochondrial** | 22.6 | 1 | 6 | 6 | 15 | 1.23E+08 | **1.46** | 5 | 25.6 | 434 | 50.1 | 8.1 | Metabolism | Mitochondrion | Nucleotidyltransferase activity | Mitochondrion |
| **C5orf51** | NP_787117.3 | 285636 | **UPF0600 protein C5orf51** | 29.9 | 1 | 4 | 4 | 6 | 7.24E+07 | **1.46** | 3 | 9.7 | 294 | 33.6 | 5.3 | Biological_process unknown | - | Molecular function unknown | - |
| **QTRTD1** | NP_078914.1 | 79691 | **queuine tRNA-ribosyltransferase subunit QTRTD1 isoform 1** | 19.8 | 4 | 4 | 4 | 5 | 6.18E+07 | **1.46** | 3 | 21.4 | 415 | 46.7 | 6.8 | Metabolism;Energy pathways | - | Catalytic activity | - |
| **HTATSF1** | NP_001156752.1 | 27336 | **HIV Tat-specific factor 1** | 20.5 | 1 | 9 | 9 | 18 | 2.14E+08 | **1.46** | 4 | 38.2 | 755 | 85.8 | 4.4 | Regulation of nucleobase, nucleoside, nucleotide and nucleic acid metabolism | Nucleus | Transcription factor activity | Nucleus |
| **ADH5** | NP_000662.3 | 128 | **alcohol dehydrogenase class-3** | 31.0 | 1 | 12 | 12 | 33 | 4.30E+08 | **1.46** | 18 | 21.9 | 374 | 39.7 | 7.5 | Metabolism;Energy pathways | Cytoplasm;Nucleus | Oxidoreductase activity | Cytoplasm |
| **FAM210A** | NP_001092271.1 | 125228 | **protein FAM210A** | 8.1 | 1 | 1 | 1 | 1 | 1.58E+07 | **1.46** | 1 | - | 272 | 30.8 | 9.8 | Biological_process unknown | Plasma membrane | Molecular function unknown | Plasma membrane |
| **PPFIA1** | NP_803172.1 | 8500 | **liprin-alpha-1 isoform a** | 21.9 | 14 | 16 | 16 | 31 | 2.51E+08 | **1.46** | 13 | 43.6 | 1185 | 133.9 | 6.3 | Cell growth and/or maintenance | Plasma membrane;Cytoplasm;Nucleus | Cytoskeletal anchoring activity | Cytoplasm |
| **TACC2** | NP_001278808.1 | 10579 | **transforming acidic coiled-coil-containing protein 2 isoform h** | 8.7 | 8 | 2 | 2 | 2 | 5.87E+06 | **1.46** | 1 | - | 575 | 64.6 | 5.3 | Cell growth and/or maintenance | Centrosome;Cytoplasm | Molecular function unknown | Centrosome |
| **TOR1A** | NP_000104.1 | 1861 | **torsin-1A precursor** | 8.1 | 1 | 2 | 2 | 5 | 2.85E+08 | **1.46** | 1 | - | 332 | 37.8 | 7.0 | Protein metabolism | Cytoplasm;Endoplasmic reticulum;Nucleus | Chaperone activity | Endoplasmic reticulum |
| **CNBP** | NP_001120668.1 | 7555 | **cellular nucleic acid-binding protein isoform 6** | 29.4 | 6 | 4 | 4 | 9 | 2.56E+08 | **1.46** | 5 | 9.3 | 170 | 18.7 | 7.7 | Regulation of nucleobase, nucleoside, nucleotide and nucleic acid metabolism | Cytoplasm;Endoplasmic reticulum | RNA binding | Cytoplasm |
| **RAB3GAP1** | NP_036365.1 | 22930 | **rab3 GTPase-activating protein catalytic subunit isoform 2** | 20.5 | 2 | 14 | 14 | 22 | 1.77E+08 | **1.46** | 12 | 30.7 | 981 | 110.5 | 5.6 | Cell communication;Signal transduction | Nucleus | GTPase activator activity | Nucleus |
| **SRSF4** | NP_005617.2 | 6429 | **serine/arginine-rich splicing factor 4** | 17.6 | 1 | 8 | 11 | 38 | 4.36E+08 | **1.46** | 8 | 19.5 | 494 | 56.6 | 11.5 | Regulation of nucleobase, nucleoside, nucleotide and nucleic acid metabolism | Nucleus | RNA binding | Nucleus |
| **SLC25A24** | NP_998816.1 | 29957 | **calcium-binding mitochondrial carrier protein SCaMC-1 isoform 2** | 33.2 | 2 | 10 | 10 | 22 | 1.64E+08 | **1.46** | 12 | 6 | 458 | 51.3 | 5.9 | Transport | Mitochondrial membrane | Transporter activity | Mitochondrial membrane |
| **TCEB2** | NP_009039.1 | 6923 | **transcription elongation factor B polypeptide 2 isoform a** | 99.2 | 2 | 10 | 10 | 35 | 1.55E+09 | **1.45** | 18 | 37.1 | 118 | 13.1 | 4.9 | Regulation of nucleobase, nucleoside, nucleotide and nucleic acid metabolism | Nucleus | Transcription regulator activity | Nucleus |
| **PGM3** | NP_056414.1 | 5238 | **phosphoacetylglucosamine mutase isoform 2** | 20.1 | 4 | 5 | 5 | 13 | 1.41E+08 | **1.45** | 7 | 13.9 | 542 | 59.8 | 6.3 | Metabolism;Energy pathways | Cytoplasm | Catalytic activity | Cytoplasm |
| **PTGR1** | NP_001139580.1 | 22949 | **prostaglandin reductase 1 isoform 1** | 50.2 | 2 | 12 | 12 | 46 | 5.75E+08 | **1.45** | 19 | 7.4 | 329 | 35.8 | 8.3 | Metabolism;Energy pathways | Cytoplasm | Catalytic activity | Cytoplasm |
| **SNRNP40** | NP_004805.2 | 9410 | **U5 small nuclear ribonucleoprotein 40 kDa protein** | 37.0 | 1 | 8 | 8 | 18 | 2.14E+08 | **1.45** | 9 | 37.3 | 357 | 39.3 | 8.1 | Regulation of nucleobase, nucleoside, nucleotide and nucleic acid metabolism | Nucleus | Ribonucleoprotein | Nucleus |
| **HK1** | NP_000179.2 | 3098 | **hexokinase-1 isoform HKI** | 45.9 | 5 | 32 | 34 | 124 | 5.56E+08 | **1.45** | 49 | 22.1 | 917 | 102.4 | 6.8 | Metabolism;Energy pathways | Cytoplasm;Mitochondrial membrane;Plasma membrane;Mitochondrion | Catalytic activity | Cytoplasm |

Supplementary Table S4. List of all proteins identified in SCC-S and SCC-R cells using SequestHT and Mascot search algorithms and their gene ontology annotation

| Gene Symbol | RefSeq_Accession | Entrez Gene ID | Description | Coverage (%) | Proteins | Unique Peptides | # Peptides | PSM | Area | SCC-R /SCC-S | SCC-R /SCC-S (Count) | SCC-R /SCC-S (Variability [%]) | # AAs | MW [kDa] | calc. pI | Biological Process | Localization | Molecular Function | Primary Localization |
|---|---|---|---|---|---|---|---|---|---|---|---|---|---|---|---|---|---|---|---|
| **ZNF622** | NP_219482.1 | 90441 | **zinc finger protein 622** | 10.3 | 1 | 2 | 2 | 3 | 1.35E+08 | **1.45** | 2 | 80.5 | 477 | 54.2 | 6.2 | Cell communication;Signal transduction | Nucleus;Cytoplasm;Nucleolus | Transcription regulator activity | Nucleus |
| **PPIB** | NP_000933.1 | 5479 | **peptidyl-prolyl cis-trans isomerase B precursor** | 63.4 | 1 | 20 | 20 | 158 | 2.52E+09 | **1.45** | 46 | 19.7 | 216 | 23.7 | 9.4 | Protein metabolism | Endoplasmic reticulum;Extracellular;Plasma membrane;Nucleus;Golgi apparatus;Nucleolus;Mitochondrion;Cytosol | Chaperone activity | Endoplasmic reticulum |
| **PSMG1** | NP_982257.1 | 8624 | **proteasome assembly chaperone 1 isoform b** | 24.0 | 3 | 6 | 6 | 15 | 1.79E+08 | **1.45** | 5 | 31.2 | 267 | 30.3 | 7.8 | Cell communication;Signal transduction | Plasma membrane | Molecular function unknown | Plasma membrane |
| **ASAH1** | NP_001120977.1 | 427 | **acid ceramidase isoform c** | 8.7 | 3 | 2 | 2 | 3 | 1.95E+07 | **1.45** | 1 | - | 389 | 44 | 7.6 | Metabolism;Energy pathways | Lysosome | Hydrolase activity | Lysosome |
| **CSTF2** | NP_001316.1 | 1478 | **cleavage stimulation factor subunit 2** | 22.9 | 1 | 5 | 7 | 19 | 2.12E+08 | **1.45** | 6 | 25 | 577 | 60.9 | 6.8 | Regulation of nucleobase, nucleoside, nucleotide and nucleic acid metabolism | Nucleus | RNA binding | Nucleus |
| **S100A13** | NP_001019384.1 | 6284 | **protein S100-A13** | 54.1 | 1 | 7 | 7 | 25 | 7.60E+08 | **1.45** | 8 | 7.1 | 98 | 11.5 | 6.2 | Cell communication;Signal transduction | Nucleus;Cytoplasm;Nucleolus;Perinuclear region | Calcium ion binding | Nucleus |
| **SOD1** | NP_000445.1 | 6647 | **superoxide dismutase [Cu-Zn]** | 90.9 | 1 | 8 | 8 | 70 | 2.94E+09 | **1.45** | 20 | 10.5 | 154 | 15.9 | 6.1 | Metabolism;Energy pathways | Peroxisome;Nucleus;Extracellular;Cytoplasm | Superoxide dismutase activity | Cytoplasm |
| **MINA** | NP_116167.3 | 84864 | **bifunctional lysine-specific demethylase and histidyl-hydroxylase MINA isoform b** | 15.3 | 2 | 5 | 5 | 8 | 5.93E+07 | **1.45** | 4 | 37.9 | 464 | 52.6 | 6.7 | Cell growth and/or maintenance | Nucleus;Nucleolus | Molecular function unknown | Nucleus |
| **PICALM** | NP_001008660.1 | 8301 | **phosphatidylinositol-binding clathrin assembly protein isoform 2** | 31.5 | 8 | 12 | 12 | 33 | 5.59E+08 | **1.45** | 18 | 23.8 | 610 | 66.4 | 8.8 | Regulation of endocytosis | Plasma membrane;Clathrin-coated vesicle;Golgi apparatus | Clathrin binding | Plasma membrane |
| **PBK** | NP_060962.2 | 55872 | **lymphokine-activated killer T-cell-originated protein kinase isoform 1** | 3.4 | 2 | 1 | 1 | 1 | 4.29E+07 | **1.44** | 1 | - | 322 | 36.1 | 5.1 | Cell communication;Signal transduction | Cytoplasm;Nucleus | Protein serine/threonine kinase activity | Cytoplasm |
| **ATP6V1C1** | NP_001686.1 | 528 | **V-type proton ATPase subunit C 1** | 16.8 | 1 | 4 | 4 | 5 | 1.71E+08 | **1.44** | 3 | 3.8 | 382 | 43.9 | 7.5 | Transport | - | ATPase activity | - |
| **NUPL1** | NP_001008564.1 | 9818 | **nucleoporin p58/p45 isoform b** | 8.4 | 2 | 3 | 3 | 6 | 1.76E+08 | **1.44** | 3 | 48.9 | 587 | 59.7 | 9.3 | Biological_process unknown | Nucleus;Mitochondrion | Molecular function unknown | Nucleus |
| **SNX3** | NP_003786.1 | 8724 | **sorting nexin-3 isoform a** | 53.7 | 2 | 5 | 7 | 14 | 5.74E+08 | **1.44** | 5 | 5.7 | 162 | 18.8 | 8.7 | Intracellular signaling cascade;Protein transport | Endosome;Cytoplasm;Integral to membrane | Protein transporter activity | Endosome |
| **LRRC8A** | NP_001120716.1 | 56262 | **leucine-rich repeat-containing protein 8A** | 1.9 | 1 | 1 | 1 | 1 | 3.57E+07 | **1.44** | 1 | - | 810 | 94.1 | 7.9 | Biological_process unknown;Immune response | Integral to membrane | Molecular function unknown | Integral to membrane |
| **RAB31** | NP_006859.2 | 11031 | **ras-related protein Rab-31** | 10.8 | 1 | 1 | 1 | 1 | 7.78E+06 | **1.44** | 1 | - | 195 | 21.7 | 7.1 | Cell communication;Signal transduction | Plasma membrane;Mitochondrion;Nucleus;Cytoplasm | GTPase activity | Plasma membrane |
| **AKAP11** | NP_057332.1 | 11215 | **A-kinase anchor protein 11** | 1.3 | 1 | 1 | 1 | 1 | 1.78E+07 | **1.44** | 1 | - | 1901 | 210.4 | 5.4 | Cell communication;Signal transduction | Peroxisome;Centrosome;Cytoplasm;Nucleus | Cytoskeletal anchoring activity | Centrosome |
| **C2orf49** | NP_076998.1 | 79074 | **ashwin isoform 1** | 25.9 | 2 | 3 | 3 | 4 | 8.14E+07 | **1.44** | 4 | 10.6 | 232 | 25.8 | 9.7 | Biological_process unknown | Nucleus | Molecular function unknown | Nucleus |
| **DTD1** | NP_543010.3 | 92675 | **D-tyrosyl-tRNA(Tyr) deacylase 1** | 7.7 | 1 | 1 | 1 | 3 | 2.46E+08 | **1.44** | 1 | - | 209 | 23.4 | 8.2 | Metabolism;Energy pathways | Nucleus;Cytoplasm | ATPase activity | Nucleus |
| **SMS** | NP_004586.2 | 6611 | **spermine synthase isoform 1** | 68.3 | 2 | 13 | 13 | 37 | 2.50E+08 | **1.44** | 9 | 30 | 366 | 41.2 | 5.0 | Metabolism;Energy pathways | - | Transferase activity | - |
| **EIF4H** | NP_114381.1 | 7458 | **eukaryotic translation initiation factor 4H isoform 2** | 66.2 | 2 | 15 | 15 | 97 | 4.02E+09 | **1.44** | 23 | 29.5 | 228 | 25.2 | 8.1 | Protein metabolism | Cytoplasm | Translation regulator activity | Cytoplasm |
| **THOC3** | NP_115737.1 | 84321 | **THO complex subunit 3** | 33.1 | 1 | 5 | 5 | 15 | 2.11E+08 | **1.44** | 6 | 11.3 | 351 | 38.7 | 6.1 | Regulation of nucleobase, nucleoside, nucleotide and nucleic acid metabolism | Nucleus | RNA binding | Nucleus |
| **TRIM32** | NP_001093149.1 | 22954 | **E3 ubiquitin-protein ligase TRIM32** | 8.9 | 1 | 3 | 3 | 4 | 1.42E+07 | **1.44** | 2 | 3.3 | 653 | 71.9 | 7.0 | Regulation of nucleobase, nucleoside, nucleotide and nucleic acid metabolism | Nucleus | Transcription regulator activity | Nucleus |
| **ATP6V1G1** | NP_004879.1 | 9550 | **V-type proton ATPase subunit G 1** | 45.8 | 1 | 4 | 4 | 15 | 3.07E+08 | **1.44** | 6 | 13.6 | 118 | 13.7 | 8.8 | Metabolism;Energy pathways | Lysosome | Transporter activity | Lysosome |
| **BCAP29** | NP_061332.2 | 55973 | **B-cell receptor-associated protein 29 isoform b** | 6.6 | 2 | 2 | 2 | 2 | 6.10E+07 | **1.44** | 1 | - | 241 | 28.3 | 9.5 | Biological_process unknown | Endoplasmic reticulum membrane | Molecular function unknown | Endoplasmic reticulum membrane |
| **GSPT1** | NP_001123478.1 | 2935 | **eukaryotic peptide chain release factor GTP-binding subunit ERF3A isoform 2** | 38.7 | 3 | 10 | 16 | 54 | 7.90E+08 | **1.44** | 15 | 21.9 | 636 | 68.5 | 5.3 | Cell communication;Signal transduction | Cytoplasm;Endoplasmic reticulum | GTPase activity | Cytoplasm |
| **SDF4** | NP_057260.2 | 51150 | **45 kDa calcium-binding protein isoform 2 precursor** | 29.3 | 2 | 5 | 5 | 9 | 9.31E+07 | **1.44** | 2 | 3.5 | 362 | 41.8 | 4.9 | Cell communication;Signal transduction | Golgi lumen | Calcium ion binding | Golgi lumen |
| **LAMTOR3** | NP_068805.1 | 8649 | **ragulator complex protein LAMTOR3 isoform 1** | 30.7 | 2 | 2 | 2 | 4 | 9.37E+07 | **1.44** | 1 | - | 124 | 13.6 | 7.3 | Cell communication;Signal transduction | Endosome;Cytoplasm | Receptor signaling complex scaffold activity | Endosome |
| **DBNL** | NP_054782.2 | 28988 | **drebrin-like protein isoform a** | 49.2 | 3 | 1 | 17 | 109 | 1.69E+09 | **1.43** | 2 | 6 | 431 | 48.3 | 5.1 | Signal transduction | Actin cytoskeleton;Dendrite;Cytoplasm | Kinase regulator activity | Actin cytoskeleton |
| **PLCXD2** | NP_695000.1 | 257068 | **PI-PLC X domain-containing protein 2 isoform b** | 1.6 | 3 | 1 | 1 | 2 | 8.36E+07 | **1.43** | 1 | - | 304 | 34.7 | 9.6 | Biological_process unknown | - | Molecular function unknown | - |
| **KPNA6** | NP_036448.1 | 23633 | **importin subunit alpha-7** | 56.2 | 2 | 16 | 19 | 64 | 6.08E+08 | **1.43** | 12 | 12.4 | 536 | 60 | 5.0 | Transport | Nucleus;Cytoplasm;Centrosome | Receptor signaling complex scaffold activity | Nucleus |
| **LIMD1** | NP_055055.1 | 8994 | **LIM domain-containing protein 1** | 23.4 | 1 | 7 | 7 | 10 | 8.78E+07 | **1.43** | 7 | 24.3 | 676 | 72.1 | 6.7 | Regulation of nucleobase, nucleoside, nucleotide and nucleic acid metabolism | Nucleus;Plasma membrane | Transcription regulator activity | Nucleus;Plasma membrane |
| **POMP** | NP_057016.1 | 51371 | **proteasome maturation protein** | 30.5 | 1 | 2 | 2 | 2 | 9.43E+07 | **1.43** | 1 | - | 141 | 15.8 | 5.1 | Biological_process unknown | - | Molecular function unknown | - |
| **PRKACA** | NP_002721.1 | 5566 | **cAMP-dependent protein kinase catalytic subunit alpha isoform 1** | 26.5 | 10 | 3 | 7 | 17 | 1.43E+08 | **1.43** | 3 | 35.4 | 351 | 40.6 | 8.8 | Signal transduction | Golgi apparatus;Nucleus;Cytoplasm | Protein serine/threonine kinase activity | Golgi apparatus;Nucleus;Cytoplasm |
| **SYNCRIP** | NP_006363.4 | 10492 | **heterogeneous nuclear ribonucleoprotein Q isoform 1** | 55.7 | 2 | 2 | 30 | 176 | 3.33E+09 | **1.43** | 2 | 41.7 | 623 | 69.6 | 8.6 | Regulation of nucleobase, nucleoside, nucleotide and nucleic acid metabolism | Cytoplasm;Nucleus;Endoplasmic reticulum;Ribosome;Nucleolus | RNA binding | Cytoplasm |
| **SHCBP1** | NP_079021.3 | 79801 | **SHC SH2 domain-binding protein 1** | 1.2 | 1 | 1 | 1 | 1 | 1.62E+07 | **1.43** | 1 | - | 672 | 75.6 | 4.8 | Biological_process unknown | - | Molecular function unknown | - |
| **CARHSP1** | NP_055131.2 | 23589 | **calcium-regulated heat stable protein 1** | 53.1 | 1 | 3 | 3 | 24 | 7.82E+08 | **1.43** | 2 | 6.8 | 147 | 15.9 | 8.2 | Cell communication;Signal transduction | Cytoplasm;Nucleus | Calcium ion binding | Cytoplasm |
| **MRGBP** | NP_060740.1 | 55257 | **MRG/MORF4L-binding protein** | 13.7 | 1 | 2 | 2 | 5 | 2.18E+07 | **1.43** | 2 | 3.9 | 204 | 22.4 | 5.8 | Regulation of nucleobase, nucleoside, nucleotide and nucleic acid metabolism | Nucleus | Transcription regulator activity | Nucleus |

| Gene Symbol | RefSeq_Accession | Entrez Gene ID | Description | Coverage (%) | Proteins | Unique Peptides | # Peptides | PSM | Area | SCC-R /SCC-S | SCC-R /SCC-S (Count) | SCC-R /SCC-S (Variability [%]) | # AAs | MW [kDa] | calc. pI | Biological Process | Localization | Molecular Function | Primary Localization |
|---|---|---|---|---|---|---|---|---|---|---|---|---|---|---|---|---|---|---|---|
| **PGK1** | NP_000282.1 | 5230 | **phosphoglycerate kinase 1** | 83.5 | 2 | 40 | 40 | 622 | 8.42E+09 | **1.43** | 118 | 19.2 | 417 | 44.6 | 8.1 | Metabolism;Energy pathways | Cytoplasmic vesicle;Cytoplasm;Nucleus;Mitochondrion | Catalytic activity | Cytoplasm |
| **NDUFB8** | NP_001271297.1 | 4714 | **NADH dehydrogenase [ubiquinone] 1 beta subcomplex subunit 8, mitochondrial isoform 3** | 14.8 | 3 | 1 | 1 | 5 | 3.67E+08 | **1.43** | 1 | - | 155 | 18.5 | 5.5 | Metabolism;Energy pathways | Mitochondrion | Oxidoreductase activity | Mitochondrion |
| **C12orf57** | NP_612434.1 | 113246 | **protein C10** | 50.8 | 1 | 4 | 4 | 16 | 8.94E+07 | **1.43** | 4 | 15.8 | 126 | 13.2 | 5.1 | Biological_process unknown | - | Molecular function unknown | - |
| **CWC22** | NP_065994.1 | 57703 | **pre-mRNA-splicing factor CWC22 homolog** | 2.1 | 1 | 1 | 1 | 1 | 2.66E+06 | **1.43** | 1 | - | 908 | 105.4 | 7.0 | Biological_process unknown | Nucleus;Cytoplasm | Molecular function unknown | Nucleus;Cytoplasm |
| **MCCC1** | NP_064551.3 | 56922 | **methylcrotonoyl-CoA carboxylase subunit alpha, mitochondrial precursor** | 10.9 | 1 | 4 | 4 | 4 | 1.04E+08 | **1.43** | 1 | - | 725 | 80.4 | 7.8 | Metabolism;Energy pathways | Mitochondrion | Ligase activity | Mitochondrion |
| **NDUFV1** | NP_001159574.1 | 4723 | **NADH dehydrogenase [ubiquinone] flavoprotein 1, mitochondrial isoform 2 precursor** | 42.9 | 2 | 11 | 11 | 20 | 1.23E+08 | **1.43** | 6 | 38.1 | 455 | 49.8 | 8.2 | Metabolism;Energy pathways | Mitochondrion | Oxidoreductase activity | Mitochondrion |
| **UBXN7** | NP_056377.1 | 26043 | **UBX domain-containing protein 7** | 22.7 | 1 | 6 | 6 | 9 | 9.03E+07 | **1.43** | 3 | 49.3 | 489 | 54.8 | 5.2 | Biological_process unknown | - | Molecular function unknown | - |
| **RBM8A** | NP_005096.1 | 9939 | **RNA-binding protein 8A** | 46.6 | 1 | 7 | 7 | 47 | 7.25E+08 | **1.43** | 11 | 30 | 174 | 19.9 | 5.7 | Regulation of nucleobase, nucleoside, nucleotide and nucleic acid metabolism | Nucleus;Cytoplasm;Nucleolus | RNA binding | Nucleus;Cytoplasm |
| **TP53BP1** | NP_005648.1 | 7158 | **tumor suppressor p53-binding protein 1 isoform 3** | 21.5 | 2 | 25 | 25 | 38 | 2.16E+08 | **1.43** | 14 | 24.8 | 1972 | 213.4 | 4.7 | Regulation of nucleobase, nucleoside, nucleotide and nucleic acid metabolism | Nucleus;Cytoplasm | Transcription regulator activity | Nucleus |
| **FAF1** | NP_008982.1 | 11124 | **FAS-associated factor 1** | 11.1 | 1 | 4 | 4 | 10 | 8.22E+07 | **1.43** | 7 | 35.9 | 650 | 73.9 | 4.9 | Apoptosis | Nucleus;Cytoplasm;Perinuclear region | Receptor signaling complex scaffold activity | Cytoplasm |
| **PURB** | NP_150093.1 | 5814 | **transcriptional activator protein Pur-beta** | 44.2 | 1 | 7 | 7 | 22 | 2.20E+08 | **1.43** | 7 | 34.3 | 312 | 33.2 | 5.4 | Regulation of nucleobase, nucleoside, nucleotide and nucleic acid metabolism | Nucleus | Transcription regulator activity | Nucleus |
| **AAK1** | NP_055726.3 | 22848 | **AP2-associated protein kinase 1** | 9.4 | 1 | 3 | 3 | 4 | 2.49E+08 | **1.43** | 1 | - | 961 | 103.8 | 6.5 | Transport | Cytoskeleton;Nucleus;Cytoplasm | Cytoskeletal protein binding | Cytoskeleton |
| **ACSL4** | NP_004449.1 | 2182 | **long-chain-fatty-acid--CoA ligase 4 isoform 1** | 37.2 | 2 | 14 | 17 | 32 | 4.46E+08 | **1.43** | 9 | 23.6 | 670 | 74.4 | 8.0 | Metabolism;Energy pathways | Microsome;Mitochondrion | Ligase activity | Microsome |
| **KLC2** | NP_073733.1 | 64837 | **kinesin light chain 2 isoform 1** | 30.2 | 6 | 5 | 13 | 38 | 2.48E+08 | **1.42** | 3 | 25.2 | 622 | 68.9 | 7.2 | Cell growth and/or maintenance | Cytoplasm;Microsome;Cytosol;Perinuclear vesicle;Cell junction;Golgi apparatus;Nucleus | Motor activity | Cytoplasm |
| **MDH2** | NP_005909.2 | 4191 | **malate dehydrogenase, mitochondrial isoform 1 precursor** | 75.7 | 3 | 21 | 21 | 350 | 7.23E+09 | **1.42** | 65 | 18.7 | 338 | 35.5 | 8.7 | Metabolism;Energy pathways | Mitochondrion;Nucleolus;Extracellular | Catalytic activity | Mitochondrion |
| **UBE2H** | NP_003335.1 | 7328 | **ubiquitin-conjugating enzyme E2 H isoform 1** | 13.1 | 2 | 2 | 2 | 2 | 5.07E+07 | **1.42** | 1 | - | 183 | 20.6 | 4.7 | Protein metabolism | Nucleus | Ubiquitin-specific protease activity | Nucleus |
| **SUMF2** | NP_056226.2 | 25870 | **sulfatase-modifying factor 2 isoform b precursor** | 38.8 | 5 | 8 | 8 | 12 | 2.85E+08 | **1.42** | 6 | 11.6 | 320 | 35.9 | 9.2 | Protein metabolism | Endoplasmic reticulum | Molecular function unknown | Endoplasmic reticulum |
| **NUDT21** | NP_008937.1 | 11051 | **cleavage and polyadenylation specificity factor subunit 5** | 55.1 | 1 | 8 | 8 | 21 | 2.84E+08 | **1.42** | 9 | 31.1 | 227 | 26.2 | 8.8 | Regulation of nucleobase, nucleoside, nucleotide and nucleic acid metabolism | Nucleus;Nucleolus | RNA binding | Nucleus |
| **PRPF38B** | NP_060531.2 | 55119 | **pre-mRNA-splicing factor 38B** | 7.5 | 1 | 1 | 1 | 1 | 1.13E+08 | **1.42** | 1 | - | 546 | 64.4 | 10.5 | Regulation of nucleobase, nucleoside, nucleotide and nucleic acid metabolism | - | RNA binding | - |
| **AP2B1** | NP_001273.1 | 163 | **AP-2 complex subunit beta isoform b** | 37.1 | 2 | 15 | 28 | 96 | 7.63E+08 | **1.42** | 20 | 35.9 | 937 | 104.5 | 5.4 | Biological_process unknown;Transport | Plasma membrane;Mitochondrion | Molecular function unknown;Transporter activity | Plasma membrane |
| **HN1L** | NP_653171.1 | 90861 | **hematological and neurological expressed 1-like protein** | 61.6 | 1 | 8 | 8 | 35 | 4.86E+08 | **1.42** | 13 | 43.8 | 190 | 20.1 | 9.3 | Regulation of nucleobase, nucleoside, nucleotide and nucleic acid metabolism | Nucleus;Cytoplasm | Transcription regulator activity | Nucleus |
| **DENND4C** | NP_060395.5 | 55667 | **DENN domain-containing protein 4C** | 2.5 | 1 | 2 | 2 | 3 | 1.61E+07 | **1.42** | 2 | 15.6 | 1909 | 212.6 | 6.8 | Biological_process unknown | Nucleus | Molecular function unknown | Nucleus |
| **FGFR1OP** | NP_001265619.1 | 11116 | **FGFR1 oncogene partner isoform c** | 9.7 | 3 | 2 | 2 | 3 | 6.06E+07 | **1.42** | 2 | 22 | 351 | 38.1 | 4.7 | Cell growth and/or maintenance | - | Molecular function unknown | - |
| **RTCA** | NP_003720.1 | 8634 | **RNA 3'-terminal phosphate cyclase isoform b** | 33.1 | 2 | 6 | 6 | 15 | 5.50E+07 | **1.42** | 4 | 7.5 | 366 | 39.3 | 7.9 | Regulation of nucleobase, nucleoside, nucleotide and nucleic acid metabolism | Nucleus;Cytoplasm | RNA binding | Nucleus |
| **GGA3** | NP_619525.1 | 23163 | **ADP-ribosylation factor-binding protein GGA3 isoform long** | 9.7 | 5 | 3 | 3 | 4 | 6.95E+06 | **1.42** | 1 | - | 723 | 78.3 | 5.6 | Transport | Golgi apparatus;Cytoplasmic vesicle;Cytoplasm;Golgi membrane;Cytosol | Transporter activity | Golgi apparatus |
| **GOPC** | NP_001017408.1 | 57120 | **Golgi-associated PDZ and coiled-coil motif-containing protein isoform b** | 3.5 | 2 | 1 | 1 | 1 | 9.74E+06 | **1.41** | 1 | - | 454 | 49.7 | 6.1 | Biological_process unknown | Golgi apparatus;Cytoplasm;Plasma membrane | Receptor signaling complex scaffold activity | Golgi apparatus |
| **PFN1** | NP_005013.1 | 5216 | **profilin-1** | 96.4 | 1 | 20 | 20 | 496 | 1.07E+10 | **1.41** | 91 | 13.6 | 140 | 15 | 8.3 | Cell growth and/or maintenance | Cytoplasm;Extracellular | Cytoskeletal protein binding | Cytoplasm |
| **PSMG2** | NP_671692.1 | 56984 | **proteasome assembly chaperone 2 isoform 2** | 14.2 | 2 | 3 | 3 | 4 | 1.08E+08 | **1.41** | 2 | 3.2 | 233 | 26.3 | 8.0 | Cell communication;Signal transduction | Nucleus | Molecular function unknown | Nucleus |
| **TUBGCP2** | NP_006650.1 | 10844 | **gamma-tubulin complex component 2 isoform 2** | 11.2 | 3 | 6 | 6 | 11 | 7.47E+07 | **1.41** | 4 | 34.3 | 902 | 102.5 | 6.8 | Cell growth and/or maintenance | Centrosome;Cytoplasm;Microtubule | Cytoskeletal protein binding | Centrosome |
| **ARFGEF2** | NP_006411.2 | 10564 | **brefeldin A-inhibited guanine nucleotide-exchange protein 2** | 4.0 | 1 | 1 | 5 | 6 | 8.38E+07 | **1.41** | 1 | - | 1785 | 201.9 | 6.3 | Cell communication;Signal transduction | Cytoplasm;Endoplasmic reticulum;Golgi apparatus;Perinuclear region;Nucleus;Microtubule;Golgi vesicle | Guanyl-nucleotide exchange factor activity | Cytoplasm |
| **FAM114A2** | NP_061161.2 | 10827 | **protein FAM114A2** | 15.8 | 1 | 4 | 4 | 8 | 9.95E+07 | **1.41** | 6 | 22.2 | 505 | 55.4 | 4.9 | Biological_process unknown | - | Molecular function unknown | - |
| **ARPC5** | NP_005708.1 | 10092 | **actin-related protein 2/3 complex subunit 5 isoform 1** | 66.9 | 2 | 6 | 6 | 35 | 7.10E+08 | **1.41** | 10 | 15.9 | 151 | 16.3 | 5.7 | Cell growth and/or maintenance | Cytoskeleton | Cytoskeletal protein binding | Cytoskeleton |
| **CCDC148** | NP_620158.3 | 130940 | **coiled-coil domain-containing protein 148 isoform 1** | 1.0 | 1 | 1 | 1 | 1 | 2.61E+07 | **1.41** | 1 | - | 591 | 71 | 8.9 | Biological_process unknown | - | Molecular function unknown | - |
| **PRDX1** | NP_002565.1 | 5052 | **peroxiredoxin-1** | 89.5 | 1 | 15 | 19 | 457 | 1.10E+10 | **1.41** | 66 | 20.2 | 199 | 22.1 | 8.1 | Metabolism;Energy pathways | Cytoplasm;Nucleus;Nucleolus;Mitochondrion | Peroxidase activity | Cytoplasm |
| **C7orf50** | NP_001127867.1 | 84310 | **uncharacterized protein C7orf50** | 35.6 | 1 | 3 | 3 | 6 | 2.10E+08 | **1.41** | 2 | 45.9 | 194 | 22.1 | 9.6 | Biological_process unknown | Nucleus | Molecular function unknown | Nucleus |
| **COMMD4** | NP_060298.2 | 54939 | **COMM domain-containing protein 4 isoform 1** | 30.2 | 4 | 3 | 3 | 8 | 3.73E+07 | **1.41** | 2 | 79.1 | 199 | 21.8 | 7.3 | Biological_process unknown | - | Molecular function unknown | - |
| **ARMC1** | NP_060590.1 | 55156 | **armadillo repeat-containing protein 1 isoform 1** | 32.6 | 2 | 4 | 4 | 14 | 1.19E+08 | **1.41** | 8 | 68.8 | 282 | 31.3 | 5.7 | Biological_process unknown | - | Molecular function unknown | - |

| Gene Symbol | RefSeq_Accession | Entrez Gene ID | Description | Coverage (%) | Proteins | Unique Peptides | # Peptides | PSM | Area | SCC-R /SCC-S | SCC-R /SCC-S (Count) | SCC-R /SCC-S (Variability [%]) | # AAs | MW [kDa] | calc. pI | Biological Process | Localization | Molecular Function | Primary Localization |
|---|---|---|---|---|---|---|---|---|---|---|---|---|---|---|---|---|---|---|---|
| **GMPS** | NP_003866.1 | 8833 | **GMP synthase [glutamine-hydrolyzing]** | 62.2 | 1 | 30 | 30 | 96 | 1.04E+09 | **1.41** | 41 | 22.3 | 693 | 76.7 | 6.9 | Metabolism;Energy pathways | - | Amidinotransferase activity | - |
| **OPA1** | NP_056375.2 | 4976 | **dynamin-like 120 kDa protein, mitochondrial isoform 1** | 35.6 | 8 | 23 | 23 | 40 | 2.51E+08 | **1.41** | 16 | 13.6 | 960 | 111.6 | 7.9 | Cell communication;Signal transduction | Mitochondrion | GTPase activity | Mitochondrion |
| **TFG** | NP_001182408.1 | 10342 | **protein TFG isoform 2** | 35.9 | 2 | 10 | 10 | 45 | 1.09E+09 | **1.41** | 12 | 17.5 | 396 | 43 | 5.2 | Signal transduction | Cytoplasm | Receptor regulator activity | Cytoplasm |
| **BID** | NP_001187.1 | 637 | **BH3-interacting domain death agonist isoform 2** | 52.8 | 3 | 8 | 8 | 63 | 2.91E+08 | **1.41** | 16 | 40 | 195 | 22 | 5.4 | Apoptosis | Mitochondrion;Cytoplasm;Cytosol;Membrane fraction;Lysosome | Receptor binding | Mitochondrion |
| **EIF2B5** | NP_003898.2 | 8893 | **translation initiation factor eIF-2B subunit epsilon** | 10.4 | 1 | 4 | 4 | 9 | 6.46E+07 | **1.41** | 1 | - | 721 | 80.3 | 5.1 | Protein metabolism | Cytoplasm;Cytosol | Translation regulator activity | Cytoplasm |
| **NDUFB4** | NP_001161803.1 | 4710 | **NADH dehydrogenase [ubiquinone] 1 beta subcomplex subunit 4 isoform 2** | 25.8 | 2 | 2 | 2 | 9 | 2.04E+08 | **1.41** | 3 | 2.3 | 120 | 14 | 9.8 | Metabolism;Energy pathways | Integral to membrane;Mitochondrion | Oxidoreductase activity | Integral to membrane |
| **ABRACL** | NP_067066.1 | 58527 | **costars family protein ABRACL** | 70.4 | 1 | 4 | 4 | 26 | 3.90E+08 | **1.41** | 2 | 13.2 | 81 | 9.1 | 6.3 | Biological_process unknown | - | Molecular function unknown | - |
| **INTS2** | NP_065799.1 | 57508 | **integrator complex subunit 2** | 3.5 | 1 | 2 | 2 | 2 | 3.40E+06 | **1.40** | 1 | - | 1204 | 134.3 | 6.1 | Biological_process unknown | - | Molecular function unknown | - |
| **MET** | NP_000236.2 | 4233 | **hepatocyte growth factor receptor isoform b precursor** | 1.4 | 2 | 1 | 1 | 1 | 1.31E+08 | **1.40** | 1 | - | 1390 | 155.4 | 7.3 | Cell communication;Signal transduction | Plasma membrane;Cytoplasm | Transmembrane receptor protein tyrosine kinase activity | Plasma membrane |
| **PFKP** | NP_002618.1 | 5214 | **6-phosphofructokinase type C isoform 1** | 49.0 | 2 | 32 | 34 | 173 | 9.95E+08 | **1.40** | 37 | 20.8 | 784 | 85.5 | 7.6 | Metabolism;Energy pathways | Cytoplasm | Catalytic activity | Cytoplasm |
| **TRIP13** | NP_004228.1 | 9319 | **pachytene checkpoint protein 2 homolog isoform 1** | 19.0 | 2 | 5 | 5 | 11 | 2.09E+08 | **1.40** | 6 | 35.1 | 432 | 48.5 | 6.1 | Regulation of nucleobase, nucleoside, nucleotide and nucleic acid metabolism | Nucleus | ATPase activity | Nucleus |
| **MEA1** | NP_055438.1 | 4201 | **male-enhanced antigen 1** | 9.2 | 1 | 1 | 1 | 2 | 5.23E+07 | **1.40** | 2 | 68 | 185 | 19.9 | 4.2 | Biological_process unknown | Cytoplasm | Molecular function unknown | Cytoplasm |
| **RABEP2** | NP_079092.2 | 79874 | **rab GTPase-binding effector protein 2** | 15.1 | 1 | 6 | 6 | 6 | 4.90E+07 | **1.40** | 4 | 11.7 | 569 | 63.5 | 4.8 | Transport | Cytoplasm;Endosome | GTPase activator activity | Cytoplasm |
| **RBMXL1** | NP_062556.2 | 494115 | **RNA binding motif protein, X-linked-like-1** | 29.2 | 2 | 1 | 12 | 48 | 7.97E+08 | **1.40** | 1 | - | 390 | 42.1 | 9.9 | - | - | - | - |
| **CUL2** | NP_001185706.1 | 8453 | **cullin-2 isoform c** | 16.5 | 3 | 8 | 8 | 15 | 9.70E+07 | **1.40** | 6 | 7.9 | 745 | 86.9 | 6.9 | Protein metabolism | Cytoplasm;Nucleus | Ubiquitin binding | Cytoplasm |
| **EBNA1BP2** | NP_006815.2 | 10969 | **probable rRNA-processing protein EBP2 isoform 2** | 29.1 | 2 | 10 | 10 | 30 | 9.72E+08 | **1.40** | 12 | 20.2 | 306 | 34.8 | 10.1 | Biological_process unknown | Nucleolus;Nucleus | Molecular function unknown | Nucleolus |
| **GFER** | NP_005253.3 | 2671 | **FAD-linked sulfhydryl oxidase ALR** | 18.1 | 1 | 2 | 2 | 5 | 4.07E+07 | **1.40** | 4 | 23.1 | 205 | 23.4 | 7.6 | Cell communication;Signal transduction | Cytoplasm;Nucleus;Extracellular | Growth factor activity | Cytoplasm |
| **LRRFIP2** | NP_001127841.1 | 9209 | **leucine-rich repeat flightless-interacting protein 2 isoform 3** | 29.7 | 4 | 7 | 8 | 15 | 1.23E+08 | **1.40** | 5 | 15.2 | 424 | 48.3 | 5.4 | Biological_process unknown | Nucleus | Molecular function unknown | Nucleus |
| **NUB1** | NP_057202.3 | 51667 | **NEDD8 ultimate buster 1 isoform 2** | 5.6 | 2 | 2 | 2 | 4 | 2.98E+07 | **1.40** | 1 | - | 625 | 71.9 | 6.6 | Protein metabolism | Nucleus | Ubiquitin-specific protease activity | Nucleus |
| **GRIPAP1** | NP_064522.3 | 56850 | **GRIP1-associated protein 1** | 31.4 | 1 | 16 | 16 | 25 | 1.79E+08 | **1.40** | 13 | 18.5 | 841 | 95.9 | 5.1 | Cell communication;Signal transduction | Endosome;Cytoplasm;Synapse;Membrane fraction;Nucleus | Guanyl-nucleotide exchange factor activity | Cytoplasm |
| **MACROD1** | NP_054786.2 | 28992 | **O-acetyl-ADP-ribose deacetylase MACROD1** | 24.0 | 1 | 4 | 4 | 6 | 3.09E+08 | **1.40** | 3 | 7.1 | 325 | 35.5 | 9.5 | Regulation of nucleobase, nucleoside, nucleotide and nucleic acid metabolism | Mitochondrion | Molecular function unknown | Mitochondrion |
| **UBE2Q1** | NP_060052.3 | 55585 | **ubiquitin-conjugating enzyme E2 Q1** | 7.4 | 1 | 1 | 1 | 2 | 3.19E+08 | **1.40** | 2 | 1.1 | 422 | 46.1 | 5.1 | Protein metabolism | - | Ubiquitin-specific protease activity | - |
| **ELOVL1** | NP_001243330.1 | 64834 | **elongation of very long chain fatty acids protein 1 isoform 2** | 9.9 | 3 | 2 | 2 | 15 | 3.65E+08 | **1.40** | 2 | 0.8 | 252 | 29.5 | 9.8 | Biological_process unknown | - | Molecular function unknown | - |
| **CALR** | NP_004334.1 | 811 | **calreticulin precursor** | 60.9 | 1 | 21 | 21 | 113 | 2.02E+09 | **1.40** | 35 | 38.3 | 417 | 48.1 | 4.4 | Protein metabolism | Endoplasmic reticulum;Nucleus;Extracellular;Cytoplasm;Plasma membrane;Sarcoplasmic | Chaperone activity | Endoplasmic reticulum |
| **FUBP3** | NP_003925.1 | 8939 | **far upstream element-binding protein 3** | 60.5 | 1 | 23 | 24 | 93 | 1.13E+09 | **1.40** | 29 | 33.4 | 572 | 61.6 | 8.4 | Regulation of nucleobase, nucleoside, nucleotide and nucleic acid metabolism | Nucleus | Transcription factor activity | Nucleus |
| **NPHP4** | NP_001278522.1 | 261734 | **nephrocystin-4 isoform b** | 1.9 | 3 | 1 | 1 | 1 | 4.92E+06 | **1.40** | 1 | - | 913 | 100.9 | 8.2 | Cell communication;Signal transduction | - | Molecular function unknown | - |
| **NUP54** | NP_001265532.1 | 53371 | **nucleoporin p54 isoform 2** | 32.0 | 2 | 11 | 11 | 16 | 1.92E+08 | **1.40** | 9 | 14.8 | 459 | 50.7 | 6.8 | Transport | Nucleus;Cytoplasm;Nuclear membrane | Transporter activity | Nucleus |
| **SUB1** | NP_006704.3 | 10923 | **activated RNA polymerase II transcriptional coactivator p15** | 39.4 | 1 | 8 | 8 | 26 | 7.58E+08 | **1.40** | 10 | 21.3 | 127 | 14.4 | 9.6 | Regulation of nucleobase, nucleoside, nucleotide and nucleic acid metabolism | Nucleus;Cytoplasm;Nucleolus;Mitochondrion | Transcription factor activity | Nucleus |
| **CAST** | NP_001035905.1 | 831 | **calpastatin isoform f** | 60.3 | 5 | 33 | 33 | 106 | 6.00E+08 | **1.39** | 40 | 20.2 | 750 | 80.2 | 5.2 | Protein metabolism | Cytoplasm;Nucleus;Perinuclear region | Protease inhibitor activity | Nucleus;Cytoplasm;Perinuclear region |
| **SNRPC** | NP_003084.1 | 6631 | **U1 small nuclear ribonucleoprotein C** | 25.8 | 1 | 3 | 3 | 11 | 1.91E+08 | **1.39** | 4 | 24.2 | 159 | 17.4 | 9.7 | Regulation of nucleobase, nucleoside, nucleotide and nucleic acid metabolism | Nucleus;Nucleolus | RNA binding | Nucleus |
| **EXOC3** | NP_009208.2 | 11336 | **exocyst complex component 3** | 9.7 | 1 | 5 | 5 | 5 | 6.89E+07 | **1.39** | 4 | 21.8 | 745 | 85.5 | 6.3 | Transport | Plasma membrane;Centrosome | Auxiliary transport protein activity | Plasma membrane |
| **SMARCC2** | NP_003066.2 | 6601 | **SWI/SNF complex subunit SMARCC2 isoform a** | 26.6 | 3 | 15 | 17 | 31 | 3.71E+08 | **1.39** | 8 | 24.3 | 1214 | 132.8 | 5.7 | Regulation of nucleobase, nucleoside, nucleotide and nucleic acid metabolism | Nucleus;Kinetochore | Transcription factor activity | Nucleus |
| **THOC7** | NP_079351.2 | 80145 | **THO complex subunit 7 homolog isoform 1** | 2.9 | 1 | 1 | 1 | 1 | 1.28E+08 | **1.39** | 1 | - | 204 | 23.7 | 5.7 | Biological_process unknown | Cytoplasm;Nucleus | Molecular function unknown | Cytoplasm |
| **USP24** | NP_056121.2 | 23358 | **ubiquitin carboxyl-terminal hydrolase 24** | 9.6 | 1 | 14 | 14 | 20 | 6.54E+07 | **1.39** | 8 | 10 | 2620 | 294.2 | 6.1 | Protein metabolism | Cytoplasm | Ubiquitin-specific protease activity | Cytoplasm |
| **AIP** | NP_003968.2 | 9049 | **AH receptor-interacting protein** | 53.9 | 1 | 12 | 12 | 56 | 1.11E+09 | **1.39** | 16 | 10.6 | 330 | 37.6 | 6.3 | Regulation of nucleobase, nucleoside, nucleotide and nucleic acid metabolism | Cytoplasm;Nucleus | Transcription regulator activity | Nucleus |
| **RANBP1** | NP_001265569.1 | 5902 | **ran-specific GTPase-activating protein isoform 3** | 64.0 | 4 | 11 | 11 | 111 | 3.87E+09 | **1.39** | 28 | 24.2 | 200 | 23.2 | 5.3 | Cell communication;Signal transduction | Centrosome;Cytoplasm;Nucleus | Transporter activity | Cytoplasm |
| **FBXW11** | NP_387449.2 | 23291 | **F-box/WD repeat-containing protein 11 isoform A** | 4.9 | 3 | 1 | 1 | 1 | 8.27E+06 | **1.39** | 1 | - | 508 | 58.4 | 7.0 | Protein metabolism | Cytoplasm;Nucleus | Molecular function unknown | Cytoplasm |

| Gene Symbol | RefSeq_Accession | Entrez Gene ID | Description | Coverage (%) | Proteins | Unique Peptides | # Peptides | PSM | Area | SCC-R /SCC-S | SCC-R /SCC-S (Count) | SCC-R /SCC-S (Variability [%]) | # AAs | MW [kDa] | calc. pI | Biological Process | Localization | Molecular Function | Primary Localization |
|---|---|---|---|---|---|---|---|---|---|---|---|---|---|---|---|---|---|---|---|
| **GTF2B** | NP_001505.1 | 2959 | **transcription initiation factor IIB** | 22.5 | 1 | 3 | 3 | 5 | 8.34E+07 | **1.39** | 1 | - | 316 | 34.8 | 8.4 | - | Nucleus;Nucleolus;Kinetochore | Transcription factor activity | Nucleus |
| **FAM91A1** | NP_659400.2 | 157769 | **protein FAM91A1** | 6.1 | 1 | 3 | 3 | 3 | 8.07E+06 | **1.39** | 1 | - | 838 | 93.9 | 6.4 | Biological_process unknown | - | Molecular function unknown | - |
| **IPO9** | NP_060555.2 | 55705 | **importin-9** | 27.5 | 1 | 15 | 15 | 54 | 2.43E+08 | **1.39** | 20 | 38 | 1041 | 115.9 | 4.8 | Transport | Cytoplasm | Transporter activity | Cytoplasm |
| **CENPH** | NP_075060.1 | 64946 | **centromere protein H** | 4.5 | 1 | 1 | 1 | 2 | 3.49E+07 | **1.39** | 1 | - | 247 | 28.5 | 5.3 | Regulation of nucleobase, nucleoside, nucleotide and nucleic acid metabolism | Centrosome;Nucleus;Kinetochore | DNA binding | Centrosome |
| **MAP3K7** | NP_663306.1 | 6885 | **mitogen-activated protein kinase kinase kinase 7 isoform D** | 6.3 | 4 | 2 | 2 | 3 | 2.92E+07 | **1.39** | 2 | 71 | 491 | 53.7 | 6.7 | Cell communication;Signal transduction | Nucleus;Cytoplasm;Plasma membrane;Cytosol | Protein serine/threonine kinase activity | Cytoplasm;Nucleus |
| **MGEA5** | NP_036347.1 | 10724 | **bifunctional protein NCOAT isoform a** | 20.7 | 2 | 11 | 11 | 29 | 4.31E+08 | **1.39** | 11 | 37.1 | 916 | 102.8 | 4.9 | Metabolism;Energy pathways | Cytoplasm;Nucleus;Integral to membrane | Hydrolase activity | Cytoplasm |
| **PSMB9** | NP_002791.1 | 5698 | **proteasome subunit beta type-9 proprotein** | 37.4 | 1 | 6 | 6 | 16 | 4.34E+08 | **1.39** | 6 | 15.8 | 219 | 23.2 | 5.0 | Cell cycle | Nucleus | Transcription regulator activity | Nucleus |
| **HGS** | NP_004703.1 | 9146 | **hepatocyte growth factor-regulated tyrosine kinase substrate** | 11.7 | 1 | 5 | 5 | 11 | 1.27E+08 | **1.38** | 5 | 15 | 777 | 86.1 | 6.2 | Cell communication;Signal transduction | Cytoplasm;Endosome | Receptor signaling complex scaffold activity | Cytoplasm |
| **PTPRF** | NP_569707.2 | 5792 | **receptor-type tyrosine-protein phosphatase F isoform 2 precursor** | 7.6 | 2 | 8 | 8 | 9 | 9.72E+07 | **1.38** | 4 | 35.1 | 1898 | 211.6 | 6.4 | Cell communication;Signal transduction | Plasma membrane;Extracellular | Receptor signaling protein tyrosine phosphatase activity | Plasma membrane |
| **COMMD10** | NP_057228.1 | 51397 | **COMM domain-containing protein 10** | 17.8 | 1 | 3 | 3 | 4 | 2.33E+07 | **1.38** | 4 | 56.2 | 202 | 23 | 6.5 | Biological_process unknown | - | Molecular function unknown | - |
| **CTNNAL1** | NP_001273903.1 | 8727 | **alpha-catulin isoform b** | 4.7 | 2 | 2 | 2 | 4 | 1.61E+07 | **1.38** | 2 | 10.4 | 718 | 80.1 | 6.7 | Apoptosis | Cytoplasm | Molecular function unknown | Cytoplasm |
| **GBAS** | NP_001474.1 | 2631 | **protein NipSnap homolog 2 isoform 1** | 5.6 | 1 | 1 | 1 | 2 | 7.83E+07 | **1.38** | 1 | - | 286 | 33.7 | 9.4 | Cell communication;Signal transduction | Plasma membrane;Mitochondrion | Molecular function unknown | Plasma membrane |
| **C16orf62** | NP_064710.4 | 57020 | **UPF0505 protein C16orf62** | 5.0 | 1 | 4 | 4 | 8 | 1.75E+07 | **1.38** | 3 | 5.1 | 1052 | 118.5 | 7.8 | Biological_process unknown | Nucleus | Molecular function unknown | Nucleus |
| **COMMD1** | NP_689729.1 | 150684 | **COMM domain-containing protein 1** | 30.0 | 1 | 3 | 3 | 6 | 8.14E+07 | **1.38** | 3 | 6.7 | 190 | 21.2 | 6.2 | Cell communication;Signal transduction | Nucleus;Cytoplasm | Molecular function unknown | Cytoplasm |
| **EYA3** | NP_001269490.1 | 2140 | **eyes absent homolog 3 isoform c** | 2.7 | 3 | 1 | 1 | 2 | 4.09E+07 | **1.38** | 1 | - | 527 | 57.8 | 5.1 | Cell communication;Signal transduction | Nucleus;Cytoplasm | Protein tyrosine phosphatase activity | Cytoplasm |
| **CHMP4B** | NP_789782.1 | 128866 | **charged multivesicular body protein 4b** | 18.3 | 1 | 4 | 4 | 10 | 9.53E+08 | **1.38** | 2 | 19 | 224 | 24.9 | 4.8 | Transport | Cytoplasm | Transporter activity | Cytoplasm |
| **CYC1** | NP_001907.2 | 1537 | **cytochrome c1, heme protein, mitochondrial precursor** | 32.3 | 1 | 7 | 7 | 44 | 7.71E+08 | **1.38** | 15 | 18 | 325 | 35.4 | 9.0 | Metabolism;Energy pathways | Mitochondrion | Molecular function unknown | Mitochondrion |
| **YWHAZ** | NP_663723.1 | 7534 | **14-3-3 protein zeta/delta** | 81.2 | 1 | 23 | 28 | 661 | 2.89E+10 | **1.38** | 66 | 9.9 | 245 | 27.7 | 4.8 | Regulation of cell cycle | Cytoplasm;Cytosol;Plasma membrane | Receptor signaling complex scaffold activity | Cytoplasm |
| **UBL5** | NP_077268.1 | 59286 | **ubiquitin-like protein 5** | 31.5 | 1 | 3 | 3 | 3 | 8.57E+07 | **1.38** | 3 | 49.7 | 73 | 8.5 | 8.4 | Protein metabolism | Cytoplasm | Ubiquitin-specific protease activity | Cytoplasm |
| **DCTPP1** | NP_077001.1 | 79077 | **dCTP pyrophosphatase 1** | 24.1 | 1 | 2 | 2 | 7 | 1.92E+08 | **1.38** | 4 | 6.5 | 170 | 18.7 | 5.0 | Biological_process unknown | - | Molecular function unknown | - |
| **PIP4K2C** | NP_001139731.1 | 79837 | **phosphatidylinositol 5-phosphate 4-kinase type-2 gamma isoform b** | 18.1 | 3 | 3 | 3 | 8 | 2.52E+08 | **1.38** | 3 | 24.2 | 403 | 45 | 6.5 | Cell communication;Signal transduction | - | Lipid kinase activity | - |
| **SMN2** | NP_075015.1 | 6607 | **survival motor neuron protein isoform c** | 16.4 | 4 | 2 | 2 | 7 | 4.74E+07 | **1.38** | 1 | - | 250 | 27.1 | 5.3 | Regulation of nucleobase, nucleoside, nucleotide and nucleic acid metabolism | Cytoplasm;Nucleus | RNA binding;Protein binding | Nucleus |
| **CHCHD3** | NP_060282.1 | 54927 | **coiled-coil-helix-coiled-coil-helix domain-containing protein 3, mitochondrial precursor** | 30.0 | 1 | 5 | 5 | 8 | 9.16E+07 | **1.38** | 2 | 55.9 | 227 | 26.1 | 8.3 | Biological_process unknown | - | Molecular function unknown | - |
| **FUS** | NP_001164408.1 | 2521 | **RNA-binding protein FUS isoform 3** | 25.7 | 3 | 10 | 12 | 60 | 2.12E+09 | **1.38** | 17 | 26.7 | 522 | 53.2 | 9.4 | RNA localization | Nucleus;Cytoplasm;Nucleolus;Mitochondrion | Nucleocytoplasmic transporter activity | Nucleus |
| **YWHAB** | NP_003395.1 | 7529 | **14-3-3 protein beta/alpha** | 76.8 | 1 | 15 | 23 | 275 | 1.87E+10 | **1.37** | 22 | 18.3 | 246 | 28.1 | 4.8 | Signal transduction | Cytoplasm;Mitochondrion | Receptor signaling complex scaffold activity | Cytoplasm |
| **BCS1L** | NP_001244272.1 | 617 | **mitochondrial chaperone BCS1** | 10.7 | 1 | 2 | 2 | 2 | 4.07E+07 | **1.37** | 2 | 44 | 419 | 47.5 | 8.5 | Metabolism;Energy pathways | Mitochondrion | Molecular function unknown | Mitochondrion |
| **HPS5** | NP_852609.1 | 11234 | **Hermansky-Pudlak syndrome 5 protein isoform b** | 3.1 | 2 | 2 | 2 | 2 | 7.55E+06 | **1.37** | 1 | - | 1015 | 114.8 | 5.4 | Cell organization and biogenesis | Cytoplasm | Molecular function unknown | Cytoplasm |
| **PM20D2** | NP_001010853.1 | 135293 | **peptidase M20 domain-containing protein 2** | 17.4 | 1 | 4 | 4 | 5 | 5.35E+07 | **1.37** | 2 | 16.3 | 436 | 47.7 | 5.9 | Protein metabolism | - | Metallopeptidase activity | - |
| **TCEA1** | NP_006747.1 | 6917 | **transcription elongation factor A protein 1 isoform 1** | 48.8 | 4 | 12 | 12 | 27 | 2.66E+08 | **1.37** | 13 | 46.8 | 301 | 33.9 | 8.4 | Transcription | Nucleus;Nucleolus;Cytoplasm | Transcription regulator activity | Nucleus;Cytoplasm |
| **ACTL6A** | NP_817126.1 | 86 | **actin-like protein 6A isoform 2** | 43.4 | 3 | 12 | 12 | 29 | 1.27E+09 | **1.37** | 13 | 30.4 | 387 | 43.2 | 6.1 | Regulation of nucleobase, nucleoside, nucleotide and nucleic acid metabolism | Cytoplasm;Chromosome;Kinetochore;Nucleus | Chromatin binding | Cytoplasm;Nucleus |
| **PFDN2** | NP_036526.2 | 5202 | **prefoldin subunit 2** | 50.7 | 1 | 6 | 6 | 44 | 6.46E+08 | **1.37** | 10 | 17.7 | 154 | 16.6 | 6.6 | Protein metabolism | - | Chaperone activity | - |
| **PANX1** | NP_056183.2 | 24145 | **pannexin-1** | 10.6 | 1 | 2 | 2 | 3 | 2.32E+07 | **1.37** | 1 | - | 426 | 48 | 6.0 | Cell growth and/or maintenance | Plasma membrane | Cell adhesion molecule activity | Plasma membrane |
| **SSSCA1** | NP_006387.1 | 10534 | **Sjoegren syndrome/scleroderma autoantigen 1** | 67.3 | 1 | 7 | 7 | 21 | 7.09E+08 | **1.37** | 8 | 62 | 199 | 21.5 | 5.2 | Cell communication;Signal transduction | Centrosome | Molecular function unknown | Centrosome |
| **PPIL3** | NP_570981.1 | 53938 | **peptidyl-prolyl cis-trans isomerase-like 3 isoform PPIL3b** | 11.8 | 2 | 1 | 1 | 4 | 3.00E+08 | **1.37** | 1 | - | 161 | 18.1 | 6.8 | Metabolism;Energy pathways | Cytoplasm | Isomerase activity | Cytoplasm |
| **UBQLN2** | NP_038472.2 | 29978 | **ubiquilin-2** | 25.6 | 1 | 6 | 9 | 40 | 5.47E+08 | **1.37** | 7 | 65.2 | 624 | 65.7 | 5.2 | Protein metabolism | Cytoplasm;Nucleus | Ubiquitin-specific protease activity | Cytoplasm |
| **DCAF7** | NP_005819.3 | 10238 | **DDB1- and CUL4-associated factor 7** | 2.6 | 1 | 1 | 1 | 1 | 4.23E+07 | **1.37** | 1 | - | 342 | 38.9 | 5.5 | Biological_process unknown;Signal transduction | Cytoplasm | Molecular function unknown | Cytoplasm |
| **EIF3K** | NP_037366.1 | 27335 | **eukaryotic translation initiation factor 3 subunit K** | 40.8 | 1 | 6 | 6 | 20 | 3.41E+08 | **1.37** | 7 | 19.4 | 218 | 25 | 4.9 | Protein metabolism | Cytoplasm;Nucleus | Translation regulator activity | Cytoplasm |

| Gene Symbol | RefSeq_Accession | Entrez Gene ID | Description | Coverage (%) | Proteins | Unique Peptides | # Peptides | PSM | Area | SCC-R /SCC-S | SCC-R /SCC-S (Count) | SCC-R /SCC-S (Variability [%]) | # AAs | MW [kDa] | calc. pI | Biological Process | Localization | Molecular Function | Primary Localization |
|---|---|---|---|---|---|---|---|---|---|---|---|---|---|---|---|---|---|---|---|
| **RSBN1** | NP_060834.2 | 54665 | **round spermatid basic protein 1** | 1.8 | 1 | 1 | 1 | 2 | 7.75E+07 | **1.37** | 1 | - | 802 | 90 | 8.6 | Transcription | Nucleus | Transcription regulator activity | Nucleus |
| **PTK2** | NP_722560.1 | 5747 | **focal adhesion kinase 1 isoform a** | 13.5 | 3 | 9 | 9 | 16 | 1.29E+08 | **1.37** | 5 | 7.9 | 1052 | 119.2 | 6.6 | Cell communication;Signal transduction | Cytoplasm;Nucleus;Axon | Protein-tyrosine kinase activity | Cytoplasm;Nucleus |
| **TSN** | NP_004613.1 | 7247 | **translin isoform 1** | 47.8 | 2 | 9 | 9 | 37 | 8.10E+08 | **1.37** | 11 | 41.2 | 228 | 26.2 | 6.4 | Regulation of nucleobase, nucleoside, nucleotide and nucleic acid metabolism | Nucleus;Cytoplasm | DNA binding | Nucleus |
| **TTC37** | NP_055454.1 | 9652 | **tetratricopeptide repeat protein 37** | 6.8 | 1 | 6 | 6 | 7 | 1.16E+08 | **1.37** | 2 | 94.3 | 1564 | 175.4 | 7.5 | Biological_process unknown | - | Molecular function unknown | - |
| **CCDC91** | NP_060788.3 | 55297 | **coiled-coil domain-containing protein 91** | 3.4 | 1 | 1 | 1 | 1 | 6.25E+07 | **1.36** | 1 | - | 441 | 49.9 | 5.1 | Biological_process unknown | Cytoplasm | Molecular function unknown | Cytoplasm |
| **PRPSAP1** | NP_002757.2 | 5635 | **phosphoribosyl pyrophosphate synthase-associated protein 1** | 20.8 | 1 | 4 | 5 | 12 | 1.50E+08 | **1.36** | 4 | 8.4 | 385 | 42.4 | 8.5 | Metabolism;Energy pathways | Cytoplasm | Enzyme regulator activity | Cytoplasm |
| **GRPEL1** | NP_079472.1 | 80273 | **grpE protein homolog 1, mitochondrial precursor** | 48.9 | 1 | 9 | 9 | 39 | 5.86E+08 | **1.36** | 18 | 13.2 | 217 | 24.3 | 8.1 | Protein metabolism | Mitochondrion | Chaperone activity | Mitochondrion |
| **HIBADH** | NP_689953.1 | 11112 | **3-hydroxyisobutyrate dehydrogenase, mitochondrial precursor** | 53.3 | 1 | 12 | 12 | 45 | 5.91E+08 | **1.36** | 19 | 35.9 | 336 | 35.3 | 8.1 | Metabolism;Energy pathways | Mitochondrion | Catalytic activity | Mitochondrion |
| **PPIA** | NP_066953.1 | 5478 | **peptidyl-prolyl cis-trans isomerase A** | 82.4 | 6 | 15 | 16 | 772 | 2.09E+10 | **1.36** | 92 | 21.9 | 165 | 18 | 7.8 | Protein folding;Peptide metabolism | Cytoplasm;Nucleolus;Mitochondrion | Isomerase activity | Cytoplasm |
| **HDGFRP2** | NP_116020.1 | 84717 | **hepatoma-derived growth factor-related protein 2 isoform 2** | 11.5 | 2 | 5 | 5 | 13 | 4.74E+08 | **1.36** | 8 | 12.6 | 670 | 74.2 | 7.5 | Cell growth and/or maintenance | Nucleus;Cytoplasm | Growth factor activity | Nucleus |
| **STAG1** | NP_005853.2 | 10274 | **cohesin subunit SA-1** | 4.9 | 1 | 2 | 3 | 7 | 9.33E+07 | **1.36** | 3 | 22.1 | 1258 | 144.3 | 5.6 | Cell communication;Signal transduction | Nucleus | Molecular function unknown | Nucleus |
| **SEPT2** | NP_004395.1 | 4735 | **septin-2 isoform a** | 63.7 | 3 | 16 | 16 | 119 | 1.51E+09 | **1.36** | 26 | 20.7 | 361 | 41.5 | 6.6 | Cell communication;Signal transduction;Cell cycle | Cytoplasm;Plasma membrane;Nucleolus;Microtubule;Mitochondrion;Nucleus | GTPase activity | Cytoplasm |
| **BOD1L1** | NP_683692.2 | 259282 | **biorientation of chromosomes in cell division protein 1-like 1** | 4.2 | 3 | 7 | 7 | 12 | 1.89E+08 | **1.36** | 5 | 41.7 | 3051 | 330.3 | 5.1 | Biological_process unknown | Nucleus;Cytoplasm | Molecular function unknown | Nucleus;Cytoplasm |
| **RANGAP1** | NP_002874.1 | 5905 | **ran GTPase-activating protein 1** | 50.8 | 1 | 17 | 17 | 64 | 7.08E+08 | **1.36** | 20 | 16.8 | 587 | 63.5 | 4.7 | Cell communication;Signal transduction | Cytoplasm;Nucleus;Kinetochore;Microtubule | GTPase activator activity | Cytoplasm |
| **MED8** | NP_001001653.1 | 112950 | **mediator of RNA polymerase II transcription subunit 8 isoform 1** | 11.2 | 3 | 1 | 1 | 4 | 3.04E+08 | **1.36** | 2 | 1.4 | 179 | 19 | 9.1 | Regulation of nucleobase, nucleoside, nucleotide and nucleic acid metabolism | Nucleus;Extracellular | Transcription regulator activity | Nucleus |
| **OS9** | NP_001248352.1 | 10956 | **protein OS-9 isoform 8 precursor** | 12.5 | 8 | 3 | 3 | 5 | 4.91E+07 | **1.36** | 2 | 12.4 | 538 | 60.8 | 4.8 | Biological_process unknown | Nucleus;Cytoplasm;Endoplasmic reticulum | Molecular function unknown | Cytoplasm;Endoplasmic reticulum |
| **ENO1** | NP_001419.1 | 2023 | **alpha-enolase isoform 1** | 81.1 | 3 | 17 | 46 | 1267 | 2.04E+10 | **1.36** | 35 | 12.7 | 434 | 47.1 | 7.4 | Metabolism;Energy pathways | Cytoplasm;Plasma membrane;Nucleus;Extracellular | Catalytic activity | Cytoplasm |
| **ANLN** | NP_001271231.1 | 54443 | **actin-binding protein anillin isoform 3** | 13.7 | 3 | 9 | 9 | 11 | 1.99E+08 | **1.36** | 6 | 27.3 | 1086 | 119.8 | 8.2 | Cell growth and/or maintenance | Nucleus;Nucleolus;Cytoplasm | Structural molecule activity | Nucleus |
| **C19orf43** | NP_076943.1 | 79002 | **uncharacterized protein C19orf43** | 20.5 | 1 | 2 | 2 | 8 | 2.31E+08 | **1.36** | 1 | - | 176 | 18.4 | 9.4 | Biological_process unknown | - | Molecular function unknown | - |
| **NDUFS5** | NP_001171908.1 | 4725 | **NADH dehydrogenase [ubiquinone] iron-sulfur protein 5** | 23.6 | 1 | 2 | 2 | 3 | 1.44E+08 | **1.36** | 1 | - | 106 | 12.5 | 9.1 | Metabolism;Energy pathways | Mitochondrion | Oxidoreductase activity | Mitochondrion |
| **MTHFR** | NP_005948.3 | 4524 | **methylenetetrahydrofolate reductase** | 3.7 | 1 | 1 | 1 | 1 | 2.92E+07 | **1.35** | 1 | - | 656 | 74.5 | 5.3 | Metabolism;Energy pathways | Extracellular | Oxidoreductase activity | Extracellular |
| **PFDN4** | NP_002614.2 | 5203 | **prefoldin subunit 4** | 20.2 | 1 | 3 | 3 | 10 | 2.20E+08 | **1.35** | 5 | 0.9 | 134 | 15.3 | 4.5 | Protein metabolism | Cytoplasm | Chaperone activity | Cytoplasm |
| **PSAT1** | NP_478059.1 | 29968 | **phosphoserine aminotransferase isoform 1** | 61.1 | 2 | 20 | 20 | 66 | 9.39E+08 | **1.35** | 23 | 15.9 | 370 | 40.4 | 7.7 | Metabolism;Energy pathways | Cytosol | Transaminase activity | Cytosol |
| **TBCA** | NP_004598.1 | 6902 | **tubulin-specific chaperone A** | 46.3 | 1 | 9 | 9 | 27 | 2.22E+09 | **1.35** | 15 | 7.6 | 108 | 12.8 | 5.3 | Protein metabolism | Cytoskeleton | Chaperone activity | Cytoskeleton |
| **GNG5** | NP_005265.1 | 2787 | **guanine nucleotide-binding protein G(I)/G(S)/G(O) subunit gamma-5 precursor** | 14.7 | 1 | 1 | 1 | 2 | 7.24E+06 | **1.35** | 1 | - | 68 | 7.3 | 9.9 | Cell communication;Signal transduction | Integral to membrane | GTPase activity | Integral to membrane |
| **GOLPH3** | NP_071413.1 | 64083 | **Golgi phosphoprotein 3** | 49.0 | 1 | 8 | 8 | 17 | 1.90E+08 | **1.35** | 9 | 24.1 | 298 | 33.8 | 6.4 | Transport | Golgi apparatus;Cytoplasm | Transporter activity | Golgi apparatus |
| **RAC2** | NP_002863.1 | 5880 | **ras-related C3 botulinum toxin substrate 2** | 37.0 | 3 | 2 | 7 | 28 | 9.28E+08 | **1.35** | 1 | - | 192 | 21.4 | 7.6 | Cell communication;Signal transduction | Plasma membrane | GTPase activity | Plasma membrane |
| **CARS2** | NP_078813.1 | 79587 | **probable cysteine--tRNA ligase, mitochondrial** | 16.5 | 1 | 4 | 5 | 18 | 1.24E+09 | **1.35** | 4 | 20.2 | 564 | 62.2 | 8.3 | Metabolism | Mitochondrion;Cytosol | Ligase activity | Mitochondrion |
| **DNAJC8** | NP_055095.2 | 22826 | **dnaJ homolog subfamily C member 8** | 45.5 | 1 | 9 | 9 | 26 | 3.13E+08 | **1.35** | 10 | 14.4 | 253 | 29.8 | 9.1 | Protein metabolism | Nucleolus | Chaperone activity | Nucleolus |
| **MRPL52** | NP_851313.1 | 122704 | **39S ribosomal protein L52, mitochondrial isoform c** | 27.1 | 2 | 1 | 1 | 1 | 2.48E+07 | **1.35** | 1 | - | 122 | 13.6 | 10.3 | Protein metabolism | Mitochondrion | Structural constituent of ribosome | Mitochondrion |
| **PDCD6IP** | NP_037506.2 | 10015 | **programmed cell death 6-interacting protein isoform 1** | 59.2 | 3 | 43 | 43 | 187 | 1.21E+09 | **1.35** | 60 | 23 | 868 | 96 | 6.5 | Apoptosis | Cytoplasm;Perinuclear region;Cytoskeleton | Molecular function unknown | Cytoplasm |
| **PLD1** | NP_001123553.1 | 5337 | **phospholipase D1 isoform b** | 1.8 | 2 | 1 | 1 | 2 | 4.75E+07 | **1.35** | 1 | - | 1036 | 119.6 | 8.7 | Cell communication;Signal transduction | Plasma membrane;Endoplasmic reticulum;Golgi apparatus;Endosome;Cytoplasm;Extracellular;Perinuclear region;Nucleus | Phosphoric diester hydrolase activity | Cytoplasm;Nucleus |
| **UBXN1** | NP_001273006.1 | 51035 | **UBX domain-containing protein 1 isoform 2** | 47.1 | 3 | 9 | 9 | 30 | 4.87E+08 | **1.35** | 11 | 10.5 | 297 | 33.3 | 5.3 | Proteolysis and peptidolysis | - | Ubiquitin binding | - |
| **CCDC22** | NP_054727.1 | 28952 | **coiled-coil domain-containing protein 22** | 16.6 | 1 | 6 | 6 | 11 | 1.41E+08 | **1.35** | 6 | 26.8 | 627 | 70.7 | 6.7 | Biological_process unknown | - | Molecular function unknown | - |
| **FNBP1** | NP_055848.1 | 23048 | **formin-binding protein 1** | 3.7 | 1 | 1 | 1 | 1 | 5.19E+07 | **1.35** | 1 | - | 617 | 71.3 | 5.7 | Biological_process unknown | Cytoplasm;Nucleus | Molecular function unknown | Nucleus |
| **GANAB** | NP_938148.1 | 23193 | **neutral alpha-glucosidase AB isoform 2 precursor** | 57.7 | 3 | 1 | 37 | 222 | 1.36E+09 | **1.35** | 3 | 0.2 | 944 | 106.8 | 6.1 | Carbohydrate metabolism | Endoplasmic reticulum;Centrosome | Hydrolase activity | Endoplasmic reticulum |
| **LARP1** | NP_056130.2 | 23367 | **la-related protein 1** | 34.6 | 1 | 19 | 19 | 54 | 4.92E+08 | **1.35** | 18 | 32.5 | 1019 | 116.4 | 9.1 | Biological_process unknown;Transport | Nucleus;Nucleolus;Cytoplasm | Molecular function unknown;Ribonucleoprotein | Nucleus;Cytoplasm |

| Gene Symbol | RefSeq_Accession | Entrez Gene ID | Description | Coverage (%) | Proteins | Unique Peptides | # Peptides | PSM | Area | SCC-R /SCC-S | SCC-R /SCC-S (Count) | SCC-R /SCC-S (Variability [%]) | # AAs | MW [kDa] | calc. pI | Biological Process | Localization | Molecular Function | Primary Localization |
|---|---|---|---|---|---|---|---|---|---|---|---|---|---|---|---|---|---|---|---|
| **YTHDC1** | NP_588611.2 | 91746 | **YTH domain-containing protein 1 isoform 2** | 4.0 | 2 | 2 | 2 | 3 | 5.42E+07 | **1.35** | 1 | - | 709 | 82.6 | 6.0 | Regulation of nucleobase, nucleoside, nucleotide and nucleic acid metabolism | Nucleus;Cytoplasm | RNA binding | Nucleus;Cytoplasm |
| **ASPH** | NP_004309.2 | 444 | **aspartyl/asparaginyl beta-hydroxylase isoform a** | 23.0 | 12 | 10 | 10 | 26 | 3.71E+08 | **1.35** | 13 | 54.1 | 758 | 85.8 | 5.0 | Protein metabolism | Endoplasmic reticulum | Catalytic activity | Endoplasmic reticulum |
| **SNRPE** | NP_003085.1 | 6635 | **small nuclear ribonucleoprotein E** | 56.5 | 1 | 3 | 3 | 50 | 6.86E+08 | **1.34** | 7 | 17.9 | 92 | 10.8 | 9.4 | Regulation of nucleobase, nucleoside, nucleotide and nucleic acid metabolism | Nucleus;Cytoplasm | Ribonucleoprotein | Nucleus |
| **ERP44** | NP_055866.1 | 23071 | **endoplasmic reticulum resident protein 44 precursor** | 34.5 | 1 | 9 | 9 | 22 | 2.73E+08 | **1.34** | 6 | 6.7 | 406 | 46.9 | 5.3 | Protein metabolism | Endoplasmic reticulum | Molecular function unknown | Endoplasmic reticulum |
| **LEO1** | NP_001273359.1 | 123169 | **RNA polymerase-associated protein LEO1 isoform 2** | 7.9 | 2 | 2 | 2 | 6 | 4.66E+07 | **1.34** | 1 | - | 606 | 68 | 4.5 | Biological_process unknown | Nucleus;Cytoplasm | Molecular function unknown | Nucleus;Cytoplasm |
| **PBXIP1** | NP_065385.2 | 57326 | **pre-B-cell leukemia transcription factor-interacting protein 1** | 5.3 | 1 | 3 | 3 | 5 | 4.70E+07 | **1.34** | 2 | 0.8 | 731 | 80.6 | 5.3 | Regulation of nucleobase, nucleoside, nucleotide and nucleic acid metabolism | Cytoplasm;Nucleus | Transcription regulator activity | Cytoplasm |
| **TRIM33** | NP_148980.2 | 51592 | **E3 ubiquitin-protein ligase TRIM33 isoform beta** | 7.2 | 2 | 4 | 4 | 9 | 8.26E+07 | **1.34** | 5 | 15.1 | 1110 | 120.5 | 6.7 | Regulation of nucleobase, nucleoside, nucleotide and nucleic acid metabolism | Nucleus | Transcription regulator activity | Nucleus |
| **OXR1** | NP_851999.2 | 55074 | **oxidation resistance protein 1 isoform 2** | 3.9 | 4 | 2 | 2 | 3 | 2.25E+07 | **1.34** | 2 | 48.7 | 839 | 93.7 | 5.2 | Biological_process unknown | Mitochondrion;Nucleus;Cytoplasm | Molecular function unknown | Mitochondrion |
| **SRM** | NP_003123.2 | 6723 | **spermidine synthase** | 33.8 | 1 | 7 | 7 | 20 | 1.62E+08 | **1.34** | 6 | 29.2 | 302 | 33.8 | 5.5 | Metabolism;Energy pathways | Cytoplasm | Transferase activity;Methyltransferase activity | Cytoplasm |
| **FKBP1A** | NP_463460.1 | 2280 | **peptidyl-prolyl cis-trans isomerase FKBP1A isoform a** | 75.0 | 2 | 6 | 6 | 28 | 9.60E+08 | **1.34** | 8 | 39.5 | 108 | 11.9 | 8.2 | Cell communication;Signal transduction | Cytoplasm;Sarcoplasmic reticulum | Isomerase activity | Cytoplasm |
| **RTN3** | NP_006045.1 | 10313 | **reticulon-3 isoform a** | 21.2 | 7 | 2 | 2 | 7 | 4.13E+08 | **1.34** | 2 | 29.8 | 236 | 25.6 | 8.5 | Biological_process unknown | Endoplasmic reticulum;Golgi apparatus;Nucleus | Molecular function unknown | Endoplasmic reticulum |
| **EFTUD1** | NP_001035700.1 | 79631 | **elongation factor Tu GTP-binding domain-containing protein 1 isoform 2** | 15.3 | 2 | 8 | 8 | 20 | 2.73E+08 | **1.34** | 11 | 18.7 | 1069 | 119.8 | 5.8 | Regulation of nucleobase, nucleoside, nucleotide and nucleic acid metabolism | - | Translation regulator activity | - |
| **DYNC1I2** | NP_001258718.1 | 1781 | **cytoplasmic dynein 1 intermediate chain 2 isoform 3** | 49.8 | 4 | 20 | 20 | 93 | 1.35E+09 | **1.34** | 27 | 16.3 | 612 | 68.4 | 5.3 | Cell growth and/or maintenance | Cytoplasm;Centrosome | Motor activity | Cytoplasm |
| **CMC2** | NP_064573.1 | 56942 | **COX assembly mitochondrial protein 2 homolog** | 27.9 | 1 | 1 | 1 | 3 | 5.34E+08 | **1.34** | 2 | 13.4 | 79 | 9.5 | 7.9 | Biological_process unknown | - | Molecular function unknown | - |
| **COMMD3-BMI1** | NP_001190991.1 | 100532731 | **COMMD3-BMI1 read-through protein** | 12.2 | 4 | 3 | 3 | 7 | 1.58E+08 | **1.34** | 2 | 12.1 | 469 | 53.3 | 7.9 | - | - | - | - |
| **TPI1** | NP_000356.1 | 7167 | **triosephosphate isomerase isoform 1** | 88.8 | 3 | 30 | 30 | 938 | 1.63E+10 | **1.34** | 118 | 21.1 | 249 | 26.7 | 6.9 | Metabolism;Energy pathways | Cytoplasm | Isomerase activity | Cytoplasm |
| **PFDN5** | NP_002615.2 | 5204 | **prefoldin subunit 5 isoform alpha** | 66.9 | 2 | 9 | 9 | 36 | 3.65E+08 | **1.34** | 13 | 24.7 | 154 | 17.3 | 6.3 | Protein metabolism | Cytoplasm;Nucleus | Chaperone activity | Nucleus |
| **SLC5A3** | NP_008864.4 | 6526 | **sodium/myo-inositol cotransporter** | 4.0 | 1 | 2 | 2 | 2 | 4.73E+07 | **1.34** | 1 | - | 718 | 79.6 | 7.5 | Transport | Plasma membrane | Transporter activity | Plasma membrane |
| **CHMP5** | NP_057494.3 | 51510 | **charged multivesicular body protein 5 isoform 1** | 52.5 | 2 | 6 | 6 | 21 | 2.87E+08 | **1.33** | 5 | 17.1 | 219 | 24.6 | 4.8 | Transport | - | Transporter activity | - |
| **CBFB** | NP_001746.1 | 865 | **core-binding factor subunit beta isoform 2** | 23.1 | 2 | 3 | 3 | 9 | 1.34E+08 | **1.33** | 3 | 7 | 182 | 21.5 | 6.6 | Regulation of nucleobase, nucleoside, nucleotide and nucleic acid metabolism | Nucleus;Cytoplasm | Transcription factor activity | Cytoplasm |
| **NDUFA5** | NP_001269350.1 | 4698 | **NADH dehydrogenase [ubiquinone] 1 alpha subcomplex subunit 5 isoform 4** | 51.3 | 6 | 5 | 5 | 27 | 3.42E+08 | **1.33** | 8 | 17 | 113 | 13.3 | 5.6 | Metabolism;Energy pathways | Mitochondrion | Oxidoreductase activity | Mitochondrion |
| **REXO4** | NP_065118.2 | 57109 | **RNA exonuclease 4 isoform 1** | 33.2 | 4 | 8 | 8 | 9 | 2.52E+08 | **1.33** | 4 | 21.8 | 422 | 46.6 | 9.8 | Regulation of nucleobase, nucleoside, nucleotide and nucleic acid metabolism | Nucleus | Translation regulator activity | Nucleus |
| **NDUFA8** | NP_055037.1 | 4702 | **NADH dehydrogenase [ubiquinone] 1 alpha subcomplex subunit 8** | 39.5 | 1 | 4 | 4 | 16 | 8.59E+07 | **1.33** | 6 | 14 | 172 | 20.1 | 7.7 | Metabolism;Energy pathways | Mitochondrion | Catalytic activity | Mitochondrion |
| **NSUN5** | NP_060514.1 | 55695 | **putative methyltransferase NSUN5 isoform 2** | 17.0 | 4 | 5 | 5 | 7 | 1.33E+08 | **1.33** | 5 | 33.9 | 429 | 46.7 | 8.6 | Cell growth and/or maintenance | - | Molecular function unknown | - |
| **TXLNA** | NP_787048.1 | 200081 | **alpha-taxilin** | 56.2 | 1 | 16 | 16 | 42 | 6.34E+08 | **1.33** | 16 | 29.9 | 546 | 61.9 | 6.5 | Cell communication;Signal transduction | Cytoplasm;Plasma membrane;Nucleus | Molecular function unknown | Cytoplasm |
| **ARPC3** | NP_001265485.1 | 10094 | **actin-related protein 2/3 complex subunit 3 isoform 1** | 54.5 | 2 | 8 | 8 | 34 | 3.62E+08 | **1.33** | 10 | 25.3 | 178 | 20.5 | 8.6 | Cell growth and/or maintenance | Cytoplasm;Nucleolus | Cytoskeletal protein binding | Cytoplasm |
| **PSMD2** | NP_002799.3 | 5708 | **26S proteasome non-ATPase regulatory subunit 2 isoform 1** | 55.8 | 3 | 34 | 34 | 159 | 1.52E+09 | **1.33** | 53 | 12.9 | 908 | 100.1 | 5.2 | Protein metabolism | Cytoplasm;Cytosol | Ubiquitin-specific protease activity | Cytoplasm |
| **TNPO3** | NP_036602.1 | 23534 | **transportin-3 isoform 1** | 26.5 | 2 | 14 | 14 | 23 | 2.16E+08 | **1.33** | 8 | 42 | 923 | 104.1 | 5.6 | Cell communication;Signal transduction | Nucleus | Ligand-dependent nuclear receptor activity | Nucleus |
| **PSMC2** | NP_002794.1 | 5701 | **26S protease regulatory subunit 7 isoform 1** | 62.6 | 2 | 21 | 21 | 76 | 6.12E+08 | **1.33** | 31 | 29.7 | 433 | 48.6 | 6.0 | Protein metabolism | Nucleus;Cytoplasm | Ubiquitin-specific protease activity | Cytoplasm |
| **RAB27B** | NP_004154.2 | 5874 | **ras-related protein Rab-27B** | 6.4 | 1 | 1 | 1 | 2 | 3.15E+06 | **1.33** | 1 | - | 218 | 24.6 | 5.5 | Cell communication;Signal transduction | Plasma membrane;Cytoplasm;Zymogen granule | GTPase activity | Plasma membrane |
| **CCDC132** | NP_001244927.1 | 55610 | **coiled-coil domain-containing protein 132 isoform c** | 3.3 | 2 | 2 | 2 | 4 | 1.14E+08 | **1.33** | 2 | 7.8 | 934 | 107.9 | 6.0 | Biological_process unknown | - | Molecular function unknown | - |
| **RAB3B** | NP_002858.2 | 5865 | **ras-related protein Rab-3B** | 26.9 | 14 | 3 | 4 | 14 | 3.05E+08 | **1.33** | 3 | 4.7 | 219 | 24.7 | 5.0 | Cell communication;Signal transduction | Plasma membrane;Cytoplasm | GTPase activity | Plasma membrane |
| **SAP30BP** | NP_037392.1 | 29115 | **SAP30-binding protein** | 10.1 | 1 | 2 | 2 | 5 | 3.81E+07 | **1.33** | 3 | 16.8 | 308 | 33.8 | 4.8 | Biological_process unknown | Nucleus | Transcription regulator activity | Nucleus |
| **LSM8** | NP_057284.1 | 51691 | **LSM8 homolog, U6 small nuclear RNA associated** | 91.7 | 1 | 6 | 6 | 26 | 2.56E+08 | **1.32** | 7 | 7.2 | 96 | 10.4 | 4.5 | Regulation of nucleobase, nucleoside, nucleotide and nucleic acid metabolism | Cytoplasm;Nucleus | RNA binding | Cytoplasm |
| **WNK1** | NP_055638.2 | 65125 | **serine/threonine-protein kinase WNK1 isoform 2** | 3.6 | 8 | 5 | 5 | 9 | 8.17E+07 | **1.32** | 2 | 10.9 | 2134 | 225.4 | 6.4 | Cell communication;Signal transduction | Cytoplasm;Nucleus | Protein serine/threonine kinase activity | Cytoplasm |
| **GNPDA2** | NP_612208.1 | 132789 | **glucosamine-6-phosphate isomerase 2 isoform 1** | 21.7 | 3 | 2 | 5 | 16 | 2.31E+08 | **1.32** | 1 | - | 276 | 31.1 | 7.0 | Metabolism;Energy pathways | - | Deaminase activity | - |
| **QRICH1** | NP_060200.2 | 54870 | **glutamine-rich protein 1** | 9.0 | 1 | 3 | 3 | 3 | 6.62E+07 | **1.32** | 2 | 42.7 | 776 | 86.4 | 5.9 | Biological_process unknown | - | Molecular function unknown | - |

Supplementary Table S4. List of all proteins identified in SCC-S and SCC-R cells using SequestHT and Mascot search algorithms and their gene ontology annotation

| Gene Symbol | RefSeq_Accession | Entrez Gene ID | Description | Coverage (%) | Proteins | Unique Peptides | # Peptides | PSM | Area | SCC-R /SCC-S | SCC-R /SCC-S (Count) | SCC-R /SCC-S (Variability [%]) | # AAs | MW [kDa] | calc. pI | Biological Process | Localization | Molecular Function | Primary Localization |
|---|---|---|---|---|---|---|---|---|---|---|---|---|---|---|---|---|---|---|---|
| **NF2** | NP_861971.1 | 4771 | **merlin isoform 8** | 11.5 | 3 | 1 | 1 | 1 | 7.93E+07 | **1.32** | 1 | - | 165 | 19.2 | 7.3 | Cell growth and/or maintenance | Nucleus;Cytoplasm;Endosome;Plasma membrane | Structural constituent of cytoskeleton | Cytoplasm;Nucleus;Plasma membrane |
| **TIMM10** | NP_036588.1 | 26519 | **mitochondrial import inner membrane translocase subunit Tim10** | 58.9 | 1 | 3 | 3 | 7 | 1.89E+08 | **1.32** | 3 | 1.5 | 90 | 10.3 | 6.3 | Protein metabolism | Mitochondrial membrane;Mitochondrion | Chaperone activity | Mitochondrial membrane |
| **CLASP1** | NP_001135746.1 | 23332 | **CLIP-associating protein 1 isoform 3** | 9.2 | 4 | 7 | 8 | 11 | 8.30E+07 | **1.32** | 4 | 130.2 | 1471 | 162 | 8.8 | Cell growth and/or maintenance | Centrosome;Nucleus;Cytoplasm;Microtubule | Cytoskeletal protein binding | Centrosome |
| **TBCE** | NP_001072983.1 | 6905 | **tubulin-specific chaperone E isoform a** | 27.9 | 3 | 7 | 7 | 15 | 1.29E+08 | **1.32** | 5 | 21.3 | 527 | 59.3 | 6.8 | Protein metabolism | Cytoplasm | Chaperone activity | Cytoplasm |
| **ERLIN1** | NP_001094096.1 | 10613 | **erlin-1** | 35.3 | 1 | 5 | 8 | 19 | 1.52E+08 | **1.32** | 3 | 2.4 | 348 | 39.1 | 7.9 | Biological_process unknown | Plasma membrane;Cytoplasm | Molecular function unknown | Plasma membrane |
| **KLC1** | NP_005543.2 | 3831 | **kinesin light chain 1 isoform 1** | 37.0 | 7 | 10 | 18 | 45 | 4.00E+08 | **1.32** | 6 | 21.7 | 560 | 63.8 | 5.9 | Signal transduction | Cytoplasm;Cytosol;Microtubule;Axon;Perinuclear vesicle;Cell junction;Golgi apparatus;Microsome | Motor activity | Cytoplasm |
| **PFDN6** | NP_001172110.1 | 10471 | **prefoldin subunit 6** | 65.1 | 1 | 8 | 8 | 22 | 3.06E+08 | **1.32** | 5 | 24.1 | 129 | 14.6 | 8.9 | Protein metabolism | - | Chaperone activity | - |
| **ROMO1** | NP_542786.1 | 140823 | **reactive oxygen species modulator 1** | 21.5 | 1 | 1 | 1 | 3 | 2.61E+07 | **1.32** | 1 | - | 79 | 8.2 | 9.3 | Biological_process unknown | - | Molecular function unknown | - |
| **STT3A** | NP_001265433.1 | 3703 | **dolichyl-diphosphooligosaccharide--protein glycosyltransferase subunit STT3A isoform b** | 10.6 | 2 | 5 | 5 | 6 | 7.61E+07 | **1.32** | 3 | 351.3 | 613 | 69.5 | 7.8 | Biological_process unknown | Plasma membrane | Molecular function unknown | Plasma membrane |
| **HDHD1** | NP_001171606.1 | 8226 | **pseudouridine-5'-monophosphatase isoform c** | 13.5 | 3 | 2 | 2 | 2 | 1.28E+07 | **1.32** | 2 | 33.8 | 208 | 23.3 | 6.9 | Biological_process unknown | - | Molecular function unknown | - |
| **ME2** | NP_002387.1 | 4200 | **NAD-dependent malic enzyme, mitochondrial isoform 1 precursor** | 12.0 | 2 | 3 | 3 | 4 | 6.76E+07 | **1.32** | 1 | - | 584 | 65.4 | 7.6 | Metabolism;Energy pathways | Mitochondrion | Carboxy-lyase activity | Mitochondrion |
| **MLLT4** | NP_001278893.1 | 4301 | **afadin isoform 4** | 5.7 | 3 | 6 | 6 | 6 | 5.96E+07 | **1.32** | 4 | 90.4 | 1781 | 201.7 | 6.5 | Cell communication;Signal transduction | Cytoplasm;Nucleus;Plasma membrane;Basolateral membrane | Cell adhesion molecule activity | Cytoplasm |
| **MZT2A** | NP_001078834.1 | 653784 | **mitotic-spindle organizing protein 2A** | 27.2 | 2 | 2 | 2 | 5 | 1.09E+08 | **1.32** | 1 | - | 158 | 16.2 | 9.6 | Biological_process unknown | - | Molecular function unknown | - |
| **PARVA** | NP_060692.2 | 55742 | **alpha-parvin** | 27.7 | 1 | 5 | 6 | 11 | 5.38E+08 | **1.32** | 3 | 3.8 | 412 | 46.6 | 8.4 | Cell growth and/or maintenance | Plasma membrane;Nucleus | Cytoskeletal protein binding | Plasma membrane;Nucleus |
| **HIF1AN** | NP_060372.2 | 55662 | **hypoxia-inducible factor 1-alpha inhibitor** | 9.2 | 1 | 2 | 2 | 3 | 1.09E+08 | **1.32** | 1 | - | 349 | 40.3 | 5.6 | Metabolism;Energy pathways | Cytoplasm;Nucleus | Catalytic activity | Cytoplasm |
| **PRCP** | NP_005031.1 | 5547 | **lysosomal Pro-X carboxypeptidase isoform 1 preproprotein** | 4.2 | 2 | 1 | 1 | 5 | 9.43E+07 | **1.32** | 3 | 7.2 | 496 | 55.8 | 7.2 | Protein metabolism | Lysosome;Extracellular matrix | Carboxypeptidase activity | Lysosome |
| **CELF1** | NP_006551.1 | 10658 | **CUGBP Elav-like family member 1 isoform 1** | 20.8 | 8 | 2 | 6 | 16 | 1.43E+08 | **1.31** | 3 | 13.9 | 482 | 51.5 | 8.5 | Regulation of nucleobase, nucleoside, nucleotide and nucleic acid metabolism;Regulation of gene expression, epigenetic | Cytoplasm;Nucleus | RNA binding | Nucleus |
| **SNX1** | NP_683758.1 | 6642 | **sorting nexin-1 isoform b** | 31.7 | 3 | 10 | 12 | 22 | 3.93E+08 | **1.31** | 6 | 76.8 | 457 | 51.8 | 5.6 | Cell communication;Signal transduction | Endosome;Plasma membrane;Cytoplasm | Transporter activity | Endosome;Plasma membrane |
| **CHM** | NP_000381.1 | 1121 | **rab proteins geranylgeranyltransferase component A 1 isoform a** | 11.0 | 1 | 3 | 3 | 3 | 1.79E+07 | **1.31** | 2 | 61.5 | 653 | 73.4 | 4.8 | Metabolism;Energy pathways | Cytoplasm | Transaminase activity | Cytoplasm |
| **LANCL1** | NP_001130046.1 | 10314 | **lanC-like protein 1** | 14.8 | 1 | 4 | 4 | 7 | 8.44E+07 | **1.31** | 3 | 7.2 | 399 | 45.3 | 7.8 | Immune response | Plasma membrane | G-protein coupled receptor activity | Plasma membrane |
| **NUTF2** | NP_005787.1 | 10204 | **nuclear transport factor 2** | 69.3 | 3 | 5 | 5 | 31 | 6.30E+08 | **1.31** | 6 | 41.2 | 127 | 14.5 | 5.4 | Transport | Cytoplasm;Nucleus | Transporter activity | Cytoplasm |
| **G3BP2** | NP_987100.1 | 9908 | **ras GTPase-activating protein-binding protein 2 isoform b** | 36.8 | 2 | 11 | 12 | 37 | 4.10E+08 | **1.31** | 14 | 23.4 | 449 | 50.8 | 5.4 | Cell communication;Signal transduction | Cytoplasm;Nucleus | Molecular function unknown | Cytoplasm;Nucleus |
| **GSN** | NP_001121138.1 | 2934 | **gelsolin isoform c** | 33.4 | 6 | 16 | 16 | 51 | 4.23E+08 | **1.31** | 15 | 16.9 | 742 | 81.9 | 5.9 | Cell growth and/or maintenance | Cytoplasm;Extracellular;Mitochondrion;Cytoskeleton | Structural constituent of cytoskeleton | Extracellular |
| **LARS2** | NP_056155.1 | 23395 | **probable leucine--tRNA ligase, mitochondrial** | 7.5 | 1 | 2 | 2 | 4 | 6.24E+07 | **1.31** | 1 | - | 903 | 101.9 | 8.2 | Metabolism;Energy pathways | Mitochondrion | Transferase activity | Mitochondrion |
| **BAX** | NP_620119.2 | 581 | **apoptosis regulator BAX isoform sigma** | 31.8 | 6 | 4 | 5 | 10 | 8.83E+07 | **1.31** | 4 | 4.5 | 179 | 19.7 | 5.4 | Apoptosis | Mitochondrion;Nucleus;Cytoplasm;Cytosol;Membrane fraction;Mitochondrial membrane | Receptor signaling complex scaffold activity | Cytoplasm;Mitochondrion |
| **HNRNPH3** | NP_036339.1 | 3189 | **heterogeneous nuclear ribonucleoprotein H3 isoform a** | 63.6 | 2 | 16 | 17 | 42 | 5.80E+08 | **1.31** | 15 | 13.6 | 346 | 36.9 | 6.9 | Regulation of nucleobase, nucleoside, nucleotide and nucleic acid metabolism | Nucleus;Nucleolus;Mitochondrion | Ribonucleoprotein | Nucleus |
| **MTHFD1** | NP_005947.3 | 4522 | **C-1-tetrahydrofolate synthase, cytoplasmic** | 54.1 | 1 | 45 | 45 | 189 | 1.07E+09 | **1.31** | 60 | 20.4 | 935 | 101.5 | 7.3 | Metabolism;Energy pathways | Cytoplasm;Cytosol;Mitochondrion | Catalytic activity | Cytoplasm |
| **NAA30** | NP_001011713.2 | 122830 | **N-alpha-acetyltransferase 30** | 5.3 | 1 | 1 | 1 | 2 | 3.81E+07 | **1.31** | 1 | - | 362 | 39.3 | 5.5 | Metabolism;Energy pathways | - | Transferase activity | - |
| **CACYBP** | NP_055227.1 | 27101 | **calcyclin-binding protein isoform 1** | 70.6 | 2 | 15 | 15 | 104 | 1.47E+09 | **1.31** | 23 | 15.1 | 228 | 26.2 | 8.3 | Protein metabolism | Cytoplasm;Nucleus | Molecular function unknown | Cytoplasm |
| **ESD** | NP_001975.1 | 2098 | **S-formylglutathione hydrolase** | 84.4 | 1 | 14 | 14 | 69 | 5.41E+08 | **1.31** | 17 | 21.1 | 282 | 31.4 | 7.0 | Metabolism;Energy pathways | Cytoplasm | Hydrolase activity | Cytoplasm |
| **WDR1** | NP_059830.1 | 9948 | **WD repeat-containing protein 1 isoform 1** | 69.3 | 2 | 31 | 31 | 144 | 7.60E+08 | **1.31** | 44 | 17.6 | 606 | 66.2 | 6.7 | Biological_process unknown | Cytoplasm | Molecular function unknown | Cytoplasm |
| **XPO1** | NP_003391.1 | 7514 | **exportin-1** | 45.1 | 1 | 36 | 36 | 158 | 5.34E+08 | **1.31** | 51 | 24.9 | 1071 | 123.3 | 6.1 | Cell communication;Signal transduction | Nucleus;Cytoplasm;Nucleolus;Kinetochore;Cytosol;Microtubule | Transporter activity | Nucleus |
| **DNAJA3** | NP_005138.3 | 9093 | **dnaJ homolog subfamily A member 3, mitochondrial isoform 1** | 21.5 | 3 | 6 | 6 | 14 | 2.08E+08 | **1.31** | 6 | 17.3 | 480 | 52.5 | 9.3 | Apoptosis | Mitochondrion;Cytoplasm;Nucleus | Chaperone activity | Mitochondrion |
| **ECI1** | NP_001910.2 | 1632 | **enoyl-CoA delta isomerase 1, mitochondrial isoform 1 precursor** | 28.5 | 2 | 5 | 5 | 31 | 4.09E+08 | **1.31** | 8 | 27.9 | 302 | 32.8 | 8.5 | Metabolism;Energy pathways | Mitochondrion | Isomerase activity | Mitochondrion |
| **GLO1** | NP_006699.2 | 2739 | **lactoylglutathione lyase** | 73.4 | 1 | 11 | 11 | 55 | 1.17E+09 | **1.31** | 20 | 12.7 | 184 | 20.8 | 5.3 | Metabolism;Energy pathways | Cytoplasm | Lyase activity | Cytoplasm |
| **GTF2E1** | NP_005504.2 | 2960 | **general transcription factor IIE subunit 1** | 7.5 | 1 | 2 | 2 | 4 | 6.14E+07 | **1.31** | 3 | 44.9 | 439 | 49.4 | 4.8 | Regulation of nucleobase, nucleoside, nucleotide and nucleic acid metabolism | Nucleus;Kinetochore | Transcription factor activity | Nucleus |
| **LSM4** | NP_036453.1 | 25804 | **U6 snRNA-associated Sm-like protein LSm4 isoform 1** | 39.6 | 2 | 6 | 6 | 40 | 5.21E+08 | **1.31** | 10 | 4.7 | 139 | 15.3 | 10.0 | Regulation of nucleobase, nucleoside, nucleotide and nucleic acid metabolism | Cytoplasm;Nucleus | RNA binding | Cytoplasm |

| Gene Symbol | RefSeq_Accession | Entrez Gene ID | Description | Coverage (%) | Proteins | Unique Peptides | # Peptides | PSM | Area | SCC-R /SCC-S | SCC-R /SCC-S (Count) | SCC-R /SCC-S (Variability [%]) | # AAs | MW [kDa] | calc. pI | Biological Process | Localization | Molecular Function | Primary Localization |
|---|---|---|---|---|---|---|---|---|---|---|---|---|---|---|---|---|---|---|---|
| SLBP | NP_006518.1 | 7884 | histone RNA hairpin-binding protein | 11.9 | 1 | 2 | 2 | 2 | 4.55E+07 | 1.31 | 1 | - | 270 | 31.3 | 7.5 | Regulation of nucleobase, nucleoside, nucleotide and nucleic acid metabolism | Nucleus;Cytoplasm;Ribosome | RNA binding | Nucleus |
| ETHE1 | NP_055112.2 | 23474 | persulfide dioxygenase ETHE1, mitochondrial | 40.6 | 1 | 6 | 6 | 23 | 3.68E+08 | 1.31 | 5 | 2.6 | 254 | 27.9 | 6.8 | Metabolism;Energy pathways | Mitochondrion;Cytoplasm;Nucleus | Molecular function unknown | Mitochondrion |
| HSF1 | NP_005517.1 | 3297 | heat shock factor protein 1 | 3.0 | 1 | 1 | 1 | 3 | 6.93E+07 | 1.31 | 1 | - | 529 | 57.2 | 5.2 | Regulation of nucleobase, nucleoside, nucleotide and nucleic acid metabolism | Cytoplasm;Nucleus | Transcription factor activity | Cytoplasm |
| HNRNPD | NP_002129.2 | 3184 | heterogeneous nuclear ribonucleoprotein D0 isoform c | 76.8 | 4 | 23 | 26 | 210 | 3.74E+09 | 1.30 | 49 | 9.8 | 306 | 32.8 | 8.2 | Regulation of nucleobase, nucleoside, nucleotide and nucleic acid metabolism | Nucleus;Cytoplasm;Mitochondrion | MRNA binding | Nucleus;Cytoplasm |
| LAMTOR2 | NP_054736.1 | 28956 | ragulator complex protein LAMTOR2 isoform 1 | 33.6 | 2 | 3 | 3 | 3 | 4.78E+07 | 1.30 | 3 | 37.3 | 125 | 13.5 | 5.4 | Cell communication;Signal transduction | Lysosome | Molecular function unknown | Lysosome |
| BANF1 | NP_001137457.1 | 8815 | barrier-to-autointegration factor | 78.7 | 1 | 9 | 9 | 71 | 2.84E+09 | 1.30 | 22 | 15.6 | 89 | 10.1 | 6.1 | Biological_process unknown | Nucleus;Nucleolus | DNA binding | Nucleus |
| SPCS1 | NP_054760.3 | 28972 | signal peptidase complex subunit 1 | 10.1 | 1 | 1 | 1 | 2 | 1.37E+08 | 1.30 | 1 | - | 169 | 18.3 | 8.7 | Protein metabolism | Endoplasmic reticulum membrane | Signal peptidase activity | Endoplasmic reticulum membrane |
| TRADD | NP_003780.1 | 8717 | tumor necrosis factor receptor type 1-associated DEATH domain protein | 11.2 | 1 | 2 | 2 | 5 | 3.57E+07 | 1.30 | 1 | - | 312 | 34.2 | 6.3 | Cell communication;Signal transduction;Apoptosis | Cytoplasm;Plasma membrane;Nucleus;Golgi apparatus;Endoplasmic reticulum | Receptor signaling complex scaffold activity | Cytoplasm |
| MTHFD2 | NP_006627.2 | 10797 | bifunctional methylenetetrahydrofolate dehydrogenase/cyclohydrolase, mitochondrial precursor | 22.0 | 1 | 4 | 4 | 4 | 1.47E+08 | 1.30 | 3 | 29.2 | 350 | 37.9 | 8.7 | Metabolism;Energy pathways | Mitochondrion | Catalytic activity | Mitochondrion |
| CBX1 | NP_006798.1 | 10951 | chromobox protein homolog 1 | 54.1 | 1 | 6 | 7 | 22 | 1.57E+08 | 1.30 | 4 | 18.1 | 185 | 21.4 | 4.9 | Regulation of nucleobase, nucleoside, nucleotide and nucleic acid metabolism | Nucleus;Cytoplasm;Nucleolus | DNA binding | Nucleus |
| HAUS5 | NP_056117.1 | 23354 | HAUS augmin-like complex subunit 5 | 1.7 | 1 | 1 | 1 | 1 | 2.28E+08 | 1.30 | 1 | - | 633 | 71.6 | 8.5 | Biological_process unknown | Cytoplasm;Centrosome;Microtubule | Molecular function unknown | - |
| MERTK | NP_006334.2 | 10461 | tyrosine-protein kinase Mer precursor | 3.1 | 1 | 2 | 2 | 6 | 1.56E+09 | 1.30 | 1 | - | 999 | 110.2 | 5.8 | Cell communication;Signal transduction | Plasma membrane | Transmembrane receptor protein tyrosine kinase activity | Plasma membrane |
| NFATC2IP | NP_116204.3 | 84901 | NFATC2-interacting protein | 4.1 | 1 | 1 | 1 | 1 | 1.79E+07 | 1.30 | 1 | - | 419 | 45.8 | 6.6 | Protein metabolism | - | Molecular function unknown | - |
| DLST | NP_001924.2 | 1743 | dihydrolipoyllysine-residue succinyltransferase component of 2-oxoglutarate dehydrogenase complex, | 37.1 | 2 | 10 | 10 | 38 | 5.31E+08 | 1.30 | 8 | 15.5 | 453 | 48.7 | 9.0 | Metabolism;Energy pathways | Mitochondrion;Plasma membrane | Acyltransferase activity | Mitochondrion |
| GSPT2 | NP_060564.2 | 23708 | eukaryotic peptide chain release factor GTP-binding subunit ERF3B | 10.4 | 1 | 1 | 7 | 20 | 8.06E+08 | 1.30 | 1 | - | 628 | 68.8 | 5.4 | Cell communication;Signal transduction | Cytoplasm | Molecular function unknown | Cytoplasm |
| MPI | NP_001276086.1 | 4351 | mannose-6-phosphate isomerase isoform 4 | 22.7 | 4 | 5 | 5 | 10 | 1.37E+08 | 1.30 | 4 | 20.1 | 362 | 39.8 | 5.4 | Metabolism;Energy pathways | Cytoplasm | Isomerase activity | Cytoplasm |
| TAF15 | NP_003478.1 | 8148 | TATA-binding protein-associated factor 2N isoform 2 | 14.1 | 2 | 3 | 5 | 27 | 6.82E+08 | 1.30 | 6 | 11.6 | 589 | 61.5 | 8.0 | Regulation of nucleobase, nucleoside, nucleotide and nucleic acid metabolism | Nucleus;Nucleolus | Transcription regulator activity | Nucleus |
| SF3B4 | NP_005841.1 | 10262 | splicing factor 3B subunit 4 | 16.3 | 1 | 5 | 5 | 23 | 3.46E+08 | 1.30 | 4 | 71.7 | 424 | 44.4 | 8.6 | Regulation of nucleobase, nucleoside, nucleotide and nucleic acid metabolism | Nucleus | Ribonucleoprotein | Nucleus |
| TUBA1C | NP_116093.1 | 84790 | tubulin alpha-1C chain | 83.3 | 1 | 5 | 34 | 1370 | 1.57E+10 | 1.30 | 11 | 10.2 | 449 | 49.9 | 5.1 | Cell growth and/or maintenance | Cytoplasm;Microtubule;Nucleus | Structural constituent of cytoskeleton | Cytoplasm |
| DBNL | NP_001014436.1 | 28988 | drebrin-like protein isoform b | 49.1 | 2 | 1 | 17 | 108 | 1.69E+09 | 1.29 | 1 | - | 430 | 48.2 | 5.1 | Signal transduction | Actin cytoskeleton;Dendrite;Cytoplasm | Kinase regulator activity | Actin cytoskeleton |
| SNAP29 | NP_004773.1 | 9342 | synaptosomal-associated protein 29 | 33.0 | 1 | 6 | 6 | 17 | 2.87E+08 | 1.29 | 8 | 25.5 | 258 | 29 | 5.8 | Transport | Plasma membrane;Nucleus;Cytoplasm;Golgi apparatus;Cytoplasmic vesicle;Zymogen granule | Auxiliary transport protein activity | Plasma membrane |
| DOCK7 | NP_001258930.1 | 85440 | dedicator of cytokinesis protein 7 isoform 4 | 9.3 | 8 | 12 | 13 | 16 | 7.84E+07 | 1.29 | 6 | 17.4 | 2098 | 238.1 | 6.8 | Cell communication;Signal transduction | - | Guanyl-nucleotide exchange factor activity | - |
| SLAIN2 | NP_065897.1 | 57606 | SLAIN motif-containing protein 2 | 10.2 | 1 | 3 | 3 | 3 | 1.55E+07 | 1.29 | 2 | 2.4 | 581 | 62.5 | 9.5 | Biological_process unknown | - | Molecular function unknown | - |
| MYO5A | NP_001135967.1 | 4644 | unconventional myosin-Va isoform 2 | 0.4 | 2 | 1 | 1 | 1 | 1.03E+06 | 1.29 | 1 | - | 1828 | 212.1 | 8.6 | Cell growth and/or maintenance | Cytoplasm | Structural molecule activity | Cytoplasm |
| PQBP1 | NP_001161461.1 | 10084 | polyglutamine-binding protein 1 isoform 4 | 46.6 | 5 | 7 | 7 | 26 | 7.10E+08 | 1.29 | 8 | 16.5 | 264 | 30.4 | 6.3 | Regulation of nucleobase, nucleoside, nucleotide and nucleic acid metabolism | Nucleus;Cytoplasm;Nucleolus | Transcription regulator activity | Nucleus |
| RABIF | NP_002862.2 | 5877 | guanine nucleotide exchange factor MSS4 | 13.0 | 1 | 1 | 1 | 1 | 1.34E+08 | 1.29 | 1 | - | 123 | 13.8 | 5.5 | Cell communication;Signal transduction | Cytoplasm | Guanyl-nucleotide exchange factor activity | Cytoplasm |
| SPATS2L | NP_001269664.1 | 26010 | SPATS2-like protein isoform a | 36.4 | 4 | 14 | 14 | 30 | 4.20E+08 | 1.29 | 11 | 18.3 | 558 | 61.7 | 9.6 | Biological_process unknown | Nucleus | Molecular function unknown | Nucleus |
| CDCA3 | NP_112589.1 | 83461 | cell division cycle-associated protein 3 | 6.7 | 1 | 1 | 1 | 1 | 4.23E+07 | 1.29 | 1 | - | 268 | 29 | 6.4 | Biological_process unknown | Cytoplasm;Nucleus | Molecular function unknown | Cytoplasm |
| UNK | NP_001073888.2 | 85451 | RING finger protein unkempt homolog | 3.6 | 1 | 1 | 1 | 2 | 6.87E+07 | 1.29 | 1 | - | 810 | 88 | 6.9 | Biological_process unknown | - | Molecular function unknown | - |
| ENDOD1 | NP_055851.1 | 23052 | endonuclease domain-containing 1 protein precursor | 11.8 | 1 | 3 | 3 | 5 | 1.31E+07 | 1.29 | 1 | - | 500 | 55 | 5.7 | Regulation of nucleobase, nucleoside, nucleotide and nucleic acid metabolism | - | Molecular function unknown | - |
| SPRYD7 | NP_065189.1 | 57213 | SPRY domain-containing protein 7 isoform 1 | 8.7 | 1 | 1 | 1 | 2 | 4.57E+07 | 1.29 | 1 | - | 196 | 21.7 | 6.7 | Biological_process unknown | - | Molecular function unknown | - |
| HSPH1 | NP_001273432.1 | 10808 | heat shock protein 105 kDa isoform 2 | 68.6 | 4 | 46 | 49 | 218 | 1.56E+09 | 1.29 | 65 | 19.7 | 814 | 92.1 | 5.6 | Protein metabolism | Cytoplasm;Cytosol;Nucleus | Heat shock protein activity;Chaperone activity | Cytoplasm |
| RUVBL1 | NP_003698.1 | 8607 | ruvB-like 1 | 71.7 | 1 | 24 | 24 | 118 | 1.28E+09 | 1.29 | 36 | 16 | 456 | 50.2 | 6.4 | Regulation of nucleobase, nucleoside, nucleotide and nucleic acid metabolism | Nucleus;Plasma membrane;Nucleolus;Chromosome | DNA binding | Nucleus |
| SEC31A | NP_001070675.1 | 22872 | protein transport protein Sec31A isoform 1 | 19.8 | 5 | 18 | 18 | 48 | 4.79E+08 | 1.29 | 13 | 26.5 | 1220 | 132.9 | 6.9 | Transport | Cytoplasm;Golgi apparatus;Endoplasmic reticulum;Nucleus | Transporter activity | Endoplasmic reticulum |
| U2AF2 | NP_001012496.1 | 11338 | splicing factor U2AF 65 kDa subunit isoform b | 49.7 | 2 | 14 | 14 | 65 | 8.28E+08 | 1.29 | 21 | 30.7 | 471 | 53.1 | 9.1 | RNA metabolism | Nucleus;Cytoplasm;Nucleolus | Spliceosomal catalysis | Nucleus |
| MPP6 | NP_057531.2 | 51678 | MAGUK p55 subfamily member 6 | 9.6 | 7 | 4 | 4 | 6 | 7.08E+07 | 1.29 | 3 | 32.3 | 540 | 61.1 | 6.2 | Cell communication;Signal transduction | Plasma membrane | Molecular function unknown | Plasma membrane |
| PHGDH | NP_006614.2 | 26227 | D-3-phosphoglycerate dehydrogenase | 49.9 | 1 | 25 | 25 | 290 | 2.33E+09 | 1.29 | 48 | 15.1 | 533 | 56.6 | 6.7 | Metabolism;Energy pathways | Extracellular;Cytosol | Catalytic activity | Extracellular |

| Gene Symbol | RefSeq_Accession | Entrez Gene ID | Description | Coverage (%) | Proteins | Unique Peptides | # Peptides | PSM | Area | SCC-R /SCC-S | SCC-R /SCC-S (Count) | SCC-R /SCC-S (Variability [%]) | # AAs | MW [kDa] | calc. pI | Biological Process | Localization | Molecular Function | Primary Localization |
|---|---|---|---|---|---|---|---|---|---|---|---|---|---|---|---|---|---|---|---|
| **SRSF7** | NP_001182375.1 | 6432 | **serine/arginine-rich splicing factor 7 isoform 2** | 25.7 | 2 | 6 | 7 | 52 | 1.50E+09 | **1.29** | 9 | 51.1 | 226 | 26 | 11.8 | Regulation of nucleobase, nucleoside, nucleotide and nucleic acid metabolism | Nucleus;Cytoplasm | RNA binding | Nucleus |
| **EXOSC6** | NP_478126.1 | 118460 | **exosome complex component MTR3** | 14.7 | 1 | 3 | 3 | 5 | 1.48E+08 | **1.29** | 3 | 38.8 | 272 | 28.2 | 6.3 | Regulation of nucleobase, nucleoside, nucleotide and nucleic acid metabolism | Nucleus | Ribonuclease activity | Nucleus |
| **PKN2** | NP_006247.1 | 5586 | **serine/threonine-protein kinase N2** | 22.8 | 1 | 12 | 12 | 15 | 7.22E+07 | **1.29** | 6 | 33.4 | 984 | 112 | 6.3 | Cell communication;Signal transduction | Cytoplasm;Extracellular;Nucleus | Protein serine/threonine kinase activity | Cytoplasm |
| **PCM1** | NP_006188.3 | 5108 | **pericentriolar material 1 protein** | 4.7 | 1 | 5 | 5 | 6 | 6.16E+07 | **1.29** | 3 | 37.4 | 2024 | 228.4 | 5.0 | Cell growth and/or maintenance | Centrosome;Cytoplasm;Nucleus | Cytoskeletal protein binding | Centrosome |
| **PSMB8** | NP_004150.1 | 5696 | **proteasome subunit beta type-8 isoform E1 proprotein** | 44.5 | 2 | 6 | 6 | 15 | 3.07E+08 | **1.29** | 7 | 6.1 | 272 | 29.8 | 5.8 | Protein metabolism | Cytoplasm;Nucleus;Endoplasmic reticulum | Ubiquitin-specific protease activity | Cytoplasm |
| **SUGT1** | NP_001124384.1 | 10910 | **suppressor of G2 allele of SKP1 homolog isoform SGT1B** | 50.7 | 2 | 12 | 12 | 24 | 6.06E+08 | **1.29** | 11 | 21.3 | 365 | 41 | 5.2 | Cell communication;Signal transduction;Protein metabolism | Nucleus | Ubiquitin-specific protease activity | Nucleus |
| **WTAP** | NP_001257460.1 | 9589 | **pre-mRNA-splicing regulator WTAP isoform 1** | 8.1 | 3 | 2 | 2 | 4 | 3.40E+07 | **1.28** | 2 | 2.2 | 396 | 44.2 | 5.2 | Biological_process unknown | Nucleus;Cytoplasm | Molecular function unknown | Nucleus;Cytoplasm |
| **AFTPH** | NP_001002243.1 | 54812 | **aftiphilin isoform c** | 4.4 | 3 | 2 | 2 | 4 | 1.90E+07 | **1.28** | 1 | - | 908 | 99.3 | 4.6 | Biological_process unknown | Cytoplasm | Molecular function unknown | Cytoplasm |
| **CSRP1** | NP_001180499.1 | 1465 | **cysteine and glycine-rich protein 1 isoform 3** | 63.6 | 2 | 9 | 9 | 37 | 7.41E+08 | **1.28** | 12 | 11 | 187 | 20 | 8.5 | Cell communication;Signal transduction | Nucleus;Cytoplasm;Nucleolus | Receptor signaling complex scaffold activity | Cytoplasm |
| **CSTF1** | NP_001028694.1 | 1477 | **cleavage stimulation factor subunit 1** | 26.7 | 1 | 6 | 6 | 18 | 4.46E+08 | **1.28** | 8 | 46.7 | 431 | 48.3 | 6.6 | Regulation of nucleobase, nucleoside, nucleotide and nucleic acid metabolism | Nucleus;Nucleolus | RNA binding | Nucleus |
| **FAM49B** | NP_001243692.1 | 51571 | **protein FAM49B** | 45.4 | 2 | 11 | 11 | 24 | 1.96E+08 | **1.28** | 10 | 14.6 | 324 | 36.7 | 6.1 | Biological_process unknown | - | Molecular function unknown | - |
| **SSBP1** | NP_003134.1 | 6742 | **single-stranded DNA-binding protein, mitochondrial precursor** | 34.5 | 1 | 6 | 6 | 14 | 8.54E+07 | **1.28** | 4 | 42.2 | 148 | 17.2 | 9.6 | Regulation of nucleobase, nucleoside, nucleotide and nucleic acid metabolism | Mitochondrion;Nucleus | DNA binding | Mitochondrion |
| **UBA6** | NP_060697.4 | 55236 | **ubiquitin-like modifier-activating enzyme 6** | 28.8 | 1 | 17 | 17 | 35 | 3.65E+08 | **1.28** | 16 | 28.2 | 1052 | 117.9 | 6.1 | Protein metabolism | - | Ubiquitin-specific protease activity | - |
| **YRDC** | NP_078916.3 | 79693 | **yrdC domain-containing protein, mitochondrial precursor** | 18.3 | 1 | 3 | 3 | 5 | 3.56E+07 | **1.28** | 3 | 35.7 | 279 | 29.3 | 8.6 | Biological_process unknown | - | Molecular function unknown | - |
| **PRRC2A** | NP_542417.2 | 7916 | **protein PRRC2A** | 12.9 | 1 | 14 | 15 | 38 | 2.28E+08 | **1.28** | 16 | 49.8 | 2157 | 228.7 | 9.5 | Biological_process unknown | - | Molecular function unknown | - |
| **CIZ1** | NP_001244905.1 | 25792 | **cip1-interacting zinc finger protein isoform 6** | 5.5 | 6 | 2 | 2 | 3 | 5.52E+07 | **1.28** | 1 | - | 797 | 88.5 | 6.0 | Regulation of nucleobase, nucleoside, nucleotide and nucleic acid metabolism | Nucleus;Cytoplasm | DNA binding | Nucleus |
| **RAP1B** | NP_001010942.1 | 5908 | **ras-related protein Rap-1b isoform 1 precursor** | 52.2 | 4 | 3 | 6 | 20 | 1.48E+08 | **1.28** | 3 | 11.1 | 184 | 20.8 | 5.8 | Cell communication;Signal transduction | Nucleus;Cytoplasm;Plasma membrane;Cell projection;Zymogen granule | GTPase activity | Nucleus |
| **TRA2B** | NP_001230808.1 | 6434 | **transformer-2 protein homolog beta isoform 2** | 54.8 | 2 | 6 | 8 | 15 | 2.62E+08 | **1.28** | 5 | 18.5 | 188 | 21.9 | 10.2 | Regulation of nucleobase, nucleoside, nucleotide and nucleic acid metabolism | Nucleus;Nucleolus;Mitochondrion;Cytoplasm | RNA binding | Nucleus |
| **VAMP7** | NP_005629.1 | 6845 | **vesicle-associated membrane protein 7 isoform 1** | 16.8 | 3 | 2 | 2 | 2 | 3.06E+07 | **1.28** | 2 | 72.5 | 220 | 24.9 | 8.6 | Transport | Plasma membrane;Cell surface;Lysosome | Transporter activity | Plasma membrane;Cell surface;Lysosome |
| **RBMS1** | NP_002888.1 | 5937 | **RNA-binding motif, single-stranded-interacting protein 1 isoform c** | 8.2 | 3 | 2 | 3 | 5 | 3.55E+07 | **1.28** | 1 | - | 403 | 44.1 | 8.7 | Regulation of nucleobase, nucleoside, nucleotide and nucleic acid metabolism | Nucleus | DNA binding | Nucleus |
| **GALNT2** | NP_001278795.1 | 2590 | **polypeptide N-acetylgalactosaminyltransferase 2 isoform 2** | 8.3 | 2 | 4 | 4 | 5 | 6.42E+07 | **1.28** | 3 | 7 | 533 | 60.8 | 7.7 | Metabolism;Energy pathways | Golgi apparatus | Galactosyltransferase activity | Golgi apparatus |
| **RPTOR** | NP_001156506.1 | 57521 | **regulatory-associated protein of mTOR isoform 2** | 4.9 | 2 | 3 | 3 | 4 | 1.60E+07 | **1.28** | 2 | 26 | 1177 | 131.4 | 7.0 | Cell communication;Signal transduction | Cytoplasm | Molecular function unknown | Cytoplasm |
| **FPGS** | NP_001018088.1 | 2356 | **folylpolyglutamate synthase, mitochondrial isoform b** | 4.5 | 3 | 1 | 1 | 1 | 9.36E+07 | **1.28** | 1 | - | 537 | 59.1 | 7.4 | Metabolism;Energy pathways;Regulation of nucleobase, nucleoside, nucleotide and nucleic acid metabolism | Cytosol;Mitochondrion | Ligase activity | Cytosol;Mitochondrion |
| **SARS** | NP_006504.2 | 6301 | **serine--tRNA ligase, cytoplasmic** | 51.2 | 1 | 26 | 26 | 115 | 1.40E+09 | **1.28** | 32 | 28.7 | 514 | 58.7 | 6.4 | Metabolism;Energy pathways | Mitochondrion;Cytoplasm | Ligase activity | Cytoplasm |
| **SGTA** | NP_003012.1 | 6449 | **small glutamine-rich tetratricopeptide repeat-containing protein alpha** | 21.1 | 1 | 5 | 5 | 18 | 4.61E+08 | **1.28** | 7 | 14.5 | 313 | 34 | 4.9 | Protein metabolism | Cytoplasm;Nucleus | Chaperone activity | Cytoplasm |
| **WDR18** | NP_077005.2 | 57418 | **WD repeat-containing protein 18** | 16.4 | 1 | 4 | 4 | 12 | 9.85E+07 | **1.28** | 6 | 10.4 | 432 | 47.4 | 6.7 | Biological_process unknown | Nucleolus | Molecular function unknown | Nucleolus |
| **TFCP2** | NP_001166924.1 | 7024 | **alpha-globin transcription factor CP2 isoform 3** | 7.8 | 3 | 1 | 2 | 9 | 5.97E+07 | **1.28** | 4 | 50.3 | 450 | 51.3 | 5.4 | Regulation of nucleobase, nucleoside, nucleotide and nucleic acid metabolism | Nucleus;Cytoplasm | Transcription factor activity | Nucleus |
| **AIFM1** | NP_665811.1 | 9131 | **apoptosis-inducing factor 1, mitochondrial isoform 2 precursor** | 43.8 | 5 | 18 | 18 | 62 | 3.33E+08 | **1.27** | 22 | 20.6 | 609 | 66.3 | 8.9 | Cell communication;Signal transduction | Mitochondrion;Cytoplasm;Nucleus | Oxidoreductase activity | Mitochondrion |
| **PDIA3** | NP_005304.3 | 2923 | **protein disulfide-isomerase A3 precursor** | 71.7 | 1 | 38 | 38 | 265 | 5.52E+09 | **1.27** | 80 | 16.2 | 505 | 56.7 | 6.4 | Protein metabolism | Endoplasmic reticulum;Cytoplasm;Plasma membrane;Mitochondrion | Isomerase activity | Endoplasmic reticulum |
| **RBM10** | NP_001191396.1 | 8241 | **RNA-binding protein 10 isoform 4** | 11.4 | 5 | 5 | 5 | 7 | 3.47E+07 | **1.27** | 3 | 43.8 | 929 | 103.4 | 6.0 | Regulation of nucleobase, nucleoside, nucleotide and nucleic acid metabolism | Nucleus | RNA binding | Nucleus |
| **TK1** | NP_003249.3 | 7083 | **thymidine kinase, cytosolic** | 36.8 | 1 | 6 | 6 | 12 | 2.42E+08 | **1.27** | 7 | 47.9 | 234 | 25.5 | 8.5 | Metabolism;Energy pathways | Cytoplasm | Catalytic activity | Cytoplasm |
| **TM9SF4** | NP_055557.2 | 9777 | **transmembrane 9 superfamily member 4 precursor** | 14.3 | 1 | 7 | 7 | 22 | 1.77E+08 | **1.27** | 10 | 12.2 | 642 | 74.5 | 6.5 | Biological_process unknown | Integral to membrane;Mitochondrion | Molecular function unknown | Integral to membrane |
| **BDH1** | NP_004042.1 | 622 | **D-beta-hydroxybutyrate dehydrogenase, mitochondrial precursor** | 9.0 | 1 | 2 | 2 | 2 | 9.26E+07 | **1.27** | 2 | 34.5 | 343 | 38.1 | 9.0 | Fatty acid metabolism | Mitochondrial membrane | Catalytic activity | Mitochondrial membrane |
| **NEDD8** | NP_006147.1 | 4738 | **NEDD8 precursor** | 49.4 | 2 | 4 | 4 | 27 | 6.49E+08 | **1.27** | 9 | 11.3 | 81 | 9.1 | 8.4 | Protein metabolism | Nucleus;Cytoplasm | Ubiquitin-specific protease activity | Nucleus |
| **OSBPL11** | NP_073613.2 | 114885 | **oxysterol-binding protein-related protein 11** | 8.0 | 1 | 4 | 4 | 5 | 3.85E+07 | **1.27** | 3 | 3.8 | 747 | 83.6 | 7.1 | Transport | Cytoplasm;Nucleus | Transporter activity | Cytoplasm |
| **SEH1L** | NP_112493.2 | 81929 | **nucleoporin SEH1 isoform 2** | 21.7 | 2 | 4 | 4 | 8 | 2.10E+08 | **1.27** | 4 | 26.9 | 360 | 39.6 | 8.1 | Biological_process unknown | Kinetochore;Nuclear membrane | Molecular function unknown | Kinetochore |
| **COPS7B** | NP_073567.1 | 64708 | **COP9 signalosome complex subunit 7b isoform b** | 34.1 | 5 | 5 | 5 | 9 | 8.31E+07 | **1.27** | 3 | 4.8 | 264 | 29.6 | 6.2 | Cell communication;Signal transduction | Cytoplasm | Molecular function unknown | Cytoplasm |

| Gene Symbol | RefSeq_Accession | Entrez Gene ID | Description | Coverage (%) | Proteins | Unique Peptides | # Peptides | PSM | Area | SCC-R /SCC-S | SCC-R /SCC-S (Count) | SCC-R /SCC-S (Variability [%]) | # AAs | MW [kDa] | calc. pI | Biological Process | Localization | Molecular Function | Primary Localization |
|---|---|---|---|---|---|---|---|---|---|---|---|---|---|---|---|---|---|---|---|
| **MKLN1** | NP_001138826.1 | 4289 | **muskelin isoform 1** | 1.7 | 2 | 1 | 1 | 1 | 5.10E+06 | **1.27** | 1 | - | 712 | 82.4 | 6.3 | Cell growth and/or maintenance | Cytoplasm;Plasma membrane | Cell adhesion molecule activity | Cytoplasm |
| **ARF1** | NP_001019398.1 | 375 | **ADP-ribosylation factor 1** | 87.9 | 7 | 12 | 19 | 140 | 2.00E+09 | **1.27** | 25 | 11.7 | 181 | 20.7 | 6.8 | Signal transduction;Endosome transport | Cytoplasm;Golgi apparatus;Plasma membrane;Endosome;Golgi membrane | GTPase activity | Golgi apparatus |
| **ASPSCR1** | NP_076988.1 | 79058 | **tether containing UBX domain for GLUT4 isoform 1** | 18.3 | 2 | 6 | 6 | 9 | 9.30E+07 | **1.27** | 4 | 10.5 | 553 | 60.1 | 6.6 | Biological_process unknown | Nucleus;Plasma membrane;Cytoplasm | Molecular function unknown | Cytoplasm |
| **CCAR1** | NP_001269888.1 | 55749 | **cell division cycle and apoptosis regulator protein 1 isoform b** | 11.1 | 2 | 8 | 8 | 20 | 6.01E+07 | **1.27** | 10 | 39.6 | 1135 | 131 | 5.8 | Cell communication;Signal transduction;Regulation of cell cycle | Cytoplasm | Molecular function unknown | Cytoplasm |
| **EPS8** | NP_004438.3 | 2059 | **epidermal growth factor receptor kinase substrate 8** | 3.0 | 1 | 2 | 2 | 4 | 3.64E+07 | **1.27** | 1 | - | 822 | 91.8 | 7.5 | Cell communication;Signal transduction | Cytoplasm;Plasma membrane;Nucleus | Receptor signaling complex scaffold activity | Cytoplasm |
| **MAGOH** | NP_002361.1 | 4116 | **protein mago nashi homolog** | 50.0 | 2 | 8 | 8 | 59 | 9.06E+08 | **1.27** | 11 | 21 | 146 | 17.2 | 6.1 | Cell communication;Signal transduction | Nucleus;Cytoplasm | Protein binding | Nucleus |
| **RUVBL2** | NP_006657.1 | 10856 | **ruvB-like 2** | 62.6 | 1 | 24 | 24 | 77 | 8.65E+08 | **1.27** | 22 | 23.9 | 463 | 51.1 | 5.6 | Regulation of nucleobase, nucleoside, nucleotide and nucleic acid metabolism | Cytoplasm;Nucleus;Nucleolus;Chromosome | Transcription regulator activity | Nucleus |
| **PPP1R8** | NP_054829.2 | 5511 | **nuclear inhibitor of protein phosphatase 1 isoform alpha** | 26.2 | 2 | 4 | 4 | 6 | 4.05E+07 | **1.27** | 3 | 6.9 | 351 | 38.5 | 7.4 | RNA metabolism | Nucleus;Cytoplasm | RNA binding | Nucleus |
| **IGBP1** | NP_001542.1 | 3476 | **immunoglobulin-binding protein 1** | 38.9 | 1 | 8 | 8 | 14 | 1.09E+08 | **1.27** | 9 | 11 | 339 | 39.2 | 5.4 | Cell communication;Signal transduction | Cytoplasm;Nucleus | Molecular function unknown | Cytoplasm |
| **RAB3GAP2** | NP_036546.2 | 25782 | **rab3 GTPase-activating protein non-catalytic subunit** | 16.1 | 1 | 13 | 13 | 28 | 9.42E+07 | **1.27** | 12 | 38.4 | 1393 | 155.9 | 5.6 | Cell communication;Signal transduction | Microsome;Cytoplasm;Mitochondrion;Cytoplasmic vesicle | GTPase activator activity | Cytoplasmic vesicle |
| **TTC1** | NP_003305.1 | 7265 | **tetratricopeptide repeat protein 1** | 13.0 | 1 | 1 | 1 | 2 | 3.22E+08 | **1.27** | 1 | - | 292 | 33.5 | 4.8 | Cell communication;Signal transduction | - | Receptor signaling complex scaffold activity | - |
| **PPP1R14B** | NP_619634.1 | 26472 | **protein phosphatase 1 regulatory subunit 14B** | 51.0 | 2 | 5 | 5 | 35 | 2.88E+08 | **1.26** | 8 | 68.5 | 147 | 15.9 | 4.9 | Metabolism;Energy pathways | Cytoplasm | Phosphatase regulator activity | Cytoplasm |
| **BMP2K** | NP_060063.2 | 55589 | **BMP-2-inducible protein kinase isoform b** | 5.7 | 2 | 2 | 2 | 4 | 2.44E+07 | **1.26** | 1 | - | 662 | 73.8 | 7.9 | Cell communication;Signal transduction | Nucleus | Protein serine/threonine kinase activity | Nucleus |
| **GSS** | NP_000169.1 | 2937 | **glutathione synthetase** | 45.6 | 1 | 16 | 16 | 49 | 7.07E+08 | **1.26** | 17 | 28.3 | 474 | 52.4 | 5.9 | Metabolism;Energy pathways | - | Ligase activity | - |
| **ATP2C1** | NP_001001485.1 | 27032 | **calcium-transporting ATPase type 2C member 1 isoform 1c** | 13.7 | 9 | 8 | 8 | 9 | 1.10E+08 | **1.26** | 8 | 38.5 | 888 | 96.9 | 6.3 | Ion transport | Golgi apparatus | ATPase activity | Golgi apparatus |
| **CD2BP2** | NP_001230575.1 | 10421 | **CD2 antigen cytoplasmic tail-binding protein 2** | 20.5 | 1 | 4 | 4 | 6 | 1.55E+08 | **1.26** | 4 | 22.9 | 341 | 37.6 | 4.6 | Cell communication;Signal transduction | Cytoplasm;Nucleus | Molecular function unknown | Cytoplasm |
| **NAA20** | NP_852668.1 | 51126 | **N-alpha-acetyltransferase 20 isoform b** | 9.0 | 2 | 1 | 1 | 1 | 1.37E+08 | **1.26** | 1 | - | 166 | 18.8 | 5.0 | Cell proliferation;Energy pathways | Cytoplasm | Acyltransferase activity | Cytoplasm |
| **PSMC4** | NP_006494.1 | 5704 | **26S protease regulatory subunit 6B isoform 1** | 62.7 | 2 | 20 | 20 | 117 | 7.06E+08 | **1.26** | 32 | 16.8 | 418 | 47.3 | 5.2 | Protein metabolism | Nucleus | Ubiquitin-specific protease activity | Nucleus |
| **LCMT1** | NP_057393.2 | 51451 | **leucine carboxyl methyltransferase 1 isoform a** | 7.8 | 2 | 2 | 2 | 2 | 1.70E+07 | **1.26** | 1 | - | 334 | 38.4 | 6.0 | Protein modification;Metabolism;Energy pathways | - | Methyltransferase activity | - |
| **MOV10** | NP_001123551.1 | 4343 | **putative helicase MOV-10 isoform 1** | 31.2 | 2 | 22 | 22 | 48 | 5.99E+08 | **1.26** | 19 | 18.6 | 1003 | 113.6 | 8.8 | Biological_process unknown | Nucleus;Cytoplasm | Molecular function unknown | Nucleus;Cytoplasm |
| **LAS1L** | NP_001164121.1 | 81887 | **ribosomal biogenesis protein LAS1L isoform 3** | 10.5 | 3 | 4 | 4 | 8 | 6.01E+07 | **1.26** | 3 | 28.8 | 675 | 76.8 | 4.7 | Cell communication;Signal transduction | Nucleus;Nucleolus | Molecular function unknown | Nucleus |
| **EIF2B4** | NP_056451.3 | 8890 | **translation initiation factor eIF-2B subunit delta isoform 3** | 15.3 | 3 | 4 | 4 | 11 | 3.89E+07 | **1.26** | 2 | 14.5 | 522 | 57.4 | 9.4 | Protein metabolism | Cytoplasm;Cytosol | Translation regulator activity | Cytoplasm |
| **ESYT2** | NP_065779.1 | 57488 | **extended synaptotagmin-2** | 18.1 | 1 | 10 | 10 | 15 | 1.67E+08 | **1.26** | 9 | 41 | 893 | 98.8 | 8.7 | Biological_process unknown | Mitochondrion | Molecular function unknown | Mitochondrion |
| **COA3** | NP_001035521.1 | 28958 | **cytochrome c oxidase assembly factor 3 homolog, mitochondrial** | 20.8 | 1 | 2 | 2 | 4 | 3.30E+08 | **1.26** | 2 | 9 | 106 | 11.7 | 9.6 | Biological_process unknown | Integral to membrane | Molecular function unknown | Integral to membrane |
| **CRELD2** | NP_001271247.1 | 79174 | **cysteine-rich with EGF-like domain protein 2 isoform d precursor** | 10.9 | 4 | 1 | 1 | 1 | 3.26E+07 | **1.26** | 1 | - | 321 | 34.9 | 4.6 | Cell communication;Signal transduction | Extracellular | Calcium ion binding | Extracellular |
| **NARS2** | NP_078954.4 | 79731 | **probable asparagine--tRNA ligase, mitochondrial isoform 1 precursor** | 10.5 | 2 | 2 | 2 | 2 | 1.42E+07 | **1.26** | 1 | - | 477 | 54.1 | 7.2 | Biological_process unknown | - | Ligase activity | - |
| **UBE4A** | NP_001191006.1 | 9354 | **ubiquitin conjugation factor E4 A isoform 2** | 2.4 | 2 | 1 | 1 | 1 | 5.01E+07 | **1.26** | 1 | - | 1066 | 122.5 | 5.2 | Protein metabolism | - | Ubiquitin-specific protease activity | - |
| **NAGK** | NP_060037.3 | 55577 | **N-acetyl-D-glucosamine kinase** | 38.0 | 1 | 9 | 9 | 19 | 2.25E+08 | **1.26** | 8 | 25.5 | 390 | 42 | 6.7 | Metabolism;Energy pathways | - | Catalytic activity | - |
| **RPRD1B** | NP_067038.1 | 58490 | **regulation of nuclear pre-mRNA domain-containing protein 1B** | 50.0 | 2 | 10 | 10 | 38 | 4.05E+08 | **1.26** | 9 | 35.3 | 326 | 36.9 | 6.0 | Regulation of nucleobase, nucleoside, nucleotide and nucleic acid metabolism | Nucleus | Molecular function unknown | Nucleus |
| **ENO1** | NP_001188412.1 | 2023 | **c-myc promoter-binding protein-1 isoform MBP-1** | 80.1 | 1 | 1 | 29 | 937 | 2.04E+10 | **1.25** | 1 | - | 341 | 36.9 | 6.3 | Metabolism;Energy pathways | Cytoplasm;Plasma membrane;Nucleus;Extracellular | Catalytic activity | Cytoplasm |
| **NDUFS3** | NP_004542.1 | 4722 | **NADH dehydrogenase [ubiquinone] iron-sulfur protein 3, mitochondrial precursor** | 37.9 | 1 | 8 | 8 | 19 | 2.98E+08 | **1.25** | 7 | 13.4 | 264 | 30.2 | 7.5 | Metabolism;Energy pathways | Mitochondrion | Catalytic activity | Mitochondrion |
| **SURF6** | NP_006744.2 | 6838 | **surfeit locus protein 6 isoform 1** | 22.4 | 2 | 3 | 3 | 11 | 3.84E+08 | **1.25** | 5 | 2.4 | 361 | 41.4 | 10.6 | Regulation of nucleobase, nucleoside, nucleotide and nucleic acid metabolism | Nucleolus;Cytoplasm | RNA binding | Nucleolus |
| **TUBA1B** | NP_006073.2 | 10376 | **tubulin alpha-1B chain** | 83.4 | 2 | 2 | 35 | 1557 | 1.68E+10 | **1.25** | 3 | 29 | 451 | 50.1 | 5.1 | Cell growth and/or maintenance | Cytoplasm;Mitochondrion;Plasma membrane | Structural molecule activity | Cytoplasm;Plasma membrane |
| **COA6** | NP_001193570.1 | 388753 | **cytochrome c oxidase assembly factor 6 homolog isoform 2** | 51.9 | 2 | 4 | 4 | 8 | 6.97E+07 | **1.25** | 5 | 58 | 79 | 9.4 | 7.7 | Metabolism;Energy pathways | Mitochondrion | Catalytic activity | Mitochondrion |
| **YWHAG** | NP_036611.2 | 7532 | **14-3-3 protein gamma** | 88.7 | 1 | 15 | 23 | 234 | 1.80E+10 | **1.25** | 24 | 19.2 | 247 | 28.3 | 4.9 | Cell communication;Signal transduction | Cytoplasm;Endoplasmic reticulum;Golgi apparatus;Nucleus | Receptor signaling complex scaffold activity | Cytoplasm |
| **HMGN5** | NP_110390.1 | 79366 | **high mobility group nucleosome-binding domain-containing protein 5** | 12.8 | 1 | 2 | 2 | 3 | 6.32E+07 | **1.25** | 2 | 19.4 | 282 | 31.5 | 4.6 | Regulation of nucleobase, nucleoside, nucleotide and nucleic acid metabolism | Nucleus | Transcription regulator activity | Nucleus |
| **PRMT1** | NP_938074.2 | 3276 | **protein arginine N-methyltransferase 1 isoform 3** | 63.5 | 5 | 18 | 18 | 80 | 1.18E+09 | **1.25** | 24 | 17.6 | 353 | 40.5 | 5.5 | Metabolism;Energy pathways | Nucleus;Cytoplasm | Methyltransferase activity | Nucleus |

| Gene Symbol | RefSeq_Accession | Entrez Gene ID | Description | Coverage (%) | Proteins | Unique Peptides | # Peptides | PSM | Area | SCC-R /SCC-S | SCC-R /SCC-S (Count) | SCC-R /SCC-S (Variability [%]) | # AAs | MW [kDa] | calc. pI | Biological Process | Localization | Molecular Function | Primary Localization |
|---|---|---|---|---|---|---|---|---|---|---|---|---|---|---|---|---|---|---|---|
| **USP40** | NP_060688.1 | 55230 | **ubiquitin carboxyl-terminal hydrolase 40** | 1.8 | 1 | 1 | 1 | 1 | 1.88E+06 | **1.25** | 1 | - | 1247 | 141.5 | 5.7 | Protein metabolism | - | Ubiquitin-specific protease activity | - |
| **PURA** | NP_005850.1 | 5813 | **transcriptional activator protein Pur-alpha** | 25.8 | 1 | 3 | 3 | 6 | 1.04E+08 | **1.25** | 3 | 40.2 | 322 | 34.9 | 6.4 | Regulation of nucleobase, nucleoside, nucleotide and nucleic acid metabolism | Nucleus;Cytoplasm | Transcription factor activity | Nucleus |
| **TSNAX** | NP_005990.1 | 7257 | **translin-associated protein X** | 45.2 | 1 | 9 | 9 | 22 | 2.70E+08 | **1.25** | 15 | 12.1 | 290 | 33.1 | 6.6 | Regulation of nucleobase, nucleoside, nucleotide and nucleic acid metabolism | Cytoplasm;Nucleus | Transporter activity | Cytoplasm |
| **CHRAC1** | NP_059140.1 | 54108 | **chromatin accessibility complex protein 1** | 44.3 | 1 | 3 | 3 | 5 | 2.31E+07 | **1.25** | 2 | 39.1 | 131 | 14.7 | 5.1 | Regulation of nucleobase, nucleoside, nucleotide and nucleic acid metabolism | Nucleus | DNA binding | Nucleus |
| **ZCCHC11** | NP_056084.1 | 23318 | **terminal uridylyltransferase 4 isoform b** | 1.3 | 2 | 1 | 1 | 2 | 1.15E+07 | **1.25** | 1 | - | 1644 | 185 | 8.0 | Regulation of nucleobase, nucleoside, nucleotide and nucleic acid metabolism | Nucleus | DNA binding;Metal ion binding | Nucleus |
| **CLNS1A** | NP_001284.1 | 1207 | **methylosome subunit pICln** | 61.2 | 1 | 8 | 8 | 22 | 2.98E+08 | **1.25** | 5 | 13.2 | 237 | 26.2 | 4.1 | Transport | Plasma membrane;Nucleus;Cytoplasm | Transporter activity | Plasma membrane |
| **MGST3** | NP_004519.1 | 4259 | **microsomal glutathione S-transferase 3** | 33.6 | 1 | 4 | 4 | 23 | 8.11E+08 | **1.25** | 7 | 8 | 152 | 16.5 | 9.4 | Metabolism;Energy pathways | Endoplasmic reticulum;Cytosol | Glutathione transferase activity | Endoplasmic reticulum |
| **PPIL4** | NP_624311.1 | 85313 | **peptidyl-prolyl cis-trans isomerase-like 4** | 29.9 | 1 | 8 | 8 | 17 | 7.66E+07 | **1.25** | 7 | 12.5 | 492 | 57.2 | 5.9 | Protein metabolism | Nucleus | Chaperone activity | Nucleus |
| **PUM1** | NP_055491.1 | 9698 | **pumilio homolog 1 isoform 2** | 6.2 | 2 | 4 | 5 | 6 | 5.32E+07 | **1.25** | 2 | 5.2 | 1186 | 126.4 | 6.8 | Regulation of translation | Nucleus;Cytoplasm | RNA binding | Nucleus;Cytoplasm |
| **MED20** | NP_004266.2 | 9477 | **mediator of RNA polymerase II transcription subunit 20** | 16.0 | 1 | 2 | 2 | 3 | 7.90E+06 | **1.25** | 2 | 7.3 | 212 | 23.2 | 6.9 | Regulation of nucleobase, nucleoside, nucleotide and nucleic acid metabolism | - | Transcription regulator activity | - |
| **STX10** | NP_001258540.1 | 8677 | **syntaxin-10 isoform 4** | 20.5 | 4 | 2 | 2 | 5 | 7.42E+07 | **1.25** | 2 | 41.9 | 200 | 22.4 | 4.6 | Transport | Golgi apparatus | Transporter activity | Golgi apparatus |
| **PSMD1** | NP_001177966.1 | 5707 | **26S proteasome non-ATPase regulatory subunit 1 isoform 2** | 49.0 | 2 | 34 | 34 | 84 | 1.62E+09 | **1.25** | 36 | 26.1 | 922 | 102.2 | 5.3 | Protein metabolism;Proteolysis and peptidolysis | Cytoplasm;Cytosol;Nucleus | Ubiquitin-specific protease activity | Cytoplasm |
| **ZRANB2** | NP_005446.2 | 9406 | **zinc finger Ran-binding domain-containing protein 2 isoform 2** | 10.3 | 2 | 3 | 3 | 5 | 1.86E+08 | **1.25** | 3 | 11 | 320 | 36.3 | 9.8 | Regulation of nucleobase, nucleoside, nucleotide and nucleic acid metabolism | Nucleus;Cytoplasm | RNA binding | Nucleus;Cytoplasm |
| **TELO2** | NP_057195.2 | 9894 | **telomere length regulation protein TEL2 homolog** | 3.9 | 1 | 2 | 2 | 2 | 4.23E+07 | **1.24** | 1 | - | 837 | 91.7 | 5.8 | Biological_process unknown | - | Molecular function unknown | - |
| **LAMC1** | NP_002284.3 | 3915 | **laminin subunit gamma-1 precursor** | 14.5 | 1 | 11 | 11 | 23 | 1.12E+08 | **1.24** | 8 | 29.1 | 1609 | 177.5 | 5.1 | Cell growth and/or maintenance | Extracellular;Plasma membrane | Extracellular matrix structural constituent | Extracellular |
| **TMEM109** | NP_076997.1 | 79073 | **transmembrane protein 109 precursor** | 4.9 | 1 | 1 | 1 | 4 | 3.38E+08 | **1.24** | 2 | 7.5 | 243 | 26.2 | 10.5 | Biological_process unknown | - | Molecular function unknown | - |
| **UBE2N** | NP_003339.1 | 7334 | **ubiquitin-conjugating enzyme E2 N** | 59.2 | 2 | 8 | 8 | 40 | 1.35E+09 | **1.24** | 14 | 30.4 | 152 | 17.1 | 6.6 | Protein metabolism | Nucleus;Plasma membrane;Cytosol | Ubiquitin-specific protease activity | Nucleus |
| **UBE2V2** | NP_003341.1 | 7336 | **ubiquitin-conjugating enzyme E2 variant 2** | 69.7 | 2 | 4 | 9 | 41 | 6.89E+08 | **1.24** | 3 | 7.6 | 145 | 16.4 | 8.1 | Protein metabolism | Cytoplasm;Nucleus | Ubiquitin-specific protease activity | Cytoplasm |
| **LRRFIP1** | NP_001131025.1 | 9208 | **leucine-rich repeat flightless-interacting protein 1 isoform 5** | 53.5 | 5 | 24 | 25 | 73 | 5.91E+08 | **1.24** | 32 | 19.8 | 752 | 82.6 | 4.6 | Regulation of nucleobase, nucleoside, nucleotide and nucleic acid metabolism | Cytoplasm;Nucleus | Transcription regulator activity | Cytoplasm |
| **NRBP1** | NP_037524.1 | 29959 | **nuclear receptor-binding protein** | 20.6 | 1 | 7 | 7 | 24 | 3.51E+08 | **1.24** | 8 | 25.6 | 535 | 59.8 | 5.1 | Cell communication;Signal transduction | Nucleus;Endosome;Plasma membrane;Cytoplasm | Receptor signaling complex scaffold activity | Nucleus |
| **LYAR** | NP_001139197.1 | 55646 | **cell growth-regulating nucleolar protein** | 38.3 | 1 | 10 | 10 | 35 | 3.08E+08 | **1.24** | 12 | 13.3 | 379 | 43.6 | 9.6 | Regulation of nucleobase, nucleoside, nucleotide and nucleic acid metabolism | Nucleolus | Transcription regulator activity | Nucleolus |
| **NUDT9** | NP_932155.1 | 53343 | **ADP-ribose pyrophosphatase, mitochondrial isoform b** | 24.3 | 3 | 4 | 4 | 6 | 4.87E+07 | **1.24** | 3 | 52.3 | 300 | 33.8 | 6.8 | Ion transport | Mitochondrion;Cytoplasm | Catalytic activity | Mitochondrion;Cytoplasm |
| **SDHB** | NP_002991.2 | 6390 | **succinate dehydrogenase [ubiquinone] iron-sulfur subunit, mitochondrial precursor** | 29.6 | 1 | 5 | 5 | 8 | 2.55E+08 | **1.24** | 5 | 2.4 | 280 | 31.6 | 8.8 | Metabolism;Energy pathways | Mitochondrion | Catalytic activity | Mitochondrion |
| **LAMTOR5** | NP_006393.2 | 10542 | **ragulator complex protein LAMTOR5** | 11.6 | 1 | 1 | 1 | 8 | 6.61E+08 | **1.24** | 2 | 10.8 | 173 | 18.1 | 5.5 | Immune response | Cytosol;Nucleus | Molecular function unknown | Cytosol |
| **PSMD6** | NP_055629.1 | 9861 | **26S proteasome non-ATPase regulatory subunit 6 isoform 2** | 37.5 | 4 | 12 | 12 | 34 | 1.16E+09 | **1.24** | 15 | 20.4 | 389 | 45.5 | 5.6 | Protein metabolism | Peroxisome | Ubiquitin-specific protease activity | Peroxisome |
| **STIP1** | NP_006810.1 | 10963 | **stress-induced-phosphoprotein 1 isoform b** | 52.5 | 3 | 34 | 34 | 206 | 2.50E+09 | **1.24** | 61 | 17.1 | 543 | 62.6 | 6.8 | Cell communication;Signal transduction | Nucleus;Golgi apparatus;Cytoplasm | Receptor signaling complex scaffold activity | Nucleus |
| **TPM4** | NP_003281.1 | 7171 | **tropomyosin alpha-4 chain isoform 2** | 81.5 | 1 | 7 | 32 | 302 | 8.25E+09 | **1.24** | 11 | 9.6 | 248 | 28.5 | 4.7 | Cell growth and/or maintenance | Cytoskeleton | Structural constituent of cytoskeleton | Cytoskeleton |
| **PRPS1** | NP_002755.1 | 5631 | **ribose-phosphate pyrophosphokinase 1 isoform 1** | 39.3 | 2 | 5 | 8 | 17 | 1.69E+08 | **1.24** | 7 | 16.1 | 318 | 34.8 | 7.0 | Metabolism;Energy pathways | - | Ligase activity | - |
| **RPE** | NP_954699.1 | 6120 | **ribulose-phosphate 3-epimerase isoform 1** | 24.1 | 6 | 4 | 4 | 13 | 1.56E+08 | **1.24** | 7 | 7.4 | 228 | 24.9 | 5.6 | Metabolism;Energy pathways | Cytoplasm | Racemase and epimerase activity | Cytoplasm |
| **EIF2B2** | NP_055054.1 | 8892 | **translation initiation factor eIF-2B subunit beta** | 4.6 | 1 | 1 | 1 | 3 | 9.60E+07 | **1.24** | 1 | - | 351 | 39 | 6.2 | Protein metabolism | Cytoplasm | Translation regulator activity | Cytoplasm |
| **FUBP1** | NP_003893.2 | 8880 | **far upstream element-binding protein 1** | 56.4 | 1 | 27 | 28 | 133 | 1.69E+09 | **1.24** | 39 | 21.6 | 644 | 67.5 | 7.6 | Regulation of nucleobase, nucleoside, nucleotide and nucleic acid metabolism | Nucleus;Cytoplasm | Transcription regulator activity | Nucleus |
| **GRB2** | NP_002077.1 | 2885 | **growth factor receptor-bound protein 2 isoform 1** | 25.4 | 2 | 3 | 3 | 4 | 3.81E+07 | **1.24** | 2 | 30.6 | 217 | 25.2 | 6.3 | Signal transduction;Apoptosis;Regulation of cell cycle | Cytoplasm;Nucleus;Plasma membrane | Receptor signaling complex scaffold activity | Cytoplasm |
| **NCAPG** | NP_071741.2 | 64151 | **condensin complex subunit 3** | 18.5 | 1 | 13 | 13 | 27 | 2.37E+08 | **1.24** | 12 | 14.3 | 1015 | 114.3 | 5.6 | Regulation of nucleobase, nucleoside, nucleotide and nucleic acid metabolism | Nucleus;Cytoplasm | DNA binding | Nucleus |
| **NELFE** | NP_002895.3 | 7936 | **negative elongation factor E** | 17.9 | 1 | 4 | 4 | 8 | 4.33E+07 | **1.24** | 3 | 5.7 | 380 | 43.2 | 9.3 | Regulation of nucleobase, nucleoside, nucleotide and nucleic acid metabolism | Nucleus;Cytoplasm | RNA binding | Nucleus;Cytoplasm |
| **SUMO1** | NP_001005781.1 | 7341 | **small ubiquitin-related modifier 1 isoform a precursor** | 28.7 | 2 | 3 | 3 | 7 | 2.30E+08 | **1.23** | 3 | 0.6 | 101 | 11.5 | 5.5 | Regulation of cell cycle;Apoptosis;Protein modification | Nucleus;Cytoplasm;Microtubule | Protein binding | Cytoplasm |
| **COPS6** | NP_006824.2 | 10980 | **COP9 signalosome complex subunit 6** | 19.3 | 1 | 4 | 4 | 11 | 1.52E+08 | **1.23** | 4 | 6.3 | 327 | 36.1 | 5.7 | Cell communication;Signal transduction | Cytoplasm;Nucleus | Protein binding | Cytoplasm |
| **DHX33** | NP_001186628.1 | 56919 | **putative ATP-dependent RNA helicase DHX33 isoform 2** | 4.7 | 2 | 1 | 1 | 2 | 5.75E+06 | **1.23** | 1 | - | 534 | 60.4 | 8.3 | Regulation of nucleobase, nucleoside, nucleotide and nucleic acid metabolism | Nucleolus;Nucleus | Molecular function unknown | Nucleolus |

Supplementary Table S4. List of all proteins identified in SCC-S and SCC-R cells using SequestHT and Mascot search algorithms and their gene ontology annotation

| Gene Symbol | RefSeq_Accession | Entrez Gene ID | Description | Coverage (%) | Proteins | Unique Peptides | # Peptides | PSM | Area | SCC-R /SCC-S | SCC-R /SCC-S (Count) | SCC-R /SCC-S (Variability [%]) | # AAs | MW [kDa] | calc. pI | Biological Process | Localization | Molecular Function | Primary Localization |
|---|---|---|---|---|---|---|---|---|---|---|---|---|---|---|---|---|---|---|---|
| **EXOSC9** | NP_005024.2 | 5393 | **exosome complex component RRP45 isoform 2** | 11.6 | 2 | 3 | 3 | 8 | 1.34E+08 | **1.23** | 2 | 53.6 | 439 | 48.9 | 5.3 | Regulation of nucleobase, nucleoside, nucleotide and nucleic acid metabolism | Nucleoplasm;Nucleolus;Nucleus;Cytoplasm | Ribonuclease activity | Nucleolus |
| **MGMT** | NP_002403.2 | 4255 | **methylated-DNA--protein-cysteine methyltransferase** | 13.5 | 1 | 2 | 2 | 3 | 2.47E+07 | **1.23** | 1 | - | 238 | 25 | 8.7 | Regulation of nucleobase, nucleoside, nucleotide and nucleic acid metabolism | Nucleus;Endoplasmic reticulum;Cytoplasm | DNA repair protein | Nucleus |
| **OGDH** | NP_002532.2 | 4967 | **2-oxoglutarate dehydrogenase, mitochondrial isoform 1 precursor** | 39.6 | 3 | 18 | 22 | 59 | 7.74E+08 | **1.23** | 20 | 9.2 | 1023 | 115.9 | 6.9 | Metabolism;Energy pathways | Mitochondrion | Catalytic activity | Mitochondrion |
| **POLR1B** | NP_001269703.1 | 84172 | **DNA-directed RNA polymerase I subunit RPA2 isoform 4** | 4.0 | 6 | 2 | 2 | 4 | 5.63E+07 | **1.23** | 2 | 53.4 | 952 | 107.8 | 7.6 | Regulation of nucleobase, nucleoside, nucleotide and nucleic acid metabolism | Nucleus | DNA-directed RNA polymerase activity | Nucleus |
| **ACAT1** | NP_000010.1 | 38 | **acetyl-CoA acetyltransferase, mitochondrial precursor** | 54.3 | 1 | 15 | 15 | 53 | 4.51E+08 | **1.23** | 19 | 19.3 | 427 | 45.2 | 8.9 | Metabolism;Energy pathways | Mitochondrion;Cytoplasm | Acyltransferase activity | Mitochondrion |
| **TSPYL1** | NP_003300.1 | 7259 | **testis-specific Y-encoded-like protein 1** | 14.7 | 1 | 3 | 3 | 7 | 1.06E+08 | **1.23** | 2 | 6.4 | 437 | 49.2 | 5.5 | Biological_process unknown | Nucleolus;Nucleus | Molecular function unknown | Nucleus |
| **DYNLT1** | NP_006510.1 | 6993 | **dynein light chain Tctex-type 1 isoform 1** | 79.7 | 3 | 5 | 5 | 24 | 1.48E+08 | **1.23** | 8 | 17.3 | 113 | 12.4 | 5.1 | Biological_process unknown | Cytoplasm;Cytosol | Molecular function unknown | Cytoplasm |
| **STX12** | NP_803173.1 | 23673 | **syntaxin-12** | 17.0 | 1 | 3 | 3 | 6 | 6.76E+07 | **1.23** | 3 | 25.5 | 276 | 31.6 | 5.6 | Transport | Endosome;Plasma membrane;Nucleus | Auxiliary transport protein activity | Endosome |
| **USP36** | NP_079366.3 | 57602 | **ubiquitin carboxyl-terminal hydrolase 36** | 4.3 | 1 | 2 | 2 | 3 | 6.13E+06 | **1.23** | 1 | - | 1123 | 122.8 | 9.7 | Protein metabolism | Nucleus;Nucleolus | Ubiquitin-specific protease activity | Nucleus |
| **MARS** | NP_004981.2 | 4141 | **methionine--tRNA ligase, cytoplasmic** | 44.8 | 1 | 26 | 26 | 93 | 9.69E+08 | **1.23** | 33 | 29.2 | 900 | 101.1 | 6.2 | Protein metabolism | Cytoplasm;Nucleus;Mitochondrion;Cytosol | Ligase activity | Cytoplasm |
| **NUBP2** | NP_001271430.1 | 10101 | **cytosolic Fe-S cluster assembly factor NUBP2 isoform 2** | 37.0 | 2 | 3 | 3 | 10 | 3.26E+07 | **1.23** | 7 | 19 | 211 | 22.8 | 5.1 | Protein metabolism | Cytoplasm | Molecular function unknown | Cytoplasm |
| **PPP2CB** | NP_001009552.1 | 5516 | **serine/threonine-protein phosphatase 2A catalytic subunit beta isoform** | 54.7 | 1 | 1 | 11 | 43 | 5.06E+08 | **1.23** | 1 | - | 309 | 35.6 | 5.4 | Cell communication;Signal transduction | Cytoplasm;Nucleus | Protein serine/threonine phosphatase activity | Cytoplasm |
| **RPLP1** | NP_000994.1 | 6176 | **60S acidic ribosomal protein P1 isoform 1** | 79.0 | 1 | 4 | 4 | 70 | 1.94E+09 | **1.23** | 13 | 54.9 | 114 | 11.5 | 4.3 | Protein metabolism | Ribosome;Cytoplasm;Mitochondrion;Nucleus | Structural constituent of ribosome | Ribosome |
| **TRIM25** | NP_005073.2 | 7706 | **E3 ubiquitin/ISG15 ligase TRIM25** | 35.7 | 1 | 15 | 15 | 47 | 5.07E+08 | **1.23** | 16 | 19.3 | 630 | 70.9 | 8.1 | Regulation of nucleobase, nucleoside, nucleotide and nucleic acid metabolism | Nucleus;Cytoplasm | Transcription factor activity | Nucleus |
| **VPS26A** | NP_004887.2 | 9559 | **vacuolar protein sorting-associated protein 26A isoform 1** | 47.1 | 2 | 10 | 10 | 35 | 6.14E+08 | **1.23** | 6 | 14.1 | 327 | 38.1 | 6.6 | Transport | Cytoplasm;Integral to membrane;Endoplasmic reticulum | Transporter activity | Cytoplasm |
| **KATNB1** | NP_005877.2 | 10300 | **katanin p80 WD40 repeat-containing subunit B1** | 1.5 | 1 | 1 | 1 | 1 | 5.31E+06 | **1.23** | 1 | - | 655 | 72.3 | 7.6 | Cell growth and/or maintenance | Centrosome;Microtubule;Nucleus | Transporter activity | Centrosome |
| **LSG1** | NP_060855.2 | 55341 | **large subunit GTPase 1 homolog** | 9.9 | 1 | 3 | 3 | 5 | 1.23E+08 | **1.23** | 2 | 59.6 | 658 | 75.2 | 6.4 | Regulation of nucleobase, nucleoside, nucleotide and nucleic acid metabolism | Nucleolus;Endoplasmic reticulum;Nucleus | Nucleotide binding;GTPase activity | Endoplasmic reticulum |
| **PSMA5** | NP_002781.2 | 5686 | **proteasome subunit alpha type-5 isoform 1** | 58.9 | 2 | 14 | 14 | 95 | 2.32E+09 | **1.23** | 24 | 28.5 | 241 | 26.4 | 4.8 | Protein metabolism | Cytoplasm;Nucleus | Ubiquitin-specific protease activity | Cytoplasm |
| **HNRNPA2B1** | NP_112533.1 | 3181 | **heterogeneous nuclear ribonucleoproteins A2/B1 isoform B1** | 85.0 | 2 | 28 | 32 | 642 | 8.61E+09 | **1.23** | 92 | 13.3 | 353 | 37.4 | 9.0 | Regulation of nucleobase, nucleoside, nucleotide and nucleic acid metabolism | Nucleus;Nucleolus;Cytoplasm;Mitochondrion | Transcription factor binding;RNA binding | Nucleus |
| **PDIA4** | NP_004902.1 | 9601 | **protein disulfide-isomerase A4 precursor** | 49.9 | 1 | 29 | 29 | 108 | 1.14E+09 | **1.23** | 32 | 12.7 | 645 | 72.9 | 5.1 | Protein metabolism | Endoplasmic reticulum;Plasma membrane;Mitochondrion | Chaperone activity | Endoplasmic reticulum |
| **LRRC20** | NP_060675.1 | 55222 | **leucine-rich repeat-containing protein 20 isoform 3** | 14.8 | 2 | 1 | 1 | 2 | 8.62E+07 | **1.22** | 2 | 3.8 | 128 | 14.1 | 9.6 | Biological_process unknown | - | Molecular function unknown | - |
| **NDUFB11** | NP_001129470.1 | 54539 | **NADH dehydrogenase [ubiquinone] 1 beta subcomplex subunit 11, mitochondrial isoform 2** | 39.2 | 2 | 3 | 3 | 8 | 1.19E+08 | **1.22** | 3 | 33.8 | 153 | 17.3 | 5.2 | Metabolism;Energy pathways | - | Oxidoreductase activity | - |
| **FNTA** | NP_002018.1 | 2339 | **protein farnesyltransferase/geranylgeranyltransferase type-1 subunit alpha** | 20.8 | 1 | 4 | 4 | 13 | 1.04E+08 | **1.22** | 5 | 1.7 | 379 | 44.4 | 5.1 | Protein modification | Nucleus;Cytoplasm | Transferase activity | Cytoplasm |
| **RAB2A** | NP_002856.1 | 5862 | **ras-related protein Rab-2A isoform a** | 57.6 | 4 | 12 | 12 | 43 | 8.23E+08 | **1.22** | 16 | 15.8 | 212 | 23.5 | 6.5 | Cell communication;Signal transduction | Golgi apparatus;Endoplasmic reticulum;Cytoplasm;Mitochondrion;Cytosol | GTPase activity | Endoplasmic reticulum |
| **USP9X** | NP_001034680.2 | 8239 | **probable ubiquitin carboxyl-terminal hydrolase FAF-X isoform 4** | 6.3 | 3 | 9 | 9 | 13 | 9.80E+07 | **1.22** | 8 | 31.7 | 2554 | 290.3 | 5.8 | Protein metabolism | Cytoplasm;Nucleus | Ubiquitin-specific protease activity | Cytoplasm;Nucleus |
| **CDC37** | NP_008996.1 | 11140 | **hsp90 co-chaperone Cdc37** | 51.3 | 1 | 20 | 20 | 116 | 2.52E+09 | **1.22** | 40 | 21.2 | 378 | 44.4 | 5.3 | Protein metabolism | Cytoplasm | Chaperone activity | Cytoplasm |
| **DYNC1LI2** | NP_006132.1 | 1783 | **cytoplasmic dynein 1 light intermediate chain 2 isoform 1** | 45.1 | 2 | 15 | 16 | 37 | 3.87E+08 | **1.22** | 19 | 17.9 | 492 | 54.1 | 6.4 | Biological_process unknown | Cytoplasm;Microtubule | Molecular function unknown | Cytoplasm |
| **GORASP2** | NP_001188357.1 | 26003 | **Golgi reassembly-stacking protein 2 isoform 2** | 31.0 | 2 | 6 | 6 | 16 | 1.78E+08 | **1.22** | 6 | 46.1 | 384 | 39.7 | 4.6 | Transport | Golgi apparatus;Nucleus;Cytoplasm | Transporter activity | Golgi apparatus |
| **LIMS1** | NP_001180412.1 | 3987 | **LIM and senescent cell antigen-like-containing domain protein 1 isoform d** | 15.1 | 5 | 3 | 3 | 10 | 1.74E+08 | **1.22** | 3 | 2 | 337 | 38.4 | 8.1 | Cell communication;Signal transduction | Cytoplasm;Nucleus | Receptor signaling complex scaffold activity | Cytoplasm |
| **YKT6** | NP_006546.1 | 10652 | **synaptobrevin homolog YKT6** | 59.6 | 1 | 7 | 7 | 22 | 2.47E+08 | **1.22** | 11 | 27.3 | 198 | 22.4 | 6.9 | Transport | Endoplasmic reticulum;Golgi apparatus | Molecular function unknown | Endoplasmic reticulum |
| **HCFC1** | NP_005325.2 | 3054 | **host cell factor 1** | 16.9 | 1 | 20 | 20 | 38 | 2.74E+08 | **1.22** | 18 | 23.8 | 2035 | 208.6 | 7.5 | Regulation of nucleobase, nucleoside, nucleotide and nucleic acid metabolism | Nucleus;Cytoplasm;Mitochondrion | Transcription factor activity | Nucleus;Cytoplasm |
| **PITPNB** | NP_036531.1 | 23760 | **phosphatidylinositol transfer protein beta isoform isoform 1** | 49.1 | 3 | 9 | 10 | 36 | 9.05E+08 | **1.22** | 14 | 15.6 | 271 | 31.5 | 6.9 | Transport | Cytoplasm | Transporter activity | Cytoplasm |
| **EXOSC7** | NP_055819.2 | 23016 | **exosome complex component RRP42** | 37.1 | 1 | 6 | 6 | 10 | 2.17E+08 | **1.22** | 5 | 9.7 | 291 | 31.8 | 5.2 | Regulation of nucleobase, nucleoside, nucleotide and nucleic acid metabolism | Nucleolus;Nucleus | Ribonuclease activity | Nucleolus |
| **SEZ6** | NP_001277131.1 | 124925 | **seizure protein 6 homolog isoform 3** | 0.7 | 3 | 1 | 1 | 1 | 9.17E+07 | **1.22** | 1 | - | 869 | 94 | 5.5 | Biological_process unknown | Integral to membrane | Molecular function unknown | Integral to membrane |
| **VTI1B** | NP_006361.1 | 10490 | **vesicle transport through interaction with t-SNAREs homolog 1B** | 15.1 | 1 | 2 | 2 | 3 | 1.93E+07 | **1.22** | 1 | - | 232 | 26.7 | 9.0 | Transport | Cytoplasmic vesicle;Golgi apparatus;Lysosome;Endosome | Molecular function unknown | Cytoplasmic vesicle |
| **KIAA1429** | NP_056311.2 | 25962 | **protein virilizer homolog isoform 1** | 5.4 | 2 | 5 | 5 | 8 | 7.80E+07 | **1.22** | 3 | 63.9 | 1812 | 201.9 | 5.0 | Biological_process unknown | Nucleus;Cytoplasm | Molecular function unknown | Nucleus;Cytoplasm |
| **MRPS18B** | NP_054765.1 | 28973 | **28S ribosomal protein S18b, mitochondrial** | 24.4 | 1 | 3 | 3 | 6 | 1.49E+07 | **1.22** | 2 | 42.5 | 258 | 29.4 | 9.4 | Protein metabolism | Mitochondrion | Structural constituent of ribosome | Mitochondrion |

| Gene Symbol | RefSeq_Accession | Entrez Gene ID | Description | Coverage (%) | Proteins | Unique Peptides | # Peptides | PSM | Area | SCC-R /SCC-S | SCC-R /SCC-S (Count) | SCC-R /SCC-S (Variability [%]) | # AAs | MW [kDa] | calc. pI | Biological Process | Localization | Molecular Function | Primary Localization |
|---|---|---|---|---|---|---|---|---|---|---|---|---|---|---|---|---|---|---|---|
| **LSM7** | NP_057283.1 | 51690 | **U6 snRNA-associated Sm-like protein LSm7** | 63.1 | 1 | 3 | 3 | 8 | 3.30E+08 | **1.22** | 2 | 25.2 | 103 | 11.6 | 5.3 | Regulation of nucleobase, nucleoside, nucleotide and nucleic acid metabolism | Cytoplasm;Nucleus | RNA binding | Cytoplasm |
| **LTN1** | NP_056380.2 | 26046 | **E3 ubiquitin-protein ligase listerin** | 3.0 | 1 | 3 | 3 | 3 | 2.93E+07 | **1.22** | 2 | 51 | 1812 | 205 | 6.5 | Protein metabolism | - | Ubiquitin-specific protease activity | - |
| **UBR7** | NP_786924.2 | 55148 | **putative E3 ubiquitin-protein ligase UBR7** | 24.7 | 1 | 4 | 4 | 8 | 1.27E+08 | **1.22** | 2 | 37.3 | 425 | 48 | 4.8 | Biological_process unknown | - | Molecular function unknown | - |
| **DLAT** | NP_001922.2 | 1737 | **dihydrolipoyllysine-residue acetyltransferase component of pyruvate dehydrogenase complex, mitochondrial** | 17.9 | 1 | 7 | 7 | 23 | 4.73E+08 | **1.22** | 9 | 44.3 | 647 | 69 | 7.8 | Metabolism;Energy pathways | Mitochondrion | Acyltransferase activity | Mitochondrion |
| **EIF5B** | NP_056988.3 | 9669 | **eukaryotic translation initiation factor 5B** | 25.6 | 1 | 18 | 18 | 53 | 3.59E+08 | **1.22** | 27 | 24.8 | 1220 | 138.7 | 5.5 | Protein metabolism | Cytoplasm;Nucleolus;Nucleus | Translation regulator activity | Cytoplasm |
| **GNB1** | NP_001269468.1 | 2782 | **guanine nucleotide-binding protein G(I)/G(S)/G(T) subunit beta-1 isoform 1** | 58.2 | 3 | 9 | 15 | 87 | 5.28E+08 | **1.22** | 10 | 17.4 | 340 | 37.4 | 6.0 | Cell communication;Signal transduction | Plasma membrane;Mitochondrion | GTPase activity | Plasma membrane |
| **NPM1** | NP_954654.1 | 4869 | **nucleophosmin isoform 2** | 83.4 | 3 | 23 | 23 | 764 | 1.01E+10 | **1.22** | 124 | 18.1 | 265 | 29.4 | 4.6 | Protein metabolism | Centrosome;Cytoplasm;Nucleolus;Nucleus | Chaperone activity | Nucleolus;Nucleus;Cytoplasm |
| **SNX30** | NP_001013012.1 | 401548 | **sorting nexin-30** | 2.8 | 1 | 1 | 1 | 1 | 3.13E+06 | **1.22** | 1 | - | 437 | 49.6 | 5.4 | Protein transport | - | Protein binding | - |
| **PATL1** | NP_689929.2 | 219988 | **protein PAT1 homolog 1** | 9.1 | 1 | 3 | 3 | 12 | 1.16E+08 | **1.21** | 4 | 13 | 770 | 86.8 | 6.7 | Biological_process unknown | Nucleus;Cytoplasm | Molecular function unknown | Nucleus;Cytoplasm |
| **TXNDC5** | NP_110437.2 | 81567 | **thioredoxin domain-containing protein 5 isoform 1 precursor** | 39.6 | 2 | 12 | 12 | 49 | 1.49E+09 | **1.21** | 17 | 10.9 | 432 | 47.6 | 6.0 | Protein metabolism | Endoplasmic reticulum;Golgi apparatus;Plasma membrane | Oxidoreductase activity | Endoplasmic reticulum |
| **UQCRH** | NP_005995.2 | 7388 | **cytochrome b-c1 complex subunit 6, mitochondrial** | 29.7 | 1 | 2 | 2 | 26 | 5.90E+08 | **1.21** | 5 | 23 | 91 | 10.7 | 4.4 | Metabolism;Energy pathways | Mitochondrion | Catalytic activity | Mitochondrion |
| **MIPOL1** | NP_001182226.1 | 145282 | **mirror-image polydactyly gene 1 protein** | 1.4 | 1 | 1 | 1 | 2 | 5.04E+07 | **1.21** | 2 | 40.2 | 442 | 51.5 | 5.7 | Biological_process unknown | - | Molecular function unknown | - |
| **MTCH1** | NP_055156.1 | 23787 | **mitochondrial carrier homolog 1 PSAP-LS** | 13.7 | 2 | 4 | 4 | 5 | 7.21E+07 | **1.21** | 1 | - | 372 | 39.9 | 9.5 | Apoptosis | Mitochondrion | Caspase activator activity | Mitochondrion |
| **SEC24D** | NP_055637.2 | 9871 | **protein transport protein Sec24D** | 3.4 | 1 | 1 | 1 | 4 | 5.71E+07 | **1.21** | 1 | - | 1032 | 112.9 | 7.3 | Transport | Endoplasmic reticulum;Cytoplasm | Transporter activity | Endoplasmic reticulum |
| **MAPRE1** | NP_036457.1 | 22919 | **microtubule-associated protein RP/EB family member 1** | 62.7 | 2 | 14 | 14 | 64 | 1.37E+09 | **1.21** | 19 | 18.3 | 268 | 30 | 5.1 | Cell communication;Signal transduction | Centrosome;Cytoplasm | Protein binding | Centrosome |
| **GTPBP1** | NP_004277.2 | 9567 | **GTP-binding protein 1** | 16.6 | 1 | 8 | 8 | 14 | 1.27E+08 | **1.21** | 7 | 16 | 669 | 72.4 | 8.3 | Cell communication;Signal transduction | Cytoplasm | GTPase activity | Cytoplasm |
| **NDUFB10** | NP_004539.1 | 4716 | **NADH dehydrogenase [ubiquinone] 1 beta subcomplex subunit 10** | 36.6 | 1 | 5 | 5 | 7 | 1.39E+08 | **1.21** | 4 | 34.3 | 172 | 20.8 | 8.5 | Metabolism;Energy pathways | Mitochondrion | Oxidoreductase activity | Mitochondrion |
| **PTGES2** | NP_079348.1 | 80142 | **prostaglandin E synthase 2 isoform 1** | 22.0 | 2 | 6 | 6 | 23 | 8.89E+08 | **1.21** | 6 | 16.7 | 377 | 41.9 | 9.2 | Metabolism;Energy pathways | Endoplasmic reticulum;Nucleus;Cytoplasm | Isomerase activity | Nucleus |
| **RPS26** | NP_001020.2 | 6231 | **40S ribosomal protein S26** | 44.4 | 2 | 4 | 4 | 32 | 1.77E+09 | **1.21** | 7 | 24 | 115 | 13 | 11.0 | Protein metabolism | Ribosome;Cytoplasm | Structural constituent of ribosome | Ribosome |
| **SF3A3** | NP_006793.1 | 10946 | **splicing factor 3A subunit 3** | 42.1 | 1 | 15 | 15 | 49 | 3.38E+08 | **1.21** | 18 | 28.2 | 501 | 58.8 | 5.4 | Protein metabolism | Nucleus;Nucleolus;Chromosome | RNA binding | Nucleus |
| **SEPT7** | NP_001011553.2 | 989 | **septin-7 isoform 2** | 51.4 | 6 | 20 | 21 | 91 | 1.74E+09 | **1.21** | 23 | 20.1 | 436 | 50.5 | 8.6 | Cell communication;Signal transduction | Cytoskeleton;Nucleolus;Mitochondrion;Nucleus;Cytoplasm | Protein binding | Cytoskeleton |
| **HMGN4** | NP_006344.1 | 10473 | **high mobility group nucleosome-binding domain-containing protein 4** | 18.9 | 1 | 1 | 1 | 4 | 6.99E+07 | **1.21** | 3 | 7 | 90 | 9.5 | 10.5 | Regulation of nucleobase, nucleoside, nucleotide and nucleic acid metabolism | Nucleus | Transcription regulator activity | Nucleus |
| **PDCL3** | NP_076970.1 | 79031 | **phosducin-like protein 3** | 33.9 | 1 | 5 | 5 | 7 | 8.90E+07 | **1.21** | 5 | 34.6 | 239 | 27.6 | 4.8 | Apoptosis | Cytoplasm;Integral to membrane | Molecular function unknown | Cytoplasm |
| **COX5B** | NP_001853.2 | 1329 | **cytochrome c oxidase subunit 5B, mitochondrial precursor** | 55.0 | 1 | 9 | 9 | 33 | 6.32E+08 | **1.21** | 10 | 6.4 | 129 | 13.7 | 8.8 | Metabolism;Energy pathways | Mitochondrion;Cytoplasm;Nucleus | Oxidoreductase activity | Mitochondrion |
| **PI4K2A** | NP_060895.1 | 55361 | **phosphatidylinositol 4-kinase type 2-alpha** | 18.0 | 1 | 5 | 5 | 15 | 1.52E+08 | **1.21** | 6 | 23.6 | 479 | 54 | 8.3 | Cell communication;Signal transduction | Endoplasmic reticulum;Golgi apparatus;Nucleus;Cytoplasm | Lipid kinase activity | Golgi apparatus |
| **RAB5A** | NP_004153.2 | 5868 | **ras-related protein Rab-5A isoform 1** | 43.7 | 1 | 5 | 6 | 8 | 5.48E+07 | **1.21** | 2 | 15.5 | 215 | 23.6 | 8.2 | Cell communication;Signal transduction | Plasma membrane;Cytoplasm;Endosome;Cell projection | GTPase activity | Plasma membrane |
| **UBAP2L** | NP_055662.3 | 9898 | **ubiquitin-associated protein 2-like isoform a** | 28.5 | 4 | 16 | 16 | 58 | 3.04E+08 | **1.21** | 19 | 52.5 | 1087 | 114.5 | 7.1 | Biological_process unknown | Nucleus;Cytoplasm | Molecular function unknown | Nucleus |
| **CAV1** | NP_001166367.1 | 857 | **caveolin-1 isoform beta** | 29.9 | 1 | 1 | 4 | 13 | 4.32E+08 | **1.20** | 2 | 3.1 | 147 | 17 | 5.5 | Cell growth and/or maintenance | Plasma membrane;Integral to membrane | Structural molecule activity | Plasma membrane |
| **ENY2** | NP_001180486.1 | 56943 | **transcription and mRNA export factor ENY2 isoform 2** | 17.7 | 2 | 1 | 1 | 7 | 1.96E+08 | **1.20** | 3 | 1.4 | 96 | 11 | 9.1 | Regulation of nucleobase, nucleoside, nucleotide and nucleic acid metabolism | Nucleus | Transcription factor activity | Nucleus |
| **INIP** | NP_067041.1 | 58493 | **SOSS complex subunit C** | 36.5 | 1 | 2 | 2 | 4 | 4.70E+07 | **1.20** | 2 | 19.6 | 104 | 11.4 | 9.3 | Biological_process unknown | - | Molecular function unknown | - |
| **NAP1L4** | NP_005960.1 | 4676 | **nucleosome assembly protein 1-like 4** | 53.1 | 1 | 12 | 14 | 54 | 1.11E+09 | **1.20** | 14 | 36.9 | 375 | 42.8 | 4.7 | Regulation of nucleobase, nucleoside, nucleotide and nucleic acid metabolism | Nucleus;Cytoplasm | Chaperone activity | Nucleus |
| **NDUFA2** | NP_002479.1 | 4695 | **NADH dehydrogenase [ubiquinone] 1 alpha subcomplex subunit 2 isoform 1** | 21.2 | 1 | 1 | 1 | 2 | 6.08E+07 | **1.20** | 2 | 23.4 | 99 | 10.9 | 9.6 | Metabolism;Energy pathways | - | Oxidoreductase activity | - |
| **U2AF1** | NP_006749.1 | 7307 | **splicing factor U2AF 35 kDa subunit isoform a** | 58.3 | 5 | 9 | 9 | 35 | 1.22E+09 | **1.20** | 9 | 25.8 | 240 | 27.9 | 8.8 | Regulation of nucleobase, nucleoside, nucleotide and nucleic acid metabolism | Nucleoplasm;Nucleus;Cytoplasm | RNA binding | Nucleus |
| **SEC16A** | NP_055681.1 | 9919 | **protein transport protein Sec16A isoform 1** | 20.8 | 1 | 1 | 25 | 56 | 8.35E+08 | **1.20** | 1 | - | 2357 | 251.7 | 5.8 | Cell motility;ER organization and biogenesis;Protein transport | Endoplasmic reticulum | Molecular function unknown | Endoplasmic reticulum |
| **TXNDC9** | NP_005774.2 | 10190 | **thioredoxin domain-containing protein 9** | 19.0 | 1 | 3 | 3 | 7 | 3.18E+07 | **1.20** | 3 | 6.5 | 226 | 26.5 | 5.9 | Biological_process unknown;Cell growth and/or maintenance | Cytoplasm;Nucleus;Centrosome | Molecular function unknown | Cytoplasm |
| **ZC3H4** | NP_055983.1 | 23211 | **zinc finger CCCH domain-containing protein 4** | 10.6 | 1 | 8 | 8 | 16 | 1.54E+08 | **1.20** | 7 | 8.4 | 1303 | 140.2 | 6.3 | Biological_process unknown | - | Molecular function unknown | - |
| **AKT1** | NP_001014431.1 | 207 | **RAC-alpha serine/threonine-protein kinase** | 14.4 | 8 | 4 | 6 | 13 | 6.02E+07 | **1.20** | 5 | 14.8 | 480 | 55.7 | 6.1 | Cell communication;Signal transduction | Plasma membrane;Cytoplasm;Nucleus | Protein serine/threonine kinase activity | Cytoplasm |

| Gene Symbol | RefSeq_Accession | Entrez Gene ID | Description | Coverage (%) | Proteins | Unique Peptides | # Peptides | PSM | Area | SCC-R /SCC-S | SCC-R /SCC-S (Count) | SCC-R /SCC-S (Variability [%]) | # AAs | MW [kDa] | calc. pI | Biological Process | Localization | Molecular Function | Primary Localization |
|---|---|---|---|---|---|---|---|---|---|---|---|---|---|---|---|---|---|---|---|
| **CCT8** | NP_006576.2 | 10694 | **T-complex protein 1 subunit theta isoform 1** | 73.5 | 4 | 36 | 36 | 265 | 3.41E+09 | **1.20** | 62 | 15 | 548 | 59.6 | 5.6 | Protein metabolism | Cytoplasm;Microtubule;Mitochondrion | Chaperone activity | Cytoplasm |
| **INF2** | NP_001026884.3 | 64423 | **inverted formin-2 isoform 2** | 13.2 | 2 | 9 | 9 | 16 | 6.92E+07 | **1.20** | 4 | 3 | 1240 | 134.5 | 5.5 | Biological_process unknown;Cytoskeleton organization and biogenesis | Nucleus;Cytoplasm | Molecular function unknown;Cytoskeletal protein binding | Nucleus;Cytoplasm |
| **MRPS22** | NP_064576.1 | 56945 | **28S ribosomal protein S22, mitochondrial** | 25.0 | 1 | 7 | 7 | 12 | 1.56E+08 | **1.20** | 5 | 48.7 | 360 | 41.3 | 7.9 | Protein metabolism | Mitochondrion | Structural constituent of ribosome | Mitochondrion |
| **NOP10** | NP_061118.1 | 55505 | **H/ACA ribonucleoprotein complex subunit 3** | 42.2 | 1 | 3 | 3 | 4 | 7.44E+07 | **1.20** | 2 | 9.2 | 64 | 7.7 | 10.0 | Regulation of nucleobase, nucleoside, nucleotide and nucleic acid metabolism | Nucleus;Nucleolus | Ribonucleoprotein | Nucleolus |
| **GNAS** | NP_000507.1 | 2778 | **protein GNAS isoform GNASL** | 18.5 | 4 | 1 | 4 | 7 | 2.20E+08 | **1.20** | 1 | - | 394 | 45.6 | 5.8 | Cell communication;Signal transduction | Plasma membrane | GTPase activity | Plasma membrane |
| **HDAC1** | NP_004955.2 | 3065 | **histone deacetylase 1** | 28.8 | 1 | 6 | 8 | 22 | 6.00E+08 | **1.20** | 7 | 6.3 | 482 | 55.1 | 5.5 | Regulation of nucleobase, nucleoside, nucleotide and nucleic acid metabolism | Nucleus;Nucleolus;Mitochondrion | Transcription regulator activity | Nucleus |
| **LMAN2** | NP_006807.1 | 10960 | **vesicular integral-membrane protein VIP36 precursor** | 46.9 | 1 | 10 | 10 | 26 | 6.86E+08 | **1.20** | 11 | 20.5 | 356 | 40.2 | 7.0 | Transport | Golgi apparatus;Cytoplasm;Mitochondrion | Transporter activity | Golgi apparatus |
| **SPECC1L** | NP_001241661.2 | 23384 | **cytospin-A isoform 2** | 4.8 | 2 | 2 | 2 | 2 | 2.62E+07 | **1.20** | 1 | - | 1078 | 120.1 | 5.8 | Biological_process unknown | - | Molecular function unknown | - |
| **VAMP3** | NP_004772.1 | 9341 | **vesicle-associated membrane protein 3** | 41.0 | 4 | 2 | 5 | 27 | 7.33E+08 | **1.20** | 2 | 0.3 | 100 | 11.3 | 8.8 | Transport | Plasma membrane | Protein binding;Transporter activity | Plasma membrane |
| **ARMC8** | NP_998819.1 | 25852 | **armadillo repeat-containing protein 8 isoform 1** | 14.8 | 5 | 3 | 3 | 5 | 4.10E+07 | **1.20** | 3 | 40.5 | 385 | 43 | 7.2 | Biological_process unknown | - | Molecular function unknown | - |
| **DYNC1H1** | NP_001367.2 | 1778 | **cytoplasmic dynein 1 heavy chain 1** | 47.7 | 1 | 172 | 172 | 684 | 1.90E+09 | **1.20** | 233 | 25.9 | 4646 | 532.1 | 6.4 | Metabolism;Energy pathways | Cytoplasm;Golgi apparatus;Endosome;Lysosome;Mitochondrion;Plasma membrane;Nucleus;Nucleolus;Microtubule | ATPase activity | Cytoplasm |
| **GAK** | NP_001273762.1 | 2580 | **cyclin-G-associated kinase isoform 2** | 9.9 | 2 | 5 | 5 | 6 | 8.95E+07 | **1.20** | 2 | 24.3 | 1213 | 132.1 | 5.7 | Cell communication;Signal transduction | Cytoplasm;Golgi apparatus;Plasma membrane;Endoplasmic reticulum | Protein serine/threonine kinase activity | Cytoplasm |
| **LMBRD2** | NP_001007528.1 | 92255 | **LMBR1 domain-containing protein 2** | 8.1 | 1 | 3 | 3 | 4 | 3.72E+07 | **1.20** | 2 | 37.2 | 695 | 81.1 | 7.5 | Biological_process unknown | - | Molecular function unknown | - |
| **SETDB1** | NP_036564.3 | 9869 | **histone-lysine N-methyltransferase SETDB1 isoform 2** | 3.8 | 2 | 3 | 3 | 4 | 4.33E+07 | **1.20** | 1 | - | 1290 | 142.9 | 6.0 | Regulation of nucleobase, nucleoside, nucleotide and nucleic acid metabolism | Nucleus;Chromosome | Transcription regulator activity | Nucleus;Chromosome |
| **SNRPD1** | NP_008869.1 | 6632 | **small nuclear ribonucleoprotein Sm D1 isoform 1** | 54.6 | 2 | 5 | 5 | 71 | 1.30E+09 | **1.20** | 12 | 24.3 | 119 | 13.3 | 11.6 | Regulation of nucleobase, nucleoside, nucleotide and nucleic acid metabolism | Cytoplasm;Nucleus | RNA binding | Nucleus |
| **WBP11** | NP_057396.1 | 51729 | **WW domain-binding protein 11** | 27.3 | 1 | 12 | 12 | 28 | 3.67E+08 | **1.20** | 9 | 33.8 | 641 | 70 | 8.4 | Regulation of nucleobase, nucleoside, nucleotide and nucleic acid metabolism | Nucleus;Integral to membrane;Nucleolus;Cytoplasm | RNA binding | Nucleus |
| **EMC2** | NP_055488.1 | 9694 | **ER membrane protein complex subunit 2** | 39.1 | 1 | 6 | 6 | 21 | 3.37E+08 | **1.20** | 6 | 27.4 | 297 | 34.8 | 6.6 | Biological_process unknown | Nucleus | Molecular function unknown | Nucleus |
| **CDC42BPB** | NP_006026.3 | 9578 | **serine/threonine-protein kinase MRCK beta** | 8.1 | 1 | 8 | 8 | 12 | 1.07E+08 | **1.20** | 5 | 22.3 | 1711 | 194.2 | 6.4 | Cell communication;Signal transduction | Cytoplasm | Protein serine/threonine kinase activity | Cytoplasm |
| **CRKL** | NP_005198.1 | 1399 | **crk-like protein** | 45.5 | 1 | 10 | 10 | 23 | 5.55E+08 | **1.20** | 9 | 13.3 | 303 | 33.8 | 6.7 | Cell communication;Signal transduction | Cytoplasm;Nucleus | Receptor signaling complex scaffold activity | Cytoplasm |
| **DDX39B** | NP_004631.1 | 7919 | **spliceosome RNA helicase DDX39B** | 66.6 | 1 | 15 | 24 | 183 | 4.93E+09 | **1.20** | 27 | 20 | 428 | 49 | 5.7 | Regulation of nucleobase, nucleoside, nucleotide and nucleic acid metabolism | Nucleus;Nucleolus | RNA binding | Nucleus |
| **GID8** | NP_060366.1 | 54994 | **glucose-induced degradation protein 8 homolog** | 37.7 | 1 | 4 | 4 | 6 | 1.23E+08 | **1.20** | 2 | 29 | 228 | 26.7 | 5.0 | Biological_process unknown | - | Molecular function unknown | - |
| **NDUFS8** | NP_002487.1 | 4728 | **NADH dehydrogenase [ubiquinone] iron-sulfur protein 8, mitochondrial precursor** | 23.8 | 1 | 4 | 4 | 8 | 1.23E+08 | **1.20** | 2 | 13.6 | 210 | 23.7 | 6.3 | Metabolism;Energy pathways | Mitochondrion;Nucleus | Oxidoreductase activity | Mitochondrion |
| **PABPC4** | NP_001129126.1 | 8761 | **polyadenylate-binding protein 4 isoform 3** | 39.6 | 5 | 9 | 20 | 105 | 1.93E+09 | **1.20** | 13 | 20.4 | 631 | 69.5 | 9.5 | Regulation of nucleobase, nucleoside, nucleotide and nucleic acid metabolism | Cytoplasm | RNA binding | Cytoplasm |
| **PSMD8** | NP_002803.2 | 5714 | **26S proteasome non-ATPase regulatory subunit 8** | 8.9 | 1 | 3 | 3 | 5 | 1.18E+08 | **1.20** | 3 | 1.2 | 350 | 39.6 | 9.7 | Protein metabolism | Cytoplasm | Ubiquitin-specific protease activity | Cytoplasm |
| **CGNL1** | NP_116255.2 | 84952 | **cingulin-like protein 1** | 0.5 | 1 | 1 | 1 | 1 | 1.56E+08 | **1.20** | 1 | - | 1302 | 149 | 5.7 | Biological_process unknown | - | Molecular function unknown | - |
| **PSMD14** | NP_005796.1 | 10213 | **26S proteasome non-ATPase regulatory subunit 14** | 47.1 | 1 | 8 | 8 | 25 | 3.04E+08 | **1.20** | 12 | 26.8 | 310 | 34.6 | 6.5 | Protein metabolism | Cytoplasm;Nucleus | Ubiquitin-specific protease activity | Cytoplasm |
| **VPS35** | NP_060676.2 | 55737 | **vacuolar protein sorting-associated protein 35** | 37.9 | 1 | 25 | 25 | 89 | 7.33E+08 | **1.20** | 25 | 13.8 | 796 | 91.6 | 5.5 | Transport | Cytoplasm;Plasma membrane;Endoplasmic reticulum;Golgi apparatus;Endosome;Membrane fraction | Transporter activity | Cytoplasm |
| **CBR3** | NP_001227.1 | 874 | **carbonyl reductase [NADPH] 3** | 17.7 | 1 | 2 | 4 | 23 | 3.76E+08 | **1.19** | 1 | - | 277 | 30.8 | 6.2 | Metabolism;Energy pathways | - | Oxidoreductase activity | - |
| **MRE11A** | NP_005582.1 | 4361 | **double-strand break repair protein MRE11A isoform 1** | 19.4 | 2 | 8 | 8 | 16 | 2.13E+08 | **1.19** | 10 | 7.9 | 708 | 80.5 | 5.9 | DNA repair | Nucleus;Cytoplasm | Exonuclease activity | Nucleus;Cytoplasm |
| **NHP2** | NP_060308.1 | 55651 | **H/ACA ribonucleoprotein complex subunit 2 isoform a** | 76.5 | 2 | 7 | 7 | 37 | 6.48E+08 | **1.19** | 13 | 24.6 | 153 | 17.2 | 8.2 | Ribosome biogenesis and assembly | Nucleus;Nucleolus | RNA binding | Nucleolus |
| **RABGAP1** | NP_036329.3 | 23637 | **rab GTPase-activating protein 1** | 26.9 | 3 | 15 | 15 | 31 | 3.11E+08 | **1.19** | 14 | 18.9 | 1069 | 121.7 | 5.3 | Cell communication;Signal transduction | Cytoplasm;Centrosome | GTPase activator activity | Cytoplasm |
| **TXLNG** | NP_060830.2 | 55787 | **gamma-taxilin isoform 1** | 5.9 | 1 | 2 | 2 | 6 | 1.21E+07 | **1.19** | 1 | - | 528 | 60.5 | 7.5 | Biological_process unknown | Nucleus;Cytoplasm | Molecular function unknown | Nucleus;Cytoplasm |
| **HTRA1** | NP_002766.1 | 5654 | **serine protease HTRA1 precursor** | 22.1 | 1 | 7 | 7 | 17 | 1.04E+08 | **1.19** | 8 | 21.9 | 480 | 51.3 | 7.8 | Protein metabolism | Extracellular | Serine-type peptidase activity | Extracellular |
| **CSRP2** | NP_001312.1 | 1466 | **cysteine and glycine-rich protein 2** | 6.7 | 1 | 1 | 1 | 1 | 3.77E+07 | **1.19** | 1 | - | 193 | 20.9 | 8.6 | Cell communication;Signal transduction | Nucleus | Receptor signaling complex scaffold activity | Nucleus |
| **BZW1** | NP_001193996.1 | 9689 | **basic leucine zipper and W2 domain-containing protein 1 isoform 1** | 29.4 | 4 | 11 | 12 | 34 | 1.24E+09 | **1.19** | 10 | 3.2 | 419 | 48 | 5.9 | Regulation of nucleobase, nucleoside, nucleotide and nucleic acid metabolism | Nucleus;Cytoplasm | Transcription factor activity | Nucleus |
| **ERGIC1** | NP_001026881.1 | 57222 | **endoplasmic reticulum-Golgi intermediate compartment protein 1** | 27.6 | 1 | 6 | 6 | 11 | 2.13E+08 | **1.19** | 5 | 11.2 | 290 | 32.6 | 7.1 | Transport | Endoplasmic reticulum;Golgi apparatus;Cytoplasm | Transporter activity | Golgi apparatus |
| **RAB11A** | NP_004654.1 | 8766 | **ras-related protein Rab-11A isoform 1** | 46.8 | 2 | 1 | 9 | 24 | 6.08E+08 | **1.19** | 1 | - | 216 | 24.4 | 6.6 | Cell communication;Signal transduction | Golgi apparatus;Mitochondrion | GTPase activity | Golgi apparatus |

Jain *et al.*, 2019. MAP2K1 is a potential therapeutic target in erlotinib resistant head and neck squamous cell carcinoma
Supplementary Table S4. List of all proteins identified in SCC-S and SCC-R cells using SequestHT and Mascot search algorithms and their gene ontology annotation

| Gene Symbol | RefSeq_Accession | Entrez Gene ID | Description | Coverage (%) | Proteins | Unique Peptides | # Peptides | PSM | Area | SCC-R /SCC-S | SCC-R /SCC-S (Count) | SCC-R /SCC-S (Variability [%]) | # AAs | MW [kDa] | calc. pI | Biological Process | Localization | Molecular Function | Primary Localization |
|---|---|---|---|---|---|---|---|---|---|---|---|---|---|---|---|---|---|---|---|
| **THRAP3** | NP_005110.2 | 9967 | **thyroid hormone receptor-associated protein 3** | 18.7 | 1 | 14 | 15 | 90 | 1.07E+09 | **1.19** | 26 | 9.8 | 955 | 108.6 | 10.2 | Regulation of nucleobase, nucleoside, nucleotide and nucleic acid metabolism | Nucleus;Cytoplasm | Transcription regulator activity | Nucleus |
| **TSPYL2** | NP_071400.1 | 64061 | **testis-specific Y-encoded-like protein 2** | 0.9 | 1 | 1 | 1 | 2 | 4.86E+08 | **1.19** | 1 | - | 693 | 79.4 | 4.6 | Cell communication;Signal transduction | Nucleus;Nucleolus;Cytoplasm | ATPase activity | Nucleolus |
| **DERA** | NP_057038.2 | 51071 | **putative deoxyribose-phosphate aldolase** | 13.8 | 1 | 2 | 2 | 2 | 1.58E+07 | **1.19** | 1 | - | 318 | 35.2 | 8.9 | Regulation of nucleobase, nucleoside, nucleotide and nucleic acid metabolism | - | Lyase activity | - |
| **INTS3** | NP_075391.3 | 65123 | **integrator complex subunit 3** | 13.4 | 1 | 7 | 7 | 10 | 1.95E+08 | **1.19** | 5 | 69.2 | 1042 | 117.9 | 5.8 | Biological_process unknown | - | Molecular function unknown | - |
| **PEX19** | NP_001180573.1 | 5824 | **peroxisomal biogenesis factor 19 isoform c** | 27.6 | 2 | 5 | 5 | 11 | 8.70E+07 | **1.19** | 5 | 29.7 | 279 | 30.8 | 4.4 | Cell communication;Signal transduction | Peroxisome;Cytoplasm | Protein binding | Cytoplasm |
| **HSPA5** | NP_005338.1 | 3309 | **78 kDa glucose-regulated protein precursor** | 48.6 | 3 | 38 | 40 | 441 | 6.80E+09 | **1.19** | 93 | 17.7 | 654 | 72.3 | 5.2 | Protein metabolism | Endoplasmic reticulum;Plasma membrane;Cytoplasm;Nucleolus;Endoplasmic reticulum lumen;Mitochondrion;Cell surface | Chaperone activity | Endoplasmic reticulum |
| **SWAP70** | NP_055870.2 | 23075 | **switch-associated protein 70** | 15.0 | 1 | 5 | 5 | 8 | 1.34E+08 | **1.19** | 4 | 25.8 | 585 | 69 | 5.9 | Cell communication;Signal transduction | Cytoplasm;Nucleus;Plasma membrane | Guanyl-nucleotide exchange factor activity | Cytoplasm |
| **ANAPC4** | NP_037499.2 | 29945 | **anaphase-promoting complex subunit 4 isoform 2** | 10.0 | 2 | 4 | 4 | 8 | 6.18E+07 | **1.19** | 1 | - | 808 | 92.1 | 5.5 | Cell communication;Signal transduction | Nucleus;Centrosome | Molecular function unknown | Nucleus |
| **RABL3** | NP_776186.2 | 285282 | **rab-like protein 3** | 20.8 | 1 | 3 | 3 | 3 | 1.60E+07 | **1.19** | 1 | - | 236 | 26.4 | 7.1 | Cell communication;Signal transduction | - | GTPase activity | - |
| **SCAMP1** | NP_004857.4 | 9522 | **secretory carrier-associated membrane protein 1 isoform 1** | 32.3 | 2 | 6 | 6 | 19 | 2.79E+08 | **1.19** | 5 | 57.4 | 338 | 37.9 | 7.4 | Transport | Cytoplasm;Plasma membrane;Golgi apparatus;Secretory granule;Synaptic vesicle | Auxiliary transport protein activity | Plasma membrane |
| **USP15** | NP_006304.1 | 9958 | **ubiquitin carboxyl-terminal hydrolase 15 isoform 2** | 10.0 | 2 | 5 | 5 | 8 | 2.39E+07 | **1.19** | 2 | 9.8 | 952 | 109.2 | 5.2 | Protein metabolism | Cytoplasm;Nucleolus | Ubiquitin-specific protease activity | Cytoplasm |
| **ALDOA** | NP_908932.1 | 226 | **fructose-bisphosphate aldolase A isoform 1** | 90.4 | 3 | 30 | 34 | 685 | 5.66E+09 | **1.19** | 86 | 18.4 | 364 | 39.4 | 8.1 | Metabolism;Energy pathways | Cytoplasm;Cytosol;Mitochondrion;Nucleus | Lyase activity | Cytoplasm |
| **LSM12** | NP_689557.1 | 124801 | **protein LSM12 homolog** | 29.7 | 1 | 4 | 4 | 7 | 5.50E+07 | **1.19** | 1 | - | 195 | 21.7 | 7.7 | Biological_process unknown | - | Molecular function unknown | - |
| **MON2** | NP_001265398.1 | 23041 | **protein MON2 homolog isoform 2** | 5.4 | 5 | 6 | 6 | 6 | 3.82E+07 | **1.19** | 2 | 1.3 | 1675 | 185.7 | 6.2 | Biological_process unknown | - | Molecular function unknown | - |
| **UBE2L3** | NP_003338.1 | 7332 | **ubiquitin-conjugating enzyme E2 L3 isoform 1** | 64.3 | 3 | 12 | 12 | 56 | 1.53E+09 | **1.19** | 21 | 18.4 | 154 | 17.9 | 8.5 | Protein metabolism | Nucleus;Cytoplasm;Perinuclear region | Ubiquitin-specific protease activity;Ubiquitin binding | Cytoplasm |
| **PPA2** | NP_789845.1 | 27068 | **inorganic pyrophosphatase 2, mitochondrial isoform 1 precursor** | 51.5 | 4 | 11 | 11 | 33 | 3.24E+08 | **1.19** | 14 | 10.7 | 334 | 37.9 | 7.4 | Metabolism;Energy pathways | Mitochondrion;Cytoplasm | Catalytic activity | Mitochondrion;Cytoplasm |
| **TIMM9** | NP_036592.1 | 26520 | **mitochondrial import inner membrane translocase subunit Tim9** | 68.5 | 1 | 4 | 4 | 10 | 2.15E+08 | **1.19** | 4 | 6.7 | 89 | 10.4 | 7.2 | Cell growth and/or maintenance | Mitochondrial membrane;Mitochondrion | Transporter activity | Mitochondrial membrane |
| **HUWE1** | NP_113584.3 | 10075 | **E3 ubiquitin-protein ligase HUWE1** | 28.8 | 1 | 71 | 71 | 185 | 6.82E+08 | **1.18** | 74 | 27.4 | 4374 | 481.6 | 5.2 | Regulation of nucleobase, nucleoside, nucleotide and nucleic acid metabolism | Nucleus;Cytoplasm | DNA binding | Nucleus |
| **MAPK3** | NP_001103361.1 | 5595 | **mitogen-activated protein kinase 3 isoform 3** | 36.1 | 3 | 5 | 8 | 28 | 9.06E+08 | **1.18** | 4 | 28 | 335 | 38.3 | 6.2 | Signal transduction | Cytoplasm;Nucleus;Cytosol;Chromosome | Protein serine/threonine kinase activity | Cytoplasm |
| **NDUFAB1** | NP_004994.1 | 4706 | **acyl carrier protein, mitochondrial precursor** | 27.6 | 1 | 4 | 4 | 16 | 7.08E+08 | **1.18** | 6 | 13.8 | 156 | 17.4 | 4.9 | Metabolism;Energy pathways | Mitochondrion | Oxidoreductase activity | Mitochondrion |
| **AP2M1** | NP_001020376.1 | 1173 | **AP-2 complex subunit mu isoform b** | 27.3 | 2 | 10 | 10 | 25 | 5.29E+08 | **1.18** | 12 | 32.3 | 433 | 49.4 | 9.5 | Transport | Plasma membrane | Receptor signaling complex scaffold activity | Plasma membrane |
| **RAD50** | NP_005723.2 | 10111 | **DNA repair protein RAD50** | 21.2 | 1 | 17 | 17 | 45 | 3.06E+08 | **1.18** | 15 | 8.9 | 1312 | 153.8 | 6.9 | Regulation of nucleobase, nucleoside, nucleotide and nucleic acid metabolism | Nucleus;Nucleolus;Cytoplasm | DNA binding | Nucleus |
| **SRRT** | NP_001122326.1 | 51593 | **serrate RNA effector molecule homolog isoform e** | 25.0 | 4 | 15 | 15 | 37 | 6.68E+08 | **1.18** | 16 | 23.5 | 871 | 100.1 | 6.0 | Biological_process unknown | Nucleus | Molecular function unknown | Nucleus |
| **USO1** | NP_003706.2 | 8615 | **general vesicular transport factor p115 isoform 2** | 27.1 | 2 | 15 | 15 | 54 | 5.50E+08 | **1.18** | 19 | 17.9 | 962 | 107.8 | 4.9 | Transport | Golgi apparatus;Cytoplasm;Nucleus;Cytosol | Transporter activity | Golgi apparatus |
| **DDX19B** | NP_009173.1 | 11269 | **ATP-dependent RNA helicase DDX19B isoform 1** | 46.1 | 5 | 3 | 12 | 43 | 2.57E+08 | **1.18** | 1 | - | 479 | 53.9 | 6.3 | Metabolism | Cytoplasm;Nucleus | ATPase activity | Cytoplasm;Nucleus |
| **EXOSC8** | NP_852480.1 | 11340 | **exosome complex component RRP43** | 24.3 | 1 | 3 | 3 | 14 | 3.72E+07 | **1.18** | 5 | 13.7 | 276 | 30 | 5.3 | Regulation of nucleobase, nucleoside, nucleotide and nucleic acid metabolism | Nucleolus | Ribonuclease activity | Nucleolus |
| **QTRT1** | NP_112486.1 | 81890 | **queuine tRNA-ribosyltransferase** | 18.4 | 1 | 4 | 4 | 6 | 9.53E+07 | **1.18** | 1 | - | 403 | 44 | 7.2 | Metabolism;Energy pathways | - | Catalytic activity | - |
| **SPTBN1** | NP_003119.2 | 6711 | **spectrin beta chain, non-erythrocytic 1 isoform 1** | 44.5 | 4 | 4 | 78 | 229 | 1.04E+09 | **1.18** | 1 | - | 2364 | 274.4 | 5.6 | Cell growth and/or maintenance | Cytoplasm;Plasma membrane;Cytoskeleton;Nucleus | Structural constituent of cytoskeleton | Cytoskeleton |
| **FXR2** | NP_004851.2 | 9513 | **fragile X mental retardation syndrome-related protein 2** | 8.0 | 1 | 1 | 3 | 15 | 7.45E+07 | **1.18** | 1 | - | 673 | 74.2 | 6.2 | Regulation of nucleobase, nucleoside, nucleotide and nucleic acid metabolism | Cytoplasm;Ribosome;Nucleolus;Nucleus | RNA binding | Cytoplasm |
| **PRKCI** | NP_002731.4 | 5584 | **protein kinase C iota type** | 40.4 | 4 | 11 | 11 | 17 | 1.59E+08 | **1.18** | 9 | 11.5 | 596 | 68.2 | 5.9 | Cell communication;Signal transduction | Cytoplasm;Nucleus;Plasma membrane;Microsome | Protein serine/threonine kinase activity | Cytoplasm |
| **USP8** | NP_001122082.1 | 9101 | **ubiquitin carboxyl-terminal hydrolase 8 isoform a** | 5.2 | 2 | 3 | 3 | 3 | 4.55E+07 | **1.18** | 1 | - | 1118 | 127.4 | 8.5 | Protein metabolism | Nucleus | Ubiquitin-specific protease activity | Nucleus |
| **COA4** | NP_057649.2 | 51287 | **cytochrome c oxidase assembly factor 4 homolog, mitochondrial** | 58.6 | 1 | 3 | 3 | 7 | 2.69E+08 | **1.18** | 2 | 11.8 | 87 | 10.1 | 6.0 | Biological_process unknown | - | Molecular function unknown | - |
| **FYTTD1** | NP_115664.2 | 84248 | **UAP56-interacting factor isoform 1** | 19.2 | 2 | 5 | 5 | 7 | 5.94E+07 | **1.18** | 3 | 10.7 | 318 | 35.8 | 11.8 | Biological_process unknown | - | Molecular function unknown | - |
| **PPIF** | NP_005720.1 | 10105 | **peptidyl-prolyl cis-trans isomerase F, mitochondrial precursor** | 44.9 | 1 | 5 | 6 | 26 | 5.99E+08 | **1.18** | 4 | 10.6 | 207 | 22 | 9.4 | Protein metabolism | Mitochondrion;Nucleolus | Isomerase activity | Mitochondrion |
| **WDR11** | NP_060587.8 | 55717 | **WD repeat-containing protein 11** | 8.8 | 1 | 6 | 6 | 7 | 1.13E+08 | **1.18** | 4 | 20.1 | 1224 | 136.6 | 6.9 | Biological_process unknown | - | Molecular function unknown | - |
| **ACTC1** | NP_005150.1 | 70 | **actin, alpha cardiac muscle 1 proprotein** | 47.8 | 5 | 9 | 26 | 1360 | 2.39E+10 | **1.18** | 27 | 14.8 | 377 | 42 | 5.4 | Cell growth and/or maintenance | Cytoplasm;Nucleus | Structural constituent of cytoskeleton | Cytoplasm |
| **NANS** | NP_061819.2 | 54187 | **sialic acid synthase** | 61.6 | 1 | 16 | 16 | 61 | 9.08E+08 | **1.18** | 19 | 17.3 | 359 | 40.3 | 6.7 | Metabolism;Energy pathways | Cytoplasm;Cytosol | Catalytic activity | Cytoplasm |

| Gene Symbol | RefSeq_Accession | Entrez Gene ID | Description | Coverage (%) | Proteins | Unique Peptides | # Peptides | PSM | Area | SCC-R /SCC-S | SCC-R /SCC-S (Count) | SCC-R /SCC-S (Variability [%]) | # AAs | MW [kDa] | calc. pI | Biological Process | Localization | Molecular Function | Primary Localization |
|---|---|---|---|---|---|---|---|---|---|---|---|---|---|---|---|---|---|---|---|
| **ND1** | YP_003024026.1 | 4535 | **NADH dehydrogenase subunit 1** | 6.3 | 1 | 2 | 2 | 8 | 1.15E+08 | **1.18** | 2 | 23.3 | 318 | 35.6 | 6.6 | Metabolism;Energy pathways | Mitochondrion | Catalytic activity | Mitochondrion |
| **UBE3A** | NP_570854.1 | 7337 | **ubiquitin-protein ligase E3A isoform 3** | 7.9 | 3 | 4 | 4 | 6 | 3.10E+07 | **1.18** | 3 | 13.2 | 872 | 100 | 5.2 | Metabolism;Energy pathways | Cytoplasm;Nucleus;Mitochondrion | Ubiquitin-specific protease activity | Cytoplasm |
| **EIF4G3** | NP_003751.2 | 8672 | **eukaryotic translation initiation factor 4 gamma 3 isoform 3** | 6.2 | 4 | 4 | 10 | 21 | 4.37E+08 | **1.18** | 3 | 2 | 1585 | 176.5 | 5.4 | Protein metabolism | Cytoplasm | Translation regulator activity | Cytoplasm |
| **GRHPR** | NP_036335.1 | 9380 | **glyoxylate reductase/hydroxypyruvate reductase** | 56.4 | 1 | 12 | 12 | 50 | 5.25E+08 | **1.18** | 12 | 32.9 | 328 | 35.6 | 7.4 | Metabolism;Energy pathways | Cytoplasm | Catalytic activity | Cytoplasm |
| **TRIP11** | NP_004230.2 | 9321 | **thyroid receptor-interacting protein 11** | 6.9 | 1 | 8 | 8 | 12 | 4.65E+07 | **1.17** | 6 | 27.1 | 1979 | 227.4 | 5.3 | Regulation of nucleobase, nucleoside, nucleotide and nucleic acid metabolism | Centrosome;Golgi apparatus;Nucleus;Cytoplasm | Receptor binding | Golgi apparatus |
| **SF3B2** | NP_006833.2 | 10992 | **splicing factor 3B subunit 2** | 56.0 | 1 | 35 | 35 | 155 | 1.02E+09 | **1.17** | 56 | 36.5 | 895 | 100.2 | 5.7 | Regulation of nucleobase, nucleoside, nucleotide and nucleic acid metabolism | Nucleus;Nucleolus;Cytoplasm | RNA binding | Nucleus |
| **BABAM1** | NP_001275686.1 | 29086 | **BRISC and BRCA1-A complex member 1 isoform 2** | 23.6 | 2 | 3 | 3 | 11 | 4.52E+07 | **1.17** | 4 | 20 | 254 | 28.1 | 4.5 | Biological_process unknown | - | Molecular function unknown | - |
| **CCDC12** | NP_001264003.1 | 151903 | **coiled-coil domain-containing protein 12 2** | 51.8 | 2 | 6 | 6 | 8 | 1.93E+08 | **1.17** | 4 | 6.5 | 166 | 19.2 | 7.3 | Biological_process unknown | - | Molecular function unknown | - |
| **ACTB** | NP_001092.1 | 60 | **actin, cytoplasmic 1** | 82.4 | 5 | 3 | 43 | 3831 | 2.82E+10 | **1.17** | 13 | 15.7 | 375 | 41.7 | 5.5 | Cell growth and/or maintenance | Cytoplasm;Golgi apparatus;Plasma membrane;Nucleolus;Cytosol;Extracellular;Cytoskeleton;Mitochondrion | Structural constituent of cytoskeleton | Cytoplasm |
| **EFTUD2** | NP_001245282.1 | 9343 | **116 kDa U5 small nuclear ribonucleoprotein component isoform a** | 64.5 | 3 | 39 | 40 | 148 | 2.28E+09 | **1.17** | 40 | 18.1 | 972 | 109.4 | 5.0 | Protein metabolism | Nucleus;Nucleolus;Cytoplasm;Chromosome | GTPase activity | Nucleus |
| **LUC7L3** | NP_057508.2 | 51747 | **luc7-like protein 3** | 19.2 | 1 | 7 | 7 | 13 | 7.21E+07 | **1.17** | 6 | 13.8 | 432 | 51.4 | 9.8 | Regulation of nucleobase, nucleoside, nucleotide and nucleic acid metabolism | Nucleus | Transcription regulator activity | Nucleus |
| **SMAP2** | NP_001185909.1 | 64744 | **stromal membrane-associated protein 2 isoform 4** | 13.5 | 4 | 2 | 2 | 3 | 3.45E+07 | **1.17** | 1 | - | 349 | 37.7 | 9.1 | Cell communication;Signal transduction | - | GTPase activator activity | - |
| **TXNDC17** | NP_116120.1 | 84817 | **thioredoxin domain-containing protein 17** | 62.6 | 1 | 7 | 7 | 27 | 8.43E+08 | **1.17** | 11 | 13.9 | 123 | 13.9 | 5.5 | Metabolism;Energy pathways | Cytoplasm | Oxidoreductase activity | Cytoplasm |
| **BAG2** | NP_004273.1 | 9532 | **BAG family molecular chaperone regulator 2** | 46.0 | 1 | 7 | 7 | 36 | 2.95E+08 | **1.17** | 13 | 22.2 | 211 | 23.8 | 6.7 | Apoptosis | Nucleus;Cytosol | Receptor signaling complex scaffold activity | Nucleus |
| **KCMF1** | NP_064507.3 | 56888 | **E3 ubiquitin-protein ligase KCMF1** | 11.8 | 1 | 4 | 4 | 10 | 4.55E+07 | **1.17** | 3 | 18.8 | 381 | 41.9 | 5.7 | Protein metabolism | Nucleus | Ubiquitin-specific protease activity | Nucleus |
| **CAPZA1** | NP_006126.1 | 829 | **F-actin-capping protein subunit alpha-1** | 70.3 | 1 | 11 | 12 | 94 | 3.28E+09 | **1.17** | 19 | 38.6 | 286 | 32.9 | 5.7 | Cell growth and/or maintenance | Cytoplasm | Structural molecule activity | Cytoplasm |
| **NUP214** | NP_005076.3 | 8021 | **nuclear pore complex protein Nup214** | 21.5 | 1 | 26 | 26 | 43 | 2.51E+08 | **1.17** | 18 | 37.1 | 2090 | 213.5 | 7.5 | Transport | Nucleus;Nuclear membrane | Transporter activity | Nucleus |
| **RIPK2** | NP_003812.1 | 8767 | **receptor-interacting serine/threonine-protein kinase 2** | 2.8 | 1 | 1 | 1 | 2 | 1.48E+07 | **1.17** | 1 | - | 540 | 61.2 | 7.1 | Cell communication;Signal transduction | Cytoplasm | Protein serine/threonine kinase activity | Cytoplasm |
| **SNX12** | NP_037478.2 | 29934 | **sorting nexin-12 isoform 1** | 29.6 | 4 | 3 | 5 | 10 | 1.44E+08 | **1.17** | 3 | 2.9 | 162 | 18.9 | 8.4 | Protein metabolism | Cytoplasm;Endosome | Molecular function unknown | Cytoplasm |
| **TMED1** | NP_006849.1 | 11018 | **transmembrane emp24 domain-containing protein 1 precursor** | 11.5 | 1 | 3 | 3 | 6 | 7.39E+07 | **1.17** | 3 | 26.7 | 227 | 25.2 | 4.5 | Cell communication;Signal transduction | Plasma membrane | Receptor binding | Plasma membrane |
| **COPG1** | NP_057212.1 | 22820 | **coatomer subunit gamma-1** | 60.0 | 1 | 29 | 30 | 138 | 7.43E+08 | **1.17** | 35 | 22.3 | 874 | 97.7 | 5.5 | Transport | Golgi apparatus | Transporter activity | Golgi apparatus |
| **NCBP2** | NP_001036005.1 | 22916 | **nuclear cap-binding protein subunit 2 isoform 2** | 21.4 | 2 | 2 | 2 | 3 | 4.76E+07 | **1.17** | 2 | 17.8 | 103 | 11.9 | 8.9 | Regulation of nucleobase, nucleoside, nucleotide and nucleic acid metabolism | Nucleus | RNA binding | Nucleus |
| **ZFYVE19** | NP_001245350.1 | 84936 | **zinc finger FYVE domain-containing protein 19 isoform 4** | 10.1 | 4 | 2 | 2 | 4 | 5.16E+07 | **1.17** | 1 | - | 296 | 32.9 | 4.7 | Protein metabolism | Cytoplasm | Ubiquitin-specific protease activity | Cytoplasm |
| **ATP1B1** | NP_001668.1 | 481 | **sodium/potassium-transporting ATPase subunit beta-1** | 26.7 | 1 | 5 | 5 | 12 | 4.30E+08 | **1.17** | 8 | 16.4 | 303 | 35 | 8.5 | Transport | Plasma membrane | ATPase activity | Plasma membrane |
| **MPHOSPH6** | NP_005783.2 | 10200 | **M-phase phosphoprotein 6** | 9.4 | 1 | 1 | 1 | 1 | 2.79E+07 | **1.17** | 1 | - | 160 | 19 | 5.2 | Cell communication;Signal transduction | Nucleus;Nucleolus;Cytoplasm | Protein binding | Nucleus |
| **PANK4** | NP_060686.1 | 55229 | **pantothenate kinase 4** | 3.1 | 1 | 2 | 2 | 3 | 7.58E+07 | **1.17** | 2 | 33.9 | 773 | 85.9 | 6.3 | Metabolism;Energy pathways | - | Catalytic activity | - |
| **WASF2** | NP_008921.1 | 10163 | **wiskott-Aldrich syndrome protein family member 2 isoform 1** | 19.7 | 4 | 5 | 5 | 15 | 1.05E+08 | **1.17** | 4 | 26.1 | 498 | 54.3 | 5.5 | Cell growth and/or maintenance | Cytoplasm | Cytoskeletal protein binding | Cytoplasm |
| **WDR33** | NP_060853.3 | 55339 | **pre-mRNA 3' end processing protein WDR33 isoform 1** | 10.9 | 3 | 7 | 7 | 9 | 3.82E+07 | **1.17** | 6 | 19.7 | 1336 | 145.8 | 9.2 | Cell growth and/or maintenance | Nucleus;Cytoplasm | Molecular function unknown | Nucleus |
| **ETFA** | NP_000117.1 | 2108 | **electron transfer flavoprotein subunit alpha, mitochondrial isoform a** | 65.2 | 2 | 15 | 15 | 65 | 1.14E+09 | **1.17** | 25 | 22.5 | 333 | 35.1 | 8.4 | Metabolism;Energy pathways | Mitochondrion | Auxiliary transport protein activity | Mitochondrion |
| **COG3** | NP_113619.2 | 83548 | **conserved oligomeric Golgi complex subunit 3** | 0.7 | 1 | 1 | 1 | 1 | 2.21E+07 | **1.16** | 1 | - | 828 | 94 | 5.6 | Transport | Golgi apparatus;Cytoplasm | Transporter activity | Golgi apparatus |
| **FAM134A** | NP_077269.3 | 79137 | **protein FAM134A** | 4.6 | 1 | 1 | 1 | 4 | 1.20E+08 | **1.16** | 1 | - | 543 | 57.8 | 4.5 | Biological_process unknown | Integral to membrane;Nucleus;Cytoplasm | Molecular function unknown | Integral to membrane |
| **GIPC1** | NP_974199.1 | 10755 | **PDZ domain-containing protein GIPC1 isoform 1** | 34.5 | 2 | 8 | 8 | 20 | 1.94E+08 | **1.16** | 6 | 32 | 333 | 36 | 6.3 | Signal transduction | Cytoplasmic vesicle membrane;Cytoplasm | Binding | Cytoplasm |
| **PTPLAD1** | NP_057479.2 | 51495 | **very-long-chain (3R)-3-hydroxyacyl-[acyl-carrier protein] dehydratase 3** | 17.1 | 1 | 4 | 4 | 16 | 1.49E+08 | **1.16** | 5 | 32.2 | 362 | 43.1 | 8.9 | Cell communication;Signal transduction | Mitochondrion;Integral to membrane | Molecular function unknown | Mitochondrion;Integral to membrane |
| **MRPS6** | NP_115865.1 | 64968 | **28S ribosomal protein S6, mitochondrial** | 10.4 | 1 | 1 | 1 | 3 | 2.03E+08 | **1.16** | 1 | - | 125 | 14.2 | 9.3 | Protein metabolism | Mitochondrion | Structural constituent of ribosome | Mitochondrion |
| **SIRT5** | NP_001229756.1 | 23408 | **NAD-dependent protein deacylase sirtuin-5, mitochondrial isoform 4** | 28.7 | 4 | 4 | 4 | 8 | 6.62E+07 | **1.16** | 2 | 11.7 | 202 | 21.7 | 6.4 | Regulation of nucleobase, nucleoside, nucleotide and nucleic acid metabolism | Nucleus | Deacetylase activity;Translation regulator activity | Nucleus |
| **TCP1** | NP_110379.2 | 6950 | **T-complex protein 1 subunit alpha isoform a** | 71.9 | 2 | 31 | 31 | 225 | 1.65E+09 | **1.16** | 57 | 27.2 | 556 | 60.3 | 6.1 | Protein metabolism | Cytoplasm;Nucleus;Cytosol;Microtubule | Chaperone activity | Cytoplasm |
| **CDC34** | NP_004350.1 | 997 | **ubiquitin-conjugating enzyme E2 R1** | 4.2 | 1 | 1 | 1 | 1 | 4.21E+07 | **1.16** | 1 | - | 236 | 26.7 | 4.5 | Protein metabolism | Nucleus;Cytoplasm | Ligase activity | Nucleus |

| Gene Symbol | RefSeq_Accession | Entrez Gene ID | Description | Coverage (%) | Proteins | Unique Peptides | # Peptides | PSM | Area | SCC-R /SCC-S | SCC-R /SCC-S (Count) | SCC-R /SCC-S (Variability [%]) | # AAs | MW [kDa] | calc. pI | Biological Process | Localization | Molecular Function | Primary Localization |
|---|---|---|---|---|---|---|---|---|---|---|---|---|---|---|---|---|---|---|---|
| **COA7** | NP_075565.2 | 65260 | **cytochrome c oxidase assembly factor 7** | 22.9 | 1 | 3 | 3 | 9 | 1.15E+08 | **1.16** | 5 | 17.8 | 231 | 25.7 | 6.0 | Biological_process unknown | - | Molecular function unknown | - |
| **PARK7** | NP_001116849.1 | 11315 | **protein DJ-1** | 83.1 | 1 | 18 | 18 | 170 | 1.55E+09 | **1.16** | 34 | 15.1 | 189 | 19.9 | 6.8 | Regulation of nucleobase, nucleoside, nucleotide and nucleic acid metabolism | Cytoplasm;Nucleus;Mitochondrion;Extracellular | RNA binding | Cytoplasm |
| **SRSF3** | NP_003008.1 | 6428 | **serine/arginine-rich splicing factor 3** | 43.9 | 1 | 9 | 10 | 100 | 2.60E+09 | **1.16** | 20 | 15.2 | 164 | 19.3 | 11.7 | Regulation of nucleobase, nucleoside, nucleotide and nucleic acid metabolism | Nucleus;Cytoplasm;Nucleolus;Mitochondrion | RNA binding | Nucleus |
| **BAG1** | NP_001165886.1 | 573 | **BAG family molecular chaperone regulator 1 isoform BAG-1S** | 4.4 | 2 | 1 | 1 | 1 | 3.96E+07 | **1.16** | 1 | - | 230 | 26 | 5.2 | Regulation of nucleobase, nucleoside, nucleotide and nucleic acid metabolism | Nucleus;Cytoplasm | Transcription factor activity | Nucleus |
| **CAPZB** | NP_004921.1 | 832 | **F-actin-capping protein subunit beta isoform 1** | 65.4 | 4 | 17 | 17 | 75 | 1.07E+09 | **1.16** | 25 | 19.5 | 272 | 30.6 | 6.0 | Cell growth and/or maintenance | Cytoskeleton;Mitochondrion | Structural constituent of cytoskeleton | Cytoskeleton |
| **CLTC** | NP_004850.1 | 1213 | **clathrin heavy chain 1 isoform 1** | 61.9 | 4 | 88 | 88 | 788 | 6.10E+09 | **1.16** | 192 | 16.9 | 1675 | 191.5 | 5.7 | Cell growth and/or maintenance | Cytoplasm;Plasma membrane;Golgi apparatus;Endosome;Microtubule;Mitochondrion | Structural molecule activity | Cytoplasm |
| **NAE1** | NP_001018169.1 | 8883 | **NEDD8-activating enzyme E1 regulatory subunit isoform b** | 23.7 | 4 | 7 | 7 | 12 | 1.20E+08 | **1.16** | 8 | 15.3 | 528 | 59.4 | 5.2 | Cell communication;Signal transduction | Cytoplasm | Receptor signaling complex scaffold activity | Cytoplasm |
| **TMEM205** | NP_001138888.1 | 374882 | **transmembrane protein 205** | 13.8 | 1 | 2 | 2 | 3 | 4.44E+07 | **1.16** | 1 | - | 189 | 21.2 | 8.6 | Biological_process unknown | Integral to membrane | Molecular function unknown | Integral to membrane |
| **SNRPG** | NP_003087.1 | 6637 | **small nuclear ribonucleoprotein G** | 26.3 | 1 | 2 | 2 | 19 | 5.26E+08 | **1.16** | 6 | 16.3 | 76 | 8.5 | 8.9 | Regulation of nucleobase, nucleoside, nucleotide and nucleic acid metabolism | Nucleus;Cytoplasm | RNA binding | Nucleus |
| **TOM1L2** | NP_001028723.1 | 146691 | **TOM1-like protein 2 isoform 1** | 12.0 | 6 | 3 | 3 | 3 | 1.70E+07 | **1.16** | 1 | - | 457 | 49.9 | 4.7 | Transport | - | Transporter activity | - |
| **VWA5B2** | NP_612354.1 | 90113 | **von Willebrand factor A domain-containing protein 5B2** | 1.9 | 1 | 1 | 2 | 4 | 1.38E+09 | **1.16** | 1 | - | 1242 | 131.6 | 6.5 | - | - | - | - |
| **ELP3** | NP_060561.3 | 55140 | **elongator complex protein 3 isoform 1** | 5.9 | 5 | 2 | 2 | 3 | 7.06E+07 | **1.16** | 2 | 24.9 | 547 | 62.2 | 8.9 | Metabolism;Energy pathways | Nucleus;Cytoplasm | Acyltransferase activity | Nucleus |
| **PIH1D1** | NP_060386.1 | 55011 | **PIH1 domain-containing protein 1** | 22.1 | 1 | 3 | 3 | 4 | 1.55E+08 | **1.16** | 1 | - | 290 | 32.3 | 5.1 | Biological_process unknown | - | Molecular function unknown | - |
| **SFT2D3** | NP_116129.3 | 84826 | **vesicle transport protein SFT2C** | 14.0 | 1 | 1 | 1 | 4 | 1.05E+08 | **1.16** | 1 | - | 215 | 21.8 | 9.9 | Vesicle-mediated transport | - | Transporter activity | - |
| **SFXN1** | NP_073591.2 | 94081 | **sideroflexin-1** | 59.9 | 1 | 12 | 14 | 71 | 3.74E+08 | **1.16** | 18 | 26.2 | 322 | 35.6 | 9.1 | Transport | Mitochondrion;Plasma membrane;Cytoplasm | Transporter activity | Mitochondrion |
| **DKC1** | NP_001354.1 | 1736 | **H/ACA ribonucleoprotein complex subunit 4 isoform 1** | 44.9 | 3 | 16 | 16 | 65 | 8.05E+08 | **1.16** | 26 | 20.1 | 514 | 57.6 | 9.4 | Regulation of nucleobase, nucleoside, nucleotide and nucleic acid metabolism | Nucleus;Nucleolus | RNA binding | Nucleus |
| **FIP1L1** | NP_112179.2 | 81608 | **pre-mRNA 3'-end-processing factor FIP1 isoform 1** | 21.0 | 3 | 5 | 5 | 13 | 7.92E+07 | **1.16** | 5 | 22.9 | 594 | 66.5 | 5.6 | Biological_process unknown | Nucleus | Molecular function unknown | Nucleus |
| **NCOR1** | NP_001177369.1 | 9611 | **nuclear receptor corepressor 1 isoform 3** | 2.3 | 2 | 3 | 3 | 4 | 1.92E+07 | **1.16** | 2 | 5.7 | 2337 | 258.4 | 6.9 | Regulation of nucleobase, nucleoside, nucleotide and nucleic acid metabolism | Nucleus;Cytoplasm | Transcription factor activity | Nucleus;Cytoplasm |
| **STRIP1** | NP_001257697.1 | 85369 | **striatin-interacting protein 1 isoform 2** | 4.7 | 2 | 1 | 2 | 2 | 3.72E+07 | **1.16** | 1 | - | 742 | 85.4 | 6.7 | Biological_process unknown | - | Molecular function unknown | - |
| **VPS8** | NP_056118.2 | 23355 | **vacuolar protein sorting-associated protein 8 homolog isoform b** | 1.3 | 2 | 1 | 1 | 1 | 1.40E+07 | **1.16** | 1 | - | 1426 | 161.5 | 5.6 | Biological_process unknown | - | Molecular function unknown | - |
| **P4HB** | NP_000909.2 | 5034 | **protein disulfide-isomerase precursor** | 74.0 | 1 | 32 | 32 | 178 | 2.42E+09 | **1.16** | 52 | 20.4 | 508 | 57.1 | 4.9 | Protein metabolism | Endoplasmic reticulum;Nucleus;Extracellular;Nucleolus;Zymogen granule;Mitochondrion | Isomerase activity | Endoplasmic reticulum |
| **ATP6V1A** | NP_001681.2 | 523 | **V-type proton ATPase catalytic subunit A** | 39.6 | 1 | 17 | 17 | 68 | 5.10E+08 | **1.15** | 23 | 16.4 | 617 | 68.3 | 5.5 | Metabolism;Energy pathways | Plasma membrane;Lysosome;Endosome;Secretory vesicle | Transporter activity | Endosome |
| **DDT** | NP_001346.1 | 1652 | **D-dopachrome decarboxylase** | 50.9 | 3 | 7 | 7 | 33 | 9.14E+08 | **1.15** | 14 | 13.7 | 118 | 12.7 | 7.3 | Immune response | Cytoplasm | Isomerase activity | Cytoplasm |
| **HRSP12** | NP_005827.1 | 10247 | **ribonuclease UK114** | 25.6 | 1 | 2 | 2 | 11 | 1.76E+08 | **1.15** | 3 | 14.8 | 137 | 14.5 | 8.7 | Protein metabolism | Plasma membrane;Cytoplasm;Golgi apparatus;Endoplasmic reticulum;Nucleus | Translation regulator activity | Cytoplasm |
| **NT5C3A** | NP_001002009.1 | 51251 | **cytosolic 5'-nucleotidase 3A isoform 2** | 33.7 | 3 | 7 | 7 | 11 | 3.31E+08 | **1.15** | 8 | 26.9 | 297 | 33.9 | 5.9 | Metabolism;Energy pathways | Cytoplasm | Hydrolase activity | Cytoplasm |
| **SNRPB** | NP_003082.1 | 6628 | **small nuclear ribonucleoprotein-associated proteins B and B' isoform B** | 23.4 | 3 | 8 | 8 | 48 | 1.27E+09 | **1.15** | 19 | 38.6 | 231 | 23.6 | 10.9 | Regulation of nucleobase, nucleoside, nucleotide and nucleic acid metabolism | Cytoplasm;Nucleus;Ribosome | RNA binding | Nucleus |
| **SNRPD2** | NP_004588.1 | 6633 | **small nuclear ribonucleoprotein Sm D2 isoform 1** | 67.8 | 2 | 11 | 11 | 77 | 1.52E+09 | **1.15** | 23 | 25.6 | 118 | 13.5 | 9.9 | Regulation of nucleobase, nucleoside, nucleotide and nucleic acid metabolism | Nucleus | RNA binding | Nucleus |
| **EPN1** | NP_037465.2 | 29924 | **epsin-1 isoform c** | 8.4 | 3 | 2 | 2 | 2 | 1.04E+07 | **1.15** | 1 | - | 550 | 57.5 | 4.9 | Cell communication;Signal transduction | Cytoplasm;Plasma membrane;Nucleus | Receptor signaling complex scaffold activity | Cytoplasm |
| **PRKACB** | NP_002722.1 | 5567 | **cAMP-dependent protein kinase catalytic subunit beta isoform 2** | 15.1 | 10 | 1 | 5 | 9 | 1.02E+08 | **1.15** | 1 | - | 351 | 40.6 | 8.8 | Cell communication;Signal transduction | Cytoplasm | Protein serine/threonine kinase activity | Cytoplasm |
| **SSU72** | NP_054907.1 | 29101 | **RNA polymerase II subunit A C-terminal domain phosphatase SSU72** | 13.4 | 1 | 2 | 2 | 2 | 3.15E+07 | **1.15** | 2 | 52.2 | 194 | 22.6 | 5.3 | Biological_process unknown | - | Molecular function unknown | - |
| **ACTR2** | NP_005713.1 | 10097 | **actin-related protein 2 isoform b** | 48.2 | 2 | 14 | 14 | 94 | 2.17E+09 | **1.15** | 26 | 15.5 | 394 | 44.7 | 6.7 | Cell motility;Cell growth and/or maintenance | Cytoplasm;Actin cytoskeleton | Structural constituent of cytoskeleton | Actin cytoskeleton |
| **CETN2** | NP_004335.1 | 1069 | **centrin-2** | 43.0 | 2 | 4 | 4 | 4 | 1.44E+07 | **1.15** | 2 | 24.4 | 172 | 19.7 | 5.0 | Cell communication;Signal transduction | Centrosome;Nucleus;Cytoplasm | Calcium ion binding | Centrosome |
| **PRPF38A** | NP_116253.2 | 84950 | **pre-mRNA-splicing factor 38A** | 15.7 | 1 | 3 | 3 | 4 | 3.07E+07 | **1.15** | 1 | - | 312 | 37.5 | 10.0 | Biological_process unknown | - | Molecular function unknown | - |
| **BOP1** | NP_056016.1 | 23246 | **ribosome biogenesis protein BOP1** | 19.0 | 1 | 6 | 6 | 17 | 1.27E+08 | **1.15** | 3 | 0.9 | 746 | 83.6 | 6.2 | Biological_process unknown | Nucleolus;Nucleus | Molecular function unknown | Nucleolus |
| **EXOC7** | NP_056034.2 | 23265 | **exocyst complex component 7 isoform 2** | 10.4 | 7 | 5 | 5 | 8 | 1.44E+08 | **1.15** | 4 | 31.8 | 653 | 74.7 | 6.4 | Biological_process unknown | Cytoplasm;Plasma membrane | Molecular function unknown | Cytoplasm |
| **NFIC** | NP_005588.2 | 4782 | **nuclear factor 1 C-type isoform 5** | 12.9 | 12 | 2 | 3 | 4 | 2.61E+07 | **1.15** | 1 | - | 428 | 47.6 | 8.2 | Regulation of nucleobase, nucleoside, nucleotide and nucleic acid metabolism | Nucleus | Transcription factor activity | Nucleus |
| **PSMC1** | NP_002793.2 | 5700 | **26S protease regulatory subunit 4** | 43.9 | 1 | 15 | 16 | 69 | 8.64E+08 | **1.15** | 19 | 12 | 440 | 49.2 | 6.2 | Protein metabolism | Nucleus;Cytoplasm | Ubiquitin-specific protease activity | Cytoplasm |

| Gene Symbol | RefSeq_Accession | Entrez Gene ID | Description | Coverage (%) | Proteins | Unique Peptides | # Peptides | PSM | Area | SCC-R /SCC-S | SCC-R /SCC-S (Count) | SCC-R /SCC-S (Variability [%]) | # AAs | MW [kDa] | calc. pI | Biological Process | Localization | Molecular Function | Primary Localization |
|---|---|---|---|---|---|---|---|---|---|---|---|---|---|---|---|---|---|---|---|
| RSL1D1 | NP_056474.2 | 26156 | ribosomal L1 domain-containing protein 1 | 48.0 | 1 | 22 | 22 | 94 | 6.45E+08 | 1.15 | 36 | 20.5 | 490 | 54.9 | 10.1 | Biological_process unknown | Nucleolus;Ribosome;Nucleus;Cytoplasm | Molecular function unknown | Nucleolus |
| ARHGAP29 | NP_004806.3 | 9411 | rho GTPase-activating protein 29 | 6.7 | 1 | 4 | 4 | 4 | 4.56E+07 | 1.15 | 1 | - | 1261 | 142 | 6.7 | Cell communication;Signal transduction | Cytoplasm | GTPase activator activity | Cytoplasm |
| BOLA1 | NP_057158.1 | 51027 | bolA-like protein 1 precursor | 16.1 | 1 | 1 | 1 | 1 | 7.44E+05 | 1.15 | 1 | - | 137 | 14.3 | 8.0 | Biological_process unknown | - | Molecular function unknown | - |
| EIF4E2 | NP_001263265.1 | 9470 | eukaryotic translation initiation factor 4E type 2 isoform C | 14.1 | 4 | 2 | 2 | 2 | 9.61E+07 | 1.15 | 1 | - | 234 | 27 | 8.0 | Protein metabolism | Cytoplasm | Translation regulator activity | Cytoplasm |
| REV3L | NP_001273361.1 | 5980 | DNA polymerase zeta catalytic subunit isoform b | 0.2 | 2 | 1 | 1 | 2 | 4.75E+07 | 1.15 | 1 | - | 3052 | 343.9 | 8.5 | Regulation of nucleobase, nucleoside, nucleotide and nucleic acid metabolism | Nucleus | DNA-directed DNA polymerase activity | Nucleus |
| SRPK1 | NP_003128.3 | 6732 | SRSF protein kinase 1 | 16.3 | 4 | 5 | 6 | 11 | 2.21E+08 | 1.15 | 5 | 32.4 | 655 | 74.3 | 6.2 | Regulation of nucleobase, nucleoside, nucleotide and nucleic acid metabolism | Nucleus;Cytoplasm | Protein threonine/tyrosine kinase activity | Nucleus |
| XRCC1 | NP_006288.2 | 7515 | DNA repair protein XRCC1 | 6.6 | 1 | 3 | 3 | 5 | 1.04E+08 | 1.15 | 4 | 4.5 | 633 | 69.5 | 6.3 | DNA repair | Nucleus;Nucleolus;Cytoplasm | DNA repair protein | Nucleus |
| ATOX1 | NP_004036.1 | 475 | copper transport protein ATOX1 | 76.5 | 1 | 5 | 5 | 18 | 1.24E+09 | 1.15 | 11 | 16.5 | 68 | 7.4 | 7.2 | Protein metabolism | Cytoplasm;Nucleus | Chaperone activity | Cytoplasm |
| NUMA1 | NP_001273490.1 | 4926 | nuclear mitotic apparatus protein 1 isoform 2 | 53.9 | 2 | 82 | 82 | 304 | 6.96E+08 | 1.15 | 109 | 16.3 | 2101 | 236.4 | 5.8 | Cell growth and/or maintenance | Nucleus;Nucleolus;Cytosol;Microtubule;Mitochondrion;Cytoplasm | Structural molecule activity | Nucleus |
| POLE3 | NP_059139.3 | 54107 | DNA polymerase epsilon subunit 3 | 33.3 | 1 | 5 | 5 | 22 | 1.90E+08 | 1.15 | 1 | - | 147 | 16.8 | 4.7 | Regulation of nucleobase, nucleoside, nucleotide and nucleic acid metabolism | Nucleus | DNA binding | Nucleus |
| HSPE1 | NP_002148.1 | 3336 | 10 kDa heat shock protein, mitochondrial | 85.3 | 1 | 16 | 16 | 123 | 3.09E+09 | 1.15 | 40 | 17.9 | 102 | 10.9 | 8.9 | Protein metabolism | Mitochondrion;Nucleolus | Heat shock protein activity | Mitochondrion |
| TPD52 | NP_005070.1 | 7163 | tumor protein D52 isoform 3 | 66.9 | 7 | 8 | 8 | 22 | 3.26E+08 | 1.15 | 8 | 27.5 | 184 | 19.9 | 5.0 | Cell proliferation | - | Molecular function unknown | - |
| UMPS | NP_000364.1 | 7372 | uridine 5'-monophosphate synthase | 36.7 | 1 | 10 | 10 | 27 | 2.96E+08 | 1.15 | 9 | 14.6 | 480 | 52.2 | 7.2 | Metabolism;Energy pathways | Nucleus | Transferase activity | Nucleus |
| COPE | NP_009194.2 | 11316 | coatomer subunit epsilon isoform a | 73.7 | 3 | 12 | 12 | 46 | 7.11E+08 | 1.15 | 17 | 24.9 | 308 | 34.5 | 5.1 | Transport | Cytoplasm;Golgi apparatus;Endoplasmic reticulum | Transporter activity | Cytoplasm |
| PRPF4 | NP_001231855.1 | 9128 | U4/U6 small nuclear ribonucleoprotein Prp4 isoform 2 | 21.5 | 2 | 7 | 7 | 14 | 3.15E+08 | 1.15 | 6 | 23.8 | 521 | 58.3 | 7.4 | Regulation of nucleobase, nucleoside, nucleotide and nucleic acid metabolism | Nucleus;Cytoplasm;Nucleolus;Ribosome | Ribonucleoprotein | Nucleus |
| ARF4 | NP_001651.1 | 378 | ADP-ribosylation factor 4 | 85.0 | 6 | 8 | 14 | 78 | 5.66E+08 | 1.14 | 8 | 22.9 | 180 | 20.5 | 7.1 | Transport | Plasma membrane;Cytoplasm;Golgi apparatus | Transporter activity | Cytoplasm |
| CCNB1 | NP_114172.1 | 891 | G2/mitotic-specific cyclin-B1 | 3.9 | 1 | 1 | 1 | 1 | 1.11E+08 | 1.14 | 1 | - | 433 | 48.3 | 7.5 | Cell communication;Signal transduction | Cytoplasm;Nucleus;Plasma membrane;Centrosome;Nucleolus | Kinase binding | Cytoplasm |
| CCT5 | NP_036205.1 | 22948 | T-complex protein 1 subunit epsilon | 85.0 | 1 | 42 | 43 | 332 | 3.65E+09 | 1.14 | 65 | 18.6 | 541 | 59.6 | 5.7 | Protein metabolism | Centrosome;Cytoplasm | Chaperone activity | Centrosome |
| PHB | NP_001268425.1 | 5245 | prohibitin isoform 1 | 70.6 | 2 | 20 | 20 | 174 | 1.10E+09 | 1.14 | 46 | 16.9 | 272 | 29.8 | 5.8 | Cell communication;Signal transduction | Mitochondrion;Plasma membrane;Nucleus;Nucleolus;Extracellular;Cytoplasm | Receptor signaling complex scaffold activity | Mitochondrion |
| SF3A1 | NP_005868.1 | 10291 | splicing factor 3A subunit 1 isoform 1 | 35.6 | 2 | 26 | 26 | 71 | 8.15E+08 | 1.14 | 30 | 25.6 | 793 | 88.8 | 5.2 | Regulation of nucleobase, nucleoside, nucleotide and nucleic acid metabolism | Nucleus | RNA binding | Nucleus |
| IWS1 | NP_060439.2 | 55677 | protein IWS1 homolog | 8.4 | 1 | 5 | 5 | 13 | 4.12E+07 | 1.14 | 6 | 54.1 | 819 | 91.9 | 4.7 | Biological_process unknown | Nucleus;Cytoplasm | Molecular function unknown | Nucleus;Cytoplasm |
| SEC23A | NP_006355.2 | 10484 | protein transport protein Sec23A | 38.8 | 1 | 17 | 18 | 45 | 2.39E+08 | 1.14 | 14 | 15 | 765 | 86.1 | 7.1 | Transport | Cytoplasm;Endoplasmic reticulum;Cytoplasmic vesicle | Transporter activity | Cytoplasm |
| TBPL1 | NP_004856.1 | 9519 | TATA box-binding protein-like protein 1 | 7.0 | 1 | 1 | 1 | 3 | 2.17E+07 | 1.14 | 1 | - | 186 | 20.9 | 9.5 | Regulation of nucleobase, nucleoside, nucleotide and nucleic acid metabolism | Nucleus;Cytoplasm | Transcription factor activity | Nucleus |
| LAMTOR1 | NP_060377.1 | 55004 | ragulator complex protein LAMTOR1 | 40.4 | 1 | 5 | 5 | 14 | 2.46E+08 | 1.14 | 4 | 12.6 | 161 | 17.7 | 5.2 | Biological_process unknown | - | Molecular function unknown | - |
| STUB1 | NP_005852.2 | 10273 | E3 ubiquitin-protein ligase CHIP | 9.6 | 1 | 2 | 2 | 4 | 3.56E+07 | 1.14 | 3 | 119.1 | 303 | 34.8 | 5.9 | Metabolism;Energy pathways | Cytoplasm;Endoplasmic reticulum;Nucleus | Ligase activity | Cytoplasm |
| TBCD | NP_005984.3 | 6904 | tubulin-specific chaperone D | 20.8 | 1 | 12 | 12 | 18 | 9.57E+07 | 1.14 | 8 | 7.6 | 1192 | 132.5 | 6.2 | Protein metabolism;Protein folding | Microtubule | Chaperone activity | Microtubule |
| NAV2 | NP_001104489.1 | 89797 | neuron navigator 2 isoform 4 | 0.4 | 5 | 1 | 1 | 2 | 1.09E+08 | 1.14 | 1 | - | 1493 | 161.8 | 8.4 | Regulation of nucleobase, nucleoside, nucleotide and nucleic acid metabolism | Nucleus;Cytoplasm | DNA binding | Nucleus |
| NHP2L1 | NP_001003796.1 | 4809 | NHP2-like protein 1 | 46.1 | 1 | 5 | 5 | 21 | 6.14E+08 | 1.14 | 7 | 18.3 | 128 | 14.2 | 8.5 | RNA metabolism | Nucleolus;Chromosome | RNA binding | Nucleolus |
| PPP2R4 | NP_821067.1 | 5524 | serine/threonine-protein phosphatase 2A activator isoform b | 60.7 | 5 | 14 | 14 | 33 | 6.32E+08 | 1.14 | 14 | 30.6 | 323 | 36.8 | 6.3 | Cell communication;Signal transduction;Regulation of cell cycle | Nucleus;Cytoplasm | Phosphatase regulator activity | Cytoplasm |
| UQCRB | NP_006285.1 | 7381 | cytochrome b-c1 complex subunit 7 isoform 1 | 52.3 | 3 | 8 | 8 | 30 | 2.99E+08 | 1.14 | 11 | 31.9 | 111 | 13.5 | 8.8 | Metabolism;Energy pathways | Mitochondrion | Catalytic activity | Mitochondrion |
| HNRNPDL | NP_001193929.1 | 9987 | heterogeneous nuclear ribonucleoprotein D-like isoform b | 47.1 | 2 | 15 | 18 | 108 | 2.19E+09 | 1.14 | 24 | 23.7 | 363 | 40 | 10.0 | Regulation of nucleobase, nucleoside, nucleotide and nucleic acid metabolism | Nucleus;Mitochondrion | RNA binding | Nucleus |
| KPNA1 | NP_002255.3 | 3836 | importin subunit alpha-5 | 31.6 | 1 | 6 | 9 | 25 | 1.33E+08 | 1.14 | 3 | 16.3 | 538 | 60.2 | 5.0 | Transport | Cytoplasm;Nucleus | Transporter activity | Cytoplasm |
| NUP188 | NP_056169.1 | 23511 | nucleoporin NUP188 homolog | 10.6 | 1 | 13 | 13 | 24 | 1.17E+08 | 1.14 | 8 | 19.2 | 1749 | 195.9 | 6.7 | Biological_process unknown | Nuclear membrane | Molecular function unknown | Nuclear membrane |
| UCHL5 | NP_057068.1 | 51377 | ubiquitin carboxyl-terminal hydrolase isozyme L5 isoform 1 | 55.3 | 4 | 11 | 11 | 29 | 3.67E+08 | 1.14 | 8 | 26.9 | 329 | 37.6 | 5.3 | Protein metabolism | Nucleus;Cytoplasm | Ubiquitin-specific protease activity | Nucleus |
| EXOC4 | NP_068579.3 | 60412 | exocyst complex component 4 isoform a | 18.1 | 2 | 12 | 12 | 21 | 2.01E+08 | 1.14 | 8 | 12.4 | 974 | 110.4 | 6.5 | Transport | Plasma membrane;Cytoplasm;Microsome;Zymogen granule | Transporter activity | Plasma membrane |
| SART1 | NP_005137.1 | 9092 | U4/U6.U5 tri-snRNP-associated protein 1 | 37.9 | 1 | 20 | 20 | 53 | 2.66E+08 | 1.14 | 19 | 18.7 | 800 | 90.2 | 6.1 | Biological_process unknown | Nucleus;Cytoplasm | Molecular function unknown | Nucleus |
| ACTR3 | NP_005712.1 | 10096 | actin-related protein 3 isoform 1 | 68.7 | 5 | 22 | 22 | 158 | 4.29E+09 | 1.14 | 41 | 14.7 | 418 | 47.3 | 5.9 | Cell growth and/or maintenance | Cytoplasm;Nucleolus | Structural constituent of cytoskeleton | Cytoplasm |

| Gene Symbol | RefSeq_Accession | Entrez Gene ID | Description | Coverage (%) | Proteins | Unique Peptides | # Peptides | PSM | Area | SCC-R /SCC-S | SCC-R /SCC-S (Count) | SCC-R /SCC-S (Variability [%]) | # AAs | MW [kDa] | calc. pI | Biological Process | Localization | Molecular Function | Primary Localization |
|---|---|---|---|---|---|---|---|---|---|---|---|---|---|---|---|---|---|---|---|
| **BCL2L13** | NP_001257662.1 | 23786 | **bcl-2-like protein 13 isoform g** | 18.1 | 5 | 1 | 1 | 2 | 1.22E+08 | **1.14** | 1 | - | 205 | 21.6 | 4.4 | Apoptosis | Mitochondrion;Cytoplasm | Molecular function unknown | Mitochondrion |
| **KIAA0391** | NP_001243609.1 | 9692 | **mitochondrial ribonuclease P protein 3 isoform 4** | 4.7 | 4 | 1 | 1 | 1 | 5.87E+07 | **1.14** | 1 | - | 211 | 24.8 | 8.7 | Biological_process unknown | Mitochondrion | Molecular function unknown | Mitochondrion |
| **OCIAD1** | NP_060300.1 | 54940 | **OCIA domain-containing protein 1 isoform 1** | 29.4 | 4 | 5 | 5 | 16 | 5.57E+07 | **1.14** | 5 | 16.9 | 245 | 27.6 | 7.5 | Biological_process unknown | Nucleus;Cytoplasm | Molecular function unknown | Nucleus;Cytoplasm |
| **TRMT112** | NP_057488.1 | 51504 | **tRNA methyltransferase 112 homolog isoform 1** | 44.0 | 3 | 4 | 4 | 14 | 2.40E+08 | **1.14** | 7 | 8.3 | 125 | 14.2 | 5.3 | Biological_process unknown | - | Molecular function unknown | - |
| **SRPK2** | NP_001265202.1 | 6733 | **SRSF protein kinase 2 isoform b precursor** | 6.5 | 5 | 2 | 3 | 6 | 7.15E+07 | **1.14** | 2 | 76.6 | 688 | 77.5 | 5.0 | Regulation of gene expression, epigenetic | Nucleus;Cytoplasm | Protein serine/threonine kinase activity | Nucleus |
| **YLPM1** | NP_062535.2 | 56252 | **YLP motif-containing protein 1** | 15.1 | 1 | 20 | 20 | 42 | 4.58E+08 | **1.14** | 17 | 19.3 | 2146 | 241.5 | 6.6 | Biological_process unknown | Nucleus | Molecular function unknown | Nucleus |
| **HN1** | NP_057269.1 | 51155 | **hematological and neurological expressed 1 protein isoform 1** | 64.9 | 3 | 2 | 5 | 26 | 1.09E+09 | **1.14** | 4 | 5.7 | 154 | 16 | 5.6 | Cell communication;Signal transduction | Nucleus;Cytoplasm | Molecular function unknown | Nucleus;Cytoplasm |
| **NCOA5** | NP_066018.1 | 57727 | **nuclear receptor coactivator 5** | 20.2 | 1 | 6 | 6 | 15 | 4.74E+07 | **1.14** | 4 | 95.3 | 579 | 65.5 | 9.6 | Regulation of nucleobase, nucleoside, nucleotide and nucleic acid metabolism | Nucleus | Transcription regulator activity | Nucleus |
| **PDF** | NP_071736.1 | 64146 | **peptide deformylase, mitochondrial precursor** | 9.1 | 1 | 1 | 1 | 1 | 9.76E+07 | **1.14** | 1 | - | 243 | 27 | 9.2 | Metabolism;Energy pathways | Mitochondrion | Hydrolase activity | Mitochondrion |
| **SCAMP3** | NP_005689.2 | 10067 | **secretory carrier-associated membrane protein 3 isoform 1** | 21.6 | 2 | 4 | 4 | 16 | 8.51E+07 | **1.14** | 4 | 12.6 | 347 | 38.3 | 7.6 | Transport | Golgi apparatus;Plasma membrane;Cytoplasm;Nucleus;Early endosome | Auxiliary transport protein activity | Plasma membrane;Nucleus |
| **SFSWAP** | NP_004583.2 | 6433 | **splicing factor, suppressor of white-apricot homolog isoform 2** | 4.4 | 2 | 1 | 1 | 2 | 1.34E+08 | **1.14** | 2 | 6.4 | 951 | 104.8 | 8.1 | Regulation of nucleobase, nucleoside, nucleotide and nucleic acid metabolism | Nucleus | RNA binding | Nucleus |
| **CWC27** | NP_005860.2 | 10283 | **peptidyl-prolyl cis-trans isomerase CWC27 homolog** | 19.5 | 1 | 5 | 5 | 7 | 2.19E+08 | **1.13** | 3 | 16.4 | 472 | 53.8 | 5.8 | Metabolism;Energy pathways | - | Isomerase activity | - |
| **DVL2** | NP_004413.1 | 1856 | **segment polarity protein dishevelled homolog DVL-2** | 14.0 | 1 | 5 | 5 | 8 | 4.40E+07 | **1.13** | 2 | 22.3 | 736 | 78.9 | 6.0 | Cell communication;Signal transduction | Cytoplasm | Receptor signaling complex scaffold activity | Cytoplasm |
| **KTI12** | NP_612426.1 | 112970 | **protein KTI12 homolog** | 35.0 | 1 | 6 | 6 | 8 | 5.85E+07 | **1.13** | 3 | 23.1 | 354 | 38.6 | 7.0 | Biological_process unknown | - | ATP binding | - |
| **NPLOC4** | NP_060391.2 | 55666 | **nuclear protein localization protein 4 homolog** | 27.8 | 1 | 11 | 11 | 28 | 2.74E+08 | **1.13** | 10 | 16.1 | 608 | 68.1 | 6.4 | Transport | Nucleus;Cytoplasm | Transporter activity | Nucleus |
| **GCLC** | NP_001489.1 | 2729 | **glutamate--cysteine ligase catalytic subunit isoform a** | 28.1 | 2 | 9 | 9 | 16 | 2.31E+08 | **1.13** | 6 | 51.2 | 637 | 72.7 | 6.1 | Metabolism;Energy pathways | - | Ligase activity | - |
| **S100A11** | NP_005611.1 | 6282 | **protein S100-A11** | 86.7 | 1 | 13 | 13 | 179 | 5.38E+09 | **1.13** | 22 | 19.2 | 105 | 11.7 | 7.1 | Cell communication;Signal transduction | Nucleus;Cytoplasm;Plasma membrane;Endosome | Calcium ion binding | Cytoplasm |
| **NPEPPS** | NP_006301.3 | 9520 | **puromycin-sensitive aminopeptidase** | 32.6 | 7 | 21 | 21 | 56 | 3.86E+08 | **1.13** | 24 | 19.9 | 919 | 103.2 | 5.7 | Protein metabolism | Cytoplasm;Golgi apparatus;Nucleus;Mitochondrion;Endosome | Aminopeptidase activity | Cytoplasm |
| **NSUN7** | NP_078953.3 | 79730 | **putative methyltransferase NSUN7** | 1.0 | 1 | 1 | 1 | 1 | 7.25E+08 | **1.13** | 1 | - | 718 | 81 | 8.6 | Biological_process unknown | - | Molecular function unknown | - |
| **PPP1CA** | NP_002699.1 | 5499 | **serine/threonine-protein phosphatase PP1-alpha catalytic subunit isoform 1** | 60.9 | 3 | 5 | 18 | 73 | 1.67E+09 | **1.13** | 12 | 11.2 | 330 | 37.5 | 6.3 | Cell proliferation | Cytoskeleton;Nucleolus;Microtubule;Mitochondrion;Cytoplasm | Protein serine/threonine phosphatase activity | Cytoskeleton |
| **TPP2** | NP_003282.2 | 7174 | **tripeptidyl-peptidase 2** | 24.8 | 1 | 22 | 22 | 33 | 2.62E+08 | **1.13** | 14 | 22.8 | 1249 | 138.3 | 6.3 | Protein metabolism | Cytoplasm;Plasma membrane | Aminopeptidase activity | Cytoplasm |
| **UBE2D2** | NP_862821.1 | 7322 | **ubiquitin-conjugating enzyme E2 D2 isoform 2** | 35.6 | 8 | 2 | 2 | 10 | 1.45E+08 | **1.13** | 4 | 30.4 | 118 | 13.6 | 8.3 | Protein metabolism | Cytoplasm | Ubiquitin-specific protease activity | Cytoplasm |
| **ANP32B** | NP_006392.1 | 10541 | **acidic leucine-rich nuclear phosphoprotein 32 family member B** | 32.3 | 1 | 7 | 9 | 97 | 1.36E+09 | **1.13** | 25 | 14.3 | 251 | 28.8 | 4.1 | Biological_process unknown | Nucleus;Nucleolus;Cytoplasm | Molecular function unknown | Nucleus |
| **ARMC6** | NP_219483.1 | 93436 | **armadillo repeat-containing protein 6 isoform 2** | 12.6 | 2 | 2 | 2 | 3 | 9.01E+07 | **1.13** | 2 | 1.1 | 476 | 51.5 | 6.1 | Signal transduction | - | Molecular function unknown | - |
| **NDUFA12** | NP_061326.1 | 55967 | **NADH dehydrogenase [ubiquinone] 1 alpha subcomplex subunit 12 isoform a** | 50.3 | 2 | 6 | 6 | 15 | 1.68E+08 | **1.13** | 7 | 13.6 | 145 | 17.1 | 9.6 | Metabolism;Energy pathways | Mitochondrion | Oxidoreductase activity | Mitochondrion |
| **ADRM1** | NP_783163.1 | 11047 | **proteasomal ubiquitin receptor ADRM1 isoform 1** | 8.9 | 2 | 3 | 3 | 6 | 2.97E+08 | **1.13** | 3 | 2 | 407 | 42.1 | 5.1 | Cell communication;Signal transduction | Nucleus;Cytoplasm;Plasma membrane;Extracellular;Microsome | Receptor activity;Molecular function unknown | Cytoplasm;Microsome |
| **WDR12** | NP_060726.3 | 55759 | **ribosome biogenesis protein WDR12** | 16.8 | 1 | 5 | 5 | 9 | 1.56E+08 | **1.13** | 4 | 21.5 | 423 | 47.7 | 5.9 | Biological_process unknown | Nucleolus | Molecular function unknown | Nucleolus |
| **MRPL39** | NP_059142.2 | 54148 | **39S ribosomal protein L39, mitochondrial isoform a** | 11.2 | 2 | 3 | 3 | 4 | 1.92E+08 | **1.13** | 2 | 8.7 | 338 | 38.7 | 7.7 | Biological_process unknown | Mitochondrion | Structural constituent of ribosome;RNA binding | Mitochondrion |
| **NDUFS2** | NP_001159631.1 | 4720 | **NADH dehydrogenase [ubiquinone] iron-sulfur protein 2, mitochondrial isoform 2 precursor** | 18.2 | 2 | 6 | 6 | 14 | 5.39E+08 | **1.13** | 6 | 10.8 | 457 | 51.8 | 8.2 | Metabolism;Energy pathways | Mitochondrion | Oxidoreductase activity | Mitochondrion |
| **NNT** | NP_892022.2 | 23530 | **NAD(P) transhydrogenase, mitochondrial** | 19.2 | 1 | 13 | 13 | 26 | 3.26E+08 | **1.13** | 10 | 37.2 | 1086 | 113.8 | 8.1 | Energy pathways | Mitochondrial membrane;Mitochondrion | Oxidoreductase activity | Mitochondrial membrane |
| **ZC3HC1** | NP_057562.3 | 51530 | **nuclear-interacting partner of ALK isoform 1** | 10.8 | 3 | 2 | 3 | 5 | 4.44E+07 | **1.13** | 2 | 0.3 | 502 | 55.2 | 5.6 | Biological_process unknown | Nucleus | Molecular function unknown | Nucleus |
| **CISD2** | NP_001008389.1 | 493856 | **CDGSH iron-sulfur domain-containing protein 2** | 21.5 | 1 | 2 | 2 | 3 | 1.15E+08 | **1.13** | 2 | 12.8 | 135 | 15.3 | 9.6 | Biological_process unknown | Integral to membrane | Molecular function unknown | Integral to membrane |
| **EXOC8** | NP_787072.2 | 149371 | **exocyst complex component 8** | 12.1 | 1 | 6 | 6 | 9 | 1.20E+08 | **1.13** | 3 | 27.6 | 725 | 81.7 | 5.5 | Transport | Plasma membrane;Cytoplasm;Nucleus | Transporter activity | Plasma membrane |
| **NUDT5** | NP_054861.2 | 11164 | **ADP-sugar pyrophosphatase** | 51.6 | 1 | 9 | 9 | 35 | 4.09E+08 | **1.13** | 13 | 31.2 | 219 | 24.3 | 4.9 | Metabolism;Energy pathways | - | Hydrolase activity | - |
| **PSMD4** | NP_002801.1 | 5710 | **26S proteasome non-ATPase regulatory subunit 4** | 38.7 | 1 | 9 | 9 | 27 | 4.35E+08 | **1.13** | 11 | 15.3 | 377 | 40.7 | 4.8 | Protein metabolism | Cytoplasm | Ubiquitin-specific protease activity | Cytoplasm |
| **HSPA9** | NP_004125.3 | 3313 | **stress-70 protein, mitochondrial precursor** | 52.7 | 1 | 32 | 32 | 287 | 3.28E+09 | **1.13** | 65 | 12.5 | 679 | 73.6 | 6.2 | Protein metabolism | Cytoplasm;Mitochondrion;Endoplasmic reticulum;Plasma membrane;Cytoplasmic vesicle;Nucleolus;Cell surface | Chaperone activity | Mitochondrion |
| **MED11** | NP_001001683.1 | 400569 | **mediator of RNA polymerase II transcription subunit 11** | 39.3 | 1 | 3 | 3 | 4 | 2.63E+07 | **1.13** | 2 | 6.8 | 117 | 13.1 | 6.0 | Biological_process unknown | - | Molecular function unknown | - |

| Gene Symbol | RefSeq_Accession | Entrez Gene ID | Description | Coverage (%) | Proteins | Unique Peptides | # Peptides | PSM | Area | SCC-R /SCC-S | SCC-R /SCC-S (Count) | SCC-R /SCC-S (Variability [%]) | # AAs | MW [kDa] | calc. pI | Biological Process | Localization | Molecular Function | Primary Localization |
|---|---|---|---|---|---|---|---|---|---|---|---|---|---|---|---|---|---|---|---|
| **TPD52L2** | NP_003279.2 | 7165 | **tumor protein D54 isoform e** | 79.1 | 6 | 2 | 12 | 73 | 1.25E+09 | **1.13** | 1 | - | 206 | 22.2 | 5.4 | Cell growth and/or maintenance;Biological_process unknown | Cytoplasm;Nucleus | Molecular function unknown | Cytoplasm;Nucleus |
| **TPM2** | NP_998839.1 | 7169 | **tropomyosin beta chain isoform 2** | 61.3 | 1 | 9 | 24 | 224 | 8.07E+09 | **1.13** | 8 | 9 | 284 | 33 | 4.7 | Cell growth and/or maintenance | Cytoskeleton;Extracellular | Structural constituent of cytoskeleton | Cytoskeleton |
| **UQCR10** | NP_037519.2 | 29796 | **cytochrome b-c1 complex subunit 9 isoform a** | 27.0 | 1 | 1 | 1 | 6 | 5.78E+08 | **1.13** | 2 | 17.6 | 63 | 7.3 | 9.5 | Electron transport | Mitochondrial membrane | Oxidoreductase activity | Mitochondrial membrane |
| **ATIC** | NP_004035.2 | 471 | **bifunctional purine biosynthesis protein PURH** | 78.7 | 1 | 35 | 35 | 191 | 4.39E+09 | **1.13** | 55 | 18.9 | 592 | 64.6 | 6.7 | Metabolism;Energy pathways | - | Hydrolase activity | - |
| **CANX** | NP_001737.1 | 821 | **calnexin precursor** | 50.8 | 1 | 25 | 25 | 167 | 3.63E+09 | **1.13** | 45 | 25.6 | 592 | 67.5 | 4.6 | Protein folding | Endoplasmic reticulum;Nuclear membrane;Endoplasmic reticulum membrane;Plasma membrane;Golgi apparatus;Lysosome;Mitochondrion | Chaperone activity | Endoplasmic reticulum membrane |
| **PSME3** | NP_005780.2 | 10197 | **proteasome activator complex subunit 3 isoform 1** | 54.7 | 3 | 8 | 8 | 18 | 2.86E+08 | **1.13** | 5 | 17.7 | 254 | 29.5 | 6.0 | Protein metabolism | Nucleus;Cytoplasm | Ubiquitin-specific protease activity | Cytoplasm |
| **COX2** | YP_003024029.1 | 4513 | **cytochrome c oxidase subunit II** | 48.0 | 1 | 7 | 7 | 28 | 6.63E+08 | **1.12** | 10 | 35.5 | 227 | 25.5 | 4.8 | Metabolism;Energy pathways | Mitochondrion | Oxidoreductase activity | Mitochondrion |
| **NDUFS1** | NP_001186912.1 | 4719 | **NADH-ubiquinone oxidoreductase 75 kDa subunit, mitochondrial isoform 4** | 50.5 | 5 | 18 | 18 | 39 | 5.28E+08 | **1.12** | 15 | 15.5 | 670 | 73.5 | 5.9 | Metabolism;Energy pathways | Mitochondrion | Oxidoreductase activity | Mitochondrion |
| **PSMB7** | NP_002790.1 | 5695 | **proteasome subunit beta type-7 proprotein** | 46.2 | 1 | 6 | 6 | 16 | 7.79E+08 | **1.12** | 6 | 30.7 | 277 | 29.9 | 7.7 | Protein metabolism | Cytoplasm;Nucleus | Ubiquitin-specific protease activity | Cytoplasm |
| **DNAJA1** | NP_001530.1 | 3301 | **dnaJ homolog subfamily A member 1** | 71.5 | 1 | 19 | 19 | 107 | 1.35E+09 | **1.12** | 43 | 22.8 | 397 | 44.8 | 7.1 | Protein metabolism | Nucleus;Nucleolus;Golgi apparatus;Acrosome | Heat shock protein activity | Acrosome |
| **WDR26** | NP_001108585.2 | 80232 | **WD repeat-containing protein 26 isoform b** | 14.6 | 2 | 5 | 5 | 9 | 1.73E+08 | **1.12** | 4 | 31.3 | 645 | 70.4 | 6.2 | Biological_process unknown | - | Molecular function unknown | - |
| **GTF2F1** | NP_002087.2 | 2962 | **general transcription factor IIF subunit 1** | 35.2 | 1 | 17 | 17 | 31 | 2.55E+08 | **1.12** | 16 | 31.8 | 517 | 58.2 | 7.5 | Regulation of nucleobase, nucleoside, nucleotide and nucleic acid metabolism | Nucleus;Nucleolus;Kinetochore | Transcription factor activity | Nucleus |
| **ING1** | NP_937861.1 | 3621 | **inhibitor of growth protein 1 isoform B** | 21.0 | 5 | 2 | 2 | 3 | 1.01E+07 | **1.12** | 1 | - | 210 | 23.7 | 8.4 | Regulation of cell growth | Nucleus | Protein binding | Nucleus |
| **CDC42** | NP_001034891.1 | 998 | **cell division control protein 42 homolog isoform 1 precursor** | 60.2 | 4 | 8 | 9 | 86 | 1.22E+09 | **1.12** | 23 | 20.3 | 191 | 21.2 | 6.6 | Cell communication;Signal transduction | Cell junction;Cytoplasm;Plasma membrane;Extracellular;Microtubule cytoskeleton;Apical membrane;Cytosol;Cell projection | GTPase activity | Cytoplasm |
| **TIMM23** | NP_006318.1 | 100287932 | **mitochondrial import inner membrane translocase subunit Tim23** | 28.7 | 3 | 4 | 4 | 6 | 7.36E+07 | **1.12** | 1 | - | 209 | 21.9 | 8.6 | - | - | - | - |
| **ZC3H18** | NP_653205.3 | 124245 | **zinc finger CCCH domain-containing protein 18** | 12.8 | 1 | 7 | 7 | 15 | 1.03E+08 | **1.12** | 7 | 65.7 | 953 | 106.3 | 8.3 | Biological_process unknown | - | Molecular function unknown | - |
| **EPS15L1** | NP_001245304.1 | 58513 | **epidermal growth factor receptor substrate 15-like 1 isoform 3** | 18.8 | 4 | 8 | 8 | 13 | 2.11E+08 | **1.12** | 7 | 27.2 | 754 | 83.2 | 5.1 | Cell communication;Signal transduction | Nucleus;Cytoplasm;Plasma membrane | Calcium ion binding | Nucleus |
| **LARS** | NP_064502.9 | 51520 | **leucine--tRNA ligase, cytoplasmic** | 43.2 | 1 | 35 | 35 | 86 | 3.87E+08 | **1.12** | 28 | 20.7 | 1176 | 134.4 | 7.3 | Metabolism;Energy pathways | Mitochondrion;Cytosol | Ligase activity | Mitochondrion |
| **EPB41** | NP_004428.1 | 2035 | **protein 4.1 isoform 6** | 6.0 | 6 | 2 | 2 | 2 | 1.95E+07 | **1.12** | 2 | 47.9 | 588 | 66.4 | 7.3 | Cell growth and/or maintenance | Plasma membrane;Nucleus;Cytoplasm;Centrosome;Cytoskeleton | Structural molecule activity | Nucleus |
| **MBNL2** | NP_997187.1 | 10150 | **muscleblind-like protein 2 isoform 3** | 15.2 | 2 | 1 | 3 | 7 | 1.16E+08 | **1.12** | 1 | - | 361 | 39.3 | 8.4 | Regulation of nucleobase, nucleoside, nucleotide and nucleic acid metabolism | Nucleus;Cytoplasm | RNA binding | Nucleus |
| **SSRP1** | NP_003137.1 | 6749 | **FACT complex subunit SSRP1** | 36.4 | 1 | 22 | 22 | 89 | 9.44E+08 | **1.12** | 26 | 20.5 | 709 | 81 | 6.9 | Regulation of nucleobase, nucleoside, nucleotide and nucleic acid metabolism | Cytoplasm;Nucleus;Nucleolus;Chromosome | Transcription factor activity | Nucleus |
| **TAF10** | NP_006275.1 | 6881 | **transcription initiation factor TFIID subunit 10** | 24.8 | 1 | 4 | 4 | 6 | 2.74E+07 | **1.12** | 3 | 21.3 | 218 | 21.7 | 6.6 | Regulation of nucleobase, nucleoside, nucleotide and nucleic acid metabolism | Nucleus | Transcription regulator activity | Nucleus |
| **DARS** | NP_001340.2 | 1615 | **aspartate--tRNA ligase, cytoplasmic** | 38.1 | 1 | 16 | 16 | 58 | 1.20E+09 | **1.12** | 15 | 23.1 | 501 | 57.1 | 6.6 | Metabolism;Energy pathways | Cytoplasm;Cytosol | ATPase activity | Cytoplasm |
| **MAP4** | NP_002366.2 | 4134 | **microtubule-associated protein 4 isoform 1** | 54.3 | 3 | 39 | 39 | 110 | 1.24E+09 | **1.12** | 40 | 26.8 | 1152 | 120.9 | 5.4 | Cell growth and/or maintenance | Cytoplasm;Microtubule;Nucleus | Cytoskeletal protein binding | Cytoplasm;Nucleus |
| **NUP43** | NP_942590.1 | 348995 | **nucleoporin Nup43** | 25.8 | 1 | 5 | 5 | 6 | 1.96E+08 | **1.12** | 4 | 2.5 | 380 | 42.1 | 5.6 | Transport | Nucleus;Nuclear membrane | Transporter activity | Nucleus |
| **SRSF10** | NP_001177938.1 | 10772 | **serine/arginine-rich splicing factor 10 isoform 6** | 45.5 | 7 | 6 | 6 | 13 | 4.33E+08 | **1.12** | 4 | 19.5 | 165 | 20.1 | 10.1 | RNA metabolism | Nucleolus;Nucleus;Cytoplasm;Nucleoplasm | RNA binding | Nucleolus;Nucleus;Cytoplasm |
| **SOAT1** | NP_003092.4 | 6646 | **sterol O-acyltransferase 1 isoform 1** | 10.0 | 3 | 3 | 3 | 7 | 6.41E+07 | **1.12** | 3 | 11.2 | 550 | 64.7 | 8.9 | Metabolism;Energy pathways | Endoplasmic reticulum;Cytoplasm;Nucleus | Acyltransferase activity | Endoplasmic reticulum |
| **SPINT2** | NP_001159575.1 | 10653 | **kunitz-type protease inhibitor 2 isoform b precursor** | 13.3 | 2 | 2 | 2 | 2 | 5.86E+07 | **1.12** | 1 | - | 195 | 21.8 | 8.4 | Protein metabolism | Plasma membrane;Extracellular;Lysosome | Protease inhibitor activity | Plasma membrane |
| **DNAJC10** | NP_001258510.1 | 54431 | **dnaJ homolog subfamily C member 10 isoform 2 precursor** | 8.7 | 2 | 4 | 4 | 6 | 3.23E+07 | **1.12** | 3 | 19.6 | 747 | 86.1 | 7.4 | Protein metabolism | Endoplasmic reticulum | Molecular function unknown | Endoplasmic reticulum |
| **FAHD1** | NP_112485.1 | 81889 | **acylpyruvase FAHD1, mitochondrial isoform 2** | 42.9 | 3 | 5 | 5 | 23 | 3.51E+08 | **1.12** | 6 | 98.8 | 224 | 24.8 | 7.4 | Biological_process unknown | - | Molecular function unknown | - |
| **NDUFA7** | NP_004992.2 | 4701 | **NADH dehydrogenase [ubiquinone] 1 alpha subcomplex subunit 7** | 25.7 | 1 | 2 | 2 | 2 | 1.41E+08 | **1.12** | 1 | - | 113 | 12.5 | 10.2 | Metabolism;Energy pathways | Mitochondrion | Molecular function unknown | Mitochondrion |
| **PRKAA1** | NP_006242.5 | 5562 | **5'-AMP-activated protein kinase catalytic subunit alpha-1 isoform 1** | 14.5 | 3 | 6 | 6 | 16 | 1.30E+08 | **1.12** | 5 | 14.3 | 559 | 64 | 8.1 | Signal transduction | Cytoplasm | Protein serine/threonine kinase activity | Cytoplasm |
| **RANBP2** | NP_006258.3 | 5903 | **E3 SUMO-protein ligase RanBP2** | 24.9 | 8 | 45 | 45 | 82 | 5.77E+08 | **1.12** | 35 | 35.1 | 3224 | 358 | 6.2 | Cell communication;Signal transduction | Nucleus;Cytoplasm;Mitochondrion;Kinetochore | Transporter activity | Nucleus |
| **RBX1** | NP_055063.1 | 9978 | **E3 ubiquitin-protein ligase RBX1** | 17.6 | 1 | 2 | 2 | 14 | 6.12E+08 | **1.11** | 1 | - | 108 | 12.3 | 7.0 | Protein metabolism | Nucleus;Cytoplasm;Cytosol | Ubiquitin-specific protease activity | Nucleus |
| **WDR44** | NP_001171894.1 | 54521 | **WD repeat-containing protein 44 isoform 2** | 15.1 | 3 | 7 | 7 | 12 | 1.45E+08 | **1.11** | 6 | 19.4 | 905 | 100.3 | 5.5 | Transport | Cytoplasm;Cytoskeleton | Transporter activity | Cytoplasm |
| **ATP6V1F** | NP_004222.2 | 9296 | **V-type proton ATPase subunit F isoform 1** | 46.2 | 2 | 4 | 4 | 6 | 3.09E+07 | **1.11** | 2 | 3 | 119 | 13.4 | 5.5 | Metabolism;Energy pathways | Plasma membrane;Mitochondrion | ATPase activity | Plasma membrane |
| **COX15** | NP_004367.2 | 1355 | **cytochrome c oxidase assembly protein COX15 homolog isoform 2** | 4.4 | 2 | 1 | 1 | 2 | 4.55E+07 | **1.11** | 1 | - | 388 | 43.8 | 9.6 | Metabolism;Energy pathways | Mitochondrion | Molecular function unknown | Mitochondrion |

| Gene Symbol | RefSeq_Accession | Entrez Gene ID | Description | Coverage (%) | Proteins | Unique Peptides | # Peptides | PSM | Area | SCC-R /SCC-S | SCC-R /SCC-S (Count) | SCC-R /SCC-S (Variability [%]) | # AAs | MW [kDa] | calc. pI | Biological Process | Localization | Molecular Function | Primary Localization |
|---|---|---|---|---|---|---|---|---|---|---|---|---|---|---|---|---|---|---|---|
| SRSF2 | NP_003007.2 | 6427 | serine/arginine-rich splicing factor 2 | 21.7 | 1 | 3 | 4 | 25 | 1.87E+09 | 1.11 | 8 | 18.8 | 221 | 25.5 | 11.9 | Regulation of nucleobase, nucleoside, nucleotide and nucleic acid metabolism | Nucleus;Nucleolus | Ribonucleoprotein | Nucleus |
| MTMR4 | NP_004678.3 | 9110 | myotubularin-related protein 4 | 0.6 | 1 | 1 | 1 | 1 | 9.67E+05 | 1.11 | 1 | - | 1195 | 133.3 | 6.0 | Cell communication;Signal transduction | Cytoplasm;Nucleus | Protein tyrosine/serine/threonine phosphatase activity | Cytoplasm |
| ADAM17 | NP_003174.3 | 6868 | disintegrin and metalloproteinase domain-containing protein 17 preproprotein | 1.8 | 1 | 1 | 1 | 1 | 1.75E+07 | 1.11 | 1 | - | 824 | 93 | 5.8 | Protein metabolism | Plasma membrane;Cell surface;Nucleus | Metallopeptidase activity | Plasma membrane |
| GOLM1 | NP_057632.2 | 51280 | Golgi membrane protein 1 | 9.2 | 1 | 3 | 3 | 3 | 2.70E+07 | 1.11 | 2 | 21.9 | 401 | 45.3 | 5.0 | Transport | Golgi apparatus | Transporter activity | Golgi apparatus |
| PRMT5 | NP_006100.2 | 10419 | protein arginine N-methyltransferase 5 isoform a | 40.5 | 6 | 19 | 19 | 70 | 5.08E+08 | 1.11 | 21 | 11.6 | 637 | 72.6 | 6.3 | Cell communication;Signal transduction | Cytoplasm | Methyltransferase activity | Cytoplasm |
| USMG5 | NP_001193356.1 | 84833 | up-regulated during skeletal muscle growth protein 5 | 27.6 | 1 | 2 | 2 | 6 | 3.57E+08 | 1.11 | 1 | - | 58 | 6.5 | 9.8 | Biological_process unknown | Integral to membrane | Molecular function unknown | Integral to membrane |
| AAAS | NP_056480.1 | 8086 | aladin isoform 1 | 8.8 | 2 | 4 | 4 | 11 | 1.05E+09 | 1.11 | 5 | 9.4 | 546 | 59.5 | 7.5 | Cell communication;Signal transduction | Peroxisome;Nuclear membrane | Molecular function unknown | Peroxisome |
| LONP1 | NP_001263409.1 | 9361 | lon protease homolog, mitochondrial isoform 3 | 43.8 | 3 | 26 | 26 | 68 | 6.89E+08 | 1.11 | 19 | 23 | 763 | 85.6 | 6.0 | Protein metabolism | Mitochondrion;Endoplasmic reticulum | Serine-type peptidase activity | Mitochondrion |
| MARCKS | NP_002347.5 | 4082 | myristoylated alanine-rich C-kinase substrate | 67.2 | 1 | 12 | 12 | 37 | 6.03E+08 | 1.11 | 14 | 31.4 | 332 | 31.5 | 4.5 | Cell growth and/or maintenance | Plasma membrane;Cytoplasm | Structural constituent of cytoskeleton | Plasma membrane |
| MRPS18C | NP_057151.1 | 51023 | 28S ribosomal protein S18c, mitochondrial | 13.4 | 1 | 1 | 1 | 3 | 2.57E+07 | 1.11 | 2 | 0 | 142 | 15.8 | 9.6 | Protein metabolism | Mitochondrion | Structural constituent of ribosome | Mitochondrion |
| PPP6C | NP_002712.1 | 5537 | serine/threonine-protein phosphatase 6 catalytic subunit isoform b | 28.9 | 3 | 5 | 5 | 10 | 1.78E+08 | 1.11 | 3 | 4.1 | 305 | 35.1 | 5.7 | Cell communication;Signal transduction | Nucleus;Cytoplasm | Protein serine/threonine phosphatase activity | Nucleus |
| AHCYL1 | NP_006612.2 | 10768 | putative adenosylhomocysteinase 2 isoform a | 27.0 | 6 | 10 | 10 | 18 | 2.22E+08 | 1.11 | 7 | 6 | 530 | 58.9 | 6.9 | Metabolism;Energy pathways | Cytoplasm;Endoplasmic reticulum;Plasma membrane | Hydrolase activity | Cytoplasm |
| PAFAH1B2 | NP_002563.1 | 5049 | platelet-activating factor acetylhydrolase IB subunit beta isoform a | 65.9 | 4 | 7 | 7 | 34 | 8.54E+08 | 1.11 | 11 | 25.4 | 229 | 25.6 | 5.9 | Cell communication;Signal transduction | Cytoplasm;Extracellular | Hydrolase activity | Cytoplasm |
| SF3B3 | NP_036558.3 | 23450 | splicing factor 3B subunit 3 | 35.9 | 1 | 33 | 33 | 101 | 5.84E+08 | 1.11 | 36 | 28.7 | 1217 | 135.5 | 5.3 | Regulation of nucleobase, nucleoside, nucleotide and nucleic acid metabolism | Nucleus;Cytoplasm | Ribonucleoprotein | Nucleus |
| ADD1 | NP_054909.2 | 118 | alpha-adducin isoform c | 20.6 | 5 | 6 | 6 | 15 | 8.84E+07 | 1.11 | 4 | 5.6 | 631 | 69.9 | 6.5 | Cell growth and/or maintenance | Nucleus;Mitochondrion;Cytoplasm | Structural molecule activity | Nucleus;Cytoplasm |
| COG8 | NP_115758.3 | 84342 | conserved oligomeric Golgi complex subunit 8 | 2.8 | 1 | 1 | 1 | 2 | 1.42E+08 | 1.11 | 1 | - | 612 | 68.4 | 5.2 | Transport | Golgi apparatus;Cytoplasm | Transporter activity | Golgi apparatus |
| KIFAP3 | NP_001191443.1 | 22920 | kinesin-associated protein 3 isoform 2 | 2.8 | 4 | 1 | 1 | 1 | 7.15E+06 | 1.11 | 1 | - | 714 | 82.1 | 4.9 | Transport | Nucleus;Cytoplasm;Endoplasmic reticulum | Transporter activity | Cytoplasm |
| LMAN1 | NP_005561.1 | 3998 | protein ERGIC-53 precursor | 23.7 | 1 | 6 | 6 | 9 | 1.29E+08 | 1.11 | 3 | 0.9 | 510 | 57.5 | 6.8 | Protein metabolism | Endoplasmic reticulum;Golgi apparatus | Chaperone activity | Endoplasmic reticulum |
| MRPS28 | NP_054737.1 | 28957 | 28S ribosomal protein S28, mitochondrial | 31.0 | 1 | 5 | 5 | 8 | 1.13E+08 | 1.11 | 3 | 47.7 | 187 | 20.8 | 9.1 | Protein metabolism | Ribosome;Mitochondrion | Structural constituent of ribosome | Ribosome |
| YEATS4 | NP_006521.1 | 8089 | YEATS domain-containing protein 4 | 11.9 | 1 | 1 | 1 | 1 | 1.85E+07 | 1.11 | 1 | - | 227 | 26.5 | 8.4 | Regulation of nucleobase, nucleoside, nucleotide and nucleic acid metabolism | Nucleus;Cytoplasm;Nucleolus | Transcription factor activity | Nucleus |
| C21orf33 | NP_004640.3 | 8209 | ES1 protein homolog, mitochondrial isoform Ia precursor | 48.9 | 14 | 10 | 10 | 40 | 3.44E+08 | 1.10 | 12 | 16.5 | 268 | 28.1 | 8.3 | Biological_process unknown | Mitochondrion | Molecular function unknown | Mitochondrion |
| SNRNP70 | NP_003080.2 | 6625 | U1 small nuclear ribonucleoprotein 70 kDa | 31.8 | 1 | 14 | 14 | 37 | 3.42E+08 | 1.10 | 11 | 17.1 | 437 | 51.5 | 9.9 | Regulation of nucleobase, nucleoside, nucleotide and nucleic acid metabolism | Nucleus;Cytoplasm | Ribonucleoprotein;RNA binding | Nucleus |
| TTI1 | NP_055472.1 | 9675 | TELO2-interacting protein 1 homolog | 3.0 | 1 | 2 | 2 | 3 | 3.09E+07 | 1.10 | 2 | 13 | 1089 | 122 | 6.0 | Biological_process unknown | Cytoplasm | Molecular function unknown | Cytoplasm |
| FAM21A | NP_001005751.1 | 387680 | WASH complex subunit FAM21A isoform 1 | 23.9 | 5 | 16 | 16 | 28 | 1.28E+08 | 1.10 | 12 | 24.1 | 1341 | 147.1 | 4.8 | Biological_process unknown | - | Molecular function unknown | - |
| RANBP3 | NP_015559.2 | 8498 | ran-binding protein 3 isoform RANBP3-b | 26.5 | 3 | 8 | 8 | 20 | 3.40E+08 | 1.10 | 8 | 15.2 | 499 | 53.4 | 4.8 | Cell communication;Signal transduction | Nucleus;Cytoplasm | Transporter activity | Nucleus |
| KHDRBS1 | NP_006550.1 | 10657 | KH domain-containing, RNA-binding, signal transduction-associated protein 1 isoform 1 | 30.7 | 4 | 11 | 11 | 66 | 1.22E+09 | 1.10 | 24 | 8.7 | 443 | 48.2 | 8.7 | Regulation of nucleobase, nucleoside, nucleotide and nucleic acid metabolism | Nucleus;Cytoplasm;Nucleolus | RNA binding | Nucleus |
| NEDD1 | NP_001128649.1 | 121441 | protein NEDD1 isoform c | 13.7 | 3 | 4 | 4 | 7 | 3.01E+07 | 1.10 | 2 | 32 | 571 | 62.3 | 8.3 | Cell communication;Signal transduction | Centrosome;Microtubule | Molecular function unknown | - |
| PSMG3 | NP_115678.1 | 84262 | proteasome assembly chaperone 3 | 32.0 | 1 | 2 | 2 | 4 | 8.81E+07 | 1.10 | 2 | 68 | 122 | 13.1 | 7.9 | Biological_process unknown | - | Molecular function unknown | - |
| IKBKG | NP_003630.1 | 8517 | NF-kappa-B essential modulator isoform a | 25.1 | 3 | 7 | 7 | 10 | 1.50E+08 | 1.10 | 4 | 23.3 | 419 | 48.2 | 5.7 | Cell communication;Signal transduction | Cytoplasm;Nucleus;Plasma membrane;Cytosol | Receptor signaling complex scaffold activity | Cytoplasm |
| RHOA | NP_001655.1 | 387 | transforming protein RhoA precursor | 74.6 | 1 | 7 | 13 | 67 | 1.44E+09 | 1.10 | 12 | 6 | 193 | 21.8 | 6.1 | Cell communication;Signal transduction | Actin cytoskeleton;Cytoplasm;Nucleus;Plasma membrane | GTPase activity | Cytoplasm |
| TRMT11 | NP_001026882.2 | 60487 | tRNA (guanine(10)-N2)-methyltransferase homolog | 6.5 | 1 | 1 | 1 | 3 | 6.96E+07 | 1.10 | 1 | - | 463 | 53.4 | 7.8 | Biological_process unknown | - | Molecular function unknown | - |
| CNPY2 | NP_055070.1 | 10330 | protein canopy homolog 2 isoform 1 precursor | 54.4 | 2 | 9 | 9 | 36 | 5.69E+08 | 1.10 | 10 | 17.4 | 182 | 20.6 | 4.9 | Cell growth and/or maintenance | Integral to membrane;Cytoplasm | Protein binding | Integral to membrane |
| DNM2 | NP_001177645.1 | 1785 | dynamin-2 isoform 5 | 37.1 | 5 | 19 | 25 | 56 | 3.30E+08 | 1.10 | 16 | 16.4 | 869 | 97.9 | 7.4 | Cell communication;Signal transduction | Cytoskeleton;Perinuclear region;Cell surface;Mitochondrion | GTPase activity | Cytoskeleton |
| ELOF1 | NP_115753.1 | 84337 | transcription elongation factor 1 homolog | 21.7 | 1 | 1 | 1 | 6 | 5.99E+08 | 1.10 | 2 | 1 | 83 | 9.5 | 8.0 | Biological_process unknown | - | Molecular function unknown | - |
| MINPP1 | NP_001171588.1 | 9562 | multiple inositol polyphosphate phosphatase 1 isoform 2 precursor | 12.2 | 2 | 1 | 1 | 1 | 2.78E+07 | 1.10 | 1 | - | 312 | 34.6 | 9.0 | Cell communication;Signal transduction | Endoplasmic reticulum;Cytoplasm | Lipid phosphatase activity | Endoplasmic reticulum |
| RNPS1 | NP_001273555.1 | 10921 | RNA-binding protein with serine-rich domain 1 isoform b | 24.5 | 3 | 5 | 5 | 14 | 2.32E+08 | 1.10 | 3 | 5.8 | 282 | 31.7 | 11.9 | Regulation of nucleobase, nucleoside, nucleotide and nucleic acid metabolism | Nucleus | RNA binding | Nucleus |
| SAAL1 | NP_612430.2 | 113174 | protein SAAL1 | 6.5 | 1 | 2 | 2 | 4 | 7.01E+07 | 1.10 | 2 | 11 | 474 | 53.5 | 4.5 | Biological_process unknown | - | Molecular function unknown | - |

| Gene Symbol | RefSeq_Accession | Entrez Gene ID | Description | Coverage (%) | Proteins | Unique Peptides | # Peptides | PSM | Area | SCC-R /SCC-S | SCC-R /SCC-S (Count) | SCC-R /SCC-S (Variability [%]) | # AAs | MW [kDa] | calc. pI | Biological Process | Localization | Molecular Function | Primary Localization |
|---|---|---|---|---|---|---|---|---|---|---|---|---|---|---|---|---|---|---|---|
| **ZDHHC5** | NP_056272.2 | 25921 | **palmitoyltransferase ZDHHC5** | 11.5 | 1 | 4 | 4 | 6 | 1.54E+07 | **1.10** | 1 | - | 715 | 77.5 | 9.0 | Biological_process unknown | Integral to membrane | Molecular function unknown | Integral to membrane |
| **EPHX1** | NP_000111.1 | 2052 | **epoxide hydrolase 1** | 15.6 | 1 | 3 | 3 | 7 | 3.33E+07 | **1.10** | 1 | - | 455 | 52.9 | 7.3 | Metabolism;Energy pathways | Endoplasmic reticulum;Nucleus;Endosome | Hydrolase activity | Endoplasmic reticulum |
| **SF3A2** | NP_009096.2 | 8175 | **splicing factor 3A subunit 2** | 16.2 | 1 | 5 | 5 | 17 | 3.13E+08 | **1.10** | 5 | 12 | 464 | 49.2 | 9.6 | Protein metabolism | Nucleus | RNA binding | Nucleus |
| **CSTF3** | NP_001317.1 | 1479 | **cleavage stimulation factor subunit 3 isoform 1** | 19.1 | 3 | 7 | 7 | 21 | 2.21E+08 | **1.10** | 8 | 22.5 | 717 | 82.9 | 8.1 | RNA metabolism | Nucleus;Cytoplasm | RNA binding | Nucleus |
| **PGRMC2** | NP_006311.2 | 10424 | **membrane-associated progesterone receptor component 2** | 10.1 | 1 | 1 | 2 | 13 | 2.20E+08 | **1.10** | 2 | 20.9 | 247 | 26.2 | 5.3 | Cell communication;Signal transduction | Plasma membrane;Nucleus;Cytoplasm | Steroid hormone receptor activity | Plasma membrane |
| **PRDX4** | NP_006397.1 | 10549 | **peroxiredoxin-4 precursor** | 41.3 | 1 | 5 | 8 | 71 | 9.98E+08 | **1.10** | 5 | 7.7 | 271 | 30.5 | 6.3 | Metabolism;Energy pathways | Cytoplasm;Endoplasmic reticulum;Golgi apparatus;Nucleus;Mitochondrion | Peroxidase activity | Cytoplasm |
| **RABL6** | NP_078994.3 | 55684 | **rab-like protein 6 isoform 1** | 9.7 | 3 | 3 | 3 | 9 | 7.69E+07 | **1.10** | 2 | 56.6 | 729 | 79.5 | 5.2 | Cell communication;Signal transduction | Nucleus | GTPase activity | Nucleus |
| **RECQL** | NP_002898.2 | 5965 | **ATP-dependent DNA helicase Q1** | 47.3 | 1 | 21 | 22 | 68 | 1.00E+09 | **1.10** | 17 | 18.8 | 649 | 73.4 | 7.9 | Regulation of nucleobase, nucleoside, nucleotide and nucleic acid metabolism | Nucleus;Mitochondrion | DNA repair protein | Nucleus |
| **TMF1** | NP_009045.2 | 7110 | **TATA element modulatory factor** | 3.8 | 1 | 2 | 2 | 2 | 2.32E+07 | **1.10** | 2 | 36.6 | 1093 | 122.8 | 4.9 | Regulation of nucleobase, nucleoside, nucleotide and nucleic acid metabolism | Golgi apparatus;Nucleus;Cytoplasm | Transcription factor activity | Nucleus |
| **CCT4** | NP_006421.2 | 10575 | **T-complex protein 1 subunit delta isoform a** | 69.0 | 2 | 29 | 30 | 293 | 1.27E+09 | **1.10** | 48 | 18.4 | 539 | 57.9 | 7.8 | Protein metabolism | Cytoplasm;Nucleolus;Microtubule;Mitochondrion | Chaperone activity | Cytoplasm |
| **EIF2A** | NP_114414.2 | 83939 | **eukaryotic translation initiation factor 2A** | 36.9 | 1 | 12 | 12 | 37 | 1.06E+09 | **1.10** | 15 | 14.9 | 585 | 64.9 | 8.9 | Protein metabolism | Cytoplasm | Translation regulator activity | Cytoplasm |
| **GATSL3** | NP_001032755.1 | 652968 | **GATS-like protein 3** | 1.8 | 1 | 1 | 1 | 13 | 3.69E+08 | **1.10** | 2 | 3.2 | 329 | 36.3 | 5.2 | Biological_process unknown | - | Molecular function unknown | - |
| **RAC1** | NP_008839.2 | 5879 | **ras-related C3 botulinum toxin substrate 1 isoform Rac1** | 42.7 | 5 | 5 | 10 | 64 | 1.37E+09 | **1.10** | 12 | 13.4 | 192 | 21.4 | 8.5 | Cell communication;Signal transduction | Cytoplasm;Cell projection;Plasma membrane;Membrane fraction;Zymogen granule | GTPase activity | Cytoplasm |
| **CD2AP** | NP_036252.1 | 23607 | **CD2-associated protein** | 27.4 | 1 | 11 | 11 | 30 | 2.01E+08 | **1.09** | 8 | 20.7 | 639 | 71.4 | 6.4 | Cell communication;Signal transduction | Cytoplasm | Receptor signaling complex scaffold activity | Cytoplasm |
| **MTHFS** | NP_001186687.1 | 10588 | **5-formyltetrahydrofolate cyclo-ligase isoform b** | 11.6 | 3 | 1 | 1 | 2 | 8.48E+07 | **1.09** | 2 | 19.7 | 146 | 16.8 | 5.0 | Metabolism;Energy pathways | Mitochondrion | Ligase activity | Mitochondrion |
| **SCFD1** | NP_057190.2 | 23256 | **sec1 family domain-containing protein 1 isoform a** | 38.5 | 5 | 13 | 13 | 31 | 4.82E+08 | **1.09** | 16 | 30.5 | 642 | 72.3 | 6.3 | Transport | Plasma membrane;Golgi apparatus;Nucleus | Auxiliary transport protein activity | Plasma membrane |
| **ADAM9** | NP_003807.1 | 8754 | **disintegrin and metalloproteinase domain-containing protein 9 precursor** | 7.5 | 1 | 3 | 3 | 3 | 2.21E+07 | **1.09** | 2 | 66.6 | 819 | 90.5 | 7.5 | Protein metabolism | Plasma membrane;Nucleus;Cytoplasm | Metallopeptidase activity | Plasma membrane;Nucleus;Cytoplasm |
| **PPIE** | NP_982281.1 | 10450 | **peptidyl-prolyl cis-trans isomerase E isoform 2** | 36.8 | 3 | 4 | 5 | 9 | 1.60E+08 | **1.09** | 3 | 17.7 | 296 | 33.1 | 5.6 | Protein metabolism;RNA metabolism | Nucleus | Isomerase activity;RNA binding | Nucleus |
| **RPS27A** | NP_001170884.1 | 6233 | **ubiquitin-40S ribosomal protein S27a precursor** | 60.3 | 4 | 9 | 9 | 72 | 1.85E+09 | **1.09** | 24 | 24.1 | 156 | 18 | 9.6 | Protein metabolism | Ribosome;Cytoplasm | Ubiquitin-specific protease activity | Ribosome |
| **TRAPPC13** | NP_001087225.1 | 80006 | **trafficking protein particle complex subunit 13 isoform 3** | 5.1 | 4 | 1 | 1 | 2 | 1.07E+08 | **1.09** | 1 | - | 411 | 45.9 | 5.7 | Biological_process unknown | - | Molecular function unknown | - |
| **BCKDK** | NP_001258855.1 | 10295 | **[3-methyl-2-oxobutanoate dehydrogenase [lipoamide]] kinase, mitochondrial isoform c** | 18.2 | 3 | 3 | 3 | 4 | 1.39E+08 | **1.09** | 2 | 11.7 | 335 | 37.7 | 9.3 | Metabolism;Energy pathways | Mitochondrion | Catalytic activity | Mitochondrion |
| **DSTN** | NP_006861.1 | 11034 | **destrin isoform a** | 47.9 | 2 | 9 | 9 | 37 | 4.50E+08 | **1.09** | 16 | 19.8 | 165 | 18.5 | 7.9 | Cell growth and/or maintenance | Nucleus | Cytoskeletal protein binding | Nucleus |
| **MRPL41** | NP_115866.1 | 64975 | **39S ribosomal protein L41, mitochondrial** | 37.2 | 1 | 3 | 3 | 6 | 1.31E+08 | **1.09** | 2 | 5.2 | 137 | 15.4 | 9.6 | Protein metabolism | Mitochondrion | Structural constituent of ribosome | Mitochondrion |
| **ARCN1** | NP_001646.2 | 372 | **coatomer subunit delta isoform 1** | 35.4 | 2 | 15 | 15 | 51 | 6.01E+08 | **1.09** | 21 | 31.1 | 511 | 57.2 | 6.2 | Transport | Cytoplasm;Golgi apparatus;Cytosol | Transporter activity | Cytoplasm |
| **COPS2** | NP_004227.1 | 9318 | **COP9 signalosome complex subunit 2 isoform 1** | 32.5 | 2 | 7 | 7 | 20 | 3.10E+08 | **1.09** | 7 | 4.7 | 443 | 51.6 | 5.5 | Regulation of nucleobase, nucleoside, nucleotide and nucleic acid metabolism | Nucleus;Cytoplasm | Transcription regulator activity | Nucleus |
| **POLR2D** | NP_004796.1 | 5433 | **DNA-directed RNA polymerase II subunit RPB4** | 43.0 | 1 | 3 | 3 | 3 | 1.27E+08 | **1.09** | 3 | 16.1 | 142 | 16.3 | 4.8 | Regulation of nucleobase, nucleoside, nucleotide and nucleic acid metabolism | Nucleus;Cytoplasm;Endoplasmic reticulum | DNA-directed RNA polymerase activity | Nucleus |
| **SBDS** | NP_057122.2 | 51119 | **ribosome maturation protein SBDS** | 47.2 | 1 | 11 | 11 | 30 | 4.69E+08 | **1.09** | 14 | 8.1 | 250 | 28.7 | 8.8 | Biological_process unknown | Nucleolus | Molecular function unknown | Nucleolus |
| **THYN1** | NP_954994.1 | 29087 | **thymocyte nuclear protein 1 isoform 2** | 21.7 | 2 | 3 | 3 | 5 | 3.42E+07 | **1.09** | 2 | 0 | 166 | 18.8 | 9.3 | Biological_process unknown | Nucleus | Molecular function unknown | Nucleus |
| **NKTR** | NP_005376.2 | 4820 | **NK-tumor recognition protein** | 1.6 | 1 | 1 | 1 | 1 | 6.12E+06 | **1.09** | 1 | - | 1462 | 165.6 | 10.0 | Immune response | Plasma membrane;Nucleus;Cytoplasm | Molecular function unknown | Plasma membrane |
| **RTF1** | NP_055953.3 | 23168 | **RNA polymerase-associated protein RTF1 homolog** | 10.3 | 1 | 8 | 8 | 13 | 8.19E+07 | **1.09** | 3 | 3.7 | 710 | 80.3 | 8.2 | Biological_process unknown | - | Molecular function unknown | - |
| **BCR** | NP_067585.2 | 613 | **breakpoint cluster region protein isoform 2** | 4.6 | 2 | 2 | 3 | 5 | 2.53E+07 | **1.09** | 1 | - | 1227 | 137.6 | 7.2 | Cell communication;Signal transduction | Cytoplasm;Nucleus | Protein serine/threonine kinase activity | Cytoplasm |
| **SPC24** | NP_872319.1 | 147841 | **kinetochore protein Spc24** | 29.4 | 1 | 3 | 3 | 8 | 1.15E+08 | **1.09** | 5 | 17 | 197 | 22.4 | 4.7 | Cell communication;Signal transduction | Kinetochore;Nucleus | Protein binding | Nucleus |
| **MYO9B** | NP_001123537.1 | 4650 | **unconventional myosin-IXb isoform 2** | 2.4 | 2 | 3 | 3 | 5 | 1.07E+08 | **1.09** | 1 | - | 2022 | 229 | 8.6 | Cell growth and/or maintenance | Cytoplasm;Nucleus | Motor activity | Cytoplasm |
| **POLR2E** | NP_002686.2 | 5434 | **DNA-directed RNA polymerases I, II, and III subunit RPABC1** | 38.1 | 1 | 4 | 4 | 13 | 1.81E+08 | **1.09** | 6 | 22.5 | 210 | 24.6 | 6.0 | Regulation of nucleobase, nucleoside, nucleotide and nucleic acid metabolism | Nucleus;Nucleolus | DNA-directed RNA polymerase activity | Nucleus |
| **SACM1L** | NP_054735.3 | 22908 | **phosphatidylinositide phosphatase SAC1** | 18.4 | 1 | 7 | 7 | 17 | 1.47E+08 | **1.09** | 6 | 17.9 | 587 | 66.9 | 7.1 | Cell communication;Signal transduction | Endoplasmic reticulum;Golgi apparatus | Lipid phosphatase activity | Endoplasmic reticulum |
| **CFDP1** | NP_006315.1 | 10428 | **craniofacial development protein 1** | 13.4 | 1 | 3 | 3 | 5 | 6.35E+07 | **1.09** | 3 | 7.4 | 299 | 33.6 | 4.8 | Cell growth and/or maintenance | Cytoplasm;Nucleus;Extracellular | Molecular function unknown | Cytoplasm |
| **CLCA1** | NP_001276.2 | 1179 | **calcium-activated chloride channel regulator 1 precursor** | 2.2 | 1 | 1 | 1 | 1 | 5.32E+08 | **1.09** | 1 | - | 914 | 100.2 | 6.4 | Transport | Plasma membrane | Auxiliary transport protein activity | Plasma membrane |

Supplementary Table S4. List of all proteins identified in SCC-S and SCC-R cells using SequestHT and Mascot search algorithms and their gene ontology annotation

| Gene Symbol | RefSeq_Accession | Entrez Gene ID | Description | Coverage (%) | Proteins | Unique Peptides | # Peptides | PSM | Area | SCC-R /SCC-S | SCC-R /SCC-S (Count) | SCC-R /SCC-S (Variability [%]) | # AAs | MW [kDa] | calc. pI | Biological Process | Localization | Molecular Function | Primary Localization |
|---|---|---|---|---|---|---|---|---|---|---|---|---|---|---|---|---|---|---|---|
| **CASP1** | NP_001214.1 | 834 | **caspase-1 isoform beta precursor** | 61.1 | 5 | 18 | 20 | 95 | 1.32E+09 | **1.09** | 21 | 53.8 | 383 | 42.9 | 6.2 | Apoptosis | Cytoplasm | Cysteine-type peptidase activity | Cytoplasm |
| **CHMP7** | NP_689485.1 | 91782 | **charged multivesicular body protein 7** | 5.5 | 1 | 2 | 2 | 3 | 1.31E+08 | **1.09** | 1 | - | 453 | 50.9 | 5.4 | Biological_process unknown | - | Molecular function unknown | - |
| **COTL1** | NP_066972.1 | 23406 | **coactosin-like protein** | 60.6 | 1 | 8 | 8 | 32 | 4.90E+08 | **1.09** | 13 | 22.5 | 142 | 15.9 | 5.7 | Cell growth and/or maintenance | Cytoplasm | Molecular function unknown | Cytoplasm |
| **ERO1L** | NP_055399.1 | 30001 | **ERO1-like protein alpha precursor** | 44.7 | 2 | 12 | 12 | 42 | 5.18E+08 | **1.09** | 15 | 34.8 | 468 | 54.4 | 5.7 | Metabolism;Energy pathways | Endoplasmic reticulum;Mitochondrion | Oxidoreductase activity | Endoplasmic reticulum |
| **IKBKAP** | NP_003631.2 | 8518 | **elongator complex protein 1** | 12.8 | 1 | 11 | 11 | 15 | 7.84E+07 | **1.09** | 6 | 27.9 | 1332 | 150.2 | 5.9 | Cell communication;Signal transduction | Cytoplasm;Nucleus | Receptor signaling complex scaffold activity | Cytoplasm |
| **NIT2** | NP_064587.1 | 56954 | **omega-amidase NIT2** | 54.4 | 1 | 9 | 9 | 32 | 6.16E+08 | **1.09** | 11 | 8.9 | 276 | 30.6 | 7.2 | Metabolism;Energy pathways | - | Hydrolase activity | - |
| **NCKAP1** | NP_038464.1 | 10787 | **nck-associated protein 1 isoform 1** | 20.1 | 2 | 14 | 14 | 32 | 3.70E+08 | **1.09** | 17 | 14.4 | 1128 | 128.7 | 6.6 | Cell communication;Signal transduction;Anti-apoptosis | Cytoplasm;Mitochondrion | Protein binding | Cytoplasm |
| **NOP16** | NP_057475.2 | 51491 | **nucleolar protein 16 isoform 3** | 32.6 | 7 | 4 | 4 | 11 | 2.55E+08 | **1.09** | 4 | 21.9 | 178 | 21.2 | 9.9 | Biological_process unknown | Nucleolus | Molecular function unknown | Nucleolus |
| **NUDC** | NP_006591.1 | 10726 | **nuclear migration protein nudC** | 27.2 | 1 | 9 | 9 | 30 | 3.29E+08 | **1.08** | 12 | 19.3 | 331 | 38.2 | 5.4 | Cell communication;Signal transduction | Cytoplasm;Nucleus | Protein binding | Cytoplasm |
| **PSMD7** | NP_002802.2 | 5713 | **26S proteasome non-ATPase regulatory subunit 7** | 39.5 | 1 | 8 | 8 | 63 | 8.05E+08 | **1.08** | 18 | 16.5 | 324 | 37 | 6.8 | Protein metabolism | Nucleus | Ubiquitin-specific protease activity | Nucleus |
| **RAB5C** | NP_958842.1 | 5878 | **ras-related protein Rab-5C isoform a** | 51.9 | 2 | 7 | 8 | 34 | 5.96E+08 | **1.08** | 10 | 15.2 | 216 | 23.5 | 8.4 | Cell communication;Signal transduction | Endosome;Cytoplasm;Mitochondrion;Cytosol | GTPase activity | Endosome |
| **SF1** | NP_973727.1 | 7536 | **splicing factor 1 isoform 3** | 26.3 | 5 | 2 | 11 | 45 | 8.89E+08 | **1.08** | 1 | - | 548 | 59.7 | 9.5 | Regulation of nucleobase, nucleoside, nucleotide and nucleic acid metabolism | Nucleus;Cytoplasm | RNA binding | Nucleus;Cytoplasm |
| **SKP1** | NP_733779.1 | 6500 | **S-phase kinase-associated protein 1 isoform b** | 36.8 | 2 | 6 | 6 | 23 | 2.50E+08 | **1.08** | 3 | 2.1 | 163 | 18.6 | 4.5 | Protein metabolism;Regulation of cell cycle;Chromosome segregation | Cytoplasm;Nucleus;Nucleolus;Chromosome | Ubiquitin-specific protease activity | Nucleus |
| **STX17** | NP_060389.2 | 55014 | **syntaxin-17** | 9.6 | 1 | 1 | 1 | 2 | 7.52E+07 | **1.08** | 2 | 28.6 | 302 | 33.4 | 6.6 | Transport | Endoplasmic reticulum;Cytoplasm | Auxiliary transport protein activity | Endoplasmic reticulum |
| **TUBB2A** | NP_001060.1 | 7280 | **tubulin beta-2A chain** | 69.9 | 2 | 5 | 32 | 1109 | 1.33E+10 | **1.08** | 5 | 4.5 | 445 | 49.9 | 4.9 | - | - | - | - |
| **VPS4B** | NP_004860.2 | 9525 | **vacuolar protein sorting-associated protein 4B** | 14.2 | 4 | 5 | 5 | 16 | 1.05E+08 | **1.08** | 7 | 21.8 | 444 | 49.3 | 7.2 | Transport | Perinuclear region | Transporter activity | Perinuclear region |
| **YWHAH** | NP_003396.1 | 7533 | **14-3-3 protein eta** | 69.5 | 1 | 17 | 22 | 250 | 1.54E+10 | **1.08** | 30 | 11.3 | 246 | 28.2 | 4.8 | Cell communication;Signal transduction | Cytoplasm;Nucleus | Receptor signaling complex scaffold activity | Cytoplasm |
| **COG5** | NP_859422.2 | 10466 | **conserved oligomeric Golgi complex subunit 5 isoform 2** | 9.7 | 3 | 5 | 5 | 9 | 3.13E+07 | **1.08** | 5 | 29.3 | 839 | 92.7 | 6.6 | Transport;Golgi organization and biogenesis | Golgi apparatus;Cytoplasm | Transporter activity;Structural molecule activity | Golgi apparatus |
| **LRRC47** | NP_065761.1 | 57470 | **leucine-rich repeat-containing protein 47** | 53.3 | 1 | 19 | 19 | 38 | 3.16E+08 | **1.08** | 18 | 14.3 | 583 | 63.4 | 8.3 | Biological_process unknown | Cytoplasm | Molecular function unknown | Cytoplasm |
| **MOB4** | NP_056202.2 | 25843 | **MOB-like protein phocein isoform 1** | 32.4 | 3 | 5 | 5 | 8 | 2.42E+08 | **1.08** | 3 | 5.5 | 225 | 26 | 5.8 | Biological_process unknown | - | Molecular function unknown | - |
| **MUT** | NP_000246.2 | 4594 | **methylmalonyl-CoA mutase, mitochondrial precursor** | 19.7 | 1 | 9 | 9 | 15 | 1.50E+08 | **1.08** | 7 | 25.8 | 750 | 83.1 | 6.9 | Metabolism;Energy pathways | Mitochondrion;Extracellular | Catalytic activity | Mitochondrion |
| **PPP4C** | NP_002711.1 | 5531 | **serine/threonine-protein phosphatase 4 catalytic subunit** | 29.6 | 1 | 4 | 5 | 16 | 1.37E+08 | **1.08** | 3 | 22.6 | 307 | 35.1 | 5.1 | Cell communication;Signal transduction | Nucleus;Centrosome;Cytoplasm | Protein serine/threonine phosphatase activity | Nucleus |
| **SCP2** | NP_001180546.1 | 6342 | **non-specific lipid-transfer protein isoform 8 proprotein** | 22.3 | 7 | 11 | 11 | 39 | 3.99E+08 | **1.08** | 16 | 33.7 | 466 | 50.3 | 7.0 | Transport;Lipid metabolism | Cytoplasm;Mitochondrion;Peroxisome;Endoplasmic reticulum;Plasma membrane;Microsome;Lysosome;Soluble fraction | Auxiliary transport protein activity;Sterol transporter activity | Cytoplasm |
| **PSEN1** | NP_015557.2 | 5663 | **presenilin-1 isoform I-463** | 4.1 | 2 | 1 | 1 | 2 | 3.55E+07 | **1.08** | 1 | - | 463 | 52.2 | 5.2 | Cell-cell adhesion | Perinuclear region;Plasma membrane;Cytoplasm;Endoplasmic reticulum;Golgi apparatus;Kinetochore;Centrosome;Nucleus | Cell adhesion molecule activity | Endoplasmic reticulum;Nucleus;Cytoplasm |
| **UBA1** | NP_003325.2 | 7317 | **ubiquitin-like modifier-activating enzyme 1** | 55.3 | 1 | 37 | 37 | 266 | 1.77E+09 | **1.08** | 60 | 27.8 | 1058 | 117.8 | 5.8 | Proteolysis and peptidolysis | Nucleus;Cytoplasm;Cytosol | Ubiquitin-specific protease activity | Nucleus |
| **IGF2R** | NP_000867.2 | 3482 | **cation-independent mannose-6-phosphate receptor precursor** | 9.4 | 1 | 14 | 14 | 27 | 4.46E+08 | **1.08** | 13 | 20.6 | 2491 | 274.1 | 5.9 | Cell communication;Signal transduction | Plasma membrane;Cytoplasm;Nucleus | Receptor activity | Plasma membrane |
| **PSMC5** | NP_002796.4 | 5705 | **26S protease regulatory subunit 8 isoform 1** | 56.2 | 2 | 16 | 17 | 64 | 6.72E+08 | **1.08** | 14 | 19.9 | 406 | 45.6 | 7.6 | Protein metabolism | Cytoplasm;Nucleus | Ubiquitin-specific protease activity | Cytoplasm |
| **SNX5** | NP_055241.1 | 27131 | **sorting nexin-5 isoform a** | 20.5 | 2 | 5 | 5 | 17 | 4.83E+08 | **1.08** | 6 | 5.3 | 404 | 46.8 | 6.8 | Transport | Endosome;Cytoplasm;Plasma membrane | Transporter activity | Endosome |
| **RHOG** | NP_001656.2 | 391 | **rho-related GTP-binding protein RhoG precursor** | 58.1 | 3 | 6 | 7 | 17 | 2.94E+08 | **1.08** | 3 | 3.9 | 191 | 21.3 | 8.1 | Cell communication;Signal transduction | Nucleus;Plasma membrane | GTPase activity | Nucleus |
| **TROVE2** | NP_001166995.1 | 6738 | **60 kDa SS-A/Ro ribonucleoprotein isoform 2** | 29.7 | 4 | 11 | 11 | 28 | 2.64E+08 | **1.08** | 12 | 20.7 | 538 | 60.6 | 8.0 | Regulation of nucleobase, nucleoside, nucleotide and nucleic acid metabolism | Cytoplasm;Nucleus;Nucleolus;Plasma membrane | RNA binding | Cytoplasm |
| **DDX46** | NP_055644.2 | 9879 | **probable ATP-dependent RNA helicase DDX46** | 24.6 | 1 | 19 | 19 | 49 | 4.57E+08 | **1.08** | 18 | 12 | 1031 | 117.3 | 9.3 | Regulation of nucleobase, nucleoside, nucleotide and nucleic acid metabolism | Nucleus;Cytoplasm | Helicase activity | Nucleus;Cytoplasm |
| **NUP88** | NP_002523.2 | 4927 | **nuclear pore complex protein Nup88** | 39.7 | 1 | 15 | 15 | 22 | 1.98E+08 | **1.08** | 13 | 18 | 741 | 83.5 | 5.7 | Transport | Nucleus;Nuclear membrane | Transporter activity | Nucleus |
| **TMEM263** | NP_689474.1 | 90488 | **transmembrane protein 263** | 75.9 | 2 | 4 | 4 | 8 | 2.62E+08 | **1.08** | 3 | 6 | 116 | 11.7 | 9.3 | Biological_process unknown | Integral to membrane | Molecular function unknown | Integral to membrane |
| **RAD21** | NP_006256.1 | 5885 | **double-strand-break repair protein rad21 homolog** | 28.2 | 1 | 9 | 9 | 22 | 2.74E+08 | **1.08** | 9 | 23.5 | 631 | 71.6 | 4.7 | Regulation of nucleobase, nucleoside, nucleotide and nucleic acid metabolism | Nucleus;Cytoplasm;Chromosome | DNA binding | Nucleus |
| **VPS29** | NP_057310.1 | 51699 | **vacuolar protein sorting-associated protein 29 isoform 1** | 36.8 | 4 | 5 | 5 | 25 | 6.46E+08 | **1.08** | 6 | 19.4 | 182 | 20.5 | 6.8 | Protein metabolism;Protein targeting | Endosome;Golgi apparatus | Hydrolase activity;Protein binding | Endosome |
| **GATC** | NP_789788.1 | 283459 | **glutamyl-tRNA(Gln) amidotransferase subunit C, mitochondrial** | 19.9 | 1 | 1 | 1 | 2 | 2.58E+08 | **1.08** | 1 | - | 136 | 15.1 | 5.1 | Biological_process unknown | - | Molecular function unknown | - |
| **ACBD3** | NP_073572.2 | 64746 | **Golgi resident protein GCP60** | 33.7 | 1 | 9 | 9 | 36 | 3.19E+08 | **1.08** | 17 | 27.8 | 528 | 60.6 | 5.1 | Cell communication;Signal transduction | Golgi apparatus;Cytoplasm | Transporter activity | Golgi apparatus |

| Gene Symbol | RefSeq_Accession | Entrez Gene ID | Description | Coverage (%) | Proteins | Unique Peptides | # Peptides | PSM | Area | SCC-R /SCC-S | SCC-R /SCC-S (Count) | SCC-R /SCC-S (Variability [%]) | # AAs | MW [kDa] | calc. pI | Biological Process | Localization | Molecular Function | Primary Localization |
|---|---|---|---|---|---|---|---|---|---|---|---|---|---|---|---|---|---|---|---|
| **PARP12** | NP_073587.1 | 64761 | **poly [ADP-ribose] polymerase 12** | 5.9 | 1 | 2 | 2 | 3 | 2.80E+07 | **1.08** | 1 | - | 701 | 79 | 8.5 | Regulation of nucleobase, nucleoside, nucleotide and nucleic acid metabolism | - | DNA binding | - |
| **WDR77** | NP_077007.1 | 79084 | **methylosome protein 50** | 16.4 | 1 | 5 | 5 | 13 | 7.70E+07 | **1.08** | 4 | 13.1 | 342 | 36.7 | 5.2 | Biological_process unknown | Nucleus | Molecular function unknown | Nucleus |
| **YIPF3** | NP_056203.2 | 25844 | **protein YIPF3** | 14.0 | 1 | 3 | 3 | 4 | 1.27E+08 | **1.08** | 1 | - | 350 | 38.2 | 5.8 | Biological_process unknown | - | Molecular function unknown | - |
| **NOSIP** | NP_057037.1 | 51070 | **nitric oxide synthase-interacting protein** | 24.9 | 1 | 4 | 4 | 6 | 1.22E+08 | **1.07** | 4 | 19 | 301 | 33.2 | 8.8 | Transport | Cytoplasmic vesicle;Nucleolus | Transporter activity | Cytoplasmic vesicle |
| **PDXK** | NP_003672.1 | 8566 | **pyridoxal kinase** | 58.0 | 1 | 9 | 9 | 31 | 3.20E+08 | **1.07** | 9 | 21.4 | 312 | 35.1 | 6.1 | Metabolism;Energy pathways | Cytoplasm | Catalytic activity | Cytoplasm |
| **PRPF40A** | NP_060362.3 | 55660 | **pre-mRNA-processing factor 40 homolog A** | 8.5 | 1 | 6 | 6 | 22 | 2.77E+08 | **1.07** | 3 | 16.4 | 930 | 105.9 | 8.0 | Regulation of nucleobase, nucleoside, nucleotide and nucleic acid metabolism | Nucleus;Nuclear matrix;Cytoplasm | RNA binding | Nucleus;Cytoplasm |
| **PTRF** | NP_036364.2 | 284119 | **polymerase I and transcript release factor** | 44.1 | 1 | 13 | 13 | 60 | 6.30E+08 | **1.07** | 23 | 11.9 | 390 | 43.4 | 5.6 | Regulation of nucleobase, nucleoside, nucleotide and nucleic acid metabolism | Nucleus;Cytoplasm | Transcription regulator activity | Nucleus;Cytoplasm |
| **SFPQ** | NP_005057.1 | 6421 | **splicing factor, proline- and glutamine-rich** | 54.3 | 1 | 32 | 33 | 214 | 5.41E+09 | **1.07** | 69 | 15 | 707 | 76.1 | 9.4 | Regulation of nucleobase, nucleoside, nucleotide and nucleic acid metabolism | Nucleus;Nucleolus;Cytoplasm | RNA binding | Nucleus |
| **TNPO2** | NP_038461.2 | 30000 | **transportin-2 isoform 2** | 21.8 | 2 | 5 | 10 | 29 | 5.60E+08 | **1.07** | 5 | 9 | 887 | 100.3 | 5.0 | Transport | Nucleoplasm;Cytoplasm | Transporter activity | Nucleoplasm |
| **BUD31** | NP_003901.2 | 8896 | **protein BUD31 homolog** | 34.7 | 1 | 5 | 5 | 15 | 1.55E+08 | **1.07** | 6 | 21 | 144 | 17 | 8.8 | Regulation of nucleobase, nucleoside, nucleotide and nucleic acid metabolism | Nucleus | Transcription regulator activity | Nucleus |
| **CCNT1** | NP_001231.2 | 904 | **cyclin-T1 isoform a** | 3.2 | 1 | 1 | 1 | 1 | 2.82E+07 | **1.07** | 1 | - | 726 | 80.6 | 8.8 | Cell communication;Signal transduction | Nucleus;Cytoplasm;Nucleolus | Kinase binding | Nucleus |
| **HEATR2** | NP_060272.3 | 54919 | **HEAT repeat-containing protein 2** | 12.1 | 1 | 4 | 4 | 9 | 1.11E+08 | **1.07** | 2 | 8.5 | 855 | 93.5 | 6.4 | Biological_process unknown | - | Molecular function unknown | - |
| **PTRHD1** | NP_001013685.1 | 391356 | **putative peptidyl-tRNA hydrolase PTRHD1** | 61.4 | 1 | 5 | 5 | 18 | 5.43E+08 | **1.07** | 6 | 21.1 | 140 | 15.8 | 9.1 | Biological_process unknown | - | Molecular function unknown | - |
| **TXN** | NP_003320.2 | 7295 | **thioredoxin isoform 1** | 51.4 | 2 | 6 | 6 | 44 | 1.63E+09 | **1.07** | 9 | 10.2 | 105 | 11.7 | 4.9 | Metabolism;Energy pathways | Cytoplasm;Nucleus;Extracellular | Catalytic activity | Cytoplasm |
| **UFSP2** | NP_060829.2 | 55325 | **ufm1-specific protease 2** | 8.1 | 1 | 2 | 2 | 2 | 2.83E+07 | **1.07** | 1 | - | 469 | 53.2 | 7.0 | Protein metabolism | - | Peptidase activity | - |
| **BCLAF1** | NP_055554.1 | 9774 | **bcl-2-associated transcription factor 1 isoform 1** | 18.6 | 3 | 14 | 15 | 30 | 1.80E+08 | **1.07** | 15 | 10.7 | 920 | 106.1 | 10.0 | Regulation of nucleobase, nucleoside, nucleotide and nucleic acid metabolism | Nucleus;Cytoplasm | Transcription factor activity | Nucleus |
| **PABPC1** | NP_002559.2 | 26986 | **polyadenylate-binding protein 1** | 60.4 | 3 | 11 | 35 | 207 | 2.99E+09 | **1.07** | 15 | 28.4 | 636 | 70.6 | 9.5 | Regulation of nucleobase, nucleoside, nucleotide and nucleic acid metabolism | Cytoplasm;Nucleus | RNA binding | Cytoplasm |
| **SLC38A2** | NP_061849.2 | 54407 | **sodium-coupled neutral amino acid transporter 2** | 7.5 | 1 | 3 | 3 | 19 | 4.09E+08 | **1.07** | 4 | 9.7 | 506 | 56 | 8.0 | Transport | Plasma membrane;Nucleus;Cytoplasm | Transporter activity | Plasma membrane |
| **C16orf13** | NP_001035251.1 | 84326 | **UPF0585 protein C16orf13 isoform c** | 16.8 | 4 | 1 | 1 | 1 | 2.82E+06 | **1.07** | 1 | - | 107 | 12 | 7.4 | Biological_process unknown | - | Molecular function unknown | - |
| **CDK7** | NP_001790.1 | 1022 | **cyclin-dependent kinase 7** | 7.5 | 1 | 1 | 1 | 1 | 2.55E+07 | **1.07** | 1 | - | 346 | 39 | 8.5 | Regulation of nucleobase, nucleoside, nucleotide and nucleic acid metabolism | Cytoplasm;Nucleus;Nucleolus | Protein serine/threonine kinase activity | Nucleus |
| **MYD88** | NP_001166040.1 | 4615 | **myeloid differentiation primary response protein MyD88 isoform 4** | 13.2 | 5 | 2 | 2 | 7 | 6.02E+07 | **1.07** | 1 | - | 204 | 22.1 | 5.1 | Cell communication;Signal transduction | Nucleus;Cytoplasm;Plasma membrane;Cytosol | Receptor signaling complex scaffold activity | Nucleus |
| **TMED4** | NP_872353.2 | 222068 | **transmembrane emp24 domain-containing protein 4 precursor** | 27.8 | 1 | 4 | 5 | 16 | 3.50E+08 | **1.07** | 5 | 19.5 | 227 | 25.9 | 8.3 | Biological_process unknown | - | Molecular function unknown | - |
| **ZFR** | NP_057191.2 | 51663 | **zinc finger RNA-binding protein** | 23.3 | 1 | 14 | 14 | 29 | 4.80E+08 | **1.07** | 13 | 12.7 | 1074 | 116.9 | 9.0 | Regulation of nucleobase, nucleoside, nucleotide and nucleic acid metabolism | Nucleus;Nucleolus | Transcription regulator activity | Nucleus |
| **KPNA3** | NP_002258.2 | 3839 | **importin subunit alpha-4** | 20.9 | 1 | 4 | 7 | 14 | 2.41E+08 | **1.07** | 3 | 1.3 | 521 | 57.8 | 4.9 | Transport | Nucleus;Cytoplasm;Nucleolus | Transporter activity | Nucleus |
| **SREK1** | NP_001070667.1 | 140890 | **splicing regulatory glutamine/lysine-rich protein 1 isoform a** | 24.0 | 4 | 9 | 9 | 19 | 1.35E+08 | **1.07** | 7 | 18 | 624 | 71.6 | 10.2 | Regulation of nucleobase, nucleoside, nucleotide and nucleic acid metabolism | Nucleus | RNA binding | Nucleus |
| **ERH** | NP_004441.1 | 2079 | **enhancer of rudimentary homolog** | 55.8 | 1 | 6 | 6 | 53 | 3.40E+09 | **1.07** | 13 | 11.6 | 104 | 12.3 | 5.9 | Regulation of nucleobase, nucleoside, nucleotide and nucleic acid metabolism | Nucleus;Nucleolus;Chromosome;Cytoplasm | Transcription regulator activity | Nucleus |
| **GNA11** | NP_002058.2 | 2767 | **guanine nucleotide-binding protein subunit alpha-11** | 19.5 | 2 | 5 | 6 | 13 | 9.17E+07 | **1.07** | 5 | 28.7 | 359 | 42.1 | 5.7 | Cell communication;Signal transduction | Plasma membrane;Cytoplasm | GTPase activity | Plasma membrane |
| **TAB1** | NP_705717.1 | 10454 | **TGF-beta-activated kinase 1 and MAP3K7-binding protein 1 isoform beta** | 17.8 | 2 | 3 | 3 | 7 | 9.99E+07 | **1.07** | 2 | 27.4 | 462 | 49.9 | 5.6 | Regulation of protein kinase activity | Cytoplasm | Kinase activity | Cytoplasm |
| **CTNNBL1** | NP_110517.2 | 56259 | **beta-catenin-like protein 1 isoform 1** | 15.3 | 2 | 8 | 8 | 20 | 2.32E+08 | **1.07** | 10 | 32.4 | 563 | 65.1 | 5.1 | Apoptosis | Nucleus;Nucleolus;Chromosome | Molecular function unknown | Nucleus |
| **PCMT1** | NP_001238982.1 | 5110 | **protein-L-isoaspartate(D-aspartate) O-methyltransferase isoform 1** | 54.7 | 5 | 10 | 10 | 55 | 1.55E+09 | **1.07** | 22 | 16.3 | 285 | 30.3 | 7.5 | Protein metabolism | Cytoplasm | Methyltransferase activity | Cytoplasm |
| **TTPAL** | NP_001248768.1 | 79183 | **alpha-tocopherol transfer protein-like isoform 2** | 5.5 | 2 | 1 | 1 | 1 | 1.59E+07 | **1.07** | 1 | - | 308 | 34.9 | 5.7 | Transport | - | Molecular function unknown | - |
| **COPG2** | NP_036265.3 | 26958 | **coatomer subunit gamma-2 isoform 1** | 19.8 | 2 | 8 | 9 | 38 | 2.19E+08 | **1.07** | 9 | 12.8 | 871 | 97.6 | 5.8 | Transport | Cytoplasm;Golgi apparatus | Transporter activity | Cytoplasm |
| **YBEY** | NP_001006114.1 | 54059 | **putative ribonuclease isoform 2** | 13.2 | 2 | 1 | 1 | 1 | 3.14E+07 | **1.07** | 1 | - | 121 | 14.1 | 6.2 | Biological_process unknown | - | Molecular function unknown | - |
| **PACS1** | NP_060496.2 | 55690 | **phosphofurin acidic cluster sorting protein 1** | 1.5 | 1 | 1 | 1 | 2 | 1.02E+08 | **1.06** | 1 | - | 963 | 104.8 | 7.7 | Transport | Cytoplasm;Golgi apparatus | Receptor signaling complex scaffold activity | Cytoplasm |
| **POLE4** | NP_063949.2 | 56655 | **DNA polymerase epsilon subunit 4** | 33.3 | 1 | 2 | 2 | 3 | 1.85E+08 | **1.06** | 1 | - | 117 | 12.2 | 4.9 | Regulation of nucleobase, nucleoside, nucleotide and nucleic acid metabolism | Nucleus | DNA-directed DNA polymerase activity | Nucleus |
| **PROSC** | NP_009129.1 | 11212 | **proline synthase co-transcribed bacterial homolog protein** | 15.6 | 1 | 3 | 3 | 10 | 8.69E+07 | **1.06** | 3 | 3.1 | 275 | 30.3 | 7.5 | Metabolism;Energy pathways | Cytoplasm | Racemase and epimerase activity | Cytoplasm |
| **SLC25A12** | NP_003696.2 | 8604 | **calcium-binding mitochondrial carrier protein Aralar1** | 14.2 | 1 | 6 | 6 | 12 | 7.22E+07 | **1.06** | 3 | 17 | 678 | 74.7 | 8.4 | Transport | Mitochondrion;Cytoplasm | Transporter activity | Mitochondrion |

| Gene Symbol | RefSeq_Accession | Entrez Gene ID | Description | Coverage (%) | Proteins | Unique Peptides | # Peptides | PSM | Area | SCC-R /SCC-S | SCC-R /SCC-S (Count) | SCC-R /SCC-S (Variability [%]) | # AAs | MW [kDa] | calc. pI | Biological Process | Localization | Molecular Function | Primary Localization |
|---|---|---|---|---|---|---|---|---|---|---|---|---|---|---|---|---|---|---|---|
| **USP10** | NP_005144.2 | 9100 | **ubiquitin carboxyl-terminal hydrolase 10 isoform 2** | 31.3 | 2 | 14 | 14 | 31 | 3.82E+08 | **1.06** | 16 | 16 | 798 | 87.1 | 5.3 | Protein metabolism | Nucleus;Cytoplasm | Ubiquitin-specific protease activity | Nucleus;Cytoplasm |
| **DNAJB3** | NP_001001394.1 | 414061 | **dnaJ homolog subfamily B member 3** | 9.0 | 3 | 1 | 1 | 2 | 1.34E+08 | **1.06** | 1 | - | 145 | 16.5 | 4.9 | Protein metabolism | - | Chaperone activity | - |
| **PLAA** | NP_001026859.1 | 9373 | **phospholipase A-2-activating protein** | 21.5 | 1 | 10 | 10 | 20 | 6.22E+07 | **1.06** | 11 | 51 | 795 | 87.1 | 6.4 | Cell communication;Signal transduction | Cytoplasm | Receptor signaling complex scaffold activity | Cytoplasm |
| **RRP15** | NP_057136.2 | 51018 | **RRP15-like protein** | 3.9 | 1 | 1 | 1 | 1 | 1.44E+07 | **1.06** | 1 | - | 282 | 31.5 | 5.5 | Biological_process unknown | Nucleolus;Cytosol | Molecular function unknown | Nucleolus |
| **TSPAN6** | NP_001265672.1 | 7105 | **tetraspanin-6 isoform d precursor** | 8.5 | 4 | 1 | 1 | 1 | 1.36E+07 | **1.06** | 1 | - | 129 | 14.9 | 7.2 | Transport | Plasma membrane | Receptor activity | Plasma membrane |
| **TTC38** | NP_060401.2 | 55020 | **tetratricopeptide repeat protein 38** | 2.6 | 1 | 1 | 1 | 1 | 6.88E+06 | **1.06** | 1 | - | 469 | 52.7 | 6.0 | Biological_process unknown | - | Molecular function unknown | - |
| **ATG4B** | NP_847896.1 | 23192 | **cysteine protease ATG4B isoform b** | 16.8 | 2 | 4 | 4 | 11 | 7.62E+07 | **1.06** | 3 | 7.9 | 380 | 42.6 | 5.5 | Proteolysis and peptidolysis | Cytoplasm;Nucleus | Peptidase activity | Cytoplasm;Nucleus |
| **CLIC4** | NP_039234.1 | 25932 | **chloride intracellular channel protein 4** | 83.8 | 5 | 21 | 22 | 105 | 1.24E+09 | **1.06** | 32 | 15.9 | 253 | 28.8 | 5.6 | Transport | Plasma membrane;Cytoplasm | Intracellular ligand-gated ion channel activity | Cytoplasm |
| **ADAR** | NP_001180424.1 | 103 | **double-stranded RNA-specific adenosine deaminase isoform d** | 38.6 | 2 | 1 | 21 | 58 | 4.08E+08 | **1.06** | 1 | - | 931 | 103.6 | 8.7 | Regulation of nucleobase, nucleoside, nucleotide and nucleic acid metabolism | Cytoplasm;Nucleolus;Nucleus | Deaminase activity | Nucleus |
| **KIAA0368** | NP_001073867.1 | 23392 | **proteasome-associated protein ECM29 homolog** | 25.9 | 1 | 32 | 32 | 69 | 2.23E+08 | **1.06** | 26 | 25.6 | 2017 | 223.6 | 8.8 | Protein metabolism | Centrosome;Endosome;Endoplasmic reticulum;Cytoplasmic vesicle | Translation regulator activity | Centrosome |
| **PPP4R2** | NP_777567.1 | 151987 | **serine/threonine-protein phosphatase 4 regulatory subunit 2** | 24.9 | 1 | 7 | 7 | 21 | 1.76E+08 | **1.06** | 5 | 16.1 | 417 | 46.9 | 4.5 | Cell communication;Signal transduction | Centrosome;Nucleus;Cytoplasm | Protein serine/threonine phosphatase activity | Centrosome |
| **PRKRIR** | NP_004696.2 | 5612 | **52 kDa repressor of the inhibitor of the protein kinase** | 1.3 | 1 | 1 | 1 | 1 | 1.31E+07 | **1.06** | 1 | - | 761 | 87.6 | 5.9 | Protein metabolism | Nucleus | Translation regulator activity | Nucleus |
| **NTPCR** | NP_115700.1 | 84284 | **cancer-related nucleoside-triphosphatase** | 40.5 | 1 | 5 | 5 | 11 | 9.73E+07 | **1.06** | 6 | 19.3 | 190 | 20.7 | 9.5 | Metabolism;Energy pathways | - | ATPase activity | - |
| **PELP1** | NP_055204.3 | 27043 | **proline-, glutamic acid- and leucine-rich protein 1 isoform 1** | 22.5 | 2 | 13 | 13 | 35 | 2.17E+08 | **1.06** | 16 | 18.5 | 1180 | 124.9 | 4.4 | Regulation of nucleobase, nucleoside, nucleotide and nucleic acid metabolism | Nucleus;Nucleolus | Transcription regulator activity | Nucleus |
| **PSPC1** | NP_001035879.1 | 55269 | **paraspeckle component 1** | 39.2 | 1 | 10 | 10 | 32 | 2.30E+08 | **1.06** | 9 | 17.6 | 523 | 58.7 | 6.7 | Regulation of nucleobase, nucleoside, nucleotide and nucleic acid metabolism | Nucleus;Nucleolus | RNA binding | Nucleus |
| **ND2** | YP_003024027.1 | 4536 | **NADH dehydrogenase subunit 2** | 2.6 | 1 | 1 | 1 | 2 | 2.81E+07 | **1.06** | 1 | - | 347 | 38.9 | 9.8 | Metabolism;Energy pathways | Mitochondrion | Oxidoreductase activity | Mitochondrion |
| **PRPF19** | NP_055317.1 | 27339 | **pre-mRNA-processing factor 19** | 66.9 | 1 | 20 | 20 | 124 | 1.60E+09 | **1.06** | 36 | 21.5 | 504 | 55.1 | 6.6 | Regulation of nucleobase, nucleoside, nucleotide and nucleic acid metabolism | Nucleus;Nucleolus | DNA repair protein | Nucleus |
| **AGK** | NP_060708.1 | 55750 | **acylglycerol kinase, mitochondrial precursor** | 13.7 | 1 | 3 | 3 | 6 | 1.14E+08 | **1.06** | 5 | 17.8 | 422 | 47.1 | 8.1 | Cell communication;Signal transduction | Integral to membrane | Lipid kinase activity | Integral to membrane |
| **ENOPH1** | NP_067027.1 | 58478 | **enolase-phosphatase E1 isoform 1** | 34.5 | 1 | 4 | 4 | 8 | 8.58E+07 | **1.06** | 3 | 30.5 | 261 | 28.9 | 4.8 | Biological_process unknown | - | Molecular function unknown | - |
| **GSK3B** | NP_001139628.1 | 2932 | **glycogen synthase kinase-3 beta isoform 2** | 19.5 | 2 | 3 | 4 | 14 | 4.55E+08 | **1.06** | 4 | 33.3 | 420 | 46.7 | 8.8 | Metabolism;Energy pathways | Cytoplasm;Nucleus;Centrosome | Protein serine/threonine kinase activity | Cytoplasm;Nucleus |
| **NDUFB9** | NP_004996.1 | 4715 | **NADH dehydrogenase [ubiquinone] 1 beta subcomplex subunit 9 isoform 1** | 49.2 | 3 | 6 | 6 | 15 | 2.84E+08 | **1.06** | 3 | 127.7 | 179 | 21.8 | 8.4 | Metabolism;Energy pathways | Mitochondrion | Oxidoreductase activity | Mitochondrion |
| **ZW10** | NP_004715.1 | 9183 | **centromere/kinetochore protein zw10 homolog** | 16.3 | 1 | 7 | 7 | 9 | 7.00E+07 | **1.06** | 3 | 17.3 | 779 | 88.8 | 6.3 | Cell communication;Signal transduction | Kinetochore;Cytoplasm;Chromosome | DNA binding | Cytoplasm |
| **ELP4** | NP_061913.3 | 26610 | **elongator complex protein 4 isoform 1** | 8.5 | 3 | 2 | 2 | 3 | 2.58E+07 | **1.06** | 2 | 34.2 | 424 | 46.6 | 8.5 | Regulation of nucleobase, nucleoside, nucleotide and nucleic acid metabolism | Nucleus | Acyltransferase activity | Nucleus |
| **SND1** | NP_055205.2 | 27044 | **staphylococcal nuclease domain-containing protein 1** | 50.0 | 1 | 38 | 38 | 219 | 9.84E+08 | **1.06** | 68 | 21.7 | 910 | 101.9 | 7.2 | Regulation of nucleobase, nucleoside, nucleotide and nucleic acid metabolism | Nucleus | Transcription regulator activity | Nucleus |
| **TPR** | NP_003283.2 | 7175 | **nucleoprotein TPR** | 36.8 | 1 | 56 | 56 | 157 | 6.09E+08 | **1.06** | 54 | 16.6 | 2363 | 267.1 | 5.0 | Regulation of nucleobase, nucleoside, nucleotide and nucleic acid metabolism | Cytoplasm;Nucleus;Nucleolus;Nuclear membrane | Molecular function unknown | Cytoplasm |
| **PTGES3** | NP_001269530.1 | 10728 | **prostaglandin E synthase 3 isoform b** | 59.0 | 6 | 9 | 9 | 50 | 1.59E+09 | **1.06** | 14 | 6.2 | 139 | 16.5 | 4.8 | Protein metabolism | Cytoplasm;Nucleus | Chaperone activity | Cytoplasm |
| **SMU1** | NP_060695.2 | 55234 | **WD40 repeat-containing protein SMU1** | 19.5 | 1 | 6 | 6 | 7 | 7.90E+07 | **1.06** | 4 | 2.8 | 513 | 57.5 | 7.2 | Regulation of nucleobase, nucleoside, nucleotide and nucleic acid metabolism | Nucleus;Cytoplasm | Molecular function unknown | Nucleus |
| **EMC3** | NP_060917.1 | 55831 | **ER membrane protein complex subunit 3** | 12.6 | 1 | 2 | 2 | 3 | 4.59E+07 | **1.05** | 1 | - | 261 | 29.9 | 6.8 | Biological_process unknown | Integral to membrane | Molecular function unknown | Integral to membrane |
| **HNRNPK** | NP_112552.1 | 3190 | **heterogeneous nuclear ribonucleoprotein K isoform b** | 70.0 | 2 | 37 | 37 | 573 | 6.91E+09 | **1.05** | 121 | 16.7 | 463 | 50.9 | 5.5 | Regulation of nucleobase, nucleoside, nucleotide and nucleic acid metabolism | Nucleolus;Cytoplasm;Mitochondrion;Nucleus | Ribonucleoprotein | Nucleus |
| **TAF2** | NP_003175.1 | 6873 | **transcription initiation factor TFIID subunit 2** | 0.9 | 1 | 1 | 1 | 1 | 3.17E+06 | **1.05** | 1 | - | 1199 | 136.9 | 8.2 | Regulation of nucleobase, nucleoside, nucleotide and nucleic acid metabolism | Nucleus;Cytoplasm | Transcription factor activity | Nucleus |
| **COPS3** | NP_003644.2 | 8533 | **COP9 signalosome complex subunit 3 isoform 1** | 17.5 | 2 | 4 | 4 | 18 | 1.64E+08 | **1.05** | 6 | 22.5 | 423 | 47.8 | 6.7 | Cell communication;Signal transduction | Cytoplasm | Transcription regulator activity | Cytoplasm |
| **DAZAP1** | NP_733829.1 | 26528 | **DAZ-associated protein 1 isoform a** | 41.3 | 2 | 11 | 11 | 42 | 1.04E+09 | **1.05** | 13 | 12.8 | 378 | 40.5 | 8.3 | Regulation of nucleobase, nucleoside, nucleotide and nucleic acid metabolism | Nucleus;Cytoplasm | RNA binding | Nucleus |
| **EXOC1** | NP_839955.1 | 55763 | **exocyst complex component 1 isoform 2** | 16.7 | 2 | 8 | 8 | 14 | 7.07E+07 | **1.05** | 9 | 22.9 | 879 | 100.2 | 6.6 | Transport | Cytoplasm | Transporter activity | Cytoplasm |
| **UBL4A** | NP_055050.1 | 8266 | **ubiquitin-like protein 4A** | 33.8 | 1 | 5 | 5 | 7 | 5.85E+07 | **1.05** | 6 | 14.5 | 157 | 17.8 | 8.7 | Protein metabolism | - | Ubiquitin-specific protease activity | - |
| **GYS1** | NP_001155059.1 | 2997 | **glycogen [starch] synthase, muscle isoform 2** | 3.7 | 2 | 2 | 2 | 3 | 1.23E+08 | **1.05** | 2 | 7.4 | 673 | 76.4 | 6.5 | Metabolism;Energy pathways | Cytoplasm;Nucleus;Cytosol | Ligase activity | Cytoplasm |
| **NTMT1** | NP_054783.2 | 28989 | **N-terminal Xaa-Pro-Lys N-methyltransferase 1 isoform a** | 27.8 | 3 | 4 | 4 | 14 | 1.24E+08 | **1.05** | 3 | 0.9 | 223 | 25.4 | 5.5 | Biological_process unknown | - | Molecular function unknown | - |
| **RANBP6** | NP_036548.1 | 26953 | **ran-binding protein 6 isoform 1** | 7.6 | 2 | 4 | 5 | 11 | 2.03E+08 | **1.05** | 1 | - | 1105 | 124.6 | 5.0 | Transport | - | Transporter activity | - |

| Gene Symbol | RefSeq_Accession | Entrez Gene ID | Description | Coverage (%) | Proteins | Unique Peptides | # Peptides | PSM | Area | SCC-R /SCC-S | SCC-R /SCC-S (Count) | SCC-R /SCC-S (Variability [%]) | # AAs | MW [kDa] | calc. pI | Biological Process | Localization | Molecular Function | Primary Localization |
|---|---|---|---|---|---|---|---|---|---|---|---|---|---|---|---|---|---|---|---|
| **CPSF6** | NP_008938.2 | 11052 | **cleavage and polyadenylation specificity factor subunit 6** | 27.6 | 1 | 11 | 11 | 42 | 3.31E+08 | **1.05** | 14 | 9.8 | 551 | 59.2 | 7.2 | Regulation of nucleobase, nucleoside, nucleotide and nucleic acid metabolism | Nucleus | RNA binding | Nucleus |
| **DDX24** | NP_065147.1 | 57062 | **ATP-dependent RNA helicase DDX24** | 34.7 | 1 | 18 | 18 | 52 | 1.65E+08 | **1.05** | 21 | 27.4 | 859 | 96.3 | 9.1 | Transport | Nucleus;Nucleolus | Transporter activity | Nucleus |
| **EIF3G** | NP_003746.2 | 8666 | **eukaryotic translation initiation factor 3 subunit G** | 55.0 | 1 | 8 | 8 | 34 | 7.39E+08 | **1.05** | 11 | 20.4 | 320 | 35.6 | 6.1 | Protein metabolism | Cytoplasm | Translation regulator activity | Cytoplasm |
| **EWSR1** | NP_001156758.1 | 2130 | **RNA-binding protein EWS isoform 4** | 14.8 | 5 | 5 | 5 | 35 | 4.03E+08 | **1.05** | 11 | 23 | 600 | 62.5 | 9.4 | Regulation of nucleobase, nucleoside, nucleotide and nucleic acid metabolism | Nucleus;Cytoplasm;Plasma membrane;Nucleolus | RNA binding | Nucleus |
| **HMGN2** | NP_005508.1 | 3151 | **non-histone chromosomal protein HMG-17** | 51.1 | 1 | 5 | 5 | 33 | 4.62E+08 | **1.05** | 11 | 22.1 | 90 | 9.4 | 10.0 | Regulation of nucleobase, nucleoside, nucleotide and nucleic acid metabolism | Nucleus;Cytoplasm | Transcription regulator activity | Nucleus |
| **MATR3** | NP_001181883.1 | 9782 | **matrin-3 isoform a** | 47.1 | 3 | 30 | 30 | 220 | 1.88E+09 | **1.05** | 63 | 19.2 | 847 | 94.6 | 6.3 | Regulation of nucleobase, nucleoside, nucleotide and nucleic acid metabolism | Nucleus;Mitochondrion;Cytoplasm | RNA binding | Nucleus |
| **NDUFA9** | NP_004993.1 | 4704 | **NADH dehydrogenase [ubiquinone] 1 alpha subcomplex subunit 9, mitochondrial precursor** | 22.6 | 1 | 7 | 7 | 12 | 9.11E+07 | **1.05** | 4 | 41.2 | 377 | 42.5 | 9.8 | Metabolism;Energy pathways | Mitochondrion | Oxidoreductase activity | Mitochondrion |
| **NDUFB7** | NP_004137.2 | 4713 | **NADH dehydrogenase [ubiquinone] 1 beta subcomplex subunit 7** | 20.4 | 1 | 2 | 2 | 3 | 1.02E+08 | **1.05** | 1 | - | 137 | 16.4 | 8.9 | Metabolism;Energy pathways | Mitochondrion | Cell adhesion molecule activity | Mitochondrion |
| **SENP3** | NP_056485.2 | 26168 | **sentrin-specific protease 3** | 6.1 | 1 | 2 | 2 | 5 | 2.68E+07 | **1.05** | 3 | 17 | 574 | 65 | 8.6 | Protein metabolism | Nucleolus;Nucleus | Peptidase activity | Nucleolus |
| **DECR1** | NP_001350.1 | 1666 | **2,4-dienoyl-CoA reductase, mitochondrial precursor** | 38.2 | 1 | 10 | 10 | 39 | 8.23E+08 | **1.05** | 14 | 21.2 | 335 | 36 | 9.3 | Metabolism;Energy pathways | Mitochondrion;Endoplasmic reticulum;Peroxisome | Catalytic activity | Mitochondrion |
| **GNL3** | NP_996561.1 | 26354 | **guanine nucleotide-binding protein-like 3 isoform 2** | 24.4 | 2 | 10 | 10 | 25 | 1.14E+08 | **1.05** | 15 | 36.6 | 537 | 60.5 | 8.8 | Regulation of cell proliferation | Nucleolus;Nucleus | GTP binding | Nucleolus |
| **RNH1** | NP_976317.1 | 6050 | **ribonuclease inhibitor** | 76.8 | 1 | 23 | 23 | 168 | 1.73E+09 | **1.05** | 35 | 10.8 | 461 | 49.9 | 4.8 | Regulation of nucleobase, nucleoside, nucleotide and nucleic acid metabolism | Cytoplasm | Translation regulator activity | Cytoplasm |
| **ABCF3** | NP_060828.2 | 55324 | **ATP-binding cassette sub-family F member 3** | 16.4 | 1 | 7 | 7 | 12 | 1.03E+08 | **1.05** | 4 | 17.2 | 709 | 79.7 | 6.3 | Transport | - | Transporter activity | - |
| **ARPC4** | NP_005709.1 | 10093 | **actin-related protein 2/3 complex subunit 4 isoform a** | 54.2 | 4 | 7 | 7 | 27 | 2.04E+09 | **1.05** | 8 | 17.9 | 168 | 19.7 | 8.4 | Cell growth and/or maintenance | Cytoskeleton;Nucleolus | Cytoskeletal protein binding | Cytoskeleton |
| **NMT1** | NP_066565.1 | 4836 | **glycylpeptide N-tetradecanoyltransferase 1** | 35.1 | 2 | 13 | 13 | 59 | 6.64E+08 | **1.05** | 21 | 19.3 | 496 | 56.8 | 7.8 | Metabolism;Energy pathways;Protein metabolism | Ribosome;Nucleus | Molecular function unknown;Acyltransferase activity | Ribosome |
| **NSUN2** | NP_001180384.1 | 54888 | **tRNA (cytosine(34)-C(5))-methyltransferase isoform 2** | 48.1 | 1 | 1 | 22 | 58 | 5.43E+08 | **1.05** | 1 | - | 732 | 82.3 | 7.0 | - | Nucleolus;Cytoplasm;Nucleus | RNA methyltransferase activity | Nucleolus |
| **CARS** | NP_644802.1 | 833 | **cysteine--tRNA ligase, cytoplasmic isoform a** | 17.6 | 4 | 9 | 9 | 26 | 3.30E+08 | **1.05** | 10 | 18.6 | 726 | 82.8 | 7.0 | Metabolism;Energy pathways | Cytoplasm | Ligase activity | Cytoplasm |
| **ENTHD2** | NP_653280.1 | 146705 | **AP-4 complex accessory subunit tepsin** | 4.8 | 1 | 1 | 1 | 1 | 5.62E+06 | **1.05** | 1 | - | 525 | 55.1 | 7.0 | Regulation of nucleobase, nucleoside, nucleotide and nucleic acid metabolism | - | Transcription regulator activity | - |
| **MPC2** | NP_056230.1 | 25874 | **mitochondrial pyruvate carrier 2** | 9.5 | 1 | 1 | 1 | 2 | 5.59E+06 | **1.05** | 1 | - | 127 | 14.3 | 10.4 | Biological_process unknown | - | Molecular function unknown | - |
| **SPR** | NP_003115.1 | 6697 | **sepiapterin reductase** | 54.8 | 1 | 11 | 11 | 45 | 3.39E+08 | **1.05** | 16 | 14.5 | 261 | 28 | 8.1 | Metabolism;Energy pathways | - | Catalytic activity | - |
| **CETN3** | NP_004356.2 | 1070 | **centrin-3** | 7.2 | 1 | 1 | 1 | 2 | 1.53E+08 | **1.05** | 1 | - | 167 | 19.5 | 4.7 | Cell communication;Signal transduction | Centrosome | Calcium ion binding | Centrosome |
| **NCAPH** | NP_056156.2 | 23397 | **condensin complex subunit 2 isoform 1** | 38.6 | 4 | 16 | 16 | 40 | 3.53E+08 | **1.05** | 7 | 25 | 741 | 82.5 | 5.1 | Cell growth and/or maintenance | Nucleolus;Kinetochore;Nucleus;Cytoplasm | Molecular function unknown | Nucleolus |
| **TMOD3** | NP_055362.1 | 29766 | **tropomodulin-3** | 54.3 | 1 | 14 | 14 | 40 | 4.52E+08 | **1.05** | 13 | 14.6 | 352 | 39.6 | 5.2 | Cell growth and/or maintenance | Cytoplasm | Cytoskeletal protein binding | Cytoplasm |
| **CCT6A** | NP_001753.1 | 908 | **T-complex protein 1 subunit zeta isoform a** | 60.5 | 5 | 33 | 33 | 280 | 4.18E+09 | **1.04** | 84 | 13 | 531 | 58 | 6.7 | Protein metabolism | Cytoplasm;Microtubule | Chaperone activity | Cytoplasm |
| **PRDX5** | NP_036226.1 | 25824 | **peroxiredoxin-5, mitochondrial isoform a precursor** | 57.0 | 3 | 12 | 12 | 83 | 1.73E+09 | **1.04** | 18 | 5.1 | 214 | 22 | 8.6 | Metabolism;Energy pathways | Mitochondrion;Peroxisome;Cytoplasm;Lysosome | Oxidoreductase activity | Mitochondrion |
| **SRRM1** | NP_005830.2 | 10250 | **serine/arginine repetitive matrix protein 1** | 6.4 | 1 | 3 | 3 | 14 | 1.22E+09 | **1.04** | 2 | 29.3 | 904 | 102.3 | 11.8 | Regulation of nucleobase, nucleoside, nucleotide and nucleic acid metabolism | Nucleus;Cytoplasm | Ribonuclease activity | Nucleus |
| **UBR4** | NP_065816.2 | 23352 | **E3 ubiquitin-protein ligase UBR4** | 13.0 | 1 | 35 | 35 | 70 | 2.12E+08 | **1.04** | 27 | 34.3 | 5183 | 573.5 | 6.0 | Biological_process unknown | Cytoplasm;Nucleus | Molecular function unknown | Cytoplasm |
| **APOBEC3C** | NP_055323.2 | 27350 | **DNA dC->dU-editing enzyme APOBEC-3C** | 6.8 | 1 | 1 | 1 | 3 | 7.51E+06 | **1.04** | 2 | 8 | 190 | 22.8 | 7.6 | Metabolism;Energy pathways | - | Deaminase activity | - |
| **PRRC2B** | NP_037450.2 | 84726 | **protein PRRC2B** | 4.9 | 1 | 4 | 5 | 9 | 8.40E+07 | **1.04** | 4 | 16.5 | 2229 | 242.8 | 8.3 | Biological_process unknown | - | Molecular function unknown | - |
| **WDR45B** | NP_062559.2 | 56270 | **WD repeat domain phosphoinositide-interacting protein 3** | 9.6 | 1 | 2 | 2 | 2 | 2.78E+07 | **1.04** | 2 | 31.9 | 344 | 38.1 | 7.6 | Biological_process unknown | - | Molecular function unknown | - |
| **ABHD11** | NP_683711.1 | 83451 | **alpha/beta hydrolase domain-containing protein 11 isoform 2** | 14.9 | 3 | 2 | 2 | 2 | 4.86E+06 | **1.04** | 1 | - | 308 | 33.8 | 9.3 | Biological_process unknown | - | Molecular function unknown | - |
| **ACIN1** | NP_001158287.1 | 22985 | **apoptotic chromatin condensation inducer in the nucleus isoform 3** | 29.4 | 5 | 25 | 25 | 72 | 5.95E+08 | **1.04** | 25 | 25.5 | 1301 | 147.3 | 6.7 | Regulation of nucleobase, nucleoside, nucleotide and nucleic acid metabolism | Nucleus;Cytoplasm | DNA binding | Nucleus;Cytoplasm |
| **CDC23** | NP_004652.2 | 8697 | **cell division cycle protein 23 homolog** | 13.1 | 1 | 4 | 4 | 7 | 5.87E+07 | **1.04** | 3 | 26.4 | 597 | 68.8 | 7.0 | Cell communication;Signal transduction;Regulation of cell cycle | Nucleus;Centrosome;Chromosome | Binding | Nucleus |
| **NBAS** | NP_056993.2 | 51594 | **neuroblastoma-amplified sequence** | 1.1 | 1 | 2 | 2 | 2 | 1.04E+08 | **1.04** | 2 | 58.7 | 2371 | 268.4 | 6.0 | Biological_process unknown | Mitochondrion | Molecular function unknown | Mitochondrion |
| **COPZ1** | NP_057141.1 | 22818 | **coatomer subunit zeta-1 isoform 1** | 37.3 | 4 | 6 | 6 | 26 | 8.84E+08 | **1.04** | 13 | 14.5 | 177 | 20.2 | 4.8 | Transport | Cytoplasm;Golgi apparatus | Transporter activity | Cytoplasm |
| **RPRD2** | NP_056018.2 | 23248 | **regulation of nuclear pre-mRNA domain-containing protein 2** | 7.0 | 1 | 5 | 5 | 7 | 1.04E+08 | **1.04** | 4 | 5.6 | 1461 | 155.9 | 7.4 | Biological_process unknown | - | Molecular function unknown | - |
| **CLINT1** | NP_001182484.1 | 9685 | **clathrin interactor 1 isoform 1** | 15.6 | 3 | 6 | 6 | 12 | 1.72E+08 | **1.04** | 6 | 16.9 | 643 | 70.2 | 6.6 | Transport | Golgi apparatus;Cytoplasm;Nucleus | Transporter activity | Golgi apparatus;Nucleus;Cytoplasm |

| Gene Symbol | RefSeq_Accession | Entrez Gene ID | Description | Coverage (%) | Proteins | Unique Peptides | # Peptides | PSM | Area | SCC-R /SCC-S | SCC-R /SCC-S (Count) | SCC-R /SCC-S (Variability [%]) | # AAs | MW [kDa] | calc. pI | Biological Process | Localization | Molecular Function | Primary Localization |
|---|---|---|---|---|---|---|---|---|---|---|---|---|---|---|---|---|---|---|---|
| COPB2 | NP_004757.1 | 9276 | coatomer subunit beta' | 40.6 | 1 | 24 | 24 | 64 | 6.85E+08 | 1.04 | 21 | 22.6 | 906 | 102.4 | 5.3 | Transport | Cytoplasm;Golgi apparatus;Cytoplasmic vesicle;Mitochondrion;Nucleus;Cytosol | Transporter activity | Cytoplasm |
| EIF3J | NP_001271265.1 | 8669 | eukaryotic translation initiation factor 3 subunit J isoform 3 | 47.4 | 3 | 10 | 10 | 32 | 1.16E+09 | 1.04 | 13 | 19.6 | 209 | 23 | 4.6 | Protein metabolism | Cytoplasm | Translation regulator activity | Cytoplasm |
| EMC1 | NP_001258358.1 | 23065 | ER membrane protein complex subunit 1 isoform 4 precursor | 20.9 | 4 | 11 | 11 | 30 | 1.95E+08 | 1.04 | 11 | 11.2 | 971 | 109.4 | 7.9 | Biological_process unknown | Mitochondrion | Molecular function unknown | Mitochondrion |
| PSMC3 | NP_002795.2 | 5702 | 26S protease regulatory subunit 6A | 58.8 | 1 | 23 | 23 | 126 | 1.15E+09 | 1.04 | 44 | 19.5 | 439 | 49.2 | 5.2 | Protein metabolism | Nucleus | Ubiquitin-specific protease activity | Nucleus |
| ARL3 | NP_004302.1 | 403 | ADP-ribosylation factor-like protein 3 | 53.3 | 1 | 7 | 7 | 16 | 1.87E+08 | 1.04 | 6 | 12.4 | 182 | 20.4 | 7.2 | Cell communication;Signal transduction | Cytoplasm;Nucleus | GTPase activity | Cytoplasm |
| PSIP1 | NP_001121689.1 | 11168 | PC4 and SFRS1-interacting protein isoform 2 | 31.5 | 2 | 11 | 11 | 41 | 6.95E+08 | 1.04 | 12 | 16.4 | 530 | 60.1 | 9.1 | Regulation of nucleobase, nucleoside, nucleotide and nucleic acid metabolism | Nucleus;Nucleoplasm;Cytoplasm | Transcription regulator activity | Nucleus;Cytoplasm |
| UBE2K | NP_005330.1 | 3093 | ubiquitin-conjugating enzyme E2 K isoform 1 | 62.5 | 3 | 9 | 9 | 32 | 5.00E+08 | 1.04 | 8 | 19.8 | 200 | 22.4 | 5.4 | Protein metabolism | Cytoplasm | Ubiquitin-specific protease activity | Cytoplasm |
| AP1B1 | NP_001118.3 | 162 | AP-1 complex subunit beta-1 isoform a | 35.9 | 3 | 15 | 28 | 105 | 8.30E+08 | 1.04 | 20 | 35.1 | 949 | 104.5 | 5.1 | Transport | Golgi apparatus | Transporter activity | Golgi apparatus |
| PSMD10 | NP_002805.1 | 5716 | 26S proteasome non-ATPase regulatory subunit 10 isoform 1 | 23.5 | 2 | 3 | 3 | 14 | 1.35E+08 | 1.04 | 5 | 5.2 | 226 | 24.4 | 6.1 | Protein metabolism | Cytoplasm;Nucleus | Ubiquitin-specific protease activity | Cytoplasm |
| STX5 | NP_001231595.1 | 6811 | syntaxin-5 isoform 2 | 14.3 | 2 | 2 | 2 | 4 | 1.28E+07 | 1.04 | 1 | - | 321 | 35.5 | 9.4 | Transport | Golgi apparatus;Endoplasmic reticulum | Transporter activity | Golgi apparatus |
| HMGB1 | NP_002119.1 | 3146 | high mobility group protein B1 | 39.5 | 1 | 14 | 14 | 183 | 3.33E+09 | 1.04 | 33 | 18.7 | 215 | 24.9 | 5.7 | Regulation of nucleobase, nucleoside, nucleotide and nucleic acid metabolism | Nucleus;Cytoplasm;Extracellular;Plasma membrane;Mitochondrion | DNA binding | Nucleus |
| ADD3 | NP_001112.2 | 120 | gamma-adducin isoform b | 10.8 | 2 | 2 | 2 | 2 | 1.43E+08 | 1.04 | 1 | - | 674 | 75.6 | 6.8 | Cell growth and/or maintenance | Plasma membrane;Nucleus;Cytoplasm | Cytoskeletal protein binding | Plasma membrane;Nucleus;Cytoplasm |
| COG7 | NP_705831.1 | 91949 | conserved oligomeric Golgi complex subunit 7 | 3.9 | 1 | 2 | 2 | 2 | 9.36E+07 | 1.03 | 2 | 5.5 | 770 | 86.3 | 5.5 | Transport | Golgi apparatus;Cytoplasm | Structural molecule activity | Golgi apparatus |
| DOCK1 | NP_001371.1 | 1793 | dedicator of cytokinesis protein 1 isoform 2 | 5.7 | 2 | 5 | 5 | 5 | 7.65E+07 | 1.03 | 2 | 12.8 | 1865 | 215.2 | 7.6 | Cell communication;Signal transduction | Cytoplasm;Plasma membrane | Guanyl-nucleotide exchange factor activity | Cytoplasm |
| MAD1L1 | NP_001013859.1 | 8379 | mitotic spindle assembly checkpoint protein MAD1 | 16.4 | 1 | 7 | 7 | 8 | 3.08E+07 | 1.03 | 2 | 27.3 | 718 | 83 | 5.9 | Cell communication;Signal transduction;Regulation of cell cycle | Kinetochore;Nucleus;Centrosome | Molecular function unknown;Transcription regulator activity | Nucleus |
| PPP2CA | NP_002706.1 | 5515 | serine/threonine-protein phosphatase 2A catalytic subunit alpha isoform | 56.0 | 1 | 3 | 13 | 47 | 4.97E+08 | 1.03 | 3 | 22.1 | 309 | 35.6 | 5.5 | Cell communication;Signal transduction | Cytoplasm;Chromosome;Mitochondrion | Protein serine/threonine phosphatase activity | Cytoplasm |
| USP5 | NP_003472.2 | 8078 | ubiquitin carboxyl-terminal hydrolase 5 isoform 2 | 60.6 | 2 | 36 | 36 | 164 | 1.05E+09 | 1.03 | 49 | 25.5 | 835 | 93.2 | 5.1 | Protein metabolism | Extracellular | Ubiquitin-specific protease activity | Extracellular |
| HARS | NP_001244970.1 | 3035 | histidine--tRNA ligase, cytoplasmic isoform 3 | 39.3 | 7 | 15 | 19 | 51 | 9.07E+08 | 1.03 | 15 | 7 | 489 | 54.8 | 5.5 | Protein metabolism | Cytoplasm;Nucleus | Ligase activity | Cytoplasm |
| HNRNPA3 | NP_919223.1 | 220988 | heterogeneous nuclear ribonucleoprotein A3 | 48.4 | 1 | 28 | 30 | 162 | 3.06E+09 | 1.03 | 65 | 18.5 | 378 | 39.6 | 9.0 | Regulation of nucleobase, nucleoside, nucleotide and nucleic acid metabolism | Nucleus;Cytoplasm | RNA binding | Nucleus |
| TARDBP | NP_031401.1 | 23435 | TAR DNA-binding protein 43 | 41.6 | 1 | 14 | 14 | 69 | 5.37E+08 | 1.03 | 21 | 23.3 | 414 | 44.7 | 6.2 | Regulation of nucleobase, nucleoside, nucleotide and nucleic acid metabolism | Nucleus | DNA binding | Nucleus |
| CCT2 | NP_006422.1 | 10576 | T-complex protein 1 subunit beta isoform 1 | 70.8 | 2 | 34 | 34 | 334 | 3.20E+09 | 1.03 | 72 | 12.9 | 535 | 57.5 | 6.5 | Protein metabolism | Cytoplasm;Nucleolus;Microtubule;Mitochondrion | Chaperone activity | Cytoplasm |
| HDAC3 | NP_003874.2 | 8841 | histone deacetylase 3 | 9.4 | 1 | 2 | 2 | 2 | 4.07E+07 | 1.03 | 1 | - | 428 | 48.8 | 5.2 | Regulation of nucleobase, nucleoside, nucleotide and nucleic acid metabolism | Nucleus;Cytoplasm | Transcription regulator activity | Nucleus |
| BAG6 | NP_001092004.1 | 7917 | large proline-rich protein BAG6 isoform b | 21.9 | 4 | 13 | 13 | 22 | 1.80E+08 | 1.03 | 9 | 11.7 | 1126 | 118.6 | 5.6 | Apoptosis | Nucleus;Cytoplasm;Cytosol | Molecular function unknown | Nucleus |
| CKAP2 | NP_001273615.1 | 26586 | cytoskeleton-associated protein 2 isoform 3 | 3.0 | 4 | 2 | 2 | 4 | 9.63E+07 | 1.03 | 1 | - | 634 | 71.2 | 9.3 | Cell growth and/or maintenance | Cytoskeleton;Microtubule | Cytoskeletal protein binding | Cytoskeleton |
| AGFG1 | NP_001128661.1 | 3267 | arf-GAP domain and FG repeat-containing protein 1 isoform 4 | 22.4 | 4 | 5 | 5 | 12 | 1.18E+08 | 1.03 | 5 | 8.2 | 522 | 54.1 | 8.9 | Regulation of nucleobase, nucleoside, nucleotide and nucleic acid metabolism | Nucleus;Cytoplasm;Nucleolus | RNA binding | Nucleus |
| ETFB | NP_001976.1 | 2109 | electron transfer flavoprotein subunit beta isoform 1 | 49.4 | 2 | 10 | 10 | 27 | 3.30E+08 | 1.03 | 5 | 25.7 | 255 | 27.8 | 8.1 | Metabolism;Energy pathways | Mitochondrion | Oxidoreductase activity | Mitochondrion |
| HP1BP3 | NP_057371.2 | 50809 | heterochromatin protein 1-binding protein 3 | 14.7 | 1 | 6 | 6 | 14 | 2.15E+08 | 1.03 | 5 | 6.1 | 553 | 61.2 | 9.7 | Regulation of nucleobase, nucleoside, nucleotide and nucleic acid metabolism | Nucleus | DNA binding | Nucleus |
| PSMD9 | NP_002804.2 | 5715 | 26S proteasome non-ATPase regulatory subunit 9 isoform 1 | 41.7 | 2 | 9 | 9 | 19 | 1.83E+08 | 1.03 | 7 | 35.7 | 223 | 24.7 | 7.0 | Protein metabolism | Cytoplasm;Nucleus | Ubiquitin-specific protease activity | Cytoplasm |
| CPSF7 | NP_001136037.1 | 79869 | cleavage and polyadenylation specificity factor subunit 7 isoform 3 | 18.4 | 3 | 5 | 5 | 21 | 2.86E+08 | 1.03 | 6 | 23.7 | 462 | 51.1 | 7.8 | Biological_process unknown | - | Molecular function unknown | - |
| PAF1 | NP_001243755.1 | 54623 | RNA polymerase II-associated factor 1 homolog isoform 2 | 24.5 | 2 | 6 | 6 | 19 | 1.11E+08 | 1.03 | 8 | 11.6 | 485 | 55.4 | 5.4 | Biological_process unknown;Cell homeostasis;Transcription;Cell development;Cell differentiation;Regulation of metabolism;Drug | Nucleus | Molecular function unknown | Nucleus |
| RICTOR | NP_001272369.1 | 253260 | rapamycin-insensitive companion of mTOR isoform 3 | 1.7 | 3 | 1 | 1 | 2 | 1.25E+08 | 1.03 | 1 | - | 1385 | 155.8 | 7.2 | Biological_process unknown | Nucleus;Myosin;Cytoplasm | Molecular function unknown | Nucleus |
| TST | NP_001257412.1 | 7263 | thiosulfate sulfurtransferase | 25.3 | 1 | 5 | 5 | 11 | 7.36E+07 | 1.03 | 7 | 38.9 | 297 | 33.4 | 7.3 | Metabolism;Energy pathways | Mitochondrion;Cytoplasm | Sulfotransferase activity | Mitochondrion |
| CSK | NP_001120662.1 | 1445 | tyrosine-protein kinase CSK | 7.8 | 1 | 2 | 2 | 3 | 6.91E+07 | 1.03 | 2 | 49.5 | 450 | 50.7 | 7.1 | Cell communication;Signal transduction | Cytoplasm;Plasma membrane;Early endosome;Late endosome | Protein-tyrosine kinase activity | Cytoplasm |
| DCTN3 | NP_001268354.1 | 11258 | dynactin subunit 3 isoform 3 | 27.2 | 5 | 3 | 3 | 4 | 7.15E+07 | 1.03 | 3 | 25.4 | 158 | 18 | 6.1 | Cell communication;Signal transduction;Cell cycle | Centrosome;Nucleus;Cytoplasm;Microtubule;Kinetochore | Cytoskeletal protein binding | Centrosome |
| PPID | NP_005029.1 | 5481 | peptidyl-prolyl cis-trans isomerase D | 29.2 | 1 | 7 | 7 | 13 | 7.57E+07 | 1.03 | 7 | 33.1 | 370 | 40.7 | 7.2 | Metabolism;Energy pathways | Nucleolus;Nucleus;Cytoplasm | Isomerase activity | Nucleolus |
| PSPH | NP_004568.2 | 5723 | phosphoserine phosphatase | 53.3 | 1 | 8 | 8 | 13 | 1.36E+08 | 1.03 | 5 | 40.1 | 225 | 25 | 5.7 | Metabolism;Energy pathways | Cytoplasm | Protein serine/threonine phosphatase activity | Cytoplasm |
| CCT7 | NP_006420.1 | 10574 | T-complex protein 1 subunit eta isoform a | 75.7 | 4 | 34 | 34 | 286 | 1.93E+09 | 1.03 | 73 | 20.3 | 543 | 59.3 | 7.7 | Protein folding | Cytoplasm;Microtubule;Mitochondrion | Chaperone activity | Cytoplasm |

| Gene Symbol | RefSeq_Accession | Entrez Gene ID | Description | Coverage (%) | Proteins | Unique Peptides | # Peptides | PSM | Area | SCC-R /SCC-S | SCC-R /SCC-S (Count) | SCC-R /SCC-S (Variability [%]) | # AAs | MW [kDa] | calc. pI | Biological Process | Localization | Molecular Function | Primary Localization |
|---|---|---|---|---|---|---|---|---|---|---|---|---|---|---|---|---|---|---|---|
| **LLPH** | NP_115714.1 | 84298 | **protein LLP homolog** | 32.6 | 1 | 4 | 4 | 5 | 6.47E+07 | **1.03** | 3 | 9.4 | 129 | 15.2 | 10.4 | Biological_process unknown | - | Molecular function unknown | - |
| **RAVER1** | NP_597709.2 | 125950 | **ribonucleoprotein PTB-binding 1** | 50.8 | 1 | 18 | 18 | 51 | 7.01E+08 | **1.03** | 23 | 32.8 | 756 | 79.5 | 8.9 | Biological_process unknown | Nucleus;Cytoplasm | Cytoskeletal protein binding | Nucleus |
| **SUPT16H** | NP_009123.1 | 11198 | **FACT complex subunit SPT16** | 42.3 | 1 | 36 | 36 | 114 | 9.69E+08 | **1.03** | 42 | 17.2 | 1047 | 119.8 | 5.7 | Regulation of nucleobase, nucleoside, nucleotide and nucleic acid metabolism | Nucleus;Cytoplasm | Transcription factor activity | Nucleus |
| **APOL2** | NP_112092.1 | 23780 | **apolipoprotein L2** | 25.8 | 1 | 7 | 7 | 26 | 1.46E+08 | **1.03** | 3 | 6.5 | 337 | 37.1 | 6.7 | Metabolism;Energy pathways;Biological_process unknown | - | Transporter activity;Molecular function unknown | - |
| **NAP1L1** | NP_631946.1 | 4673 | **nucleosome assembly protein 1-like 1** | 47.1 | 1 | 15 | 17 | 135 | 2.12E+09 | **1.03** | 28 | 24.9 | 391 | 45.3 | 4.5 | Regulation of nucleobase, nucleoside, nucleotide and nucleic acid metabolism | Nucleus;Mitochondrion | DNA binding | Nucleus |
| **NDUFC2** | NP_004540.1 | 4718 | **NADH dehydrogenase [ubiquinone] 1 subunit C2 isoform 1** | 29.4 | 5 | 3 | 3 | 4 | 3.23E+07 | **1.03** | 3 | 2.6 | 119 | 14.2 | 9.0 | Metabolism;Energy pathways | Mitochondrion | Oxidoreductase activity | Mitochondrion |
| **NUP93** | NP_055484.3 | 9688 | **nuclear pore complex protein Nup93 isoform 1** | 40.4 | 2 | 18 | 18 | 43 | 4.30E+08 | **1.03** | 12 | 36.2 | 819 | 93.4 | 5.7 | Transport | Nucleus;Mitochondrion;Nuclear membrane | Auxiliary transport protein activity | Nucleus |
| **ALDH6A1** | NP_001265523.1 | 4329 | **methylmalonate-semialdehyde dehydrogenase [acylating], mitochondrial isoform 3** | 32.8 | 3 | 5 | 5 | 13 | 1.30E+08 | **1.02** | 1 | - | 381 | 41.1 | 7.7 | Metabolism;Energy pathways | Mitochondrion;Extracellular | Catalytic activity | Mitochondrion |
| **NCLN** | NP_064555.2 | 56926 | **nicalin precursor** | 12.1 | 1 | 5 | 5 | 8 | 5.52E+07 | **1.02** | 2 | 80.5 | 563 | 62.9 | 6.9 | Protein metabolism | Plasma membrane | Peptidase activity | Plasma membrane |
| **RAE1** | NP_001015885.1 | 8480 | **mRNA export factor** | 54.1 | 1 | 12 | 12 | 28 | 3.98E+08 | **1.02** | 14 | 18.7 | 368 | 40.9 | 7.8 | Regulation of nucleobase, nucleoside, nucleotide and nucleic acid metabolism | Nucleus;Cytoplasm;Cytoskeleton;Microtubule;Mitochondrion | RNA binding | Nucleus |
| **RNF7** | NP_001188299.1 | 9616 | **RING-box protein 2 isoform 4** | 35.1 | 3 | 2 | 2 | 6 | 2.29E+07 | **1.02** | 1 | - | 97 | 10.9 | 6.3 | Anti-apoptosis | Nucleus;Cytoplasm | Ligase activity | Nucleus |
| **SORD** | NP_003095.2 | 6652 | **sorbitol dehydrogenase** | 35.0 | 1 | 7 | 7 | 14 | 9.63E+07 | **1.02** | 3 | 28.2 | 357 | 38.3 | 8.0 | Metabolism;Energy pathways | Plasma membrane;Cytoplasm | Catalytic activity | Plasma membrane |
| **FRMPD3** | NP_115804.1 | 84443 | **FERM and PDZ domain-containing protein 3** | 0.6 | 1 | 1 | 1 | 2 | 5.75E+08 | **1.02** | 1 | - | 1810 | 199.1 | 8.2 | - | - | - | - |
| **TXN2** | NP_036605.2 | 25828 | **thioredoxin, mitochondrial precursor** | 33.1 | 1 | 3 | 3 | 8 | 4.49E+07 | **1.02** | 1 | - | 166 | 18.4 | 8.3 | Metabolism;Energy pathways | Mitochondrion | Oxidoreductase activity | Mitochondrion |
| **ACOT13** | NP_001153566.1 | 55856 | **acyl-coenzyme A thioesterase 13 isoform 2** | 42.7 | 2 | 3 | 3 | 4 | 2.35E+08 | **1.02** | 3 | 32.5 | 117 | 12.4 | 8.5 | Metabolism;Energy pathways | - | Hydrolase activity | - |
| **CYTB** | YP_003024038.1 | 4519 | **cytochrome b** | 2.4 | 1 | 1 | 1 | 1 | 6.36E+07 | **1.02** | 1 | - | 380 | 42.7 | 8.1 | Metabolism;Energy pathways | Mitochondrion | Oxidoreductase activity | Mitochondrion |
| **GAPDH** | NP_002037.2 | 2597 | **glyceraldehyde-3-phosphate dehydrogenase isoform 1** | 92.5 | 2 | 38 | 39 | 3595 | 4.82E+10 | **1.02** | 459 | 18.2 | 335 | 36 | 8.5 | Metabolism;Energy pathways | Cytoplasm;Nucleus;Nucleolus;Extracellular;Mitochondrion | Catalytic activity | Cytoplasm |
| **CEBPD** | NP_005186.2 | 1052 | **CCAAT/enhancer-binding protein delta** | 9.7 | 1 | 1 | 1 | 1 | 7.39E+07 | **1.02** | 1 | - | 269 | 28.4 | 8.3 | Regulation of nucleobase, nucleoside, nucleotide and nucleic acid metabolism | Nucleus | Transcription factor activity | Nucleus |
| **GNB2** | NP_005264.2 | 2783 | **guanine nucleotide-binding protein G(I)/G(S)/G(T) subunit beta-2** | 77.9 | 2 | 9 | 16 | 92 | 7.15E+08 | **1.02** | 11 | 26 | 340 | 37.3 | 6.0 | Cell communication;Signal transduction | Cytoplasm | GTPase activity | Cytoplasm |
| **NAA10** | NP_003482.1 | 8260 | **N-alpha-acetyltransferase 10 isoform 1** | 62.1 | 4 | 8 | 8 | 18 | 2.60E+08 | **1.02** | 5 | 32.4 | 235 | 26.4 | 5.6 | Metabolism;Energy pathways | Cytoplasm | Acyltransferase activity | Cytoplasm |
| **NIT1** | NP_001172023.1 | 4817 | **nitrilase homolog 1 isoform 4** | 13.1 | 4 | 2 | 2 | 3 | 7.11E+07 | **1.02** | 2 | 69.1 | 291 | 31.8 | 6.9 | Metabolism;Energy pathways | - | Molecular function unknown | - |
| **RPAP1** | NP_056355.2 | 26015 | **RNA polymerase II-associated protein 1** | 4.7 | 1 | 3 | 3 | 6 | 5.85E+07 | **1.02** | 2 | 13.7 | 1393 | 152.7 | 6.4 | Biological_process unknown | - | Molecular function unknown | - |
| **SRSF1** | NP_001071634.1 | 6426 | **serine/arginine-rich splicing factor 1 isoform 2** | 46.8 | 2 | 17 | 17 | 90 | 1.26E+09 | **1.02** | 21 | 15.8 | 201 | 22.4 | 7.9 | Protein metabolism | Nucleus;Cytoplasm;Nucleolus | RNA binding | Nucleus |
| **XRN1** | NP_001269786.1 | 54464 | **5'-3' exoribonuclease 1 isoform c** | 4.7 | 3 | 4 | 4 | 5 | 6.63E+07 | **1.02** | 3 | 6.6 | 1694 | 192.7 | 7.3 | Regulation of nucleobase, nucleoside, nucleotide and nucleic acid metabolism | Cytoplasm | Ribonuclease activity | Cytoplasm |
| **ECSIT** | NP_057665.2 | 51295 | **evolutionarily conserved signaling intermediate in Toll pathway, mitochondrial isoform 1 precursor** | 9.7 | 4 | 2 | 2 | 2 | 2.99E+06 | **1.02** | 1 | - | 431 | 49.1 | 6.3 | Cell communication;Signal transduction | Cytoplasm;Nucleus;Mitochondrion | Receptor signaling complex scaffold activity | Cytoplasm;Mitochondrion |
| **GAA** | NP_001073272.1 | 2548 | **lysosomal alpha-glucosidase preproprotein** | 9.1 | 1 | 5 | 5 | 12 | 2.10E+08 | **1.02** | 4 | 16.7 | 952 | 105.3 | 6.0 | Metabolism;Energy pathways | Lysosome | Glucosidase activity | Lysosome |
| **MRPS12** | NP_066930.1 | 6183 | **28S ribosomal protein S12, mitochondrial precursor** | 18.1 | 1 | 1 | 1 | 1 | 1.28E+07 | **1.02** | 1 | - | 138 | 15.2 | 10.3 | Protein metabolism;Ribosome biogenesis and assembly | Mitochondrion | Structural constituent of ribosome | Mitochondrion |
| **CNIH4** | NP_001264128.1 | 29097 | **protein cornichon homolog 4 isoform 4** | 26.3 | 3 | 1 | 1 | 8 | 1.51E+09 | **1.02** | 3 | 16.6 | 76 | 9 | 8.2 | Biological_process unknown | Integral to membrane | Molecular function unknown | Integral to membrane |
| **MSL3** | NP_006791.2 | 10943 | **male-specific lethal 3 homolog isoform c** | 3.1 | 5 | 1 | 1 | 1 | 4.03E+06 | **1.02** | 1 | - | 355 | 40.6 | 8.9 | Regulation of nucleobase, nucleoside, nucleotide and nucleic acid metabolism | Nucleus;Cytoplasm | Transcription factor activity | Nucleus |
| **EIF4ENIF1** | NP_001157974.1 | 56478 | **eukaryotic translation initiation factor 4E transporter isoform b** | 4.0 | 2 | 1 | 2 | 16 | 5.14E+09 | **1.02** | 1 | - | 811 | 88.2 | 7.4 | Protein metabolism | Perinuclear region;Cytoplasm;Nucleus | Translation regulator activity | Cytoplasm |
| **MED4** | NP_054885.1 | 29079 | **mediator of RNA polymerase II transcription subunit 4 isoform 1** | 10.7 | 1 | 2 | 2 | 3 | 9.58E+06 | **1.02** | 2 | 19.4 | 270 | 29.7 | 5.1 | Cell communication;Signal transduction | Nucleus | Translation regulator activity | Nucleus |
| **AP3D1** | NP_003929.4 | 8943 | **AP-3 complex subunit delta-1 isoform 2** | 24.5 | 2 | 18 | 18 | 62 | 3.68E+08 | **1.02** | 23 | 26.1 | 1153 | 130.1 | 8.5 | Transport | Cytoplasm;Golgi apparatus | Transporter activity | Cytoplasm |
| **CCT3** | NP_005989.3 | 7203 | **T-complex protein 1 subunit gamma isoform a** | 66.2 | 2 | 35 | 35 | 194 | 4.90E+09 | **1.02** | 70 | 17.8 | 545 | 60.5 | 6.5 | Protein metabolism | Cytoplasm;Nucleolus;Microtubule | Chaperone activity | Cytoplasm |
| **EIF2S1** | NP_004085.1 | 1965 | **eukaryotic translation initiation factor 2 subunit 1** | 62.2 | 1 | 22 | 22 | 86 | 3.59E+08 | **1.02** | 29 | 40.9 | 315 | 36.1 | 5.1 | Protein metabolism | Cytoplasm;Ribosome;Nucleolus;Mitochondrion | Translation regulator activity | Cytoplasm |
| **NONO** | NP_001138881.1 | 4841 | **non-POU domain-containing octamer-binding protein isoform 1** | 61.4 | 2 | 26 | 27 | 164 | 1.53E+09 | **1.02** | 50 | 22.9 | 471 | 54.2 | 9.0 | Regulation of nucleobase, nucleoside, nucleotide and nucleic acid metabolism | Nucleus;Nucleolus;Mitochondrion | RNA binding | Nucleus |
| **SEC24A** | NP_068817.1 | 10802 | **protein transport protein Sec24A isoform 1** | 5.9 | 2 | 4 | 4 | 8 | 4.86E+07 | **1.02** | 5 | 18.1 | 1093 | 119.7 | 7.7 | Transport | Endoplasmic reticulum | Transporter activity | Endoplasmic reticulum |
| **SNRPB2** | NP_003083.1 | 6629 | **U2 small nuclear ribonucleoprotein B''** | 42.2 | 1 | 5 | 7 | 15 | 1.67E+08 | **1.02** | 6 | 23.4 | 225 | 25.5 | 9.7 | RNA metabolism | Nucleus;Cytoplasm | RNA binding | Nucleus |

| Gene Symbol | RefSeq_Accession | Entrez Gene ID | Description | Coverage (%) | Proteins | Unique Peptides | # Peptides | PSM | Area | SCC-R /SCC-S | SCC-R /SCC-S (Count) | SCC-R /SCC-S (Variability [%]) | # AAs | MW [kDa] | calc. pI | Biological Process | Localization | Molecular Function | Primary Localization |
|---|---|---|---|---|---|---|---|---|---|---|---|---|---|---|---|---|---|---|---|
| **TNPO1** | NP_694858.1 | 3842 | **transportin-1 isoform 2** | 40.6 | 2 | 18 | 23 | 91 | 8.87E+08 | **1.02** | 22 | 21.7 | 890 | 101.2 | 5.0 | Transport | Cytoplasm;Nuclear membrane;Centrosome | Transporter activity | Cytoplasm |
| **CRELD1** | NP_001070883.1 | 78987 | **cysteine-rich with EGF-like domain protein 1 isoform 2 precursor** | 1.7 | 1 | 1 | 1 | 1 | 2.34E+06 | **1.02** | 1 | - | 420 | 45.4 | 4.9 | Cell development | Plasma membrane;Extracellular | Cell adhesion molecule activity | Plasma membrane |
| **OTUD7B** | NP_064590.2 | 56957 | **OTU domain-containing protein 7B** | 1.9 | 1 | 1 | 1 | 3 | 3.05E+07 | **1.02** | 2 | 2.6 | 843 | 92.5 | 6.7 | Protein metabolism | Cytoplasm | Ubiquitin-specific protease activity | Cytoplasm |
| **PMM2** | NP_000294.1 | 5373 | **phosphomannomutase 2** | 13.4 | 1 | 2 | 2 | 3 | 4.14E+07 | **1.02** | 2 | 16.7 | 246 | 28.1 | 6.8 | Metabolism;Energy pathways | Cytoplasm;Nucleus | Catalytic activity | Cytoplasm |
| **RIC8A** | NP_001273063.1 | 60626 | **synembryn-A isoform 2** | 27.3 | 3 | 9 | 9 | 22 | 1.96E+08 | **1.02** | 9 | 22.4 | 531 | 59.7 | 5.3 | Cell communication;Signal transduction | Cytoplasm;Plasma membrane | Guanyl-nucleotide exchange factor activity | Cytoplasm |
| **SYNRG** | NP_001157019.1 | 11276 | **synergin gamma isoform 7** | 5.1 | 7 | 2 | 2 | 2 | 2.25E+07 | **1.02** | 1 | - | 1108 | 119.4 | 5.1 | Transport | Plasma membrane;Golgi apparatus | Transporter activity | Golgi apparatus |
| **FNBP1L** | NP_060207.2 | 54874 | **formin-binding protein 1-like isoform 2** | 3.7 | 3 | 1 | 1 | 2 | 6.12E+07 | **1.02** | 1 | - | 547 | 63.5 | 6.2 | Signal transduction | Microtubule;Nucleus | Cytoskeletal protein binding | Microtubule |
| **HSD17B10** | NP_004484.1 | 3028 | **3-hydroxyacyl-CoA dehydrogenase type-2 isoform 1** | 90.0 | 2 | 19 | 19 | 199 | 1.41E+09 | **1.02** | 39 | 21.6 | 261 | 26.9 | 7.8 | Metabolism;Energy pathways | Mitochondrion;Endoplasmic reticulum;Plasma membrane | Catalytic activity | Mitochondrion |
| **NCBP1** | NP_002477.1 | 4686 | **nuclear cap-binding protein subunit 1** | 23.8 | 1 | 12 | 12 | 26 | 1.59E+08 | **1.02** | 9 | 42.7 | 790 | 91.8 | 6.4 | Regulation of nucleobase, nucleoside, nucleotide and nucleic acid metabolism | Nucleus;Cytoplasm | RNA binding | Nucleus |
| **NT5DC1** | NP_689942.2 | 221294 | **5'-nucleotidase domain-containing protein 1** | 42.6 | 1 | 11 | 11 | 20 | 1.62E+08 | **1.02** | 10 | 39.3 | 455 | 51.8 | 6.4 | Biological_process unknown | Cytoplasm | Molecular function unknown | Cytoplasm |
| **FRYL** | NP_055845.1 | 285527 | **protein furry homolog-like** | 2.6 | 1 | 4 | 4 | 5 | 2.73E+07 | **1.01** | 1 | - | 3013 | 339.4 | 5.6 | Biological_process unknown | - | Molecular function unknown | - |
| **TATDN1** | NP_001139632.1 | 83940 | **putative deoxyribonuclease TATDN1 isoform b** | 27.6 | 2 | 4 | 4 | 5 | 1.44E+08 | **1.01** | 1 | - | 250 | 28.1 | 6.5 | Biological_process unknown | - | Molecular function unknown | - |
| **ASMTL** | NP_001166944.1 | 8623 | **N-acetylserotonin O-methyltransferase-like protein isoform 2** | 4.1 | 3 | 1 | 1 | 2 | 9.55E+07 | **1.01** | 2 | 20.7 | 563 | 62.4 | 5.6 | Metabolism;Energy pathways | Cytoplasm | Acyltransferase activity | Cytoplasm |
| **EXOSC1** | NP_057130.1 | 51013 | **exosome complex component CSL4** | 15.4 | 1 | 2 | 2 | 4 | 3.46E+07 | **1.01** | 1 | - | 195 | 21.4 | 8.2 | Regulation of nucleobase, nucleoside, nucleotide and nucleic acid metabolism | Nucleus;Cytoplasm;Nucleolus | Ribonuclease activity | Nucleus |
| **PPCS** | NP_078940.2 | 79717 | **phosphopantothenate--cysteine ligase isoform a** | 22.8 | 4 | 5 | 5 | 9 | 1.18E+08 | **1.01** | 4 | 24.6 | 311 | 34 | 6.7 | Metabolism | - | Ligase activity | - |
| **CALM3** | NP_005175.2 | 808 | **calmodulin** | 83.9 | 3 | 9 | 9 | 61 | 1.68E+09 | **1.01** | 19 | 13.3 | 149 | 16.8 | 4.2 | Cell communication;Signal transduction | Cytoplasm;Nucleus;Plasma membrane | Calcium ion binding | Cytoplasm |
| **GINS3** | NP_001119602.1 | 64785 | **DNA replication complex GINS protein PSF3 isoform c** | 20.3 | 3 | 1 | 1 | 2 | 1.04E+08 | **1.01** | 1 | - | 138 | 15.6 | 4.9 | Biological_process unknown | - | Molecular function unknown | - |
| **WARS2** | NP_056651.1 | 10352 | **tryptophan--tRNA ligase, mitochondrial isoform 1 precursor** | 18.9 | 2 | 3 | 3 | 3 | 1.31E+07 | **1.01** | 2 | 8.8 | 360 | 40.1 | 9.3 | Protein metabolism | Mitochondrion;Nucleus;Cytoplasm | Translation regulator activity;TRNA ligase activity | Mitochondrion |
| **ZYG11B** | NP_078922.1 | 79699 | **protein zyg-11 homolog B** | 2.8 | 1 | 2 | 2 | 7 | 9.26E+08 | **1.01** | 2 | 34.7 | 744 | 83.9 | 6.9 | Biological_process unknown | - | Molecular function unknown | - |
| **ACTR10** | NP_060947.1 | 55860 | **actin-related protein 10** | 12.5 | 1 | 3 | 3 | 3 | 4.90E+07 | **1.01** | 3 | 63.7 | 417 | 46.3 | 7.4 | Cell growth and/or maintenance | Cytoskeleton;Cytoplasm;Centrosome;Kinetochore;Microtubule | Cytoskeletal protein binding | Cytoskeleton |
| **C20orf24** | NP_061328.1 | 55969 | **uncharacterized protein C20orf24 isoform a** | 11.6 | 3 | 1 | 1 | 1 | 4.11E+07 | **1.01** | 1 | - | 129 | 14.8 | 4.8 | Biological_process unknown | - | Molecular function unknown | - |
| **DAD1** | NP_001335.1 | 1603 | **dolichyl-diphosphooligosaccharide--protein glycosyltransferase subunit DAD1** | 10.6 | 1 | 1 | 1 | 2 | 6.20E+06 | **1.01** | 1 | - | 113 | 12.5 | 7.1 | Apoptosis | Endoplasmic reticulum | Receptor signaling complex scaffold activity | Endoplasmic reticulum |
| **GFPT1** | NP_002047.2 | 2673 | **glutamine--fructose-6-phosphate aminotransferase [isomerizing] 1 isoform 2** | 33.6 | 3 | 16 | 16 | 32 | 2.15E+08 | **1.01** | 18 | 24.5 | 681 | 76.7 | 6.8 | Metabolism;Energy pathways | Cytoplasm | Transaminase activity | Cytoplasm |
| **EEF1A1** | NP_001393.1 | 1915 | **elongation factor 1-alpha 1** | 82.0 | 2 | 41 | 41 | 1604 | 2.45E+10 | **1.01** | 275 | 30.9 | 462 | 50.1 | 9.0 | Regulation of cell cycle | Cytoplasm;Nucleus;Nucleolus;Mitochondrion;Ribosome | Transcription regulator activity | Cytoplasm |
| **EXOSC2** | NP_055100.2 | 23404 | **exosome complex component RRP4 isoform 1** | 34.5 | 3 | 6 | 6 | 15 | 1.10E+08 | **1.01** | 3 | 23.4 | 293 | 32.8 | 7.5 | Regulation of nucleobase, nucleoside, nucleotide and nucleic acid metabolism | Nucleus;Cytoplasm;Nucleolus | Ribonuclease activity | Nucleus |
| **MZT1** | NP_001065243.1 | 440145 | **mitotic-spindle organizing protein 1** | 23.2 | 1 | 1 | 1 | 2 | 8.04E+07 | **1.01** | 1 | - | 82 | 8.5 | 4.8 | Biological_process unknown | - | Molecular function unknown | - |
| **NUP50** | NP_705931.1 | 10762 | **nuclear pore complex protein Nup50 isoform a** | 39.6 | 2 | 10 | 10 | 23 | 1.91E+08 | **1.01** | 9 | 19.9 | 440 | 46.8 | 8.3 | Transport | Nucleoplasm;Cytoplasm;Nucleus | Transporter activity | Nucleoplasm |
| **PACSIN2** | NP_001171900.1 | 11252 | **protein kinase C and casein kinase substrate in neurons protein 2 isoform B** | 21.6 | 2 | 6 | 6 | 14 | 9.57E+07 | **1.01** | 7 | 9.1 | 445 | 51.3 | 5.4 | Cell communication;Signal transduction | Cytoplasm | Receptor signaling complex scaffold activity | Cytoplasm |
| **UBE3C** | NP_055486.2 | 9690 | **ubiquitin-protein ligase E3C** | 6.8 | 1 | 4 | 4 | 7 | 8.18E+07 | **1.01** | 4 | 23.6 | 1083 | 123.8 | 6.7 | Protein metabolism | - | Ubiquitin-specific protease activity | - |
| **ZFAND6** | NP_001229847.1 | 54469 | **AN1-type zinc finger protein 6 isoform c** | 5.9 | 4 | 1 | 1 | 1 | 9.69E+06 | **1.01** | 1 | - | 170 | 18.3 | 5.5 | Cell communication;Signal transduction | Cytoplasm | Receptor signaling complex scaffold activity | Cytoplasm |
| **CERS2** | NP_859530.1 | 29956 | **ceramide synthase 2** | 14.5 | 1 | 4 | 4 | 10 | 2.49E+08 | **1.01** | 5 | 24.2 | 380 | 44.8 | 9.0 | Regulation of cell growth | Integral to membrane | Molecular function unknown | Integral to membrane |
| **TET1** | NP_085128.2 | 80312 | **methylcytosine dioxygenase TET1** | 0.4 | 1 | 1 | 1 | 7 | 7.44E+08 | **1.01** | 2 | 1.3 | 2136 | 235.2 | 8.2 | Regulation of nucleobase, nucleoside, nucleotide and nucleic acid metabolism | Nucleus | DNA binding | Nucleus |
| **PDLIM5** | NP_006448.4 | 10611 | **PDZ and LIM domain protein 5 isoform a** | 42.0 | 8 | 18 | 18 | 54 | 4.89E+08 | **1.01** | 23 | 22.5 | 596 | 63.9 | 8.2 | Cell communication;Signal transduction | Cytoplasm;Plasma membrane;Nucleus | Receptor signaling complex scaffold activity | Cytoplasm |
| **SPIN1** | NP_006708.2 | 10927 | **spindlin-1** | 16.0 | 1 | 2 | 2 | 3 | 7.30E+07 | **1.01** | 1 | - | 262 | 29.6 | 7.0 | Biological_process unknown | Cytoplasm | Molecular function unknown | Cytoplasm |
| **PPIL1** | NP_057143.1 | 51645 | **peptidyl-prolyl cis-trans isomerase-like 1** | 57.2 | 1 | 5 | 6 | 20 | 3.79E+08 | **1.01** | 5 | 3.6 | 166 | 18.2 | 8.0 | Metabolism;Energy pathways | Cytoplasm | Isomerase activity | Cytoplasm |
| **TM9SF3** | NP_064508.3 | 56889 | **transmembrane 9 superfamily member 3 precursor** | 18.5 | 1 | 8 | 8 | 19 | 2.82E+08 | **1.01** | 10 | 24.1 | 589 | 67.8 | 7.2 | Transport | Plasma membrane | Auxiliary transport protein activity | Plasma membrane |
| **ARFGAP1** | NP_060679.1 | 55738 | **ADP-ribosylation factor GTPase-activating protein 1 isoform a** | 38.4 | 5 | 8 | 8 | 12 | 1.07E+08 | **1.01** | 5 | 25.3 | 406 | 44.6 | 5.7 | Lipid metabolism;Protein transport | Golgi apparatus;Cytoplasm | GTPase activator activity | Golgi apparatus |

Supplementary Table S4. List of all proteins identified in SCC-S and SCC-R cells using SequestHT and Mascot search algorithms and their gene ontology annotation

| Gene Symbol | RefSeq_Accession | Entrez Gene ID | Description | Coverage (%) | Proteins | Unique Peptides | # Peptides | PSM | Area | SCC-R /SCC-S | SCC-R /SCC-S (Count) | SCC-R /SCC-S (Variability [%]) | # AAs | MW [kDa] | calc. pI | Biological Process | Localization | Molecular Function | Primary Localization |
|---|---|---|---|---|---|---|---|---|---|---|---|---|---|---|---|---|---|---|---|
| **GFM1** | NP_079272.4 | 85476 | **elongation factor G, mitochondrial** | 21.6 | 1 | 8 | 8 | 22 | 3.63E+08 | **1.00** | 10 | 18.4 | 751 | 83.4 | 7.0 | Protein metabolism | Mitochondrion | Translation regulator activity | Mitochondrion |
| **TOE1** | NP_079353.3 | 114034 | **target of EGR1 protein 1** | 15.9 | 1 | 4 | 4 | 6 | 2.43E+07 | **1.00** | 2 | 3.4 | 510 | 56.5 | 7.2 | Regulation of nucleobase, nucleoside, nucleotide and nucleic acid metabolism | Nucleus;Nucleolus | DNA binding | Nucleolus |
| **PTMS** | NP_002815.3 | 5763 | **parathymosin** | 29.4 | 1 | 6 | 6 | 29 | 6.18E+08 | **1.00** | 5 | 6.7 | 102 | 11.5 | 4.2 | Regulation of nucleobase, nucleoside, nucleotide and nucleic acid metabolism | Nucleus | DNA binding | Nucleus |
| **RALY** | NP_057951.1 | 22913 | **RNA-binding protein Raly isoform 1** | 36.3 | 5 | 7 | 7 | 33 | 6.00E+08 | **1.00** | 12 | 16.1 | 306 | 32.4 | 9.2 | Regulation of nucleobase, nucleoside, nucleotide and nucleic acid metabolism | Mitochondrion;Nucleus;Cytoplasm | RNA binding | Mitochondrion;Nucleus;Cytoplasm |
| **SDHA** | NP_004159.2 | 6389 | **succinate dehydrogenase [ubiquinone] flavoprotein subunit, mitochondrial** | 43.5 | 1 | 15 | 15 | 70 | 4.55E+08 | **1.00** | 20 | 17.8 | 664 | 72.6 | 7.4 | Metabolism;Energy pathways | Mitochondrion;Cytosol | Catalytic activity | Mitochondrion |
| **XRN2** | NP_036387.2 | 22803 | **5'-3' exoribonuclease 2** | 32.6 | 1 | 16 | 16 | 37 | 3.14E+08 | **1.00** | 9 | 18.3 | 950 | 108.5 | 7.5 | Regulation of nucleobase, nucleoside, nucleotide and nucleic acid metabolism | Nucleolus;Nucleus;Cytoplasm | Ribonuclease activity | Nucleolus;Nucleus;Cytoplasm |
| **YIPF5** | NP_001258661.1 | 81555 | **protein YIPF5 isoform b** | 4.4 | 2 | 1 | 1 | 1 | 6.16E+07 | **1.00** | 1 | - | 203 | 21.9 | 4.6 | Biological_process unknown | Golgi apparatus | Molecular function unknown | Golgi apparatus |
| **AK1** | NP_000467.1 | 203 | **adenylate kinase isoenzyme 1** | 62.4 | 1 | 9 | 9 | 30 | 1.94E+08 | **1.00** | 16 | 16.8 | 194 | 21.6 | 8.6 | Metabolism;Energy pathways | Plasma membrane;Cytoplasm | Catalytic activity | Cytoplasm |
| **CUL4A** | NP_001008895.1 | 8451 | **cullin-4A isoform 1** | 24.4 | 3 | 10 | 13 | 29 | 2.26E+08 | **1.00** | 12 | 27.5 | 759 | 87.6 | 8.1 | Protein metabolism | Cytoplasm;Nucleus;Cytosol | Ubiquitin-specific protease activity | Cytoplasm |
| **SPATA20** | NP_001245302.1 | 64847 | **spermatogenesis-associated protein 20 isoform 3** | 3.4 | 3 | 1 | 1 | 1 | 2.17E+06 | **1.00** | 1 | - | 742 | 83.3 | 6.8 | Reproduction;Embryonic development | - | Molecular function unknown | - |
| **SRSF11** | NP_001177916.1 | 9295 | **serine/arginine-rich splicing factor 11 isoform 2** | 19.3 | 2 | 7 | 7 | 30 | 5.40E+08 | **1.00** | 11 | 12.7 | 483 | 53.4 | 10.5 | Regulation of nucleobase, nucleoside, nucleotide and nucleic acid metabolism | Nucleus;Cytoplasm | Transcription regulator activity | Nucleus |
| **CARM1** | NP_954592.1 | 10498 | **histone-arginine methyltransferase CARM1** | 19.9 | 1 | 6 | 6 | 8 | 2.27E+08 | **1.00** | 4 | 21.8 | 608 | 65.8 | 6.7 | Metabolism;Energy pathways | Nucleus | Methyltransferase activity | Nucleus |
| **MED16** | NP_005472.2 | 10025 | **mediator of RNA polymerase II transcription subunit 16** | 2.1 | 1 | 1 | 1 | 2 | 1.87E+08 | **1.00** | 1 | - | 877 | 96.7 | 7.4 | Regulation of nucleobase, nucleoside, nucleotide and nucleic acid metabolism | Nucleus | Transcription factor activity | Nucleus |
| **GTF2F2** | NP_004119.1 | 2963 | **general transcription factor IIF subunit 2** | 29.3 | 1 | 4 | 4 | 9 | 2.68E+08 | **1.00** | 3 | 3.8 | 249 | 28.4 | 9.2 | Regulation of nucleobase, nucleoside, nucleotide and nucleic acid metabolism | Nucleus;Nucleolus | Transcription factor activity | Nucleus |
| **PSMC6** | NP_002797.3 | 5706 | **26S protease regulatory subunit 10B** | 52.6 | 1 | 16 | 16 | 58 | 6.54E+08 | **1.00** | 20 | 12.1 | 403 | 45.8 | 7.8 | Protein metabolism | Cytoplasm | Ubiquitin-specific protease activity | Cytoplasm |
| **DTYMK** | NP_001158503.1 | 1841 | **thymidylate kinase isoform 2** | 45.7 | 2 | 7 | 7 | 15 | 1.88E+08 | **1.00** | 9 | 24.2 | 188 | 21.1 | 8.8 | Metabolism;Energy pathways | Cytoplasm | Catalytic activity | Cytoplasm |
| **PTDSS1** | NP_001277154.1 | 9791 | **phosphatidylserine synthase 1 isoform 2** | 5.8 | 2 | 1 | 1 | 1 | 9.55E+05 | **1.00** | 1 | - | 327 | 38.2 | 8.7 | Metabolism;Energy pathways | Mitochondrion;Nucleus;Cytoplasm | Transferase activity | Mitochondrion |
| **SMARCA1** | NP_003060.2 | 6594 | **probable global transcription activator SNF2L1 isoform a** | 10.9 | 3 | 7 | 11 | 21 | 2.46E+08 | **1.00** | 7 | 38.3 | 1054 | 122.5 | 8.1 | Regulation of nucleobase, nucleoside, nucleotide and nucleic acid metabolism | Nucleus | Transcription regulator activity | Nucleus |
| **C9orf64** | NP_115683.3 | 84267 | **UPF0553 protein C9orf64** | 6.7 | 1 | 1 | 1 | 1 | 4.55E+07 | **1.00** | 1 | - | 341 | 39 | 5.9 | Biological_process unknown | - | Molecular function unknown | - |
| **WDR74** | NP_060563.2 | 54663 | **WD repeat-containing protein 74** | 7.8 | 1 | 2 | 2 | 2 | 8.41E+06 | **1.00** | 1 | - | 385 | 42.4 | 8.3 | Cell communication;Signal transduction | Nucleus | Molecular function unknown | Nucleus |
| **ZFPL1** | NP_006773.2 | 7542 | **zinc finger protein-like 1** | 26.1 | 1 | 4 | 4 | 7 | 2.70E+08 | **1.00** | 5 | 33.9 | 310 | 34.1 | 8.1 | Regulation of nucleobase, nucleoside, nucleotide and nucleic acid metabolism | Nucleus | Transcription regulator activity | Nucleus |
| **MNAT1** | NP_002422.1 | 4331 | **CDK-activating kinase assembly factor MAT1 isoform 1** | 24.9 | 2 | 4 | 4 | 5 | 8.49E+07 | **1.00** | 3 | 3.4 | 309 | 35.8 | 6.1 | Cell communication;Signal transduction | Cytoplasm;Nucleus | Kinase binding | Nucleus |
| **FBL** | NP_001427.2 | 2091 | **rRNA 2'-O-methyltransferase fibrillarin** | 62.0 | 1 | 16 | 16 | 89 | 1.07E+09 | **1.00** | 23 | 15.6 | 321 | 33.8 | 10.2 | Regulation of nucleobase, nucleoside, nucleotide and nucleic acid metabolism | Nucleolus;Nucleus | Ribonucleoprotein | Nucleolus |
| **PAIP2** | NP_001028284.1 | 51247 | **polyadenylate-binding protein-interacting protein 2** | 80.3 | 1 | 4 | 4 | 8 | 1.09E+08 | **1.00** | 2 | 19.7 | 127 | 15 | 4.1 | Regulation of nucleobase, nucleoside, nucleotide and nucleic acid metabolism | Cytoplasm | Translation regulator activity | Cytoplasm |
| **POLD2** | NP_001243808.1 | 5425 | **DNA polymerase delta subunit 2 isoform 1** | 24.5 | 2 | 6 | 6 | 12 | 9.37E+07 | **1.00** | 5 | 47.3 | 469 | 51.3 | 5.6 | Regulation of nucleobase, nucleoside, nucleotide and nucleic acid metabolism | Nucleus | DNA-directed DNA polymerase activity | Nucleus |
| **CYFIP1** | NP_055423.1 | 23191 | **cytoplasmic FMR1-interacting protein 1 isoform a** | 19.6 | 5 | 19 | 19 | 28 | 2.83E+08 | **1.00** | 13 | 17.9 | 1253 | 145.1 | 6.9 | Biological_process unknown | Cytoplasm;Nucleus | Molecular function unknown | Cytoplasm |
| **HSPA14** | NP_057383.2 | 51182 | **heat shock 70 kDa protein 14 isoform 1** | 37.3 | 2 | 10 | 10 | 17 | 2.47E+08 | **1.00** | 6 | 10 | 509 | 54.8 | 5.6 | Biological_process unknown | - | Chaperone activity | - |
| **NUDT4** | NP_061967.3 | 11163 | **diphosphoinositol polyphosphate phosphohydrolase 2 isoform alpha** | 28.3 | 4 | 2 | 2 | 2 | 1.69E+07 | **1.00** | 1 | - | 180 | 20.3 | 6.4 | Biological_process unknown | - | Molecular function unknown | - |
| **KIF16B** | NP_001186794.1 | 55614 | **kinesin-like protein KIF16B isoform 3** | 2.7 | 3 | 1 | 2 | 4 | 4.05E+07 | **0.99** | 1 | - | 1266 | 146.2 | 6.2 | Transport | - | Auxiliary transport protein activity | - |
| **NFS1** | NP_066923.3 | 9054 | **cysteine desulfurase, mitochondrial isoform a precursor** | 20.8 | 2 | 6 | 6 | 18 | 4.18E+07 | **0.99** | 6 | 49 | 457 | 50.2 | 8.3 | Metabolism;Energy pathways | Mitochondrion;Cytoplasm;Nucleus | Sulfotransferase activity | Mitochondrion |
| **PGAM1** | NP_002620.1 | 5223 | **phosphoglycerate mutase 1** | 82.7 | 3 | 21 | 21 | 256 | 5.16E+09 | **0.99** | 43 | 26.5 | 254 | 28.8 | 7.2 | Metabolism;Energy pathways | Cytoplasm | Catalytic activity | Cytoplasm |
| **ALMS1** | NP_055935.4 | 7840 | **Alstrom syndrome protein 1** | 1.4 | 1 | 3 | 3 | 4 | 3.69E+07 | **0.99** | 1 | - | 4169 | 460.9 | 6.3 | Biological_process unknown | Nucleus | Molecular function unknown | Nucleus |
| **COX5A** | NP_004246.2 | 9377 | **cytochrome c oxidase subunit 5A, mitochondrial precursor** | 60.7 | 1 | 9 | 9 | 37 | 4.83E+08 | **0.99** | 11 | 11.5 | 150 | 16.8 | 6.8 | Metabolism;Energy pathways | Mitochondrion | Oxidoreductase activity | Mitochondrion |
| **MFAP1** | NP_005917.2 | 4236 | **microfibrillar-associated protein 1** | 14.6 | 1 | 4 | 4 | 4 | 5.89E+07 | **0.99** | 3 | 10.4 | 439 | 51.9 | 5.0 | Cell growth and/or maintenance | Extracellular;Nucleus;Cytoplasm | Extracellular matrix structural constituent | Extracellular |
| **OGFOD1** | NP_060703.3 | 55239 | **2-oxoglutarate and iron-dependent oxygenase domain-containing protein 1** | 12.6 | 1 | 4 | 4 | 7 | 8.25E+07 | **0.99** | 3 | 2.8 | 542 | 63.2 | 5.1 | Metabolism;Energy pathways | - | Oxidoreductase activity | - |
| **PDIA6** | NP_001269636.1 | 10130 | **protein disulfide-isomerase A6 isoform e precursor** | 63.6 | 5 | 20 | 20 | 152 | 1.58E+09 | **0.99** | 47 | 22.2 | 437 | 47.8 | 5.1 | Protein metabolism | Endoplasmic reticulum;Endoplasmic reticulum lumen;Extracellular;Mitochondrion | Isomerase activity | Endoplasmic reticulum |
| **PPME1** | NP_057231.1 | 51400 | **protein phosphatase methylesterase 1 isoform a** | 42.2 | 2 | 8 | 8 | 27 | 4.10E+08 | **0.99** | 11 | 16.9 | 386 | 42.3 | 6.0 | Metabolism;Energy pathways | - | Methyltransferase activity | - |

**Supplementary Table S4. List of all proteins identified in SCC-S and SCC-R cells using SequestHT and Mascot search algorithms and their gene ontology annotation**

| Gene Symbol | RefSeq_Accession | Entrez Gene ID | Description | Coverage (%) | Proteins | Unique Peptides | # Peptides | PSM | Area | SCC-R /SCC-S | SCC-R /SCC-S (Count) | SCC-R /SCC-S (Variability [%]) | # AAs | MW [kDa] | calc. pI | Biological Process | Localization | Molecular Function | Primary Localization |
|---|---|---|---|---|---|---|---|---|---|---|---|---|---|---|---|---|---|---|---|
| **RBM7** | NP_001272974.1 | 10179 | **RNA-binding protein 7 isoform a** | 17.2 | 2 | 2 | 2 | 4 | 2.05E+07 | **0.99** | 2 | 44.1 | 267 | 30.6 | 9.6 | Regulation of nucleobase, nucleoside, nucleotide and nucleic acid metabolism | - | RNA binding | - |
| **DHX36** | NP_001107869.1 | 170506 | **ATP-dependent RNA helicase DHX36 isoform 2** | 17.9 | 2 | 10 | 10 | 22 | 2.64E+08 | **0.99** | 10 | 24 | 994 | 113.1 | 7.7 | Regulation of nucleobase, nucleoside, nucleotide and nucleic acid metabolism | Plasma membrane | RNA binding | Plasma membrane |
| **QRSL1** | NP_060762.3 | 55278 | **glutamyl-tRNA(Gln) amidotransferase subunit A, mitochondrial** | 3.8 | 1 | 1 | 1 | 1 | 2.52E+07 | **0.99** | 1 | - | 528 | 57.4 | 5.7 | Metabolism;Energy pathways | - | Amidinotransferase activity | - |
| **GNA13** | NP_006563.2 | 10672 | **guanine nucleotide-binding protein subunit alpha-13 isoform 1** | 11.7 | 3 | 3 | 3 | 6 | 2.91E+07 | **0.99** | 3 | 19.1 | 377 | 44 | 8.0 | Cell communication;Signal transduction | Plasma membrane | GTPase activity | Plasma membrane |
| **SETD2** | NP_054878.5 | 29072 | **histone-lysine N-methyltransferase SETD2** | 1.6 | 1 | 2 | 2 | 4 | 3.43E+07 | **0.99** | 2 | 26.6 | 2564 | 287.4 | 6.1 | Regulation of nucleobase, nucleoside, nucleotide and nucleic acid metabolism | Nucleus;Cytoplasm | DNA binding | Nucleus |
| **EDF1** | NP_001268227.1 | 8721 | **endothelial differentiation-related factor 1 isoform 4** | 48.0 | 5 | 6 | 6 | 23 | 4.71E+08 | **0.99** | 6 | 12.9 | 127 | 14.1 | 9.8 | Regulation of nucleobase, nucleoside, nucleotide and nucleic acid metabolism | Nucleus;Cytoplasm | Transcription regulator activity | Nucleus;Cytoplasm |
| **RAB27A** | NP_899058.1 | 5873 | **ras-related protein Rab-27A** | 4.5 | 1 | 1 | 1 | 1 | 3.85E+07 | **0.99** | 1 | - | 221 | 24.9 | 5.2 | Immune response;Protein localization | Plasma membrane;Cytosol | GTPase activity | Plasma membrane |
| **SRSF5** | NP_001034554.1 | 6430 | **serine/arginine-rich splicing factor 5** | 19.5 | 1 | 3 | 4 | 14 | 1.62E+08 | **0.99** | 1 | - | 272 | 31.2 | 11.6 | Regulation of nucleobase, nucleoside, nucleotide and nucleic acid metabolism | Nucleus;Nucleolus | RNA binding | Nucleus |
| **CDC16** | NP_003894.3 | 8881 | **cell division cycle protein 16 homolog** | 15.0 | 1 | 4 | 4 | 7 | 1.10E+08 | **0.99** | 4 | 10.3 | 620 | 71.6 | 5.9 | Cell communication;Signal transduction | Centrosome | Protein binding | Centrosome |
| **DUT** | NP_001939.1 | 1854 | **deoxyuridine 5'-triphosphate nucleotidohydrolase, mitochondrial isoform 2** | 68.3 | 3 | 9 | 9 | 60 | 6.51E+08 | **0.99** | 10 | 15.2 | 164 | 17.7 | 6.6 | DNA replication | Cytoplasm;Nucleus;Mitochondrion | Hydrolase activity | Cytoplasm;Nucleus;Mitochondrion |
| **GUSB** | NP_001271219.1 | 2990 | **beta-glucuronidase isoform 2 precursor** | 1.2 | 2 | 1 | 1 | 1 | 1.83E+07 | **0.99** | 1 | - | 505 | 58.3 | 7.0 | Metabolism;Energy pathways | Lysosome;Endoplasmic reticulum;Golgi apparatus;Extracellular | Hydrolase activity | Lysosome |
| **SBNO2** | NP_001093592.1 | 22904 | **protein strawberry notch homolog 2 isoform 2** | 0.5 | 2 | 1 | 1 | 1 | 1.11E+08 | **0.99** | 1 | - | 1309 | 144.1 | 6.6 | RNA metabolism | - | Helicase activity | - |
| **SF3B5** | NP_112577.1 | 83443 | **splicing factor 3B subunit 5** | 87.2 | 1 | 4 | 4 | 21 | 4.11E+08 | **0.99** | 8 | 15.6 | 86 | 10.1 | 6.4 | Biological_process unknown | - | Molecular function unknown | - |
| **CPSF3** | NP_057291.1 | 51692 | **cleavage and polyadenylation specificity factor subunit 3** | 27.1 | 1 | 8 | 8 | 18 | 3.76E+08 | **0.99** | 7 | 13.2 | 684 | 77.4 | 5.6 | Regulation of nucleobase, nucleoside, nucleotide and nucleic acid metabolism | Nucleus | RNA binding | Nucleus |
| **PDDC1** | NP_872418.1 | 347862 | **Parkinson disease 7 domain-containing protein 1 precursor** | 41.8 | 1 | 4 | 4 | 8 | 8.74E+07 | **0.99** | 4 | 22.9 | 220 | 23.3 | 6.6 | Biological_process unknown | - | Molecular function unknown | - |
| **GSR** | NP_000628.2 | 2936 | **glutathione reductase, mitochondrial isoform 1 precursor** | 19.5 | 4 | 6 | 6 | 12 | 1.36E+08 | **0.99** | 7 | 24.6 | 522 | 56.2 | 8.5 | Metabolism;Energy pathways | Cytoplasm;Mitochondrion | Catalytic activity | Mitochondrion |
| **SYAP1** | NP_116185.2 | 94056 | **synapse-associated protein 1** | 46.6 | 1 | 7 | 7 | 10 | 1.43E+08 | **0.99** | 6 | 94.7 | 352 | 39.9 | 4.5 | Cell communication;Signal transduction | Nucleus;Cytoplasm | Transporter activity | Nucleus;Cytoplasm |
| **CBR1** | NP_001748.1 | 873 | **carbonyl reductase [NADPH] 1 isoform 1** | 71.8 | 2 | 16 | 18 | 84 | 4.39E+08 | **0.99** | 23 | 14.3 | 277 | 30.4 | 8.3 | Metabolism;Energy pathways | Cytoplasm | Oxidoreductase activity | Cytoplasm |
| **DLGAP5** | NP_001139487.1 | 9787 | **disks large-associated protein 5 isoform b** | 3.4 | 2 | 2 | 2 | 2 | 1.42E+07 | **0.99** | 1 | - | 842 | 94.1 | 9.1 | Cell communication;Signal transduction;Regulation of cell cycle | Centrosome;Cytoplasm;Microtubule;Nucleus | Phosphoprotein phosphatase activity | Centrosome |
| **TBK1** | NP_037386.1 | 29110 | **serine/threonine-protein kinase TBK1** | 14.4 | 1 | 6 | 6 | 11 | 9.91E+07 | **0.99** | 6 | 5.4 | 729 | 83.6 | 6.8 | Cell communication;Signal transduction | Cytoplasm | Protein serine/threonine kinase activity | Cytoplasm |
| **ARL6IP4** | NP_001265309.1 | 51329 | **ADP-ribosylation factor-like protein 6-interacting protein 4 isoform 7** | 23.0 | 7 | 3 | 3 | 8 | 1.89E+08 | **0.99** | 2 | 32 | 226 | 25.3 | 10.9 | Regulation of nucleobase, nucleoside, nucleotide and nucleic acid metabolism | Nucleus;Nucleolus;Cytoplasm | Molecular function unknown | Nucleus;Cytoplasm |
| **ASNA1** | NP_004308.2 | 439 | **ATPase ASNA1** | 51.4 | 1 | 11 | 11 | 32 | 2.26E+08 | **0.99** | 11 | 19.8 | 348 | 38.8 | 4.9 | Metabolism;Energy pathways | Cytoplasm;Nucleus;Nucleolus | ATPase activity | Cytoplasm |
| **MORC2** | NP_055756.1 | 22880 | **MORC family CW-type zinc finger protein 2** | 2.2 | 1 | 1 | 1 | 1 | 7.43E+07 | **0.99** | 1 | - | 970 | 110.7 | 8.6 | Biological_process unknown | Nucleus;Cytoplasm | Molecular function unknown | Nucleus;Cytoplasm |
| **NUBP1** | NP_001265435.1 | 4682 | **cytosolic Fe-S cluster assembly factor NUBP1 isoform 2** | 25.6 | 2 | 5 | 5 | 13 | 1.61E+08 | **0.99** | 4 | 41.1 | 309 | 33.4 | 5.2 | Regulation of nucleobase, nucleoside, nucleotide and nucleic acid metabolism | Nucleus | DNA binding | Nucleus |
| **TP53RK** | NP_291028.3 | 112858 | **TP53-regulating kinase** | 17.4 | 1 | 3 | 3 | 5 | 7.33E+07 | **0.99** | 3 | 25.7 | 253 | 28.1 | 9.5 | Cell communication;Signal transduction | Nucleus | Protein serine/threonine kinase activity | Nucleus |
| **ARPC5L** | NP_112240.1 | 81873 | **actin-related protein 2/3 complex subunit 5-like protein** | 51.0 | 1 | 3 | 3 | 21 | 3.00E+08 | **0.98** | 5 | 3.2 | 153 | 16.9 | 6.6 | Biological_process unknown | - | Molecular function unknown | - |
| **HBS1L** | NP_006611.1 | 10767 | **HBS1-like protein isoform 1** | 11.1 | 3 | 3 | 3 | 7 | 7.07E+07 | **0.98** | 2 | 26.2 | 684 | 75.4 | 6.6 | Protein metabolism | Cytoplasm | Translation regulator activity | Cytoplasm |
| **ITPR2** | NP_002214.2 | 3709 | **inositol 1,4,5-trisphosphate receptor type 2** | 0.7 | 4 | 1 | 2 | 2 | 8.02E+07 | **0.98** | 1 | - | 2701 | 307.9 | 6.4 | Cell communication;Signal transduction | Plasma membrane | Transporter activity | Plasma membrane |
| **KHSRP** | NP_003676.2 | 8570 | **far upstream element-binding protein 2** | 43.6 | 1 | 22 | 22 | 118 | 9.01E+08 | **0.98** | 37 | 27.4 | 711 | 73.1 | 7.3 | Regulation of nucleobase, nucleoside, nucleotide and nucleic acid metabolism | Nucleus;Cytoplasm | Transcription regulator activity | Nucleus |
| **SNRPA1** | NP_003081.2 | 6627 | **U2 small nuclear ribonucleoprotein A'** | 59.6 | 1 | 15 | 15 | 41 | 2.65E+08 | **0.98** | 16 | 11.4 | 255 | 28.4 | 8.6 | Regulation of nucleobase, nucleoside, nucleotide and nucleic acid metabolism | Nucleus;Nucleolus;Chromosome | RNA binding | Nucleus |
| **SNRPD3** | NP_004166.1 | 6634 | **small nuclear ribonucleoprotein Sm D3** | 54.8 | 1 | 6 | 6 | 50 | 3.83E+08 | **0.98** | 9 | 7.2 | 126 | 13.9 | 10.3 | Regulation of nucleobase, nucleoside, nucleotide and nucleic acid metabolism | Nucleus;Nucleolus | RNA binding | Nucleus |
| **C12orf10** | NP_067653.3 | 60314 | **UPF0160 protein MYG1, mitochondrial precursor** | 20.0 | 1 | 5 | 5 | 9 | 8.88E+07 | **0.98** | 3 | 48.9 | 376 | 42.4 | 6.7 | Biological_process unknown | Cytosol | Molecular function unknown | Cytosol |
| **CUL4B** | NP_001073341.1 | 8450 | **cullin-4B isoform 2** | 15.9 | 2 | 8 | 11 | 20 | 1.11E+08 | **0.98** | 7 | 71.7 | 895 | 102.2 | 7.9 | Protein metabolism | Nucleus | Ubiquitin-specific protease activity | Nucleus |
| **RRS1** | NP_055984.1 | 23212 | **ribosome biogenesis regulatory protein homolog** | 37.5 | 1 | 11 | 11 | 20 | 3.45E+08 | **0.98** | 9 | 18.9 | 365 | 41.2 | 10.7 | Cell growth and/or maintenance;Biological_process unknown | Nucleus;Nucleolus;Ribosome | Ribonucleoprotein;Molecular function unknown | Nucleus |
| **MLKL** | NP_689862.1 | 197259 | **mixed lineage kinase domain-like protein isoform 1** | 20.8 | 2 | 7 | 7 | 8 | 1.26E+08 | **0.98** | 4 | 13.1 | 471 | 54.4 | 8.8 | Cell communication;Signal transduction | - | Protein-tyrosine kinase activity | - |
| **MYCBP** | NP_036465.2 | 26292 | **C-Myc-binding protein** | 38.8 | 1 | 3 | 3 | 5 | 1.50E+08 | **0.98** | 3 | 11.1 | 103 | 12 | 5.9 | Regulation of nucleobase, nucleoside, nucleotide and nucleic acid metabolism | Cytoplasm;Nucleus;Mitochondrion | Transcription regulator activity | Cytoplasm |
| **HINT3** | NP_612638.3 | 135114 | **histidine triad nucleotide-binding protein 3** | 10.4 | 1 | 1 | 1 | 1 | 1.31E+08 | **0.98** | 1 | - | 182 | 20.3 | 6.6 | Biological_process unknown | - | Molecular function unknown | - |

| Gene Symbol | RefSeq_Accession | Entrez Gene ID | Description | Coverage (%) | Proteins | Unique Peptides | # Peptides | PSM | Area | SCC-R /SCC-S | SCC-R /SCC-S (Count) | SCC-R /SCC-S (Variability [%]) | # AAs | MW [kDa] | calc. pI | Biological Process | Localization | Molecular Function | Primary Localization |
|---|---|---|---|---|---|---|---|---|---|---|---|---|---|---|---|---|---|---|---|
| **KIF2A** | NP_001230881.1 | 3796 | **kinesin-like protein KIF2A isoform 3** | 15.7 | 5 | 7 | 7 | 8 | 2.90E+07 | **0.98** | 2 | 49 | 686 | 77.7 | 6.6 | Cell growth and/or maintenance | Nucleus;Cytoplasm;Microtubule | Motor activity | Cytoplasm |
| **PPP1R21** | NP_001129101.1 | 129285 | **protein phosphatase 1 regulatory subunit 21 isoform 1** | 7.2 | 3 | 3 | 3 | 4 | 4.53E+07 | **0.98** | 2 | 8.3 | 780 | 88.3 | 6.8 | Biological_process unknown | - | Molecular function unknown | - |
| **S100A10** | NP_002957.1 | 6281 | **protein S100-A10** | 57.7 | 1 | 7 | 7 | 123 | 6.02E+09 | **0.98** | 21 | 11.7 | 97 | 11.2 | 7.4 | Cell communication;Signal transduction | Plasma membrane;Nucleus;Cytoplasm | Calcium ion binding | Plasma membrane |
| **MRPL12** | NP_002940.2 | 6182 | **39S ribosomal protein L12, mitochondrial** | 29.3 | 1 | 4 | 4 | 34 | 2.42E+09 | **0.98** | 8 | 14.9 | 198 | 21.3 | 8.9 | Regulation of nucleobase, nucleoside, nucleotide and nucleic acid metabolism | Mitochondrion;Nucleolus | Structural constituent of ribosome | Mitochondrion |
| **MRPS5** | NP_114108.1 | 64969 | **28S ribosomal protein S5, mitochondrial** | 7.4 | 1 | 2 | 2 | 2 | 7.76E+07 | **0.98** | 1 | - | 430 | 48 | 9.9 | Protein metabolism | Mitochondrion | Ribonucleoprotein | Mitochondrion |
| **SRSF9** | NP_003760.1 | 8683 | **serine/arginine-rich splicing factor 9** | 44.8 | 1 | 11 | 11 | 40 | 4.96E+08 | **0.98** | 8 | 8.5 | 221 | 25.5 | 8.7 | Protein metabolism | Nucleus;Nucleolus | RNA binding | Nucleus |
| **ZFYVE16** | NP_001271166.1 | 9765 | **zinc finger FYVE domain-containing protein 16 isoform b** | 9.3 | 2 | 4 | 4 | 6 | 3.95E+07 | **0.98** | 2 | 30.3 | 809 | 88.4 | 4.6 | Transport | Endosome | Auxiliary transport protein activity | Endosome |
| **ARPC1B** | NP_005711.1 | 10095 | **actin-related protein 2/3 complex subunit 1B** | 53.5 | 1 | 16 | 17 | 79 | 1.15E+09 | **0.98** | 25 | 12.8 | 372 | 40.9 | 8.4 | Cell growth and/or maintenance | Plasma membrane;Nucleolus;Cytoplasm | Cytoskeletal protein binding | Plasma membrane |
| **AKR1E2** | NP_001035267.1 | 83592 | **1,5-anhydro-D-fructose reductase isoform 1** | 2.5 | 1 | 1 | 1 | 2 | 7.14E+08 | **0.98** | 1 | - | 320 | 36.6 | 7.5 | Metabolism;Energy pathways | - | Oxidoreductase activity | - |
| **CHMP4C** | NP_689497.1 | 92421 | **charged multivesicular body protein 4c** | 6.0 | 1 | 1 | 1 | 2 | 8.26E+07 | **0.98** | 2 | 20.4 | 233 | 26.4 | 6.1 | Biological_process unknown | - | Molecular function unknown | - |
| **NOP9** | NP_777573.1 | 161424 | **nucleolar protein 9 isoform 1** | 21.7 | 2 | 8 | 8 | 15 | 5.34E+07 | **0.98** | 5 | 21.1 | 636 | 69.4 | 7.3 | Biological_process unknown | Nucleolus;Cytoplasm | Molecular function unknown | Nucleolus |
| **RBBP7** | NP_002884.1 | 5931 | **histone-binding protein RBBP7 isoform 2** | 56.5 | 2 | 9 | 11 | 52 | 5.09E+08 | **0.98** | 15 | 18 | 425 | 47.8 | 5.1 | Regulation of nucleobase, nucleoside, nucleotide and nucleic acid metabolism | Nucleus;Nucleolus | Transcription regulator activity | Nucleus |
| **SMEK1** | NP_001271210.1 | 55671 | **serine/threonine-protein phosphatase 4 regulatory subunit 3A isoform 3** | 15.0 | 1 | 2 | 7 | 13 | 1.06E+08 | **0.98** | 2 | 10.9 | 594 | 67.8 | 5.2 | Biological_process unknown | Cytoplasm;Nucleus | Molecular function unknown | Cytoplasm |
| **GEMIN7** | NP_078983.1 | 79760 | **gem-associated protein 7** | 20.6 | 1 | 1 | 1 | 2 | 1.15E+08 | **0.98** | 1 | - | 131 | 14.5 | 7.4 | Regulation of nucleobase, nucleoside, nucleotide and nucleic acid metabolism | Cytoplasm;Nucleus | Ribonucleoprotein | Cytoplasm |
| **PHF5A** | NP_116147.1 | 84844 | **PHD finger-like domain-containing protein 5A** | 53.6 | 1 | 5 | 5 | 11 | 3.31E+08 | **0.98** | 4 | 13.3 | 110 | 12.4 | 8.4 | Regulation of nucleobase, nucleoside, nucleotide and nucleic acid metabolism | Nucleus | Transcription regulator activity | Nucleus |
| **FKBP5** | NP_004108.1 | 2289 | **peptidyl-prolyl cis-trans isomerase FKBP5 isoform 1** | 13.1 | 2 | 4 | 4 | 7 | 6.43E+07 | **0.98** | 5 | 22.1 | 457 | 51.2 | 5.9 | Metabolism;Energy pathways | Cytoplasm | Isomerase activity | Cytoplasm |
| **GLRX5** | NP_057501.2 | 51218 | **glutaredoxin-related protein 5, mitochondrial precursor** | 21.0 | 1 | 2 | 2 | 13 | 3.12E+08 | **0.98** | 3 | 5.4 | 157 | 16.6 | 6.8 | Metabolism;Energy pathways | Mitochondrion | Oxidoreductase activity | Mitochondrion |
| **WDR82** | NP_079498.2 | 80335 | **WD repeat-containing protein 82** | 35.1 | 1 | 7 | 7 | 15 | 1.08E+08 | **0.98** | 6 | 16.5 | 313 | 35.1 | 7.7 | Biological_process unknown | Nucleus | Molecular function unknown | Nucleus |
| **NBN** | NP_002476.2 | 4683 | **nibrin** | 11.9 | 1 | 5 | 5 | 10 | 9.67E+07 | **0.97** | 2 | 32.8 | 754 | 84.9 | 6.9 | DNA repair | Nucleus;Extracellular;Nucleolus | DNA binding | Nucleus |
| **TXNRD2** | NP_006431.2 | 10587 | **thioredoxin reductase 2, mitochondrial isoform 1 precursor** | 26.3 | 2 | 7 | 8 | 13 | 1.65E+08 | **0.97** | 4 | 11.4 | 524 | 56.5 | 7.5 | Cell death;Cell proliferation | Mitochondrion;Nucleus | Catalytic activity | Mitochondrion |
| **IVD** | NP_001152980.1 | 3712 | **isovaleryl-CoA dehydrogenase, mitochondrial isoform 2 precursor** | 15.2 | 2 | 3 | 3 | 6 | 1.24E+08 | **0.97** | 3 | 25.6 | 396 | 43 | 7.7 | Metabolism;Energy pathways | Mitochondrion | Catalytic activity | Mitochondrion |
| **MCMBP** | NP_079110.1 | 79892 | **mini-chromosome maintenance complex-binding protein isoform 1** | 36.6 | 3 | 14 | 14 | 24 | 3.13E+08 | **0.97** | 7 | 25.8 | 642 | 72.9 | 5.9 | Biological_process unknown | - | Molecular function unknown | - |
| **MRPL9** | NP_113608.1 | 65005 | **39S ribosomal protein L9, mitochondrial** | 16.5 | 1 | 3 | 3 | 5 | 3.43E+07 | **0.97** | 1 | - | 267 | 30.2 | 10.1 | Protein metabolism | Mitochondrion | Structural constituent of ribosome | Mitochondrion |
| **VPS52** | NP_072047.4 | 6293 | **vacuolar protein sorting-associated protein 52 homolog isoform 1** | 17.2 | 4 | 7 | 7 | 9 | 5.89E+07 | **0.97** | 4 | 19 | 723 | 82.2 | 6.0 | Transport | Cytoplasm | Transporter activity | Cytoplasm |
| **COPB1** | NP_001137533.1 | 1315 | **coatomer subunit beta** | 42.8 | 1 | 30 | 30 | 91 | 9.14E+08 | **0.97** | 38 | 16.8 | 953 | 107.1 | 6.1 | Transport | Cytoplasm;Golgi apparatus;Endoplasmic reticulum;Endosome;Mitochondrion | Transporter activity | Cytoplasm |
| **LOC102288414** | NP_001273015.1 | 102288414 | **uncharacterized protein LOC102288414** | 35.8 | 1 | 4 | 4 | 8 | 2.81E+08 | **0.97** | 5 | 6.1 | 123 | 14.2 | 11.5 | - | - | - | - |
| **ORC6** | NP_055136.1 | 23594 | **origin recognition complex subunit 6** | 4.0 | 1 | 1 | 1 | 1 | 3.16E+07 | **0.97** | 1 | - | 252 | 28.1 | 8.7 | Regulation of nucleobase, nucleoside, nucleotide and nucleic acid metabolism | Cytoplasm;Nucleus | DNA binding | Cytoplasm |
| **ANKMY2** | NP_064715.1 | 57037 | **ankyrin repeat and MYND domain-containing protein 2** | 2.5 | 1 | 1 | 1 | 2 | 4.64E+07 | **0.97** | 1 | - | 441 | 49.3 | 6.3 | Biological_process unknown | - | Molecular function unknown | - |
| **DBR1** | NP_057300.2 | 51163 | **lariat debranching enzyme** | 13.1 | 1 | 4 | 4 | 7 | 2.23E+08 | **0.97** | 3 | 5.5 | 544 | 61.5 | 5.5 | Regulation of nucleobase, nucleoside, nucleotide and nucleic acid metabolism | Nucleus;Cytoplasm | Hydrolase activity | Nucleus |
| **TMED5** | NP_057124.3 | 50999 | **transmembrane emp24 domain-containing protein 5 isoform 1 precursor** | 10.0 | 1 | 2 | 2 | 4 | 5.44E+07 | **0.97** | 2 | 1.6 | 229 | 26 | 4.8 | Biological_process unknown | Membrane fraction | Molecular function unknown | Membrane fraction |
| **NUP153** | NP_001265139.1 | 9972 | **nuclear pore complex protein Nup153 isoform 3** | 15.6 | 3 | 12 | 12 | 24 | 9.47E+07 | **0.97** | 5 | 41.5 | 1433 | 149.3 | 8.8 | Transport | Nucleus;Nuclear membrane | Transporter activity | Nucleus |
| **FAM136A** | NP_116211.2 | 84908 | **protein FAM136A** | 63.8 | 2 | 7 | 7 | 20 | 2.20E+08 | **0.97** | 9 | 15.6 | 138 | 15.6 | 7.6 | Regulation of nucleobase, nucleoside, nucleotide and nucleic acid metabolism | Nucleus | Transcription regulator activity | Nucleus |
| **STX8** | NP_004844.1 | 9482 | **syntaxin-8** | 11.9 | 1 | 2 | 2 | 7 | 5.11E+07 | **0.97** | 3 | 9.9 | 236 | 26.9 | 5.0 | Transport | Endoplasmic reticulum;Plasma membrane;Endosome;Golgi apparatus;Cytoplasm | Auxiliary transport protein activity | Plasma membrane |
| **ATP5D** | NP_001678.1 | 513 | **ATP synthase subunit delta, mitochondrial precursor** | 35.7 | 1 | 3 | 3 | 27 | 1.62E+09 | **0.97** | 3 | 3.3 | 168 | 17.5 | 5.5 | Ion transport | Mitochondrial membrane;Mitochondrion | ATPase activity | Mitochondrial membrane |
| **MICAL3** | NP_001116203.1 | 57553 | **protein-methionine sulfoxide oxidase MICAL3 isoform 3** | 0.8 | 3 | 1 | 1 | 2 | 3.32E+06 | **0.97** | 1 | - | 966 | 109 | 7.9 | Cell motility;Transport | Cytoplasm;Microtubule | Cytoskeletal protein binding | Cytoplasm |
| **PSMD12** | NP_002807.1 | 5718 | **26S proteasome non-ATPase regulatory subunit 12 isoform 1** | 54.2 | 2 | 25 | 25 | 69 | 8.95E+08 | **0.97** | 20 | 19.5 | 456 | 52.9 | 7.7 | Protein metabolism | Cytoplasm | Ubiquitin-specific protease activity | Cytoplasm |
| **TRAPPC2L** | NP_057293.1 | 51693 | **trafficking protein particle complex subunit 2-like protein** | 9.3 | 1 | 1 | 1 | 1 | 2.26E+07 | **0.97** | 1 | - | 140 | 16.1 | 6.8 | Cell communication;Signal transduction | - | Molecular function unknown | - |

| Gene Symbol | RefSeq_Accession | Entrez Gene ID | Description | Coverage (%) | Proteins | Unique Peptides | # Peptides | PSM | Area | SCC-R /SCC-S | SCC-R /SCC-S (Count) | SCC-R /SCC-S (Variability [%]) | # AAs | MW [kDa] | calc. pI | Biological Process | Localization | Molecular Function | Primary Localization |
|---|---|---|---|---|---|---|---|---|---|---|---|---|---|---|---|---|---|---|---|
| **ANXA1** | NP_000691.1 | 301 | **annexin A1** | 75.1 | 1 | 35 | 35 | 774 | 8.59E+09 | **0.97** | 142 | 11.4 | 346 | 38.7 | 7.0 | Cell communication;Signal transduction | Cytoplasm;Nucleus;Plasma membrane;Mitochondrion | Calcium ion binding | Plasma membrane |
| **FNTB** | NP_002019.1 | 2342 | **protein farnesyltransferase subunit beta** | 9.4 | 3 | 2 | 2 | 4 | 5.01E+07 | **0.97** | 3 | 4.1 | 437 | 48.7 | 5.8 | Protein metabolism;Protein modification | Nucleus;Cytoplasm | Molecular function unknown;Transferase activity | Cytoplasm |
| **GLOD4** | NP_057164.3 | 51031 | **glyoxalase domain-containing protein 4** | 57.1 | 1 | 13 | 13 | 42 | 3.89E+08 | **0.97** | 10 | 14.4 | 298 | 33.2 | 5.6 | Biological_process unknown | Mitochondrion | Molecular function unknown | Mitochondrion |
| **RAB1B** | NP_112243.1 | 81876 | **ras-related protein Rab-1B** | 80.1 | 16 | 8 | 15 | 134 | 2.66E+09 | **0.97** | 18 | 15.5 | 201 | 22.2 | 5.7 | Cell communication;Signal transduction | Cytoplasmic vesicle;Endoplasmic reticulum;Golgi apparatus;Mitochondrion;Cytosol | GTPase activity | Golgi apparatus |
| **UBR1** | NP_777576.1 | 197131 | **E3 ubiquitin-protein ligase UBR1** | 3.8 | 1 | 3 | 3 | 4 | 8.94E+06 | **0.97** | 1 | - | 1749 | 200.1 | 6.0 | Protein metabolism | - | Ubiquitin-specific protease activity | - |
| **AGFG2** | NP_006067.3 | 3268 | **arf-GAP domain and FG repeat-containing protein 2** | 7.5 | 1 | 2 | 2 | 4 | 4.92E+07 | **0.97** | 3 | 57 | 481 | 48.9 | 9.1 | Biological_process unknown | - | Molecular function unknown | - |
| **BCAS2** | NP_005863.1 | 10286 | **pre-mRNA-splicing factor SPF27** | 67.1 | 1 | 10 | 10 | 37 | 5.63E+08 | **0.97** | 13 | 10.5 | 225 | 26.1 | 5.7 | Cell communication;Signal transduction;Regulation of nucleobase, nucleoside, nucleotide and nucleic acid metabolism | Nucleus;Chromosome | Pre-mRNA splicing factor activity | Nucleus |
| **CBX3** | NP_009207.2 | 11335 | **chromobox protein homolog 3** | 64.5 | 1 | 8 | 10 | 61 | 1.80E+09 | **0.97** | 9 | 6.4 | 183 | 20.8 | 5.3 | Regulation of gene expression, epigenetic | Nucleus;Nucleolus;Cytoplasm | DNA binding | Nucleus |
| **ACSF3** | NP_001120686.1 | 197322 | **acyl-CoA synthetase family member 3, mitochondrial isoform 1 precursor** | 10.6 | 2 | 4 | 4 | 8 | 7.56E+07 | **0.97** | 4 | 49.6 | 576 | 64.1 | 8.4 | Biological_process unknown | - | Molecular function unknown | - |
| **PGP** | NP_001035830.1 | 283871 | **phosphoglycolate phosphatase** | 30.2 | 1 | 7 | 7 | 13 | 1.17E+08 | **0.97** | 8 | 15 | 321 | 34 | 6.1 | Biological_process unknown | - | Molecular function unknown | - |
| **SPCS2** | NP_055567.2 | 9789 | **signal peptidase complex subunit 2** | 34.5 | 1 | 4 | 4 | 16 | 4.30E+08 | **0.96** | 8 | 15.2 | 226 | 25 | 8.5 | Protein metabolism | Microsome | Peptidase activity | Microsome |
| **HTRA2** | NP_037379.1 | 27429 | **serine protease HTRA2, mitochondrial isoform 1 preproprotein** | 18.3 | 3 | 4 | 4 | 7 | 5.31E+07 | **0.96** | 2 | 8.7 | 458 | 48.8 | 10.1 | Protein metabolism | Mitochondrion;Endoplasmic reticulum;Cytoplasm | Serine-type peptidase activity | Mitochondrion |
| **POP4** | NP_006618.1 | 10775 | **ribonuclease P protein subunit p29** | 6.4 | 1 | 1 | 1 | 1 | 4.35E+06 | **0.96** | 1 | - | 220 | 25.4 | 10.1 | Protein metabolism | Nucleolus;Nucleus | Ribonuclease activity | Nucleolus |
| **BRCC3** | NP_001018065.1 | 79184 | **lys-63-specific deubiquitinase BRCC36 isoform 2** | 22.0 | 3 | 4 | 4 | 8 | 5.74E+07 | **0.96** | 3 | 14.6 | 291 | 33.1 | 6.0 | Protein metabolism | - | Ligase activity | - |
| **SAFB2** | NP_055464.1 | 9667 | **scaffold attachment factor B2** | 25.6 | 1 | 9 | 19 | 48 | 3.88E+08 | **0.96** | 8 | 17.8 | 953 | 107.4 | 6.2 | Biological_process unknown | Cytoplasm;Nucleus | Molecular function unknown | Cytoplasm |
| **SDF2L1** | NP_071327.2 | 23753 | **stromal cell-derived factor 2-like protein 1 precursor** | 24.9 | 1 | 3 | 3 | 5 | 9.83E+07 | **0.96** | 2 | 3.6 | 221 | 23.6 | 7.0 | Biological_process unknown | Endoplasmic reticulum;Mitochondrion | Molecular function unknown | Endoplasmic reticulum |
| **DDX42** | NP_987095.1 | 11325 | **ATP-dependent RNA helicase DDX42** | 37.1 | 1 | 19 | 19 | 29 | 3.17E+08 | **0.96** | 13 | 25 | 938 | 102.9 | 7.0 | Regulation of nucleobase, nucleoside, nucleotide and nucleic acid metabolism | Nucleus;Cytoplasm | RNA binding | Nucleus |
| **RAD23B** | NP_002865.1 | 5887 | **UV excision repair protein RAD23 homolog B isoform 1** | 33.3 | 3 | 9 | 9 | 40 | 1.02E+09 | **0.96** | 13 | 11.9 | 409 | 43.1 | 4.8 | Regulation of nucleobase, nucleoside, nucleotide and nucleic acid metabolism | Nucleus;Cytoplasm | DNA binding | Nucleus |
| **TSFM** | NP_005717.3 | 10102 | **elongation factor Ts, mitochondrial isoform 2 precursor** | 28.9 | 4 | 6 | 6 | 17 | 2.08E+08 | **0.96** | 3 | 2.5 | 325 | 35.4 | 8.4 | Protein metabolism | Mitochondrion | Translation regulator activity | Mitochondrion |
| **GOLGA4** | NP_002069.2 | 2803 | **Golgin subfamily A member 4 isoform 2** | 10.9 | 2 | 14 | 14 | 21 | 9.12E+07 | **0.96** | 10 | 17.8 | 2230 | 261 | 5.4 | Transport | Golgi apparatus;Extracellular;Nucleus | Transporter activity | Golgi apparatus;Nucleus |
| **MTMR1** | NP_003819.1 | 8776 | **myotubularin-related protein 1** | 10.2 | 1 | 3 | 3 | 3 | 8.58E+06 | **0.96** | 2 | 44.7 | 665 | 74.6 | 7.1 | Cell communication;Signal transduction | - | Protein tyrosine/serine/threonine phosphatase activity;Lipid phosphatase activity | - |
| **DCTN1** | NP_001177765.1 | 1639 | **dynactin subunit 1 isoform 5** | 44.1 | 6 | 38 | 38 | 84 | 4.73E+08 | **0.96** | 34 | 27.3 | 1236 | 136.7 | 5.4 | Transport | Cytoplasm;Centrosome;Endoplasmic reticulum;Kinetochore;Microtubule | Structural constituent of cytoskeleton | Centrosome |
| **DCTN4** | NP_057305.1 | 51164 | **dynactin subunit 4 isoform b** | 34.8 | 3 | 9 | 9 | 13 | 2.09E+08 | **0.96** | 8 | 24.9 | 460 | 52.3 | 7.3 | Biological_process unknown | Cytoplasm;Centrosome;Nucleus;Microtubule;Kinetochore | Molecular function unknown | Cytoplasm |
| **GNAI3** | NP_006487.1 | 2773 | **guanine nucleotide-binding protein G(k) subunit alpha** | 41.8 | 2 | 11 | 13 | 43 | 1.79E+08 | **0.96** | 10 | 10.3 | 354 | 40.5 | 5.7 | Cell communication;Signal transduction | Plasma membrane;Cytoplasm | GTPase activity | Plasma membrane |
| **RB1** | NP_000312.2 | 5925 | **retinoblastoma-associated protein** | 12.4 | 1 | 7 | 7 | 13 | 5.71E+07 | **0.96** | 4 | 10.9 | 928 | 106.1 | 7.9 | Regulation of nucleobase, nucleoside, nucleotide and nucleic acid metabolism | Nucleus | Transcription regulator activity | Nucleus |
| **SAP18** | NP_005861.2 | 10284 | **histone deacetylase complex subunit SAP18** | 20.9 | 1 | 3 | 3 | 5 | 1.69E+07 | **0.96** | 2 | 8.5 | 172 | 19.5 | 9.8 | Regulation of nucleobase, nucleoside, nucleotide and nucleic acid metabolism | Nucleus;Nucleolus | Transcription regulator activity | Nucleus |
| **THOP1** | NP_003240.1 | 7064 | **thimet oligopeptidase** | 18.3 | 1 | 7 | 7 | 21 | 2.16E+08 | **0.96** | 7 | 32.1 | 689 | 78.8 | 6.1 | Protein metabolism | Cytoplasm;Extracellular;Plasma membrane | Metallopeptidase activity | Cytoplasm |
| **EEF1D** | NP_001951.2 | 1936 | **elongation factor 1-delta isoform 2** | 53.7 | 3 | 2 | 13 | 236 | 4.60E+09 | **0.96** | 7 | 26.5 | 281 | 31.1 | 5.0 | Cell communication;Signal transduction | Endoplasmic reticulum;Mitochondrion;Nucleus;Cytoplasm | Guanyl-nucleotide exchange factor activity | Endoplasmic reticulum |
| **OGT** | NP_858058.1 | 8473 | **UDP-N-acetylglucosamine--peptide N-acetylglucosaminyltransferase 110 kDa subunit isoform 1** | 23.4 | 2 | 15 | 15 | 27 | 3.19E+08 | **0.96** | 11 | 12.1 | 1046 | 116.8 | 6.7 | Protein metabolism | Nucleus;Mitochondrion;Cytoplasm | Transferase activity | Nucleus |
| **PDHB** | NP_000916.2 | 5162 | **pyruvate dehydrogenase E1 component subunit beta, mitochondrial isoform 1 precursor** | 31.8 | 2 | 10 | 10 | 31 | 2.36E+08 | **0.96** | 7 | 8.9 | 359 | 39.2 | 6.7 | Metabolism;Energy pathways | Mitochondrion | Carboxy-lyase activity | Mitochondrion |
| **REPS1** | NP_001273541.1 | 85021 | **ralBP1-associated Eps domain-containing protein 1 isoform d** | 8.2 | 4 | 4 | 4 | 4 | 1.29E+07 | **0.96** | 1 | - | 705 | 76.8 | 5.8 | Cell communication;Signal transduction | Cytoplasm | Calcium ion binding | Cytoplasm |
| **SRA1** | NP_001030312.2 | 10011 | **steroid receptor RNA activator 1 isoform 1** | 35.6 | 2 | 5 | 5 | 6 | 2.04E+07 | **0.96** | 1 | - | 236 | 25.7 | 7.0 | Steroid hormone receptor signaling pathway;Apoptosis;Cell proliferation | Nucleus;Cytoplasm | Steroid hormone receptor activity;Molecular function unknown | Cytoplasm |
| **TUBB4B** | NP_006079.1 | 10383 | **tubulin beta-4B chain** | 84.7 | 3 | 2 | 36 | 1355 | 1.51E+10 | **0.96** | 4 | 4.9 | 445 | 49.8 | 4.9 | Cell growth and/or maintenance | Cytoplasm;Nucleolus;Microtubule | Structural molecule activity | Cytoplasm |
| **ANP32E** | NP_001129951.1 | 81611 | **acidic leucine-rich nuclear phosphoprotein 32 family member E isoform 3** | 35.5 | 5 | 7 | 7 | 23 | 7.39E+08 | **0.96** | 10 | 12.4 | 220 | 25.1 | 3.7 | Biological_process unknown | Nucleus;Nucleolus | Molecular function unknown | Nucleus |
| **CNOT10** | NP_001243670.1 | 25904 | **CCR4-NOT transcription complex subunit 10 isoform 2** | 2.1 | 3 | 1 | 1 | 1 | 5.16E+07 | **0.96** | 1 | - | 717 | 79.3 | 7.8 | Biological_process unknown | - | Molecular function unknown | - |
| **MAPK1** | NP_620407.1 | 5594 | **mitogen-activated protein kinase 1** | 51.1 | 1 | 9 | 12 | 61 | 1.14E+09 | **0.96** | 10 | 17.5 | 360 | 41.4 | 7.0 | Cell communication;Signal transduction | Cytoplasm;Nucleus | Protein serine/threonine kinase activity | Cytoplasm |
| **NELFA** | NP_005654.3 | 7469 | **negative elongation factor A** | 7.8 | 1 | 3 | 3 | 6 | 6.54E+07 | **0.96** | 3 | 9.9 | 539 | 58.5 | 9.3 | Regulation of nucleobase, nucleoside, nucleotide and nucleic acid metabolism | Nucleus | Transcription regulator activity | Nucleus |

Supplementary Table S4. List of all proteins identified in SCC-S and SCC-R cells using SequestHT and Mascot search algorithms and their gene ontology annotation

| Gene Symbol | RefSeq_Accession | Entrez Gene ID | Description | Coverage (%) | Proteins | Unique Peptides | # Peptides | PSM | Area | SCC-R /SCC-S | SCC-R /SCC-S (Count) | SCC-R /SCC-S (Variability [%]) | # AAs | MW [kDa] | calc. pI | Biological Process | Localization | Molecular Function | Primary Localization |
|---|---|---|---|---|---|---|---|---|---|---|---|---|---|---|---|---|---|---|---|
| PGRMC1 | NP_006658.1 | 10857 | membrane-associated progesterone receptor component 1 isoform 1 | 54.9 | 2 | 7 | 8 | 25 | 1.18E+09 | 0.96 | 10 | 30.1 | 195 | 21.7 | 4.7 | Cell communication;Signal transduction | Plasma membrane | Receptor activity | Plasma membrane |
| UGT2B15 | NP_001067.2 | 7366 | UDP-glucuronosyltransferase 2B15 precursor | 1.3 | 2 | 1 | 1 | 3 | 7.43E+07 | 0.96 | 2 | 1.8 | 530 | 61 | 8.8 | Metabolism;Energy pathways | Endoplasmic reticulum | Transferase activity | Endoplasmic reticulum |
| ISOC1 | NP_057132.2 | 51015 | isochorismatase domain-containing protein 1 | 23.8 | 1 | 5 | 5 | 10 | 1.20E+08 | 0.96 | 5 | 17.6 | 298 | 32.2 | 7.4 | Biological_process unknown | - | Molecular function unknown | - |
| PCDHGB4 | NP_115269.1 | 8641 | protocadherin gamma-B4 isoform 2 precursor | 0.8 | 4 | 1 | 1 | 2 | 1.43E+06 | 0.96 | 1 | - | 803 | 87.4 | 4.8 | Cell growth and/or maintenance | Integral to membrane | Cell adhesion molecule activity | Integral to membrane |
| SLC4A7 | NP_001245309.1 | 9497 | sodium bicarbonate cotransporter 3 isoform c | 8.3 | 3 | 5 | 5 | 9 | 2.87E+08 | 0.96 | 3 | 3.1 | 1090 | 123 | 6.9 | Transport | Plasma membrane | Transporter activity | Plasma membrane |
| ZC2HC1A | NP_057094.2 | 51101 | zinc finger C2HC domain-containing protein 1A | 7.4 | 1 | 1 | 1 | 1 | 1.37E+07 | 0.96 | 1 | - | 325 | 35.1 | 9.8 | Biological_process unknown | - | Molecular function unknown | - |
| HNRNPAB | NP_004490.2 | 3182 | heterogeneous nuclear ribonucleoprotein A/B isoform b | 65.3 | 2 | 14 | 15 | 142 | 2.69E+09 | 0.96 | 29 | 13.9 | 285 | 30.6 | 7.9 | Regulation of nucleobase, nucleoside, nucleotide and nucleic acid metabolism | Nucleolus;Nucleus | MRNA binding | Nucleolus |
| TNRC6B | NP_055903.2 | 23112 | trinucleotide repeat-containing gene 6B protein isoform 2 | 2.7 | 2 | 2 | 2 | 2 | 4.62E+06 | 0.96 | 1 | - | 1723 | 182.7 | 7.0 | Biological_process unknown | - | Molecular function unknown | - |
| IDH3A | NP_005521.1 | 3419 | isocitrate dehydrogenase [NAD] subunit alpha, mitochondrial precursor | 36.6 | 1 | 9 | 9 | 52 | 5.28E+08 | 0.95 | 20 | 7.9 | 366 | 39.6 | 6.9 | Metabolism;Energy pathways | Mitochondrion;Extracellular | Catalytic activity | Mitochondrion |
| HIGD2A | NP_620175.1 | 192286 | HIG1 domain family member 2A, mitochondrial | 23.6 | 1 | 1 | 1 | 5 | 5.59E+07 | 0.95 | 1 | - | 106 | 11.5 | 10.2 | Biological_process unknown | Integral to membrane | Molecular function unknown | Integral to membrane |
| SMEK1 | NP_001271209.1 | 55671 | serine/threonine-protein phosphatase 4 regulatory subunit 3A isoform 1 | 11.1 | 1 | 1 | 6 | 14 | 2.15E+08 | 0.95 | 1 | - | 820 | 93.8 | 4.9 | Biological_process unknown | Cytoplasm;Nucleus | Molecular function unknown | Cytoplasm |
| PBDC1 | NP_057584.2 | 51260 | protein PBDC1 | 57.1 | 1 | 8 | 8 | 14 | 3.07E+08 | 0.95 | 7 | 8.8 | 233 | 26 | 4.8 | Biological_process unknown | - | Molecular function unknown | - |
| ARHGAP17 | NP_001006635.1 | 55114 | rho GTPase-activating protein 17 isoform 1 | 15.6 | 2 | 5 | 5 | 8 | 2.24E+08 | 0.95 | 1 | - | 881 | 95.4 | 7.6 | Cell communication;Signal transduction | Cytoplasm;Nucleus | GTPase activator activity | Cytoplasm |
| CIAO1 | NP_004795.1 | 9391 | probable cytosolic iron-sulfur protein assembly protein CIAO1 | 27.1 | 1 | 6 | 6 | 10 | 5.79E+07 | 0.95 | 5 | 32.3 | 339 | 37.8 | 5.0 | Regulation of nucleobase, nucleoside, nucleotide and nucleic acid metabolism | Nucleus;Cytoplasm | Transcription regulator activity | Nucleus |
| PSMA2 | NP_002778.1 | 5683 | proteasome subunit alpha type-2 | 60.3 | 1 | 10 | 10 | 97 | 8.20E+08 | 0.95 | 23 | 21.3 | 234 | 25.9 | 7.4 | Protein metabolism | - | Ubiquitin-specific protease activity | - |
| PSMB4 | NP_002787.2 | 5692 | proteasome subunit beta type-4 | 36.4 | 1 | 6 | 6 | 49 | 6.51E+08 | 0.95 | 8 | 4.4 | 264 | 29.2 | 6.0 | Protein metabolism | Cytoplasm | Ubiquitin-specific protease activity | Cytoplasm |
| SMAP1 | NP_001268368.1 | 60682 | stromal membrane-associated protein 1 isoform C | 2.8 | 4 | 1 | 1 | 2 | 1.78E+08 | 0.95 | 1 | - | 436 | 47.8 | 8.4 | Cell growth and/or maintenance | Integral to membrane | Binding | Integral to membrane |
| DNPEP | NP_036232.2 | 23549 | aspartyl aminopeptidase | 37.3 | 1 | 10 | 10 | 27 | 3.34E+08 | 0.95 | 13 | 34.4 | 485 | 53.4 | 7.6 | Protein metabolism | Cytoplasm | Aminopeptidase activity | Cytoplasm |
| NDUFAF4 | NP_054884.1 | 29078 | NADH dehydrogenase [ubiquinone] 1 alpha subcomplex assembly factor 4 | 31.4 | 1 | 5 | 5 | 7 | 4.95E+07 | 0.95 | 4 | 15.2 | 175 | 20.3 | 8.8 | Cell communication;Signal transduction | - | Molecular function unknown | - |
| RAB21 | NP_055814.1 | 23011 | ras-related protein Rab-21 | 44.9 | 1 | 8 | 8 | 33 | 3.90E+08 | 0.95 | 11 | 7.7 | 225 | 24.3 | 7.9 | Cell communication;Signal transduction | Endoplasmic reticulum;Cytoplasm;Mitochondrion | GTPase activity | Endoplasmic reticulum |
| URI1 | NP_001239570.1 | 8725 | unconventional prefoldin RPB5 interactor 1 isoform c | 12.0 | 2 | 3 | 3 | 4 | 2.43E+07 | 0.95 | 1 | - | 475 | 53.4 | 5.2 | Regulation of nucleobase, nucleoside, nucleotide and nucleic acid metabolism;Regulation of gene expression, epigenetic | Cytoplasm;Endoplasmic reticulum;Nucleus;Microsome | Transcription regulator activity | Cytoplasm |
| HSP90AB1 | NP_001258901.1 | 3326 | heat shock protein HSP 90-beta isoform c | 73.1 | 3 | 45 | 69 | 1680 | 2.90E+10 | 0.95 | 121 | 16.8 | 714 | 82.3 | 5.1 | Cell communication;Signal transduction | Cytoplasm;Nucleus;Nucleolus;Cytosol;Microtubule;Mitochondrion | Chaperone activity | Cytoplasm |
| ND4 | YP_003024035.1 | 4538 | NADH dehydrogenase subunit 4 | 10.0 | 1 | 2 | 2 | 5 | 1.76E+08 | 0.95 | 2 | 14.5 | 459 | 51.5 | 9.4 | Metabolism;Energy pathways | Mitochondrion | Catalytic activity | Mitochondrion |
| BCKDHB | NP_898871.1 | 594 | 2-oxoisovalerate dehydrogenase subunit beta, mitochondrial precursor | 4.9 | 1 | 1 | 1 | 2 | 1.02E+08 | 0.95 | 1 | - | 392 | 43.1 | 6.3 | Metabolism | Mitochondrion | Catalytic activity | Mitochondrion |
| FKBP3 | NP_002004.1 | 2287 | peptidyl-prolyl cis-trans isomerase FKBP3 | 53.6 | 1 | 13 | 13 | 60 | 1.40E+09 | 0.95 | 27 | 11.8 | 224 | 25.2 | 9.3 | Metabolism;Energy pathways | Nucleus;Cytoplasm | Isomerase activity | Nucleus |
| GPR144 | XP_006710279.1 | 347088 | probable G-protein coupled receptor 144 | 1.6 | 3 | 1 | 1 | 1 | 1.01E+08 | 0.95 | 1 | - | 963 | 104 | 8.0 | Cell communication;Signal transduction | Integral to membrane | G-protein coupled receptor activity | Integral to membrane |
| WBSCR22 | NP_059998.2 | 114049 | ribosome biogenesis methyltransferase WBSCR22 isoform 2 | 7.8 | 2 | 1 | 1 | 1 | 3.72E+07 | 0.95 | 1 | - | 281 | 31.9 | 8.7 | Metabolism;Energy pathways | Nucleus;Nucleolus | Methyltransferase activity | Nucleolus |
| ROCK2 | NP_004841.2 | 9475 | rho-associated protein kinase 2 | 15.0 | 1 | 12 | 13 | 19 | 1.84E+08 | 0.95 | 10 | 8.4 | 1388 | 160.8 | 6.0 | Cell communication;Signal transduction | Nucleus;Cytoplasm | Protein serine/threonine kinase activity | Nucleus;Cytoplasm |
| VPS28 | NP_057292.1 | 51160 | vacuolar protein sorting-associated protein 28 homolog isoform 1 | 29.9 | 2 | 4 | 4 | 6 | 1.20E+08 | 0.95 | 2 | 29.4 | 221 | 25.4 | 5.5 | Transport | Cytoplasm;Endosome | Transporter activity | Cytoplasm |
| CDS2 | NP_003809.1 | 8760 | phosphatidate cytidylyltransferase 2 | 11.2 | 1 | 2 | 2 | 6 | 1.69E+08 | 0.95 | 3 | 12.4 | 445 | 51.4 | 7.1 | Metabolism;Energy pathways | Mitochondrion;Nucleus;Cytoplasm | Nucleotidyltransferase activity | Mitochondrion;Nucleus;Cytoplasm |
| RPSA | NP_001012321.1 | 3921 | 40S ribosomal protein SA | 64.1 | 1 | 18 | 18 | 271 | 5.22E+09 | 0.95 | 43 | 13.8 | 295 | 32.8 | 4.9 | Cell communication;Signal transduction;Cell adhesion | Cytoplasm;Ribosome;Nucleolus;Cytosol | Structural constituent of ribosome;Cell adhesion molecule activity | Cytoplasm |
| RRP1 | NP_003674.1 | 8568 | ribosomal RNA processing protein 1 homolog A | 11.9 | 1 | 3 | 3 | 6 | 6.07E+07 | 0.95 | 2 | 3.9 | 461 | 52.8 | 9.3 | Regulation of nucleobase, nucleoside, nucleotide and nucleic acid metabolism | Nucleolus;Nucleus | Molecular function unknown | Nucleolus |
| SUMO2 | NP_001005849.1 | 6613 | small ubiquitin-related modifier 2 isoform b precursor | 45.1 | 7 | 4 | 4 | 29 | 1.08E+09 | 0.95 | 6 | 1.5 | 71 | 8.1 | 5.4 | Biological_process unknown | Nucleus;Cytoplasm;Nuclear membrane | Molecular function unknown | Nucleus |
| NACA | NP_001106673.1 | 4666 | nascent polypeptide-associated complex subunit alpha isoform b | 34.9 | 2 | 9 | 9 | 147 | 1.73E+09 | 0.95 | 28 | 8.9 | 215 | 23.4 | 4.6 | Protein metabolism | Nucleus;Cytoplasm;Ribosome | Chaperone activity | Nucleus |
| DHX9 | NP_001348.2 | 1660 | ATP-dependent RNA helicase A | 46.7 | 1 | 43 | 43 | 300 | 2.39E+09 | 0.94 | 74 | 20.7 | 1270 | 140.9 | 6.8 | Regulation of nucleobase, nucleoside, nucleotide and nucleic acid metabolism | Cytoplasm;Nucleus;Nucleolus;Cytoplasmic vesicle;Integral to membrane | Transcription factor activity | Nucleus |
| MGME1 | NP_443097.1 | 92667 | mitochondrial genome maintenance exonuclease 1 | 16.9 | 1 | 4 | 4 | 9 | 4.45E+07 | 0.94 | 3 | 36.7 | 344 | 39.4 | 7.7 | Biological_process unknown | Mitochondrion | Molecular function unknown | Mitochondrion |
| PHOSPHO2 | NP_001008489.1 | 493911 | pyridoxal phosphate phosphatase PHOSPHO2 | 2.5 | 1 | 1 | 1 | 1 | 1.04E+08 | 0.94 | 1 | - | 241 | 27.8 | 6.8 | Biological_process unknown | - | Molecular function unknown | - |

| Gene Symbol | RefSeq_Accession | Entrez Gene ID | Description | Coverage (%) | Proteins | Unique Peptides | # Peptides | PSM | Area | SCC-R /SCC-S | SCC-R /SCC-S (Count) | SCC-R /SCC-S (Variability [%]) | # AAs | MW [kDa] | calc. pI | Biological Process | Localization | Molecular Function | Primary Localization |
|---|---|---|---|---|---|---|---|---|---|---|---|---|---|---|---|---|---|---|---|
| ARPC2 | NP_690601.1 | 10109 | actin-related protein 2/3 complex subunit 2 | 66.3 | 1 | 15 | 15 | 61 | 1.30E+09 | 0.94 | 20 | 27.2 | 300 | 34.3 | 7.4 | Cell growth and/or maintenance;Cytoskeleton organization and biogenesis | Actin cytoskeleton;Nucleolus | Cytoskeletal protein binding | Actin cytoskeleton |
| GET4 | NP_057033.2 | 51608 | Golgi to ER traffic protein 4 homolog | 8.6 | 1 | 2 | 2 | 6 | 5.30E+07 | 0.94 | 1 | - | 327 | 36.5 | 5.4 | Biological_process unknown | - | Molecular function unknown | - |
| DYNLL1 | NP_001032583.1 | 8655 | dynein light chain 1, cytoplasmic | 62.9 | 1 | 4 | 6 | 31 | 9.13E+08 | 0.94 | 6 | 4.9 | 89 | 10.4 | 7.4 | Cell growth and/or maintenance | Cytoplasm;Nucleus;Perinuclear region;Microtubule;Centrosome;Kinetochore | Motor activity | Cytoplasm |
| GTF2H2D | XP_005276855.1 | 730394 | general transcription factor IIH subunit 2-like protein isoform X8 | 8.9 | 5 | 1 | 1 | 1 | 1.63E+07 | 0.94 | 1 | - | 259 | 28.5 | 6.5 | Biological_process unknown | Nucleus | Molecular function unknown | Nucleus |
| IAH1 | NP_001034702.1 | 285148 | isoamyl acetate-hydrolyzing esterase 1 homolog precursor | 29.0 | 1 | 4 | 4 | 7 | 2.60E+07 | 0.94 | 2 | 37.2 | 248 | 27.6 | 5.3 | Energy pathways;Metabolism | - | Hydrolase activity | - |
| PPIG | NP_004783.2 | 9360 | peptidyl-prolyl cis-trans isomerase G | 4.6 | 1 | 3 | 3 | 4 | 1.06E+08 | 0.94 | 3 | 28.8 | 754 | 88.6 | 10.3 | Metabolism;Energy pathways | Nucleus;Cytoplasm | Isomerase activity | Nucleus |
| TM9SF2 | NP_004791.1 | 9375 | transmembrane 9 superfamily member 2 precursor | 10.4 | 1 | 5 | 5 | 21 | 3.75E+08 | 0.94 | 5 | 2.4 | 663 | 75.7 | 7.4 | Transport | Endosome;Mitochondrion | Molecular function unknown | Endosome |
| DHX15 | NP_001349.2 | 1665 | putative pre-mRNA-splicing factor ATP-dependent RNA helicase DHX15 | 37.4 | 1 | 22 | 22 | 82 | 4.47E+08 | 0.94 | 27 | 21.9 | 795 | 90.9 | 7.5 | Regulation of nucleobase, nucleoside, nucleotide and nucleic acid metabolism | Nucleus;Nucleolus | RNA binding | Nucleus |
| PDXDC1 | NP_001272377.1 | 23042 | pyridoxal-dependent decarboxylase domain-containing protein 1 isoform 5 | 22.8 | 11 | 8 | 8 | 11 | 1.47E+08 | 0.94 | 4 | 20.1 | 697 | 76.5 | 5.5 | Metabolism;Energy pathways | Nucleus;Cytoplasm | Carboxy-lyase activity | Nucleus;Cytoplasm |
| PSMA7 | NP_002783.1 | 5688 | proteasome subunit alpha type-7 | 58.1 | 4 | 14 | 14 | 78 | 1.19E+09 | 0.94 | 19 | 11.9 | 248 | 27.9 | 8.5 | Protein metabolism | Cytoplasm;Nucleus;Nucleolus | Ubiquitin-specific protease activity | Cytoplasm |
| RBM45 | NP_694453.2 | 129831 | RNA-binding protein 45 | 4.4 | 1 | 1 | 1 | 1 | 4.74E+07 | 0.94 | 1 | - | 474 | 53.3 | 7.2 | Regulation of nucleobase, nucleoside, nucleotide and nucleic acid metabolism | Cytoplasm;Nucleus | RNA binding | Cytoplasm |
| CIT | NP_009105.1 | 11113 | citron Rho-interacting kinase isoform 2 | 1.8 | 2 | 2 | 2 | 3 | 2.51E+07 | 0.94 | 2 | 31.9 | 2027 | 231.3 | 6.6 | Cell communication;Signal transduction | Cytoplasm;Nucleus | Protein serine/threonine kinase activity | Cytoplasm;Nucleus |
| COX6C | NP_004365.1 | 1345 | cytochrome c oxidase subunit 6C proprotein | 17.3 | 1 | 2 | 2 | 9 | 3.40E+08 | 0.94 | 4 | 3.6 | 75 | 8.8 | 10.4 | Metabolism;Energy pathways | Mitochondrion | Oxidoreductase activity | Mitochondrion |
| PSMD3 | NP_002800.2 | 5709 | 26S proteasome non-ATPase regulatory subunit 3 | 42.9 | 1 | 16 | 16 | 71 | 5.36E+08 | 0.94 | 22 | 18.7 | 534 | 60.9 | 8.4 | Protein metabolism | Cytoplasm;Peroxisome | Ubiquitin-specific protease activity | Cytoplasm |
| RABEP1 | NP_001278510.1 | 9135 | rab GTPase-binding effector protein 1 isoform 3 | 12.7 | 4 | 6 | 6 | 10 | 1.25E+08 | 0.94 | 5 | 19.6 | 819 | 94.1 | 5.0 | Cell communication;Signal transduction | Cytoplasm;Endosome;Nucleus | GTPase activator activity | Cytoplasm;Nucleus |
| COX7A2 | NP_001856.2 | 1347 | cytochrome c oxidase subunit 7A2, mitochondrial precursor | 20.0 | 1 | 3 | 3 | 11 | 2.79E+08 | 0.94 | 2 | 40.8 | 115 | 12.8 | 9.1 | Metabolism;Energy pathways | Mitochondrion;Cytoplasm | Oxidoreductase activity | Mitochondrion |
| EMC6 | NP_001014764.1 | 83460 | ER membrane protein complex subunit 6 | 10.9 | 1 | 1 | 1 | 1 | 1.42E+08 | 0.94 | 1 | - | 110 | 12 | 10.1 | Biological_process unknown | - | Molecular function unknown | - |
| KPNB1 | NP_002256.2 | 3837 | importin subunit beta-1 isoform 1 | 54.8 | 2 | 40 | 40 | 360 | 2.64E+09 | 0.94 | 84 | 16.6 | 876 | 97.1 | 4.8 | Transport | Cytoplasm;Nucleus;Extracellular;Mitochondrion;Centrosome | Transporter activity | Cytoplasm |
| LUZP1 | NP_361013.3 | 7798 | leucine zipper protein 1 | 8.9 | 1 | 6 | 6 | 8 | 2.21E+07 | 0.94 | 2 | 69.4 | 1076 | 120.2 | 8.5 | Regulation of nucleobase, nucleoside, nucleotide and nucleic acid metabolism | Nucleus;Mitochondrion;Cytoplasm | Transcription regulator activity | Nucleus |
| DHX8 | NP_004932.1 | 1659 | ATP-dependent RNA helicase DHX8 | 1.4 | 1 | 1 | 1 | 1 | 1.06E+08 | 0.94 | 1 | - | 1220 | 139.2 | 8.3 | Regulation of nucleobase, nucleoside, nucleotide and nucleic acid metabolism | Nucleus;Nucleolus | RNA binding | Nucleus |
| FAF2 | NP_055428.1 | 23197 | FAS-associated factor 2 | 35.5 | 1 | 10 | 10 | 33 | 2.21E+08 | 0.94 | 7 | 57.9 | 445 | 52.6 | 5.6 | Apoptosis | Cytoplasm | Molecular function unknown | Cytoplasm |
| GOLGA1 | NP_002068.1 | 2800 | Golgin subfamily A member 1 | 10.7 | 1 | 5 | 5 | 6 | 7.05E+07 | 0.94 | 3 | 19.1 | 767 | 88.1 | 5.3 | Cell growth and/or maintenance | Golgi apparatus;Endosome | Structural molecule activity | Golgi apparatus |
| GORASP1 | NP_001265718.1 | 64689 | Golgi reassembly-stacking protein 1 isoform 2 | 7.8 | 2 | 1 | 1 | 2 | 1.91E+08 | 0.94 | 1 | - | 345 | 35.7 | 4.2 | Cell growth and/or maintenance | Golgi apparatus | Structural molecule activity | Golgi apparatus |
| MED23 | NP_001257450.1 | 9439 | mediator of RNA polymerase II transcription subunit 23 isoform c | 1.3 | 4 | 1 | 1 | 1 | 5.41E+06 | 0.94 | 1 | - | 1359 | 155.5 | 7.4 | Regulation of nucleobase, nucleoside, nucleotide and nucleic acid metabolism | Nucleus | Transcription regulator activity | Nucleus |
| PGM1 | NP_002624.2 | 5236 | phosphoglucomutase-1 isoform 1 | 69.6 | 3 | 33 | 33 | 119 | 6.53E+08 | 0.94 | 44 | 30.2 | 562 | 61.4 | 6.8 | Metabolism;Energy pathways | Cytoplasm;Nucleus | Catalytic activity | Cytoplasm |
| PNN | NP_002678.2 | 5411 | pinin | 16.3 | 1 | 11 | 11 | 22 | 5.86E+08 | 0.94 | 11 | 21.5 | 717 | 81.6 | 7.1 | Cell communication;Signal transduction | Plasma membrane;Nucleus;Nucleolus | Cell adhesion molecule activity | Plasma membrane;Nucleus |
| RPA2 | NP_002937.1 | 6118 | replication protein A 32 kDa subunit isoform 1 | 64.1 | 2 | 8 | 8 | 20 | 3.15E+08 | 0.94 | 11 | 19.9 | 270 | 29.2 | 6.2 | Regulation of nucleobase, nucleoside, nucleotide and nucleic acid metabolism | Nucleus;Cytoplasm | DNA binding | Nucleus |
| KIAA1143 | NP_065747.1 | 57456 | uncharacterized protein KIAA1143 | 10.4 | 1 | 1 | 1 | 1 | 1.66E+08 | 0.94 | 1 | - | 154 | 17.5 | 6.1 | Biological_process unknown | - | Molecular function unknown | - |
| LDHB | NP_001167568.1 | 3945 | L-lactate dehydrogenase B chain | 79.0 | 2 | 26 | 27 | 564 | 9.38E+09 | 0.94 | 93 | 16.2 | 334 | 36.6 | 6.1 | Metabolism;Energy pathways | Cytoplasm | Catalytic activity | Cytoplasm |
| RDX | NP_002897.1 | 5962 | radixin isoform 2 | 45.3 | 5 | 10 | 22 | 85 | 7.03E+09 | 0.94 | 13 | 23.4 | 583 | 68.5 | 6.4 | Cell growth and/or maintenance | Plasma membrane;Cytoplasm | Cytoskeletal protein binding | Plasma membrane |
| ATP1B3 | NP_001670.1 | 483 | sodium/potassium-transporting ATPase subunit beta-3 | 23.7 | 1 | 5 | 5 | 17 | 9.75E+08 | 0.94 | 6 | 19.8 | 279 | 31.5 | 8.4 | Transport | Plasma membrane | ATPase activity | Plasma membrane |
| CASP3 | NP_116786.1 | 836 | caspase-3 preproprotein | 6.9 | 1 | 2 | 2 | 3 | 8.91E+07 | 0.94 | 1 | - | 277 | 31.6 | 6.5 | Apoptosis | Mitochondrion;Cytoplasm;Nucleus | Cysteine-type peptidase activity | Cytoplasm |
| CECR5 | NP_060299.4 | 27440 | cat eye syndrome critical region protein 5 isoform 1 | 22.4 | 2 | 5 | 5 | 14 | 2.14E+08 | 0.94 | 2 | 9.8 | 393 | 43.6 | 6.9 | Biological_process unknown | Mitochondrion | Molecular function unknown | Mitochondrion |
| DUOX1 | NP_059130.2 | 53905 | dual oxidase 1 precursor | 2.4 | 1 | 3 | 3 | 6 | 3.91E+07 | 0.94 | 3 | 17.7 | 1551 | 177.1 | 7.9 | Metabolism | Plasma membrane;Endoplasmic reticulum | Catalytic activity | Plasma membrane |
| PNKP | NP_009185.2 | 11284 | bifunctional polynucleotide phosphatase/kinase | 4.8 | 1 | 2 | 2 | 2 | 6.50E+07 | 0.94 | 2 | 2.2 | 521 | 57 | 8.5 | Regulation of nucleobase, nucleoside, nucleotide and nucleic acid metabolism | Nucleus;Nucleolus | DNA repair protein | Nucleus |
| PPP6R3 | NP_001157636.1 | 55291 | serine/threonine-protein phosphatase 6 regulatory subunit 3 isoform 3 | 29.1 | 10 | 16 | 16 | 35 | 1.73E+08 | 0.94 | 14 | 26.5 | 791 | 88.9 | 4.6 | Biological_process unknown | Cytoplasm;Nucleus | Molecular function unknown | Cytoplasm |
| RPL29 | NP_000983.1 | 6159 | 60S ribosomal protein L29 | 10.1 | 1 | 2 | 2 | 33 | 2.38E+09 | 0.94 | 4 | 6.1 | 159 | 17.7 | 11.7 | Protein metabolism | Plasma membrane;Nucleus;Cytoplasm;Nucleolus;Mitochondrion | Structural constituent of ribosome | Plasma membrane |

| Gene Symbol | RefSeq_Accession | Entrez Gene ID | Description | Coverage (%) | Proteins | Unique Peptides | # Peptides | PSM | Area | SCC-R /SCC-S | SCC-R /SCC-S (Count) | SCC-R /SCC-S (Variability [%]) | # AAs | MW [kDa] | calc. pI | Biological Process | Localization | Molecular Function | Primary Localization |
|---|---|---|---|---|---|---|---|---|---|---|---|---|---|---|---|---|---|---|---|
| **DNAJB11** | NP_057390.1 | 51726 | **dnaJ homolog subfamily B member 11 precursor** | 30.2 | 1 | 9 | 9 | 25 | 2.49E+08 | **0.94** | 7 | 9.2 | 358 | 40.5 | 6.2 | Protein metabolism | Endoplasmic reticulum;Mitochondrion | Chaperone activity | Endoplasmic reticulum |
| **LYPLA2** | NP_009191.1 | 11313 | **acyl-protein thioesterase 2** | 43.3 | 1 | 5 | 5 | 15 | 2.60E+08 | **0.94** | 7 | 10.1 | 231 | 24.7 | 7.2 | Metabolism;Energy pathways | - | Phospholipase activity | - |
| **OLA1** | NP_037473.3 | 29789 | **obg-like ATPase 1 isoform 1** | 63.6 | 2 | 18 | 18 | 112 | 4.70E+08 | **0.93** | 39 | 13.9 | 396 | 44.7 | 7.8 | Biological_process unknown | Nucleolus | Molecular function unknown | Nucleolus |
| **ABCA13** | NP_689914.3 | 154664 | **ATP-binding cassette sub-family A member 13** | 0.2 | 1 | 2 | 2 | 3 | 2.74E+07 | **0.93** | 1 | - | 5058 | 575.8 | 6.4 | Transport | Plasma membrane | Transporter activity | Plasma membrane |
| **EIF4E** | NP_001959.1 | 1977 | **eukaryotic translation initiation factor 4E isoform 1** | 16.6 | 3 | 3 | 3 | 14 | 3.15E+08 | **0.93** | 5 | 6.7 | 217 | 25.1 | 6.2 | Protein metabolism | Perinuclear region;Nucleus;Cytoplasm | Translation regulator activity | Nucleus |
| **POLR2C** | NP_116558.1 | 5432 | **DNA-directed RNA polymerase II subunit RPB3** | 38.2 | 1 | 5 | 5 | 9 | 4.55E+07 | **0.93** | 4 | 21.9 | 275 | 31.4 | 4.9 | Regulation of nucleobase, nucleoside, nucleotide and nucleic acid metabolism | Nucleus | Transcription regulator activity | Nucleus |
| **PTBP1** | NP_114368.1 | 5725 | **polypyrimidine tract-binding protein 1 isoform c** | 85.7 | 4 | 23 | 28 | 515 | 3.52E+09 | **0.93** | 83 | 16.5 | 531 | 57.2 | 9.2 | Regulation of nucleobase, nucleoside, nucleotide and nucleic acid metabolism | Nucleus;Cytoplasm;Nucleolus | Ribonucleoprotein | Nucleus |
| **STAU1** | NP_001032405.1 | 6780 | **double-stranded RNA-binding protein Staufen homolog 1 isoform c** | 60.6 | 3 | 18 | 18 | 54 | 4.03E+08 | **0.93** | 22 | 19.7 | 502 | 55.6 | 9.5 | Protein metabolism | Endoplasmic reticulum;Cytoplasm;Nucleus | RNA binding;Transporter activity | Endoplasmic reticulum |
| **PDE12** | NP_808881.3 | 201626 | **2',5'-phosphodiesterase 12** | 11.8 | 1 | 3 | 3 | 5 | 1.97E+07 | **0.93** | 2 | 3.3 | 609 | 67.3 | 6.6 | Metabolism;Energy pathways | - | Phosphoric diester hydrolase activity | - |
| **PSMA1** | NP_002777.1 | 5682 | **proteasome subunit alpha type-1 isoform 2** | 65.4 | 3 | 15 | 15 | 85 | 8.34E+08 | **0.93** | 25 | 19.8 | 263 | 29.5 | 6.6 | Protein metabolism | Nucleus;Cytoplasm | Peptidase activity | Cytoplasm |
| **PSMD13** | NP_002808.3 | 5719 | **26S proteasome non-ATPase regulatory subunit 13 isoform 1** | 57.5 | 2 | 17 | 17 | 64 | 1.24E+09 | **0.93** | 27 | 9.8 | 376 | 42.9 | 5.8 | Protein metabolism | Cytoplasm | Ubiquitin-specific protease activity | Cytoplasm |
| **SCLY** | NP_057594.4 | 51540 | **selenocysteine lyase** | 8.0 | 1 | 2 | 2 | 3 | 8.82E+06 | **0.93** | 1 | - | 453 | 48.8 | 7.1 | Metabolism;Energy pathways | Cytoplasm | Lyase activity | Cytoplasm |
| **UBA3** | NP_003959.3 | 9039 | **NEDD8-activating enzyme E1 catalytic subunit isoform 1** | 32.6 | 2 | 7 | 7 | 12 | 1.10E+08 | **0.93** | 6 | 19.8 | 463 | 51.8 | 5.5 | Protein metabolism | Nucleus;Cytoplasm | Ubiquitin-specific protease activity | Nucleus |
| **KIFC1** | NP_002254.2 | 3833 | **kinesin-like protein KIFC1** | 2.1 | 1 | 1 | 1 | 1 | 2.57E+07 | **0.93** | 1 | - | 673 | 73.7 | 9.0 | Microtubule-based process | Microtubule | Motor activity | Microtubule |
| **NLN** | NP_065777.1 | 57486 | **neurolysin, mitochondrial** | 19.6 | 1 | 9 | 9 | 19 | 1.54E+08 | **0.93** | 7 | 30 | 704 | 80.6 | 6.6 | Protein metabolism | Cytoplasm;Mitochondrion;Plasma membrane | Metallopeptidase activity | Cytoplasm |
| **NUP98** | NP_624358.2 | 4928 | **nuclear pore complex protein Nup98-Nup96 isoform 4** | 13.4 | 6 | 16 | 17 | 29 | 3.78E+08 | **0.93** | 12 | 32.1 | 1726 | 187.1 | 6.5 | Transport | Nucleus;Cytoplasm;Nuclear membrane;Mitochondrion | Transporter activity | Nucleus;Nuclear membrane;Cytoplasm |
| **TOMM22** | NP_064628.1 | 56993 | **mitochondrial import receptor subunit TOM22 homolog** | 66.2 | 1 | 6 | 6 | 28 | 5.64E+08 | **0.93** | 6 | 22.6 | 142 | 15.5 | 4.3 | Transport | Mitochondrion | Auxiliary transport protein activity | Mitochondrion |
| **ACOT9** | NP_001028755.2 | 23597 | **acyl-coenzyme A thioesterase 9, mitochondrial isoform b precursor** | 18.5 | 2 | 6 | 6 | 13 | 1.10E+08 | **0.93** | 5 | 8.7 | 439 | 49.9 | 8.6 | Metabolism | Mitochondrion | Hydrolase activity | Mitochondrion |
| **ERCC4** | NP_005227.1 | 2072 | **DNA repair endonuclease XPF** | 2.6 | 1 | 1 | 1 | 2 | 1.71E+07 | **0.93** | 1 | - | 916 | 104.4 | 6.9 | Regulation of nucleobase, nucleoside, nucleotide and nucleic acid metabolism | Nucleus;Cytoplasm | DNA repair protein | Nucleus |
| **KIAA0101** | NP_055551.1 | 9768 | **PCNA-associated factor isoform 1** | 36.0 | 2 | 3 | 3 | 4 | 6.07E+07 | **0.93** | 2 | 18.5 | 111 | 12 | 9.8 | Biological_process unknown | - | Molecular function unknown | - |
| **C1orf122** | NP_940848.2 | 127687 | **uncharacterized protein C1orf122 isoform 1** | 25.5 | 2 | 2 | 2 | 2 | 4.52E+07 | **0.93** | 2 | 55 | 110 | 11.5 | 6.7 | Biological_process unknown | - | Molecular function unknown | - |
| **CORO1C** | NP_001263400.1 | 23603 | **coronin-1C isoform b** | 51.7 | 3 | 24 | 24 | 76 | 5.83E+08 | **0.93** | 27 | 14.5 | 474 | 53.2 | 7.1 | Cell growth and/or maintenance | Cytoplasm | Cytoskeletal protein binding | Cytoplasm |
| **OSGEP** | NP_060277.1 | 55644 | **probable tRNA N6-adenosine threonylcarbamoyltransferase** | 11.0 | 1 | 3 | 3 | 6 | 4.81E+07 | **0.93** | 3 | 4.5 | 335 | 36.4 | 6.4 | Protein metabolism | - | Peptidase activity | - |
| **DLD** | NP_000099.2 | 1738 | **dihydrolipoyl dehydrogenase, mitochondrial isoform 1 precursor** | 36.0 | 4 | 15 | 15 | 72 | 9.25E+08 | **0.93** | 26 | 12.3 | 509 | 54.1 | 7.9 | Metabolism;Energy pathways | Mitochondrion | Catalytic activity | Mitochondrion |
| **MALSU1** | NP_612455.1 | 115416 | **mitochondrial assembly of ribosomal large subunit protein 1** | 5.1 | 1 | 1 | 1 | 1 | 2.04E+07 | **0.93** | 1 | - | 234 | 26.2 | 5.5 | Biological_process unknown | - | Molecular function unknown | - |
| **NIPA2** | NP_001008894.1 | 81614 | **magnesium transporter NIPA2 isoform b** | 3.8 | 2 | 1 | 1 | 1 | 2.29E+07 | **0.93** | 1 | - | 341 | 37 | 8.2 | Biological_process unknown | Integral to membrane | Molecular function unknown | Integral to membrane |
| **GOLGA3** | NP_005886.2 | 2802 | **Golgin subfamily A member 3 isoform 1** | 11.8 | 1 | 3 | 12 | 19 | 6.77E+07 | **0.93** | 2 | 42.4 | 1498 | 167.3 | 5.4 | Cell growth and/or maintenance | Golgi apparatus;Cytoplasm;Nucleus | Structural molecule activity | Golgi apparatus;Nucleus |
| **HAT1** | NP_003633.1 | 8520 | **histone acetyltransferase type B catalytic subunit** | 31.3 | 1 | 8 | 8 | 21 | 2.89E+08 | **0.93** | 7 | 3.5 | 419 | 49.5 | 5.7 | Metabolism | Nucleus;Cytoplasm | Acyltransferase activity | Nucleus |
| **HDAC2** | NP_001518.3 | 3066 | **histone deacetylase 2** | 17.0 | 1 | 3 | 5 | 15 | 3.59E+08 | **0.93** | 5 | 20.9 | 488 | 55.3 | 5.9 | Regulation of nucleobase, nucleoside, nucleotide and nucleic acid metabolism | Nucleus;Nucleolus;Cytoplasm | Transcription regulator activity;DNA binding | Nucleus |
| **LYPLA1** | NP_001266285.1 | 10434 | **acyl-protein thioesterase 1 isoform 2** | 48.5 | 6 | 4 | 4 | 12 | 3.89E+08 | **0.93** | 5 | 6.2 | 196 | 21.1 | 6.3 | Metabolism;Energy pathways | Endoplasmic reticulum;Nucleus | Phospholipase activity | Endoplasmic reticulum |
| **METAP2** | NP_006829.1 | 10988 | **methionine aminopeptidase 2** | 62.8 | 1 | 21 | 21 | 74 | 1.13E+09 | **0.93** | 24 | 20.8 | 478 | 52.9 | 5.8 | Protein metabolism | - | Translation regulator activity | - |
| **NUP155** | NP_004289.1 | 9631 | **nuclear pore complex protein Nup155 isoform 2** | 29.8 | 3 | 22 | 22 | 65 | 2.84E+08 | **0.93** | 24 | 25.7 | 1332 | 148.9 | 6.3 | Transport | Nucleus;Cytoplasm;Mitochondrion;Nuclear membrane | Transporter activity | Nucleus |
| **SUPT6H** | NP_003161.2 | 6830 | **transcription elongation factor SPT6** | 16.6 | 1 | 16 | 16 | 27 | 1.91E+08 | **0.93** | 7 | 13.9 | 1726 | 198.9 | 4.9 | Regulation of nucleobase, nucleoside, nucleotide and nucleic acid metabolism | Nucleus;Cytoplasm | Transcription factor activity | Nucleus |
| **CNDP2** | NP_060705.2 | 55748 | **cytosolic non-specific dipeptidase isoform 1** | 45.1 | 2 | 16 | 16 | 56 | 5.55E+08 | **0.93** | 23 | 32.4 | 475 | 52.8 | 6.0 | Protein metabolism | Cytoplasm | Metallopeptidase activity | Cytoplasm |
| **EIF3M** | NP_006351.2 | 10480 | **eukaryotic translation initiation factor 3 subunit M** | 57.0 | 1 | 15 | 15 | 79 | 5.76E+08 | **0.93** | 23 | 20.9 | 374 | 42.5 | 5.6 | Biological_process unknown | - | Molecular function unknown | - |
| **PSMB3** | NP_002786.2 | 5691 | **proteasome subunit beta type-3** | 47.3 | 1 | 7 | 7 | 41 | 1.84E+09 | **0.93** | 14 | 15.4 | 205 | 22.9 | 6.6 | Protein metabolism | Nucleus;Cytoplasm | Ubiquitin-specific protease activity | Cytoplasm |
| **SSNA1** | NP_003722.2 | 8636 | **Sjoegren syndrome nuclear autoantigen 1** | 48.7 | 1 | 4 | 4 | 8 | 1.31E+08 | **0.93** | 4 | 19 | 119 | 13.6 | 5.4 | Biological_process unknown | Nucleus | Molecular function unknown | Nucleus |

| Gene Symbol | RefSeq_Accession | Entrez Gene ID | Description | Coverage (%) | Proteins | Unique Peptides | # Peptides | PSM | Area | SCC-R /SCC-S | SCC-R /SCC-S (Count) | SCC-R /SCC-S (Variability [%]) | # AAs | MW [kDa] | calc. pI | Biological Process | Localization | Molecular Function | Primary Localization |
|---|---|---|---|---|---|---|---|---|---|---|---|---|---|---|---|---|---|---|---|
| **CWC15** | NP_057487.2 | 51503 | **spliceosome-associated protein CWC15 homolog** | 29.7 | 1 | 5 | 5 | 8 | 1.88E+08 | **0.93** | 4 | 40.5 | 229 | 26.6 | 5.7 | Biological_process unknown | Nucleus | Molecular function unknown | Nucleus |
| **NDUFA6** | NP_002481.2 | 4700 | **NADH dehydrogenase [ubiquinone] 1 alpha subcomplex subunit 6** | 15.6 | 1 | 2 | 2 | 3 | 9.80E+07 | **0.93** | 2 | 3.3 | 154 | 17.9 | 10.1 | Metabolism;Energy pathways | Mitochondrion | Oxidoreductase activity | Mitochondrion |
| **POLR2H** | NP_006223.2 | 5437 | **DNA-directed RNA polymerases I, II, and III subunit RPABC3 isoform 2** | 32.0 | 5 | 4 | 4 | 20 | 8.78E+07 | **0.93** | 3 | 15.2 | 150 | 17.1 | 4.7 | Regulation of nucleobase, nucleoside, nucleotide and nucleic acid metabolism | Nucleus;Nucleolus | DNA-directed RNA polymerase activity | Nucleus |
| **STAMBP** | NP_006454.1 | 10617 | **STAM-binding protein** | 11.1 | 1 | 3 | 3 | 5 | 7.68E+07 | **0.93** | 3 | 16.4 | 424 | 48 | 6.3 | Cell communication;Signal transduction | Cytoplasm;Nucleus | Receptor signaling complex scaffold activity | Cytoplasm |
| **IPO5** | NP_002262.3 | 3843 | **importin-5** | 58.7 | 1 | 41 | 42 | 212 | 1.11E+09 | **0.92** | 59 | 16.6 | 1115 | 125.5 | 4.9 | Transport | Cytoplasm;Nucleus;Mitochondrion;Centrosome | Transporter activity | Cytoplasm |
| **MRPL13** | NP_054797.2 | 28998 | **39S ribosomal protein L13, mitochondrial** | 30.9 | 1 | 3 | 3 | 4 | 1.57E+08 | **0.92** | 4 | 60.8 | 178 | 20.7 | 9.2 | Protein metabolism | Mitochondrion | Structural constituent of ribosome | Mitochondrion |
| **PRKRA** | NP_001132990.1 | 8575 | **interferon-inducible double-stranded RNA-dependent protein kinase activator A isoform 3** | 31.6 | 3 | 5 | 5 | 15 | 3.83E+07 | **0.92** | 6 | 21.8 | 288 | 31.6 | 8.4 | Regulation of nucleobase, nucleoside, nucleotide and nucleic acid metabolism | Cytoplasm | RNA binding | Cytoplasm |
| **RPN1** | NP_002941.1 | 6184 | **dolichyl-diphosphooligosaccharide--protein glycosyltransferase subunit 1 precursor** | 58.0 | 1 | 28 | 28 | 153 | 5.45E+09 | **0.92** | 42 | 30.2 | 607 | 68.5 | 6.4 | Protein metabolism | Endoplasmic reticulum;Nucleus;Cytoplasm;Mitochondrion;Cytosol | Ubiquitin-specific protease activity | Endoplasmic reticulum;Nucleus;Cytosol |
| **URAD** | NP_001099047.1 | 646625 | **putative 2-oxo-4-hydroxy-4-carboxy-5-ureidoimidazoline decarboxylase** | 3.5 | 1 | 1 | 1 | 1 | 4.52E+07 | **0.92** | 1 | - | 173 | 19.1 | 6.1 | - | - | - | - |
| **CRK** | NP_005197.3 | 1398 | **adapter molecule crk isoform b** | 63.2 | 2 | 9 | 9 | 31 | 6.60E+08 | **0.92** | 12 | 18.1 | 204 | 22.9 | 5.5 | Cell communication;Signal transduction | Cytoplasm;Nucleus;Plasma membrane | Receptor signaling complex scaffold activity | Cytoplasm |
| **DCUN1D1** | NP_065691.2 | 54165 | **DCN1-like protein 1** | 17.8 | 1 | 3 | 3 | 8 | 1.21E+08 | **0.92** | 5 | 29.6 | 259 | 30.1 | 5.3 | Biological_process unknown | - | Molecular function unknown | - |
| **STOML2** | NP_038470.1 | 30968 | **stomatin-like protein 2, mitochondrial isoform a** | 66.6 | 4 | 16 | 16 | 87 | 6.95E+08 | **0.92** | 30 | 16.4 | 356 | 38.5 | 7.4 | Transport | Plasma membrane;Mitochondrion;Integral to membrane | Auxiliary transport protein activity | Plasma membrane |
| **EIF4G2** | NP_001409.3 | 1982 | **eukaryotic translation initiation factor 4 gamma 2 isoform 1** | 38.7 | 2 | 26 | 26 | 68 | 7.61E+08 | **0.92** | 35 | 14.2 | 907 | 102.3 | 7.1 | Protein metabolism | Ribosome;Cytoplasm | Translation regulator activity | Cytoplasm |
| **GDI2** | NP_001485.2 | 2665 | **rab GDP dissociation inhibitor beta isoform 1** | 75.5 | 2 | 28 | 39 | 241 | 1.03E+09 | **0.92** | 45 | 21.7 | 445 | 50.6 | 6.5 | Transport | Cytoplasm;Plasma membrane;Endoplasmic reticulum;Centrosome | Auxiliary transport protein activity | Cytoplasm |
| **SLC25A36** | NP_060625.2 | 55186 | **solute carrier family 25 member 36 isoform b** | 9.4 | 2 | 1 | 1 | 2 | 7.16E+06 | **0.92** | 1 | - | 310 | 34.1 | 8.7 | Metabolism | Mitochondrion | Transporter activity | Mitochondrion |
| **EIF3I** | NP_003748.1 | 8668 | **eukaryotic translation initiation factor 3 subunit I** | 72.6 | 1 | 18 | 18 | 59 | 8.39E+08 | **0.92** | 20 | 18.3 | 325 | 36.5 | 5.6 | Protein metabolism | Ribosome | Translation regulator activity | Ribosome |
| **HSP90B1** | NP_003290.1 | 7184 | **endoplasmin precursor** | 65.5 | 1 | 51 | 53 | 549 | 7.30E+09 | **0.92** | 98 | 16.8 | 803 | 92.4 | 4.8 | Protein metabolism | Endoplasmic reticulum;Mitochondrion;Cytosol | Heat shock protein activity | Endoplasmic reticulum |
| **MRPS17** | NP_057053.1 | 51373 | **28S ribosomal protein S17, mitochondrial** | 30.8 | 1 | 2 | 2 | 8 | 2.09E+08 | **0.92** | 1 | - | 130 | 14.5 | 9.9 | Protein metabolism | Mitochondrion;Nucleus | Structural constituent of ribosome | Mitochondrion |
| **NSMCE2** | NP_775956.1 | 286053 | **E3 SUMO-protein ligase NSE2** | 6.1 | 1 | 1 | 1 | 1 | 2.24E+07 | **0.92** | 1 | - | 247 | 27.9 | 7.7 | DNA repair | - | DNA repair protein | - |
| **RALGAPB** | NP_001269847.1 | 57148 | **ral GTPase-activating protein subunit beta isoform 2** | 1.7 | 2 | 1 | 1 | 1 | 6.27E+07 | **0.92** | 1 | - | 1491 | 166.4 | 6.7 | Cell communication;Signal transduction | Smooth microsome;Plasma membrane;Rough microsome;Cytosol | GTPase activator activity | Smooth microsome |
| **RBM14** | NP_006319.1 | 10432 | **RNA-binding protein 14 isoform 1** | 25.9 | 4 | 12 | 12 | 52 | 3.28E+08 | **0.92** | 16 | 24.1 | 669 | 69.4 | 9.7 | Regulation of nucleobase, nucleoside, nucleotide and nucleic acid metabolism | Nucleus;Nucleolus;Cytoplasm | RNA binding | Nucleus |
| **SRC** | NP_005408.1 | 6714 | **proto-oncogene tyrosine-protein kinase Src** | 8.8 | 1 | 2 | 4 | 4 | 7.42E+07 | **0.92** | 2 | 8.2 | 536 | 59.8 | 7.4 | Signal transduction | Nucleus;Plasma membrane;Cytoplasm | Protein-tyrosine kinase activity | Cytoplasm |
| **DENND2D** | NP_001258762.1 | 79961 | **DENN domain-containing protein 2D isoform b** | 4.1 | 1 | 1 | 1 | 1 | 1.14E+08 | **0.92** | 1 | - | 468 | 53.1 | 7.8 | Biological_process unknown | - | Molecular function unknown | - |
| **PAPSS1** | NP_005434.4 | 9061 | **bifunctional 3'-phosphoadenosine 5'-phosphosulfate synthase 1** | 28.4 | 1 | 9 | 10 | 18 | 2.02E+08 | **0.92** | 9 | 6.7 | 624 | 70.8 | 6.9 | Metabolism;Energy pathways | Nucleus | Nucleotidyltransferase activity | Nucleus |
| **PCID2** | NP_060856.2 | 55795 | **PCI domain-containing protein 2 isoform 1** | 11.8 | 3 | 3 | 3 | 5 | 2.58E+07 | **0.92** | 1 | - | 399 | 46 | 8.5 | Biological_process unknown | - | Molecular function unknown | - |
| **APEX1** | NP_542379.1 | 328 | **DNA-(apurinic or apyrimidinic site) lyase** | 58.2 | 1 | 16 | 16 | 109 | 1.05E+09 | **0.92** | 31 | 26.7 | 318 | 35.5 | 8.1 | Regulation of nucleobase, nucleoside, nucleotide and nucleic acid metabolism | Cytoplasm;Nucleus;Endoplasmic reticulum;Mitochondrion;Nucleolus | DNA repair protein;Transcription regulator activity | Cytoplasm |
| **EIF5A** | NP_001137233.1 | 1984 | **eukaryotic translation initiation factor 5A-1 isoform B** | 72.7 | 3 | 7 | 16 | 203 | 5.23E+09 | **0.92** | 14 | 22.8 | 154 | 16.8 | 5.2 | Protein metabolism | Cytoplasm;Nucleus;Nucleolus;Mitochondrion | Translation factor activity, nucleic acid binding | Cytoplasm |
| **GNL2** | NP_037417.1 | 29889 | **nucleolar GTP-binding protein 2** | 3.8 | 1 | 2 | 2 | 4 | 5.33E+07 | **0.92** | 2 | 5 | 731 | 83.6 | 9.3 | Cell communication;Signal transduction | Nucleolus;Ribosome;Nucleus | GTPase activity | Nucleolus |
| **MRPS21** | NP_114107.1 | 54460 | **28S ribosomal protein S21, mitochondrial** | 13.8 | 1 | 1 | 1 | 2 | 3.58E+07 | **0.92** | 1 | - | 87 | 10.7 | 10.2 | Protein metabolism | Mitochondrion | Structural constituent of ribosome | Mitochondrion |
| **NDUFA10** | NP_004535.1 | 4705 | **NADH dehydrogenase [ubiquinone] 1 alpha subcomplex subunit 10, mitochondrial precursor** | 26.2 | 1 | 5 | 5 | 15 | 1.98E+08 | **0.92** | 5 | 4.1 | 355 | 40.7 | 8.5 | Metabolism;Energy pathways | Mitochondrion | Oxidoreductase activity | Mitochondrion |
| **OSBP** | NP_002547.1 | 5007 | **oxysterol-binding protein 1** | 25.5 | 3 | 11 | 13 | 36 | 5.00E+08 | **0.92** | 15 | 23.1 | 807 | 89.4 | 7.3 | Transport | Cytoplasm;Golgi apparatus;Nucleus | Transporter activity | Cytoplasm |
| **PFKL** | NP_002617.3 | 5211 | **6-phosphofructokinase, liver type isoform b** | 34.9 | 2 | 16 | 18 | 67 | 3.32E+08 | **0.92** | 14 | 35.7 | 780 | 85 | 7.5 | Metabolism;Energy pathways | Cytoplasm | Catalytic activity | Cytoplasm |
| **SNAPIN** | NP_036569.1 | 23557 | **SNARE-associated protein Snapin** | 24.3 | 1 | 2 | 2 | 5 | 6.53E+07 | **0.92** | 1 | - | 136 | 14.9 | 9.3 | Transport | Golgi apparatus;Cytoplasm | Auxiliary transport protein activity | Cytoplasm |
| **TPX2** | NP_036244.2 | 22974 | **targeting protein for Xklp2** | 2.1 | 1 | 1 | 1 | 1 | 6.21E+07 | **0.92** | 1 | - | 747 | 85.6 | 9.2 | Cell growth and/or maintenance;Spindle assembly | Nucleus;Microtubule | ATP binding | Nucleus |
| **AKAP8** | NP_005849.1 | 10270 | **A-kinase anchor protein 8** | 18.2 | 1 | 5 | 5 | 10 | 2.57E+07 | **0.92** | 3 | 10.4 | 692 | 76.1 | 5.2 | Cell communication;Signal transduction | Nucleus;Cytoplasm | Chromatin binding | Nucleus |
| **KIAA1671** | NP_001138678.1 | 85379 | **uncharacterized protein KIAA1671** | 5.8 | 1 | 5 | 5 | 6 | 4.45E+07 | **0.92** | 3 | 22.7 | 1806 | 196.6 | 8.5 | Biological_process unknown | - | Molecular function unknown | - |
| **PSMB1** | NP_002784.1 | 5689 | **proteasome subunit beta type-1** | 70.1 | 1 | 15 | 15 | 100 | 1.22E+09 | **0.92** | 31 | 12.6 | 241 | 26.5 | 8.1 | Protein metabolism | - | Ubiquitin-specific protease activity | - |

**Supplementary Table S4. List of all proteins identified in SCC-S and SCC-R cells using SequestHT and Mascot search algorithms and their gene ontology annotation**

| Gene Symbol | RefSeq_Accession | Entrez Gene ID | Description | Coverage (%) | Proteins | Unique Peptides | # Peptides | PSM | Area | SCC-R /SCC-S | SCC-R /SCC-S (Count) | SCC-R /SCC-S (Variability [%]) | # AAs | MW [kDa] | calc. pI | Biological Process | Localization | Molecular Function | Primary Localization |
|---|---|---|---|---|---|---|---|---|---|---|---|---|---|---|---|---|---|---|---|
| VPS53 | NP_001121631.1 | 55275 | vacuolar protein sorting-associated protein 53 homolog isoform 1 | 8.9 | 2 | 5 | 5 | 11 | 5.27E+07 | 0.92 | 5 | 123.2 | 832 | 94.3 | 6.7 | Biological_process unknown | - | Molecular function unknown | - |
| VTI1A | NP_660207.2 | 143187 | vesicle transport through interaction with t-SNAREs homolog 1A | 24.0 | 1 | 2 | 2 | 8 | 1.13E+08 | 0.92 | 4 | 33.6 | 217 | 25.2 | 6.4 | Transport | Integral to membrane | Transporter activity | Integral to membrane |
| DYNLL2 | NP_542408.1 | 140735 | dynein light chain 2, cytoplasmic | 62.9 | 1 | 3 | 5 | 8 | 1.21E+08 | 0.92 | 2 | 83 | 89 | 10.3 | 7.4 | Cell growth and/or maintenance | Cytoplasm | Motor activity | Cytoplasm |
| HOOK3 | NP_115786.1 | 84376 | protein Hook homolog 3 | 2.2 | 1 | 1 | 1 | 1 | 1.21E+07 | 0.92 | 1 | - | 718 | 83.1 | 5.2 | Cell growth and/or maintenance | Golgi apparatus;Nucleus;Cytoplasm;Endoplasmic reticulum | Cytoskeletal protein binding | Cytoplasm |
| METAP1 | NP_055958.2 | 23173 | methionine aminopeptidase 1 | 32.9 | 1 | 6 | 6 | 17 | 3.25E+08 | 0.92 | 8 | 34.6 | 386 | 43.2 | 7.2 | Protein metabolism | Nucleolus | Aminopeptidase activity | Nucleolus |
| TOR1AIP2 | NP_659471.1 | 163590 | torsin-1A-interacting protein 2 isoform b | 30.6 | 1 | 7 | 7 | 12 | 1.33E+08 | 0.92 | 8 | 26.9 | 470 | 51.2 | 5.0 | Biological_process unknown | Endoplasmic reticulum | Molecular function unknown | Endoplasmic reticulum |
| APOA1BP | NP_658985.2 | 128240 | NAD(P)H-hydrate epimerase precursor | 24.7 | 1 | 4 | 4 | 22 | 2.11E+08 | 0.92 | 9 | 37.6 | 288 | 31.7 | 7.7 | Biological_process unknown | Endoplasmic reticulum;Extracellular | Molecular function unknown | Endoplasmic reticulum |
| PAIP1 | NP_899152.1 | 10605 | polyadenylate-binding protein-interacting protein 1 isoform 3 | 25.6 | 1 | 1 | 5 | 16 | 2.29E+08 | 0.92 | 1 | - | 367 | 42 | 4.5 | Protein metabolism;Regulation of translation | Cytoplasm;Nucleus | Translation regulator activity;Protein binding | Cytoplasm |
| CCDC86 | NP_077003.1 | 79080 | coiled-coil domain-containing protein 86 | 12.5 | 1 | 2 | 2 | 6 | 5.78E+07 | 0.92 | 2 | 11.3 | 360 | 40.2 | 10.3 | Biological_process unknown | Nucleus;Cytoplasm | Molecular function unknown | Nucleus |
| CUL1 | NP_003583.2 | 8454 | cullin-1 | 14.7 | 1 | 9 | 9 | 18 | 8.54E+07 | 0.92 | 9 | 2.9 | 776 | 89.6 | 8.0 | Protein metabolism | Nucleus;Cytoplasm | Ubiquitin-specific protease activity | Nucleus |
| EMD | NP_000108.1 | 2010 | emerin | 53.2 | 1 | 7 | 7 | 20 | 1.91E+08 | 0.92 | 4 | 4 | 254 | 29 | 5.5 | Cell growth and/or maintenance | Nucleus;Plasma membrane;Cytoplasm;Endoplasmic reticulum;Mitochondrion | Structural molecule activity | Nucleus |
| HYOU1 | NP_006380.1 | 10525 | hypoxia up-regulated protein 1 precursor | 35.4 | 1 | 24 | 25 | 95 | 6.34E+08 | 0.92 | 34 | 11.3 | 999 | 111.3 | 5.2 | Protein metabolism | Endoplasmic reticulum;Cytoplasm;Mitochondrion | Chaperone activity | Endoplasmic reticulum |
| AIMP2 | NP_006294.2 | 7965 | aminoacyl tRNA synthase complex-interacting multifunctional protein 2 | 47.8 | 1 | 8 | 8 | 36 | 6.53E+08 | 0.91 | 8 | 19 | 320 | 35.3 | 8.2 | Protein metabolism | Cytoplasm;Nucleus | Translation regulator activity | Cytoplasm |
| ASH2L | NP_001269201.1 | 9070 | set1/Ash2 histone methyltransferase complex subunit ASH2 isoform d | 4.9 | 4 | 1 | 1 | 1 | 1.66E+07 | 0.91 | 1 | - | 489 | 55.3 | 8.1 | Regulation of nucleobase, nucleoside, nucleotide and nucleic acid metabolism | Nucleus | DNA binding | Nucleus |
| ATG16L1 | NP_001177196.1 | 55054 | autophagy-related protein 16-1 isoform 5 | 12.0 | 5 | 3 | 3 | 6 | 1.13E+08 | 0.91 | 2 | 13.5 | 491 | 54.5 | 6.1 | Transport | Cytosol;Integral to membrane | Intracellular transporter activity | Integral to membrane |
| CLPP | NP_006003.1 | 8192 | putative ATP-dependent Clp protease proteolytic subunit, mitochondrial precursor | 28.2 | 1 | 4 | 4 | 10 | 1.74E+08 | 0.91 | 4 | 23.1 | 277 | 30.2 | 8.1 | Protein metabolism | Mitochondrion | Peptidase activity | Mitochondrion |
| ERC1 | NP_829883.1 | 23085 | ELKS/Rab6-interacting/CAST family member 1 isoform delta | 18.3 | 3 | 14 | 14 | 28 | 1.62E+08 | 0.91 | 11 | 23.9 | 1088 | 124.8 | 6.1 | Energy pathways | Cytoplasm;Golgi apparatus | Protein binding | Cytoplasm |
| SEC24B | NP_001036199.1 | 10427 | protein transport protein Sec24B isoform b | 23.1 | 2 | 15 | 15 | 22 | 9.41E+07 | 0.91 | 11 | 22.7 | 1233 | 133.5 | 7.4 | Transport | Cytoplasm | Transporter activity | Cytoplasm |
| TRMT1L | NP_001189352.1 | 81627 | TRMT1-like protein isoform 2 | 18.4 | 2 | 6 | 6 | 10 | 4.88E+07 | 0.91 | 3 | 9.3 | 577 | 65 | 8.5 | Regulation of nucleobase, nucleoside, nucleotide and nucleic acid metabolism | Nucleus;Nucleolus | DNA binding | Nucleus |
| ARIH1 | NP_005735.2 | 25820 | E3 ubiquitin-protein ligase ARIH1 | 11.7 | 1 | 4 | 4 | 6 | 1.07E+08 | 0.91 | 1 | - | 557 | 64.1 | 5.1 | Protein metabolism | Nucleus;Cytoplasm | Ubiquitin-specific protease activity | Nucleus |
| G3BP1 | NP_938405.1 | 10146 | ras GTPase-activating protein-binding protein 1 | 59.0 | 1 | 17 | 18 | 148 | 2.11E+09 | 0.91 | 39 | 21.7 | 466 | 52.1 | 5.5 | Regulation of nucleobase, nucleoside, nucleotide and nucleic acid metabolism;Signal transduction | Cytosol;Nucleus;Plasma membrane;Cytoplasm | Ribonuclease activity;RNA binding | Cytosol |
| KIAA0196 | NP_055661.3 | 9897 | WASH complex subunit strumpellin | 12.1 | 1 | 9 | 9 | 17 | 1.32E+08 | 0.91 | 9 | 7.6 | 1159 | 134.2 | 7.0 | Biological_process unknown | - | Molecular function unknown | - |
| EXOSC5 | NP_064543.3 | 56915 | exosome complex component RRP46 | 10.2 | 1 | 1 | 1 | 1 | 3.00E+07 | 0.91 | 1 | - | 235 | 25.2 | 7.6 | Regulation of nucleobase, nucleoside, nucleotide and nucleic acid metabolism | Nucleolus;Nucleus;Cytoplasm | Ribonuclease activity | Nucleolus |
| GLG1 | NP_001139139.1 | 2734 | Golgi apparatus protein 1 isoform 3 precursor | 17.9 | 3 | 13 | 13 | 30 | 3.00E+08 | 0.91 | 11 | 20.4 | 1179 | 134.5 | 6.9 | Biological_process unknown | Golgi apparatus;Plasma membrane;Mitochondrion | Receptor binding | Golgi apparatus |
| PABPN1 | NP_004634.1 | 8106 | polyadenylate-binding protein 2 | 40.5 | 3 | 7 | 7 | 18 | 6.49E+08 | 0.91 | 5 | 17.8 | 306 | 32.7 | 5.1 | Regulation of nucleobase, nucleoside, nucleotide and nucleic acid metabolism | Nucleus;Nucleoplasm | RNA binding | Nucleus |
| RPS2 | NP_002943.2 | 6187 | 40S ribosomal protein S2 | 41.0 | 1 | 13 | 13 | 87 | 2.86E+09 | 0.91 | 22 | 22.7 | 293 | 31.3 | 10.2 | Protein metabolism | Ribosome;Nucleolus;Mitochondrion;Cytoplasm | Structural constituent of ribosome | Ribosome |
| CNOT3 | NP_055331.1 | 4849 | CCR4-NOT transcription complex subunit 3 | 4.9 | 1 | 3 | 3 | 3 | 1.68E+07 | 0.91 | 2 | 123 | 753 | 81.8 | 6.2 | Regulation of nucleobase, nucleoside, nucleotide and nucleic acid metabolism | Nucleus | Transcription regulator activity | Nucleus |
| IARS | NP_002152.2 | 3376 | isoleucine--tRNA ligase, cytoplasmic | 34.2 | 1 | 29 | 29 | 85 | 8.96E+08 | 0.91 | 26 | 11.2 | 1262 | 144.4 | 6.2 | Protein metabolism | Cytoplasm;Mitochondrion;Ribosome | Ligase activity | Cytoplasm |
| ADRBK1 | NP_001610.2 | 156 | beta-adrenergic receptor kinase 1 | 6.1 | 1 | 2 | 2 | 2 | 4.25E+06 | 0.91 | 1 | - | 689 | 79.5 | 7.3 | Cell communication;Signal transduction | Cytoplasm;Plasma membrane | Protein serine/threonine kinase activity | Cytoplasm |
| BLOC1S6 | NP_036520.1 | 26258 | biogenesis of lysosome-related organelles complex 1 subunit 6 | 11.1 | 1 | 1 | 1 | 3 | 1.04E+08 | 0.91 | 1 | - | 172 | 19.7 | 6.4 | Cell growth and/or maintenance | Cytoplasmic vesicle;Plasma membrane | Molecular function unknown | Cytoplasmic vesicle |
| PDHA1 | NP_000275.1 | 5160 | pyruvate dehydrogenase E1 component subunit alpha, somatic form, mitochondrial isoform 1 precursor | 44.1 | 5 | 12 | 12 | 29 | 2.80E+08 | 0.91 | 7 | 11.3 | 390 | 43.3 | 8.1 | Metabolism;Energy pathways | Mitochondrion;Nucleus | Catalytic activity | Mitochondrion |
| FTSJ3 | NP_060117.3 | 117246 | pre-rRNA processing protein FTSJ3 | 27.2 | 1 | 15 | 15 | 36 | 2.13E+08 | 0.91 | 12 | 8.6 | 847 | 96.5 | 8.4 | Biological_process unknown | Nucleolus;Nucleus;Cytoplasm | Molecular function unknown | Nucleolus |
| HSPD1 | NP_002147.2 | 3329 | 60 kDa heat shock protein, mitochondrial | 83.4 | 1 | 62 | 62 | 1882 | 8.77E+09 | 0.91 | 235 | 15.4 | 573 | 61 | 5.9 | Protein metabolism;Protein folding;Apoptosis;Regulation of immune response;Signal transduction | Mitochondrion;Cytoplasm;Plasma membrane;Mitochondrial matrix;Endoplasmic reticulum;Peroxisomal matrix;Secretory | Heat shock protein activity | Mitochondrial matrix |
| MRPL16 | NP_060310.1 | 54948 | 39S ribosomal protein L16, mitochondrial | 19.9 | 1 | 3 | 3 | 6 | 9.54E+07 | 0.91 | 3 | 63.6 | 251 | 28.4 | 10.1 | Protein metabolism | Mitochondrion | Structural constituent of ribosome | Mitochondrion |
| PSMB2 | NP_002785.1 | 5690 | proteasome subunit beta type-2 isoform 1 | 64.2 | 3 | 10 | 10 | 41 | 1.80E+09 | 0.91 | 10 | 15.9 | 201 | 22.8 | 7.0 | Protein metabolism | Cytoplasm | Ubiquitin-specific protease activity | Cytoplasm |
| TRIM28 | NP_005753.1 | 10155 | transcription intermediary factor 1-beta | 66.6 | 1 | 28 | 28 | 179 | 2.01E+09 | 0.91 | 40 | 24.2 | 835 | 88.5 | 5.8 | Regulation of nucleobase, nucleoside, nucleotide and nucleic acid metabolism | Nucleus;Nucleolus;Mitochondrion | Transcription regulator activity | Nucleus |
| DNAJC7 | NP_003306.3 | 7266 | dnaJ homolog subfamily C member 7 isoform 1 | 23.9 | 2 | 8 | 8 | 22 | 1.15E+08 | 0.91 | 13 | 13.1 | 494 | 56.4 | 7.0 | Protein metabolism;Protein folding | Cytoplasm;Nucleus | Chaperone activity | Cytoplasm |

| Gene Symbol | RefSeq_Accession | Entrez Gene ID | Description | Coverage (%) | Proteins | Unique Peptides | # Peptides | PSM | Area | SCC-R /SCC-S | SCC-R /SCC-S (Count) | SCC-R /SCC-S (Variability [%]) | # AAs | MW [kDa] | calc. pI | Biological Process | Localization | Molecular Function | Primary Localization |
|---|---|---|---|---|---|---|---|---|---|---|---|---|---|---|---|---|---|---|---|
| **EIF3B** | NP_003742.2 | 8662 | **eukaryotic translation initiation factor 3 subunit B** | 45.0 | 1 | 25 | 25 | 87 | 1.05E+09 | **0.91** | 30 | 25.5 | 814 | 92.4 | 5.0 | Protein metabolism | Ribosome;Nucleus;Cytoplasm | Translation regulator activity | Ribosome |
| **NUP205** | NP_055950.1 | 23165 | **nuclear pore complex protein Nup205** | 16.4 | 1 | 19 | 19 | 34 | 2.78E+08 | **0.91** | 17 | 23 | 2012 | 227.8 | 6.2 | Biological_process unknown | Nucleus;Nuclear membrane | Molecular function unknown | Nucleus |
| **PAWR** | NP_002574.2 | 5074 | **PRKC apoptosis WT1 regulator protein** | 9.4 | 1 | 3 | 3 | 5 | 2.26E+07 | **0.91** | 3 | 22.6 | 340 | 36.5 | 5.4 | Regulation of nucleobase, nucleoside, nucleotide and nucleic acid metabolism | Nucleus;Cytoplasm;Endoplasmic reticulum;Plasma membrane | Transcription regulator activity | Nucleus |
| **RTCB** | NP_055121.1 | 51493 | **tRNA-splicing ligase RtcB homolog** | 46.3 | 1 | 18 | 18 | 67 | 5.20E+08 | **0.91** | 25 | 21.1 | 505 | 55.2 | 7.2 | Biological_process unknown | - | Molecular function unknown | - |
| **SPATA5** | NP_660208.2 | 166378 | **spermatogenesis-associated protein 5** | 13.9 | 1 | 6 | 7 | 13 | 2.40E+08 | **0.91** | 5 | 13 | 893 | 97.8 | 5.7 | Metabolism;Energy pathways | Mitochondrion | ATPase activity | Mitochondrion |
| **SSB** | NP_003133.1 | 6741 | **lupus La protein** | 65.2 | 1 | 27 | 27 | 116 | 9.02E+08 | **0.91** | 33 | 16.1 | 408 | 46.8 | 7.1 | Regulation of nucleobase, nucleoside, nucleotide and nucleic acid metabolism | Nucleus;Plasma membrane;Cytoplasm | RNA binding | Nucleus |
| **CCDC43** | NP_001092695.1 | 124808 | **coiled-coil domain-containing protein 43 isoform 2** | 10.4 | 2 | 1 | 1 | 1 | 6.79E+07 | **0.91** | 1 | - | 154 | 16.7 | 4.5 | Biological_process unknown | - | Molecular function unknown | - |
| **CKS2** | NP_001818.1 | 1164 | **cyclin-dependent kinases regulatory subunit 2** | 7.6 | 1 | 1 | 1 | 1 | 4.36E+07 | **0.91** | 1 | - | 79 | 9.9 | 8.5 | Cell communication;Signal transduction | - | Molecular function unknown | - |
| **ELF1** | NP_001138825.1 | 1997 | **ETS-related transcription factor Elf-1 isoform b** | 3.0 | 2 | 1 | 1 | 1 | 4.43E+07 | **0.91** | 1 | - | 595 | 64.9 | 5.4 | Regulation of nucleobase, nucleoside, nucleotide and nucleic acid metabolism | Nucleus;Cytoplasm | Transcription factor activity | Nucleus |
| **PPP1R7** | NP_002703.1 | 5510 | **protein phosphatase 1 regulatory subunit 7 isoform 1** | 37.2 | 7 | 9 | 9 | 24 | 1.58E+08 | **0.91** | 8 | 18.3 | 360 | 41.5 | 4.9 | Regulation of nucleobase, nucleoside, nucleotide and nucleic acid metabolism | Nucleus;Cytoplasm | Protein serine/threonine phosphatase activity | Nucleus |
| **FAM203A** | NP_057542.2 | 51236 | **protein FAM203A** | 15.1 | 1 | 3 | 3 | 13 | 5.41E+07 | **0.91** | 6 | 15.4 | 390 | 42.1 | 4.8 | Biological_process unknown | - | Molecular function unknown | - |
| **NAA40** | NP_079047.2 | 79829 | **N-alpha-acetyltransferase 40** | 5.1 | 1 | 1 | 1 | 2 | 1.04E+08 | **0.91** | 1 | - | 237 | 27.2 | 7.4 | Metabolism | - | Molecular function unknown | - |
| **NFU1** | NP_001002756.1 | 27247 | **NFU1 iron-sulfur cluster scaffold homolog, mitochondrial isoform 3** | 36.3 | 3 | 2 | 2 | 6 | 4.68E+07 | **0.91** | 2 | 105.6 | 113 | 12.3 | 4.0 | Biological_process unknown | Mitochondrion;Cytosol;Nucleus | Molecular function unknown | Mitochondrion;Cytosol |
| **TOMM40** | NP_001122389.1 | 10452 | **mitochondrial import receptor subunit TOM40 homolog** | 27.2 | 1 | 5 | 5 | 17 | 1.36E+08 | **0.91** | 4 | 14.6 | 361 | 37.9 | 7.3 | Transport | Mitochondrion | Transporter activity | Mitochondrion |
| **COPS5** | NP_006828.2 | 10987 | **COP9 signalosome complex subunit 5** | 19.5 | 1 | 5 | 5 | 10 | 4.72E+07 | **0.90** | 4 | 37.7 | 334 | 37.6 | 6.5 | Protein metabolism | Nucleus;Cytoplasm;Plasma membrane | Ubiquitin-specific protease activity | Nucleus |
| **FARSB** | NP_005678.3 | 10056 | **phenylalanine--tRNA ligase beta subunit** | 33.5 | 1 | 14 | 14 | 28 | 3.38E+08 | **0.90** | 16 | 14.1 | 589 | 66.1 | 6.8 | Protein metabolism | Cytoplasm;Nucleolus | Ligase activity | Cytoplasm |
| **RNASEH2C** | NP_115569.2 | 84153 | **ribonuclease H2 subunit C** | 23.8 | 1 | 2 | 2 | 7 | 6.76E+07 | **0.90** | 1 | - | 164 | 17.8 | 5.0 | Biological_process unknown | - | Molecular function unknown | - |
| **CNTNAP4** | NP_620481.2 | 85445 | **contactin-associated protein-like 4 isoform 2** | 0.6 | 2 | 1 | 1 | 9 | 1.15E+09 | **0.90** | 4 | 12.3 | 1235 | 137.4 | 6.6 | Cell communication;Signal transduction;Cell recognition | - | Cell adhesion molecule activity | - |
| **AGL** | NP_000636.2 | 178 | **glycogen debranching enzyme isoform 2** | 12.2 | 3 | 9 | 9 | 11 | 1.02E+08 | **0.90** | 6 | 13.2 | 1515 | 172.5 | 6.7 | Carbohydrate metabolism;Energy pathways | Cytoplasm;Extracellular | Glucosidase activity | Cytoplasm |
| **CAB39** | NP_001124321.1 | 51719 | **calcium-binding protein 39** | 18.8 | 1 | 5 | 5 | 13 | 2.18E+08 | **0.90** | 7 | 38.5 | 341 | 39.8 | 6.9 | Biological_process unknown | - | Calcium ion binding | - |
| **DNM3** | NP_001265181.1 | 26052 | **dynamin-3 isoform c** | 11.9 | 3 | 1 | 5 | 15 | 9.78E+07 | **0.90** | 2 | 7.8 | 555 | 62.8 | 8.7 | Cell communication;Signal transduction | - | GTPase activity | - |
| **HTATIP2** | NP_001091990.1 | 10553 | **oxidoreductase HTATIP2 isoform a precursor** | 30.4 | 3 | 7 | 7 | 10 | 1.05E+08 | **0.90** | 5 | 20.5 | 276 | 30.1 | 8.4 | Regulation of nucleobase, nucleoside, nucleotide and nucleic acid metabolism | Nucleus | Transcription regulator activity | Nucleus |
| **NQO1** | NP_001020604.1 | 1728 | **NAD(P)H dehydrogenase [quinone] 1 isoform b** | 45.8 | 4 | 13 | 13 | 59 | 1.23E+09 | **0.90** | 20 | 15.6 | 240 | 27.3 | 8.7 | Metabolism;Energy pathways;Synaptic transmission;Xenobiotic metabolism | Mitochondrion;Microsome;Plasma membrane;Cytoplasm;Nucleus | Oxidoreductase activity | Mitochondrion |
| **TSSC1** | NP_003301.1 | 7260 | **protein TSSC1** | 5.2 | 1 | 1 | 1 | 1 | 1.04E+08 | **0.90** | 1 | - | 387 | 43.6 | 5.1 | Biological_process unknown | - | Molecular function unknown | - |
| **ARFGEF1** | NP_006412.2 | 10565 | **brefeldin A-inhibited guanine nucleotide-exchange protein 1** | 11.3 | 1 | 8 | 12 | 18 | 1.03E+08 | **0.90** | 6 | 18.7 | 1849 | 208.6 | 5.9 | Cell communication;Signal transduction | Golgi apparatus | Guanyl-nucleotide exchange factor activity | Golgi apparatus |
| **DHX40** | NP_001159773.1 | 79665 | **probable ATP-dependent RNA helicase DHX40 isoform 2** | 1.7 | 2 | 1 | 1 | 1 | 5.44E+06 | **0.90** | 1 | - | 702 | 80.2 | 8.5 | Regulation of nucleobase, nucleoside, nucleotide and nucleic acid metabolism | Nucleus | ATPase activity | Nucleus |
| **SEC61A1** | NP_037468.1 | 29927 | **protein transport protein Sec61 subunit alpha isoform 1** | 8.8 | 4 | 4 | 4 | 26 | 3.09E+08 | **0.90** | 7 | 43.2 | 476 | 52.2 | 8.1 | Transport | Endoplasmic reticulum;Golgi apparatus;Integral to membrane | Protein translocase activity;Intracellular transporter activity | Endoplasmic reticulum |
| **TSG101** | NP_006283.1 | 7251 | **tumor susceptibility gene 101 protein** | 20.3 | 1 | 5 | 5 | 9 | 1.78E+08 | **0.90** | 6 | 23.5 | 390 | 43.9 | 6.5 | Protein metabolism | Cytoplasm;Nucleus;Golgi apparatus | Ubiquitin-specific protease activity | Cytoplasm |
| **ACBD5** | NP_001035938.1 | 91452 | **acyl-CoA-binding domain-containing protein 5 isoform 2** | 3.5 | 3 | 1 | 1 | 2 | 1.01E+08 | **0.90** | 1 | - | 490 | 54.8 | 5.2 | Biological_process unknown | Nucleus | Molecular function unknown | Nucleus |
| **ANAPC1** | NP_073153.1 | 64682 | **anaphase-promoting complex subunit 1** | 7.5 | 5 | 8 | 8 | 10 | 7.56E+07 | **0.90** | 4 | 3.8 | 1944 | 216.4 | 6.3 | Cell communication;Signal transduction | Chromosome;Centrosome;Microtubule | Protein binding | Chromosome |
| **KIF13B** | NP_056069.2 | 23303 | **kinesin-like protein KIF13B** | 1.5 | 1 | 1 | 2 | 4 | 2.55E+07 | **0.90** | 1 | - | 1826 | 202.7 | 5.9 | Cell growth and/or maintenance | Cytoplasm | Motor activity | Cytoplasm |
| **LOC101928852** | XP_005255809.1 | 101928852 | **protein FRG2-like-2-like** | 6.7 | 1 | 1 | 1 | 1 | 7.70E+07 | **0.90** | 1 | - | 120 | 13.6 | 9.3 | - | - | - | - |
| **NPHS1** | NP_004637.1 | 4868 | **nephrin precursor** | 0.6 | 1 | 1 | 1 | 1 | 1.07E+07 | **0.90** | 1 | - | 1241 | 134.7 | 5.6 | Cell communication;Signal transduction | Plasma membrane;Endoplasmic reticulum;Extracellular | Cell adhesion molecule activity | Plasma membrane |
| **CSNK2A2** | NP_001887.1 | 1459 | **casein kinase II subunit alpha'** | 52.0 | 1 | 13 | 14 | 37 | 3.15E+08 | **0.90** | 11 | 15.3 | 350 | 41.2 | 8.6 | Cell communication;Signal transduction | Nucleus;Cytoplasm;Plasma membrane;Nucleolus | Protein serine/threonine kinase activity | Nucleus |
| **IMMT** | NP_001093640.1 | 10989 | **mitochondrial inner membrane protein isoform 3** | 42.8 | 3 | 24 | 24 | 65 | 2.80E+08 | **0.90** | 29 | 30.5 | 747 | 82.6 | 6.6 | Cell growth and/or maintenance | Mitochondrion | Motor activity | Mitochondrion |
| **MED15** | NP_056973.2 | 51586 | **mediator of RNA polymerase II transcription subunit 15 isoform b** | 23.1 | 2 | 7 | 7 | 15 | 1.22E+08 | **0.90** | 9 | 38 | 748 | 82.5 | 9.3 | Biological_process unknown | Cytoplasm;Nucleus;Nucleoplasm;Nucleolus | Transcription regulator activity | Cytoplasm |
| **MIF** | NP_002406.1 | 4282 | **macrophage migration inhibitory factor** | 88.7 | 1 | 5 | 5 | 53 | 3.81E+09 | **0.90** | 23 | 46.1 | 115 | 12.5 | 7.9 | Cell communication;Signal transduction | Extracellular;Cytoplasm | Cytokine activity | Extracellular |

| Gene Symbol | RefSeq_Accession | Entrez Gene ID | Description | Coverage (%) | Proteins | Unique Peptides | # Peptides | PSM | Area | SCC-R /SCC-S | SCC-R /SCC-S (Count) | SCC-R /SCC-S (Variability [%]) | # AAs | MW [kDa] | calc. pI | Biological Process | Localization | Molecular Function | Primary Localization |
|---|---|---|---|---|---|---|---|---|---|---|---|---|---|---|---|---|---|---|---|
| **MRPS23** | NP_057154.2 | 51649 | **28S ribosomal protein S23, mitochondrial** | 17.9 | 1 | 2 | 2 | 2 | 6.74E+07 | **0.90** | 2 | 21 | 190 | 21.8 | 8.9 | Protein metabolism | Mitochondrion | Structural constituent of ribosome | Mitochondrion |
| **RPA1** | NP_002936.1 | 6117 | **replication protein A 70 kDa DNA-binding subunit** | 40.1 | 1 | 17 | 17 | 43 | 1.83E+08 | **0.90** | 15 | 35.8 | 616 | 68.1 | 7.2 | Regulation of nucleobase, nucleoside, nucleotide and nucleic acid metabolism | Nucleus;Cytoplasm | DNA binding | Nucleus |
| **MARCH5** | NP_060294.1 | 54708 | **E3 ubiquitin-protein ligase MARCH5** | 5.8 | 1 | 1 | 1 | 1 | 3.11E+07 | **0.90** | 1 | - | 278 | 31.2 | 8.7 | Biological_process unknown | Mitochondrial membrane;Mitochondrion;Integral to membrane | Ligase activity | Mitochondrial membrane |
| **ARHGAP23** | NP_001186346.1 | 57636 | **rho GTPase-activating protein 23** | 1.1 | 1 | 1 | 1 | 1 | 3.95E+07 | **0.90** | 1 | - | 1491 | 162.1 | 9.2 | - | - | - | - |
| **CDC5L** | NP_001244.1 | 988 | **cell division cycle 5-like protein** | 49.3 | 1 | 26 | 26 | 57 | 6.61E+08 | **0.90** | 22 | 29.4 | 802 | 92.2 | 8.2 | Cell communication;Signal transduction | Nucleus;Cytoplasm | Protein binding | Nucleus |
| **GRWD1** | NP_113673.3 | 83743 | **glutamate-rich WD repeat-containing protein 1** | 26.7 | 1 | 5 | 5 | 10 | 6.63E+07 | **0.90** | 5 | 20.1 | 446 | 49.4 | 4.9 | Cell growth and/or maintenance | Nucleus;Nucleolus;Cytoplasm | Molecular function unknown | Nucleus |
| **HSBP1** | NP_001528.1 | 3281 | **heat shock factor-binding protein 1** | 40.8 | 1 | 1 | 1 | 2 | 1.61E+08 | **0.90** | 1 | - | 76 | 8.5 | 4.4 | Regulation of nucleobase, nucleoside, nucleotide and nucleic acid metabolism | Nucleus | Transcription regulator activity | Nucleus |
| **LETM1** | NP_036450.1 | 3954 | **LETM1 and EF-hand domain-containing protein 1, mitochondrial precursor** | 27.6 | 1 | 12 | 12 | 31 | 5.47E+08 | **0.90** | 13 | 18.7 | 739 | 83.3 | 6.7 | Cell communication;Signal transduction | Plasma membrane;Mitochondrion | Calcium ion binding | Plasma membrane |
| **PTMA** | NP_002814.3 | 5757 | **prothymosin alpha isoform 2** | 40.9 | 4 | 9 | 9 | 225 | 6.13E+09 | **0.90** | 24 | 13.3 | 110 | 12.1 | 3.8 | Cell proliferation | Nucleus;Cytoplasm;Perinuclear region | Molecular function unknown | Nucleus |
| **SYF2** | NP_056299.1 | 25949 | **pre-mRNA-splicing factor SYF2 isoform 1** | 23.9 | 2 | 3 | 3 | 4 | 2.99E+07 | **0.90** | 3 | 0.7 | 243 | 28.7 | 8.4 | Biological_process unknown | Nucleus | Molecular function unknown | Nucleus |
| **SIN3A** | NP_056292.1 | 25942 | **paired amphipathic helix protein Sin3a** | 10.4 | 1 | 7 | 7 | 12 | 1.36E+08 | **0.90** | 5 | 65.6 | 1273 | 145.1 | 7.3 | Regulation of nucleobase, nucleoside, nucleotide and nucleic acid metabolism | Cytoplasm;Nucleus | Transcription regulator activity;DNA binding | Nucleus |
| **ACTR1A** | NP_005727.1 | 10121 | **alpha-centractin** | 56.1 | 1 | 11 | 14 | 60 | 3.66E+08 | **0.89** | 13 | 18.6 | 376 | 42.6 | 6.6 | Cell growth and/or maintenance | Cytoplasm;Centrosome;Kinetochore;Microtubule | Structural constituent of cytoskeleton | Centrosome |
| **BRE** | NP_954664.1 | 9577 | **BRCA1-A complex subunit BRE isoform 2** | 21.4 | 4 | 4 | 4 | 6 | 7.23E+07 | **0.89** | 2 | 18.2 | 383 | 43.5 | 5.8 | Cell proliferation;Regulation of signal transduction | Cytosol;Nucleus | Binding | Cytosol |
| **NCAPD2** | NP_055680.3 | 9918 | **condensin complex subunit 1** | 34.6 | 1 | 31 | 31 | 62 | 3.63E+08 | **0.89** | 29 | 22.8 | 1401 | 157.1 | 6.6 | Regulation of nucleobase, nucleoside, nucleotide and nucleic acid metabolism | Nucleus;Cytoplasm;Microtubule;Mitochondrion | DNA binding | Cytoplasm |
| **AAR2** | NP_001258803.1 | 25980 | **protein AAR2 homolog** | 16.2 | 1 | 4 | 4 | 7 | 2.85E+07 | **0.89** | 2 | 6.5 | 384 | 43.4 | 6.0 | Biological_process unknown | - | Molecular function unknown | - |
| **SEPHS1** | NP_036379.2 | 22929 | **selenide, water dikinase 1 isoform 1** | 36.0 | 3 | 6 | 6 | 19 | 2.45E+08 | **0.89** | 6 | 33.5 | 392 | 42.9 | 6.0 | Metabolism;Energy pathways | - | ATP binding | - |
| **SPTAN1** | NP_003118.2 | 6709 | **spectrin alpha chain, non-erythrocytic 1 isoform 2** | 48.6 | 3 | 83 | 83 | 281 | 7.14E+08 | **0.89** | 83 | 25.7 | 2472 | 284.4 | 5.4 | Cell growth and/or maintenance | Cytoplasm;Plasma membrane;Cytosol;Nucleus | Structural constituent of cytoskeleton | Cytoplasm;Nucleus |
| **PIP5K1A** | NP_001129109.1 | 8394 | **phosphatidylinositol 4-phosphate 5-kinase type-1 alpha isoform 4** | 2.8 | 4 | 1 | 1 | 2 | 4.32E+07 | **0.89** | 1 | - | 500 | 56 | 7.5 | Cell communication;Signal transduction | Plasma membrane;Nucleus;Golgi apparatus;Cytoplasm;Cytosol;Microtubule;Actin filament | Lipid kinase activity | Plasma membrane |
| **FAM162A** | NP_055182.3 | 26355 | **protein FAM162A** | 11.7 | 1 | 2 | 2 | 3 | 5.58E+07 | **0.89** | 2 | 40.7 | 154 | 17.3 | 9.8 | Biological_process unknown | - | Molecular function unknown | - |
| **LOC101930154** | XP_006726924.1 | 101930154 | **WAS protein family homolog 6-like** | 5.8 | 1 | 1 | 2 | 5 | 6.05E+06 | **0.89** | 2 | 5.8 | 451 | 48.3 | 6.0 | - | - | - | - |
| **MRPL19** | NP_055578.2 | 9801 | **39S ribosomal protein L19, mitochondrial** | 3.1 | 1 | 1 | 1 | 1 | 3.17E+07 | **0.89** | 1 | - | 292 | 33.5 | 9.5 | Protein metabolism | Mitochondrion | Structural constituent of ribosome | Mitochondrion |
| **PITHD1** | NP_065095.2 | 57095 | **PITH domain-containing protein 1** | 23.7 | 1 | 3 | 3 | 4 | 6.31E+07 | **0.89** | 2 | 1.2 | 211 | 24.2 | 5.7 | Biological_process unknown | - | Molecular function unknown | - |
| **SLC35B2** | NP_001273440.1 | 347734 | **adenosine 3'-phospho 5'-phosphosulfate transporter 1 isoform c** | 11.5 | 6 | 4 | 4 | 7 | 9.98E+07 | **0.89** | 3 | 29.7 | 383 | 42.1 | 9.4 | Transport | Golgi apparatus | Auxiliary transport protein activity | Golgi apparatus |
| **ADCK3** | NP_064632.2 | 56997 | **chaperone activity of bc1 complex-like, mitochondrial** | 1.9 | 1 | 1 | 1 | 2 | 4.05E+07 | **0.89** | 1 | - | 647 | 71.9 | 7.0 | Apoptosis | Mitochondrion | Chaperone activity | Mitochondrion |
| **DCUN1D5** | NP_115675.1 | 84259 | **DCN1-like protein 5** | 32.5 | 1 | 4 | 4 | 5 | 5.93E+07 | **0.89** | 2 | 23.9 | 237 | 27.5 | 5.6 | Biological_process unknown | - | Molecular function unknown | - |
| **DPP3** | NP_005691.2 | 10072 | **dipeptidyl peptidase 3 isoform 1** | 43.0 | 2 | 20 | 20 | 97 | 1.04E+09 | **0.89** | 26 | 24.9 | 737 | 82.5 | 5.1 | Protein metabolism | Cytoplasm | Aminopeptidase activity | Cytoplasm |
| **DPY30** | NP_115963.1 | 84661 | **protein dpy-30 homolog** | 76.8 | 1 | 4 | 4 | 60 | 2.81E+08 | **0.89** | 18 | 13.3 | 99 | 11.2 | 4.9 | Biological_process unknown | - | Molecular function unknown | - |
| **NARS** | NP_004530.1 | 4677 | **asparagine--tRNA ligase, cytoplasmic** | 19.0 | 1 | 8 | 8 | 18 | 4.29E+08 | **0.89** | 9 | 7.6 | 548 | 62.9 | 6.3 | Protein metabolism | Cytoplasm | Ligase activity | Cytoplasm |
| **SASS6** | NP_919268.1 | 163786 | **spindle assembly abnormal protein 6 homolog** | 1.1 | 1 | 1 | 1 | 2 | 1.01E+07 | **0.89** | 1 | - | 657 | 74.4 | 7.6 | Biological_process unknown | - | Molecular function unknown | - |
| **SCLT1** | NP_653244.2 | 132320 | **sodium channel and clathrin linker 1** | 1.2 | 1 | 1 | 1 | 1 | 1.02E+08 | **0.89** | 1 | - | 688 | 80.9 | 6.1 | Regulation of endocytosis | Golgi apparatus;Lysosome | Protein channel activity | Golgi apparatus |
| **TUFM** | NP_003312.3 | 7284 | **elongation factor Tu, mitochondrial precursor** | 66.2 | 1 | 31 | 31 | 163 | 2.24E+09 | **0.89** | 58 | 13.9 | 455 | 49.8 | 7.6 | Protein metabolism | Mitochondrion;Nucleolus | Translation regulator activity | Mitochondrion |
| **FKBP14** | NP_060416.1 | 55033 | **peptidyl-prolyl cis-trans isomerase FKBP14 precursor** | 5.7 | 1 | 1 | 1 | 1 | 6.14E+07 | **0.89** | 1 | - | 211 | 24.2 | 6.1 | Metabolism;Energy pathways | Endoplasmic reticulum | Isomerase activity | Endoplasmic reticulum |
| **GNB2L1** | NP_006089.1 | 10399 | **guanine nucleotide-binding protein subunit beta-2-like 1** | 79.5 | 1 | 25 | 25 | 288 | 2.59E+09 | **0.89** | 52 | 15.3 | 317 | 35.1 | 7.7 | Cell communication;Signal transduction | Cytoplasm;Nucleus;Plasma membrane;Cytoskeleton | Receptor signaling complex scaffold activity | Cytoplasm |
| **MPHOSPH10** | NP_005782.1 | 10199 | **U3 small nucleolar ribonucleoprotein protein MPP10** | 18.1 | 1 | 7 | 7 | 15 | 1.22E+08 | **0.89** | 8 | 28.9 | 681 | 78.8 | 4.9 | Regulation of nucleobase, nucleoside, nucleotide and nucleic acid metabolism | Nucleolus;Cytoplasm;Nucleus | Ribonucleoprotein | Nucleolus |
| **NDUFA13** | NP_057049.5 | 51079 | **NADH dehydrogenase [ubiquinone] 1 alpha subcomplex subunit 13** | 35.4 | 1 | 3 | 4 | 9 | 1.50E+08 | **0.89** | 5 | 18.3 | 144 | 16.7 | 8.4 | Metabolism;Energy pathways | Mitochondrion;Nucleus;Perinuclear region;Cytoplasm | Oxidoreductase activity | Mitochondrion |
| **PSMA6** | NP_002782.1 | 5687 | **proteasome subunit alpha type-6 isoform a** | 63.0 | 3 | 13 | 13 | 125 | 7.65E+08 | **0.89** | 25 | 11.5 | 246 | 27.4 | 6.8 | Protein metabolism | Cytoplasm;Nucleus;Ribosome | Ubiquitin-specific protease activity | Cytoplasm |
| **SAFB** | NP_002958.2 | 6294 | **scaffold attachment factor B1 isoform 3** | 29.8 | 4 | 12 | 22 | 63 | 4.35E+08 | **0.89** | 13 | 7.1 | 915 | 102.6 | 5.5 | Regulation of nucleobase, nucleoside, nucleotide and nucleic acid metabolism | Nucleus;Cytoplasm | Transcription factor activity | Nucleus |

| Gene Symbol | RefSeq_Accession | Entrez Gene ID | Description | Coverage (%) | Proteins | Unique Peptides | # Peptides | PSM | Area | SCC-R /SCC-S | SCC-R /SCC-S (Count) | SCC-R /SCC-S (Variability [%]) | # AAs | MW [kDa] | calc. pI | Biological Process | Localization | Molecular Function | Primary Localization |
|---|---|---|---|---|---|---|---|---|---|---|---|---|---|---|---|---|---|---|---|
| TUBB6 | NP_115914.1 | 84617 | tubulin beta-6 chain | 79.8 | 1 | 15 | 30 | 405 | 4.25E+09 | 0.89 | 39 | 27.4 | 446 | 49.8 | 4.9 | Cell growth and/or maintenance | - | Structural constituent of cytoskeleton | - |
| USP7 | NP_003461.2 | 7874 | ubiquitin carboxyl-terminal hydrolase 7 isoform 1 | 23.6 | 3 | 15 | 15 | 30 | 2.57E+08 | 0.89 | 13 | 10.4 | 1102 | 128.2 | 5.6 | Protein metabolism | Nucleus;Cytosol | Ubiquitin-specific protease activity | Nucleus |
| ELAVL1 | NP_001410.2 | 1994 | ELAV-like protein 1 | 60.4 | 8 | 16 | 16 | 61 | 1.86E+09 | 0.89 | 20 | 13.5 | 326 | 36.1 | 9.2 | Regulation of nucleobase, nucleoside, nucleotide and nucleic acid metabolism | Cytoplasm;Nucleus;Nucleolus;Mitochondrion | RNA binding | Nucleus |
| GLTP | NP_057517.1 | 51228 | glycolipid transfer protein | 12.9 | 1 | 2 | 2 | 4 | 1.09E+08 | 0.89 | 3 | 3.5 | 209 | 23.8 | 7.4 | Transport | Cytoplasm | Transporter activity | Cytoplasm |
| HNRNPM | NP_005959.2 | 4670 | heterogeneous nuclear ribonucleoprotein M isoform a | 54.0 | 2 | 33 | 33 | 265 | 2.41E+09 | 0.89 | 66 | 19.9 | 730 | 77.5 | 8.7 | Regulation of nucleobase, nucleoside, nucleotide and nucleic acid metabolism | Plasma membrane;Nucleolus;Nucleus;Mitochondrion | Ribonucleoprotein | Nucleolus;Nucleus |
| MRPL45 | NP_001265208.2 | 84311 | 39S ribosomal protein L45, mitochondrial isoform 2 | 9.0 | 2 | 2 | 2 | 2 | 5.51E+07 | 0.89 | 2 | 1.5 | 256 | 29.3 | 8.9 | Protein metabolism | Mitochondrion | Structural constituent of ribosome | Mitochondrion |
| MRPL50 | NP_061924.1 | 54534 | 39S ribosomal protein L50, mitochondrial | 34.2 | 1 | 3 | 3 | 3 | 4.80E+07 | 0.89 | 2 | 5.5 | 158 | 18.3 | 7.9 | Protein metabolism | Mitochondrion | Structural constituent of ribosome | Mitochondrion |
| NUSAP1 | NP_001230073.1 | 51203 | nucleolar and spindle-associated protein 1 isoform 6 | 5.8 | 5 | 1 | 1 | 1 | 4.82E+06 | 0.89 | 1 | - | 378 | 42.3 | 9.9 | Cell cycle | Nucleus;Nucleolus;Cytoplasm;Cytoskeleton | Cytokine activity | Nucleus |
| RBM17 | NP_116294.1 | 84991 | splicing factor 45 | 21.0 | 1 | 5 | 5 | 10 | 1.19E+08 | 0.89 | 5 | 8.7 | 401 | 44.9 | 6.0 | Regulation of nucleobase, nucleoside, nucleotide and nucleic acid metabolism | Nucleus | RNA binding | Nucleus |
| CLPTM1 | NP_001269104.1 | 1209 | cleft lip and palate transmembrane protein 1 isoform 3 | 8.1 | 3 | 4 | 4 | 13 | 9.14E+07 | 0.89 | 2 | 10.8 | 655 | 75.1 | 6.6 | Biological_process unknown | Integral to membrane | Molecular function unknown | Integral to membrane |
| GNAS | NP_536351.1 | 2778 | protein GNAS isoform GNASS | 17.4 | 2 | 1 | 4 | 8 | 2.22E+08 | 0.89 | 1 | - | 380 | 44.2 | 6.3 | Cell communication;Signal transduction | Plasma membrane | GTPase activity | Plasma membrane |
| NFKBIA | NP_065390.1 | 4792 | NF-kappa-B inhibitor alpha | 5.7 | 1 | 1 | 1 | 2 | 2.87E+07 | 0.89 | 1 | - | 317 | 35.6 | 4.7 | Regulation of nucleobase, nucleoside, nucleotide and nucleic acid metabolism | Nucleus;Mitochondrion;Cytoplasm | Transcription regulator activity | Cytoplasm |
| PAICS | NP_006443.1 | 10606 | multifunctional protein ADE2 isoform 2 | 73.9 | 2 | 29 | 29 | 194 | 1.27E+09 | 0.89 | 53 | 20.3 | 425 | 47 | 7.2 | Metabolism;Energy pathways | Nucleus | Ligase activity | Nucleus |
| PLRG1 | NP_002660.1 | 5356 | pleiotropic regulator 1 isoform 1 | 25.7 | 2 | 6 | 6 | 13 | 1.64E+08 | 0.89 | 5 | 13.8 | 514 | 57.2 | 9.2 | Cell communication;Signal transduction | Nucleus;Nucleolus | Transcription regulator activity | Nucleus |
| RIPK1 | NP_003795.2 | 8737 | receptor-interacting serine/threonine-protein kinase 1 | 12.1 | 1 | 5 | 5 | 5 | 3.01E+07 | 0.89 | 1 | - | 671 | 75.9 | 6.3 | Cell communication | Cytoplasm | Protein serine/threonine kinase activity | Cytoplasm |
| WRNIP1 | NP_569079.1 | 56897 | ATPase WRNIP1 isoform 2 | 25.2 | 2 | 9 | 9 | 14 | 5.41E+07 | 0.89 | 7 | 29.6 | 640 | 69.4 | 6.3 | Regulation of nucleobase, nucleoside, nucleotide and nucleic acid metabolism | Nucleus;Cytoplasm | DNA repair protein | Nucleus;Cytoplasm |
| GIT2 | NP_055591.2 | 9815 | ARF GTPase-activating protein GIT2 isoform 3 | 18.3 | 6 | 5 | 5 | 7 | 1.04E+08 | 0.89 | 2 | 20.3 | 679 | 75.7 | 7.2 | Cell communication;Signal transduction | Nucleus;Golgi apparatus;Endoplasmic reticulum;Focal adhesion | GTPase activator activity | Nucleus |
| MPDZ | NP_001248336.1 | 8777 | multiple PDZ domain protein isoform 3 | 1.8 | 3 | 2 | 2 | 2 | 1.35E+08 | 0.89 | 1 | - | 2008 | 214.7 | 5.0 | Cell growth and/or maintenance | Cytoplasm;Nucleus;Plasma membrane | Cell adhesion molecule activity | Plasma membrane |
| MYH9 | NP_002464.1 | 4627 | myosin-9 | 68.2 | 8 | 160 | 184 | 1675 | 6.10E+09 | 0.89 | 373 | 12.8 | 1960 | 226.4 | 5.6 | Cell growth and/or maintenance | Cytoplasm;Cytosol;Nucleus;Mitochondrion | Structural molecule activity | Cytoplasm |
| RNF113A | NP_008909.1 | 7737 | RING finger protein 113A | 12.5 | 1 | 2 | 2 | 6 | 8.21E+07 | 0.89 | 3 | 21.2 | 343 | 38.8 | 5.7 | Regulation of nucleobase, nucleoside, nucleotide and nucleic acid metabolism | Nucleus | DNA binding | Nucleus |
| SKIV2L | NP_008860.4 | 6499 | helicase SKI2W | 7.7 | 1 | 5 | 5 | 9 | 4.38E+07 | 0.89 | 4 | 21.1 | 1246 | 137.7 | 6.1 | Regulation of nucleobase, nucleoside, nucleotide and nucleic acid metabolism | Nucleolus;Cytoplasm;Ribosome;Nucleus | RNA binding | Nucleolus |
| AFF4 | NP_055238.1 | 27125 | AF4/FMR2 family member 4 | 2.7 | 1 | 2 | 2 | 3 | 1.49E+07 | 0.89 | 1 | - | 1163 | 127.4 | 9.3 | Regulation of nucleobase, nucleoside, nucleotide and nucleic acid metabolism | Nucleus;Cytoplasm | Transcription factor activity | Nucleus |
| COPA | NP_004362.2 | 1314 | coatomer subunit alpha isoform 2 | 44.2 | 2 | 39 | 39 | 119 | 1.07E+09 | 0.89 | 39 | 21.9 | 1224 | 138.3 | 7.7 | Transport | Cytoplasm;Golgi apparatus;Endoplasmic reticulum;Mitochondrion | Transporter activity | Cytoplasm |
| LANCL2 | NP_061167.1 | 55915 | lanC-like protein 2 | 31.1 | 1 | 7 | 7 | 12 | 1.23E+08 | 0.89 | 5 | 6.6 | 450 | 50.8 | 7.4 | Biological_process unknown | Plasma membrane;Nucleus;Cytoplasm | Molecular function unknown | Plasma membrane |
| NDUFS7 | NP_077718.3 | 374291 | NADH dehydrogenase [ubiquinone] iron-sulfur protein 7, mitochondrial | 22.1 | 1 | 3 | 3 | 5 | 1.92E+08 | 0.89 | 1 | - | 213 | 23.5 | 10.0 | Metabolism;Energy pathways | Mitochondrion;Nucleus | Oxidoreductase activity | Mitochondrion |
| SLC25A13 | NP_055066.1 | 10165 | calcium-binding mitochondrial carrier protein Aralar2 isoform 2 | 26.8 | 2 | 10 | 10 | 24 | 3.57E+08 | 0.89 | 9 | 15 | 675 | 74.1 | 8.6 | Transport | Mitochondrion | Transporter activity;Carrier activity | Mitochondrion |
| TXNL1 | NP_004777.1 | 9352 | thioredoxin-like protein 1 | 30.1 | 1 | 5 | 5 | 9 | 1.08E+08 | 0.89 | 3 | 33.8 | 289 | 32.2 | 5.0 | Metabolism;Energy pathways | Cytoplasm | Oxidoreductase activity | Cytoplasm |
| AK4 | NP_037542.1 | 205 | adenylate kinase 4, mitochondrial | 30.5 | 1 | 4 | 4 | 6 | 1.56E+08 | 0.88 | 4 | 29.7 | 223 | 25.3 | 8.4 | Metabolism;Energy pathways | Mitochondrion | Catalytic activity | Mitochondrion |
| RBM28 | NP_001159607.1 | 55131 | RNA-binding protein 28 isoform 2 | 13.1 | 2 | 6 | 6 | 7 | 4.68E+07 | 0.88 | 5 | 3.4 | 618 | 69.9 | 8.5 | Regulation of nucleobase, nucleoside, nucleotide and nucleic acid metabolism | Nucleolus;Nucleus | RNA binding | Nucleolus |
| RPL15 | NP_001240309.1 | 6138 | 60S ribosomal protein L15 isoform 1 | 35.3 | 2 | 8 | 8 | 50 | 6.77E+08 | 0.88 | 13 | 18.8 | 204 | 24.1 | 11.6 | Protein metabolism | Ribosome;Nucleolus | Structural constituent of ribosome | Ribosome |
| SLC30A6 | NP_001180444.1 | 55676 | zinc transporter 6 isoform 4 | 3.7 | 4 | 1 | 1 | 1 | 2.93E+06 | 0.88 | 1 | - | 432 | 47.6 | 8.3 | Transport | Golgi apparatus;Cytoplasmic vesicle;Endosome;Nucleus | Transporter activity | Golgi apparatus |
| FAM32A | NP_054796.1 | 26017 | protein FAM32A | 11.6 | 1 | 1 | 1 | 1 | 1.84E+07 | 0.88 | 1 | - | 112 | 13.2 | 10.0 | Biological_process unknown | - | Molecular function unknown | - |
| FAM98B | NP_775882.2 | 283742 | protein FAM98B | 31.0 | 1 | 5 | 6 | 12 | 5.50E+07 | 0.88 | 5 | 18.7 | 433 | 45.5 | 8.7 | Biological_process unknown | - | Molecular function unknown | - |
| ITSN2 | NP_671494.2 | 50618 | intersectin-2 isoform 2 | 1.8 | 3 | 1 | 1 | 1 | 9.64E+06 | 0.88 | 1 | - | 1249 | 141.7 | 7.8 | Protein transport | Endosome;Cytoplasm | Protein domain specific binding | Endosome;Cytoplasm |
| MANF | NP_006001.4 | 7873 | mesencephalic astrocyte-derived neurotrophic factor precursor | 34.6 | 1 | 5 | 5 | 21 | 5.65E+08 | 0.88 | 6 | 11.7 | 185 | 21.1 | 8.9 | Biological_process unknown | Cytoplasm;Nucleus;Nucleolus | Molecular function unknown | Cytoplasm |
| MRPL32 | NP_114109.1 | 64983 | 39S ribosomal protein L32, mitochondrial precursor | 5.3 | 1 | 1 | 1 | 3 | 6.57E+06 | 0.88 | 1 | - | 188 | 21.4 | 9.7 | Protein metabolism | Mitochondrion | Structural constituent of ribosome | Mitochondrion |
| MRPL38 | NP_115867.2 | 64978 | 39S ribosomal protein L38, mitochondrial | 17.4 | 1 | 4 | 4 | 6 | 1.77E+08 | 0.88 | 5 | 42.6 | 380 | 44.6 | 7.5 | Protein metabolism | Mitochondrion | Structural constituent of ribosome | Mitochondrion |

| Gene Symbol | RefSeq_Accession | Entrez Gene ID | Description | Coverage (%) | Proteins | Unique Peptides | # Peptides | PSM | Area | SCC-R /SCC-S | SCC-R /SCC-S (Count) | SCC-R /SCC-S (Variability [%]) | # AAs | MW [kDa] | calc. pI | Biological Process | Localization | Molecular Function | Primary Localization |
|---|---|---|---|---|---|---|---|---|---|---|---|---|---|---|---|---|---|---|---|
| **DHFR** | NP_000782.1 | 1719 | **dihydrofolate reductase isoform 1** | 54.6 | 4 | 5 | 6 | 8 | 2.45E+08 | **0.88** | 4 | 48 | 187 | 21.4 | 7.4 | Regulation of nucleobase, nucleoside, nucleotide and nucleic acid metabolism | Cytoplasm;Nucleus | Oxidoreductase activity | Cytoplasm |
| **ERAP2** | NP_001123612.1 | 64167 | **endoplasmic reticulum aminopeptidase 2** | 7.4 | 1 | 6 | 6 | 11 | 1.59E+08 | **0.88** | 4 | 12.3 | 960 | 110.4 | 6.7 | Protein metabolism | Endoplasmic reticulum | Aminopeptidase activity | Endoplasmic reticulum |
| **MARCKSL1** | NP_075385.1 | 65108 | **MARCKS-related protein** | 68.7 | 1 | 5 | 5 | 9 | 6.19E+07 | **0.88** | 2 | 8.2 | 195 | 19.5 | 4.7 | Cell communication;Signal transduction | - | Receptor signaling complex scaffold activity | - |
| **TIMM8A** | NP_004076.1 | 1678 | **mitochondrial import inner membrane translocase subunit Tim8 A isoform 1** | 49.5 | 2 | 3 | 3 | 13 | 3.64E+08 | **0.88** | 2 | 3.3 | 97 | 11 | 5.2 | Metabolism;Energy pathways | Mitochondrion | Catalytic activity | Mitochondrion |
| **UBR5** | NP_001269802.1 | 51366 | **E3 ubiquitin-protein ligase UBR5 isoform 2** | 4.9 | 2 | 6 | 6 | 10 | 7.77E+07 | **0.88** | 5 | 4.9 | 2798 | 309 | 5.9 | Protein metabolism | Nucleus | Ubiquitin-specific protease activity | Nucleus |
| **FLAD1** | NP_001171820.1 | 80308 | **FAD synthase isoform 3** | 13.9 | 4 | 3 | 3 | 5 | 3.56E+07 | **0.88** | 3 | 10.5 | 446 | 49.2 | 6.0 | Biological_process unknown | - | Molecular function unknown | - |
| **LRCH1** | NP_001157685.1 | 23143 | **leucine-rich repeat and calponin homology domain-containing protein 1 isoform 3** | 5.9 | 3 | 2 | 2 | 3 | 6.12E+07 | **0.88** | 1 | - | 696 | 77.2 | 5.9 | Biological_process unknown | - | Molecular function unknown | - |
| **NUP160** | NP_056046.1 | 23279 | **nuclear pore complex protein Nup160** | 12.4 | 1 | 11 | 11 | 28 | 3.90E+08 | **0.88** | 16 | 23.6 | 1436 | 162 | 5.5 | Transport | Nucleus;Nuclear membrane;Microtubule | Transporter activity | Nucleus |
| **SMC3** | NP_005436.1 | 9126 | **structural maintenance of chromosomes protein 3** | 32.6 | 1 | 30 | 30 | 95 | 3.30E+08 | **0.88** | 39 | 17 | 1217 | 141.5 | 7.2 | Biological_process unknown | Nucleus;Nucleolus;Chromosome;Mitochondrion;Kinetochore | Molecular function unknown | Nucleus |
| **SRP68** | NP_055045.2 | 6730 | **signal recognition particle subunit SRP68 isoform 1** | 36.0 | 3 | 16 | 16 | 46 | 5.36E+08 | **0.88** | 22 | 46.4 | 627 | 70.7 | 8.6 | Protein metabolism | Nucleolus;Cytoplasm;Endoplasmic reticulum;Cytosol | RNA binding | Nucleolus |
| **SRP72** | NP_008878.3 | 6731 | **signal recognition particle subunit SRP72 isoform 1** | 48.9 | 2 | 25 | 25 | 79 | 3.72E+08 | **0.88** | 24 | 17.2 | 671 | 74.6 | 9.3 | Protein metabolism | Nucleolus;Cytoplasm;Cytosol | RNA binding | Nucleolus |
| **DHX16** | NP_003578.2 | 8449 | **putative pre-mRNA-splicing factor ATP-dependent RNA helicase DHX16 isoform 1** | 9.5 | 2 | 7 | 7 | 15 | 1.33E+08 | **0.88** | 5 | 5.5 | 1041 | 119.2 | 6.8 | Regulation of nucleobase, nucleoside, nucleotide and nucleic acid metabolism | Nucleus | ATPase activity | Nucleus |
| **EHMT1** | NP_079033.4 | 79813 | **histone-lysine N-methyltransferase EHMT1 isoform 1** | 4.2 | 2 | 3 | 3 | 3 | 4.55E+07 | **0.88** | 1 | - | 1298 | 141.4 | 5.8 | Regulation of nucleobase, nucleoside, nucleotide and nucleic acid metabolism | Nucleus | Methyltransferase activity | Nucleus |
| **NLE1** | NP_060566.2 | 54475 | **notchless protein homolog 1 isoform a** | 13.2 | 2 | 4 | 4 | 8 | 8.24E+07 | **0.88** | 3 | 4.3 | 485 | 53.3 | 7.3 | Biological_process unknown | Nucleolus | Molecular function unknown | Nucleolus |
| **SARNP** | NP_149073.1 | 84324 | **SAP domain-containing ribonucleoprotein** | 61.4 | 1 | 8 | 8 | 26 | 3.50E+08 | **0.88** | 5 | 11.9 | 210 | 23.7 | 6.4 | Biological_process unknown | Nucleus;Cytoplasm;Nucleolus | Molecular function unknown | Nucleus |
| **TARS** | NP_689508.3 | 6897 | **threonine--tRNA ligase, cytoplasmic isoform 1** | 51.9 | 2 | 33 | 35 | 143 | 9.33E+08 | **0.88** | 49 | 15.8 | 723 | 83.4 | 6.7 | Metabolism;Energy pathways | Cytoplasm;Mitochondrion;Ribosome | Ligase activity | Cytoplasm |
| **FKBP15** | NP_056073.1 | 23307 | **FK506-binding protein 15** | 18.2 | 1 | 15 | 15 | 33 | 1.92E+08 | **0.88** | 9 | 14.3 | 1219 | 133.5 | 5.2 | Cell growth and/or maintenance | Axon;Actin cytoskeleton | Isomerase activity | Axon |
| **GMPPB** | NP_068806.1 | 29925 | **mannose-1-phosphate guanyltransferase beta isoform 2** | 25.6 | 2 | 6 | 6 | 12 | 1.82E+08 | **0.88** | 4 | 48 | 360 | 39.8 | 6.8 | Metabolism | - | Phosphorylase activity | - |
| **LARP4B** | NP_055970.1 | 23185 | **la-related protein 4B** | 7.6 | 1 | 2 | 2 | 6 | 7.11E+07 | **0.88** | 3 | 36.9 | 738 | 80.5 | 6.9 | Regulation of nucleobase, nucleoside, nucleotide and nucleic acid metabolism | Nucleus;Cytoplasm | RNA binding | Nucleus;Cytoplasm |
| **MYL12A** | NP_006462.1 | 10627 | **myosin regulatory light chain 12A** | 62.6 | 5 | 4 | 10 | 252 | 1.53E+09 | **0.88** | 11 | 15 | 171 | 19.8 | 4.8 | Cell communication;Signal transduction | Cytoskeleton | Calcium ion binding | Cytoskeleton |
| **C5orf22** | NP_060826.2 | 55322 | **UPF0489 protein C5orf22** | 17.2 | 1 | 4 | 4 | 10 | 7.51E+07 | **0.88** | 4 | 5.8 | 442 | 49.9 | 4.8 | Biological_process unknown | - | Molecular function unknown | - |
| **RPL34** | NP_000986.2 | 6164 | **60S ribosomal protein L34** | 20.5 | 1 | 3 | 3 | 7 | 1.58E+08 | **0.88** | 3 | 22.2 | 117 | 13.3 | 11.5 | Protein metabolism | Ribosome;Nucleolus;Mitochondrion | Structural constituent of ribosome | Ribosome |
| **PYCARD** | NP_660183.1 | 29108 | **apoptosis-associated speck-like protein containing a CARD isoform b** | 36.4 | 2 | 6 | 6 | 32 | 2.92E+08 | **0.88** | 12 | 38 | 176 | 20 | 6.0 | Apoptosis | Cytoplasm;Perinuclear region | Caspase activator activity | Cytoplasm |
| **RBMX** | NP_002130.2 | 27316 | **RNA-binding motif protein, X chromosome isoform 1** | 35.8 | 4 | 6 | 17 | 92 | 1.28E+09 | **0.88** | 16 | 9.5 | 391 | 42.3 | 10.1 | Regulation of nucleobase, nucleoside, nucleotide and nucleic acid metabolism | Nucleus;Nucleolus | RNA binding | Nucleus |
| **TMEM38B** | NP_060582.1 | 55151 | **trimeric intracellular cation channel type B** | 7.6 | 1 | 1 | 1 | 1 | 1.22E+08 | **0.88** | 1 | - | 291 | 32.5 | 9.1 | Biological_process unknown | - | Molecular function unknown | - |
| **DMAP1** | NP_001029196.1 | 55929 | **DNA methyltransferase 1-associated protein 1** | 5.8 | 1 | 2 | 2 | 2 | 2.96E+07 | **0.88** | 1 | - | 467 | 53 | 9.5 | Regulation of nucleobase, nucleoside, nucleotide and nucleic acid metabolism | Nucleus | Transcription regulator activity | Nucleus |
| **MAGT1** | NP_115497.4 | 84061 | **magnesium transporter protein 1** | 2.7 | 1 | 1 | 1 | 1 | 6.05E+07 | **0.88** | 1 | - | 367 | 41.5 | 9.9 | Biological_process unknown | - | Molecular function unknown | - |
| **NIFK** | NP_115766.3 | 84365 | **MKI67 FHA domain-interacting nucleolar phosphoprotein** | 39.6 | 1 | 9 | 9 | 14 | 3.77E+08 | **0.88** | 7 | 27.6 | 293 | 34.2 | 9.9 | Regulation of nucleobase, nucleoside, nucleotide and nucleic acid metabolism | Nucleolus;Cytoplasm;Nucleus | RNA binding | Nucleolus |
| **NRD1** | NP_001095132.1 | 4898 | **nardilysin isoform b precursor** | 12.7 | 3 | 9 | 9 | 18 | 1.23E+08 | **0.88** | 11 | 14 | 1151 | 131.6 | 5.0 | Protein metabolism | Plasma membrane | Metallopeptidase activity | Plasma membrane |
| **RNF2** | NP_009143.1 | 6045 | **E3 ubiquitin-protein ligase RING2** | 8.9 | 2 | 2 | 2 | 2 | 1.86E+07 | **0.88** | 2 | 44.4 | 336 | 37.6 | 6.8 | Protein metabolism | Cytoplasm;Nucleoplasm | Ubiquitin-specific protease activity | Cytoplasm |
| **CAV1** | NP_001744.2 | 857 | **caveolin-1 isoform alpha** | 37.6 | 1 | 4 | 7 | 30 | 6.05E+08 | **0.88** | 9 | 21.4 | 178 | 20.5 | 6.0 | Cell growth and/or maintenance | Plasma membrane;Integral to membrane | Structural molecule activity | Plasma membrane |
| **DIS3L2** | NP_689596.4 | 129563 | **DIS3-like exonuclease 2 isoform 1** | 6.1 | 2 | 3 | 3 | 3 | 2.27E+07 | **0.88** | 1 | - | 885 | 99.2 | 6.1 | Biological_process unknown | - | Molecular function unknown | - |
| **EIF1AY** | NP_001265541.1 | 9086 | **eukaryotic translation initiation factor 1A, Y-chromosomal isoform 2** | 12.6 | 3 | 1 | 1 | 14 | 5.81E+08 | **0.88** | 2 | 27.6 | 127 | 14.4 | 5.3 | Protein metabolism | - | Translation regulator activity | - |
| **ITGA9** | NP_002198.2 | 3680 | **integrin alpha-9 precursor** | 0.6 | 1 | 1 | 1 | 2 | 1.79E+08 | **0.88** | 2 | 20.9 | 1035 | 114.4 | 6.1 | Cell growth and/or maintenance | Plasma membrane | Cell adhesion molecule activity | Plasma membrane |
| **MAN1A2** | NP_006690.1 | 10905 | **mannosyl-oligosaccharide 1,2-alpha-mannosidase IB** | 3.0 | 1 | 1 | 1 | 2 | 8.15E+06 | **0.88** | 2 | 19.5 | 641 | 73 | 7.6 | Metabolism;Energy pathways | Golgi apparatus | Hydrolase activity | Golgi apparatus |
| **MDH1** | NP_005908.1 | 4190 | **malate dehydrogenase, cytoplasmic isoform 2** | 50.6 | 3 | 13 | 13 | 130 | 4.07E+09 | **0.88** | 29 | 10.6 | 334 | 36.4 | 7.4 | Metabolism;Energy pathways | Cytoplasm;Cytosol;Mitochondrion | Catalytic activity | Cytoplasm |
| **RRP1B** | NP_055871.1 | 23076 | **ribosomal RNA processing protein 1 homolog B** | 29.2 | 1 | 12 | 12 | 25 | 1.13E+08 | **0.88** | 9 | 11.1 | 758 | 84.4 | 9.8 | Biological_process unknown | Nucleolus;Nucleus | Molecular function unknown | Nucleolus |

| Gene Symbol | RefSeq_Accession | Entrez Gene ID | Description | Coverage (%) | Proteins | Unique Peptides | # Peptides | PSM | Area | SCC-R /SCC-S | SCC-R /SCC-S (Count) | SCC-R /SCC-S (Variability [%]) | # AAs | MW [kDa] | calc. pI | Biological Process | Localization | Molecular Function | Primary Localization |
|---|---|---|---|---|---|---|---|---|---|---|---|---|---|---|---|---|---|---|---|
| **UBE2R2** | NP_060281.2 | 54926 | **ubiquitin-conjugating enzyme E2 R2** | 4.6 | 1 | 1 | 1 | 1 | 6.93E+07 | **0.88** | 1 | - | 238 | 27.1 | 4.4 | Protein metabolism | - | Ubiquitin-specific protease activity | - |
| **BLOC1S1** | NP_001478.2 | 2647 | **biogenesis of lysosome-related organelles complex 1 subunit 1** | 25.5 | 1 | 2 | 2 | 5 | 4.21E+07 | **0.87** | 2 | 0.4 | 153 | 17.3 | 9.3 | Regulation of nucleobase, nucleoside, nucleotide and nucleic acid metabolism | - | Acyltransferase activity | - |
| **BOLA2** | NP_001026997.1 | 552900 | **BolA-like protein 2** | 34.9 | 1 | 5 | 5 | 24 | 2.13E+09 | **0.87** | 12 | 5.5 | 152 | 16.9 | 8.2 | Biological_process unknown | - | Molecular function unknown | - |
| **BST2** | NP_004326.1 | 684 | **bone marrow stromal antigen 2 precursor** | 5.6 | 1 | 1 | 1 | 2 | 1.58E+08 | **0.87** | 1 | - | 180 | 19.8 | 5.6 | Immune response | Plasma membrane | Molecular function unknown | Plasma membrane |
| **CDK1** | NP_001777.1 | 983 | **cyclin-dependent kinase 1 isoform 1** | 46.1 | 3 | 9 | 11 | 40 | 9.21E+08 | **0.87** | 14 | 22.7 | 297 | 34.1 | 8.4 | Cell communication;Signal transduction | Cytoplasm;Nucleus;Centrosome;Nucleolus;Microtubule;Mitochondrion | Protein serine/threonine kinase activity | Cytoplasm |
| **IPO7** | NP_006382.1 | 10527 | **importin-7** | 28.5 | 1 | 20 | 21 | 89 | 8.06E+08 | **0.87** | 27 | 22.4 | 1038 | 119.4 | 4.8 | Transport | Nucleus | Transporter activity | Nucleus |
| **PPL** | NP_002696.3 | 5493 | **periplakin** | 16.7 | 1 | 21 | 21 | 42 | 2.23E+08 | **0.87** | 16 | 8.7 | 1756 | 204.6 | 5.6 | Cell growth and/or maintenance | Cell surface;Plasma membrane;Cytoplasm | Structural constituent of cytoskeleton | Plasma membrane |
| **RTFDC1** | NP_057491.2 | 51507 | **protein RTF2 homolog isoform b** | 10.1 | 3 | 2 | 2 | 3 | 5.42E+07 | **0.87** | 2 | 36.9 | 306 | 33.9 | 8.6 | Biological_process unknown | Nucleolus | Molecular function unknown | Nucleolus |
| **SLC39A7** | NP_001275706.1 | 7922 | **zinc transporter SLC39A7 isoform 2** | 4.1 | 2 | 1 | 1 | 2 | 4.72E+07 | **0.87** | 2 | 8.2 | 344 | 36.3 | 7.8 | Transport | Plasma membrane;Endoplasmic reticulum | Auxiliary transport protein activity | Plasma membrane |
| **TNKS1BP1** | NP_203754.2 | 85456 | **182 kDa tankyrase-1-binding protein** | 52.9 | 1 | 56 | 56 | 180 | 7.75E+08 | **0.87** | 56 | 18.8 | 1729 | 181.7 | 4.9 | Regulation of nucleobase, nucleoside, nucleotide and nucleic acid metabolism | Nucleus;Cytoplasm | Molecular function unknown | Nucleus |
| **UQCRQ** | NP_055217.2 | 27089 | **cytochrome b-c1 complex subunit 8** | 15.9 | 1 | 1 | 1 | 5 | 4.83E+08 | **0.87** | 2 | 3.7 | 82 | 9.9 | 10.1 | Biological_process unknown | - | Molecular function unknown | - |
| **WDR89** | NP_001245201.1 | 112840 | **WD repeat-containing protein 89** | 4.7 | 1 | 1 | 1 | 1 | 4.44E+07 | **0.87** | 1 | - | 387 | 43.2 | 6.1 | Biological_process unknown | Nucleolus | Molecular function unknown | Nucleolus |
| **CDK5RAP3** | NP_788276.1 | 80279 | **CDK5 regulatory subunit-associated protein 3 isoform b** | 16.6 | 5 | 7 | 7 | 15 | 1.08E+08 | **0.87** | 6 | 5.8 | 506 | 56.9 | 4.8 | Cell communication;Signal transduction;Regulation of cell cycle | - | Molecular function unknown | - |
| **KLHL36** | NP_079007.2 | 79786 | **kelch-like protein 36** | 1.1 | 1 | 1 | 1 | 1 | 2.52E+07 | **0.87** | 1 | - | 616 | 69.9 | 6.2 | Biological_process unknown | - | Molecular function unknown | - |
| **LARP4** | NP_954660.1 | 113251 | **la-related protein 4 isoform c** | 15.0 | 6 | 6 | 6 | 15 | 5.90E+07 | **0.87** | 7 | 31.7 | 653 | 72.4 | 6.3 | Regulation of nucleobase, nucleoside, nucleotide and nucleic acid metabolism | Nucleus;Cytoplasm | RNA binding | Nucleus;Cytoplasm |
| **NHLRC2** | NP_940916.2 | 374354 | **NHL repeat-containing protein 2** | 11.6 | 1 | 4 | 4 | 10 | 4.79E+07 | **0.87** | 4 | 41.5 | 726 | 79.4 | 5.6 | Biological_process unknown | - | Molecular function unknown | - |
| **ACSF2** | NP_001275899.1 | 80221 | **acyl-CoA synthetase family member 2, mitochondrial isoform 4** | 24.7 | 5 | 7 | 7 | 8 | 1.33E+08 | **0.87** | 3 | 38.6 | 572 | 63.6 | 7.4 | Biological_process unknown | - | Molecular function unknown | - |
| **ANKRD17** | NP_001273700.1 | 26057 | **ankyrin repeat domain-containing protein 17 isoform c** | 6.6 | 3 | 6 | 8 | 11 | 5.10E+07 | **0.87** | 3 | 93.5 | 2490 | 263.4 | 6.5 | Biological_process unknown | Nucleus;Cytoplasm | Molecular function unknown | Nucleus |
| **BICD2** | NP_056065.1 | 23299 | **protein bicaudal D homolog 2 isoform 2** | 7.2 | 2 | 4 | 4 | 6 | 7.76E+07 | **0.87** | 4 | 91.1 | 824 | 93.5 | 5.4 | Vesicle-mediated transport | Cytoplasm;Golgi apparatus;Centrosome;Microtubule;Nucleus | Protein binding | Golgi apparatus;Nucleus;Cytoplasm |
| **DDX6** | NP_001244120.1 | 1656 | **probable ATP-dependent RNA helicase DDX6** | 36.0 | 1 | 10 | 11 | 42 | 6.11E+08 | **0.87** | 11 | 15.9 | 483 | 54.4 | 8.7 | Regulation of nucleobase, nucleoside, nucleotide and nucleic acid metabolism | Cytoplasm;Nucleus | RNA binding | Cytoplasm |
| **HM13** | NP_110416.1 | 81502 | **minor histocompatibility antigen H13 isoform 1** | 14.1 | 4 | 5 | 5 | 26 | 3.63E+08 | **0.87** | 8 | 17.8 | 377 | 41.5 | 6.4 | Immune response | Integral to membrane | Peptidase activity | Integral to membrane |
| **LDHA** | NP_005557.1 | 3939 | **L-lactate dehydrogenase A chain isoform 1** | 92.8 | 8 | 34 | 35 | 766 | 8.74E+09 | **0.87** | 120 | 17.9 | 332 | 36.7 | 8.3 | Metabolism;Energy pathways | Cytoplasm;Nucleolus;Cytosol | Catalytic activity | Cytoplasm |
| **NUMB** | NP_001005745.1 | 8650 | **protein numb homolog isoform 4** | 6.6 | 4 | 2 | 2 | 3 | 4.52E+07 | **0.87** | 1 | - | 592 | 64.5 | 8.4 | Development | Plasma membrane;Endosome;Golgi apparatus;Cytoplasmic vesicle;Cytoplasm;Cytosol;Nucleus | Molecular function unknown | Plasma membrane;Nucleus;Cytoplasm |
| **SAP130** | NP_078821.2 | 79595 | **histone deacetylase complex subunit SAP130 isoform b** | 2.2 | 2 | 2 | 2 | 2 | 1.12E+07 | **0.87** | 1 | - | 1048 | 110.3 | 9.8 | Regulation of nucleobase, nucleoside, nucleotide and nucleic acid metabolism | Nucleus | Transcription regulator activity | Nucleus |
| **CPSF2** | NP_059133.1 | 53981 | **cleavage and polyadenylation specificity factor subunit 2** | 16.6 | 1 | 6 | 6 | 9 | 1.09E+08 | **0.87** | 3 | 26.3 | 782 | 88.4 | 5.1 | Regulation of nucleobase, nucleoside, nucleotide and nucleic acid metabolism | Nucleus;Cytoplasm | RNA binding | Nucleus |
| **MTMR9** | NP_056273.2 | 66036 | **myotubularin-related protein 9** | 4.0 | 1 | 1 | 1 | 2 | 3.49E+07 | **0.87** | 1 | - | 549 | 63.4 | 6.4 | Cell communication;Signal transduction | Cytoplasm | Lipid phosphatase activity | Cytoplasm |
| **DDB1** | NP_001914.3 | 1642 | **DNA damage-binding protein 1** | 27.8 | 1 | 24 | 24 | 83 | 4.91E+08 | **0.87** | 29 | 20.3 | 1140 | 126.9 | 5.3 | Regulation of nucleobase, nucleoside, nucleotide and nucleic acid metabolism | Nucleus;Cytoplasm;Nucleolus;Cytosol | DNA repair protein | Cytoplasm |
| **DDX17** | NP_006377.2 | 10521 | **probable ATP-dependent RNA helicase DDX17 isoform 1** | 50.8 | 2 | 24 | 30 | 167 | 2.54E+09 | **0.87** | 39 | 25.8 | 729 | 80.2 | 8.3 | Regulation of nucleobase, nucleoside, nucleotide and nucleic acid metabolism | Nucleus;Nucleolus | ATPase activity | Nucleus |
| **GPS1** | NP_004118.3 | 2873 | **COP9 signalosome complex subunit 1 isoform 2** | 22.8 | 2 | 7 | 7 | 13 | 4.11E+08 | **0.87** | 6 | 17.2 | 491 | 55.5 | 6.7 | Cell communication;Signal transduction | Nucleus;Cytoplasm;Golgi apparatus | Signal transducer activity | Nucleus |
| **LMNB2** | NP_116126.3 | 84823 | **lamin-B2** | 59.7 | 1 | 33 | 38 | 132 | 7.20E+08 | **0.87** | 45 | 13.3 | 620 | 69.9 | 5.6 | Cell growth and/or maintenance | Nucleus;Cytoplasm | Structural molecule activity | Nucleus |
| **MFHAS1** | NP_004216.2 | 9258 | **malignant fibrous histiocytoma-amplified sequence 1** | 1.0 | 1 | 1 | 1 | 2 | 8.17E+07 | **0.87** | 1 | - | 1052 | 116.9 | 7.9 | Biological_process unknown | - | Molecular function unknown | - |
| **PEX14** | NP_004556.1 | 5195 | **peroxisomal membrane protein PEX14** | 22.6 | 1 | 4 | 4 | 8 | 1.93E+08 | **0.87** | 3 | 14.9 | 377 | 41.2 | 4.9 | Cell communication;Signal transduction | Peroxisome;Nucleus | Protein binding | Peroxisome |
| **USP34** | NP_055524.3 | 9736 | **ubiquitin carboxyl-terminal hydrolase 34** | 0.5 | 1 | 1 | 1 | 1 | 1.46E+07 | **0.87** | 1 | - | 3546 | 404 | 5.8 | Protein metabolism | - | Ubiquitin-specific protease activity | - |
| **YES1** | NP_005424.1 | 7525 | **tyrosine-protein kinase Yes** | 16.4 | 44 | 3 | 6 | 8 | 2.91E+08 | **0.87** | 3 | 1.8 | 543 | 60.8 | 6.7 | Cell communication;Signal transduction | Cytoplasm;Plasma membrane | Protein-tyrosine kinase activity | Cytoplasm |
| **AQR** | NP_055506.1 | 9716 | **intron-binding protein aquarius** | 8.7 | 3 | 8 | 8 | 13 | 6.92E+07 | **0.87** | 9 | 29.9 | 1485 | 171.2 | 6.4 | Biological_process unknown;RNA metabolism | Nucleus | Molecular function unknown | Nucleus |
| **ATP6AP1** | NP_001174.2 | 537 | **V-type proton ATPase subunit S1 precursor** | 8.9 | 1 | 3 | 3 | 3 | 3.56E+07 | **0.87** | 2 | 102.9 | 470 | 52 | 6.1 | Transport | - | Transporter activity | - |
| **SEC61G** | NP_001012474.1 | 23480 | **protein transport protein Sec61 subunit gamma** | 17.7 | 1 | 1 | 1 | 4 | 6.93E+08 | **0.87** | 3 | 15.5 | 68 | 7.7 | 10.0 | Transport | Endoplasmic reticulum;Golgi apparatus;Perinuclear region;Microsome | Transporter activity | Endoplasmic reticulum |

| Gene Symbol | RefSeq_Accession | Entrez Gene ID | Description | Coverage (%) | Proteins | Unique Peptides | # Peptides | PSM | Area | SCC-R /SCC-S | SCC-R /SCC-S (Count) | SCC-R /SCC-S (Variability [%]) | # AAs | MW [kDa] | calc. pI | Biological Process | Localization | Molecular Function | Primary Localization |
|---|---|---|---|---|---|---|---|---|---|---|---|---|---|---|---|---|---|---|---|
| **AKR1A1** | NP_001189343.1 | 10327 | **alcohol dehydrogenase [NADP(+)]** | 60.6 | 1 | 13 | 14 | 86 | 6.68E+08 | **0.87** | 16 | 24.9 | 325 | 36.5 | 6.8 | Metabolism | Cytoplasm;Plasma membrane;Mitochondrion | Oxidoreductase activity | Cytoplasm |
| **ATP5I** | NP_009031.1 | 521 | **ATP synthase subunit e, mitochondrial** | 30.4 | 1 | 2 | 2 | 6 | 1.44E+08 | **0.87** | 3 | 6.2 | 69 | 7.9 | 9.4 | Metabolism;Energy pathways | Mitochondrion;Extracellular | Transporter activity | Mitochondrion |
| **DEK** | NP_003463.1 | 7913 | **protein DEK isoform 1** | 20.8 | 2 | 8 | 8 | 27 | 6.04E+08 | **0.87** | 12 | 9.4 | 375 | 42.6 | 8.6 | Regulation of nucleobase, nucleoside, nucleotide and nucleic acid metabolism | Nucleus;Cytoplasm | DNA binding | Nucleus |
| **ANXA11** | NP_001265338.1 | 311 | **annexin A11 isoform 2** | 29.9 | 4 | 14 | 14 | 53 | 5.17E+08 | **0.87** | 19 | 21 | 472 | 51.2 | 7.7 | Signal transduction;Cell communication | Cytoplasm;Nucleus | Calcium ion binding | Cytoplasm |
| **DDX19A** | NP_060802.1 | 55308 | **ATP-dependent RNA helicase DDX19A** | 45.0 | 2 | 4 | 13 | 50 | 2.76E+08 | **0.87** | 6 | 15 | 478 | 53.9 | 6.6 | Regulation of nucleobase, nucleoside, nucleotide and nucleic acid metabolism | - | Molecular function unknown | - |
| **GMEB1** | NP_077808.1 | 10691 | **glucocorticoid modulatory element-binding protein 1 isoform 2** | 5.2 | 2 | 2 | 2 | 2 | 3.73E+07 | **0.87** | 1 | - | 563 | 61.3 | 4.8 | Regulation of nucleobase, nucleoside, nucleotide and nucleic acid metabolism | Nucleus | Transcription regulator activity | Nucleus |
| **ILF2** | NP_001254738.1 | 3608 | **interleukin enhancer-binding factor 2 isoform 2** | 84.7 | 2 | 24 | 24 | 286 | 1.69E+09 | **0.87** | 42 | 13.2 | 352 | 38.9 | 4.9 | Regulation of nucleobase, nucleoside, nucleotide and nucleic acid metabolism | Nucleus;Cytoplasm;Nucleolus | Transcription factor activity | Nucleus |
| **RALA** | NP_005393.2 | 5898 | **ras-related protein Ral-A precursor** | 31.1 | 1 | 2 | 5 | 14 | 2.83E+08 | **0.87** | 3 | 19.9 | 206 | 23.6 | 7.1 | Cell communication;Signal transduction | Plasma membrane;Membrane fraction;Mitochondrion | GTPase activity | Plasma membrane |
| **AP3M1** | NP_036227.1 | 26985 | **AP-3 complex subunit mu-1** | 23.0 | 2 | 6 | 6 | 13 | 1.96E+08 | **0.87** | 6 | 10 | 418 | 46.9 | 6.9 | Protein targeting | Golgi apparatus;Cytoplasm;Lysosome | Protein transporter activity | Golgi apparatus |
| **DFFA** | NP_998731.1 | 1676 | **DNA fragmentation factor subunit alpha isoform 2** | 25.4 | 2 | 4 | 4 | 7 | 1.11E+08 | **0.87** | 3 | 2.8 | 268 | 29.4 | 4.6 | Protein metabolism | Cytoplasm;Nucleus | Chaperone activity | Cytoplasm |
| **HNRNPH2** | NP_062543.1 | 3188 | **heterogeneous nuclear ribonucleoprotein H2** | 53.2 | 2 | 9 | 17 | 96 | 2.28E+09 | **0.86** | 10 | 23.8 | 449 | 49.2 | 6.3 | Regulation of nucleobase, nucleoside, nucleotide and nucleic acid metabolism | Nucleus;Cytoplasm | RNA binding | Nucleus |
| **NADSYN1** | NP_060631.2 | 55191 | **glutamine-dependent NAD(+) synthetase** | 9.5 | 1 | 5 | 5 | 6 | 8.82E+07 | **0.86** | 3 | 0.5 | 706 | 79.2 | 6.4 | Metabolism;Energy pathways | - | Ligase activity | - |
| **PAFAH1B1** | NP_000421.1 | 5048 | **platelet-activating factor acetylhydrolase IB subunit alpha** | 52.4 | 1 | 16 | 16 | 63 | 7.06E+08 | **0.86** | 20 | 10.5 | 410 | 46.6 | 7.4 | Metabolism;Energy pathways | Cytoplasm;Centrosome | Hydrolase activity | Cytoplasm |
| **SCAF11** | NP_004710.2 | 9169 | **protein SCAF11** | 4.9 | 1 | 3 | 3 | 4 | 2.69E+07 | **0.86** | 1 | - | 1463 | 164.6 | 8.4 | Regulation of nucleobase, nucleoside, nucleotide and nucleic acid metabolism | Nucleus;Cytoplasm | RNA binding | Nucleus |
| **TAF4** | NP_003176.2 | 6874 | **transcription initiation factor TFIID subunit 4** | 3.9 | 1 | 2 | 2 | 2 | 5.34E+07 | **0.86** | 2 | 7.7 | 1085 | 110 | 9.9 | Regulation of nucleobase, nucleoside, nucleotide and nucleic acid metabolism | Nucleus | Transcription factor activity | Nucleus |
| **KIAA1033** | NP_056090.1 | 23325 | **WASH complex subunit 7** | 14.8 | 1 | 10 | 10 | 27 | 2.22E+08 | **0.86** | 11 | 40.3 | 1173 | 136.3 | 7.4 | Biological_process unknown | - | Molecular function unknown | - |
| **MPP5** | NP_001243479.1 | 64398 | **MAGUK p55 subfamily member 5 isoform 2** | 7.0 | 2 | 2 | 2 | 4 | 2.20E+07 | **0.86** | 1 | - | 641 | 73.4 | 6.3 | Cell communication;Signal transduction | Plasma membrane | Molecular function unknown | Plasma membrane |
| **RQCD1** | NP_001258564.1 | 9125 | **cell differentiation protein RCD1 homolog isoform 3** | 15.1 | 3 | 2 | 2 | 2 | 5.11E+08 | **0.86** | 2 | 33.7 | 258 | 29.1 | 8.0 | Regulation of nucleobase, nucleoside, nucleotide and nucleic acid metabolism | Nucleus | Transcription regulator activity | Nucleus |
| **SERPINB7** | NP_001248760.1 | 8710 | **serpin B7 isoform 2** | 2.8 | 2 | 1 | 1 | 4 | 2.50E+08 | **0.86** | 2 | 7.9 | 363 | 41.1 | 6.7 | Protein metabolism | Cytoplasm | Protease inhibitor activity | Cytoplasm |
| **WTH3DI** | NP_001071105.1 | 150786 | **RAB6C-like** | 15.8 | 2 | 1 | 3 | 9 | 2.64E+08 | **0.86** | 1 | - | 254 | 28.2 | 6.7 | Biological_process unknown | - | Molecular function unknown | - |
| **ACP1** | NP_009030.1 | 52 | **low molecular weight phosphotyrosine protein phosphatase isoform b** | 58.9 | 1 | 1 | 6 | 11 | 4.16E+08 | **0.86** | 1 | - | 158 | 18 | 6.9 | Metabolism;Energy pathways | Cytoplasm | Acid phosphatase activity | Cytoplasm |
| **CDC27** | NP_001247.3 | 996 | **cell division cycle protein 27 homolog isoform 2** | 6.2 | 2 | 2 | 2 | 4 | 7.33E+07 | **0.86** | 4 | 14.9 | 824 | 91.8 | 7.0 | Cell communication;Signal transduction | Nucleus;Kinetochore;Centrosome | Protein binding | Nucleus |
| **COG6** | NP_001138551.1 | 57511 | **conserved oligomeric Golgi complex subunit 6 isoform 2** | 12.7 | 2 | 4 | 4 | 6 | 6.12E+07 | **0.86** | 2 | 35.4 | 615 | 68.4 | 5.9 | Transport | Golgi apparatus;Cytoplasm | Structural molecule activity | Golgi apparatus |
| **IDH3B** | NP_008830.2 | 3420 | **isocitrate dehydrogenase [NAD] subunit beta, mitochondrial isoform a precursor** | 33.0 | 3 | 8 | 8 | 22 | 3.34E+08 | **0.86** | 7 | 22.1 | 385 | 42.2 | 8.5 | Metabolism;Energy pathways | Mitochondrion | Catalytic activity | Mitochondrion |
| **POLG** | NP_001119603.1 | 5428 | **DNA polymerase subunit gamma-1** | 1.2 | 1 | 1 | 1 | 1 | 1.94E+07 | **0.86** | 1 | - | 1239 | 139.5 | 6.9 | Regulation of nucleobase, nucleoside, nucleotide and nucleic acid metabolism | Mitochondrion | DNA-directed DNA polymerase activity | Mitochondrion |
| **PRRC2C** | NP_055987.2 | 23215 | **protein PRRC2C** | 11.2 | 1 | 15 | 16 | 35 | 1.16E+09 | **0.86** | 10 | 15.8 | 2817 | 308.4 | 9.1 | Biological_process unknown | Cytoplasm | Molecular function unknown | Cytoplasm |
| **RDH14** | NP_065956.1 | 57665 | **retinol dehydrogenase 14** | 6.3 | 1 | 2 | 2 | 2 | 3.07E+07 | **0.86** | 1 | - | 336 | 36.8 | 8.8 | Metabolism;Energy pathways | Endoplasmic reticulum | Catalytic activity | Endoplasmic reticulum |
| **VPS13C** | NP_060154.3 | 54832 | **vacuolar protein sorting-associated protein 13C isoform 1A** | 1.6 | 4 | 4 | 4 | 5 | 3.15E+07 | **0.86** | 2 | 30.4 | 3710 | 417 | 6.6 | Biological_process unknown | Nucleus | Molecular function unknown | Nucleus |
| **AP1G1** | NP_001119.3 | 164 | **AP-1 complex subunit gamma-1 isoform b** | 33.2 | 2 | 19 | 19 | 43 | 1.99E+08 | **0.86** | 18 | 32.6 | 822 | 91.3 | 6.8 | Vesicle-mediated transport | Cytosol;Plasma membrane;Golgi apparatus | Transporter activity | Golgi apparatus |
| **PAK1IP1** | NP_060376.2 | 55003 | **p21-activated protein kinase-interacting protein 1** | 14.8 | 1 | 4 | 4 | 5 | 5.21E+07 | **0.86** | 3 | 1 | 392 | 43.9 | 8.9 | Cell communication;Signal transduction | Cytoplasm;Nucleolus | Cytoskeletal protein binding | Cytoplasm |
| **SMC4** | NP_001002800.1 | 10051 | **structural maintenance of chromosomes protein 4 isoform 1** | 22.2 | 2 | 17 | 17 | 45 | 1.96E+08 | **0.86** | 23 | 34.6 | 1288 | 147.1 | 6.8 | Chromosome organization and biogenesis (sensu Eukarya) | Nucleus;Nucleolus;Mitochondrion;Cytoplasm | Structural constituent of chromatin | Nucleus |
| **YTHDF3** | NP_689971.4 | 253943 | **YTH domain-containing family protein 3 isoform a** | 19.3 | 3 | 4 | 8 | 15 | 2.66E+08 | **0.86** | 3 | 35.6 | 585 | 63.8 | 9.0 | Biological_process unknown | - | Molecular function unknown | - |
| **COPS4** | NP_057213.2 | 51138 | **COP9 signalosome complex subunit 4 isoform 1** | 30.1 | 2 | 7 | 7 | 11 | 1.44E+08 | **0.86** | 5 | 65 | 406 | 46.2 | 5.8 | Protein metabolism | - | Molecular function unknown | - |
| **CPSF1** | NP_037423.2 | 29894 | **cleavage and polyadenylation specificity factor subunit 1** | 10.3 | 1 | 10 | 10 | 20 | 1.73E+08 | **0.86** | 9 | 36.3 | 1443 | 160.8 | 6.4 | Regulation of nucleobase, nucleoside, nucleotide and nucleic acid metabolism | Nucleus | RNA binding | Nucleus |
| **EIF4G1** | NP_937887.1 | 1981 | **eukaryotic translation initiation factor 4 gamma 1 isoform 2** | 40.0 | 3 | 2 | 50 | 196 | 7.94E+08 | **0.86** | 2 | 47 | 1512 | 166.5 | 5.2 | Protein metabolism | Nucleus;Cytoplasm | Translation regulator activity | Nucleus;Cytoplasm |
| **PSMA3** | NP_687033.1 | 5684 | **proteasome subunit alpha type-3 isoform 2** | 40.7 | 2 | 10 | 10 | 37 | 6.28E+08 | **0.86** | 11 | 7.7 | 248 | 27.6 | 5.3 | Protein metabolism | Cytoplasm;Nucleus | Ubiquitin-specific protease activity | Cytoplasm |
| **SF3B1** | NP_036565.2 | 23451 | **splicing factor 3B subunit 1 isoform 1** | 44.3 | 2 | 42 | 42 | 146 | 8.59E+08 | **0.86** | 54 | 25.7 | 1304 | 145.7 | 7.1 | Regulation of nucleobase, nucleoside, nucleotide and nucleic acid metabolism | Nucleus | Ribonucleoprotein | Nucleus |

| Gene Symbol | RefSeq_Accession | Entrez Gene ID | Description | Coverage (%) | Proteins | Unique Peptides | # Peptides | PSM | Area | SCC-R /SCC-S | SCC-R /SCC-S (Count) | SCC-R /SCC-S (Variability [%]) | # AAs | MW [kDa] | calc. pI | Biological Process | Localization | Molecular Function | Primary Localization |
|---|---|---|---|---|---|---|---|---|---|---|---|---|---|---|---|---|---|---|---|
| **SMCHD1** | NP_056110.2 | 23347 | **structural maintenance of chromosomes flexible hinge domain-containing protein 1** | 11.6 | 1 | 15 | 15 | 26 | 2.54E+08 | **0.86** | 15 | 25.4 | 2005 | 226.2 | 7.3 | Biological_process unknown | - | Molecular function unknown | - |
| **ATF7** | NP_001123532.1 | 11016 | **cyclic AMP-dependent transcription factor ATF-7 isoform 3** | 4.8 | 2 | 1 | 1 | 1 | 1.08E+08 | **0.86** | 1 | - | 462 | 49.6 | 8.3 | Cell communication;Signal transduction | Nucleus;Cytoplasm | Transcription factor activity | Nucleus;Cytoplasm |
| **EIF5** | NP_892116.2 | 1983 | **eukaryotic translation initiation factor 5** | 45.2 | 1 | 16 | 16 | 53 | 7.82E+08 | **0.86** | 15 | 10.7 | 431 | 49.2 | 5.6 | Protein metabolism | Cytoplasm | Translation regulator activity;GTPase activator activity | Cytoplasm |
| **PHF14** | NP_055475.2 | 9678 | **PHD finger protein 14** | 1.4 | 1 | 1 | 1 | 2 | 1.89E+07 | **0.86** | 1 | - | 888 | 100 | 5.3 | Biological_process unknown | Nucleus;Cytoplasm | Molecular function unknown | Nucleus;Cytoplasm |
| **RNF126** | NP_919442.1 | 55658 | **E3 ubiquitin-protein ligase RNF126** | 10.3 | 1 | 1 | 1 | 2 | 1.33E+08 | **0.86** | 1 | - | 311 | 33.8 | 5.5 | Protein metabolism | - | Ubiquitin-specific protease activity | - |
| **RPL4** | NP_000959.2 | 6124 | **60S ribosomal protein L4** | 51.8 | 1 | 27 | 27 | 300 | 5.14E+09 | **0.86** | 56 | 19.7 | 427 | 47.7 | 11.1 | Protein metabolism | Ribosome;Nucleolus | Structural constituent of ribosome | Ribosome |
| **SCRIB** | NP_056171.3 | 23513 | **protein scribble homolog isoform b** | 21.0 | 2 | 14 | 15 | 27 | 7.95E+07 | **0.86** | 9 | 25.7 | 1630 | 174.8 | 5.1 | Protein metabolism | Plasma membrane;Cytoplasm;Synapse;Basolateral membrane;Cell junction | Ubiquitin-specific protease activity | Cytoplasm |
| **AFG3L2** | NP_006787.2 | 10939 | **AFG3-like protein 2** | 20.1 | 1 | 9 | 9 | 14 | 1.57E+08 | **0.86** | 7 | 38.9 | 797 | 88.5 | 8.7 | Transport | Mitochondrion | ATPase activity | Mitochondrion |
| **ATXN3** | NP_001121169.2 | 4287 | **ataxin-3 isoform e** | 20.0 | 10 | 4 | 4 | 8 | 9.75E+07 | **0.86** | 4 | 3.8 | 310 | 35.1 | 4.6 | Protein metabolism | Cytoplasm;Nucleus | Ubiquitin-specific protease activity | Nucleus |
| **GPHN** | NP_001019389.1 | 10243 | **gephyrin isoform 2** | 3.7 | 2 | 2 | 2 | 3 | 3.96E+07 | **0.86** | 2 | 10.7 | 736 | 79.7 | 5.4 | Cell communication;Signal transduction;Synapse organization and biogenesis | Dendrite;Nucleus;Synapse;Cytosol;Cytoplasm | Molecular function unknown;Receptor binding | Cytosol |
| **MTDH** | NP_848927.2 | 92140 | **protein LYRIC** | 35.6 | 1 | 13 | 13 | 32 | 4.06E+08 | **0.86** | 10 | 14.9 | 582 | 63.8 | 9.3 | Biological_process unknown | Endoplasmic reticulum;Nucleus;Nucleolus;Plasma membrane;Cytoplasm | Molecular function unknown | Endoplasmic reticulum |
| **NUP107** | NP_065134.1 | 57122 | **nuclear pore complex protein Nup107** | 13.7 | 1 | 8 | 8 | 17 | 1.95E+08 | **0.86** | 6 | 10.3 | 925 | 106.3 | 5.4 | Transport | Nucleus;Kinetochore;Microtubule;Nuclear membrane | Transporter activity | Nucleus |
| **SCPEP1** | NP_067639.1 | 59342 | **retinoid-inducible serine carboxypeptidase precursor** | 5.8 | 1 | 2 | 2 | 6 | 6.29E+07 | **0.86** | 3 | 50.3 | 452 | 50.8 | 5.8 | Protein metabolism | Cytoplasm;Extracellular | Carboxypeptidase activity | Cytoplasm |
| **STRAP** | NP_009109.3 | 11171 | **serine-threonine kinase receptor-associated protein** | 62.3 | 1 | 17 | 17 | 94 | 1.49E+09 | **0.86** | 25 | 23.7 | 350 | 38.4 | 5.1 | Cell communication;Signal transduction | Cytoplasm;Nucleus | Receptor binding | Cytoplasm |
| **EIF1** | NP_005792.1 | 10209 | **eukaryotic translation initiation factor 1** | 82.3 | 1 | 4 | 9 | 40 | 2.92E+09 | **0.86** | 6 | 9.8 | 113 | 12.7 | 7.4 | Protein metabolism | Cytoplasm | Translation regulator activity | Cytoplasm |
| **POLR1C** | NP_976035.1 | 9533 | **DNA-directed RNA polymerases I and III subunit RPAC1** | 22.3 | 1 | 5 | 5 | 13 | 8.12E+07 | **0.86** | 5 | 40.8 | 346 | 39.2 | 5.5 | Regulation of nucleobase, nucleoside, nucleotide and nucleic acid metabolism | Nucleolus | DNA-directed RNA polymerase activity | Nucleolus |
| **RPS21** | NP_001015.1 | 6227 | **40S ribosomal protein S21** | 71.1 | 1 | 7 | 7 | 137 | 2.66E+09 | **0.86** | 10 | 7.7 | 83 | 9.1 | 8.5 | Protein metabolism | Ribosome | Structural constituent of ribosome | Ribosome |
| **STIM1** | NP_003147.2 | 6786 | **stromal interaction molecule 1 isoform 2 precursor** | 10.1 | 3 | 4 | 4 | 7 | 5.10E+07 | **0.86** | 4 | 30.7 | 685 | 77.4 | 6.7 | Cell communication;Signal transduction | Plasma membrane;Endoplasmic reticulum;Nucleus;Cytoplasm | Cell adhesion molecule activity | Plasma membrane |
| **TACO1** | NP_057444.2 | 51204 | **translational activator of cytochrome c oxidase 1** | 36.4 | 1 | 6 | 6 | 20 | 5.25E+08 | **0.86** | 5 | 23.1 | 297 | 32.5 | 8.1 | Biological_process unknown | - | Molecular function unknown | - |
| **EFR3A** | NP_055952.2 | 23167 | **protein EFR3 homolog A** | 3.1 | 1 | 1 | 1 | 1 | 9.29E+07 | **0.86** | 1 | - | 821 | 92.9 | 6.7 | Biological_process unknown | - | Molecular function unknown | - |
| **INTS5** | NP_085131.1 | 80789 | **integrator complex subunit 5** | 1.9 | 1 | 1 | 1 | 2 | 7.61E+06 | **0.86** | 1 | - | 1019 | 107.9 | 7.1 | RNA metabolism;Transcription | Nucleus;Cytosol | Protein binding | Nucleus |
| **MRPL28** | NP_006419.2 | 10573 | **39S ribosomal protein L28, mitochondrial** | 26.6 | 1 | 5 | 5 | 6 | 5.56E+07 | **0.86** | 4 | 19.2 | 256 | 30.1 | 8.3 | Protein metabolism;RNA metabolism | Mitochondrion | Structural molecule activity;Ribonucleoprotein | Mitochondrion |
| **SURF2** | NP_001265857.1 | 6835 | **surfeit locus protein 2 isoform 2** | 10.6 | 2 | 2 | 2 | 3 | 5.69E+07 | **0.86** | 1 | - | 255 | 29.5 | 9.1 | Biological_process unknown | Cytoplasm | Molecular function unknown | Cytoplasm |
| **PMPCA** | NP_055975.1 | 23203 | **mitochondrial-processing peptidase subunit alpha isoform 1 precursor** | 25.9 | 3 | 8 | 8 | 32 | 4.50E+08 | **0.86** | 13 | 9.8 | 525 | 58.2 | 6.9 | Protein metabolism | Mitochondrion | Peptidase activity | Mitochondrion |
| **SUCLG1** | NP_003840.2 | 8802 | **succinyl-CoA ligase [ADP/GDP-forming] subunit alpha, mitochondrial precursor** | 37.6 | 1 | 10 | 10 | 31 | 5.98E+08 | **0.86** | 14 | 22.8 | 346 | 36.2 | 8.8 | Metabolism;Energy pathways | Mitochondrion | Ligase activity | Mitochondrion |
| **CKS1B** | NP_001817.1 | 1163 | **cyclin-dependent kinases regulatory subunit 1** | 20.3 | 1 | 2 | 2 | 2 | 9.92E+06 | **0.85** | 1 | - | 79 | 9.7 | 8.9 | Cell communication;Signal transduction | Mitochondrion | Protein binding | Mitochondrion |
| **CLASP2** | NP_055912.2 | 23122 | **CLIP-associating protein 2 isoform 1** | 8.3 | 2 | 7 | 8 | 18 | 8.44E+07 | **0.85** | 6 | 4.6 | 1515 | 165.8 | 8.3 | Cell growth and/or maintenance | Cytoplasm;Golgi apparatus;Microtubule | Cytoskeletal protein binding | Cytoplasm |
| **CS** | NP_004068.2 | 1431 | **citrate synthase, mitochondrial precursor** | 51.9 | 1 | 17 | 17 | 99 | 2.03E+09 | **0.85** | 28 | 17.3 | 466 | 51.7 | 8.3 | Metabolism;Energy pathways | Mitochondrial matrix;Mitochondrion;Cytosol | Acyltransferase activity | Mitochondrial matrix |
| **EIF3F** | NP_003745.1 | 8665 | **eukaryotic translation initiation factor 3 subunit F** | 38.9 | 1 | 8 | 8 | 50 | 3.89E+08 | **0.85** | 17 | 11.1 | 357 | 37.5 | 5.5 | Protein metabolism | Cytoplasm;Nucleus | Translation regulator activity | Cytoplasm |
| **ILKAP** | NP_110395.1 | 80895 | **integrin-linked kinase-associated serine/threonine phosphatase 2C** | 12.0 | 1 | 3 | 3 | 4 | 2.99E+07 | **0.85** | 2 | 2.7 | 392 | 42.9 | 7.1 | Cell death | Cytoplasm;Nucleus | Protein serine/threonine phosphatase activity | Cytoplasm |
| **MRPL15** | NP_054894.1 | 29088 | **39S ribosomal protein L15, mitochondrial** | 25.7 | 1 | 5 | 5 | 16 | 7.97E+07 | **0.85** | 4 | 3.7 | 296 | 33.4 | 10.0 | Protein metabolism | Mitochondrion | Structural constituent of ribosome | Mitochondrion |
| **RBM4** | NP_002887.2 | 5936 | **RNA-binding protein 4 isoform 1** | 56.0 | 4 | 6 | 12 | 49 | 4.98E+08 | **0.85** | 9 | 14.5 | 364 | 40.3 | 7.1 | Regulation of nucleobase, nucleoside, nucleotide and nucleic acid metabolism | Nucleus;Nucleolus | RNA binding | Nucleus |
| **C14orf166** | NP_057123.1 | 51637 | **UPF0568 protein C14orf166** | 65.6 | 1 | 14 | 14 | 50 | 2.87E+08 | **0.85** | 21 | 16 | 244 | 28.1 | 6.7 | Regulation of nucleobase, nucleoside, nucleotide and nucleic acid metabolism | Nucleus;Nucleolus;Cytosol | Transcription regulator activity | Nucleus |
| **CAND1** | NP_060918.2 | 55832 | **cullin-associated NEDD8-dissociated protein 1** | 48.3 | 3 | 49 | 49 | 178 | 2.24E+09 | **0.85** | 57 | 14.5 | 1230 | 136.3 | 5.8 | Regulation of nucleobase, nucleoside, nucleotide and nucleic acid metabolism | Nucleus;Mitochondrion | Transcription regulator activity | Nucleus |
| **DNAJC3** | NP_006251.1 | 5611 | **dnaJ homolog subfamily C member 3 precursor** | 9.1 | 1 | 4 | 4 | 7 | 1.72E+08 | **0.85** | 4 | 35.1 | 504 | 57.5 | 6.2 | Protein metabolism | Cytoplasm | Translation regulator activity | Cytoplasm |
| **MTX2** | NP_006545.1 | 10651 | **metaxin-2** | 36.9 | 1 | 5 | 5 | 14 | 7.54E+07 | **0.85** | 3 | 9.9 | 263 | 29.7 | 6.3 | Transport | Mitochondrion | Molecular function unknown | Mitochondrion |
| **NOP58** | NP_057018.1 | 51602 | **nucleolar protein 58** | 32.1 | 1 | 13 | 13 | 55 | 3.70E+08 | **0.85** | 18 | 20.8 | 529 | 59.5 | 8.9 | Regulation of nucleobase, nucleoside, nucleotide and nucleic acid metabolism | Nucleolus;Ribosome | Ribonucleoprotein | Nucleolus |

| Gene Symbol | RefSeq_Accession | Entrez Gene ID | Description | Coverage (%) | Proteins | Unique Peptides | # Peptides | PSM | Area | SCC-R /SCC-S | SCC-R /SCC-S (Count) | SCC-R /SCC-S (Variability [%]) | # AAs | MW [kDa] | calc. pI | Biological Process | Localization | Molecular Function | Primary Localization |
|---|---|---|---|---|---|---|---|---|---|---|---|---|---|---|---|---|---|---|---|
| **RBM25** | NP_067062.1 | 58517 | **RNA-binding protein 25** | 31.0 | 1 | 15 | 15 | 36 | 5.41E+08 | **0.85** | 15 | 8.8 | 843 | 100.1 | 6.3 | Regulation of nucleobase, nucleoside, nucleotide and nucleic acid metabolism | Nucleus | RNA binding | Nucleus |
| **RILP** | NP_113618.2 | 83547 | **rab-interacting lysosomal protein** | 2.5 | 1 | 1 | 1 | 1 | 7.20E+07 | **0.85** | 1 | - | 401 | 44.2 | 5.6 | Transport | Lysosome | Transporter activity | Lysosome |
| **RPS12** | NP_001007.2 | 6206 | **40S ribosomal protein S12** | 81.8 | 1 | 13 | 13 | 318 | 3.32E+09 | **0.85** | 54 | 18.9 | 132 | 14.5 | 7.2 | Protein metabolism | Ribosome;Nucleolus | Structural constituent of ribosome | Ribosome |
| **SNRNP200** | NP_054733.2 | 23020 | **U5 small nuclear ribonucleoprotein 200 kDa helicase** | 40.1 | 1 | 64 | 64 | 177 | 7.87E+08 | **0.85** | 72 | 23.8 | 2136 | 244.4 | 6.1 | Regulation of nucleobase, nucleoside, nucleotide and nucleic acid metabolism | Nucleus;Nucleolus;Mitochondrion | Ribonucleoprotein | Nucleus |
| **TOMM5** | NP_001001790.1 | 401505 | **mitochondrial import receptor subunit TOM5 homolog isoform 1** | 13.7 | 3 | 1 | 1 | 2 | 1.17E+08 | **0.85** | 1 | - | 51 | 6 | 9.7 | Biological_process unknown | - | Molecular function unknown | - |
| **GUF1** | NP_068746.2 | 60558 | **translation factor GUF1, mitochondrial** | 7.9 | 1 | 3 | 3 | 3 | 3.38E+07 | **0.85** | 2 | 88.9 | 669 | 74.3 | 8.6 | Biological_process unknown | - | Molecular function unknown | - |
| **MRPL54** | NP_758455.1 | 116541 | **39S ribosomal protein L54, mitochondrial** | 22.5 | 1 | 1 | 1 | 2 | 1.21E+08 | **0.85** | 1 | - | 138 | 15.8 | 9.6 | Protein metabolism | Mitochondrion | Structural constituent of ribosome | Mitochondrion |
| **OVCA2** | NP_543012.1 | 124641 | **ovarian cancer-associated gene 2 protein** | 30.8 | 1 | 3 | 3 | 4 | 8.53E+06 | **0.85** | 1 | - | 227 | 24.4 | 6.9 | Cell communication;Signal transduction | - | Molecular function unknown | - |
| **PPP1R10** | NP_002705.2 | 5514 | **serine/threonine-protein phosphatase 1 regulatory subunit 10** | 4.4 | 1 | 2 | 2 | 3 | 1.70E+07 | **0.85** | 1 | - | 940 | 99 | 9.2 | Cell communication;Signal transduction | Nucleus | Protein serine/threonine phosphatase activity | Nucleus |
| **SMARCE1** | NP_003070.3 | 6605 | **SWI/SNF-related matrix-associated actin-dependent regulator of chromatin subfamily E member 1** | 36.7 | 1 | 8 | 8 | 19 | 1.57E+08 | **0.85** | 11 | 47.3 | 411 | 46.6 | 4.9 | Regulation of nucleobase, nucleoside, nucleotide and nucleic acid metabolism | Nucleus;Kinetochore | DNA binding | Nucleus |
| **TVP23C** | NP_001128508.1 | 201158 | **Golgi apparatus membrane protein TVP23 homolog C isoform 2** | 5.9 | 2 | 1 | 1 | 1 | 2.58E+06 | **0.85** | 1 | - | 203 | 23.4 | 8.3 | Biological_process unknown | - | Molecular function unknown | - |
| **ASUN** | NP_060634.2 | 55726 | **protein asunder homolog** | 8.6 | 1 | 4 | 4 | 5 | 3.70E+07 | **0.85** | 3 | 21.7 | 706 | 80.2 | 6.7 | Biological_process unknown | - | Molecular function unknown | - |
| **CPT2** | NP_000089.1 | 1376 | **carnitine O-palmitoyltransferase 2, mitochondrial precursor** | 3.8 | 1 | 2 | 2 | 2 | 6.76E+07 | **0.85** | 2 | 10.6 | 658 | 73.7 | 8.2 | Metabolism;Energy pathways | Mitochondrion | Palmitoyltransferase activity | Mitochondrion |
| **CTR9** | NP_055448.1 | 9646 | **RNA polymerase-associated protein CTR9 homolog** | 6.0 | 1 | 4 | 4 | 5 | 4.70E+07 | **0.85** | 3 | 65.7 | 1173 | 133.4 | 6.8 | Regulation of nucleobase, nucleoside, nucleotide and nucleic acid metabolism | Nucleus;Cytoplasm | Transcription regulator activity | Nucleus |
| **NME1-NME2** | NP_001018146.1 | 654364 | **NME1-NME2 protein** | 78.7 | 3 | 8 | 17 | 408 | 5.40E+09 | **0.85** | 29 | 26.6 | 267 | 30.1 | 8.9 | - | - | - | - |
| **UBAP2** | NP_060919.3 | 55833 | **ubiquitin-associated protein 2 isoform 1** | 19.0 | 3 | 9 | 9 | 19 | 1.70E+08 | **0.85** | 8 | 16.6 | 1119 | 117.1 | 7.3 | Biological_process unknown | - | Molecular function unknown | - |
| **ZC3H13** | NP_055885.3 | 23091 | **zinc finger CCCH domain-containing protein 13** | 1.2 | 1 | 1 | 1 | 1 | 2.89E+07 | **0.85** | 1 | - | 1564 | 184.8 | 9.5 | Regulation of nucleobase, nucleoside, nucleotide and nucleic acid metabolism | - | Transcription regulator activity | - |
| **KDM2A** | NP_036440.1 | 22992 | **lysine-specific demethylase 2A isoform a** | 5.8 | 2 | 4 | 4 | 5 | 3.92E+07 | **0.85** | 3 | 10.1 | 1162 | 132.7 | 7.6 | Protein metabolism;Regulation of gene expression, epigenetic | Nucleus;Nucleolus | Ubiquitin-specific protease activity | Nucleus |
| **RPP40** | NP_001273062.1 | 10799 | **ribonuclease P protein subunit p40 isoform c** | 21.5 | 3 | 5 | 5 | 9 | 6.14E+07 | **0.85** | 3 | 1.1 | 321 | 36.8 | 6.4 | Regulation of nucleobase, nucleoside, nucleotide and nucleic acid metabolism | Nucleus;Nucleolus | Ribonuclease activity | Nucleus |
| **RPS28** | NP_001022.1 | 6234 | **40S ribosomal protein S28** | 60.9 | 1 | 8 | 8 | 91 | 1.90E+09 | **0.85** | 16 | 11.5 | 69 | 7.8 | 10.7 | Protein metabolism | Ribosome | Structural constituent of ribosome | Ribosome |
| **RPS8** | NP_001003.1 | 6202 | **40S ribosomal protein S8** | 61.5 | 3 | 19 | 19 | 140 | 3.66E+09 | **0.85** | 38 | 14.5 | 208 | 24.2 | 10.3 | Protein metabolism | Cytoplasm;Nucleolus | Structural constituent of ribosome | Cytoplasm |
| **TRA2A** | NP_001269688.1 | 29896 | **transformer-2 protein homolog alpha isoform 3** | 41.1 | 3 | 5 | 6 | 7 | 1.44E+08 | **0.85** | 5 | 37.6 | 180 | 20.6 | 10.2 | Regulation of nucleobase, nucleoside, nucleotide and nucleic acid metabolism | Nucleus;Mitochondrion;Cytoplasm | RNA binding | Nucleus |
| **EIF4B** | NP_001408.2 | 1975 | **eukaryotic translation initiation factor 4B** | 37.8 | 1 | 17 | 17 | 52 | 6.70E+08 | **0.85** | 20 | 30.8 | 611 | 69.1 | 5.7 | Protein metabolism | Cytoplasm;Ribosome | Translation regulator activity | Cytoplasm |
| **NCL** | NP_005372.2 | 4691 | **nucleolin** | 43.2 | 1 | 45 | 45 | 678 | 4.80E+09 | **0.85** | 113 | 13.1 | 710 | 76.6 | 4.7 | Regulation of nucleobase, nucleoside, nucleotide and nucleic acid metabolism | Nucleolus;Nucleus;Cytoplasm;Plasma membrane | RNA binding | Nucleolus |
| **RBKS** | NP_071411.1 | 64080 | **ribokinase isoform 1** | 3.1 | 1 | 1 | 1 | 1 | 3.55E+07 | **0.85** | 1 | - | 322 | 34.1 | 5.1 | Metabolism;Energy pathways | - | Catalytic activity | - |
| **ADSL** | NP_000017.1 | 158 | **adenylosuccinate lyase isoform a** | 44.6 | 2 | 15 | 15 | 34 | 1.96E+08 | **0.85** | 14 | 14.6 | 484 | 54.9 | 7.1 | Metabolism;Energy pathways | Cytoplasm | Lyase activity | Cytoplasm |
| **EEF1D** | NP_001123528.1 | 1936 | **elongation factor 1-delta isoform 4** | 53.7 | 1 | 1 | 12 | 180 | 2.74E+09 | **0.85** | 2 | 4.8 | 257 | 28.5 | 4.9 | Cell communication;Signal transduction | Endoplasmic reticulum;Mitochondrion;Nucleus;Cytoplasm | Guanyl-nucleotide exchange factor activity | Endoplasmic reticulum |
| **EPRS** | NP_004437.2 | 2058 | **bifunctional glutamate/proline--tRNA ligase** | 57.3 | 1 | 71 | 71 | 234 | 1.04E+09 | **0.85** | 96 | 21.2 | 1512 | 170.5 | 7.3 | Protein metabolism | Cytoplasm;Nucleus | Ligase activity | Cytoplasm |
| **GOT2** | NP_002071.2 | 2806 | **aspartate aminotransferase, mitochondrial isoform 1 precursor** | 49.5 | 2 | 20 | 20 | 120 | 1.88E+09 | **0.85** | 37 | 21.1 | 430 | 47.5 | 9.0 | Metabolism;Energy pathways | Mitochondrion;Nucleolus | Transaminase activity | Mitochondrion |
| **HNRNPC** | NP_004491.2 | 3183 | **heterogeneous nuclear ribonucleoproteins C1/C2 isoform b** | 65.9 | 5 | 2 | 22 | 175 | 5.14E+09 | **0.85** | 3 | 7.6 | 293 | 32.3 | 5.1 | Regulation of nucleobase, nucleoside, nucleotide and nucleic acid metabolism | Nucleus;Cytoplasm;Nucleolus;Mitochondrion | RNA binding | Nucleus |
| **P4HA2** | NP_001017974.1 | 8974 | **prolyl 4-hydroxylase subunit alpha-2 isoform 2 precursor** | 17.5 | 2 | 6 | 6 | 9 | 9.19E+07 | **0.85** | 5 | 79.4 | 533 | 60.6 | 5.7 | Metabolism;Energy pathways;Peptide metabolism | Endoplasmic reticulum | Catalytic activity | Endoplasmic reticulum |
| **GTF2A1** | NP_056943.1 | 2957 | **transcription initiation factor IIA subunit 1 isoform 1** | 5.9 | 1 | 2 | 2 | 9 | 1.23E+08 | **0.85** | 3 | 55.8 | 376 | 41.5 | 4.6 | Regulation of nucleobase, nucleoside, nucleotide and nucleic acid metabolism | Kinetochore;Nucleus;Cytoplasm | Transcription factor activity | Nucleus |
| **SPATA5L1** | NP_076968.2 | 79029 | **spermatogenesis-associated protein 5-like protein 1** | 11.8 | 1 | 4 | 4 | 5 | 7.21E+07 | **0.85** | 3 | 3.4 | 753 | 80.7 | 8.1 | Biological_process unknown | - | Molecular function unknown | - |
| **AIMP1** | NP_004748.2 | 9255 | **aminoacyl tRNA synthase complex-interacting multifunctional protein 1 isoform a precursor** | 53.2 | 2 | 11 | 11 | 50 | 6.43E+08 | **0.85** | 16 | 31.1 | 312 | 34.3 | 8.4 | Immune response | Nucleus;Cytoplasm;Extracellular;Microtubule;Ribosome | Cytokine activity | Extracellular |
| **ARHGEF7** | NP_003890.1 | 8874 | **rho guanine nucleotide exchange factor 7 isoform a** | 19.0 | 5 | 8 | 8 | 17 | 8.68E+07 | **0.85** | 5 | 7.4 | 646 | 73.1 | 7.2 | Cell communication;Signal transduction;Regulation of signal transduction | Cytoplasm;Synapse;Plasma membrane | Guanyl-nucleotide exchange factor activity;Molecular function unknown | Cytoplasm |
| **CLPB** | NP_001245323.1 | 81570 | **caseinolytic peptidase B protein homolog isoform 4** | 12.2 | 4 | 6 | 6 | 7 | 3.52E+08 | **0.85** | 4 | 25.5 | 662 | 74.5 | 8.7 | Metabolism;Energy pathways | Nucleus | ATPase activity | Nucleus |
| **PLEK2** | NP_057529.1 | 26499 | **pleckstrin-2** | 12.8 | 1 | 2 | 2 | 3 | 5.35E+07 | **0.85** | 2 | 49.3 | 353 | 39.9 | 9.4 | Cell growth and/or maintenance | Plasma membrane | Cytoskeletal protein binding | Plasma membrane |

| Gene Symbol | RefSeq_Accession | Entrez Gene ID | Description | Coverage (%) | Proteins | Unique Peptides | # Peptides | PSM | Area | SCC-R /SCC-S | SCC-R /SCC-S (Count) | SCC-R /SCC-S (Variability [%]) | # AAs | MW [kDa] | calc. pI | Biological Process | Localization | Molecular Function | Primary Localization |
|---|---|---|---|---|---|---|---|---|---|---|---|---|---|---|---|---|---|---|---|
| **PPP3CA** | NP_001124163.1 | 5530 | **serine/threonine-protein phosphatase 2B catalytic subunit alpha isoform isoform 2** | 24.9 | 11 | 8 | 8 | 15 | 2.17E+08 | **0.85** | 5 | 49.2 | 511 | 57.6 | 6.3 | Cell communication;Signal transduction | Cytoplasm;Nucleus | Protein serine/threonine phosphatase activity | Cytoplasm |
| **PPP4R1** | NP_005125.1 | 9989 | **serine/threonine-protein phosphatase 4 regulatory subunit 1 isoform b** | 16.2 | 2 | 7 | 7 | 11 | 1.91E+08 | **0.85** | 6 | 26.2 | 933 | 105.1 | 4.8 | Cell communication;Signal transduction | Centrosome | Enzyme regulator activity | Centrosome |
| **FSCN1** | NP_003079.1 | 6624 | **fascin** | 68.0 | 1 | 27 | 27 | 199 | 2.66E+09 | **0.85** | 36 | 27.3 | 493 | 54.5 | 7.2 | Cell growth and/or maintenance | Plasma membrane;Cytoplasm;Cytosol | Structural molecule activity | Cytoplasm |
| **SCAMP2** | NP_005688.2 | 10066 | **secretory carrier-associated membrane protein 2** | 9.7 | 1 | 2 | 2 | 5 | 9.35E+07 | **0.85** | 2 | 27.2 | 329 | 36.6 | 6.1 | Transport | Plasma membrane;Cytoplasm;Secretory granule | Auxiliary transport protein activity | Plasma membrane |
| **UBXN4** | NP_055422.1 | 23190 | **UBX domain-containing protein 4** | 31.3 | 1 | 7 | 7 | 13 | 1.10E+08 | **0.85** | 7 | 28.1 | 508 | 56.7 | 6.4 | Biological_process unknown | - | Molecular function unknown | - |
| **CIAPIN1** | NP_064709.2 | 57019 | **anamorsin** | 42.0 | 1 | 8 | 8 | 23 | 5.98E+08 | **0.84** | 10 | 17.9 | 312 | 33.6 | 5.6 | Cell growth and/or maintenance | Cytoplasm | Molecular function unknown | Cytoplasm |
| **MORF4L2** | NP_001135900.1 | 9643 | **mortality factor 4-like protein 2** | 6.3 | 1 | 1 | 1 | 2 | 6.35E+07 | **0.84** | 1 | - | 288 | 32.3 | 9.7 | Regulation of nucleobase, nucleoside, nucleotide and nucleic acid metabolism | Nucleus;Cytoplasm | Transcription regulator activity | Nucleus |
| **NOM1** | NP_612409.1 | 64434 | **nucleolar MIF4G domain-containing protein 1** | 5.6 | 1 | 3 | 3 | 6 | 3.58E+07 | **0.84** | 2 | 3.8 | 860 | 96.2 | 8.1 | Biological_process unknown | Nucleolus | Molecular function unknown | Nucleolus |
| **PI4KA** | NP_477352.3 | 5297 | **phosphatidylinositol 4-kinase alpha isoform 1** | 4.1 | 2 | 5 | 5 | 6 | 2.43E+07 | **0.84** | 3 | 35.4 | 2102 | 236.7 | 7.1 | Cell communication;Signal transduction | Golgi apparatus;Cytoplasm;Membrane fraction | Lipid kinase activity | Golgi apparatus |
| **RCN2** | NP_002893.1 | 5955 | **reticulocalbin-2 isoform a precursor** | 35.0 | 2 | 7 | 7 | 27 | 2.38E+08 | **0.84** | 4 | 9.5 | 317 | 36.9 | 4.4 | Cell communication;Signal transduction | Endoplasmic reticulum | Calcium ion binding | Endoplasmic reticulum |
| **C9orf78** | NP_057604.1 | 51759 | **uncharacterized protein C9orf78** | 17.7 | 1 | 3 | 3 | 7 | 6.68E+07 | **0.84** | 2 | 12.6 | 289 | 33.7 | 6.7 | Biological_process unknown | Nucleus;Cytoplasm | Molecular function unknown | Nucleus;Cytoplasm |
| **DIP2B** | NP_775873.2 | 57609 | **disco-interacting protein 2 homolog B** | 2.4 | 1 | 2 | 2 | 3 | 3.34E+07 | **0.84** | 1 | - | 1576 | 171.4 | 8.1 | Biological_process unknown | - | Molecular function unknown | - |
| **MTX1** | NP_002446.3 | 4580 | **metaxin-1 isoform 1** | 11.6 | 2 | 4 | 4 | 6 | 6.84E+07 | **0.84** | 1 | - | 466 | 51.4 | 9.8 | Transport | Mitochondrion;Cytosol;Perinuclear region | Auxiliary transport protein activity;Transporter activity | Mitochondrion |
| **SEL1L** | NP_005056.3 | 6400 | **protein sel-1 homolog 1 isoform 1 precursor** | 5.4 | 1 | 2 | 2 | 4 | 6.29E+07 | **0.84** | 1 | - | 794 | 88.7 | 5.4 | Biological_process unknown | Cytoplasmic vesicle | Binding | Cytoplasmic vesicle |
| **XPO7** | NP_055839.3 | 23039 | **exportin-7** | 12.7 | 1 | 8 | 8 | 14 | 8.68E+07 | **0.84** | 7 | 18.1 | 1087 | 123.8 | 6.3 | Transport | Nucleus;Cytoplasm | Transporter activity | Nucleus |
| **DDX27** | NP_060365.7 | 55661 | **probable ATP-dependent RNA helicase DDX27** | 17.6 | 1 | 11 | 11 | 15 | 5.86E+07 | **0.84** | 8 | 34.1 | 796 | 89.8 | 9.3 | Regulation of nucleobase, nucleoside, nucleotide and nucleic acid metabolism | Nucleus;Nucleolus | Helicase activity | Nucleus |
| **KIF1B** | NP_904325.2 | 23095 | **kinesin-like protein KIF1B isoform alpha** | 1.7 | 1 | 1 | 2 | 2 | 1.14E+07 | **0.84** | 1 | - | 1153 | 130.3 | 8.4 | Cell growth and/or maintenance | Cytoplasmic vesicle;Mitochondrion;Microtubule;Cytoplasm | Motor activity | Cytoplasmic vesicle |
| **PC** | NP_071504.2 | 5091 | **pyruvate carboxylase, mitochondrial precursor** | 6.5 | 1 | 4 | 4 | 6 | 2.51E+07 | **0.84** | 2 | 7.5 | 1178 | 129.6 | 6.8 | Metabolism;Energy pathways | Mitochondrion | Ligase activity | Mitochondrion |
| **PLCB3** | NP_001171812.1 | 5331 | **1-phosphatidylinositol 4,5-bisphosphate phosphodiesterase beta-3 isoform 2** | 17.5 | 2 | 12 | 12 | 21 | 2.31E+08 | **0.84** | 9 | 45.1 | 1167 | 131.1 | 5.9 | Metabolism;Energy pathways | Cytoplasm;Extracellular;Nucleus | Phospholipase activity | Cytoplasm |
| **PRC1** | NP_001254509.1 | 9055 | **protein regulator of cytokinesis 1 isoform 4** | 2.1 | 3 | 1 | 1 | 2 | 5.66E+07 | **0.84** | 1 | - | 525 | 61.4 | 7.7 | Cell communication;Signal transduction;Cell cycle | Nucleus;Cytoskeleton;Microtubule cytoskeleton;Extracellular space;Microtubule | Cytoskeletal protein binding | Nucleus;Microtubule |
| **TEX10** | NP_001155056.1 | 54881 | **testis-expressed sequence 10 protein isoform 2** | 19.5 | 2 | 11 | 11 | 14 | 9.25E+07 | **0.84** | 8 | 20.7 | 913 | 103.8 | 9.4 | Biological_process unknown | - | Molecular function unknown | - |
| **HSP90AA1** | NP_005339.3 | 3320 | **heat shock protein HSP 90-alpha isoform 2** | 74.0 | 2 | 44 | 66 | 1785 | 2.90E+10 | **0.84** | 161 | 19.2 | 732 | 84.6 | 5.0 | Protein metabolism | Cytoplasm;Nucleus;Extracellular space;Microtubule;Mitochondrion | Chaperone activity | Cytoplasm |
| **MRPS26** | NP_110438.1 | 64949 | **28S ribosomal protein S26, mitochondrial** | 16.1 | 1 | 2 | 2 | 4 | 6.46E+07 | **0.84** | 3 | 8.8 | 205 | 24.2 | 10.4 | Protein metabolism | Mitochondrion | Structural constituent of ribosome | Mitochondrion |
| **NSA2** | NP_055701.1 | 10412 | **ribosome biogenesis protein NSA2 homolog isoform 1** | 16.9 | 2 | 5 | 5 | 7 | 1.06E+08 | **0.84** | 4 | 16.7 | 260 | 30 | 10.3 | Regulation of nucleobase, nucleoside, nucleotide and nucleic acid metabolism | Nucleus;Nucleolus;Ribosome | Molecular function unknown | Nucleus |
| **POLD3** | NP_006582.1 | 10714 | **DNA polymerase delta subunit 3** | 14.8 | 1 | 5 | 5 | 5 | 6.77E+07 | **0.84** | 4 | 6.6 | 466 | 51.4 | 9.4 | Regulation of nucleobase, nucleoside, nucleotide and nucleic acid metabolism | Nucleus | DNA repair protein | Nucleus |
| **POLR2B** | NP_000929.1 | 5431 | **DNA-directed RNA polymerase II subunit RPB2** | 20.5 | 1 | 13 | 13 | 27 | 2.16E+08 | **0.84** | 11 | 23.5 | 1174 | 133.8 | 6.9 | Regulation of nucleobase, nucleoside, nucleotide and nucleic acid metabolism | Nucleus | DNA-directed RNA polymerase activity | Nucleus |
| **SUCLG2** | NP_003839.2 | 8801 | **succinyl-CoA ligase [GDP-forming] subunit beta, mitochondrial isoform 2 precursor** | 30.3 | 2 | 10 | 10 | 13 | 2.23E+08 | **0.84** | 7 | 26.1 | 432 | 46.5 | 6.4 | Metabolism;Energy pathways | Mitochondrion | Ligase activity | Mitochondrion |
| **C19orf70** | NP_991330.1 | 125988 | **protein QIL1 precursor** | 58.5 | 1 | 4 | 4 | 10 | 6.74E+07 | **0.84** | 3 | 50.5 | 118 | 13.1 | 9.4 | Biological_process unknown | - | Molecular function unknown | - |
| **CCDC137** | NP_954981.1 | 339230 | **coiled-coil domain-containing protein 137** | 9.7 | 1 | 2 | 2 | 2 | 5.31E+07 | **0.84** | 1 | - | 289 | 33.2 | 10.9 | Biological_process unknown | - | Molecular function unknown | - |
| **GALK1** | NP_000145.1 | 2584 | **galactokinase** | 35.7 | 1 | 7 | 7 | 20 | 2.30E+08 | **0.84** | 8 | 25.5 | 392 | 42.2 | 6.5 | Metabolism;Energy pathways | Cytoplasm | Catalytic activity | Cytoplasm |
| **GEMIN5** | NP_001239085.1 | 25929 | **gem-associated protein 5 isoform 2** | 4.8 | 2 | 4 | 4 | 7 | 5.02E+07 | **0.84** | 3 | 42.5 | 1507 | 168.4 | 6.7 | Regulation of nucleobase, nucleoside, nucleotide and nucleic acid metabolism | Cytoplasm;Nucleus | Ribonucleoprotein | Cytoplasm |
| **MRTO4** | NP_057267.2 | 51154 | **mRNA turnover protein 4 homolog** | 24.3 | 1 | 5 | 5 | 21 | 2.17E+08 | **0.84** | 5 | 14 | 239 | 27.5 | 8.3 | Biological_process unknown | Nucleolus | Molecular function unknown | Nucleolus |
| **NELFCD** | NP_945327.2 | 51497 | **negative elongation factor C/D** | 5.0 | 1 | 2 | 2 | 2 | 4.46E+06 | **0.84** | 1 | - | 599 | 67.3 | 5.2 | Regulation of nucleobase, nucleoside, nucleotide and nucleic acid metabolism | Nucleus;Cytoplasm | Transcription regulator activity | Nucleus |
| **P4HA1** | NP_001136068.1 | 5033 | **prolyl 4-hydroxylase subunit alpha-1 isoform 3 precursor** | 21.3 | 3 | 7 | 7 | 18 | 1.68E+08 | **0.84** | 10 | 21.1 | 516 | 58.9 | 6.0 | Metabolism;Energy pathways;Protein metabolism | Endoplasmic reticulum | Oxidoreductase activity | Endoplasmic reticulum |
| **PPP2R1A** | NP_055040.2 | 5518 | **serine/threonine-protein phosphatase 2A 65 kDa regulatory subunit A alpha isoform** | 56.2 | 1 | 25 | 31 | 156 | 2.25E+09 | **0.84** | 39 | 15.2 | 589 | 65.3 | 5.1 | Cell communication;Signal transduction | Cytoplasm;Chromosome;Membrane fraction;Microtubule | Protein serine/threonine phosphatase activity | Cytoplasm |
| **TDRD5** | NP_775804.2 | 163589 | **tudor domain-containing protein 5 isoform 2** | 0.8 | 2 | 1 | 1 | 1 | 1.99E+08 | **0.84** | 1 | - | 981 | 109.7 | 8.0 | Regulation of nucleobase, nucleoside, nucleotide and nucleic acid metabolism | - | RNA binding | - |
| **XRCC5** | NP_066964.1 | 7520 | **X-ray repair cross-complementing protein 5** | 61.9 | 1 | 33 | 33 | 283 | 1.56E+09 | **0.84** | 74 | 17.3 | 732 | 82.7 | 5.8 | Regulation of nucleobase, nucleoside, nucleotide and nucleic acid metabolism | Nucleus;Cytoplasm;Nucleolus;Mitochondrion | DNA repair protein | Nucleus |

| Gene Symbol | RefSeq_Accession | Entrez Gene ID | Description | Coverage (%) | Proteins | Unique Peptides | # Peptides | PSM | Area | SCC-R /SCC-S | SCC-R /SCC-S (Count) | SCC-R /SCC-S (Variability [%]) | # AAs | MW [kDa] | calc. pI | Biological Process | Localization | Molecular Function | Primary Localization |
|---|---|---|---|---|---|---|---|---|---|---|---|---|---|---|---|---|---|---|---|
| **DDOST** | NP_005207.2 | 1650 | **dolichyl-diphosphooligosaccharide--protein glycosyltransferase 48 kDa subunit precursor** | 34.2 | 1 | 8 | 8 | 18 | 7.82E+08 | **0.84** | 9 | 44 | 456 | 50.7 | 6.4 | Metabolism;Energy pathways | Endoplasmic reticulum;Mitochondrion | Galactosyltransferase activity | Endoplasmic reticulum |
| **E2F4** | NP_001941.2 | 1874 | **transcription factor E2F4** | 5.1 | 1 | 1 | 1 | 1 | 6.44E+07 | **0.84** | 1 | - | 413 | 43.9 | 4.8 | Regulation of nucleobase, nucleoside, nucleotide and nucleic acid metabolism | Cytoplasm;Nucleus;Nucleolus | Transcription factor activity | Nucleus |
| **ESYT1** | NP_056107.1 | 23344 | **extended synaptotagmin-1 isoform 2** | 33.0 | 2 | 24 | 24 | 79 | 4.08E+08 | **0.84** | 31 | 23.2 | 1104 | 122.8 | 5.8 | Cell communication;Signal transduction | Plasma membrane;Microsome;Golgi apparatus;Endoplasmic reticulum;Mitochondrion | Calcium ion binding | Plasma membrane |
| **KRI1** | NP_075384.3 | 65095 | **protein KRI1 homolog** | 14.3 | 1 | 5 | 5 | 7 | 3.02E+07 | **0.84** | 4 | 21 | 709 | 83.2 | 5.2 | Biological_process unknown | Nucleus;Cytoplasm | Molecular function unknown | Nucleus;Cytoplasm |
| **NFYB** | NP_006157.1 | 4801 | **nuclear transcription factor Y subunit beta** | 10.1 | 1 | 1 | 1 | 1 | 2.68E+06 | **0.84** | 1 | - | 207 | 22.8 | 4.6 | Regulation of nucleobase, nucleoside, nucleotide and nucleic acid metabolism | Nucleus | Transcription factor activity | Nucleus |
| **TIMM13** | NP_036590.1 | 26517 | **mitochondrial import inner membrane translocase subunit Tim13** | 43.2 | 1 | 2 | 2 | 4 | 9.35E+07 | **0.84** | 2 | 10.4 | 95 | 10.5 | 8.2 | Transport | Mitochondrion | Transporter activity | Mitochondrion |
| **COIL** | NP_004636.1 | 8161 | **coilin** | 10.8 | 1 | 4 | 4 | 6 | 6.46E+07 | **0.84** | 3 | 4 | 576 | 62.6 | 9.1 | Regulation of nucleobase, nucleoside, nucleotide and nucleic acid metabolism | Nucleus;Nucleolus | RNA binding | Nucleus |
| **OTUB1** | NP_060140.2 | 55611 | **ubiquitin thioesterase OTUB1** | 37.3 | 1 | 8 | 8 | 24 | 6.33E+08 | **0.84** | 8 | 11.2 | 271 | 31.3 | 4.9 | Protein metabolism | - | Ubiquitin-specific protease activity | - |
| **PI4KB** | NP_001185702.1 | 5298 | **phosphatidylinositol 4-kinase beta isoform 2** | 5.1 | 3 | 2 | 2 | 2 | 3.13E+07 | **0.84** | 2 | 32 | 801 | 89.8 | 6.4 | Cell communication;Signal transduction | Cytoplasm;Mitochondrion;Endoplasmic reticulum;Golgi apparatus;Plasma membrane | Lipid kinase activity | Cytoplasm |
| **RPL8** | NP_000964.1 | 6132 | **60S ribosomal protein L8** | 52.9 | 1 | 12 | 12 | 108 | 1.26E+09 | **0.84** | 32 | 21.6 | 257 | 28 | 11.0 | Protein metabolism | Nucleolus;Mitochondrion | Structural constituent of ribosome | Nucleolus |
| **EIF3A** | NP_003741.1 | 8661 | **eukaryotic translation initiation factor 3 subunit A** | 30.0 | 1 | 38 | 38 | 132 | 1.45E+09 | **0.84** | 49 | 22 | 1382 | 166.5 | 6.8 | Protein metabolism | Cytoplasm | Translation regulator activity | Cytoplasm |
| **PHB2** | NP_001138303.1 | 11331 | **prohibitin-2 isoform 1** | 58.9 | 2 | 15 | 15 | 96 | 1.46E+09 | **0.84** | 32 | 18.1 | 299 | 33.3 | 9.8 | Regulation of nucleobase, nucleoside, nucleotide and nucleic acid metabolism | Mitochondrion;Cytoplasm;Nucleus;Cytosol | Transcription regulator activity | Nucleus |
| **RPS15** | NP_001009.1 | 6209 | **40S ribosomal protein S15** | 55.9 | 1 | 5 | 5 | 105 | 2.54E+09 | **0.84** | 27 | 21.5 | 145 | 17 | 10.4 | Regulation of nucleobase, nucleoside, nucleotide and nucleic acid metabolism | Ribosome | Structural constituent of ribosome | Ribosome |
| **VCP** | NP_009057.1 | 7415 | **transitional endoplasmic reticulum ATPase** | 84.6 | 2 | 66 | 66 | 548 | 4.31E+09 | **0.84** | 140 | 21.7 | 806 | 89.3 | 5.3 | Metabolism;Energy pathways | Endoplasmic reticulum;Cytoplasm;Nucleus;Golgi apparatus;Nucleolus;Perinuclear region;Mitochondrion | ATPase activity | Cytoplasm;Nucleus;Nucleolus;Perinuclear region |
| **ATP5B** | NP_001677.2 | 506 | **ATP synthase subunit beta, mitochondrial precursor** | 69.2 | 1 | 26 | 26 | 360 | 2.35E+09 | **0.84** | 66 | 21.5 | 529 | 56.5 | 5.4 | Metabolism;Energy pathways | Mitochondrion;Plasma membrane;Nucleolus;Extracellular;Zymogen granule | Transporter activity | Mitochondrion |
| **FLII** | NP_002009.1 | 2314 | **protein flightless-1 homolog isoform 1** | 23.3 | 3 | 17 | 17 | 53 | 4.00E+08 | **0.84** | 19 | 22.1 | 1269 | 144.7 | 6.1 | Cell growth and/or maintenance | Cytoplasm;Nucleus | Cytoskeletal protein binding | Nucleus |
| **NPC2** | NP_006423.1 | 10577 | **epididymal secretory protein E1 precursor** | 41.1 | 1 | 5 | 5 | 32 | 4.76E+08 | **0.84** | 8 | 51 | 151 | 16.6 | 7.7 | Metabolism;Energy pathways | Cytoplasm;Lysosome | Transporter activity | Lysosome |
| **PRPF6** | NP_036601.2 | 24148 | **pre-mRNA-processing factor 6** | 20.8 | 1 | 15 | 15 | 40 | 4.93E+08 | **0.84** | 15 | 22.4 | 941 | 106.9 | 8.3 | Cell communication;Signal transduction | Nucleus | Receptor signaling complex scaffold activity | Nucleus |
| **RILPL1** | NP_847884.2 | 353116 | **RILP-like protein 1** | 10.9 | 1 | 2 | 2 | 2 | 2.23E+07 | **0.84** | 1 | - | 403 | 47.1 | 5.2 | Biological_process unknown | - | Molecular function unknown | - |
| **NDRG1** | NP_001128714.1 | 10397 | **protein NDRG1 isoform 1** | 43.2 | 3 | 10 | 10 | 28 | 1.81E+08 | **0.84** | 7 | 30.1 | 394 | 42.8 | 5.8 | Cell communication;Signal transduction | Cytoplasm;Nucleus | Molecular function unknown | Cytoplasm |
| **BROX** | NP_653296.2 | 148362 | **BRO1 domain-containing protein BROX isoform a** | 29.2 | 3 | 7 | 7 | 18 | 1.66E+08 | **0.83** | 8 | 24.5 | 411 | 46.4 | 7.7 | Biological_process unknown | - | Molecular function unknown | - |
| **NME3** | NP_002504.2 | 4832 | **nucleoside diphosphate kinase 3 precursor** | 10.1 | 1 | 1 | 1 | 1 | 4.17E+07 | **0.83** | 1 | - | 169 | 19 | 7.8 | Metabolism;Energy pathways | Mitochondrion;Cytoplasm | Catalytic activity | Mitochondrion |
| **API5** | NP_006586.1 | 8539 | **apoptosis inhibitor 5 isoform b** | 42.7 | 4 | 21 | 21 | 63 | 3.84E+08 | **0.83** | 23 | 19.4 | 504 | 56.7 | 5.9 | Apoptosis | Nucleus;Cytoplasm;Nucleolus | Molecular function unknown | Nucleus |
| **GNL1** | NP_005266.2 | 2794 | **guanine nucleotide-binding protein-like 1** | 20.4 | 1 | 9 | 9 | 19 | 1.87E+08 | **0.83** | 8 | 24.8 | 607 | 68.6 | 5.8 | Cell communication;Signal transduction | Nucleus;Cytoplasm | Molecular function unknown | Nucleus;Cytoplasm |
| **IKBIP** | NP_963906.1 | 121457 | **inhibitor of nuclear factor kappa-B kinase-interacting protein isoform 2** | 10.0 | 1 | 2 | 3 | 4 | 3.70E+07 | **0.83** | 2 | 26.4 | 350 | 39.3 | 9.2 | Apoptosis | Endoplasmic reticulum | Molecular function unknown | Endoplasmic reticulum |
| **KCNK13** | NP_071337.2 | 56659 | **potassium channel subfamily K member 13** | 2.0 | 1 | 1 | 1 | 1 | 7.08E+07 | **0.83** | 1 | - | 408 | 45.4 | 8.4 | Transport | Plasma membrane | Inward rectifier channel | Plasma membrane |
| **RPL27** | NP_000979.1 | 6155 | **60S ribosomal protein L27** | 39.0 | 1 | 5 | 5 | 40 | 8.43E+08 | **0.83** | 7 | 10.6 | 136 | 15.8 | 10.6 | Protein metabolism | Cytoplasm;Nucleolus | Structural constituent of ribosome | Cytoplasm |
| **SF3B6** | NP_057131.1 | 51639 | **splicing factor 3B subunit 6** | 22.4 | 1 | 2 | 2 | 14 | 1.45E+08 | **0.83** | 3 | 5.7 | 125 | 14.6 | 9.4 | Regulation of nucleobase, nucleoside, nucleotide and nucleic acid metabolism | Nucleus;Nucleolus | RNA binding | Nucleus |
| **BCAP31** | NP_001132913.1 | 10134 | **B-cell receptor-associated protein 31 isoform b** | 24.0 | 2 | 6 | 6 | 24 | 3.17E+08 | **0.83** | 11 | 31.9 | 246 | 28 | 8.4 | Transport | Endoplasmic reticulum;Golgi apparatus;Mitochondrion | Transporter activity | Endoplasmic reticulum |
| **MED1** | NP_004765.2 | 5469 | **mediator of RNA polymerase II transcription subunit 1** | 2.7 | 1 | 2 | 2 | 4 | 7.66E+06 | **0.83** | 2 | 25.5 | 1581 | 168.4 | 8.7 | Cell communication;Signal transduction | Nucleus;Kinetochore | Transcription regulator activity | Nucleus |
| **PTCD3** | NP_060422.4 | 55037 | **pentatricopeptide repeat domain-containing protein 3, mitochondrial precursor** | 20.3 | 1 | 8 | 8 | 13 | 1.03E+08 | **0.83** | 5 | 57.6 | 689 | 78.5 | 6.4 | Biological_process unknown | - | Protein binding | - |
| **RABGGTA** | NP_878256.1 | 5875 | **geranylgeranyl transferase type-2 subunit alpha** | 13.2 | 1 | 5 | 5 | 11 | 5.85E+07 | **0.83** | 6 | 18.3 | 567 | 65 | 5.7 | Protein modification | Cytoplasm | Transferase activity | Cytoplasm |
| **TRMT2B** | NP_001161443.1 | 79979 | **tRNA (uracil(54)-C(5))-methyltransferase homolog isoform b** | 3.3 | 2 | 1 | 1 | 2 | 9.49E+06 | **0.83** | 1 | - | 459 | 51.5 | 8.5 | Regulation of nucleobase, nucleoside, nucleotide and nucleic acid metabolism | - | Methyltransferase activity | - |
| **MAPK9** | NP_620707.1 | 5601 | **mitogen-activated protein kinase 9 isoform alpha1** | 7.1 | 5 | 1 | 2 | 2 | 5.06E+07 | **0.83** | 1 | - | 382 | 44 | 6.4 | Regulation of cell cycle;Apoptosis;Signal transduction | Nucleus;Cytoplasm | Protein serine/threonine kinase activity | Nucleus |
| **PIN4** | NP_001164218.1 | 5303 | **peptidyl-prolyl cis-trans isomerase NIMA-interacting 4 isoform 2** | 10.5 | 2 | 1 | 1 | 3 | 1.84E+08 | **0.83** | 1 | - | 133 | 14.2 | 10.2 | Metabolism;Energy pathways | Nucleus;Cytoplasm;Mitochondrion | Isomerase activity | Nucleus |
| **PLXNB2** | NP_036533.2 | 23654 | **plexin-B2 precursor** | 10.1 | 1 | 10 | 10 | 18 | 2.37E+08 | **0.83** | 11 | 23.8 | 1838 | 205 | 6.2 | Cell communication;Signal transduction | Plasma membrane | Receptor activity | Plasma membrane |
| **PRPSAP2** | NP_001230870.1 | 5636 | **phosphoribosyl pyrophosphate synthase-associated protein 2 isoform 4** | 33.2 | 4 | 5 | 6 | 18 | 1.47E+08 | **0.83** | 9 | 32.6 | 283 | 31.3 | 7.2 | Regulation of nucleobase, nucleoside, nucleotide and nucleic acid metabolism | Nucleus;Cytoplasm | Molecular function unknown | Nucleus;Cytoplasm |

| Gene Symbol | RefSeq_Accession | Entrez Gene ID | Description | Coverage (%) | Proteins | Unique Peptides | # Peptides | PSM | Area | SCC-R /SCC-S | SCC-R /SCC-S (Count) | SCC-R /SCC-S (Variability [%]) | # AAs | MW [kDa] | calc. pI | Biological Process | Localization | Molecular Function | Primary Localization |
|---|---|---|---|---|---|---|---|---|---|---|---|---|---|---|---|---|---|---|---|
| **TTC7B** | NP_001010854.1 | 145567 | **tetratricopeptide repeat protein 7B** | 3.2 | 1 | 1 | 1 | 1 | 6.03E+06 | **0.83** | 1 | - | 843 | 94.1 | 6.9 | Biological_process unknown | - | Molecular function unknown | - |
| **WDR61** | NP_079510.1 | 80349 | **WD repeat-containing protein 61** | 38.0 | 1 | 8 | 8 | 15 | 1.68E+08 | **0.83** | 8 | 26 | 305 | 33.6 | 5.5 | Biological_process unknown | - | Molecular function unknown | - |
| **BET1L** | NP_057610.2 | 51272 | **BET1-like protein isoform 2** | 16.7 | 2 | 1 | 1 | 1 | 5.82E+07 | **0.83** | 1 | - | 102 | 11.1 | 7.3 | Transport | Golgi apparatus;Endosome | Transporter activity | Golgi apparatus |
| **CNOT1** | NP_057368.3 | 23019 | **CCR4-NOT transcription complex subunit 1 isoform a** | 15.1 | 3 | 23 | 23 | 40 | 1.03E+08 | **0.83** | 14 | 43.2 | 2376 | 266.8 | 7.1 | Transcription | Nucleus | Transcription regulator activity | Nucleus |
| **CNOT2** | NP_001186232.1 | 4848 | **CCR4-NOT transcription complex subunit 2** | 3.2 | 1 | 1 | 1 | 1 | 6.72E+07 | **0.83** | 1 | - | 540 | 59.7 | 7.7 | Regulation of nucleobase, nucleoside, nucleotide and nucleic acid metabolism | Nucleus | Transcription regulator activity | Nucleus |
| **FXR1** | NP_005078.2 | 8087 | **fragile X mental retardation syndrome-related protein 1 isoform a** | 34.9 | 2 | 3 | 16 | 55 | 3.38E+08 | **0.83** | 1 | - | 621 | 69.7 | 6.2 | Regulation of nucleobase, nucleoside, nucleotide and nucleic acid metabolism;Regulation of translation | Cytoplasm;Nucleus;Nucleolus | RNA binding | Nucleolus |
| **RNASEH2B** | NP_078846.2 | 79621 | **ribonuclease H2 subunit B isoform 1** | 21.8 | 2 | 4 | 4 | 7 | 3.54E+07 | **0.83** | 3 | 6.4 | 312 | 35.1 | 9.1 | Biological_process unknown | - | Molecular function unknown | - |
| **CUL5** | NP_003469.2 | 8065 | **cullin-5** | 1.3 | 1 | 1 | 1 | 2 | 2.25E+07 | **0.83** | 1 | - | 780 | 90.9 | 7.9 | Protein metabolism | Plasma membrane;Cytoplasm;Nucleus | Ubiquitin-specific protease activity | Cytoplasm |
| **HSPA8** | NP_006588.1 | 3312 | **heat shock cognate 71 kDa protein isoform 1** | 62.4 | 2 | 33 | 46 | 904 | 1.67E+10 | **0.83** | 119 | 21.9 | 646 | 70.9 | 5.5 | Protein metabolism | Cytoplasm;Nucleus;Nucleolus;Mitochondrion;Cytosol | Heat shock protein activity | Nucleolus;Cytoplasm |
| **MRPS30** | NP_057724.2 | 10884 | **28S ribosomal protein S30, mitochondrial** | 12.1 | 1 | 3 | 3 | 4 | 1.04E+08 | **0.83** | 3 | 3.1 | 439 | 50.3 | 8.0 | Protein metabolism | Mitochondrion | Structural molecule activity | Mitochondrion |
| **NIPSNAP1** | NP_001189431.1 | 8508 | **protein NipSnap homolog 1 isoform 2** | 23.1 | 2 | 5 | 5 | 20 | 5.77E+08 | **0.83** | 7 | 12.7 | 264 | 31.4 | 9.1 | Biological_process unknown | Mitochondrion | Molecular function unknown | Mitochondrion |
| **SURF4** | NP_001267718.1 | 6836 | **surfeit locus protein 4 isoform 3** | 31.5 | 6 | 4 | 4 | 9 | 2.49E+08 | **0.83** | 6 | 6.6 | 159 | 18 | 6.1 | Transport | Cytoplasm;Endoplasmic reticulum;Mitochondrion | Auxiliary transport protein activity | Endoplasmic reticulum |
| **AP2A1** | NP_570603.2 | 160 | **AP-2 complex subunit alpha-1 isoform 2** | 28.7 | 2 | 14 | 17 | 45 | 3.87E+08 | **0.83** | 9 | 40.3 | 955 | 105.3 | 7.7 | Transport | Plasma membrane;Mitochondrion | Transporter activity | Plasma membrane |
| **ASL** | NP_001020115.1 | 435 | **argininosuccinate lyase isoform 2** | 13.7 | 3 | 5 | 5 | 5 | 8.20E+07 | **0.83** | 3 | 22.1 | 444 | 49.5 | 6.4 | Metabolism;Energy pathways | - | Lyase activity | - |
| **POGZ** | NP_665739.3 | 23126 | **pogo transposable element with ZNF domain isoform 3** | 2.3 | 5 | 2 | 2 | 4 | 3.06E+07 | **0.83** | 2 | 4.1 | 1315 | 145.2 | 7.2 | Regulation of nucleobase, nucleoside, nucleotide and nucleic acid metabolism | Nucleus | Transcription regulator activity | Nucleus |
| **BCAS3** | NP_060149.3 | 54828 | **breast carcinoma-amplified sequence 3 isoform 2** | 1.9 | 2 | 1 | 1 | 3 | 1.01E+07 | **0.83** | 2 | 13.9 | 913 | 99.6 | 6.6 | Cell communication;Signal transduction | - | Molecular function unknown | - |
| **MEAF6** | NP_001257804.1 | 64769 | **chromatin modification-related protein MEAF6 isoform 2** | 12.0 | 3 | 1 | 1 | 1 | 9.99E+07 | **0.83** | 1 | - | 191 | 21.6 | 9.3 | Biological_process unknown | - | Molecular function unknown | - |
| **PELO** | NP_057030.3 | 53918 | **protein pelota homolog** | 10.9 | 1 | 3 | 3 | 9 | 7.31E+07 | **0.83** | 5 | 35.8 | 385 | 43.3 | 6.3 | Cell communication;Signal transduction | Nucleus;Nucleolus;Cytoplasm | Molecular function unknown | Nucleus |
| **PRPF8** | NP_006436.3 | 10594 | **pre-mRNA-processing-splicing factor 8** | 38.1 | 1 | 63 | 63 | 184 | 1.22E+09 | **0.83** | 71 | 27.5 | 2335 | 273.4 | 8.8 | Regulation of nucleobase, nucleoside, nucleotide and nucleic acid metabolism | Cytoplasm;Nucleus;Nucleolus;Chromosome | RNA binding | Nucleus |
| **TCF20** | NP_852469.1 | 6942 | **transcription factor 20 isoform 2** | 1.2 | 2 | 1 | 1 | 1 | 2.13E+07 | **0.83** | 1 | - | 1938 | 209.5 | 9.1 | Regulation of nucleobase, nucleoside, nucleotide and nucleic acid metabolism | Nucleus;Cytoplasm | Transcription factor activity | Nucleus;Cytoplasm |
| **ACTR5** | NP_079131.3 | 79913 | **actin-related protein 5** | 9.6 | 1 | 3 | 3 | 3 | 2.95E+07 | **0.83** | 1 | - | 607 | 68.3 | 5.3 | Biological_process unknown | Chromosome | Molecular function unknown | Chromosome |
| **C12orf29** | NP_001009894.2 | 91298 | **uncharacterized protein C12orf29** | 15.1 | 1 | 2 | 2 | 3 | 1.44E+08 | **0.83** | 2 | 8.1 | 325 | 37.5 | 7.1 | Biological_process unknown | - | Molecular function unknown | - |
| **DDX20** | NP_009135.4 | 11218 | **probable ATP-dependent RNA helicase DDX20** | 14.6 | 1 | 5 | 5 | 7 | 8.03E+07 | **0.83** | 4 | 15 | 824 | 92.2 | 7.0 | Metabolism;Energy pathways | Cytoplasm;Nucleus | ATPase activity | Nucleus |
| **KARS** | NP_005539.1 | 3735 | **lysine--tRNA ligase isoform 2** | 56.1 | 2 | 25 | 26 | 84 | 9.15E+08 | **0.83** | 24 | 21.3 | 597 | 68 | 6.4 | TRNA aminoacylation | Mitochondrion;Cytoplasm;Nucleus;Plasma membrane | Ligase activity | Cytoplasm |
| **KDM3B** | NP_057688.2 | 51780 | **lysine-specific demethylase 3B** | 15.0 | 1 | 12 | 12 | 17 | 1.03E+08 | **0.83** | 7 | 15.6 | 1761 | 191.5 | 7.2 | Biological_process unknown | Nucleus;Cytoplasm;Nucleolus | Molecular function unknown | Nucleus |
| **MBD3** | NP_001268383.1 | 53615 | **methyl-CpG-binding domain protein 3 isoform 2** | 17.4 | 2 | 5 | 5 | 11 | 2.86E+08 | **0.83** | 4 | 7.3 | 259 | 29 | 5.2 | Regulation of nucleobase, nucleoside, nucleotide and nucleic acid metabolism | Nucleus;Mitochondrion;Cytoplasm | Transcription regulator activity | Nucleus;Cytoplasm |
| **NAA50** | NP_079422.1 | 80218 | **N-alpha-acetyltransferase 50** | 22.5 | 1 | 3 | 3 | 4 | 7.89E+07 | **0.83** | 2 | 9.3 | 169 | 19.4 | 8.8 | Biological_process unknown | - | Acyltransferase activity | - |
| **NUCB1** | NP_006175.2 | 4924 | **nucleobindin-1 precursor** | 26.7 | 1 | 10 | 10 | 15 | 1.29E+08 | **0.83** | 9 | 25.5 | 461 | 53.8 | 5.3 | Cell communication;Signal transduction | Golgi apparatus;Extracellular;Cytoplasm;Nucleus | Calcium ion binding | Golgi apparatus |
| **PA2G4** | NP_006182.2 | 5036 | **proliferation-associated protein 2G4** | 60.4 | 1 | 23 | 23 | 203 | 3.49E+09 | **0.83** | 48 | 16.5 | 394 | 43.8 | 6.6 | Regulation of nucleobase, nucleoside, nucleotide and nucleic acid metabolism | Nucleus;Nucleolus;Cytosol;Cytoplasm | Transcription regulator activity;DNA binding | Nucleus |
| **POM121C** | NP_001092885.2 | 100101267 | **nuclear envelope pore membrane protein POM 121C** | 3.7 | 3 | 2 | 2 | 2 | 5.70E+07 | **0.83** | 1 | - | 987 | 99.1 | 9.9 | Biological_process unknown | - | Molecular function unknown | - |
| **RAB14** | NP_057406.2 | 51552 | **ras-related protein Rab-14** | 80.0 | 14 | 15 | 16 | 49 | 9.06E+08 | **0.83** | 16 | 13.3 | 215 | 23.9 | 6.2 | Cell communication;Signal transduction | Integral to membrane;Golgi apparatus;Early endosome;Mitochondrion | GTPase activity | Integral to membrane |
| **USP39** | NP_006581.2 | 10713 | **U4/U6.U5 tri-snRNP-associated protein 2 isoform 1** | 20.4 | 4 | 7 | 7 | 24 | 1.96E+08 | **0.83** | 7 | 25.4 | 565 | 65.3 | 8.9 | Protein metabolism | Nucleus;Cytoplasm | Ubiquitin-specific protease activity | Nucleus |
| **XPNPEP3** | NP_071381.1 | 63929 | **probable Xaa-Pro aminopeptidase 3 isoform 1** | 6.7 | 1 | 2 | 2 | 9 | 2.20E+08 | **0.83** | 3 | 3.1 | 507 | 57 | 6.8 | Biological_process unknown | - | Molecular function unknown | - |
| **BRAP** | NP_006759.3 | 8315 | **BRCA1-associated protein** | 5.1 | 1 | 2 | 2 | 4 | 3.58E+07 | **0.83** | 1 | - | 592 | 67.3 | 5.9 | Cell communication;Signal transduction | Cytoplasm | Receptor signaling complex scaffold activity | Cytoplasm |
| **CCAR2** | NP_066997.3 | 57805 | **cell cycle and apoptosis regulator protein 2** | 35.2 | 1 | 19 | 19 | 50 | 4.61E+08 | **0.83** | 13 | 24.3 | 923 | 102.8 | 5.2 | Signal transduction | Nucleus;Cytoplasm;Mitochondrion | Calcium ion binding | Nucleus |
| **COPRS** | NP_060875.2 | 55352 | **coordinator of PRMT5 and differentiation stimulator** | 8.7 | 1 | 1 | 1 | 2 | 4.97E+06 | **0.83** | 1 | - | 184 | 20.1 | 4.2 | Biological_process unknown | - | Molecular function unknown | - |
| **NT5C2** | NP_036361.1 | 22978 | **cytosolic purine 5'-nucleotidase** | 32.1 | 1 | 10 | 10 | 18 | 3.18E+08 | **0.83** | 6 | 25.7 | 561 | 64.9 | 6.1 | Metabolism;Energy pathways | Cytoplasm | Hydrolase activity | Cytoplasm |

| Gene Symbol | RefSeq_Accession | Entrez Gene ID | Description | Coverage (%) | Proteins | Unique Peptides | # Peptides | PSM | Area | SCC-R /SCC-S | SCC-R /SCC-S (Count) | SCC-R /SCC-S (Variability [%]) | # AAs | MW [kDa] | calc. pI | Biological Process | Localization | Molecular Function | Primary Localization |
|---|---|---|---|---|---|---|---|---|---|---|---|---|---|---|---|---|---|---|---|
| **PSMD5** | NP_005038.1 | 5711 | **26S proteasome non-ATPase regulatory subunit 5 isoform 1** | 53.2 | 2 | 18 | 18 | 64 | 4.71E+08 | **0.83** | 21 | 20 | 504 | 56.2 | 5.5 | Protein metabolism | Cytoplasm | Ubiquitin-specific protease activity | Cytoplasm |
| **ATXN2** | NP_002964.3 | 6311 | **ataxin-2** | 6.2 | 1 | 4 | 5 | 7 | 7.44E+07 | **0.82** | 1 | - | 1313 | 140.2 | 9.6 | Regulation of nucleobase, nucleoside, nucleotide and nucleic acid metabolism | Cytoplasm;Nucleus | RNA binding | Cytoplasm |
| **SUGP1** | NP_757386.2 | 57794 | **SURP and G-patch domain-containing protein 1** | 2.3 | 1 | 1 | 1 | 1 | 3.03E+07 | **0.82** | 1 | - | 645 | 72.4 | 7.6 | Regulation of nucleobase, nucleoside, nucleotide and nucleic acid metabolism | Nucleus | RNA binding | Nucleus |
| **GGPS1** | NP_001032354.1 | 9453 | **geranylgeranyl pyrophosphate synthase** | 3.3 | 1 | 1 | 1 | 1 | 5.92E+07 | **0.82** | 1 | - | 300 | 34.8 | 6.1 | Metabolism;Energy pathways | Cytoplasm | Transferase activity | Cytoplasm |
| **LYRM4** | NP_065141.3 | 57128 | **LYR motif-containing protein 4 isoform 1** | 35.2 | 3 | 2 | 2 | 4 | 8.94E+07 | **0.82** | 1 | - | 91 | 10.8 | 10.7 | Biological_process unknown | - | Molecular function unknown | - |
| **ARFIP1** | NP_001020766.1 | 27236 | **arfaptin-1 isoform 1** | 40.0 | 2 | 7 | 7 | 8 | 1.23E+08 | **0.82** | 6 | 41.7 | 373 | 41.7 | 6.7 | Signal transduction | Cytoplasm;Golgi apparatus | Regulator of G-protein signaling activity | Cytoplasm |
| **GOLGA5** | NP_005104.3 | 9950 | **Golgin subfamily A member 5** | 17.7 | 1 | 8 | 8 | 12 | 1.45E+08 | **0.82** | 5 | 16.6 | 731 | 82.9 | 5.8 | Transport | Golgi apparatus;Endoplasmic reticulum;Nucleus;Cytoplasm | Auxiliary transport protein activity | Golgi apparatus;Nucleus;Cytoplasm |
| **SDAD1** | NP_001275912.1 | 55153 | **protein SDA1 homolog isoform 2** | 12.0 | 3 | 5 | 5 | 6 | 6.15E+07 | **0.82** | 3 | 32.5 | 650 | 75.4 | 9.2 | Biological_process unknown | Nucleolus;Nucleus;Cytoplasm | Molecular function unknown | Nucleolus |
| **UQCRFS1** | NP_005994.2 | 7386 | **cytochrome b-c1 complex subunit Rieske, mitochondrial** | 31.4 | 1 | 6 | 6 | 25 | 3.49E+08 | **0.82** | 11 | 43 | 274 | 29.6 | 8.3 | Metabolism;Energy pathways | Mitochondrion | Catalytic activity | Mitochondrion |
| **AVEN** | NP_065104.1 | 57099 | **cell death regulator Aven** | 18.2 | 1 | 3 | 3 | 5 | 5.59E+07 | **0.82** | 4 | 8.5 | 362 | 38.5 | 5.0 | Apoptosis | Nucleus;Integral to membrane | Receptor signaling complex scaffold activity | Nucleus |
| **CD3EAP** | NP_036231.1 | 10849 | **DNA-directed RNA polymerase I subunit RPA34** | 19.8 | 1 | 4 | 4 | 6 | 1.49E+07 | **0.82** | 1 | - | 510 | 55 | 8.5 | Regulation of nucleobase, nucleoside, nucleotide and nucleic acid metabolism | Nucleolus;Nucleus;Cytoplasm | Transcription regulator activity | Nucleolus |
| **RMDN3** | NP_060615.1 | 55177 | **regulator of microtubule dynamics protein 3** | 6.2 | 1 | 2 | 2 | 3 | 6.05E+07 | **0.82** | 1 | - | 470 | 52.1 | 5.1 | Apoptosis | Mitochondrion;Nucleus | Apoptotic protease activator activity | Mitochondrion |
| **UQCRC2** | NP_003357.2 | 7385 | **cytochrome b-c1 complex subunit 2, mitochondrial precursor** | 55.6 | 1 | 14 | 14 | 80 | 6.69E+08 | **0.82** | 23 | 25.1 | 453 | 48.4 | 8.6 | Metabolism;Energy pathways | Mitochondrion | Catalytic activity | Mitochondrion |
| **DNM1L** | NP_036192.2 | 10059 | **dynamin-1-like protein isoform 1** | 62.4 | 4 | 1 | 30 | 103 | 2.62E+08 | **0.82** | 1 | - | 736 | 81.8 | 6.8 | Mitochondrion organization and biogenesis | Endoplasmic reticulum;Golgi apparatus;Cytoplasm | GTPase activity | Cytoplasm |
| **RAB22A** | NP_065724.1 | 57403 | **ras-related protein Rab-22A** | 10.8 | 1 | 1 | 1 | 2 | 3.28E+07 | **0.82** | 1 | - | 194 | 21.8 | 8.2 | Cell communication;Signal transduction | Endosome;Golgi apparatus | GTPase activity | Endosome |
| **TTC19** | NP_001258349.1 | 54902 | **tetratricopeptide repeat protein 19, mitochondrial isoform 2** | 4.4 | 2 | 1 | 1 | 2 | 6.39E+07 | **0.82** | 1 | - | 273 | 31.3 | 5.4 | Biological_process unknown | - | Molecular function unknown | - |
| **WDR55** | NP_060176.2 | 54853 | **WD repeat-containing protein 55** | 20.1 | 1 | 3 | 3 | 3 | 6.72E+07 | **0.82** | 2 | 3.6 | 383 | 42.1 | 4.9 | Biological_process unknown | - | Molecular function unknown | - |
| **BLVRA** | NP_000703.2 | 644 | **biliverdin reductase A precursor** | 48.7 | 1 | 13 | 13 | 55 | 9.51E+08 | **0.82** | 15 | 21.2 | 296 | 33.4 | 6.4 | Metabolism;Energy pathways | Cytoplasm;Nucleus | Oxidoreductase activity | Cytoplasm |
| **PES1** | NP_001269256.1 | 23481 | **pescadillo homolog isoform 3** | 30.3 | 3 | 11 | 11 | 26 | 2.95E+08 | **0.82** | 13 | 24.4 | 449 | 51.4 | 5.2 | Cell communication;Signal transduction | Nucleus;Nucleolus;Ribosome | Protein binding | Nucleus |
| **REEP6** | NP_612402.1 | 92840 | **receptor expression-enhancing protein 6** | 5.4 | 1 | 1 | 1 | 1 | 3.36E+08 | **0.82** | 1 | - | 184 | 20.7 | 8.6 | Biological_process unknown | Integral to membrane | Molecular function unknown | Integral to membrane |
| **RPLP2** | NP_000995.1 | 6181 | **60S acidic ribosomal protein P2** | 80.9 | 1 | 12 | 12 | 250 | 4.40E+09 | **0.82** | 37 | 15 | 115 | 11.7 | 4.5 | Protein metabolism | Cytoplasm;Nucleolus | Structural constituent of ribosome | Cytoplasm |
| **SPAG7** | NP_004881.2 | 9552 | **sperm-associated antigen 7** | 10.1 | 1 | 2 | 2 | 2 | 3.54E+07 | **0.82** | 2 | 4.2 | 227 | 26 | 7.9 | Regulation of nucleobase, nucleoside, nucleotide and nucleic acid metabolism | - | Molecular function unknown | - |
| **TRAF2** | NP_066961.2 | 7186 | **TNF receptor-associated factor 2** | 8.6 | 1 | 4 | 4 | 8 | 3.98E+07 | **0.82** | 5 | 56.9 | 501 | 55.8 | 7.5 | Cell communication;Signal transduction | Cytoplasm;Plasma membrane | Receptor signaling complex scaffold activity | Cytoplasm |
| **ZFP91** | NP_001183980.1 | 80829 | **E3 ubiquitin-protein ligase ZFP91 isoform 2** | 10.4 | 2 | 3 | 3 | 6 | 1.41E+08 | **0.82** | 4 | 9 | 569 | 63.3 | 7.4 | Regulation of nucleobase, nucleoside, nucleotide and nucleic acid metabolism | Nucleus;Cytoplasm | Transcription factor activity | Nucleus |
| **ELP6** | NP_001026873.2 | 54859 | **elongator complex protein 6** | 5.6 | 1 | 1 | 1 | 2 | 1.28E+08 | **0.82** | 1 | - | 266 | 29.8 | 6.8 | Biological_process unknown | - | Molecular function unknown | - |
| **HNRNPUL2** | NP_001073027.1 | 221092 | **heterogeneous nuclear ribonucleoprotein U-like protein 2** | 43.6 | 1 | 23 | 23 | 88 | 1.01E+09 | **0.82** | 26 | 13.2 | 747 | 85.1 | 4.9 | Regulation of nucleobase, nucleoside, nucleotide and nucleic acid metabolism | Nucleus | RNA binding | Nucleus |
| **NUP35** | NP_001274513.1 | 129401 | **nucleoporin NUP53 isoform b** | 30.1 | 3 | 6 | 6 | 11 | 9.67E+07 | **0.82** | 5 | 31.6 | 309 | 33.1 | 9.4 | Transport | Nuclear membrane;Nucleus | Transporter activity | Nuclear membrane |
| **C11orf54** | NP_001273000.1 | 28970 | **ester hydrolase C11orf54 isoform d** | 29.3 | 4 | 3 | 3 | 7 | 1.18E+08 | **0.82** | 5 | 25.8 | 256 | 28.7 | 7.0 | Biological_process unknown | - | Molecular function unknown | - |
| **MYBPC2** | NP_004524.3 | 4606 | **myosin-binding protein C, fast-type** | 0.5 | 1 | 1 | 1 | 2 | 9.27E+07 | **0.82** | 1 | - | 1141 | 128 | 7.5 | Cell growth and/or maintenance | Cytoplasm | Structural molecule activity | Cytoplasm |
| **SEC11A** | NP_001258849.1 | 23478 | **signal peptidase complex catalytic subunit SEC11A isoform 4** | 29.3 | 6 | 4 | 4 | 8 | 1.52E+08 | **0.82** | 5 | 4.4 | 164 | 19 | 8.4 | Protein metabolism | - | Aminopeptidase activity | - |
| **COG1** | NP_061184.1 | 9382 | **conserved oligomeric Golgi complex subunit 1** | 6.1 | 1 | 4 | 4 | 4 | 3.67E+07 | **0.82** | 3 | 21.9 | 980 | 108.9 | 7.3 | Transport | Golgi apparatus;Cytoplasm | Structural molecule activity | Golgi apparatus |
| **EEF1B2** | NP_001950.1 | 1933 | **elongation factor 1-beta** | 44.0 | 1 | 7 | 7 | 62 | 1.46E+09 | **0.82** | 15 | 10.7 | 225 | 24.7 | 4.7 | Protein metabolism | Cytoplasm | Translation regulator activity | Cytoplasm |
| **IPO11** | NP_057422.3 | 51194 | **importin-11 isoform 2** | 9.5 | 2 | 4 | 4 | 13 | 5.94E+07 | **0.82** | 3 | 12.9 | 975 | 112.5 | 5.3 | Cell communication;Signal transduction | Nucleus;Cytoplasm | Transporter activity | Nucleus |
| **ITGAE** | NP_002199.3 | 3682 | **integrin alpha-E precursor** | 0.7 | 1 | 1 | 1 | 3 | 6.13E+08 | **0.82** | 2 | 10.3 | 1179 | 130.1 | 5.7 | Cell communication;Signal transduction | Plasma membrane | Receptor activity | Plasma membrane |
| **PRCC** | NP_005964.3 | 5546 | **proline-rich protein PRCC** | 2.2 | 1 | 1 | 1 | 2 | 6.88E+07 | **0.82** | 1 | - | 491 | 52.4 | 5.1 | Cell communication;Signal transduction | Nucleus;Extracellular;Cytoplasm | Molecular function unknown | Nucleus |
| **RFX1** | NP_002909.4 | 5989 | **MHC class II regulatory factor RFX1** | 3.5 | 1 | 2 | 2 | 2 | 1.46E+07 | **0.82** | 1 | - | 979 | 104.7 | 6.3 | Regulation of nucleobase, nucleoside, nucleotide and nucleic acid metabolism | Nucleus | Transcription regulator activity | Nucleus |
| **ACAA1** | NP_001598.1 | 30 | **3-ketoacyl-CoA thiolase, peroxisomal isoform a** | 50.5 | 2 | 11 | 11 | 23 | 2.07E+08 | **0.82** | 9 | 14.2 | 424 | 44.3 | 8.4 | Metabolism;Energy pathways | Peroxisome;Cytoplasm;Mitochondrion | Acyltransferase activity | Peroxisome |

| Gene Symbol | RefSeq_Accession | Entrez Gene ID | Description | Coverage (%) | Proteins | Unique Peptides | # Peptides | PSM | Area | SCC-R /SCC-S | SCC-R /SCC-S (Count) | SCC-R /SCC-S (Variability [%]) | # AAs | MW [kDa] | calc. pI | Biological Process | Localization | Molecular Function | Primary Localization |
|---|---|---|---|---|---|---|---|---|---|---|---|---|---|---|---|---|---|---|---|
| **EIF3H** | NP_003747.1 | 8667 | **eukaryotic translation initiation factor 3 subunit H** | 56.0 | 1 | 17 | 17 | 56 | 3.84E+08 | **0.82** | 19 | 18.1 | 352 | 39.9 | 6.5 | Protein metabolism;Translation | Ribosome;Cytoplasm;Cytosol | Translation regulator activity | Cytoplasm |
| **HSPA4** | NP_002145.3 | 3308 | **heat shock 70 kDa protein 4** | 77.1 | 1 | 60 | 62 | 258 | 9.87E+08 | **0.82** | 84 | 26.7 | 840 | 94.3 | 5.2 | Protein metabolism | Golgi apparatus;Plasma membrane;Extracellular;Nucleus;Cytoplasm;Cell surface | Chaperone activity | Golgi apparatus;Cytoplasm |
| **RBM33** | NP_444271.2 | 155435 | **RNA-binding protein 33** | 5.0 | 1 | 3 | 3 | 3 | 2.91E+07 | **0.82** | 2 | 34.1 | 1170 | 129.9 | 6.9 | Regulation of nucleobase, nucleoside, nucleotide and nucleic acid metabolism | Nucleus;Cytoplasm | RNA binding | Nucleus |
| **RPS17** | NP_001012.1 | 6218 | **40S ribosomal protein S17** | 60.0 | 1 | 8 | 8 | 122 | 1.88E+09 | **0.82** | 15 | 9.1 | 135 | 15.5 | 9.9 | Protein metabolism | Ribosome;Nucleolus | Structural constituent of ribosome | Ribosome |
| **SMARCB1** | NP_001007469.1 | 6598 | **SWI/SNF-related matrix-associated actin-dependent regulator of chromatin subfamily B member 1 isoform b** | 28.5 | 2 | 6 | 6 | 15 | 2.92E+08 | **0.81** | 7 | 8.5 | 376 | 43.1 | 5.6 | Regulation of nucleobase, nucleoside, nucleotide and nucleic acid metabolism | Nucleus;Kinetochore | DNA binding | Nucleus |
| **UFL1** | NP_056138.1 | 23376 | **E3 UFM1-protein ligase 1** | 25.3 | 1 | 15 | 15 | 22 | 1.25E+08 | **0.81** | 10 | 20.3 | 794 | 89.5 | 6.8 | Biological_process unknown | Mitochondrion;Nucleus;Cytoplasm | Molecular function unknown | Mitochondrion;Nucleus;Cytoplasm |
| **BPNT1** | NP_001273079.1 | 10380 | **3'(2'),5'-bisphosphate nucleotidase 1 isoform 3** | 44.5 | 3 | 10 | 10 | 30 | 5.36E+08 | **0.81** | 10 | 14.7 | 272 | 29.2 | 8.3 | Cell communication;Signal transduction | - | Hydrolase activity | - |
| **EIF3L** | NP_001229852.1 | 51386 | **eukaryotic translation initiation factor 3 subunit L isoform 2** | 33.0 | 2 | 13 | 13 | 57 | 3.77E+08 | **0.81** | 17 | 28.4 | 516 | 61 | 7.0 | Protein metabolism | Cytoplasm | Translation regulator activity | Cytoplasm |
| **GMPPA** | NP_995319.1 | 29926 | **mannose-1-phosphate guanyltransferase alpha** | 6.4 | 1 | 3 | 3 | 3 | 1.06E+08 | **0.81** | 2 | 2.2 | 420 | 46.3 | 7.2 | Carbohydrate mediated signaling;Metabolism | - | Phosphorylase activity | - |
| **PSMA4** | NP_002780.1 | 5685 | **proteasome subunit alpha type-4 isoform 1** | 54.4 | 2 | 10 | 10 | 70 | 9.59E+08 | **0.81** | 20 | 14 | 261 | 29.5 | 7.7 | Protein metabolism | Cytoplasm;Endoplasmic reticulum;Nucleus | Ubiquitin-specific protease activity | Cytoplasm |
| **RCC1** | NP_001041664.1 | 1104 | **regulator of chromosome condensation isoform c** | 47.0 | 3 | 11 | 11 | 47 | 8.47E+08 | **0.81** | 23 | 16.2 | 421 | 44.9 | 7.5 | Cell communication;Signal transduction | Nucleus;Nucleolus;Cytoplasm | Guanyl-nucleotide exchange factor activity | Nucleus |
| **SLC7A1** | NP_003036.1 | 6541 | **high affinity cationic amino acid transporter 1** | 3.0 | 1 | 1 | 1 | 1 | 2.17E+08 | **0.81** | 1 | - | 629 | 67.6 | 5.4 | Transport | Plasma membrane | Auxiliary transport protein activity | Plasma membrane |
| **COL4A3BP** | NP_112729.1 | 10087 | **collagen type IV alpha-3-binding protein isoform 2** | 17.6 | 3 | 4 | 4 | 8 | 1.14E+08 | **0.81** | 3 | 0.7 | 598 | 68 | 5.5 | Signal transduction | Cytoplasm;Plasma membrane;Extracellular | Protein serine/threonine kinase activity | Cytoplasm |
| **DAK** | NP_056348.2 | 26007 | **bifunctional ATP-dependent dihydroxyacetone kinase/FAD-AMP lyase (cyclizing)** | 17.0 | 1 | 5 | 5 | 9 | 1.74E+08 | **0.81** | 6 | 26.8 | 575 | 58.9 | 7.5 | Metabolism;Energy pathways | - | Catalytic activity | - |
| **ERAL1** | NP_005693.1 | 26284 | **GTPase Era, mitochondrial** | 11.7 | 1 | 3 | 3 | 5 | 1.64E+08 | **0.81** | 2 | 12.4 | 437 | 48.3 | 8.8 | Cell communication;Signal transduction | Endoplasmic reticulum;Nucleus | GTPase activity | Endoplasmic reticulum |
| **MRPL40** | NP_003767.2 | 64976 | **39S ribosomal protein L40, mitochondrial** | 16.5 | 1 | 2 | 2 | 9 | 1.79E+08 | **0.81** | 3 | 9.5 | 206 | 24.5 | 9.6 | Protein metabolism | Ribosome;Mitochondrion | Structural constituent of ribosome | Ribosome |
| **SERPINH1** | NP_001193943.1 | 871 | **serpin H1 precursor** | 57.7 | 1 | 21 | 21 | 219 | 3.33E+09 | **0.81** | 46 | 12.3 | 418 | 46.4 | 8.7 | Protein metabolism | Endoplasmic reticulum;Plasma membrane;Nucleolus | Heat shock protein activity | Endoplasmic reticulum |
| **STK38** | NP_009202.1 | 11329 | **serine/threonine-protein kinase 38** | 6.7 | 1 | 2 | 2 | 6 | 2.09E+08 | **0.81** | 4 | 1.5 | 465 | 54.2 | 7.2 | Cell communication;Signal transduction | Nucleus | Protein serine/threonine kinase activity | Nucleus |
| **UPF1** | NP_002902.2 | 5976 | **regulator of nonsense transcripts 1** | 36.9 | 1 | 33 | 33 | 76 | 3.16E+08 | **0.81** | 31 | 17.8 | 1118 | 123 | 6.7 | Regulation of translation;RNA metabolism | Cytoplasm;Nucleus | Helicase activity | Cytoplasm;Nucleus |
| **HNRNPU** | NP_114032.2 | 3192 | **heterogeneous nuclear ribonucleoprotein U isoform a** | 52.5 | 2 | 38 | 38 | 370 | 8.93E+09 | **0.81** | 100 | 16.6 | 825 | 90.5 | 6.0 | Regulation of nucleobase, nucleoside, nucleotide and nucleic acid metabolism | Nucleus;Mitochondrion;Cytoplasm | RNA binding | Nucleus;Cytoplasm |
| **NSFL1C** | NP_057227.2 | 55968 | **NSFL1 cofactor p47 isoform a** | 35.4 | 3 | 8 | 8 | 34 | 2.69E+08 | **0.81** | 14 | 16 | 370 | 40.5 | 5.1 | Cell growth and/or maintenance | Cytoplasm;Nucleus;Golgi apparatus | Molecular function unknown | Nucleus;Cytoplasm |
| **SYMPK** | NP_004810.2 | 8189 | **symplekin** | 13.8 | 1 | 10 | 10 | 24 | 7.22E+07 | **0.81** | 12 | 28.5 | 1274 | 141.1 | 6.1 | Cell growth and/or maintenance | Plasma membrane;Cytoplasm;Nucleus | Cell adhesion molecule activity | Plasma membrane |
| **ALDH9A1** | NP_000687.3 | 223 | **4-trimethylaminobutyraldehyde dehydrogenase** | 33.0 | 1 | 12 | 12 | 23 | 2.46E+08 | **0.81** | 8 | 19.3 | 518 | 56.3 | 6.6 | Metabolism;Energy pathways | Cytoplasm | Catalytic activity | Cytoplasm |
| **MTA1** | NP_004680.2 | 9112 | **metastasis-associated protein MTA1 isoform MTA1** | 15.1 | 2 | 5 | 8 | 12 | 1.64E+08 | **0.81** | 4 | 6.1 | 715 | 80.7 | 9.3 | Regulation of nucleobase, nucleoside, nucleotide and nucleic acid metabolism | Nucleus;Cytoplasm | Transcription regulator activity | Nucleus |
| **RBM22** | NP_060517.1 | 55696 | **pre-mRNA-splicing factor RBM22** | 17.1 | 1 | 4 | 4 | 14 | 2.03E+08 | **0.81** | 5 | 21.9 | 420 | 46.9 | 8.5 | Biological_process unknown | - | Molecular function unknown | - |
| **TWF1** | NP_002813.3 | 5756 | **twinfilin-1 isoform 2** | 32.9 | 2 | 9 | 9 | 49 | 5.22E+08 | **0.81** | 14 | 16.3 | 350 | 40.3 | 7.0 | Cell communication;Signal transduction | Actin cytoskeleton;Cytoplasm | Protein-tyrosine kinase activity | Cytoplasm |
| **LHPP** | NP_071409.3 | 64077 | **phospholysine phosphohistidine inorganic pyrophosphate phosphatase isoform 1** | 18.5 | 1 | 2 | 2 | 4 | 1.97E+07 | **0.81** | 4 | 11.3 | 270 | 29.1 | 6.2 | Biological_process unknown | - | Molecular function unknown | - |
| **ANKS1A** | NP_056060.2 | 23294 | **ankyrin repeat and SAM domain-containing protein 1A** | 2.5 | 1 | 2 | 2 | 2 | 1.70E+07 | **0.81** | 1 | - | 1134 | 123 | 6.4 | Cell communication;Signal transduction | Cytoplasm | Receptor signaling complex scaffold activity | Cytoplasm |
| **HIST1H2BB** | NP_066406.1 | 3018 | **histone H2B type 1-B** | 67.5 | 6 | 2 | 15 | 322 | 1.75E+10 | **0.81** | 3 | 9.3 | 126 | 13.9 | 10.3 | Regulation of nucleobase, nucleoside, nucleotide and nucleic acid metabolism | Nucleus | DNA binding | Nucleus |
| **MRPL11** | NP_733934.1 | 65003 | **39S ribosomal protein L11, mitochondrial isoform b** | 31.3 | 3 | 5 | 5 | 8 | 1.10E+08 | **0.81** | 5 | 13.2 | 166 | 18.2 | 9.7 | Protein metabolism | Mitochondrion | Structural constituent of ribosome | Mitochondrion |
| **RPF1** | NP_079341.2 | 80135 | **ribosome production factor 1** | 19.2 | 1 | 3 | 3 | 7 | 1.17E+08 | **0.81** | 1 | - | 349 | 40.1 | 10.0 | Regulation of nucleobase, nucleoside, nucleotide and nucleic acid metabolism | Nucleolus | Molecular function unknown | Nucleolus |
| **XPNPEP1** | NP_065116.3 | 7511 | **xaa-Pro aminopeptidase 1 isoform 1** | 31.1 | 2 | 11 | 11 | 22 | 6.27E+08 | **0.81** | 11 | 15.1 | 666 | 74.8 | 5.9 | Protein metabolism | Cytoplasm | Aminopeptidase activity | Cytoplasm |
| **CBX5** | NP_001120793.1 | 23468 | **chromobox protein homolog 5** | 61.3 | 1 | 9 | 10 | 29 | 5.22E+08 | **0.81** | 12 | 17.4 | 191 | 22.2 | 5.9 | Regulation of nucleobase, nucleoside, nucleotide and nucleic acid metabolism | Nucleus;Nucleolus;Chromosome;Cytoplasm | DNA binding | Nucleus |
| **PEBP1** | NP_002558.1 | 5037 | **phosphatidylethanolamine-binding protein 1 preproprotein** | 88.8 | 1 | 17 | 17 | 184 | 1.96E+09 | **0.81** | 44 | 19.6 | 187 | 21 | 7.5 | Cell communication;Signal transduction | Cytoplasm;Plasma membrane | Protease inhibitor activity | Cytoplasm |
| **TRIAP1** | NP_057483.1 | 51499 | **TP53-regulated inhibitor of apoptosis 1** | 40.8 | 1 | 2 | 2 | 3 | 7.16E+07 | **0.81** | 2 | 63.9 | 76 | 8.8 | 5.5 | Anti-apoptosis;DNA repair | Perinuclear region;Mitochondrion | Enzyme inhibitor activity | Perinuclear region |
| **DNAJC21** | NP_001012339.2 | 134218 | **dnaJ homolog subfamily C member 21 isoform 2** | 3.0 | 2 | 1 | 1 | 2 | 5.13E+07 | **0.81** | 1 | - | 531 | 62 | 5.5 | Regulation of nucleobase, nucleoside, nucleotide and nucleic acid metabolism | - | DNA binding | - |
| **NOTCH1** | NP_060087.3 | 4851 | **neurogenic locus notch homolog protein 1 preproprotein** | 1.0 | 1 | 1 | 1 | 1 | 4.84E+06 | **0.81** | 1 | - | 2555 | 272.3 | 5.1 | Anti-apoptosis | Plasma membrane;Nucleus;Cytosol | Transcription regulator activity | Plasma membrane |

| Gene Symbol | RefSeq_Accession | Entrez Gene ID | Description | Coverage (%) | Proteins | Unique Peptides | # Peptides | PSM | Area | SCC-R /SCC-S | SCC-R /SCC-S (Count) | SCC-R /SCC-S (Variability [%]) | # AAs | MW [kDa] | calc. pI | Biological Process | Localization | Molecular Function | Primary Localization |
|---|---|---|---|---|---|---|---|---|---|---|---|---|---|---|---|---|---|---|---|
| **HEXB** | NP_001278933.1 | 3074 | **beta-hexosaminidase subunit beta isoform 2** | 26.0 | 2 | 5 | 5 | 15 | 1.65E+08 | **0.80** | 6 | 37.8 | 331 | 38.2 | 6.4 | Metabolism;Energy pathways | Lysosome;Golgi apparatus;Endoplasmic reticulum | Hydrolase activity | Lysosome |
| **SCO1** | NP_004580.1 | 6341 | **protein SCO1 homolog, mitochondrial** | 9.3 | 1 | 2 | 2 | 5 | 1.57E+08 | **0.80** | 1 | - | 301 | 33.8 | 8.9 | Metabolism;Energy pathways | Mitochondrion | Molecular function unknown | Mitochondrion |
| **DHRS7** | NP_057113.1 | 51635 | **dehydrogenase/reductase SDR family member 7 precursor** | 19.8 | 1 | 3 | 3 | 14 | 2.97E+08 | **0.80** | 4 | 9.1 | 339 | 38.3 | 8.3 | Metabolism;Energy pathways | - | Catalytic activity | - |
| **IARS2** | NP_060530.3 | 55699 | **isoleucine--tRNA ligase, mitochondrial precursor** | 33.7 | 1 | 19 | 19 | 42 | 5.87E+08 | **0.80** | 16 | 11.3 | 1012 | 113.7 | 7.2 | Metabolism;Energy pathways;Translation | Mitochondrion;Cytosol | TRNA ligase activity | Mitochondrion |
| **RAB13** | NP_002861.1 | 5872 | **ras-related protein Rab-13 isoform 1** | 21.7 | 2 | 2 | 4 | 15 | 4.34E+08 | **0.80** | 3 | 3.6 | 203 | 22.8 | 9.2 | Cell communication;Signal transduction | Cytoplasmic vesicle;Plasma membrane | GTPase activity | Cytoplasmic vesicle |
| **TPM1** | NP_001018006.1 | 7168 | **tropomyosin alpha-1 chain isoform 4** | 52.5 | 5 | 8 | 24 | 219 | 8.07E+09 | **0.80** | 7 | 15.4 | 284 | 32.9 | 4.8 | Cell growth and/or maintenance | Perinuclear region;Cytoplasm;Actin cytoskeleton | Cytoskeletal protein binding | Actin cytoskeleton |
| **UQCRC1** | NP_003356.2 | 7384 | **cytochrome b-c1 complex subunit 1, mitochondrial precursor** | 51.9 | 1 | 18 | 18 | 81 | 1.08E+09 | **0.80** | 30 | 18.5 | 480 | 52.6 | 6.4 | Metabolism;Energy pathways | Mitochondrion;Nucleus | Catalytic activity | Mitochondrion |
| **AARS** | NP_001596.2 | 16 | **alanine--tRNA ligase, cytoplasmic** | 58.4 | 1 | 42 | 42 | 222 | 1.32E+09 | **0.80** | 60 | 13.4 | 968 | 106.7 | 5.5 | Protein metabolism;Translation | Cytoplasm;Mitochondrion;Cytosol | Ligase activity | Cytoplasm |
| **CUL3** | NP_003581.1 | 8452 | **cullin-3 isoform 1** | 5.5 | 3 | 3 | 3 | 4 | 1.35E+08 | **0.80** | 1 | - | 768 | 88.9 | 8.5 | Protein metabolism | Nucleus;Golgi apparatus | Ubiquitin-specific protease activity | Nucleus |
| **GGA1** | NP_001166158.1 | 26088 | **ADP-ribosylation factor-binding protein GGA1 isoform 4** | 23.3 | 4 | 7 | 7 | 15 | 1.36E+08 | **0.80** | 7 | 20.5 | 635 | 69.8 | 5.2 | Biological_process unknown;Transport | Cytosol;Golgi membrane;Perinuclear region | Molecular function unknown;Transporter activity | Golgi membrane |
| **PSME1** | NP_006254.1 | 5720 | **proteasome activator complex subunit 1 isoform 1** | 68.7 | 4 | 18 | 18 | 180 | 1.46E+09 | **0.80** | 39 | 20.2 | 249 | 28.7 | 6.0 | Protein metabolism | Nucleus;Cytoplasm | Ubiquitin-specific protease activity | Nucleus |
| **TOMM6** | NP_001127965.1 | 100188893 | **mitochondrial import receptor subunit TOM6 homolog** | 56.8 | 1 | 2 | 2 | 4 | 1.70E+08 | **0.80** | 1 | - | 74 | 8 | 4.9 | Protein transport | Mitochondrial membrane | Protein transporter activity | Mitochondrial membrane |
| **TRRAP** | NP_003487.1 | 8295 | **transformation/transcription domain-associated protein isoform 2** | 1.6 | 2 | 4 | 4 | 5 | 2.82E+07 | **0.80** | 1 | - | 3830 | 434.1 | 8.2 | Regulation of nucleobase, nucleoside, nucleotide and nucleic acid metabolism | Nucleus;Mitochondrion;Extracellular | Transcription regulator activity | Nucleus |
| **POLR2A** | NP_000928.1 | 5430 | **DNA-directed RNA polymerase II subunit RPB1** | 9.5 | 1 | 9 | 9 | 15 | 1.20E+08 | **0.80** | 10 | 21.4 | 1970 | 217.1 | 7.4 | Regulation of nucleobase, nucleoside, nucleotide and nucleic acid metabolism | Nucleus | DNA-directed RNA polymerase II activity | Nucleus |
| **CDKN2AIP** | NP_060102.1 | 55602 | **CDKN2A-interacting protein** | 13.1 | 1 | 5 | 5 | 8 | 4.81E+07 | **0.80** | 5 | 36 | 580 | 61.1 | 9.0 | Regulation of nucleobase, nucleoside, nucleotide and nucleic acid metabolism | Nucleolus;Nucleus | RNA binding | Nucleus |
| **GATAD2A** | NP_060130.3 | 54815 | **transcriptional repressor p66-alpha** | 12.0 | 1 | 4 | 5 | 13 | 3.22E+07 | **0.80** | 2 | 29.8 | 633 | 68 | 9.9 | Regulation of nucleobase, nucleoside, nucleotide and nucleic acid metabolism | Nucleus;Cytoplasm | Transcription regulator activity | Nucleus |
| **LPP** | NP_005569.1 | 4026 | **lipoma-preferred partner isoform a** | 49.4 | 2 | 14 | 14 | 35 | 2.59E+08 | **0.80** | 6 | 26.1 | 612 | 65.7 | 7.4 | Cell growth and/or maintenance | Nucleus;Cytoplasm;Plasma membrane | Molecular function unknown | Cytoplasm |
| **RANBP10** | NP_065901.1 | 57610 | **ran-binding protein 10** | 10.0 | 1 | 4 | 4 | 6 | 2.08E+07 | **0.80** | 4 | 2.9 | 620 | 67.2 | 6.8 | Cell communication;Signal transduction | - | Molecular function unknown | - |
| **TOMM34** | NP_006800.2 | 10953 | **mitochondrial import receptor subunit TOM34** | 39.5 | 1 | 10 | 10 | 35 | 1.53E+08 | **0.80** | 11 | 14.5 | 309 | 34.5 | 9.0 | Transport | Cytoplasm;Mitochondrion | Transporter activity | Cytoplasm |
| **GLRX3** | NP_006532.2 | 10539 | **glutaredoxin-3** | 63.9 | 1 | 18 | 18 | 52 | 5.76E+08 | **0.80** | 12 | 28 | 335 | 37.4 | 5.4 | Biological_process unknown | Cytoplasm | Molecular function unknown | Cytoplasm |
| **RNASEH2A** | NP_006388.2 | 10535 | **ribonuclease H2 subunit A** | 11.0 | 1 | 2 | 2 | 4 | 4.30E+07 | **0.80** | 1 | - | 299 | 33.4 | 5.3 | Regulation of nucleobase, nucleoside, nucleotide and nucleic acid metabolism | Nucleus | Ribonuclease activity | Nucleus |
| **TIPRL** | NP_690866.1 | 261726 | **TIP41-like protein isoform 1** | 66.5 | 2 | 11 | 11 | 21 | 4.03E+08 | **0.80** | 10 | 39.9 | 272 | 31.4 | 5.9 | Biological_process unknown | - | Molecular function unknown | - |
| **CHMP1B** | NP_065145.2 | 57132 | **charged multivesicular body protein 1b** | 7.0 | 1 | 2 | 2 | 2 | 1.01E+08 | **0.80** | 1 | - | 199 | 22.1 | 8.1 | Transport | Endosome | Transporter activity | Endosome |
| **CYCS** | NP_061820.1 | 54205 | **cytochrome c** | 51.4 | 1 | 11 | 11 | 44 | 9.40E+08 | **0.80** | 17 | 13.2 | 105 | 11.7 | 9.6 | Metabolism;Energy pathways | Mitochondrion;Cytoplasm;Cytosol | Catalytic activity | Mitochondrion |
| **ERCC6L** | NP_060139.2 | 54821 | **DNA excision repair protein ERCC-6-like** | 8.4 | 1 | 5 | 5 | 7 | 1.47E+08 | **0.80** | 3 | 60.4 | 1250 | 141 | 5.3 | Biological_process unknown | - | Molecular function unknown | - |
| **MT1X** | NP_005943.1 | 4501 | **metallothionein-1X** | 67.2 | 4 | 1 | 4 | 20 | 1.15E+09 | **0.80** | 2 | 3.8 | 61 | 6.1 | 8.0 | Transport | Cytoplasm;Nucleus;Extracellular | Transporter activity | Cytoplasm |
| **NUP85** | NP_079120.1 | 79902 | **nuclear pore complex protein Nup85** | 15.1 | 1 | 5 | 5 | 6 | 4.54E+07 | **0.80** | 3 | 4.2 | 656 | 75 | 5.6 | Cell growth and/or maintenance | Centrosome | Cytoskeletal anchoring activity | Centrosome |
| **SMARCC1** | NP_003065.3 | 6599 | **SWI/SNF complex subunit SMARCC1** | 9.8 | 1 | 5 | 7 | 10 | 2.31E+08 | **0.80** | 3 | 84 | 1105 | 122.8 | 5.8 | Regulation of nucleobase, nucleoside, nucleotide and nucleic acid metabolism | Nucleus;Kinetochore;Cytoplasm | Transcription factor activity | Nucleus |
| **TUBG1** | NP_001061.2 | 7283 | **tubulin gamma-1 chain** | 28.8 | 2 | 7 | 7 | 16 | 2.11E+08 | **0.80** | 5 | 5 | 451 | 51.1 | 6.1 | Cell growth and/or maintenance | Nucleus;Nucleolus;Centrosome;Cytosol;Microtubule | Structural constituent of cytoskeleton | Nucleus |
| **ATP5J** | NP_001003696.1 | 522 | **ATP synthase-coupling factor 6, mitochondrial isoform a precursor** | 50.9 | 2 | 5 | 5 | 8 | 2.16E+08 | **0.80** | 3 | 7.5 | 108 | 12.6 | 9.5 | Metabolism;Energy pathways | Mitochondrion;Extracellular | ATPase activity;Protein binding | Mitochondrion |
| **CDC42EP1** | NP_689449.1 | 11135 | **cdc42 effector protein 1** | 16.4 | 1 | 3 | 3 | 4 | 8.19E+07 | **0.80** | 3 | 2.5 | 391 | 40.3 | 7.2 | Cell growth and/or maintenance | Extracellular;Plasma membrane;Cytoplasm;Nucleus | Structural constituent of cytoskeleton | Extracellular |
| **MAVS** | NP_065797.2 | 57506 | **mitochondrial antiviral-signaling protein isoform 1** | 36.7 | 2 | 8 | 8 | 14 | 1.71E+08 | **0.80** | 6 | 7.6 | 540 | 56.5 | 5.5 | Biological_process unknown | - | Molecular function unknown | - |
| **PDCD10** | NP_665859.1 | 11235 | **programmed cell death protein 10** | 22.6 | 1 | 4 | 4 | 7 | 1.77E+08 | **0.80** | 3 | 27.1 | 212 | 24.7 | 8.2 | Apoptosis | - | Molecular function unknown | - |
| **RAB9A** | NP_001182257.1 | 9367 | **ras-related protein Rab-9A** | 5.5 | 2 | 1 | 1 | 1 | 8.78E+07 | **0.80** | 1 | - | 201 | 22.8 | 5.5 | Cell communication;Signal transduction | Endosome;Golgi apparatus;Cytoplasm;Nucleus | Molecular function unknown | Endosome |
| **EXOC6** | NP_061926.3 | 54536 | **exocyst complex component 6 isoform a** | 1.9 | 1 | 1 | 1 | 1 | 8.01E+07 | **0.80** | 1 | - | 804 | 93.7 | 6.2 | Transport | Cytoplasm | Auxiliary transport protein activity | Cytoplasm |
| **EXOSC3** | NP_001002269.1 | 51010 | **exosome complex component RRP40 isoform 2** | 31.1 | 2 | 3 | 3 | 5 | 1.69E+08 | **0.80** | 3 | 4.7 | 164 | 17.2 | 9.3 | Regulation of nucleobase, nucleoside, nucleotide and nucleic acid metabolism | Nucleolus;Nucleus;Cytoplasm | Ribonuclease activity | Nucleolus |
| **NOC2L** | NP_056473.2 | 26155 | **nucleolar complex protein 2 homolog** | 10.3 | 1 | 5 | 5 | 9 | 7.89E+07 | **0.80** | 5 | 15.1 | 749 | 84.9 | 5.6 | Biological_process unknown | Nucleolus;Nucleus;Cytoplasm | Molecular function unknown | Nucleolus |

| Gene Symbol | RefSeq_Accession | Entrez Gene ID | Description | Coverage (%) | Proteins | Unique Peptides | # Peptides | PSM | Area | SCC-R /SCC-S | SCC-R /SCC-S (Count) | SCC-R /SCC-S (Variability [%]) | # AAs | MW [kDa] | calc. pI | Biological Process | Localization | Molecular Function | Primary Localization |
|---|---|---|---|---|---|---|---|---|---|---|---|---|---|---|---|---|---|---|---|
| OSBPL8 | NP_001003712.1 | 114882 | oxysterol-binding protein-related protein 8 isoform b | 4.1 | 4 | 2 | 2 | 3 | 1.58E+08 | 0.80 | 1 | - | 847 | 96.9 | 7.6 | Transport | Mitochondrion | Transporter activity | Mitochondrion |
| PKNOX1 | NP_001273187.1 | 5316 | homeobox protein PKNOX1 isoform 2 | 4.7 | 2 | 1 | 1 | 1 | 2.66E+07 | 0.80 | 1 | - | 319 | 34.5 | 6.3 | Transcription | Nucleus;Cytoplasm | DNA binding | Nucleus |
| DDX18 | NP_006764.3 | 8886 | ATP-dependent RNA helicase DDX18 | 44.2 | 1 | 17 | 17 | 49 | 2.35E+08 | 0.79 | 17 | 22.2 | 670 | 75.4 | 9.5 | Transport | Nucleus;Nucleolus | Transporter activity | Nucleus |
| EIF3D | NP_003744.1 | 8664 | eukaryotic translation initiation factor 3 subunit D | 53.5 | 1 | 19 | 19 | 89 | 1.05E+09 | 0.79 | 33 | 15.4 | 548 | 63.9 | 6.1 | Protein metabolism | Cytoplasm | Translation regulator activity | Cytoplasm |
| EMC8 | NP_006058.1 | 10328 | ER membrane protein complex subunit 8 isoform 1 | 14.3 | 2 | 2 | 2 | 6 | 8.89E+07 | 0.79 | 1 | - | 210 | 23.8 | 6.4 | Biological_process unknown | Nucleus;Cytoplasm;Mitochondrion | Molecular function unknown | Nucleus |
| GCDH | NP_000150.1 | 2639 | glutaryl-CoA dehydrogenase, mitochondrial isoform a precursor | 27.2 | 2 | 6 | 6 | 9 | 3.06E+08 | 0.79 | 6 | 10.7 | 438 | 48.1 | 8.1 | Metabolism;Energy pathways | Mitochondrion | Catalytic activity | Mitochondrion |
| MRPS36 | NP_150597.1 | 92259 | 28S ribosomal protein S36, mitochondrial | 45.6 | 1 | 3 | 3 | 10 | 9.44E+07 | 0.79 | 4 | 27.6 | 103 | 11.5 | 10.0 | Protein metabolism | Mitochondrion | Structural constituent of ribosome | Mitochondrion |
| NGDN | NP_056329.1 | 25983 | neuroguidin isoform 2 | 14.8 | 2 | 3 | 3 | 10 | 6.48E+07 | 0.79 | 5 | 7.4 | 311 | 35.2 | 9.3 | Biological_process unknown | Nucleolus;Nucleus | Molecular function unknown | Nucleolus;Nucleus |
| HDHD3 | NP_112496.1 | 81932 | haloacid dehalogenase-like hydrolase domain-containing protein 3 | 8.0 | 1 | 1 | 1 | 1 | 8.60E+07 | 0.79 | 1 | - | 251 | 28 | 6.7 | Metabolism;Energy pathways | - | Hydrolase activity | - |
| MYLK2 | NP_149109.1 | 85366 | myosin light chain kinase 2, skeletal/cardiac muscle | 1.3 | 3 | 1 | 1 | 3 | 1.43E+08 | 0.79 | 2 | 7.2 | 596 | 64.6 | 7.0 | Cell communication;Signal transduction | - | Protein serine/threonine kinase activity | - |
| NOC3L | NP_071896.8 | 64318 | nucleolar complex protein 3 homolog | 9.1 | 1 | 5 | 5 | 6 | 5.09E+07 | 0.79 | 3 | 10.1 | 800 | 92.5 | 9.2 | Biological_process unknown | - | Molecular function unknown | - |
| NXN | NP_071908.2 | 64359 | nucleoredoxin isoform 1 | 39.8 | 2 | 10 | 10 | 31 | 2.23E+08 | 0.79 | 12 | 24.6 | 435 | 48.4 | 5.0 | Metabolism;Energy pathways | Nucleus;Cytoplasm | Oxidoreductase activity | Nucleus |
| PAK1 | NP_002567.3 | 5058 | serine/threonine-protein kinase PAK 1 isoform 2 | 19.3 | 2 | 4 | 9 | 22 | 2.66E+08 | 0.79 | 5 | 23.9 | 545 | 60.6 | 5.8 | Cell communication;Signal transduction | Plasma membrane;Cytoplasm;Cell projection | Protein serine/threonine kinase activity | Plasma membrane;Cytoplasm |
| RNF123 | NP_071347.2 | 63891 | E3 ubiquitin-protein ligase RNF123 | 0.5 | 1 | 1 | 1 | 2 | 2.49E+08 | 0.79 | 1 | - | 1314 | 148.4 | 6.7 | Protein metabolism | Cytoplasm | Ubiquitin-specific protease activity | Cytoplasm |
| RPLP0 | NP_000993.1 | 6175 | 60S acidic ribosomal protein P0 | 67.2 | 1 | 20 | 20 | 177 | 2.80E+09 | 0.79 | 42 | 21.5 | 317 | 34.3 | 6.0 | Protein metabolism | Nucleus;Ribosome;Nucleolus;Cytoplasm;Cytosol;Mitochondrion | Structural constituent of ribosome | Ribosome |
| UFD1L | NP_005650.2 | 7353 | ubiquitin fusion degradation protein 1 homolog isoform A | 33.6 | 2 | 6 | 6 | 21 | 2.19E+08 | 0.79 | 5 | 75.4 | 307 | 34.5 | 6.7 | Protein metabolism | Nucleus;Cytoplasm | Ubiquitin-specific protease activity | Nucleus |
| ATP1A1 | NP_000692.2 | 476 | sodium/potassium-transporting ATPase subunit alpha-1 isoform a | 36.7 | 11 | 26 | 26 | 117 | 9.64E+08 | 0.79 | 31 | 15.1 | 1023 | 112.8 | 5.5 | Transport | Plasma membrane;Mitochondrion | ATPase activity | Plasma membrane |
| NOMO2 | NP_775885.1 | 283820 | nodal modulator 2 isoform 2 precursor | 20.7 | 6 | 15 | 15 | 28 | 3.92E+08 | 0.79 | 11 | 22.1 | 1222 | 134.1 | 5.8 | Biological_process unknown | - | Molecular function unknown | - |
| PRPF4B | NP_003904.3 | 8899 | serine/threonine-protein kinase PRP4 homolog | 8.7 | 1 | 9 | 9 | 12 | 5.86E+07 | 0.79 | 6 | 27.1 | 1007 | 116.9 | 10.3 | Regulation of nucleobase, nucleoside, nucleotide and nucleic acid metabolism;Signal transduction | Nucleus;Cytoplasm | Transcription regulator activity;Protein serine/threonine kinase activity | Nucleus |
| PSME2 | NP_002809.2 | 5721 | proteasome activator complex subunit 2 | 65.7 | 1 | 13 | 13 | 137 | 1.29E+09 | 0.79 | 39 | 18.7 | 239 | 27.4 | 5.7 | Protein metabolism | Cytoplasm;Nucleus;Endoplasmic reticulum;Nucleolus | Ubiquitin-specific protease activity | Cytoplasm |
| RRM2 | NP_001025.1 | 6241 | ribonucleoside-diphosphate reductase subunit M2 isoform 2 | 32.7 | 5 | 10 | 10 | 18 | 1.84E+08 | 0.79 | 10 | 19 | 389 | 44.8 | 5.4 | Regulation of nucleobase, nucleoside, nucleotide and nucleic acid metabolism | Cytoplasm;Nucleus | Oxidoreductase activity | Nucleus |
| RRP9 | NP_004695.1 | 9136 | U3 small nucleolar RNA-interacting protein 2 | 16.8 | 1 | 4 | 4 | 6 | 1.34E+08 | 0.79 | 2 | 21.6 | 475 | 51.8 | 7.9 | Regulation of nucleobase, nucleoside, nucleotide and nucleic acid metabolism | Nucleolus;Nucleus;Cytoplasm | Ribonucleoprotein | Nucleolus |
| SCYL1 | NP_001041683.1 | 57410 | N-terminal kinase-like protein isoform B | 26.7 | 2 | 14 | 14 | 27 | 1.93E+08 | 0.79 | 15 | 41.7 | 791 | 88 | 6.3 | Cell communication;Signal transduction;Regulation of nucleobase, nucleoside, nucleotide and nucleic acid metabolism | Centrosome;Cytoplasm | Transcription regulator activity | Centrosome |
| TEAD4 | NP_958851.1 | 7004 | transcriptional enhancer factor TEF-3 isoform 3 | 3.9 | 5 | 1 | 1 | 1 | 2.73E+07 | 0.79 | 1 | - | 305 | 34.1 | 6.5 | Regulation of nucleobase, nucleoside, nucleotide and nucleic acid metabolism | Nucleus | Transcription factor activity | Nucleus |
| ACTR1B | NP_005726.1 | 10120 | beta-centractin | 20.0 | 1 | 1 | 4 | 6 | 1.46E+08 | 0.79 | 1 | - | 376 | 42.3 | 6.4 | Cell growth and/or maintenance | Cytoplasm;Centrosome;Kinetochore;Microtubule | Structural constituent of cytoskeleton | Cytoplasm |
| GADD45GIP1 | NP_443082.2 | 90480 | growth arrest and DNA damage-inducible proteins-interacting protein 1 | 38.3 | 1 | 6 | 6 | 8 | 8.11E+07 | 0.79 | 2 | 32.4 | 222 | 25.4 | 10.0 | Cell communication;Signal transduction | Nucleus;Mitochondrion | Protein binding | Nucleus |
| PRPF31 | NP_056444.3 | 26121 | U4/U6 small nuclear ribonucleoprotein Prp31 | 23.3 | 1 | 6 | 6 | 7 | 7.89E+07 | 0.79 | 5 | 35 | 499 | 55.4 | 5.8 | Regulation of nucleobase, nucleoside, nucleotide and nucleic acid metabolism | Nucleus | RNA binding | Nucleus |
| TMEM70 | NP_060336.3 | 54968 | transmembrane protein 70, mitochondrial isoform a | 3.5 | 1 | 1 | 1 | 1 | 1.40E+08 | 0.79 | 1 | - | 260 | 29 | 8.9 | Biological_process unknown | Mitochondrion | Molecular function unknown | Mitochondrion |
| XAB2 | NP_064581.2 | 56949 | pre-mRNA-splicing factor SYF1 | 12.8 | 1 | 6 | 6 | 9 | 9.95E+07 | 0.79 | 5 | 23.7 | 855 | 99.9 | 6.2 | Regulation of nucleobase, nucleoside, nucleotide and nucleic acid metabolism | Nucleus | DNA repair protein | Nucleus |
| COX4I1 | NP_001852.1 | 1327 | cytochrome c oxidase subunit 4 isoform 1, mitochondrial precursor | 37.3 | 1 | 7 | 7 | 18 | 1.05E+09 | 0.79 | 7 | 25 | 169 | 19.6 | 9.5 | Metabolism;Energy pathways | Nucleus;Cytoplasm;Mitochondrion | Oxidoreductase activity | Mitochondrion |
| CSE1L | NP_001307.2 | 1434 | exportin-2 isoform 1 | 43.6 | 2 | 33 | 33 | 179 | 1.36E+09 | 0.79 | 51 | 15.1 | 971 | 110.3 | 5.8 | Transport | Nucleus;Cytoplasm;Extracellular;Mitochondrion | Transporter activity | Nucleus |
| ENAH | NP_060682.2 | 55740 | protein enabled homolog isoform b | 24.0 | 2 | 8 | 8 | 16 | 9.55E+07 | 0.79 | 7 | 54.1 | 570 | 63.9 | 6.4 | Cell growth and/or maintenance | Cytoplasm;Plasma membrane;Nucleus | Structural constituent of cytoskeleton | Cytoplasm;Nucleus |
| ENSA | NP_996927.1 | 2029 | alpha-endosulfine isoform 4 | 66.7 | 9 | 6 | 6 | 14 | 2.25E+08 | 0.79 | 6 | 26.1 | 117 | 13 | 8.3 | Transport | Nucleus | Receptor binding | Nucleus |
| FNDC3A | NP_055738.3 | 22862 | fibronectin type-III domain-containing protein 3A isoform 2 | 1.8 | 2 | 1 | 1 | 1 | 1.32E+07 | 0.79 | 1 | - | 1142 | 125.7 | 7.1 | Cell growth and/or maintenance | Integral to membrane;Nucleus;Cytoplasm | Molecular function unknown | Integral to membrane |
| MRPL24 | NP_078816.2 | 79590 | 39S ribosomal protein L24, mitochondrial | 29.6 | 1 | 5 | 5 | 10 | 1.90E+08 | 0.79 | 5 | 25.8 | 216 | 24.9 | 9.3 | Protein metabolism | Mitochondrion | Structural constituent of ribosome | Mitochondrion |
| SPTLC1 | NP_006406.1 | 10558 | serine palmitoyltransferase 1 isoform a | 25.4 | 3 | 8 | 8 | 14 | 1.57E+08 | 0.79 | 8 | 14.7 | 473 | 52.7 | 6.0 | Metabolism;Energy pathways | Endoplasmic reticulum | Palmitoyltransferase activity | Endoplasmic reticulum |
| CDK13 | NP_112557.2 | 8621 | cyclin-dependent kinase 13 isoform 2 | 3.9 | 2 | 2 | 3 | 7 | 4.12E+07 | 0.79 | 4 | 37.1 | 1452 | 158.3 | 9.7 | Cell communication;Signal transduction | Nucleus;Nucleolus;Cytoplasm | Protein serine/threonine kinase activity | Nucleus;Cytoplasm |

| Gene Symbol | RefSeq_Accession | Entrez Gene ID | Description | Coverage (%) | Proteins | Unique Peptides | # Peptides | PSM | Area | SCC-R /SCC-S | SCC-R /SCC-S (Count) | SCC-R /SCC-S (Variability [%]) | # AAs | MW [kDa] | calc. pI | Biological Process | Localization | Molecular Function | Primary Localization |
|---|---|---|---|---|---|---|---|---|---|---|---|---|---|---|---|---|---|---|---|
| **CMBL** | NP_620164.1 | 134147 | **carboxymethylenebutenolidase homolog** | 6.5 | 1 | 1 | 1 | 2 | 4.70E+07 | **0.79** | 1 | - | 245 | 28 | 7.2 | Biological_process unknown | - | Molecular function unknown | - |
| **HMBS** | NP_001245138.1 | 3145 | **porphobilinogen deaminase isoform 4** | 13.2 | 4 | 2 | 2 | 8 | 1.01E+08 | **0.79** | 4 | 28.5 | 304 | 33.2 | 8.0 | Metabolism;Energy pathways | Cytoplasm;Nucleus | Transferase activity | Cytoplasm |
| **SLC25A29** | NP_001034444.1 | 123096 | **mitochondrial carnitine/acylcarnitine carrier protein CACL isoform 1** | 8.3 | 1 | 1 | 1 | 2 | 1.30E+07 | **0.79** | 1 | - | 303 | 32 | 8.8 | Transport | Mitochondrion | Transporter activity | Mitochondrion |
| **CASK** | NP_001119527.1 | 8573 | **peripheral plasma membrane protein CASK isoform 3** | 3.9 | 3 | 2 | 2 | 2 | 3.04E+07 | **0.79** | 1 | - | 897 | 102 | 6.4 | Cell communication;Signal transduction | Plasma membrane;Nucleus;Cytoplasm | Protein serine/threonine kinase activity | Plasma membrane |
| **HSPA1A** | NP_005336.3 | 3303 | **heat shock 70 kDa protein 1A/1B** | 68.6 | 1 | 28 | 43 | 522 | 1.18E+10 | **0.79** | 69 | 18.8 | 641 | 70 | 5.7 | Protein metabolism | Cytoplasm;Nucleolus;Cytosol;Nucleus | Chaperone activity | Cytoplasm |
| **PEX3** | NP_003621.1 | 8504 | **peroxisomal biogenesis factor 3** | 9.4 | 1 | 2 | 2 | 8 | 2.09E+07 | **0.79** | 4 | 7.2 | 373 | 42.1 | 8.2 | Cell communication;Signal transduction | Peroxisome;Mitochondrion | Protein binding | Peroxisome |
| **RPL7A** | NP_000963.1 | 6130 | **60S ribosomal protein L7a** | 53.0 | 1 | 20 | 20 | 197 | 4.03E+09 | **0.79** | 42 | 22.9 | 266 | 30 | 10.6 | Protein metabolism | Nucleus;Nucleolus;Cytoplasm;Extracellular;Ribosome | Structural constituent of ribosome | Cytoplasm |
| **WASH1** | NP_878908.4 | 100287171 | **WAS protein family homolog 1** | 5.6 | 1 | 1 | 2 | 3 | 1.10E+08 | **0.79** | 1 | - | 465 | 50.3 | 5.7 | - | - | - | - |
| **C17orf85** | NP_001107590.1 | 55421 | **uncharacterized protein C17orf85** | 9.5 | 1 | 5 | 5 | 7 | 5.15E+07 | **0.79** | 4 | 10.4 | 620 | 70.5 | 5.7 | Biological_process unknown | Nucleus;Cytoplasm | Molecular function unknown | Nucleus;Cytoplasm |
| **HEATR6** | NP_071353.4 | 63897 | **HEAT repeat-containing protein 6** | 12.9 | 1 | 7 | 7 | 9 | 3.51E+07 | **0.79** | 5 | 1.2 | 1181 | 128.7 | 7.0 | Biological_process unknown | - | Molecular function unknown | - |
| **INTS9** | NP_001166033.1 | 55756 | **integrator complex subunit 9 isoform 3** | 5.7 | 3 | 2 | 2 | 5 | 2.14E+07 | **0.79** | 3 | 0.9 | 634 | 71.1 | 6.0 | Biological_process unknown | Nucleus | Molecular function unknown | Nucleus |
| **RPS13** | NP_001008.1 | 6207 | **40S ribosomal protein S13** | 45.0 | 1 | 11 | 11 | 115 | 2.62E+09 | **0.79** | 41 | 22.3 | 151 | 17.2 | 10.5 | Protein metabolism | Ribosome;Nucleolus | Structural constituent of ribosome | Ribosome |
| **COMMD9** | NP_001095123.1 | 29099 | **COMM domain-containing protein 9 isoform 2** | 46.2 | 2 | 3 | 3 | 14 | 5.07E+07 | **0.79** | 4 | 17.9 | 156 | 17.3 | 6.9 | Biological_process unknown | - | Molecular function unknown | - |
| **HADH** | NP_005318.3 | 3033 | **hydroxyacyl-coenzyme A dehydrogenase, mitochondrial isoform 2 precursor** | 58.3 | 2 | 11 | 11 | 22 | 2.30E+08 | **0.79** | 12 | 15.9 | 314 | 34.3 | 8.9 | Biological_process unknown | Mitochondrion;Endoplasmic reticulum;Nucleolus | Molecular function unknown | Mitochondrion |
| **RPL6** | NP_000961.2 | 6128 | **60S ribosomal protein L6** | 50.4 | 1 | 17 | 17 | 352 | 3.29E+09 | **0.79** | 69 | 12.4 | 288 | 32.7 | 10.6 | Protein metabolism | Nucleolus;Mitochondrion;Ribosome | Structural constituent of ribosome | Nucleolus |
| **RPS9** | NP_001004.2 | 6203 | **40S ribosomal protein S9** | 44.3 | 1 | 12 | 12 | 49 | 1.12E+09 | **0.79** | 20 | 13 | 194 | 22.6 | 10.7 | Protein metabolism | Ribosome;Nucleolus | Structural constituent of ribosome | Ribosome |
| **SEC23IP** | NP_009121.1 | 11196 | **SEC23-interacting protein** | 23.6 | 1 | 12 | 12 | 21 | 5.85E+08 | **0.79** | 9 | 14.2 | 1000 | 111 | 5.5 | Transport | Endoplasmic reticulum;Cytoplasm;Nucleus;Golgi apparatus | Transporter activity | Endoplasmic reticulum |
| **SENP1** | NP_001254523.1 | 29843 | **sentrin-specific protease 1** | 2.8 | 1 | 1 | 1 | 1 | 2.92E+07 | **0.79** | 1 | - | 644 | 73.4 | 8.5 | Protein metabolism | Nucleus | Peptidase activity | Nucleus |
| **OXSM** | NP_001138863.1 | 54995 | **3-oxoacyl-[acyl-carrier-protein] synthase, mitochondrial isoform 2 precursor** | 18.9 | 2 | 3 | 3 | 4 | 6.49E+07 | **0.78** | 1 | - | 376 | 40 | 7.9 | Biological_process unknown | - | Molecular function unknown | - |
| **FAM96B** | NP_057146.1 | 51647 | **mitotic spindle-associated MMXD complex subunit MIP18** | 30.7 | 1 | 3 | 3 | 6 | 3.00E+08 | **0.78** | 3 | 0.1 | 163 | 17.7 | 5.2 | Biological_process unknown | - | Molecular function unknown | - |
| **ITGAV** | NP_001138471.1 | 3685 | **integrin alpha-V isoform 2 precursor** | 11.8 | 2 | 1 | 5 | 10 | 4.81E+07 | **0.78** | 1 | - | 1002 | 111.1 | 5.6 | Cell communication;Signal transduction;Cell adhesion | Plasma membrane;Cell surface;Nucleus;Cytoplasm | Receptor activity | Plasma membrane |
| **RBM15** | NP_001188474.1 | 64783 | **putative RNA-binding protein 15 isoform 2** | 8.7 | 2 | 5 | 5 | 7 | 2.84E+07 | **0.78** | 2 | 17 | 969 | 106.3 | 10.1 | Regulation of nucleobase, nucleoside, nucleotide and nucleic acid metabolism | - | RNA binding | - |
| **UQCR11** | NP_006821.1 | 10975 | **cytochrome b-c1 complex subunit 10** | 21.4 | 1 | 1 | 1 | 1 | 9.27E+06 | **0.78** | 1 | - | 56 | 6.6 | 9.9 | Metabolism;Energy pathways | Mitochondrion | Catalytic activity | Mitochondrion |
| **RBM39** | NP_001229529.1 | 9584 | **RNA-binding protein 39 isoform d** | 42.4 | 7 | 14 | 14 | 34 | 9.26E+08 | **0.78** | 10 | 9.6 | 502 | 56.3 | 10.0 | Regulation of nucleobase, nucleoside, nucleotide and nucleic acid metabolism | Nucleolus;Nucleus | RNA binding;Transcription regulator activity | Nucleus |
| **RPA3** | NP_002938.1 | 6119 | **replication protein A 14 kDa subunit** | 55.4 | 1 | 4 | 4 | 23 | 3.86E+08 | **0.78** | 4 | 10.9 | 121 | 13.6 | 5.1 | Regulation of nucleobase, nucleoside, nucleotide and nucleic acid metabolism | Nucleolus;Cytoplasm | DNA binding | Nucleolus |
| **BPHL** | NP_004323.2 | 670 | **valacyclovir hydrolase precursor** | 24.4 | 1 | 3 | 3 | 4 | 6.92E+07 | **0.78** | 3 | 67.3 | 291 | 32.5 | 9.1 | Metabolism;Energy pathways | - | Hydrolase activity | - |
| **DNTTIP2** | NP_055412.2 | 30836 | **deoxynucleotidyltransferase terminal-interacting protein 2** | 20.4 | 1 | 9 | 9 | 17 | 1.41E+08 | **0.78** | 8 | 8.1 | 756 | 84.4 | 6.2 | Regulation of nucleobase, nucleoside, nucleotide and nucleic acid metabolism | Nucleus;Cytoplasm | DNA binding | Nucleus |
| **MAK16** | NP_115898.2 | 84549 | **protein MAK16 homolog** | 10.7 | 1 | 2 | 2 | 6 | 1.41E+08 | **0.78** | 1 | - | 300 | 35.3 | 5.4 | Regulation of nucleobase, nucleoside, nucleotide and nucleic acid metabolism | Nucleolus;Nucleus;Cytoplasm | RNA binding | Nucleolus |
| **EML3** | NP_694997.2 | 256364 | **echinoderm microtubule-associated protein-like 3** | 1.8 | 1 | 1 | 1 | 2 | 8.41E+06 | **0.78** | 1 | - | 896 | 95.1 | 7.1 | Cytoskeleton organization and biogenesis | - | Cytoskeletal protein binding | - |
| **ETF1** | NP_004721.1 | 2107 | **eukaryotic peptide chain release factor subunit 1 isoform 1** | 38.0 | 3 | 13 | 13 | 35 | 3.85E+08 | **0.78** | 11 | 13.1 | 437 | 49 | 5.7 | Protein metabolism | Ribosome;Cytoplasm;Nucleolus | Translation regulator activity | Ribosome |
| **KIAA1279** | NP_056449.1 | 26128 | **KIF1-binding protein** | 17.7 | 1 | 5 | 6 | 14 | 7.91E+08 | **0.78** | 2 | 61.9 | 621 | 71.8 | 5.5 | Biological_process unknown | - | Molecular function unknown | - |
| **MCM2** | NP_004517.2 | 4171 | **DNA replication licensing factor MCM2** | 39.6 | 1 | 28 | 28 | 79 | 1.07E+09 | **0.78** | 28 | 30.2 | 904 | 101.8 | 5.5 | Regulation of nucleobase, nucleoside, nucleotide and nucleic acid metabolism | Nucleus;Nucleolus;Chromosome | DNA binding | Nucleus |
| **CDK2AP2** | NP_001258778.1 | 10263 | **cyclin-dependent kinase 2-associated protein 2 isoform 2** | 11.3 | 4 | 1 | 1 | 1 | 2.87E+07 | **0.78** | 1 | - | 71 | 7.9 | 9.3 | Regulation of nucleobase, nucleoside, nucleotide and nucleic acid metabolism | - | Molecular function unknown | - |
| **SUGP2** | NP_001017392.2 | 10147 | **SURP and G-patch domain-containing protein 2** | 5.6 | 1 | 3 | 3 | 4 | 3.12E+07 | **0.78** | 1 | - | 1082 | 120.1 | 7.3 | Protein metabolism | Nucleus | RNA binding | Nucleus |
| **C17orf59** | NP_060092.2 | 54785 | **uncharacterized protein C17orf59** | 27.2 | 1 | 5 | 5 | 7 | 2.22E+07 | **0.78** | 6 | 23.8 | 357 | 37.2 | 5.8 | Biological_process unknown | - | Molecular function unknown | - |
| **PRPS2** | NP_002756.1 | 5634 | **ribose-phosphate pyrophosphokinase 2 isoform 2** | 33.7 | 2 | 4 | 7 | 13 | 1.23E+08 | **0.78** | 3 | 15.1 | 318 | 34.7 | 6.6 | Metabolism;Energy pathways | Cytoplasm | Ligase activity | Cytoplasm |
| **RBM27** | NP_061862.1 | 54439 | **RNA-binding protein 27** | 6.9 | 1 | 3 | 4 | 12 | 1.64E+08 | **0.78** | 3 | 1.1 | 1060 | 118.6 | 9.2 | Transcription | - | RNA binding | - |

| Gene Symbol | RefSeq_Accession | Entrez Gene ID | Description | Coverage (%) | Proteins | Unique Peptides | # Peptides | PSM | Area | SCC-R /SCC-S | SCC-R /SCC-S (Count) | SCC-R /SCC-S (Variability [%]) | # AAs | MW [kDa] | calc. pI | Biological Process | Localization | Molecular Function | Primary Localization |
|---|---|---|---|---|---|---|---|---|---|---|---|---|---|---|---|---|---|---|---|
| SART3 | NP_055521.1 | 9733 | squamous cell carcinoma antigen recognized by T-cells 3 | 29.6 | 1 | 19 | 19 | 57 | 3.03E+08 | 0.78 | 24 | 24.5 | 963 | 109.9 | 5.6 | Regulation of nucleobase, nucleoside, nucleotide and nucleic acid metabolism | Nucleus;Cytoplasm | RNA binding | Nucleus |
| SBF1 | NP_002963.2 | 6305 | myotubularin-related protein 5 | 2.5 | 1 | 3 | 3 | 5 | 1.46E+07 | 0.78 | 1 | - | 1893 | 210.9 | 7.1 | Cell communication;Signal transduction | Cytoplasm;Nucleus | Molecular function unknown | Cytoplasm |
| SERPINB6 | NP_004559.4 | 5269 | serpin B6 isoform a | 61.7 | 6 | 18 | 18 | 70 | 4.59E+08 | 0.78 | 19 | 21.9 | 376 | 42.6 | 5.3 | Protein metabolism | Cytoplasm | Protease inhibitor activity | Cytoplasm |
| SLC44A2 | NP_001138528.1 | 57153 | choline transporter-like protein 2 isoform 2 | 1.7 | 2 | 1 | 1 | 1 | 3.50E+07 | 0.78 | 1 | - | 704 | 79.8 | 8.5 | Cell communication;Signal transduction | Integral to membrane | Signal transducer activity | Integral to membrane |
| ALCAM | NP_001230210.1 | 214 | CD166 antigen isoform 3 precursor | 42.2 | 4 | 15 | 15 | 55 | 2.31E+08 | 0.78 | 16 | 24 | 555 | 61.9 | 6.2 | Immune response | Plasma membrane;Cytoplasm | Cell adhesion molecule activity | Plasma membrane |
| COG4 | NP_001182068.1 | 25839 | conserved oligomeric Golgi complex subunit 4 isoform 2 | 7.8 | 2 | 4 | 4 | 8 | 4.99E+07 | 0.78 | 4 | 24.7 | 768 | 87.1 | 5.2 | Transport | Golgi apparatus;Cytoplasm | Transporter activity | Golgi apparatus |
| GIT1 | NP_054749.2 | 28964 | ARF GTPase-activating protein GIT1 isoform 2 | 8.0 | 2 | 3 | 3 | 3 | 3.28E+07 | 0.78 | 2 | 27.8 | 761 | 84.3 | 6.8 | Cell communication;Signal transduction | Cytoplasm | GTPase activator activity | Cytoplasm |
| PRDX6 | NP_004896.1 | 9588 | peroxiredoxin-6 | 71.9 | 1 | 21 | 21 | 225 | 3.71E+09 | 0.78 | 49 | 15.6 | 224 | 25 | 6.4 | Metabolism;Energy pathways | Cytoplasm;Lysosome | Peroxidase activity | Lysosome |
| PRIM2 | NP_000938.2 | 5558 | DNA primase large subunit isoform a | 3.0 | 1 | 1 | 1 | 1 | 4.14E+07 | 0.78 | 1 | - | 509 | 58.8 | 7.9 | Regulation of nucleobase, nucleoside, nucleotide and nucleic acid metabolism | Nucleus | DNA-directed RNA polymerase activity | Nucleus |
| TMPO | NP_003267.1 | 7112 | thymopoietin isoform alpha | 55.8 | 1 | 13 | 24 | 93 | 1.14E+09 | 0.78 | 8 | 10.6 | 694 | 75.4 | 7.7 | Nuclear organization and biogenesis;Regulation of cell cycle | Nucleus;Nuclear membrane;Mitochondrion;Cytoplasm | Molecular function unknown | Nucleus;Nuclear membrane;Mitochondrion;Cytoplasm |
| CDK9 | NP_001252.1 | 1025 | cyclin-dependent kinase 9 | 18.8 | 1 | 5 | 5 | 7 | 6.17E+07 | 0.78 | 3 | 13.9 | 372 | 42.8 | 8.8 | Regulation of nucleobase, nucleoside, nucleotide and nucleic acid metabolism | Nucleus;Cytoplasm;Nucleolus | Protein serine/threonine kinase activity | Nucleus;Cytoplasm |
| RFC4 | NP_853551.1 | 5984 | replication factor C subunit 4 | 40.2 | 1 | 11 | 11 | 29 | 4.00E+08 | 0.78 | 9 | 6.9 | 363 | 39.7 | 8.0 | Regulation of nucleobase, nucleoside, nucleotide and nucleic acid metabolism | Nucleus;Nucleolus | DNA binding | Nucleus |
| RPS7 | NP_001002.1 | 6201 | 40S ribosomal protein S7 | 62.9 | 1 | 15 | 15 | 186 | 4.02E+09 | 0.78 | 48 | 16 | 194 | 22.1 | 10.1 | Protein metabolism | Ribosome | Structural constituent of ribosome | Ribosome |
| RSL24D1 | NP_057388.1 | 51187 | probable ribosome biogenesis protein RLP24 | 16.6 | 1 | 2 | 2 | 3 | 5.38E+07 | 0.78 | 1 | - | 163 | 19.6 | 10.0 | Biological_process unknown | Nucleus;Cytoplasm;Ribosome;Nucleolus | Molecular function unknown | Ribosome |
| CLPTM1L | NP_110409.2 | 81037 | cleft lip and palate transmembrane protein 1-like protein | 5.0 | 1 | 2 | 2 | 6 | 7.38E+07 | 0.78 | 3 | 17.1 | 538 | 62.2 | 8.6 | Biological_process unknown | Integral to membrane;Mitochondrion | Molecular function unknown | Integral to membrane |
| DDX55 | NP_065987.1 | 57696 | ATP-dependent RNA helicase DDX55 | 2.8 | 1 | 1 | 1 | 2 | 6.83E+06 | 0.78 | 1 | - | 600 | 68.5 | 9.3 | Regulation of nucleobase, nucleoside, nucleotide and nucleic acid metabolism | Nucleus;Nucleolus | RNA binding;Molecular function unknown | Nucleus |
| PITRM1 | NP_055704.2 | 10531 | presequence protease, mitochondrial isoform 2 precursor | 15.0 | 3 | 9 | 9 | 14 | 1.29E+08 | 0.78 | 7 | 21 | 1037 | 117.3 | 6.9 | Protein metabolism | Extracellular | Metallopeptidase activity | Extracellular |
| RPF2 | NP_115570.1 | 84154 | ribosome production factor 2 homolog isoform 1 | 25.8 | 2 | 6 | 6 | 14 | 3.47E+08 | 0.78 | 7 | 9.3 | 306 | 35.6 | 10.0 | Biological_process unknown | Nucleolus;Nucleus | Molecular function unknown | Nucleolus |
| DDX1 | NP_004930.1 | 1653 | ATP-dependent RNA helicase DDX1 | 46.6 | 1 | 26 | 26 | 144 | 1.64E+09 | 0.77 | 41 | 20.6 | 740 | 82.4 | 7.2 | Regulation of nucleobase, nucleoside, nucleotide and nucleic acid metabolism | Nucleus;Nucleolus | RNA binding | Nucleus |
| GIGYF2 | NP_001096618.1 | 26058 | PERQ amino acid-rich with GYF domain-containing protein 2 isoform c | 7.5 | 3 | 6 | 6 | 16 | 1.34E+08 | 0.77 | 4 | 6 | 1293 | 149.4 | 5.5 | Cell communication;Signal transduction | Nucleus;Cytoplasm | Molecular function unknown | Nucleus |
| MIA3 | NP_940953.2 | 375056 | melanoma inhibitory activity protein 3 precursor | 5.5 | 1 | 5 | 5 | 7 | 5.02E+07 | 0.77 | 3 | 3.5 | 1907 | 213.6 | 4.8 | Biological_process unknown | - | Molecular function unknown | - |
| PPP1CB | NP_002700.1 | 5500 | serine/threonine-protein phosphatase PP1-beta catalytic subunit isoform 1 | 37.9 | 1 | 2 | 14 | 51 | 4.73E+08 | 0.77 | 1 | - | 327 | 37.2 | 6.2 | Cell growth and/or maintenance | Nucleolus;Microtubule | Protein serine/threonine phosphatase activity | Nucleolus |
| RPL18 | NP_001257419.1 | 6141 | 60S ribosomal protein L18 isoform 2 | 44.7 | 2 | 9 | 9 | 97 | 3.00E+09 | 0.77 | 17 | 13.3 | 159 | 18.1 | 11.8 | Protein metabolism | Ribosome;Nucleolus;Mitochondrion | Structural constituent of ribosome | Ribosome |
| VAV2 | NP_003362.2 | 7410 | guanine nucleotide exchange factor VAV2 isoform 2 | 9.5 | 2 | 5 | 5 | 5 | 7.26E+07 | 0.77 | 3 | 32.1 | 839 | 97 | 6.9 | Cell communication;Signal transduction | Cytoplasm;Nucleus | Guanyl-nucleotide exchange factor activity | Cytoplasm |
| VPS26B | NP_443107.1 | 112936 | vacuolar protein sorting-associated protein 26B | 3.6 | 1 | 1 | 1 | 2 | 6.11E+07 | 0.77 | 1 | - | 336 | 39.1 | 7.4 | Transport | Plasma membrane;Cytosol | Transporter activity | Plasma membrane |
| ATP6V1H | NP_998784.1 | 51606 | V-type proton ATPase subunit H isoform 2 | 20.7 | 2 | 6 | 6 | 17 | 2.44E+08 | 0.77 | 7 | 13 | 465 | 54.1 | 6.5 | Transport | - | ATPase activity | - |
| MRPL37 | NP_057575.2 | 51253 | 39S ribosomal protein L37, mitochondrial | 32.2 | 1 | 7 | 7 | 16 | 2.92E+08 | 0.77 | 5 | 2.2 | 423 | 48.1 | 8.6 | Protein metabolism | Mitochondrion | Structural constituent of ribosome | Mitochondrion |
| GPC3 | NP_001158091.1 | 2719 | glypican-3 isoform 4 precursor | 1.3 | 4 | 1 | 1 | 5 | 1.44E+08 | 0.77 | 2 | 19.6 | 526 | 59.4 | 6.3 | Cell communication;Signal transduction | Extracellular;Plasma membrane | Molecular function unknown | Extracellular |
| MCM7 | NP_005907.3 | 4176 | DNA replication licensing factor MCM7 isoform 1 | 51.5 | 2 | 30 | 30 | 134 | 8.48E+08 | 0.77 | 43 | 19.7 | 719 | 81.3 | 6.5 | Regulation of nucleobase, nucleoside, nucleotide and nucleic acid metabolism;DNA replication | Nucleus;Nucleolus;Chromosome | Helicase activity | Nucleus |
| MPV17 | NP_002428.1 | 4358 | protein Mpv17 | 13.1 | 1 | 1 | 1 | 1 | 4.68E+07 | 0.77 | 1 | - | 176 | 19.7 | 9.5 | Biological_process unknown | Peroxisome;Mitochondrion | Molecular function unknown | Peroxisome;Mitochondrion |
| YWHAE | NP_006752.1 | 7531 | 14-3-3 protein epsilon | 82.0 | 1 | 23 | 26 | 479 | 1.73E+10 | 0.77 | 69 | 14.2 | 255 | 29.2 | 4.7 | Cell communication;Signal transduction | Cytoplasm;Nucleus;Golgi apparatus;Nucleolus;Mitochondrion | Receptor signaling complex scaffold activity | Cytoplasm |
| RPS4X | NP_000998.1 | 6191 | 40S ribosomal protein S4, X isoform X isoform | 71.5 | 3 | 24 | 24 | 151 | 2.64E+09 | 0.77 | 40 | 9.8 | 263 | 29.6 | 10.2 | Protein metabolism | Ribosome;Nucleolus;Cytosol | Structural constituent of ribosome | Ribosome |
| SDR39U1 | NP_064580.2 | 56948 | epimerase family protein SDR39U1 isoform 1 | 7.2 | 1 | 1 | 1 | 1 | 4.54E+06 | 0.77 | 1 | - | 293 | 31.1 | 9.0 | Biological_process unknown | - | Molecular function unknown | - |
| VCL | NP_003364.1 | 7414 | vinculin isoform VCL | 59.1 | 2 | 49 | 49 | 285 | 3.79E+09 | 0.77 | 61 | 18 | 1066 | 116.6 | 6.1 | Cell growth and/or maintenance | Cytoplasm;Plasma membrane;Nucleus | Cytoskeletal protein binding | Cytoplasm |
| BUB3 | NP_001007794.1 | 9184 | mitotic checkpoint protein BUB3 isoform b | 37.1 | 2 | 8 | 8 | 41 | 5.37E+08 | 0.77 | 7 | 20.3 | 326 | 36.9 | 6.8 | Regulation of cell cycle;Signal transduction | Kinetochore;Cytoplasm;Nucleus;Nucleolus;Chromosome;Mitochondrion | Protein transporter activity | Cytoplasm |
| COMT | NP_009294.1 | 1312 | catechol O-methyltransferase isoform S-COMT | 82.8 | 2 | 16 | 16 | 57 | 6.33E+08 | 0.77 | 23 | 18.2 | 221 | 24.4 | 5.3 | Metabolism;Energy pathways | Endoplasmic reticulum;Cytoplasm | Acyltransferase activity | Endoplasmic reticulum;Cytoplasm |
| LDLR | NP_001182732.1 | 3949 | low-density lipoprotein receptor isoform 6 precursor | 15.3 | 5 | 6 | 6 | 15 | 1.12E+08 | 0.77 | 5 | 24.7 | 682 | 75.8 | 5.2 | Cell communication;Signal transduction | Plasma membrane;Lysosome;Endosome | Receptor activity | Plasma membrane |

| Gene Symbol | RefSeq_Accession | Entrez Gene ID | Description | Coverage (%) | Proteins | Unique Peptides | # Peptides | PSM | Area | SCC-R /SCC-S | SCC-R /SCC-S (Count) | SCC-R /SCC-S (Variability [%]) | # AAs | MW [kDa] | calc. pI | Biological Process | Localization | Molecular Function | Primary Localization |
|---|---|---|---|---|---|---|---|---|---|---|---|---|---|---|---|---|---|---|---|
| **MAPKAPK2** | NP_004750.1 | 9261 | **MAP kinase-activated protein kinase 2 isoform 1** | 4.6 | 2 | 1 | 1 | 1 | 2.28E+07 | **0.77** | 1 | - | 370 | 42.2 | 8.3 | Cell communication;Signal transduction | Cytoplasm;Nucleus | Protein serine/threonine kinase activity | Nucleus |
| **TMEM258** | NP_055021.1 | 746 | **transmembrane protein 258** | 10.1 | 1 | 1 | 1 | 2 | 1.74E+08 | **0.77** | 1 | - | 79 | 9.1 | 5.8 | Biological_process unknown | Integral to membrane | Molecular function unknown | Integral to membrane |
| **UNC45A** | NP_001034764.1 | 55898 | **protein unc-45 homolog A isoform 3** | 30.9 | 2 | 19 | 19 | 44 | 6.88E+08 | **0.77** | 20 | 20.1 | 929 | 101.6 | 6.1 | Biological_process unknown | Nucleus | Molecular function unknown | Nucleus |
| **BZW2** | NP_054757.1 | 28969 | **basic leucine zipper and W2 domain-containing protein 2** | 26.3 | 1 | 6 | 7 | 12 | 7.57E+07 | **0.77** | 4 | 6.4 | 419 | 48.1 | 6.7 | Protein metabolism | Nucleolus | Translation regulator activity | Nucleolus |
| **CDK11B** | NP_277024.2 | 984 | **cyclin-dependent kinase 11B isoform 5** | 5.9 | 8 | 4 | 4 | 7 | 5.35E+07 | **0.77** | 4 | 17.2 | 748 | 87.1 | 5.4 | Cell communication;Signal transduction | Nucleus;Cytoplasm | Protein serine/threonine kinase activity | Nucleus;Cytoplasm |
| **PRKAR2A** | NP_004148.1 | 5576 | **cAMP-dependent protein kinase type II-alpha regulatory subunit** | 34.2 | 1 | 8 | 10 | 19 | 1.06E+08 | **0.77** | 8 | 30 | 404 | 45.5 | 5.1 | Cell communication;Signal transduction | Cytoplasm;Nucleus | Protein serine/threonine kinase activity | Cytoplasm |
| **TCEAL4** | NP_001006938.1 | 79921 | **transcription elongation factor A protein-like 4** | 14.0 | 2 | 2 | 2 | 2 | 2.89E+07 | **0.77** | 2 | 9.8 | 215 | 24.6 | 5.2 | Biological_process unknown | - | Molecular function unknown | - |
| **DDX23** | NP_004809.2 | 9416 | **probable ATP-dependent RNA helicase DDX23** | 13.2 | 1 | 8 | 8 | 11 | 1.82E+08 | **0.77** | 8 | 12.5 | 820 | 95.5 | 9.6 | Protein metabolism | Nucleus;Nucleolus;Cytoplasm | RNA binding | Nucleus |
| **EMG1** | NP_006322.4 | 10436 | **ribosomal RNA small subunit methyltransferase NEP1** | 35.3 | 1 | 5 | 5 | 21 | 1.73E+08 | **0.77** | 6 | 25.4 | 244 | 26.7 | 9.2 | Protein metabolism | Nucleus;Nucleolus | Translation regulator activity | Nucleus |
| **RPL12** | NP_000967.1 | 6136 | **60S ribosomal protein L12** | 58.8 | 1 | 8 | 8 | 123 | 4.19E+09 | **0.77** | 22 | 8.2 | 165 | 17.8 | 9.4 | Protein metabolism | Nucleus;Nucleolus;Mitochondrion;Ribosome | Structural constituent of ribosome | Nucleus |
| **RRN3** | NP_060897.3 | 54700 | **RNA polymerase I-specific transcription initiation factor RRN3** | 4.0 | 1 | 1 | 1 | 1 | 1.76E+07 | **0.77** | 1 | - | 651 | 74.1 | 5.6 | Regulation of nucleobase, nucleoside, nucleotide and nucleic acid metabolism | Nucleolus;Nucleus | Transcription factor activity | Nucleolus |
| **YWHAQ** | NP_006817.1 | 10971 | **14-3-3 protein theta** | 76.3 | 1 | 19 | 24 | 283 | 1.83E+10 | **0.77** | 35 | 12.2 | 245 | 27.7 | 4.8 | Cell communication;Signal transduction | Cytoplasm;Nucleus;Cytosol | Receptor signaling complex scaffold activity | Cytoplasm |
| **AKAP9** | NP_671714.1 | 10142 | **A-kinase anchor protein 9 isoform 3** | 0.4 | 2 | 1 | 1 | 2 | 4.07E+07 | **0.77** | 1 | - | 3899 | 451.8 | 5.0 | Cell communication;Signal transduction | Cytoplasm;Golgi apparatus;Centrosome | Receptor signaling complex scaffold activity | Centrosome |
| **DIMT1** | NP_055288.1 | 27292 | **probable dimethyladenosine transferase** | 12.8 | 1 | 2 | 2 | 5 | 7.17E+07 | **0.77** | 2 | 9.1 | 313 | 35.2 | 10.0 | Biological_process unknown | - | Molecular function unknown | - |
| **PWP1** | NP_008993.1 | 11137 | **periodic tryptophan protein 1 homolog** | 12.6 | 1 | 4 | 4 | 8 | 1.00E+08 | **0.77** | 3 | 7 | 501 | 55.8 | 4.8 | Cell growth and/or maintenance | Nucleus;Endoplasmic reticulum;Nucleolus | Transcription regulator activity | Nucleus |
| **RBM26** | NP_071401.3 | 64062 | **RNA-binding protein 26 isoform 3** | 13.7 | 2 | 1 | 7 | 12 | 1.26E+08 | **0.77** | 1 | - | 980 | 110.6 | 9.1 | Regulation of nucleobase, nucleoside, nucleotide and nucleic acid metabolism | Nucleus | RNA binding | Nucleus |
| **GLS** | NP_001243239.1 | 2744 | **glutaminase kidney isoform, mitochondrial isoform 2** | 31.4 | 2 | 11 | 11 | 20 | 2.14E+08 | **0.77** | 8 | 10 | 598 | 65.4 | 7.9 | Metabolism;Energy pathways | Mitochondrion;Golgi apparatus | Deaminase activity | Mitochondrion |
| **NME1** | NP_000260.1 | 4830 | **nucleoside diphosphate kinase A isoform b** | 86.2 | 2 | 2 | 11 | 241 | 3.58E+09 | **0.77** | 1 | - | 152 | 17.1 | 6.2 | Metabolism;Energy pathways | Cytoplasm;Nucleus;Extracellular;Mitochondrion | Catalytic activity | Cytoplasm |
| **OXA1L** | NP_005006.3 | 5018 | **mitochondrial inner membrane protein OXA1L** | 2.4 | 1 | 1 | 1 | 2 | 2.17E+07 | **0.77** | 1 | - | 495 | 55.2 | 9.7 | Metabolism;Energy pathways | Mitochondrion;Cytoplasm;Nucleus | Catalytic activity | Mitochondrion |
| **PTPN2** | NP_536347.1 | 5771 | **tyrosine-protein phosphatase non-receptor type 2 isoform 2** | 9.0 | 4 | 2 | 2 | 3 | 4.48E+07 | **0.77** | 1 | - | 387 | 45.1 | 8.1 | Cell communication;Signal transduction | Endoplasmic reticulum;Nucleus;Cytoplasm | Protein tyrosine phosphatase activity | Endoplasmic reticulum;Nucleus |
| **RPN2** | NP_001129243.1 | 6185 | **dolichyl-diphosphooligosaccharide--protein glycosyltransferase subunit 2 isoform 2 precursor** | 43.6 | 2 | 18 | 18 | 77 | 2.63E+08 | **0.77** | 23 | 21.9 | 615 | 67.7 | 6.1 | Protein metabolism | Endoplasmic reticulum;Mitochondrion | Transferase activity | Endoplasmic reticulum |
| **SRCIN1** | NP_079524.2 | 80725 | **SRC kinase signaling inhibitor 1** | 0.6 | 1 | 1 | 1 | 1 | 1.35E+09 | **0.77** | 1 | - | 1183 | 127 | 9.4 | Cell communication;Signal transduction | Cytoplasm;Plasma membrane | Molecular function unknown | Cytoplasm |
| **FAU** | NP_001988.1 | 2197 | **ubiquitin-like protein fubi and ribosomal protein S30 precursor** | 8.3 | 1 | 2 | 2 | 39 | 2.78E+09 | **0.76** | 8 | 12.3 | 133 | 14.4 | 10.2 | Protein metabolism | Ribosome;Nucleolus | Ubiquitin-specific protease activity | Ribosome |
| **GLUD1** | NP_005262.1 | 2746 | **glutamate dehydrogenase 1, mitochondrial precursor** | 64.9 | 2 | 27 | 27 | 120 | 1.16E+09 | **0.76** | 33 | 20.6 | 558 | 61.4 | 7.8 | Metabolism;Energy pathways | Mitochondrion;Cytosol | Catalytic activity | Mitochondrion |
| **GTPBP10** | NP_149098.2 | 85865 | **GTP-binding protein 10 isoform 2** | 11.1 | 2 | 3 | 3 | 5 | 4.25E+07 | **0.76** | 3 | 20.4 | 387 | 42.9 | 9.0 | Regulation of nucleobase, nucleoside, nucleotide and nucleic acid metabolism | Nucleus | RNA binding | Nucleus |
| **JMJD7** | NP_001108104.1 | 100137047 | **jmjC domain-containing protein 7** | 7.6 | 1 | 1 | 1 | 2 | 1.22E+08 | **0.76** | 1 | - | 316 | 35.9 | 5.4 | Biological_process unknown | - | Molecular function unknown | - |
| **MTMR2** | NP_958438.1 | 8898 | **myotubularin-related protein 2 isoform 2** | 7.0 | 2 | 2 | 2 | 3 | 1.63E+08 | **0.76** | 2 | 28.7 | 571 | 65.9 | 7.9 | Protein metabolism | Cytoplasm;Nucleus | Protein tyrosine phosphatase activity | Cytoplasm |
| **SMC2** | NP_001036015.1 | 10592 | **structural maintenance of chromosomes protein 2** | 36.3 | 1 | 35 | 35 | 93 | 4.02E+08 | **0.76** | 44 | 14.6 | 1197 | 135.6 | 8.4 | Biological_process unknown | Nucleus;Nucleolus;Mitochondrion | Molecular function unknown | Nucleus |
| **STK11IP** | NP_443134.2 | 114790 | **serine/threonine-protein kinase 11-interacting protein** | 1.4 | 1 | 1 | 1 | 1 | 1.62E+08 | **0.76** | 1 | - | 1099 | 121.3 | 5.4 | Cell communication;Signal transduction | Cytoplasm;Nucleus | Molecular function unknown | Cytoplasm |
| **GFOD2** | NP_001230579.1 | 81577 | **glucose-fructose oxidoreductase domain-containing protein 2 isoform 2 precursor** | 6.3 | 2 | 1 | 1 | 1 | 2.12E+07 | **0.76** | 1 | - | 112 | 12.1 | 6.0 | Biological_process unknown | Nucleus | Molecular function unknown | Nucleus |
| **GPS2** | NP_004480.1 | 2874 | **G protein pathway suppressor 2** | 2.5 | 1 | 1 | 1 | 1 | 2.58E+07 | **0.76** | 1 | - | 327 | 36.7 | 9.5 | Cell communication;Signal transduction | Nucleus | GTPase regulator activity | Nucleus |
| **HECTD1** | NP_056197.3 | 25831 | **E3 ubiquitin-protein ligase HECTD1** | 2.1 | 1 | 3 | 3 | 4 | 8.12E+07 | **0.76** | 1 | - | 2610 | 289.2 | 5.4 | Protein metabolism | - | Ubiquitin-specific protease activity | - |
| **CSNK2A1** | NP_001886.1 | 1457 | **casein kinase II subunit alpha isoform a** | 34.3 | 3 | 9 | 10 | 43 | 6.12E+08 | **0.76** | 16 | 19.5 | 391 | 45.1 | 7.7 | Signal transduction;Protein modification | Nucleus;Cytoplasm;Plasma membrane | Protein serine/threonine kinase activity | Nucleus |
| **DHX38** | NP_054722.2 | 9785 | **pre-mRNA-splicing factor ATP-dependent RNA helicase PRP16** | 1.5 | 1 | 1 | 1 | 1 | 1.01E+07 | **0.76** | 1 | - | 1227 | 140.4 | 6.5 | Regulation of nucleobase, nucleoside, nucleotide and nucleic acid metabolism | Nucleus | RNA binding | Nucleus |
| **NAA15** | NP_476516.1 | 80155 | **N-alpha-acetyltransferase 15, NatA auxiliary subunit** | 32.8 | 4 | 20 | 20 | 55 | 4.00E+08 | **0.76** | 17 | 23 | 866 | 101.2 | 7.4 | Metabolism;Energy pathways | Cytoplasm;Nucleus | Transferase activity | Cytoplasm;Nucleus |
| **AP1S1** | NP_001274.1 | 1174 | **AP-1 complex subunit sigma-1A** | 15.8 | 1 | 2 | 2 | 6 | 8.61E+08 | **0.76** | 3 | 9.5 | 158 | 18.7 | 5.7 | Transport | Golgi apparatus | Transporter activity | Golgi apparatus |
| **CIRBP** | NP_001271.1 | 1153 | **cold-inducible RNA-binding protein** | 44.2 | 1 | 6 | 6 | 21 | 3.13E+08 | **0.76** | 6 | 9 | 172 | 18.6 | 9.5 | Cell communication;Signal transduction | Nucleus | RNA binding | Nucleus |

| Gene Symbol | RefSeq_Accession | Entrez Gene ID | Description | Coverage (%) | Proteins | Unique Peptides | # Peptides | PSM | Area | SCC-R /SCC-S | SCC-R /SCC-S (Count) | SCC-R /SCC-S (Variability [%]) | # AAs | MW [kDa] | calc. pI | Biological Process | Localization | Molecular Function | Primary Localization |
|---|---|---|---|---|---|---|---|---|---|---|---|---|---|---|---|---|---|---|---|
| **COX7C** | NP_001858.1 | 1350 | **cytochrome c oxidase subunit 7C, mitochondrial precursor** | 23.8 | 1 | 2 | 2 | 6 | 1.33E+08 | **0.76** | 2 | 31.4 | 63 | 7.2 | 10.3 | Metabolism;Energy pathways | Mitochondrion | Molecular function unknown | Mitochondrion |
| **MRPL3** | NP_009139.1 | 11222 | **39S ribosomal protein L3, mitochondrial** | 20.1 | 1 | 3 | 3 | 8 | 1.80E+08 | **0.76** | 4 | 17.2 | 348 | 38.6 | 9.5 | Protein metabolism | Nucleolus;Ribosome | Structural constituent of ribosome | Nucleolus |
| **RPL19** | NP_000972.1 | 6143 | **60S ribosomal protein L19** | 21.9 | 1 | 6 | 6 | 60 | 1.09E+09 | **0.76** | 10 | 12.7 | 196 | 23.5 | 11.5 | Protein metabolism | Ribosome | Structural constituent of ribosome | Ribosome |
| **RPL27A** | NP_000981.1 | 6157 | **60S ribosomal protein L27a** | 32.4 | 1 | 6 | 6 | 97 | 2.26E+09 | **0.76** | 18 | 14.5 | 148 | 16.6 | 11.0 | Protein metabolism | Ribosome;Nucleolus;Mitochondrion | Structural constituent of ribosome | Ribosome |
| **ANAPC16** | NP_775744.1 | 119504 | **anaphase-promoting complex subunit 16 isoform 1** | 10.9 | 1 | 1 | 1 | 1 | 1.40E+08 | **0.76** | 1 | - | 110 | 11.7 | 5.0 | Biological_process unknown | - | Molecular function unknown | - |
| **CHTOP** | NP_056422.2 | 26097 | **chromatin target of PRMT1 protein isoform 1** | 21.8 | 3 | 5 | 5 | 14 | 2.51E+08 | **0.76** | 2 | 3 | 248 | 26.4 | 12.2 | Biological_process unknown | Nucleus | Molecular function unknown | Nucleus |
| **MCTS1** | NP_054779.1 | 28985 | **malignant T-cell-amplified sequence 1 isoform 1** | 59.1 | 2 | 7 | 7 | 82 | 6.68E+08 | **0.76** | 15 | 17 | 181 | 20.5 | 8.8 | Cell communication;Signal transduction | Cytoplasm | DNA binding | Cytoplasm |
| **UTP3** | NP_065101.1 | 57050 | **something about silencing protein 10** | 14.8 | 1 | 5 | 5 | 9 | 4.40E+07 | **0.76** | 5 | 45.8 | 479 | 54.5 | 5.6 | Biological_process unknown | Nucleolus | Molecular function unknown | Nucleolus |
| **EIF3C** | NP_001186071.1 | 8663 | **eukaryotic translation initiation factor 3 subunit C isoform a** | 34.3 | 3 | 29 | 29 | 80 | 8.66E+08 | **0.76** | 27 | 18.6 | 913 | 105.3 | 5.7 | Protein metabolism | Cytoplasm;Ribosome | Translation regulator activity | Cytoplasm |
| **HSPB1** | NP_001531.1 | 3315 | **heat shock protein beta-1** | 90.2 | 1 | 17 | 17 | 230 | 6.47E+09 | **0.76** | 47 | 38.8 | 205 | 22.8 | 6.4 | Protein metabolism | Cytoplasm;Nucleus;Plasma membrane;Microtubule | Chaperone activity | Cytoplasm |
| **LIMCH1** | NP_001106191.1 | 22998 | **LIM and calponin homology domains-containing protein 1 isoform e** | 1.4 | 7 | 1 | 1 | 1 | 6.41E+06 | **0.76** | 1 | - | 890 | 100.7 | 6.3 | Biological_process unknown | Nucleus;Cytoplasm | Molecular function unknown | Nucleus;Cytoplasm |
| **PDHX** | NP_001128496.1 | 8050 | **pyruvate dehydrogenase protein X component, mitochondrial isoform 2** | 9.3 | 3 | 3 | 3 | 5 | 1.25E+07 | **0.76** | 3 | 25.2 | 486 | 51.4 | 6.3 | Metabolism;Energy pathways | Mitochondrion | Catalytic activity | Mitochondrion |
| **FKBP8** | NP_036313.3 | 23770 | **peptidyl-prolyl cis-trans isomerase FKBP8** | 13.8 | 1 | 4 | 4 | 9 | 1.87E+08 | **0.76** | 2 | 0.9 | 413 | 44.6 | 4.8 | Apoptosis | Mitochondrion;Cytoplasm;Nucleus | Receptor signaling complex scaffold activity;Protease inhibitor activity | Mitochondrion |
| **NRM** | NP_001257637.1 | 11270 | **nurim isoform 3** | 18.0 | 4 | 1 | 1 | 1 | 4.54E+07 | **0.76** | 1 | - | 111 | 11.9 | 9.0 | Biological_process unknown | Nucleus | Molecular function unknown | Nucleus |
| **RPL5** | NP_000960.2 | 6125 | **60S ribosomal protein L5** | 59.3 | 1 | 17 | 17 | 164 | 2.82E+09 | **0.76** | 40 | 19 | 297 | 34.3 | 9.7 | Protein metabolism | Ribosome;Nucleolus;Cytosol | Structural constituent of ribosome | Ribosome |
| **CHERP** | NP_006378.3 | 10523 | **calcium homeostasis endoplasmic reticulum protein** | 14.7 | 1 | 8 | 8 | 22 | 2.24E+08 | **0.76** | 8 | 25.9 | 916 | 103.6 | 9.0 | Regulation of cell proliferation;Homeostasis | Cytoplasm;Endoplasmic reticulum membrane;Perinuclear region | Molecular function unknown | Endoplasmic reticulum membrane |
| **TUBGCP3** | NP_001273206.1 | 10426 | **gamma-tubulin complex component 3 isoform 2** | 9.6 | 4 | 5 | 5 | 9 | 9.45E+07 | **0.76** | 6 | 8.4 | 897 | 102.4 | 8.1 | Cell growth and/or maintenance | Cytoplasm;Centrosome;Microtubule | Cytoskeletal protein binding | Centrosome |
| **APRT** | NP_000476.1 | 353 | **adenine phosphoribosyltransferase isoform a** | 71.1 | 2 | 10 | 10 | 34 | 8.07E+08 | **0.76** | 13 | 14.4 | 180 | 19.6 | 6.0 | Purine salvage | - | Transferase activity | - |
| **LRRC57** | NP_694992.2 | 255252 | **leucine-rich repeat-containing protein 57** | 5.4 | 1 | 1 | 1 | 1 | 4.47E+07 | **0.76** | 1 | - | 239 | 26.7 | 8.4 | Biological_process unknown | - | Molecular function unknown | - |
| **MLH1** | NP_001245200.1 | 4292 | **DNA mismatch repair protein Mlh1 isoform 4** | 3.8 | 4 | 2 | 2 | 2 | 8.93E+07 | **0.76** | 1 | - | 687 | 76.7 | 6.2 | Regulation of nucleobase, nucleoside, nucleotide and nucleic acid metabolism | Nucleus | DNA repair protein | Nucleus |
| **EIF3E** | NP_001559.1 | 3646 | **eukaryotic translation initiation factor 3 subunit E** | 36.0 | 1 | 15 | 15 | 55 | 1.39E+09 | **0.75** | 26 | 18.7 | 445 | 52.2 | 6.0 | Protein metabolism | Nucleus;Cytoplasm | Translation regulator activity | Cytoplasm |
| **ST13** | NP_001265518.1 | 6767 | **hsc70-interacting protein isoform 2** | 30.1 | 2 | 12 | 12 | 45 | 6.33E+08 | **0.75** | 17 | 29.7 | 359 | 40.1 | 5.3 | Cell communication;Signal transduction | Lysosome;Cytoplasm | Receptor signaling complex scaffold activity | Lysosome |
| **STX18** | NP_058626.1 | 53407 | **syntaxin-18** | 9.0 | 1 | 2 | 2 | 2 | 3.19E+07 | **0.75** | 1 | - | 335 | 38.6 | 5.5 | Transport | Endoplasmic reticulum;Golgi apparatus | Auxiliary transport protein activity | Endoplasmic reticulum |
| **TRMT5** | NP_065861.3 | 57570 | **tRNA (guanine(37)-N1)-methyltransferase** | 7.9 | 1 | 2 | 2 | 3 | 1.93E+07 | **0.75** | 1 | - | 509 | 58.2 | 8.6 | Metabolism;Energy pathways | - | Methyltransferase activity | - |
| **EIF4A3** | NP_055555.1 | 9775 | **eukaryotic initiation factor 4A-III** | 58.4 | 1 | 20 | 23 | 151 | 2.56E+09 | **0.75** | 24 | 28.1 | 411 | 46.8 | 6.7 | Regulation of nucleobase, nucleoside, nucleotide and nucleic acid metabolism | Nucleus;Nucleolus | Molecular function unknown | Nucleus |
| **FARSA** | NP_004452.1 | 2193 | **phenylalanine--tRNA ligase alpha subunit** | 33.1 | 1 | 10 | 10 | 39 | 2.89E+08 | **0.75** | 13 | 19.5 | 508 | 57.5 | 7.8 | Protein metabolism | Cytoplasm | Ligase activity | Cytoplasm |
| **PCDH15** | NP_001136242.1 | 65217 | **protocadherin-15 isoform CD2-2 precursor** | 0.4 | 12 | 1 | 1 | 2 | 8.82E+07 | **0.75** | 1 | - | 1539 | 171.2 | 5.1 | Cell communication;Signal transduction | Plasma membrane | Cell adhesion molecule activity | Plasma membrane |
| **STBD1** | NP_003934.1 | 8987 | **starch-binding domain-containing protein 1** | 6.4 | 1 | 1 | 1 | 3 | 1.14E+07 | **0.75** | 1 | - | 358 | 39 | 5.7 | Biological_process unknown | Sarcoplasmic reticulum;Plasma membrane | Molecular function unknown | Plasma membrane |
| **SET** | NP_003002.2 | 6418 | **protein SET isoform 2** | 55.2 | 4 | 3 | 14 | 226 | 3.66E+09 | **0.75** | 4 | 23.1 | 277 | 32.1 | 4.2 | Regulation of nucleobase, nucleoside, nucleotide and nucleic acid metabolism | Cytoplasm;Endoplasmic reticulum;Nucleus;Plasma membrane;Cytosol;Nuclear membrane | MHC class I receptor activity;MHC class II receptor activity | Nucleus |
| **CHAMP1** | NP_001157616.1 | 283489 | **chromosome alignment-maintaining phosphoprotein 1** | 7.0 | 1 | 3 | 3 | 3 | 7.72E+06 | **0.75** | 1 | - | 812 | 89 | 8.4 | Regulation of nucleobase, nucleoside, nucleotide and nucleic acid metabolism | Nucleus;Cytoplasm | DNA binding | Nucleus |
| **IKBIP** | NP_710154.1 | 121457 | **inhibitor of nuclear factor kappa-B kinase-interacting protein isoform 1** | 21.0 | 1 | 4 | 5 | 7 | 8.59E+07 | **0.75** | 1 | - | 377 | 43.1 | 4.9 | Apoptosis | Endoplasmic reticulum | Molecular function unknown | Endoplasmic reticulum |
| **MRPL46** | NP_071446.2 | 26589 | **39S ribosomal protein L46, mitochondrial** | 50.5 | 1 | 8 | 8 | 18 | 9.67E+07 | **0.75** | 7 | 11.6 | 279 | 31.7 | 7.1 | Protein metabolism | Mitochondrion | Structural constituent of ribosome | Mitochondrion |
| **VDAC3** | NP_005653.3 | 7419 | **voltage-dependent anion-selective channel protein 3 isoform 1** | 35.7 | 2 | 8 | 8 | 18 | 9.45E+07 | **0.75** | 3 | 5.1 | 283 | 30.6 | 8.7 | Transport | Mitochondrion | Voltage-gated ion channel activity | Mitochondrion |
| **FTO** | NP_001073901.1 | 79068 | **alpha-ketoglutarate-dependent dioxygenase FTO** | 15.5 | 1 | 5 | 5 | 10 | 5.84E+07 | **0.75** | 7 | 32.9 | 505 | 58.2 | 5.2 | Biological_process unknown | - | Molecular function unknown | - |
| **PPP2R2A** | NP_002708.1 | 5520 | **serine/threonine-protein phosphatase 2A 55 kDa regulatory subunit B alpha isoform isoform 1** | 37.6 | 14 | 9 | 9 | 22 | 2.07E+08 | **0.75** | 11 | 37.6 | 447 | 51.7 | 6.2 | Cell communication;Signal transduction | Cytoplasm;Chromosome | Protein serine/threonine phosphatase activity | Cytoplasm |
| **CHID1** | NP_001136149.1 | 66005 | **chitinase domain-containing protein 1 isoform c precursor** | 21.6 | 3 | 4 | 4 | 7 | 1.42E+08 | **0.75** | 3 | 1.8 | 362 | 41.7 | 8.6 | Transport;Cell proliferation;Cell activation;Cell differentiation | Lysosome;Late endosome;Golgi apparatus;Nucleus;Cytoplasm | Transporter activity | Lysosome |
| **CTU1** | NP_660275.2 | 90353 | **cytoplasmic tRNA 2-thiolation protein 1** | 5.8 | 1 | 2 | 2 | 2 | 2.35E+06 | **0.75** | 2 | 44.1 | 348 | 36.4 | 9.2 | Biological_process unknown | - | Molecular function unknown | - |

| Gene Symbol | RefSeq_Accession | Entrez Gene ID | Description | Coverage (%) | Proteins | Unique Peptides | # Peptides | PSM | Area | SCC-R /SCC-S | SCC-R /SCC-S (Count) | SCC-R /SCC-S (Variability [%]) | # AAs | MW [kDa] | calc. pI | Biological Process | Localization | Molecular Function | Primary Localization |
|---|---|---|---|---|---|---|---|---|---|---|---|---|---|---|---|---|---|---|---|
| **HK2** | NP_000180.2 | 3099 | **hexokinase-2** | 16.1 | 1 | 10 | 12 | 22 | 2.73E+08 | **0.75** | 6 | 16.6 | 917 | 102.3 | 6.1 | Metabolism;Energy pathways | Mitochondrion | Catalytic activity | Mitochondrion |
| **ISY1** | NP_065752.1 | 57461 | **pre-mRNA-splicing factor ISY1 homolog isoform 2** | 15.4 | 3 | 3 | 3 | 5 | 3.36E+07 | **0.75** | 3 | 34.5 | 285 | 33 | 5.2 | Biological_process unknown | - | Molecular function unknown | - |
| **KCTD10** | NP_114160.1 | 83892 | **BTB/POZ domain-containing adapter for CUL3-mediated RhoA degradation protein 3** | 3.5 | 1 | 1 | 1 | 2 | 1.61E+07 | **0.75** | 1 | - | 313 | 35.4 | 6.3 | Transport | - | Ion channel activity | - |
| **KIAA1715** | NP_085153.1 | 80856 | **protein lunapark** | 14.3 | 1 | 3 | 3 | 6 | 1.49E+07 | **0.75** | 3 | 85.7 | 428 | 47.7 | 5.1 | Biological_process unknown | Integral to membrane | Protein binding | Integral to membrane |
| **TBL2** | NP_036585.1 | 26608 | **transducin beta-like protein 2 precursor** | 15.2 | 1 | 5 | 5 | 12 | 8.18E+07 | **0.75** | 4 | 15.1 | 447 | 49.8 | 9.4 | Cell communication;Signal transduction | Nucleus | Molecular function unknown | Nucleus |
| **ALYREF** | NP_005773.3 | 10189 | **THO complex subunit 4** | 40.5 | 1 | 6 | 6 | 75 | 1.58E+09 | **0.75** | 15 | 15 | 264 | 27.5 | 11.1 | Protein metabolism | Nucleus;Nucleolus;Mitochondrion | Chaperone activity | Nucleus |
| **COASY** | NP_079509.5 | 80347 | **bifunctional coenzyme A synthase isoform a precursor** | 18.4 | 2 | 5 | 5 | 10 | 5.16E+07 | **0.75** | 4 | 12.1 | 564 | 62.3 | 7.0 | Metabolism;Energy pathways | Cytoplasm;Mitochondrion | Transferase activity | Cytoplasm |
| **DNAJA2** | NP_005871.1 | 10294 | **dnaJ homolog subfamily A member 2** | 41.3 | 1 | 12 | 12 | 56 | 1.18E+09 | **0.75** | 20 | 31.1 | 412 | 45.7 | 6.5 | Protein metabolism | Cytoplasm;Nucleus;Mitochondrion;Microsome | Chaperone activity | Cytoplasm |
| **MCM6** | NP_005906.2 | 4175 | **DNA replication licensing factor MCM6** | 41.9 | 1 | 32 | 32 | 90 | 9.08E+08 | **0.75** | 38 | 20.7 | 821 | 92.8 | 5.4 | Regulation of nucleobase, nucleoside, nucleotide and nucleic acid metabolism | Nucleus;Nucleolus;Chromosome | Protein binding | Nucleus |
| **OTUD4** | NP_001096123.1 | 54726 | **OTU domain-containing protein 4 isoform 3** | 1.5 | 1 | 1 | 1 | 1 | 9.10E+07 | **0.75** | 1 | - | 1049 | 117 | 6.7 | Biological_process unknown | Cytoplasm | Molecular function unknown | Cytoplasm |
| **SLC35E1** | NP_079157.3 | 79939 | **solute carrier family 35 member E1** | 5.4 | 1 | 1 | 1 | 2 | 2.32E+08 | **0.75** | 1 | - | 410 | 44.7 | 9.8 | Transport | Integral to membrane | Auxiliary transport protein activity | Integral to membrane |
| **UBTF** | NP_001070152.1 | 7343 | **nucleolar transcription factor 1 isoform b** | 15.0 | 2 | 7 | 7 | 10 | 1.96E+08 | **0.75** | 6 | 15.6 | 727 | 84.9 | 5.6 | Regulation of nucleobase, nucleoside, nucleotide and nucleic acid metabolism | Nucleolus;Nucleus | Transcription factor activity | Nucleolus |
| **EZR** | NP_003370.2 | 7430 | **ezrin** | 58.9 | 1 | 28 | 38 | 254 | 7.71E+09 | **0.75** | 51 | 11.4 | 586 | 69.4 | 6.3 | Cell growth and/or maintenance | Cytoplasm;Plasma membrane;Mitochondrion | Cytoskeletal anchoring activity | Cytoplasm |
| **MCAT** | NP_055322.1 | 27349 | **malonyl-CoA-acyl carrier protein transacylase, mitochondrial isoform b precursor** | 9.4 | 2 | 1 | 1 | 1 | 2.65E+07 | **0.75** | 1 | - | 180 | 19.2 | 6.2 | Fatty acid metabolism | Mitochondrion;Nucleus | Acyltransferase activity | Mitochondrion |
| **PREP** | NP_002717.3 | 5550 | **prolyl endopeptidase** | 26.1 | 1 | 12 | 12 | 32 | 2.23E+08 | **0.75** | 9 | 15.4 | 710 | 80.6 | 5.9 | Protein metabolism | Cytoplasm;Mitochondrion;Nucleus | Serine-type peptidase activity | Cytoplasm |
| **GPI** | NP_000166.2 | 2821 | **glucose-6-phosphate isomerase isoform 2** | 57.7 | 4 | 33 | 33 | 278 | 7.02E+09 | **0.75** | 84 | 19.7 | 558 | 63.1 | 8.3 | Metabolism;Energy pathways | Cytoplasm;Mitochondrion | Isomerase activity | Cytoplasm |
| **RPL3** | NP_000958.1 | 6122 | **60S ribosomal protein L3 isoform a** | 43.9 | 3 | 22 | 22 | 190 | 1.70E+09 | **0.75** | 48 | 13 | 403 | 46.1 | 10.2 | Protein metabolism | Cytoplasm;Nucleolus;Ribosome | Structural constituent of ribosome | Cytoplasm |
| **CDC26** | NP_644815.1 | 246184 | **anaphase-promoting complex subunit CDC26** | 14.1 | 1 | 1 | 1 | 2 | 1.53E+08 | **0.74** | 1 | - | 85 | 9.8 | 6.8 | Cell communication;Signal transduction | Centrosome | Molecular function unknown | - |
| **DDX54** | NP_076977.3 | 79039 | **ATP-dependent RNA helicase DDX54 isoform 2** | 26.1 | 2 | 13 | 13 | 26 | 2.27E+08 | **0.74** | 12 | 22.1 | 881 | 98.5 | 10.0 | Regulation of nucleobase, nucleoside, nucleotide and nucleic acid metabolism | Nucleolus;Nucleus;Cytoplasm | RNA binding | Nucleolus |
| **DNAJB1** | NP_006136.1 | 3337 | **dnaJ homolog subfamily B member 1** | 33.8 | 1 | 8 | 9 | 29 | 4.23E+08 | **0.74** | 6 | 14 | 340 | 38 | 8.6 | Protein metabolism | Cytoplasm;Nucleus;Endoplasmic reticulum;Cytosol | Heat shock protein activity | Cytoplasm |
| **MCM4** | NP_877423.1 | 4173 | **DNA replication licensing factor MCM4** | 59.6 | 5 | 31 | 31 | 96 | 1.03E+09 | **0.74** | 32 | 34.1 | 863 | 96.5 | 6.7 | Regulation of nucleobase, nucleoside, nucleotide and nucleic acid metabolism | Nucleus;Nucleolus;Chromosome | DNA binding | Nucleus |
| **NOL9** | NP_078930.3 | 79707 | **polynucleotide 5'-hydroxyl-kinase NOL9** | 15.5 | 1 | 5 | 5 | 9 | 2.56E+08 | **0.74** | 3 | 35.7 | 702 | 79.2 | 9.2 | Biological_process unknown | Nucleolus | Molecular function unknown | Nucleolus |
| **RPL28** | NP_000982.2 | 6158 | **60S ribosomal protein L28 isoform 2** | 46.0 | 5 | 9 | 9 | 44 | 8.78E+08 | **0.74** | 15 | 11 | 137 | 15.7 | 12.0 | Protein metabolism | Ribosome;Nucleolus | Structural constituent of ribosome | Ribosome |
| **RPS6** | NP_001001.2 | 6194 | **40S ribosomal protein S6** | 37.4 | 1 | 12 | 12 | 105 | 3.35E+09 | **0.74** | 20 | 21.1 | 249 | 28.7 | 10.8 | Protein metabolism | Ribosome;Nucleolus;Cytosol | Structural constituent of ribosome | Ribosome |
| **TMED9** | NP_059980.2 | 54732 | **transmembrane emp24 domain-containing protein 9 precursor** | 31.1 | 1 | 7 | 8 | 29 | 4.94E+08 | **0.74** | 5 | 44.3 | 235 | 27.3 | 8.0 | Biological_process unknown | Endoplasmic reticulum;Mitochondrion | Molecular function unknown | Endoplasmic reticulum |
| **PSMB10** | NP_002792.1 | 5699 | **proteasome subunit beta type-10 precursor** | 15.0 | 1 | 3 | 3 | 8 | 5.46E+07 | **0.74** | 3 | 32.7 | 273 | 28.9 | 7.8 | Protein metabolism | Cytoplasm | Ubiquitin-specific protease activity | Cytoplasm |
| **RPL18A** | NP_000971.1 | 6142 | **60S ribosomal protein L18a** | 43.8 | 1 | 10 | 10 | 54 | 1.69E+09 | **0.74** | 11 | 8.6 | 176 | 20.7 | 10.7 | Regulation of nucleobase, nucleoside, nucleotide and nucleic acid metabolism | Cytoplasm;Nucleolus;Mitochondrion | RNA binding | Cytoplasm |
| **IDH3G** | NP_004126.1 | 3421 | **isocitrate dehydrogenase [NAD] subunit gamma, mitochondrial isoform a precursor** | 36.1 | 2 | 7 | 7 | 15 | 2.10E+08 | **0.74** | 7 | 15.5 | 393 | 42.8 | 8.5 | Metabolism;Energy pathways | Mitochondrion | Catalytic activity | Mitochondrion |
| **MYO18B** | NP_115997.5 | 84700 | **unconventional myosin-XVIIIb** | 0.7 | 1 | 2 | 2 | 2 | 1.23E+08 | **0.74** | 1 | - | 2567 | 285 | 6.9 | Cell growth and/or maintenance | Cytoplasm;Nucleus | Motor activity | Cytoplasm;Nucleus |
| **ANXA7** | NP_001147.1 | 310 | **annexin A7 isoform 1** | 31.1 | 2 | 12 | 12 | 70 | 1.59E+09 | **0.74** | 17 | 12.4 | 466 | 50.3 | 6.6 | Transport | Nucleus;Plasma membrane;Endoplasmic reticulum;Cytoplasmic vesicle;Cytoplasm | Calcium ion binding | Nucleus |
| **AGO2** | NP_001158095.1 | 27161 | **protein argonaute-2 isoform 2** | 13.8 | 5 | 4 | 7 | 9 | 8.42E+07 | **0.74** | 1 | - | 825 | 93.6 | 9.3 | Protein metabolism | Cytoplasm | Translation regulator activity | Cytoplasm |
| **PUS7** | NP_061915.2 | 54517 | **pseudouridylate synthase 7 homolog** | 12.9 | 1 | 4 | 4 | 5 | 2.60E+07 | **0.74** | 2 | 28.9 | 661 | 75 | 6.4 | Regulation of nucleobase, nucleoside, nucleotide and nucleic acid metabolism | - | Lyase activity | - |
| **STAT3** | NP_998827.1 | 6774 | **signal transducer and activator of transcription 3 isoform 3** | 19.4 | 3 | 8 | 8 | 16 | 1.29E+08 | **0.74** | 3 | 0.9 | 722 | 83.1 | 7.1 | Regulation of nucleobase, nucleoside, nucleotide and nucleic acid metabolism | Cytoplasm;Nucleus;Mitochondrion;Cell surface;Extracellular matrix | Transcription factor activity | Nucleus;Extracellular matrix |
| **ACTG1** | NP_001186883.1 | 71 | **actin, cytoplasmic 2** | 82.4 | 5 | 2 | 42 | 3758 | 2.82E+10 | **0.74** | 4 | 25.3 | 375 | 41.8 | 5.5 | Cell growth and/or maintenance | Cytoplasm;Cytosol | Structural constituent of cytoskeleton | Cytoplasm |
| **CTBP2** | NP_001320.1 | 1488 | **C-terminal-binding protein 2 isoform 1** | 12.8 | 2 | 4 | 4 | 6 | 1.76E+08 | **0.74** | 4 | 18.6 | 445 | 48.9 | 7.0 | Cell communication;Signal transduction | Cytoplasm;Nucleus | Receptor signaling complex scaffold activity | Nucleus |
| **PLS3** | NP_001129497.1 | 5358 | **plastin-3 isoform 1** | 67.9 | 5 | 34 | 37 | 171 | 1.25E+09 | **0.74** | 51 | 18.1 | 630 | 70.8 | 5.6 | Cell growth and/or maintenance | Cytoplasm | Structural molecule activity | Cytoplasm |
| **SET** | NP_001116293.1 | 6418 | **protein SET isoform 1** | 55.2 | 4 | 2 | 13 | 233 | 3.66E+09 | **0.74** | 4 | 13.6 | 290 | 33.5 | 4.3 | Regulation of nucleobase, nucleoside, nucleotide and nucleic acid metabolism | Cytoplasm;Endoplasmic reticulum;Nucleus;Plasma membrane;Cytosol;Nuclear membrane | MHC class I receptor activity;MHC class II receptor activity | Nucleus |

| Gene Symbol | RefSeq_Accession | Entrez Gene ID | Description | Coverage (%) | Proteins | Unique Peptides | # Peptides | PSM | Area | SCC-R /SCC-S | SCC-R /SCC-S (Count) | SCC-R /SCC-S (Variability [%]) | # AAs | MW [kDa] | calc. pI | Biological Process | Localization | Molecular Function | Primary Localization |
|---|---|---|---|---|---|---|---|---|---|---|---|---|---|---|---|---|---|---|---|
| **SPATS2** | NP_075559.2 | 65244 | **spermatogenesis-associated serine-rich protein 2** | 11.0 | 1 | 3 | 3 | 5 | 2.72E+07 | **0.74** | 3 | 17.6 | 545 | 59.5 | 8.9 | Biological_process unknown | Cytoplasm | Molecular function unknown | Cytoplasm |
| **AKAP1** | NP_001229832.1 | 8165 | **A-kinase anchor protein 1, mitochondrial precursor** | 1.4 | 1 | 1 | 1 | 2 | 1.56E+07 | **0.74** | 1 | - | 903 | 97.3 | 4.9 | Cell growth and/or maintenance | Cytoplasm;Nucleus;Endoplasmic reticulum;Mitochondrion | Cytoskeletal anchoring activity | Cytoplasm |
| **EEA1** | NP_003557.2 | 8411 | **early endosome antigen 1** | 27.8 | 1 | 23 | 23 | 45 | 2.43E+08 | **0.74** | 19 | 25.1 | 1411 | 162.4 | 5.7 | Transport | Endosome;Cytoplasm;Nucleus;Cytosol;Centrosome | Auxiliary transport protein activity | Endosome |
| **NUP133** | NP_060700.2 | 55746 | **nuclear pore complex protein Nup133** | 19.6 | 1 | 13 | 13 | 24 | 3.19E+08 | **0.74** | 14 | 34.7 | 1156 | 128.9 | 5.1 | Transport | Nucleus;Cytoplasm;Kinetochore;Microtubule | Transporter activity | Nucleus |
| **RPL7** | NP_000962.2 | 6129 | **60S ribosomal protein L7** | 52.8 | 1 | 17 | 17 | 190 | 3.68E+09 | **0.74** | 43 | 16.5 | 248 | 29.2 | 10.7 | Protein metabolism | Cytoplasm;Nucleus;Ribosome;Nucleolus;Cytosol;Mitochondrion | Structural constituent of ribosome | Ribosome |
| **C14orf169** | NP_078920.2 | 79697 | **bifunctional lysine-specific demethylase and histidyl-hydroxylase NO66** | 4.4 | 1 | 1 | 1 | 1 | 1.24E+07 | **0.74** | 1 | - | 641 | 71 | 6.5 | Regulation of nucleobase, nucleoside, nucleotide and nucleic acid metabolism | Nucleolus;Nucleus | Transcription regulator activity | Nucleolus |
| **CAPRIN1** | NP_976240.1 | 4076 | **caprin-1 isoform 2** | 23.8 | 2 | 12 | 12 | 59 | 9.46E+08 | **0.74** | 17 | 21.9 | 694 | 76.8 | 5.1 | Transport | Plasma membrane | Molecular function unknown | Plasma membrane |
| **RNF40** | NP_001193962.1 | 9810 | **E3 ubiquitin-protein ligase BRE1B isoform 2** | 14.3 | 3 | 7 | 8 | 12 | 1.55E+08 | **0.74** | 9 | 42.7 | 1000 | 113.5 | 6.4 | Protein metabolism | Cytoplasm;Integral to membrane;Nucleus | Ubiquitin-specific protease activity | Nucleus |
| **RPL36** | NP_056229.2 | 25873 | **60S ribosomal protein L36** | 32.4 | 1 | 7 | 7 | 64 | 1.78E+09 | **0.74** | 13 | 11.1 | 105 | 12.2 | 11.6 | Protein metabolism | Ribosome;Nucleolus | Structural constituent of ribosome | Ribosome |
| **ABHD14B** | NP_001241682.1 | 84836 | **alpha/beta hydrolase domain-containing protein 14B isoform 3** | 12.8 | 2 | 1 | 1 | 2 | 3.38E+08 | **0.74** | 1 | - | 172 | 18 | 6.7 | Biological_process unknown | - | Molecular function unknown | - |
| **DAP3** | NP_001186780.1 | 7818 | **28S ribosomal protein S29, mitochondrial isoform 3** | 20.7 | 3 | 5 | 5 | 14 | 1.36E+08 | **0.74** | 5 | 18 | 357 | 41 | 9.0 | Apoptosis | Mitochondrion;Cytoplasm;Nucleus | Ribonucleoprotein | Mitochondrion |
| **RAB6A** | NP_002860.2 | 5870 | **ras-related protein Rab-6A isoform a** | 51.9 | 18 | 7 | 10 | 38 | 5.00E+08 | **0.74** | 6 | 19.1 | 208 | 23.5 | 5.5 | Cell communication;Signal transduction | Plasma membrane;Cytoplasmic vesicle;Golgi apparatus | GTPase activity | Golgi apparatus |
| **RBBP9** | NP_006597.2 | 10741 | **putative hydrolase RBBP9** | 55.9 | 1 | 5 | 5 | 11 | 1.39E+08 | **0.74** | 4 | 23.3 | 186 | 21 | 6.2 | Biological_process unknown;Cell differentiation | Nucleus | Molecular function unknown | Nucleus |
| **SLC12A4** | NP_001139436.1 | 6560 | **solute carrier family 12 member 4 isoform e** | 1.5 | 5 | 1 | 1 | 1 | 5.37E+07 | **0.74** | 1 | - | 1054 | 117 | 6.8 | Transport | Plasma membrane | Ion transporter activity | Plasma membrane |
| **ZFYVE1** | NP_001268663.1 | 53349 | **zinc finger FYVE domain-containing protein 1 isoform 3** | 1.6 | 2 | 1 | 1 | 1 | 2.34E+07 | **0.74** | 1 | - | 763 | 85.5 | 7.4 | Biological_process unknown | Cytoplasmic vesicle;Endoplasmic reticulum;Golgi apparatus;Cytosol;Perinuclear region | Lipid binding | Golgi apparatus |
| **POLE2** | NP_001184259.1 | 5427 | **DNA polymerase epsilon subunit 2 isoform 2** | 4.0 | 3 | 1 | 1 | 1 | 6.14E+07 | **0.73** | 1 | - | 501 | 56.4 | 6.1 | Regulation of nucleobase, nucleoside, nucleotide and nucleic acid metabolism | Nucleus | DNA-directed DNA polymerase activity | Nucleus |
| **TCF25** | NP_055787.1 | 22980 | **transcription factor 25** | 8.6 | 1 | 3 | 3 | 3 | 3.50E+07 | **0.73** | 1 | - | 676 | 76.6 | 6.4 | Biological_process unknown | - | Molecular function unknown | - |
| **ZNF207** | NP_001027464.1 | 7756 | **BUB3-interacting and GLEBS motif-containing protein ZNF207 isoform b** | 13.8 | 3 | 5 | 5 | 12 | 2.70E+08 | **0.73** | 6 | 15.8 | 463 | 49.7 | 9.1 | Regulation of nucleobase, nucleoside, nucleotide and nucleic acid metabolism;Transcription | Nucleus;Nucleolus | DNA binding;Transcription factor activity | Nucleus |
| **DDX39A** | NP_005795.2 | 10212 | **ATP-dependent RNA helicase DDX39A** | 55.0 | 1 | 11 | 20 | 79 | 2.11E+09 | **0.73** | 12 | 21.1 | 427 | 49.1 | 5.7 | Regulation of nucleobase, nucleoside, nucleotide and nucleic acid metabolism | Nucleus;Nucleolus | Helicase activity | Nucleus |
| **FBXL18** | NP_079239.3 | 80028 | **F-box/LRR-repeat protein 18** | 21.3 | 1 | 9 | 9 | 19 | 1.32E+08 | **0.73** | 9 | 16.3 | 718 | 78.9 | 8.3 | Biological_process unknown | - | Molecular function unknown | - |
| **IK** | NP_006074.2 | 3550 | **protein Red** | 36.8 | 1 | 11 | 11 | 19 | 2.06E+08 | **0.73** | 8 | 11.7 | 557 | 65.6 | 6.6 | Immune response | Nucleus | Molecular function unknown | Nucleus |
| **RPL10** | NP_006004.2 | 6134 | **60S ribosomal protein L10 isoform a** | 42.1 | 4 | 12 | 12 | 94 | 2.01E+09 | **0.73** | 19 | 11.5 | 214 | 24.6 | 10.1 | Protein metabolism | Cytoplasm;Endoplasmic reticulum;Ribosome;Nucleolus | Structural constituent of ribosome | Ribosome |
| **RPS20** | NP_001014.1 | 6224 | **40S ribosomal protein S20 isoform 2** | 34.5 | 2 | 5 | 5 | 25 | 4.41E+09 | **0.73** | 8 | 6.3 | 119 | 13.4 | 9.9 | Protein metabolism | Ribosome | Structural constituent of ribosome | Ribosome |
| **RPS23** | NP_001016.1 | 6228 | **40S ribosomal protein S23** | 43.4 | 1 | 6 | 6 | 137 | 4.81E+08 | **0.73** | 16 | 20.3 | 143 | 15.8 | 10.5 | Protein metabolism | Ribosome;Nucleolus | Structural constituent of ribosome | Ribosome |
| **AATF** | NP_036270.1 | 26574 | **protein AATF** | 9.5 | 1 | 3 | 3 | 4 | 4.56E+07 | **0.73** | 1 | - | 560 | 63.1 | 4.9 | Apoptosis | Nucleus;Cytoplasm;Nucleolus | Transcription factor activity | Nucleus |
| **STAG2** | NP_001269347.1 | 10735 | **cohesin subunit SA-2 isoform b** | 13.8 | 2 | 8 | 10 | 19 | 1.73E+08 | **0.73** | 11 | 34.4 | 1231 | 141.2 | 5.4 | Cell growth and/or maintenance | Nucleus;Chromosome;Cytoplasm | Molecular function unknown | Nucleus |
| **TMBIM1** | NP_071435.2 | 64114 | **protein lifeguard 3** | 4.5 | 1 | 1 | 1 | 3 | 2.02E+08 | **0.73** | 1 | - | 311 | 34.6 | 7.7 | Biological_process unknown | - | Molecular function unknown | - |
| **WDR70** | NP_060504.1 | 55100 | **WD repeat-containing protein 70** | 11.9 | 1 | 5 | 5 | 6 | 1.04E+08 | **0.73** | 4 | 12.3 | 654 | 73.2 | 6.3 | Biological_process unknown | Cytoplasm | Molecular function unknown | Cytoplasm |
| **GATAD2B** | NP_065750.1 | 57459 | **transcriptional repressor p66-beta** | 24.3 | 1 | 8 | 9 | 29 | 1.12E+08 | **0.73** | 4 | 16.4 | 593 | 65.2 | 9.7 | Regulation of nucleobase, nucleoside, nucleotide and nucleic acid metabolism | Nucleus | Transcription regulator activity | Nucleus |
| **KDM5C** | NP_001140174.1 | 8242 | **lysine-specific demethylase 5C isoform 2** | 1.3 | 5 | 1 | 2 | 2 | 9.96E+07 | **0.73** | 1 | - | 1379 | 155 | 5.6 | Regulation of nucleobase, nucleoside, nucleotide and nucleic acid metabolism | Nucleus;Cytoplasm | DNA binding | Nucleus;Cytoplasm |
| **ERGIC3** | NP_057050.1 | 51614 | **endoplasmic reticulum-Golgi intermediate compartment protein 3 isoform b** | 6.8 | 2 | 1 | 1 | 3 | 1.89E+07 | **0.73** | 1 | - | 383 | 43.2 | 6.1 | Biological_process unknown | Golgi apparatus | Molecular function unknown | Golgi apparatus |
| **KIF5B** | NP_004512.1 | 3799 | **kinesin-1 heavy chain** | 46.3 | 2 | 36 | 36 | 106 | 1.02E+09 | **0.73** | 39 | 14.7 | 963 | 109.6 | 6.5 | Cell growth and/or maintenance | Mitochondrion;Microtubule | Motor activity | Mitochondrion |
| **PRKAG1** | NP_001193639.1 | 5571 | **5'-AMP-activated protein kinase subunit gamma-1 isoform 4** | 10.7 | 3 | 4 | 4 | 13 | 6.65E+07 | **0.73** | 7 | 5.2 | 299 | 34.1 | 8.5 | Signal transduction | - | Molecular function unknown | - |
| **CNN3** | NP_001830.1 | 1266 | **calponin-3 isoform 1** | 45.6 | 3 | 8 | 8 | 35 | 6.19E+08 | **0.73** | 9 | 30.1 | 329 | 36.4 | 6.1 | Cell growth and/or maintenance | Cytoplasm;Nucleus | Cytoskeletal protein binding | Cytoplasm |
| **HDLBP** | NP_005327.1 | 3069 | **vigilin isoform a** | 38.7 | 2 | 35 | 35 | 123 | 1.18E+09 | **0.73** | 42 | 17.7 | 1268 | 141.4 | 6.9 | Transport | Nucleus;Plasma membrane;Extracellular;Cytoplasm;Ribosome;Nucleolus | Transporter activity | Cytoplasm;Plasma membrane |
| **RPL23A** | NP_000975.2 | 6147 | **60S ribosomal protein L23a** | 52.6 | 1 | 12 | 12 | 95 | 2.28E+09 | **0.73** | 23 | 17.6 | 156 | 17.7 | 10.5 | Regulation of nucleobase, nucleoside, nucleotide and nucleic acid metabolism | Cytoplasm;Nucleolus;Mitochondrion | RNA binding | Cytoplasm |
| **RRP8** | NP_056139.1 | 23378 | **ribosomal RNA-processing protein 8** | 4.2 | 1 | 1 | 1 | 4 | 4.09E+07 | **0.73** | 2 | 89.2 | 456 | 50.7 | 9.4 | Metabolism;Energy pathways | Nucleolus | Methyltransferase activity | Nucleolus |

| Gene Symbol | RefSeq_Accession | Entrez Gene ID | Description | Coverage (%) | Proteins | Unique Peptides | # Peptides | PSM | Area | SCC-R /SCC-S | SCC-R /SCC-S (Count) | SCC-R /SCC-S (Variability [%]) | # AAs | MW [kDa] | calc. pI | Biological Process | Localization | Molecular Function | Primary Localization |
|---|---|---|---|---|---|---|---|---|---|---|---|---|---|---|---|---|---|---|---|
| **EIF4A1** | NP_001407.1 | 1973 | **eukaryotic initiation factor 4A-I isoform 1** | 77.8 | 2 | 21 | 32 | 399 | 9.08E+09 | **0.73** | 64 | 13.9 | 406 | 46.1 | 5.5 | Protein metabolism | Nucleus;Cytoplasm;Ribosome | Translation regulator activity | Cytoplasm |
| **TALDO1** | NP_006746.1 | 6888 | **transaldolase** | 54.3 | 1 | 22 | 22 | 71 | 1.16E+09 | **0.73** | 30 | 18.8 | 337 | 37.5 | 6.8 | Metabolism;Energy pathways | Nucleus | Transferase activity, transferring aldehyde or ketonic groups | Nucleus |
| **U2SURP** | NP_001073884.1 | 23350 | **U2 snRNP-associated SURP motif-containing protein** | 18.6 | 1 | 10 | 10 | 22 | 1.70E+08 | **0.73** | 10 | 12.5 | 1029 | 118.2 | 8.5 | Biological_process unknown | - | Molecular function unknown | - |
| **CPNE3** | NP_003900.1 | 8895 | **copine-3** | 36.3 | 8 | 13 | 14 | 47 | 9.51E+08 | **0.73** | 17 | 23.4 | 537 | 60.1 | 5.9 | Transport | Cytoplasm | Transporter activity | Cytoplasm |
| **DDRGK1** | NP_076424.1 | 65992 | **DDRGK domain-containing protein 1 precursor** | 10.2 | 1 | 2 | 2 | 3 | 1.53E+08 | **0.73** | 2 | 19.2 | 314 | 35.6 | 5.1 | Biological_process unknown | - | Molecular function unknown | - |
| **HSPA1L** | NP_005518.3 | 3305 | **heat shock 70 kDa protein 1-like** | 30.3 | 1 | 1 | 17 | 278 | 1.02E+10 | **0.73** | 6 | 12.9 | 641 | 70.3 | 6.0 | Protein metabolism | Cytoplasm;Nucleus;Mitochondrion | Heat shock protein activity | Cytoplasm |
| **PFAS** | NP_036525.1 | 5198 | **phosphoribosylformylglycinamidine synthase** | 38.3 | 1 | 28 | 28 | 67 | 5.15E+08 | **0.73** | 27 | 29.8 | 1338 | 144.6 | 5.8 | Metabolism;Energy pathways | Cytoplasm | Ligase activity | Cytoplasm |
| **RPL10A** | NP_009035.3 | 4736 | **60S ribosomal protein L10a** | 40.6 | 1 | 10 | 10 | 140 | 2.05E+09 | **0.73** | 39 | 14.1 | 217 | 24.8 | 9.9 | Protein metabolism | Ribosome;Nucleolus | Structural constituent of ribosome | Ribosome |
| **RPS18** | NP_072045.1 | 6222 | **40S ribosomal protein S18** | 50.0 | 1 | 11 | 11 | 80 | 2.12E+09 | **0.73** | 30 | 10.7 | 152 | 17.7 | 11.0 | Protein metabolism | Ribosome;Nucleolus | Structural constituent of ribosome | Ribosome |
| **SAP30** | NP_003855.1 | 8819 | **histone deacetylase complex subunit SAP30** | 8.2 | 1 | 1 | 1 | 2 | 1.28E+08 | **0.73** | 1 | - | 220 | 23.3 | 9.2 | Regulation of nucleobase, nucleoside, nucleotide and nucleic acid metabolism | Nucleus | Protein binding | Nucleus |
| **TMED10** | NP_006818.3 | 10972 | **transmembrane emp24 domain-containing protein 10 precursor** | 43.4 | 1 | 11 | 11 | 51 | 2.06E+09 | **0.73** | 14 | 21.7 | 219 | 25 | 7.4 | Transport | Integral to membrane;Endoplasmic reticulum;Golgi apparatus;Microsome;Plasma membrane;Zymogen granule;Mitochondrion | Transporter activity | Integral to membrane |
| **TMX1** | NP_110382.3 | 81542 | **thioredoxin-related transmembrane protein 1 precursor** | 20.7 | 1 | 5 | 5 | 22 | 2.02E+08 | **0.73** | 5 | 40.3 | 280 | 31.8 | 5.0 | Metabolism;Energy pathways | Endoplasmic reticulum;Microsome;Plasma membrane;Mitochondrion | Oxidoreductase activity | Endoplasmic reticulum |
| **MTOR** | NP_004949.1 | 2475 | **serine/threonine-protein kinase mTOR** | 5.7 | 1 | 8 | 8 | 11 | 5.45E+07 | **0.73** | 7 | 25.9 | 2549 | 288.7 | 7.2 | Cell communication;Signal transduction | Plasma membrane;Cytoplasm;Nucleus;Mitochondrion;Cytosol | Kinase activity | Nucleus |
| **H2AFZ** | NP_002097.1 | 3015 | **histone H2A.Z** | 56.3 | 6 | 5 | 7 | 225 | 6.75E+09 | **0.72** | 23 | 9.7 | 128 | 13.5 | 10.6 | Regulation of nucleobase, nucleoside, nucleotide and nucleic acid metabolism | Nucleus | DNA binding | Nucleus |
| **MRPL44** | NP_075066.1 | 65080 | **39S ribosomal protein L44, mitochondrial** | 16.0 | 1 | 4 | 4 | 13 | 6.71E+07 | **0.72** | 5 | 52.8 | 332 | 37.5 | 8.4 | Protein metabolism | Mitochondrion | Structural constituent of ribosome | Mitochondrion |
| **TOR1B** | NP_055321.1 | 27348 | **torsin-1B precursor** | 4.2 | 1 | 1 | 1 | 2 | 1.50E+08 | **0.72** | 1 | - | 336 | 38 | 8.5 | Protein metabolism | Endoplasmic reticulum;Perinuclear region | Chaperone activity | Perinuclear region |
| **ABCB7** | NP_001258626.1 | 22 | **ATP-binding cassette sub-family B member 7, mitochondrial isoform 3** | 3.4 | 5 | 1 | 1 | 1 | 2.16E+07 | **0.72** | 1 | - | 712 | 78.1 | 9.1 | Transport | Mitochondrion | Transporter activity | Mitochondrion |
| **PCBP1** | NP_006187.2 | 5093 | **poly(rC)-binding protein 1** | 81.7 | 3 | 12 | 16 | 216 | 2.38E+09 | **0.72** | 43 | 18.5 | 356 | 37.5 | 7.1 | Regulation of nucleobase, nucleoside, nucleotide and nucleic acid metabolism | Nucleus;Cytoplasm;Nucleolus;Mitochondrion | RNA binding | Nucleus |
| **PRPF3** | NP_004689.1 | 9129 | **U4/U6 small nuclear ribonucleoprotein Prp3** | 17.6 | 1 | 7 | 7 | 17 | 1.44E+08 | **0.72** | 7 | 31.9 | 683 | 77.5 | 9.5 | Regulation of nucleobase, nucleoside, nucleotide and nucleic acid metabolism | Nucleus;Nucleolus;Ribosome;Cytoplasm | RNA binding | Nucleus |
| **RPAP3** | NP_001139548.1 | 79657 | **RNA polymerase II-associated protein 3 isoform 3** | 10.7 | 3 | 3 | 3 | 5 | 2.80E+07 | **0.72** | 3 | 123 | 506 | 57.1 | 7.7 | Apoptosis | Cytoplasm | Molecular function unknown | Cytoplasm |
| **RPL30** | NP_000980.1 | 6156 | **60S ribosomal protein L30** | 64.4 | 1 | 7 | 7 | 97 | 1.60E+09 | **0.72** | 20 | 7.3 | 115 | 12.8 | 9.6 | Protein metabolism | - | Structural constituent of ribosome | - |
| **SAR1A** | NP_001136120.1 | 56681 | **GTP-binding protein SAR1a** | 71.2 | 1 | 8 | 10 | 52 | 1.22E+09 | **0.72** | 13 | 17 | 198 | 22.4 | 6.7 | Cell communication;Signal transduction | Endoplasmic reticulum | GTPase activity | Endoplasmic reticulum |
| **AHSA1** | NP_036243.1 | 10598 | **activator of 90 kDa heat shock protein ATPase homolog 1** | 60.1 | 1 | 17 | 17 | 80 | 1.45E+09 | **0.72** | 17 | 15.6 | 338 | 38.3 | 5.5 | Biological_process unknown | Cytoplasm;Endoplasmic reticulum | Molecular function unknown | Cytoplasm |
| **MCM3** | NP_002379.3 | 4172 | **DNA replication licensing factor MCM3 isoform 1** | 40.6 | 2 | 27 | 27 | 100 | 1.40E+09 | **0.72** | 32 | 7.9 | 853 | 95.8 | 5.8 | Regulation of nucleobase, nucleoside, nucleotide and nucleic acid metabolism | Nucleus;Nucleolus;Chromosome;Mitochondrion | DNA binding | Nucleus |
| **RPS3A** | NP_000997.1 | 6189 | **40S ribosomal protein S3a isoform 1** | 56.4 | 2 | 26 | 26 | 205 | 2.60E+09 | **0.72** | 48 | 13.2 | 264 | 29.9 | 9.7 | Protein metabolism | Cytoplasm;Nucleus;Nucleolus;Cytosol;Ribosome | Structural constituent of ribosome | Cytoplasm |
| **TPT1** | NP_003286.1 | 7178 | **translationally-controlled tumor protein isoform 2** | 77.3 | 4 | 13 | 13 | 61 | 1.36E+09 | **0.72** | 18 | 17.1 | 172 | 19.6 | 4.9 | Cell growth and/or maintenance | Nucleus;Extracellular;Cytoplasm;Cytoskeleton;Nucleolus;Plasma membrane;Mitochondrion | Calcium ion binding | Nucleus |
| **DDX56** | NP_001244118.1 | 54606 | **probable ATP-dependent RNA helicase DDX56 isoform 2** | 31.0 | 2 | 8 | 9 | 20 | 2.16E+08 | **0.72** | 4 | 7.4 | 507 | 57.2 | 9.2 | Regulation of nucleobase, nucleoside, nucleotide and nucleic acid metabolism | Cytoplasm;Nucleus;Nucleolus | Helicase activity | Nucleolus |
| **GTF2I** | NP_001509.3 | 2969 | **general transcription factor II-I isoform 4** | 45.4 | 6 | 31 | 31 | 65 | 3.56E+08 | **0.72** | 20 | 33.4 | 957 | 107.9 | 7.9 | Regulation of nucleobase, nucleoside, nucleotide and nucleic acid metabolism | Cytoplasm;Nucleolus;Nucleus | Transcription factor activity | Nucleus |
| **MDC1** | NP_055456.2 | 9656 | **mediator of DNA damage checkpoint protein 1** | 3.8 | 1 | 4 | 4 | 5 | 1.05E+08 | **0.72** | 5 | 37.2 | 2089 | 226.5 | 5.5 | Regulation of nucleobase, nucleoside, nucleotide and nucleic acid metabolism | Nucleus;Cytoplasm | DNA repair protein | Nucleus;Cytoplasm |
| **RPS3** | NP_001243731.1 | 6188 | **40S ribosomal protein S3 isoform 1** | 71.6 | 3 | 22 | 22 | 191 | 4.08E+09 | **0.72** | 52 | 12.3 | 243 | 26.7 | 9.7 | Protein metabolism | Cytoplasm;Nucleus;Ribosome | Structural constituent of ribosome | Cytoplasm |
| **CDKN2AIPNL** | NP_542387.1 | 91368 | **CDKN2AIP N-terminal-like protein** | 15.5 | 1 | 1 | 1 | 3 | 8.02E+06 | **0.72** | 1 | - | 116 | 13.2 | 5.0 | Biological_process unknown | - | Molecular function unknown | - |
| **SCCPDH** | NP_057086.2 | 51097 | **saccharopine dehydrogenase-like oxidoreductase** | 22.6 | 1 | 5 | 5 | 10 | 2.15E+08 | **0.72** | 6 | 21.2 | 429 | 47.1 | 9.1 | Biological_process unknown | Mitochondrion | Molecular function unknown | Mitochondrion |
| **SRP54** | NP_003127.1 | 6729 | **signal recognition particle 54 kDa protein isoform 1** | 33.5 | 2 | 13 | 13 | 38 | 3.44E+08 | **0.72** | 14 | 9.5 | 504 | 55.7 | 8.8 | Protein metabolism | Cytoplasm | RNA binding | Cytoplasm |
| **DCTN5** | NP_001185940.1 | 84516 | **dynactin subunit 5 isoform 2** | 46.4 | 3 | 3 | 3 | 6 | 1.63E+08 | **0.72** | 2 | 38.8 | 84 | 9.7 | 9.1 | Biological_process unknown | Centrosome;Kinetochore;Microtubule | Molecular function unknown | - |
| **RABEPK** | NP_001167624.1 | 10244 | **rab9 effector protein with kelch motifs isoform b** | 11.2 | 2 | 2 | 2 | 5 | 4.33E+07 | **0.72** | 1 | - | 321 | 34.8 | 6.0 | Transport | Cytoplasm;Endosome | Transporter activity | Cytoplasm |
| **SRP19** | NP_003126.1 | 6728 | **signal recognition particle 19 kDa protein isoform 1** | 48.6 | 4 | 6 | 6 | 10 | 7.38E+07 | **0.72** | 6 | 17.5 | 144 | 16.1 | 9.9 | Protein metabolism | Nucleolus;Cytoplasm;Nucleus | RNA binding | Cytoplasm |
| **STXBP2** | NP_001120868.1 | 6813 | **syntaxin-binding protein 2 isoform b** | 31.2 | 3 | 11 | 11 | 29 | 2.04E+08 | **0.72** | 10 | 53.9 | 590 | 66.1 | 6.6 | Cell communication;Signal transduction | Plasma membrane;Cytoplasm;Secretory granule | Molecular function unknown | Cytoplasm |

Supplementary Table S4. List of all proteins identified in SCC-S and SCC-R cells using SequestHT and Mascot search algorithms and their gene ontology annotation

| Gene Symbol | RefSeq_Accession | Entrez Gene ID | Description | Coverage (%) | Proteins | Unique Peptides | # Peptides | PSM | Area | SCC-R /SCC-S | SCC-R /SCC-S (Count) | SCC-R /SCC-S (Variability [%]) | # AAs | MW [kDa] | calc. pI | Biological Process | Localization | Molecular Function | Primary Localization |
|---|---|---|---|---|---|---|---|---|---|---|---|---|---|---|---|---|---|---|---|
| ARHGAP1 | NP_004299.1 | 392 | rho GTPase-activating protein 1 | 44.4 | 1 | 11 | 11 | 26 | 3.72E+08 | 0.72 | 9 | 14.6 | 439 | 50.4 | 6.3 | Cell communication;Signal transduction | Cytoplasm;Extracellular | GTPase activator activity | Cytoplasm |
| GSTZ1 | NP_001504.2 | 2954 | maleylacetoacetate isomerase isoform 3 | 24.2 | 3 | 4 | 4 | 5 | 9.66E+07 | 0.72 | 2 | 3.4 | 161 | 17.9 | 6.0 | Metabolism;Energy pathways | - | Glutathione transferase activity | - |
| PVR | NP_001129241.1 | 5817 | poliovirus receptor isoform gamma precursor | 6.0 | 4 | 1 | 1 | 1 | 5.42E+06 | 0.72 | 1 | - | 364 | 39.3 | 6.2 | Cell communication;Signal transduction | Plasma membrane;Extracellular | Receptor activity | Plasma membrane |
| BAG5 | NP_001015048.1 | 9529 | BAG family molecular chaperone regulator 5 isoform b | 32.4 | 2 | 8 | 8 | 14 | 9.44E+07 | 0.72 | 5 | 12.3 | 447 | 51.2 | 6.1 | Biological_process unknown | - | Molecular function unknown | - |
| FAM175B | NP_115558.3 | 23172 | BRISC complex subunit Abro1 | 23.1 | 1 | 5 | 5 | 8 | 2.85E+07 | 0.72 | 3 | 10.1 | 415 | 46.9 | 6.2 | Biological_process unknown | Cytoplasm | Molecular function unknown | Cytoplasm |
| RPL22 | NP_000974.1 | 6146 | 60S ribosomal protein L22 proprotein | 62.5 | 1 | 8 | 8 | 69 | 4.12E+09 | 0.72 | 11 | 14 | 128 | 14.8 | 9.2 | Protein metabolism | Ribosome;Cytoplasm;Nucleolus;Nucleus | Structural constituent of ribosome | Ribosome |
| SRSF8 | NP_115285.1 | 10929 | serine/arginine-rich splicing factor 8 | 8.9 | 1 | 1 | 2 | 6 | 6.41E+08 | 0.72 | 1 | - | 282 | 32.3 | 11.7 | Regulation of nucleobase, nucleoside, nucleotide and nucleic acid metabolism | Nucleus | Transcription regulator activity | Nucleus |
| AKR7A2 | NP_003680.2 | 8574 | aflatoxin B1 aldehyde reductase member 2 | 22.8 | 1 | 4 | 4 | 15 | 1.65E+08 | 0.72 | 3 | 8 | 359 | 39.6 | 7.2 | Metabolism;Energy pathways | Cytoplasm | Catalytic activity | Cytoplasm |
| FKBP4 | NP_002005.1 | 2288 | peptidyl-prolyl cis-trans isomerase FKBP4 | 73.0 | 1 | 33 | 33 | 147 | 1.08E+09 | 0.72 | 44 | 26 | 459 | 51.8 | 5.4 | Metabolism;Energy pathways | Cytoplasm;Nucleus | Isomerase activity | Cytoplasm |
| MAP4K5 | NP_942089.1 | 11183 | mitogen-activated protein kinase kinase kinase kinase 5 | 6.5 | 1 | 3 | 3 | 3 | 1.01E+08 | 0.72 | 3 | 19 | 846 | 95 | 7.7 | Cell communication;Signal transduction | Cytoplasm | Protein serine/threonine kinase activity | Cytoplasm |
| PSMD11 | NP_001257411.1 | 5717 | 26S proteasome non-ATPase regulatory subunit 11 | 41.2 | 1 | 14 | 14 | 43 | 4.90E+08 | 0.72 | 16 | 11.2 | 422 | 47.4 | 6.5 | Protein metabolism | Cytoplasm;Nucleus | Ubiquitin-specific protease activity | Cytoplasm |
| PUM2 | NP_001269720.1 | 23369 | pumilio homolog 2 isoform 4 | 11.0 | 4 | 4 | 5 | 7 | 2.27E+08 | 0.72 | 3 | 1.2 | 929 | 99.6 | 7.3 | Protein metabolism | Cytoplasm;Nucleus | RNA binding | Cytoplasm |
| RHOT2 | NP_620124.1 | 89941 | mitochondrial Rho GTPase 2 | 10.7 | 1 | 3 | 3 | 6 | 3.48E+07 | 0.72 | 1 | - | 618 | 68.1 | 5.9 | Cell communication;Signal transduction | Mitochondrion | GTPase activity | Mitochondrion |
| ABCE1 | NP_001035809.1 | 6059 | ATP-binding cassette sub-family E member 1 | 42.1 | 1 | 17 | 17 | 45 | 8.57E+08 | 0.71 | 15 | 16.3 | 599 | 67.3 | 8.3 | Transport | Mitochondrion;Cytoplasm | Transporter activity | Cytoplasm |
| BBX | NP_064620.2 | 56987 | HMG box transcription factor BBX isoform 2 | 2.2 | 2 | 1 | 1 | 2 | 1.90E+07 | 0.71 | 1 | - | 911 | 101.5 | 8.5 | Regulation of nucleobase, nucleoside, nucleotide and nucleic acid metabolism | Nucleus | Transcription factor activity | Nucleus |
| GSTK1 | NP_057001.1 | 373156 | glutathione S-transferase kappa 1 isoform a | 36.3 | 4 | 7 | 7 | 20 | 3.05E+08 | 0.71 | 8 | 19.7 | 226 | 25.5 | 8.4 | Metabolism | Peroxisome;Mitochondrion | Glutathione transferase activity | Peroxisome;Mitochondrion |
| ICT1 | NP_001536.1 | 3396 | peptidyl-tRNA hydrolase ICT1, mitochondrial precursor | 5.8 | 1 | 1 | 1 | 1 | 9.34E+07 | 0.71 | 1 | - | 206 | 23.6 | 10.1 | Immune response | - | Molecular function unknown | - |
| TTC12 | NP_060338.3 | 54970 | tetratricopeptide repeat protein 12 | 1.0 | 1 | 1 | 1 | 1 | 2.33E+08 | 0.71 | 1 | - | 705 | 78.7 | 5.7 | Biological_process unknown | - | Molecular function unknown | - |
| ANAPC7 | NP_001131136.1 | 51434 | anaphase-promoting complex subunit 7 isoform b | 10.1 | 2 | 3 | 3 | 5 | 5.67E+07 | 0.71 | 3 | 22.3 | 537 | 60 | 8.4 | Cell communication;Signal transduction | Cytoplasm;Microtubule;Centrosome | Protein binding | Cytoplasm |
| NFRKB | NP_001137307.1 | 4798 | nuclear factor related to kappa-B-binding protein isoform 1 | 1.0 | 2 | 1 | 1 | 1 | 1.06E+07 | 0.71 | 1 | - | 1299 | 138.9 | 9.3 | Regulation of nucleobase, nucleoside, nucleotide and nucleic acid metabolism | Nucleus;Chromosome | Transcription factor activity | Nucleus |
| POLA2 | NP_002680.2 | 23649 | DNA polymerase alpha subunit B | 11.5 | 1 | 4 | 4 | 5 | 4.84E+07 | 0.71 | 3 | 11.5 | 598 | 65.9 | 5.2 | Regulation of nucleobase, nucleoside, nucleotide and nucleic acid metabolism | Nucleus | DNA-directed DNA polymerase activity | Nucleus |
| RPS24 | NP_148982.1 | 6229 | 40S ribosomal protein S24 isoform a | 36.9 | 5 | 6 | 6 | 41 | 1.12E+09 | 0.71 | 10 | 20.9 | 130 | 15.1 | 10.9 | Protein metabolism | Ribosome | Structural constituent of ribosome | Ribosome |
| XPO5 | NP_065801.1 | 57510 | exportin-5 | 27.6 | 1 | 22 | 22 | 51 | 2.55E+08 | 0.71 | 17 | 32.5 | 1204 | 136.2 | 5.8 | Transport | Nucleus | Transporter activity | Nucleus |
| CCNB2 | NP_004692.1 | 9133 | G2/mitotic-specific cyclin-B2 | 2.8 | 1 | 1 | 1 | 1 | 8.20E+06 | 0.71 | 1 | - | 398 | 45.3 | 8.9 | Cell communication;Signal transduction | Golgi apparatus;Cytoplasm;Extracellular | Protein binding | Golgi apparatus |
| RFC5 | NP_853556.2 | 5985 | replication factor C subunit 5 isoform 2 | 33.9 | 4 | 6 | 6 | 17 | 1.14E+08 | 0.71 | 5 | 39.1 | 319 | 36.1 | 7.0 | DNA replication | Nucleolus;Nucleus;Mitochondrion | DNA binding | Nucleus |
| RPL36A | NP_066357.2 | 6173 | 60S ribosomal protein L36a isoform a | 17.6 | 5 | 3 | 3 | 4 | 3.97E+07 | 0.71 | 4 | 4.7 | 142 | 16.4 | 10.4 | Protein metabolism | Cytoplasm | Structural constituent of ribosome | Cytoplasm |
| EIF2S2 | NP_003899.2 | 8894 | eukaryotic translation initiation factor 2 subunit 2 | 49.6 | 1 | 18 | 18 | 58 | 7.95E+08 | 0.71 | 20 | 19.2 | 333 | 38.4 | 5.8 | Protein metabolism | Cytoplasm;Nucleolus | Translation regulator activity | Cytoplasm |
| MRI1 | NP_115661.1 | 84245 | methylthioribose-1-phosphate isomerase isoform 2 | 20.8 | 2 | 4 | 4 | 8 | 8.44E+07 | 0.71 | 5 | 20.8 | 322 | 34.5 | 5.7 | Protein metabolism;Biological_process unknown | - | Translation regulator activity;Molecular function unknown | - |
| RPL11 | NP_000966.2 | 6135 | 60S ribosomal protein L11 isoform 1 | 27.0 | 1 | 2 | 6 | 62 | 2.34E+09 | 0.71 | 1 | - | 178 | 20.2 | 9.6 | Protein metabolism | Ribosome;Nucleolus;Nucleoplasm;Mitochondrion | Structural constituent of ribosome | Ribosome |
| SEC61B | NP_006799.1 | 10952 | protein transport protein Sec61 subunit beta | 37.5 | 1 | 3 | 3 | 7 | 1.88E+08 | 0.71 | 4 | 24.6 | 96 | 10 | 11.6 | Transport | Endoplasmic reticulum | Auxiliary transport protein activity | Endoplasmic reticulum |
| ALDH1B1 | NP_000683.3 | 219 | aldehyde dehydrogenase X, mitochondrial precursor | 29.2 | 1 | 11 | 11 | 32 | 1.18E+08 | 0.71 | 10 | 18.2 | 517 | 57.2 | 7.0 | Metabolism;Energy pathways | Mitochondrion | Catalytic activity | Mitochondrion |
| ALG6 | NP_037471.2 | 29929 | dolichyl pyrophosphate Man9GlcNAc2 alpha-1,3-glucosyltransferase precursor | 1.8 | 1 | 1 | 1 | 1 | 3.04E+07 | 0.71 | 1 | - | 507 | 58.1 | 8.5 | Metabolism;Energy pathways | Endoplasmic reticulum;Golgi apparatus | Transferase activity | Endoplasmic reticulum |
| EIF2S3 | NP_001406.1 | 1968 | eukaryotic translation initiation factor 2 subunit 3 | 51.7 | 1 | 20 | 20 | 93 | 1.03E+09 | 0.71 | 24 | 14.7 | 472 | 51.1 | 8.4 | Protein metabolism | Cytoplasm;Nucleolus | Translation regulator activity | Cytoplasm |
| FSTL1 | NP_009016.1 | 11167 | follistatin-related protein 1 precursor | 10.4 | 1 | 2 | 2 | 5 | 4.24E+07 | 0.71 | 3 | 34 | 308 | 35 | 5.5 | Protein metabolism | Extracellular | Protease inhibitor activity | Extracellular |
| ITSN1 | NP_001001132.1 | 6453 | intersectin-1 isoform ITSN-s | 1.6 | 2 | 1 | 1 | 1 | 9.78E+07 | 0.71 | 1 | - | 1220 | 137.6 | 7.3 | Signal transduction;Vesicle-mediated transport | Axon;Dendrite;Golgi apparatus;Cytoplasm;Endosome;Cytosol;Cytoplasmic vesicle;Plasma membrane;Caveola;Cell | Receptor signaling complex scaffold activity | Cytoplasm;Nucleus |
| MRPS14 | NP_071383.1 | 63931 | 28S ribosomal protein S14, mitochondrial | 21.9 | 1 | 2 | 2 | 4 | 1.60E+08 | 0.71 | 1 | - | 128 | 15.1 | 11.4 | Protein metabolism | Mitochondrion | Structural constituent of ribosome | Mitochondrion |
| SEPT8 | NP_055961.1 | 23176 | septin-8 isoform b | 40.1 | 5 | 10 | 14 | 31 | 6.12E+08 | 0.71 | 12 | 13.8 | 429 | 49.8 | 6.1 | Cell communication;Signal transduction;Regulation of exocytosis;Vesicle-mediated transport | Cytoplasmic vesicle;Cytoplasm;Cell surface | GTPase activity | Cytoplasm |

| Gene Symbol | RefSeq_Accession | Entrez Gene ID | Description | Coverage (%) | Proteins | Unique Peptides | # Peptides | PSM | Area | SCC-R /SCC-S | SCC-R /SCC-S (Count) | SCC-R /SCC-S (Variability [%]) | # AAs | MW [kDa] | calc. pI | Biological Process | Localization | Molecular Function | Primary Localization |
|---|---|---|---|---|---|---|---|---|---|---|---|---|---|---|---|---|---|---|---|
| CBL | NP_005179.2 | 867 | E3 ubiquitin-protein ligase CBL | 5.3 | 2 | 3 | 3 | 3 | 1.42E+07 | 0.71 | 1 | - | 906 | 99.6 | 6.5 | Cell communication;Signal transduction;Protein metabolism | Cytosol;Endosome;Cytoplasm;Plasma membrane;Nucleus;Cell projection | Ubiquitin-specific protease activity | Cytoplasm |
| RPL13A | NP_036555.1 | 23521 | 60S ribosomal protein L13a isoform 1 | 38.9 | 2 | 10 | 10 | 57 | 1.72E+09 | 0.71 | 12 | 13.4 | 203 | 23.6 | 10.9 | Protein metabolism | Ribosome;Nucleolus;Cytoplasm | Structural constituent of ribosome | Ribosome |
| GPKOW | NP_056513.2 | 27238 | G patch domain and KOW motifs-containing protein | 33.4 | 1 | 9 | 9 | 19 | 2.13E+08 | 0.71 | 5 | 33.2 | 476 | 52.2 | 6.2 | Biological_process unknown | Cytoplasm | Molecular function unknown | Cytoplasm |
| IMP4 | NP_219484.1 | 92856 | U3 small nucleolar ribonucleoprotein protein IMP4 | 13.4 | 1 | 2 | 2 | 4 | 6.16E+07 | 0.71 | 2 | 0.6 | 291 | 33.7 | 9.5 | Regulation of nucleobase, nucleoside, nucleotide and nucleic acid metabolism | Nucleolus;Cytoplasm | Ribonucleoprotein | Nucleolus |
| NOL6 | NP_075068.2 | 65083 | nucleolar protein 6 alpha isoform | 10.3 | 2 | 6 | 6 | 18 | 1.16E+08 | 0.71 | 5 | 35.5 | 1146 | 127.5 | 7.6 | Regulation of nucleobase, nucleoside, nucleotide and nucleic acid metabolism | Nucleolus;Nucleus | RNA binding | Nucleolus |
| ADSS | NP_001117.2 | 159 | adenylosuccinate synthetase isozyme 2 | 29.8 | 1 | 8 | 9 | 27 | 3.51E+08 | 0.71 | 8 | 28.9 | 456 | 50.1 | 6.6 | Metabolism;Energy pathways | Cytoplasm | Ligase activity | Cytoplasm |
| RBMX2 | NP_057108.2 | 51634 | RNA-binding motif protein, X-linked 2 | 6.8 | 1 | 2 | 2 | 4 | 1.29E+07 | 0.71 | 2 | 3 | 322 | 37.3 | 9.8 | Regulation of nucleobase, nucleoside, nucleotide and nucleic acid metabolism | Nucleus;Cytoplasm | RNA binding | Nucleus;Cytoplasm |
| RPS25 | NP_001019.1 | 6230 | 40S ribosomal protein S25 | 36.8 | 1 | 5 | 5 | 45 | 1.34E+09 | 0.71 | 7 | 4.7 | 125 | 13.7 | 10.1 | Protein metabolism | Nucleus;Ribosome;Nucleolus | Structural constituent of ribosome | Ribosome |
| TUBAL3 | NP_001165335.1 | 79861 | tubulin alpha chain-like 3 isoform 2 | 9.4 | 2 | 1 | 5 | 30 | 8.87E+08 | 0.71 | 1 | - | 406 | 45.5 | 6.3 | Cell growth and/or maintenance | - | Structural molecule activity | - |
| ATAD3A | NP_001164006.1 | 55210 | ATPase family AAA domain-containing protein 3A isoform 2 | 22.4 | 4 | 8 | 8 | 16 | 8.35E+07 | 0.70 | 5 | 12.1 | 586 | 66.2 | 9.3 | Metabolism;Energy pathways | - | ATPase activity | - |
| C4orf27 | NP_060337.2 | 54969 | UPF0609 protein C4orf27 | 7.2 | 1 | 2 | 2 | 2 | 6.58E+07 | 0.70 | 2 | 0.9 | 346 | 39.4 | 6.8 | Biological_process unknown | - | Molecular function unknown | - |
| NVL | NP_001230076.1 | 4931 | nuclear valosin-containing protein-like isoform 4 | 6.0 | 4 | 4 | 4 | 6 | 4.82E+07 | 0.70 | 2 | 1.4 | 765 | 85 | 6.2 | Metabolism;Energy pathways | Nucleoplasm | ATPase activity | Nucleoplasm |
| WLS | NP_001180263.1 | 79971 | protein wntless homolog isoform 3 | 4.0 | 3 | 1 | 1 | 1 | 1.43E+08 | 0.70 | 1 | - | 450 | 51.5 | 7.3 | Cell communication;Signal transduction | - | Molecular function unknown | - |
| CROT | NP_066974.2 | 54677 | peroxisomal carnitine O-octanoyltransferase isoform 2 | 2.8 | 2 | 1 | 1 | 1 | 3.57E+07 | 0.70 | 1 | - | 612 | 70.1 | 7.1 | Metabolism;Energy pathways | Peroxisome | Acyltransferase activity | Peroxisome |
| GLE1 | NP_001490.1 | 2733 | nucleoporin GLE1 isoform 2 | 6.2 | 2 | 2 | 2 | 2 | 6.13E+06 | 0.70 | 1 | - | 659 | 75.4 | 7.0 | Transport | Nuclear membrane;Nucleoplasm;Cytoplasm | Transporter activity | Nuclear membrane |
| DENR | NP_003668.2 | 8562 | density-regulated protein | 35.9 | 1 | 5 | 5 | 18 | 9.72E+08 | 0.70 | 7 | 9.4 | 198 | 22.1 | 5.3 | Cell communication;Signal transduction | Nucleus | Molecular function unknown | Nucleus |
| HIST1H2AE | NP_066390.1 | 3012 | histone H2A type 1-B/E | 37.7 | 4 | 2 | 7 | 909 | 9.70E+09 | 0.70 | 3 | 12.1 | 130 | 14.1 | 11.1 | Regulation of nucleobase, nucleoside, nucleotide and nucleic acid metabolism | Nucleus | DNA binding | Nucleus |
| NCAPG2 | NP_060230.5 | 54892 | condensin-2 complex subunit G2 isoform a | 3.0 | 2 | 2 | 2 | 3 | 5.36E+06 | 0.70 | 2 | 8.7 | 1143 | 130.9 | 6.9 | Cell growth and/or maintenance | - | Molecular function unknown | - |
| POLR1A | NP_056240.2 | 25885 | DNA-directed RNA polymerase I subunit RPA1 | 5.6 | 2 | 6 | 6 | 8 | 2.45E+08 | 0.70 | 4 | 3.5 | 1720 | 194.7 | 7.0 | Regulation of nucleobase, nucleoside, nucleotide and nucleic acid metabolism | Nucleolus | DNA-directed RNA polymerase activity | Nucleolus |
| PYCR1 | NP_001269209.1 | 5831 | pyrroline-5-carboxylate reductase 1, mitochondrial isoform 1 | 25.4 | 4 | 4 | 5 | 8 | 1.36E+08 | 0.70 | 3 | 2.3 | 319 | 33.3 | 7.6 | Metabolism;Energy pathways | Cytoplasm;Mitochondrion;Nucleus;Microsome | Oxidoreductase activity | Cytoplasm |
| TCERG1 | NP_001035095.1 | 10915 | transcription elongation regulator 1 isoform 2 | 5.7 | 2 | 5 | 5 | 10 | 1.19E+08 | 0.70 | 4 | 19.5 | 1077 | 121.6 | 8.5 | Regulation of nucleobase, nucleoside, nucleotide and nucleic acid metabolism | Nucleus;Nucleolus | Transcription factor activity | Nucleus |
| WRAP53 | NP_001137464.1 | 55135 | telomerase Cajal body protein 1 | 11.5 | 1 | 3 | 3 | 4 | 9.75E+07 | 0.70 | 3 | 6.4 | 548 | 59.3 | 4.6 | Biological_process unknown | Nucleus | Molecular function unknown | Nucleus |
| CPSF3L | NP_001243392.1 | 54973 | integrator complex subunit 11 isoform 5 | 5.8 | 5 | 2 | 2 | 2 | 3.17E+07 | 0.70 | 2 | 37.3 | 499 | 56 | 8.3 | Regulation of cell cycle | Nucleus;Cytoplasm | Endonuclease activity | Nucleus |
| G6PD | NP_001035810.1 | 2539 | glucose-6-phosphate 1-dehydrogenase isoform b | 56.3 | 2 | 25 | 25 | 110 | 1.20E+09 | 0.70 | 41 | 17.6 | 515 | 59.2 | 6.8 | Metabolism;Energy pathways | Endoplasmic reticulum;Cytoplasm | Catalytic activity | Cytoplasm |
| IL18 | NP_001553.1 | 3606 | interleukin-18 isoform 1 proprotein | 64.3 | 2 | 8 | 8 | 24 | 3.19E+08 | 0.70 | 7 | 23.9 | 193 | 22.3 | 4.7 | Immune response | Extracellular;Cytoplasm | Cytokine activity | Extracellular |
| COPS7A | NP_001157566.1 | 50813 | COP9 signalosome complex subunit 7a | 16.7 | 1 | 2 | 2 | 3 | 2.82E+07 | 0.70 | 2 | 16.7 | 275 | 30.3 | 8.2 | Regulation of nucleobase, nucleoside, nucleotide and nucleic acid metabolism | Nucleus | Transcription regulator activity | Nucleus |
| TBL3 | NP_006444.2 | 10607 | transducin beta-like protein 3 | 24.6 | 1 | 11 | 11 | 17 | 2.00E+08 | 0.70 | 7 | 48.4 | 808 | 89 | 6.9 | Biological_process unknown | Nucleolus | Molecular function unknown | Nucleolus |
| NOL7 | NP_057251.2 | 51406 | nucleolar protein 7 | 7.0 | 1 | 2 | 2 | 5 | 3.99E+07 | 0.70 | 2 | 6.2 | 257 | 29.4 | 9.7 | Biological_process unknown | Nucleolus | Molecular function unknown | Nucleolus |
| PTPN23 | NP_056281.1 | 25930 | tyrosine-protein phosphatase non-receptor type 23 | 4.9 | 1 | 5 | 5 | 9 | 4.53E+07 | 0.70 | 3 | 53.9 | 1636 | 178.9 | 6.9 | Cell communication;Signal transduction | Cytoplasm;Nucleus | Protein tyrosine phosphatase activity | Cytoplasm |
| RFC2 | NP_852136.1 | 5982 | replication factor C subunit 2 isoform 1 | 45.5 | 5 | 9 | 9 | 22 | 1.92E+08 | 0.70 | 11 | 34.6 | 354 | 39.1 | 6.4 | DNA replication | Nucleolus;Nucleus | DNA binding | Nucleolus |
| PYGL | NP_001157412.1 | 5836 | glycogen phosphorylase, liver form isoform 2 | 54.1 | 2 | 32 | 37 | 103 | 5.42E+08 | 0.70 | 32 | 26.5 | 813 | 93.1 | 7.3 | Metabolism;Energy pathways | - | Phosphorylase activity | - |
| SRP14 | NP_003125.3 | 6727 | signal recognition particle 14 kDa protein | 43.4 | 1 | 6 | 6 | 18 | 1.89E+08 | 0.70 | 10 | 12.8 | 136 | 14.6 | 10.0 | Protein metabolism | Cytoplasm;Nucleolus;Cytosol | RNA binding | Cytoplasm |
| SLC1A5 | NP_005619.1 | 6510 | neutral amino acid transporter B(0) isoform 1 | 17.0 | 3 | 5 | 5 | 18 | 2.60E+08 | 0.70 | 8 | 62.7 | 541 | 56.6 | 5.5 | Transport | Plasma membrane | Auxiliary transport protein activity | Plasma membrane |
| BSG | NP_940991.1 | 682 | basigin isoform 2 | 39.8 | 4 | 7 | 7 | 22 | 1.05E+09 | 0.70 | 11 | 38.4 | 269 | 29.2 | 5.6 | Cell communication;Signal transduction | Plasma membrane;Nucleus;Cytoplasm | Receptor activity | Plasma membrane;Nucleus;Cytoplasm |
| GTPBP4 | NP_036473.2 | 23560 | nucleolar GTP-binding protein 1 | 22.2 | 2 | 11 | 11 | 31 | 5.09E+08 | 0.70 | 7 | 13 | 634 | 73.9 | 9.5 | Cell communication;Signal transduction | Nucleus;Nucleolus;Ribosome | GTPase activity | Nucleus |
| PCBP2 | NP_005007.2 | 5094 | poly(rC)-binding protein 2 isoform a | 75.4 | 5 | 1 | 14 | 205 | 3.68E+09 | 0.70 | 2 | 20.8 | 366 | 38.6 | 6.8 | Regulation of nucleobase, nucleoside, nucleotide and nucleic acid metabolism | Nucleus;Cytoplasm | RNA binding;DNA binding | Nucleus |
| SMC1A | NP_006297.2 | 8243 | structural maintenance of chromosomes protein 1A isoform 1 | 28.2 | 2 | 25 | 25 | 55 | 5.86E+08 | 0.70 | 22 | 18.6 | 1233 | 143.1 | 7.6 | Cell communication;Signal transduction | Nucleus;Nucleolus;Chromosome;Cytoplasm;Kinetochore | Chromatin binding | Nucleus |

| Gene Symbol | RefSeq_Accession | Entrez Gene ID | Description | Coverage (%) | Proteins | Unique Peptides | # Peptides | PSM | Area | SCC-R /SCC-S | SCC-R /SCC-S (Count) | SCC-R /SCC-S (Variability [%]) | # AAs | MW [kDa] | calc. pI | Biological Process | Localization | Molecular Function | Primary Localization |
|---|---|---|---|---|---|---|---|---|---|---|---|---|---|---|---|---|---|---|---|
| **ARL1** | NP_001168.1 | 400 | **ADP-ribosylation factor-like protein 1** | 49.2 | 1 | 7 | 7 | 29 | 2.54E+08 | **0.69** | 14 | 18.5 | 181 | 20.4 | 5.7 | Cell communication;Signal transduction | Golgi apparatus | GTPase activity | Golgi apparatus |
| **CDK12** | NP_055898.1 | 51755 | **cyclin-dependent kinase 12 isoform 2** | 6.8 | 2 | 5 | 6 | 10 | 1.08E+08 | **0.69** | 6 | 38.2 | 1481 | 163.1 | 9.5 | Cell communication;Signal transduction | Nucleus | Protein serine/threonine kinase activity | Nucleus |
| **CYBA** | NP_000092.2 | 1535 | **cytochrome b-245 light chain** | 15.9 | 1 | 1 | 1 | 3 | 1.84E+08 | **0.69** | 1 | - | 195 | 21 | 9.5 | Metabolism;Energy pathways | Plasma membrane;Lysosome | Catalytic activity | Plasma membrane |
| **EEF1G** | NP_001395.1 | 1937 | **elongation factor 1-gamma** | 73.5 | 1 | 27 | 27 | 270 | 1.09E+10 | **0.69** | 64 | 26 | 437 | 50.1 | 6.7 | Protein metabolism | Nucleolus;Plasma membrane;Cytoplasm;Mitochondrion | Translation regulator activity | Cytoplasm |
| **GAPVD1** | NP_001269609.1 | 26130 | **GTPase-activating protein and VPS9 domain-containing protein 1 isoform 2** | 15.4 | 3 | 13 | 13 | 25 | 9.45E+07 | **0.69** | 7 | 48.5 | 1460 | 162.8 | 5.2 | Biological_process unknown | - | Molecular function unknown | - |
| **SSR4** | NP_006271.1 | 6748 | **translocon-associated protein subunit delta isoform 2 precursor** | 24.9 | 3 | 3 | 3 | 11 | 5.62E+08 | **0.69** | 5 | 21.7 | 173 | 19 | 6.2 | Transport | Endoplasmic reticulum;Microsome;Mitochondrion | Auxiliary transport protein activity | Endoplasmic reticulum |
| **RPL13** | NP_150254.1 | 6137 | **60S ribosomal protein L13 isoform 1** | 48.8 | 3 | 13 | 13 | 57 | 8.94E+08 | **0.69** | 20 | 17.6 | 211 | 24.2 | 11.7 | Protein metabolism | Ribosome;Nucleolus;Mitochondrion;Nucleus | RNA binding | Ribosome |
| **UBAC1** | NP_057256.2 | 10422 | **ubiquitin-associated domain-containing protein 1** | 4.7 | 1 | 1 | 1 | 1 | 6.17E+07 | **0.69** | 1 | - | 405 | 45.3 | 4.9 | Cell growth and/or maintenance | Cytoplasm;Integral to membrane;Nucleus;Cytoskeleton | Molecular function unknown | Cytoplasm |
| **GUK1** | NP_001152862.1 | 2987 | **guanylate kinase isoform a** | 11.0 | 3 | 2 | 2 | 3 | 1.74E+07 | **0.69** | 1 | - | 218 | 23.8 | 7.5 | Cell communication;Signal transduction | Mitochondrion;Cytoplasm | Catalytic activity | Cytoplasm |
| **DNAJC11** | NP_060668.2 | 55735 | **dnaJ homolog subfamily C member 11** | 6.1 | 1 | 3 | 3 | 3 | 7.26E+07 | **0.69** | 3 | 22.2 | 559 | 63.2 | 8.4 | Biological_process unknown | - | Molecular function unknown | - |
| **MTCH2** | NP_055157.1 | 23788 | **mitochondrial carrier homolog 2** | 38.0 | 1 | 7 | 7 | 36 | 4.99E+08 | **0.69** | 12 | 16.3 | 303 | 33.3 | 8.0 | Cell communication;Signal transduction | Mitochondrion | Molecular function unknown | Mitochondrion |
| **ATP6V1D** | NP_057078.1 | 51382 | **V-type proton ATPase subunit D** | 31.6 | 1 | 4 | 4 | 7 | 9.14E+07 | **0.69** | 1 | - | 247 | 28.2 | 9.4 | Metabolism;Energy pathways | Integral to membrane;Cytosol | ATPase activity | Integral to membrane |
| **CD46** | NP_758860.1 | 4179 | **membrane cofactor protein isoform 14 precursor** | 6.6 | 8 | 2 | 2 | 3 | 1.47E+08 | **0.69** | 3 | 17.7 | 349 | 39.3 | 5.3 | Cell communication;Signal transduction | Plasma membrane | Receptor activity | Plasma membrane |
| **ERP29** | NP_006808.1 | 10961 | **endoplasmic reticulum resident protein 29 isoform 1 precursor** | 59.4 | 2 | 14 | 14 | 106 | 1.82E+09 | **0.69** | 25 | 14.6 | 261 | 29 | 7.3 | Protein folding | Endoplasmic reticulum;Mitochondrion | Chaperone activity | Endoplasmic reticulum |
| **FH** | NP_000134.2 | 2271 | **fumarate hydratase, mitochondrial** | 50.6 | 1 | 15 | 15 | 93 | 1.02E+09 | **0.69** | 26 | 14.1 | 510 | 54.6 | 8.8 | Metabolism;Energy pathways | Mitochondrion;Cytoplasm | Catalytic activity | Mitochondrion |
| **GEMIN4** | NP_056536.2 | 50628 | **gem-associated protein 4** | 7.7 | 1 | 5 | 5 | 12 | 2.31E+08 | **0.69** | 7 | 65.5 | 1058 | 120 | 6.0 | Reproductive behavior | Nucleus;Cytoplasm;Nucleolus;Cytosol | Molecular function unknown | Nucleus |
| **GINS2** | NP_057179.1 | 51659 | **DNA replication complex GINS protein PSF2** | 13.5 | 1 | 1 | 1 | 1 | 5.26E+07 | **0.69** | 1 | - | 185 | 21.4 | 5.4 | Biological_process unknown | - | Molecular function unknown | - |
| **TFB1M** | NP_057104.2 | 51106 | **dimethyladenosine transferase 1, mitochondrial** | 11.0 | 1 | 2 | 2 | 3 | 3.96E+07 | **0.69** | 2 | 95.9 | 346 | 39.5 | 9.3 | Regulation of nucleobase, nucleoside, nucleotide and nucleic acid metabolism | Mitochondrion | Transcription factor activity | Mitochondrion |
| **MRPL53** | NP_444278.1 | 116540 | **39S ribosomal protein L53, mitochondrial** | 16.1 | 1 | 1 | 1 | 7 | 4.33E+07 | **0.69** | 3 | 26.6 | 112 | 12.1 | 8.8 | Protein metabolism | Mitochondrion | Structural constituent of ribosome | Mitochondrion |
| **PRRC1** | NP_570721.1 | 133619 | **protein PRRC1 isoform 2** | 28.8 | 2 | 6 | 6 | 10 | 3.34E+08 | **0.69** | 8 | 9 | 445 | 46.7 | 5.8 | Biological_process unknown | Golgi apparatus;Cytoplasm | Molecular function unknown | Golgi apparatus |
| **RPS27** | NP_001021.1 | 6232 | **40S ribosomal protein S27** | 40.5 | 1 | 3 | 5 | 39 | 1.01E+09 | **0.69** | 7 | 13.8 | 84 | 9.5 | 9.5 | Regulation of nucleobase, nucleoside, nucleotide and nucleic acid metabolism | Nucleus;Cytoplasm;Extracellular;Nucleolus | Structural constituent of ribosome | Cytoplasm |
| **ADA** | NP_000013.2 | 100 | **adenosine deaminase** | 2.2 | 1 | 1 | 1 | 1 | 2.50E+07 | **0.69** | 1 | - | 363 | 40.7 | 6.0 | Metabolism;Energy pathways | Plasma membrane;Cytoplasm | Deaminase activity | Plasma membrane |
| **ADAM10** | NP_001101.1 | 102 | **disintegrin and metalloproteinase domain-containing protein 10 precursor** | 6.2 | 1 | 3 | 3 | 6 | 5.54E+07 | **0.69** | 1 | - | 748 | 84.1 | 7.8 | Protein metabolism | Plasma membrane;Golgi apparatus;Nucleus | Metallopeptidase activity | Plasma membrane |
| **ATP5O** | NP_001688.1 | 539 | **ATP synthase subunit O, mitochondrial precursor** | 52.6 | 1 | 10 | 10 | 36 | 4.02E+08 | **0.69** | 11 | 16.3 | 213 | 23.3 | 10.0 | Metabolism;Energy pathways | Mitochondrion | Transporter activity | Mitochondrion |
| **ATRX** | NP_000480.3 | 546 | **transcriptional regulator ATRX isoform 1** | 7.4 | 2 | 10 | 10 | 15 | 7.27E+07 | **0.69** | 8 | 16.3 | 2492 | 282.4 | 6.6 | Regulation of nucleobase, nucleoside, nucleotide and nucleic acid metabolism | Nucleus;Nucleolus;Cytoplasm | Transcription regulator activity | Nucleus;Cytoplasm |
| **CYB5R1** | NP_057327.2 | 51706 | **NADH-cytochrome b5 reductase 1** | 12.5 | 1 | 3 | 3 | 4 | 6.64E+07 | **0.69** | 2 | 2.9 | 305 | 34.1 | 9.4 | Metabolism;Energy pathways | Endoplasmic reticulum;Cytoplasm | Catalytic activity | Endoplasmic reticulum |
| **HNRNPA1** | NP_002127.1 | 3178 | **heterogeneous nuclear ribonucleoprotein A1 isoform a** | 76.3 | 2 | 1 | 34 | 428 | 6.04E+09 | **0.69** | 3 | 3.5 | 320 | 34.2 | 9.2 | Regulation of nucleobase, nucleoside, nucleotide and nucleic acid metabolism | Nucleus;Cytoplasm;Nucleolus;Mitochondrion | RNA binding | Nucleus |
| **RPL21** | NP_000973.2 | 6144 | **60S ribosomal protein L21** | 43.1 | 1 | 8 | 8 | 47 | 1.51E+09 | **0.69** | 13 | 6.6 | 160 | 18.6 | 10.5 | Protein metabolism | Ribosome;Nucleolus | Structural constituent of ribosome | Ribosome |
| **YME1L1** | NP_001240795.1 | 10730 | **ATP-dependent zinc metalloprotease YME1L1 isoform 4** | 14.8 | 3 | 7 | 7 | 9 | 2.85E+07 | **0.69** | 4 | 3.6 | 683 | 75.9 | 8.6 | Protein metabolism | Mitochondrion;Cytoplasm | Metallopeptidase activity | Mitochondrion |
| **RPS15A** | NP_001010.2 | 6210 | **40S ribosomal protein S15a** | 73.9 | 1 | 12 | 12 | 84 | 2.26E+09 | **0.69** | 21 | 19.6 | 130 | 14.8 | 10.1 | Protein metabolism | Ribosome | Structural constituent of ribosome | Ribosome |
| **LIG1** | NP_001275992.1 | 3978 | **DNA ligase 1 isoform 2** | 5.4 | 3 | 2 | 2 | 3 | 1.51E+08 | **0.69** | 2 | 36.8 | 888 | 98.2 | 5.5 | Regulation of nucleobase, nucleoside, nucleotide and nucleic acid metabolism | Nucleus | DNA ligase activity | Nucleus |
| **LRBA** | NP_001186211.2 | 987 | **lipopolysaccharide-responsive and beige-like anchor protein isoform 1** | 4.6 | 2 | 8 | 8 | 12 | 1.56E+08 | **0.69** | 6 | 12.7 | 2851 | 317.5 | 5.6 | Cell communication;Signal transduction | Golgi apparatus;Lysosome;Plasma membrane;Endoplasmic reticulum;Cytoplasmic vesicle | Cytoskeletal anchoring activity | Golgi apparatus |
| **OSTF1** | NP_036515.4 | 26578 | **osteoclast-stimulating factor 1** | 33.2 | 1 | 4 | 4 | 10 | 1.16E+08 | **0.69** | 4 | 30.7 | 214 | 23.8 | 5.7 | Cell communication;Signal transduction | Nucleus;Cytoskeleton;Cytoplasm | Receptor signaling complex scaffold activity | Nucleus |
| **C20orf27** | NP_001245358.1 | 54976 | **UPF0687 protein C20orf27 isoform 2** | 29.9 | 2 | 3 | 3 | 5 | 6.77E+07 | **0.68** | 1 | - | 174 | 19.3 | 6.8 | Biological_process unknown | - | Molecular function unknown | - |
| **ECHS1** | NP_004083.3 | 1892 | **enoyl-CoA hydratase, mitochondrial** | 64.1 | 1 | 12 | 12 | 116 | 2.07E+09 | **0.68** | 32 | 14.5 | 290 | 31.4 | 8.1 | Metabolism;Energy pathways | Mitochondrion | Catalytic activity | Mitochondrion |
| **FAM120A** | NP_001273651.1 | 23196 | **constitutive coactivator of PPAR-gamma-like protein 1 isoform b** | 36.2 | 4 | 23 | 23 | 56 | 2.78E+08 | **0.68** | 20 | 58.6 | 1117 | 121.7 | 8.9 | Biological_process unknown | Nucleus | Molecular function unknown | Nucleus |
| **MECP2** | NP_004983.1 | 4204 | **methyl-CpG-binding protein 2 isoform 1** | 2.9 | 2 | 1 | 1 | 3 | 1.62E+08 | **0.68** | 2 | 7 | 486 | 52.4 | 10.0 | Regulation of nucleobase, nucleoside, nucleotide and nucleic acid metabolism | Nucleus | DNA binding | Nucleus |

**Supplementary Table S4. List of all proteins identified in SCC-S and SCC-R cells using SequestHT and Mascot search algorithms and their gene ontology annotation**

| Gene Symbol | RefSeq_Accession | Entrez Gene ID | Description | Coverage (%) | Proteins | Unique Peptides | # Peptides | PSM | Area | SCC-R /SCC-S | SCC-R /SCC-S (Count) | SCC-R /SCC-S (Variability [%]) | # AAs | MW [kDa] | calc. pI | Biological Process | Localization | Molecular Function | Primary Localization |
|---|---|---|---|---|---|---|---|---|---|---|---|---|---|---|---|---|---|---|---|
| **CCDC6** | NP_005427.2 | 8030 | **coiled-coil domain-containing protein 6** | 26.0 | 1 | 8 | 8 | 21 | 2.11E+08 | **0.68** | 4 | 4 | 474 | 53.3 | 7.3 | Apoptosis | Cytoplasm;Nucleus | Structural constituent of cytoskeleton | Cytoplasm |
| **PPIL2** | NP_055152.1 | 23759 | **peptidyl-prolyl cis-trans isomerase-like 2 isoform a** | 9.4 | 2 | 2 | 3 | 3 | 3.36E+07 | **0.68** | 1 | - | 520 | 58.8 | 8.8 | Protein metabolism | Nucleus;Cytoplasm;Plasma membrane | Chaperone activity | Nucleus |
| **HNRNPA1** | NP_112420.1 | 3178 | **heterogeneous nuclear ribonucleoprotein A1 isoform b** | 62.4 | 2 | 1 | 34 | 421 | 6.04E+09 | **0.68** | 1 | - | 372 | 38.7 | 9.1 | Regulation of nucleobase, nucleoside, nucleotide and nucleic acid metabolism | Nucleus;Cytoplasm;Nucleolus;Mitochondrion | RNA binding | Nucleus |
| **CDYL** | NP_001137443.1 | 9425 | **chromodomain Y-like protein isoform d** | 7.3 | 2 | 2 | 2 | 4 | 2.73E+07 | **0.68** | 2 | 19.5 | 412 | 45.1 | 8.7 | Regulation of nucleobase, nucleoside, nucleotide and nucleic acid metabolism | Nucleus | Transcription regulator activity | Nucleus |
| **MTX3** | NP_001010891.4 | 345778 | **metaxin-3 isoform 2** | 3.2 | 2 | 1 | 1 | 1 | 5.69E+07 | **0.68** | 1 | - | 248 | 27.9 | 7.8 | Biological_process unknown | - | Molecular function unknown | - |
| **RAN** | NP_006316.1 | 5901 | **GTP-binding nuclear protein Ran** | 81.5 | 1 | 16 | 16 | 224 | 1.31E+10 | **0.68** | 44 | 14.4 | 216 | 24.4 | 7.5 | Cell communication;Signal transduction | Nucleus;Cytoplasm;Mitochondrion | GTPase activity | Nucleus |
| **BAZ2A** | NP_038477.2 | 11176 | **bromodomain adjacent to zinc finger domain protein 2A** | 4.4 | 1 | 5 | 5 | 7 | 6.75E+07 | **0.68** | 4 | 12.1 | 1905 | 211.1 | 6.6 | Regulation of nucleobase, nucleoside, nucleotide and nucleic acid metabolism | Nucleolus;Nucleus | Transcription regulator activity | Nucleolus |
| **RPL32** | NP_001007074.1 | 6161 | **60S ribosomal protein L32** | 33.3 | 1 | 5 | 5 | 48 | 1.33E+09 | **0.68** | 12 | 18 | 135 | 15.8 | 11.3 | Protein metabolism | Ribosome | Structural constituent of ribosome | Ribosome |
| **ARF5** | NP_001653.1 | 381 | **ADP-ribosylation factor 5** | 68.9 | 6 | 7 | 14 | 70 | 4.93E+08 | **0.68** | 6 | 5.3 | 180 | 20.5 | 6.8 | Cell communication;Signal transduction | Plasma membrane;Cytoplasm;Golgi apparatus | GTPase activity | Cytoplasm |
| **DDX10** | NP_004389.2 | 1662 | **probable ATP-dependent RNA helicase DDX10** | 6.1 | 1 | 3 | 3 | 4 | 8.91E+07 | **0.68** | 3 | 15.6 | 875 | 100.8 | 8.6 | Regulation of nucleobase, nucleoside, nucleotide and nucleic acid metabolism | Nucleus;Nucleolus | RNA binding | Nucleus |
| **MRPL4** | NP_057040.2 | 51073 | **39S ribosomal protein L4, mitochondrial isoform a** | 27.0 | 2 | 5 | 5 | 15 | 1.61E+08 | **0.68** | 5 | 38.5 | 311 | 34.9 | 9.7 | Protein metabolism | Mitochondrion | Structural constituent of ribosome | Mitochondrion |
| **NISCH** | NP_001263223.1 | 11188 | **nischarin isoform 3** | 3.3 | 3 | 1 | 1 | 1 | 5.35E+07 | **0.68** | 1 | - | 515 | 56.8 | 5.8 | Cell growth and/or maintenance | Plasma membrane;Cytoplasm | Receptor activity | Plasma membrane |
| **PMPCB** | NP_004270.2 | 9512 | **mitochondrial-processing peptidase subunit beta precursor** | 22.9 | 1 | 8 | 8 | 12 | 1.42E+08 | **0.68** | 4 | 33.8 | 489 | 54.3 | 6.8 | Protein metabolism | Mitochondrion | Peptidase activity | Mitochondrion |
| **ZNF346** | NP_036411.1 | 23567 | **zinc finger protein 346** | 16.3 | 1 | 3 | 3 | 4 | 2.76E+07 | **0.68** | 2 | 37.4 | 294 | 32.9 | 9.1 | Regulation of nucleobase, nucleoside, nucleotide and nucleic acid metabolism | Nucleolus;Nucleus;Cytoplasm | RNA binding | Nucleolus |
| **CEP97** | NP_078824.2 | 79598 | **centrosomal protein of 97 kDa** | 1.5 | 1 | 1 | 1 | 2 | 2.98E+07 | **0.68** | 1 | - | 865 | 96.9 | 5.0 | Biological_process unknown | Centriole | Molecular function unknown | Centriole |
| **GCLM** | NP_002052.1 | 2730 | **glutamate--cysteine ligase regulatory subunit** | 27.4 | 1 | 5 | 5 | 12 | 9.11E+07 | **0.68** | 3 | 18.8 | 274 | 30.7 | 6.0 | Metabolism;Energy pathways | - | Ligase activity | - |
| **NEMF** | NP_004704.2 | 9147 | **nuclear export mediator factor NEMF** | 3.8 | 1 | 2 | 2 | 3 | 2.71E+07 | **0.68** | 1 | - | 1076 | 122.9 | 6.4 | Biological_process unknown | Nucleus;Cytoplasm | Molecular function unknown | Nucleus |
| **RPL17** | NP_000976.1 | 6139 | **60S ribosomal protein L17 isoform a** | 50.0 | 4 | 11 | 11 | 89 | 3.37E+09 | **0.68** | 19 | 23.6 | 184 | 21.4 | 10.2 | Protein metabolism | Ribosome;Nucleolus | Structural constituent of ribosome | Ribosome |
| **RPP30** | NP_006404.1 | 10556 | **ribonuclease P protein subunit p30 isoform b** | 30.2 | 2 | 5 | 5 | 7 | 3.84E+07 | **0.68** | 3 | 2.6 | 268 | 29.3 | 8.9 | Regulation of nucleobase, nucleoside, nucleotide and nucleic acid metabolism | Nucleus;Nucleolus;Cytoplasm | Ribonuclease activity | Nucleus |
| **RPS11** | NP_001006.1 | 6205 | **40S ribosomal protein S11** | 62.7 | 1 | 11 | 11 | 91 | 3.19E+09 | **0.68** | 23 | 8.5 | 158 | 18.4 | 10.3 | Protein metabolism | Ribosome;Nucleolus | Structural constituent of ribosome | Ribosome |
| **MMGT1** | NP_775741.1 | 93380 | **membrane magnesium transporter 1 precursor** | 18.3 | 1 | 1 | 1 | 9 | 1.23E+09 | **0.68** | 4 | 6 | 131 | 14.7 | 9.2 | Biological_process unknown | - | Molecular function unknown | - |
| **NOL12** | NP_077289.1 | 79159 | **nucleolar protein 12** | 10.3 | 1 | 1 | 1 | 3 | 2.06E+07 | **0.68** | 2 | 97 | 213 | 24.6 | 10.2 | Biological_process unknown | Nucleus | Molecular function unknown | Nucleus |
| **PSMB6** | NP_002789.1 | 5694 | **proteasome subunit beta type-6 isoform 1 proprotein** | 42.7 | 2 | 7 | 7 | 38 | 9.83E+08 | **0.68** | 9 | 32.5 | 239 | 25.3 | 4.9 | Protein metabolism | Cytoplasm | Ubiquitin-specific protease activity | Cytoplasm |
| **CHD2** | NP_001262.3 | 1106 | **chromodomain-helicase-DNA-binding protein 2 isoform 1** | 0.8 | 1 | 1 | 2 | 2 | 1.08E+08 | **0.68** | 1 | - | 1828 | 211.2 | 8.1 | Regulation of nucleobase, nucleoside, nucleotide and nucleic acid metabolism | Nucleus | DNA binding | Nucleus |
| **INTS1** | NP_001073922.2 | 26173 | **integrator complex subunit 1** | 3.7 | 1 | 5 | 5 | 7 | 3.33E+07 | **0.68** | 3 | 14.9 | 2190 | 244.1 | 6.1 | RNA metabolism;Transcription | Nucleus | Molecular function unknown | Nucleus |
| **MCU** | NP_001257609.1 | 90550 | **calcium uniporter protein, mitochondrial isoform 3** | 21.2 | 3 | 4 | 4 | 8 | 7.80E+07 | **0.68** | 3 | 42.5 | 302 | 35.1 | 7.5 | Biological_process unknown | - | Molecular function unknown | - |
| **RPS5** | NP_001000.2 | 6193 | **40S ribosomal protein S5** | 54.4 | 1 | 14 | 14 | 188 | 2.55E+09 | **0.68** | 25 | 11.8 | 204 | 22.9 | 9.7 | Protein metabolism | Ribosome;Nucleolus | Structural constituent of ribosome | Ribosome |
| **RRM1** | NP_001024.1 | 6240 | **ribonucleoside-diphosphate reductase large subunit** | 42.6 | 1 | 20 | 20 | 51 | 2.96E+08 | **0.68** | 21 | 34.6 | 792 | 90 | 7.2 | Regulation of nucleobase, nucleoside, nucleotide and nucleic acid metabolism | Cytoplasm | Oxidoreductase activity | Cytoplasm |
| **MRPS27** | NP_001273680.1 | 23107 | **28S ribosomal protein S27, mitochondrial isoform 3** | 41.9 | 3 | 9 | 9 | 17 | 1.69E+08 | **0.67** | 7 | 32.5 | 358 | 41.3 | 5.5 | Protein metabolism | Mitochondrion | Structural constituent of ribosome | Mitochondrion |
| **MRPL51** | NP_057581.2 | 51258 | **39S ribosomal protein L51, mitochondrial** | 4.7 | 1 | 1 | 1 | 1 | 7.86E+07 | **0.67** | 1 | - | 128 | 15.1 | 11.3 | Protein metabolism | Mitochondrion | Structural constituent of ribosome | Mitochondrion |
| **TRAP1** | NP_001258978.1 | 10131 | **heat shock protein 75 kDa, mitochondrial isoform 2** | 34.1 | 2 | 14 | 15 | 147 | 8.04E+09 | **0.67** | 19 | 15.9 | 651 | 74.2 | 7.9 | Protein metabolism | Mitochondrion | Heat shock protein activity | Mitochondrion |
| **ZCCHC8** | NP_060082.2 | 55596 | **zinc finger CCHC domain-containing protein 8** | 31.8 | 1 | 12 | 12 | 20 | 1.08E+08 | **0.67** | 11 | 30.1 | 707 | 78.5 | 4.9 | Biological_process unknown | Nucleus | Molecular function unknown | Nucleus |
| **PHKB** | NP_001027005.1 | 5257 | **phosphorylase b kinase regulatory subunit beta isoform b** | 1.2 | 2 | 1 | 1 | 1 | 9.67E+05 | **0.67** | 1 | - | 1086 | 123.7 | 6.6 | Biological_process unknown | - | Molecular function unknown | - |
| **TMED2** | NP_006806.1 | 10959 | **transmembrane emp24 domain-containing protein 2 precursor** | 24.9 | 1 | 6 | 6 | 27 | 3.61E+08 | **0.67** | 8 | 7.1 | 201 | 22.7 | 5.2 | Transport | Golgi apparatus;Microsome;Plasma membrane | Transporter activity | Golgi apparatus |
| **MRPL21** | NP_852616.1 | 219927 | **39S ribosomal protein L21, mitochondrial isoform a** | 35.8 | 2 | 3 | 3 | 7 | 1.13E+08 | **0.67** | 4 | 15.3 | 120 | 13.7 | 10.1 | Protein metabolism | Mitochondrion | Structural constituent of ribosome | Mitochondrion |
| **MTMR3** | NP_694691.1 | 8897 | **myotubularin-related protein 3 isoform b** | 1.1 | 3 | 1 | 1 | 1 | 1.57E+07 | **0.67** | 1 | - | 1161 | 129.4 | 6.1 | Cell communication;Signal transduction | Nucleus;Cytoplasm;Plasma membrane | Protein tyrosine/serine/threonine phosphatase activity | Nucleus |
| **RAB35** | NP_006852.1 | 11021 | **ras-related protein Rab-35 isoform 1** | 40.8 | 16 | 5 | 7 | 27 | 7.66E+08 | **0.67** | 5 | 7.4 | 201 | 23 | 8.3 | Cell communication;Signal transduction | - | GTPase activity | - |

Supplementary Table S4. List of all proteins identified in SCC-S and SCC-R cells using SequestHT and Mascot search algorithms and their gene ontology annotation

| Gene Symbol | RefSeq_Accession | Entrez Gene ID | Description | Coverage (%) | Proteins | Unique Peptides | # Peptides | PSM | Area | SCC-R /SCC-S | SCC-R /SCC-S (Count) | SCC-R /SCC-S (Variability [%]) | # AAs | MW [kDa] | calc. pI | Biological Process | Localization | Molecular Function | Primary Localization |
|---|---|---|---|---|---|---|---|---|---|---|---|---|---|---|---|---|---|---|---|
| **RBM26** | NP_001273561.1 | 64062 | **RNA-binding protein 26 isoform 2** | 12.4 | 1 | 1 | 7 | 13 | 1.31E+08 | **0.67** | 1 | - | 983 | 111 | 9.1 | Regulation of nucleobase, nucleoside, nucleotide and nucleic acid metabolism | Nucleus | RNA binding | Nucleus |
| **VPS25** | NP_115729.1 | 84313 | **vacuolar protein-sorting-associated protein 25** | 8.5 | 1 | 1 | 1 | 2 | 3.75E+07 | **0.67** | 1 | - | 176 | 20.7 | 6.3 | Transport | Endosome;Nucleus | Transporter activity | Endosome |
| **PPAN** | NP_064615.3 | 56342 | **suppressor of SWI4 1 homolog** | 19.9 | 3 | 6 | 6 | 14 | 1.80E+08 | **0.67** | 8 | 44.1 | 473 | 53.2 | 10.1 | Cell communication;Signal transduction | Nucleolus;Nucleus | Ribonucleoprotein | Nucleolus |
| **CCS** | NP_005116.1 | 9973 | **copper chaperone for superoxide dismutase** | 28.1 | 1 | 6 | 6 | 10 | 8.48E+07 | **0.67** | 7 | 42.7 | 274 | 29 | 5.6 | Protein metabolism | Cytoplasm;Nucleus | Chaperone activity | Cytoplasm |
| **MED12** | NP_005111.2 | 9968 | **mediator of RNA polymerase II transcription subunit 12** | 0.7 | 1 | 1 | 1 | 1 | 1.05E+07 | **0.67** | 1 | - | 2177 | 242.9 | 7.1 | Cell communication;Signal transduction | Nucleus | Transcription regulator activity | Nucleus |
| **PHF3** | NP_001277188.1 | 23469 | **PHD finger protein 3 isoform 2** | 1.4 | 3 | 2 | 2 | 2 | 3.25E+07 | **0.67** | 2 | 526.8 | 1951 | 219.7 | 7.5 | Regulation of nucleobase, nucleoside, nucleotide and nucleic acid metabolism | Nucleus;Cytoplasm | Transcription factor activity | Nucleus |
| **PPM1G** | NP_817092.1 | 5496 | **protein phosphatase 1G** | 51.5 | 1 | 19 | 19 | 58 | 4.71E+08 | **0.67** | 18 | 25.7 | 546 | 59.2 | 4.4 | Cell communication;Signal transduction;Protein modification | Cytoplasm;Nucleoplasm | Protein serine/threonine phosphatase activity | Nucleoplasm |
| **RPL26** | NP_000978.1 | 6154 | **60S ribosomal protein L26** | 39.3 | 2 | 9 | 9 | 50 | 1.00E+09 | **0.67** | 22 | 11.3 | 145 | 17.2 | 10.6 | Protein metabolism | Ribosome;Nucleolus | Structural constituent of ribosome | Ribosome |
| **SDE2** | NP_689821.3 | 163859 | **protein SDE2 homolog** | 2.4 | 1 | 1 | 1 | 1 | 1.09E+07 | **0.67** | 1 | - | 451 | 49.7 | 6.1 | Biological_process unknown | Nucleus | Molecular function unknown | Nucleus |
| **STK3** | NP_006272.2 | 6788 | **serine/threonine-protein kinase 3 isoform 1** | 5.3 | 2 | 1 | 2 | 6 | 4.95E+07 | **0.67** | 1 | - | 491 | 56.3 | 5.2 | Cell communication;Signal transduction | Cytoplasm;Nucleus | Protein serine/threonine kinase activity | Cytoplasm |
| **TAX1BP3** | NP_055419.1 | 30851 | **tax1-binding protein 3 isoform 1** | 39.5 | 2 | 4 | 4 | 7 | 2.87E+08 | **0.67** | 2 | 7 | 124 | 13.7 | 8.5 | Regulation of nucleobase, nucleoside, nucleotide and nucleic acid metabolism | Cytoplasm;Nucleus | Transcription regulator activity | Cytoplasm |
| **GOSR2** | NP_004278.2 | 9570 | **Golgi SNAP receptor complex member 2 isoform A** | 45.8 | 3 | 5 | 5 | 12 | 2.01E+08 | **0.67** | 5 | 46.2 | 212 | 24.8 | 8.1 | Transport | Golgi apparatus;Endoplasmic reticulum | Auxiliary transport protein activity | Golgi apparatus |
| **APPL1** | NP_036228.1 | 26060 | **DCC-interacting protein 13-alpha** | 12.0 | 1 | 4 | 4 | 6 | 2.55E+07 | **0.67** | 3 | 24.3 | 709 | 79.6 | 5.4 | Cell communication;Signal transduction | Cytoplasm | Receptor signaling complex scaffold activity | Cytoplasm |
| **ATP5J2** | NP_001034267.1 | 9551 | **ATP synthase subunit f, mitochondrial isoform 2d** | 49.0 | 5 | 2 | 2 | 8 | 3.59E+08 | **0.67** | 2 | 7.8 | 49 | 5.7 | 9.7 | Electron transport | Mitochondrion | ATPase activity | Mitochondrion |
| **EIF2D** | NP_008824.2 | 1939 | **eukaryotic translation initiation factor 2D isoform 1** | 21.1 | 2 | 6 | 6 | 10 | 5.54E+07 | **0.67** | 5 | 9.2 | 584 | 64.7 | 7.7 | Cell growth and/or maintenance | Plasma membrane | Receptor activity | Plasma membrane |
| **NUP37** | NP_076962.2 | 79023 | **nucleoporin Nup37** | 22.4 | 1 | 5 | 5 | 12 | 3.22E+08 | **0.67** | 5 | 23.4 | 326 | 36.7 | 5.9 | Transport | Nucleus;Nuclear membrane | Transporter activity | Nucleus |
| **PRDX3** | NP_054817.2 | 10935 | **thioredoxin-dependent peroxide reductase, mitochondrial isoform b** | 44.5 | 2 | 10 | 10 | 74 | 1.33E+09 | **0.67** | 13 | 35.1 | 238 | 25.8 | 7.5 | Metabolism;Energy pathways | Mitochondrion | Peroxidase activity | Mitochondrion |
| **TRAPPC3** | NP_001257824.1 | 27095 | **trafficking protein particle complex subunit 3 isoform 3** | 6.0 | 3 | 1 | 1 | 1 | 1.02E+07 | **0.67** | 1 | - | 134 | 15 | 4.9 | Transport | Golgi apparatus | Transporter activity | Golgi apparatus |
| **DNASE2** | NP_001366.1 | 1777 | **deoxyribonuclease-2-alpha precursor** | 3.1 | 1 | 1 | 1 | 2 | 8.04E+07 | **0.67** | 1 | - | 360 | 39.6 | 8.1 | Regulation of nucleobase, nucleoside, nucleotide and nucleic acid metabolism | Lysosome;Nucleus | Deoxyribonuclease activity | Lysosome |
| **PPP2R5E** | NP_001269109.1 | 5529 | **serine/threonine-protein phosphatase 2A 56 kDa regulatory subunit epsilon isoform isoform b** | 10.0 | 3 | 3 | 3 | 3 | 1.93E+07 | **0.67** | 1 | - | 462 | 54 | 6.6 | Cell communication;Signal transduction | Cytoplasm;Chromosome | Molecular function unknown | Cytoplasm |
| **DIAPH1** | NP_001073280.1 | 1729 | **protein diaphanous homolog 1 isoform 2** | 32.5 | 2 | 28 | 28 | 74 | 4.74E+08 | **0.66** | 30 | 20.3 | 1263 | 140.2 | 5.4 | Cell growth and/or maintenance | Cytoplasm;Mitochondrion | Cytoskeletal protein binding | Cytoplasm |
| **MRPL22** | NP_054899.2 | 29093 | **39S ribosomal protein L22, mitochondrial isoform a** | 19.4 | 2 | 3 | 3 | 7 | 3.95E+07 | **0.66** | 2 | 32.1 | 206 | 23.6 | 9.9 | Protein metabolism | Mitochondrion | Structural constituent of ribosome | Mitochondrion |
| **UPF2** | NP_056357.1 | 26019 | **regulator of nonsense transcripts 2** | 3.5 | 1 | 2 | 2 | 3 | 1.35E+08 | **0.66** | 2 | 11.3 | 1272 | 147.7 | 5.7 | Regulation of nucleobase, nucleoside, nucleotide and nucleic acid metabolism;RNA metabolism | Cytoplasm;Nucleus | RNA binding;MRNA binding | Cytoplasm |
| **ASS1** | NP_000041.2 | 445 | **argininosuccinate synthase** | 44.2 | 1 | 15 | 15 | 58 | 4.52E+08 | **0.66** | 18 | 23.7 | 412 | 46.5 | 8.0 | Metabolism;Energy pathways | Cytosol;Nucleus;Microsome | Ligase activity;Catalytic activity | Cytosol |
| **ATP5A1** | NP_001001935.1 | 498 | **ATP synthase subunit alpha, mitochondrial isoform c** | 53.9 | 3 | 26 | 26 | 165 | 2.40E+09 | **0.66** | 36 | 19.3 | 503 | 54.5 | 8.2 | Metabolism;Energy pathways | Mitochondrion;Extracellular;Zymogen granule | Transporter activity | Mitochondrion |
| **CELF1** | NP_001166110.1 | 10658 | **CUGBP Elav-like family member 1 isoform 4** | 16.2 | 5 | 1 | 5 | 10 | 1.27E+08 | **0.66** | 1 | - | 512 | 55 | 8.4 | Regulation of nucleobase, nucleoside, nucleotide and nucleic acid metabolism;Regulation of gene expression, epigenetic | Cytoplasm;Nucleus | RNA binding | Nucleus |
| **PMF1-BGLAP** | NP_001186593.1 | 100527963 | **PMF1-BGLAP protein isoform 4** | 8.5 | 7 | 1 | 1 | 1 | 3.75E+07 | **0.66** | 1 | - | 142 | 15.7 | 9.4 | - | - | - | - |
| **RPL11** | NP_001186731.1 | 6135 | **60S ribosomal protein L11 isoform 2** | 24.9 | 1 | 1 | 5 | 47 | 2.05E+09 | **0.66** | 1 | - | 177 | 20.1 | 9.6 | Protein metabolism | Ribosome;Nucleolus;Nucleoplasm;Mitochondrion | Structural constituent of ribosome | Ribosome |
| **ARMCX3** | NP_808817.1 | 51566 | **armadillo repeat-containing X-linked protein 3** | 15.8 | 1 | 3 | 3 | 7 | 5.54E+07 | **0.66** | 4 | 30.8 | 379 | 42.5 | 8.4 | Immune response;Embryonic development | Plasma membrane;Cytoplasm | Molecular function unknown | Plasma membrane |
| **KPNA2** | NP_002257.1 | 3838 | **importin subunit alpha-1** | 59.0 | 1 | 18 | 18 | 92 | 1.11E+09 | **0.66** | 26 | 20.6 | 529 | 57.8 | 5.4 | Cell communication;Signal transduction | Cytoplasm;Nucleus;Plasma membrane;Nucleolus;Centrosome | Transporter activity | Cytoplasm |
| **SKIV2L2** | NP_056175.3 | 23517 | **superkiller viralicidic activity 2-like 2** | 27.3 | 1 | 21 | 21 | 45 | 2.94E+08 | **0.66** | 20 | 30.7 | 1042 | 117.7 | 6.5 | Regulation of nucleobase, nucleoside, nucleotide and nucleic acid metabolism | Nucleus;Nucleolus | Helicase activity | Nucleus |
| **BLOC1S2** | NP_001269368.1 | 282991 | **biogenesis of lysosome-related organelles complex 1 subunit 2 isoform 4** | 24.8 | 3 | 2 | 2 | 2 | 9.92E+07 | **0.66** | 1 | - | 141 | 15.8 | 4.9 | Biological_process unknown | - | Molecular function unknown | - |
| **STRIP2** | NP_001127808.1 | 57464 | **striatin-interacting protein 2 isoform b** | 4.2 | 2 | 1 | 2 | 2 | 3.63E+07 | **0.66** | 1 | - | 758 | 86 | 6.6 | Biological_process unknown | - | Molecular function unknown | - |
| **TOMM20** | NP_055580.1 | 9804 | **mitochondrial import receptor subunit TOM20 homolog** | 27.6 | 1 | 2 | 2 | 4 | 9.69E+08 | **0.66** | 1 | - | 145 | 16.3 | 8.6 | Transport | Mitochondrion | Auxiliary transport protein activity | Mitochondrion |
| **DPF2** | NP_006259.1 | 5977 | **zinc finger protein ubi-d4** | 10.2 | 1 | 3 | 3 | 7 | 4.36E+07 | **0.66** | 1 | - | 391 | 44.1 | 6.3 | Regulation of nucleobase, nucleoside, nucleotide and nucleic acid metabolism | Nucleus;Cytoplasm | DNA binding | Nucleus |
| **POLD1** | NP_002682.2 | 5424 | **DNA polymerase delta catalytic subunit** | 10.7 | 1 | 7 | 7 | 7 | 8.35E+07 | **0.66** | 5 | 17.8 | 1107 | 123.6 | 7.0 | Regulation of nucleobase, nucleoside, nucleotide and nucleic acid metabolism | Nucleus | DNA-directed DNA polymerase activity | Nucleus |
| **UGP2** | NP_006750.3 | 7360 | **UTP--glucose-1-phosphate uridylyltransferase isoform a** | 38.6 | 2 | 14 | 14 | 44 | 3.53E+08 | **0.66** | 17 | 19.8 | 508 | 56.9 | 8.2 | Metabolism;Energy pathways | - | Nucleotidyltransferase activity | - |

Jain *et al.*, 2019. MAP2K1 is a potential therapeutic target in erlotinib resistant head and neck squamous cell carcinoma
Supplementary Table S4. List of all proteins identified in SCC-S and SCC-R cells using SequestHT and Mascot search algorithms and their gene ontology annotation

| Gene Symbol | RefSeq_Accession | Entrez Gene ID | Description | Coverage (%) | Proteins | Unique Peptides | # Peptides | PSM | Area | SCC-R /SCC-S | SCC-R /SCC-S (Count) | SCC-R /SCC-S (Variability [%]) | # AAs | MW [kDa] | calc. pI | Biological Process | Localization | Molecular Function | Primary Localization |
|---|---|---|---|---|---|---|---|---|---|---|---|---|---|---|---|---|---|---|---|
| **RPS14** | NP_005608.1 | 6208 | **40S ribosomal protein S14** | 72.2 | 2 | 14 | 14 | 84 | 1.74E+09 | **0.66** | 21 | 24.8 | 151 | 16.3 | 10.1 | Protein metabolism | Cytoplasm;Nucleolus;Ribosome | Structural constituent of ribosome | Ribosome |
| **HMGN1** | NP_004956.5 | 3150 | **non-histone chromosomal protein HMG-14** | 52.0 | 1 | 6 | 6 | 10 | 1.12E+08 | **0.66** | 7 | 3.8 | 100 | 10.7 | 9.6 | Regulation of nucleobase, nucleoside, nucleotide and nucleic acid metabolism | Nucleus;Nucleolus | Transcription regulator activity | Nucleus |
| **HSPBP1** | NP_001123578.1 | 23640 | **hsp70-binding protein 1** | 20.3 | 1 | 6 | 6 | 14 | 6.44E+07 | **0.66** | 4 | 9.8 | 359 | 39.3 | 5.2 | Protein metabolism | - | Molecular function unknown | - |
| **LIN7C** | NP_060832.1 | 55327 | **protein lin-7 homolog C** | 40.1 | 2 | 4 | 4 | 5 | 5.74E+07 | **0.66** | 4 | 5.8 | 197 | 21.8 | 8.4 | Cell communication;Signal transduction | Extracellular | Molecular function unknown | Extracellular |
| **SMNDC1** | NP_005862.1 | 10285 | **survival of motor neuron-related-splicing factor 30** | 15.6 | 1 | 3 | 3 | 7 | 2.13E+08 | **0.66** | 3 | 6.7 | 238 | 26.7 | 7.2 | Cell growth and/or maintenance | Nucleus;Nucleolus | Structural molecule activity | Nucleus |
| **DNAJC2** | NP_001123359.1 | 27000 | **dnaJ homolog subfamily C member 2 isoform 2** | 4.6 | 2 | 2 | 2 | 2 | 2.35E+07 | **0.66** | 1 | - | 568 | 65.9 | 9.1 | Regulation of cell cycle | Ribosome;Microtubule;Cytoplasm;Nucleus;Cytosol | Chaperone activity | Nucleus |
| **HECTD3** | NP_078878.3 | 79654 | **E3 ubiquitin-protein ligase HECTD3** | 5.8 | 1 | 3 | 3 | 4 | 3.51E+07 | **0.66** | 2 | 35.7 | 861 | 97.1 | 5.6 | Protein metabolism | - | Ubiquitin-specific protease activity | - |
| **MAN2C1** | NP_001243425.1 | 4123 | **alpha-mannosidase 2C1 isoform 4** | 3.0 | 4 | 1 | 1 | 4 | 6.98E+07 | **0.66** | 2 | 7.5 | 941 | 104.6 | 6.5 | Metabolism;Energy pathways | Cytoplasm;Nucleus | Hydrolase activity | Cytoplasm |
| **NXF1** | NP_006353.2 | 10482 | **nuclear RNA export factor 1 isoform 1** | 17.9 | 2 | 7 | 7 | 10 | 5.02E+07 | **0.66** | 4 | 15 | 619 | 70.1 | 8.5 | Regulation of nucleobase, nucleoside, nucleotide and nucleic acid metabolism | Nucleus | RNA binding | Nucleus |
| **MAN2A1** | NP_002363.2 | 4124 | **alpha-mannosidase 2** | 8.0 | 1 | 5 | 5 | 10 | 1.86E+08 | **0.66** | 6 | 9.5 | 1144 | 131.1 | 7.6 | Metabolism;Energy pathways | Golgi apparatus | Catalytic activity | Golgi apparatus |
| **PET112** | NP_004555.1 | 5188 | **glutamyl-tRNA(Gln) amidotransferase subunit B, mitochondrial precursor** | 5.4 | 1 | 1 | 1 | 1 | 1.90E+08 | **0.66** | 1 | - | 557 | 61.8 | 8.6 | Protein metabolism | Mitochondrion | Translation regulator activity | Mitochondrion |
| **GMPR2** | NP_001269952.1 | 51292 | **GMP reductase 2 isoform 5** | 15.3 | 5 | 3 | 3 | 9 | 1.36E+08 | **0.65** | 2 | 10.9 | 320 | 34.6 | 7.7 | Metabolism | Cytoplasm | Catalytic activity | Cytoplasm |
| **CYB5B** | NP_085056.2 | 80777 | **cytochrome b5 type B** | 51.3 | 1 | 4 | 4 | 22 | 1.09E+09 | **0.65** | 6 | 9.6 | 150 | 16.7 | 5.0 | Transport | Mitochondrion | Transporter activity | Mitochondrion |
| **FBXO22** | NP_671717.1 | 26263 | **F-box only protein 22 isoform a** | 12.2 | 1 | 2 | 2 | 2 | 1.42E+08 | **0.65** | 1 | - | 403 | 44.5 | 7.0 | Protein metabolism | - | Ubiquitin-specific protease activity | - |
| **NKAP** | NP_078804.2 | 79576 | **NF-kappa-B-activating protein** | 10.8 | 1 | 2 | 2 | 2 | 7.20E+07 | **0.65** | 1 | - | 415 | 47.1 | 10.1 | Biological_process unknown | Nucleus;Cytoplasm | Molecular function unknown | Nucleus |
| **STK39** | NP_037365.2 | 27347 | **STE20/SPS1-related proline-alanine-rich protein kinase** | 8.1 | 1 | 1 | 3 | 7 | 7.78E+07 | **0.65** | 1 | - | 545 | 59.4 | 6.3 | Cell communication;Signal transduction | Cytoplasm;Nucleus | Protein serine/threonine kinase activity | Cytoplasm |
| **UCHL3** | NP_005993.1 | 7347 | **ubiquitin carboxyl-terminal hydrolase isozyme L3 isoform 2** | 56.5 | 2 | 9 | 9 | 29 | 3.95E+08 | **0.65** | 7 | 20.3 | 230 | 26.2 | 4.9 | Protein metabolism | Cytoplasm | Ubiquitin-specific protease activity | Cytoplasm |
| **HNRNPR** | NP_005817.1 | 10236 | **heterogeneous nuclear ribonucleoprotein R isoform 2** | 45.5 | 4 | 20 | 26 | 130 | 1.57E+09 | **0.65** | 24 | 15.7 | 633 | 70.9 | 8.1 | Regulation of nucleobase, nucleoside, nucleotide and nucleic acid metabolism | Nucleus;Nucleolus;Mitochondrion | RNA binding | Nucleus |
| **NIP7** | NP_057185.1 | 51388 | **60S ribosome subunit biogenesis protein NIP7 homolog isoform 1** | 40.6 | 2 | 3 | 3 | 6 | 3.23E+07 | **0.65** | 1 | - | 180 | 20.4 | 8.5 | Cell growth and/or maintenance | Nucleolus;Nucleus | RNA binding | Nucleolus |
| **RBM34** | NP_055829.2 | 23029 | **RNA-binding protein 34 isoform 1** | 24.9 | 2 | 6 | 6 | 13 | 7.21E+07 | **0.65** | 4 | 25.1 | 430 | 48.5 | 10.1 | Regulation of nucleobase, nucleoside, nucleotide and nucleic acid metabolism | Nucleolus;Nucleus | RNA binding | Nucleolus |
| **HSPA2** | NP_068814.2 | 3306 | **heat shock-related 70 kDa protein 2** | 30.7 | 1 | 6 | 20 | 300 | 1.51E+10 | **0.65** | 6 | 23.4 | 639 | 70 | 5.7 | Protein metabolism | Cytoplasm;Nucleolus;Nucleus | Heat shock protein activity | Nucleus |
| **NDUFB5** | NP_001186886.1 | 4711 | **NADH dehydrogenase [ubiquinone] 1 beta subcomplex subunit 5, mitochondrial isoform 2** | 26.3 | 2 | 2 | 2 | 2 | 2.45E+07 | **0.65** | 1 | - | 137 | 15.7 | 7.6 | Metabolism;Energy pathways | Mitochondrion | Oxidoreductase activity | Mitochondrion |
| **PSMB5** | NP_002788.1 | 5693 | **proteasome subunit beta type-5 isoform 1** | 38.4 | 3 | 8 | 8 | 45 | 2.66E+08 | **0.65** | 10 | 14.1 | 263 | 28.5 | 6.9 | Protein metabolism;Proteolysis and peptidolysis | Nucleus;Cytoplasm;Cytosol | Ubiquitin-specific protease activity | Cytoplasm |
| **ALAD** | NP_000022.3 | 210 | **delta-aminolevulinic acid dehydratase** | 20.0 | 1 | 5 | 5 | 10 | 2.16E+08 | **0.65** | 4 | 15.8 | 330 | 36.3 | 6.8 | Metabolism;Energy pathways | Cytoplasm | Hydro-lyase activity | Cytoplasm |
| **APOL3** | NP_663617.1 | 80833 | **apolipoprotein L3 isoform 3** | 3.5 | 2 | 1 | 1 | 1 | 3.36E+07 | **0.65** | 1 | - | 202 | 21.6 | 8.8 | Lipoprotein metabolism | Cytoplasm;Extracellular | Lipid transporter activity | Extracellular |
| **MADD** | NP_569830.2 | 8567 | **MAP kinase-activating death domain protein isoform f** | 1.1 | 10 | 1 | 1 | 1 | 3.84E+07 | **0.65** | 1 | - | 1479 | 163.9 | 6.3 | Anti-apoptosis;Cell communication;Signal transduction | Cytoplasm;Plasma membrane | Kinase regulator activity;Receptor signaling complex scaffold activity | Plasma membrane |
| **SRSF6** | NP_006266.2 | 6431 | **serine/arginine-rich splicing factor 6** | 21.8 | 1 | 6 | 9 | 39 | 1.43E+09 | **0.65** | 11 | 9.5 | 344 | 39.6 | 11.4 | Regulation of nucleobase, nucleoside, nucleotide and nucleic acid metabolism | Nucleus;Nucleolus;Cytoplasm | RNA binding | Nucleus |
| **AASS** | NP_005754.2 | 10157 | **alpha-aminoadipic semialdehyde synthase, mitochondrial** | 9.0 | 1 | 5 | 5 | 6 | 4.68E+07 | **0.65** | 2 | 54.4 | 926 | 102.1 | 6.6 | Metabolism;Energy pathways | Mitochondrion | Oxidoreductase activity | Mitochondrion |
| **FARP1** | NP_005757.1 | 10160 | **FERM, RhoGEF and pleckstrin domain-containing protein 1 isoform 1** | 6.5 | 2 | 4 | 4 | 5 | 6.34E+07 | **0.65** | 3 | 25.9 | 1045 | 118.6 | 8.2 | Cell growth and/or maintenance | - | Guanyl-nucleotide exchange factor activity | - |
| **KIAA1919** | NP_699200.2 | 91749 | **sodium-dependent glucose transporter 1** | 6.6 | 1 | 1 | 1 | 1 | 1.46E+07 | **0.65** | 1 | - | 518 | 56.2 | 5.4 | Transport | Plasma membrane | Auxiliary transport protein activity | Plasma membrane |
| **REEP5** | NP_005660.4 | 7905 | **receptor expression-enhancing protein 5** | 22.2 | 1 | 4 | 4 | 7 | 5.86E+07 | **0.65** | 4 | 29.7 | 189 | 21.5 | 8.1 | Biological_process unknown | - | Molecular function unknown | - |
| **HLTF** | NP_003062.2 | 6596 | **helicase-like transcription factor** | 9.9 | 1 | 5 | 5 | 8 | 3.39E+07 | **0.65** | 3 | 2.5 | 1009 | 113.9 | 8.6 | Regulation of nucleobase, nucleoside, nucleotide and nucleic acid metabolism | Nucleus;Nucleolus;Cytoplasm | DNA binding | Nucleus |
| **DUS3L** | NP_064560.2 | 56931 | **tRNA-dihydrouridine(47) synthase [NAD(P)(+)]-like isoform 1** | 8.8 | 1 | 4 | 4 | 7 | 8.38E+07 | **0.65** | 3 | 17.7 | 650 | 72.5 | 8.1 | Regulation of nucleobase, nucleoside, nucleotide and nucleic acid metabolism | Cytoplasm | DNA binding | Cytoplasm |
| **SLC31A1** | NP_001850.1 | 1317 | **high affinity copper uptake protein 1** | 10.0 | 1 | 1 | 1 | 2 | 1.07E+08 | **0.65** | 1 | - | 190 | 21.1 | 7.4 | Transport | Plasma membrane;Endoplasmic reticulum;Golgi apparatus | Transporter activity | Plasma membrane |
| **ANAPC5** | NP_001131031.1 | 51433 | **anaphase-promoting complex subunit 5 isoform b** | 2.6 | 2 | 1 | 1 | 1 | 1.79E+07 | **0.65** | 1 | - | 643 | 72.5 | 6.7 | Cell communication;Signal transduction | Nucleus;Centrosome | Ubiquitin-specific protease activity | Nucleus |
| **NAT10** | NP_078938.2 | 55226 | **N-acetyltransferase 10 isoform a** | 35.1 | 2 | 25 | 25 | 50 | 5.60E+08 | **0.65** | 15 | 26.6 | 1025 | 115.6 | 8.3 | Metabolism;Energy pathways | Nucleus | Acyltransferase activity | Nucleus |
| **SEMA5B** | NP_001243277.1 | 54437 | **semaphorin-5B isoform 3 precursor** | 0.7 | 3 | 1 | 1 | 3 | 3.60E+08 | **0.65** | 2 | 14.3 | 1057 | 116 | 7.5 | Signal transduction;Cell communication | Integral to membrane | Molecular function unknown | Integral to membrane |

Supplementary Table S4. List of all proteins identified in SCC-S and SCC-R cells using SequestHT and Mascot search algorithms and their gene ontology annotation

| Gene Symbol | RefSeq_Accession | Entrez Gene ID | Description | Coverage (%) | Proteins | Unique Peptides | # Peptides | PSM | Area | SCC-R /SCC-S | SCC-R /SCC-S (Count) | SCC-R /SCC-S (Variability [%]) | # AAs | MW [kDa] | calc. pI | Biological Process | Localization | Molecular Function | Primary Localization |
|---|---|---|---|---|---|---|---|---|---|---|---|---|---|---|---|---|---|---|---|
| **SLC25A6** | NP_001627.2 | 293 | **ADP/ATP translocase 3** | 52.4 | 2 | 3 | 17 | 123 | 3.69E+09 | **0.65** | 6 | 10.6 | 298 | 32.8 | 9.7 | Transport | Mitochondrion | Transporter activity | Mitochondrion |
| **TRMT1** | NP_001136026.1 | 55621 | **tRNA (guanine(26)-N(2))-dimethyltransferase isoform 2** | 16.8 | 2 | 6 | 6 | 14 | 3.53E+08 | **0.65** | 6 | 5.4 | 630 | 69.3 | 7.2 | Regulation of nucleobase, nucleoside, nucleotide and nucleic acid metabolism | - | RNA methyltransferase activity | - |
| **POLR1E** | NP_071935.1 | 64425 | **DNA-directed RNA polymerase I subunit RPA49 isoform 1** | 1.9 | 1 | 1 | 1 | 1 | 1.19E+08 | **0.65** | 1 | - | 419 | 47.2 | 8.9 | Regulation of nucleobase, nucleoside, nucleotide and nucleic acid metabolism | Nucleolus;Nucleus | Transcription factor activity | Nucleolus |
| **RARS** | NP_002878.2 | 5917 | **arginine--tRNA ligase, cytoplasmic** | 40.6 | 1 | 26 | 26 | 49 | 2.96E+08 | **0.64** | 20 | 18.4 | 660 | 75.3 | 6.7 | Protein metabolism | Cytoplasm;Cytosol | Ligase activity | Cytoplasm |
| **SAMM50** | NP_056195.3 | 25813 | **sorting and assembly machinery component 50 homolog** | 12.8 | 1 | 4 | 4 | 5 | 6.65E+07 | **0.64** | 3 | 1.4 | 469 | 51.9 | 6.9 | Biological_process unknown | Mitochondrion | Molecular function unknown | Mitochondrion |
| **GRSF1** | NP_002083.3 | 2926 | **G-rich sequence factor 1 isoform 1** | 46.5 | 2 | 15 | 15 | 40 | 7.98E+08 | **0.64** | 18 | 42.9 | 480 | 53.1 | 6.2 | Protein metabolism | Cytoplasm;Nucleolus;Mitochondrion | RNA binding | Cytoplasm |
| **WDR4** | NP_001247404.1 | 10785 | **tRNA (guanine-N(7)-)-methyltransferase non-catalytic subunit WDR4 isoform 3** | 8.7 | 3 | 1 | 1 | 2 | 1.03E+08 | **0.64** | 1 | - | 266 | 29.9 | 7.4 | Biological_process unknown | - | Molecular function unknown | - |
| **CAMK2G** | NP_001213.2 | 818 | **calcium/calmodulin-dependent protein kinase type II subunit gamma isoform 4** | 20.8 | 16 | 4 | 5 | 15 | 1.60E+08 | **0.64** | 1 | - | 495 | 55.9 | 7.2 | Cell communication;Signal transduction | Cytoplasm;Nucleus | Protein serine/threonine kinase activity | Cytoplasm |
| **PPP1CC** | NP_002701.1 | 5501 | **serine/threonine-protein phosphatase PP1-gamma catalytic subunit isoform 1** | 43.0 | 2 | 2 | 15 | 53 | 4.88E+08 | **0.64** | 2 | 26.5 | 323 | 37 | 6.5 | Cell communication;Signal transduction | Nucleus;Cytoplasm;Nucleolus;Microtubule | Protein serine/threonine phosphatase activity | Nucleus |
| **TJP1** | NP_783297.2 | 7082 | **tight junction protein ZO-1 isoform b** | 29.3 | 2 | 29 | 29 | 65 | 3.87E+08 | **0.64** | 21 | 15.8 | 1668 | 186.9 | 6.8 | Cell growth and/or maintenance | Cell surface;Cytoplasm;Nucleus;Extracellular | Cell adhesion molecule activity | Cell surface;Nucleus;Cytoplasm |
| **VPS33A** | NP_075067.2 | 65082 | **vacuolar protein sorting-associated protein 33A** | 11.1 | 1 | 3 | 3 | 6 | 1.30E+07 | **0.64** | 2 | 5.9 | 596 | 67.6 | 7.0 | Transport | Golgi apparatus;Lysosome | Transporter activity | Golgi apparatus |
| **MSH3** | NP_002430.3 | 4437 | **DNA mismatch repair protein Msh3** | 3.6 | 1 | 2 | 2 | 2 | 7.95E+06 | **0.64** | 1 | - | 1137 | 127.3 | 8.0 | Regulation of nucleobase, nucleoside, nucleotide and nucleic acid metabolism | Nucleus | DNA repair protein | Nucleus |
| **SHPK** | NP_037408.2 | 23729 | **sedoheptulokinase** | 11.3 | 1 | 3 | 3 | 6 | 6.31E+07 | **0.64** | 2 | 4.5 | 478 | 51.5 | 6.8 | Cell communication;Signal transduction | Cytoplasm | Molecular function unknown | Cytoplasm |
| **SLC25A22** | NP_078974.1 | 79751 | **mitochondrial glutamate carrier 1** | 15.5 | 1 | 3 | 3 | 6 | 1.28E+08 | **0.64** | 2 | 36.8 | 323 | 34.4 | 9.3 | Transport | Mitochondrion | Auxiliary transport protein activity | Mitochondrion |
| **BAX** | NP_001278360.1 | 581 | **apoptosis regulator BAX isoform zeta** | 9.7 | 2 | 1 | 2 | 2 | 4.78E+07 | **0.64** | 1 | - | 114 | 12.9 | 8.2 | Apoptosis | Mitochondrion;Nucleus;Cytoplasm;Cytosol;Membrane fraction;Mitochondrial membrane | Receptor signaling complex scaffold activity | Cytoplasm;Mitochondrion |
| **DDX5** | NP_004387.1 | 1655 | **probable ATP-dependent RNA helicase DDX5** | 49.0 | 1 | 21 | 27 | 141 | 2.40E+09 | **0.64** | 28 | 19 | 614 | 69.1 | 8.9 | Regulation of nucleobase, nucleoside, nucleotide and nucleic acid metabolism | Nucleus;Nucleolus | RNA binding | Nucleus |
| **GEMIN2** | NP_001009183.1 | 8487 | **gem-associated protein 2 isoform gamma** | 12.8 | 3 | 2 | 2 | 2 | 2.11E+07 | **0.64** | 1 | - | 250 | 28.1 | 6.8 | Regulation of nucleobase, nucleoside, nucleotide and nucleic acid metabolism | Nucleus;Cytoplasm | Ribonucleoprotein | Nucleus |
| **PPA1** | NP_066952.1 | 5464 | **inorganic pyrophosphatase** | 74.7 | 1 | 16 | 16 | 99 | 2.23E+09 | **0.64** | 27 | 13.5 | 289 | 32.6 | 5.9 | Metabolism;Energy pathways | Cytoplasm;Mitochondrion | Catalytic activity | Cytoplasm |
| **SON** | NP_620305.2 | 6651 | **protein SON isoform F** | 10.7 | 4 | 12 | 12 | 26 | 2.93E+08 | **0.64** | 11 | 24.9 | 2426 | 263.7 | 5.6 | Regulation of nucleobase, nucleoside, nucleotide and nucleic acid metabolism | Nucleus;Cytoplasm | Transcription factor activity | Nucleus |
| **ATP6** | YP_003024031.1 | 4508 | **ATP synthase F0 subunit 6** | 4.4 | 1 | 1 | 1 | 2 | 1.39E+08 | **0.64** | 1 | - | 226 | 24.8 | 10.1 | Metabolism;Energy pathways | Mitochondrion | Transporter activity | Mitochondrion |
| **PAPD5** | NP_001035375.2 | 64282 | **non-canonical poly(A) RNA polymerase PAPD5 isoform b** | 3.8 | 2 | 1 | 1 | 2 | 6.99E+07 | **0.64** | 1 | - | 651 | 70.8 | 9.5 | Biological_process unknown | Nucleus;Nucleolus | Molecular function unknown | Nucleus |
| **PTRH2** | NP_057161.1 | 51651 | **peptidyl-tRNA hydrolase 2, mitochondrial precursor** | 45.3 | 1 | 5 | 5 | 14 | 1.51E+08 | **0.64** | 7 | 13.4 | 179 | 19.2 | 8.7 | Apoptosis | Cytoplasm;Mitochondrion | Hydrolase activity | Mitochondrion |
| **SH3GLB2** | NP_064530.1 | 56904 | **endophilin-B2 isoform b** | 23.8 | 2 | 6 | 6 | 7 | 1.21E+08 | **0.64** | 4 | 37 | 395 | 43.9 | 6.0 | Biological_process unknown | Cytoplasm | Molecular function unknown | Cytoplasm |
| **GEMIN6** | NP_079051.9 | 79833 | **gem-associated protein 6** | 22.2 | 1 | 3 | 3 | 4 | 5.24E+07 | **0.64** | 2 | 3.7 | 167 | 18.8 | 5.1 | Regulation of nucleobase, nucleoside, nucleotide and nucleic acid metabolism | Cytoplasm;Nucleus | Ribonucleoprotein | Cytoplasm |
| **KDM1A** | NP_055828.2 | 23028 | **lysine-specific histone demethylase 1A isoform b** | 15.0 | 2 | 7 | 7 | 18 | 3.39E+08 | **0.64** | 7 | 15.1 | 852 | 92.8 | 6.5 | Regulation of nucleobase, nucleoside, nucleotide and nucleic acid metabolism | Nucleus;Cytoplasm | Transcription regulator activity;Deacetylase activity | Nucleus;Cytoplasm |
| **MXRA7** | NP_940932.2 | 439921 | **matrix-remodeling-associated protein 7 isoform 3 precursor** | 8.8 | 1 | 2 | 2 | 4 | 8.34E+07 | **0.64** | 2 | 8.6 | 170 | 17.5 | 4.2 | Biological_process unknown | - | Molecular function unknown | - |
| **NAA25** | NP_079229.2 | 80018 | **N-alpha-acetyltransferase 25, NatB auxiliary subunit** | 14.3 | 1 | 7 | 7 | 17 | 1.70E+08 | **0.64** | 6 | 39.8 | 972 | 112.2 | 6.6 | Biological_process unknown | - | Molecular function unknown | - |
| **RSRC2** | NP_075388.2 | 65117 | **arginine/serine-rich coiled-coil protein 2** | 3.7 | 1 | 1 | 1 | 2 | 5.10E+06 | **0.64** | 1 | - | 434 | 50.5 | 11.3 | Biological_process unknown;Cell proliferation | Nucleus | Molecular function unknown;Transcription factor activity | Nucleus |
| **PUF60** | NP_001258029.1 | 22827 | **poly(U)-binding-splicing factor PUF60 isoform h** | 58.7 | 4 | 1 | 18 | 106 | 1.13E+09 | **0.64** | 2 | 5 | 499 | 54 | 5.3 | Regulation of nucleobase, nucleoside, nucleotide and nucleic acid metabolism | Nucleus | RNA binding | Nucleus |
| **CSDE1** | NP_001229822.1 | 7812 | **cold shock domain-containing protein E1 isoform 2** | 30.6 | 4 | 17 | 17 | 35 | 4.19E+08 | **0.64** | 16 | 15.7 | 767 | 85.7 | 6.2 | Regulation of gene expression, epigenetic | Cytoplasm | RNA binding | Cytoplasm |
| **ECI2** | NP_001159482.1 | 10455 | **enoyl-CoA delta isomerase 2, mitochondrial isoform 1** | 31.9 | 2 | 8 | 8 | 17 | 3.21E+08 | **0.64** | 9 | 22.3 | 364 | 40.2 | 8.8 | Fatty acid metabolism;Metabolism;Energy pathways | Peroxisome | Isomerase activity | Peroxisome |
| **ACSS2** | NP_001229322.1 | 55902 | **acetyl-coenzyme A synthetase, cytoplasmic isoform 3** | 7.8 | 3 | 4 | 4 | 5 | 2.98E+07 | **0.64** | 3 | 2.5 | 606 | 67.6 | 6.2 | Metabolism;Energy pathways | Cytoplasm | Catalytic activity | Cytoplasm |
| **HMG20A** | NP_060670.1 | 10363 | **high mobility group protein 20A** | 8.7 | 1 | 1 | 1 | 1 | 8.89E+06 | **0.64** | 1 | - | 347 | 40.1 | 6.5 | Regulation of nucleobase, nucleoside, nucleotide and nucleic acid metabolism | Nucleus | Transcription regulator activity | Nucleus |
| **MOB1B** | NP_775739.1 | 92597 | **MOB kinase activator 1B isoform 2** | 18.5 | 3 | 1 | 4 | 7 | 1.67E+08 | **0.64** | 1 | - | 216 | 25.1 | 6.7 | Biological_process unknown | - | Molecular function unknown | - |
| **SLC25A1** | NP_005975.1 | 6576 | **tricarboxylate transport protein, mitochondrial isoform a precursor** | 14.8 | 3 | 4 | 4 | 14 | 6.04E+08 | **0.64** | 3 | 0.3 | 311 | 34 | 9.9 | Transport | Mitochondrion | Transporter activity | Mitochondrion |
| **EIF2B1** | NP_001405.1 | 1967 | **translation initiation factor eIF-2B subunit alpha** | 44.9 | 1 | 9 | 9 | 28 | 2.68E+08 | **0.63** | 11 | 22.3 | 305 | 33.7 | 7.3 | Protein metabolism | Cytoplasm;Plasma membrane | Translation regulator activity | Cytoplasm |
| **HARS2** | NP_001265660.1 | 23438 | **probable histidine--tRNA ligase, mitochondrial isoform 2** | 16.2 | 3 | 3 | 7 | 11 | 4.80E+08 | **0.63** | 1 | - | 481 | 54.1 | 8.0 | Protein metabolism | Cytoplasm;Nucleus | Ligase activity | Cytoplasm |

Supplementary Table S4. List of all proteins identified in SCC-S and SCC-R cells using SequestHT and Mascot search algorithms and their gene ontology annotation

| Gene Symbol | RefSeq_Accession | Entrez Gene ID | Description | Coverage (%) | Proteins | Unique Peptides | # Peptides | PSM | Area | SCC-R /SCC-S | SCC-R /SCC-S (Count) | SCC-R /SCC-S (Variability [%]) | # AAs | MW [kDa] | calc. pI | Biological Process | Localization | Molecular Function | Primary Localization |
|---|---|---|---|---|---|---|---|---|---|---|---|---|---|---|---|---|---|---|---|
| **MAT2A** | NP_005902.1 | 4144 | **S-adenosylmethionine synthase isoform type-2** | 54.2 | 2 | 12 | 12 | 37 | 3.29E+08 | **0.63** | 10 | 20.5 | 395 | 43.6 | 6.5 | Metabolism;Energy pathways | Cytoplasm | Catalytic activity | Cytoplasm |
| **VDAC2** | NP_001171752.1 | 7417 | **voltage-dependent anion-selective channel protein 2 isoform 2** | 61.9 | 2 | 14 | 14 | 103 | 1.94E+09 | **0.63** | 31 | 11.7 | 294 | 31.5 | 7.6 | Transport | Mitochondrion;Nucleolus;Zymogen granule;Extracellular;Nucleus;Cytoplasm | Voltage-gated ion channel activity | Mitochondrion |
| **DIABLO** | NP_001265233.1 | 56616 | **diablo homolog, mitochondrial isoform 2** | 18.8 | 5 | 3 | 3 | 13 | 2.38E+08 | **0.63** | 2 | 19 | 186 | 21.2 | 4.8 | Cell communication;Signal transduction;Apoptosis | Mitochondrion;Cytoplasm | Caspase activator activity | Mitochondrion |
| **MRPL2** | NP_057034.2 | 51069 | **39S ribosomal protein L2, mitochondrial** | 23.9 | 1 | 4 | 4 | 8 | 8.34E+07 | **0.63** | 2 | 94.3 | 305 | 33.3 | 11.3 | Protein metabolism | Mitochondrion | Structural constituent of ribosome | Mitochondrion |
| **GART** | NP_000810.1 | 2618 | **trifunctional purine biosynthetic protein adenosine-3 isoform 1** | 52.3 | 2 | 38 | 38 | 147 | 1.42E+09 | **0.63** | 35 | 19.6 | 1010 | 107.7 | 6.7 | Regulation of nucleobase, nucleoside, nucleotide and nucleic acid metabolism | Cytosol | Ligase activity | Cytosol |
| **PDCD2L** | NP_115722.1 | 84306 | **programmed cell death protein 2-like** | 3.4 | 1 | 1 | 1 | 2 | 3.67E+07 | **0.63** | 1 | - | 358 | 39.4 | 4.9 | Apoptosis | Cytoplasm | Molecular function unknown | Cytoplasm |
| **SNF8** | NP_009172.2 | 11267 | **vacuolar-sorting protein SNF8** | 10.9 | 1 | 2 | 2 | 2 | 6.22E+07 | **0.63** | 1 | - | 258 | 28.8 | 6.7 | Regulation of nucleobase, nucleoside, nucleotide and nucleic acid metabolism | - | Transcription regulator activity | - |
| **SUPT7L** | NP_001269661.1 | 9913 | **STAGA complex 65 subunit gamma isoform c** | 9.7 | 3 | 1 | 1 | 1 | 3.47E+07 | **0.63** | 1 | - | 279 | 30.9 | 4.9 | Apoptosis | Nucleus | Transcription regulator activity | Nucleus |
| **ARHGEF18** | NP_056133.2 | 23370 | **rho guanine nucleotide exchange factor 18 isoform a** | 3.1 | 2 | 2 | 2 | 3 | 5.43E+07 | **0.63** | 2 | 3.3 | 1015 | 114 | 6.2 | Cell communication;Signal transduction | - | Guanyl-nucleotide exchange factor activity | - |
| **TMCO1** | NP_001243094.1 | 54499 | **transmembrane and coiled-coil domain-containing protein 1 isoform c** | 21.6 | 3 | 3 | 3 | 27 | 4.54E+08 | **0.63** | 5 | 8.9 | 176 | 19.9 | 9.8 | Biological_process unknown | Endoplasmic reticulum;Golgi apparatus;Cytosol | Molecular function unknown | Endoplasmic reticulum |
| **UBE4B** | NP_006039.2 | 10277 | **ubiquitin conjugation factor E4 B isoform 2** | 7.0 | 2 | 4 | 4 | 6 | 4.90E+07 | **0.63** | 2 | 16.6 | 1173 | 133.2 | 6.1 | Protein metabolism | - | Ubiquitin-specific protease activity | - |
| **ARAF** | NP_001645.1 | 369 | **serine/threonine-protein kinase A-Raf isoform 1** | 3.5 | 3 | 1 | 2 | 4 | 7.77E+07 | **0.63** | 1 | - | 606 | 67.5 | 9.0 | Cell communication;Signal transduction | Mitochondrion;Cytoplasm;Nucleus | Protein serine/threonine kinase activity | Cytoplasm |
| **ATF7IP** | NP_001273444.1 | 55729 | **activating transcription factor 7-interacting protein 1 isoform 4** | 6.6 | 4 | 4 | 4 | 5 | 7.48E+07 | **0.63** | 1 | - | 1105 | 118.6 | 4.6 | Regulation of nucleobase, nucleoside, nucleotide and nucleic acid metabolism | Nucleus | Transcription regulator activity | Nucleus |
| **C12orf5** | NP_065108.1 | 57103 | **fructose-2,6-bisphosphatase TIGAR** | 43.3 | 1 | 6 | 6 | 19 | 8.84E+07 | **0.63** | 5 | 33.5 | 270 | 30 | 7.7 | Metabolism;Energy pathways | - | Hydrolase activity | - |
| **NOP14** | NP_001278908.1 | 8602 | **nucleolar protein 14 isoform 2** | 13.5 | 2 | 9 | 9 | 19 | 6.56E+07 | **0.63** | 8 | 22 | 806 | 91.3 | 6.6 | Biological_process unknown | Nucleus | Molecular function unknown | Nucleus |
| **WDR5** | NP_438172.1 | 11091 | **WD repeat-containing protein 5** | 44.3 | 1 | 9 | 9 | 20 | 1.79E+08 | **0.63** | 8 | 11.3 | 334 | 36.6 | 8.3 | Cell growth and/or maintenance | Cytoplasm;Nucleolus;Nucleus | Histone binding | Cytoplasm |
| **C6orf211** | NP_078849.1 | 79624 | **UPF0364 protein C6orf211 isoform a** | 35.6 | 2 | 8 | 8 | 19 | 8.24E+07 | **0.63** | 4 | 10.3 | 441 | 51.1 | 5.8 | Biological_process unknown | - | Molecular function unknown | - |
| **LMNB1** | NP_001185486.1 | 4001 | **lamin-B1 isoform 2** | 47.3 | 1 | 1 | 18 | 68 | 8.72E+08 | **0.63** | 1 | - | 376 | 43 | 5.1 | Cell growth and/or maintenance | Nucleus;Nucleolus;Mitochondrion;Cytoplasm | Structural molecule activity | Nucleus |
| **ZNF281** | NP_001268223.1 | 23528 | **zinc finger protein 281 isoform 2** | 1.6 | 2 | 1 | 1 | 1 | 2.15E+07 | **0.63** | 1 | - | 859 | 93.2 | 8.4 | Regulation of nucleobase, nucleoside, nucleotide and nucleic acid metabolism | Nucleus | Transcription regulator activity | Nucleus |
| **MTA2** | NP_004730.2 | 9219 | **metastasis-associated protein MTA2** | 34.1 | 1 | 15 | 17 | 38 | 3.34E+08 | **0.63** | 12 | 18.6 | 668 | 75 | 9.7 | Regulation of nucleobase, nucleoside, nucleotide and nucleic acid metabolism;Chromosome organization and biogenesis (sensu Eukarya) | Nucleus;Golgi apparatus | DNA binding | Nucleus |
| **PAPOLA** | NP_116021.2 | 10914 | **poly(A) polymerase alpha isoform 1** | 15.3 | 4 | 7 | 7 | 11 | 1.12E+08 | **0.63** | 8 | 19.3 | 745 | 82.8 | 7.4 | Regulation of nucleobase, nucleoside, nucleotide and nucleic acid metabolism | - | RNA binding | - |
| **DARS2** | NP_060592.2 | 55157 | **aspartate--tRNA ligase, mitochondrial** | 7.3 | 1 | 4 | 4 | 6 | 1.36E+08 | **0.63** | 5 | 12.2 | 645 | 73.5 | 8.0 | Biological_process unknown | Mitochondrion | Molecular function unknown | Mitochondrion |
| **GGCT** | NP_076956.1 | 79017 | **gamma-glutamylcyclotransferase isoform 1** | 69.7 | 4 | 9 | 9 | 17 | 1.28E+08 | **0.63** | 8 | 29.2 | 188 | 21 | 5.1 | Biological_process unknown | - | Molecular function unknown | - |
| **NOL11** | NP_056277.2 | 25926 | **nucleolar protein 11** | 8.5 | 1 | 3 | 3 | 8 | 6.06E+07 | **0.63** | 6 | 7.4 | 719 | 81.1 | 6.1 | Biological_process unknown | Nucleolus | Molecular function unknown | Nucleolus |
| **RAB18** | NP_067075.1 | 22931 | **ras-related protein Rab-18 isoform 1** | 25.2 | 5 | 4 | 4 | 10 | 9.26E+07 | **0.63** | 4 | 28.8 | 206 | 23 | 5.2 | Cell communication;Signal transduction | Plasma membrane | GTPase activity | Plasma membrane |
| **RNF20** | NP_062538.5 | 56254 | **E3 ubiquitin-protein ligase BRE1A** | 9.1 | 1 | 5 | 6 | 9 | 1.49E+08 | **0.63** | 5 | 38.2 | 975 | 113.6 | 5.9 | Protein metabolism | Nucleus;Nucleolus | Ubiquitin-specific protease activity | Nucleus |
| **SNW1** | NP_036377.1 | 22938 | **SNW domain-containing protein 1** | 36.2 | 1 | 14 | 14 | 35 | 1.40E+08 | **0.63** | 10 | 7.1 | 536 | 61.5 | 9.5 | Regulation of nucleobase, nucleoside, nucleotide and nucleic acid metabolism | Nucleus;Nucleolus | Transcription regulator activity | Nucleus |
| **USE1** | NP_060937.1 | 55850 | **vesicle transport protein USE1** | 12.7 | 1 | 2 | 2 | 4 | 4.73E+07 | **0.63** | 2 | 6.1 | 259 | 29.3 | 9.1 | Biological_process unknown | - | Molecular function unknown | - |
| **ALDH16A1** | NP_001138868.1 | 126133 | **aldehyde dehydrogenase family 16 member A1 isoform 2** | 22.8 | 2 | 7 | 7 | 17 | 3.36E+08 | **0.63** | 6 | 18.6 | 751 | 79.9 | 7.6 | Biological_process unknown | - | Molecular function unknown | - |
| **VAPB** | NP_004729.1 | 9217 | **vesicle-associated membrane protein-associated protein B/C isoform 1** | 39.5 | 2 | 6 | 7 | 19 | 3.35E+08 | **0.63** | 8 | 31.4 | 243 | 27.2 | 7.3 | Transport | Plasma membrane;Membrane fraction;Mitochondrion | Auxiliary transport protein activity | Plasma membrane |
| **WIBG** | NP_001137325.1 | 84305 | **partner of Y14 and mago isoform 2** | 50.7 | 2 | 6 | 6 | 23 | 2.46E+08 | **0.63** | 9 | 28.4 | 203 | 22.7 | 9.4 | Biological_process unknown | - | Molecular function unknown | - |
| **PPP2R1B** | NP_002707.3 | 5519 | **serine/threonine-protein phosphatase 2A 65 kDa regulatory subunit A beta isoform isoform a** | 28.6 | 5 | 5 | 11 | 55 | 2.13E+08 | **0.62** | 4 | 12.4 | 601 | 66.2 | 4.9 | Cell communication;Signal transduction;Regulation of cell proliferation | Membrane fraction;Cytoplasm;Nucleus;Microtubule | Protein serine/threonine phosphatase activity;Enzyme regulator activity | Cytoplasm |
| **SUCLA2** | NP_003841.1 | 8803 | **succinyl-CoA ligase [ADP-forming] subunit beta, mitochondrial precursor** | 27.2 | 1 | 8 | 8 | 18 | 4.43E+08 | **0.62** | 10 | 32 | 463 | 50.3 | 7.4 | Metabolism;Energy pathways | Mitochondrion;Cytoplasm | Ligase activity | Mitochondrion |
| **TBRG4** | NP_004740.2 | 9238 | **protein TBRG4 isoform 1** | 21.9 | 3 | 10 | 10 | 27 | 3.34E+08 | **0.62** | 14 | 18.8 | 631 | 70.7 | 7.4 | Cell communication;Signal transduction | Mitochondrion;Nucleus | Molecular function unknown | Mitochondrion;Nucleus |
| **RPL31** | NP_000984.1 | 6160 | **60S ribosomal protein L31 isoform 1** | 48.0 | 3 | 7 | 7 | 60 | 1.28E+09 | **0.62** | 9 | 21.5 | 125 | 14.5 | 10.5 | Protein metabolism | Ribosome;Nucleolus | Structural constituent of ribosome | Ribosome |
| **CCDC124** | NP_612451.1 | 115098 | **coiled-coil domain-containing protein 124** | 20.6 | 1 | 3 | 3 | 3 | 1.28E+08 | **0.62** | 3 | 11.5 | 223 | 25.8 | 9.5 | Biological_process unknown | - | Molecular function unknown | - |
| **MYL6** | NP_524147.2 | 4637 | **myosin light polypeptide 6 isoform 2** | 87.4 | 4 | 1 | 13 | 408 | 6.64E+09 | **0.62** | 1 | - | 151 | 17 | 4.6 | Cell growth and/or maintenance | Cytoskeleton;Cytoplasm | Structural constituent of cytoskeleton | Cytoplasm |

| Gene Symbol | RefSeq_Accession | Entrez Gene ID | Description | Coverage (%) | Proteins | Unique Peptides | # Peptides | PSM | Area | SCC-R /SCC-S | SCC-R /SCC-S (Count) | SCC-R /SCC-S (Variability [%]) | # AAs | MW [kDa] | calc. pI | Biological Process | Localization | Molecular Function | Primary Localization |
|---|---|---|---|---|---|---|---|---|---|---|---|---|---|---|---|---|---|---|---|
| **PANK3** | NP_078870.1 | 79646 | **pantothenate kinase 3** | 3.8 | 2 | 1 | 1 | 1 | 4.14E+06 | **0.62** | 1 | - | 370 | 41.1 | 6.6 | Metabolism;Energy pathways | - | Catalytic activity | - |
| **RPL9** | NP_000652.2 | 6133 | **60S ribosomal protein L9** | 67.2 | 1 | 12 | 12 | 66 | 1.07E+09 | **0.62** | 19 | 11.5 | 192 | 21.8 | 10.0 | Protein metabolism | Ribosome;Nucleolus;Mitochondrion | Structural constituent of ribosome | Ribosome |
| **RRBP1** | NP_001036041.1 | 6238 | **ribosome-binding protein 1** | 45.9 | 1 | 34 | 34 | 104 | 5.61E+08 | **0.62** | 40 | 22.3 | 977 | 108.6 | 5.5 | Transport | Endoplasmic reticulum;Nucleus;Cytoplasm | Transmembrane receptor activity | Endoplasmic reticulum;Nucleus;Cytoplasm |
| **SEC22A** | NP_036562.2 | 26984 | **vesicle-trafficking protein SEC22a** | 8.8 | 1 | 1 | 1 | 1 | 1.61E+07 | **0.62** | 1 | - | 307 | 34.9 | 8.2 | Transport | Integral to membrane;Endoplasmic reticulum;Golgi apparatus | Transporter activity | Integral to membrane |
| **TRMT10C** | NP_060289.2 | 54931 | **mitochondrial ribonuclease P protein 1 precursor** | 20.1 | 1 | 6 | 6 | 15 | 2.35E+08 | **0.62** | 8 | 16.4 | 403 | 47.3 | 9.4 | Regulation of nucleobase, nucleoside, nucleotide and nucleic acid metabolism | Nucleus | RNA methyltransferase activity | Nucleus |
| **TTLL12** | NP_055955.1 | 23170 | **tubulin--tyrosine ligase-like protein 12** | 36.0 | 1 | 13 | 13 | 30 | 5.41E+08 | **0.62** | 11 | 15.7 | 644 | 74.4 | 5.5 | Metabolism;Energy pathways | Nucleus;Cytoplasm | Ligase activity | Nucleus;Cytoplasm |
| **KIAA0020** | NP_055693.4 | 9933 | **pumilio domain-containing protein KIAA0020** | 31.9 | 1 | 14 | 14 | 30 | 1.75E+08 | **0.62** | 15 | 20 | 648 | 73.5 | 9.6 | Regulation of nucleobase, nucleoside, nucleotide and nucleic acid metabolism | Nucleus;Nucleolus | RNA binding | Nucleus |
| **PUS1** | NP_001002019.1 | 80324 | **tRNA pseudouridine synthase A, mitochondrial isoform 2** | 15.5 | 2 | 4 | 4 | 5 | 9.66E+07 | **0.62** | 3 | 27.6 | 399 | 44.3 | 7.7 | Regulation of nucleobase, nucleoside, nucleotide and nucleic acid metabolism | Nucleus;Cytoplasm;Mitochondrion | Catalytic activity | Nucleus |
| **RAB10** | NP_057215.3 | 10890 | **ras-related protein Rab-10** | 38.0 | 15 | 6 | 9 | 46 | 8.14E+08 | **0.62** | 13 | 24.3 | 200 | 22.5 | 8.4 | Cell communication;Signal transduction | Nucleus;Cytoplasm;Golgi apparatus;Endosome;Golgi vesicle;Mitochondrion | GTPase activity | Nucleus;Golgi vesicle |
| **THOC2** | NP_001075019.1 | 57187 | **THO complex subunit 2** | 5.9 | 1 | 8 | 8 | 14 | 1.10E+08 | **0.62** | 3 | 7.1 | 1593 | 182.7 | 8.4 | Regulation of nucleobase, nucleoside, nucleotide and nucleic acid metabolism | Nucleus;Mitochondrion;Cytoplasm | Transcription factor activity | Nucleus |
| **VDAC1** | NP_003365.1 | 7416 | **voltage-dependent anion-selective channel protein 1** | 59.4 | 1 | 13 | 13 | 55 | 1.86E+09 | **0.62** | 23 | 9.6 | 283 | 30.8 | 8.5 | Transport | Mitochondrion;Plasma membrane;Endosome;Nucleolus;Zymogen granule | Voltage-gated ion channel activity | Mitochondrion |
| **KIF2C** | NP_006836.2 | 11004 | **kinesin-like protein KIF2C** | 4.0 | 1 | 2 | 2 | 3 | 3.57E+07 | **0.62** | 1 | - | 725 | 81.3 | 7.8 | Cell growth and/or maintenance | Cytoplasm;Nucleus;Nucleolus;Kinetochore | Motor activity | Cytoplasm |
| **SPECC1** | NP_001028727.1 | 92521 | **cytospin-B isoform 2** | 6.5 | 5 | 4 | 4 | 5 | 1.66E+07 | **0.62** | 1 | - | 987 | 110.2 | 5.9 | Biological_process unknown | Nucleus;Cytoplasm | Molecular function unknown | Nucleus;Cytoplasm |
| **ZZEF1** | NP_055928.3 | 23140 | **zinc finger ZZ-type and EF-hand domain-containing protein 1** | 3.1 | 1 | 5 | 5 | 6 | 2.21E+07 | **0.62** | 3 | 25.8 | 2961 | 330.9 | 6.0 | Biological_process unknown | - | Molecular function unknown | - |
| **VAPA** | NP_919415.2 | 9218 | **vesicle-associated membrane protein-associated protein A isoform 2** | 53.0 | 2 | 11 | 12 | 48 | 5.27E+08 | **0.62** | 20 | 25.9 | 249 | 27.9 | 8.6 | Vesicle docking;Transport | Plasma membrane;Endoplasmic reticulum membrane;Golgi membrane;Mitochondrion | Auxiliary transport protein activity | Endoplasmic reticulum membrane;Golgi membrane |
| **AARS2** | NP_065796.1 | 57505 | **alanine--tRNA ligase, mitochondrial** | 4.4 | 1 | 3 | 3 | 5 | 3.11E+07 | **0.62** | 3 | 18.6 | 985 | 107.3 | 6.3 | Metabolism;Energy pathways;Biological_process unknown | Mitochondrion | Molecular function unknown | Mitochondrion |
| **AGPS** | NP_003650.1 | 8540 | **alkyldihydroxyacetonephosphate synthase, peroxisomal precursor** | 32.1 | 1 | 13 | 13 | 31 | 2.24E+08 | **0.62** | 9 | 12.2 | 658 | 72.9 | 7.3 | Metabolism;Energy pathways | Peroxisome;Mitochondrion | Transferase activity | Peroxisome |
| **AURKB** | NP_001243763.1 | 9212 | **aurora kinase B isoform 2** | 14.2 | 3 | 3 | 3 | 6 | 3.13E+07 | **0.62** | 3 | 20.3 | 303 | 34.7 | 9.1 | Cell communication;Signal transduction | Centrosome;Nucleolus;Chromosome;Mitochondrion;Kinetochore | Protein serine/threonine kinase activity | Centrosome |
| **EGFR** | NP_005219.2 | 1956 | **epidermal growth factor receptor isoform a precursor** | 34.2 | 10 | 27 | 27 | 77 | 5.38E+08 | **0.62** | 29 | 24.6 | 1210 | 134.2 | 6.7 | Cell communication;Signal transduction | Plasma membrane;Endosome;Clathrin-coated vesicle;Cytoplasm;Nucleus | Transmembrane receptor protein tyrosine kinase activity | Plasma membrane;Nucleus;Cytoplasm |
| **CLIP1** | NP_937883.1 | 6249 | **CAP-Gly domain-containing linker protein 1 isoform b** | 23.6 | 3 | 17 | 19 | 35 | 3.03E+08 | **0.62** | 16 | 25.2 | 1392 | 156.7 | 5.4 | Cell growth and/or maintenance;Mitosis | Microtubule;Kinetochore;Endosome;Cytoplasm | Structural molecule activity | Microtubule;Cytoplasm |
| **MLLT10** | NP_004632.1 | 8028 | **protein AF-10 isoform a** | 2.4 | 2 | 1 | 1 | 1 | 3.43E+07 | **0.62** | 1 | - | 1027 | 109 | 8.4 | Regulation of nucleobase, nucleoside, nucleotide and nucleic acid metabolism | Nucleus;Cytoplasm | Transcription factor activity | Nucleus |
| **PSMF1** | NP_848693.2 | 9491 | **proteasome inhibitor PI31 subunit** | 32.8 | 1 | 5 | 5 | 25 | 6.31E+08 | **0.62** | 11 | 14.7 | 271 | 29.8 | 5.7 | Protein metabolism | Nuclear membrane;Endoplasmic reticulum | Protease inhibitor activity | Nuclear membrane |
| **RPL7L1** | NP_940888.2 | 285855 | **60S ribosomal protein L7-like 1** | 29.7 | 1 | 6 | 6 | 14 | 1.04E+08 | **0.62** | 7 | 27.9 | 246 | 28.6 | 10.5 | Protein metabolism | - | Structural constituent of ribosome | - |
| **COX7A2L** | NP_004709.2 | 9167 | **cytochrome c oxidase subunit 7A-related protein, mitochondrial** | 11.4 | 1 | 1 | 1 | 3 | 6.51E+07 | **0.61** | 1 | - | 114 | 12.6 | 9.4 | Metabolism;Energy pathways | Golgi apparatus;Mitochondrion | Catalytic activity | Mitochondrion |
| **ESF1** | NP_057733.2 | 51575 | **ESF1 homolog** | 7.8 | 1 | 4 | 4 | 5 | 1.61E+07 | **0.61** | 1 | - | 851 | 98.7 | 5.1 | Biological_process unknown | Nucleolus;Nucleus;Cytoplasm | Molecular function unknown | Nucleolus |
| **MYH10** | NP_005955.3 | 4628 | **myosin-10 isoform 2** | 43.8 | 10 | 57 | 79 | 295 | 2.41E+09 | **0.61** | 57 | 16.9 | 1976 | 228.9 | 5.5 | Cell growth and/or maintenance | Cytoplasm | Structural molecule activity | Cytoplasm |
| **MYO1C** | NP_203693.3 | 4641 | **unconventional myosin-Ic isoform c** | 31.4 | 3 | 27 | 27 | 71 | 5.51E+08 | **0.61** | 30 | 16 | 1028 | 117.8 | 9.4 | Cell growth and/or maintenance | Plasma membrane;Cytoplasm;Nucleolus;Cytoskeleton | Motor activity | Plasma membrane |
| **NUFIP2** | NP_065823.1 | 57532 | **nuclear fragile X mental retardation-interacting protein 2** | 8.6 | 1 | 4 | 4 | 11 | 9.63E+07 | **0.61** | 3 | 44.6 | 695 | 76.1 | 8.7 | Regulation of nucleobase, nucleoside, nucleotide and nucleic acid metabolism | Nucleus;Cytoplasm | RNA binding | Nucleus |
| **BAG3** | NP_004272.2 | 9531 | **BAG family molecular chaperone regulator 3** | 40.2 | 1 | 11 | 11 | 21 | 1.96E+08 | **0.61** | 10 | 51 | 575 | 61.6 | 7.0 | Apoptosis | Cytoplasm | Receptor signaling complex scaffold activity | Cytoplasm |
| **GCC1** | NP_078799.2 | 79571 | **GRIP and coiled-coil domain-containing protein 1** | 2.6 | 1 | 1 | 1 | 5 | 3.96E+07 | **0.61** | 2 | 44.5 | 775 | 87.8 | 5.5 | Cell growth and/or maintenance | Golgi apparatus | Structural molecule activity | Golgi apparatus |
| **GNPNAT1** | NP_932332.1 | 64841 | **glucosamine 6-phosphate N-acetyltransferase** | 37.5 | 1 | 5 | 5 | 14 | 7.38E+07 | **0.61** | 6 | 9.2 | 184 | 20.7 | 8.0 | Metabolism;Energy pathways | - | Acyltransferase activity | - |
| **PGD** | NP_002622.2 | 5226 | **6-phosphogluconate dehydrogenase, decarboxylating** | 57.1 | 1 | 21 | 21 | 100 | 1.75E+09 | **0.61** | 31 | 22.2 | 483 | 53.1 | 7.2 | Metabolism;Energy pathways | Cytoplasm | Catalytic activity | Cytoplasm |
| **RPP38** | NP_006405.2 | 10557 | **ribonuclease P protein subunit p38** | 20.9 | 1 | 3 | 3 | 4 | 1.70E+07 | **0.61** | 3 | 4 | 283 | 31.8 | 9.9 | RNA metabolism | Nucleolus | Ribonuclease activity | Nucleolus |
| **SAR1B** | NP_001028675.1 | 51128 | **GTP-binding protein SAR1b** | 50.0 | 1 | 5 | 7 | 27 | 1.80E+08 | **0.61** | 4 | 15.4 | 198 | 22.4 | 6.1 | Cell communication;Signal transduction | Endoplasmic reticulum;Golgi apparatus | GTPase activity | Endoplasmic reticulum |
| **WAPAL** | NP_055860.1 | 23063 | **wings apart-like protein homolog** | 12.8 | 1 | 10 | 10 | 16 | 8.58E+07 | **0.61** | 7 | 68.7 | 1190 | 132.9 | 5.4 | Biological_process unknown | Nucleus;Nucleolus;Chromosome;Cytoplasm | Molecular function unknown | Nucleus |
| **BMS1** | NP_055568.3 | 9790 | **ribosome biogenesis protein BMS1 homolog** | 9.4 | 1 | 8 | 8 | 15 | 2.44E+08 | **0.61** | 6 | 37.4 | 1282 | 145.7 | 6.4 | Biological_process unknown | Nucleolus;Ribosome;Nucleus | Molecular function unknown | Nucleolus |
| **PDE8A** | NP_001230066.1 | 5151 | **high affinity cAMP-specific and IBMX-insensitive 3',5'-cyclic phosphodiesterase 8A isoform 3** | 0.9 | 3 | 1 | 1 | 1 | 2.16E+06 | **0.61** | 1 | - | 757 | 86 | 6.0 | CAMP-mediated signaling;Energy pathways | Membrane fraction;Cytoplasm | Phosphoric diester hydrolase activity | Cytoplasm |

| Gene Symbol | RefSeq_Accession | Entrez Gene ID | Description | Coverage (%) | Proteins | Unique Peptides | # Peptides | PSM | Area | SCC-R /SCC-S | SCC-R /SCC-S (Count) | SCC-R /SCC-S (Variability [%]) | # AAs | MW [kDa] | calc. pI | Biological Process | Localization | Molecular Function | Primary Localization |
|---|---|---|---|---|---|---|---|---|---|---|---|---|---|---|---|---|---|---|---|
| **YARS2** | NP_001035526.1 | 51067 | **tyrosine--tRNA ligase, mitochondrial precursor** | 41.9 | 1 | 11 | 11 | 18 | 2.21E+08 | **0.61** | 9 | 29.1 | 477 | 53.2 | 9.0 | Biological_process unknown | - | Molecular function unknown | - |
| **RFC3** | NP_853536.2 | 5983 | **replication factor C subunit 3 isoform 2** | 29.5 | 2 | 5 | 5 | 7 | 1.94E+08 | **0.61** | 6 | 20.9 | 305 | 34.7 | 8.4 | DNA replication | Nucleolus;Nucleus | DNA binding | Nucleolus |
| **UBE2O** | NP_071349.3 | 63893 | **ubiquitin-conjugating enzyme E2 O** | 6.1 | 1 | 5 | 5 | 8 | 1.25E+08 | **0.61** | 5 | 13.9 | 1292 | 141.2 | 5.1 | Protein metabolism | Cytoplasm | Ubiquitin-specific protease activity | Cytoplasm |
| **L2HGDH** | NP_079160.1 | 79944 | **L-2-hydroxyglutarate dehydrogenase, mitochondrial precursor** | 10.6 | 1 | 3 | 3 | 5 | 3.37E+07 | **0.61** | 3 | 11.6 | 463 | 50.3 | 8.2 | Metabolism;Energy pathways | Mitochondrion | Catalytic activity | Mitochondrion |
| **PLEKHF2** | NP_078889.1 | 79666 | **pleckstrin homology domain-containing family F member 2** | 25.7 | 2 | 4 | 4 | 5 | 1.11E+08 | **0.61** | 2 | 53.3 | 249 | 27.8 | 8.2 | Biological_process unknown | Cytoplasm | Molecular function unknown | Cytoplasm |
| **RAB1A** | NP_004152.1 | 5861 | **ras-related protein Rab-1A isoform 1** | 75.1 | 17 | 7 | 14 | 106 | 9.40E+08 | **0.61** | 10 | 15 | 205 | 22.7 | 6.2 | Cell communication;Signal transduction | Endoplasmic reticulum;Golgi apparatus | GTPase activity | Golgi apparatus |
| **SLK** | NP_055535.2 | 9748 | **STE20-like serine/threonine-protein kinase** | 33.5 | 1 | 21 | 22 | 52 | 2.84E+08 | **0.61** | 24 | 28.1 | 1235 | 142.6 | 5.2 | Cell communication;Signal transduction | Cytoplasm | Protein serine/threonine kinase activity | Cytoplasm |
| **ANKEF1** | NP_942093.1 | 63926 | **ankyrin repeat and EF-hand domain-containing protein 1** | 0.8 | 1 | 1 | 1 | 4 | 4.43E+07 | **0.61** | 1 | - | 776 | 86.6 | 8.3 | Biological_process unknown | - | Molecular function unknown;Calcium ion binding | - |
| **DDX41** | NP_057306.2 | 51428 | **probable ATP-dependent RNA helicase DDX41** | 13.7 | 1 | 6 | 6 | 7 | 4.90E+07 | **0.60** | 5 | 20.7 | 622 | 69.8 | 6.8 | Regulation of nucleobase, nucleoside, nucleotide and nucleic acid metabolism | Nucleolus;Nucleus;Cytoplasm | Helicase activity | Nucleolus |
| **POLR1D** | NP_057056.1 | 51082 | **DNA-directed RNA polymerases I and III subunit RPAC2 isoform 1** | 48.9 | 2 | 4 | 4 | 7 | 3.38E+07 | **0.60** | 1 | - | 133 | 15.2 | 5.8 | Regulation of nucleobase, nucleoside, nucleotide and nucleic acid metabolism | Nucleolus;Nucleus | DNA-directed RNA polymerase activity | Nucleolus |
| **TMEM87A** | NP_001273416.1 | 25963 | **transmembrane protein 87A isoform 3** | 9.5 | 2 | 3 | 3 | 4 | 6.37E+07 | **0.60** | 2 | 6.4 | 494 | 56.7 | 5.7 | Biological_process unknown | Integral to membrane | Molecular function unknown | Integral to membrane |
| **PIGK** | NP_005473.1 | 10026 | **GPI-anchor transamidase precursor** | 22.5 | 1 | 5 | 5 | 7 | 4.66E+07 | **0.60** | 3 | 14.1 | 395 | 45.2 | 6.2 | Protein metabolism | Endoplasmic reticulum | Cysteine-type peptidase activity | Endoplasmic reticulum |
| **PTRH1** | NP_001002913.1 | 138428 | **probable peptidyl-tRNA hydrolase** | 11.7 | 1 | 1 | 1 | 6 | 1.02E+08 | **0.60** | 1 | - | 214 | 22.9 | 10.6 | Metabolism;Energy pathways | - | Hydrolase activity | - |
| **CNNM2** | NP_951059.1 | 54805 | **metal transporter CNNM2 isoform 3** | 2.5 | 4 | 1 | 1 | 1 | 9.39E+06 | **0.60** | 1 | - | 552 | 60.4 | 7.1 | Cell communication;Signal transduction | Nucleus | Molecular function unknown | Nucleus |
| **MCM5** | NP_006730.2 | 4174 | **DNA replication licensing factor MCM5** | 33.4 | 1 | 21 | 21 | 69 | 8.56E+08 | **0.60** | 24 | 27.5 | 734 | 82.2 | 8.4 | Regulation of nucleobase, nucleoside, nucleotide and nucleic acid metabolism | Nucleus;Nucleolus;Chromosome;Mitochondrion | DNA binding | Nucleus |
| **TAF6L** | NP_006464.1 | 10629 | **TAF6-like RNA polymerase II p300/CBP-associated factor-associated factor 65 kDa subunit 6L** | 7.2 | 1 | 2 | 2 | 2 | 1.70E+07 | **0.60** | 1 | - | 622 | 67.8 | 9.0 | Regulation of nucleobase, nucleoside, nucleotide and nucleic acid metabolism | Nucleus | DNA-directed RNA polymerase activity | Nucleus |
| **ZMPSTE24** | NP_005848.2 | 10269 | **CAAX prenyl protease 1 homolog** | 12.2 | 1 | 4 | 4 | 9 | 6.95E+07 | **0.60** | 3 | 19.9 | 475 | 54.8 | 7.5 | Protein metabolism | Endoplasmic reticulum;Golgi apparatus;Mitochondrion | Metallopeptidase activity | Endoplasmic reticulum |
| **ATL3** | NP_056274.3 | 25923 | **atlastin-3 isoform 1** | 32.4 | 2 | 10 | 10 | 19 | 1.42E+08 | **0.60** | 9 | 48.9 | 541 | 60.5 | 5.7 | Immune response | Mitochondrion | Molecular function unknown | Mitochondrion |
| **ATP5F1** | NP_001679.2 | 515 | **ATP synthase F(0) complex subunit B1, mitochondrial precursor** | 10.6 | 1 | 2 | 2 | 11 | 2.07E+08 | **0.60** | 4 | 27.3 | 256 | 28.9 | 9.4 | Metabolism;Energy pathways | Mitochondrion | Transporter activity | Mitochondrion |
| **CAPN1** | NP_001185798.1 | 823 | **calpain-1 catalytic subunit** | 36.1 | 1 | 18 | 18 | 61 | 1.47E+09 | **0.60** | 25 | 23 | 714 | 81.8 | 5.7 | Protein metabolism | Cytoplasm;Plasma membrane;Nucleus | Cysteine-type peptidase activity | Cytoplasm |
| **DDX47** | NP_057439.2 | 51202 | **probable ATP-dependent RNA helicase DDX47 isoform 1** | 30.1 | 2 | 11 | 11 | 29 | 2.85E+08 | **0.60** | 11 | 37.9 | 455 | 50.6 | 9.1 | Regulation of nucleobase, nucleoside, nucleotide and nucleic acid metabolism;Ribosome biogenesis and assembly | Nucleus;Cytoplasm;Nucleolus | Molecular function unknown | Nucleus |
| **MTFR1** | NP_001139310.1 | 9650 | **mitochondrial fission regulator 1 isoform 2** | 8.0 | 3 | 1 | 1 | 1 | 7.08E+06 | **0.60** | 1 | - | 300 | 33.2 | 7.5 | Biological_process unknown | Mitochondrion | Molecular function unknown | Mitochondrion |
| **ZHX1** | NP_001017926.1 | 11244 | **zinc fingers and homeoboxes protein 1** | 6.6 | 1 | 2 | 2 | 2 | 2.75E+07 | **0.60** | 1 | - | 873 | 98 | 6.1 | Regulation of nucleobase, nucleoside, nucleotide and nucleic acid metabolism | Nucleus | Transcription factor activity | Nucleus |
| **CCDC53** | NP_057137.1 | 51019 | **WASH complex subunit CCDC53** | 12.4 | 1 | 1 | 1 | 1 | 7.55E+07 | **0.60** | 1 | - | 194 | 21.2 | 4.5 | Biological_process unknown | - | Molecular function unknown | - |
| **DOCK6** | NP_065863.2 | 57572 | **dedicator of cytokinesis protein 6** | 1.0 | 1 | 1 | 2 | 2 | 2.02E+07 | **0.60** | 1 | - | 2047 | 229.4 | 6.7 | Cell communication;Signal transduction | - | Guanyl-nucleotide exchange factor activity | - |
| **EXOC6B** | NP_056004.1 | 23233 | **exocyst complex component 6B** | 2.3 | 1 | 1 | 1 | 4 | 4.24E+07 | **0.60** | 2 | 3.5 | 811 | 94.1 | 6.5 | Cell-cell signaling | - | Molecular function unknown | - |
| **HSD17B4** | NP_000405.1 | 3295 | **peroxisomal multifunctional enzyme type 2 isoform 2** | 34.2 | 4 | 16 | 16 | 43 | 2.90E+08 | **0.60** | 12 | 29.2 | 736 | 79.6 | 8.8 | Metabolism;Energy pathways | Peroxisome;Cytoplasm;Nucleolus;Mitochondrion | Catalytic activity | Peroxisome |
| **INPP1** | NP_002185.1 | 3628 | **inositol polyphosphate 1-phosphatase** | 15.0 | 1 | 4 | 4 | 6 | 4.62E+07 | **0.60** | 1 | - | 399 | 44 | 5.3 | Cell communication;Signal transduction | Cytoplasm | Catalytic activity | Cytoplasm |
| **MORC3** | NP_056173.1 | 23515 | **MORC family CW-type zinc finger protein 3** | 6.1 | 1 | 3 | 3 | 3 | 1.08E+08 | **0.60** | 2 | 3.6 | 939 | 107 | 5.6 | Regulation of nucleobase, nucleoside, nucleotide and nucleic acid metabolism | Nucleus | RNA binding | Nucleus |
| **RCL1** | NP_005763.3 | 10171 | **RNA 3'-terminal phosphate cyclase-like protein isoform a** | 11.5 | 2 | 3 | 3 | 6 | 1.05E+08 | **0.60** | 3 | 29.5 | 373 | 40.8 | 9.3 | Regulation of nucleobase, nucleoside, nucleotide and nucleic acid metabolism | Nucleus;Nucleolus | RNA binding | Nucleus |
| **SEC24C** | NP_004913.2 | 9632 | **protein transport protein Sec24C** | 32.5 | 1 | 21 | 21 | 52 | 6.54E+08 | **0.60** | 11 | 9.3 | 1094 | 118.2 | 7.1 | Transport | Endoplasmic reticulum;Cytoplasm | Transporter activity | Endoplasmic reticulum |
| **FLOT1** | NP_005794.1 | 10211 | **flotillin-1** | 42.9 | 1 | 12 | 12 | 27 | 3.08E+08 | **0.60** | 12 | 10.8 | 427 | 47.3 | 7.5 | Cell growth and/or maintenance | Plasma membrane;Kinetochore;Cytoplasm | Molecular function unknown | Plasma membrane |
| **NACC1** | NP_443108.1 | 112939 | **nucleus accumbens-associated protein 1** | 5.7 | 2 | 2 | 2 | 4 | 3.15E+07 | **0.60** | 2 | 14.8 | 527 | 57.2 | 5.7 | Biological_process unknown | Nucleus | Molecular function unknown | Nucleus |
| **GTF3C5** | NP_036219.2 | 9328 | **general transcription factor 3C polypeptide 5 isoform 2** | 12.5 | 3 | 4 | 4 | 5 | 2.55E+07 | **0.60** | 2 | 7 | 519 | 59.5 | 6.9 | Regulation of nucleobase, nucleoside, nucleotide and nucleic acid metabolism | Nucleus | Transcription factor activity | Nucleus |
| **HIST2H2AB** | NP_778235.1 | 317772 | **histone H2A type 2-B** | 37.7 | 2 | 3 | 5 | 74 | 1.72E+09 | **0.60** | 16 | 21.5 | 130 | 14 | 10.9 | Regulation of nucleobase, nucleoside, nucleotide and nucleic acid metabolism | - | DNA binding | - |
| **UTP14A** | NP_001159693.1 | 10813 | **U3 small nucleolar RNA-associated protein 14 homolog A isoform 2** | 30.9 | 3 | 17 | 17 | 35 | 2.11E+08 | **0.60** | 13 | 16.4 | 719 | 82 | 7.9 | Biological_process unknown | Ribosome | Molecular function unknown | Ribosome |
| **VTA1** | NP_001273300.1 | 51534 | **vacuolar protein sorting-associated protein VTA1 homolog isoform b** | 22.5 | 3 | 4 | 4 | 11 | 1.43E+08 | **0.60** | 5 | 23.8 | 280 | 31.1 | 6.4 | Biological_process unknown | - | Molecular function unknown | - |

| Gene Symbol | RefSeq_Accession | Entrez Gene ID | Description | Coverage (%) | Proteins | Unique Peptides | # Peptides | PSM | Area | SCC-R /SCC-S | SCC-R /SCC-S (Count) | SCC-R /SCC-S (Variability [%]) | # AAs | MW [kDa] | calc. pI | Biological Process | Localization | Molecular Function | Primary Localization |
|---|---|---|---|---|---|---|---|---|---|---|---|---|---|---|---|---|---|---|---|
| **MRPL55** | NP_852121.1 | 128308 | **39S ribosomal protein L55, mitochondrial isoform a** | 25.0 | 2 | 2 | 2 | 5 | 1.45E+08 | **0.59** | 4 | 13.3 | 128 | 15.1 | 11.2 | Protein metabolism | Mitochondrion | Structural constituent of ribosome | Mitochondrion |
| **PPFIBP1** | NP_001185845.1 | 8496 | **liprin-beta-1 isoform 4** | 20.6 | 4 | 12 | 12 | 23 | 2.70E+08 | **0.59** | 7 | 6.4 | 980 | 110.2 | 5.4 | Cell communication;Signal transduction | Plasma membrane;Cytoplasm;Nucleus | Cytoskeletal anchoring activity | Plasma membrane |
| **RPL24** | NP_000977.1 | 6152 | **60S ribosomal protein L24** | 26.1 | 1 | 6 | 6 | 45 | 2.23E+09 | **0.59** | 14 | 13.4 | 157 | 17.8 | 11.3 | Protein metabolism | Ribosome | Structural constituent of ribosome | Ribosome |
| **ABCF2** | NP_009120.1 | 10061 | **ATP-binding cassette sub-family F member 2 isoform a** | 22.8 | 2 | 11 | 11 | 24 | 3.06E+08 | **0.59** | 10 | 37 | 623 | 71.2 | 7.4 | Immune response | Mitochondrion;Peroxisome | Molecular function unknown | Mitochondrion |
| **METTL2A** | NP_859076.3 | 339175 | **methyltransferase-like protein 2A** | 4.0 | 2 | 1 | 1 | 1 | 1.37E+06 | **0.59** | 1 | - | 378 | 43.5 | 6.0 | Biological_process unknown | - | Molecular function unknown | - |
| **POR** | NP_000932.3 | 5447 | **NADPH--cytochrome P450 reductase** | 26.2 | 1 | 10 | 10 | 24 | 2.44E+08 | **0.59** | 14 | 27.4 | 680 | 77 | 5.6 | Metabolism;Energy pathways | Endoplasmic reticulum;Plasma membrane;Mitochondrion | Catalytic activity | Endoplasmic reticulum |
| **UTP15** | NP_115551.2 | 84135 | **U3 small nucleolar RNA-associated protein 15 homolog isoform 1** | 15.8 | 3 | 5 | 5 | 9 | 6.89E+07 | **0.59** | 3 | 95.3 | 518 | 58.4 | 9.1 | Cell communication | - | Molecular function unknown | - |
| **C10orf76** | NP_078817.2 | 79591 | **UPF0668 protein C10orf76** | 7.7 | 1 | 3 | 3 | 7 | 5.12E+07 | **0.59** | 5 | 31.2 | 689 | 78.7 | 6.6 | Biological_process unknown | - | Molecular function unknown | - |
| **MSH2** | NP_001245210.1 | 4436 | **DNA mismatch repair protein Msh2 isoform 2** | 13.3 | 2 | 9 | 9 | 24 | 1.93E+08 | **0.59** | 9 | 10.3 | 868 | 97.3 | 5.8 | Regulation of nucleobase, nucleoside, nucleotide and nucleic acid metabolism | Nucleus | DNA repair protein | Nucleus |
| **AK3** | NP_057366.2 | 50808 | **GTP:AMP phosphotransferase AK3, mitochondrial isoform a** | 18.5 | 3 | 3 | 3 | 3 | 4.41E+07 | **0.59** | 1 | - | 227 | 25.5 | 9.2 | Metabolism;Energy pathways | Mitochondrion | Catalytic activity | Mitochondrion |
| **TMEM33** | NP_060596.2 | 55161 | **transmembrane protein 33** | 17.8 | 1 | 3 | 3 | 18 | 1.72E+08 | **0.59** | 7 | 34.6 | 247 | 28 | 9.7 | Biological_process unknown | Membrane fraction;Integral to membrane | Molecular function unknown | Membrane fraction |
| **DRG1** | NP_004138.1 | 4733 | **developmentally-regulated GTP-binding protein 1** | 27.5 | 1 | 8 | 8 | 18 | 3.45E+08 | **0.59** | 8 | 11.7 | 367 | 40.5 | 8.9 | Cell growth and/or maintenance | Cytoplasm | Molecular function unknown | Cytoplasm |
| **RPL35** | NP_009140.1 | 11224 | **60S ribosomal protein L35** | 22.8 | 1 | 4 | 4 | 41 | 3.28E+09 | **0.59** | 15 | 9.8 | 123 | 14.5 | 11.1 | Protein metabolism | Ribosome;Nucleolus | Structural constituent of ribosome | Ribosome |
| **UBE2F** | NP_001265237.1 | 140739 | **NEDD8-conjugating enzyme UBE2F isoform 4** | 8.5 | 2 | 1 | 1 | 1 | 4.44E+07 | **0.59** | 1 | - | 164 | 18.6 | 8.1 | Protein metabolism | - | Ubiquitin-specific protease activity | - |
| **VPS45** | NP_001266284.1 | 11311 | **vacuolar protein sorting-associated protein 45 isoform 4** | 22.2 | 4 | 5 | 5 | 5 | 7.59E+07 | **0.59** | 2 | 218.4 | 447 | 50.7 | 8.9 | Transport | Golgi apparatus | Transporter activity | Golgi apparatus |
| **ACTN1** | NP_001123477.1 | 87 | **alpha-actinin-1 isoform c** | 71.1 | 3 | 41 | 69 | 644 | 5.31E+09 | **0.59** | 89 | 15.3 | 887 | 102.6 | 5.5 | Cell growth and/or maintenance | Cytoplasm;Mitochondrial membrane;Cytosol;Cytoskeleton;Mitochondrion;Nucleus | Cytoskeletal protein binding | Cytoplasm |
| **MRPL49** | NP_004918.1 | 740 | **39S ribosomal protein L49, mitochondrial** | 23.5 | 1 | 3 | 3 | 8 | 4.54E+07 | **0.59** | 1 | - | 166 | 19.2 | 9.5 | Protein metabolism | Mitochondrion | Structural constituent of ribosome | Mitochondrion |
| **PTPN1** | NP_002818.1 | 5770 | **tyrosine-protein phosphatase non-receptor type 1 isoform 1** | 33.1 | 2 | 8 | 8 | 14 | 1.83E+08 | **0.59** | 5 | 10.4 | 435 | 49.9 | 6.3 | Cell communication;Signal transduction | Endoplasmic reticulum;Cytoplasm | Protein tyrosine phosphatase activity | Endoplasmic reticulum |
| **C12orf43** | NP_001273127.1 | 64897 | **uncharacterized protein C12orf43 isoform g** | 29.0 | 7 | 3 | 3 | 8 | 3.21E+07 | **0.59** | 2 | 31.8 | 221 | 23.9 | 9.7 | Biological_process unknown | Nucleus | Molecular function unknown | Nucleus |
| **NSUN6** | NP_872349.1 | 221078 | **putative methyltransferase NSUN6** | 4.9 | 1 | 1 | 1 | 2 | 8.51E+06 | **0.59** | 1 | - | 469 | 51.7 | 8.0 | Biological_process unknown | - | Molecular function unknown | - |
| **TES** | NP_690042.1 | 26136 | **testin isoform 2** | 32.5 | 2 | 11 | 11 | 30 | 5.28E+08 | **0.59** | 13 | 14 | 412 | 46.9 | 7.6 | Cell growth and/or maintenance | Extracellular;Plasma membrane | Molecular function unknown | Extracellular |
| **WDR43** | NP_055946.1 | 23160 | **WD repeat-containing protein 43** | 31.6 | 1 | 11 | 11 | 30 | 3.48E+08 | **0.59** | 13 | 30 | 677 | 74.8 | 5.6 | Biological_process unknown | - | Molecular function unknown | - |
| **KMT2C** | NP_733751.2 | 58508 | **histone-lysine N-methyltransferase 2C** | 0.1 | 1 | 1 | 1 | 1 | 7.38E+07 | **0.59** | 1 | - | 4911 | 541 | 6.5 | Regulation of nucleobase, nucleoside, nucleotide and nucleic acid metabolism | Nucleus | Transcription regulator activity | Nucleus |
| **SH3GL1** | NP_003016.1 | 6455 | **endophilin-A2 isoform 1** | 40.0 | 4 | 12 | 12 | 25 | 3.32E+08 | **0.59** | 13 | 20.3 | 368 | 41.5 | 5.4 | Cell communication;Signal transduction | Cytoplasm;Nucleus;Cytosol | Molecular function unknown | Cytoplasm |
| **TMEM41B** | NP_001158502.1 | 440026 | **transmembrane protein 41B isoform 2** | 15.0 | 2 | 1 | 1 | 3 | 3.67E+07 | **0.59** | 1 | - | 127 | 14.1 | 9.4 | Biological_process unknown | Integral to membrane | Molecular function unknown | Integral to membrane |
| **TOMM70A** | NP_055635.3 | 9868 | **mitochondrial import receptor subunit TOM70** | 48.2 | 1 | 17 | 17 | 33 | 1.79E+08 | **0.59** | 14 | 52.2 | 608 | 67.4 | 7.1 | Transport | Mitochondrion | Auxiliary transport protein activity | Mitochondrion |
| **GBF1** | NP_001186308.1 | 8729 | **Golgi-specific brefeldin A-resistance guanine nucleotide exchange factor 1 isoform 3** | 13.9 | 3 | 13 | 14 | 38 | 9.82E+08 | **0.59** | 11 | 11.9 | 1855 | 205.9 | 5.8 | Cell communication;Signal transduction | Golgi apparatus;Cytoplasm;Nucleus | Guanyl-nucleotide exchange factor activity | Golgi apparatus |
| **NAA35** | NP_078911.3 | 60560 | **N-alpha-acetyltransferase 35, NatC auxiliary subunit** | 8.7 | 1 | 4 | 4 | 6 | 6.82E+07 | **0.59** | 3 | 44.6 | 725 | 83.6 | 7.1 | Cell growth and/or maintenance | - | Molecular function unknown | - |
| **NOP56** | NP_006383.2 | 10528 | **nucleolar protein 56** | 41.9 | 1 | 16 | 16 | 52 | 5.34E+08 | **0.59** | 21 | 19.5 | 594 | 66 | 9.2 | Regulation of nucleobase, nucleoside, nucleotide and nucleic acid metabolism | Nucleolus;Ribosome | Molecular function unknown | Nucleolus |
| **SETD3** | NP_954574.1 | 84193 | **histone-lysine N-methyltransferase setd3 isoform b** | 19.9 | 2 | 5 | 5 | 6 | 1.04E+08 | **0.59** | 5 | 5.8 | 296 | 33.7 | 7.0 | Biological_process unknown | - | Methyltransferase activity | - |
| **USP47** | NP_060414.3 | 55031 | **ubiquitin carboxyl-terminal hydrolase 47 isoform b** | 5.2 | 2 | 4 | 4 | 4 | 6.56E+07 | **0.59** | 2 | 6.4 | 1287 | 147.1 | 5.1 | Protein metabolism | - | Ubiquitin-specific protease activity | - |
| **CLPX** | NP_006651.2 | 10845 | **ATP-dependent Clp protease ATP-binding subunit clpX-like, mitochondrial precursor** | 21.6 | 1 | 9 | 9 | 15 | 1.73E+08 | **0.58** | 7 | 25.5 | 633 | 69.2 | 7.6 | Protein metabolism | Mitochondrion | Peptidase activity | Mitochondrion |
| **RPL14** | NP_001030168.1 | 9045 | **60S ribosomal protein L14** | 27.4 | 1 | 5 | 5 | 22 | 7.03E+08 | **0.58** | 6 | 9 | 215 | 23.4 | 10.9 | Protein metabolism | Ribosome;Nucleolus | Structural constituent of ribosome | Ribosome |
| **ABCD3** | NP_002849.1 | 5825 | **ATP-binding cassette sub-family D member 3 isoform a** | 21.2 | 2 | 8 | 8 | 14 | 1.52E+08 | **0.58** | 8 | 28.3 | 659 | 75.4 | 9.4 | Transport | Peroxisome;Mitochondrion | ATP binding | Peroxisome |
| **ALKBH5** | NP_060228.3 | 54890 | **RNA demethylase ALKBH5** | 11.4 | 1 | 2 | 2 | 2 | 2.70E+07 | **0.58** | 1 | - | 394 | 44.2 | 9.1 | Biological_process unknown | Nucleus | Molecular function unknown | Nucleus |
| **EML2** | NP_036287.1 | 24139 | **echinoderm microtubule-associated protein-like 2 isoform 2** | 11.6 | 3 | 4 | 4 | 5 | 7.78E+07 | **0.58** | 3 | 30.8 | 649 | 70.6 | 6.3 | Cell growth and/or maintenance | Nucleus | Cytoskeletal protein binding | Nucleus |
| **MRPS31** | NP_005821.2 | 10240 | **28S ribosomal protein S31, mitochondrial** | 15.7 | 1 | 4 | 4 | 9 | 2.94E+07 | **0.58** | 3 | 47.8 | 395 | 45.3 | 9.3 | Protein metabolism | Mitochondrion | Structural constituent of ribosome | Mitochondrion |

| Gene Symbol | RefSeq_Accession | Entrez Gene ID | Description | Coverage (%) | Proteins | Unique Peptides | # Peptides | PSM | Area | SCC-R /SCC-S | SCC-R /SCC-S (Count) | SCC-R /SCC-S (Variability [%]) | # AAs | MW [kDa] | calc. pI | Biological Process | Localization | Molecular Function | Primary Localization |
|---|---|---|---|---|---|---|---|---|---|---|---|---|---|---|---|---|---|---|---|
| **POLB** | NP_002681.1 | 5423 | **DNA polymerase beta** | 8.1 | 1 | 2 | 2 | 2 | 2.07E+07 | **0.58** | 2 | 38.5 | 335 | 38.2 | 9.0 | Regulation of nucleobase, nucleoside, nucleotide and nucleic acid metabolism | Nucleus | DNA-directed DNA polymerase activity | Nucleus |
| **RPS16** | NP_001011.1 | 6217 | **40S ribosomal protein S16** | 39.7 | 1 | 7 | 7 | 97 | 3.32E+09 | **0.58** | 17 | 18.8 | 146 | 16.4 | 10.2 | Protein metabolism | Ribosome;Nucleolus | Structural constituent of ribosome | Ribosome |
| **MRPL14** | NP_115487.2 | 64928 | **39S ribosomal protein L14, mitochondrial** | 29.7 | 1 | 3 | 3 | 5 | 6.57E+07 | **0.58** | 3 | 39.8 | 145 | 15.9 | 10.2 | Protein metabolism | Mitochondrion | Structural constituent of ribosome | Mitochondrion |
| **MYO6** | NP_004990.3 | 4646 | **unconventional myosin-VI** | 17.7 | 1 | 11 | 11 | 17 | 1.22E+08 | **0.58** | 8 | 31.6 | 1285 | 148.6 | 8.5 | Cell growth and/or maintenance | Golgi apparatus;Cytoplasm | Motor activity | Golgi apparatus |
| **NOC4L** | NP_076983.1 | 79050 | **nucleolar complex protein 4 homolog** | 20.5 | 1 | 5 | 5 | 9 | 5.17E+07 | **0.58** | 3 | 24.1 | 516 | 58.4 | 7.5 | Biological_process unknown | - | Molecular function unknown | - |
| **MGST2** | NP_001191297.1 | 4258 | **microsomal glutathione S-transferase 2 isoform 3 precursor** | 18.2 | 2 | 1 | 1 | 2 | 2.68E+08 | **0.58** | 1 | - | 77 | 8.4 | 9.5 | Metabolism;Energy pathways | Endoplasmic reticulum | Glutathione transferase activity | Endoplasmic reticulum |
| **AP3B1** | NP_003655.3 | 8546 | **AP-3 complex subunit beta-1 isoform 1** | 20.8 | 5 | 14 | 14 | 30 | 1.79E+09 | **0.58** | 10 | 15 | 1094 | 121.2 | 6.0 | Protein metabolism | Plasma membrane;Cytoplasm;Nucleus | Receptor signaling complex scaffold activity | Plasma membrane;Nucleus;Cytoplasm |
| **HIST2H2AC** | NP_003508.1 | 8338 | **histone H2A type 2-C** | 60.5 | 8 | 4 | 9 | 1095 | 1.29E+10 | **0.58** | 41 | 16.7 | 129 | 14 | 10.9 | Regulation of nucleobase, nucleoside, nucleotide and nucleic acid metabolism | Nucleus | DNA binding | Nucleus |
| **LUC7L2** | NP_057103.2 | 51631 | **putative RNA-binding protein Luc7-like 2 isoform 1** | 36.7 | 4 | 7 | 11 | 40 | 2.83E+08 | **0.58** | 7 | 52.6 | 392 | 46.5 | 10.0 | Biological_process unknown | Nucleolus;Nucleus;Cytoplasm | Molecular function unknown | Nucleolus |
| **GTF3C4** | NP_036336.2 | 9329 | **general transcription factor 3C polypeptide 4** | 26.5 | 1 | 11 | 11 | 20 | 8.54E+07 | **0.58** | 7 | 34.8 | 822 | 91.9 | 6.7 | Regulation of nucleobase, nucleoside, nucleotide and nucleic acid metabolism | Nucleus | Transcription factor activity | Nucleus |
| **IPO4** | NP_078934.3 | 79711 | **importin-4** | 26.8 | 1 | 19 | 19 | 55 | 5.22E+08 | **0.58** | 26 | 30.7 | 1081 | 118.6 | 5.0 | Cell communication;Signal transduction | Cytoplasm;Nucleus | Transporter activity | Cytoplasm |
| **TMA7** | NP_057017.1 | 51372 | **translation machinery-associated protein 7** | 18.8 | 1 | 2 | 2 | 4 | 1.04E+08 | **0.58** | 3 | 25.2 | 64 | 7.1 | 10.0 | DNA repair;DNA replication;Hemopoiesis;Cell proliferation;Cell differentiation | Nucleus | Transcription cofactor activity | Nucleus |
| **XRCC6** | NP_001275905.1 | 2547 | **X-ray repair cross-complementing protein 6 isoform 1** | 67.0 | 3 | 44 | 44 | 265 | 2.14E+09 | **0.58** | 84 | 21.8 | 609 | 69.8 | 6.6 | Regulation of nucleobase, nucleoside, nucleotide and nucleic acid metabolism | Nucleus;Cytoplasm;Nucleolus | DNA binding | Nucleus |
| **DOLPP1** | NP_001129389.1 | 57171 | **dolichyldiphosphatase 1 isoform b** | 9.7 | 2 | 1 | 1 | 1 | 3.51E+07 | **0.58** | 1 | - | 195 | 22.1 | 9.6 | Metabolism;Energy pathways | Endoplasmic reticulum | Acid phosphatase activity | Endoplasmic reticulum |
| **RAB5B** | NP_002859.1 | 5869 | **ras-related protein Rab-5B isoform 1** | 18.1 | 2 | 2 | 3 | 7 | 1.65E+08 | **0.57** | 2 | 7.2 | 215 | 23.7 | 8.1 | Cell communication;Signal transduction | Plasma membrane;Endosome | GTPase activity | Plasma membrane |
| **RPL35A** | NP_000987.2 | 6165 | **60S ribosomal protein L35a** | 15.5 | 1 | 4 | 4 | 16 | 2.57E+08 | **0.57** | 6 | 5.6 | 110 | 12.5 | 11.1 | Protein metabolism | Ribosome;Nucleolus | Structural constituent of ribosome | Ribosome |
| **ARID1A** | NP_624361.1 | 8289 | **AT-rich interactive domain-containing protein 1A isoform b** | 3.1 | 2 | 3 | 3 | 3 | 2.56E+07 | **0.57** | 1 | - | 2068 | 218.2 | 6.5 | Regulation of nucleobase, nucleoside, nucleotide and nucleic acid metabolism | Cytoplasm;Nucleus | RNA binding | Cytoplasm |
| **CD9** | NP_001760.1 | 928 | **CD9 antigen** | 20.6 | 1 | 3 | 3 | 26 | 1.23E+09 | **0.57** | 6 | 17.4 | 228 | 25.4 | 7.2 | Immune response | Plasma membrane;Cell surface | Molecular function unknown | Cell surface |
| **DNAJC9** | NP_056005.1 | 23234 | **dnaJ homolog subfamily C member 9** | 45.8 | 1 | 8 | 8 | 19 | 3.09E+08 | **0.57** | 8 | 15.9 | 260 | 29.9 | 5.7 | Protein metabolism | - | Chaperone activity | - |
| **MTIF3** | NP_001159735.1 | 219402 | **translation initiation factor IF-3, mitochondrial** | 7.6 | 1 | 1 | 1 | 3 | 7.98E+07 | **0.57** | 1 | - | 278 | 31.7 | 9.7 | Protein metabolism | Mitochondrion | Translation regulator activity | Mitochondrion |
| **HNRNPA0** | NP_006796.1 | 10949 | **heterogeneous nuclear ribonucleoprotein A0** | 48.2 | 1 | 10 | 11 | 45 | 8.88E+08 | **0.57** | 16 | 16.7 | 305 | 30.8 | 9.3 | Regulation of nucleobase, nucleoside, nucleotide and nucleic acid metabolism | Nucleus;Nucleolus;Cytoplasm;Mitochondrion | RNA binding | Nucleus |
| **DDX3X** | NP_001180345.1 | 1654 | **ATP-dependent RNA helicase DDX3X isoform 2** | 48.3 | 8 | 22 | 23 | 83 | 1.34E+09 | **0.57** | 29 | 23.1 | 661 | 73.1 | 7.2 | Regulation of nucleobase, nucleoside, nucleotide and nucleic acid metabolism | Nucleus;Cytoplasm;Nucleolus | RNA binding | Nucleus |
| **HSPA4L** | NP_055093.2 | 22824 | **heat shock 70 kDa protein 4L** | 29.1 | 1 | 14 | 17 | 53 | 3.84E+08 | **0.57** | 12 | 24.9 | 839 | 94.5 | 5.9 | Protein metabolism | Cytoplasm | Heat shock protein activity | Cytoplasm |
| **RAB8A** | NP_005361.2 | 4218 | **ras-related protein Rab-8A** | 33.8 | 16 | 4 | 7 | 29 | 6.86E+08 | **0.57** | 6 | 17 | 207 | 23.7 | 9.1 | Cell communication;Signal transduction | Nucleus;Plasma membrane;Cytoplasm;Golgi apparatus;Zymogen granule;Mitochondrion | GTPase activity | Nucleus |
| **DHX30** | NP_055781.2 | 22907 | **putative ATP-dependent RNA helicase DHX30 isoform 2** | 16.8 | 2 | 10 | 10 | 12 | 1.09E+08 | **0.57** | 7 | 55 | 1155 | 129.4 | 8.5 | RNA metabolism | Nucleus;Mitochondrion | RNA binding | Nucleus |
| **KIF3A** | NP_008985.3 | 11127 | **kinesin-like protein KIF3A** | 3.2 | 1 | 1 | 1 | 1 | 3.97E+07 | **0.57** | 1 | - | 699 | 80 | 6.5 | Cell growth and/or maintenance | Cytoplasm | Motor activity | Cytoplasm |
| **TJP2** | NP_001163886.1 | 9414 | **tight junction protein ZO-2 isoform 4** | 21.8 | 6 | 14 | 14 | 24 | 1.10E+08 | **0.57** | 6 | 16.7 | 1157 | 130.4 | 7.6 | Cell communication;Signal transduction | Cytoplasm;Nucleus;Cell junction | Cell adhesion molecule activity | Cytoplasm |
| **YBX3** | NP_003642.3 | 8531 | **Y-box-binding protein 3 isoform a** | 71.0 | 2 | 3 | 19 | 92 | 9.22E+08 | **0.57** | 2 | 10.2 | 372 | 40.1 | 9.8 | Regulation of nucleobase, nucleoside, nucleotide and nucleic acid metabolism | Nucleus;Cytoplasm | DNA binding | Nucleus |
| **GCN1L1** | NP_006827.1 | 10985 | **translational activator GCN1** | 41.4 | 1 | 83 | 83 | 202 | 1.08E+09 | **0.57** | 82 | 23.6 | 2671 | 292.5 | 7.4 | Protein metabolism | Nucleolus;Nucleus | Translation regulator activity | Nucleolus |
| **RAB3IP** | NP_783323.1 | 117177 | **rab-3A-interacting protein isoform beta 1** | 5.6 | 4 | 1 | 1 | 1 | 3.85E+06 | **0.57** | 1 | - | 395 | 43.4 | 5.2 | Biological_process unknown;Signal transduction | Cytoplasm;Nucleus | Molecular function unknown;Guanyl-nucleotide exchange factor activity | Cytoplasm |
| **CRNKL1** | NP_001265556.1 | 51340 | **crooked neck-like protein 1 isoform c** | 9.6 | 4 | 4 | 4 | 9 | 7.44E+07 | **0.57** | 5 | 17.2 | 564 | 68.6 | 6.2 | Regulation of nucleobase, nucleoside, nucleotide and nucleic acid metabolism | Nucleus;Cytoplasm | RNA binding | Nucleus |
| **DDX50** | NP_076950.1 | 79009 | **ATP-dependent RNA helicase DDX50** | 18.5 | 1 | 7 | 9 | 22 | 3.88E+08 | **0.57** | 6 | 11.1 | 737 | 82.5 | 9.2 | Regulation of nucleobase, nucleoside, nucleotide and nucleic acid metabolism | Cytoplasm;Nucleolus;Nucleus;Mitochondrion | RNA binding | Nucleolus |
| **EP300** | NP_001420.2 | 2033 | **histone acetyltransferase p300** | 1.5 | 1 | 2 | 2 | 2 | 2.73E+07 | **0.57** | 1 | - | 2414 | 264 | 8.5 | Regulation of nucleobase, nucleoside, nucleotide and nucleic acid metabolism | Cytoplasm;Nucleus | Transcription regulator activity | Nucleus |
| **GOLGA2** | NP_004477.3 | 2801 | **Golgin subfamily A member 2** | 20.8 | 1 | 10 | 10 | 14 | 1.68E+08 | **0.57** | 7 | 11.2 | 1002 | 113 | 5.0 | Cell growth and/or maintenance | Golgi apparatus;Golgi membrane | Structural molecule activity | Golgi apparatus;Golgi membrane |
| **MRPS25** | NP_071942.1 | 64432 | **28S ribosomal protein S25, mitochondrial** | 28.3 | 1 | 4 | 4 | 6 | 8.98E+07 | **0.57** | 1 | - | 173 | 20.1 | 8.8 | Protein metabolism | Mitochondrion | Structural constituent of ribosome | Mitochondrion |
| **RRP36** | NP_149103.1 | 88745 | **ribosomal RNA processing protein 36 homolog** | 19.7 | 1 | 3 | 3 | 6 | 4.28E+07 | **0.57** | 4 | 4.3 | 259 | 29.8 | 10.2 | Biological_process unknown | Nucleolus;Nucleus | Molecular function unknown | Nucleolus |
| **ALG5** | NP_001135836.1 | 29880 | **dolichyl-phosphate beta-glucosyltransferase isoform 2** | 18.7 | 2 | 4 | 4 | 9 | 9.98E+07 | **0.57** | 3 | 7.7 | 294 | 33.5 | 9.4 | Protein metabolism | Endoplasmic reticulum | Transferase activity | Endoplasmic reticulum |

| Gene Symbol | RefSeq_Accession | Entrez Gene ID | Description | Coverage (%) | Proteins | Unique Peptides | # Peptides | PSM | Area | SCC-R /SCC-S | SCC-R /SCC-S (Count) | SCC-R /SCC-S (Variability [%]) | # AAs | MW [kDa] | calc. pI | Biological Process | Localization | Molecular Function | Primary Localization |
|---|---|---|---|---|---|---|---|---|---|---|---|---|---|---|---|---|---|---|---|
| AHCY | NP_000678.1 | 191 | adenosylhomocysteinase isoform 1 | 47.2 | 2 | 21 | 21 | 116 | 1.70E+09 | 0.57 | 37 | 13.3 | 432 | 47.7 | 6.3 | Metabolism;Energy pathways | Cytoplasm | Hydrolase activity | Cytoplasm |
| LOC100132731 | XP_006710216.1 | 100132731 | uncharacterized protein FLJ43738 isoform X3 | 2.9 | 4 | 2 | 2 | 4 | 6.33E+08 | 0.57 | 1 | - | 654 | 75.2 | 7.5 | - | - | - | - |
| SCYL2 | NP_060458.3 | 55681 | SCY1-like protein 2 | 14.4 | 1 | 7 | 7 | 11 | 1.55E+08 | 0.57 | 4 | 16.7 | 929 | 103.6 | 8.2 | Regulation of endocytosis | Cytoplasm;Perinuclear region;Clathrin-coated vesicle;Golgi vesicle | Kinase activity | Perinuclear region |
| BCCIP | NP_510868.1 | 56647 | BRCA2 and CDKN1A-interacting protein isoform BCCIPbeta | 38.2 | 3 | 8 | 8 | 27 | 3.71E+08 | 0.56 | 8 | 19.1 | 314 | 36 | 4.6 | Cell growth and/or maintenance;Signal transduction;DNA repair | Nucleus | Peptide binding | Nucleus |
| CLTB | NP_001825.1 | 1212 | clathrin light chain B isoform a | 16.1 | 2 | 4 | 4 | 10 | 3.05E+08 | 0.56 | 5 | 20.1 | 211 | 23.2 | 4.7 | Cell growth and/or maintenance | Cytoplasm;Plasma membrane;Golgi apparatus | Structural molecule activity | Cytoplasm |
| GALNT6 | NP_009141.2 | 11226 | polypeptide N-acetylgalactosaminyltransferase 6 | 3.1 | 1 | 1 | 1 | 1 | 4.16E+07 | 0.56 | 1 | - | 622 | 71.1 | 8.2 | Protein metabolism | Golgi apparatus | Transferase activity | Golgi apparatus |
| ZDHHC13 | NP_061901.2 | 54503 | palmitoyltransferase ZDHHC13 isoform 1 | 4.7 | 3 | 3 | 3 | 3 | 4.92E+08 | 0.56 | 2 | 3.7 | 622 | 70.8 | 8.1 | Biological_process unknown | Integral to membrane | Molecular function unknown | Integral to membrane |
| DNTTIP1 | NP_443183.1 | 116092 | deoxynucleotidyltransferase terminal-interacting protein 1 | 5.5 | 1 | 1 | 1 | 1 | 1.72E+07 | 0.56 | 1 | - | 329 | 37 | 9.0 | Immune response | Nucleus | DNA binding | Nucleus |
| RPS19 | NP_001013.1 | 6223 | 40S ribosomal protein S19 | 58.6 | 1 | 11 | 11 | 83 | 2.61E+09 | 0.56 | 21 | 20.4 | 145 | 16.1 | 10.3 | Protein metabolism | Nucleolus;Mitochondrion | Structural constituent of ribosome | Nucleolus |
| VAMP8 | NP_003752.2 | 8673 | vesicle-associated membrane protein 8 | 47.0 | 1 | 4 | 4 | 12 | 3.30E+08 | 0.56 | 4 | 2.6 | 100 | 11.4 | 7.3 | Transport | Plasma membrane;Endosome;Secretory granule;Golgi apparatus;Endoplasmic reticulum;Zymogen granule | Auxiliary transport protein activity | Endosome |
| DHX29 | NP_061903.2 | 54505 | ATP-dependent RNA helicase DHX29 | 7.6 | 1 | 7 | 7 | 7 | 5.82E+07 | 0.56 | 3 | 24.4 | 1369 | 155.1 | 8.1 | Regulation of nucleobase, nucleoside, nucleotide and nucleic acid metabolism | - | Helicase activity | - |
| EEF2 | NP_001952.1 | 1938 | elongation factor 2 | 73.1 | 1 | 62 | 63 | 1116 | 9.36E+09 | 0.56 | 205 | 16.1 | 858 | 95.3 | 6.8 | Protein metabolism;Translation | Cytoplasm;Nucleolus;Cytosol;Mitochondrion;Ribosome;Nucleus | Translation regulator activity | Cytoplasm |
| H1F0 | NP_005309.1 | 3005 | histone H1.0 | 23.7 | 1 | 5 | 5 | 30 | 7.96E+08 | 0.56 | 6 | 35.4 | 194 | 20.9 | 10.8 | Regulation of nucleobase, nucleoside, nucleotide and nucleic acid metabolism | Nucleus;Cytoplasm | DNA binding | Nucleus |
| MTHFD1L | NP_001229697.1 | 25902 | monofunctional C1-tetrahydrofolate synthase, mitochondrial isoform 3 | 13.4 | 16 | 8 | 8 | 10 | 9.92E+07 | 0.56 | 6 | 15.6 | 913 | 99.2 | 7.3 | Metabolism | Mitochondrion | Catalytic activity | Mitochondrion |
| NBEAL1 | NP_001107604.1 | 65065 | neurobeachin-like protein 1 | 0.7 | 1 | 1 | 1 | 1 | 1.30E+07 | 0.56 | 1 | - | 2694 | 307 | 6.4 | Cell-cell signaling | Lysosome | Molecular function unknown | Lysosome |
| NOL10 | NP_001248323.1 | 79954 | nucleolar protein 10 isoform 3 | 16.9 | 3 | 5 | 5 | 8 | 8.43E+07 | 0.56 | 4 | 8.6 | 638 | 74.5 | 8.4 | Biological_process unknown | Nucleolus | Molecular function unknown | Nucleolus |
| RBM19 | NP_057280.2 | 9904 | probable RNA-binding protein 19 | 7.9 | 1 | 5 | 5 | 6 | 3.46E+07 | 0.56 | 2 | 8.6 | 960 | 107.3 | 6.5 | Regulation of nucleobase, nucleoside, nucleotide and nucleic acid metabolism | Nucleolus | RNA binding | Nucleolus |
| RNPEP | NP_064601.3 | 6051 | aminopeptidase B | 38.9 | 1 | 17 | 17 | 45 | 5.52E+08 | 0.56 | 17 | 10 | 650 | 72.5 | 5.7 | Protein metabolism | Golgi apparatus;Plasma membrane;Extracellular;Nucleus | Aminopeptidase activity | Golgi apparatus |
| CHD8 | NP_065971.2 | 57680 | chromodomain-helicase-DNA-binding protein 8 isoform 2 | 0.7 | 2 | 1 | 1 | 1 | 3.99E+07 | 0.56 | 1 | - | 2302 | 262.2 | 6.7 | Regulation of nucleobase, nucleoside, nucleotide and nucleic acid metabolism | Nucleus;Cytoplasm | Transcription regulator activity | Nucleus |
| RPL23 | NP_000969.1 | 9349 | 60S ribosomal protein L23 | 50.0 | 1 | 6 | 6 | 76 | 1.72E+09 | 0.56 | 18 | 11.8 | 140 | 14.9 | 10.5 | Protein metabolism | Ribosome | Structural constituent of ribosome | Ribosome |
| SLC2A1 | NP_006507.2 | 6513 | solute carrier family 2, facilitated glucose transporter member 1 | 12.4 | 1 | 6 | 6 | 16 | 5.06E+08 | 0.56 | 4 | 6.1 | 492 | 54 | 8.7 | Transport | Plasma membrane;Cytoplasm;Golgi apparatus | Auxiliary transport protein activity | Plasma membrane |
| EED | NP_694536.1 | 8726 | polycomb protein EED isoform b | 2.8 | 2 | 1 | 1 | 1 | 4.56E+07 | 0.56 | 1 | - | 400 | 45.5 | 6.7 | Regulation of gene expression, epigenetic | Nucleus;Cytoplasm | Transcription regulator activity | Nucleus |
| PCNA | NP_872590.1 | 5111 | proliferating cell nuclear antigen | 74.7 | 1 | 18 | 18 | 148 | 2.12E+09 | 0.56 | 37 | 15.1 | 261 | 28.8 | 4.7 | DNA repair | Nucleus;Cytoplasm;Nucleolus | DNA binding | Nucleus |
| BRD3 | NP_031397.1 | 8019 | bromodomain-containing protein 3 | 6.9 | 1 | 3 | 3 | 3 | 1.86E+07 | 0.56 | 2 | 47.5 | 726 | 79.5 | 9.4 | Regulation of nucleobase, nucleoside, nucleotide and nucleic acid metabolism | Nucleus | Transcription regulator activity | Nucleus |
| CEBPZ | NP_005751.2 | 10153 | CCAAT/enhancer-binding protein zeta | 13.1 | 1 | 8 | 8 | 10 | 1.56E+08 | 0.56 | 4 | 24.4 | 1054 | 120.9 | 5.9 | Regulation of nucleobase, nucleoside, nucleotide and nucleic acid metabolism | Nucleus;Nucleolus | Transcription regulator activity | Nucleus |
| LMNB1 | NP_005564.1 | 4001 | lamin-B1 isoform 1 | 60.2 | 1 | 21 | 40 | 161 | 9.13E+08 | 0.56 | 34 | 16.2 | 586 | 66.4 | 5.2 | Cell growth and/or maintenance | Nucleus;Nucleolus;Mitochondrion;Cytoplasm | Structural molecule activity | Nucleus |
| LRRC16A | NP_001167448.1 | 55604 | leucine-rich repeat-containing protein 16A isoform 2 | 2.9 | 2 | 2 | 2 | 2 | 4.04E+07 | 0.56 | 1 | - | 1365 | 150.8 | 7.8 | Biological_process unknown | - | Molecular function unknown | - |
| RPS10 | NP_001190174.1 | 6204 | 40S ribosomal protein S10 | 52.1 | 2 | 8 | 8 | 72 | 1.17E+09 | 0.56 | 11 | 34.6 | 165 | 18.9 | 10.2 | Protein metabolism | Ribosome;Nucleolus | Structural constituent of ribosome | Ribosome |
| PIGS | NP_149975.1 | 94005 | GPI transamidase component PIG-S | 15.7 | 1 | 5 | 5 | 10 | 9.24E+07 | 0.56 | 4 | 8.5 | 555 | 61.6 | 6.5 | Cell growth and/or maintenance | Microsome;Mitochondrion | Cytoskeletal anchoring activity | Microsome |
| GPD2 | NP_000399.3 | 2820 | glycerol-3-phosphate dehydrogenase, mitochondrial precursor | 47.9 | 1 | 24 | 24 | 47 | 1.16E+09 | 0.55 | 24 | 33.3 | 727 | 80.8 | 7.7 | Metabolism;Energy pathways | Mitochondrion | Catalytic activity | Mitochondrion |
| HMOX2 | NP_002125.3 | 3163 | heme oxygenase 2 isoform b | 31.0 | 3 | 5 | 5 | 11 | 7.62E+07 | 0.55 | 4 | 32.6 | 316 | 36 | 5.4 | Metabolism;Energy pathways | Endoplasmic reticulum;Mitochondrion | Catalytic activity | Endoplasmic reticulum |
| IDH1 | NP_001269315.1 | 3417 | isocitrate dehydrogenase [NADP] cytoplasmic | 61.8 | 1 | 22 | 23 | 91 | 9.54E+08 | 0.55 | 32 | 20.2 | 414 | 46.6 | 7.0 | Metabolism;Energy pathways | Cytoplasm;Peroxisome;Mitochondrion | Catalytic activity | Cytoplasm |
| ADNP | NP_852107.1 | 23394 | activity-dependent neuroprotector homeobox protein | 7.8 | 1 | 5 | 5 | 5 | 7.88E+07 | 0.55 | 2 | 46.1 | 1102 | 123.5 | 7.3 | Regulation of nucleobase, nucleoside, nucleotide and nucleic acid metabolism | Nucleus;Cytoplasm | Transcription factor activity | Nucleus |
| DBT | NP_001909.3 | 1629 | lipoamide acyltransferase component of branched-chain alpha-keto acid dehydrogenase complex, mitochondrial | 11.2 | 1 | 4 | 4 | 5 | 3.17E+07 | 0.55 | 2 | 14 | 482 | 53.5 | 8.5 | Metabolism;Energy pathways | Mitochondrion | Acyltransferase activity | Mitochondrion |
| FOXRED1 | NP_060017.1 | 55572 | FAD-dependent oxidoreductase domain-containing protein 1 | 14.6 | 1 | 3 | 3 | 4 | 4.62E+07 | 0.55 | 2 | 1.8 | 486 | 53.8 | 7.8 | Biological_process unknown | Mitochondrion | Molecular function unknown | Mitochondrion |
| MRPL1 | NP_064621.3 | 65008 | 39S ribosomal protein L1, mitochondrial precursor | 39.1 | 1 | 8 | 8 | 12 | 1.81E+08 | 0.55 | 4 | 11.7 | 325 | 36.9 | 8.8 | Protein metabolism | Mitochondrion | Structural constituent of ribosome | Mitochondrion |
| BYSL | NP_004044.3 | 705 | bystin | 27.7 | 1 | 9 | 9 | 22 | 2.06E+08 | 0.55 | 11 | 17.1 | 437 | 49.6 | 8.1 | Cell growth and/or maintenance | Cytoplasm;Nucleolus;Cytoskeleton;Nucleus | Cell adhesion molecule activity | Cytoplasm |

| Gene Symbol | RefSeq_Accession | Entrez Gene ID | Description | Coverage (%) | Proteins | Unique Peptides | # Peptides | PSM | Area | SCC-R /SCC-S | SCC-R /SCC-S (Count) | SCC-R /SCC-S (Variability [%]) | # AAs | MW [kDa] | calc. pI | Biological Process | Localization | Molecular Function | Primary Localization |
|---|---|---|---|---|---|---|---|---|---|---|---|---|---|---|---|---|---|---|---|
| **MYOF** | NP_579899.1 | 26509 | **myoferlin isoform b** | 46.0 | 16 | 72 | 72 | 213 | 9.08E+08 | **0.55** | 75 | 23 | 2048 | 233.3 | 6.2 | Cell growth and/or maintenance | Plasma membrane;Nucleus;Nucleolus | Molecular function unknown | Plasma membrane;Nucleus |
| **PSME4** | NP_055429.2 | 23198 | **proteasome activator complex subunit 4** | 4.0 | 1 | 4 | 4 | 4 | 5.28E+07 | **0.55** | 2 | 0.1 | 1843 | 211.2 | 6.9 | Protein metabolism | Nucleus | Ubiquitin-specific protease activity | Nucleus |
| **TIMM44** | NP_006342.2 | 10469 | **mitochondrial import inner membrane translocase subunit TIM44** | 18.6 | 1 | 7 | 7 | 22 | 2.74E+08 | **0.55** | 9 | 27.4 | 452 | 51.3 | 8.3 | Transport | Mitochondrion | Catalytic activity | Mitochondrion |
| **FAS** | NP_690610.1 | 355 | **tumor necrosis factor receptor superfamily member 6 isoform 2 precursor** | 13.1 | 3 | 2 | 2 | 2 | 4.42E+07 | **0.55** | 2 | 64.4 | 314 | 35.4 | 7.9 | Cell communication;Signal transduction;Anti-apoptosis | Plasma membrane;Cytoplasm | Receptor activity;Binding | Plasma membrane |
| **MMS19** | NP_001276332.1 | 64210 | **MMS19 nucleotide excision repair protein homolog isoform 2** | 41.8 | 3 | 20 | 20 | 40 | 2.92E+08 | **0.55** | 15 | 25.7 | 987 | 108.3 | 6.4 | Regulation of nucleobase, nucleoside, nucleotide and nucleic acid metabolism | Nucleus | Transcription regulator activity | Nucleus |
| **MRPS7** | NP_057055.2 | 51081 | **28S ribosomal protein S7, mitochondrial** | 30.6 | 1 | 5 | 5 | 14 | 3.93E+08 | **0.55** | 7 | 36.8 | 242 | 28.1 | 10.0 | Protein metabolism | Mitochondrion | Structural constituent of ribosome | Mitochondrion |
| **LIN54** | NP_001275926.1 | 132660 | **protein lin-54 homolog isoform b** | 2.3 | 2 | 1 | 1 | 1 | 1.94E+07 | **0.55** | 1 | - | 528 | 56.2 | 9.3 | Biological_process unknown | - | Molecular function unknown | - |
| **PPM1B** | NP_002697.1 | 5495 | **protein phosphatase 1B isoform 1** | 25.7 | 5 | 7 | 8 | 9 | 9.69E+07 | **0.55** | 4 | 18.5 | 479 | 52.6 | 5.1 | Signal transduction | Cytoplasm | Protein serine/threonine phosphatase activity | Cytoplasm |
| **RAB34** | NP_001243205.1 | 83871 | **ras-related protein Rab-34 isoform 6** | 21.9 | 7 | 4 | 4 | 8 | 5.25E+07 | **0.55** | 2 | 17.2 | 237 | 26.7 | 7.0 | Cell communication;Signal transduction | Plasma membrane;Endosome;Cytoplasm;Golgi apparatus;Cytosol | GTPase activity | Golgi apparatus |
| **ZBTB7A** | NP_056982.1 | 51341 | **zinc finger and BTB domain-containing protein 7A** | 3.3 | 1 | 1 | 1 | 3 | 6.05E+07 | **0.55** | 1 | - | 584 | 61.4 | 5.2 | Regulation of nucleobase, nucleoside, nucleotide and nucleic acid metabolism | Nucleus;Nucleolus;Cytoplasm | Transcription factor activity | Nucleus |
| **ATR** | NP_001175.2 | 545 | **serine/threonine-protein kinase ATR** | 0.5 | 1 | 1 | 1 | 1 | 2.25E+07 | **0.55** | 1 | - | 2644 | 301.2 | 7.4 | Cell communication;Signal transduction | Nucleus;Nucleolus | Protein serine/threonine kinase activity | Nucleus |
| **C6orf106** | NP_073595.2 | 64771 | **uncharacterized protein C6orf106 isoform b** | 8.6 | 2 | 1 | 1 | 1 | 2.48E+07 | **0.55** | 1 | - | 232 | 25.7 | 4.5 | Biological_process unknown | - | Molecular function unknown | - |
| **C1QBP** | NP_001203.1 | 708 | **complement component 1 Q subcomponent-binding protein, mitochondrial precursor** | 44.3 | 1 | 8 | 8 | 144 | 2.67E+09 | **0.54** | 15 | 15.5 | 282 | 31.3 | 4.8 | Immune response | Mitochondrion;Endoplasmic reticulum;Golgi apparatus;Cytoplasm;Plasma membrane;Nucleus | Complement receptor activity | Mitochondrion;Nucleus |
| **CARKD** | NP_001229811.1 | 55739 | **ATP-dependent (S)-NAD(P)H-hydrate dehydratase isoform c** | 12.8 | 3 | 2 | 2 | 2 | 3.78E+07 | **0.54** | 1 | - | 329 | 35.1 | 8.6 | Metabolism | - | Transporter activity | - |
| **TFRC** | NP_003225.2 | 7037 | **transferrin receptor protein 1** | 39.6 | 1 | 27 | 27 | 173 | 1.09E+09 | **0.54** | 41 | 27.3 | 760 | 84.8 | 6.6 | Transport | Plasma membrane;Endosome;Extracellular;Mitochondrion;Integral to membrane;Nucleus | Auxiliary transport protein activity | Plasma membrane |
| **WDHD1** | NP_009017.1 | 11169 | **WD repeat and HMG-box DNA-binding protein 1 isoform 1** | 7.5 | 2 | 5 | 5 | 9 | 5.38E+07 | **0.54** | 3 | 11.3 | 1129 | 125.9 | 5.6 | Regulation of nucleobase, nucleoside, nucleotide and nucleic acid metabolism | Nucleus;Cytoplasm | DNA binding | Nucleus |
| **LTA4H** | NP_000886.1 | 4048 | **leukotriene A-4 hydrolase isoform 1** | 52.9 | 3 | 27 | 27 | 78 | 1.09E+09 | **0.54** | 34 | 19 | 611 | 69.2 | 6.2 | Protein metabolism | Cytoplasm;Nucleus | Hydrolase activity | Cytoplasm |
| **ZC3H15** | NP_060941.2 | 55854 | **zinc finger CCCH domain-containing protein 15** | 21.4 | 1 | 6 | 6 | 11 | 1.11E+08 | **0.54** | 5 | 35.7 | 426 | 48.6 | 5.3 | Biological_process unknown | Nucleus;Cytoplasm | DNA binding | Nucleus;Cytoplasm |
| **ERCC2** | NP_000391.1 | 2068 | **TFIIH basal transcription factor complex helicase XPD subunit isoform 1** | 7.2 | 2 | 2 | 2 | 4 | 4.72E+07 | **0.54** | 1 | - | 760 | 86.9 | 7.2 | Regulation of nucleobase, nucleoside, nucleotide and nucleic acid metabolism | Nucleus;Cytoplasm;Nucleolus | DNA repair protein | Nucleus |
| **DRG2** | NP_001379.1 | 1819 | **developmentally-regulated GTP-binding protein 2** | 6.3 | 1 | 2 | 2 | 4 | 4.12E+07 | **0.54** | 2 | 32 | 364 | 40.7 | 8.9 | Cell communication;Signal transduction | - | Molecular function unknown | - |
| **FAM169A** | NP_056381.1 | 26049 | **soluble lamin-associated protein of 75 kDa** | 3.3 | 1 | 1 | 1 | 2 | 2.01E+08 | **0.54** | 1 | - | 670 | 74.9 | 4.6 | Biological_process unknown | - | Molecular function unknown | - |
| **GOT1** | NP_002070.1 | 2805 | **aspartate aminotransferase, cytoplasmic** | 58.8 | 1 | 18 | 18 | 70 | 1.07E+09 | **0.54** | 26 | 20.2 | 413 | 46.2 | 7.0 | Metabolism;Energy pathways | Cytoplasm;Mitochondrion | Transaminase activity | Cytoplasm |
| **RGS10** | NP_002916.1 | 6001 | **regulator of G-protein signaling 10 isoform b** | 40.7 | 2 | 6 | 6 | 15 | 3.36E+08 | **0.54** | 7 | 17.2 | 167 | 19.6 | 5.9 | Cell communication;Signal transduction | Cytoplasm;Plasma membrane;Nucleus | GTPase activator activity | Plasma membrane |
| **PRDX2** | NP_005800.3 | 7001 | **peroxiredoxin-2** | 85.9 | 1 | 15 | 16 | 99 | 3.08E+09 | **0.54** | 23 | 22.6 | 198 | 21.9 | 6.0 | Metabolism;Energy pathways | Cytoplasm;Plasma membrane | Peroxidase activity | Cytoplasm |
| **SSR1** | NP_003135.2 | 6745 | **translocon-associated protein subunit alpha isoform 1 precursor** | 19.9 | 2 | 5 | 5 | 12 | 3.45E+08 | **0.54** | 2 | 18.3 | 286 | 32.2 | 4.5 | Transport | Endoplasmic reticulum;Microsome;Mitochondrion;Nucleus | Auxiliary transport protein activity | Endoplasmic reticulum |
| **PTGR2** | NP_689657.1 | 145482 | **prostaglandin reductase 2** | 13.4 | 1 | 3 | 3 | 4 | 1.07E+07 | **0.54** | 1 | - | 351 | 38.5 | 5.4 | Metabolism;Energy pathways | - | Catalytic activity | - |
| **SLIRP** | NP_001254792.1 | 81892 | **SRA stem-loop-interacting RNA-binding protein, mitochondrial isoform 2 precursor** | 70.1 | 3 | 7 | 7 | 33 | 8.61E+08 | **0.54** | 14 | 21.3 | 107 | 12.1 | 10.2 | Regulation of nucleobase, nucleoside, nucleotide and nucleic acid metabolism | Nucleus | RNA binding | Nucleus |
| **PDCD4** | NP_001186421.1 | 27250 | **programmed cell death protein 4 isoform 3** | 35.0 | 3 | 10 | 10 | 22 | 1.89E+08 | **0.54** | 7 | 27.8 | 455 | 50.2 | 5.5 | Apoptosis | Nucleus;Cytoplasm | Molecular function unknown | Nucleus;Cytoplasm |
| **SEPT9** | NP_001106963.1 | 10801 | **septin-9 isoform a** | 56.1 | 6 | 26 | 26 | 76 | 4.31E+08 | **0.53** | 29 | 48.9 | 586 | 65.4 | 9.0 | Cell proliferation | Cytoskeleton;Nucleus;Microtubule | GTP binding | Cytoskeleton;Nucleus |
| **ARHGAP5** | NP_001164.2 | 394 | **rho GTPase-activating protein 5 isoform b** | 1.6 | 2 | 1 | 1 | 1 | 5.07E+07 | **0.53** | 1 | - | 1501 | 172.2 | 6.6 | Cell communication;Signal transduction | Cytoplasm;Plasma membrane | GTPase activator activity | Cytoplasm |
| **EML4** | NP_001138548.1 | 27436 | **echinoderm microtubule-associated protein-like 4 isoform b** | 7.7 | 2 | 5 | 5 | 9 | 1.37E+08 | **0.53** | 2 | 34.7 | 923 | 102.4 | 6.1 | Cell growth and/or maintenance | Cytoplasm;Microtubule | Structural molecule activity | Cytoplasm |
| **ERCC3** | NP_000113.1 | 2071 | **TFIIH basal transcription factor complex helicase XPB subunit** | 10.9 | 1 | 5 | 5 | 8 | 9.09E+07 | **0.53** | 4 | 72.1 | 782 | 89.2 | 7.2 | Regulation of nucleobase, nucleoside, nucleotide and nucleic acid metabolism | Nucleus;Cytoplasm | DNA repair protein | Nucleus |
| **PREX1** | NP_065871.2 | 57580 | **phosphatidylinositol 3,4,5-trisphosphate-dependent Rac exchanger 1 protein** | 0.4 | 1 | 1 | 1 | 1 | 1.63E+07 | **0.53** | 1 | - | 1659 | 186.1 | 6.4 | Cell communication;Signal transduction | Cytoplasm;Plasma membrane | Guanyl-nucleotide exchange factor activity | Cytoplasm |
| **TRIP4** | NP_057297.2 | 9325 | **activating signal cointegrator 1** | 4.8 | 1 | 1 | 1 | 2 | 3.92E+07 | **0.53** | 1 | - | 581 | 66.1 | 7.9 | Regulation of nucleobase, nucleoside, nucleotide and nucleic acid metabolism | Nucleus;Cytoplasm | Transcription regulator activity | Nucleus |
| **ALDH2** | NP_001191818.1 | 217 | **aldehyde dehydrogenase, mitochondrial isoform 2 precursor** | 20.0 | 2 | 6 | 6 | 11 | 8.69E+07 | **0.53** | 5 | 26.9 | 470 | 51 | 6.2 | Metabolism;Energy pathways | Mitochondrion;Cytoplasm | Catalytic activity | Mitochondrion |
| **GLYR1** | NP_115958.2 | 84656 | **putative oxidoreductase GLYR1** | 5.6 | 1 | 2 | 2 | 4 | 8.40E+07 | **0.53** | 3 | 39.8 | 553 | 60.5 | 9.2 | Metabolism;Energy pathways | Nucleus | Catalytic activity | Nucleus |
| **LPCAT1** | NP_079106.3 | 79888 | **lysophosphatidylcholine acyltransferase 1** | 6.2 | 1 | 2 | 2 | 4 | 9.29E+07 | **0.53** | 2 | 39.6 | 534 | 59.1 | 6.0 | Cell organization and biogenesis | Endoplasmic reticulum;Golgi apparatus | Acyltransferase activity | Endoplasmic reticulum |

| Gene Symbol | RefSeq_Accession | Entrez Gene ID | Description | Coverage (%) | Proteins | Unique Peptides | # Peptides | PSM | Area | SCC-R /SCC-S | SCC-R /SCC-S (Count) | SCC-R /SCC-S (Variability [%]) | # AAs | MW [kDa] | calc. pI | Biological Process | Localization | Molecular Function | Primary Localization |
|---|---|---|---|---|---|---|---|---|---|---|---|---|---|---|---|---|---|---|---|
| **STX6** | NP_005810.1 | 10228 | **syntaxin-6 isoform 1** | 14.1 | 2 | 2 | 2 | 6 | 8.56E+07 | **0.53** | 1 | - | 255 | 29.2 | 4.9 | Cell communication;Signal transduction | Plasma membrane;Golgi apparatus | Auxiliary transport protein activity | Plasma membrane |
| **HEATR3** | NP_891552.1 | 55027 | **HEAT repeat-containing protein 3** | 3.8 | 1 | 2 | 2 | 3 | 1.10E+07 | **0.53** | 1 | - | 680 | 74.5 | 5.1 | Biological_process unknown | - | Molecular function unknown | - |
| **RWDD1** | NP_001007465.1 | 51389 | **RWD domain-containing protein 1 isoform b** | 17.7 | 2 | 2 | 2 | 3 | 2.74E+07 | **0.53** | 2 | 115.1 | 147 | 17.1 | 4.4 | Biological_process | - | Molecular function unknown | - |
| **TMED7-TICAM2** | NP_001157941.1 | 100302736 | **TRAM adaptor with GOLD domain isoform 2 precursor** | 26.1 | 3 | 3 | 3 | 12 | 6.06E+07 | **0.53** | 2 | 4.9 | 188 | 21.2 | 6.2 | - | - | - | - |
| **ACADVL** | NP_001029031.1 | 37 | **very long-chain specific acyl-CoA dehydrogenase, mitochondrial isoform 2 precursor** | 43.3 | 4 | 18 | 18 | 39 | 4.74E+08 | **0.53** | 14 | 22.4 | 633 | 68 | 8.6 | Energy pathways | Mitochondrion | Catalytic activity | Mitochondrion |
| **GPT2** | NP_597700.1 | 84706 | **alanine aminotransferase 2 isoform 1** | 18.4 | 3 | 5 | 5 | 6 | 4.06E+07 | **0.53** | 4 | 47.7 | 523 | 57.9 | 7.7 | Metabolism;Energy pathways | Cytoplasm;Mitochondrion | Transaminase activity | Cytoplasm |
| **HADHB** | NP_001268442.1 | 3032 | **trifunctional enzyme subunit beta, mitochondrial isoform 3** | 56.6 | 3 | 17 | 17 | 73 | 1.78E+09 | **0.53** | 26 | 33.7 | 452 | 48.8 | 9.2 | Metabolism;Energy pathways | Mitochondrion | Catalytic activity | Mitochondrion |
| **NFXL1** | NP_001265553.1 | 152518 | **NF-X1-type zinc finger protein NFXL1** | 2.4 | 1 | 2 | 2 | 3 | 1.89E+07 | **0.53** | 1 | - | 911 | 101.3 | 8.4 | Regulation of nucleobase, nucleoside, nucleotide and nucleic acid metabolism | Cytoplasm | Transcription factor activity | Cytoplasm |
| **SLC16A1** | NP_001159968.1 | 6566 | **monocarboxylate transporter 1** | 7.2 | 1 | 2 | 2 | 7 | 3.72E+08 | **0.53** | 3 | 22.9 | 500 | 53.9 | 8.7 | Transport | Plasma membrane;Mitochondrion;Nucleus | Auxiliary transport protein activity | Plasma membrane |
| **NDRG3** | NP_071922.2 | 57446 | **protein NDRG3 isoform b** | 31.7 | 2 | 8 | 8 | 13 | 1.70E+08 | **0.53** | 7 | 22.5 | 363 | 40 | 5.4 | Cell communication;Signal transduction;Cell differentiation | Cytoplasm | Molecular function unknown | Cytoplasm |
| **SLC25A5** | NP_001143.2 | 292 | **ADP/ATP translocase 2** | 44.3 | 2 | 10 | 21 | 256 | 3.69E+09 | **0.53** | 20 | 25.2 | 298 | 32.8 | 9.7 | Metabolism;Energy pathways | Mitochondrion | ATP binding | Mitochondrion |
| **IDE** | NP_004960.2 | 3416 | **insulin-degrading enzyme isoform 1** | 18.3 | 2 | 10 | 10 | 18 | 1.77E+08 | **0.53** | 8 | 37 | 1019 | 117.9 | 6.6 | Protein metabolism | Cytoplasm;Plasma membrane;Extracellular;Peroxisome;Endosome | Metallopeptidase activity | Cytoplasm |
| **ATP5H** | NP_001003785.1 | 10476 | **ATP synthase subunit d, mitochondrial isoform b** | 38.7 | 2 | 4 | 4 | 28 | 5.96E+08 | **0.53** | 5 | 18.2 | 137 | 15.8 | 7.2 | Metabolism;Energy pathways;Ion transport | Mitochondrial membrane;Mitochondrion | Transporter activity | Mitochondrial membrane |
| **DPM1** | NP_003850.1 | 8813 | **dolichol-phosphate mannosyltransferase subunit 1** | 26.5 | 1 | 5 | 5 | 10 | 1.29E+08 | **0.53** | 4 | 2.1 | 260 | 29.6 | 9.6 | Metabolism;Energy pathways | Plasma membrane;Nucleus;Cytoplasm | Mannosyltransferase activity | Plasma membrane |
| **STK24** | NP_001273578.1 | 8428 | **serine/threonine-protein kinase 24 isoform c precursor** | 38.4 | 3 | 9 | 11 | 31 | 4.99E+08 | **0.53** | 5 | 48.1 | 412 | 45.8 | 5.6 | Signal transduction | Cytoplasm;Nucleus | Protein serine/threonine kinase activity | Cytoplasm;Nucleus |
| **ATG12** | NP_004698.3 | 9140 | **ubiquitin-like protein ATG12 isoform 1** | 4.3 | 1 | 1 | 1 | 2 | 1.13E+07 | **0.53** | 1 | - | 140 | 15.1 | 5.1 | Biological_process unknown | Cytoplasm | Molecular function unknown | Cytoplasm |
| **ATP5C1** | NP_001001973.1 | 509 | **ATP synthase subunit gamma, mitochondrial isoform L (liver) precursor** | 35.2 | 2 | 7 | 7 | 21 | 2.35E+08 | **0.53** | 6 | 7.9 | 298 | 33 | 9.2 | Metabolism;Energy pathways | Mitochondrion | Transporter activity | Mitochondrion |
| **HNRNPF** | NP_004957.1 | 3185 | **heterogeneous nuclear ribonucleoprotein F** | 52.8 | 1 | 12 | 14 | 95 | 2.05E+09 | **0.53** | 16 | 20.4 | 415 | 45.6 | 5.6 | Regulation of nucleobase, nucleoside, nucleotide and nucleic acid metabolism | Nucleus;Cytoplasm;Nucleolus | Ribonucleoprotein | Nucleus |
| **HSDL2** | NP_001182751.1 | 84263 | **hydroxysteroid dehydrogenase-like protein 2 isoform 2** | 49.3 | 2 | 10 | 10 | 29 | 2.12E+08 | **0.53** | 15 | 32.5 | 345 | 37.3 | 6.1 | Biological_process unknown | - | Molecular function unknown | - |
| **TERF2** | NP_005643.2 | 7014 | **telomeric repeat-binding factor 2** | 13.3 | 1 | 5 | 5 | 6 | 5.82E+07 | **0.53** | 2 | 17.8 | 542 | 59.6 | 9.4 | Regulation of nucleobase, nucleoside, nucleotide and nucleic acid metabolism | Nucleus | DNA binding | Nucleus |
| **KIF11** | NP_004514.2 | 3832 | **kinesin-like protein KIF11** | 8.2 | 1 | 5 | 5 | 7 | 5.77E+07 | **0.53** | 2 | 1 | 1056 | 119.1 | 5.6 | Cell growth and/or maintenance | Cytoplasm;Centrosome;Microtubule | Motor activity | Cytoplasm |
| **UTP18** | NP_057085.2 | 51096 | **U3 small nucleolar RNA-associated protein 18 homolog** | 21.4 | 1 | 7 | 7 | 16 | 1.76E+08 | **0.53** | 8 | 16.7 | 556 | 62 | 8.8 | Regulation of nucleobase, nucleoside, nucleotide and nucleic acid metabolism | Nucleolus | Molecular function unknown | Nucleolus |
| **LRPPRC** | NP_573566.2 | 10128 | **leucine-rich PPR motif-containing protein, mitochondrial precursor** | 65.9 | 1 | 77 | 77 | 345 | 2.54E+09 | **0.52** | 121 | 24.7 | 1394 | 157.8 | 6.1 | Regulation of nucleobase, nucleoside, nucleotide and nucleic acid metabolism | Nucleus;Cytoplasm;Plasma membrane | RNA binding | Nucleus |
| **SNX17** | NP_001253989.1 | 9784 | **sorting nexin-17 isoform 3** | 8.3 | 4 | 2 | 2 | 3 | 5.60E+07 | **0.52** | 2 | 82.2 | 445 | 50 | 7.0 | Cell communication;Signal transduction | Cytoplasm;Integral to membrane;Endosome | Receptor signaling complex scaffold activity | Cytoplasm |
| **TIMM22** | NP_037469.2 | 29928 | **mitochondrial import inner membrane translocase subunit Tim22** | 10.3 | 1 | 1 | 1 | 1 | 2.79E+08 | **0.52** | 1 | - | 194 | 20 | 7.6 | Transport | Mitochondrion;Mitochondrial membrane | Transporter activity | Mitochondrial membrane |
| **ELF2** | NP_001263388.1 | 1998 | **ETS-related transcription factor Elf-2 isoform 5** | 4.9 | 5 | 1 | 1 | 2 | 3.64E+07 | **0.52** | 1 | - | 492 | 53 | 8.6 | Regulation of nucleobase, nucleoside, nucleotide and nucleic acid metabolism | Nucleus | Transcription factor activity | Nucleus |
| **HYPK** | NP_057484.3 | 25764 | **huntingtin-interacting protein K isoform 1** | 46.5 | 1 | 5 | 5 | 15 | 2.31E+08 | **0.52** | 3 | 27.8 | 129 | 14.7 | 4.9 | Biological_process unknown | - | Molecular function unknown | - |
| **KDELC1** | NP_076994.2 | 79070 | **KDEL motif-containing protein 1 precursor** | 10.0 | 1 | 3 | 3 | 4 | 2.66E+07 | **0.52** | 1 | - | 502 | 58 | 7.7 | Biological_process unknown | Endoplasmic reticulum | Molecular function unknown | Endoplasmic reticulum |
| **MFSD10** | NP_001139541.1 | 10227 | **major facilitator superfamily domain-containing protein 10** | 10.6 | 1 | 3 | 3 | 5 | 8.50E+07 | **0.52** | 1 | - | 455 | 48.3 | 9.6 | Biological_process unknown | - | Molecular function unknown | - |
| **RPS6KA1** | NP_002944.2 | 6195 | **ribosomal protein S6 kinase alpha-1 isoform a** | 14.3 | 5 | 6 | 6 | 12 | 1.27E+08 | **0.52** | 3 | 20.3 | 735 | 82.7 | 7.8 | Cell communication;Signal transduction | Nucleus;Cytosol;Membrane fraction | Protein serine/threonine kinase activity | Cytosol |
| **TRIP12** | NP_001271145.1 | 9320 | **E3 ubiquitin-protein ligase TRIP12 isoform d** | 7.4 | 4 | 8 | 8 | 10 | 5.89E+07 | **0.52** | 4 | 8.2 | 1722 | 192 | 6.5 | Regulation of nucleobase, nucleoside, nucleotide and nucleic acid metabolism | Nucleus | Transcription regulator activity | Nucleus |
| **ILVBL** | NP_006835.2 | 10994 | **acetolactate synthase-like protein** | 31.5 | 1 | 11 | 11 | 27 | 2.33E+08 | **0.52** | 13 | 29.7 | 632 | 67.8 | 8.2 | Metabolism;Energy pathways | - | Coenzyme binding | - |
| **PGAM5** | NP_001164014.1 | 192111 | **serine/threonine-protein phosphatase PGAM5, mitochondrial isoform 1** | 28.7 | 3 | 7 | 7 | 13 | 2.56E+08 | **0.52** | 6 | 11 | 289 | 32 | 8.7 | Metabolism;Energy pathways | Cytosol | Catalytic activity | Cytosol |
| **PTPMT1** | NP_001137456.1 | 114971 | **phosphatidylglycerophosphatase and protein-tyrosine phosphatase 1 isoform 2** | 8.6 | 2 | 1 | 1 | 3 | 5.58E+07 | **0.52** | 1 | - | 151 | 16.4 | 11.0 | Cell communication;Signal transduction | - | Protein tyrosine/serine/threonine phosphatase activity | - |
| **ITPA** | NP_258412.1 | 3704 | **inosine triphosphate pyrophosphatase isoform a** | 26.8 | 3 | 3 | 3 | 5 | 9.11E+07 | **0.52** | 2 | 47.5 | 194 | 21.4 | 5.7 | Metabolism;Energy pathways | Cytoplasm | Hydrolase activity | Cytoplasm |
| **PDCD11** | NP_055791.1 | 22984 | **protein RRP5 homolog** | 19.9 | 1 | 23 | 23 | 38 | 1.87E+08 | **0.52** | 21 | 18.4 | 1871 | 208.6 | 8.9 | Apoptosis | Cytoplasm;Nucleus | Transcription factor binding | Nucleus |
| **PPP2R5D** | NP_001257405.1 | 5528 | **serine/threonine-protein phosphatase 2A 56 kDa regulatory subunit delta isoform isoform 4** | 20.0 | 9 | 5 | 5 | 8 | 8.93E+07 | **0.52** | 3 | 18 | 451 | 53.1 | 6.4 | Cell communication;Signal transduction | Chromosome;Nucleus;Cytoplasm;Microtubule | Protein serine/threonine phosphatase activity | Nucleus;Cytoplasm |

Supplementary Table S4. List of all proteins identified in SCC-S and SCC-R cells using SequestHT and Mascot search algorithms and their gene ontology annotation

| Gene Symbol | RefSeq_Accession | Entrez Gene ID | Description | Coverage (%) | Proteins | Unique Peptides | # Peptides | PSM | Area | SCC-R /SCC-S | SCC-R /SCC-S (Count) | SCC-R /SCC-S (Variability [%]) | # AAs | MW [kDa] | calc. pI | Biological Process | Localization | Molecular Function | Primary Localization |
|---|---|---|---|---|---|---|---|---|---|---|---|---|---|---|---|---|---|---|---|
| **SEC14L2** | NP_001191133.1 | 23541 | **SEC14-like protein 2 isoform 3** | 23.8 | 4 | 4 | 4 | 5 | 3.41E+07 | **0.52** | 3 | 16.4 | 320 | 36.6 | 8.6 | Regulation of nucleobase, nucleoside, nucleotide and nucleic acid metabolism | Cytoplasm;Nucleus | Transcription regulator activity | Cytoplasm |
| **FUCA2** | NP_114409.2 | 2519 | **plasma alpha-L-fucosidase precursor** | 2.4 | 1 | 1 | 1 | 1 | 2.30E+07 | **0.52** | 1 | - | 467 | 54 | 6.3 | Metabolism;Energy pathways | Lysosome | Hydrolase activity | Lysosome |
| **ITCH** | NP_001244067.1 | 83737 | **E3 ubiquitin-protein ligase Itchy homolog isoform 3** | 6.0 | 3 | 3 | 3 | 4 | 3.90E+07 | **0.52** | 2 | 39 | 752 | 86.4 | 5.6 | Regulation of signal transduction | Plasma membrane;Endosome;Vesicle | Ubiquitin-specific protease activity | Endosome |
| **MAP4K3** | NP_001257354.1 | 8491 | **mitogen-activated protein kinase kinase kinase kinase 3 isoform 2** | 4.6 | 2 | 2 | 2 | 2 | 2.04E+07 | **0.52** | 2 | 83.4 | 873 | 98.9 | 7.9 | Cell communication;Signal transduction | Cytoplasm | Protein serine/threonine kinase activity | Cytoplasm |
| **SRP9** | NP_003124.1 | 6726 | **signal recognition particle 9 kDa protein isoform 2** | 46.5 | 2 | 5 | 5 | 29 | 1.19E+09 | **0.52** | 4 | 46.2 | 86 | 10.1 | 8.0 | Protein metabolism | Cytoplasm;Nucleolus | RNA binding | Cytoplasm |
| **EEFSEC** | NP_068756.2 | 60678 | **selenocysteine-specific elongation factor** | 3.0 | 1 | 1 | 1 | 2 | 8.62E+07 | **0.52** | 1 | - | 596 | 65.3 | 8.4 | Protein metabolism | Cytoplasm | Translation regulator activity | Cytoplasm |
| **MIPEP** | NP_005923.2 | 4285 | **mitochondrial intermediate peptidase** | 3.2 | 1 | 1 | 1 | 2 | 1.47E+07 | **0.52** | 1 | - | 713 | 80.6 | 7.1 | Protein metabolism | Mitochondrion | Metallopeptidase activity | Mitochondrion |
| **SEC23B** | NP_001166217.1 | 10483 | **protein transport protein Sec23B isoform 2** | 34.7 | 2 | 13 | 14 | 32 | 1.92E+08 | **0.52** | 13 | 23.1 | 749 | 84.4 | 7.0 | Transport | Endoplasmic reticulum;Golgi apparatus;Cytoplasm | Transporter activity | Endoplasmic reticulum |
| **MRPL42** | NP_054769.1 | 28977 | **39S ribosomal protein L42, mitochondrial precursor** | 13.4 | 1 | 2 | 2 | 2 | 4.42E+07 | **0.51** | 1 | - | 142 | 16.7 | 8.4 | Protein metabolism | Mitochondrion | Structural constituent of ribosome | Mitochondrion |
| **CHD4** | NP_001264.2 | 1108 | **chromodomain-helicase-DNA-binding protein 4** | 23.9 | 1 | 27 | 29 | 45 | 2.16E+08 | **0.51** | 20 | 25.7 | 1912 | 217.9 | 5.9 | Regulation of nucleobase, nucleoside, nucleotide and nucleic acid metabolism | Nucleus;Nucleolus;Mitochondrion;Cytoplasm | DNA binding | Nucleus;Cytoplasm |
| **FEN1** | NP_004102.1 | 2237 | **flap endonuclease 1** | 44.2 | 1 | 13 | 13 | 59 | 1.11E+09 | **0.51** | 20 | 31.1 | 380 | 42.6 | 8.6 | Regulation of nucleobase, nucleoside, nucleotide and nucleic acid metabolism | Nucleus;Nucleolus | Deoxyribonuclease activity | Nucleus |
| **MSH6** | NP_000170.1 | 2956 | **DNA mismatch repair protein Msh6 isoform 1** | 16.5 | 3 | 13 | 13 | 24 | 2.39E+08 | **0.51** | 12 | 26.5 | 1360 | 152.7 | 6.9 | DNA repair | Nucleus;Cytoplasm | DNA binding | Nucleus |
| **SSR3** | NP_009038.1 | 6747 | **translocon-associated protein subunit gamma** | 7.6 | 1 | 1 | 1 | 5 | 4.43E+08 | **0.51** | 2 | 18.5 | 185 | 21.1 | 9.6 | Transport | Microsome;Endoplasmic reticulum;Mitochondrion;Cytoplasm | Auxiliary transport protein activity | Microsome |
| **ORC3** | NP_001184188.1 | 23595 | **origin recognition complex subunit 3 isoform 3** | 2.1 | 3 | 1 | 1 | 2 | 6.21E+06 | **0.51** | 1 | - | 568 | 65.9 | 7.6 | Regulation of nucleobase, nucleoside, nucleotide and nucleic acid metabolism | Nucleus | DNA binding | Nucleus |
| **MARCH2** | NP_060368.2 | 54996 | **mitochondrial amidoxime reducing component 2 precursor** | 4.5 | 2 | 1 | 1 | 3 | 1.78E+08 | **-** | - | - | 335 | 38 | 9.2 | Drug metabolism | Mitochondrial membrane | Catalytic activity | Mitochondrial membrane |
| **MARCH3** | NP_848545.1 | 115123 | **E3 ubiquitin-protein ligase MARCH3** | 2.8 | 1 | 1 | 1 | 2 | 3.56E+07 | **-** | - | - | 253 | 28.5 | 8.2 | Protein metabolism | Integral to membrane | Ubiquitin-specific protease activity | Integral to membrane |
| **AAED1** | NP_714542.1 | 195827 | **thioredoxin-like protein AAED1** | 15.0 | 1 | 1 | 1 | 1 | 2.45E+06 | **-** | - | - | 226 | 24.8 | 8.9 | Biological_process unknown | - | Molecular function unknown | - |
| **AAGAB** | NP_001258815.1 | 79719 | **alpha- and gamma-adaptin-binding protein p34 isoform 2** | 13.6 | 2 | 1 | 1 | 2 | 9.62E+07 | **-** | - | - | 206 | 22.7 | 4.7 | Biological_process unknown | Nucleus | Molecular function unknown | Nucleus |
| **ABCA2** | NP_001597.2 | 20 | **ATP-binding cassette sub-family A member 2 isoform a** | 1.1 | 2 | 1 | 1 | 1 | 4.95E+08 | **-** | - | - | 2436 | 269.8 | 6.8 | Transport | Lysosome;Cytoplasm | Transporter activity | Lysosome |
| **ABCB6** | NP_005680.1 | 10058 | **ATP-binding cassette sub-family B member 6, mitochondrial** | 11.2 | 1 | 4 | 4 | 4 | 6.83E+06 | **-** | - | - | 842 | 93.8 | 8.5 | Transport | Mitochondrion | Auxiliary transport protein activity | Mitochondrion |
| **ABI1** | NP_001171596.1 | 10006 | **abl interactor 1 isoform l** | 10.9 | 12 | 2 | 2 | 6 | 2.68E+07 | **-** | - | - | 329 | 35.9 | 5.1 | Regulation of cell growth;Signal transduction | Cytosol | Binding | Cytosol |
| **ACAD10** | NP_079523.3 | 80724 | **acyl-CoA dehydrogenase family member 10 isoform b** | 2.0 | 2 | 1 | 1 | 1 | 6.08E+07 | **-** | - | - | 1059 | 118.8 | 8.1 | Metabolism;Energy pathways | - | Catalytic activity | - |
| **ACADS** | NP_000008.1 | 35 | **short-chain specific acyl-CoA dehydrogenase, mitochondrial precursor** | 7.5 | 1 | 1 | 1 | 1 | 0.00E+00 | **-** | - | - | 412 | 44.3 | 8.0 | Metabolism;Energy pathways | Mitochondrion | Oxidoreductase activity | Mitochondrion |
| **ACBD7** | NP_001034933.1 | 414149 | **acyl-CoA-binding domain-containing protein 7** | 15.9 | 1 | 1 | 1 | 1 | 3.69E+06 | **-** | - | - | 88 | 9.8 | 6.7 | Biological_process unknown | - | Molecular function unknown | - |
| **ACSBG2** | NP_001276108.1 | 81616 | **long-chain-fatty-acid--CoA ligase ACSBG2 isoform b** | 3.1 | 2 | 1 | 1 | 1 | 4.49E+08 | **-** | - | - | 638 | 71.2 | 8.7 | Metabolism;Energy pathways | - | Molecular function unknown | - |
| **ACTBL2** | NP_001017992.1 | 345651 | **beta-actin-like protein 2** | 20.7 | 1 | 1 | 10 | 564 | 1.29E+10 | **-** | - | - | 376 | 42 | 5.6 | Biological_process unknown | - | Molecular function unknown | - |
| **ADAL** | NP_001012987.1 | 161823 | **adenosine deaminase-like protein isoform 2** | 4.5 | 2 | 1 | 1 | 1 | 2.51E+07 | **-** | - | - | 267 | 30 | 6.6 | Biological_process unknown | - | Molecular function unknown | - |
| **ADAMTS12** | NP_112217.2 | 81792 | **A disintegrin and metalloproteinase with thrombospondin motifs 12 preproprotein** | 0.3 | 1 | 1 | 1 | 1 | 1.01E+07 | **-** | - | - | 1594 | 177.6 | 7.9 | Protein metabolism | Extracellular;Nucleus | Metallopeptidase activity | Extracellular |
| **ADAMTSL1** | NP_001035362.3 | 92949 | **ADAMTS-like protein 1 isoform 4 precursor** | 0.7 | 1 | 1 | 1 | 1 | 2.24E+07 | **-** | - | - | 1762 | 193.3 | 7.8 | Biological_process unknown | Extracellular | Molecular function unknown | Extracellular |
| **ADAR** | NP_056655.2 | 103 | **double-stranded RNA-specific adenosine deaminase isoform b** | 30.3 | 2 | 1 | 21 | 57 | 3.56E+08 | **-** | - | - | 1200 | 133.1 | 8.5 | Regulation of nucleobase, nucleoside, nucleotide and nucleic acid metabolism | Cytoplasm;Nucleolus;Nucleus | Deaminase activity | Nucleus |
| **ADCK4** | NP_079152.3 | 79934 | **aarF domain-containing protein kinase 4 isoform a** | 1.7 | 1 | 1 | 1 | 1 | 2.09E+07 | **-** | - | - | 544 | 60 | 7.2 | Biological_process unknown | - | Molecular function unknown | - |
| **ADO** | NP_116193.2 | 84890 | **2-aminoethanethiol dioxygenase** | 4.1 | 1 | 1 | 1 | 1 | 1.65E+06 | **-** | - | - | 270 | 29.7 | 6.0 | Biological_process unknown | - | Molecular function unknown | - |
| **ADRA2C** | NP_000674.2 | 152 | **alpha-2C adrenergic receptor** | 5.6 | 1 | 1 | 1 | 1 | 1.95E+07 | **-** | - | - | 462 | 49.5 | 10.3 | Cell communication;Signal transduction | Integral to membrane;Endosome | G-protein coupled receptor activity | Integral to membrane |
| **ADSSL1** | NP_689541.1 | 122622 | **adenylosuccinate synthetase isozyme 1 isoform 2** | 5.9 | 2 | 1 | 2 | 2 | 6.25E+06 | **-** | - | - | 457 | 50.2 | 8.6 | Metabolism;Energy pathways | Cytoplasm | Molecular function unknown | Cytoplasm |
| **AFAP1L2** | NP_115939.1 | 84632 | **actin filament-associated protein 1-like 2 isoform 2** | 8.0 | 3 | 4 | 4 | 5 | 5.10E+07 | **-** | - | - | 814 | 90.8 | 5.3 | Signal transduction | Cytoplasm | Transcription regulator activity | Cytoplasm |
| **AGAP3** | NP_001036000.1 | 116988 | **arf-GAP with GTPase, ANK repeat and PH domain-containing protein 3 isoform b** | 4.0 | 5 | 1 | 1 | 2 | 7.33E+06 | **-** | - | - | 396 | 42.5 | 7.7 | Cell communication;Signal transduction | - | Molecular function unknown | - |
| **AGGF1** | NP_060516.2 | 55109 | **angiogenic factor with G patch and FHA domains 1** | 2.8 | 1 | 1 | 1 | 1 | 1.53E+09 | **-** | - | - | 714 | 80.9 | 5.4 | Cell communication;Signal transduction | Extracellular;Cytoplasm;Nucleus | Growth factor activity | Extracellular;Nucleus |

Supplementary Table S4. List of all proteins identified in SCC-S and SCC-R cells using SequestHT and Mascot search algorithms and their gene ontology annotation

| Gene Symbol | RefSeq_Accession | Entrez Gene ID | Description | Coverage (%) | Proteins | Unique Peptides | # Peptides | PSM | Area | SCC-R /SCC-S | SCC-R /SCC-S (Count) | SCC-R /SCC-S (Variability [%]) | # AAs | MW [kDa] | calc. pI | Biological Process | Localization | Molecular Function | Primary Localization |
|---|---|---|---|---|---|---|---|---|---|---|---|---|---|---|---|---|---|---|---|
| **AGPAT1** | NP_116130.2 | 10554 | **1-acyl-sn-glycerol-3-phosphate acyltransferase alpha** | 7.4 | 1 | 1 | 1 | 1 | 1.22E+07 | **-** | - | - | 283 | 31.7 | 9.4 | Metabolism;Energy pathways | Endoplasmic reticulum membrane | Acyltransferase activity | Endoplasmic reticulum membrane |
| **AHSG** | NP_001613.2 | 197 | **alpha-2-HS-glycoprotein preproprotein** | 3.5 | 1 | 2 | 2 | 7 | 1.49E+08 | **-** | - | - | 367 | 39.3 | 5.7 | Cell communication;Signal transduction | Extracellular | Defense/immunity protein activity | Extracellular |
| **AIF1L** | NP_113614.1 | 83543 | **allograft inflammatory factor 1-like isoform 1** | 10.0 | 2 | 1 | 1 | 1 | 6.44E+07 | **-** | - | - | 150 | 17.1 | 7.2 | Biological_process unknown | Chromosome | Calcium ion binding | - |
| **AIFM2** | NP_116186.1 | 84883 | **apoptosis-inducing factor 2** | 13.7 | 1 | 2 | 2 | 6 | 3.12E+07 | **-** | - | - | 373 | 40.5 | 9.1 | Apoptosis | Cytoplasm;Mitochondrion | NADP binding;DNA binding | Mitochondrion |
| **AIM1** | NP_001615.1 | 202 | **absent in melanoma 1 protein** | 7.4 | 1 | 8 | 8 | 12 | 1.16E+08 | **-** | - | - | 1723 | 188.5 | 5.9 | Cell communication;Regulation of cell proliferation;Signal transduction | Cytoskeleton | Calcium ion binding | Cytoskeleton |
| **AK5** | NP_036225.2 | 26289 | **adenylate kinase isoenzyme 5 isoform 2** | 1.9 | 2 | 1 | 1 | 1 | 8.69E+07 | **-** | - | - | 536 | 60.3 | 5.1 | Metabolism;Energy pathways | Cytoplasm | ATPase activity | Cytoplasm |
| **AKAP17A** | NP_005079.2 | 8227 | **A-kinase anchor protein 17A isoform 1** | 4.0 | 1 | 1 | 1 | 2 | 2.11E+07 | **-** | - | - | 695 | 80.7 | 9.7 | Cell communication;Signal transduction | Plasma membrane;Nucleus;Cytoplasm | Molecular function unknown | Plasma membrane |
| **AKAP6** | NP_004265.3 | 9472 | **A-kinase anchor protein 6** | 0.6 | 1 | 1 | 1 | 1 | 6.22E+08 | **-** | - | - | 2319 | 256.6 | 5.0 | Cell growth and/or maintenance | Nucleus;Sarcoplasmic reticulum | Cytoskeletal anchoring activity | Nucleus |
| **AKIRIN1** | NP_001129747.1 | 79647 | **akirin-1 isoform 2** | 8.2 | 2 | 1 | 1 | 2 | 3.97E+07 | **-** | - | - | 147 | 16.5 | 9.0 | Biological_process unknown | - | Molecular function unknown | - |
| **AKR1C1** | NP_001344.2 | 1645 | **aldo-keto reductase family 1 member C1** | 32.2 | 6 | 2 | 7 | 23 | 8.58E+08 | **-** | - | - | 323 | 36.8 | 7.9 | Metabolism;Energy pathways | Cytoplasm | Catalytic activity | Cytoplasm |
| **AKR1C3** | NP_003730.4 | 8644 | **aldo-keto reductase family 1 member C3 isoform 1** | 29.7 | 6 | 2 | 7 | 21 | 7.38E+08 | **-** | - | - | 323 | 36.8 | 7.9 | Metabolism;Energy pathways | Cytoplasm | Catalytic activity | Cytoplasm |
| **ALB** | NP_000468.1 | 213 | **serum albumin preproprotein** | 5.6 | 1 | 1 | 4 | 5 | 3.13E+08 | **-** | - | - | 609 | 69.3 | 6.3 | Transport | Extracellular | Transporter activity | Extracellular |
| **ALDOC** | NP_005156.1 | 230 | **fructose-bisphosphate aldolase C** | 18.7 | 2 | 2 | 6 | 66 | 1.64E+09 | **-** | - | - | 364 | 39.4 | 6.9 | Metabolism;Energy pathways | Mitochondrion | Lyase activity | Mitochondrion |
| **ALG13** | NP_001244164.1 | 79868 | **putative bifunctional UDP-N-acetylglucosamine transferase and deubiquitinase ALG13 isoform 7** | 41.0 | 3 | 1 | 1 | 1 | 1.03E+07 | **-** | - | - | 61 | 6.9 | 7.9 | Biological_process unknown | - | Molecular function unknown | - |
| **ALG9** | NP_001071160.1 | 79796 | **alpha-1,2-mannosyltransferase ALG9 isoform d precursor** | 3.6 | 4 | 1 | 1 | 1 | 2.79E+06 | **-** | - | - | 440 | 50.8 | 8.7 | Metabolism;Energy pathways | Endoplasmic reticulum | Transferase activity | Endoplasmic reticulum |
| **ALPP** | NP_001623.3 | 250 | **alkaline phosphatase, placental type preproprotein** | 13.3 | 2 | 4 | 4 | 4 | 3.68E+07 | **-** | - | - | 535 | 57.9 | 6.3 | Metabolism;Energy pathways | Extracellular;Nucleus | Hydrolase activity | Extracellular |
| **AMIGO2** | NP_862830.1 | 347902 | **amphoterin-induced protein 2 precursor** | 5.4 | 1 | 1 | 1 | 2 | 4.60E+09 | **-** | - | - | 522 | 57.9 | 8.4 | Cell growth and/or maintenance | Plasma membrane;Nucleus;Cytoplasm;Microsome | Cell adhesion molecule activity | Plasma membrane |
| **ANAPC10** | NP_001243641.1 | 10393 | **anaphase-promoting complex subunit 10 isoform 4** | 15.7 | 3 | 1 | 1 | 1 | 7.75E+05 | **-** | - | - | 83 | 9.3 | 5.2 | Cell growth and/or maintenance | Nucleus;Centrosome;Microtubule | Protein binding | Nucleus |
| **ANAPC15** | NP_001265417.1 | 25906 | **anaphase-promoting complex subunit 15 isoform b** | 8.3 | 2 | 1 | 1 | 1 | 0.00E+00 | **-** | - | - | 121 | 14.3 | 3.6 | Biological_process unknown | - | Molecular function unknown | - |
| **ANK1** | NP_065210.2 | 286 | **ankyrin-1 isoform 2** | 0.4 | 5 | 1 | 1 | 1 | 4.56E+06 | **-** | - | - | 1719 | 188.9 | 6.6 | Cell communication;Signal transduction | Sarcoplasmic reticulum;Cytoplasm;Integral to membrane;Plasma membrane | Receptor signaling complex scaffold activity | Plasma membrane |
| **ANK2** | NP_001139.3 | 287 | **ankyrin-2 isoform 1** | 0.3 | 1 | 1 | 1 | 1 | 2.81E+08 | **-** | - | - | 3957 | 433.4 | 5.1 | Cell growth and/or maintenance | Plasma membrane;Endoplasmic reticulum;Cytoplasmic vesicle;Nucleus;Cytoplasm | Cytoskeletal protein binding | Plasma membrane;Cytoplasm |
| **ANK3** | NP_001191332.1 | 288 | **ankyrin-3 isoform 3** | 4.2 | 4 | 3 | 3 | 3 | 7.31E+07 | **-** | - | - | 1861 | 202.5 | 6.7 | Cell communication;Signal transduction | Plasma membrane;Cytoplasm | Receptor signaling complex scaffold activity | Plasma membrane |
| **ANKAR** | NP_653309.3 | 150709 | **ankyrin and armadillo repeat-containing protein** | 0.4 | 1 | 1 | 1 | 6 | 7.47E+08 | **-** | - | - | 1434 | 161.9 | 8.1 | Biological_process unknown | - | Molecular function unknown | - |
| **ANKRD12** | NP_001077094.1 | 23253 | **ankyrin repeat domain-containing protein 12 isoform 2** | 0.6 | 2 | 1 | 1 | 1 | 1.01E+08 | **-** | - | - | 2039 | 232.9 | 7.1 | Biological_process unknown | Nucleus | Molecular function unknown | Nucleus |
| **ANKRD2** | NP_001123453.1 | 26287 | **ankyrin repeat domain-containing protein 2 isoform b** | 42.5 | 4 | 7 | 7 | 8 | 2.29E+08 | **-** | - | - | 327 | 36.2 | 6.0 | Regulation of nucleobase, nucleoside, nucleotide and nucleic acid metabolism | Nucleus;Cytoplasm | Transcription regulator activity | Nucleus |
| **ANKRD24** | NP_597732.1 | 170961 | **ankyrin repeat domain-containing protein 24** | 1.9 | 1 | 1 | 1 | 1 | 2.07E+08 | **-** | - | - | 1146 | 124.1 | 5.0 | Biological_process unknown | - | Molecular function unknown | - |
| **ANKRD50** | NP_001161354.1 | 57182 | **ankyrin repeat domain-containing protein 50 isoform 2** | 1.8 | 2 | 1 | 1 | 1 | 2.19E+07 | **-** | - | - | 1250 | 136.4 | 6.4 | Biological_process unknown | - | Molecular function unknown | - |
| **ANKRD54** | NP_620152.1 | 129138 | **ankyrin repeat domain-containing protein 54** | 10.3 | 1 | 1 | 1 | 1 | 3.92E+07 | **-** | - | - | 300 | 32.5 | 6.3 | Biological_process unknown | Nucleus | Protein binding | Nucleus |
| **ANKZF1** | NP_001269721.1 | 55139 | **ankyrin repeat and zinc finger domain-containing protein 1 isoform 2** | 4.8 | 2 | 1 | 1 | 1 | 1.41E+07 | **-** | - | - | 516 | 57.5 | 9.5 | Biological_process unknown | Cytoplasm | Molecular function unknown | Cytoplasm |
| **ANXA8L1** | NP_001092315.2 | 728113 | **annexin A8-like protein 1 isoform 1** | 61.2 | 3 | 3 | 21 | 108 | 3.17E+09 | **-** | - | - | 327 | 36.9 | 5.8 | Signal transduction | - | Calcium ion binding | - |
| **AP1M2** | NP_005489.2 | 10053 | **AP-1 complex subunit mu-2** | 22.9 | 1 | 6 | 7 | 13 | 3.29E+08 | **-** | - | - | 423 | 48.1 | 8.2 | Cell communication;Signal transduction | Cytoplasm;Golgi apparatus | Receptor signaling complex scaffold activity | Cytoplasm |
| **APOA5** | NP_001160070.1 | 116519 | **apolipoprotein A-V precursor** | 4.9 | 1 | 1 | 1 | 1 | 8.61E+07 | **-** | - | - | 366 | 41.2 | 6.4 | Transport | Extracellular | Transporter activity | Extracellular |
| **APOO** | NP_077027.1 | 79135 | **apolipoprotein O precursor** | 10.1 | 1 | 1 | 1 | 1 | 0.00E+00 | **-** | - | - | 198 | 22.3 | 9.1 | Biological_process unknown | Mitochondrion | Molecular function unknown | Mitochondrion |
| **APTX** | NP_001182183.1 | 54840 | **aprataxin isoform h** | 13.5 | 7 | 2 | 2 | 2 | 1.76E+07 | **-** | - | - | 288 | 33.1 | 9.3 | DNA repair | Nucleoplasm;Nucleolus | Protein binding;Hydrolase activity | Nucleoplasm |
| **ARAP1** | NP_001128662.1 | 116985 | **arf-GAP with Rho-GAP domain, ANK repeat and PH domain-containing protein 1 isoform d** | 2.9 | 3 | 2 | 2 | 4 | 1.23E+08 | **-** | - | - | 1133 | 127.6 | 6.1 | Cell communication;Signal transduction | Golgi apparatus;Nucleus;Cytoplasm | GTPase activator activity | Golgi apparatus |
| **ARFGAP3** | NP_001135765.1 | 26286 | **ADP-ribosylation factor GTPase-activating protein 3 isoform 2** | 2.3 | 2 | 1 | 1 | 1 | 1.31E+07 | **-** | - | - | 472 | 52 | 6.1 | Protein metabolism | Cytoplasm | GTPase activator activity | Cytoplasm |
| **ARFIP2** | NP_036534.1 | 23647 | **arfaptin-2 isoform 2** | 7.6 | 4 | 2 | 2 | 2 | 1.27E+07 | **-** | - | - | 341 | 37.8 | 6.0 | Cell growth and/or maintenance | Nucleus;Cytoplasm | Structural molecule activity | Nucleus |

| Gene Symbol | RefSeq_Accession | Entrez Gene ID | Description | Coverage (%) | Proteins | Unique Peptides | # Peptides | PSM | Area | SCC-R /SCC-S | SCC-R /SCC-S (Count) | SCC-R /SCC-S (Variability [%]) | # AAs | MW [kDa] | calc. pI | Biological Process | Localization | Molecular Function | Primary Localization |
|---|---|---|---|---|---|---|---|---|---|---|---|---|---|---|---|---|---|---|---|
| ARFRP1 | NP_003215.1 | 10139 | ADP-ribosylation factor-related protein 1 isoform a | 11.4 | 5 | 2 | 2 | 3 | 7.41E+06 | - | - | - | 201 | 22.6 | 7.6 | Cell communication;Signal transduction | Plasma membrane | GTPase activity | Plasma membrane |
| ARG2 | NP_001163.1 | 384 | arginase-2, mitochondrial precursor | 6.8 | 1 | 1 | 1 | 1 | 2.22E+07 | - | - | - | 354 | 38.6 | 6.5 | Metabolism;Energy pathways | Mitochondrion | Hydrolase activity | Mitochondrion |
| ARHGAP18 | NP_277050.2 | 93663 | rho GTPase-activating protein 18 | 2.0 | 1 | 1 | 1 | 1 | 1.12E+07 | - | - | - | 663 | 74.9 | 6.4 | Cell communication;Signal transduction | - | GTPase activator activity | - |
| ARHGAP22 | NP_001242955.1 | 58504 | rho GTPase-activating protein 22 isoform 4 | 5.4 | 4 | 1 | 1 | 1 | 1.18E+08 | - | - | - | 608 | 66.6 | 7.2 | Cell communication;Signal transduction | - | GTPase activator activity | - |
| ARHGAP26 | NP_001129080.1 | 23092 | rho GTPase-activating protein 26 isoform b | 1.5 | 2 | 1 | 1 | 3 | 1.12E+09 | - | - | - | 759 | 86.1 | 6.7 | Cell communication;Signal transduction | Cytoplasm | GTPase activator activity | Cytoplasm |
| ARHGAP31 | NP_065805.2 | 57514 | rho GTPase-activating protein 31 | 1.8 | 1 | 1 | 1 | 1 | 4.42E+06 | - | - | - | 1444 | 156.9 | 5.8 | Cell communication;Signal transduction | - | GTPase activator activity | - |
| ARHGEF12 | NP_001185594.1 | 23365 | rho guanine nucleotide exchange factor 12 isoform 2 | 0.6 | 2 | 1 | 1 | 2 | 4.05E+07 | - | - | - | 1525 | 171 | 5.7 | Cell communication;Signal transduction | Cytoplasm;Plasma membrane | Guanyl-nucleotide exchange factor activity | Cytoplasm |
| ARHGEF2 | NP_004714.2 | 9181 | rho guanine nucleotide exchange factor 2 isoform 3 | 2.3 | 3 | 2 | 2 | 2 | 7.97E+06 | - | - | - | 958 | 108.2 | 6.9 | Cell communication;Signal transduction | Cytoskeleton;Microtubule;Cytoplasm | Guanyl-nucleotide exchange factor activity | Cytoskeleton |
| ARL6IP6 | NP_689735.1 | 151188 | ADP-ribosylation factor-like protein 6-interacting protein 6 | 17.3 | 1 | 1 | 1 | 1 | 3.55E+06 | - | - | - | 226 | 24.7 | 6.3 | Biological_process unknown | - | Molecular function unknown | - |
| ARMC9 | NP_079415.3 | 80210 | lisH domain-containing protein ARMC9 isoform 2 | 1.1 | 2 | 1 | 1 | 3 | 4.80E+07 | - | - | - | 665 | 75.5 | 5.5 | Biological_process unknown | - | Molecular function unknown | - |
| ARMCX4 | NP_001243084.2 | 100131755 | armadillo repeat-containing X-linked protein 4 | 1.0 | 1 | 1 | 1 | 1 | 1.94E+08 | - | - | - | 2290 | 235.8 | 5.8 | - | - | - | - |
| ARNT | NP_001184254.1 | 405 | aryl hydrocarbon receptor nuclear translocator isoform 4 | 1.7 | 5 | 1 | 1 | 1 | 1.19E+07 | - | - | - | 773 | 84.8 | 6.7 | Regulation of nucleobase, nucleoside, nucleotide and nucleic acid metabolism | Nucleus | Transcription factor activity | Nucleus |
| ARRB2 | NP_001244258.1 | 409 | beta-arrestin-2 isoform 4 | 9.4 | 5 | 1 | 1 | 1 | 2.16E+06 | - | - | - | 330 | 36.6 | 8.8 | Cell communication;Signal transduction | Cytoplasm;Nucleus;Plasma membrane | Receptor signaling complex scaffold activity | Cytoplasm |
| ARRDC3 | NP_065852.1 | 57561 | arrestin domain-containing protein 3 | 1.7 | 1 | 1 | 1 | 10 | 4.55E+09 | - | - | - | 414 | 46.4 | 6.6 | Cell communication;Signal transduction | - | Molecular function unknown | - |
| ASF1B | NP_060624.1 | 55723 | histone chaperone ASF1B | 7.4 | 1 | 1 | 1 | 1 | 7.35E+06 | - | - | - | 202 | 22.4 | 4.6 | Protein metabolism | Nucleus;Cytoplasm | Chaperone activity | Nucleus |
| ASPM | NP_060606.3 | 259266 | abnormal spindle-like microcephaly-associated protein isoform 1 | 0.2 | 1 | 1 | 1 | 1 | 4.22E+06 | - | - | - | 3477 | 409.5 | 10.5 | Biological_process unknown | Nucleus;Microtubule | Molecular function unknown | Nucleus |
| ASXL2 | NP_060733.4 | 55252 | putative Polycomb group protein ASXL2 | 1.5 | 1 | 1 | 1 | 1 | 3.93E+08 | - | - | - | 1435 | 153.7 | 8.8 | Regulation of nucleobase, nucleoside, nucleotide and nucleic acid metabolism | - | DNA binding | - |
| ATAD2B | NP_001229267.1 | 54454 | ATPase family AAA domain-containing protein 2B isoform 2 | 1.8 | 2 | 1 | 1 | 1 | 0.00E+00 | - | - | - | 1453 | 164.2 | 6.9 | Biological_process unknown | - | ATP binding | - |
| ATG2B | NP_060506.5 | 55102 | autophagy-related protein 2 homolog B | 1.5 | 1 | 1 | 1 | 1 | 2.14E+07 | - | - | - | 2078 | 232.6 | 5.8 | Biological_process unknown | - | Molecular function unknown | - |
| ATG4C | NP_116241.2 | 84938 | cysteine protease ATG4C | 2.8 | 1 | 1 | 1 | 1 | 2.50E+07 | - | - | - | 458 | 52.5 | 6.0 | Protein metabolism | - | Endopeptidase activity | - |
| ATP11A | NP_056020.2 | 23250 | probable phospholipid-transporting ATPase IH isoform a | 0.7 | 2 | 1 | 1 | 1 | 1.08E+09 | - | - | - | 1134 | 129.7 | 6.6 | Ion transport | Integral to membrane | ATPase activity;Ion transporter activity | Integral to membrane |
| ATP5L | NP_006467.4 | 10632 | ATP synthase subunit g, mitochondrial | 12.6 | 1 | 1 | 1 | 2 | 3.98E+07 | - | - | - | 103 | 11.4 | 9.6 | Metabolism;Energy pathways | Mitochondrion | ATPase activity | Mitochondrion |
| ATP6V0A2 | NP_036595.2 | 23545 | V-type proton ATPase 116 kDa subunit a isoform 2 | 2.0 | 1 | 1 | 1 | 2 | 1.10E+07 | - | - | - | 856 | 98 | 6.6 | Metabolism;Energy pathways | Integral to membrane;Early endosome;Nucleus | ATPase activity | Integral to membrane |
| ATP8 | YP_003024030.1 | 4509 | ATP synthase F0 subunit 8 | 16.2 | 1 | 1 | 1 | 1 | 4.07E+07 | - | - | - | 68 | 8 | 9.9 | - | - | - | - |
| ATP8A2 | NP_057613.4 | 51761 | probable phospholipid-transporting ATPase IB | 0.8 | 1 | 1 | 1 | 1 | 0.00E+00 | - | - | - | 1188 | 133.5 | 8.1 | Transport | - | ATPase activity | - |
| ATPAF2 | NP_663729.1 | 91647 | ATP synthase mitochondrial F1 complex assembly factor 2 | 4.5 | 1 | 1 | 1 | 1 | 5.17E+06 | - | - | - | 289 | 32.8 | 7.1 | Metabolism;Energy pathways | Mitochondrion | Molecular function unknown | Mitochondrion |
| ATXN7L3B | NP_001129734.1 | 552889 | putative ataxin-7-like protein 3B | 24.7 | 1 | 1 | 1 | 1 | 0.00E+00 | - | - | - | 97 | 10.8 | 4.4 | Biological_process unknown | - | Molecular function unknown | - |
| AUP1 | NP_853553.1 | 550 | ancient ubiquitous protein 1 | 6.1 | 1 | 2 | 2 | 2 | 8.64E+07 | - | - | - | 410 | 45.8 | 8.7 | Cell communication;Signal transduction | Cytoplasm | Molecular function unknown | Cytoplasm |
| AVL9 | NP_055875.1 | 23080 | late secretory pathway protein AVL9 homolog | 7.7 | 1 | 3 | 3 | 3 | 2.12E+06 | - | - | - | 648 | 71.9 | 6.2 | Biological_process unknown | - | Molecular function unknown | - |
| B3GAT2 | NP_542780.1 | 135152 | galactosylgalactosylxylosylprotein 3-beta-glucuronosyltransferase 2 | 3.4 | 1 | 1 | 1 | 1 | 0.00E+00 | - | - | - | 323 | 36.9 | 10.6 | Metabolism;Energy pathways;Carbohydrate metabolism | Golgi apparatus | Transferase activity | Golgi apparatus |
| B3GNT1 | NP_006867.1 | 11041 | N-acetyllactosaminide beta-1,3-N-acetylglucosaminyltransferase | 10.6 | 1 | 2 | 2 | 3 | 4.89E+07 | - | - | - | 415 | 47.1 | 7.2 | Metabolism;Energy pathways | Golgi apparatus | Transferase activity | Golgi apparatus |
| BACH1 | NP_996749.1 | 571 | transcription regulator protein BACH1 | 3.7 | 1 | 2 | 2 | 2 | 1.55E+07 | - | - | - | 736 | 81.9 | 5.1 | Regulation of nucleobase, nucleoside, nucleotide and nucleic acid metabolism | Nucleus;Cytoplasm | Transcription regulator activity | Nucleus |
| BAK1 | NP_001179.1 | 578 | bcl-2 homologous antagonist/killer | 11.9 | 1 | 1 | 1 | 1 | 2.22E+07 | - | - | - | 211 | 23.4 | 6.0 | Apoptosis | Mitochondrion;Endoplasmic reticulum;Membrane fraction | Receptor signaling complex scaffold activity | Mitochondrion |
| BANP | NP_001167012.1 | 54971 | protein BANP isoform e | 2.8 | 7 | 1 | 1 | 1 | 2.83E+06 | - | - | - | 466 | 50.9 | 5.5 | Regulation of nucleobase, nucleoside, nucleotide and nucleic acid metabolism;Signal transduction;Cell communication | Nucleus | Transcription factor activity;Signal transducer activity | Nucleus |
| BAZ1A | NP_872589.1 | 11177 | bromodomain adjacent to zinc finger domain protein 1A isoform b | 3.6 | 2 | 3 | 3 | 3 | 9.80E+06 | - | - | - | 1524 | 175.3 | 6.8 | Regulation of nucleobase, nucleoside, nucleotide and nucleic acid metabolism | Nucleus | DNA binding | Nucleus |
| BCL10 | NP_003912.1 | 8915 | B-cell lymphoma/leukemia 10 | 18.0 | 1 | 2 | 2 | 2 | 6.40E+07 | - | - | - | 233 | 26.2 | 5.7 | Cell communication;Signal transduction | Cytoplasm;Nucleus | Receptor signaling complex scaffold activity | Cytoplasm |
| BCL7A | NP_001019979.1 | 605 | B-cell CLL/lymphoma 7 protein family member A isoform b | 21.9 | 6 | 2 | 2 | 4 | 1.19E+08 | - | - | - | 210 | 22.8 | 5.1 | Cell communication;Signal transduction | Cytoplasm | Receptor signaling complex scaffold activity | Cytoplasm |

Supplementary Table S4. List of all proteins identified in SCC-S and SCC-R cells using SequestHT and Mascot search algorithms and their gene ontology annotation

| Gene Symbol | RefSeq_Accession | Entrez Gene ID | Description | Coverage (%) | Proteins | Unique Peptides | # Peptides | PSM | Area | SCC-R /SCC-S | SCC-R /SCC-S (Count) | SCC-R /SCC-S (Variability [%]) | # AAs | MW [kDa] | calc. pI | Biological Process | Localization | Molecular Function | Primary Localization |
|---|---|---|---|---|---|---|---|---|---|---|---|---|---|---|---|---|---|---|---|
| BCL9L | NP_872363.1 | 283149 | B-cell CLL/lymphoma 9-like protein | 2.0 | 1 | 1 | 1 | 1 | 2.18E+07 | - | - | - | 1499 | 157 | 8.6 | Biological_process unknown | Nucleus;Cytoplasm | Molecular function unknown | Nucleus |
| BDH2 | NP_064524.3 | 56898 | 3-hydroxybutyrate dehydrogenase type 2 | 6.1 | 1 | 1 | 1 | 1 | 4.19E+07 | - | - | - | 245 | 26.7 | 7.7 | Metabolism;Energy pathways | - | Oxidoreductase activity | - |
| BICC1 | NP_001073981.1 | 80114 | protein bicaudal C homolog 1 | 2.1 | 1 | 1 | 1 | 1 | 3.05E+06 | - | - | - | 974 | 104.8 | 8.5 | Biological_process unknown | - | RNA binding | - |
| BNIP1 | NP_053581.2 | 662 | vesicle transport protein SEC20 isoform BNIP1-a | 9.8 | 4 | 1 | 1 | 1 | 1.23E+06 | - | - | - | 194 | 22.3 | 9.0 | Apoptosis | Cytoplasm;Nucleus | Receptor signaling complex scaffold activity | Cytoplasm |
| BPIFA1 | NP_570913.1 | 51297 | BPI fold-containing family A member 1 precursor | 4.3 | 1 | 1 | 1 | 2 | 8.43E+06 | - | - | - | 256 | 26.7 | 5.8 | Inflammatory response | Extracellular | Molecular function unknown | Extracellular |
| BRAT1 | NP_689956.2 | 221927 | BRCA1-associated ATM activator 1 | 5.7 | 1 | 2 | 2 | 2 | 1.23E+06 | - | - | - | 821 | 88.1 | 5.3 | Biological_process unknown | - | Molecular function unknown | - |
| BRD8 | NP_001157798.1 | 10902 | bromodomain-containing protein 8 isoform 4 | 1.3 | 3 | 1 | 1 | 1 | 1.87E+07 | - | - | - | 866 | 94.2 | 4.7 | Cell communication;Signal transduction;Regulation of nucleobase, nucleoside, nucleotide and nucleic acid metabolism | Nucleus | Transcription regulator activity | Nucleus |
| BSN | NP_003449.2 | 8927 | protein bassoon | 0.5 | 1 | 1 | 1 | 2 | 1.13E+08 | - | - | - | 3926 | 416.2 | 7.6 | Regulation of nucleobase, nucleoside, nucleotide and nucleic acid metabolism | Nucleus | Transcription factor activity | Nucleus |
| BTBD8 | NP_899065.2 | 284697 | BTB/POZ domain-containing protein 8 | 2.9 | 1 | 1 | 1 | 1 | 7.02E+06 | - | - | - | 378 | 42.8 | 6.0 | Biological_process unknown | - | Molecular function unknown | - |
| BTD | NP_001268655.1 | 686 | biotinidase isoform 5 | 9.5 | 5 | 1 | 1 | 1 | 2.02E+07 | - | - | - | 179 | 20.1 | 6.4 | Metabolism;Energy pathways | Extracellular | Hydrolase activity | Extracellular |
| BTF3 | NP_001032726.1 | 689 | transcription factor BTF3 isoform A | 62.6 | 1 | 1 | 9 | 89 | 1.36E+09 | - | - | - | 206 | 22.2 | 9.4 | Regulation of nucleobase, nucleoside, nucleotide and nucleic acid metabolism | Nucleus | Transcription factor activity | Nucleus |
| BTN2A1 | NP_001184162.1 | 11120 | butyrophilin subfamily 2 member A1 isoform 3 | 5.6 | 1 | 1 | 1 | 1 | 4.72E+07 | - | - | - | 466 | 53.2 | 6.7 | Immune response | Integral to membrane | Antigen binding | Integral to membrane |
| C10orf11 | NP_114413.1 | 83938 | leucine-rich repeat-containing protein C10orf11 | 5.6 | 1 | 1 | 1 | 1 | 1.21E+06 | - | - | - | 198 | 22.6 | 6.7 | Biological_process unknown | - | Molecular function unknown | - |
| C11orf88 | NP_997313.2 | 399949 | UPF0722 protein C11orf88 isoform 1 | 3.1 | 1 | 1 | 1 | 2 | 5.98E+07 | - | - | - | 196 | 22.5 | 8.9 | Biological_process unknown | - | Molecular function unknown | - |
| C14orf119 | NP_060394.1 | 55017 | uncharacterized protein C14orf119 | 8.6 | 1 | 1 | 1 | 1 | 2.30E+06 | - | - | - | 140 | 16 | 4.9 | Biological_process unknown | - | Molecular function unknown | - |
| C14orf2 | NP_004885.1 | 9556 | 6.8 kDa mitochondrial proteolipid isoform 1 | 12.1 | 1 | 1 | 1 | 1 | 3.60E+06 | - | - | - | 58 | 6.7 | 10.1 | Biological_process unknown | - | Molecular function unknown | - |
| C14orf93 | NP_001269899.1 | 60686 | uncharacterized protein C14orf93 isoform c | 2.6 | 2 | 1 | 1 | 1 | 7.66E+06 | - | - | - | 498 | 54.1 | 5.4 | Biological_process unknown | - | Molecular function unknown | - |
| C16orf80 | NP_037374.1 | 29105 | UPF0468 protein C16orf80 | 8.8 | 1 | 1 | 1 | 2 | 9.58E+06 | - | - | - | 193 | 22.8 | 9.8 | Cell growth and/or maintenance | - | Transcription factor activity | - |
| C16orf91 | NP_001258980.1 | 283951 | protein CCSMST1 precursor | 17.4 | 1 | 1 | 1 | 1 | 8.19E+07 | - | - | - | 132 | 15 | 7.0 | Biological_process unknown | - | Molecular function unknown | - |
| C19orf25 | NP_689695.2 | 148223 | UPF0449 protein C19orf25 | 17.0 | 1 | 1 | 1 | 1 | 2.23E+07 | - | - | - | 118 | 12.9 | 5.1 | Biological_process unknown | - | Molecular function unknown | - |
| C19orf33 | NP_277055.1 | 64073 | immortalization up-regulated protein | 25.5 | 1 | 1 | 1 | 7 | 5.19E+08 | - | - | - | 106 | 10.9 | 9.7 | Regulation of nucleobase, nucleoside, nucleotide and nucleic acid metabolism | Nucleus;Cytoplasm | Transcription regulator activity | Nucleus |
| C19orf54 | NP_940878.3 | 284325 | UPF0692 protein C19orf54 | 8.0 | 1 | 1 | 1 | 1 | 8.78E+07 | - | - | - | 351 | 37.8 | 7.5 | Biological_process unknown | - | Molecular function unknown | - |
| C1orf116 | NP_001077393.1 | 79098 | specifically androgen-regulated gene protein isoform 2 | 4.2 | 2 | 1 | 1 | 1 | 4.76E+06 | - | - | - | 355 | 37.5 | 10.1 | Biological_process unknown | Cytoplasm | Molecular function unknown | Cytoplasm |
| C1orf131 | NP_689592.2 | 128061 | uncharacterized protein C1orf131 | 3.8 | 1 | 1 | 1 | 1 | 9.93E+07 | - | - | - | 293 | 32.6 | 9.9 | Biological_process unknown | - | Molecular function unknown | - |
| C2CD5 | NP_055617.1 | 9847 | C2 domain-containing protein 5 isoform e | 5.4 | 5 | 2 | 2 | 3 | 5.06E+06 | - | - | - | 1000 | 110.4 | 5.7 | Biological_process unknown | Nucleus;Cytoplasm | Molecular function unknown | Nucleus;Cytoplasm |
| C2orf43 | NP_001269652.1 | 60526 | UPF0554 protein C2orf43 isoform e | 8.4 | 2 | 1 | 1 | 2 | 1.43E+06 | - | - | - | 166 | 18.7 | 8.7 | Biological_process unknown | - | Molecular function unknown | - |
| C6orf132 | NP_001157918.1 | 647024 | uncharacterized protein C6orf132 | 0.9 | 1 | 1 | 1 | 1 | 3.67E+06 | - | - | - | 1188 | 124 | 9.5 | - | - | - | - |
| C7orf65 | NP_001116537.1 | 401335 | uncharacterized protein C7orf65 | 8.6 | 1 | 1 | 1 | 1 | 1.70E+07 | - | - | - | 151 | 16.8 | 9.4 | - | - | - | - |
| C8orf82 | NP_001001795.1 | 414919 | UPF0598 protein C8orf82 | 8.3 | 1 | 1 | 1 | 1 | 1.28E+07 | - | - | - | 216 | 23.9 | 9.1 | Biological_process unknown | - | Molecular function unknown | - |
| CA13 | NP_940986.1 | 377677 | carbonic anhydrase 13 | 9.5 | 1 | 1 | 1 | 1 | 2.51E+06 | - | - | - | 262 | 29.4 | 7.0 | Metabolism;Energy pathways | Cytoplasm | Catalytic activity | Cytoplasm |
| CABLES1 | NP_001243367.1 | 91768 | CDK5 and ABL1 enzyme substrate 1 isoform 3 | 2.3 | 3 | 1 | 1 | 1 | 1.35E+07 | - | - | - | 306 | 34.6 | 9.3 | Cell growth and/or maintenance | Nucleus;Cytoplasm;Integral to membrane;Mitochondrion | Receptor signaling complex scaffold activity | Nucleus |
| CACNA1A | NP_001120694.1 | 773 | voltage-dependent P/Q-type calcium channel subunit alpha-1A isoform 4 | 1.0 | 2 | 1 | 1 | 1 | 4.37E+08 | - | - | - | 2506 | 282.4 | 8.8 | Transport | Integral to membrane | Voltage-gated ion channel activity | Integral to membrane |
| CACNA2D1 | NP_000713.2 | 781 | voltage-dependent calcium channel subunit alpha-2/delta-1 precursor | 1.8 | 1 | 1 | 1 | 1 | 2.56E+07 | - | - | - | 1091 | 123.1 | 5.3 | Transport | Plasma membrane | Voltage-gated ion channel activity | Plasma membrane |
| CADM1 | NP_001091987.1 | 23705 | cell adhesion molecule 1 isoform 2 precursor | 5.8 | 2 | 1 | 1 | 1 | 1.85E+07 | - | - | - | 414 | 45.6 | 5.1 | Cell communication;Signal transduction | Plasma membrane;Nucleus | Cell adhesion molecule activity | Plasma membrane |
| CALD1 | NP_149129.2 | 800 | caldesmon isoform 1 | 28.6 | 3 | 1 | 19 | 49 | 4.21E+08 | - | - | - | 793 | 93.2 | 5.7 | Cell growth and/or maintenance | Cytoplasm;Plasma membrane;Nucleus | Cytoskeletal protein binding | Cytoplasm;Nucleus |
| CALML5 | NP_059118.2 | 51806 | calmodulin-like protein 5 | 11.6 | 1 | 1 | 1 | 1 | 1.04E+08 | - | - | - | 146 | 15.9 | 4.4 | Cell communication;Signal transduction | Extracellular | Calcium ion binding | Extracellular |
| CALY | NP_056537.1 | 50632 | neuron-specific vesicular protein calcyon | 11.5 | 1 | 1 | 1 | 1 | 8.76E+08 | - | - | - | 217 | 23.4 | 6.9 | Cell communication | Plasma membrane;Cytoplasm;Golgi apparatus;Endoplasmic reticulum;Dendrite | Receptor activity | Plasma membrane |

| Gene Symbol | RefSeq_Accession | Entrez Gene ID | Description | Coverage (%) | Proteins | Unique Peptides | # Peptides | PSM | Area | SCC-R /SCC-S | SCC-R /SCC-S (Count) | SCC-R /SCC-S (Variability [%]) | # AAs | MW [kDa] | calc. pI | Biological Process | Localization | Molecular Function | Primary Localization |
|---|---|---|---|---|---|---|---|---|---|---|---|---|---|---|---|---|---|---|---|
| CAPS | NP_542157.1 | 828 | calcyphosin isoform b | 5.6 | 2 | 1 | 1 | 1 | 2.41E+06 | - | - | - | 162 | 17.8 | 5.1 | Cell communication;Signal transduction | Cytoplasm | Calcium ion binding | Cytoplasm |
| CARD16 | NP_443121.1 | 114769 | caspase recruitment domain-containing protein 16 isoform 2 | 51.6 | 2 | 2 | 4 | 7 | 8.83E+08 | - | - | - | 97 | 10.7 | 5.2 | Apoptosis | - | Molecular function unknown | - |
| CASC4 | NP_816929.1 | 113201 | protein CASC4 isoform b | 4.5 | 2 | 1 | 1 | 2 | 0.00E+00 | - | - | - | 380 | 43.3 | 6.7 | Biological_process unknown | - | Molecular function unknown | - |
| CASP6 | NP_116787.1 | 839 | caspase-6 isoform beta | 9.8 | 2 | 1 | 1 | 1 | 2.83E+07 | - | - | - | 204 | 22.6 | 7.2 | Apoptosis | Cytoplasm;Nucleus | Cysteine-type peptidase activity | Cytoplasm |
| CASS4 | NP_001157586.1 | 57091 | cas scaffolding protein family member 4 isoform b | 1.5 | 2 | 1 | 1 | 1 | 2.60E+06 | - | - | - | 732 | 81.1 | 7.1 | Cell communication;Signal transduction | - | Molecular function unknown | - |
| CAV2 | NP_001193677.1 | 858 | caveolin-2 isoform d | 20.3 | 3 | 1 | 1 | 1 | 2.77E+07 | - | - | - | 79 | 8.7 | 8.2 | Cell communication;Signal transduction | Plasma membrane;Golgi apparatus | Protein binding | Plasma membrane |
| CC2D2A | NP_001073991.2 | 57545 | coiled-coil and C2 domain-containing protein 2A isoform a | 1.3 | 1 | 1 | 1 | 1 | 0.00E+00 | - | - | - | 1620 | 186.1 | 6.7 | Biological_process unknown | - | Molecular function unknown | - |
| CCDC116 | NP_689825.2 | 164592 | coiled-coil domain-containing protein 116 | 1.3 | 1 | 1 | 1 | 1 | 4.91E+06 | - | - | - | 613 | 67.9 | 8.5 | Biological_process unknown | - | Molecular function unknown | - |
| CCDC122 | NP_659411.2 | 160857 | coiled-coil domain-containing protein 122 | 4.8 | 1 | 1 | 1 | 1 | 4.53E+07 | - | - | - | 273 | 32.2 | 7.0 | Biological_process unknown | - | Molecular function unknown | - |
| CCDC51 | NP_001243895.1 | 79714 | coiled-coil domain-containing protein 51 isoform 2 | 7.3 | 2 | 1 | 1 | 1 | 1.04E+06 | - | - | - | 302 | 33.6 | 7.0 | Biological_process unknown | - | Molecular function unknown | - |
| CCDC59 | NP_054886.2 | 29080 | thyroid transcription factor 1-associated protein 26 | 7.5 | 1 | 1 | 1 | 2 | 3.96E+07 | - | - | - | 241 | 28.7 | 9.9 | Biological_process unknown | - | Molecular function unknown | - |
| CCDC64B | NP_001096645.1 | 146439 | bicaudal D-related protein 2 | 2.4 | 1 | 1 | 1 | 1 | 0.00E+00 | - | - | - | 508 | 56.8 | 5.0 | Biological_process unknown | - | Molecular function unknown | - |
| CCDC85C | NP_001138467.1 | 317762 | coiled-coil domain-containing protein 85C | 4.3 | 1 | 1 | 1 | 1 | 2.81E+07 | - | - | - | 419 | 45.2 | 7.0 | Biological_process unknown | - | Molecular function unknown | - |
| CCDC88A | NP_001241872.1 | 55704 | girdin isoform 3 | 0.7 | 3 | 1 | 1 | 2 | 2.37E+07 | - | - | - | 1796 | 207.7 | 6.0 | Cell growth and/or maintenance;Cell migration | Cytoskeleton;Plasma membrane | Structural constituent of cytoskeleton | Cytoskeleton |
| CCDC9 | NP_056418.1 | 26093 | coiled-coil domain-containing protein 9 | 3.6 | 1 | 1 | 1 | 1 | 1.02E+08 | - | - | - | 531 | 59.7 | 5.4 | Biological_process unknown | Nucleus | Molecular function unknown | Nucleus |
| CCDC93 | NP_061917.3 | 54520 | coiled-coil domain-containing protein 93 | 5.6 | 1 | 2 | 2 | 2 | 1.15E+07 | - | - | - | 631 | 73.2 | 8.2 | Biological_process unknown | - | Molecular function unknown | - |
| CCDC94 | NP_060544.2 | 55702 | coiled-coil domain-containing protein 94 | 5.9 | 1 | 1 | 1 | 1 | 4.88E+06 | - | - | - | 323 | 37.1 | 5.9 | Biological_process unknown | Nucleus | Molecular function unknown | Nucleus |
| CCDC97 | NP_443080.1 | 90324 | coiled-coil domain-containing protein 97 | 18.7 | 1 | 3 | 3 | 3 | 1.47E+07 | - | - | - | 343 | 38.9 | 4.6 | Biological_process unknown | Nucleus;Cytoplasm | Molecular function unknown | Nucleus;Cytoplasm |
| CCND1 | NP_444284.1 | 595 | G1/S-specific cyclin-D1 | 5.1 | 1 | 1 | 1 | 1 | 6.47E+06 | - | - | - | 295 | 33.7 | 5.0 | Cell communication;Signal transduction | Nucleus;Cytoplasm | Kinase binding | Nucleus |
| CCNDBP1 | NP_036274.3 | 23582 | cyclin-D1-binding protein 1 | 3.6 | 1 | 1 | 1 | 1 | 8.69E+07 | - | - | - | 360 | 40.2 | 4.8 | Cell communication;Signal transduction;Biological_process unknown | Extracellular;Nucleus | Molecular function unknown | Extracellular |
| CCNH | NP_001230.1 | 902 | cyclin-H isoform 1 | 9.6 | 1 | 2 | 2 | 2 | 2.40E+07 | - | - | - | 323 | 37.6 | 7.2 | Regulation of nucleobase, nucleoside, nucleotide and nucleic acid metabolism | Nucleus | Transcription regulator activity | Nucleus |
| CD276 | NP_001019907.1 | 80381 | CD276 antigen isoform a precursor | 5.2 | 2 | 1 | 1 | 1 | 9.27E+06 | - | - | - | 534 | 57.2 | 4.9 | Immune response | Plasma membrane | Receptor binding | Plasma membrane |
| CD58 | NP_001138294.1 | 965 | lymphocyte function-associated antigen 3 isoform 2 | 4.8 | 2 | 1 | 1 | 1 | 1.09E+08 | - | - | - | 248 | 27.9 | 7.0 | Immune response | Plasma membrane;Cytoplasm | Molecular function unknown | Plasma membrane |
| CDA | NP_001776.1 | 978 | cytidine deaminase | 25.3 | 1 | 2 | 2 | 2 | 1.48E+07 | - | - | - | 146 | 16.2 | 6.9 | Metabolism;Energy pathways | Nucleus;Cytoplasm | Deaminase activity | Nucleus |
| CDC123 | NP_006014.2 | 8872 | cell division cycle protein 123 homolog | 1.8 | 1 | 1 | 1 | 1 | 1.74E+07 | - | - | - | 336 | 39.1 | 4.8 | Cell communication;Signal transduction | Cytoplasm | Molecular function unknown | Cytoplasm |
| CDC37L1 | NP_060383.2 | 55664 | hsp90 co-chaperone Cdc37-like 1 | 11.9 | 1 | 2 | 2 | 2 | 2.12E+07 | - | - | - | 337 | 38.8 | 5.3 | Protein metabolism | Cytoplasm | Heat shock protein activity | Cytoplasm |
| CDC40 | NP_056975.1 | 51362 | pre-mRNA-processing factor 17 | 2.1 | 1 | 1 | 1 | 1 | 2.28E+06 | - | - | - | 579 | 65.5 | 7.1 | Regulation of nucleobase, nucleoside, nucleotide and nucleic acid metabolism | Nucleus;Cytoplasm | Transcription regulator activity | Nucleus |
| CDC42BPA | NP_055641.3 | 8476 | serine/threonine-protein kinase MRCK alpha isoform A | 1.7 | 2 | 1 | 1 | 1 | 0.00E+00 | - | - | - | 1638 | 186 | 6.3 | Cell communication;Signal transduction;Cytoskeleton organization and biogenesis | Cytoplasm;Cell junction | Protein serine/threonine kinase activity | Cell junction |
| CDC42BPG | NP_059995.2 | 55561 | serine/threonine-protein kinase MRCK gamma | 1.2 | 1 | 1 | 1 | 3 | 9.01E+07 | - | - | - | 1551 | 172.4 | 6.3 | Cell communication;Signal transduction | Cytoplasm | Protein serine/threonine kinase activity | Cytoplasm |
| CDC42SE2 | NP_064625.1 | 56990 | CDC42 small effector protein 2 | 44.1 | 1 | 1 | 1 | 2 | 9.42E+06 | - | - | - | 84 | 9.2 | 8.4 | Cell communication;Signal transduction | Plasma membrane | Molecular function unknown | Plasma membrane |
| CDH1 | NP_004351.1 | 999 | cadherin-1 preproprotein | 4.4 | 1 | 2 | 2 | 3 | 1.36E+08 | - | - | - | 882 | 97.4 | 4.7 | Cell communication;Signal transduction | Plasma membrane;Cytoplasm;Extracellular;Cell junction | Cell adhesion molecule activity | Plasma membrane |
| CDH19 | NP_001257957.1 | 28513 | cadherin-19 isoform 2 preproprotein | 4.3 | 2 | 1 | 1 | 1 | 1.63E+08 | - | - | - | 490 | 55.3 | 4.9 | Cell communication;Signal transduction | Plasma membrane | Cell adhesion molecule activity | Plasma membrane |
| CDH3 | NP_001784.2 | 1001 | cadherin-3 preproprotein | 19.4 | 1 | 8 | 8 | 17 | 6.00E+08 | - | - | - | 829 | 91.4 | 4.8 | Cell communication;Signal transduction | Plasma membrane;Cytoplasm;Extracellular | Cell adhesion molecule activity | Plasma membrane |
| CDH5 | NP_001786.2 | 1003 | cadherin-5 preproprotein | 1.2 | 1 | 1 | 1 | 1 | 0.00E+00 | - | - | - | 784 | 87.5 | 5.4 | Cell growth and/or maintenance | Plasma membrane;Cell junction | Cell adhesion molecule activity | Plasma membrane |
| CDX2 | NP_001256.3 | 1045 | homeobox protein CDX-2 | 2.6 | 1 | 1 | 1 | 1 | 2.96E+07 | - | - | - | 313 | 33.5 | 9.6 | Regulation of nucleobase, nucleoside, nucleotide and nucleic acid metabolism | Nucleus | Transcription factor activity | Nucleus |
| CEBPB | NP_001272807.1 | 1051 | CCAAT/enhancer-binding protein beta isoform b | 17.4 | 3 | 3 | 3 | 4 | 8.37E+07 | - | - | - | 322 | 33.6 | 8.2 | Regulation of nucleobase, nucleoside, nucleotide and nucleic acid metabolism | Nucleus;Cytoplasm | Transcription factor activity | Nucleus |
| CEBPG | NP_001239225.1 | 1054 | CCAAT/enhancer-binding protein gamma | 20.7 | 1 | 1 | 1 | 1 | 2.45E+06 | - | - | - | 150 | 16.4 | 9.8 | Regulation of nucleobase, nucleoside, nucleotide and nucleic acid metabolism | Nucleus | Transcription regulator activity | Nucleus |

| Gene Symbol | RefSeq_Accession | Entrez Gene ID | Description | Coverage (%) | Proteins | Unique Peptides | # Peptides | PSM | Area | SCC-R /SCC-S | SCC-R /SCC-S (Count) | SCC-R /SCC-S (Variability [%]) | # AAs | MW [kDa] | calc. pI | Biological Process | Localization | Molecular Function | Primary Localization |
|---|---|---|---|---|---|---|---|---|---|---|---|---|---|---|---|---|---|---|---|
| CENPB | NP_001801.1 | 1059 | major centromere autoantigen B | 2.8 | 1 | 1 | 1 | 2 | 0.00E+00 | - | - | - | 599 | 65.1 | 4.6 | Regulation of nucleobase, nucleoside, nucleotide and nucleic acid metabolism | Nucleus;Nucleolus;Chromosome;Kinetochore | DNA binding | Nucleus |
| CENPC | NP_001803.2 | 1060 | centromere protein C | 1.5 | 1 | 1 | 1 | 1 | 6.48E+06 | - | - | - | 943 | 106.8 | 9.4 | Regulation of nucleobase, nucleoside, nucleotide and nucleic acid metabolism | Nucleus;Nucleolus;Kinetochore | DNA binding | Nucleus |
| CENPV | NP_859067.2 | 201161 | centromere protein V | 5.2 | 1 | 1 | 1 | 1 | 1.81E+07 | - | - | - | 272 | 29.7 | 9.7 | Regulation of nucleobase, nucleoside, nucleotide and nucleic acid metabolism | Nucleus;Cytoplasm | DNA binding | Nucleus |
| CEP152 | NP_055800.2 | 22995 | centrosomal protein of 152 kDa isoform 2 | 0.7 | 2 | 1 | 1 | 1 | 3.80E+06 | - | - | - | 1654 | 189 | 5.4 | Biological_process unknown | Centrosome | Molecular function unknown | Centrosome |
| CEP41 | NP_001244088.1 | 95681 | centrosomal protein of 41 kDa isoform 3 | 6.3 | 3 | 1 | 1 | 1 | 2.68E+06 | - | - | - | 285 | 31.6 | 7.5 | Biological_process unknown | Centrosome | Molecular function unknown | Centrosome |
| CEP85 | NP_073615.2 | 64793 | centrosomal protein of 85 kDa isoform 1 | 3.2 | 1 | 1 | 1 | 1 | 2.06E+07 | - | - | - | 762 | 85.6 | 6.0 | Biological_process unknown | - | Molecular function unknown | - |
| CFB | NP_001701.2 | 629 | complement factor B preproprotein | 3.1 | 1 | 1 | 1 | 1 | 1.35E+07 | - | - | - | 764 | 85.5 | 7.1 | Immune response | Extracellular | Complement activity | Extracellular |
| CFLAR | NP_001189447.1 | 8837 | CASP8 and FADD-like apoptosis regulator isoform 6 | 5.5 | 3 | 1 | 1 | 1 | 0.00E+00 | - | - | - | 366 | 41.3 | 7.2 | Cell communication;Signal transduction | Cytoplasm;Nucleus | Receptor signaling complex scaffold activity | Cytoplasm |
| CHCHD1 | NP_976043.1 | 118487 | coiled-coil-helix-coiled-coil-helix domain-containing protein 1 | 9.3 | 1 | 1 | 1 | 1 | 1.15E+07 | - | - | - | 118 | 13.5 | 10.2 | Biological_process unknown | Nucleus | Molecular function unknown | Nucleus |
| CHCHD10 | NP_998885.1 | 400916 | coiled-coil-helix-coiled-coil-helix domain-containing protein 10, mitochondrial precursor | 18.3 | 1 | 1 | 1 | 1 | 9.68E+06 | - | - | - | 142 | 14.1 | 7.8 | Biological_process unknown | - | Molecular function unknown | - |
| CHCHD6 | NP_115719.1 | 84303 | coiled-coil-helix-coiled-coil-helix domain-containing protein 6, mitochondrial | 10.2 | 1 | 1 | 1 | 1 | 8.49E+07 | - | - | - | 235 | 26.4 | 8.9 | Biological_process unknown | - | Molecular function unknown | - |
| CHD1 | NP_001261.2 | 1105 | chromodomain-helicase-DNA-binding protein 1 | 4.8 | 1 | 4 | 4 | 7 | 3.24E+07 | - | - | - | 1710 | 196.6 | 7.1 | Regulation of nucleobase, nucleoside, nucleotide and nucleic acid metabolism | Nucleus;Cytoplasm | DNA binding | Nucleus |
| CHMP1A | NP_002759.2 | 5119 | charged multivesicular body protein 1a isoform 2 | 4.6 | 1 | 1 | 1 | 3 | 3.10E+07 | - | - | - | 196 | 21.7 | 8.1 | Protein metabolism | - | Metallopeptidase activity | - |
| CHMP6 | NP_078867.2 | 79643 | charged multivesicular body protein 6 | 6.5 | 1 | 1 | 1 | 3 | 1.13E+07 | - | - | - | 201 | 23.5 | 5.3 | Transport | - | Molecular function unknown | - |
| CHRDL1 | NP_001137455.2 | 91851 | chordin-like protein 1 isoform 4 precursor | 1.6 | 4 | 1 | 1 | 1 | 9.24E+07 | - | - | - | 378 | 43.5 | 8.4 | Cell communication;Signal transduction | - | Molecular function unknown | - |
| CHST14 | NP_569735.1 | 113189 | carbohydrate sulfotransferase 14 | 2.4 | 1 | 1 | 1 | 1 | 7.10E+06 | - | - | - | 376 | 43 | 9.5 | Metabolism;Energy pathways | Integral to membrane | Sulfotransferase activity | Integral to membrane |
| CHTF18 | NP_071375.1 | 63922 | chromosome transmission fidelity protein 18 homolog | 1.5 | 1 | 1 | 1 | 1 | 3.27E+06 | - | - | - | 975 | 107.3 | 7.2 | Regulation of nucleobase, nucleoside, nucleotide and nucleic acid metabolism | Nucleus | DNA binding | Nucleus |
| CHUK | NP_001269.3 | 1147 | inhibitor of nuclear factor kappa-B kinase subunit alpha | 3.4 | 1 | 1 | 1 | 1 | 4.15E+06 | - | - | - | 745 | 84.6 | 6.7 | Cell communication;Signal transduction | Cytoplasm;Plasma membrane;Nucleus;Cytosol | Protein serine/threonine kinase activity | Cytoplasm |
| CIC | NP_055940.3 | 23152 | protein capicua homolog | 1.0 | 1 | 1 | 1 | 1 | 2.64E+07 | - | - | - | 1608 | 163.7 | 8.6 | Regulation of nucleobase, nucleoside, nucleotide and nucleic acid metabolism | Nucleus;Cytoplasm | Transcription factor activity | Nucleus;Cytoplasm |
| CLDN7 | NP_001171952.1 | 1366 | claudin-7 isoform 2 precursor | 11.7 | 2 | 1 | 1 | 3 | 2.78E+08 | - | - | - | 145 | 15.1 | 8.6 | Cell growth and/or maintenance | Plasma membrane | Cell adhesion molecule activity | Plasma membrane |
| CLGN | NP_001124147.1 | 1047 | calmegin precursor | 8.2 | 1 | 2 | 2 | 2 | 1.13E+08 | - | - | - | 610 | 70 | 4.7 | Protein metabolism | Endoplasmic reticulum;Cytoplasm | Chaperone activity | Endoplasmic reticulum |
| CLMN | NP_079010.2 | 79789 | calmin | 2.4 | 1 | 1 | 1 | 1 | 4.01E+07 | - | - | - | 1002 | 111.6 | 4.9 | Biological_process unknown | Integral to membrane;Cytoplasm | Molecular function unknown | Integral to membrane |
| CMC4 | NP_001018024.1 | 100272147 | cx9C motif-containing protein 4 | 25.0 | 1 | 1 | 1 | 1 | 6.17E+07 | - | - | - | 68 | 7.7 | 8.2 | - | - | - | - |
| CMIP | NP_938204.2 | 80790 | C-Maf-inducing protein isoform C-Mip | 3.5 | 1 | 1 | 1 | 1 | 1.62E+07 | - | - | - | 773 | 86.3 | 6.7 | Cell communication;Signal transduction | Cytoplasm | Receptor signaling complex scaffold activity | Cytoplasm |
| CMPK2 | NP_001243407.1 | 129607 | UMP-CMP kinase 2, mitochondrial isoform 3 precursor | 3.8 | 3 | 1 | 1 | 1 | 2.46E+07 | - | - | - | 366 | 39.5 | 7.3 | Biological_process unknown | - | Molecular function unknown | - |
| CMTM6 | NP_060271.1 | 54918 | CKLF-like MARVEL transmembrane domain-containing protein 6 | 5.5 | 1 | 1 | 1 | 1 | 2.04E+08 | - | - | - | 183 | 20.4 | 5.3 | Cell communication;Signal transduction | Plasma membrane | Chemokine activity | Plasma membrane |
| CMTR1 | NP_055865.1 | 23070 | cap-specific mRNA (nucleoside-2'-O-)-methyltransferase 1 | 8.6 | 1 | 4 | 4 | 5 | 8.67E+07 | - | - | - | 835 | 95.3 | 7.1 | Regulation of nucleobase, nucleoside, nucleotide and nucleic acid metabolism | Cytoplasm | RNA methyltransferase activity | Cytoplasm |
| CNNM3 | NP_951060.1 | 26505 | metal transporter CNNM3 isoform 2 precursor | 4.9 | 2 | 1 | 1 | 2 | 1.01E+07 | - | - | - | 659 | 70.6 | 7.1 | Cell communication;Signal transduction | Nucleus | Molecular function unknown | Nucleus |
| CNOT7 | NP_473367.2 | 29883 | CCR4-NOT transcription complex subunit 7 isoform 2 | 8.2 | 2 | 1 | 1 | 1 | 1.34E+07 | - | - | - | 244 | 28.2 | 4.9 | Regulation of nucleobase, nucleoside, nucleotide and nucleic acid metabolism | Nucleus;Cytoplasm | Transcription regulator activity | Nucleus |
| CNPY4 | NP_689968.1 | 245812 | protein canopy homolog 4 precursor | 17.3 | 1 | 2 | 2 | 2 | 9.97E+06 | - | - | - | 248 | 28.3 | 4.6 | Biological_process unknown | - | Molecular function unknown | - |
| COBLL1 | NP_001265389.1 | 22837 | cordon-bleu protein-like 1 isoform 3 | 0.9 | 2 | 1 | 1 | 1 | 1.14E+08 | - | - | - | 1174 | 128.5 | 7.0 | Biological_process unknown | - | Molecular function unknown | - |
| COG2 | NP_031383.1 | 22796 | conserved oligomeric Golgi complex subunit 2 isoform 1 | 13.8 | 2 | 5 | 5 | 5 | 1.21E+07 | - | - | - | 738 | 83.2 | 6.6 | Transport | Cytoplasm;Golgi apparatus | Structural molecule activity | Golgi apparatus |
| COL18A1 | NP_569712.2 | 80781 | collagen alpha-1(XVIII) chain isoform 2 precursor | 2.3 | 2 | 1 | 1 | 1 | 2.09E+07 | - | - | - | 1336 | 135.4 | 6.5 | Cell growth and/or maintenance | Extracellular | Extracellular matrix structural constituent | Extracellular |
| COL24A1 | NP_690850.2 | 255631 | collagen alpha-1(XXIV) chain precursor | 1.2 | 1 | 1 | 1 | 1 | 9.65E+06 | - | - | - | 1714 | 175.4 | 8.3 | Cell growth and/or maintenance | Extracellular | Extracellular matrix structural constituent | Extracellular |
| COL26A1 | NP_597714.2 | 136227 | collagen alpha-1(XXVI) chain isoform 2 precursor | 5.2 | 2 | 1 | 1 | 1 | 0.00E+00 | - | - | - | 439 | 45.1 | 7.3 | Cell growth and/or maintenance | Extracellular;Endoplasmic reticulum;Golgi apparatus | Extracellular matrix structural constituent | Extracellular |
| COL4A1 | NP_001836.2 | 1282 | collagen alpha-1(IV) chain preproprotein | 1.2 | 1 | 1 | 1 | 1 | 5.78E+07 | - | - | - | 1669 | 160.5 | 8.3 | Cell growth and/or maintenance | Extracellular;Extracellular matrix | Extracellular matrix structural constituent | Extracellular |
| COLQ | NP_536800.2 | 8292 | acetylcholinesterase collagenic tail peptide isoform III precursor | 4.3 | 3 | 1 | 1 | 1 | 1.04E+07 | - | - | - | 421 | 44.2 | 8.3 | Signal transduction;Cell communication | Extracellular | Catalytic activity | Extracellular |

| Gene Symbol | RefSeq_Accession | Entrez Gene ID | Description | Coverage (%) | Proteins | Unique Peptides | # Peptides | PSM | Area | SCC-R /SCC-S | SCC-R /SCC-S (Count) | SCC-R /SCC-S (Variability [%]) | # AAs | MW [kDa] | calc. pI | Biological Process | Localization | Molecular Function | Primary Localization |
|---|---|---|---|---|---|---|---|---|---|---|---|---|---|---|---|---|---|---|---|
| COMMD5 | NP_054785.2 | 28991 | COMM domain-containing protein 5 | 14.3 | 2 | 2 | 2 | 4 | 4.20E+07 | - | - | - | 224 | 24.7 | 7.0 | Biological_process unknown | Nucleus;Cytoplasm | Molecular function unknown | Nucleus |
| COMMD8 | NP_060315.1 | 54951 | COMM domain-containing protein 8 | 6.6 | 1 | 1 | 1 | 1 | 2.54E+08 | - | - | - | 183 | 21.1 | 5.4 | Biological_process unknown | - | Molecular function unknown | - |
| COQ3 | NP_059117.3 | 51805 | hexaprenyldihydroxybenzoate methyltransferase, mitochondrial | 4.6 | 1 | 1 | 1 | 1 | 4.89E+07 | - | - | - | 369 | 41 | 7.4 | Metabolism;Energy pathways | Mitochondrion | Methyltransferase activity | Mitochondrion |
| COQ5 | NP_115690.3 | 84274 | 2-methoxy-6-polyprenyl-1,4-benzoquinol methylase, mitochondrial precursor | 4.3 | 1 | 1 | 1 | 1 | 6.42E+06 | - | - | - | 327 | 37.1 | 7.0 | Biological_process unknown | - | Molecular function unknown | - |
| CPA4 | NP_001156918.1 | 51200 | carboxypeptidase A4 isoform 2 preproprotein | 9.5 | 2 | 2 | 2 | 2 | 7.44E+07 | - | - | - | 388 | 43.5 | 8.3 | Protein metabolism | - | Metallopeptidase activity | - |
| CPNE5 | NP_065990.1 | 57699 | copine-5 | 4.1 | 8 | 1 | 2 | 7 | 1.25E+09 | - | - | - | 593 | 65.7 | 6.0 | Transport | Cytoplasm | Transporter activity | Cytoplasm |
| CPNE7 | NP_705900.1 | 27132 | copine-7 isoform a | 7.2 | 9 | 2 | 3 | 6 | 5.24E+08 | - | - | - | 558 | 61.9 | 5.7 | Cell communication;Signal transduction | - | Transporter activity | - |
| CREB1 | NP_004370.1 | 1385 | cyclic AMP-responsive element-binding protein 1 isoform A | 18.0 | 9 | 2 | 4 | 8 | 1.43E+08 | - | - | - | 327 | 35.1 | 5.3 | Regulation of nucleobase, nucleoside, nucleotide and nucleic acid metabolism | Nucleus | Transcription factor activity | Nucleus |
| CREG1 | NP_003842.1 | 8804 | protein CREG1 precursor | 9.6 | 1 | 1 | 1 | 1 | 2.07E+08 | - | - | - | 220 | 24.1 | 7.6 | Cell communication;Signal transduction | Endoplasmic reticulum;Golgi apparatus;Extracellular;Nucleus | Transcription factor activity | Extracellular |
| CROCC | NP_055490.4 | 9696 | rootletin | 1.7 | 1 | 2 | 2 | 7 | 1.77E+08 | - | - | - | 2017 | 228.3 | 5.5 | Cell growth and/or maintenance | Cytoplasm;Endoplasmic reticulum | Structural molecule activity | Cytoplasm |
| CSAD | NP_001231635.1 | 51380 | cysteine sulfinic acid decarboxylase isoform 3 | 8.5 | 3 | 1 | 1 | 1 | 9.35E+06 | - | - | - | 260 | 29.2 | 6.7 | Metabolism;Energy pathways | - | Carboxy-lyase activity | - |
| CSF1 | NP_757350.1 | 1435 | macrophage colony-stimulating factor 1 isoform c precursor | 7.0 | 3 | 1 | 1 | 2 | 3.18E+07 | - | - | - | 256 | 29.1 | 5.4 | Immune response;Cell proliferation;Cell differentiation;Signal transduction;Hemopoiesis | Extracellular | Cytokine activity | Extracellular |
| CST6 | NP_001314.1 | 1474 | cystatin-M precursor | 14.1 | 1 | 1 | 1 | 1 | 0.00E+00 | - | - | - | 149 | 16.5 | 8.1 | Protein metabolism | Extracellular | Protease inhibitor activity | Extracellular |
| CTDSPL | NP_005799.2 | 10217 | CTD small phosphatase-like protein isoform 2 | 5.3 | 2 | 1 | 1 | 1 | 1.88E+07 | - | - | - | 265 | 29.9 | 5.5 | Metabolism;Energy pathways;Regulation of cell growth | Cytoplasm;Nucleus | Catalytic activity | Cytoplasm;Nucleus |
| CTH | NP_001177392.1 | 1491 | cystathionine gamma-lyase isoform 3 | 3.5 | 2 | 1 | 1 | 1 | 1.33E+07 | - | - | - | 373 | 41.2 | 6.9 | Protein metabolism | Cytoplasm | Cysteine-type peptidase activity | Cytoplasm |
| CTSH | NP_004381.2 | 1512 | pro-cathepsin H preproprotein | 11.0 | 1 | 1 | 1 | 1 | 3.55E+07 | - | - | - | 335 | 37.4 | 8.1 | Protein metabolism | Microsome;Nucleus;Mitochondrion;Lysosome | Cysteine-type peptidase activity | Lysosome |
| CYHR1 | NP_612505.1 | 50626 | cysteine and histidine-rich protein 1 isoform 1 precursor | 3.9 | 1 | 1 | 1 | 1 | 1.83E+07 | - | - | - | 362 | 40.7 | 7.2 | Biological_process unknown | Integral to membrane | Molecular function unknown | Integral to membrane |
| CYP2J2 | NP_000766.2 | 1573 | cytochrome P450 2J2 | 2.6 | 1 | 1 | 1 | 1 | 2.28E+06 | - | - | - | 502 | 57.6 | 8.6 | Metabolism;Energy pathways | Endoplasmic reticulum | Catalytic activity | Endoplasmic reticulum |
| D2HGDH | NP_689996.4 | 728294 | D-2-hydroxyglutarate dehydrogenase, mitochondrial isoform 1 precursor | 1.2 | 1 | 1 | 1 | 1 | 1.01E+07 | - | - | - | 521 | 56.4 | 8.0 | - | - | - | - |
| DAPK1 | NP_001275659.1 | 1612 | death-associated protein kinase 1 | 0.5 | 1 | 1 | 1 | 1 | 1.23E+08 | - | - | - | 1430 | 159.9 | 6.8 | Cell communication;Signal transduction | Cytoplasm;Plasma membrane | Protein serine/threonine kinase activity | Cytoplasm |
| DAXX | NP_001241646.1 | 1616 | death domain-associated protein 6 isoform c | 4.7 | 3 | 1 | 1 | 1 | 5.64E+06 | - | - | - | 665 | 73.5 | 4.9 | Cell communication;Signal transduction | Nucleus;Cytoplasm | Receptor signaling complex scaffold activity | Nucleus |
| DBI | NP_001073331.1 | 1622 | acyl-CoA-binding protein isoform 3 | 18.4 | 1 | 1 | 2 | 2 | 1.37E+08 | - | - | - | 87 | 10 | 6.6 | Metabolism;Energy pathways | Extracellular;Cytoplasm;Mitochondrion | Receptor binding | Mitochondrion |
| DCAKD | NP_001122103.1 | 79877 | dephospho-CoA kinase domain-containing protein | 4.8 | 1 | 1 | 1 | 1 | 8.45E+06 | - | - | - | 231 | 26.5 | 9.6 | Biological_process unknown | - | Kinase activity | - |
| DCTD | NP_001912.2 | 1635 | deoxycytidylate deaminase isoform b | 9.0 | 2 | 1 | 1 | 1 | 4.81E+06 | - | - | - | 178 | 20 | 7.6 | Metabolism;Energy pathways | - | Deaminase activity | - |
| DCTN2 | NP_001248342.1 | 10540 | dynactin subunit 2 isoform 3 | 72.1 | 2 | 1 | 20 | 86 | 7.26E+08 | - | - | - | 401 | 44.2 | 5.2 | Cell growth and/or maintenance | Cytoplasm;Zymogen granule;Centrosome;Microtubule;Kinetochore | Motor activity | Cytoplasm |
| DCTN6 | NP_006562.1 | 10671 | dynactin subunit 6 | 9.5 | 1 | 1 | 1 | 1 | 0.00E+00 | - | - | - | 190 | 20.7 | 6.3 | Biological_process unknown | Mitochondrion;Centrosome;Kinetochore;Microtubule | Molecular function unknown | Mitochondrion |
| DDI2 | NP_115717.3 | 84301 | protein DDI1 homolog 2 | 10.8 | 1 | 2 | 2 | 2 | 0.00E+00 | - | - | - | 399 | 44.5 | 5.1 | Protein metabolism | Cytoplasm | Ubiquitin-specific protease activity | Cytoplasm |
| DDN | NP_055901.2 | 23109 | dendrin | 3.5 | 1 | 1 | 1 | 2 | 5.51E+06 | - | - | - | 711 | 75.9 | 10.2 | Biological_process unknown | Cytoplasm | Molecular function unknown | Cytoplasm |
| DDR1 | NP_001945.3 | 780 | epithelial discoidin domain-containing receptor 1 isoform 1 precursor | 5.7 | 6 | 3 | 3 | 3 | 2.83E+07 | - | - | - | 876 | 97.1 | 6.5 | Cell communication;Signal transduction | Plasma membrane | Transmembrane receptor protein tyrosine kinase activity | Plasma membrane |
| DDX11 | NP_689651.1 | 1663 | probable ATP-dependent RNA helicase DDX11 isoform 3 | 3.6 | 1 | 1 | 1 | 1 | 0.00E+00 | - | - | - | 970 | 108.2 | 7.3 | Regulation of nucleobase, nucleoside, nucleotide and nucleic acid metabolism | Nucleus;Nucleolus | RNA binding | Nucleolus |
| DDX26B | NP_872346.3 | 203522 | protein DDX26B | 2.0 | 3 | 1 | 1 | 1 | 7.00E+06 | - | - | - | 861 | 96.6 | 8.7 | Biological_process unknown | - | Molecular function unknown | - |
| DDX28 | NP_060850.2 | 55794 | probable ATP-dependent RNA helicase DDX28 | 2.6 | 1 | 1 | 1 | 1 | 2.80E+06 | - | - | - | 540 | 59.5 | 10.4 | Regulation of nucleobase, nucleoside, nucleotide and nucleic acid metabolism | Nucleus;Mitochondrion | RNA binding | Mitochondrion |
| DDX31 | NP_619526.1 | 64794 | probable ATP-dependent RNA helicase DDX31 isoform 2 | 2.1 | 2 | 1 | 1 | 1 | 0.00E+00 | - | - | - | 585 | 64.1 | 9.9 | Regulation of nucleobase, nucleoside, nucleotide and nucleic acid metabolism | Nucleolus;Nucleus | RNA binding | Nucleolus |
| DDX51 | NP_778236.2 | 317781 | ATP-dependent RNA helicase DDX51 | 3.3 | 1 | 1 | 1 | 1 | 3.93E+08 | - | - | - | 666 | 72.4 | 8.2 | Regulation of nucleobase, nucleoside, nucleotide and nucleic acid metabolism | Nucleolus;Nucleus | Helicase activity | Nucleolus |
| DEF6 | NP_071330.3 | 50619 | differentially expressed in FDCP 6 homolog | 4.8 | 1 | 1 | 1 | 1 | 5.83E+08 | - | - | - | 631 | 73.9 | 6.1 | Cell communication;Signal transduction | Cytoplasm;Plasma membrane | Guanyl-nucleotide exchange factor activity | Cytoplasm |
| DEGS1 | NP_003667.1 | 8560 | sphingolipid delta(4)-desaturase DES1 | 5.6 | 1 | 1 | 1 | 1 | 0.00E+00 | - | - | - | 323 | 37.8 | 7.5 | Energy pathways;Lipid metabolism | Endoplasmic reticulum membrane | Oxidoreductase activity | Endoplasmic reticulum membrane |
| DERL1 | NP_001128143.1 | 79139 | derlin-1 isoform b | 4.8 | 2 | 1 | 1 | 1 | 7.21E+07 | - | - | - | 231 | 26.4 | 9.5 | Transport | Endoplasmic reticulum | Transporter activity | Endoplasmic reticulum |

| Gene Symbol | RefSeq_Accession | Entrez Gene ID | Description | Coverage (%) | Proteins | Unique Peptides | # Peptides | PSM | Area | SCC-R /SCC-S | SCC-R /SCC-S (Count) | SCC-R /SCC-S (Variability [%]) | # AAs | MW [kDa] | calc. pI | Biological Process | Localization | Molecular Function | Primary Localization |
|---|---|---|---|---|---|---|---|---|---|---|---|---|---|---|---|---|---|---|---|
| DGUOK | NP_550440.1 | 1716 | deoxyguanosine kinase, mitochondrial isoform b precursor | 13.2 | 2 | 1 | 1 | 1 | 4.61E+06 | - | - | - | 189 | 21.4 | 9.5 | Metabolism;Energy pathways | Mitochondrion;Cytoplasm | Catalytic activity | Mitochondrion |
| DHRS4L1 | NP_001264793.1 | 728635 | putative dehydrogenase/reductase SDR family member 4-like 2 | 6.7 | 3 | 1 | 1 | 1 | 4.47E+07 | - | - | - | 150 | 16.3 | 7.8 | Biological_process unknown | - | Molecular function unknown | - |
| DIAPH2 | NP_009293.1 | 1730 | protein diaphanous homolog 2 isoform 12C | 7.2 | 2 | 4 | 4 | 5 | 2.33E+07 | - | - | - | 1096 | 124.7 | 6.4 | Cell communication;Signal transduction | Cytoplasm;Endosome;Nucleus | Receptor binding | Cytoplasm |
| DIEXF | NP_055203.4 | 27042 | digestive organ expansion factor homolog | 3.0 | 1 | 1 | 1 | 1 | 6.24E+05 | - | - | - | 756 | 87 | 5.9 | Organogenesis | - | Molecular function unknown | - |
| DKK3 | NP_056965.3 | 27122 | dickkopf-related protein 3 precursor | 6.6 | 1 | 1 | 1 | 1 | 1.36E+07 | - | - | - | 350 | 38.4 | 4.7 | Cell communication;Signal transduction | Extracellular;Cytoplasm | Receptor binding;Lipid binding | Extracellular |
| DLC1 | NP_079043.3 | 10395 | rho GTPase-activating protein 7 isoform 3 | 1.9 | 2 | 1 | 1 | 1 | 6.04E+08 | - | - | - | 463 | 50.9 | 5.2 | Regulation of cell proliferation;Apoptosis;Regulation of cell growth | Cytoplasm | GTPase activator activity | Cytoplasm |
| DLGAP1 | NP_001229691.1 | 9229 | disks large-associated protein 1 isoform 4 | 1.7 | 6 | 1 | 1 | 1 | 3.56E+07 | - | - | - | 661 | 74 | 5.8 | Biological_process unknown | Synapse | Molecular function unknown | Synapse |
| DLGAP4 | NP_001035951.1 | 22839 | disks large-associated protein 4 isoform c | 6.7 | 3 | 1 | 1 | 2 | 1.45E+07 | - | - | - | 285 | 31.6 | 5.9 | Cell communication;Signal transduction | Synapse;Cytosol;Plasma membrane | Molecular function unknown | Cytosol |
| DMP1 | NP_001073380.1 | 1758 | dentin matrix acidic phosphoprotein 1 isoform 2 precursor | 2.2 | 2 | 1 | 1 | 1 | 3.81E+07 | - | - | - | 497 | 54.1 | 4.2 | Cell growth and/or maintenance | Extracellular | Extracellular matrix structural constituent | Extracellular |
| DNAH8 | NP_001193856.1 | 1769 | dynein heavy chain 8, axonemal | 0.3 | 1 | 1 | 1 | 1 | 0.00E+00 | - | - | - | 4707 | 538.3 | 6.1 | Cell growth and/or maintenance | Cytoplasm | Motor activity | Cytoplasm |
| DNAJB14 | NP_001265239.1 | 79982 | dnaJ homolog subfamily B member 14 isoform 2 | 3.5 | 2 | 1 | 1 | 1 | 8.50E+05 | - | - | - | 312 | 35.4 | 8.8 | Protein metabolism;Protein folding | - | Chaperone activity | - |
| DNAJB4 | NP_008965.2 | 11080 | dnaJ homolog subfamily B member 4 | 5.6 | 1 | 1 | 2 | 3 | 4.63E+08 | - | - | - | 337 | 37.8 | 8.5 | Protein metabolism | - | Heat shock protein activity | - |
| DNAJC17 | NP_060633.1 | 55192 | dnaJ homolog subfamily C member 17 | 6.9 | 1 | 1 | 1 | 1 | 8.65E+06 | - | - | - | 304 | 34.7 | 8.5 | Biological_process unknown | Nucleus | Molecular function unknown | Nucleus |
| DNAJC19 | NP_001177162.1 | 131118 | mitochondrial import inner membrane translocase subunit TIM14 isoform 2 | 15.4 | 2 | 1 | 1 | 2 | 3.40E+07 | - | - | - | 91 | 10.1 | 10.1 | Mitochondrial transport | Mitochondrial membrane | Protein translocase activity | Mitochondrial membrane |
| DNAJC5 | NP_079495.1 | 80331 | dnaJ homolog subfamily C member 5 | 24.8 | 1 | 2 | 2 | 5 | 6.36E+07 | - | - | - | 198 | 22.1 | 5.1 | Protein metabolism | Cytoplasmic vesicle;Endoplasmic reticulum;Plasma membrane;Nucleus;Zymogen granule | Chaperone activity | Cytoplasmic vesicle |
| DNM1L | NP_001265392.1 | 10059 | dynamin-1-like protein isoform 4 | 63.2 | 3 | 1 | 30 | 100 | 2.62E+08 | - | - | - | 725 | 80.5 | 6.7 | Mitochondrion organization and biogenesis | Endoplasmic reticulum;Golgi apparatus;Cytoplasm | GTPase activity | Cytoplasm |
| DNMT3A | NP_783329.1 | 1788 | DNA (cytosine-5)-methyltransferase 3A isoform c | 11.5 | 2 | 1 | 1 | 1 | 3.97E+07 | - | - | - | 166 | 16.9 | 4.8 | Regulation of nucleobase, nucleoside, nucleotide and nucleic acid metabolism | Nucleus;Cytoplasm | DNA-methyltransferase activity | Nucleus |
| DPF1 | NP_001128628.1 | 8193 | zinc finger protein neuro-d4 isoform c | 6.6 | 4 | 1 | 1 | 1 | 2.55E+07 | - | - | - | 332 | 37.8 | 7.2 | Regulation of nucleobase, nucleoside, nucleotide and nucleic acid metabolism | Nucleus | DNA binding | Nucleus |
| DPH1 | NP_001374.3 | 1801 | diphthamide biosynthesis protein 1 | 10.2 | 1 | 2 | 2 | 2 | 6.16E+07 | - | - | - | 443 | 48.8 | 8.2 | Protein metabolism | Cytoplasm;Nucleus | Translation regulator activity | Cytoplasm |
| DPH5 | NP_057042.2 | 51611 | diphthine synthase isoform a | 21.4 | 2 | 3 | 3 | 4 | 3.88E+07 | - | - | - | 285 | 31.6 | 5.3 | Biological_process unknown | - | Molecular function unknown | - |
| DPPA4 | NP_060659.3 | 55211 | developmental pluripotency-associated protein 4 | 3.0 | 1 | 1 | 1 | 1 | 0.00E+00 | - | - | - | 304 | 33.5 | 9.5 | Biological_process unknown | Nucleus | Molecular function unknown | Nucleus |
| DSG3 | NP_001935.2 | 1830 | desmoglein-3 preproprotein | 5.2 | 1 | 3 | 3 | 4 | 2.76E+07 | - | - | - | 999 | 107.5 | 5.0 | Cell communication;Signal transduction | Plasma membrane | Cell adhesion molecule activity | Plasma membrane |
| DTWD2 | NP_775937.1 | 285605 | DTW domain-containing protein 2 | 9.1 | 1 | 1 | 1 | 1 | 4.78E+06 | - | - | - | 298 | 33.4 | 8.7 | Biological_process unknown | - | Molecular function unknown | - |
| DUSP12 | NP_009171.1 | 11266 | dual specificity protein phosphatase 12 | 5.6 | 1 | 1 | 1 | 1 | 2.32E+07 | - | - | - | 340 | 37.7 | 6.8 | Cell communication;Signal transduction | Nucleus;Cytoplasm | Protein tyrosine/serine/threonine phosphatase activity | Nucleus |
| DUSP28 | NP_001028747.1 | 285193 | dual specificity phosphatase 28 | 7.4 | 1 | 1 | 1 | 1 | 4.88E+08 | - | - | - | 176 | 18.3 | 8.3 | Biological_process unknown | - | Molecular function unknown | - |
| DUSP9 | NP_001386.1 | 1852 | dual specificity protein phosphatase 9 | 5.7 | 1 | 1 | 1 | 1 | 9.06E+06 | - | - | - | 384 | 41.8 | 6.1 | Cell communication;Signal transduction | Cytoplasm;Nucleus | Protein tyrosine/serine/threonine phosphatase activity | Cytoplasm |
| DYM | NP_060123.3 | 54808 | dymeclin | 0.9 | 1 | 1 | 1 | 1 | 1.40E+07 | - | - | - | 669 | 75.9 | 5.9 | Biological_process unknown | - | Molecular function unknown | - |
| ECT2 | NP_060568.3 | 1894 | protein ECT2 isoform b | 1.5 | 2 | 1 | 1 | 1 | 1.07E+06 | - | - | - | 883 | 100 | 7.5 | Cell communication;Signal transduction | Cytoplasm;Nucleus;Membrane fraction;Chromosome | Guanyl-nucleotide exchange factor activity | Nucleus |
| EDEM3 | NP_079467.3 | 80267 | ER degradation-enhancing alpha-mannosidase-like protein 3 precursor | 2.3 | 1 | 1 | 1 | 1 | 7.09E+06 | - | - | - | 932 | 104.6 | 4.9 | Metabolism;Energy pathways | Endoplasmic reticulum | Hydrolase activity | Endoplasmic reticulum |
| EEF2K | NP_037434.1 | 29904 | eukaryotic elongation factor 2 kinase | 1.7 | 1 | 1 | 1 | 1 | 6.89E+05 | - | - | - | 725 | 82.1 | 5.4 | Protein metabolism | Cytoplasm | Protein serine/threonine kinase activity | Cytoplasm |
| EFEMP1 | NP_001034438.1 | 2202 | EGF-containing fibulin-like extracellular matrix protein 1 precursor | 5.9 | 1 | 1 | 1 | 1 | 4.50E+06 | - | - | - | 493 | 54.6 | 5.1 | Cell growth and/or maintenance | Extracellular | Extracellular matrix structural constituent | Extracellular |
| EHBP1 | NP_001136088.1 | 23301 | EH domain-binding protein 1 isoform 3 | 0.5 | 3 | 1 | 1 | 1 | 3.24E+08 | - | - | - | 1160 | 132.2 | 5.9 | Biological_process unknown | - | Molecular function unknown | - |
| EHD3 | NP_055415.1 | 30845 | EH domain-containing protein 3 | 8.8 | 1 | 1 | 5 | 5 | 1.39E+08 | - | - | - | 535 | 60.8 | 6.6 | Cell communication;Signal transduction | Cytoplasmic vesicle;Nucleus | Cytoskeletal protein binding | Cytoplasmic vesicle |
| EHMT2 | NP_079532.5 | 10919 | histone-lysine N-methyltransferase EHMT2 isoform b | 2.6 | 2 | 1 | 1 | 2 | 0.00E+00 | - | - | - | 1176 | 128.9 | 5.5 | Regulation of nucleobase, nucleoside, nucleotide and nucleic acid metabolism;Protein modification | Nucleus;Cytoplasm | Methyltransferase activity | Nucleus |
| EIF2AK4 | NP_001013725.2 | 440275 | eukaryotic translation initiation factor 2-alpha kinase 4 | 0.9 | 1 | 1 | 1 | 2 | 2.77E+08 | - | - | - | 1649 | 186.8 | 6.3 | Cell communication;Signal transduction | Cytoplasm | Transmembrane receptor protein tyrosine kinase activity | Cytoplasm |
| EIF4EBP2 | NP_004087.1 | 1979 | eukaryotic translation initiation factor 4E-binding protein 2 | 25.8 | 1 | 1 | 1 | 1 | 7.10E+07 | - | - | - | 120 | 12.9 | 6.7 | Protein metabolism | - | Translation regulator activity | - |
| EIF4G1 | NP_001278086.1 | 1981 | eukaryotic translation initiation factor 4 gamma 1 isoform 7 | 40.3 | 6 | 2 | 50 | 195 | 7.94E+08 | - | - | - | 1559 | 171.4 | 5.3 | Protein metabolism | Nucleus;Cytoplasm | Translation regulator activity | Nucleus;Cytoplasm |

| Gene Symbol | RefSeq_Accession | Entrez Gene ID | Description | Coverage (%) | Proteins | Unique Peptides | # Peptides | PSM | Area | SCC-R /SCC-S | SCC-R /SCC-S (Count) | SCC-R /SCC-S (Variability [%]) | # AAs | MW [kDa] | calc. pI | Biological Process | Localization | Molecular Function | Primary Localization |
|---|---|---|---|---|---|---|---|---|---|---|---|---|---|---|---|---|---|---|---|
| EIF5A2 | NP_065123.1 | 56648 | eukaryotic translation initiation factor 5A-2 | 47.1 | 1 | 2 | 11 | 145 | 5.23E+09 | - | - | - | 153 | 16.8 | 5.6 | Protein metabolism | Cytoplasm;Nucleolus | Translation regulator activity | Cytoplasm |
| ELF3 | NP_001107781.1 | 1999 | ETS-related transcription factor Elf-3 | 4.0 | 1 | 1 | 1 | 1 | 1.11E+07 | - | - | - | 371 | 41.4 | 5.8 | Regulation of nucleobase, nucleoside, nucleotide and nucleic acid metabolism | Nucleus | Transcription factor activity | Nucleus |
| ELMO1 | NP_569709.1 | 9844 | engulfment and cell motility protein 1 isoform 2 | 7.3 | 2 | 1 | 1 | 1 | 2.65E+06 | - | - | - | 247 | 28.7 | 6.4 | Cytoskeleton organization and biogenesis;Signal transduction;Cell growth and/or maintenance;Cell migration | Cytoplasm;Plasma membrane | Protein domain specific binding;Motor activity | Cytoplasm |
| ELMO3 | NP_078988.2 | 79767 | engulfment and cell motility protein 3 | 5.4 | 1 | 2 | 2 | 2 | 4.32E+07 | - | - | - | 773 | 87 | 7.7 | Cell growth and/or maintenance | - | Motor activity | - |
| ELMSAN1 | NP_001036783.1 | 91748 | ELM2 and SANT domain-containing protein 1 | 2.0 | 1 | 1 | 1 | 1 | 6.13E+07 | - | - | - | 1045 | 114.9 | 9.2 | Regulation of nucleobase, nucleoside, nucleotide and nucleic acid metabolism | Nucleus | DNA binding | Nucleus |
| ELP2 | NP_001229807.1 | 55250 | elongator complex protein 2 isoform 5 | 1.7 | 6 | 1 | 1 | 2 | 2.10E+07 | - | - | - | 756 | 84.8 | 6.0 | Cell communication;Signal transduction | Nucleus | Receptor signaling complex scaffold activity | Nucleus |
| ELP5 | NP_981960.1 | 23587 | elongator complex protein 5 isoform 1 | 4.1 | 1 | 1 | 1 | 1 | 7.76E+07 | - | - | - | 316 | 34.8 | 5.0 | Biological_process unknown | - | Molecular function unknown | - |
| EMC4 | NP_001273349.1 | 51234 | ER membrane protein complex subunit 4 isoform b | 10.7 | 2 | 1 | 1 | 3 | 3.53E+07 | - | - | - | 149 | 16.7 | 10.1 | Biological_process unknown | Nucleus | Molecular function unknown | Nucleus |
| EMC7 | NP_064539.1 | 56851 | ER membrane protein complex subunit 7 precursor | 16.5 | 1 | 2 | 2 | 2 | 1.26E+07 | - | - | - | 242 | 26.5 | 9.3 | Biological_process unknown | - | Molecular function unknown | - |
| EMILIN2 | NP_114437.2 | 84034 | EMILIN-2 precursor | 1.6 | 1 | 1 | 1 | 2 | 8.38E+08 | - | - | - | 1053 | 115.6 | 6.5 | Cell growth and/or maintenance | Extracellular | Extracellular matrix structural constituent | Extracellular |
| ENPP1 | NP_006199.2 | 5167 | ectonucleotide pyrophosphatase/phosphodiesterase family member 1 | 3.1 | 1 | 1 | 1 | 1 | 2.56E+07 | - | - | - | 925 | 104.9 | 7.1 | Metabolism;Energy pathways | Plasma membrane | ATPase activity | Plasma membrane |
| ENTPD6 | NP_001107561.1 | 955 | ectonucleoside triphosphate diphosphohydrolase 6 isoform 2 | 3.9 | 2 | 1 | 1 | 1 | 0.00E+00 | - | - | - | 467 | 51.1 | 8.9 | Metabolism;Energy pathways | Extracellular;Plasma membrane | Hydrolase activity | Extracellular |
| EPB41L2 | NP_001186317.1 | 2037 | band 4.1-like protein 2 isoform c | 20.9 | 7 | 1 | 14 | 27 | 2.98E+08 | - | - | - | 852 | 95.5 | 5.8 | Cell communication;Signal transduction | Plasma membrane;Nucleus;Cytoplasm;Mitochondrion | Structural constituent of cytoskeleton | Plasma membrane |
| EPHB2 | NP_059145.2 | 2048 | ephrin type-B receptor 2 isoform 1 precursor | 4.2 | 11 | 1 | 2 | 2 | 1.95E+08 | - | - | - | 986 | 109.8 | 5.6 | Cell communication;Signal transduction | Plasma membrane | Transmembrane receptor protein tyrosine kinase activity | Plasma membrane |
| EPM2AIP1 | NP_055620.1 | 9852 | EPM2A-interacting protein 1 | 2.1 | 1 | 1 | 1 | 1 | 4.85E+06 | - | - | - | 607 | 70.3 | 6.1 | Biological_process unknown | Endoplasmic reticulum | Molecular function unknown | Endoplasmic reticulum |
| EPN2 | NP_683723.2 | 22905 | epsin-2 isoform a | 4.6 | 2 | 2 | 2 | 2 | 2.29E+07 | - | - | - | 584 | 62.3 | 7.5 | Cell communication;Signal transduction;Biological_process unknown | Cytoplasmic vesicle;Golgi apparatus | Receptor signaling complex scaffold activity;Molecular function unknown | Cytoplasmic vesicle |
| EPT1 | NP_277040.1 | 85465 | ethanolaminephosphotransferase 1 | 4.5 | 1 | 1 | 1 | 6 | 1.68E+08 | - | - | - | 397 | 45.2 | 6.6 | Biological_process unknown | - | Molecular function unknown | - |
| ERBB2IP | NP_001006600.1 | 55914 | protein LAP2 isoform 7 | 3.2 | 6 | 2 | 2 | 3 | 2.14E+07 | - | - | - | 1302 | 146 | 5.3 | Signal transduction;Cell communication | Plasma membrane;Cytoplasm;Nucleus | Protein binding;Receptor signaling complex scaffold activity | Plasma membrane |
| ERCC5 | NP_000114.2 | 2073 | DNA repair protein complementing XP-G cells | 2.3 | 2 | 1 | 1 | 1 | 2.10E+07 | - | - | - | 1186 | 133.2 | 5.3 | Regulation of nucleobase, nucleoside, nucleotide and nucleic acid metabolism | Nucleus | DNA repair protein | Nucleus |
| ERCC6-PGBD3 | NP_001263988.1 | 101243544 | Cockayne syndrome B-piggyBac fusion protein | 1.8 | 2 | 1 | 1 | 2 | 1.88E+07 | - | - | - | 1061 | 119.4 | 6.6 | - | - | - | - |
| ERF | NP_006485.2 | 2077 | ETS domain-containing transcription factor ERF | 8.2 | 1 | 2 | 2 | 2 | 4.32E+07 | - | - | - | 548 | 58.7 | 7.3 | Cell communication;Signal transduction | Nucleus;Cytoplasm | Transcription factor activity | Nucleus |
| ERGIC2 | NP_057654.2 | 51290 | endoplasmic reticulum-Golgi intermediate compartment protein 2 | 5.8 | 1 | 1 | 1 | 1 | 7.77E+06 | - | - | - | 377 | 42.5 | 6.8 | Biological_process unknown | Nucleus;Cytoplasm | Molecular function unknown | Nucleus |
| ERICH6B | NP_872348.2 | 220081 | glutamate-rich protein 6B | 1.2 | 1 | 1 | 1 | 2 | 2.17E+08 | - | - | - | 696 | 81.6 | 4.8 | Biological_process unknown | - | Molecular function unknown | - |
| ERLIN2 | NP_009106.1 | 11160 | erlin-2 isoform 1 | 21.8 | 2 | 2 | 5 | 12 | 1.53E+08 | - | - | - | 339 | 37.8 | 5.6 | Biological_process unknown | - | Molecular function unknown | - |
| ERRFI1 | NP_061821.1 | 54206 | ERBB receptor feedback inhibitor 1 | 12.6 | 1 | 3 | 3 | 4 | 6.19E+07 | - | - | - | 462 | 50.5 | 8.1 | Cell growth and/or maintenance | Cytoplasm | Structural constituent of cytoskeleton | Cytoplasm |
| ESPNL | NP_919288.2 | 339768 | espin-like protein | 0.7 | 1 | 1 | 1 | 1 | 3.10E+07 | - | - | - | 1005 | 108.1 | 6.5 | Biological_process unknown | - | Molecular function unknown | - |
| ESRP1 | NP_001116299.1 | 54845 | epithelial splicing regulatory protein 1 isoform 5 | 18.5 | 5 | 4 | 5 | 12 | 2.00E+08 | - | - | - | 604 | 67.2 | 6.6 | Biological_process unknown | Nucleus | RNA binding | Nucleus |
| ETV3 | NP_001138784.1 | 2117 | ETS translocation variant 3 isoform 1 | 2.5 | 1 | 1 | 1 | 1 | 3.78E+08 | - | - | - | 512 | 57 | 8.6 | Regulation of nucleobase, nucleoside, nucleotide and nucleic acid metabolism | Nucleus | Transcription factor activity | Nucleus |
| EVA1C | NP_001273485.1 | 59271 | protein eva-1 homolog C isoform 2 precursor | 1.8 | 2 | 1 | 1 | 1 | 9.09E+06 | - | - | - | 438 | 49.1 | 7.1 | Biological_process unknown | Integral to membrane | Molecular function unknown | Integral to membrane |
| EVC | NP_714928.1 | 2121 | ellis-van Creveld syndrome protein | 0.6 | 1 | 1 | 1 | 1 | 5.80E+08 | - | - | - | 992 | 111.9 | 6.7 | Biological_process unknown | - | Molecular function unknown | - |
| EXOC2 | NP_060773.3 | 55770 | exocyst complex component 2 | 3.6 | 1 | 2 | 2 | 3 | 2.41E+05 | - | - | - | 924 | 104 | 6.9 | Transport | Plasma membrane;Nucleus;Cytoplasm | Auxiliary transport protein activity | Plasma membrane |
| F11 | NP_000119.1 | 2160 | coagulation factor XI precursor | 1.0 | 1 | 1 | 1 | 2 | 9.27E+07 | - | - | - | 625 | 70.1 | 8.1 | Protein metabolism | Extracellular;Plasma membrane | Catalytic activity | Plasma membrane |
| FABP5 | NP_001435.1 | 2171 | fatty acid-binding protein, epidermal | 31.9 | 1 | 3 | 3 | 3 | 5.21E+07 | - | - | - | 135 | 15.2 | 7.0 | Transport | Cytoplasm;Endoplasmic reticulum | Transporter activity | Cytoplasm |
| FADS3 | NP_068373.1 | 3995 | fatty acid desaturase 3 | 4.3 | 1 | 1 | 1 | 1 | 1.32E+07 | - | - | - | 445 | 51.1 | 7.8 | Metabolism;Energy pathways | - | Oxidoreductase activity | - |
| FAM115A | NP_001193870.1 | 9747 | protein FAM115A isoform 3 | 4.8 | 3 | 1 | 1 | 1 | 1.34E+06 | - | - | - | 497 | 56.6 | 8.2 | Biological_process unknown | - | Molecular function unknown | - |
| FAM120B | NP_115824.1 | 84498 | constitutive coactivator of peroxisome proliferator-activated receptor gamma isoform c | 2.6 | 3 | 1 | 1 | 1 | 3.09E+07 | - | - | - | 910 | 103.7 | 5.7 | Biological_process unknown | - | Molecular function unknown | - |
| FAM120C | NP_060318.4 | 54954 | constitutive coactivator of PPAR-gamma-like protein 2 isoform 1 | 3.6 | 1 | 2 | 2 | 2 | 1.67E+07 | - | - | - | 1096 | 120.5 | 9.0 | Biological_process unknown | Nucleus | Molecular function unknown | Nucleus |

| Gene Symbol | RefSeq_Accession | Entrez Gene ID | Description | Coverage (%) | Proteins | Unique Peptides | # Peptides | PSM | Area | SCC-R /SCC-S | SCC-R /SCC-S (Count) | SCC-R /SCC-S (Variability [%]) | # AAs | MW [kDa] | calc. pI | Biological Process | Localization | Molecular Function | Primary Localization |
|---|---|---|---|---|---|---|---|---|---|---|---|---|---|---|---|---|---|---|---|
| FAM122B | NP_001160071.1 | 159090 | protein FAM122B isoform 2 | 17.4 | 3 | 1 | 1 | 1 | 0.00E+00 | - | - | - | 247 | 26.9 | 6.4 | Biological_process unknown | - | Molecular function unknown | - |
| FAM160A2 | NP_001092264.1 | 84067 | FTS and Hook-interacting protein isoform 2 | 2.4 | 1 | 1 | 1 | 1 | 2.72E+08 | - | - | - | 972 | 105.5 | 6.8 | Biological_process unknown | - | Molecular function unknown | - |
| FAM160B1 | NP_001128523.1 | 57700 | protein FAM160B1 isoform b | 1.9 | 2 | 1 | 1 | 1 | 3.73E+07 | - | - | - | 738 | 83.7 | 5.3 | Biological_process unknown | - | Molecular function unknown | - |
| FAM167A | NP_444509.2 | 83648 | protein FAM167A | 12.6 | 1 | 1 | 1 | 1 | 1.05E+07 | - | - | - | 214 | 24.2 | 6.3 | Biological_process unknown | - | Molecular function unknown | - |
| FAM168B | NP_001009993.2 | 130074 | myelin-associated neurite-outgrowth inhibitor | 9.2 | 1 | 1 | 1 | 2 | 1.26E+07 | - | - | - | 195 | 20.3 | 9.2 | Biological_process unknown | - | Molecular function unknown | - |
| FAM177A1 | NP_001072987.1 | 283635 | protein FAM177A1 isoform 2 | 17.4 | 1 | 1 | 1 | 2 | 7.10E+09 | - | - | - | 213 | 23.7 | 4.5 | Biological_process unknown | - | Molecular function unknown | - |
| FAM193B | NP_001177875.1 | 54540 | protein FAM193B | 0.9 | 1 | 1 | 1 | 1 | 3.88E+08 | - | - | - | 822 | 87.5 | 7.2 | Regulation of nucleobase, nucleoside, nucleotide and nucleic acid metabolism | Nucleus | DNA binding | Nucleus |
| FAM207A | NP_478070.1 | 85395 | protein FAM207A | 15.7 | 1 | 1 | 1 | 1 | 1.41E+06 | - | - | - | 230 | 25.4 | 11.1 | Biological_process unknown | Nucleolus | Molecular function unknown | Nucleolus |
| FAM20B | NP_055679.1 | 9917 | glycosaminoglycan xylosylkinase | 4.7 | 1 | 1 | 1 | 1 | 9.38E+06 | - | - | - | 409 | 46.4 | 6.9 | Biological_process unknown | Extracellular | Molecular function unknown | Extracellular |
| FAM64A | NP_061886.2 | 54478 | protein FAM64A isoform 1 | 8.8 | 2 | 1 | 1 | 1 | 1.91E+07 | - | - | - | 238 | 26.2 | 9.9 | Biological_process unknown | - | Molecular function unknown | - |
| FAM83B | NP_001010872.1 | 222584 | protein FAM83B | 5.8 | 1 | 3 | 3 | 3 | 3.19E+07 | - | - | - | 1011 | 114.7 | 9.0 | Biological_process unknown | - | Molecular function unknown | - |
| FAM98C | NP_777565.3 | 147965 | protein FAM98C | 4.3 | 1 | 1 | 1 | 1 | 1.38E+07 | - | - | - | 349 | 37.3 | 7.2 | Biological_process unknown | - | Molecular function unknown | - |
| FANCI | NP_060663.2 | 55215 | Fanconi anemia group I protein isoform 2 | 3.2 | 2 | 2 | 2 | 2 | 3.05E+07 | - | - | - | 1268 | 142.5 | 6.8 | Biological_process unknown | Nucleus;Mitochondrion | DNA repair protein | Nucleus |
| FARP2 | NP_001269913.1 | 9855 | FERM, RhoGEF and pleckstrin domain-containing protein 2 isoform c | 5.2 | 3 | 1 | 1 | 1 | 0.00E+00 | - | - | - | 638 | 72.2 | 8.9 | Cell communication;Signal transduction | Cytoskeleton | Structural constituent of cytoskeleton | Cytoskeleton |
| FASTKD2 | NP_055744.2 | 22868 | FAST kinase domain-containing protein 2 | 1.8 | 1 | 1 | 1 | 1 | 1.44E+07 | - | - | - | 710 | 81.4 | 8.1 | Biological_process unknown | - | Molecular function unknown | - |
| FASTKD5 | NP_068598.1 | 60493 | FAST kinase domain-containing protein 5 | 2.0 | 1 | 1 | 1 | 1 | 9.63E+07 | - | - | - | 764 | 86.5 | 8.1 | Biological_process unknown | - | Molecular function unknown | - |
| FAT2 | NP_001438.1 | 2196 | protocadherin Fat 2 precursor | 3.2 | 1 | 7 | 7 | 7 | 1.33E+08 | - | - | - | 4349 | 479 | 5.2 | Cell communication;Signal transduction | - | Cell adhesion molecule activity | - |
| FBRSL1 | NP_001136113.1 | 57666 | fibrosin-1-like protein | 1.5 | 1 | 1 | 1 | 1 | 5.56E+08 | - | - | - | 1045 | 110.8 | 9.7 | Biological_process unknown | - | Molecular function unknown | - |
| FBXO2 | NP_036300.2 | 26232 | F-box only protein 2 | 13.5 | 1 | 2 | 2 | 2 | 4.10E+07 | - | - | - | 296 | 33.3 | 4.4 | Protein metabolism | Cytoplasm | Ubiquitin-specific protease activity | Cytoplasm |
| FBXO21 | NP_055817.1 | 23014 | F-box only protein 21 isoform 2 | 7.9 | 2 | 2 | 2 | 2 | 4.22E+07 | - | - | - | 621 | 71.4 | 6.0 | Protein metabolism | - | Ubiquitin-specific protease activity | - |
| FCHSD1 | NP_258260.1 | 89848 | FCH and double SH3 domains protein 1 | 1.9 | 1 | 1 | 1 | 1 | 9.52E+05 | - | - | - | 690 | 76.9 | 5.3 | Cell communication;Signal transduction | - | Receptor signaling complex scaffold activity | - |
| FGA | NP_068657.1 | 2243 | fibrinogen alpha chain isoform alpha preproprotein | 1.4 | 2 | 2 | 2 | 6 | 5.65E+07 | - | - | - | 644 | 69.7 | 8.1 | Protein metabolism | Extracellular | Protein binding | Extracellular |
| FGD6 | NP_060821.3 | 55785 | FYVE, RhoGEF and PH domain-containing protein 6 | 1.0 | 1 | 1 | 1 | 1 | 1.02E+09 | - | - | - | 1430 | 160.7 | 7.0 | Biological_process unknown | - | Molecular function unknown | - |
| FGFBP1 | NP_005121.1 | 9982 | fibroblast growth factor-binding protein 1 precursor | 27.4 | 1 | 3 | 3 | 6 | 2.16E+08 | - | - | - | 234 | 26.2 | 9.1 | Cell communication;Signal transduction | Extracellular;Cytoplasm | Chaperone activity | Extracellular |
| FHOD1 | NP_037373.2 | 29109 | FH1/FH2 domain-containing protein 1 | 2.5 | 1 | 2 | 2 | 2 | 4.56E+06 | - | - | - | 1164 | 126.5 | 6.4 | Cell growth and/or maintenance | Cytoplasm;Perinuclear region;Actin cytoskeleton | Cytoskeletal protein binding | Cytoplasm |
| FKBP9 | NP_001271272.1 | 11328 | peptidyl-prolyl cis-trans isomerase FKBP9 isoform 3 | 4.1 | 3 | 1 | 1 | 2 | 3.33E+07 | - | - | - | 338 | 37 | 4.8 | Protein metabolism | Endoplasmic reticulum | Chaperone activity | Endoplasmic reticulum |
| FKRP | NP_001034974.1 | 79147 | fukutin-related protein | 2.6 | 1 | 1 | 1 | 1 | 2.00E+06 | - | - | - | 495 | 54.5 | 7.4 | Biological_process unknown | Golgi apparatus | Molecular function unknown | Golgi apparatus |
| FLT4 | NP_002011.2 | 2324 | vascular endothelial growth factor receptor 3 isoform 2 precursor | 1.1 | 2 | 1 | 1 | 1 | 1.23E+08 | - | - | - | 1298 | 145.5 | 6.3 | Cell communication;Signal transduction | Plasma membrane;Cytoplasm | Transmembrane receptor protein tyrosine kinase activity | Plasma membrane |
| FLVCR1 | NP_054772.1 | 28982 | feline leukemia virus subgroup C receptor-related protein 1 | 2.3 | 1 | 1 | 1 | 1 | 1.18E+07 | - | - | - | 555 | 59.8 | 6.0 | Transport | Plasma membrane | Transporter activity | Plasma membrane |
| FMNL1 | NP_005883.2 | 752 | formin-like protein 1 | 5.3 | 1 | 3 | 4 | 5 | 2.59E+08 | - | - | - | 1100 | 121.8 | 5.7 | Cell growth and/or maintenance | Cytoplasm;Plasma membrane | Cell adhesion molecule activity | Cytoplasm |
| FMNL3 | NP_944489.2 | 91010 | formin-like protein 3 isoform 2 | 1.3 | 3 | 1 | 1 | 1 | 0.00E+00 | - | - | - | 976 | 111.2 | 6.9 | Biological_process unknown | - | Molecular function unknown | - |
| FN1 | NP_997639.1 | 2335 | fibronectin isoform 6 preproprotein | 1.0 | 5 | 1 | 1 | 1 | 8.32E+06 | - | - | - | 2176 | 239.5 | 5.9 | Cell growth and/or maintenance | Extracellular;Endoplasmic reticulum;Nucleus | Extracellular matrix structural constituent | Extracellular;Nucleus |
| FN3KRP | NP_078895.2 | 79672 | ketosamine-3-kinase | 3.9 | 1 | 1 | 1 | 1 | 2.42E+07 | - | - | - | 309 | 34.4 | 7.3 | Metabolism;Energy pathways | - | Catalytic activity | - |
| FOCAD | NP_060264.4 | 54914 | focadhesin | 0.3 | 1 | 1 | 1 | 1 | 1.62E+08 | - | - | - | 1801 | 199.9 | 6.6 | Biological_process unknown | - | Molecular function unknown | - |
| FOXF1 | NP_001442.2 | 2294 | forkhead box protein F1 | 9.2 | 1 | 1 | 1 | 1 | 4.10E+07 | - | - | - | 379 | 40.1 | 9.1 | Regulation of nucleobase, nucleoside, nucleotide and nucleic acid metabolism | Nucleus | Transcription factor activity | Nucleus |
| FOXP3 | NP_001107849.1 | 50943 | forkhead box protein P3 isoform b | 7.8 | 1 | 1 | 1 | 1 | 0.00E+00 | - | - | - | 396 | 43.4 | 9.3 | Regulation of nucleobase, nucleoside, nucleotide and nucleic acid metabolism;Cell proliferation | Nucleus | Transcription regulator activity | Nucleus |
| FRG1 | NP_004468.1 | 2483 | protein FRG1 | 4.3 | 1 | 1 | 1 | 1 | 1.19E+07 | - | - | - | 258 | 29.2 | 9.0 | Biological_process unknown | Nucleolus | Molecular function unknown | Nucleolus |

| Gene Symbol | RefSeq_Accession | Entrez Gene ID | Description | Coverage (%) | Proteins | Unique Peptides | # Peptides | PSM | Area | SCC-R /SCC-S | SCC-R /SCC-S (Count) | SCC-R /SCC-S (Variability [%]) | # AAs | MW [kDa] | calc. pI | Biological Process | Localization | Molecular Function | Primary Localization |
|---|---|---|---|---|---|---|---|---|---|---|---|---|---|---|---|---|---|---|---|
| FRMD6 | NP_001035946.1 | 122786 | FERM domain-containing protein 6 isoform 1 | 2.9 | 2 | 1 | 1 | 2 | 2.66E+07 | - | - | - | 614 | 70.9 | 7.5 | Biological_process unknown | Plasma membrane | Molecular function unknown | Plasma membrane |
| FRMD8 | NP_114110.1 | 83786 | FERM domain-containing protein 8 | 3.5 | 1 | 1 | 1 | 1 | 2.17E+06 | - | - | - | 464 | 51.2 | 6.2 | Cell growth and/or maintenance | - | Cytoskeletal protein binding | - |
| FTSJ1 | NP_803188.1 | 24140 | putative tRNA (cytidine(32)/guanosine(34)-2'-O)-methyltransferase isoform b | 4.9 | 2 | 1 | 1 | 1 | 4.83E+07 | - | - | - | 327 | 35.8 | 5.9 | Regulation of nucleobase, nucleoside, nucleotide and nucleic acid metabolism | Nucleolus | Methyltransferase activity | Nucleolus |
| FUNDC2 | NP_076423.2 | 65991 | FUN14 domain-containing protein 2 | 4.8 | 1 | 1 | 1 | 1 | 8.53E+07 | - | - | - | 189 | 20.7 | 9.7 | Biological_process unknown | Nucleus | Molecular function unknown | Nucleus |
| FUT7 | NP_004470.1 | 2529 | alpha-(1,3)-fucosyltransferase 7 | 1.8 | 1 | 1 | 1 | 1 | 9.45E+08 | - | - | - | 342 | 39.2 | 10.0 | Metabolism;Energy pathways | Golgi apparatus | Fucosyltransferase activity | Golgi apparatus |
| FUT8 | NP_004471.4 | 2530 | alpha-(1,6)-fucosyltransferase isoform b | 6.3 | 2 | 1 | 1 | 1 | 1.54E+07 | - | - | - | 412 | 47.2 | 7.1 | Metabolism;Energy pathways | Golgi apparatus | Fucosyltransferase activity | Golgi apparatus |
| FXR1 | NP_001013456.1 | 8087 | fragile X mental retardation syndrome-related protein 1 isoform b | 37.3 | 1 | 1 | 14 | 51 | 3.18E+08 | - | - | - | 539 | 60.8 | 6.4 | Regulation of nucleobase, nucleoside, nucleotide and nucleic acid metabolism;Regulation of translation | Cytoplasm;Nucleus;Nucleolus | RNA binding | Nucleolus |
| FXYD3 | NP_001129483.1 | 5349 | FXYD domain-containing ion transport regulator 3 isoform 1 precursor | 21.8 | 3 | 1 | 1 | 2 | 2.59E+08 | - | - | - | 87 | 9.3 | 7.3 | Transport | Plasma membrane;Cytoplasm | Ion channel activity | Plasma membrane |
| GAPDHS | NP_055179.1 | 26330 | glyceraldehyde-3-phosphate dehydrogenase, testis-specific | 3.4 | 1 | 1 | 2 | 7 | 1.56E+09 | - | - | - | 408 | 44.5 | 8.2 | Metabolism;Energy pathways | Cell projection | Catalytic activity | Cell projection |
| GBGT1 | NP_001275501.1 | 26301 | globoside alpha-1,3-N-acetylgalactosaminyltransferase 1 isoform 4 | 7.0 | 3 | 1 | 1 | 1 | 4.56E+08 | - | - | - | 300 | 34.7 | 8.5 | Immune response | Integral to membrane | Transferase activity | Integral to membrane |
| GCFC2 | NP_001188263.1 | 6936 | GC-rich sequence DNA-binding factor 2 isoform 2 | 2.8 | 2 | 1 | 1 | 1 | 1.48E+07 | - | - | - | 612 | 71.3 | 5.9 | Regulation of nucleobase, nucleoside, nucleotide and nucleic acid metabolism | Nucleus;Cytoplasm | Transcription factor activity | Nucleus |
| GDPD3 | NP_077283.2 | 79153 | glycerophosphodiester phosphodiesterase domain-containing protein 3 | 6.6 | 1 | 1 | 1 | 1 | 1.84E+06 | - | - | - | 318 | 36.6 | 8.0 | Biological_process unknown | - | Molecular function unknown | - |
| GGH | NP_003869.1 | 8836 | gamma-glutamyl hydrolase precursor | 7.6 | 1 | 1 | 1 | 2 | 7.60E+07 | - | - | - | 318 | 35.9 | 7.1 | Metabolism;Energy pathways | Lysosome;Extracellular;Plasma membrane | Hydrolase activity | Lysosome |
| GIGYF1 | NP_072096.2 | 64599 | PERQ amino acid-rich with GYF domain-containing protein 1 | 5.1 | 1 | 2 | 2 | 2 | 3.47E+07 | - | - | - | 1035 | 114.5 | 5.4 | Cell communication;Signal transduction | - | Molecular function unknown | - |
| GLB1 | NP_001073279.1 | 2720 | beta-galactosidase isoform b | 17.3 | 2 | 1 | 6 | 12 | 1.97E+08 | - | - | - | 647 | 72.7 | 6.3 | Metabolism;Energy pathways | Lysosome | Catalytic activity | Lysosome |
| GLB1 | NP_001129074.1 | 2720 | beta-galactosidase isoform c preproprotein | 23.4 | 1 | 1 | 6 | 11 | 2.37E+08 | - | - | - | 546 | 60.5 | 7.0 | Metabolism;Energy pathways | Lysosome | Catalytic activity | Lysosome |
| GLI1 | NP_001153517.1 | 2735 | zinc finger protein GLI1 isoform 2 | 1.6 | 3 | 1 | 1 | 1 | 5.23E+07 | - | - | - | 978 | 104.6 | 7.1 | Cell communication;Signal transduction | Cytoplasm;Nucleus | Transcription factor activity | Cytoplasm |
| GLMN | NP_444504.1 | 11146 | glomulin | 2.5 | 1 | 1 | 1 | 1 | 2.27E+06 | - | - | - | 594 | 68.2 | 5.3 | Cell communication;Signal transduction;Regulation of cell proliferation | Cytoplasm | Receptor binding;Protein domain specific binding | Cytoplasm |
| GLRA2 | NP_001112357.1 | 2742 | glycine receptor subunit alpha-2 isoform A precursor | 4.7 | 2 | 1 | 1 | 1 | 0.00E+00 | - | - | - | 452 | 52 | 8.9 | Transport | Plasma membrane | Extracellular ligand-gated ion channel activity | Plasma membrane |
| GLT8D1 | NP_001010983.1 | 55830 | glycosyltransferase 8 domain-containing protein 1 | 5.9 | 1 | 1 | 1 | 1 | 8.11E+07 | - | - | - | 371 | 41.9 | 9.4 | Metabolism;Energy pathways;Biological_process unknown | - | Molecular function unknown | - |
| GM2A | NP_000396.2 | 2760 | ganglioside GM2 activator isoform 1 precursor | 10.4 | 1 | 1 | 1 | 1 | 3.34E+06 | - | - | - | 193 | 20.8 | 5.3 | Metabolism;Energy pathways | Lysosome;Extracellular | Transporter activity | Lysosome |
| GMCL1 | NP_848526.1 | 64395 | germ cell-less protein-like 1 | 4.1 | 1 | 1 | 1 | 2 | 8.24E+07 | - | - | - | 515 | 58.6 | 7.4 | Regulation of nucleobase, nucleoside, nucleotide and nucleic acid metabolism | Nucleus | Transcription regulator activity | Nucleus |
| GMIP | NP_001275927.1 | 51291 | GEM-interacting protein isoform 2 | 1.7 | 3 | 1 | 1 | 1 | 4.25E+07 | - | - | - | 941 | 103.6 | 5.9 | Cell communication;Signal transduction | Plasma membrane | GTPase activator activity | Plasma membrane |
| GNAL | NP_001248373.1 | 2774 | guanine nucleotide-binding protein G(olf) subunit alpha isoform 3 | 4.0 | 1 | 1 | 1 | 1 | 7.88E+07 | - | - | - | 174 | 20.5 | 6.3 | Cell communication;Signal transduction | Secretory granule;Golgi apparatus;Plasma membrane | GTPase activity | Secretory granule |
| GNAQ | NP_002063.2 | 2776 | guanine nucleotide-binding protein G(q) subunit alpha | 13.1 | 1 | 2 | 3 | 3 | 8.65E+06 | - | - | - | 359 | 42.1 | 5.7 | Cell communication;Signal transduction | Plasma membrane;Cytoplasm;Golgi apparatus | GTPase activity | Plasma membrane |
| GNE | NP_001177313.1 | 10020 | bifunctional UDP-N-acetylglucosamine 2-epimerase/N-acetylmannosamine kinase isoform 5 | 5.1 | 5 | 1 | 1 | 1 | 1.09E+07 | - | - | - | 612 | 66.7 | 6.8 | Metabolism;Energy pathways | Cytoplasm;Cytosol | Racemase and epimerase activity | Cytoplasm |
| GNL3L | NP_001171748.1 | 54552 | guanine nucleotide-binding protein-like 3-like protein | 5.0 | 1 | 2 | 2 | 2 | 4.43E+07 | - | - | - | 582 | 65.5 | 8.4 | Biological_process unknown | - | Molecular function unknown | - |
| GOLGA3 | NP_001166028.1 | 2802 | Golgin subfamily A member 3 isoform 2 | 13.8 | 1 | 1 | 10 | 15 | 6.70E+07 | - | - | - | 1134 | 126.1 | 5.3 | Cell growth and/or maintenance | Golgi apparatus;Cytoplasm;Nucleus | Structural molecule activity | Golgi apparatus;Nucleus |
| GOLIM4 | NP_055313.1 | 27333 | Golgi integral membrane protein 4 | 8.9 | 1 | 3 | 3 | 3 | 3.45E+07 | - | - | - | 696 | 81.8 | 4.8 | Cell communication;Signal transduction | Golgi apparatus;Endoplasmic reticulum;Nucleus;Cytoplasm | Molecular function unknown | Golgi apparatus |
| GPAA1 | NP_003792.1 | 8733 | glycosylphosphatidylinositol anchor attachment 1 protein | 2.1 | 1 | 1 | 1 | 1 | 7.99E+07 | - | - | - | 621 | 67.6 | 8.1 | Protein metabolism | Endoplasmic reticulum;Mitochondrion;Cytoplasm | Cytoskeletal anchoring activity | Endoplasmic reticulum |
| GPALPP1 | NP_061029.2 | 55425 | GPALPP motifs-containing protein 1 | 3.5 | 1 | 1 | 1 | 1 | 5.29E+06 | - | - | - | 340 | 38.1 | 5.4 | Biological_process unknown | - | Molecular function unknown | - |
| GPC1 | NP_002072.2 | 2817 | glypican-1 precursor | 5.6 | 1 | 2 | 2 | 2 | 1.25E+08 | - | - | - | 558 | 61.6 | 7.3 | Cell communication;Signal transduction | Plasma membrane;Extracellular | Receptor activity | Plasma membrane |
| GPR124 | NP_116166.9 | 25960 | G-protein coupled receptor 124 precursor | 2.2 | 1 | 1 | 1 | 1 | 1.94E+08 | - | - | - | 1338 | 142.6 | 8.4 | Cell communication;Signal transduction | Plasma membrane | G-protein coupled receptor activity | Plasma membrane |
| GPR179 | NP_001004334.3 | 440435 | probable G-protein coupled receptor 179 precursor | 1.2 | 1 | 1 | 1 | 1 | 5.21E+09 | - | - | - | 2367 | 257.2 | 5.7 | Cell communication;Signal transduction | Integral to membrane | Molecular function unknown | Integral to membrane |
| GPR56 | NP_958933.1 | 9289 | G-protein coupled receptor 56 isoform b precursor | 5.0 | 5 | 2 | 2 | 2 | 1.11E+07 | - | - | - | 687 | 77 | 8.4 | Signal transduction | Plasma membrane | G-protein coupled receptor activity | Plasma membrane |
| GPR89A | NP_001091082.2 | 653519 | Golgi pH regulator A isoform 2 | 6.3 | 2 | 1 | 1 | 2 | 0.00E+00 | - | - | - | 430 | 50 | 9.4 | Signal transduction | - | G-protein coupled receptor activity | - |
| GPRIN1 | NP_443131.2 | 114787 | G protein-regulated inducer of neurite outgrowth 1 | 4.7 | 1 | 2 | 2 | 2 | 6.82E+07 | - | - | - | 1008 | 102.3 | 8.1 | Biological_process unknown | - | Molecular function unknown | - |

| Gene Symbol | RefSeq_Accession | Entrez Gene ID | Description | Coverage (%) | Proteins | Unique Peptides | # Peptides | PSM | Area | SCC-R /SCC-S | SCC-R /SCC-S (Count) | SCC-R /SCC-S (Variability [%]) | # AAs | MW [kDa] | calc. pI | Biological Process | Localization | Molecular Function | Primary Localization |
|---|---|---|---|---|---|---|---|---|---|---|---|---|---|---|---|---|---|---|---|
| GPX1 | NP_000572.2 | 2876 | glutathione peroxidase 1 isoform 1 | 8.9 | 1 | 1 | 1 | 3 | 6.06E+07 | - | - | - | 203 | 22.1 | 6.6 | Anti-apoptosis | Cytoplasm;Mitochondrion;Cytosol | Peroxidase activity | Cytosol |
| GRB7 | NP_001229372.1 | 2886 | growth factor receptor-bound protein 7 isoform a | 2.8 | 2 | 1 | 1 | 2 | 5.26E+07 | - | - | - | 532 | 59.6 | 8.5 | Cell migration | Plasma membrane;Cytoplasm | Binding | Plasma membrane |
| GRHL2 | NP_079191.2 | 79977 | grainyhead-like protein 2 homolog | 7.0 | 2 | 2 | 2 | 2 | 3.67E+06 | - | - | - | 625 | 71.1 | 6.4 | Regulation of nucleobase, nucleoside, nucleotide and nucleic acid metabolism | Nucleus | Transcription factor activity | Nucleus |
| GSX1 | NP_663632.1 | 219409 | GS homeobox 1 | 9.9 | 1 | 1 | 1 | 6 | 1.35E+08 | - | - | - | 264 | 27.9 | 8.9 | Regulation of nucleobase, nucleoside, nucleotide and nucleic acid metabolism | Nucleus | Transcription factor activity | Nucleus |
| GTF2E2 | NP_002086.1 | 2961 | transcription initiation factor IIE subunit beta | 6.5 | 1 | 1 | 1 | 1 | 7.93E+06 | - | - | - | 291 | 33 | 9.7 | Regulation of nucleobase, nucleoside, nucleotide and nucleic acid metabolism | Nucleus;Kinetochore | Transcription factor activity | Nucleus |
| GTPBP3 | NP_001122327.1 | 84705 | tRNA modification GTPase GTPBP3, mitochondrial isoform III | 4.7 | 4 | 1 | 1 | 2 | 7.88E+07 | - | - | - | 471 | 49.8 | 6.8 | Regulation of nucleobase, nucleoside, nucleotide and nucleic acid metabolism | Mitochondrion | GTPase activity | Mitochondrion |
| GUCY2D | NP_000171.1 | 3000 | retinal guanylyl cyclase 1 | 1.9 | 1 | 1 | 1 | 1 | 3.24E+06 | - | - | - | 1103 | 120 | 7.4 | Cell communication;Signal transduction | Plasma membrane | Guanylate cyclase activity | Plasma membrane |
| H2AFY2 | NP_061119.1 | 55506 | core histone macro-H2A.2 | 33.1 | 1 | 7 | 9 | 24 | 1.48E+08 | - | - | - | 372 | 40 | 9.7 | Regulation of nucleobase, nucleoside, nucleotide and nucleic acid metabolism | Nucleus | DNA binding | Nucleus |
| HAUS4 | NP_001159742.1 | 54930 | HAUS augmin-like complex subunit 4 isoform 2 | 5.0 | 2 | 1 | 1 | 1 | 0.00E+00 | - | - | - | 318 | 37.2 | 6.3 | Biological_process unknown | Centrosome;Microtubule | Molecular function unknown | - |
| HBD | NP_000510.1 | 3045 | hemoglobin subunit delta | 36.1 | 2 | 1 | 6 | 33 | 5.04E+08 | - | - | - | 147 | 16 | 8.1 | Transport | - | Transporter activity | - |
| HBG1 | NP_000550.2 | 3047 | hemoglobin subunit gamma-1 | 17.7 | 3 | 1 | 2 | 9 | 4.12E+08 | - | - | - | 147 | 16.1 | 7.2 | Transport | - | Transporter activity | - |
| HCAR2 | NP_808219.1 | 338442 | hydroxycarboxylic acid receptor 2 | 3.9 | 2 | 1 | 1 | 1 | 1.98E+07 | - | - | - | 363 | 41.8 | 9.1 | Cell communication;Signal transduction | Plasma membrane | G-protein coupled receptor activity | Plasma membrane |
| HDAC4 | NP_006028.2 | 9759 | histone deacetylase 4 | 2.6 | 1 | 1 | 1 | 1 | 2.36E+07 | - | - | - | 1084 | 119 | 7.0 | Regulation of nucleobase, nucleoside, nucleotide and nucleic acid metabolism | Cytoplasm;Nucleus | Transcription regulator activity | Cytoplasm |
| HDAC9 | NP_848510.1 | 9734 | histone deacetylase 9 isoform 4 | 0.7 | 2 | 1 | 1 | 7 | 6.48E+08 | - | - | - | 1066 | 117.1 | 6.6 | Regulation of gene expression, epigenetic | Nucleus;Cytoplasm | Transcription regulator activity | Nucleus |
| HDDC3 | NP_940929.1 | 374659 | guanosine-3',5'-bis(diphosphate) 3'-pyrophosphohydrolase MESH1 isoform 2 | 12.1 | 2 | 1 | 1 | 1 | 6.59E+06 | - | - | - | 140 | 15.7 | 6.5 | Metabolism;Energy pathways | - | Catalytic activity | - |
| HDGFRP3 | NP_057157.1 | 50810 | hepatoma-derived growth factor-related protein 3 | 15.8 | 1 | 1 | 1 | 2 | 6.00E+07 | - | - | - | 203 | 22.6 | 8.0 | Cell growth and/or maintenance | Nucleus | Growth factor activity | Nucleus |
| HEATR5A | NP_056288.2 | 25938 | HEAT repeat-containing protein 5A | 0.5 | 1 | 1 | 1 | 1 | 7.56E+06 | - | - | - | 2046 | 222.6 | 6.6 | Biological_process unknown | - | Molecular function unknown | - |
| HERC1 | NP_003913.3 | 8925 | probable E3 ubiquitin-protein ligase HERC1 | 0.5 | 1 | 1 | 1 | 1 | 8.96E+05 | - | - | - | 4861 | 531.9 | 6.0 | Transport | Cytoplasm;Perinuclear region | Auxiliary transport protein activity | Cytoplasm |
| HERC5 | NP_057407.2 | 51191 | E3 ISG15--protein ligase HERC5 | 9.9 | 1 | 7 | 7 | 9 | 2.06E+08 | - | - | - | 1024 | 116.8 | 7.7 | Cell communication;Signal transduction | Cytoplasm;Nucleus | Ligase activity | Cytoplasm |
| HERC6 | NP_060382.3 | 55008 | probable E3 ubiquitin-protein ligase HERC6 isoform 1 | 2.6 | 1 | 1 | 1 | 1 | 2.31E+07 | - | - | - | 1022 | 115.1 | 8.0 | Protein metabolism | Cytoplasm | Ubiquitin-specific protease activity | Cytoplasm |
| HIGD1C | NP_001103089.1 | 613227 | HIG1 domain family member 1C | 34.0 | 1 | 1 | 1 | 1 | 3.32E+08 | - | - | - | 97 | 11.1 | 9.7 | Biological_process unknown | - | Molecular function unknown | - |
| HIP1 | NP_001230127.1 | 3092 | huntingtin-interacting protein 1 isoform 2 | 7.3 | 2 | 4 | 4 | 6 | 3.55E+07 | - | - | - | 986 | 110.6 | 5.3 | Cell death | Integral to membrane;Golgi apparatus | Structural molecule activity | Integral to membrane |
| HJURP | NP_001269892.1 | 55355 | Holliday junction recognition protein isoform c | 2.1 | 3 | 1 | 1 | 1 | 0.00E+00 | - | - | - | 663 | 74.3 | 9.6 | Biological_process unknown | - | Molecular function unknown | - |
| HMCES | NP_064572.2 | 56941 | embryonic stem cell-specific 5-hydroxymethylcytosine-binding protein | 3.4 | 1 | 1 | 1 | 2 | 2.54E+06 | - | - | - | 354 | 40.5 | 8.2 | Biological_process unknown | - | Molecular function unknown | - |
| HMOX1 | NP_002124.1 | 3162 | heme oxygenase 1 | 20.5 | 1 | 3 | 3 | 3 | 6.19E+06 | - | - | - | 288 | 32.8 | 8.3 | Metabolism;Energy pathways | Endoplasmic reticulum;Microsome;Cytosol | Catalytic activity | Endoplasmic reticulum;Microsome |
| HMP19 | NP_057064.1 | 51617 | neuron-specific protein family member 2 | 3.5 | 1 | 1 | 1 | 5 | 5.06E+08 | - | - | - | 171 | 19.1 | 9.4 | Biological_process unknown | Golgi apparatus | Molecular function unknown | Golgi apparatus |
| HN1 | NP_001002033.1 | 51155 | hematological and neurological expressed 1 protein isoform 3 | 69.4 | 1 | 1 | 4 | 19 | 6.79E+08 | - | - | - | 108 | 11 | 4.8 | Cell communication;Signal transduction | Nucleus;Cytoplasm | Molecular function unknown | Nucleus;Cytoplasm |
| HNF1B | NP_001159395.1 | 6928 | hepatocyte nuclear factor 1-beta isoform 2 | 3.6 | 2 | 1 | 1 | 1 | 0.00E+00 | - | - | - | 531 | 58.3 | 8.2 | Regulation of nucleobase, nucleoside, nucleotide and nucleic acid metabolism | Nucleus | Transcription factor activity | Nucleus |
| HNRNPLL | NP_001136122.1 | 92906 | heterogeneous nuclear ribonucleoprotein L-like isoform 2 | 14.2 | 2 | 4 | 4 | 7 | 4.01E+07 | - | - | - | 537 | 59.6 | 7.7 | Regulation of nucleobase, nucleoside, nucleotide and nucleic acid metabolism | Nucleolus | RNA binding | Nucleolus |
| HOXC6 | NP_710160.1 | 3223 | homeobox protein Hox-C6 isoform 2 | 15.7 | 2 | 1 | 1 | 1 | 0.00E+00 | - | - | - | 153 | 17.8 | 9.7 | Regulation of nucleobase, nucleoside, nucleotide and nucleic acid metabolism;Regulation of gene expression, epigenetic | Nucleus | Transcription factor activity | Nucleus |
| HPS6 | NP_079023.2 | 79803 | Hermansky-Pudlak syndrome 6 protein | 2.1 | 1 | 1 | 1 | 1 | 0.00E+00 | - | - | - | 775 | 82.9 | 6.3 | Biological_process unknown | - | Molecular function unknown | - |
| HS1BP3 | NP_071905.3 | 64342 | HCLS1-binding protein 3 | 1.8 | 1 | 1 | 1 | 1 | 7.71E+06 | - | - | - | 392 | 42.8 | 5.0 | Biological_process unknown | - | Molecular function unknown | - |
| HS2ST1 | NP_001127964.1 | 9653 | heparan sulfate 2-O-sulfotransferase 1 isoform 2 | 7.0 | 2 | 1 | 1 | 1 | 2.11E+07 | - | - | - | 229 | 26.7 | 8.7 | Metabolism;Energy pathways | - | Sulfotransferase activity | - |
| HSD17B8 | NP_055049.1 | 7923 | estradiol 17-beta-dehydrogenase 8 | 5.0 | 1 | 1 | 1 | 1 | 1.02E+07 | - | - | - | 261 | 27 | 6.5 | Metabolism;Energy pathways | - | Catalytic activity | - |
| HTT | NP_002102.4 | 3064 | huntingtin | 1.2 | 1 | 2 | 2 | 7 | 1.34E+07 | - | - | - | 3144 | 347.6 | 6.2 | Regulation of nucleobase, nucleoside, nucleotide and nucleic acid metabolism | Plasma membrane;Golgi apparatus;Cytoplasm;Perinuclear region | DNA binding | - |
| HUS1B | NP_683762.2 | 135458 | checkpoint protein HUS1B | 10.4 | 1 | 1 | 1 | 1 | 0.00E+00 | - | - | - | 278 | 31 | 9.3 | Cell communication;Signal transduction | - | Protein binding | - |
| IBA57 | NP_001010867.1 | 200205 | putative transferase CAF17, mitochondrial precursor | 6.7 | 1 | 1 | 1 | 1 | 9.69E+06 | - | - | - | 356 | 38.1 | 9.8 | Biological_process unknown | - | Molecular function unknown | - |

Supplementary Table S4. List of all proteins identified in SCC-S and SCC-R cells using SequestHT and Mascot search algorithms and their gene ontology annotation

| Gene Symbol | RefSeq_Accession | Entrez Gene ID | Description | Coverage (%) | Proteins | Unique Peptides | # Peptides | PSM | Area | SCC-R /SCC-S | SCC-R /SCC-S (Count) | SCC-R /SCC-S (Variability [%]) | # AAs | MW [kDa] | calc. pI | Biological Process | Localization | Molecular Function | Primary Localization |
|---|---|---|---|---|---|---|---|---|---|---|---|---|---|---|---|---|---|---|---|
| **ICOSLG** | NP_001269981.1 | 23308 | **ICOS ligand isoform d** | 6.5 | 1 | 1 | 1 | 1 | 1.11E+08 | **-** | - | - | 217 | 23.7 | 5.3 | Immune response | Plasma membrane | Receptor binding | Plasma membrane |
| **ID1** | NP_851998.1 | 3397 | **DNA-binding protein inhibitor ID-1 isoform b** | 12.1 | 2 | 1 | 1 | 2 | 2.29E+06 | **-** | - | - | 149 | 15.6 | 7.8 | Regulation of immune response;Regulation of nucleobase, nucleoside, nucleotide and nucleic acid metabolism;Regulation of gene expression, | Centrosome;Cytoplasm;Nucleus | Transcription regulator activity | Nucleus |
| **IFI44L** | NP_006811.2 | 10964 | **interferon-induced protein 44-like** | 1.3 | 1 | 1 | 1 | 1 | 3.73E+07 | **-** | - | - | 452 | 51.3 | 6.8 | Biological_process unknown | - | Molecular function unknown | - |
| **IFIT3** | NP_001276687.1 | 3437 | **interferon-induced protein with tetratricopeptide repeats 3 isoform b** | 78.5 | 1 | 2 | 29 | 85 | 2.20E+09 | **-** | - | - | 438 | 49.8 | 5.2 | Immune response | Cytoplasm | Molecular function unknown | Cytoplasm |
| **IFIT3** | NP_001026853.1 | 3437 | **interferon-induced protein with tetratricopeptide repeats 3 isoform a** | 75.3 | 1 | 3 | 30 | 91 | 2.20E+09 | **-** | - | - | 490 | 55.9 | 5.2 | Immune response | Cytoplasm | Molecular function unknown | Cytoplasm |
| **IFITM1** | NP_003632.3 | 8519 | **interferon-induced transmembrane protein 1** | 29.6 | 2 | 2 | 3 | 8 | 9.12E+08 | **-** | - | - | 125 | 13.9 | 7.9 | Cell communication;Signal transduction | Plasma membrane | Molecular function unknown | Plasma membrane |
| **IFITM3** | NP_066362.2 | 10410 | **interferon-induced transmembrane protein 3** | 48.9 | 2 | 3 | 4 | 15 | 4.81E+08 | **-** | - | - | 133 | 14.6 | 7.0 | Immune response | Plasma membrane | Molecular function unknown | Plasma membrane |
| **IFNGR1** | NP_000407.1 | 3459 | **interferon gamma receptor 1 precursor** | 4.9 | 1 | 1 | 1 | 1 | 3.76E+07 | **-** | - | - | 489 | 54.4 | 4.9 | Immune response | Plasma membrane | Transmembrane receptor activity | Plasma membrane |
| **IGFBP4** | NP_001543.2 | 3487 | **insulin-like growth factor-binding protein 4 precursor** | 14.0 | 1 | 1 | 1 | 1 | 2.29E+08 | **-** | - | - | 258 | 27.9 | 7.2 | Cell communication;Signal transduction | Extracellular | Cell adhesion molecule activity | Extracellular |
| **IGSF5** | NP_001073913.1 | 150084 | **immunoglobulin superfamily member 5** | 9.8 | 1 | 1 | 1 | 3 | 0.00E+00 | **-** | - | - | 407 | 44.6 | 7.8 | Cell adhesion;Cell-cell adhesion | Cell junction | Cell adhesion molecule activity | Cell junction |
| **IGSF9B** | NP_001264214.1 | 22997 | **protein turtle homolog B precursor** | 0.4 | 1 | 1 | 1 | 1 | 0.00E+00 | **-** | - | - | 1437 | 156.8 | 7.0 | Cell communication;Signal transduction | - | Molecular function unknown | - |
| **IL12RB1** | NP_714912.1 | 3594 | **interleukin-12 receptor subunit beta-1 isoform 2 precursor** | 1.6 | 4 | 1 | 1 | 3 | 8.54E+08 | **-** | - | - | 381 | 42.3 | 7.1 | Signal transduction | Plasma membrane | Transmembrane receptor activity | Plasma membrane |
| **IL1B** | NP_000567.1 | 3553 | **interleukin-1 beta proprotein** | 8.6 | 1 | 1 | 1 | 1 | 1.43E+08 | **-** | - | - | 269 | 30.7 | 4.8 | Immune response | Extracellular;Plasma membrane;Cytoplasm;Nucleus | Cytokine activity | Extracellular |
| **IL1RAP** | NP_001161400.1 | 3556 | **interleukin-1 receptor accessory protein isoform 1 precursor** | 1.2 | 1 | 1 | 1 | 2 | 3.79E+07 | **-** | - | - | 570 | 65.4 | 8.1 | Immune response | Plasma membrane;Soluble fraction | Receptor activity | Plasma membrane;Soluble fraction |
| **IL22RA1** | NP_067081.2 | 58985 | **interleukin-22 receptor subunit alpha-1 precursor** | 1.6 | 1 | 1 | 1 | 1 | 0.00E+00 | **-** | - | - | 574 | 63 | 6.2 | Cell communication;Signal transduction | Plasma membrane | Transmembrane receptor activity | Plasma membrane |
| **ILF3** | NP_060090.2 | 3609 | **interleukin enhancer-binding factor 3 isoform d** | 62.6 | 2 | 2 | 48 | 273 | 1.53E+09 | **-** | - | - | 898 | 95.7 | 8.8 | Regulation of nucleobase, nucleoside, nucleotide and nucleic acid metabolism;Regulation of gene expression, epigenetic | Nucleus;Cytoplasm;Ribosome;Nucleolus;Mitochondrion | Transcription factor activity;Transcription regulator activity | Nucleus |
| **ILF3** | NP_036350.2 | 3609 | **interleukin enhancer-binding factor 3 isoform a** | 63.3 | 3 | 2 | 48 | 274 | 1.53E+09 | **-** | - | - | 894 | 95.3 | 8.8 | Regulation of nucleobase, nucleoside, nucleotide and nucleic acid metabolism;Regulation of gene expression, epigenetic | Nucleus;Cytoplasm;Ribosome;Nucleolus;Mitochondrion | Transcription factor activity;Transcription regulator activity | Nucleus |
| **INADL** | NP_795352.2 | 10207 | **inaD-like protein** | 0.3 | 1 | 1 | 1 | 1 | 1.57E+08 | **-** | - | - | 1801 | 196.3 | 4.9 | Cell growth and/or maintenance | Plasma membrane;Actin cytoskeleton | Cell adhesion molecule activity | Plasma membrane |
| **ING5** | NP_115705.2 | 84289 | **inhibitor of growth protein 5** | 9.2 | 1 | 1 | 1 | 1 | 0.00E+00 | **-** | - | - | 240 | 27.7 | 7.6 | Cell growth and/or maintenance | Nucleus | Transcription regulator activity | Nucleus |
| **INPP5K** | NP_001129114.1 | 51763 | **inositol polyphosphate 5-phosphatase K isoform 2** | 6.7 | 2 | 2 | 2 | 2 | 1.33E+06 | **-** | - | - | 372 | 42.8 | 6.5 | Regulation of signal transduction | Plasma membrane;Golgi apparatus;Endoplasmic reticulum;Cytoplasm;Perinuclear region | Lipid phosphatase activity | Golgi apparatus;Endoplasmic reticulum |
| **INTS12** | NP_065128.2 | 57117 | **integrator complex subunit 12** | 2.8 | 1 | 1 | 1 | 1 | 6.54E+06 | **-** | - | - | 462 | 48.8 | 9.7 | Regulation of nucleobase, nucleoside, nucleotide and nucleic acid metabolism | Nucleus | Transcription regulator activity | Nucleus |
| **IPO13** | NP_055467.3 | 9670 | **importin-13** | 1.5 | 1 | 1 | 1 | 1 | 8.39E+06 | **-** | - | - | 963 | 108.1 | 5.3 | Cell communication;Signal transduction | Cytoplasm;Nucleus | Ligand-dependent nuclear receptor activity | Cytoplasm |
| **IQCB1** | NP_001018865.2 | 9657 | **IQ calmodulin-binding motif-containing protein 1 isoform c** | 1.5 | 2 | 1 | 1 | 1 | 1.33E+08 | **-** | - | - | 465 | 53.7 | 8.5 | Biological_process unknown | - | Molecular function unknown | - |
| **IRAK2** | NP_001561.3 | 3656 | **interleukin-1 receptor-associated kinase-like 2** | 2.1 | 1 | 1 | 1 | 1 | 5.35E+07 | **-** | - | - | 625 | 69.4 | 5.7 | Cell communication;Signal transduction | Cytoplasm | Protein serine/threonine kinase activity | Cytoplasm |
| **IRF2BP1** | NP_056464.1 | 26145 | **interferon regulatory factor 2-binding protein 1** | 6.2 | 1 | 1 | 1 | 1 | 1.02E+07 | **-** | - | - | 584 | 61.6 | 8.2 | Regulation of nucleobase, nucleoside, nucleotide and nucleic acid metabolism | Nucleus;Cytoplasm | Transcription regulator activity | Nucleus |
| **IRF2BPL** | NP_078772.1 | 64207 | **interferon regulatory factor 2-binding protein-like** | 2.5 | 1 | 1 | 1 | 1 | 1.12E+07 | **-** | - | - | 796 | 82.6 | 8.2 | Biological_process unknown | Nucleus;Cytoplasm | Molecular function unknown | Nucleus |
| **IRF6** | NP_006138.1 | 3664 | **interferon regulatory factor 6 isoform 1** | 19.7 | 2 | 5 | 5 | 8 | 9.70E+07 | **-** | - | - | 467 | 53.1 | 5.3 | Regulation of nucleobase, nucleoside, nucleotide and nucleic acid metabolism | Nucleus | Transcription factor activity | Nucleus |
| **IRF9** | NP_006075.3 | 10379 | **interferon regulatory factor 9** | 7.1 | 1 | 1 | 1 | 2 | 4.37E+07 | **-** | - | - | 393 | 43.7 | 5.8 | Regulation of nucleobase, nucleoside, nucleotide and nucleic acid metabolism | Cytoplasm;Nucleus | Transcription factor activity | Nucleus |
| **IRS2** | NP_003740.2 | 8660 | **insulin receptor substrate 2** | 1.9 | 1 | 1 | 1 | 1 | 1.65E+07 | **-** | - | - | 1338 | 137.2 | 8.7 | Cell communication;Signal transduction | Cytoplasm;Nucleus | Receptor signaling complex scaffold activity | Cytoplasm |
| **ISCA2** | NP_919255.2 | 122961 | **iron-sulfur cluster assembly 2 homolog, mitochondrial isoform 1 precursor** | 13.0 | 1 | 1 | 1 | 1 | 2.18E+07 | **-** | - | - | 154 | 16.5 | 5.3 | Biological_process unknown | - | Molecular function unknown | - |
| **ISG20** | NP_002192.2 | 3669 | **interferon-stimulated gene 20 kDa protein** | 13.3 | 1 | 2 | 2 | 2 | 1.61E+07 | **-** | - | - | 181 | 20.4 | 8.9 | Regulation of nucleobase, nucleoside, nucleotide and nucleic acid metabolism | Nucleus | RNA binding | Nucleus |
| **ISOC2** | NP_001129674.1 | 79763 | **isochorismatase domain-containing protein 2, mitochondrial isoform 3** | 19.3 | 3 | 1 | 1 | 1 | 3.91E+06 | **-** | - | - | 135 | 14.8 | 9.0 | Metabolism;Energy pathways | - | Molecular function unknown | - |
| **IST1** | NP_001257906.1 | 9798 | **IST1 homolog isoform d** | 8.4 | 4 | 2 | 2 | 2 | 0.00E+00 | **-** | - | - | 335 | 36.6 | 5.4 | Biological_process unknown | - | Molecular function unknown | - |
| **ITFG2** | NP_060933.3 | 55846 | **integrin-alpha FG-GAP repeat-containing protein 2** | 5.6 | 1 | 1 | 1 | 1 | 4.34E+06 | **-** | - | - | 447 | 49.3 | 5.2 | Cell communication;Signal transduction | - | Cell adhesion molecule activity | - |
| **ITGAV** | NP_001138472.1 | 3685 | **integrin alpha-V isoform 3 precursor** | 10.4 | 1 | 1 | 5 | 10 | 6.22E+07 | **-** | - | - | 1012 | 112.2 | 5.9 | Cell communication;Signal transduction;Cell adhesion | Plasma membrane;Cell surface;Nucleus;Cytoplasm | Receptor activity | Plasma membrane |
| **ITGB5** | NP_002204.2 | 3693 | **integrin beta-5 precursor** | 7.4 | 1 | 2 | 2 | 2 | 6.58E+07 | **-** | - | - | 799 | 88 | 6.1 | Cell communication;Signal transduction | Plasma membrane;Cytoplasm;Endosome | Cell adhesion molecule activity | Plasma membrane |
| **ITM2B** | NP_068839.1 | 9445 | **integral membrane protein 2B** | 10.9 | 1 | 2 | 2 | 2 | 1.08E+07 | **-** | - | - | 266 | 30.3 | 5.1 | Cell growth and/or maintenance | Plasma membrane | Molecular function unknown | Plasma membrane |

Jain *et al.*, 2019. MAP2K1 is a potential therapeutic target in erlotinib resistant head and neck squamous cell carcinoma
Supplementary Table S4. List of all proteins identified in SCC-S and SCC-R cells using SequestHT and Mascot search algorithms and their gene ontology annotation

| Gene Symbol | RefSeq_Accession | Entrez Gene ID | Description | Coverage (%) | Proteins | Unique Peptides | # Peptides | PSM | Area | SCC-R /SCC-S | SCC-R /SCC-S (Count) | SCC-R /SCC-S (Variability [%]) | # AAs | MW [kDa] | calc. pI | Biological Process | Localization | Molecular Function | Primary Localization |
|---|---|---|---|---|---|---|---|---|---|---|---|---|---|---|---|---|---|---|---|
| ITPK1 | NP_001136065.1 | 3705 | inositol-tetrakisphosphate 1-kinase isoform a | 6.0 | 2 | 2 | 2 | 2 | 7.39E+07 | - | - | - | 414 | 45.6 | 6.2 | Cell communication;Signal transduction | - | Catalytic activity | - |
| JUNB | NP_002220.1 | 3726 | transcription factor jun-B | 7.2 | 1 | 1 | 1 | 1 | 1.21E+07 | - | - | - | 347 | 35.9 | 9.2 | Regulation of nucleobase, nucleoside, nucleotide and nucleic acid metabolism | Nucleus | Transcription factor activity | Nucleus |
| JUND | NP_001273897.1 | 3727 | transcription factor jun-D isoform deltaJunD | 6.6 | 2 | 1 | 1 | 1 | 1.07E+08 | - | - | - | 304 | 31.2 | 8.4 | Regulation of nucleobase, nucleoside, nucleotide and nucleic acid metabolism | Nucleus | Transcription factor activity | Nucleus |
| KANK1 | NP_694856.1 | 23189 | KN motif and ankyrin repeat domain-containing protein 1 isoform b | 1.5 | 2 | 1 | 1 | 1 | 4.36E+07 | - | - | - | 1194 | 129.7 | 5.1 | Biological_process unknown | Cytoplasm | Molecular function unknown | Cytoplasm |
| KANK4 | NP_859063.3 | 163782 | KN motif and ankyrin repeat domain-containing protein 4 | 8.7 | 1 | 5 | 5 | 6 | 6.30E+07 | - | - | - | 995 | 107.3 | 5.3 | Biological_process unknown | - | Molecular function unknown | - |
| KATNA1 | NP_008975.1 | 11104 | katanin p60 ATPase-containing subunit A1 isoform 1 | 2.9 | 1 | 1 | 1 | 1 | 1.15E+07 | - | - | - | 491 | 55.9 | 6.9 | Cell growth and/or maintenance | Centrosome | ATPase activity | Centrosome |
| KCNAB2 | NP_001186792.1 | 8514 | voltage-gated potassium channel subunit beta-2 isoform 4 | 31.0 | 4 | 4 | 4 | 6 | 7.02E+07 | - | - | - | 300 | 33.6 | 8.4 | Transport | Cytoplasm | Voltage-gated ion channel activity | Cytoplasm |
| KCNAB3 | NP_004723.2 | 9196 | voltage-gated potassium channel subunit beta-3 | 1.7 | 1 | 1 | 1 | 1 | 1.70E+06 | - | - | - | 404 | 43.6 | 8.6 | Transport | Cytoplasm | Voltage-gated ion channel activity | Cytoplasm |
| KCTD4 | NP_940686.2 | 386618 | BTB/POZ domain-containing protein KCTD4 | 3.1 | 1 | 1 | 1 | 7 | 2.46E+09 | - | - | - | 259 | 29.9 | 7.1 | Biological_process unknown | - | Molecular function unknown | - |
| KCTD5 | NP_061865.1 | 54442 | BTB/POZ domain-containing protein KCTD5 | 5.1 | 1 | 1 | 1 | 2 | 7.99E+06 | - | - | - | 234 | 26.1 | 6.2 | Transport | Chromosome | Ion channel activity | - |
| KDM6A | NP_001278347.1 | 7403 | lysine-specific demethylase 6A isoform 5 | 3.0 | 5 | 1 | 1 | 1 | 0.00E+00 | - | - | - | 1322 | 145.1 | 7.4 | Biological_process unknown | Nucleus | Molecular function unknown | Nucleus |
| KDM7A | NP_085150.1 | 80853 | lysine-specific demethylase 7A | 1.0 | 1 | 1 | 1 | 1 | 1.67E+07 | - | - | - | 941 | 106.5 | 8.1 | Biological_process unknown | - | Molecular function unknown | - |
| KDSR | NP_002026.1 | 2531 | 3-ketodihydrosphingosine reductase precursor | 4.8 | 1 | 1 | 1 | 1 | 5.83E+07 | - | - | - | 332 | 36.2 | 7.1 | Regulation of nucleobase, nucleoside, nucleotide and nucleic acid metabolism | Extracellular | Oxidoreductase activity | Extracellular |
| KIAA1217 | NP_001091970.1 | 56243 | sickle tail protein homolog isoform 2 | 1.7 | 4 | 1 | 1 | 1 | 0.00E+00 | - | - | - | 1264 | 137.8 | 9.2 | Biological_process unknown | - | Molecular function unknown | - |
| KIAA1468 | NP_065905.2 | 57614 | lisH domain and HEAT repeat-containing protein KIAA1468 | 1.7 | 1 | 1 | 1 | 1 | 3.16E+06 | - | - | - | 1216 | 134.5 | 5.5 | Biological_process unknown | Cytoplasm | Molecular function unknown | Cytoplasm |
| KIAA2013 | NP_612355.1 | 90231 | uncharacterized protein KIAA2013 precursor | 7.4 | 2 | 3 | 3 | 5 | 4.99E+07 | - | - | - | 634 | 69.1 | 8.2 | Biological_process unknown | Integral to membrane | Molecular function unknown | Integral to membrane |
| KIF13A | NP_001099038.1 | 63971 | kinesin-like protein KIF13A isoform d | 1.6 | 4 | 1 | 2 | 3 | 2.15E+07 | - | - | - | 1749 | 195.9 | 5.7 | Cell growth and/or maintenance | Golgi apparatus;Plasma membrane | Motor activity | Golgi apparatus |
| KIF17 | NP_001274141.1 | 57576 | kinesin-like protein KIF17 isoform c | 2.5 | 3 | 2 | 2 | 2 | 4.97E+08 | - | - | - | 929 | 104 | 5.5 | Cell growth and/or maintenance | Cytoplasm;Nucleus;Cell projection;Cytoskeleton | Motor activity | Cytoplasm |
| KIF20A | NP_005724.1 | 10112 | kinesin-like protein KIF20A | 2.1 | 1 | 1 | 1 | 1 | 4.52E+07 | - | - | - | 890 | 100.2 | 6.9 | Cell growth and/or maintenance | Golgi apparatus;Microtubule;Nucleus | Motor activity | Golgi apparatus |
| KIF27 | NP_001258856.1 | 55582 | kinesin-like protein KIF27 isoform B | 0.9 | 2 | 1 | 1 | 2 | 2.88E+07 | - | - | - | 1335 | 152.2 | 6.8 | Cell growth and/or maintenance | - | Motor activity | - |
| KLF5 | NP_001721.2 | 688 | Krueppel-like factor 5 isoform 1 | 2.4 | 1 | 1 | 1 | 1 | 3.18E+07 | - | - | - | 457 | 50.8 | 8.6 | Regulation of nucleobase, nucleoside, nucleotide and nucleic acid metabolism | Nucleus | Transcription factor activity | Nucleus |
| KLHL21 | NP_055666.2 | 9903 | kelch-like protein 21 | 3.9 | 1 | 1 | 1 | 1 | 1.07E+06 | - | - | - | 597 | 66.6 | 5.4 | Biological_process unknown | - | Molecular function unknown | - |
| KLHL28 | NP_060128.2 | 54813 | kelch-like protein 28 | 4.2 | 1 | 1 | 1 | 1 | 1.30E+06 | - | - | - | 571 | 64.1 | 6.1 | Biological_process unknown | - | Molecular function unknown | - |
| KLK14 | NP_071329.2 | 43847 | kallikrein-14 preproprotein | 10.1 | 1 | 1 | 1 | 1 | 9.27E+08 | - | - | - | 267 | 29.1 | 9.2 | Protein metabolism | Extracellular | Serine-type peptidase activity | Extracellular |
| KNOP1 | NP_001013009.2 | 400506 | lysine-rich nucleolar protein 1 | 6.8 | 1 | 2 | 2 | 2 | 2.20E+06 | - | - | - | 458 | 51.6 | 9.9 | Biological_process unknown | Nucleus | Molecular function unknown | Nucleus |
| KRAS | NP_004976.2 | 3845 | GTPase KRas isoform b | 33.5 | 3 | 1 | 4 | 7 | 1.63E+08 | - | - | - | 188 | 21.4 | 8.1 | Signal transduction | Mitochondrion;Plasma membrane | GTPase activity | Plasma membrane |
| KRIT1 | NP_001013424.1 | 889 | krev interaction trapped protein 1 isoform 2 | 0.9 | 2 | 1 | 1 | 2 | 2.60E+08 | - | - | - | 688 | 78.6 | 8.8 | Signal transduction | Tubulin;Cytoplasm | Molecular function unknown | Tubulin |
| KRT1 | NP_006112.3 | 3848 | keratin, type II cytoskeletal 1 | 5.0 | 1 | 1 | 4 | 15 | 3.52E+08 | - | - | - | 644 | 66 | 8.1 | Cell growth and/or maintenance | Plasma membrane;Cytoskeleton | Structural molecule activity | Plasma membrane |
| KRT15 | NP_002266.2 | 3866 | keratin, type I cytoskeletal 15 | 55.0 | 5 | 11 | 27 | 174 | 8.33E+09 | - | - | - | 456 | 49.2 | 4.8 | Cell growth and/or maintenance | Cytoplasm | Structural constituent of cytoskeleton | Cytoplasm |
| KRT16 | NP_005548.2 | 3868 | keratin, type I cytoskeletal 16 | 35.1 | 9 | 2 | 19 | 173 | 8.64E+09 | - | - | - | 473 | 51.2 | 5.1 | Cell growth and/or maintenance | Cytoplasm | Structural constituent of cytoskeleton | Cytoplasm |
| KRT2 | NP_000414.2 | 3849 | keratin, type II cytoskeletal 2 epidermal | 9.2 | 4 | 1 | 8 | 116 | 6.37E+09 | - | - | - | 639 | 65.4 | 8.0 | Cell growth and/or maintenance | Cytoplasm;Cytoskeleton | Cytoskeletal protein binding | Cytoplasm |
| KRT36 | NP_003762.1 | 8689 | keratin, type I cuticular Ha6 | 3.9 | 3 | 1 | 2 | 12 | 1.77E+09 | - | - | - | 467 | 52.2 | 4.9 | Cell growth and/or maintenance | - | Structural molecule activity | - |
| KRT4 | NP_002263.3 | 3851 | keratin, type II cytoskeletal 4 | 18.7 | 1 | 6 | 10 | 32 | 3.33E+09 | - | - | - | 520 | 56.1 | 6.6 | Cell growth and/or maintenance | Cytoplasm | Structural constituent of cytoskeleton | Cytoplasm |
| KRT6A | NP_005545.1 | 3853 | keratin, type II cytoskeletal 6A | 46.6 | 8 | 10 | 28 | 212 | 1.31E+10 | - | - | - | 564 | 60 | 8.0 | Cell growth and/or maintenance | Cytoplasm | Structural molecule activity | Cytoplasm |
| KRT71 | NP_258259.1 | 112802 | keratin, type II cytoskeletal 71 | 5.9 | 2 | 1 | 3 | 17 | 2.05E+09 | - | - | - | 523 | 57.3 | 6.6 | Cell growth and/or maintenance | Cytoskeleton | Structural molecule activity | Cytoskeleton |
| KRT80 | NP_001074961.1 | 144501 | keratin, type II cytoskeletal 80 isoform K80.1 | 4.7 | 2 | 1 | 3 | 18 | 9.91E+07 | - | - | - | 422 | 47.2 | 5.3 | Cell growth and/or maintenance | - | Structural constituent of cytoskeleton | - |
| KSR1 | NP_055053.1 | 8844 | kinase suppressor of Ras 1 | 1.1 | 1 | 1 | 1 | 1 | 3.65E+08 | - | - | - | 762 | 84.6 | 8.6 | Regulation of signal transduction;Anti-apoptosis;Signal transduction | Plasma membrane;Cell surface;Cytoplasm | Kinase activity;Protein kinase activity | Cytoplasm |

| Gene Symbol | RefSeq_Accession | Entrez Gene ID | Description | Coverage (%) | Proteins | Unique Peptides | # Peptides | PSM | Area | SCC-R /SCC-S | SCC-R /SCC-S (Count) | SCC-R /SCC-S (Variability [%]) | # AAs | MW [kDa] | calc. pI | Biological Process | Localization | Molecular Function | Primary Localization |
|---|---|---|---|---|---|---|---|---|---|---|---|---|---|---|---|---|---|---|---|
| KYNU | NP_001186170.1 | 8942 | kynureninase isoform a | 10.1 | 2 | 2 | 2 | 3 | 5.63E+07 | - | - | - | 465 | 52.3 | 7.0 | Metabolism;Energy pathways | - | Hydrolase activity | - |
| L3HYPDH | NP_653182.1 | 112849 | trans-L-3-hydroxyproline dehydratase | 4.8 | 1 | 1 | 1 | 1 | 3.43E+06 | - | - | - | 354 | 38.1 | 6.7 | Biological_process unknown | - | Molecular function unknown | - |
| LAD1 | NP_005549.2 | 3898 | ladinin-1 | 41.8 | 1 | 13 | 13 | 18 | 2.84E+08 | - | - | - | 517 | 57.1 | 9.7 | Cell growth and/or maintenance | Plasma membrane | Cytoskeletal anchoring activity | Plasma membrane |
| LAMA3 | NP_001121190.1 | 3909 | laminin subunit alpha-3 isoform 4 precursor | 0.9 | 4 | 1 | 1 | 1 | 8.71E+06 | - | - | - | 1668 | 183.9 | 8.2 | Cell growth and/or maintenance | Extracellular | Extracellular matrix structural constituent | Extracellular |
| LAMA4 | NP_002281.3 | 3910 | laminin subunit alpha-4 isoform 2 precursor | 1.0 | 2 | 1 | 1 | 1 | 1.83E+06 | - | - | - | 1816 | 201.7 | 6.3 | Cell growth and/or maintenance | Extracellular;Plasma membrane | Cell adhesion molecule activity | Extracellular |
| LAMB4 | NP_031382.2 | 22798 | laminin subunit beta-4 precursor | 1.9 | 1 | 1 | 1 | 1 | 8.82E+08 | - | - | - | 1761 | 193.4 | 6.4 | Biological_process unknown | - | Molecular function unknown | - |
| LAPTM4A | NP_055528.1 | 9741 | lysosomal-associated transmembrane protein 4A | 11.6 | 1 | 1 | 1 | 1 | 1.29E+07 | - | - | - | 233 | 26.8 | 6.5 | Transport | Golgi apparatus | Auxiliary transport protein activity | Golgi apparatus |
| LARP6 | NP_932062.1 | 55323 | la-related protein 6 isoform 2 | 12.9 | 2 | 1 | 1 | 1 | 1.14E+07 | - | - | - | 93 | 9.8 | 4.6 | Regulation of nucleobase, nucleoside, nucleotide and nucleic acid metabolism | - | RNA binding | - |
| LCLAT1 | NP_001002257.1 | 253558 | lysocardiolipin acyltransferase 1 isoform 2 | 4.8 | 2 | 1 | 1 | 1 | 1.21E+08 | - | - | - | 376 | 44.5 | 8.6 | Metabolism | Mitochondrion;Microsome;Endoplasmic reticulum | Acyltransferase activity | Endoplasmic reticulum |
| LDLRAP1 | NP_056442.2 | 26119 | low density lipoprotein receptor adapter protein 1 | 3.9 | 1 | 1 | 1 | 1 | 2.99E+06 | - | - | - | 308 | 33.9 | 6.7 | Cell communication;Signal transduction | Plasma membrane;Endosome | Receptor signaling complex scaffold activity | Plasma membrane |
| LEMD2 | NP_001137416.1 | 221496 | LEM domain-containing protein 2 isoform 2 | 6.5 | 2 | 1 | 1 | 1 | 5.49E+06 | - | - | - | 201 | 23.5 | 6.2 | Biological_process unknown | Integral to membrane;Nucleus | Molecular function unknown | Integral to membrane |
| LENG8 | NP_443157.1 | 114823 | leukocyte receptor cluster member 8 | 0.8 | 1 | 1 | 1 | 1 | 5.52E+07 | - | - | - | 800 | 88.1 | 9.1 | Biological_process unknown | - | Molecular function unknown | - |
| LIAS | NP_001265519.1 | 11019 | lipoyl synthase, mitochondrial isoform 3 precursor | 5.2 | 2 | 1 | 1 | 1 | 0.00E+00 | - | - | - | 329 | 37.1 | 8.9 | Metabolism;Energy pathways | Mitochondrion | Sulfotransferase activity | Mitochondrion |
| LIMA1 | NP_001107018.1 | 51474 | LIM domain and actin-binding protein 1 isoform 1 | 32.5 | 2 | 1 | 14 | 29 | 4.18E+08 | - | - | - | 760 | 85.2 | 6.8 | Cell growth and/or maintenance | Cytoplasm;Cytoskeleton;Nucleus | Structural constituent of cytoskeleton | Cytoplasm |
| LMAN2L | NP_110432.1 | 81562 | VIP36-like protein isoform 2 precursor | 12.9 | 2 | 3 | 3 | 3 | 2.21E+07 | - | - | - | 348 | 39.7 | 8.4 | Transport | Plasma membrane;Golgi apparatus;Endoplasmic reticulum | Transporter activity | Endoplasmic reticulum |
| LMX1B | NP_002307.2 | 4010 | LIM homeobox transcription factor 1-beta isoform 1 | 7.3 | 3 | 1 | 1 | 2 | 1.28E+09 | - | - | - | 395 | 44.1 | 6.7 | Regulation of nucleobase, nucleoside, nucleotide and nucleic acid metabolism | Nucleus | Transcription factor activity | Nucleus |
| LNPEP | NP_005566.2 | 4012 | leucyl-cystinyl aminopeptidase isoform 1 | 5.4 | 2 | 2 | 2 | 4 | 1.51E+08 | - | - | - | 1025 | 117.3 | 5.7 | Protein metabolism | Plasma membrane;Extracellular | Aminopeptidase activity | Plasma membrane |
| LOC101060602 | XP_006726777.1 | 101060602 | multidrug and toxin extrusion protein 2-like isoform X1 | 4.0 | 1 | 1 | 1 | 2 | 4.24E+06 | - | - | - | 201 | 22.6 | 4.6 | - | - | - | - |
| LOC101060817 | XP_003960941.1 | 101060817 | glycine cleavage system H protein, mitochondrial-like | 11.6 | 2 | 1 | 1 | 7 | 1.47E+08 | - | - | - | 173 | 18.8 | 4.9 | - | - | - | - |
| LOC101927614 | XP_005275984.1 | 101927614 | uncharacterized protein LOC101927614 | 16.3 | 1 | 2 | 2 | 3 | 3.07E+08 | - | - | - | 178 | 19.4 | 9.2 | - | - | - | - |
| LOC101929070 | XP_006721479.1 | 101929070 | uncharacterized transmembrane protein DDB_G0289901-like isoform X71 | 4.0 | 5 | 1 | 1 | 1 | 5.09E+07 | - | - | - | 601 | 69.2 | 10.7 | - | - | - | - |
| LOC101929991 | XP_006726839.1 | 101929991 | putative zinc finger protein 66-like | 6.2 | 1 | 1 | 1 | 4 | 1.36E+07 | - | - | - | 289 | 32.7 | 9.3 | - | - | - | - |
| LOC102724073 | XP_006710087.1 | 102724073 | spermatid maturation protein 1-like isoform X7 | 4.8 | 2 | 1 | 1 | 1 | 9.64E+07 | - | - | - | 145 | 16.3 | 8.9 | - | - | - | - |
| LOC102724147 | XP_006719789.1 | 102724147 | uncharacterized protein LOC102724147 | 6.9 | 1 | 1 | 1 | 1 | 1.56E+10 | - | - | - | 363 | 38.4 | 10.0 | - | - | - | - |
| LOC102724709 | XP_006726703.1 | 102724709 | fibroin heavy chain-like | 19.0 | 2 | 1 | 1 | 1 | 0.00E+00 | - | - | - | 232 | 23.4 | 5.2 | - | - | - | - |
| LOC102724975 | XP_006721419.1 | 102724975 | collagen alpha-3(IV) chain-like | 14.2 | 1 | 1 | 1 | 1 | 2.22E+08 | - | - | - | 260 | 26.6 | 11.3 | - | - | - | - |
| LOC102725469 | XP_006710059.1 | 102725469 | interleukin-9 receptor-like | 12.2 | 1 | 1 | 1 | 1 | 2.63E+07 | - | - | - | 189 | 21 | 10.1 | - | - | - | - |
| LOC728763 | XP_006710208.1 | 728763 | rootletin-like | 2.7 | 3 | 1 | 1 | 1 | 7.70E+06 | - | - | - | 666 | 74.4 | 6.9 | - | - | - | - |
| LOH12CR1 | NP_477517.1 | 118426 | loss of heterozygosity 12 chromosomal region 1 protein | 6.6 | 1 | 1 | 1 | 1 | 2.62E+06 | - | - | - | 196 | 22.2 | 6.4 | Biological_process unknown | - | Molecular function unknown | - |
| LOX | NP_002308.2 | 4015 | protein-lysine 6-oxidase isoform 1 preproprotein | 4.3 | 1 | 2 | 2 | 2 | 4.44E+07 | - | - | - | 417 | 46.9 | 8.1 | Metabolism;Energy pathways | Extracellular;Cytoplasm;Nucleus | Catalytic activity | Extracellular |
| LPCAT3 | NP_005759.4 | 10162 | lysophospholipid acyltransferase 5 | 8.0 | 1 | 2 | 2 | 2 | 1.74E+06 | - | - | - | 487 | 56 | 8.7 | Biological_process unknown | Integral to membrane | Molecular function unknown | Integral to membrane |
| LPIN1 | NP_663731.1 | 23175 | phosphatidate phosphatase LPIN1 isoform 1 | 2.6 | 3 | 1 | 1 | 1 | 1.13E+07 | - | - | - | 890 | 98.6 | 6.6 | Biological_process unknown | Nucleus | Molecular function unknown | Nucleus |
| LPIN2 | NP_055461.1 | 9663 | phosphatidate phosphatase LPIN2 | 1.6 | 1 | 1 | 1 | 1 | 2.76E+07 | - | - | - | 896 | 99.3 | 5.3 | Cell growth and/or maintenance | Nucleus;Cytoplasm | Molecular function unknown | Nucleus |
| LRCH3 | NP_116162.1 | 84859 | leucine-rich repeat and calponin homology domain-containing protein 3 precursor | 2.1 | 1 | 1 | 1 | 1 | 2.98E+07 | - | - | - | 712 | 78.8 | 6.6 | Biological_process unknown | - | Molecular function unknown | - |
| LRRC1 | NP_060684.4 | 55227 | leucine-rich repeat-containing protein 1 | 12.2 | 1 | 2 | 3 | 6 | 2.62E+07 | - | - | - | 524 | 59.2 | 5.0 | Biological_process unknown | - | Molecular function unknown | - |
| LRRC14 | NP_001258965.1 | 9684 | leucine-rich repeat-containing protein 14 | 1.6 | 1 | 1 | 1 | 1 | 1.05E+07 | - | - | - | 493 | 54.5 | 6.9 | Biological_process unknown | - | Molecular function unknown | - |
| LRRC4C | NP_065980.1 | 57689 | leucine-rich repeat-containing protein 4C precursor | 0.9 | 1 | 1 | 1 | 2 | 2.26E+08 | - | - | - | 640 | 71.9 | 7.2 | Cell communication;Signal transduction | Integral to membrane;Plasma membrane | Protein binding | Integral to membrane |

| Gene Symbol | RefSeq_Accession | Entrez Gene ID | Description | Coverage (%) | Proteins | Unique Peptides | # Peptides | PSM | Area | SCC-R /SCC-S | SCC-R /SCC-S (Count) | SCC-R /SCC-S (Variability [%]) | # AAs | MW [kDa] | calc. pI | Biological Process | Localization | Molecular Function | Primary Localization |
|---|---|---|---|---|---|---|---|---|---|---|---|---|---|---|---|---|---|---|---|
| LRRC8C | NP_115646.2 | 84230 | leucine-rich repeat-containing protein 8C | 2.4 | 1 | 1 | 1 | 1 | 8.08E+05 | - | - | - | 803 | 92.3 | 7.7 | Cell growth and/or maintenance | Endoplasmic reticulum | Cytoskeletal anchoring activity | Endoplasmic reticulum |
| LRWD1 | NP_690852.1 | 222229 | leucine-rich repeat and WD repeat-containing protein 1 | 2.6 | 1 | 1 | 1 | 1 | 5.52E+07 | - | - | - | 647 | 70.8 | 7.2 | Biological_process unknown | Nucleus | Molecular function unknown | Nucleus |
| LSM1 | NP_055277.1 | 27257 | U6 snRNA-associated Sm-like protein LSm1 | 12.8 | 1 | 1 | 1 | 2 | 0.00E+00 | - | - | - | 133 | 15.2 | 5.2 | Regulation of nucleobase, nucleoside, nucleotide and nucleic acid metabolism | Cytoplasm;Nucleus | RNA binding | Cytoplasm |
| LSM14B | NP_653304.2 | 149986 | protein LSM14 homolog B | 11.7 | 1 | 2 | 2 | 3 | 3.46E+07 | - | - | - | 385 | 42 | 9.7 | Biological_process unknown | - | Molecular function unknown | - |
| LSR | NP_001247419.1 | 51599 | lipolysis-stimulated lipoprotein receptor isoform 5 | 7.8 | 5 | 3 | 3 | 4 | 2.53E+07 | - | - | - | 541 | 59.7 | 9.4 | Lipoprotein metabolism | Plasma membrane | Receptor activity | Plasma membrane |
| LTV1 | NP_116249.2 | 84946 | protein LTV1 homolog | 7.2 | 1 | 1 | 1 | 2 | 4.15E+07 | - | - | - | 475 | 54.8 | 4.9 | Biological_process unknown | Cytoplasm | Molecular function unknown | Cytoplasm |
| LY6D | NP_003686.1 | 8581 | lymphocyte antigen 6D precursor | 10.9 | 1 | 1 | 1 | 1 | 0.00E+00 | - | - | - | 128 | 13.3 | 8.2 | Cell communication;Signal transduction | Plasma membrane | Cell adhesion molecule activity | Plasma membrane |
| LYPLAL1 | NP_620149.1 | 127018 | lysophospholipase-like protein 1 | 19.0 | 1 | 2 | 2 | 3 | 1.53E+07 | - | - | - | 237 | 26.3 | 7.8 | Biological_process unknown | - | Molecular function unknown | - |
| LYRM7 | NP_859056.2 | 90624 | complex III assembly factor LYRM7 | 14.4 | 1 | 1 | 1 | 2 | 0.00E+00 | - | - | - | 104 | 11.9 | 9.7 | Biological_process unknown | - | Molecular function unknown | - |
| LYZ | NP_000230.1 | 4069 | lysozyme C precursor | 27.0 | 1 | 2 | 2 | 2 | 8.42E+06 | - | - | - | 148 | 16.5 | 9.2 | Metabolism;Energy pathways | Extracellular | Hydrolase activity | Extracellular |
| LZIC | NP_115744.2 | 84328 | protein LZIC | 16.3 | 1 | 2 | 2 | 6 | 3.68E+07 | - | - | - | 190 | 21.5 | 4.9 | Biological_process unknown | - | Molecular function unknown | - |
| MAD2L1 | NP_002349.1 | 4085 | mitotic spindle assembly checkpoint protein MAD2A | 7.8 | 1 | 1 | 1 | 1 | 2.69E+07 | - | - | - | 205 | 23.5 | 5.1 | Cell communication;Signal transduction | Kinetochore;Nucleus;Chromosome;Cytoplasm;Nuclear membrane | Protein binding | Nucleus |
| MAEA | NP_005873.2 | 10296 | macrophage erythroblast attacher isoform 2 | 4.5 | 2 | 1 | 1 | 1 | 1.41E+07 | - | - | - | 355 | 40.4 | 8.8 | Cell adhesion | Plasma membrane | Cell adhesion molecule activity | Plasma membrane |
| MAGEB2 | NP_002355.2 | 4113 | melanoma-associated antigen B2 | 7.8 | 1 | 1 | 1 | 1 | 0.00E+00 | - | - | - | 319 | 35.3 | 8.8 | Immune response | Nucleus | Molecular function unknown | Nucleus |
| MAGI2 | NP_036433.2 | 9863 | membrane-associated guanylate kinase, WW and PDZ domain-containing protein 2 | 2.4 | 1 | 1 | 1 | 1 | 4.94E+08 | - | - | - | 1455 | 158.7 | 6.4 | Cell communication;Signal transduction | Cytoplasm | Molecular function unknown | Cytoplasm |
| MAN1A1 | NP_005898.2 | 4121 | mannosyl-oligosaccharide 1,2-alpha-mannosidase IA | 4.1 | 1 | 1 | 1 | 1 | 1.61E+07 | - | - | - | 653 | 72.9 | 6.5 | Metabolism;Energy pathways | Endoplasmic reticulum;Golgi apparatus | Hydrolase activity | Endoplasmic reticulum |
| MAP1B | NP_005900.2 | 4131 | microtubule-associated protein 1B | 20.0 | 1 | 23 | 23 | 36 | 2.25E+08 | - | - | - | 2468 | 270.5 | 4.8 | Cell growth and/or maintenance | Cytoplasm;Plasma membrane;Nucleus | Cytoskeletal protein binding | Cytoplasm |
| MAP1S | NP_060644.4 | 55201 | microtubule-associated protein 1S | 1.8 | 1 | 1 | 1 | 1 | 0.00E+00 | - | - | - | 1059 | 112.1 | 7.3 | Cell growth and/or maintenance | Microtubule | Structural constituent of cytoskeleton | Microtubule |
| MAP2K3 | NP_002747.2 | 5606 | dual specificity mitogen-activated protein kinase kinase 3 isoform A | 8.5 | 3 | 1 | 2 | 3 | 1.84E+07 | - | - | - | 318 | 36.1 | 6.3 | Signal transduction | Nucleus;Cytoplasm | Protein serine/threonine kinase activity | Nucleus |
| MAP2K6 | NP_002749.2 | 5608 | dual specificity mitogen-activated protein kinase kinase 6 | 15.3 | 2 | 2 | 3 | 6 | 4.39E+07 | - | - | - | 334 | 37.5 | 7.4 | Signal transduction;Regulation of cell cycle | Nucleus;Cytoplasm | Protein serine/threonine kinase activity | Nucleus |
| MAP3K12 | NP_006292.3 | 7786 | mitogen-activated protein kinase kinase kinase 12 isoform 2 | 0.8 | 2 | 1 | 1 | 1 | 8.67E+07 | - | - | - | 859 | 93.2 | 6.5 | Cell communication;Signal transduction | Golgi apparatus;Nucleus;Cytoplasm;Plasma membrane | Protein serine/threonine kinase activity | Golgi apparatus |
| MAP3K2 | NP_006600.3 | 10746 | mitogen-activated protein kinase kinase kinase 2 | 3.1 | 1 | 1 | 1 | 1 | 8.52E+05 | - | - | - | 619 | 69.7 | 8.0 | Cell communication;Signal transduction | - | Protein serine/threonine kinase activity | - |
| MAPKAP1 | NP_001006619.1 | 79109 | target of rapamycin complex 2 subunit MAPKAP1 isoform 5 | 4.3 | 4 | 1 | 1 | 1 | 3.76E+07 | - | - | - | 323 | 36.2 | 7.8 | Cell communication;Signal transduction | Cytoplasm;Nucleus;Plasma membrane | Molecular function unknown;Kinase binding | Cytoplasm |
| MAPKAPK3 | NP_001230855.1 | 7867 | MAP kinase-activated protein kinase 3 | 11.5 | 1 | 2 | 2 | 2 | 1.93E+07 | - | - | - | 382 | 43 | 7.3 | Cell communication;Signal transduction | Cytoplasm;Nucleus | Protein serine/threonine kinase activity | Nucleus |
| MARVELD2 | NP_001231663.1 | 153562 | MARVEL domain-containing protein 2 isoform 2 | 4.0 | 2 | 1 | 1 | 1 | 2.88E+07 | - | - | - | 546 | 62.8 | 7.2 | Biological_process unknown | Nucleus | Molecular function unknown | Nucleus |
| MAU2 | NP_056144.3 | 23383 | MAU2 chromatid cohesion factor homolog | 2.5 | 1 | 1 | 1 | 1 | 2.31E+07 | - | - | - | 613 | 69 | 7.3 | Biological_process unknown | Chromosome;Nucleus;Microtubule | Molecular function unknown | Chromosome |
| MAX | NP_660087.1 | 4149 | protein max isoform b | 13.9 | 2 | 1 | 1 | 1 | 1.35E+07 | - | - | - | 151 | 17.2 | 6.6 | Regulation of nucleobase, nucleoside, nucleotide and nucleic acid metabolism | Nucleus;Cytoplasm | Transcription factor activity | Nucleus;Cytoplasm |
| MBNL1 | NP_997176.1 | 4154 | muscleblind-like protein 1 isoform c | 18.6 | 2 | 1 | 4 | 10 | 1.46E+08 | - | - | - | 388 | 41.8 | 8.9 | Regulation of gene expression, epigenetic | Nucleus;Cytoplasm | RNA binding | Nucleus;Cytoplasm |
| MBNL1 | NP_997177.1 | 4154 | muscleblind-like protein 1 isoform d | 25.8 | 6 | 1 | 4 | 9 | 1.31E+08 | - | - | - | 302 | 33 | 8.8 | Regulation of gene expression, epigenetic | Nucleus;Cytoplasm | RNA binding | Nucleus;Cytoplasm |
| MBOAT2 | NP_620154.2 | 129642 | lysophospholipid acyltransferase 2 | 5.8 | 1 | 1 | 1 | 2 | 2.16E+07 | - | - | - | 520 | 59.5 | 8.0 | Biological_process unknown | Plasma membrane | Molecular function unknown | Plasma membrane |
| MCM3AP | NP_003897.2 | 8888 | germinal-center associated nuclear protein | 0.9 | 1 | 1 | 1 | 2 | 7.82E+06 | - | - | - | 1980 | 218.3 | 6.4 | Cell communication;Signal transduction | Nucleus;Cytoplasm | Chromatin binding | Nucleus |
| MDK | NP_001257481.1 | 4192 | midkine isoform b precursor | 16.1 | 2 | 1 | 1 | 1 | 9.44E+06 | - | - | - | 87 | 9.5 | 10.1 | Cell communication;Signal transduction | Extracellular;Nucleus;Nucleolus;Cytoplasm | Cytokine activity | Extracellular |
| MECR | NP_001019903.2 | 51102 | trans-2-enoyl-CoA reductase, mitochondrial isoform b | 3.7 | 2 | 1 | 1 | 1 | 1.73E+06 | - | - | - | 297 | 32.2 | 7.2 | Metabolism;Energy pathways | Mitochondrion;Nucleus | Catalytic activity | Mitochondrion |
| MED17 | NP_004259.3 | 9440 | mediator of RNA polymerase II transcription subunit 17 | 9.2 | 1 | 4 | 4 | 5 | 8.53E+06 | - | - | - | 651 | 72.8 | 7.4 | Regulation of nucleobase, nucleoside, nucleotide and nucleic acid metabolism | Nucleus | Transcription factor activity | Nucleus |
| MED19 | NP_703151.1 | 219541 | mediator of RNA polymerase II transcription subunit 19 | 15.5 | 1 | 1 | 1 | 1 | 4.25E+07 | - | - | - | 194 | 20.4 | 9.4 | Cell growth and/or maintenance | Nucleus | Structural molecule activity | Nucleus |
| MED24 | NP_001072986.1 | 9862 | mediator of RNA polymerase II transcription subunit 24 isoform 2 | 3.2 | 2 | 2 | 2 | 3 | 2.49E+07 | - | - | - | 976 | 108.9 | 6.9 | Regulation of nucleobase, nucleoside, nucleotide and nucleic acid metabolism | Nucleus | Transcription regulator activity | Nucleus |
| MED25 | NP_112235.2 | 81857 | mediator of RNA polymerase II transcription subunit 25 | 3.2 | 1 | 1 | 1 | 1 | 5.62E+07 | - | - | - | 747 | 78.1 | 8.3 | Regulation of nucleobase, nucleoside, nucleotide and nucleic acid metabolism | Nucleus | Transcription regulator activity | Nucleus |

| Gene Symbol | RefSeq_Accession | Entrez Gene ID | Description | Coverage (%) | Proteins | Unique Peptides | # Peptides | PSM | Area | SCC-R /SCC-S | SCC-R /SCC-S (Count) | SCC-R /SCC-S (Variability [%]) | # AAs | MW [kDa] | calc. pI | Biological Process | Localization | Molecular Function | Primary Localization |
|---|---|---|---|---|---|---|---|---|---|---|---|---|---|---|---|---|---|---|---|
| MED7 | NP_004261.1 | 9443 | mediator of RNA polymerase II transcription subunit 7 | 20.2 | 1 | 1 | 1 | 1 | 9.54E+05 | - | - | - | 233 | 27.2 | 5.8 | Regulation of nucleobase, nucleoside, nucleotide and nucleic acid metabolism | Nucleus | Transcription factor activity | Nucleus |
| MEFV | NP_000234.1 | 4210 | pyrin isoform 1 | 3.1 | 1 | 1 | 1 | 2 | 0.00E+00 | - | - | - | 781 | 86.4 | 8.0 | Regulation of nucleobase, nucleoside, nucleotide and nucleic acid metabolism | Cytoplasm;Nucleus;Golgi apparatus | Transcription factor activity | Cytoplasm |
| MEST | NP_001240831.1 | 4232 | mesoderm-specific transcript homolog protein isoform d precursor | 6.5 | 4 | 1 | 1 | 1 | 1.26E+07 | - | - | - | 292 | 33.7 | 9.7 | Metabolism;Energy pathways | Cytoplasm | Hydrolase activity | Cytoplasm |
| METTL13 | NP_055770.1 | 51603 | methyltransferase-like protein 13 isoform 2 | 2.8 | 2 | 1 | 1 | 1 | 7.80E+06 | - | - | - | 613 | 68.6 | 6.7 | Biological_process unknown | - | Molecular function unknown | - |
| METTL16 | NP_076991.3 | 79066 | methyltransferase-like protein 16 | 6.2 | 1 | 2 | 2 | 2 | 2.90E+07 | - | - | - | 562 | 63.6 | 7.9 | Biological_process unknown | - | Molecular function unknown | - |
| METTL7A | NP_054752.3 | 25840 | methyltransferase-like protein 7A precursor | 15.6 | 1 | 2 | 2 | 2 | 4.16E+07 | - | - | - | 244 | 28.3 | 8.4 | Metabolism;Energy pathways | - | Methyltransferase activity | - |
| MEX3D | NP_976049.3 | 399664 | RNA-binding protein MEX3D isoform 1 | 1.7 | 1 | 1 | 1 | 1 | 6.67E+07 | - | - | - | 651 | 64.8 | 5.4 | Regulation of nucleobase, nucleoside, nucleotide and nucleic acid metabolism | Cytoplasm;Nucleus;Perinuclear region | RNA binding | Nucleus |
| MFN1 | NP_284941.2 | 55669 | mitofusin-1 | 2.8 | 1 | 1 | 1 | 2 | 7.35E+07 | - | - | - | 741 | 84.1 | 6.3 | Mitochondrion organization and biogenesis | Mitochondrion | GTPase activity | Mitochondrion |
| MGAT5B | NP_653278.2 | 146664 | alpha-1,6-mannosylglycoprotein 6-beta-N-acetylglucosaminyltransferase B isoform 1 | 0.9 | 3 | 1 | 1 | 1 | 1.23E+06 | - | - | - | 790 | 89.2 | 8.4 | Cell adhesion | - | Transferase activity | - |
| MGLL | NP_001003794.1 | 11343 | monoglyceride lipase isoform 2 | 31.4 | 3 | 6 | 6 | 10 | 1.06E+08 | - | - | - | 303 | 33.2 | 7.0 | Lipid metabolism | - | Lipase activity | - |
| MICU1 | NP_001182447.1 | 10367 | calcium uptake protein 1, mitochondrial isoform 2 | 4.0 | 2 | 1 | 1 | 1 | 1.50E+06 | - | - | - | 476 | 54.3 | 8.4 | Immune response | Extracellular | Molecular function unknown | Extracellular |
| MKL1 | NP_001269591.1 | 57591 | MKL/myocardin-like protein 1 isoform 3 | 3.6 | 3 | 1 | 1 | 2 | 4.12E+05 | - | - | - | 798 | 85 | 7.8 | Regulation of nucleobase, nucleoside, nucleotide and nucleic acid metabolism | Nucleus;Cytoplasm | Transcription factor activity | Nucleus |
| MKL2 | NP_054767.3 | 57496 | MKL/myocardin-like protein 2 | 6.0 | 1 | 3 | 3 | 3 | 5.37E+06 | - | - | - | 1049 | 114 | 6.0 | Regulation of nucleobase, nucleoside, nucleotide and nucleic acid metabolism | Nucleus;Cytoplasm | Transcription factor activity | Nucleus |
| MLST8 | NP_001186104.1 | 64223 | target of rapamycin complex subunit LST8 isoform b | 5.5 | 2 | 1 | 1 | 1 | 1.76E+06 | - | - | - | 325 | 35.7 | 5.9 | Cell communication;Signal transduction | Cytoplasm;Integral to membrane | Receptor signaling complex scaffold activity | Cytoplasm |
| MOB1A | NP_060691.2 | 55233 | MOB kinase activator 1A | 18.5 | 2 | 1 | 4 | 6 | 1.67E+08 | - | - | - | 216 | 25.1 | 7.0 | Biological_process unknown | Cytoplasm | Molecular function unknown | Cytoplasm |
| MORN3 | NP_776254.3 | 283385 | MORN repeat-containing protein 3 | 7.5 | 1 | 1 | 1 | 1 | 2.19E+07 | - | - | - | 240 | 27.6 | 8.4 | Biological_process unknown | - | Molecular function unknown | - |
| MPG | NP_001015054.1 | 4350 | DNA-3-methyladenine glycosylase isoform c | 9.6 | 3 | 1 | 1 | 2 | 7.66E+06 | - | - | - | 281 | 30.8 | 9.2 | DNA repair | Nucleus;Cytoplasm | Hydrolase activity | Nucleus |
| MPLKIP | NP_619646.1 | 136647 | M-phase-specific PLK1-interacting protein | 14.0 | 1 | 1 | 1 | 1 | 8.31E+06 | - | - | - | 179 | 19.1 | 10.2 | Biological_process unknown | Cytoplasm | Molecular function unknown | Cytoplasm |
| MPZL2 | NP_005788.1 | 10205 | myelin protein zero-like protein 2 precursor | 6.1 | 1 | 1 | 1 | 1 | 2.04E+07 | - | - | - | 215 | 24.5 | 7.0 | Cell adhesion;Organogenesis | Plasma membrane | Cell adhesion molecule activity | Plasma membrane |
| MROH2A | NP_001274324.1 | 339766 | maestro heat-like repeat-containing protein family member 2A | 0.6 | 1 | 1 | 1 | 1 | 1.97E+08 | - | - | - | 1688 | 191.1 | 6.9 | - | - | - | - |
| MRPL23 | NP_066957.3 | 6150 | 39S ribosomal protein L23, mitochondrial | 24.2 | 2 | 2 | 2 | 5 | 1.03E+08 | - | - | - | 153 | 17.8 | 9.7 | Protein metabolism | Ribosome;Mitochondrion | Structural constituent of ribosome | Ribosome |
| MRPL30 | NP_660213.1 | 51263 | 39S ribosomal protein L30, mitochondrial precursor | 17.4 | 1 | 2 | 2 | 2 | 9.69E+07 | - | - | - | 161 | 18.5 | 10.0 | Protein metabolism | Mitochondrion | Structural constituent of ribosome | Mitochondrion |
| MRPL48 | NP_057139.1 | 51642 | 39S ribosomal protein L48, mitochondrial | 6.1 | 1 | 1 | 1 | 1 | 2.40E+07 | - | - | - | 212 | 23.9 | 9.0 | Protein metabolism | Mitochondrion | Structural constituent of ribosome | Mitochondrion |
| MRPS10 | NP_060611.2 | 55173 | 28S ribosomal protein S10, mitochondrial | 9.5 | 1 | 1 | 1 | 3 | 4.31E+07 | - | - | - | 201 | 23 | 8.0 | Protein metabolism | Mitochondrion | Structural constituent of ribosome | Mitochondrion |
| MRPS11 | NP_073750.2 | 64963 | 28S ribosomal protein S11, mitochondrial isoform a | 23.2 | 2 | 2 | 2 | 2 | 9.61E+06 | - | - | - | 194 | 20.6 | 10.8 | Protein metabolism | Mitochondrion | Structural constituent of ribosome | Mitochondrion |
| MRPS15 | NP_112570.2 | 64960 | 28S ribosomal protein S15, mitochondrial | 3.9 | 1 | 1 | 1 | 1 | 2.03E+08 | - | - | - | 257 | 29.8 | 10.5 | Protein metabolism | Mitochondrion;Ribosome | Structural constituent of ribosome | Mitochondrion |
| MRPS18A | NP_001180272.1 | 55168 | 28S ribosomal protein S18a, mitochondrial isoform 2 precursor | 17.3 | 2 | 1 | 1 | 1 | 9.58E+06 | - | - | - | 127 | 14 | 9.5 | Protein metabolism | Mitochondrion | Structural constituent of ribosome | Mitochondrion |
| MRPS2 | NP_057118.1 | 51116 | 28S ribosomal protein S2, mitochondrial | 6.4 | 1 | 1 | 1 | 1 | 2.91E+07 | - | - | - | 296 | 33.2 | 9.3 | Protein metabolism | Mitochondrion | Structural constituent of ribosome | Mitochondrion |
| MS4A14 | NP_001073160.1 | 84689 | membrane-spanning 4-domains subfamily A member 14 isoform 2 | 1.1 | 4 | 1 | 1 | 1 | 1.33E+07 | - | - | - | 662 | 74.6 | 5.5 | Cell communication;Signal transduction | Plasma membrane | Molecular function unknown | Plasma membrane |
| MSRB3 | NP_001180390.1 | 253827 | methionine-R-sulfoxide reductase B3 isoform 2 precursor | 6.0 | 1 | 1 | 1 | 1 | 0.00E+00 | - | - | - | 185 | 20 | 7.2 | Metabolism;Energy pathways | Endoplasmic reticulum;Mitochondrion | Catalytic activity | Endoplasmic reticulum |
| MT1G | NP_005941.1 | 4495 | metallothionein-1G | 67.2 | 4 | 1 | 4 | 12 | 1.09E+09 | - | - | - | 61 | 6.1 | 8.0 | Biological_process unknown | - | Molecular function unknown | - |
| MTFR1L | NP_001093097.1 | 56181 | mitochondrial fission regulator 1-like isoform b | 5.9 | 2 | 1 | 1 | 1 | 5.10E+06 | - | - | - | 205 | 22.8 | 10.6 | Biological_process unknown | - | Molecular function unknown | - |
| MTG2 | NP_056481.1 | 26164 | mitochondrial ribosome-associated GTPase 2 | 3.5 | 1 | 1 | 1 | 1 | 5.27E+06 | - | - | - | 406 | 43.9 | 9.5 | Biological_process unknown | Mitochondrion | Molecular function unknown | Mitochondrion |
| MTIF2 | NP_001005369.1 | 4528 | translation initiation factor IF-2, mitochondrial precursor | 2.3 | 1 | 1 | 1 | 1 | 5.57E+06 | - | - | - | 727 | 81.3 | 7.2 | Protein metabolism | Mitochondrion | Translation regulator activity | Mitochondrion |
| MTMR12 | NP_001035536.1 | 54545 | myotubularin-related protein 12 | 2.8 | 1 | 1 | 1 | 1 | 5.63E+06 | - | - | - | 747 | 86.1 | 6.6 | Cell communication;Signal transduction | Cytoplasm | Receptor signaling complex scaffold activity | Cytoplasm |
| MTUS2 | NP_001028774.2 | 23281 | microtubule-associated tumor suppressor candidate 2 isoform a | 1.5 | 1 | 1 | 2 | 2 | 4.58E+07 | - | - | - | 1379 | 151.1 | 6.7 | Biological_process unknown;Cytoskeleton organization and biogenesis | - | Molecular function unknown;Cytoskeletal anchoring activity | - |
| MUC16 | NP_078966.2 | 94025 | mucin-16 | 0.3 | 1 | 1 | 1 | 1 | 0.00E+00 | - | - | - | 14507 | 1518.2 | 5.3 | Cell communication;Signal transduction | Plasma membrane;Endoplasmic reticulum;Golgi apparatus;Extracellular | Molecular function unknown | Plasma membrane |

| Gene Symbol | RefSeq_Accession | Entrez Gene ID | Description | Coverage (%) | Proteins | Unique Peptides | # Peptides | PSM | Area | SCC-R /SCC-S | SCC-R /SCC-S (Count) | SCC-R /SCC-S (Variability [%]) | # AAs | MW [kDa] | calc. pI | Biological Process | Localization | Molecular Function | Primary Localization |
|---|---|---|---|---|---|---|---|---|---|---|---|---|---|---|---|---|---|---|---|
| MUC2 | NP_002448.3 | 4583 | mucin-2 precursor | 0.8 | 1 | 1 | 1 | 1 | 2.04E+09 | - | - | - | 5179 | 539.8 | 5.8 | Biological_process unknown | Endoplasmic reticulum;Golgi apparatus;Extracellular;Nucleus | Molecular function unknown | Endoplasmic reticulum |
| MUC4 | NP_060876.5 | 4585 | mucin-4 isoform a precursor | 0.3 | 1 | 1 | 1 | 1 | 1.02E+09 | - | - | - | 5412 | 542 | 5.3 | Cell communication;Signal transduction | Plasma membrane;Extracellular | Molecular function unknown | Plasma membrane |
| MX2 | NP_002454.1 | 4600 | interferon-induced GTP-binding protein Mx2 | 28.4 | 1 | 11 | 15 | 74 | 2.32E+09 | - | - | - | 715 | 82 | 8.8 | Immune response | Nucleus;Cytoplasm | GTPase activity | Nucleus |
| MYEOV2 | NP_001156896.1 | 150678 | myeloma-overexpressed gene 2 protein isoform 2 | 100.0 | 1 | 2 | 2 | 3 | 0.00E+00 | - | - | - | 57 | 6.2 | 3.8 | Biological_process unknown | - | Molecular function unknown | - |
| MYH13 | NP_003793.2 | 8735 | myosin-13 | 1.3 | 8 | 2 | 3 | 6 | 3.19E+08 | - | - | - | 1938 | 223.5 | 5.7 | Cell growth and/or maintenance | Cytoplasm | Motor activity | Cytoplasm |
| MYH4 | NP_060003.2 | 4622 | myosin-4 | 1.0 | 8 | 1 | 2 | 5 | 3.91E+08 | - | - | - | 1939 | 222.9 | 5.9 | Cell growth and/or maintenance | - | Structural molecule activity | - |
| MYL6 | NP_066299.2 | 4637 | myosin light polypeptide 6 isoform 1 | 87.4 | 4 | 1 | 13 | 337 | 6.64E+09 | - | - | - | 151 | 16.9 | 4.7 | Cell growth and/or maintenance | Cytoskeleton;Cytoplasm | Structural constituent of cytoskeleton | Cytoplasm |
| MYO1D | NP_056009.1 | 4642 | unconventional myosin-Id | 16.9 | 1 | 10 | 10 | 20 | 1.83E+08 | - | - | - | 1006 | 116.1 | 9.4 | Cell growth and/or maintenance | Cytoplasm | Structural molecule activity | Cytoplasm |
| MYPN | NP_001243197.1 | 84665 | myopalladin isoform b | 2.1 | 2 | 1 | 2 | 4 | 1.22E+08 | - | - | - | 1026 | 112.6 | 7.1 | Cell growth and/or maintenance | Nucleus | Structural constituent of cytoskeleton | Nucleus |
| MYT1L | NP_055840.2 | 23040 | myelin transcription factor 1-like protein | 0.6 | 1 | 1 | 1 | 1 | 1.30E+09 | - | - | - | 1184 | 132.8 | 5.0 | Regulation of nucleobase, nucleoside, nucleotide and nucleic acid metabolism | Nucleus | Transcription factor activity | Nucleus |
| NAALADL2 | NP_996898.2 | 254827 | inactive N-acetylated-alpha-linked acidic dipeptidase-like protein 2 | 0.8 | 1 | 1 | 1 | 2 | 1.10E+08 | - | - | - | 795 | 88.6 | 6.2 | Biological_process unknown | Integral to membrane | Molecular function unknown | Integral to membrane |
| NADK2 | NP_694558.1 | 133686 | NAD kinase 2, mitochondrial isoform 2 | 10.0 | 3 | 2 | 2 | 2 | 8.48E+06 | - | - | - | 279 | 31.7 | 6.7 | Biological_process unknown | - | Molecular function unknown | - |
| NANP | NP_689880.1 | 140838 | N-acylneuraminate-9-phosphatase | 10.1 | 1 | 1 | 1 | 2 | 1.70E+07 | - | - | - | 248 | 27.8 | 6.4 | Metabolism;Energy pathways | - | Hydrolase activity | - |
| NAV3 | NP_055718.4 | 89795 | neuron navigator 3 isoform 2 | 1.9 | 2 | 1 | 1 | 2 | 3.75E+08 | - | - | - | 2363 | 252.9 | 8.6 | Regulation of nucleobase, nucleoside, nucleotide and nucleic acid metabolism | Nucleus | Molecular function unknown | Nucleus |
| NBEAL2 | NP_055990.1 | 23218 | neurobeachin-like protein 2 | 1.0 | 1 | 1 | 1 | 1 | 1.06E+07 | - | - | - | 2754 | 302.3 | 6.4 | Biological_process unknown;Signal transduction | - | Molecular function unknown | - |
| NCAPD3 | NP_056076.1 | 23310 | condensin-2 complex subunit D3 | 4.9 | 1 | 3 | 3 | 3 | 1.66E+07 | - | - | - | 1498 | 168.8 | 7.5 | Biological_process unknown | Nucleolus;Mitochondrion;Nucleus | Molecular function unknown | Nucleolus |
| NDE1 | NP_060138.1 | 54820 | nuclear distribution protein nudE homolog 1 | 6.0 | 1 | 1 | 1 | 1 | 7.42E+06 | - | - | - | 335 | 37.7 | 5.2 | Cell growth and/or maintenance | Nucleus;Centrosome;Cytoplasm;Chromosome | Cytoskeletal protein binding | Nucleus |
| NDN | NP_002478.1 | 4692 | necdin | 2.8 | 1 | 1 | 1 | 1 | 0.00E+00 | - | - | - | 321 | 36.1 | 8.8 | Regulation of nucleobase, nucleoside, nucleotide and nucleic acid metabolism | Nucleus;Cytoplasm | DNA binding | Nucleus |
| NDNL2 | NP_619649.1 | 56160 | melanoma-associated antigen G1 | 2.0 | 1 | 1 | 1 | 2 | 8.98E+07 | - | - | - | 304 | 34.3 | 9.3 | Biological_process unknown | Nucleolus | Molecular function unknown | Nucleolus |
| NDUFA3 | NP_004533.1 | 4696 | NADH dehydrogenase [ubiquinone] 1 alpha subcomplex subunit 3 | 41.7 | 1 | 1 | 1 | 1 | 0.00E+00 | - | - | - | 84 | 9.3 | 8.5 | Metabolism;Energy pathways | Mitochondrion | Oxidoreductase activity | Mitochondrion |
| NDUFA4 | NP_002480.1 | 4697 | NADH dehydrogenase [ubiquinone] 1 alpha subcomplex subunit 4 | 27.2 | 1 | 2 | 2 | 5 | 5.78E+07 | - | - | - | 81 | 9.4 | 9.4 | Metabolism;Energy pathways | Mitochondrion | Oxidoreductase activity | Mitochondrion |
| NDUFAF1 | NP_057097.2 | 51103 | complex I intermediate-associated protein 30, mitochondrial precursor | 5.8 | 1 | 1 | 1 | 1 | 8.54E+06 | - | - | - | 327 | 37.7 | 7.6 | Biological_process unknown | Mitochondrion | Chaperone activity | Mitochondrion |
| NDUFB6 | NP_001186916.1 | 4712 | NADH dehydrogenase [ubiquinone] 1 beta subcomplex subunit 6 isoform 3 | 10.3 | 3 | 1 | 1 | 1 | 1.56E+08 | - | - | - | 97 | 11.7 | 9.5 | Metabolism;Energy pathways;Electron transport | Mitochondrial membrane;Mitochondrion | Electron transporter activity | Mitochondrial membrane |
| NDUFS6 | NP_004544.1 | 4726 | NADH dehydrogenase [ubiquinone] iron-sulfur protein 6, mitochondrial precursor | 31.5 | 1 | 3 | 3 | 3 | 4.29E+07 | - | - | - | 124 | 13.7 | 8.3 | Metabolism;Energy pathways | Mitochondrion | Catalytic activity | Mitochondrion |
| NEB | NP_004534.2 | 4703 | nebulin isoform 3 | 0.2 | 4 | 1 | 1 | 1 | 9.14E+07 | - | - | - | 6669 | 772.4 | 9.1 | Cell growth and/or maintenance | Cytoplasm;Extracellular | Structural constituent of cytoskeleton | Cytoplasm |
| NEBL | NP_006384.1 | 10529 | nebulette isoform 1 | 2.2 | 1 | 1 | 1 | 1 | 1.05E+09 | - | - | - | 1014 | 116.4 | 8.0 | Cell growth and/or maintenance | Cytoskeleton | Cytoskeletal protein binding | Cytoskeleton |
| NECAP1 | NP_056324.2 | 25977 | adaptin ear-binding coat-associated protein 1 | 24.4 | 1 | 3 | 3 | 10 | 6.30E+08 | - | - | - | 275 | 29.7 | 6.8 | Biological_process unknown | - | Molecular function unknown | - |
| NEO1 | NP_001166094.1 | 4756 | neogenin isoform 2 precursor | 1.6 | 3 | 1 | 1 | 1 | 3.03E+07 | - | - | - | 1408 | 154.2 | 6.4 | Cell communication;Signal transduction | Plasma membrane | Receptor activity | Plasma membrane |
| NES | NP_006608.1 | 10763 | nestin | 2.0 | 1 | 2 | 2 | 2 | 9.08E+07 | - | - | - | 1621 | 177.3 | 4.4 | Cell growth and/or maintenance | Cytoplasm;Nucleus | Structural constituent of cytoskeleton | Cytoplasm |
| NEURL3 | NP_001272415.1 | 93082 | E3 ubiquitin-protein ligase NEURL3 isoform b | 23.4 | 1 | 1 | 1 | 1 | 0.00E+00 | - | - | - | 128 | 13.9 | 8.3 | Biological_process unknown | - | Molecular function unknown | - |
| NFAT5 | NP_775322.1 | 10725 | nuclear factor of activated T-cells 5 isoform a | 3.2 | 4 | 1 | 1 | 1 | 3.53E+06 | - | - | - | 1455 | 157.9 | 5.4 | Regulation of nucleobase, nucleoside, nucleotide and nucleic acid metabolism | Nucleus;Cytoplasm | Transcription factor activity | Nucleus;Cytoplasm |
| NFATC4 | NP_001185894.1 | 4776 | nuclear factor of activated T-cells, cytoplasmic 4 isoform 3 | 0.9 | 6 | 1 | 1 | 3 | 4.54E+07 | - | - | - | 794 | 84.2 | 5.4 | Regulation of nucleobase, nucleoside, nucleotide and nucleic acid metabolism | Nucleus;Cytoplasm | Transcription factor activity | Nucleus |
| NFIL3 | NP_005375.2 | 4783 | nuclear factor interleukin-3-regulated protein | 6.3 | 1 | 1 | 1 | 2 | 9.62E+08 | - | - | - | 462 | 51.4 | 7.2 | Regulation of nucleobase, nucleoside, nucleotide and nucleic acid metabolism | Nucleus | Transcription factor activity | Nucleus |
| NFIX | NP_001257973.1 | 4784 | nuclear factor 1 X-type isoform 3 | 5.8 | 10 | 1 | 2 | 2 | 2.61E+07 | - | - | - | 433 | 47.9 | 8.5 | Regulation of nucleobase, nucleoside, nucleotide and nucleic acid metabolism | Nucleus | Transcription factor activity | Nucleus |
| NGLY1 | NP_001138767.1 | 55768 | peptide-N(4)-(N-acetyl-beta-glucosaminyl)asparagine amidase isoform 4 | 12.2 | 4 | 3 | 3 | 3 | 2.23E+07 | - | - | - | 558 | 63.8 | 8.1 | Metabolism;Energy pathways | - | Catalytic activity | - |
| NLRP13 | NP_789780.2 | 126204 | NACHT, LRR and PYD domains-containing protein 13 | 1.0 | 1 | 1 | 1 | 1 | 4.24E+07 | - | - | - | 1043 | 118.8 | 5.7 | Biological_process unknown | - | Molecular function unknown | - |
| NLRP8 | NP_789781.2 | 126205 | NACHT, LRR and PYD domains-containing protein 8 | 1.4 | 1 | 1 | 1 | 1 | 5.56E+08 | - | - | - | 1048 | 119.4 | 8.0 | Biological_process unknown | - | Molecular function unknown | - |

Supplementary Table S4. List of all proteins identified in SCC-S and SCC-R cells using SequestHT and Mascot search algorithms and their gene ontology annotation

| Gene Symbol | RefSeq_Accession | Entrez Gene ID | Description | Coverage (%) | Proteins | Unique Peptides | # Peptides | PSM | Area | SCC-R /SCC-S | SCC-R /SCC-S (Count) | SCC-R /SCC-S (Variability [%]) | # AAs | MW [kDa] | calc. pI | Biological Process | Localization | Molecular Function | Primary Localization |
|---|---|---|---|---|---|---|---|---|---|---|---|---|---|---|---|---|---|---|---|
| **NOS2** | NP_000616.3 | 4843 | **nitric oxide synthase, inducible** | 1.6 | 1 | 1 | 1 | 1 | 0.00E+00 | **-** | - | - | 1153 | 131 | 8.0 | Metabolism;Energy pathways | Cytoplasm;Plasma membrane | Oxidoreductase activity | Cytoplasm |
| **NOTCH2** | NP_077719.2 | 4853 | **neurogenic locus notch homolog protein 2 isoform 1 preproprotein** | 1.5 | 1 | 1 | 1 | 1 | 2.29E+08 | **-** | - | - | 2471 | 265.2 | 5.1 | Cell communication;Signal transduction | Plasma membrane;Golgi apparatus;Endoplasmic reticulum;Nucleus | Receptor activity | Plasma membrane |
| **NR2C2AP** | NP_795361.1 | 126382 | **nuclear receptor 2C2-associated protein** | 18.7 | 1 | 1 | 1 | 2 | 1.56E+08 | **-** | - | - | 139 | 15.9 | 6.2 | Biological_process unknown | Nucleus | Molecular function unknown | Nucleus |
| **NRAS** | NP_002515.1 | 4893 | **GTPase NRas** | 31.8 | 1 | 1 | 4 | 7 | 1.65E+08 | **-** | - | - | 189 | 21.2 | 5.2 | Cell communication;Signal transduction | Plasma membrane;Golgi apparatus | GTPase activity | Plasma membrane |
| **NRG1** | NP_001153473.1 | 3084 | **pro-neuregulin-1, membrane-bound isoform isoform HRG-beta1d** | 3.7 | 6 | 1 | 1 | 1 | 3.23E+07 | **-** | - | - | 590 | 65.4 | 8.5 | Cell communication;Signal transduction | Extracellular;Plasma membrane;Cytoplasm | Growth factor activity | Extracellular |
| **NRG2** | NP_001171864.1 | 9542 | **pro-neuregulin-2, membrane-bound isoform isoform 7** | 4.1 | 5 | 1 | 1 | 1 | 1.84E+09 | **-** | - | - | 784 | 84.3 | 9.4 | Signal transduction;Cell growth and/or maintenance | Integral to membrane | Growth factor activity | Integral to membrane |
| **NRP1** | NP_001231902.1 | 8829 | **neuropilin-1 isoform e precursor** | 7.1 | 5 | 3 | 3 | 4 | 2.55E+07 | **-** | - | - | 916 | 102.3 | 6.0 | Signal transduction;Cell migration;Morphogenesis;Immune response | Plasma membrane;Extracellular | Receptor activity | Plasma membrane |
| **NSRP1** | NP_001248396.1 | 84081 | **nuclear speckle splicing regulatory protein 1 isoform 2** | 11.7 | 2 | 4 | 4 | 4 | 4.32E+07 | **-** | - | - | 504 | 60.4 | 8.9 | Biological_process unknown | Nucleus;Cytoplasm | Molecular function unknown | Nucleus;Cytoplasm |
| **NSUN2** | NP_060225.4 | 54888 | **tRNA (cytosine(34)-C(5))-methyltransferase isoform 1** | 47.2 | 1 | 1 | 22 | 60 | 5.43E+08 | **-** | - | - | 767 | 86.4 | 6.8 | - | Nucleolus;Cytoplasm;Nucleus | RNA methyltransferase activity | Nucleolus |
| **NSUN4** | NP_001243056.1 | 387338 | **5-methylcytosine rRNA methyltransferase NSUN4 isoform b** | 10.2 | 2 | 1 | 1 | 1 | 7.52E+07 | **-** | - | - | 335 | 37.3 | 6.5 | Biological_process unknown | - | Molecular function unknown | - |
| **NT5C** | NP_001239306.1 | 30833 | **5'(3')-deoxyribonucleotidase, cytosolic type isoform 2** | 7.5 | 2 | 1 | 1 | 1 | 2.60E+07 | **-** | - | - | 161 | 18.7 | 6.8 | Metabolism;Energy pathways | Cytoplasm;Nucleus | Hydrolase activity | Cytoplasm |
| **NTRK2** | NP_001018074.1 | 4915 | **BDNF/NT-3 growth factors receptor isoform c precursor** | 0.7 | 2 | 1 | 1 | 1 | 3.51E+07 | **-** | - | - | 822 | 91.9 | 6.5 | Cell communication;Signal transduction | Integral to membrane | Transmembrane receptor protein tyrosine kinase activity | Integral to membrane |
| **NUDCD2** | NP_660309.1 | 134492 | **nudC domain-containing protein 2** | 5.7 | 1 | 1 | 1 | 1 | 0.00E+00 | **-** | - | - | 157 | 17.7 | 5.1 | Biological_process unknown | - | Molecular function unknown | - |
| **NUDT15** | NP_060753.1 | 55270 | **probable 8-oxo-dGTP diphosphatase NUDT15** | 11.0 | 1 | 2 | 2 | 2 | 8.01E+06 | **-** | - | - | 164 | 18.6 | 6.1 | Metabolism;Energy pathways | Nucleus | Hydrolase activity | Nucleus |
| **NUPL2** | NP_031368.1 | 11097 | **nucleoporin-like protein 2** | 5.0 | 1 | 1 | 1 | 1 | 4.37E+06 | **-** | - | - | 423 | 44.8 | 9.2 | Transport | Nuclear membrane | Transporter activity | Nuclear membrane |
| **NUPR1** | NP_036517.1 | 26471 | **nuclear protein 1 isoform b** | 19.5 | 2 | 1 | 1 | 2 | 1.13E+07 | **-** | - | - | 82 | 8.9 | 10.0 | Cell growth and/or maintenance | Nucleus | DNA binding | Nucleus |
| **NXT1** | NP_037380.1 | 29107 | **NTF2-related export protein 1** | 14.3 | 1 | 1 | 1 | 2 | 1.61E+07 | **-** | - | - | 140 | 15.8 | 5.0 | Transport | Nucleus;Cytoplasm | Transporter activity | Nucleus |
| **OAS1** | NP_002525.2 | 4938 | **2'-5'-oligoadenylate synthase 1 isoform 2** | 6.3 | 3 | 1 | 1 | 1 | 0.00E+00 | **-** | - | - | 364 | 41.7 | 8.9 | Immune response | Cytoplasm;Nucleus | Ligase activity | Cytoplasm |
| **OAS2** | NP_002526.2 | 4939 | **2'-5'-oligoadenylate synthase 2 isoform 2** | 34.9 | 3 | 19 | 19 | 30 | 3.40E+08 | **-** | - | - | 687 | 78.7 | 8.0 | Immune response | Cytoplasm;Nucleus;Plasma membrane;Endoplasmic reticulum;Nucleolus | Ligase activity | Cytoplasm |
| **OASL** | NP_003724.1 | 8638 | **2'-5'-oligoadenylate synthase-like protein isoform a** | 27.8 | 3 | 9 | 9 | 14 | 1.10E+08 | **-** | - | - | 514 | 59.2 | 7.9 | Regulation of nucleobase, nucleoside, nucleotide and nucleic acid metabolism | Nucleolus;Cytoplasm;Nucleus | Transcription regulator activity | Nucleus |
| **OGDHL** | NP_060715.2 | 55753 | **2-oxoglutarate dehydrogenase-like, mitochondrial isoform a** | 8.1 | 3 | 1 | 5 | 5 | 8.79E+07 | **-** | - | - | 1010 | 114.4 | 6.7 | Biological_process unknown | - | Molecular function unknown | - |
| **OGN** | NP_148935.1 | 4969 | **mimecan preproprotein** | 5.4 | 1 | 1 | 1 | 2 | 5.61E+06 | **-** | - | - | 298 | 33.9 | 5.6 | Organogenesis;Cell communication;Signal transduction | Extracellular | Growth factor activity | Extracellular |
| **OLFML2B** | NP_056256.1 | 25903 | **olfactomedin-like protein 2B precursor** | 2.4 | 1 | 1 | 1 | 1 | 0.00E+00 | **-** | - | - | 750 | 83.9 | 5.2 | Biological_process unknown | Extracellular | Molecular function unknown | Extracellular |
| **OLR1** | NP_002534.1 | 4973 | **oxidized low-density lipoprotein receptor 1 isoform 1** | 14.7 | 3 | 2 | 2 | 3 | 2.63E+07 | **-** | - | - | 273 | 30.9 | 7.3 | Transport | Plasma membrane | Receptor activity | Plasma membrane |
| **OOEP** | NP_001073976.1 | 441161 | **oocyte-expressed protein homolog** | 8.1 | 1 | 1 | 1 | 1 | 8.99E+07 | **-** | - | - | 149 | 17.2 | 7.1 | Biological_process unknown | - | Molecular function unknown | - |
| **OR5AC2** | NP_473447.1 | 81050 | **olfactory receptor 5AC2** | 2.3 | 1 | 1 | 1 | 5 | 1.08E+08 | **-** | - | - | 309 | 35.3 | 8.9 | Cell communication;Signal transduction | Integral to membrane | G-protein coupled receptor activity | Integral to membrane |
| **ORMDL2** | NP_054901.1 | 29095 | **ORM1-like protein 2** | 9.8 | 2 | 1 | 1 | 3 | 1.83E+08 | **-** | - | - | 153 | 17.4 | 9.6 | Biological_process unknown | Endoplasmic reticulum | Molecular function unknown | Endoplasmic reticulum |
| **ORMDL3** | NP_644809.1 | 94103 | **ORM1-like protein 3** | 9.8 | 1 | 1 | 1 | 1 | 3.41E+07 | **-** | - | - | 153 | 17.5 | 9.6 | Protein metabolism | Endoplasmic reticulum | Molecular function unknown | Endoplasmic reticulum |
| **OSBP2** | NP_001269670.1 | 23762 | **oxysterol-binding protein 2 isoform f** | 6.1 | 6 | 1 | 3 | 5 | 3.32E+07 | **-** | - | - | 659 | 74.6 | 6.5 | Transport | Plasma membrane;Cytoplasm | Transporter activity | Cytoplasm |
| **OSBPL3** | NP_663162.1 | 26031 | **oxysterol-binding protein-related protein 3 isoform d** | 3.5 | 4 | 2 | 2 | 2 | 1.46E+07 | **-** | - | - | 820 | 93.7 | 6.5 | Steroid metabolism | Golgi apparatus;Cytoplasm;Plasma membrane;Nucleus;Endoplasmic reticulum membrane | Steroid binding | Endoplasmic reticulum membrane |
| **OSBPL9** | NP_683704.2 | 114883 | **oxysterol-binding protein-related protein 9 isoform b** | 13.6 | 6 | 3 | 3 | 3 | 6.02E+07 | **-** | - | - | 719 | 81.1 | 5.8 | Transport | Cytoplasm;Nucleus | Transporter activity | Cytoplasm;Nucleus |
| **OSTC** | NP_001254746.1 | 58505 | **oligosaccharyltransferase complex subunit OSTC isoform 2** | 14.5 | 3 | 1 | 1 | 2 | 2.95E+08 | **-** | - | - | 83 | 9.4 | 7.4 | Biological_process unknown | Integral to membrane | Molecular function unknown | Integral to membrane |
| **OTC** | NP_000522.3 | 5009 | **ornithine carbamoyltransferase, mitochondrial precursor** | 2.0 | 1 | 1 | 1 | 1 | 2.20E+08 | **-** | - | - | 354 | 39.9 | 8.6 | Metabolism;Energy pathways | Mitochondrion;Cytoplasm | Carboxyl- and carbamoyltransferase activity | Mitochondrion |
| **OTOF** | NP_919224.1 | 9381 | **otoferlin isoform a** | 0.4 | 2 | 1 | 1 | 2 | 1.10E+08 | **-** | - | - | 1997 | 226.6 | 5.7 | Cell communication;Signal transduction;Vesicle-mediated transport | Integral to membrane;Cytoplasm | Calcium ion binding | Cytoplasm |
| **OXLD1** | NP_001034931.1 | 339229 | **oxidoreductase-like domain-containing protein 1** | 10.2 | 1 | 1 | 1 | 1 | 2.78E+07 | **-** | - | - | 147 | 15.8 | 8.2 | Biological_process unknown | - | Molecular function unknown | - |
| **P4HA3** | NP_001275677.1 | 283208 | **prolyl 4-hydroxylase subunit alpha-3 isoform 2 precursor** | 3.3 | 1 | 1 | 1 | 1 | 5.33E+08 | **-** | - | - | 604 | 67.5 | 7.2 | Metabolism;Energy pathways | - | Oxidoreductase activity | - |
| **P4HTM** | NP_808808.1 | 54681 | **transmembrane prolyl 4-hydroxylase isoform a** | 5.0 | 2 | 1 | 1 | 1 | 1.28E+07 | **-** | - | - | 502 | 56.6 | 6.1 | Metabolism;Energy pathways | Endoplasmic reticulum | Catalytic activity | Endoplasmic reticulum |

| Gene Symbol | RefSeq_Accession | Entrez Gene ID | Description | Coverage (%) | Proteins | Unique Peptides | # Peptides | PSM | Area | SCC-R /SCC-S | SCC-R /SCC-S (Count) | SCC-R /SCC-S (Variability [%]) | # AAs | MW [kDa] | calc. pI | Biological Process | Localization | Molecular Function | Primary Localization |
|---|---|---|---|---|---|---|---|---|---|---|---|---|---|---|---|---|---|---|---|
| **PABPC3** | NP_112241.2 | 5042 | **polyadenylate-binding protein 3** | 25.5 | 1 | 1 | 17 | 100 | 2.03E+09 | - | - | - | 631 | 70 | 9.7 | Regulation of nucleobase, nucleoside, nucleotide and nucleic acid metabolism | Cytoplasm;Nucleus | RNA binding | Nucleus |
| **PALLD** | NP_001159582.1 | 23022 | **palladin isoform 4** | 26.5 | 1 | 1 | 9 | 16 | 2.35E+08 | - | - | - | 672 | 73.3 | 7.7 | Cell growth and/or maintenance | Cytoplasm;Nucleus;Actin filament | Molecular function unknown | Cytoplasm |
| **PALLD** | NP_001159581.1 | 23022 | **palladin isoform 3** | 21.9 | 3 | 1 | 9 | 15 | 2.35E+08 | - | - | - | 777 | 85.8 | 6.9 | Cell growth and/or maintenance | Cytoplasm;Nucleus;Actin filament | Molecular function unknown | Cytoplasm |
| **PARG** | NP_003622.2 | 8505 | **poly(ADP-ribose) glycohydrolase** | 1.3 | 1 | 1 | 1 | 1 | 4.23E+06 | - | - | - | 976 | 111 | 6.4 | Metabolism;Energy pathways | Cytoplasm;Nucleus;Centrosome | Hydrolase activity | Cytoplasm |
| **PARN** | NP_001127949.1 | 5073 | **poly(A)-specific ribonuclease PARN isoform 2** | 2.9 | 3 | 1 | 1 | 1 | 6.04E+06 | - | - | - | 578 | 66.5 | 6.4 | Regulation of nucleobase, nucleoside, nucleotide and nucleic acid metabolism | Cytoplasm;Nucleus;Nucleolus | Ribonuclease activity | Cytoplasm |
| **PATZ1** | NP_114440.1 | 23598 | **POZ-, AT hook-, and zinc finger-containing protein 1 short isoform** | 4.5 | 4 | 2 | 2 | 2 | 2.74E+07 | - | - | - | 537 | 57.6 | 8.5 | Regulation of nucleobase, nucleoside, nucleotide and nucleic acid metabolism | Nucleus | Transcription regulator activity | Nucleus |
| **PBX1** | NP_001191890.1 | 5087 | **pre-B-cell leukemia transcription factor 1 isoform 2** | 8.7 | 3 | 2 | 2 | 2 | 8.59E+07 | - | - | - | 347 | 38.4 | 8.4 | Regulation of nucleobase, nucleoside, nucleotide and nucleic acid metabolism | Nucleus | Transcription regulator activity | Nucleus |
| **PCBP2** | NP_001122383.1 | 5094 | **poly(rC)-binding protein 2 isoform d** | 75.3 | 4 | 0 | 14 | 197 | 3.68E+09 | - | - | - | 365 | 38.6 | 6.8 | Regulation of nucleobase, nucleoside, nucleotide and nucleic acid metabolism | Nucleus;Cytoplasm | RNA binding;DNA binding | Nucleus |
| **PCBP2** | NP_001122384.1 | 5094 | **poly(rC)-binding protein 2 isoform e** | 75.1 | 6 | 1 | 14 | 201 | 3.68E+09 | - | - | - | 361 | 38.1 | 6.8 | Regulation of nucleobase, nucleoside, nucleotide and nucleic acid metabolism | Nucleus;Cytoplasm | RNA binding;DNA binding | Nucleus |
| **PCF11** | NP_056969.2 | 51585 | **pre-mRNA cleavage complex 2 protein Pcf11** | 1.9 | 1 | 2 | 2 | 6 | 2.90E+07 | - | - | - | 1555 | 172.9 | 8.5 | Regulation of nucleobase, nucleoside, nucleotide and nucleic acid metabolism | Nucleus | RNA binding | Nucleus |
| **PCMTD1** | NP_001273712.1 | 115294 | **protein-L-isoaspartate O-methyltransferase domain-containing protein 1 isoform 3** | 4.4 | 3 | 1 | 1 | 1 | 0.00E+00 | - | - | - | 181 | 21 | 9.1 | Biological_process unknown | - | Molecular function unknown | - |
| **PCNXL3** | NP_115599.2 | 399909 | **pecanex-like protein 3** | 1.2 | 1 | 1 | 1 | 1 | 6.19E+06 | - | - | - | 2034 | 221.9 | 6.6 | Biological_process unknown | - | Molecular function unknown | - |
| **PDE1B** | NP_000915.1 | 5153 | **calcium/calmodulin-dependent 3',5'-cyclic nucleotide phosphodiesterase 1B isoform 1** | 3.9 | 1 | 1 | 1 | 1 | 6.54E+08 | - | - | - | 536 | 61.3 | 5.5 | Cell communication;Signal transduction | Cytoplasm | Phosphoric diester hydrolase activity | Cytoplasm |
| **PDE4DIP** | NP_001185763.3 | 9659 | **myomegalin isoform 9** | 1.1 | 8 | 2 | 2 | 3 | 3.35E+08 | - | - | - | 2362 | 267 | 5.5 | Transport | Golgi apparatus;Centrosome;Cytoplasm;Nucleus | Transporter activity | Golgi apparatus |
| **PDE5A** | NP_246273.2 | 8654 | **cGMP-specific 3',5'-cyclic phosphodiesterase isoform 3** | 2.9 | 3 | 1 | 1 | 1 | 1.08E+07 | - | - | - | 823 | 93.6 | 6.1 | Cell communication;Signal transduction | Nucleus;Cytoplasm | Phosphoric diester hydrolase activity | Nucleus |
| **PDE6B** | NP_001138764.1 | 5158 | **rod cGMP-specific 3',5'-cyclic phosphodiesterase subunit beta isoform 3** | 1.2 | 3 | 1 | 1 | 1 | 3.98E+07 | - | - | - | 575 | 66.5 | 5.4 | Metabolism;Energy pathways | Plasma membrane | Hydrolase activity | Plasma membrane |
| **PDE9A** | NP_001001576.1 | 5152 | **high affinity cGMP-specific 3',5'-cyclic phosphodiesterase 9A isoform j** | 2.7 | 15 | 1 | 1 | 1 | 5.72E+08 | - | - | - | 376 | 44.1 | 5.6 | CGMP-mediated signaling | - | Phosphoric diester hydrolase activity | - |
| **PDIA2** | NP_006840.2 | 64714 | **protein disulfide-isomerase A2 precursor** | 1.5 | 1 | 1 | 1 | 2 | 1.17E+08 | - | - | - | 525 | 58.2 | 5.0 | Metabolism;Energy pathways | Endoplasmic reticulum | Isomerase activity | Endoplasmic reticulum |
| **PDIA5** | NP_006801.1 | 10954 | **protein disulfide-isomerase A5 precursor** | 5.8 | 1 | 2 | 2 | 2 | 1.98E+07 | - | - | - | 519 | 59.6 | 7.9 | Protein metabolism | Endoplasmic reticulum | Chaperone activity | Endoplasmic reticulum |
| **PDLIM3** | NP_001244892.1 | 27295 | **PDZ and LIM domain protein 3 isoform d** | 9.1 | 4 | 1 | 1 | 1 | 1.29E+07 | - | - | - | 197 | 21.7 | 8.7 | Cell growth and/or maintenance | Cytoplasm | Molecular function unknown | Cytoplasm |
| **PDXP** | NP_064711.1 | 57026 | **pyridoxal phosphate phosphatase** | 8.8 | 1 | 1 | 1 | 1 | 1.32E+07 | - | - | - | 296 | 31.7 | 6.6 | Metabolism;Energy pathways | - | Acid phosphatase activity | - |
| **PDZD11** | NP_057568.1 | 51248 | **PDZ domain-containing protein 11** | 35.7 | 1 | 2 | 2 | 2 | 2.93E+07 | - | - | - | 140 | 16.1 | 7.2 | Biological_process unknown | - | Molecular function unknown | - |
| **PDZD8** | NP_776152.1 | 118987 | **PDZ domain-containing protein 8** | 1.0 | 1 | 1 | 1 | 1 | 2.86E+07 | - | - | - | 1154 | 128.5 | 6.1 | Biological_process unknown | Nucleus | Molecular function unknown | Nucleus |
| **PEAR1** | NP_001073940.1 | 375033 | **platelet endothelial aggregation receptor 1 precursor** | 1.5 | 1 | 1 | 1 | 1 | 2.21E+07 | - | - | - | 1037 | 110.6 | 6.8 | Signal transduction | - | Receptor signaling protein activity | - |
| **PELI1** | NP_065702.2 | 57162 | **E3 ubiquitin-protein ligase pellino homolog 1** | 5.7 | 1 | 1 | 1 | 1 | 0.00E+00 | - | - | - | 418 | 46.3 | 8.0 | Cell communication;Signal transduction | - | Receptor signaling complex scaffold activity | - |
| **PEX11B** | NP_001171724.1 | 8799 | **peroxisomal membrane protein 11B isoform 2** | 4.9 | 2 | 1 | 1 | 1 | 7.85E+06 | - | - | - | 245 | 26.7 | 9.9 | Cell communication;Signal transduction | Peroxisome | Molecular function unknown | Peroxisome |
| **PEX12** | NP_000277.1 | 5193 | **peroxisome assembly protein 12** | 2.2 | 1 | 1 | 1 | 1 | 0.00E+00 | - | - | - | 359 | 40.8 | 9.1 | Cell communication;Signal transduction | Peroxisome | Protein binding | Peroxisome |
| **PEX13** | NP_002609.1 | 5194 | **peroxisome biogenesis factor 13** | 2.5 | 1 | 1 | 1 | 1 | 2.22E+06 | - | - | - | 403 | 44.1 | 8.1 | Cell communication;Signal transduction | Peroxisome | Protein binding | Peroxisome |
| **PEX5** | NP_001124496.1 | 5830 | **peroxisomal biogenesis factor 5 isoform c** | 5.3 | 4 | 2 | 2 | 4 | 1.38E+07 | - | - | - | 602 | 66.8 | 4.5 | Cell communication;Signal transduction | Cytoplasm;Peroxisome | Protein binding | Cytoplasm |
| **PEX5L** | NP_001243681.1 | 51555 | **PEX5-related protein isoform 4** | 3.4 | 2 | 1 | 1 | 1 | 0.00E+00 | - | - | - | 591 | 65.9 | 5.3 | Biological_process unknown | Cytoplasm | Molecular function unknown | Cytoplasm |
| **PFKFB2** | NP_001018063.1 | 5208 | **6-phosphofructo-2-kinase/fructose-2, 6-bisphosphatase 2 isoform b** | 2.3 | 2 | 1 | 1 | 1 | 0.00E+00 | - | - | - | 471 | 54.4 | 7.6 | Metabolism;Energy pathways | Mitochondrion | Catalytic activity | Mitochondrion |
| **PHACTR4** | NP_001041648.1 | 65979 | **phosphatase and actin regulator 4 isoform 1** | 5.1 | 2 | 2 | 2 | 2 | 1.40E+07 | - | - | - | 702 | 78.2 | 6.6 | Signal transduction | Perinuclear region;Synapse;Cytoplasm | Phosphatase regulator activity | Perinuclear region |
| **PHAX** | NP_115553.2 | 51808 | **phosphorylated adapter RNA export protein** | 2.8 | 1 | 1 | 1 | 7 | 6.22E+07 | - | - | - | 394 | 44.4 | 5.4 | Regulation of nucleobase, nucleoside, nucleotide and nucleic acid metabolism | Nucleus;Cytoplasm | RNA binding | Nucleus |
| **PHC2** | NP_004418.2 | 1912 | **polyhomeotic-like protein 2 isoform b** | 4.0 | 2 | 1 | 1 | 1 | 3.15E+07 | - | - | - | 323 | 35.7 | 8.6 | Protein metabolism | Nucleus | Ubiquitin-specific protease activity | Nucleus |
| **PHC3** | NP_079223.3 | 80012 | **polyhomeotic-like protein 3** | 1.3 | 1 | 1 | 1 | 2 | 4.62E+06 | - | - | - | 995 | 107.4 | 6.6 | Biological_process unknown | Nucleus;Cytoplasm | Molecular function unknown | Nucleus |
| **PHF23** | NP_001271446.1 | 79142 | **PHD finger protein 23 isoform 2** | 8.0 | 2 | 1 | 1 | 1 | 5.29E+06 | - | - | - | 336 | 36.7 | 5.4 | Regulation of nucleobase, nucleoside, nucleotide and nucleic acid metabolism | - | Transcription regulator activity | - |
| **PHF8** | NP_001171827.1 | 23133 | **histone lysine demethylase PHF8 isoform 4** | 5.2 | 4 | 3 | 3 | 3 | 2.62E+07 | - | - | - | 878 | 98.2 | 6.9 | Regulation of nucleobase, nucleoside, nucleotide and nucleic acid metabolism | Nucleus | DNA binding | Nucleus |

| Gene Symbol | RefSeq_Accession | Entrez Gene ID | Description | Coverage (%) | Proteins | Unique Peptides | # Peptides | PSM | Area | SCC-R /SCC-S | SCC-R /SCC-S (Count) | SCC-R /SCC-S (Variability [%]) | # AAs | MW [kDa] | calc. pI | Biological Process | Localization | Molecular Function | Primary Localization |
|---|---|---|---|---|---|---|---|---|---|---|---|---|---|---|---|---|---|---|---|
| PIGR | NP_002635.2 | 5284 | polymeric immunoglobulin receptor precursor | 2.1 | 1 | 1 | 1 | 1 | 2.21E+06 | - | - | - | 764 | 83.2 | 5.7 | Immune response | Plasma membrane;Extracellular | Receptor activity | Plasma membrane |
| PIGT | NP_001171659.1 | 51604 | GPI transamidase component PIG-T isoform 4 precursor | 8.6 | 4 | 3 | 3 | 4 | 1.58E+07 | - | - | - | 476 | 54.1 | 8.2 | Protein metabolism | Endoplasmic reticulum | Transaminase activity | Endoplasmic reticulum |
| PIK3R2 | NP_005018.1 | 5296 | phosphatidylinositol 3-kinase regulatory subunit beta | 4.0 | 1 | 1 | 1 | 2 | 4.26E+07 | - | - | - | 728 | 81.6 | 6.5 | Cell communication;Signal transduction | Plasma membrane;Cytoplasm | Kinase regulator activity | Cytoplasm |
| PIK3R4 | NP_055417.1 | 30849 | phosphoinositide 3-kinase regulatory subunit 4 | 1.6 | 1 | 1 | 1 | 1 | 8.01E+06 | - | - | - | 1358 | 153 | 7.2 | Protein metabolism | - | Receptor signaling complex scaffold activity | - |
| PINX1 | NP_060354.4 | 54984 | PIN2/TERF1-interacting telomerase inhibitor 1 isoform 1 | 5.8 | 1 | 1 | 1 | 1 | 2.23E+07 | - | - | - | 328 | 37 | 9.6 | Biological_process unknown | Cytoplasm;Nucleus;Nucleolus | Molecular function unknown | Nucleus |
| PJA2 | NP_055634.3 | 9867 | E3 ubiquitin-protein ligase Praja-2 | 2.5 | 1 | 1 | 1 | 1 | 4.75E+07 | - | - | - | 708 | 78.2 | 4.4 | Protein metabolism | Endoplasmic reticulum;Golgi apparatus | Ubiquitin-specific protease activity | Endoplasmic reticulum |
| PKDREJ | NP_006062.1 | 10343 | polycystic kidney disease and receptor for egg jelly-related protein precursor | 0.4 | 1 | 1 | 1 | 1 | 3.32E+07 | - | - | - | 2253 | 255.3 | 9.1 | Transport | Plasma membrane | Ion channel activity | Plasma membrane |
| PKP1 | NP_001005337.1 | 5317 | plakophilin-1 isoform 1a | 20.1 | 2 | 9 | 9 | 10 | 1.59E+08 | - | - | - | 726 | 80.4 | 9.0 | Intercellular junction assembly and/or maintenance | Nucleus;Desmosome | Cell adhesion molecule activity | Nucleus |
| PKP4 | NP_001005476.1 | 8502 | plakophilin-4 isoform b | 2.8 | 2 | 2 | 2 | 2 | 6.49E+06 | - | - | - | 1149 | 127.1 | 9.0 | Cell growth and/or maintenance | Plasma membrane;Cytoplasm;Desmosome;Nucleus | Cell adhesion molecule activity | Plasma membrane;Nucleus;Cytoplasm |
| PLAUR | NP_002650.1 | 5329 | urokinase plasminogen activator surface receptor isoform 1 precursor | 26.3 | 3 | 5 | 5 | 9 | 8.44E+07 | - | - | - | 335 | 37 | 6.7 | Cell communication;Signal transduction | Plasma membrane | Receptor activity | Plasma membrane |
| PLBD2 | NP_775813.2 | 196463 | putative phospholipase B-like 2 isoform 1 precursor | 8.0 | 2 | 2 | 2 | 5 | 2.90E+07 | - | - | - | 589 | 65.4 | 6.8 | Biological_process unknown | Lysosome | Molecular function unknown | Lysosome |
| PLEKHO2 | NP_079477.2 | 80301 | pleckstrin homology domain-containing family O member 2 isoform 1 | 5.5 | 2 | 2 | 2 | 2 | 3.06E+07 | - | - | - | 490 | 53.3 | 5.4 | Biological_process unknown | - | Molecular function unknown | - |
| PLGRKT | NP_060935.2 | 55848 | plasminogen receptor (KT) | 10.2 | 1 | 1 | 1 | 1 | 8.21E+07 | - | - | - | 147 | 17.2 | 9.6 | Biological_process unknown | Integral to membrane | Molecular function unknown | Integral to membrane |
| PLIN4 | NP_001073869.1 | 729359 | perilipin-4 | 2.4 | 1 | 1 | 1 | 1 | 1.12E+09 | - | - | - | 1357 | 134.3 | 8.7 | Biological_process unknown | Cytoplasm | Molecular function unknown | Cytoplasm |
| PLP2 | NP_002659.1 | 5355 | proteolipid protein 2 | 8.6 | 1 | 1 | 1 | 9 | 7.12E+09 | - | - | - | 152 | 16.7 | 7.2 | Transport | Endoplasmic reticulum | Ion channel activity | Endoplasmic reticulum |
| PLXNA2 | NP_079455.3 | 5362 | plexin-A2 precursor | 1.5 | 1 | 1 | 1 | 1 | 4.21E+07 | - | - | - | 1894 | 211 | 6.5 | Cell communication;Signal transduction | Plasma membrane | Receptor activity | Plasma membrane |
| PML | NP_150242.1 | 5371 | protein PML isoform 9 | 32.7 | 6 | 1 | 16 | 25 | 4.32E+08 | - | - | - | 829 | 90.7 | 6.9 | Regulation of gene expression, epigenetic | Nucleus;Cytoplasm;Nucleolus;Centrosome;Nuclear matrix | Transcription regulator activity;Transporter activity | Nucleus;Cytoplasm |
| PNISR | NP_116259.2 | 25957 | arginine/serine-rich protein PNISR | 1.5 | 1 | 1 | 1 | 1 | 8.38E+06 | - | - | - | 805 | 92.5 | 10.0 | Biological_process unknown | - | Molecular function unknown | - |
| PNPLA2 | NP_065109.1 | 57104 | patatin-like phospholipase domain-containing protein 2 | 3.2 | 1 | 1 | 1 | 1 | 1.45E+07 | - | - | - | 504 | 55.3 | 7.1 | Metabolism;Energy pathways | Cytoplasm | Hydrolase activity | Cytoplasm |
| POC1A | NP_001155052.1 | 25886 | POC1 centriolar protein homolog A isoform 2 | 2.2 | 3 | 1 | 1 | 1 | 3.40E+08 | - | - | - | 359 | 39.8 | 8.2 | Cell communication;Signal transduction | - | Molecular function unknown | - |
| POFUT2 | NP_056042.1 | 23275 | GDP-fucose protein O-fucosyltransferase 2 isoform A precursor | 3.3 | 2 | 1 | 1 | 2 | 4.60E+07 | - | - | - | 424 | 48.9 | 6.8 | Development | Cytoplasm;Golgi apparatus | Fucosyltransferase activity | Cytoplasm |
| POGLUT1 | NP_689518.1 | 56983 | protein O-glucosyltransferase 1 precursor | 3.1 | 1 | 1 | 1 | 3 | 2.45E+07 | - | - | - | 392 | 46.2 | 8.7 | Biological_process unknown | Extracellular | Molecular function unknown | Extracellular |
| POLA1 | NP_058633.2 | 5422 | DNA polymerase alpha catalytic subunit | 0.9 | 1 | 1 | 1 | 1 | 8.81E+06 | - | - | - | 1462 | 165.8 | 5.9 | Regulation of nucleobase, nucleoside, nucleotide and nucleic acid metabolism | Nucleus | DNA-directed DNA polymerase activity | Nucleus |
| POLDIP2 | NP_056399.1 | 26073 | polymerase delta-interacting protein 2 isoform 1 | 13.9 | 2 | 4 | 4 | 5 | 3.78E+07 | - | - | - | 368 | 42 | 8.6 | Biological_process unknown | Nucleus | Molecular function unknown | Nucleus |
| POLR2G | NP_002687.1 | 5436 | DNA-directed RNA polymerase II subunit RPB7 | 7.0 | 1 | 1 | 1 | 1 | 4.34E+06 | - | - | - | 172 | 19.3 | 5.5 | Regulation of nucleobase, nucleoside, nucleotide and nucleic acid metabolism | Nucleus | DNA-directed RNA polymerase activity | Nucleus |
| POLR2J3 | NP_001091084.2 | 548644 | DNA-directed RNA polymerase II subunit RPB11-b2 | 33.0 | 3 | 2 | 2 | 2 | 2.85E+06 | - | - | - | 115 | 13.1 | 6.3 | Biological_process unknown | - | Molecular function unknown | - |
| POLR2L | NP_066951.1 | 5441 | DNA-directed RNA polymerases I, II, and III subunit RPABC5 | 44.8 | 1 | 1 | 1 | 2 | 8.48E+06 | - | - | - | 67 | 7.6 | 7.8 | Regulation of nucleobase, nucleoside, nucleotide and nucleic acid metabolism | Nucleus | DNA-directed RNA polymerase activity | Nucleus |
| POLR3K | NP_057394.3 | 51728 | DNA-directed RNA polymerase III subunit RPC10 | 24.1 | 1 | 1 | 1 | 1 | 9.06E+06 | - | - | - | 108 | 12.3 | 7.8 | Regulation of nucleobase, nucleoside, nucleotide and nucleic acid metabolism | Nucleus | DNA-directed RNA polymerase activity | Nucleus |
| POLRMT | NP_005026.3 | 5442 | DNA-directed RNA polymerase, mitochondrial precursor | 3.2 | 1 | 2 | 2 | 2 | 1.71E+07 | - | - | - | 1230 | 138.5 | 9.0 | Regulation of nucleobase, nucleoside, nucleotide and nucleic acid metabolism | Mitochondrion | DNA-directed RNA polymerase activity | Mitochondrion |
| POP5 | NP_937845.1 | 51367 | ribonuclease P/MRP protein subunit POP5 isoform c | 6.2 | 2 | 1 | 1 | 1 | 2.17E+07 | - | - | - | 113 | 12.8 | 5.3 | Regulation of nucleobase, nucleoside, nucleotide and nucleic acid metabolism | Nucleolus;Nucleus;Cytoplasm | Ribonuclease activity | Nucleolus;Cytoplasm |
| PPEF2 | NP_006230.2 | 5470 | serine/threonine-protein phosphatase with EF-hands 2 | 1.3 | 1 | 1 | 1 | 1 | 1.16E+08 | - | - | - | 753 | 86.5 | 7.1 | Cell communication;Signal transduction | - | Protein serine/threonine phosphatase activity | - |
| PPHLN1 | NP_001137261.1 | 51535 | periphilin-1 isoform 8 | 12.0 | 5 | 1 | 3 | 4 | 7.03E+07 | - | - | - | 293 | 33.9 | 6.6 | Cell growth and/or maintenance | Nucleus;Plasma membrane;Cytoplasm | Structural molecule activity | Nucleus;Cytoplasm |
| PPHLN1 | NP_001137259.1 | 51535 | periphilin-1 isoform 6 | 9.5 | 2 | 1 | 3 | 3 | 2.30E+07 | - | - | - | 434 | 49.6 | 8.7 | Cell growth and/or maintenance | Nucleus;Plasma membrane;Cytoplasm | Structural molecule activity | Nucleus;Cytoplasm |
| PPIAL4G | NP_001116540.1 | 644591 | peptidyl-prolyl cis-trans isomerase A-like 4G | 26.2 | 4 | 1 | 2 | 29 | 1.69E+09 | - | - | - | 164 | 18.2 | 9.1 | Protein folding | - | Isomerase activity | - |
| PPIH | NP_006338.1 | 10465 | peptidyl-prolyl cis-trans isomerase H | 20.3 | 1 | 2 | 2 | 7 | 1.34E+08 | - | - | - | 177 | 19.2 | 8.1 | Protein metabolism | Nucleus;Cytoplasm;Nucleolus | Chaperone activity | Nucleus |
| PPOX | NP_000300.1 | 5498 | protoporphyrinogen oxidase | 6.5 | 1 | 1 | 1 | 1 | 6.16E+06 | - | - | - | 477 | 50.7 | 8.2 | Metabolism;Energy pathways | Mitochondrion | Catalytic activity | Mitochondrion |
| PPP1R11 | NP_068778.1 | 6992 | protein phosphatase 1 regulatory subunit 11 | 21.4 | 1 | 2 | 2 | 3 | 5.58E+07 | - | - | - | 126 | 13.9 | 7.0 | Biological_process unknown | Cytoplasm | Enzyme inhibitor activity | Cytoplasm |

Jain *et al.* , 2019. MAP2K1 is a potential therapeutic target in erlotinib resistant head and neck squamous cell carcinoma
Supplementary Table S4. List of all proteins identified in SCC-S and SCC-R cells using SequestHT and Mascot search algorithms and their gene ontology annotation

| Gene Symbol | RefSeq_Accession | Entrez Gene ID | Description | Coverage (%) | Proteins | Unique Peptides | # Peptides | PSM | Area | SCC-R /SCC-S | SCC-R /SCC-S (Count) | SCC-R /SCC-S (Variability [%]) | # AAs | MW [kDa] | calc. pI | Biological Process | Localization | Molecular Function | Primary Localization |
|---|---|---|---|---|---|---|---|---|---|---|---|---|---|---|---|---|---|---|---|
| PPP1R36 | NP_758953.1 | 145376 | protein phosphatase 1 regulatory subunit 36 | 2.8 | 1 | 1 | 1 | 1 | 1.95E+08 | - | - | - | 422 | 49.4 | 9.4 | Biological_process unknown | - | Molecular function unknown | - |
| PPP4R4 | NP_478144.1 | 57718 | serine/threonine-protein phosphatase 4 regulatory subunit 4 isoform 1 | 0.7 | 1 | 1 | 1 | 3 | 6.34E+08 | - | - | - | 873 | 99.4 | 7.8 | Biological_process unknown | Cytoplasm | Molecular function unknown | Cytoplasm |
| PPP6R1 | NP_055746.3 | 22870 | serine/threonine-protein phosphatase 6 regulatory subunit 1 | 3.6 | 1 | 1 | 1 | 1 | 9.39E+06 | - | - | - | 881 | 96.7 | 4.6 | Biological_process unknown | Cytoplasm | Molecular function unknown | Cytoplasm |
| PREPL | NP_001165084.1 | 9581 | prolyl endopeptidase-like isoform 4 | 7.8 | 4 | 2 | 2 | 2 | 2.73E+06 | - | - | - | 638 | 73.3 | 5.4 | Protein metabolism | - | Serine-type peptidase activity | - |
| PRKAR2B | NP_002727.2 | 5577 | cAMP-dependent protein kinase type II-beta regulatory subunit | 19.9 | 1 | 3 | 5 | 11 | 5.80E+07 | - | - | - | 418 | 46.3 | 4.9 | Cell communication;Signal transduction | Nucleus;Cytoplasm;Golgi apparatus | Protein serine/threonine kinase activity | Cytoplasm |
| PRKCA | NP_002728.1 | 5578 | protein kinase C alpha type | 10.4 | 1 | 3 | 3 | 3 | 7.74E+06 | - | - | - | 672 | 76.7 | 7.1 | Cell communication;Signal transduction | Nucleus;Cytoplasm;Plasma membrane;Synapse;Perinuclear region | Protein serine/threonine kinase activity | Cytoplasm |
| PRKCDBP | NP_659477.2 | 112464 | protein kinase C delta-binding protein | 10.7 | 1 | 2 | 2 | 2 | 6.66E+07 | - | - | - | 261 | 27.7 | 6.4 | Cell communication;Signal transduction | Nucleus | Molecular function unknown | Nucleus |
| PRKD2 | NP_001073351.1 | 25865 | serine/threonine-protein kinase D2 isoform B | 2.2 | 2 | 1 | 1 | 2 | 1.21E+07 | - | - | - | 721 | 80.1 | 7.3 | Cell communication;Signal transduction | Cytoplasm;Plasma membrane | Protein serine/threonine kinase activity | Cytoplasm |
| PROCR | NP_006395.2 | 10544 | endothelial protein C receptor precursor | 3.8 | 1 | 1 | 1 | 1 | 1.19E+07 | - | - | - | 238 | 26.7 | 7.2 | Immune response | Plasma membrane;Centrosome | Antigen binding | Plasma membrane |
| PRR14L | NP_775837.2 | 253143 | protein PRR14L | 0.8 | 1 | 1 | 1 | 1 | 8.31E+07 | - | - | - | 2151 | 237.2 | 6.3 | Biological_process unknown | - | Molecular function unknown | - |
| PRSS1 | NP_002760.1 | 5644 | trypsin-1 preproprotein | 4.1 | 1 | 1 | 1 | 86 | 2.76E+10 | - | - | - | 247 | 26.5 | 6.5 | Protein metabolism | Extracellular | Serine-type peptidase activity | Extracellular |
| PRSS23 | NP_009104.1 | 11098 | serine protease 23 precursor | 6.8 | 1 | 2 | 2 | 2 | 1.19E+07 | - | - | - | 383 | 43 | 9.4 | Protein metabolism | - | Serine-type peptidase activity | - |
| PRUNE | NP_067045.1 | 58497 | protein prune homolog | 4.6 | 1 | 1 | 1 | 1 | 7.52E+06 | - | - | - | 453 | 50.2 | 5.5 | Biological_process unknown | Cytoplasm;Nucleus | Molecular function unknown | Cytoplasm |
| PSMG4 | NP_001129222.1 | 389362 | proteasome assembly chaperone 4 isoform c | 24.0 | 3 | 1 | 1 | 1 | 0.00E+00 | - | - | - | 104 | 11.2 | 6.5 | Biological_process unknown | - | Molecular function unknown | - |
| PTDSS2 | NP_110410.1 | 81490 | phosphatidylserine synthase 2 | 3.1 | 1 | 1 | 1 | 1 | 1.69E+08 | - | - | - | 487 | 56.2 | 6.3 | Metabolism;Energy pathways | Mitochondrion;Nucleus;Cytoplasm | Transferase activity | Mitochondrion |
| PTGES | NP_004869.1 | 9536 | prostaglandin E synthase | 6.6 | 1 | 1 | 1 | 1 | 6.24E+07 | - | - | - | 152 | 17.1 | 9.5 | Lipid metabolism | Microsome;Integral to membrane | Molecular function unknown | Microsome |
| PTP4A2 | NP_001182030.1 | 8073 | protein tyrosine phosphatase type IVA 2 isoform 4 | 22.1 | 3 | 2 | 2 | 2 | 1.54E+06 | - | - | - | 136 | 15.7 | 8.5 | Cell communication;Signal transduction | Perinuclear region;Nucleus;Endosome;Plasma membrane;Cytoplasm | Protein tyrosine phosphatase activity | Nucleus |
| PTPLAD2 | NP_001010915.2 | 401494 | very-long-chain (3R)-3-hydroxyacyl-[acyl-carrier protein] dehydratase 4 | 3.9 | 1 | 1 | 1 | 1 | 6.51E+06 | - | - | - | 232 | 27.5 | 8.6 | Biological_process unknown | Integral to membrane | Molecular function unknown | Integral to membrane |
| PTPN11 | NP_002825.3 | 5781 | tyrosine-protein phosphatase non-receptor type 11 isoform 1 | 31.4 | 1 | 3 | 12 | 19 | 1.01E+08 | - | - | - | 593 | 68 | 7.3 | Cell communication;Signal transduction | Cytoplasm;Plasma membrane | Protein tyrosine phosphatase activity | Cytoplasm |
| PTPN11 | NP_542168.1 | 5781 | tyrosine-protein phosphatase non-receptor type 11 isoform 2 | 35.2 | 1 | 1 | 10 | 16 | 1.45E+08 | - | - | - | 460 | 52.8 | 7.4 | Cell communication;Signal transduction | Cytoplasm;Plasma membrane | Protein tyrosine phosphatase activity | Cytoplasm |
| PTPN13 | NP_542415.1 | 5783 | tyrosine-protein phosphatase non-receptor type 13 isoform 3 | 1.4 | 4 | 2 | 2 | 2 | 1.51E+07 | - | - | - | 2294 | 255.8 | 6.2 | Cell communication;Signal transduction;Apoptosis | Plasma membrane;Cytoplasm;Early endosome | Protein tyrosine phosphatase activity | Cytoplasm |
| PTPN9 | NP_002824.1 | 5780 | tyrosine-protein phosphatase non-receptor type 9 | 2.2 | 1 | 1 | 1 | 1 | 3.09E+07 | - | - | - | 593 | 68 | 8.0 | Cell communication;Signal transduction | Cytoplasm | Protein tyrosine phosphatase activity | Cytoplasm |
| PTPRB | NP_001193901.1 | 5787 | receptor-type tyrosine-protein phosphatase beta isoform d precursor | 1.1 | 3 | 1 | 1 | 1 | 0.00E+00 | - | - | - | 1907 | 214.4 | 7.6 | Cell communication;Signal transduction | Plasma membrane;Nucleus | Receptor signaling protein tyrosine phosphatase activity | Plasma membrane |
| PTTG1 | NP_001269312.1 | 9232 | securin | 7.9 | 1 | 1 | 1 | 1 | 6.45E+05 | - | - | - | 202 | 22 | 6.6 | Regulation of nucleobase, nucleoside, nucleotide and nucleic acid metabolism | Cytoplasm;Nucleus | Transcription regulator activity | Cytoplasm |
| PTX3 | NP_002843.2 | 5806 | pentraxin-related protein PTX3 precursor | 7.4 | 1 | 2 | 2 | 2 | 5.14E+07 | - | - | - | 381 | 41.9 | 5.0 | Immune response | Extracellular | Defense/immunity protein activity | Extracellular |
| PUF60 | NP_001129505.1 | 22827 | poly(U)-binding-splicing factor PUF60 isoform c | 60.1 | 4 | 1 | 18 | 107 | 1.13E+09 | - | - | - | 516 | 55.7 | 5.3 | Regulation of nucleobase, nucleoside, nucleotide and nucleic acid metabolism | Nucleus | RNA binding | Nucleus |
| PVRL2 | NP_001036189.1 | 5819 | poliovirus receptor-related protein 2 isoform delta precursor | 4.7 | 1 | 1 | 1 | 1 | 7.20E+07 | - | - | - | 538 | 57.7 | 4.8 | Cell communication;Signal transduction | Plasma membrane;Nucleus | Coreceptor activity | Plasma membrane;Nucleus |
| PVRL3 | NP_056295.1 | 25945 | poliovirus receptor-related protein 3 isoform 1 precursor | 2.0 | 1 | 1 | 1 | 1 | 1.11E+08 | - | - | - | 549 | 61 | 6.2 | Cell growth and/or maintenance | Plasma membrane | Cell adhesion molecule activity | Plasma membrane |
| PXN | NP_001074324.1 | 5829 | paxillin isoform 2 | 44.0 | 2 | 1 | 14 | 48 | 3.70E+08 | - | - | - | 591 | 64.5 | 6.2 | Signal transduction | Focal adhesion;Golgi apparatus;Plasma membrane;Cytoplasm;Extracellular matrix;Cytosol;Membrane fraction;Soluble fraction | Cell adhesion molecule activity | Focal adhesion |
| PXN | NP_002850.2 | 5829 | paxillin isoform 1 | 44.9 | 3 | 1 | 14 | 48 | 3.70E+08 | - | - | - | 557 | 60.9 | 6.4 | Signal transduction | Focal adhesion;Golgi apparatus;Plasma membrane;Cytoplasm;Extracellular matrix;Cytosol;Membrane fraction;Soluble fraction | Cell adhesion molecule activity | Focal adhesion |
| PYDC2 | NP_001076777.1 | 152138 | pyrin domain-containing protein 2 | 6.2 | 1 | 1 | 1 | 2 | 1.85E+08 | - | - | - | 97 | 10.8 | 5.4 | Biological_process unknown;Regulation of signal transduction | Nucleus;Cytoplasm;Perinuclear region | Transcription regulator activity | Nucleus |
| PYURF | NP_116295.1 | 100996939 | protein preY, mitochondrial precursor | 20.2 | 1 | 2 | 2 | 2 | 1.25E+07 | - | - | - | 114 | 12.6 | 9.3 | - | - | - | - |
| RAB11B | NP_004209.2 | 9230 | ras-related protein Rab-11B | 46.3 | 2 | 1 | 9 | 23 | 6.08E+08 | - | - | - | 218 | 24.5 | 5.9 | Cell communication;Signal transduction | Nucleus;Golgi apparatus;Plasma membrane | GTPase activator activity | Nucleus |
| RAB25 | NP_065120.2 | 57111 | ras-related protein Rab-25 | 5.2 | 1 | 1 | 1 | 1 | 2.50E+07 | - | - | - | 213 | 23.5 | 6.0 | Cell communication;Signal transduction | Golgi apparatus | GTPase activity | Golgi apparatus |
| RAB26 | NP_055168.2 | 25837 | ras-related protein Rab-26 | 8.6 | 1 | 1 | 1 | 1 | 8.96E+08 | - | - | - | 256 | 27.9 | 9.4 | Cell communication;Signal transduction | Secretory granule | GTPase activity | Secretory granule |
| RAB3IL1 | NP_037533.2 | 5866 | guanine nucleotide exchange factor for Rab-3A isoform 1 | 9.2 | 2 | 2 | 2 | 3 | 6.65E+06 | - | - | - | 382 | 42.6 | 6.5 | Biological_process unknown | - | Molecular function unknown | - |
| RABAC1 | NP_006414.2 | 10567 | prenylated Rab acceptor protein 1 | 12.4 | 1 | 1 | 1 | 2 | 1.93E+08 | - | - | - | 185 | 20.6 | 7.3 | Transport | Plasma membrane;Cytoplasm;Golgi apparatus | GTPase activator activity | Golgi apparatus |

| Gene Symbol | RefSeq_Accession | Entrez Gene ID | Description | Coverage (%) | Proteins | Unique Peptides | # Peptides | PSM | Area | SCC-R /SCC-S | SCC-R /SCC-S (Count) | SCC-R /SCC-S (Variability [%]) | # AAs | MW [kDa] | calc. pI | Biological Process | Localization | Molecular Function | Primary Localization |
|---|---|---|---|---|---|---|---|---|---|---|---|---|---|---|---|---|---|---|---|
| RABGEF1 | NP_001273991.1 | 27342 | rab5 GDP/GTP exchange factor isoform c | 6.5 | 3 | 2 | 2 | 4 | 8.58E+07 | - | - | - | 491 | 56.9 | 7.0 | Cell communication;Signal transduction | - | Guanyl-nucleotide exchange factor activity | - |
| RABGGTB | NP_004573.2 | 5876 | geranylgeranyl transferase type-2 subunit beta | 9.7 | 1 | 3 | 3 | 4 | 2.74E+07 | - | - | - | 331 | 36.9 | 5.0 | Metabolism;Energy pathways | Cytoplasm | Transferase activity | Cytoplasm |
| RABL2A | NP_009013.1 | 11159 | rab-like protein 2A | 9.7 | 4 | 1 | 1 | 1 | 1.17E+08 | - | - | - | 228 | 26.1 | 4.9 | Cell communication;Signal transduction | Plasma membrane | GTPase activity | Plasma membrane |
| RACGAP1 | NP_001119575.1 | 29127 | rac GTPase-activating protein 1 | 4.1 | 1 | 1 | 1 | 1 | 1.21E+07 | - | - | - | 632 | 71 | 8.9 | Cell communication;Signal transduction | Nucleus;Microtubule;Chromosome | GTPase activator activity | Nucleus |
| RAF1 | NP_002871.1 | 5894 | RAF proto-oncogene serine/threonine-protein kinase | 4.9 | 2 | 1 | 2 | 5 | 1.23E+08 | - | - | - | 648 | 73 | 9.2 | Cell communication;Signal transduction | Plasma membrane;Nucleus;Cytoplasm | Protein serine/threonine kinase activity | Cytoplasm |
| RALBP1 | NP_006779.1 | 10928 | ralA-binding protein 1 | 3.7 | 1 | 1 | 1 | 1 | 0.00E+00 | - | - | - | 655 | 76 | 5.9 | Cell communication;Signal transduction | Plasma membrane;Nucleus;Cytoplasm | GTPase activator activity | Plasma membrane |
| RANBP9 | NP_005484.2 | 10048 | ran-binding protein 9 | 4.1 | 1 | 2 | 2 | 3 | 3.12E+07 | - | - | - | 729 | 77.8 | 6.8 | Cell growth and/or maintenance | Nucleus | Cytoskeletal protein binding | Nucleus |
| RAP1A | NP_001010935.1 | 5906 | ras-related protein Rap-1A precursor | 31.0 | 1 | 2 | 5 | 13 | 1.15E+08 | - | - | - | 184 | 21 | 6.7 | Cell communication;Signal transduction | Nucleus;Plasma membrane;Late endosome;Lysosome;Perinuclear region;Cytoplasm;Mitochondrion | GTPase activity | Plasma membrane |
| RASA1 | NP_072179.1 | 5921 | ras GTPase-activating protein 1 isoform 2 | 4.8 | 2 | 2 | 2 | 2 | 2.50E+07 | - | - | - | 870 | 100.3 | 7.6 | Cell communication;Signal transduction | Cytoplasm;Plasma membrane | GTPase activator activity | Cytoplasm |
| RASAL3 | NP_075055.1 | 64926 | RAS protein activator like-3 | 2.0 | 1 | 1 | 2 | 2 | 4.31E+08 | - | - | - | 1011 | 111.8 | 8.8 | Biological_process unknown | - | Molecular function unknown | - |
| RBBP4 | NP_005601.1 | 5928 | histone-binding protein RBBP4 isoform a | 66.1 | 2 | 2 | 12 | 49 | 2.08E+09 | - | - | - | 425 | 47.6 | 4.9 | Regulation of nucleobase, nucleoside, nucleotide and nucleic acid metabolism | Nucleus;Nucleolus;Mitochondrion | Transcription regulator activity | Nucleus |
| RBBP5 | NP_001180201.1 | 5929 | retinoblastoma-binding protein 5 isoform 2 | 6.8 | 3 | 2 | 2 | 3 | 8.76E+06 | - | - | - | 500 | 55 | 5.0 | Regulation of nucleobase, nucleoside, nucleotide and nucleic acid metabolism | Nucleus;Cytoplasm | Transcription regulator activity | Nucleus |
| RBBP6 | NP_061173.1 | 5930 | E3 ubiquitin-protein ligase RBBP6 isoform 2 | 0.7 | 2 | 1 | 1 | 1 | 1.02E+07 | - | - | - | 1758 | 197.2 | 9.6 | Protein metabolism | Nucleus;Cytoplasm | Ubiquitin-specific protease activity | Nucleus;Cytoplasm |
| RBBP8NL | NP_543023.2 | 140893 | RBBP8 N-terminal-like protein | 3.3 | 1 | 1 | 1 | 1 | 3.87E+07 | - | - | - | 664 | 71.4 | 6.5 | Biological_process unknown | - | Molecular function unknown | - |
| RBCK1 | NP_006453.1 | 10616 | ranBP-type and C3HC4-type zinc finger-containing protein 1 isoform 1 | 5.8 | 2 | 1 | 1 | 1 | 1.70E+07 | - | - | - | 468 | 52.9 | 5.5 | Protein metabolism;Regulation of nucleobase, nucleoside, nucleotide and nucleic acid metabolism | Cytoplasm;Nucleus | Ubiquitin binding;Transcription factor activity | Cytoplasm |
| RBM4B | NP_113680.1 | 83759 | RNA-binding protein 4B isoform 1 | 29.0 | 2 | 1 | 7 | 17 | 2.88E+08 | - | - | - | 359 | 40.1 | 6.7 | Regulation of nucleobase, nucleoside, nucleotide and nucleic acid metabolism | Cytoplasm | RNA binding | Cytoplasm |
| RC3H1 | NP_742068.1 | 149041 | roquin-1 | 2.1 | 1 | 1 | 1 | 1 | 1.56E+07 | - | - | - | 1133 | 125.7 | 7.3 | Protein metabolism | Cytoplasm | Ubiquitin binding | Cytoplasm |
| RCCD1 | NP_001017919.1 | 91433 | RCC1 domain-containing protein 1 | 2.9 | 1 | 1 | 1 | 2 | 1.78E+07 | - | - | - | 376 | 40.1 | 5.3 | Biological_process unknown | - | Molecular function unknown | - |
| RECQL4 | NP_004251.3 | 9401 | ATP-dependent DNA helicase Q4 | 1.3 | 1 | 1 | 1 | 1 | 4.97E+06 | - | - | - | 1208 | 133 | 8.1 | DNA repair | Nucleus | Helicase activity | Nucleus |
| RGS8 | NP_203131.1 | 85397 | regulator of G-protein signaling 8 isoform 1 | 9.6 | 1 | 1 | 1 | 1 | 0.00E+00 | - | - | - | 198 | 23 | 9.2 | Cell communication;Signal transduction | Nucleus;Plasma membrane | GTPase activator activity | Nucleus |
| RHBDD1 | NP_001161080.1 | 84236 | rhomboid-related protein 4 | 5.4 | 1 | 1 | 1 | 1 | 5.75E+06 | - | - | - | 315 | 35.8 | 8.2 | Biological_process unknown | - | Molecular function unknown | - |
| RHOB | NP_004031.1 | 388 | rho-related GTP-binding protein RhoB precursor | 25.5 | 1 | 1 | 2 | 16 | 0.00E+00 | - | - | - | 196 | 22.1 | 5.2 | Cell communication;Signal transduction | Plasma membrane;Golgi apparatus | GTPase activity | Plasma membrane |
| RIOK2 | NP_001153221.1 | 55781 | serine/threonine-protein kinase RIO2 isoform 2 | 8.0 | 2 | 2 | 2 | 2 | 2.16E+07 | - | - | - | 474 | 54.5 | 5.4 | Biological_process unknown | Nucleus;Cytoplasm | Molecular function unknown | Nucleus;Cytoplasm |
| RLTPR | NP_001013860.1 | 146206 | leucine-rich repeat-containing protein 16C | 0.8 | 1 | 1 | 1 | 2 | 8.01E+06 | - | - | - | 1435 | 154.6 | 6.8 | Signal transduction;Cell communication | - | Molecular function unknown | - |
| RNF149 | NP_775918.2 | 284996 | E3 ubiquitin-protein ligase RNF149 precursor | 6.3 | 1 | 1 | 1 | 1 | 4.77E+07 | - | - | - | 400 | 43.1 | 6.5 | Protein metabolism | - | Ubiquitin-specific protease activity | - |
| RNF181 | NP_057578.1 | 51255 | E3 ubiquitin-protein ligase RNF181 | 12.4 | 1 | 1 | 1 | 1 | 4.80E+05 | - | - | - | 153 | 17.9 | 5.1 | Biological_process unknown | - | Molecular function unknown | - |
| RNF34 | NP_001243787.1 | 80196 | E3 ubiquitin-protein ligase RNF34 isoform 3 | 7.2 | 3 | 1 | 1 | 1 | 2.83E+07 | - | - | - | 180 | 20.4 | 4.5 | Apoptosis | Cytoplasm;Nucleus | Ubiquitin-specific protease activity | Cytoplasm |
| RNF5 | NP_008844.1 | 6048 | E3 ubiquitin-protein ligase RNF5 | 8.3 | 1 | 1 | 1 | 1 | 1.67E+06 | - | - | - | 180 | 19.9 | 6.7 | Protein metabolism | Plasma membrane;Nucleus;Endoplasmic reticulum membrane | Ligase activity | Plasma membrane |
| RNPC3 | NP_060089.1 | 55599 | RNA-binding protein 40 | 5.0 | 1 | 1 | 1 | 1 | 1.48E+07 | - | - | - | 517 | 58.5 | 7.7 | Regulation of nucleobase, nucleoside, nucleotide and nucleic acid metabolism | Nucleus | RNA binding | Nucleus |
| RPAP2 | NP_079089.2 | 79871 | putative RNA polymerase II subunit B1 CTD phosphatase RPAP2 | 2.8 | 1 | 1 | 1 | 1 | 9.79E+08 | - | - | - | 612 | 69.5 | 7.8 | Biological_process unknown | - | Molecular function unknown | - |
| RPS6KB2 | NP_003943.2 | 6199 | ribosomal protein S6 kinase beta-2 | 7.1 | 6 | 1 | 2 | 2 | 1.99E+07 | - | - | - | 482 | 53.4 | 7.3 | Protein metabolism;Signal transduction | Cytoplasm;Nucleus;Nucleoplasm | Protein serine/threonine kinase activity | Cytoplasm |
| RPUSD2 | NP_001273336.1 | 27079 | RNA pseudouridylate synthase domain-containing protein 2 isoform 2 | 7.9 | 2 | 2 | 2 | 3 | 3.50E+07 | - | - | - | 484 | 54.1 | 6.7 | Biological_process unknown | - | Molecular function unknown | - |
| RRAGB | NP_006055.3 | 10325 | ras-related GTP-binding protein B short isoform | 7.8 | 2 | 1 | 2 | 2 | 1.70E+08 | - | - | - | 346 | 40.1 | 6.1 | Cell communication;Signal transduction | Cytoplasm | Guanyl-nucleotide exchange factor activity | Cytoplasm |
| RRAGC | NP_001258780.1 | 64121 | ras-related GTP-binding protein C isoform 2 | 5.8 | 2 | 1 | 1 | 1 | 1.76E+06 | - | - | - | 365 | 40.2 | 5.1 | Cell communication;Signal transduction | Cytoplasm;Nucleus | GTPase activity | Cytoplasm |
| RRAS2 | NP_001170785.1 | 22800 | ras-related protein R-Ras2 isoform c | 19.5 | 3 | 1 | 3 | 9 | 6.01E+07 | - | - | - | 169 | 19.8 | 5.5 | Cell communication;Signal transduction | Plasma membrane;Cytoplasm;Endoplasmic reticulum;Nucleus | GTPase activity | Plasma membrane |
| RSRC1 | NP_001258763.1 | 51319 | serine/Arginine-related protein 53 isoform 2 | 7.3 | 2 | 1 | 1 | 1 | 1.82E+07 | - | - | - | 276 | 31.5 | 10.7 | Biological_process unknown | - | Molecular function unknown | - |
| RTKN | NP_001015056.1 | 6242 | rhotekin isoform c | 9.8 | 5 | 3 | 3 | 4 | 2.03E+07 | - | - | - | 513 | 56.6 | 6.9 | Cell communication;Signal transduction | Cytoplasm | Transcription regulator activity | Cytoplasm |

| Gene Symbol | RefSeq_Accession | Entrez Gene ID | Description | Coverage (%) | Proteins | Unique Peptides | # Peptides | PSM | Area | SCC-R /SCC-S | SCC-R /SCC-S (Count) | SCC-R /SCC-S (Variability [%]) | # AAs | MW [kDa] | calc. pI | Biological Process | Localization | Molecular Function | Primary Localization |
|---|---|---|---|---|---|---|---|---|---|---|---|---|---|---|---|---|---|---|---|
| **RUFY2** | NP_001035882.1 | 55680 | **RUN and FYVE domain-containing protein 2 isoform b** | 2.5 | 1 | 1 | 1 | 1 | 7.01E+07 | - | - | - | 403 | 46.1 | 5.2 | Biological_process unknown | - | Molecular function unknown | - |
| **RYR1** | NP_001036188.1 | 6261 | **ryanodine receptor 1 isoform 2** | 0.5 | 2 | 1 | 1 | 1 | 7.64E+08 | - | - | - | 5033 | 564.3 | 5.3 | Transport | Endoplasmic reticulum;Sarcoplasmic reticulum;Nucleus | Intracellular ligand-gated ion channel activity | Sarcoplasmic reticulum |
| **S100A14** | NP_065723.1 | 57402 | **protein S100-A14** | 74.0 | 1 | 7 | 7 | 26 | 6.29E+08 | - | - | - | 104 | 11.7 | 5.2 | Biological_process unknown | Cytoplasm;Plasma membrane | Calcium ion binding | Cytoplasm |
| **S100A3** | NP_002951.1 | 6274 | **protein S100-A3** | 20.8 | 1 | 1 | 1 | 2 | 1.54E+07 | - | - | - | 101 | 11.7 | 4.8 | Cell communication;Signal transduction | Cytoplasm | Calcium ion binding | Cytoplasm |
| **SAC3D1** | NP_037431.3 | 29901 | **SAC3 domain-containing protein 1** | 9.5 | 1 | 2 | 2 | 2 | 1.07E+07 | - | - | - | 358 | 38.6 | 7.6 | Cell communication;Signal transduction | Centrosome;Nucleus | Molecular function unknown | Centrosome |
| **SAMD1** | NP_612361.1 | 90378 | **atherin** | 3.2 | 1 | 1 | 1 | 1 | 4.07E+07 | - | - | - | 538 | 56 | 7.6 | Biological_process unknown | - | Molecular function unknown | - |
| **SCAF8** | NP_001273128.1 | 22828 | **protein SCAF8 isoform d** | 4.3 | 4 | 3 | 3 | 3 | 1.82E+07 | - | - | - | 1271 | 140.4 | 8.3 | Regulation of nucleobase, nucleoside, nucleotide and nucleic acid metabolism | Nucleus | RNA binding | Nucleus |
| **SCAP** | NP_036367.2 | 22937 | **sterol regulatory element-binding protein cleavage-activating protein** | 1.8 | 1 | 1 | 1 | 1 | 6.56E+08 | - | - | - | 1279 | 139.6 | 6.9 | Metabolism;Energy pathways | Endoplasmic reticulum;Golgi apparatus;Nucleus | Chaperone activity | Endoplasmic reticulum |
| **SCN1A** | NP_001159436.1 | 6323 | **sodium channel protein type 1 subunit alpha isoform 3** | 1.2 | 3 | 1 | 1 | 1 | 1.00E+08 | - | - | - | 1981 | 226 | 5.9 | Transport | Plasma membrane | Voltage-gated ion channel activity | Plasma membrane |
| **SCN3A** | NP_001075145.1 | 6328 | **sodium channel protein type 3 subunit alpha isoform 2** | 1.9 | 3 | 1 | 1 | 1 | 1.11E+08 | - | - | - | 1951 | 221.3 | 5.7 | Transport | Plasma membrane | Voltage-gated ion channel activity | Plasma membrane |
| **SCO2** | NP_005129.2 | 9997 | **protein SCO2 homolog, mitochondrial precursor** | 11.3 | 1 | 1 | 1 | 1 | 1.39E+06 | - | - | - | 266 | 29.8 | 8.9 | Protein metabolism | Mitochondrion | Chaperone activity | Mitochondrion |
| **SCOC** | NP_001147162.1 | 60592 | **short coiled-coil protein isoform 6** | 29.8 | 6 | 2 | 2 | 2 | 2.05E+06 | - | - | - | 94 | 10.7 | 5.1 | Biological_process unknown | Golgi apparatus;Plasma membrane | Molecular function unknown | Golgi apparatus |
| **SDK1** | NP_689957.3 | 221935 | **protein sidekick-1 isoform 1** | 1.2 | 1 | 1 | 1 | 1 | 0.00E+00 | - | - | - | 2213 | 242 | 6.4 | Cell growth and/or maintenance | Plasma membrane | Cell adhesion molecule activity | Plasma membrane |
| **SEC16A** | NP_001263347.1 | 9919 | **protein transport protein Sec16A isoform 2** | 21.3 | 1 | 1 | 25 | 55 | 8.35E+08 | - | - | - | 2334 | 249.3 | 5.9 | Cell motility;ER organization and biogenesis;Protein transport | Endoplasmic reticulum | Molecular function unknown | Endoplasmic reticulum |
| **SEC62** | NP_003253.1 | 7095 | **translocation protein SEC62** | 4.5 | 1 | 2 | 2 | 2 | 9.87E+07 | - | - | - | 399 | 45.8 | 7.1 | Protein metabolism | Endoplasmic reticulum;Nucleus;Cytoplasm | Transporter activity | Endoplasmic reticulum |
| **SEL1L3** | NP_056002.2 | 23231 | **protein sel-1 homolog 3** | 0.5 | 1 | 1 | 1 | 1 | 0.00E+00 | - | - | - | 1132 | 128.5 | 7.3 | Biological_process unknown | - | Molecular function unknown | - |
| **SEMA3B** | NP_001005914.1 | 7869 | **semaphorin-3B isoform 2 precursor** | 5.1 | 1 | 1 | 1 | 1 | 1.11E+07 | - | - | - | 748 | 83 | 8.9 | Cell communication;Signal transduction;Apoptosis | Extracellular;Endoplasmic reticulum | Molecular function unknown | Extracellular |
| **SEMA3C** | NP_006370.1 | 10512 | **semaphorin-3C precursor** | 12.8 | 1 | 5 | 5 | 6 | 3.34E+07 | - | - | - | 751 | 85.2 | 8.7 | Cell communication;Signal transduction | Extracellular | Receptor binding | Extracellular |
| **SEPSECS** | NP_058651.3 | 51091 | **O-phosphoseryl-tRNA(Sec) selenium transferase** | 6.8 | 1 | 2 | 2 | 2 | 9.44E+06 | - | - | - | 501 | 55.7 | 8.1 | Biological_process unknown | - | Molecular function unknown | - |
| **SERPINB13** | NP_036529.1 | 5275 | **serpin B13** | 7.2 | 1 | 2 | 2 | 2 | 1.38E+07 | - | - | - | 391 | 44.2 | 5.7 | Protein metabolism | Cytoplasm | Protease inhibitor activity | Cytoplasm |
| **SERPINB2** | NP_002566.1 | 5055 | **plasminogen activator inhibitor 2** | 2.2 | 1 | 1 | 1 | 1 | 8.68E+07 | - | - | - | 415 | 46.6 | 5.6 | Protein metabolism;Anti-apoptosis | Cytoplasm;Extracellular;Nucleus | Protease inhibitor activity | Cytoplasm |
| **SERPINB3** | NP_008850.1 | 6317 | **serpin B3** | 31.5 | 2 | 8 | 8 | 12 | 5.00E+07 | - | - | - | 390 | 44.5 | 6.8 | Protein metabolism | Cytoplasm;Extracellular | Protease inhibitor activity | Cytoplasm |
| **SEZ6L2** | NP_001107572.1 | 26470 | **seizure 6-like protein 2 isoform 4 precursor** | 3.8 | 6 | 1 | 1 | 1 | 2.62E+06 | - | - | - | 809 | 86.8 | 4.8 | Biological_process unknown | Plasma membrane | Molecular function unknown | Plasma membrane |
| **SF1** | NP_001171501.1 | 7536 | **splicing factor 1 isoform 6** | 21.1 | 2 | 1 | 10 | 41 | 8.89E+08 | - | - | - | 673 | 71.7 | 9.6 | Regulation of nucleobase, nucleoside, nucleotide and nucleic acid metabolism | Nucleus;Cytoplasm | RNA binding | Nucleus;Cytoplasm |
| **SFTPB** | NP_000533.3 | 6439 | **pulmonary surfactant-associated protein B precursor** | 5.6 | 1 | 1 | 1 | 1 | 0.00E+00 | - | - | - | 393 | 43.3 | 5.7 | Immune response;Cellular defense response | Extracellular;Endoplasmic reticulum;Golgi apparatus | Molecular function unknown | Extracellular |
| **SFXN4** | NP_998814.1 | 119559 | **sideroflexin-4** | 4.2 | 1 | 1 | 1 | 1 | 8.44E+07 | - | - | - | 337 | 38 | 9.2 | Transport | Integral to membrane | Transporter activity | Integral to membrane |
| **SGCZ** | NP_631906.2 | 137868 | **zeta-sarcoglycan** | 5.5 | 1 | 1 | 1 | 1 | 3.01E+07 | - | - | - | 312 | 34.5 | 7.0 | Cell growth and/or maintenance | Plasma membrane | Cell adhesion molecule activity | Plasma membrane |
| **SH3PXD2A** | NP_055446.2 | 9644 | **SH3 and PX domain-containing protein 2A** | 2.0 | 1 | 1 | 1 | 1 | 1.00E+07 | - | - | - | 1105 | 121.8 | 8.4 | Cell communication;Signal transduction | Nucleus | Receptor signaling complex scaffold activity | Nucleus |
| **SHARPIN** | NP_112236.3 | 81858 | **sharpin** | 3.4 | 1 | 1 | 1 | 1 | 4.08E+07 | - | - | - | 387 | 39.9 | 5.8 | Biological_process unknown | Plasma membrane;Cytoplasm | Molecular function unknown | Plasma membrane |
| **SHISA2** | NP_001007539.1 | 387914 | **protein shisa-2 homolog precursor** | 7.1 | 1 | 1 | 1 | 1 | 1.01E+08 | - | - | - | 295 | 31.4 | 7.7 | Biological_process unknown | Integral to membrane | Molecular function unknown | Integral to membrane |
| **SIGMAR1** | NP_001269138.1 | 10280 | **sigma non-opioid intracellular receptor 1 isoform 10** | 7.3 | 5 | 1 | 1 | 1 | 7.28E+07 | - | - | - | 110 | 12.5 | 6.5 | Cell communication;Signal transduction | Endoplasmic reticulum;Nuclear membrane;Plasma membrane | Transmembrane receptor activity | Plasma membrane |
| **SIRT1** | NP_001135970.1 | 23411 | **NAD-dependent protein deacetylase sirtuin-1 isoform b** | 3.1 | 2 | 1 | 1 | 1 | 1.85E+07 | - | - | - | 452 | 50.5 | 4.8 | Metabolism;Energy pathways | Nucleus;Cytoplasm | Deacetylase activity | Nucleus |
| **SIRT2** | NP_001180215.1 | 22933 | **NAD-dependent protein deacetylase sirtuin-2 isoform 3** | 4.3 | 3 | 1 | 1 | 1 | 6.44E+07 | - | - | - | 234 | 26.7 | 6.5 | Cell communication;Signal transduction | Cytoskeleton;Cytoplasm;Nucleus | Deacetylase activity | Cytoplasm |
| **SIRT6** | NP_057623.2 | 51548 | **NAD-dependent protein deacetylase sirtuin-6 isoform 1** | 6.5 | 1 | 1 | 1 | 1 | 2.22E+07 | - | - | - | 355 | 39.1 | 9.2 | Regulation of nucleobase, nucleoside, nucleotide and nucleic acid metabolism | Nucleus | Deacetylase activity | Nucleus |
| **SLC11A2** | NP_001167601.1 | 4891 | **natural resistance-associated macrophage protein 2 isoform 4** | 5.0 | 4 | 1 | 1 | 1 | 4.48E+07 | - | - | - | 557 | 61 | 6.6 | Transport | Endosome;Lysosome;Plasma membrane;Cytoplasm;Nucleus | Transporter activity | Endosome |
| **SLC12A2** | NP_001243390.1 | 6558 | **solute carrier family 12 member 2 isoform 2** | 1.4 | 2 | 1 | 1 | 1 | 3.54E+07 | - | - | - | 1196 | 129.6 | 6.5 | Transport | Plasma membrane;Extracellular | Auxiliary transport protein activity | Plasma membrane |
| **SLC12A9** | NP_064631.2 | 56996 | **solute carrier family 12 member 9 isoform 1** | 2.1 | 1 | 1 | 1 | 1 | 1.74E+08 | - | - | - | 914 | 96 | 8.1 | Transport | Plasma membrane | Auxiliary transport protein activity | Plasma membrane |

Supplementary Table S4. List of all proteins identified in SCC-S and SCC-R cells using SequestHT and Mascot search algorithms and their gene ontology annotation

| Gene Symbol | RefSeq_Accession | Entrez Gene ID | Description | Coverage (%) | Proteins | Unique Peptides | # Peptides | PSM | Area | SCC-R /SCC-S | SCC-R /SCC-S (Count) | SCC-R /SCC-S (Variability [%]) | # AAs | MW [kDa] | calc. pI | Biological Process | Localization | Molecular Function | Primary Localization |
|---|---|---|---|---|---|---|---|---|---|---|---|---|---|---|---|---|---|---|---|
| SLC15A3 | NP_057666.1 | 51296 | solute carrier family 15 member 3 | 2.8 | 1 | 1 | 1 | 1 | 1.35E+07 | - | - | - | 581 | 63.5 | 9.1 | Transport | Lysosome | Auxiliary transport protein activity | Lysosome |
| SLC17A5 | NP_036566.1 | 26503 | sialin | 5.5 | 1 | 1 | 1 | 1 | 1.26E+07 | - | - | - | 495 | 54.6 | 8.3 | Transport | Lysosome | Transporter activity | Lysosome |
| SLC1A4 | NP_003029.2 | 6509 | neutral amino acid transporter A isoform 1 | 2.1 | 1 | 1 | 1 | 1 | 3.67E+08 | - | - | - | 532 | 55.7 | 6.3 | Transport | Plasma membrane;Cytoplasm | Transporter activity | Plasma membrane |
| SLC20A2 | NP_001244110.1 | 6575 | sodium-dependent phosphate transporter 2 | 3.4 | 1 | 1 | 1 | 1 | 9.85E+06 | - | - | - | 652 | 70.3 | 6.5 | Transport | Plasma membrane | Auxiliary transport protein activity | Plasma membrane |
| SLC22A10 | NP_001034841.3 | 387775 | solute carrier family 22 member 10 | 1.3 | 1 | 1 | 1 | 1 | 2.80E+07 | - | - | - | 541 | 60.2 | 8.6 | Transport | Integral to membrane | Ion transporter activity | Integral to membrane |
| SLC22A18 | NP_002546.3 | 5002 | solute carrier family 22 member 18 | 2.8 | 1 | 1 | 1 | 1 | 7.10E+07 | - | - | - | 424 | 44.8 | 9.6 | Transport;Ion transport | Plasma membrane | Auxiliary transport protein activity;Ion transporter activity | Plasma membrane |
| SLC25A15 | NP_055067.1 | 10166 | mitochondrial ornithine transporter 1 | 5.7 | 1 | 1 | 1 | 1 | 1.93E+07 | - | - | - | 301 | 32.7 | 9.1 | Transport | Mitochondrion | Transporter activity | Mitochondrion |
| SLC25A4 | NP_001142.2 | 291 | ADP/ATP translocase 1 | 43.6 | 2 | 1 | 12 | 99 | 1.20E+09 | - | - | - | 298 | 33 | 9.8 | Transport | Mitochondrion;Nucleus | Transporter activity | Mitochondrion |
| SLC27A2 | NP_001153101.1 | 11001 | very long-chain acyl-CoA synthetase isoform 2 | 14.8 | 2 | 4 | 4 | 4 | 4.84E+07 | - | - | - | 567 | 64.6 | 8.5 | Transport | Peroxisome;Microsome;Endoplasmic reticulum | Auxiliary transport protein activity | Peroxisome |
| SLC27A3 | NP_077306.1 | 11000 | long-chain fatty acid transport protein 3 | 4.0 | 1 | 2 | 2 | 2 | 9.89E+06 | - | - | - | 730 | 78.6 | 7.5 | Transport | Integral to membrane | Auxiliary transport protein activity | Integral to membrane |
| SLC2A14 | NP_001273165.1 | 144195 | solute carrier family 2, facilitated glucose transporter member 14 isoform c | 4.9 | 5 | 1 | 1 | 1 | 1.35E+08 | - | - | - | 411 | 44.9 | 7.8 | Transport | - | Transporter activity | - |
| SLC30A1 | NP_067017.2 | 7779 | zinc transporter 1 | 2.4 | 1 | 1 | 1 | 1 | 5.55E+06 | - | - | - | 507 | 55.3 | 6.5 | Transport | Integral to membrane | Auxiliary transport protein activity | Integral to membrane |
| SLC35A2 | NP_001269577.1 | 7355 | UDP-galactose translocator isoform e | 6.9 | 2 | 1 | 1 | 1 | 4.60E+08 | - | - | - | 218 | 23.5 | 10.6 | Transport | Golgi apparatus;Endoplasmic reticulum | Transporter activity | Golgi apparatus |
| SLC35E4 | NP_001001479.1 | 339665 | solute carrier family 35 member E4 | 7.4 | 1 | 1 | 1 | 1 | 4.59E+07 | - | - | - | 350 | 36.7 | 9.4 | Biological_process unknown | - | Molecular function unknown | - |
| SLC36A4 | NP_001273068.1 | 120103 | proton-coupled amino acid transporter 4 isoform 2 | 6.2 | 2 | 1 | 1 | 1 | 2.98E+08 | - | - | - | 369 | 41.4 | 8.3 | Transport | Integral to membrane | Auxiliary transport protein activity | Integral to membrane |
| SLC38A7 | NP_060701.1 | 55238 | putative sodium-coupled neutral amino acid transporter 7 | 2.6 | 1 | 1 | 1 | 1 | 7.19E+06 | - | - | - | 462 | 49.9 | 5.1 | Transport | - | Transporter activity | - |
| SLC39A11 | NP_631916.2 | 201266 | zinc transporter ZIP11 isoform 2 | 5.1 | 2 | 1 | 1 | 1 | 2.88E+06 | - | - | - | 335 | 34.7 | 5.4 | Transport | Integral to membrane | Transporter activity | Integral to membrane |
| SLC3A2 | NP_001012680.1 | 6520 | 4F2 cell-surface antigen heavy chain isoform b | 47.4 | 2 | 1 | 22 | 159 | 5.33E+09 | - | - | - | 631 | 68.1 | 5.1 | Transport | Plasma membrane;Mitochondrion;Extracellular;Nucleus;Cytoplasm | Auxiliary transport protein activity | Plasma membrane;Nucleus;Cytoplasm |
| SLC3A2 | NP_002385.3 | 6520 | 4F2 cell-surface antigen heavy chain isoform c | 42.9 | 2 | 1 | 22 | 159 | 5.33E+09 | - | - | - | 630 | 68 | 5.0 | Transport | Plasma membrane;Mitochondrion;Extracellular;Nucleus;Cytoplasm | Auxiliary transport protein activity | Plasma membrane;Nucleus;Cytoplasm |
| SLC4A1 | NP_000333.1 | 6521 | band 3 anion transport protein | 2.2 | 1 | 1 | 1 | 1 | 2.48E+05 | - | - | - | 911 | 101.7 | 5.2 | Transport | Plasma membrane;Nucleus;Golgi apparatus;Mitochondrion | Auxiliary transport protein activity | Plasma membrane |
| SLC5A11 | NP_001245343.1 | 115584 | sodium/myo-inositol cotransporter 2 isoform 5 | 5.2 | 1 | 1 | 1 | 1 | 0.00E+00 | - | - | - | 519 | 57.1 | 7.1 | Transport | Integral to membrane | Auxiliary transport protein activity;Transporter activity | Integral to membrane |
| SLC7A6 | NP_001070253.1 | 9057 | Y+L amino acid transporter 2 | 5.8 | 1 | 1 | 1 | 1 | 1.05E+08 | - | - | - | 515 | 56.8 | 5.9 | Transport | Plasma membrane | Auxiliary transport protein activity | Plasma membrane |
| SLC9C1 | NP_898884.1 | 285335 | sodium/hydrogen exchanger 10 | 1.1 | 1 | 1 | 1 | 1 | 7.00E+07 | - | - | - | 1177 | 135.1 | 7.1 | Transport | - | Auxiliary transport protein activity | - |
| SLCO4A1 | NP_057438.3 | 28231 | solute carrier organic anion transporter family member 4A1 | 3.3 | 1 | 1 | 1 | 1 | 1.89E+10 | - | - | - | 722 | 77.1 | 7.8 | Transport | Plasma membrane | Auxiliary transport protein activity | Plasma membrane |
| SLU7 | NP_006416.3 | 10569 | pre-mRNA-splicing factor SLU7 | 4.6 | 1 | 1 | 1 | 2 | 0.00E+00 | - | - | - | 586 | 68.3 | 7.1 | Regulation of nucleobase, nucleoside, nucleotide and nucleic acid metabolism | Nucleus | Ribonucleoprotein | Nucleus |
| SLX4 | NP_115820.2 | 84464 | structure-specific endonuclease subunit SLX4 | 0.7 | 1 | 1 | 1 | 2 | 1.43E+08 | - | - | - | 1834 | 199.9 | 6.1 | Biological_process unknown | Nucleus | Molecular function unknown | - |
| SMARCA2 | NP_001276326.1 | 6595 | probable global transcription activator SNF2L2 isoform c | 4.7 | 6 | 1 | 6 | 10 | 5.12E+06 | - | - | - | 1514 | 172.5 | 7.1 | Regulation of nucleobase, nucleoside, nucleotide and nucleic acid metabolism | Nucleus;Extracellular;Cytoplasm | Transcription factor activity | Nucleus;Cytoplasm |
| SMARCAD1 | NP_064544.2 | 56916 | SWI/SNF-related matrix-associated actin-dependent regulator of chromatin subfamily A containing DEAD/H box 1 | 3.7 | 2 | 2 | 2 | 4 | 5.05E+07 | - | - | - | 1026 | 117.3 | 5.6 | Regulation of nucleobase, nucleoside, nucleotide and nucleic acid metabolism | Nucleus | Helicase activity | Nucleus |
| SMC6 | NP_078900.1 | 79677 | structural maintenance of chromosomes protein 6 | 1.5 | 1 | 1 | 1 | 2 | 8.11E+07 | - | - | - | 1091 | 126.2 | 7.0 | Regulation of nucleobase, nucleoside, nucleotide and nucleic acid metabolism | Nucleus | Molecular function unknown | Nucleus |
| SMG5 | NP_056142.2 | 23381 | protein SMG5 | 2.4 | 1 | 1 | 1 | 1 | 1.04E+08 | - | - | - | 1016 | 113.9 | 5.9 | Biological_process unknown | Cytoplasm;Nucleus | Molecular function unknown | Cytoplasm |
| SMG7 | NP_963862.1 | 9887 | protein SMG7 isoform 2 | 2.1 | 3 | 1 | 1 | 1 | 1.10E+07 | - | - | - | 1091 | 121.9 | 8.3 | Biological_process unknown | - | Molecular function unknown | - |
| SMG8 | NP_060619.4 | 55181 | protein SMG8 | 3.3 | 1 | 1 | 1 | 1 | 5.65E+08 | - | - | - | 991 | 109.6 | 7.7 | Biological_process unknown | - | Molecular function unknown | - |
| SMG9 | NP_061981.2 | 56006 | protein SMG9 | 17.3 | 1 | 4 | 4 | 10 | 2.08E+07 | - | - | - | 520 | 57.6 | 7.0 | Biological_process unknown | Cytoplasm | Molecular function unknown | Cytoplasm |
| SMIM13 | NP_001129047.1 | 221710 | small integral membrane protein 13 | 33.0 | 1 | 1 | 1 | 1 | 4.22E+06 | - | - | - | 91 | 10.3 | 6.7 | Biological_process unknown | - | Molecular function unknown | - |
| SMPD3 | NP_061137.1 | 55512 | sphingomyelin phosphodiesterase 3 | 3.4 | 1 | 1 | 1 | 1 | 4.36E+08 | - | - | - | 655 | 71 | 5.8 | Metabolism;Energy pathways | Golgi apparatus | Catalytic activity | Golgi apparatus |
| SMTN | NP_008863.3 | 6525 | smoothelin isoform c | 5.7 | 5 | 2 | 2 | 2 | 1.26E+07 | - | - | - | 917 | 99 | 9.1 | Cell growth and/or maintenance | Cytoplasm | Cytoskeletal protein binding | Cytoplasm |
| SNUPN | NP_001036046.1 | 10073 | snurportin-1 | 7.5 | 1 | 1 | 1 | 1 | 7.59E+07 | - | - | - | 360 | 41.1 | 6.6 | Transport | Cytoplasm;Nucleus | Receptor signaling complex scaffold activity | Cytoplasm |

| Gene Symbol | RefSeq_Accession | Entrez Gene ID | Description | Coverage (%) | Proteins | Unique Peptides | # Peptides | PSM | Area | SCC-R /SCC-S | SCC-R /SCC-S (Count) | SCC-R /SCC-S (Variability [%]) | # AAs | MW [kDa] | calc. pI | Biological Process | Localization | Molecular Function | Primary Localization |
|---|---|---|---|---|---|---|---|---|---|---|---|---|---|---|---|---|---|---|---|
| **SNURF** | NP_073715.1 | 8926 | **SNRPN upstream reading frame protein** | 15.5 | 1 | 1 | 1 | 1 | 7.15E+07 | - | - | - | 71 | 8.4 | 10.6 | Biological_process unknown | Nucleus | Molecular function unknown | Nucleus |
| **SNX14** | NP_065201.1 | 57231 | **sorting nexin-14 isoform b** | 1.2 | 2 | 1 | 1 | 1 | 2.68E+06 | - | - | - | 893 | 103.7 | 6.9 | Transport | Endoplasmic reticulum | Transporter activity | Endoplasmic reticulum |
| **SNX33** | NP_695003.1 | 257364 | **sorting nexin-33** | 5.6 | 1 | 1 | 1 | 1 | 8.87E+06 | - | - | - | 574 | 65.2 | 6.8 | Cell communication;Signal transduction | - | Molecular function unknown | - |
| **SNX8** | NP_037453.1 | 29886 | **sorting nexin-8** | 5.6 | 1 | 1 | 1 | 1 | 8.64E+07 | - | - | - | 465 | 52.5 | 7.4 | Transport | - | Transporter activity | - |
| **SNX9** | NP_057308.1 | 51429 | **sorting nexin-9** | 6.2 | 1 | 1 | 1 | 2 | 9.66E+07 | - | - | - | 595 | 66.5 | 5.6 | Cell communication;Signal transduction | Plasma membrane;Cell surface;Golgi apparatus;Perinuclear region;Clathrin-coated vesicle | Receptor signaling complex scaffold activity | - |
| **SOX15** | NP_008873.1 | 6665 | **protein SOX-15** | 13.3 | 1 | 2 | 2 | 2 | 2.54E+07 | - | - | - | 233 | 25.2 | 9.8 | Regulation of nucleobase, nucleoside, nucleotide and nucleic acid metabolism | Nucleus | Transcription factor activity | Nucleus |
| **SP1** | NP_001238754.1 | 6667 | **transcription factor Sp1 isoform c** | 2.4 | 2 | 1 | 1 | 1 | 5.01E+07 | - | - | - | 737 | 75.8 | 8.1 | Regulation of nucleobase, nucleoside, nucleotide and nucleic acid metabolism | Nucleus | Transcription factor activity | Nucleus |
| **SP3** | NP_001017371.3 | 6670 | **transcription factor Sp3 isoform 2** | 3.9 | 3 | 1 | 1 | 2 | 1.67E+08 | - | - | - | 713 | 75.3 | 5.3 | Regulation of nucleobase, nucleoside, nucleotide and nucleic acid metabolism | Nucleus;Nuclear matrix | Transcription factor activity | Nucleus |
| **SPAG1** | NP_003105.2 | 6674 | **sperm-associated antigen 1** | 3.7 | 1 | 2 | 2 | 4 | 2.49E+07 | - | - | - | 926 | 103.6 | 6.9 | Reproduction | Plasma membrane;Cytoplasm | GTPase activity | Cytoplasm |
| **SPAG17** | NP_996879.1 | 200162 | **sperm-associated antigen 17** | 0.6 | 1 | 1 | 1 | 1 | 8.51E+07 | - | - | - | 2223 | 251.6 | 6.2 | Biological_process unknown | - | Molecular function unknown | - |
| **SPATA17** | NP_620151.1 | 128153 | **spermatogenesis-associated protein 17** | 4.4 | 1 | 1 | 1 | 1 | 1.63E+08 | - | - | - | 361 | 43.5 | 9.9 | Apoptosis | Cytoplasm;Nucleus | Protein binding | Cytoplasm |
| **SPATA31A3** | NP_001076593.1 | 727830 | **spermatogenesis-associated protein 31A3** | 3.1 | 1 | 1 | 1 | 1 | 0.00E+00 | - | - | - | 1347 | 148.6 | 8.7 | Biological_process unknown | - | Molecular function unknown | - |
| **SPATA31C2** | NP_001159609.1 | 645961 | **uncharacterized protein LOC645961** | 2.5 | 2 | 1 | 1 | 1 | 5.85E+08 | - | - | - | 1134 | 124.4 | 9.3 | - | - | - | - |
| **SPATA31E1** | NP_849150.3 | 286234 | **spermatogenesis-associated protein 31E1** | 1.0 | 1 | 1 | 2 | 7 | 9.64E+08 | - | - | - | 1445 | 157 | 9.1 | Biological_process unknown | - | Molecular function unknown | - |
| **SPEF2** | NP_079143.3 | 79925 | **sperm flagellar protein 2 isoform 1** | 0.3 | 1 | 1 | 1 | 1 | 5.58E+07 | - | - | - | 1822 | 209.7 | 5.5 | Biological_process unknown | - | Molecular function unknown | - |
| **SPG21** | NP_001121362.1 | 51324 | **maspardin isoform b** | 6.4 | 2 | 1 | 1 | 2 | 2.01E+07 | - | - | - | 281 | 31.6 | 6.4 | Cell growth and/or maintenance | Cytoplasm;Endosome;Golgi apparatus | Structural constituent of cytoskeleton | Cytoplasm |
| **SPRY4** | NP_001120968.1 | 81848 | **protein sprouty homolog 4 isoform 2** | 15.4 | 2 | 2 | 2 | 2 | 3.35E+07 | - | - | - | 299 | 32.5 | 7.9 | Cell communication;Signal transduction | Cytoplasm | Molecular function unknown | Cytoplasm |
| **SRBD1** | NP_060549.4 | 55133 | **S1 RNA-binding domain-containing protein 1** | 7.2 | 1 | 3 | 3 | 3 | 7.45E+07 | - | - | - | 995 | 111.7 | 8.7 | Biological_process unknown | Cytoplasm | Molecular function unknown | Cytoplasm |
| **SRFBP1** | NP_689759.2 | 153443 | **serum response factor-binding protein 1** | 3.5 | 1 | 1 | 1 | 1 | 5.90E+06 | - | - | - | 429 | 48.6 | 9.6 | Metabolism | Nucleus;Cytosol;Perinuclear region;Cytoplasm | Transcription regulator activity | Nucleus |
| **SRGAP1** | NP_065813.1 | 57522 | **SLIT-ROBO Rho GTPase-activating protein 1** | 3.1 | 1 | 1 | 2 | 3 | 6.95E+07 | - | - | - | 1085 | 124.2 | 6.8 | Cell communication;Signal transduction | Cytoplasm;Nucleus | GTPase activator activity | Cytoplasm |
| **SSFA2** | NP_001274433.1 | 6744 | **sperm-specific antigen 2 isoform 4** | 4.0 | 4 | 2 | 2 | 2 | 4.61E+06 | - | - | - | 1084 | 119.1 | 5.3 | Biological_process unknown | Plasma membrane;Nucleus;Cytoplasm | Molecular function unknown | Plasma membrane |
| **ST14** | NP_068813.1 | 6768 | **suppressor of tumorigenicity 14 protein** | 1.8 | 1 | 1 | 1 | 1 | 3.68E+07 | - | - | - | 855 | 94.7 | 6.6 | Protein metabolism | Plasma membrane | Serine-type peptidase activity | Plasma membrane |
| **STARD10** | NP_006636.2 | 10809 | **PCTP-like protein** | 7.2 | 1 | 1 | 1 | 2 | 8.56E+06 | - | - | - | 291 | 33 | 7.1 | Biological_process unknown | - | Molecular function unknown | - |
| **STARD7** | NP_064536.2 | 56910 | **stAR-related lipid transfer protein 7, mitochondrial precursor** | 6.2 | 1 | 1 | 1 | 1 | 9.23E+07 | - | - | - | 370 | 43.1 | 9.0 | Signal transduction | - | Lipid binding | - |
| **STARD9** | NP_065810.2 | 57519 | **stAR-related lipid transfer protein 9** | 0.6 | 1 | 2 | 3 | 4 | 2.91E+08 | - | - | - | 4700 | 516 | 6.3 | Biological_process unknown | - | Molecular function unknown | - |
| **STAT5B** | NP_036580.2 | 6777 | **signal transducer and activator of transcription 5B** | 3.8 | 1 | 1 | 1 | 1 | 0.00E+00 | - | - | - | 787 | 89.8 | 6.1 | Regulation of nucleobase, nucleoside, nucleotide and nucleic acid metabolism | Cytoplasm;Nucleus | Transcription factor activity | Cytoplasm |
| **STC2** | NP_003705.1 | 8614 | **stanniocalcin-2 precursor** | 2.3 | 1 | 1 | 1 | 1 | 4.50E+07 | - | - | - | 302 | 33.2 | 7.3 | Cell communication;Signal transduction | Extracellular | Hormone activity | Extracellular |
| **STON2** | NP_149095.2 | 85439 | **stonin-2 isoform 1** | 1.0 | 2 | 1 | 1 | 2 | 2.13E+07 | - | - | - | 905 | 101.1 | 5.4 | Biological_process unknown | Cytoplasm;Plasma membrane | Molecular function unknown | Plasma membrane |
| **STRN3** | NP_001077362.1 | 29966 | **striatin-3 isoform 1** | 26.4 | 1 | 1 | 10 | 13 | 4.89E+08 | - | - | - | 797 | 87.2 | 5.4 | Cell communication;Signal transduction | Nucleus;Cytoplasm;Integral to membrane | Calcium ion binding | Nucleus |
| **STRN3** | NP_055389.3 | 29966 | **striatin-3 isoform 2** | 28.3 | 1 | 1 | 10 | 13 | 1.23E+08 | - | - | - | 713 | 77.7 | 5.3 | Cell communication;Signal transduction | Nucleus;Cytoplasm;Integral to membrane | Calcium ion binding | Nucleus |
| **STRN4** | NP_037535.2 | 29888 | **striatin-4 isoform 1** | 5.4 | 2 | 1 | 1 | 1 | 4.36E+06 | - | - | - | 753 | 80.5 | 5.4 | Cell communication;Signal transduction | Cytoplasm | Molecular function unknown | Cytoplasm |
| **STT3B** | NP_849193.1 | 201595 | **dolichyl-diphosphooligosaccharide--protein glycosyltransferase subunit STT3B** | 3.3 | 1 | 1 | 1 | 1 | 1.17E+07 | - | - | - | 826 | 93.6 | 8.9 | Biological_process unknown | Integral to membrane;Mitochondrion;Nucleus;Cytoplasm | Catalytic activity;Phosphoprotein phosphatase activity | Integral to membrane |
| **STX3** | NP_001171511.1 | 6809 | **syntaxin-3 isoform 2** | 10.8 | 2 | 1 | 1 | 1 | 3.39E+06 | - | - | - | 277 | 32 | 5.5 | Transport | Plasma membrane | Transporter activity | Plasma membrane |
| **STXBP5L** | NP_055795.1 | 9515 | **syntaxin-binding protein 5-like** | 2.5 | 1 | 1 | 1 | 1 | 9.87E+07 | - | - | - | 1186 | 131.8 | 7.0 | Transport | - | Transporter activity | - |
| **SUN1** | NP_001165415.1 | 23353 | **SUN domain-containing protein 1 isoform c** | 1.8 | 3 | 1 | 1 | 1 | 4.42E+07 | - | - | - | 682 | 76.4 | 6.6 | Cell growth and/or maintenance | Nucleus;Cytoplasm | Intracellular transporter activity | Nucleus |
| **SUPV3L1** | NP_003162.2 | 6832 | **ATP-dependent RNA helicase SUPV3L1, mitochondrial precursor** | 3.8 | 1 | 2 | 2 | 5 | 5.90E+07 | - | - | - | 786 | 87.9 | 8.0 | Regulation of nucleobase, nucleoside, nucleotide and nucleic acid metabolism | Mitochondrion;Cytoplasm | RNA binding | Mitochondrion |
| **SURF1** | NP_003163.1 | 6834 | **surfeit locus protein 1 isoform 1** | 5.3 | 1 | 1 | 1 | 2 | 2.76E+07 | - | - | - | 300 | 33.3 | 9.6 | Metabolism;Energy pathways | Mitochondrion | Transporter activity | Mitochondrion |

| Gene Symbol | RefSeq_Accession | Entrez Gene ID | Description | Coverage (%) | Proteins | Unique Peptides | # Peptides | PSM | Area | SCC-R /SCC-S | SCC-R /SCC-S (Count) | SCC-R /SCC-S (Variability [%]) | # AAs | MW [kDa] | calc. pI | Biological Process | Localization | Molecular Function | Primary Localization |
|---|---|---|---|---|---|---|---|---|---|---|---|---|---|---|---|---|---|---|---|
| SVOPL | NP_777619.1 | 136306 | putative transporter SVOPL isoform 2 | 2.7 | 1 | 1 | 1 | 2 | 2.32E+06 | - | - | - | 340 | 36.9 | 9.2 | Transport | - | Molecular function unknown | - |
| SYN1 | NP_598006.1 | 6853 | synapsin-1 isoform Ib | 3.6 | 2 | 1 | 1 | 1 | 6.63E+06 | - | - | - | 669 | 70 | 9.9 | Transport | Cytoplasm | Transporter activity | Cytoplasm |
| SYNCRIP | NP_001153148.1 | 10492 | heterogeneous nuclear ribonucleoprotein Q isoform 5 | 55.6 | 5 | 1 | 29 | 173 | 3.33E+09 | - | - | - | 561 | 62.5 | 7.6 | Regulation of nucleobase, nucleoside, nucleotide and nucleic acid metabolism | Cytoplasm;Nucleus;Endoplasmic reticulum;Ribosome;Nucleolus | RNA binding | Cytoplasm |
| SYT11 | NP_689493.3 | 23208 | synaptotagmin-11 | 5.3 | 1 | 1 | 1 | 1 | 2.18E+07 | - | - | - | 431 | 48.3 | 9.0 | Cell communication;Signal transduction | Cytoplasmic vesicle;Cytoplasm | Calcium ion binding | Cytoplasmic vesicle |
| SYT4 | NP_065834.1 | 6860 | synaptotagmin-4 | 2.1 | 1 | 1 | 1 | 1 | 5.77E+07 | - | - | - | 425 | 47.9 | 8.5 | Cell communication;Signal transduction | Cytoplasmic vesicle | Calcium ion binding | Cytoplasmic vesicle |
| TAF7 | NP_005633.2 | 6879 | transcription initiation factor TFIID subunit 7 | 4.6 | 1 | 1 | 1 | 1 | 0.00E+00 | - | - | - | 349 | 40.2 | 5.2 | Regulation of nucleobase, nucleoside, nucleotide and nucleic acid metabolism | Nucleus;Cytoplasm | Transcription factor activity | Nucleus |
| TAF9 | NP_003178.1 | 6880 | transcription initiation factor TFIID subunit 9 | 5.3 | 1 | 1 | 1 | 2 | 5.17E+07 | - | - | - | 264 | 29 | 8.7 | Transcription;Energy pathways | Nucleus;Nucleoplasm | Transcription regulator activity | Nucleoplasm |
| TAF9B | NP_057059.2 | 51616 | transcription initiation factor TFIID subunit 9B | 10.4 | 1 | 1 | 1 | 1 | 1.17E+07 | - | - | - | 251 | 27.6 | 9.6 | Regulation of nucleobase, nucleoside, nucleotide and nucleic acid metabolism | Nucleus | Transcription regulator activity | Nucleus |
| TANC1 | NP_203752.2 | 85461 | protein TANC1 isoform 1 | 0.6 | 1 | 1 | 1 | 1 | 3.45E+07 | - | - | - | 1861 | 202.1 | 8.3 | Biological_process unknown | Extracellular | Molecular function unknown | Extracellular |
| TANK | NP_597841.1 | 10010 | TRAF family member-associated NF-kappa-B activator isoform b | 21.9 | 2 | 1 | 1 | 1 | 0.00E+00 | - | - | - | 119 | 13.6 | 5.0 | Signal transduction | Cytoplasm | Receptor signaling complex scaffold activity | Cytoplasm |
| TAOK1 | NP_079418.1 | 57551 | serine/threonine-protein kinase TAO1 isoform 2 | 1.5 | 2 | 1 | 1 | 2 | 7.85E+07 | - | - | - | 853 | 97.5 | 6.9 | Cell communication;Signal transduction | Nucleus | Protein serine/threonine kinase activity | Nucleus |
| TBC1D22A | NP_001271234.1 | 25771 | TBC1 domain family member 22A isoform d | 6.2 | 3 | 1 | 1 | 1 | 0.00E+00 | - | - | - | 470 | 53.9 | 5.4 | Cell communication;Signal transduction | Nucleus | GTPase activator activity | Nucleus |
| TBC1D23 | NP_060779.2 | 55773 | TBC1 domain family member 23 isoform 2 | 7.3 | 2 | 2 | 2 | 2 | 1.67E+06 | - | - | - | 684 | 76.5 | 5.4 | Biological_process unknown | - | Molecular function unknown | - |
| TBL1Y | NP_599020.1 | 90665 | F-box-like/WD repeat-containing protein TBL1Y | 11.5 | 1 | 1 | 3 | 4 | 6.30E+07 | - | - | - | 522 | 56.7 | 5.6 | Regulation of nucleobase, nucleoside, nucleotide and nucleic acid metabolism | Nucleus | Transcription regulator activity | Nucleus |
| TCN2 | NP_001171655.1 | 6948 | transcobalamin-2 isoform 2 precursor | 10.5 | 2 | 1 | 1 | 1 | 4.05E+09 | - | - | - | 400 | 44.4 | 6.9 | Transport | Extracellular | Transporter activity | Extracellular |
| TCOF1 | NP_001128717.1 | 6949 | treacle protein isoform f | 20.5 | 4 | 2 | 21 | 54 | 4.06E+08 | - | - | - | 1412 | 144.3 | 9.0 | Transcription;Cell migration;Ribosome biogenesis and assembly | Nucleoplasm;Nucleolus;Cytoplasm;Nucleus | Transcription regulator activity | Nucleolus |
| TCP11L1 | NP_060863.3 | 55346 | T-complex protein 11-like protein 1 | 6.9 | 1 | 2 | 2 | 2 | 5.83E+07 | - | - | - | 509 | 57 | 5.6 | Biological_process unknown | - | Molecular function unknown | - |
| TDRD7 | NP_055105.2 | 23424 | tudor domain-containing protein 7 | 5.6 | 1 | 3 | 3 | 3 | 2.36E+07 | - | - | - | 1098 | 123.5 | 7.2 | Cell communication;Signal transduction | Mitochondrion;Cytoplasm;Nucleus | Receptor signaling complex scaffold activity | Mitochondrion |
| TDRD9 | NP_694591.2 | 122402 | putative ATP-dependent RNA helicase TDRD9 | 1.5 | 1 | 1 | 1 | 1 | 0.00E+00 | - | - | - | 1382 | 155.6 | 7.1 | Regulation of nucleobase, nucleoside, nucleotide and nucleic acid metabolism | - | RNA binding | - |
| TDRKH | NP_001077433.1 | 11022 | tudor and KH domain-containing protein isoform b | 2.3 | 2 | 1 | 1 | 3 | 1.41E+07 | - | - | - | 516 | 57.2 | 4.9 | Regulation of nucleobase, nucleoside, nucleotide and nucleic acid metabolism | - | RNA binding | - |
| TEFM | NP_078959.3 | 79736 | transcription elongation factor, mitochondrial | 3.6 | 1 | 1 | 1 | 1 | 3.81E+07 | - | - | - | 360 | 41.6 | 9.3 | Biological_process unknown | - | Molecular function unknown | - |
| TERF2IP | NP_061848.2 | 54386 | telomeric repeat-binding factor 2-interacting protein 1 | 27.8 | 1 | 4 | 4 | 4 | 1.72E+08 | - | - | - | 399 | 44.2 | 4.7 | Cell communication;Signal transduction;Chromosome organization and biogenesis (sensu Eukarya) | Nucleus | DNA binding | Nucleus |
| TEX15 | NP_112561.2 | 56154 | testis-expressed sequence 15 protein | 0.7 | 1 | 1 | 2 | 3 | 2.37E+08 | - | - | - | 2789 | 315.1 | 6.2 | Biological_process unknown | - | Molecular function unknown | - |
| TFAP2A | NP_001027451.1 | 7020 | transcription factor AP-2-alpha isoform b | 8.4 | 5 | 2 | 2 | 2 | 9.95E+06 | - | - | - | 431 | 47.2 | 8.4 | Development;Apoptosis;Cell differentiation;Regulation of gene expression, epigenetic | Nucleus | Transcription factor activity | Nucleus |
| TFDP1 | NP_009042.1 | 7027 | transcription factor Dp-1 | 4.4 | 1 | 1 | 1 | 1 | 2.41E+06 | - | - | - | 410 | 45 | 6.1 | Regulation of nucleobase, nucleoside, nucleotide and nucleic acid metabolism | Nucleus | Transcription factor activity | Nucleus |
| TFIP11 | NP_001008697.1 | 24144 | tuftelin-interacting protein 11 | 1.8 | 1 | 1 | 1 | 1 | 3.18E+06 | - | - | - | 837 | 96.8 | 5.7 | Secretory pathway | Nucleus;Cytoplasm;Extracellular | Extracellular matrix structural constituent | Nucleus |
| TGFB1 | NP_000651.3 | 7040 | transforming growth factor beta-1 precursor | 4.6 | 1 | 1 | 1 | 1 | 6.86E+06 | - | - | - | 390 | 44.3 | 8.5 | Cell communication;Signal transduction | Extracellular | Receptor binding | Extracellular |
| THEM6 | NP_057731.1 | 51337 | protein THEM6 precursor | 5.3 | 1 | 1 | 1 | 1 | 2.20E+06 | - | - | - | 208 | 23.8 | 9.6 | Biological_process unknown | Secreted | Molecular function unknown | Secreted |
| THG1L | NP_060342.2 | 54974 | probable tRNA(His) guanylyltransferase | 3.4 | 1 | 1 | 1 | 1 | 2.17E+07 | - | - | - | 298 | 34.8 | 8.0 | Cell growth and/or maintenance | Cytoplasm;Centrosome;Nucleus | Molecular function unknown | Cytoplasm |
| TIAL1 | NP_001029097.1 | 7073 | nucleolysin TIAR isoform 2 | 35.5 | 1 | 1 | 8 | 23 | 3.38E+08 | - | - | - | 392 | 43.4 | 8.0 | Regulation of nucleobase, nucleoside, nucleotide and nucleic acid metabolism | Nucleus;Cytoplasm;Lysosome | RNA binding | Nucleus |
| TIAL1 | NP_003243.1 | 7073 | nucleolysin TIAR isoform 1 | 35.2 | 1 | 1 | 8 | 26 | 3.38E+08 | - | - | - | 375 | 41.6 | 7.7 | Regulation of nucleobase, nucleoside, nucleotide and nucleic acid metabolism | Nucleus;Cytoplasm;Lysosome | RNA binding | Nucleus |
| TIMM17A | NP_006326.1 | 10440 | mitochondrial import inner membrane translocase subunit Tim17-A | 19.3 | 1 | 1 | 1 | 1 | 1.93E+06 | - | - | - | 171 | 18 | 7.9 | Transport | Mitochondrion | Catalytic activity | Mitochondrion |
| TIMM8B | NP_036591.2 | 26521 | mitochondrial import inner membrane translocase subunit Tim8 B | 11.2 | 1 | 1 | 1 | 2 | 1.96E+07 | - | - | - | 98 | 11.1 | 8.1 | Transport | Mitochondrion | Auxiliary transport protein activity | Mitochondrion |
| TIMP1 | NP_003245.1 | 7076 | metalloproteinase inhibitor 1 precursor | 9.7 | 1 | 1 | 1 | 1 | 6.75E+07 | - | - | - | 207 | 23.2 | 8.1 | Cell growth and/or maintenance | Extracellular;Cell surface | Extracellular matrix structural constituent | Extracellular |
| TIPARP | NP_001171647.1 | 25976 | TCDD-inducible poly [ADP-ribose] polymerase | 4.3 | 1 | 1 | 1 | 1 | 0.00E+00 | - | - | - | 657 | 76.2 | 6.7 | Regulation of nucleobase, nucleoside, nucleotide and nucleic acid metabolism | - | DNA binding | - |
| TM9SF1 | NP_001014842.1 | 10548 | transmembrane 9 superfamily member 1 isoform b precursor | 7.2 | 2 | 2 | 2 | 2 | 4.87E+07 | - | - | - | 489 | 55.2 | 7.1 | Signal transduction | Plasma membrane | Receptor activity | Plasma membrane |
| TMA16 | NP_060822.2 | 55319 | translation machinery-associated protein 16 | 10.8 | 1 | 1 | 1 | 1 | 3.86E+07 | - | - | - | 203 | 23.8 | 9.3 | Biological_process unknown | - | Molecular function unknown | - |

| Gene Symbol | RefSeq_Accession | Entrez Gene ID | Description | Coverage (%) | Proteins | Unique Peptides | # Peptides | PSM | Area | SCC-R /SCC-S | SCC-R /SCC-S (Count) | SCC-R /SCC-S (Variability [%]) | # AAs | MW [kDa] | calc. pI | Biological Process | Localization | Molecular Function | Primary Localization |
|---|---|---|---|---|---|---|---|---|---|---|---|---|---|---|---|---|---|---|---|
| TMCO6 | NP_060972.3 | 55374 | transmembrane and coiled-coil domain-containing protein 6 | 5.9 | 1 | 1 | 1 | 1 | 3.65E+06 | - | - | - | 493 | 54.4 | 5.8 | Biological_process unknown | - | Molecular function unknown | - |
| TMEM106B | NP_001127704.1 | 54664 | transmembrane protein 106B | 4.7 | 1 | 1 | 1 | 1 | 2.19E+06 | - | - | - | 274 | 31.1 | 7.0 | Biological_process unknown | Integral to membrane | Molecular function unknown | Integral to membrane |
| TMEM11 | NP_003867.1 | 8834 | transmembrane protein 11, mitochondrial | 8.3 | 1 | 1 | 1 | 3 | 1.87E+08 | - | - | - | 192 | 21.5 | 7.4 | Cell communication;Signal transduction | Plasma membrane | Molecular function unknown | Plasma membrane |
| TMEM123 | NP_443164.2 | 114908 | porimin precursor | 4.3 | 1 | 1 | 1 | 1 | 2.32E+09 | - | - | - | 208 | 21.5 | 8.7 | Cell communication;Signal transduction | Plasma membrane | Receptor activity | Plasma membrane |
| TMEM126A | NP_115649.1 | 84233 | transmembrane protein 126A isoform 1 | 14.9 | 1 | 2 | 2 | 3 | 3.06E+07 | - | - | - | 195 | 21.5 | 9.3 | Biological_process unknown | - | Molecular function unknown | - |
| TMEM131 | NP_056163.1 | 23505 | transmembrane protein 131 | 1.2 | 1 | 1 | 1 | 1 | 2.87E+08 | - | - | - | 1883 | 205 | 8.6 | Biological_process unknown | - | Molecular function unknown | - |
| TMEM183A | NP_612400.3 | 92703 | transmembrane protein 183A | 5.3 | 2 | 1 | 1 | 1 | 6.84E+08 | - | - | - | 376 | 42.8 | 9.1 | Biological_process unknown | Integral to membrane | Molecular function unknown | Integral to membrane |
| TMEM194A | NP_056072.2 | 23306 | transmembrane protein 194A isoform b | 3.2 | 2 | 1 | 1 | 1 | 2.83E+06 | - | - | - | 371 | 42 | 6.2 | Biological_process unknown | - | Molecular function unknown | - |
| TMEM209 | NP_116231.2 | 84928 | transmembrane protein 209 | 2.9 | 1 | 1 | 1 | 1 | 7.06E+07 | - | - | - | 561 | 62.9 | 8.6 | Biological_process unknown | - | Molecular function unknown | - |
| TMEM63A | NP_055513.2 | 9725 | transmembrane protein 63A | 5.3 | 1 | 1 | 1 | 1 | 2.71E+08 | - | - | - | 807 | 92.1 | 7.3 | Biological_process unknown | Integral to membrane | Molecular function unknown | Integral to membrane |
| TMEM63B | NP_060896.1 | 55362 | transmembrane protein 63B | 1.1 | 1 | 1 | 1 | 1 | 1.68E+06 | - | - | - | 832 | 94.9 | 7.4 | Biological_process unknown | Integral to membrane;Kinetochore | Molecular function unknown | Integral to membrane |
| TMPPE | NP_001129710.1 | 643853 | transmembrane protein with metallophosphoesterase domain isoform 2 | 4.8 | 2 | 1 | 1 | 1 | 9.48E+06 | - | - | - | 316 | 34.1 | 6.5 | Biological_process unknown | - | Molecular function unknown | - |
| TNFAIP8 | NP_055165.2 | 25816 | tumor necrosis factor alpha-induced protein 8 isoform a | 9.6 | 1 | 1 | 1 | 1 | 6.96E+07 | - | - | - | 198 | 23 | 7.9 | Cell growth and/or maintenance | Cytoplasm | Molecular function unknown | Cytoplasm |
| TNIP1 | NP_001245385.1 | 10318 | TNFAIP3-interacting protein 1 isoform 6 | 5.4 | 6 | 1 | 1 | 1 | 4.22E+07 | - | - | - | 556 | 62.6 | 7.7 | Cell growth and/or maintenance | Cytoplasm;Nucleus | Cytoskeletal anchoring activity | Cytoplasm |
| TNK1 | NP_003976.2 | 8711 | non-receptor tyrosine-protein kinase TNK1 isoform 2 | 3.8 | 2 | 1 | 1 | 1 | 2.77E+07 | - | - | - | 661 | 71.9 | 9.5 | Cell communication;Signal transduction | Cytoplasm;Integral to membrane | Protein-tyrosine kinase activity | Cytoplasm |
| TOM1L1 | NP_005477.2 | 10040 | TOM1-like protein 1 | 6.9 | 1 | 1 | 1 | 1 | 0.00E+00 | - | - | - | 476 | 53 | 5.2 | Cell communication;Signal transduction | Endosome;Golgi apparatus;Lysosome;Cytoplasm | Receptor signaling complex scaffold activity | Endosome |
| TOMM7 | NP_061932.1 | 54543 | mitochondrial import receptor subunit TOM7 homolog | 30.9 | 1 | 1 | 1 | 1 | 3.43E+08 | - | - | - | 55 | 6.2 | 10.3 | Transport | Mitochondrion | Transporter activity | Mitochondrion |
| TOP3A | NP_004609.1 | 7156 | DNA topoisomerase 3-alpha | 1.2 | 1 | 1 | 1 | 1 | 1.15E+07 | - | - | - | 1001 | 112.3 | 8.3 | Regulation of nucleobase, nucleoside, nucleotide and nucleic acid metabolism | Nucleus;Mitochondrion;Nucleolus | DNA topoisomerase activity | Nucleus |
| TP63 | NP_001108452.1 | 8626 | tumor protein 63 isoform 4 | 6.8 | 6 | 3 | 3 | 4 | 7.45E+07 | - | - | - | 586 | 65.7 | 7.7 | Regulation of nucleobase, nucleoside, nucleotide and nucleic acid metabolism | Nucleus;Endoplasmic reticulum | Transcription factor activity | Nucleus |
| TPBG | NP_001159864.1 | 7162 | trophoblast glycoprotein precursor | 4.5 | 1 | 1 | 1 | 3 | 0.00E+00 | - | - | - | 420 | 46 | 6.8 | Cell growth and/or maintenance | Plasma membrane | Molecular function unknown | Plasma membrane |
| TPD52L2 | NP_955391.1 | 7165 | tumor protein D54 isoform f | 79.0 | 5 | 1 | 11 | 71 | 1.25E+09 | - | - | - | 186 | 19.9 | 5.5 | Cell growth and/or maintenance;Biological_process unknown | Cytoplasm;Nucleus | Molecular function unknown | Cytoplasm;Nucleus |
| TPP1 | NP_000382.3 | 1200 | tripeptidyl-peptidase 1 preproprotein | 11.6 | 1 | 2 | 2 | 5 | 2.37E+07 | - | - | - | 563 | 61.2 | 6.5 | Protein metabolism | Lysosome;Nucleus | Serine-type peptidase activity | Lysosome |
| TRANK1 | NP_055646.2 | 9881 | TPR and ankyrin repeat-containing protein 1 | 0.4 | 1 | 1 | 1 | 1 | 1.94E+08 | - | - | - | 2925 | 336 | 6.8 | Biological_process unknown | - | Molecular function unknown | - |
| TRAPPC5 | NP_001035927.1 | 126003 | trafficking protein particle complex subunit 5 | 14.4 | 1 | 1 | 1 | 2 | 0.00E+00 | - | - | - | 188 | 20.8 | 9.7 | Biological_process unknown | - | Molecular function unknown | - |
| TRIM24 | NP_003843.3 | 8805 | transcription intermediary factor 1-alpha isoform b | 0.9 | 2 | 1 | 1 | 1 | 0.00E+00 | - | - | - | 1016 | 112.9 | 6.8 | Regulation of nucleobase, nucleoside, nucleotide and nucleic acid metabolism | Nucleus;Cytoplasm | Transcription regulator activity | Nucleus;Cytoplasm |
| TRIM27 | NP_006501.1 | 5987 | zinc finger protein RFP | 5.3 | 1 | 1 | 1 | 1 | 9.62E+05 | - | - | - | 513 | 58.5 | 6.2 | Cell growth and/or maintenance;Cell differentiation;Apoptosis | Nucleus;Cytoplasm | DNA binding | Nucleus |
| TRIM3 | NP_001234936.1 | 10612 | tripartite motif-containing protein 3 isoform 2 | 3.2 | 2 | 1 | 1 | 1 | 0.00E+00 | - | - | - | 625 | 67.7 | 8.6 | Cell growth and/or maintenance | Cytoplasm | Metal ion binding | Cytoplasm |
| TRIML2 | NP_775824.1 | 205860 | probable E3 ubiquitin-protein ligase TRIML2 | 3.1 | 1 | 1 | 1 | 1 | 4.48E+06 | - | - | - | 387 | 44 | 5.7 | Biological_process unknown | - | Molecular function unknown | - |
| TRMT10B | NP_001273882.1 | 158234 | tRNA methyltransferase 10 homolog B isoform e | 5.1 | 1 | 1 | 1 | 1 | 2.43E+08 | - | - | - | 196 | 22.5 | 8.4 | Regulation of nucleobase, nucleoside, nucleotide and nucleic acid metabolism | - | RNA methyltransferase activity | - |
| TRMT13 | NP_061956.2 | 54482 | tRNA:m(4)X modification enzyme TRM13 homolog | 3.5 | 1 | 1 | 1 | 1 | 0.00E+00 | - | - | - | 481 | 54.2 | 8.1 | Biological_process unknown | - | Molecular function unknown | - |
| TRMT61B | NP_060380.3 | 55006 | tRNA (adenine(58)-N(1))-methyltransferase, mitochondrial precursor | 5.5 | 1 | 1 | 1 | 1 | 0.00E+00 | - | - | - | 477 | 52.9 | 6.6 | Metabolism;Energy pathways | - | Methyltransferase activity | - |
| TRNAU1AP | NP_060316.1 | 54952 | tRNA selenocysteine 1-associated protein 1 | 7.3 | 1 | 1 | 1 | 2 | 1.38E+06 | - | - | - | 287 | 32.5 | 4.7 | Regulation of nucleobase, nucleoside, nucleotide and nucleic acid metabolism | Nucleus;Cytoplasm | RNA binding | Nucleus |
| TRNP1 | NP_001013664.1 | 388610 | TMF-regulated nuclear protein 1 | 11.9 | 1 | 1 | 1 | 1 | 0.00E+00 | - | - | - | 227 | 23.4 | 11.5 | Biological_process unknown | - | Molecular function unknown | - |
| TRPM4 | NP_001182156.1 | 54795 | transient receptor potential cation channel subfamily M member 4 isoform 2 | 0.7 | 3 | 1 | 1 | 1 | 2.27E+07 | - | - | - | 1069 | 118.6 | 7.9 | Transport | Plasma membrane | Intracellular ligand-gated ion channel activity | Plasma membrane |
| TSKU | NP_001245139.1 | 25987 | tsukushin precursor | 10.2 | 1 | 1 | 1 | 1 | 2.45E+05 | - | - | - | 353 | 37.8 | 6.9 | Biological_process unknown | - | Molecular function unknown | - |
| TSPAN4 | NP_001020410.1 | 7106 | tetraspanin-4 isoform b | 14.4 | 2 | 1 | 1 | 1 | 1.33E+07 | - | - | - | 174 | 19.4 | 5.4 | Biological_process unknown | Cell surface | Molecular function unknown | Cell surface |
| TSR2 | NP_477511.1 | 90121 | pre-rRNA-processing protein TSR2 homolog | 15.2 | 1 | 1 | 1 | 5 | 9.94E+07 | - | - | - | 191 | 20.9 | 4.4 | Biological_process unknown | - | Molecular function unknown | - |

| Gene Symbol | RefSeq_Accession | Entrez Gene ID | Description | Coverage (%) | Proteins | Unique Peptides | # Peptides | PSM | Area | SCC-R /SCC-S | SCC-R /SCC-S (Count) | SCC-R /SCC-S (Variability [%]) | # AAs | MW [kDa] | calc. pI | Biological Process | Localization | Molecular Function | Primary Localization |
|---|---|---|---|---|---|---|---|---|---|---|---|---|---|---|---|---|---|---|---|
| **TSSC4** | NP_005697.2 | 10078 | **protein TSSC4** | 4.0 | 1 | 1 | 1 | 1 | 9.40E+07 | **-** | - | - | 329 | 34.3 | 5.2 | Biological_process unknown | Cytoplasm | Molecular function unknown | Cytoplasm |
| **TTC21A** | NP_001098983.2 | 199223 | **tetratricopeptide repeat protein 21A isoform 1** | 0.7 | 2 | 1 | 1 | 1 | 1.92E+08 | **-** | - | - | 1272 | 145.5 | 7.1 | Cell communication;Signal transduction | - | Molecular function unknown | - |
| **TTC25** | NP_113609.1 | 83538 | **tetratricopeptide repeat protein 25** | 1.0 | 1 | 1 | 1 | 3 | 2.50E+08 | **-** | - | - | 672 | 76.6 | 5.6 | Cell communication;Signal transduction | Cytoplasm;Nucleus | Molecular function unknown | Cytoplasm |
| **TTC28** | NP_001138890.1 | 23331 | **tetratricopeptide repeat protein 28** | 3.3 | 1 | 4 | 4 | 6 | 1.55E+07 | **-** | - | - | 2481 | 270.7 | 6.9 | Biological_process unknown | - | Molecular function unknown | - |
| **TTK** | NP_001160163.1 | 7272 | **dual specificity protein kinase TTK isoform 2** | 1.5 | 2 | 1 | 1 | 1 | 4.26E+06 | **-** | - | - | 856 | 96.9 | 8.2 | Cell communication;Signal transduction | Centrosome | Protein threonine/tyrosine kinase activity | Centrosome |
| **TTN** | NP_001254479.1 | 7273 | **titin isoform IC** | 0.0 | 1 | 1 | 1 | 1 | 3.13E+07 | **-** | - | - | 35991 | 3992.1 | 6.4 | Transport;Muscle development;Muscle contraction | Cytoplasm;Nucleus;Sarcoplasmic reticulum | Structural molecule activity;Kinase activity | Cytoplasm |
| **TUBA3C** | NP_005992.1 | 7278 | **tubulin alpha-3C/D chain** | 62.9 | 3 | 1 | 24 | 1095 | 1.57E+10 | **-** | - | - | 450 | 49.9 | 5.1 | Cell growth and/or maintenance | Cytoplasm | Structural molecule activity | Cytoplasm |
| **TUBA8** | NP_001180343.1 | 51807 | **tubulin alpha-8 chain isoform 2** | 42.0 | 3 | 0 | 14 | 594 | 1.40E+10 | **-** | - | - | 383 | 42.9 | 5.1 | Cell growth and/or maintenance | Cytoskeleton;Microtubule | Cytoskeletal protein binding | Cytoskeleton |
| **TUBB4A** | NP_006078.2 | 10382 | **tubulin beta-4A chain isoform 3** | 77.7 | 4 | 2 | 29 | 1140 | 1.50E+10 | **-** | - | - | 444 | 49.6 | 4.9 | Cell growth and/or maintenance | Microtubule | Structural constituent of cytoskeleton | Microtubule |
| **TUBE1** | NP_057346.1 | 51175 | **tubulin epsilon chain** | 4.8 | 1 | 1 | 1 | 1 | 8.61E+07 | **-** | - | - | 475 | 52.9 | 6.6 | Cell growth and/or maintenance | Centrosome | Structural constituent of cytoskeleton | Centrosome |
| **TUBGCP6** | NP_065194.2 | 85378 | **gamma-tubulin complex component 6** | 1.4 | 1 | 1 | 1 | 1 | 0.00E+00 | **-** | - | - | 1819 | 200.4 | 6.3 | Cell growth and/or maintenance | Centrosome;Microtubule | Structural constituent of cytoskeleton | Centrosome |
| **TUSC2** | NP_009206.1 | 11334 | **tumor suppressor candidate 2** | 10.9 | 1 | 1 | 1 | 1 | 1.83E+06 | **-** | - | - | 110 | 12.1 | 9.7 | Cell communication;Signal transduction | Cytoplasm | Molecular function unknown | Cytoplasm |
| **TXNRD1** | NP_877419.1 | 7296 | **thioredoxin reductase 1, cytoplasmic isoform 2** | 68.7 | 1 | 1 | 24 | 135 | 1.93E+09 | **-** | - | - | 499 | 54.7 | 6.5 | Metabolism;Energy pathways | Cytoplasm;Extracellular;Plasma membrane;Nucleus | Oxidoreductase activity | Cytoplasm |
| **TXNRD1** | NP_001248375.1 | 7296 | **thioredoxin reductase 1, cytoplasmic isoform 5** | 67.7 | 1 | 1 | 22 | 121 | 1.93E+09 | **-** | - | - | 461 | 50.8 | 6.6 | Metabolism;Energy pathways | Cytoplasm;Extracellular;Plasma membrane;Nucleus | Oxidoreductase activity | Cytoplasm |
| **TYSND1** | NP_001035363.1 | 219743 | **peroxisomal leader peptide-processing protease isoform b** | 4.3 | 2 | 1 | 1 | 1 | 1.33E+07 | **-** | - | - | 398 | 41.5 | 7.1 | Biological_process unknown | - | Molecular function unknown | - |
| **UAP1L1** | NP_997192.2 | 91373 | **UDP-N-acetylhexosamine pyrophosphorylase-like protein 1** | 2.0 | 1 | 1 | 1 | 1 | 1.27E+07 | **-** | - | - | 507 | 57 | 6.3 | Metabolism;Energy pathways | - | Catalytic activity | - |
| **UBA7** | NP_003326.2 | 7318 | **ubiquitin-like modifier-activating enzyme 7** | 2.7 | 1 | 1 | 1 | 1 | 1.89E+07 | **-** | - | - | 1012 | 111.6 | 6.0 | Protein metabolism | Cytoplasm;Nucleus | Ubiquitin-specific protease activity | Cytoplasm |
| **UBAC2** | NP_001137544.1 | 337867 | **ubiquitin-associated domain-containing protein 2 isoform 1 precursor** | 3.2 | 1 | 1 | 1 | 1 | 3.21E+07 | **-** | - | - | 344 | 38.9 | 9.0 | Biological_process unknown | - | Molecular function unknown | - |
| **UBAP1** | NP_001164675.1 | 51271 | **ubiquitin-associated protein 1 isoform 1** | 4.0 | 3 | 1 | 1 | 2 | 5.19E+07 | **-** | - | - | 502 | 55 | 5.1 | Biological_process unknown | - | Molecular function unknown | - |
| **UBE2G2** | NP_001189418.1 | 7327 | **ubiquitin-conjugating enzyme E2 G2 isoform 3** | 48.4 | 3 | 2 | 2 | 3 | 2.22E+07 | **-** | - | - | 95 | 10.8 | 5.2 | Protein metabolism | Nucleus;Cytoplasm;Endoplasmic reticulum | Ubiquitin-specific protease activity | Nucleus |
| **UBE2V1** | NP_001244324.1 | 7335 | **ubiquitin-conjugating enzyme E2 variant 1 isoform e** | 63.2 | 9 | 4 | 9 | 38 | 5.78E+08 | **-** | - | - | 144 | 16.2 | 6.7 | Cell differentiation | Nucleus;Cytoplasm | Transcription factor activity | Nucleus |
| **UBL7** | NP_957717.1 | 84993 | **ubiquitin-like protein 7 isoform a** | 6.1 | 2 | 1 | 1 | 1 | 0.00E+00 | **-** | - | - | 380 | 40.5 | 5.1 | Protein metabolism | - | Ubiquitin-like-protein-specific protease activity | - |
| **UBXN6** | NP_079517.1 | 80700 | **UBX domain-containing protein 6 isoform 1** | 15.7 | 2 | 3 | 3 | 3 | 1.42E+07 | **-** | - | - | 441 | 49.7 | 6.9 | Biological_process unknown | Cytoplasm;Membrane fraction;Nucleus;Centrosome | Molecular function unknown | Cytoplasm;Membrane fraction |
| **UCK1** | NP_001248379.1 | 83549 | **uridine-cytidine kinase 1 isoform c** | 12.7 | 3 | 1 | 2 | 2 | 3.72E+07 | **-** | - | - | 268 | 30.7 | 7.1 | Metabolism;Energy pathways | - | Phosphorylase activity | - |
| **UFC1** | NP_057490.2 | 51506 | **ubiquitin-fold modifier-conjugating enzyme 1** | 5.4 | 1 | 1 | 1 | 2 | 1.24E+08 | **-** | - | - | 167 | 19.4 | 7.4 | Protein metabolism | - | Molecular function unknown | - |
| **UHRF2** | NP_690856.1 | 115426 | **E3 ubiquitin-protein ligase UHRF2** | 1.9 | 1 | 1 | 1 | 1 | 2.46E+06 | **-** | - | - | 802 | 89.9 | 8.2 | Protein metabolism | Nucleus | Ubiquitin-specific protease activity | Nucleus |
| **UNC119** | NP_473376.1 | 9094 | **protein unc-119 homolog A isoform b** | 9.1 | 2 | 1 | 1 | 1 | 4.73E+06 | **-** | - | - | 220 | 24.1 | 7.3 | Cell communication;Signal transduction | Cytoplasm;Plasma membrane | Molecular function unknown;Kinase regulator activity | Cytoplasm |
| **UNC119B** | NP_001074002.1 | 84747 | **protein unc-119 homolog B** | 8.0 | 1 | 1 | 1 | 1 | 4.32E+06 | **-** | - | - | 251 | 28.1 | 5.7 | Biological_process unknown | - | Molecular function unknown | - |
| **UPF3A** | NP_542418.1 | 65110 | **regulator of nonsense transcripts 3A isoform hUpf3pdelta** | 4.7 | 2 | 1 | 1 | 1 | 0.00E+00 | **-** | - | - | 443 | 51 | 8.8 | Regulation of nucleobase, nucleoside, nucleotide and nucleic acid metabolism | Cytoplasm;Nucleus | RNA binding | Nucleus |
| **UPRT** | NP_659489.1 | 139596 | **uracil phosphoribosyltransferase homolog** | 3.6 | 1 | 1 | 1 | 1 | 3.03E+06 | **-** | - | - | 309 | 33.8 | 6.0 | Biological_process unknown | - | Molecular function unknown | - |
| **USP11** | NP_004642.2 | 8237 | **ubiquitin carboxyl-terminal hydrolase 11** | 1.3 | 1 | 1 | 1 | 1 | 0.00E+00 | **-** | - | - | 963 | 109.7 | 5.5 | Protein metabolism | Nucleus | Ubiquitin-specific protease activity | Nucleus |
| **USP18** | NP_059110.2 | 11274 | **ubl carboxyl-terminal hydrolase 18** | 9.1 | 1 | 2 | 2 | 4 | 4.57E+07 | **-** | - | - | 372 | 43 | 7.8 | Cell communication;Signal transduction | Nucleus | Ubiquitin-specific protease activity | Nucleus |
| **USP19** | NP_006668.1 | 10869 | **ubiquitin carboxyl-terminal hydrolase 19 isoform 4** | 2.2 | 4 | 1 | 1 | 1 | 2.41E+06 | **-** | - | - | 1318 | 145.6 | 6.3 | Protein metabolism | - | Ubiquitin-specific protease activity | - |
| **USP25** | NP_037528.3 | 29761 | **ubiquitin carboxyl-terminal hydrolase 25 isoform USP25a** | 1.2 | 3 | 1 | 1 | 1 | 1.43E+07 | **-** | - | - | 1055 | 122.1 | 5.3 | Protein metabolism | Cytoplasm | Ubiquitin-specific protease activity | Cytoplasm |
| **USP28** | NP_065937.1 | 57646 | **ubiquitin carboxyl-terminal hydrolase 28** | 1.4 | 1 | 1 | 1 | 1 | 3.28E+07 | **-** | - | - | 1077 | 122.4 | 5.2 | Protein metabolism | - | Ubiquitin-specific protease activity | - |
| **USP38** | NP_001277255.1 | 84640 | **ubiquitin carboxyl-terminal hydrolase 38 isoform 3** | 2.2 | 3 | 1 | 1 | 1 | 9.09E+05 | **-** | - | - | 587 | 65.1 | 5.6 | Protein metabolism | - | Ubiquitin-specific protease activity | - |
| **USP4** | NP_955475.1 | 7375 | **ubiquitin carboxyl-terminal hydrolase 4 isoform b** | 2.0 | 2 | 1 | 1 | 1 | 1.21E+06 | **-** | - | - | 916 | 103.9 | 5.5 | Protein metabolism | Cytoplasm;Nucleus | Ubiquitin-specific protease activity | Cytoplasm;Nucleus |

| Gene Symbol | RefSeq_Accession | Entrez Gene ID | Description | Coverage (%) | Proteins | Unique Peptides | # Peptides | PSM | Area | SCC-R /SCC-S | SCC-R /SCC-S (Count) | SCC-R /SCC-S (Variability [%]) | # AAs | MW [kDa] | calc. pI | Biological Process | Localization | Molecular Function | Primary Localization |
|---|---|---|---|---|---|---|---|---|---|---|---|---|---|---|---|---|---|---|---|
| USP43 | NP_001254505.1 | 124739 | ubiquitin carboxyl-terminal hydrolase 43 isoform 2 | 1.3 | 2 | 1 | 1 | 1 | 1.72E+07 | - | - | - | 1118 | 122.2 | 9.2 | Proteolysis and peptidolysis | - | Ubiquitin-like-protein-specific protease activity | - |
| UTY | NP_872601.1 | 7404 | histone demethylase UTY isoform 1 | 2.3 | 25 | 1 | 1 | 1 | 4.12E+08 | - | - | - | 1079 | 118 | 7.9 | Immune response | - | Molecular function unknown | - |
| UVSSA | NP_065945.2 | 57654 | UV-stimulated scaffold protein A | 2.4 | 1 | 1 | 1 | 2 | 3.42E+08 | - | - | - | 709 | 80.5 | 6.3 | Biological_process unknown | - | Molecular function unknown | - |
| UXS1 | NP_001240805.1 | 80146 | UDP-glucuronic acid decarboxylase 1 isoform 3 | 9.9 | 3 | 1 | 1 | 1 | 3.56E+07 | - | - | - | 252 | 28.3 | 9.0 | Metabolism;Energy pathways | Golgi apparatus | Carboxy-lyase activity | Golgi apparatus |
| VAMP2 | NP_055047.2 | 6844 | vesicle-associated membrane protein 2 | 34.5 | 4 | 1 | 4 | 18 | 6.18E+08 | - | - | - | 116 | 12.7 | 8.1 | Transport | Plasma membrane;Golgi apparatus;Endosome;Zymogen granule | Auxiliary transport protein activity | Plasma membrane |
| VKORC1 | NP_996560.1 | 79001 | vitamin K epoxide reductase complex subunit 1 isoform 2 precursor | 14.1 | 2 | 1 | 1 | 4 | 2.30E+07 | - | - | - | 92 | 9.9 | 8.4 | Protein metabolism;Vitamin metabolism | Endoplasmic reticulum | Catalytic activity | Endoplasmic reticulum |
| VN1R5 | NP_776257.1 | 317705 | vomeronasal type-1 receptor 5 | 2.0 | 1 | 1 | 1 | 1 | 7.09E+07 | - | - | - | 357 | 40.8 | 9.2 | Cell communication;Signal transduction | Integral to membrane | G-protein coupled receptor activity | Integral to membrane |
| VPS13A | NP_001018048.1 | 23230 | vacuolar protein sorting-associated protein 13A isoform D | 0.8 | 4 | 1 | 1 | 2 | 7.73E+07 | - | - | - | 3069 | 347.5 | 6.2 | Protein metabolism;Transport | - | Transporter activity | - |
| VPS18 | NP_065908.1 | 57617 | vacuolar protein sorting-associated protein 18 homolog | 1.3 | 1 | 1 | 1 | 1 | 0.00E+00 | - | - | - | 973 | 110.1 | 6.1 | Transport | Cytoplasm;Lysosome | Transporter activity | Cytoplasm |
| VPS33B | NP_061138.3 | 26276 | vacuolar protein sorting-associated protein 33B isoform 1 | 2.3 | 1 | 1 | 1 | 1 | 7.84E+06 | - | - | - | 617 | 70.5 | 6.7 | Transport | Cytoplasm | Transporter activity | Cytoplasm |
| VTN | NP_000629.3 | 7448 | vitronectin precursor | 9.0 | 1 | 2 | 2 | 12 | 1.05E+09 | - | - | - | 478 | 54.3 | 5.8 | Cell growth and/or maintenance | Extracellular | Extracellular matrix structural constituent | Extracellular |
| WBP4 | NP_009118.1 | 11193 | WW domain-binding protein 4 | 4.5 | 1 | 1 | 1 | 1 | 2.28E+06 | - | - | - | 376 | 42.5 | 5.6 | Regulation of nucleobase, nucleoside, nucleotide and nucleic acid metabolism | Nucleus | RNA binding | Nucleus |
| WDFY4 | NP_065996.1 | 57705 | WD repeat- and FYVE domain-containing protein 4 | 0.6 | 1 | 1 | 1 | 1 | 1.37E+08 | - | - | - | 3184 | 353.4 | 6.3 | Biological_process unknown | - | Binding | - |
| WDR41 | NP_060738.2 | 55255 | WD repeat-containing protein 41 | 4.8 | 1 | 1 | 1 | 1 | 7.30E+07 | - | - | - | 459 | 51.7 | 5.4 | Biological_process unknown | - | Molecular function unknown | - |
| WDR48 | NP_065890.1 | 57599 | WD repeat-containing protein 48 | 9.2 | 1 | 3 | 3 | 3 | 1.89E+07 | - | - | - | 677 | 76.2 | 7.0 | Cell communication;Signal transduction | Endosome;Lysosome;Cytoplasm | Molecular function unknown | Endosome |
| WDR59 | NP_085058.3 | 79726 | WD repeat-containing protein 59 | 1.0 | 1 | 1 | 1 | 1 | 0.00E+00 | - | - | - | 974 | 109.7 | 7.9 | Biological_process unknown | - | Molecular function unknown | - |
| WDR62 | NP_001077430.1 | 284403 | WD repeat-containing protein 62 isoform 1 | 2.6 | 2 | 2 | 2 | 2 | 4.79E+06 | - | - | - | 1523 | 166.4 | 5.9 | Biological_process unknown | Cytoplasm | Molecular function unknown | Cytoplasm |
| WDR73 | NP_116245.2 | 84942 | WD repeat-containing protein 73 | 6.4 | 1 | 1 | 1 | 1 | 1.92E+07 | - | - | - | 378 | 41.7 | 5.8 | Biological_process unknown | - | Molecular function unknown | - |
| WDR87 | NP_114157.4 | 83889 | WD repeat-containing protein 87 isoform 2 | 0.4 | 2 | 1 | 1 | 1 | 9.19E+07 | - | - | - | 2873 | 333 | 7.3 | Cell communication;Signal transduction | - | Molecular function unknown | - |
| WDR91 | NP_054868.3 | 29062 | WD repeat-containing protein 91 | 1.6 | 1 | 1 | 1 | 1 | 2.62E+07 | - | - | - | 747 | 83.3 | 6.6 | Biological_process unknown | - | Molecular function unknown | - |
| WDSUB1 | NP_689741.2 | 151525 | WD repeat, SAM and U-box domain-containing protein 1 | 3.8 | 1 | 1 | 1 | 1 | 5.91E+06 | - | - | - | 476 | 52.8 | 6.4 | Cell communication;Signal transduction | - | Molecular function unknown | - |
| WRN | NP_000544.2 | 7486 | Werner syndrome ATP-dependent helicase | 0.8 | 1 | 1 | 1 | 3 | 5.77E+08 | - | - | - | 1432 | 162.4 | 6.3 | Regulation of nucleobase, nucleoside, nucleotide and nucleic acid metabolism | Nucleolus;Nucleus | Deoxyribonuclease activity | Nucleolus |
| XAF1 | NP_954590.1 | 54739 | XIAP-associated factor 1 isoform 2 | 9.6 | 2 | 2 | 2 | 2 | 4.44E+07 | - | - | - | 282 | 32.4 | 8.2 | Apoptosis | Nucleus | Ubiquitin-specific protease activity | Nucleus |
| XIAP | NP_001191330.1 | 331 | E3 ubiquitin-protein ligase XIAP | 2.8 | 1 | 1 | 1 | 1 | 1.40E+07 | - | - | - | 497 | 56.6 | 6.7 | Anti-apoptosis | Cytoplasm;Nucleus | Ligase activity | Cytoplasm |
| XIRP2 | NP_001186074.1 | 129446 | xin actin-binding repeat-containing protein 2 isoform 5 | 1.0 | 3 | 1 | 1 | 1 | 4.23E+07 | - | - | - | 716 | 81.8 | 6.7 | Cytoskeletal anchoring | - | Cytoskeletal protein binding | - |
| XPA | NP_000371.1 | 7507 | DNA repair protein complementing XP-A cells | 16.9 | 1 | 2 | 2 | 2 | 1.45E+07 | - | - | - | 273 | 31.3 | 6.7 | DNA repair | Nucleus | DNA repair protein | Nucleus |
| XPR1 | NP_001129141.1 | 9213 | xenotropic and polytropic retrovirus receptor 1 isoform 2 | 7.6 | 2 | 2 | 2 | 2 | 8.47E+07 | - | - | - | 631 | 73.9 | 7.5 | Cell communication;Signal transduction | Plasma membrane | Receptor activity | Plasma membrane |
| YIF1B | NP_001034762.1 | 90522 | protein YIF1B isoform 4 | 30.6 | 6 | 4 | 4 | 4 | 7.04E+08 | - | - | - | 311 | 33.9 | 8.9 | Biological_process unknown | - | Molecular function unknown | - |
| YIPF2 | NP_076934.1 | 78992 | protein YIPF2 | 9.2 | 1 | 1 | 1 | 1 | 0.00E+00 | - | - | - | 316 | 35.1 | 5.7 | Biological_process unknown | - | Molecular function unknown | - |
| YIPF6 | NP_776195.2 | 286451 | protein YIPF6 isoform A | 17.8 | 1 | 1 | 1 | 1 | 2.28E+07 | - | - | - | 236 | 26.2 | 5.6 | Biological_process unknown | - | Molecular function unknown | - |
| YPEL5 | NP_001120873.1 | 51646 | protein yippee-like 5 | 11.6 | 1 | 1 | 1 | 1 | 2.76E+08 | - | - | - | 121 | 13.8 | 7.3 | Biological_process unknown | - | Molecular function unknown | - |
| YTHDF1 | NP_060268.2 | 54915 | YTH domain-containing family protein 1 | 17.2 | 1 | 2 | 6 | 10 | 2.10E+08 | - | - | - | 559 | 60.8 | 8.8 | Biological_process unknown | - | Molecular function unknown | - |
| ZBTB12 | NP_862825.1 | 221527 | zinc finger and BTB domain-containing protein 12 | 2.4 | 1 | 1 | 1 | 1 | 1.83E+08 | - | - | - | 459 | 49.1 | 7.5 | Regulation of nucleobase, nucleoside, nucleotide and nucleic acid metabolism | - | DNA binding | - |
| ZBTB9 | NP_689948.1 | 221504 | zinc finger and BTB domain-containing protein 9 | 4.4 | 1 | 1 | 1 | 1 | 2.39E+08 | - | - | - | 473 | 50.6 | 6.8 | Regulation of nucleobase, nucleoside, nucleotide and nucleic acid metabolism | Nucleus | Transcription factor activity | Nucleus |
| ZC3H3 | NP_055932.2 | 23144 | zinc finger CCCH domain-containing protein 3 | 2.2 | 1 | 1 | 1 | 2 | 4.64E+08 | - | - | - | 948 | 101.9 | 11.0 | Regulation of nucleobase, nucleoside, nucleotide and nucleic acid metabolism | Nucleus | DNA binding | Nucleus |
| ZC3H7A | NP_054872.2 | 29066 | zinc finger CCCH domain-containing protein 7A | 12.9 | 1 | 4 | 4 | 5 | 2.59E+07 | - | - | - | 971 | 110.5 | 7.3 | Regulation of nucleobase, nucleoside, nucleotide and nucleic acid metabolism | Nucleus | DNA binding | Nucleus |
| ZDHHC20 | NP_001273567.1 | 253832 | probable palmitoyltransferase ZDHHC20 isoform 2 | 6.5 | 2 | 1 | 1 | 1 | 0.00E+00 | - | - | - | 292 | 33.7 | 8.0 | Biological_process unknown | - | Molecular function unknown | - |

| Gene Symbol | RefSeq_Accession | Entrez Gene ID | Description | Coverage (%) | Proteins | Unique Peptides | # Peptides | PSM | Area | SCC-R /SCC-S | SCC-R /SCC-S (Count) | SCC-R /SCC-S (Variability [%]) | # AAs | MW [kDa] | calc. pI | Biological Process | Localization | Molecular Function | Primary Localization |
|---|---|---|---|---|---|---|---|---|---|---|---|---|---|---|---|---|---|---|---|
| **ZDHHC3** | NP_001128651.1 | 51304 | **palmitoyltransferase ZDHHC3 isoform 1** | 5.4 | 2 | 1 | 1 | 1 | 1.49E+08 | - | - | - | 299 | 34.1 | 8.2 | Transport | Golgi apparatus | Molecular function unknown | Golgi apparatus |
| **ZFAND2B** | NP_001257928.1 | 130617 | **AN1-type zinc finger protein 2B isoform 2** | 11.3 | 2 | 1 | 1 | 1 | 6.00E+07 | - | - | - | 213 | 23.2 | 6.9 | Biological_process unknown | - | Molecular function unknown | - |
| **ZFHX4** | NP_078997.4 | 79776 | **zinc finger homeobox protein 4** | 0.8 | 1 | 1 | 1 | 1 | 4.60E+07 | - | - | - | 3616 | 398.7 | 6.3 | Regulation of nucleobase, nucleoside, nucleotide and nucleic acid metabolism | Nucleus | Transcription factor activity | Nucleus |
| **ZMYM2** | NP_932072.1 | 7750 | **zinc finger MYM-type protein 2** | 0.9 | 1 | 1 | 1 | 1 | 5.02E+06 | - | - | - | 1377 | 154.8 | 6.3 | Regulation of nucleobase, nucleoside, nucleotide and nucleic acid metabolism;Biological_process unknown | Nucleolus;Nucleus | Transcription regulator activity;Molecular function unknown | Nucleus |
| **ZMYM3** | NP_001164634.1 | 9203 | **zinc finger MYM-type protein 3 isoform 3** | 11.9 | 3 | 2 | 2 | 3 | 5.31E+07 | - | - | - | 495 | 52.5 | 5.2 | Regulation of nucleobase, nucleoside, nucleotide and nucleic acid metabolism | Nucleus;Cytoplasm | DNA binding | Nucleus |
| **ZNF114** | NP_705836.1 | 163071 | **zinc finger protein 114** | 7.9 | 1 | 3 | 3 | 5 | 4.87E+07 | - | - | - | 417 | 47.7 | 8.6 | Regulation of nucleobase, nucleoside, nucleotide and nucleic acid metabolism | Nucleus | DNA binding | Nucleus |
| **ZNF140** | NP_003431.2 | 7699 | **zinc finger protein 140** | 1.3 | 2 | 1 | 1 | 1 | 1.13E+07 | - | - | - | 457 | 53 | 8.7 | Regulation of nucleobase, nucleoside, nucleotide and nucleic acid metabolism | Nucleus | DNA binding | Nucleus |
| **ZNF148** | NP_068799.2 | 7707 | **zinc finger protein 148** | 2.5 | 1 | 1 | 1 | 1 | 6.21E+07 | - | - | - | 794 | 88.9 | 6.5 | Regulation of nucleobase, nucleoside, nucleotide and nucleic acid metabolism | Nucleus | Transcription factor activity | Nucleus |
| **ZNF181** | NP_001139137.1 | 339318 | **zinc finger protein 181 isoform 2** | 3.7 | 2 | 1 | 1 | 1 | 0.00E+00 | - | - | - | 570 | 65.7 | 8.9 | Regulation of nucleobase, nucleoside, nucleotide and nucleic acid metabolism | - | Transcription factor activity | - |
| **ZNF214** | NP_037381.2 | 7761 | **zinc finger protein 214** | 1.2 | 1 | 1 | 1 | 1 | 6.15E+07 | - | - | - | 606 | 70.9 | 8.5 | Regulation of nucleobase, nucleoside, nucleotide and nucleic acid metabolism | Nucleus | DNA binding | Nucleus |
| **ZNF219** | NP_001095142.1 | 51222 | **zinc finger protein 219** | 1.4 | 1 | 1 | 1 | 1 | 2.59E+09 | - | - | - | 722 | 76.8 | 9.4 | Regulation of nucleobase, nucleoside, nucleotide and nucleic acid metabolism | Nucleus | Transcription regulator activity | Nucleus |
| **ZNF33A** | NP_001265107.1 | 7581 | **zinc finger protein 33A isoform d** | 2.9 | 8 | 1 | 1 | 1 | 0.00E+00 | - | - | - | 699 | 81.1 | 8.3 | Regulation of nucleobase, nucleoside, nucleotide and nucleic acid metabolism | Nucleus | Transcription factor activity | Nucleus |
| **ZNF384** | NP_001035009.1 | 171017 | **zinc finger protein 384 isoform c** | 5.4 | 3 | 1 | 1 | 2 | 3.71E+07 | - | - | - | 461 | 50.5 | 8.5 | Regulation of nucleobase, nucleoside, nucleotide and nucleic acid metabolism | Nucleus | Transcription factor activity | Nucleus |
| **ZNF428** | NP_872304.2 | 126299 | **zinc finger protein 428** | 14.9 | 1 | 2 | 2 | 3 | 9.54E+06 | - | - | - | 188 | 20.5 | 4.2 | Regulation of nucleobase, nucleoside, nucleotide and nucleic acid metabolism | Nucleus | DNA binding | Nucleus |
| **ZNF484** | NP_001248388.1 | 83744 | **zinc finger protein 484 isoform b** | 0.7 | 3 | 1 | 1 | 3 | 4.99E+08 | - | - | - | 816 | 93.9 | 8.4 | Regulation of nucleobase, nucleoside, nucleotide and nucleic acid metabolism | Nucleus | Transcription regulator activity | Nucleus |
| **ZNF511** | NP_665805.2 | 118472 | **zinc finger protein 511** | 9.9 | 1 | 1 | 1 | 2 | 0.00E+00 | - | - | - | 252 | 28.2 | 7.7 | Regulation of nucleobase, nucleoside, nucleotide and nucleic acid metabolism | - | Molecular function unknown | - |
| **ZNF521** | NP_056276.1 | 25925 | **zinc finger protein 521** | 1.1 | 1 | 1 | 1 | 2 | 5.94E+08 | - | - | - | 1311 | 147.8 | 7.0 | Cell growth and/or maintenance | Nucleus | Transcription regulator activity | Nucleus |
| **ZNF646** | NP_055514.3 | 9726 | **zinc finger protein 646** | 0.7 | 1 | 1 | 1 | 1 | 5.82E+07 | - | - | - | 1832 | 200.7 | 7.2 | Regulation of nucleobase, nucleoside, nucleotide and nucleic acid metabolism | Nucleus | Ligand-dependent nuclear receptor activity | Nucleus |
| **ZNF687** | NP_065883.1 | 57592 | **zinc finger protein 687** | 2.2 | 1 | 1 | 1 | 2 | 8.19E+06 | - | - | - | 1237 | 129.4 | 8.2 | Regulation of nucleobase, nucleoside, nucleotide and nucleic acid metabolism | Nucleus;Cytoplasm | DNA binding | Nucleus |
| **ZNF706** | NP_001035975.1 | 51123 | **zinc finger protein 706** | 15.8 | 1 | 1 | 1 | 1 | 1.18E+08 | - | - | - | 76 | 8.5 | 10.0 | Regulation of nucleobase, nucleoside, nucleotide and nucleic acid metabolism | - | DNA binding | - |
| **ZNF770** | NP_054825.2 | 54989 | **zinc finger protein 770** | 4.8 | 1 | 1 | 1 | 1 | 1.80E+08 | - | - | - | 691 | 80 | 9.6 | Regulation of nucleobase, nucleoside, nucleotide and nucleic acid metabolism | - | DNA binding | - |
| **ZNHIT6** | NP_060423.3 | 54680 | **box C/D snoRNA protein 1 isoform 1** | 5.7 | 1 | 1 | 1 | 1 | 2.09E+07 | - | - | - | 470 | 53.9 | 5.8 | Biological_process unknown | - | Molecular function unknown | - |
| **ZWINT** | NP_001005413.1 | 11130 | **ZW10 interactor isoform b** | 5.2 | 2 | 1 | 1 | 1 | 0.00E+00 | - | - | - | 230 | 25.5 | 4.9 | Cell cycle | Nucleus;Cytoplasm;Kinetochore | Molecular function unknown | Nucleus |
| **ZXDA** | NP_009087.1 | 7789 | **zinc finger X-linked protein ZXDA** | 2.1 | 2 | 1 | 1 | 1 | 4.74E+08 | - | - | - | 799 | 84.7 | 7.5 | Regulation of nucleobase, nucleoside, nucleotide and nucleic acid metabolism | Nucleus | Transcription factor activity | Nucleus |

**Supplementary Table S5:List of phosphopeptides identified in SCC-S and SCC-R cells using SequestHT and Mascot search algorithms**

| GeneSymbol | NP_Accession | Gene ID | ProteinDescription | PhosphoSite (Protein) | SCC-R / SCC-S R1 | SCC-R / SCC-S R2 | SCC-R / SCC-S Total Proteome | Sequence | # PSMs | # Proteins | # Protein Groups | # Missed Cleavages | Modifications | phosphoRS Site Probabilities | Phosphowindow | PhosphoSite (Peptide) | MH+ [Da] | PhosphositePlusEvidence |
|---|---|---|---|---|---|---|---|---|---|---|---|---|---|---|---|---|---|---|
| **AAAS** | NP_001166937.1 | 8086 | aladin isoform 2 | **S462** | 0.94 | 0.94 | 1.11 | FsPVLGR | 2 | 2 | 1 | 0 | S2(Phospho) | S(2): 100.0 | NAQFPRFsPVLGRAQ | S2; | 855.4 | No |
| **AAAS** | NP_001166937.1 | 8086 | aladin isoform 2 | **S462** | 1.15 | 1.34 | 1.11 | IAHIPLYFVNAQFPRFsPVLGR | 2 | 2 | 1 | 1 | S17(Phospho) | Y(7): 0.0; S(17): 100.0 | NAQFPRFsPVLGRAQ | S17; | 2622.4 | No |
| **AAK1** | NP_055726.3 | 22848 | AP2-associated protein kinase 1 | **S637** | **2.56** | | 1.42 | ILsDVTHSAVFGVPASK | 3 | 1 | 1 | 0 | S3(Phospho); K17(Label:13C(6)15N(2)) | S(3): 100.0; T(6): 0.0; S(8): 0.0; S(16): 0.0 | AGHRRILsDVTHSAV | S3; | 1815.9 | Yes |
| **AAK1** | NP_055726.3 | 22848 | AP2-associated protein kinase 1 | **S637** | **2.68** | **2.64** | 1.42 | ILsDVTHSAVFGVPASK | 8 | 1 | 1 | 0 | S3(Phospho) | S(3): 100.0; T(6): 0.0; S(8): 0.0; S(16): 0.0 | AGHRRILsDVTHSAV | S3; | 1807.9 | Yes |
| **AAK1** | NP_055726.3 | 22848 | AP2-associated protein kinase 1 | **T606** | 1.2 | 1.09 | 1.42 | VQTtPPPAVQGQK | 6 | 1 | 1 | 0 | T4(Phospho) | T(3): 0.0; T(4): 100.0 | QQPKVQTtPPPAVQG | T4; | 1430.7 | Yes |
| **AAK1** | NP_055726.3 | 22848 | AP2-associated protein kinase 1 | **S637** | **2.44** | **2.54** | 1.42 | RILsDVTHSAVFGVPASK | 6 | 1 | 1 | 1 | S4(Phospho) | S(4): 100.0; T(7): 0.0; S(9): 0.0; S(17): 0.0 | AGHRRILsDVTHSAV | S4; | 1964.0 | Yes |
| **AAK1** | NP_055726.3 | 22848 | AP2-associated protein kinase 1 | **S637** | | 1.91 | 1.42 | RILsDVTHSAVFGVPASK | 3 | 1 | 1 | 1 | R1(Label:13C(6)); S4(Phospho); K18(Label:13C(6)15N(2)) | S(4): 100.0; T(7): 0.0; S(9): 0.0; S(17): 0.0 | AGHRRILsDVTHSAV | S4; | 1978.0 | Yes |
| **AAK1** | NP_055726.3 | 22848 | AP2-associated protein kinase 1 | **T620; S624** | 1.76 | | 1.42 | VGSLtPPSsPKTQR | 2 | 1 | 1 | 1 | T5(Phospho); S9(Phospho) | S(3): 0.0; T(5): 100.0; S(8): 2.0; S(9): 98.0; T(12): 0.0 | GQKVGSLtPPSSPKT; GSLTPPSsPKTQRAG | T5; S9; | 1614.7 | Yes;Yes |
| **AAK1** | NP_055726.3 | 22848 | AP2-associated protein kinase 1 | **T620; S624** | | 1.63 | 1.42 | VGSLtPPSsPK | 2 | 1 | 1 | 0 | T5(Phospho); S9(Phospho) | S(3): 0.0; T(5): 100.0; S(8): 1.0; S(9): 99.0 | GQKVGSLtPPSSPKT; GSLTPPSsPKTQRAG | T5; S9; | 1229.5 | Yes;Yes |
| **AAK1** | NP_055726.3 | 22848 | AP2-associated protein kinase 1 | **T606** | | 1.23 | 1.42 | VQTtPPPAVQGQK | 5 | 1 | 1 | 0 | T4(Phospho); K13(Label:13C(6)15N(2)) | T(3): 0.0; T(4): 100.0 | QQPKVQTtPPPAVQG | T4; | 1438.7 | Yes |
| **AAK1** | NP_055726.3 | 22848 | AP2-associated protein kinase 1 | **S20** | | **2.26** | 1.42 | REQGGSGLGSGsSGGGGSTSG LGSGYIGR | 2 | 1 | 1 | 1 | S13(Phospho) | S(6): 0.0; S(10): 0.0; S(12): 97.8; S(13): 2.2; S(18): 0.0; T(19): 0.0; S(20): 0.0; S(24): 0.0; Y(26): 0.0 | GSGLGSGsSGGGGST | S12; | 2622.2 | Yes |
| **AAK1** | NP_055726.3 | 22848 | AP2-associated protein kinase 1 | **T389** | | **4.53** | 1.42 | AGQTQPNPGILPIQPALtPR | 2 | 1 | 1 | 0 | T18(Phospho) | T(4): 0.0; T(18): 100.0 | LPIQPALtPRKRATV | T18; | 2149.1 | Yes |
| **AATF** | NP_036270.1 | 26574 | protein AATF | **S203** | **0.33** | **0.26** | 0.73 | AGDRNsEDDGVVMTFSSVK | 4 | 1 | 1 | 1 | S6(Phospho) | S(6): 100.0; T(14): 0.0; S(16): 0.0; S(17): 0.0 | DRAGDRNsEDDGVVM | S6; | 2093.9 | Yes |
| **AATF** | NP_036270.1 | 26574 | protein AATF | **S203** | **0.33** | **0.27** | 0.73 | AGDRNsEDDGVVMTFSSVK | 6 | 1 | 1 | 1 | R4(Label:13C(6)); S6(Phospho); K19(Label:13C(6)15N(2)) | S(6): 100.0; T(14): 0.0; S(16): 0.0; S(17): 0.0 | DRAGDRNsEDDGVVM | S6; | 2107.9 | Yes |
| **AATF** | NP_036270.1 | 26574 | protein AATF | **S203** | | | 0.73 | AGDRNsEDDGVVMTFSSVK | 1 | 1 | 1 | 1 | R4(Label:13C(6)); S6(Phospho); M13(Oxidation); K19(Label:13C(6)15N(2)) | S(6): 100.0; T(14): 0.0; S(16): 0.0; S(17): 0.0 | DRAGDRNsEDDGVVM | S6; | 2123.9 | Yes |
| **ABCA7** | NP_061985.2 | 10347 | ATP-binding cassette sub-family A member 7 | **S941; S958** | | | #N/A | HLsGGMQRKLSVAIAFVGGsQVV ILDEPTAGVDPASR | 1 | 1 | 1 | 2 | K9(Label:13C(6)15N(2)); S11(Phospho); S20(Phospho); R37(Label:13C(6)) | S(3): 90.1; S(11): 10.9; S(20): 89.1; T(29): 9.8; S(36): 0.0 | SVQTRHLsGGMQRKL; AIAFVGGsQVVILDE | S3; S20; | 3937.0 | No;Yes |
| **ABCF1** | NP_001020262.1 | 23 | ATP-binding cassette sub-family F member 1 isoform a | **S109** | 0.61 | | **0.47** | LSVPTsDEEDEVPAPKPR | 5 | 2 | 1 | 0 | S6(Phospho) | S(2): 0.0; T(5): 0.0; S(6): 100.0 | KKLSVPTsDEEDEVP | S6; | 2045.9 | Yes |
| **ABCF1** | NP_001020262.1 | 23 | ATP-binding cassette sub-family F member 1 isoform a | **S109** | 0.53 | | **0.47** | KLSVPTsDEEDEVPAPKPR | 2 | 2 | 1 | 1 | K1(Label:13C(6)15N(2)); S7(Phospho); K17(Label:13C(6)15N(2)); R19(Label:13C(6)) | S(3): 0.0; T(6): 0.0; S(7): 100.0 | KKLSVPTsDEEDEVP | S7; | 2196.1 | Yes |
| **ABCF1** | NP_001020262.1 | 23 | ATP-binding cassette sub-family F member 1 isoform a | **S109** | 1.02 | | **0.47** | LSVPTsDEEDEVPAPKPR | 5 | 2 | 1 | 0 | S6(Phospho); K16(Label:13C(6)15N(2)); R18(Label:13C(6)) | S(2): 0.0; T(5): 0.0; S(6): 100.0 | KKLSVPTsDEEDEVP | S6; | 2060.0 | Yes |
| **ABCF1** | NP_001020262.1 | 23 | ATP-binding cassette sub-family F member 1 isoform a | **S105; T108; S109** | 1.58 | 0.95 | **0.47** | LKKLsVPtsDEEDEVPAPKPR | 2 | 2 | 1 | 2 | S5(Phospho); T8(Phospho); S9(Phospho) | S(5): 100.0; T(8): 100.0; S(9): 100.0 | MERLKKLsVPTSDEE;LKKLSVPtSDEEDEV;KKLSVPTsDEEDEVP | S5; T8; S9; | 2575.2 | Yes;Yes;Yes |
| **ABCF1** | NP_001020262.1 | 23 | ATP-binding cassette sub-family F member 1 isoform a | **S166** | **0.4** | | **0.47** | AVsEEQQPALK | 1 | 2 | 1 | 0 | S3(Phospho); K11(Label:13C(6)15N(2)) | S(3): 100.0 | NRINKAVsEEQQPAL | S3; | 1287.6 | Yes |
| **ABCF1** | NP_001020262.1 | 23 | ATP-binding cassette sub-family F member 1 isoform a | **T108; S109** | | 0.59 | **0.47** | LSVPtsDEEDEVPAPKPR | 3 | 2 | 1 | 0 | T5(Phospho); S6(Phospho) | S(2): 0.0; T(5): 100.0; S(6): 100.0 | LKKLSVPtSDEEDEV;KKLSVPTsDEEDEVP | T5; S6; | 2125.9 | Yes;Yes |
| **ABCF1** | NP_001020262.1 | 23 | ATP-binding cassette sub-family F member 1 isoform a | **S228** | | **0.46** | **0.47** | KAEQGsEEEGEGEEEEEEGGES K | 1 | 1 | 1 | 1 | K1(Label:13C(6)15N(2)); S6(Phospho); K23(Label:13C(6)15N(2)) | S(6): 100.0; S(22): 0.0 | AKKAEQGsEEEGEGE | S6; | 2578.0 | Yes |
| **ABCF1** | NP_001020262.1 | 23 | ATP-binding cassette sub-family F member 1 isoform a | **S228** | | 0.52 | **0.47** | KAEQGsEEEGEGEEEEEEGGES KADDPYAHLSK | 2 | 1 | 1 | 2 | S6(Phospho) | S(6): 100.0; S(22): 0.0; Y(28): 0.0; S(32): 0.0 | AKKAEQGsEEEGEGE | S6; | 3659.5 | Yes |
| **ABCF1** | NP_001020262.1 | 23 | ATP-binding cassette sub-family F member 1 isoform a | **S228** | | | **0.47** | KAEQGsEEEGEGEEEEEEGGES KADDPYAHLSK | 2 | 1 | 1 | 2 | K1(Label:13C(6)15N(2)); S6(Phospho); K23(Label:13C(6)15N(2)); K33(Label:13C(6)15N(2)) | S(6): 100.0; S(22): 0.0; Y(28): 0.0; S(32): 0.0 | AKKAEQGsEEEGEGE | S6; | 3683.5 | Yes |
| **ABCF1** | NP_001020262.1 | 23 | ATP-binding cassette sub-family F member 1 isoform a | **T108; S109** | | | **0.47** | LSVPtsDEEDEVPAPKPR | 1 | 2 | 1 | 0 | T5(Phospho); S6(Phospho); K16(Label:13C(6)15N(2)); R18(Label:13C(6)) | S(2): 3.7; T(5): 98.1; S(6): 98.1 | LKKLSVPtSDEEDEV;KKLSVPTsDEEDEVP | T5; S6; | 2139.9 | Yes;Yes |
| **ABI1** | NP_001171596.1 | 10006 | abl interactor 1 isoform l | **S161** | 0.68 | 0.79 | #NQ | LGSQHsPGR | 8 | 12 | 1 | 0 | S6(Phospho) | S(3): 0.0; S(6): 100.0 | ARLGSQHsPGRTASL | S6; | 1018.4 | No |
| **ABI1** | NP_001171596.1 | 10006 | abl interactor 1 isoform l | **S161** | 0.71 | 1.01 | #NQ | LGSQHsPGR | 3 | 12 | 1 | 0 | S6(Phospho); R9(Label:13C(6)) | S(3): 0.0; S(6): 100.0 | ARLGSQHsPGRTASL | S6; | 1024.5 | No |
| **ABI2** | NP_001269861.1 | 10152 | abl interactor 2 isoform e | **S165** | 1.11 | 0.85 | **2.52** | NMAPSQQsPVR | 4 | 5 | 1 | 0 | S8(Phospho) | S(5): 0.0; S(8): 100.0 | NMAPSQQsPVRTASV | S8; | 1294.6 | No |
| **ABI2** | NP_001269861.1 | 10152 | abl interactor 2 isoform e | **S165** | 1 | 0.97 | **2.52** | NMAPSQQsPVR | 2 | 5 | 1 | 0 | S8(Phospho); R11(Label:13C(6)) | S(5): 0.0; S(8): 100.0 | NMAPSQQsPVRTASV | S8; | 1300.6 | No |
| **ABL1** | NP_005148.2 | 25 | tyrosine-protein kinase ABL1 isoform a | **S569** | | | #N/A | GQGESDPLDHEPAVsPLLPR | 2 | 2 | 1 | 0 | S15(Phospho) | S(5): 0.0; S(15): 100.0 | LDHEPAVsPLLPRKE | S15; | 2194.0 | Yes |
| **ABL2** | NP_001161711.1 | 27 | Abelson tyrosine-protein kinase 2 isoform i | **S584** | | **2.43** | #N/A | GAQASSGsPALPR | 2 | 7 | 1 | 0 | S8(Phospho) | S(5): 0.0; S(6): 0.0; S(8): 100.0 | GAQASSGsPALPRKQ | S8; | 1278.6 | No |
| **ABLIM1** | NP_006711.3 | 3983 | actin-binding LIM protein 1 isoform s | **S383** | | **2.25** | #N/A | GVsMPNMLEPK | 2 | 4 | 1 | 0 | S3(Phospho) | S(3): 100.0 | MRMDRGVsMPNMLEP | S3; | 1282.6 | No |
| **ABLIM1** | NP_006711.3 | 3983 | actin-binding LIM protein 1 isoform s | **S217** | | **0.47** | #N/A | sSEDIIK | 1 | 4 | 1 | 0 | S1(Phospho); K7(Label:13C(6)15N(2)) | S(1): 99.0; S(2): 1.0 | ALAAQSKsSEDIIKF | S1; | 879.4 | No |
| **ABLIM3** | NP_055760.1 | 22885 | actin-binding LIM protein 3 | **T276; S282** | | | #N/A | HRRtSETSIsPPGSSIGSPNR | 1 | 1 | 1 | 2 | R2(Label:13C(6)); R3(Label:13C(6)); S5(Phospho); S8(Phospho) | T(4): 84.5; S(5): 10.4; T(7): 10.4; S(8): 5.5; S(10): 89.1; S(14): 0.1; S(15): 0.1; S(18): 0.0 | KKLKHRRtSETSISP;RTSETSIsPPGSSIG | T4; S10; | 2395.1 | Yes;Yes |
| **ACACA** | NP_942136.1 | 31 | acetyl-CoA carboxylase 1 isoform 2 | **S29** | | | **0.24** | FIIGSVSEDNsEDEISNLVK | 4 | 2 | 1 | 0 | S11(Phospho); K20(Label:13C(6)15N(2)) | S(5): 0.0; S(7): 0.0; S(11): 100.0; S(16): 0.0 | GSVSEDNsEDEISNL | S11; | 2283.0 | Yes |
| **ACACA** | NP_942136.1 | 31 | acetyl-CoA carboxylase 1 isoform 2 | **S1259** | | **0.27** | **0.24** | IFDEVMGCFSDsPPQSPTFPEAG HTSLYDEDKVPR | 1 | 4 | 1 | 1 | C8(Carbamidomethyl); S12(Phospho); K32(Label:13C(6)15N(2)); R35(Label:13C(6)) | S(10): 0.0; S(12): 100.0; S(16): 0.0; T(18): 0.0; T(25): 0.0; S(26): 0.0; Y(28): 0.0 | VMGCFSDsPPQSPTF | S12; | 4049.8 | Yes |
| **ACAP2** | NP_036419.3 | 23527 | arf-GAP with coiled-coil, ANK repeat and PH domain-containing protein 2 | **S521** | | | 1.52 | FVDKYSISLsPPEQQK | 2 | 1 | 1 | 1 | S10(Phospho) | Y(5): 0.0; S(6): 0.0; S(8): 0.0; S(10): 100.0 | DKYSISLsPPEQQKK | S10; | 1945.9 | Yes |
| **ACAP2** | NP_036419.3 | 23527 | arf-GAP with coiled-coil, ANK repeat and PH domain-containing protein 2 | **S521** | | | 1.52 | FVDKYSISLsPPEQQK | 1 | 1 | 1 | 1 | K4(Label:13C(6)15N(2)); S10(Phospho); K16(Label:13C(6)15N(2)) | Y(5): 0.0; S(6): 0.0; S(8): 0.0; S(10): 100.0 | DKYSISLsPPEQQKK | S10; | 1962.0 | Yes |
| **ACIN1** | NP_001158287.1 | 22985 | apoptotic chromatin condensation inducer in the nucleus isoform 3 | **S670** | **2.84** | 1.94 | 1.04 | RLsQPESAEK | 5 | 3 | 1 | 1 | S3(Phospho) | S(3): 100.0; S(7): 0.0 | SVQARRLsQPESAEK | S3; | 1224.6 | No |
| **ACIN1** | NP_001158287.1 | 22985 | apoptotic chromatin condensation inducer in the nucleus isoform 3 | **S200; S203** | 1.33 | | 1.04 | AAKLsEGsQPAEEEEDQETPSR | 2 | 3 | 1 | 1 | S5(Phospho); S8(Phospho) | S(5): 100.0; S(8): 100.0; T(19): 0.0; S(21): 0.0 | QARAAKLsEGSQPAE; AAKLSEGsQPAEEEE | S5; S8; | 2548.0 | No;No |
| **ACIN1** | NP_001158287.1 | 22985 | apoptotic chromatin condensation inducer in the nucleus isoform 3 | **S413** | | **2.1** | 1.04 | EASSPPPHPQLHSEEEIEPMEGP APPVLIQLsPPNTDADTR | 4 | 3 | 1 | 0 | S13(Phospho); S32(Phospho) | S(3): 49.1; S(4): 49.1; S(13): 1.8; S(32): 100.0; T(36): 0.0; T(40): 0.0 | PPVLIQLsPPNTDAD | S32; | 4570.1 | No |
| **ACIN1** | NP_001158287.1 | 22985 | apoptotic chromatin condensation inducer in the nucleus isoform 3 | **S450** | 1.3 | | 1.04 | AEsPAEKVPEESVLPLVQK | 3 | 3 | 1 | 1 | S3(Phospho) | S(3): 100.0; S(12): 0.0 | PSDTKAEsPAEKVPE | S3; | 2130.1 | No |
| **ACIN1** | NP_001158287.1 | 22985 | apoptotic chromatin condensation inducer in the nucleus isoform 3 | **S288** | **2.53** | | 1.04 | sQEQEVLER | 2 | 3 | 1 | 0 | S1(Phospho) | S(1): 100.0 | DERPKTRsQEQEVLE | S1; | 1197.5 | No |
| **ACIN1** | NP_001158287.1 | 22985 | apoptotic chromatin condensation inducer in the nucleus isoform 3 | **S450** | 1.37 | 1.21 | 1.04 | AEsPAEKVPEESVLPLVQK | 4 | 3 | 1 | 1 | S3(Phospho); K7(Label:13C(6)15N(2)); K19(Label:13C(6)15N(2)) | S(3): 100.0; S(12): 0.0 | PSDTKAEsPAEKVPE | S3; | 2146.1 | No |
| **ACIN1** | NP_001158287.1 | 22985 | apoptotic chromatin condensation inducer in the nucleus isoform 3 | **S670** | **2.23** | | 1.04 | RLsQPESAEKHVTQR | 1 | 3 | 1 | 2 | S3(Phospho) | S(3): 100.0; S(7): 0.0; T(13): 0.0 | SVQARRLsQPESAEK | S3; | 1845.9 | No |
| **ACIN1** | NP_001158287.1 | 22985 | apoptotic chromatin condensation inducer in the nucleus isoform 3 | **S670** | | **2.61** | 1.04 | RLsQPESAEK | 2 | 3 | 1 | 1 | R1(Label:13C(6)); S3(Phospho); K10(Label:13C(6)15N(2)) | S(3): 100.0; S(7): 0.0 | SVQARRLsQPESAEK | S3; | 1238.6 | No |
| **ACIN1** | NP_001158287.1 | 22985 | apoptotic chromatin condensation inducer in the nucleus isoform 3 | **S1289; S1291; T1292** | 0.93 | | 1.04 | sRstPVRDR | 1 | 5 | 1 | 2 | S1(Phospho); S3(Phospho); T4(Phospho) | S(1): 100.0; S(3): 100.0; T(4): 100.0 | KRHSRSRsRSTPVRD; HSRSRSRsTPVRDRG; SRSRSRStPVRDRGG | S1; S3; T4; | 1313.5 | No;No;No |
| **ACIN1** | NP_001158287.1 | 22985 | apoptotic chromatin condensation inducer in the nucleus isoform 3 | **S798** | | 0.99 | 1.04 | GVPAGNsDTEGGQPGR | 4 | 5 | 1 | 0 | S7(Phospho) | S(7): 100.0; T(9): 0.0 | KGVPAGNsDTEGGQP | S7; | 1578.7 | No |

| GeneSymbol | NP_Accession | Gene ID | ProteinDescription | PhosphoSite (Protein) | SCC-R / SCC-S R1 | SCC-R / SCC-S R2 | SCC-R / SCC-S Total Proteome | Sequence | # PSMs | # Proteins | # Protein Groups | # Missed Cleavages | Modifications | phosphoRS Site Probabilities | Phosphowindow | PhosphoSite (Peptide) | MH+ [Da] | PhosphositePlusEvidence |
|---|---|---|---|---|---|---|---|---|---|---|---|---|---|---|---|---|---|---|
| **ACIN1** | NP_001158287.1 | 22985 | apoptotic chromatin condensation inducer in the nucleus isoform 3 | **S798** | | 1.06 | 1.04 | GVPAGNsDTEGGQPGRK | 3 | 5 | 1 | 1 | S7(Phospho) | S(7): 100.0; T(9): 0.0 | KGVPAGNsDTEGGQP | S7; | 1706.7 | No |
| **ACIN1** | NP_001158287.1 | 22985 | apoptotic chromatin condensation inducer in the nucleus isoform 3 | **S438** | | **0.47** | 1.04 | ELLVSQHTVQLVGGLsPLSSPSDTK | 2 | 3 | 1 | 0 | S16(Phospho) | S(5): 0.0; T(8): 0.0; S(16): 100.0; S(19): 0.0; S(20): 0.0; S(22): 0.0; T(24): 0.0 | VQLVGGLsPLSSPSD | S16; | 2672.4 | No |
| **ACIN1** | NP_001158287.1 | 22985 | apoptotic chromatin condensation inducer in the nucleus isoform 3 | **S438** | | **2.18** | 1.04 | ELLVSQHTVQLVGGLsPLSSPSDTKAESPAEKVPEESVLPLVQK | 3 | 3 | 1 | 2 | S16(Phospho); S19(Phospho) | S(5): 0.0; T(8): 0.0; S(16): 99.7; S(19): 0.2; S(20): 0.2; S(22): 49.0; T(24): 49.0; S(28): 1.9; S(37): 0.0 | VQLVGGLsPLSSPSD | S16; | 4783.4 | No |
| **ACIN1** | NP_001158287.1 | 22985 | apoptotic chromatin condensation inducer in the nucleus isoform 3 | **S370** | | | 1.04 | ASLVALPEQTAsEEETPPPLLTK | 2 | 3 | 1 | 0 | S12(Phospho); K23(Label:13C(6)15N(2)) | S(2): 0.0; T(10): 0.1; S(12): 99.9; T(16): 0.0; T(22): 0.0 | ALPEQTAsEEETPPP | S12; | 2509.3 | No |
| **ACIN1** | NP_001158287.1 | 22985 | apoptotic chromatin condensation inducer in the nucleus isoform 3 | **S438** | | 1.36 | 1.04 | ELLVSQHTVQLVGGLsPLSSPSDTKAESPAEKVPEESVLPLVQK | 3 | 3 | 1 | 2 | S16(Phospho); K25(Label:13C(6)15N(2)); K32(Label:13C(6)15N(2)); K44(Label:13C(6)15N(2)) | S(5): 0.0; T(8): 0.0; S(16): 100.0; S(19): 0.0; S(20): 0.0; S(22): 0.0; T(24): 0.0; S(28): 0.0; S(37): 0.0 | VQLVGGLsPLSSPSD | S16; | 4727.5 | No |
| **ACIN1** | NP_001158287.1 | 22985 | apoptotic chromatin condensation inducer in the nucleus isoform 3 | **S370** | | 1.73 | 1.04 | ASLVALPEQTAsEEETPPPLLTK | 3 | 3 | 1 | 0 | S12(Phospho) | S(2): 0.0; T(10): 0.1; S(12): 99.9; T(16): 0.0; T(22): 0.0 | ALPEQTAsEEETPPP | S12; | 2501.2 | No |
| **ACIN1** | NP_001158287.1 | 22985 | apoptotic chromatin condensation inducer in the nucleus isoform 3 | **S384; S385; S413** | | **3.59** | 1.04 | EAssPPPHPQLHSEEEIEPMEGPAPPVLIQLsPPNTDADTR | 1 | 3 | 1 | 0 | S4(Phospho); S13(Phospho); S32(Phospho) | S(3): 99.5; S(4): 99.5; S(13): 0.9; S(32): 100.0; T(36): 0.0; T(40): 0.0 | PLLTKEAsSPPPHPQ;LLTKEASsPPPHPQL;PPVLIQLsPPNTDAD | S3; S4; S32; | 4650.0 | No;No;No;No |
| **ACIN1** | NP_001158287.1 | 22985 | apoptotic chromatin condensation inducer in the nucleus isoform 3 | **S176** | | 0.74 | 1.04 | SSSISEEKGDsDDEKPR | 2 | 3 | 1 | 1 | S11(Phospho) | S(1): 0.0; S(2): 0.0; S(3): 0.0; S(5): 0.0; S(11): 100.0 | ISEEKGDsDDEKPRK | S11; | 1945.8 | No |
| **ACIN1** | NP_001158287.1 | 22985 | apoptotic chromatin condensation inducer in the nucleus isoform 3 | **S521** | | | 1.04 | RAsHTLLPSHR | 1 | 3 | 1 | 1 | S3(Phospho) | S(3): 100.0; T(5): 0.0; S(9): 0.0 | QKEGRRAsHTLLPSH | S3; | 1354.7 | No |
| **ACIN1** | NP_001158287.1 | 22985 | apoptotic chromatin condensation inducer in the nucleus isoform 3 | **S438** | | | 1.04 | ELLVSQHTVQLVGGLsPLSSPSDTKAESPAEKVPEESVLPLVQK | 2 | 3 | 1 | 2 | S16(Phospho) | S(5): 0.0; T(8): 0.0; S(16): 91.1; S(19): 4.2; S(20): 4.2; S(22): 0.2; T(24): 0.2; S(28): 0.2; S(37): 0.0 | VQLVGGLsPLSSPSD | S16; | 4703.4 | No |
| **ACIN1** | NP_001158287.1 | 22985 | apoptotic chromatin condensation inducer in the nucleus isoform 3 | **S615; S617** | | 0.84 | 1.04 | sLsPGVSR | 2 | 3 | 1 | 0 | S1(Phospho); S3(Phospho) | S(1): 100.0; S(3): 100.0; S(7): 0.0 | ASSNSRKsLSPGVSR;SNSRKSLsPGVSRDS | S1; S3; | 962.4 | No;No |
| **ACIN1** | NP_001158287.1 | 22985 | apoptotic chromatin condensation inducer in the nucleus isoform 3 | **S855** | | | 1.04 | SLIPDIKPLAGQEAVVDLHADDsRISEDETER | 1 | 4 | 1 | 1 | K7(Label:13C(6)15N(2)); R24(Label:13C(6)); S26(Phospho); R32(Label:13C(6)) | S(1): 0.0; S(23): 96.6; S(26): 3.3; T(30): 0.1 | VDLHADDsRISEDET | S23; | 3618.8 | No |
| **ACIN1** | NP_001158287.1 | 22985 | apoptotic chromatin condensation inducer in the nucleus isoform 3 | **S176** | | 0.76 | 1.04 | SSSISEEKGDsDDEKPR | 1 | 3 | 1 | 1 | K8(Label:13C(6)15N(2)); S11(Phospho); K15(Label:13C(6)15N(2)); R17(Label:13C(6)) | S(1): 0.0; S(2): 0.0; S(3): 0.0; S(5): 0.0; S(11): 100.0 | ISEEKGDsDDEKPRK | S11; | 1967.9 | No |
| **ACIN1** | NP_001158287.1 | 22985 | apoptotic chromatin condensation inducer in the nucleus isoform 3 | **S798** | | | 1.04 | GVPAGNsDTEGGQPGRK | 1 | 5 | 1 | 1 | S7(Phospho); R16(Label:13C(6)); K17(Label:13C(6)15N(2)) | S(7): 98.9; T(9): 1.1 | KGVPAGNsDTEGGQP | S7; | 1720.8 | No |
| **ACIN1** | NP_001158287.1 | 22985 | apoptotic chromatin condensation inducer in the nucleus isoform 3 | **S798** | | 1.02 | 1.04 | GVPAGNsDTEGGQPGR | 2 | 5 | 1 | 0 | S7(Phospho); R16(Label:13C(6)) | S(7): 100.0; T(9): 0.0 | KGVPAGNsDTEGGQP | S7; | 1584.7 | No |
| **ACLY** | NP_001087.2 | 47 | ATP-citrate synthase isoform 1 | **S481** | **0.38** | **0.36** | **0.44** | AKPAMPQDSVPsPR | 7 | 1 | 1 | 0 | S12(Phospho) | S(9): 0.0; S(12): 100.0 | MPQDSVPsPRSLQGK | S12; | 1560.7 | Yes |
| **ACLY** | NP_001087.2 | 47 | ATP-citrate synthase isoform 1 | **S481** | | **0.37** | **0.44** | AKPAMPQDSVPsPR | 5 | 1 | 1 | 0 | K2(Label:13C(6)15N(2)); S12(Phospho); R14(Label:13C(6)) | S(9): 0.0; S(12): 100.0 | MPQDSVPsPRSLQGK | S12; | 1574.8 | Yes |
| **ACLY** | NP_001087.2 | 47 | ATP-citrate synthase isoform 1 | **S455** | | 0.58 | **0.44** | TAsFSESR | 1 | 2 | 1 | 0 | S3(Phospho) | T(1): 0.0; S(3): 100.0; S(5): 0.0; S(7): 0.0 | PAPSRTAsFSESRAD | S3; | 964.4 | Yes |
| **ACLY** | NP_001087.2 | 47 | ATP-citrate synthase isoform 1 | **S455** | | 0.6 | **0.44** | TAsFSESR | 1 | 2 | 1 | 0 | S3(Phospho); R8(Label:13C(6)) | T(1): 0.0; S(3): 100.0; S(5): 0.0; S(7): 0.0 | PAPSRTAsFSESRAD | S3; | 970.4 | Yes |
| **ACSL3** | NP_004448.2 | 2181 | long-chain-fatty-acid--CoA ligase 3 | **S683** | | **0.17** | **0.37** | IRLsPEPWTPETGLVTDAFK | 2 | 1 | 1 | 1 | S4(Phospho) | S(4): 100.0; T(9): 0.0; T(12): 0.0; T(16): 0.0 | IPVKIRLsPEPWTPE | S4; | 2337.2 | Yes |
| **ACSS2** | NP_061147.1 | 55902 | acetyl-coenzyme A synthetase, cytoplasmic isoform 1 | **S267** | **0.42** | | 0.63 | AELGMGDSTSQsPPIKR | 2 | 3 | 1 | 1 | S12(Phospho) | S(8): 0.0; T(9): 0.0; S(10): 0.0; S(12): 100.0 | MGDSTSQsPPIKRSC | S12; | 1853.8 | Yes |
| **ACSS2** | NP_061147.1 | 55902 | acetyl-coenzyme A synthetase, cytoplasmic isoform 1 | **S267** | **0.43** | **0.32** | 0.63 | AELGMGDSTSQsPPIKR | 4 | 3 | 1 | 1 | S12(Phospho); K16(Label:13C(6)15N(2)); R17(Label:13C(6)) | S(8): 0.0; T(9): 0.0; S(10): 0.0; S(12): 100.0 | MGDSTSQsPPIKRSC | S12; | 1867.9 | Yes |
| **ACSS2** | NP_061147.1 | 55902 | acetyl-coenzyme A synthetase, cytoplasmic isoform 1 | **S30** | | 0.62 | 0.63 | SWsPPPEVSR | 2 | 2 | 1 | 0 | S3(Phospho) | S(1): 0.0; S(3): 100.0; S(9): 0.0 | GGRARSWsPPPEVSR | S3; | 1221.5 | Yes |
| **ACTL6A** | NP_817126.1 | 86 | actin-like protein 6A isoform 2 | **S191** | **1.95** | | 1.37 | EAVREGsPANWK | 2 | 2 | 1 | 1 | S7(Phospho) | S(7): 100.0 | KEAVREGsPANWKRK | S7; | 1423.6 | No |
| **ACTL6A** | NP_817126.1 | 86 | actin-like protein 6A isoform 2 | **S191** | | 1.46 | 1.37 | EGsPANWK | 1 | 2 | 1 | 0 | S3(Phospho) | S(3): 100.0 | KEAVREGsPANWKRK | S3; | 968.4 | No |
| **ACTR10** | NP_060947.1 | 55860 | actin-related protein 10 | **S414** | 0.65 | | 1.01 | AFsTEK | 1 | 1 | 1 | 0 | S3(Phospho); K6(Label:13C(6)15N(2)) | S(3): 98.8; T(4): 1.2 | PLMKRAFsTEK_ | S3; | 770.3 | Yes |
| **ACTR8** | NP_075050.3 | 93973 | actin-related protein 8 | **S132** | | | #N/A | IPVsPEQAR | 1 | 1 | 1 | 0 | S4(Phospho) | S(4): 100.0 | GTRRIPVsPEQARSY | S4; | 1076.5 | Yes |
| **ADAM17** | NP_003174.3 | 6868 | disintegrin and metalloproteinase domain-containing protein 17 preproprotein | **S791** | **0.25** | **0.22** | 1.11 | sFEDLTDHPVTR | 3 | 1 | 1 | 0 | S1(Phospho); R12(Label:13C(6)) | S(1): 100.0; T(6): 0.0; T(11): 0.0 | NSSTAAKsFEDLTDH | S1; | 1502.7 | Yes |
| **ADAM17** | NP_003174.3 | 6868 | disintegrin and metalloproteinase domain-containing protein 17 preproprotein | **T735** | | **5.52** | 1.11 | IIKPFPAPQtPGR | 1 | 1 | 1 | 0 | T10(Phospho) | T(10): 100.0 | KPFPAPQtPGRLQPA | T10; | 1501.8 | Yes |
| **ADAM9** | NP_003807.1 | 8754 | disintegrin and metalloproteinase domain-containing protein 9 precursor | **S758** | **5.17** | | 1.09 | HVsPVTPPR | 2 | 1 | 1 | 0 | S3(Phospho); R9(Label:13C(6)) | S(3): 100.0; T(6): 0.0 | GSVPRHVsPVTPPRE | S3; | 1075.5 | Yes |
| **ADAM9** | NP_003807.1 | 8754 | disintegrin and metalloproteinase domain-containing protein 9 precursor | **S758** | | | 1.09 | HVsPVTPPR | 1 | 1 | 1 | 0 | S3(Phospho) | S(3): 100.0; T(6): 0.0 | GSVPRHVsPVTPPRE | S3; | 1069.5 | Yes |
| **ADCY2** | NP_065433.2 | 108 | adenylate cyclase type 2 | **S178** | | | #N/A | DAIIASVLTSSSHTIVLSVCLsATPGGK | 1 | 1 | 1 | 0 | S18(Phospho); C20(Carbamidomethyl) | S(6): 0.0; T(9): 0.0; S(10): 0.0; S(11): 0.0; S(12): 0.0; T(14): 0.0; S(18): 3.9; S(22): 92.1; T(24): 3.9 | IVLSVCLsATPGGKE | S22; | 2864.4 | No |
| **ADCY6** | NP_066193.1 | 112 | adenylate cyclase type 6 isoform b | **S54** | **2.5** | **2.1** | #N/A | DAEPPsPTPAGPPR | 4 | 2 | 1 | 0 | S6(Phospho) | S(6): 98.8; T(8): 1.2 | LRDAEPPsPTPAGPP | S6; | 1468.6 | Yes |
| **ADCY6** | NP_066193.1 | 112 | adenylate cyclase type 6 isoform b | **S54** | | **2.17** | #N/A | DAEPPsPTPAGPPR | 2 | 2 | 1 | 0 | S6(Phospho); R14(Label:13C(6)) | S(6): 100.0; T(8): 0.0 | LRDAEPPsPTPAGPP | S6; | 1474.7 | Yes |
| **ADCY6** | NP_066193.1 | 112 | adenylate cyclase type 6 isoform b | **S576** | | 0.75 | #N/A | ANsMEGLMPR | 2 | 2 | 1 | 0 | S3(Phospho) | S(3): 100.0 | LQRTRANsMEGLMPR | S3; | 1185.5 | Yes |
| **ADCY6** | NP_066193.1 | 112 | adenylate cyclase type 6 isoform b | **S576** | | 0.77 | #N/A | ANsMEGLMPR | 1 | 2 | 1 | 0 | S3(Phospho); R10(Label:13C(6)) | S(3): 100.0 | LQRTRANsMEGLMPR | S3; | 1191.5 | Yes |
| **ADD1** | NP_054909.2 | 118 | alpha-adducin isoform c | **S12** | **2.13** | **2.4** | 1.11 | AAVVTsPPPTTAPHK | 3 | 5 | 1 | 0 | S6(Phospho) | T(5): 0.0; S(6): 100.0; T(10): 0.0; T(11): 0.0 | SRAAVVTsPPPTTAP | S6; | 1553.8 | Yes |
| **ADD1** | NP_054909.2 | 118 | alpha-adducin isoform c | **S12** | | | 1.11 | AAVVTsPPPTTAPHK | 1 | 5 | 1 | 0 | S6(Phospho); K15(Label:13C(6)15N(2)) | T(5): 0.0; S(6): 100.0; T(10): 0.0; T(11): 0.0 | SRAAVVTsPPPTTAP | S6; | 1561.8 | Yes |
| **ADD1** | NP_054909.2 | 118 | alpha-adducin isoform c | **S465** | 0.94 | | 1.11 | WLNSGRGDEASEEGQNGSsPK | 4 | 5 | 1 | 1 | R6(Label:13C(6)); S19(Phospho); K21(Label:13C(6)15N(2)) | S(4): 0.0; S(11): 0.0; S(18): 1.3; S(19): 98.7 | EEGQNGSsPKSKTKW | S19; | 2299.0 | Yes |
| **ADD1** | NP_054909.2 | 118 | alpha-adducin isoform c | **S465** | | 0.94 | 1.11 | WLNSGRGDEASEEGQNGSsPK | 5 | 5 | 1 | 1 | S19(Phospho) | S(4): 0.0; S(11): 0.0; S(18): 0.0; S(19): 100.0 | EEGQNGSsPKSKTKW | S19; | 2284.9 | Yes |
| **ADD1** | NP_054909.2 | 118 | alpha-adducin isoform c | **S586** | | | 1.11 | KQKGsEENLDEAR | 1 | 5 | 1 | 2 | K1(Label:13C(6)15N(2)); K3(Label:13C(6)15N(2)); S5(Phospho); R13(Label:13C(6)) | S(5): 100.0 | VERKQKGsEENLDEA | S5; | 1605.8 | Yes |
| **ADD1** | NP_054909.2 | 118 | alpha-adducin isoform c | **S12** | 1.75 | | 1.11 | AAVVTsPPPTTAPHKER | 3 | 5 | 1 | 1 | S6(Phospho); K15(Label:13C(6)15N(2)); R17(Label:13C(6)) | T(5): 1.4; S(6): 98.6; T(10): 0.0; T(11): 0.0 | SRAAVVTsPPPTTAP | S6; | 1853.0 | Yes |
| **ADD1** | NP_054909.2 | 118 | alpha-adducin isoform c | **S586** | **0.17** | | 1.11 | QKGsEENLDEAR | 2 | 5 | 1 | 1 | K2(Label:13C(6)15N(2)); S4(Phospho); R12(Label:13C(6)) | S(4): 100.0 | VERKQKGsEENLDEA | S4; | 1469.6 | Yes |
| **ADD1** | NP_054909.2 | 118 | alpha-adducin isoform c | **S358** | | **1.97** | 1.11 | SPGsPVGEGTGSPPK | 2 | 5 | 1 | 0 | S4(Phospho) | S(1): 0.0; S(4): 100.0; T(10): 0.0; S(12): 0.0 | AKSRSPGsPVGEGTG | S4; | 1433.6 | Yes |
| **ADD1** | NP_054909.2 | 118 | alpha-adducin isoform c | **S358** | | | 1.11 | SPGsPVGEGTGSPPK | 2 | 5 | 1 | 0 | S4(Phospho); K15(Label:13C(6)15N(2)) | S(1): 1.8; S(4): 98.2; T(10): 0.0; S(12): 0.0 | AKSRSPGsPVGEGTG | S4; | 1441.6 | Yes |
| **ADD1** | NP_054909.2 | 118 | alpha-adducin isoform c | **S358** | | 1.64 | 1.11 | SPGsPVGEGTGSPPKWQIGEQEFEALMR | 1 | 5 | 1 | 1 | S4(Phospho); K15(Label:13C(6)15N(2)); R28(Label:13C(6)) | S(1): 1.4; S(4): 98.6; T(10): 0.0; S(12): 0.0 | AKSRSPGsPVGEGTG | S4; | 3065.4 | Yes |
| **ADD2** | NP_001171983.1 | 119 | beta-adducin isoform a | **T611** | | | #N/A | EAEtKSPLVSPSK | 2 | 1 | 1 | 1 | S10(Phospho); S12(Phospho) | T(4): 93.6; S(6): 4.4; S(10): 51.0; S(12): 51.0 | SPAKEAEtKSPLVSP | T4; | 1532.7 | Yes |
| **ADD3** | NP_001112.2 | 120 | gamma-adducin isoform b | **S42** | | 0.79 | 1.03 | NMsPDLR | 1 | 2 | 1 | 0 | S3(Phospho) | S(3): 100.0 | YIRERNMsPDLRQDF | S3; | 912.4 | Yes |
| **ADGB** | NP_078970.3 | 79747 | androglobin | **S1431** | | | #N/A | KTSDAESPPISEsQTKPK | 1 | 1 | 1 | 1 | K1(Label:13C(6)15N(2)); T15(Phospho) | T(2): 0.0; S(3): 0.0; S(7): 2.5; S(11): 2.5; S(13): 92.6; T(15): 2.5 | ESPPISEsQTKPKEE | S13; | 2017.9 | No |

| GeneSymbol | NP_Accession | Gene ID | ProteinDescription | PhosphoSite (Protein) | SCC-R / SCC-S R1 | SCC-R / SCC-S R2 | SCC-R / SCC-S Total Proteome | Sequence | # PSMs | # Proteins | # Protein Groups | # Missed Cleavages | Modifications | phosphoRS Site Probabilities | Phosphowindow | PhosphoSite (Peptide) | MH+ [Da] | PhosphositePlusEvidence |
|---|---|---|---|---|---|---|---|---|---|---|---|---|---|---|---|---|---|---|
| **ADH1C** | NP_000660.1 | 126 | alcohol dehydrogenase 1C | **T191; S207** | | | #N/A | VAKVtPGSTCAVFGLGGVGLsVVMGCK | 1 | 1 | 1 | 1 | K3(Label:13C(6)15N(2)); T5(Phospho); C10(Carbamidomethyl); S21(Phospho); C26(Carbamidomethyl); K27(Label:13C(6)15N(2)) | T(5): 99.4; S(8): 0.3; T(9): 0.3; S(21): 100.0 | AVKVAKVtPGSTCAV; GLGGVGLsVVMGCKA | T5; S21; | 2827.3 | No;No |
| **ADNP** | NP_852107.1 | 23394 | activity-dependent neuroprotector homeobox protein | **S953** | | 0.31 | 0.55 | LMHNAsDSEVDQDDVVEWK | 4 | 1 | 1 | 0 | S6(Phospho); K19(Label:13C(6)15N(2)) | S(6): 100.0; S(8): 0.0 | TKLMHNAsDSEVDQD | S6; | 2305.0 | Yes |
| **ADNP** | NP_852107.1 | 23394 | activity-dependent neuroprotector homeobox protein | **S953** | | | 0.55 | LMHNAsDSEVDQDDVVEWK | 2 | 1 | 1 | 0 | S6(Phospho) | S(6): 98.4; S(8): 1.6 | TKLMHNAsDSEVDQD | S6; | 2296.9 | Yes |
| **ADNP** | NP_852107.1 | 23394 | activity-dependent neuroprotector homeobox protein | **S953; S955** | | | 0.55 | LMHNAsDsEVDQDDVVEWK | 1 | 1 | 1 | 0 | S6(Phospho); S8(Phospho); K19(Label:13C(6)15N(2)) | S(6): 100.0; S(8): 100.0 | TKLMHNAsDSEVDQD; LMHNASDsEVDQDDV | S6; S8; | 2384.9 | Yes;Yes |
| **ADRB2** | NP_000015.1 | 154 | beta-2 adrenergic receptor | **S246** | | 10.66 | #N/A | FHVQNLsQVEQDGR | 2 | 1 | 1 | 0 | S7(Phospho) | S(7): 100.0 | RFHVQNLsQVEQDGR | S7; | 1736.8 | Yes |
| **ADRBK1** | NP_001610.2 | 156 | beta-adrenergic receptor kinase 1 | **S670** | | 0.91 | 0.91 | sPVVELSKVPLVQR | 2 | 1 | 1 | 1 | S1(Phospho) | S(1): 100.0; S(7): 0.0 | KMKNKPRsPVVELSK | S1; | 1630.9 | Yes |
| **ADRBK1** | NP_001610.2 | 156 | beta-adrenergic receptor kinase 1 | **S670** | | | 0.91 | sPVVELSKVPLVQR | 1 | 1 | 1 | 1 | S1(Phospho); K8(Label:13C(6)15N(2)); R14(Label:13C(6)) | S(1): 100.0; S(7): 0.0 | KMKNKPRsPVVELSK | S1; | 1644.9 | Yes |
| **AEBP2** | NP_694939.2 | 121536 | zinc finger protein AEBP2 isoform a | **S24** | 1.35 | | #N/A | LSPLPPGsPGSAAR | 1 | 2 | 1 | 0 | S8(Phospho); R14(Label:13C(6)) | S(2): 0.0; S(8): 100.0; S(11): 0.0 | LSPLPPGsPGSAARG | S8; | 1392.7 | Yes |
| **AFAP1L1** | NP_001139809.1 | 134265 | actin filament-associated protein 1-like 1 isoform 2 | **S329** | | | #N/A | EVSKPVGGAEGVEVPRsPVLLCK | 1 | 2 | 1 | 1 | S17(Phospho); C22(Carbamidomethyl) | S(3): 0.0; S(17): 100.0 | EGVEVPRsPVLLCKL | S17; | 2487.3 | Yes |
| **AFAP1L2** | NP_115939.1 | 84632 | actin filament-associated protein 1-like 2 isoform 2 | **Y413** | | | #NQ | VAQQPLSLVGCEVVPDPSPDHLySFR | 2 | 3 | 1 | 0 | C11(Carbamidomethyl); Y23(Phospho); R26(Label:13C(6)) | S(7): 0.0; S(18): 0.0; Y(23): 98.2; S(24): 1.8 | DPSPDHLySFRILHK | Y23; | 2996.4 | Yes |
| **AFAP1L2** | NP_115939.1 | 84632 | actin filament-associated protein 1-like 2 isoform 2 | **S640** | | | #NQ | ISFPPSCPDAVVATPPGAsPPVKDR | 2 | 3 | 1 | 1 | C7(Carbamidomethyl); S19(Phospho); K23(Label:13C(6)15N(2)); R25(Label:13C(6)) | S(2): 0.0; S(6): 0.0; T(14): 0.0; S(19): 100.0 | VATPPGAsPPVKDRL | S19; | 2656.3 | No |
| **AFF1** | NP_005926.1 | 4299 | AF4/FMR2 family member 1 isoform 2 | **S750** | 0.39 | 0.54 | #N/A | LLsPLRDTPPPQSLMVK | 2 | 2 | 1 | 1 | S3(Phospho); R6(Label:13C(6)); K17(Label:13C(6)15N(2)) | S(3): 100.0; T(8): 0.0; S(13): 0.0 | LRDTKLLsPLRDTPP | S3; | 1986.1 | Yes |
| **AFF1** | NP_005926.1 | 4299 | AF4/FMR2 family member 1 isoform 2 | **S750** | | | #N/A | LLsPLRDTPPPQSLMVK | 1 | 2 | 1 | 1 | S3(Phospho) | S(3): 100.0; T(8): 0.0; S(13): 0.0 | LRDTKLLsPLRDTPP | S3; | 1972.0 | Yes |
| **AFF4** | NP_055238.1 | 27125 | AF4/FMR2 family member 4 | **S1058** | 0.84 | 0.87 | 0.88 | AVGMPSPVsPK | 11 | 1 | 1 | 0 | S9(Phospho) | S(6): 0.0; S(9): 100.0 | VGMPSPVsPKLSPGN | S9; | 1149.5 | Yes |
| **AFF4** | NP_055238.1 | 27125 | AF4/FMR2 family member 4 | **S1058** | | | 0.88 | AVGMPSPVsPK | 7 | 1 | 1 | 0 | S9(Phospho); K11(Label:13C(6)15N(2)) | S(6): 0.0; S(9): 100.0 | VGMPSPVsPKLSPGN | S9; | 1157.6 | Yes |
| **AFF4** | NP_055238.1 | 27125 | AF4/FMR2 family member 4 | **S1058** | 0.72 | | 0.88 | AVGMPSPVsPK | 3 | 1 | 1 | 0 | M4(Oxidation); S9(Phospho); K11(Label:13C(6)15N(2)) | S(6): 0.0; S(9): 100.0 | VGMPSPVsPKLSPGN | S9; | 1173.5 | Yes |
| **AFF4** | NP_055238.1 | 27125 | AF4/FMR2 family member 4 | **S1058** | | | 0.88 | AVGMPSPVsPK | 4 | 1 | 1 | 0 | M4(Oxidation); S9(Phospho) | S(6): 0.0; S(9): 100.0 | VGMPSPVsPKLSPGN | S9; | 1165.5 | Yes |
| **AFF4** | NP_055238.1 | 27125 | AF4/FMR2 family member 4 | **S694** | | 0.66 | 0.88 | MFsPMEEK | 2 | 1 | 1 | 0 | S3(Phospho) | S(3): 100.0 | FFRQRMFsPMEEKEL | S3; | 1078.4 | Yes |
| **AFF4** | NP_055238.1 | 27125 | AF4/FMR2 family member 4 | **S694** | | 0.68 | 0.88 | MFsPMEEK | 3 | 1 | 1 | 0 | S3(Phospho); K8(Label:13C(6)15N(2)) | S(3): 100.0 | FFRQRMFsPMEEKEL | S3; | 1086.4 | Yes |
| **AFF4** | NP_055238.1 | 27125 | AF4/FMR2 family member 4 | **S1043** | | 0.78 | 0.88 | NSYNNSQAPsPGLGSK | 2 | 1 | 1 | 0 | S10(Phospho) | S(2): 0.0; Y(3): 0.0; S(6): 0.0; S(10): 98.2; S(15): 1.8 | YNNSQAPsPGLGSKA | S10; | 1700.7 | Yes |
| **AFF4** | NP_055238.1 | 27125 | AF4/FMR2 family member 4 | **S694** | | 0.66 | 0.88 | MFsPMEEK | 2 | 1 | 1 | 0 | S3(Phospho); M5(Oxidation); K8(Label:13C(6)15N(2)) | S(3): 100.0 | FFRQRMFsPMEEKEL | S3; | 1102.4 | Yes |
| **AGAP1** | NP_055729.2 | 116987 | arf-GAP with GTPase, ANK repeat and PH domain-containing protein 1 isoform 2 | **T783** | 4.4 | | #N/A | FVLMAtPNLSR | 4 | 2 | 1 | 0 | T6(Phospho) | T(6): 100.0; S(10): 0.0 | ERFVLMAtPNLSRRN | T6; | 1328.6 | No |
| **AGAP1** | NP_055729.2 | 116987 | arf-GAP with GTPase, ANK repeat and PH domain-containing protein 1 isoform 2 | **S468** | 1.78 | 1.86 | #N/A | LDPPPsPHANR | 3 | 2 | 1 | 0 | S6(Phospho) | S(6): 100.0 | PKLDPPPsPHANRKK | S6; | 1280.6 | No |
| **AGAP1** | NP_055729.2 | 116987 | arf-GAP with GTPase, ANK repeat and PH domain-containing protein 1 isoform 2 | **T783** | | | #N/A | FVLMAtPNLSR | 1 | 2 | 1 | 0 | T6(Phospho); R11(Label:13C(6)) | T(6): 100.0; S(10): 0.0 | ERFVLMAtPNLSRRN | T6; | 1334.7 | No |
| **AGAP2** | NP_055585.1 | 116986 | arf-GAP with GTPase, ANK repeat and PH domain-containing protein 2 isoform PIKE-A | **S184** | 1.19 | | #N/A | IsASSPR | 1 | 2 | 1 | 0 | S2(Phospho) | S(2): 100.0; S(4): 0.0; S(5): 0.0 | VGTQDRIsASSPRVV | S2; | 797.4 | No |
| **AGAP3** | NP_114152.3 | 116988 | arf-GAP with GTPase, ANK repeat and PH domain-containing protein 3 isoform a | **S574** | 1.25 | 1.15 | #NQ | LDPPPsPHSNR | 5 | 1 | 1 | 0 | S6(Phospho) | S(6): 100.0; S(9): 0.0 | PKLDPPPsPHSNRKK | S6; | 1296.6 | No |
| **AGAP3** | NP_114152.3 | 116988 | arf-GAP with GTPase, ANK repeat and PH domain-containing protein 3 isoform a | **T362** | | 3.21 | #NQ | IETIAASSTPtPIR | 2 | 3 | 1 | 0 | T11(Phospho) | T(3): 0.0; S(7): 0.0; S(8): 0.0; T(9): 1.1; T(11): 98.9 | IAASSTPtPIRKQSK | T11; | 1536.8 | No |
| **AGAP3** | NP_114152.3 | 116988 | arf-GAP with GTPase, ANK repeat and PH domain-containing protein 3 isoform a | **T360** | | 3.3 | #NQ | IETIAASStPTPIRK | 3 | 3 | 1 | 1 | T9(Phospho) | T(3): 0.0; S(7): 0.0; S(8): 0.0; T(9): 100.0; T(11): 0.0 | ETIAASStPTPIRKQ | T9; | 1664.9 | No |
| **AGAP3** | NP_114152.3 | 116988 | arf-GAP with GTPase, ANK repeat and PH domain-containing protein 3 isoform a | **S574** | | 1.39 | #NQ | LDPPPsPHSNR | 1 | 1 | 1 | 0 | S6(Phospho); R11(Label:13C(6)) | S(6): 100.0; S(9): 0.0 | PKLDPPPsPHSNRKK | S6; | 1302.6 | No |
| **AGAP3** | NP_114152.3 | 116988 | arf-GAP with GTPase, ANK repeat and PH domain-containing protein 3 isoform a | **T478** | | 0.92 | #NQ | ATPATAPGtSPR | 1 | 2 | 1 | 0 | S10(Phospho); R12(Label:13C(6)) | T(2): 0.0; T(5): 0.0; T(9): 99.1; S(10): 0.9 | TPATAPGtSPRANGL | T9; | 1212.6 | No |
| **AGAP3** | NP_114152.3 | 116988 | arf-GAP with GTPase, ANK repeat and PH domain-containing protein 3 isoform a | **S479** | | | #NQ | ATPATAPGTsPR | 1 | 2 | 1 | 0 | S10(Phospho) | T(2): 0.0; T(5): 0.0; T(9): 0.0; S(10): 100.0 | PATAPGTsPRANGLS | S10; | 1206.6 | No |
| **AGAP3** | NP_114152.3 | 116988 | arf-GAP with GTPase, ANK repeat and PH domain-containing protein 3 isoform a | **T362** | | | #NQ | IETIAASSTPtPIR | 1 | 3 | 1 | 0 | T9(Phospho); R14(Label:13C(6)) | T(3): 0.0; S(7): 0.0; S(8): 0.0; T(9): 0.0; T(11): 100.0 | IAASSTPtPIRKQSK | T11; | 1542.8 | No |
| **AGBL2** | NP_079059.2 | 79841 | cytosolic carboxypeptidase 2 | **S168** | | | #N/A | EPQELFsILSTK | 2 | 1 | 1 | 0 | S7(Phospho); S10(Phospho) | S(7): 100.0; S(10): 50.0; T(11): 50.0 | REPQELFsILSTKRP | S7; | 1551.7 | No |
| **AGFG1** | NP_001128661.1 | 3267 | arf-GAP domain and FG repeat-containing protein 1 isoform 4 | **S181** | 1.26 | 1.36 | 1.03 | GTPSQsPVVGR | 6 | 4 | 1 | 0 | S6(Phospho) | T(2): 0.0; S(4): 0.0; S(6): 100.0 | NKGTPSQsPVVGRSQ | S6; | 1164.5 | Yes |
| **AGFG1** | NP_001128661.1 | 3267 | arf-GAP domain and FG repeat-containing protein 1 isoform 4 | **T177; S181** | 2.41 | 2.32 | 1.03 | SLLGDSAPTLHLNKGtPSQsPVVGR | 6 | 4 | 1 | 1 | T16(Phospho); S20(Phospho) | S(1): 0.0; S(6): 0.0; T(9): 0.0; T(16): 100.0; S(18): 0.0; S(20): 100.0 | TLHLNKGtPSQSPVV;NKGTPSQsPVVGRSQ | T16; S20; | 2691.3 | Yes;Yes |
| **AGFG1** | NP_001128661.1 | 3267 | arf-GAP domain and FG repeat-containing protein 1 isoform 4 | **T177; S181** | | | 1.03 | SLLGDSAPTLHLNKGtPSQsPVVGR | 2 | 4 | 1 | 1 | K14(Label:13C(6)15N(2)); S18(Phospho); S20(Phospho); R25(Label:13C(6)) | S(1): 0.0; S(6): 0.0; T(9): 0.0; T(16): 100.0; S(18): 0.0; S(20): 100.0 | TLHLNKGtPSQSPVV;NKGTPSQsPVVGRSQ | T16; S20; | 2705.3 | Yes;Yes |
| **AGFG1** | NP_001128661.1 | 3267 | arf-GAP domain and FG repeat-containing protein 1 isoform 4 | **S181** | 1.32 | | 1.03 | GTPSQsPVVGR | 3 | 4 | 1 | 0 | S6(Phospho); R11(Label:13C(6)) | T(2): 0.0; S(4): 0.0; S(6): 100.0 | NKGTPSQsPVVGRSQ | S6; | 1170.6 | Yes |
| **AGPS** | NP_003650.1 | 8540 | alkyldihydroxyacetonephosphate synthase, peroxisomal precursor | **S65** | 1.68 | | 0.62 | RAAsAATAAPTATPAAQESGTIPK | 1 | 1 | 1 | 1 | R1(Label:13C(6)); S4(Phospho); K24(Label:13C(6)15N(2)) | S(4): 100.0; T(7): 0.0; T(11): 0.0; T(13): 0.0; S(19): 0.0; T(21): 0.0 | CKARRAAsAATAAPT | S4; | 2333.2 | Yes |
| **AGTPBP1** | NP_001273646.1 | 23287 | cytosolic carboxypeptidase 1 isoform c | **T31** | | | #N/A | MRTGSAASSAAAAAAAAAASASPATGVCMKtPGGGR | 1 | 2 | 1 | 2 | R2(Label:13C(6)); T25(Phospho); C28(Carbamidomethyl); M29(Oxidation); T31(Phospho) | T(3): 43.5; S(5): 43.5; S(8): 5.8; S(9): 5.8; S(20): 10.4; S(22): 10.4; T(25): 2.1; T(31): 78.6 | ATGVCMKtPGGGRRG | T31; | 3374.5 | No |
| **AHCTF1** | NP_056261.4 | 25909 | protein ELYS | **S1907** | | | 0.32 | VSTVTsPSR | 1 | 1 | 1 | 0 | S6(Phospho); R9(Label:13C(6)) | S(2): 0.0; T(3): 0.0; T(5): 1.1; S(6): 97.9; S(8): 1.1 | LKVSTVTsPSRMIRK | S6; | 1019.5 | No |
| **AHCTF1** | NP_056261.4 | 25909 | protein ELYS | **S1550** | | 0.23 | 0.32 | NLsFNELYPSGTLK | 2 | 1 | 1 | 0 | S3(Phospho); K14(Label:13C(6)15N(2)) | S(3): 100.0; Y(8): 0.0; S(10): 0.0; T(12): 0.0 | GEEARNLsFNELYPS | S3; | 1670.8 | No |
| **AHCTF1** | NP_056261.4 | 25909 | protein ELYS | **S1550** | | | 0.32 | NLsFNELYPSGTLK | 1 | 1 | 1 | 0 | S3(Phospho) | S(3): 100.0; Y(8): 0.0; S(10): 0.0; T(12): 0.0 | GEEARNLsFNELYPS | S3; | 1662.8 | No |
| **AHCTF1** | NP_056261.4 | 25909 | protein ELYS | **S1953** | | | 0.32 | GREVsPSDVR | 1 | 1 | 1 | 1 | R2(Label:13C(6)); S5(Phospho); R10(Label:13C(6)) | S(5): 100.0; S(7): 0.0 | RVRGREVsPSDVRED | S5; | 1193.6 | No |
| **AHCTF1** | NP_056261.4 | 25909 | protein ELYS | **S537** | | 0.52 | 0.32 | CLVAGLLsPR | 1 | 1 | 1 | 0 | C1(Carbamidomethyl); S8(Phospho); R10(Label:13C(6)) | S(8): 100.0 | CLVAGLLsPRFVDVQ | S8; | 1171.6 | No |
| **AHDC1** | NP_001025053.1 | 27245 | AT-hook DNA-binding motif-containing protein 1 | **S1064** | | | #N/A | ASTVsPGGYMVPK | 2 | 1 | 1 | 0 | S5(Phospho); K13(Label:13C(6)15N(2)) | S(2): 0.0; T(3): 0.0; S(5): 100.0; Y(9): 0.0 | DSRASTVsPGGYMVP | S5; | 1381.6 | Yes |
| **AHDC1** | NP_001025053.1 | 27245 | AT-hook DNA-binding motif-containing protein 1 | **T887; S916** | | | #N/A | GLAtFPSRGAKASPVAVGSSGAGADPSFQPVLsAR | 1 | 1 | 1 | 2 | T4(Phospho); S33(Phospho) | T(4): 91.4; S(7): 8.4; S(13): 0.2; S(19): 0.2; S(20): 7.7; S(27): 7.7; S(33): 84.5 | PAQRGLAtFPSRGAK; PSFQPVLsARQTFPP | T4; S33; | 3473.6 | No;No |
| **AHNAK** | NP_001611.1 | 79026 | neuroblast differentiation-associated protein AHNAK isoform 1 | **S5752** | 1.9 | | 1.70 | ASLGsLEGEAEAEASSPK | 3 | 1 | 1 | 0 | S5(Phospho) | S(2): 0.0; S(5): 100.0; S(15): 0.0; S(16): 0.0 | SSKASLGsLEGEAEA | S5; | 1812.8 | Yes |
| **AHNAK** | NP_001611.1 | 79026 | neuroblast differentiation-associated protein AHNAK isoform 1 | **S216** | 1.41 | 1.81 | 1.70 | LPSGSGAAsPTGSAVDIR | 22 | 1 | 1 | 0 | S9(Phospho) | S(3): 0.0; S(5): 0.0; S(9): 100.0; T(11): 0.0; S(13): 0.0 | PSGSGAAsPTGSAVD | S9; | 1722.8 | Yes |
| **AHNAK** | NP_001611.1 | 79026 | neuroblast differentiation-associated protein AHNAK isoform 1 | **S511** | 1.91 | 1.2 | 1.70 | ISMQDVDLSLGsPK | 8 | 1 | 1 | 0 | S12(Phospho) | S(2): 0.0; S(9): 0.0; S(12): 100.0 | DVDLSLGsPKLKGDI | S12; | 1569.7 | Yes |
| **AHNAK** | NP_001611.1 | 79026 | neuroblast differentiation-associated protein AHNAK isoform 1 | **T4564** | 4.16 | | 1.70 | VGIDtPDIDIHGPEGK | 4 | 1 | 1 | 0 | T5(Phospho) | T(5): 100.0 | GPKVGIDtPDIDIHG | T5; | 1742.8 | Yes |
| **AHNAK** | NP_001611.1 | 79026 | neuroblast differentiation-associated protein AHNAK isoform 1 | **S212; S216** | 4.74 | | 1.70 | LPSGsGAAsPTGSAVDIR | 4 | 1 | 1 | 0 | S5(Phospho); S9(Phospho) | S(3): 0.0; S(5): 100.0; S(9): 100.0; T(11): 0.0; S(13): 0.0 | VIRLPSGsGAASPTG;PSGSGAAsPTGSAVD | S5; S9; | 1802.8 | Yes;Yes |
| **AHNAK** | NP_001611.1 | 79026 | neuroblast differentiation-associated protein AHNAK isoform 1 | **S135** | 2.27 | 2.22 | 1.70 | LKsEDGVEGDLGETQSR | 12 | 1 | 1 | 1 | S3(Phospho) | S(3): 100.0; T(14): 0.0; S(16): 0.0 | KIKPRLKsEDGVEGD | S3; | 1899.8 | Yes |

| GeneSymbol | NP_Accession | Gene ID | ProteinDescription | PhosphoSite (Protein) | SCC-R / SCC-S R1 | SCC-R / SCC-S R2 | SCC-R / SCC-S Total Proteome | Sequence | # PSMs | # Proteins | # Protein Groups | # Missed Cleavages | Modifications | phosphoRS Site Probabilities | Phosphowindow | PhosphoSite (Peptide) | MH+ [Da] | PhosphositePlusEvidence |
|---|---|---|---|---|---|---|---|---|---|---|---|---|---|---|---|---|---|---|
| AHNAK | NP_001611.1 | 79026 | neuroblast differentiation-associated protein AHNAK isoform 1 | S5752 | | | 1.70 | ASLGsLEGEAEAEASSPK | 2 | 1 | 1 | 0 | S5(Phospho); K18(Label:13C(6)15N(2)) | S(2): 0.0; S(5): 100.0; S(15): 0.0; S(16): 0.0 | SSKASLGsLEGEAEA | S5; | 1820.8 | Yes |
| AHNAK | NP_001611.1 | 79026 | neuroblast differentiation-associated protein AHNAK isoform 1 | S5782 | 1.21 | 1.26 | 1.70 | HRSNsFSDER | 5 | 1 | 1 | 1 | S5(Phospho) | S(3): 0.0; S(5): 100.0; S(7): 0.0 | KPRHRSNsFSDEREF | S5; | 1314.5 | Yes |
| AHNAK | NP_001611.1 | 79026 | neuroblast differentiation-associated protein AHNAK isoform 1 | S216 | 1.25 | 1.29 | 1.70 | TVIRLPSGSGAAsPTGSAVDIR | 6 | 1 | 1 | 1 | S13(Phospho) | T(1): 0.0; S(7): 0.0; S(9): 0.0; S(13): 100.0; T(15): 0.0; S(17): 0.0 | PSGSGAAsPTGSAVD | S13; | 2192.1 | Yes |
| AHNAK | NP_001611.1 | 79026 | neuroblast differentiation-associated protein AHNAK isoform 1 | S5790 | | 1.97 | 1.70 | EFsGPSTPTGTLEFEGGEVSLEGKVK | 4 | 1 | 1 | 1 | S3(Phospho) | S(3): 99.9; S(6): 0.0; T(7): 0.1; T(9): 0.0; T(11): 0.0; S(20): 0.0 | FSDEREFsGPSTPTG | S3; | 2819.3 | Yes |
| AHNAK | NP_001611.1 | 79026 | neuroblast differentiation-associated protein AHNAK isoform 1 | S5448 | 1.34 | 1.34 | 1.70 | IsAPNVDFNLEGPKVK | 4 | 1 | 1 | 1 | S2(Phospho) | S(2): 100.0 | NLKGPRIsAPNVDFN | S2; | 1807.9 | Yes |
| AHNAK | NP_001611.1 | 79026 | neuroblast differentiation-associated protein AHNAK isoform 1 | S511 | 2.27 | 2.03 | 1.70 | ISMQDVDLSLGsPKLKGDIK | 3 | 1 | 1 | 2 | S12(Phospho) | S(2): 0.0; S(9): 0.0; S(12): 100.0 | DVDLSLGsPKLKGDI | S12; | 2224.1 | Yes |
| AHNAK | NP_001611.1 | 79026 | neuroblast differentiation-associated protein AHNAK isoform 1 | S5731 | 2.52 | 1.9 | 1.70 | GGVTGsPEASISGSKGDLK | 5 | 1 | 1 | 1 | S6(Phospho) | T(4): 0.0; S(6): 100.0; S(10): 0.0; S(12): 0.0; S(14): 0.0 | GKGGVTGsPEASISG | S6; | 1826.9 | Yes |
| AHNAK | NP_001611.1 | 79026 | neuroblast differentiation-associated protein AHNAK isoform 1 | T5824 | 3.81 | 3.15 | 1.70 | FGtFGGLGSK | 14 | 1 | 1 | 0 | T3(Phospho) | T(3): 100.0; S(9): 0.0 | HGKLKFGtFGGLGSK | T3; | 1050.5 | Yes |
| AHNAK | NP_001611.1 | 79026 | neuroblast differentiation-associated protein AHNAK isoform 1 | S93 | | | 1.70 | KGDRsPEPGQTWTR | 6 | 2 | 1 | 2 | S5(Phospho) | S(5): 100.0; T(11): 0.0; T(13): 0.0 | LHRKGDRsPEPGQTW | S5; | 1694.8 | Yes |
| AHNAK | NP_001611.1 | 79026 | neuroblast differentiation-associated protein AHNAK isoform 1 | S5790 | | 2.03 | 1.70 | EFsGPSTPTGTLEFEGGEVSLEGKVK | 4 | 1 | 1 | 1 | S3(Phospho); K25(Label:13C(6)15N(2)); K27(Label:13C(6)15N(2)) | S(3): 100.0; S(6): 0.0; T(7): 0.0; T(9): 0.0; T(11): 0.0; S(20): 0.0 | FSDEREFsGPSTPTG | S3; | 2835.3 | Yes |
| AHNAK | NP_001611.1 | 79026 | neuroblast differentiation-associated protein AHNAK isoform 1 | T4564 | | | 1.70 | VGIDtPDIDIHGPEGK | 2 | 1 | 1 | 0 | T5(Phospho); K16(Label:13C(6)15N(2)) | T(5): 100.0 | GPKVGIDtPDIDIHG | T5; | 1750.8 | Yes |
| AHNAK | NP_001611.1 | 79026 | neuroblast differentiation-associated protein AHNAK isoform 1 | S5110 | | 5.07 | 1.70 | FKAEAPLPsPK | 15 | 1 | 1 | 1 | S9(Phospho) | S(9): 100.0 | KAEAPLPsPKLEGEL | S9; | 1264.6 | Yes |
| AHNAK | NP_001611.1 | 79026 | neuroblast differentiation-associated protein AHNAK isoform 1 | S5099 | 6.16 | 7.67 | 1.70 | MYFPDVEFDIKsPK | 4 | 1 | 1 | 1 | S12(Phospho) | Y(2): 0.0; S(12): 100.0 | DVEFDIKsPKFKAEA | S12; | 1795.8 | Yes |
| AHNAK | NP_001611.1 | 79026 | neuroblast differentiation-associated protein AHNAK isoform 1 | S511 | 2.35 | 2.55 | 1.70 | ISMQDVDLSLGsPKLK | 3 | 1 | 1 | 1 | S12(Phospho) | S(2): 0.0; S(9): 0.0; S(12): 100.0 | DVDLSLGsPKLKGDI | S12; | 1810.9 | Yes |
| AHNAK | NP_001611.1 | 79026 | neuroblast differentiation-associated protein AHNAK isoform 1 | S5731; S5739 | 1.43 | | 1.70 | GGVTGsPEASISGsKGDLK | 1 | 1 | 1 | 1 | S6(Phospho); S14(Phospho) | T(4): 0.0; S(6): 100.0; S(10): 0.0; S(12): 0.0; S(14): 100.0 | GKGGVTGsPEASISG; PEASISGsKGDLKSS | S6; S14; | 1906.8 | Yes;Yes |
| AHNAK | NP_001611.1 | 79026 | neuroblast differentiation-associated protein AHNAK isoform 1 | S3426 | | | 1.70 | VSMPDVELNLKsPK | 5 | 1 | 1 | 1 | S12(Phospho) | S(2): 0.0; S(12): 100.0 | DVELNLKsPKVKGDL | S12; | 1636.8 | Yes |
| AHNAK | NP_001611.1 | 79026 | neuroblast differentiation-associated protein AHNAK isoform 1 | S511 | | | 1.70 | ISMQDVDLSLGsPKLK | 2 | 1 | 1 | 1 | S12(Phospho); K14(Label:13C(6)15N(2)); K16(Label:13C(6)15N(2)) | S(2): 0.0; S(9): 0.0; S(12): 100.0 | DVDLSLGsPKLKGDI | S12; | 1826.9 | Yes |
| AHNAK | NP_001611.1 | 79026 | neuroblast differentiation-associated protein AHNAK isoform 1 | S177 | 2.73 | 3.21 | 1.70 | DIDISsPEFKIK | 5 | 1 | 1 | 1 | S6(Phospho) | S(5): 0.0; S(6): 100.0 | AKDIDISsPEFKIKI | S6; | 1471.7 | Yes |
| AHNAK | NP_001611.1 | 79026 | neuroblast differentiation-associated protein AHNAK isoform 1 | S5110 | 4.94 | 11.2 | 1.70 | AEAPLPsPK | 5 | 1 | 1 | 0 | S7(Phospho) | S(7): 100.0 | KAEAPLPsPKLEGEL | S7; | 989.5 | Yes |
| AHNAK | NP_001611.1 | 79026 | neuroblast differentiation-associated protein AHNAK isoform 1 | S4986 | | 2.83 | 1.70 | FGFGAKsPK | 4 | 1 | 1 | 1 | S7(Phospho) | S(7): 100.0 | KFGFGAKsPKADIKS | S7; | 1018.5 | Yes |
| AHNAK | NP_001611.1 | 79026 | neuroblast differentiation-associated protein AHNAK isoform 1 | S5110 | 6.71 | 6.82 | 1.70 | FKAEAPLPsPKLEGELQAPDLELSLPAIHVEGLDIK | 10 | 1 | 1 | 2 | S9(Phospho) | S(9): 100.0; S(24): 0.0 | KAEAPLPsPKLEGEL | S9; | 3945.1 | Yes |
| AHNAK | NP_001611.1 | 79026 | neuroblast differentiation-associated protein AHNAK isoform 1 | S93 | 3.2 | | 1.70 | GDRsPEPGQTWTR | 3 | 2 | 1 | 1 | S4(Phospho) | S(4): 100.0; T(10): 0.0; T(12): 0.0 | LHRKGDRsPEPGQTW | S4; | 1566.7 | Yes |
| AHNAK | NP_001611.1 | 79026 | neuroblast differentiation-associated protein AHNAK isoform 1 | S511 | | | 1.70 | ISMQDVDLSLGsPKLKGDIK | 2 | 1 | 1 | 2 | S12(Phospho); K14(Label:13C(6)15N(2)); K16(Label:13C(6)15N(2)); K20(Label:13C(6)15N(2)) | S(2): 0.0; S(9): 0.0; S(12): 100.0 | DVDLSLGsPKLKGDI | S12; | 2248.2 | Yes |
| AHNAK | NP_001611.1 | 79026 | neuroblast differentiation-associated protein AHNAK isoform 1 | S177 | 2.82 | 2.68 | 1.70 | DIDISsPEFK | 2 | 1 | 1 | 0 | S6(Phospho); K10(Label:13C(6)15N(2)) | S(5): 0.8; S(6): 99.2 | AKDIDISsPEFKIKI | S6; | 1238.5 | Yes |
| AHNAK | NP_001611.1 | 79026 | neuroblast differentiation-associated protein AHNAK isoform 1 | S1445 | | | 1.70 | MPEMsIK | 2 | 1 | 1 | 0 | S5(Phospho) | S(5): 100.0 | KFKMPEMsIKPQKIS | S5; | 915.4 | Yes |
| AHNAK | NP_001611.1 | 79026 | neuroblast differentiation-associated protein AHNAK isoform 1 | S216 | 1.38 | 1.23 | 1.70 | LPSGSGAAsPTGSAVDIR | 5 | 1 | 1 | 0 | S9(Phospho); R18(Label:13C(6)) | S(3): 0.0; S(5): 0.0; S(9): 100.0; T(11): 0.0; S(13): 0.0 | PSGSGAAsPTGSAVD | S9; | 1728.8 | Yes |
| AHNAK | NP_001611.1 | 79026 | neuroblast differentiation-associated protein AHNAK isoform 1 | S216 | | | 1.70 | TVIRLPSGSGAAsPTGSAVDIR | 2 | 1 | 1 | 1 | R4(Label:13C(6)); S13(Phospho); R22(Label:13C(6)) | T(1): 0.0; S(7): 0.0; S(9): 0.0; S(13): 100.0; T(15): 0.0; S(17): 0.0 | PSGSGAAsPTGSAVD | S13; | 2204.1 | Yes |
| AHNAK | NP_001611.1 | 79026 | neuroblast differentiation-associated protein AHNAK isoform 1 | S135 | | | 1.70 | LKsEDGVEGDLGETQSR | 4 | 1 | 1 | 1 | K2(Label:13C(6)15N(2)); S3(Phospho); R17(Label:13C(6)) | S(3): 100.0; T(14): 0.0; S(16): 0.0 | KIKPRLKsEDGVEGD | S3; | 1913.9 | Yes |
| AHNAK | NP_001611.1 | 79026 | neuroblast differentiation-associated protein AHNAK isoform 1 | S5782 | 1.27 | | 1.70 | HRSNsFSDER | 1 | 1 | 1 | 1 | R2(Label:13C(6)); S5(Phospho); R10(Label:13C(6)) | S(3): 0.0; S(5): 100.0; S(7): 0.0 | KPRHRSNsFSDEREF | S5; | 1326.6 | Yes |
| AHNAK | NP_001611.1 | 79026 | neuroblast differentiation-associated protein AHNAK isoform 1 | S3412 | 4.48 | 4.68 | 1.70 | MPFLSISsPK | 8 | 1 | 1 | 0 | S8(Phospho) | S(5): 0.0; S(7): 0.8; S(8): 99.2 | MPFLSISsPKVSMPD | S8; | 1186.6 | Yes |
| AHNAK | NP_001611.1 | 79026 | neuroblast differentiation-associated protein AHNAK isoform 1 | S177 | 2.92 | 4.07 | 1.70 | EGAKDIDISsPEFK | 2 | 1 | 1 | 1 | S10(Phospho) | S(9): 0.0; S(10): 100.0 | AKDIDISsPEFKIKI | S10; | 1615.7 | Yes |
| AHNAK | NP_001611.1 | 79026 | neuroblast differentiation-associated protein AHNAK isoform 1 | S3412 | | | 1.70 | MPFLSISsPK | 2 | 1 | 1 | 0 | S8(Phospho); K10(Label:13C(6)15N(2)) | S(5): 0.0; S(7): 0.9; S(8): 99.1 | MPFLSISsPKVSMPD | S8; | 1194.6 | Yes |
| AHNAK | NP_001611.1 | 79026 | neuroblast differentiation-associated protein AHNAK isoform 1 | S5110 | | | 1.70 | FKAEAPLPsPK | 3 | 1 | 1 | 1 | K2(Label:13C(6)15N(2)); S9(Phospho); K11(Label:13C(6)15N(2)) | S(9): 100.0 | KAEAPLPsPKLEGEL | S9; | 1280.7 | Yes |
| AHNAK | NP_001611.1 | 79026 | neuroblast differentiation-associated protein AHNAK isoform 1 | S5731 | 1.4 | | 1.70 | GGVTGsPEASISGSK | 1 | 1 | 1 | 0 | S6(Phospho); K15(Label:13C(6)15N(2)) | T(4): 0.0; S(6): 100.0; S(10): 0.0; S(12): 0.0; S(14): 0.0 | GKGGVTGsPEASISG | S6; | 1421.6 | Yes |
| AHNAK | NP_001611.1 | 79026 | neuroblast differentiation-associated protein AHNAK isoform 1 | S212; S216 | | | 1.70 | LPSGsGAAsPTGSAVDIR | 1 | 1 | 1 | 0 | S5(Phospho); S9(Phospho); R18(Label:13C(6)) | S(3): 0.0; S(5): 100.0; S(9): 100.0; T(11): 0.0; S(13): 0.0 | VIRLPSGsGAASPTG;PSGSGAAsPTGSAVD | S5; S9; | 1808.8 | Yes;Yes |
| AHNAK | NP_001611.1 | 79026 | neuroblast differentiation-associated protein AHNAK isoform 1 | S5752 | | | 1.70 | SSKASLGsLEGEAEAEASSPK | 2 | 1 | 1 | 1 | K3(Label:13C(6)15N(2)); S5(Phospho); S8(Phospho); K21(Label:13C(6)15N(2)) | S(1): 49.5; S(2): 49.5; S(5): 1.0; S(8): 100.0; S(18): 0.0; S(19): 0.0 | SSKASLGsLEGEAEA | S8; | 2210.9 | Yes |
| AHNAK | NP_001611.1 | 79026 | neuroblast differentiation-associated protein AHNAK isoform 1 | S5782 | | 1.65 | 1.70 | SNsFSDEREFSGPSTPTGTLEFEGGEVSLEGGKVK | 1 | 1 | 1 | 2 | S11(Phospho) | S(1): 2.7; S(3): 97.3; S(5): 0.0; S(11): 0.0; S(14): 0.0; T(15): 0.0; T(17): 0.0; T(19): 0.0; S(28): 0.0 | KPRHRSNsFSDEREF | S3; | 3741.7 | Yes |
| AHNAK | NP_001611.1 | 79026 | neuroblast differentiation-associated protein AHNAK isoform 1 | S5830 | | | 1.70 | FGTFGGLGsK | 2 | 1 | 1 | 0 | S9(Phospho); K10(Label:13C(6)15N(2)) | T(3): 0.0; S(9): 100.0 | GTFGGLGsKSKGHYE | S9; | 1058.5 | Yes |
| AHNAK | NP_001611.1 | 79026 | neuroblast differentiation-associated protein AHNAK isoform 1 | S5077 | | 3.91 | 1.70 | VDVKsPK | 4 | 1 | 1 | 1 | S5(Phospho) | S(5): 100.0 | ALKVDVKsPKTKKTM | S5; | 852.4 | Yes |
| AHNAK | NP_001611.1 | 79026 | neuroblast differentiation-associated protein AHNAK isoform 1 | S5782 | | 1.23 | 1.70 | SNsFSDER | 2 | 1 | 1 | 0 | S3(Phospho) | S(1): 0.0; S(3): 100.0; S(5): 0.0 | KPRHRSNsFSDEREF | S3; | 1021.4 | Yes |
| AHNAK | NP_001611.1 | 79026 | neuroblast differentiation-associated protein AHNAK isoform 1 | S3360 | | | 1.70 | FNFsGSK | 1 | 1 | 1 | 0 | S4(Phospho) | S(4): 99.0; S(6): 1.0 | KLPKFNFsGSKVQTP | S4; | 866.3 | Yes |
| AHNAK | NP_001611.1 | 79026 | neuroblast differentiation-associated protein AHNAK isoform 1 | S2397 | | 4.44 | 1.70 | ISMPDLDLHLKsPK | 6 | 1 | 1 | 1 | S12(Phospho) | S(2): 0.0; S(12): 100.0 | DLDLHLKsPKAKGEV | S12; | 1673.8 | Yes |
| AHNAK | NP_001611.1 | 79026 | neuroblast differentiation-associated protein AHNAK isoform 1 | S5031 | | | 1.70 | IHMsGPK | 1 | 1 | 1 | 0 | S4(Phospho) | S(4): 100.0 | KMPKIHMsGPKIKAK | S4; | 849.4 | Yes |
| AHNAK | NP_001611.1 | 79026 | neuroblast differentiation-associated protein AHNAK isoform 1 | T5845 | | | 1.70 | GHYEVTGSDDEtGKLQGSGVSLASK | 1 | 1 | 1 | 1 | T12(Phospho) | Y(3): 0.0; T(6): 2.7; S(8): 2.7; T(12): 94.6; S(18): 0.0; S(21): 0.0; S(24): 0.0 | VTGSDDEtGKLQGSG | T12; | 2602.2 | Yes |
| AHNAK | NP_001611.1 | 79026 | neuroblast differentiation-associated protein AHNAK isoform 1 | S2397 | | 3.33 | 1.70 | ISMPDLDLHLKsPK | 2 | 1 | 1 | 1 | K11(Label:13C(6)15N(2)); S12(Phospho); K14(Label:13C(6)15N(2)) | S(2): 0.0; S(12): 100.0 | DLDLHLKsPKAKGEV | S12; | 1689.9 | Yes |
| AHNAK | NP_001611.1 | 79026 | neuroblast differentiation-associated protein AHNAK isoform 1 | S559 | | 1.92 | 1.70 | LGsPSGKTGTCR | 1 | 1 | 1 | 1 | S3(Phospho); C11(Carbamidomethyl) | S(3): 100.0; S(5): 0.0; T(8): 0.0; T(10): 0.0 | LTGPRLGsPSGKTGT | S3; | 1300.6 | Yes |
| AHNAK | NP_001611.1 | 79026 | neuroblast differentiation-associated protein AHNAK isoform 1 | S5739 | | 1.32 | 1.70 | GGVTGSPEASISGsKGDLK | 3 | 1 | 1 | 1 | S14(Phospho); K15(Label:13C(6)15N(2)); K19(Label:13C(6)15N(2)) | T(4): 0.0; S(6): 0.0; S(10): 0.0; S(12): 0.0; S(14): 100.0 | PEASISGsKGDLKSS | S14; | 1842.9 | Yes |

Supplementary Table S5:List of phosphopeptides identified in SCC-S and SCC-R cells using SequestHT and Mascot search algorithms

| GeneSymbol | NP_Accession | Gene ID | ProteinDescription | PhosphoSite (Protein) | SCC-R / SCC-S R1 | SCC-R / SCC-S R2 | SCC-R / SCC-S Total Proteome | Sequence | # PSMs | # Proteins | # Protein Groups | # Missed Cleavages | Modifications | phosphoRS Site Probabilities | Phosphowindow | PhosphoSite (Peptide) | MH+ [Da] | PhosphositePlusEvidence |
|---|---|---|---|---|---|---|---|---|---|---|---|---|---|---|---|---|---|---|
| **AHNAK** | NP_001611.1 | 79026 | neuroblast differentiation-associated protein AHNAK isoform 1 | **S4903** | | | 1.70 | LSGPsLK | 1 | 1 | 1 | 0 | S5(Phospho) | S(2): 0.0; S(5): 100.0 | DAKLSGPsLKMPSLE | S5; | 781.4 | Yes |
| **AHNAK** | NP_001611.1 | 79026 | neuroblast differentiation-associated protein AHNAK isoform 1 | **S177** | | | 1.70 | DIDISsPEFK | 1 | 1 | 1 | 0 | S6(Phospho) | S(5): 0.0; S(6): 100.0 | AKDIDISsPEFKIKI | S6; | 1230.5 | Yes |
| **AHNAK** | NP_001611.1 | 79026 | neuroblast differentiation-associated protein AHNAK isoform 1 | **S5763** | | | 1.70 | ASLGSLEGEAEAEASsPKGK | 1 | 1 | 1 | 1 | S16(Phospho) | S(2): 0.0; S(5): 0.0; S(15): 2.0; S(16): 98.0 | EAEAEASsPKGKFSL | S16; | 1997.9 | Yes |
| **AHNAK** | NP_001611.1 | 79026 | neuroblast differentiation-associated protein AHNAK isoform 1 | **S1158; S1170** | | | 1.70 | ADVVVsGPKVDIEAPDVsLEGPEGK | 1 | 1 | 1 | 1 | S6(Phospho); S18(Phospho) | S(6): 100.0; S(18): 100.0 | PKADVVVsGPKVDIE;DIEAPDVsLEGPEGK | S6; S18; | 2667.2 | Yes;Yes |
| **AHNAK** | NP_001611.1 | 79026 | neuroblast differentiation-associated protein AHNAK isoform 1 | **S5749; S5763** | | | 1.70 | AsLGSLEGEAEAEASsPKGK | 1 | 1 | 1 | 1 | S5(Phospho); S15(Phospho) | S(2): 95.6; S(5): 4.4; S(15): 4.4; S(16): 95.6 | DLKSSKAsLGSLEGE;EAEAEASsPKGKFSL | S2; S16; | 2077.9 | Yes;Yes |
| **AHNAK** | NP_001611.1 | 79026 | neuroblast differentiation-associated protein AHNAK isoform 1 | **S210; S212** | | | 1.70 | TVIRLPsGsGAASPTGSAVDIR | 1 | 1 | 1 | 1 | S9(Phospho); S13(Phospho) | T(1): 0.0; S(7): 96.1; S(9): 96.1; S(13): 7.8; T(15): 0.0; S(17): 0.0 | KTVIRLPsGSGAASP;VIRLPSGsGAASPTG | S7; S9; | 2272.1 | Yes;Yes |
| **AHNAK** | NP_001611.1 | 79026 | neuroblast differentiation-associated protein AHNAK isoform 1 | **S2397** | | **5.68** | 1.70 | ISMPDLDLHLKsPK | 1 | 1 | 1 | 1 | M3(Oxidation); S12(Phospho) | S(2): 0.0; S(12): 100.0 | DLDLHLKsPKAKGEV | S12; | 1689.8 | Yes |
| **AHNAK** | NP_001611.1 | 79026 | neuroblast differentiation-associated protein AHNAK isoform 1 | **S5763** | | | 1.70 | ASLGSLEGEAEAEASsPKGK | 1 | 1 | 1 | 1 | S16(Phospho); K18(Label:13C(6)15N(2)); K20(Label:13C(6)15N(2)) | S(2): 0.0; S(5): 0.0; S(15): 2.4; S(16): 97.6 | EAEAEASsPKGKFSL | S16; | 2013.9 | Yes |
| **AHNAK2** | NP_612429.2 | 113146 | protein AHNAK2 | **S842** | **80.55** | **82.31** | **39.91** | MPsFGVSAPGK | 16 | 1 | 1 | 0 | S3(Phospho) | S(3): 100.0; S(7): 0.0 | MPKFKMPsFGVSAPG | S3; | 1157.5 | Yes |
| **AHNAK2** | NP_612429.2 | 113146 | protein AHNAK2 | **S294** | | | **39.91** | DAHDVsPTSTDTEAQLTVER | 2 | 1 | 1 | 0 | S6(Phospho) | S(6): 100.0; T(8): 0.0; S(9): 0.0; T(10): 0.0; T(12): 0.0; T(17): 0.0 | PRDAHDVsPTSTDTE | S6; | 2252.0 | Yes |
| **AHNAK2** | NP_612429.2 | 113146 | protein AHNAK2 | **S842** | | | **39.91** | FKMPsFGVSAPGK | 12 | 1 | 1 | 1 | S5(Phospho) | S(5): 100.0; S(9): 0.0 | MPKFKMPsFGVSAPG | S5; | 1432.7 | Yes |
| **AHNAK2** | NP_612429.2 | 113146 | protein AHNAK2 | **S2327** | | | **39.91** | MLsFGVSALGK | 6 | 1 | 1 | 0 | S3(Phospho) | S(3): 100.0; S(7): 0.0 | MPKFKMLsFGVSALG | S3; | 1189.6 | No |
| **AHNAK2** | NP_612429.2 | 113146 | protein AHNAK2 | **S1172** | | | **39.91** | MPsFGASAPGK | 6 | 1 | 1 | 0 | S3(Phospho) | S(3): 100.0; S(7): 0.0 | MPKFKMPsFGASAPG | S3; | 1129.5 | Yes |
| **AHNAK2** | NP_612429.2 | 113146 | protein AHNAK2 | **S842** | | | **39.91** | FKMPsFGVSAPGK | 3 | 1 | 1 | 1 | M3(Oxidation); S5(Phospho) | S(5): 100.0; S(9): 0.0 | MPKFKMPsFGVSAPG | S5; | 1448.7 | Yes |
| **AHNAK2** | NP_612429.2 | 113146 | protein AHNAK2 | **T408** | | | **39.91** | LCEGtPQEGGLR | 4 | 1 | 1 | 0 | C2(Carbamidomethyl); T5(Phospho) | T(5): 100.0 | DPRLCEGtPQEGGLR | T5; | 1396.6 | Yes |
| **AHNAK2** | NP_612429.2 | 113146 | protein AHNAK2 | **S1997** | | | **39.91** | MPsFGVSAPGR | 8 | 1 | 1 | 0 | S3(Phospho) | S(3): 100.0; S(7): 0.0 | MPKFKMPsFGVSAPG | S3; | 1185.5 | Yes |
| **AHNAK2** | NP_612429.2 | 113146 | protein AHNAK2 | **S4472** | | | **39.91** | MPsFGMLSPGK | 4 | 1 | 1 | 0 | S3(Phospho) | S(3): 100.0; S(8): 0.0 | MPKFKMPsFGMLSPG | S3; | 1231.5 | Yes |
| **AHNAK2** | NP_612429.2 | 113146 | protein AHNAK2 | **T330** | | | **39.91** | FRtGSGQGPSSTGQPGR | 4 | 1 | 1 | 1 | T3(Phospho) | T(3): 98.9; S(5): 1.1; S(10): 0.0; S(11): 0.0; T(12): 0.0 | FLNLRFRtGSGQGPS | T3; | 1756.8 | Yes |
| **AHNAK2** | NP_612429.2 | 113146 | protein AHNAK2 | **S842** | | | **39.91** | MPsFGVSAPGK | 2 | 1 | 1 | 0 | S3(Phospho); K11(Label:13C(6)15N(2)) | S(3): 100.0; S(7): 0.0 | MPKFKMPsFGVSAPG | S3; | 1165.5 | Yes |
| **AHNAK2** | NP_612429.2 | 113146 | protein AHNAK2 | **S4710** | | | **39.91** | VsFSSTK | 1 | 1 | 1 | 0 | S2(Phospho) | S(2): 100.0; S(4): 0.0; S(5): 0.0; T(6): 0.0 | HFKVPKVsFSSTKTP | S2; | 835.4 | No |
| **AHNAK2** | NP_612429.2 | 113146 | protein AHNAK2 | **S1997** | | | **39.91** | FKMPsFGVSAPGR | 2 | 1 | 1 | 1 | S5(Phospho) | S(5): 100.0; S(9): 0.0 | MPKFKMPsFGVSAPG | S5; | 1460.7 | Yes |
| **AHNAK2** | NP_612429.2 | 113146 | protein AHNAK2 | **S509** | **14.01** | **50.54** | **39.91** | RLsTPQR | 4 | 1 | 1 | 1 | S3(Phospho) | S(3): 100.0; T(4): 0.0 | PERERRLsTPQRGKR | S3; | 937.5 | Yes |
| **AHNAK2** | NP_612429.2 | 113146 | protein AHNAK2 | **S1253** | | | **39.91** | VQMPsLK | 1 | 1 | 1 | 0 | S5(Phospho) | S(5): 100.0 | LPKVQMPsLKMPKVD | S5; | 882.4 | Yes |
| **AHNAK2** | NP_612429.2 | 113146 | protein AHNAK2 | **S2657** | | | **39.91** | MPsFR | 6 | 1 | 1 | 0 | S3(Phospho) | S(3): 100.0 | MPKFKMPsFRVSAPG | S3; | 717.3 | Yes |
| **AHNAK2** | NP_612429.2 | 113146 | protein AHNAK2 | **S5199** | **54.05** | | **39.91** | MPsLRR | 2 | 1 | 1 | 1 | S3(Phospho) | S(3): 100.0 | MPKFRMPsLRRSFRD | S3; | 839.4 | Yes |
| **AHNAK2** | NP_612429.2 | 113146 | protein AHNAK2 | **S4442; T4443** | | | **39.91** | VEVTSPNLDVSLPSMEVDIQAPGAKLDstR | 1 | 1 | 1 | 1 | S14(Phospho); S28(Phospho); R30(Label:13C(6)) | T(4): 0.0; S(5): 0.0; S(11): 0.1; S(14): 10.8; S(28): 94.5; T(29): 94.5 | APGAKLDsTRLEGDL;PGAKLDStRLEGDLS | S28; T29; | 3334.6 | No;No |
| **AHNAK2** | NP_612429.2 | 113146 | protein AHNAK2 | **S1172** | | | **39.91** | FKMPsFGASAPGK | 1 | 1 | 1 | 1 | S5(Phospho) | S(5): 100.0; S(9): 0.0 | MPKFKMPsFGASAPG | S5; | 1404.6 | Yes |
| **AHNAK2** | NP_612429.2 | 113146 | protein AHNAK2 | **S593** | | | **39.91** | FKIPSLGWsPSK | 4 | 1 | 1 | 1 | S9(Phospho) | S(5): 0.0; S(9): 99.1; S(11): 0.9 | KIPSLGWsPSKHTKT | S9; | 1426.7 | Yes |
| **AHNAK2** | NP_612429.2 | 113146 | protein AHNAK2 | **S3408** | | | **39.91** | VDLKsPQVDIKGPK | 2 | 1 | 1 | 2 | S5(Phospho) | S(5): 100.0 | MPKVDLKsPQVDIKG | S5; | 1603.8 | Yes |
| **AHNAK2** | NP_612429.2 | 113146 | protein AHNAK2 | **S4897** | | | **39.91** | FVFSVPQMAVPEGDLHAAVGAPVMSPLsPGER | 3 | 1 | 1 | 0 | S28(Phospho) | S(4): 0.0; S(25): 0.0; S(28): 100.0 | APVMSPLsPGERVQC | S28; | 3372.6 | Yes |
| **AHNAK2** | NP_612429.2 | 113146 | protein AHNAK2 | **S4472** | | | **39.91** | FKMPsFGMLSPGK | 2 | 1 | 1 | 1 | S5(Phospho) | S(5): 100.0; S(10): 0.0 | MPKFKMPsFGMLSPG | S5; | 1506.7 | Yes |
| **AHNAK2** | NP_612429.2 | 113146 | protein AHNAK2 | **S497** | | | **39.91** | FAFsTEKEPER | 2 | 1 | 1 | 1 | S4(Phospho) | S(4): 99.1; T(5): 0.9 | KTPKFAFsTEKEPER | S4; | 1420.6 | Yes |
| **AHNAK2** | NP_612429.2 | 113146 | protein AHNAK2 | **S4962** | | | **39.91** | IKLPsFR | 1 | 1 | 1 | 1 | S5(Phospho) | S(5): 100.0 | MPKIKLPsFRWSPKK | S5; | 940.5 | Yes |
| **AHNAK2** | NP_612429.2 | 113146 | protein AHNAK2 | **S497** | | | **39.91** | FAFsTEKEPERER | 1 | 1 | 1 | 2 | S4(Phospho) | S(4): 98.2; T(5): 1.8 | KTPKFAFsTEKEPER | S4; | 1705.8 | Yes |
| **AHNAK2** | NP_612429.2 | 113146 | protein AHNAK2 | **S1583** | | | **39.91** | VQMPsFK | 2 | 1 | 1 | 0 | S5(Phospho) | S(5): 100.0 | LPKVQMPsFKMPKVD | S5; | 916.4 | Yes |
| **AHSG** | NP_001613.2 | 197 | alpha-2-HS-glycoprotein preproprotein | **S138** | | | #NQ | CDSSPDsAEDVRK | 11 | 1 | 1 | 1 | C1(Carbamidomethyl); S7(Phospho) | S(3): 0.0; S(4): 0.0; S(7): 100.0 | KCDSSPDsAEDVRKV | S7; | 1545.6 | Yes |
| **AHSG** | NP_001613.2 | 197 | alpha-2-HS-glycoprotein preproprotein | **S138** | | | #NQ | CDSSPDsAEDVR | 5 | 1 | 1 | 0 | C1(Carbamidomethyl); S7(Phospho) | S(3): 0.0; S(4): 0.0; S(7): 100.0 | KCDSSPDsAEDVRKV | S7; | 1417.5 | Yes |
| **AIFM1** | NP_665811.1 | 9131 | apoptosis-inducing factor 1, mitochondrial isoform 2 precursor | **S264** | **2.8** | | 1.27 | SLsAIDR | 1 | 3 | 1 | 0 | S3(Phospho) | S(1): 0.0; S(3): 100.0 | GGTPRSLsAIDRAGA | S3; | 841.4 | No |
| **AIFM1** | NP_665811.1 | 9131 | apoptosis-inducing factor 1, mitochondrial isoform 2 precursor | **S264** | | **2.56** | 1.27 | SLsAIDRAGAEVK | 2 | 3 | 1 | 1 | S3(Phospho) | S(1): 0.0; S(3): 100.0 | GGTPRSLsAIDRAGA | S3; | 1396.7 | No |
| **AIMP2** | NP_006294.2 | 7965 | aminoacyl tRNA synthase complex-interacting multifunctional protein 2 | **T82** | | | 0.91 | MIQtPDADLDVTNIIQADEPTTLTTNALDLNSVLGK | 1 | 1 | 1 | 0 | T4(Phospho) | T(4): 99.6; T(12): 0.1; T(21): 0.1; T(22): 0.1; T(24): 0.0; T(25): 0.0; S(32): 0.0 | GLSKMIQtPDADLDV | T4; | 3920.9 | Yes |
| **AJUBA** | NP_116265.1 | 84962 | LIM domain-containing protein ajuba isoform 1 | **S263** | **0.3** | **0.27** | **0.32** | GAQPGRHsVTGYGDCAVGAR | 2 | 2 | 1 | 1 | R6(Label:13C(6)); S8(Phospho); C15(Carbamidomethyl); R20(Label:13C(6)) | S(8): 100.0; T(10): 0.0; Y(12): 0.0 | GAQPGRHsVTGYGDC | S8; | 2108.0 | Yes |
| **AJUBA** | NP_116265.1 | 84962 | LIM domain-containing protein ajuba isoform 1 | **S119** | | **0.44** | **0.32** | LEPTAPALsPR | 2 | 2 | 1 | 0 | S9(Phospho) | T(4): 0.0; S(9): 100.0 | EPTAPALsPRSSFAS | S9; | 1231.6 | Yes |
| **AJUBA** | NP_116265.1 | 84962 | LIM domain-containing protein ajuba isoform 1 | **S230** | | **0.22** | **0.32** | HsYPPALGSPGALAGAGVGAAGPLER | 2 | 2 | 1 | 0 | S2(Phospho) | S(2): 100.0; Y(3): 0.0; S(9): 0.0 | GCQESRHsYPPALGS | S2; | 2453.2 | Yes |
| **AJUBA** | NP_116265.1 | 84962 | LIM domain-containing protein ajuba isoform 1 | **S230** | | **0.35** | **0.32** | HsYPPALGSPGALAGAGVGAAGPLER | 3 | 2 | 1 | 0 | S2(Phospho); R26(Label:13C(6)) | S(2): 100.0; Y(3): 0.0; S(9): 0.0 | GCQESRHsYPPALGS | S2; | 2459.2 | Yes |
| **AJUBA** | NP_116265.1 | 84962 | LIM domain-containing protein ajuba isoform 1 | **T114** | | 0.62 | **0.32** | ALPLPQSLPPDFRLEPtAPALSPR | 2 | 2 | 1 | 1 | S22(Phospho) | S(7): 0.0; T(17): 97.7; S(22): 2.3 | PDFRLEPtAPALSPR | T17; | 2663.4 | No |
| **AJUBA** | NP_116265.1 | 84962 | LIM domain-containing protein ajuba isoform 1 | **T114** | | | **0.32** | ALPLPQSLPPDFRLEPtAPALSPR | 1 | 2 | 1 | 1 | R13(Label:13C(6)); S22(Phospho); R24(Label:13C(6)) | S(7): 0.0; T(17): 98.2; S(22): 1.8 | PDFRLEPtAPALSPR | T17; | 2675.4 | No |
| **AJUBA** | NP_116265.1 | 84962 | LIM domain-containing protein ajuba isoform 1 | **S119** | | **0.46** | **0.32** | LEPTAPALsPR | 3 | 2 | 1 | 0 | S9(Phospho); R11(Label:13C(6)) | T(4): 0.0; S(9): 100.0 | EPTAPALsPRSSFAS | S9; | 1237.6 | Yes |
| **AK5** | NP_036225.2 | 26289 | adenylate kinase isoenzyme 5 isoform 2 | **T245** | | | #NQ | AEQQGRPDDNVKAtQR | 1 | 2 | 1 | 1 | K12(Label:13C(6)15N(2)); T14(Phospho) | T(14): 100.0 | PDDNVKAtQRRLMNF | T14; | 1900.9 | No |
| **AKAP1** | NP_001229832.1 | 8165 | A-kinase anchor protein 1, mitochondrial precursor | **T533** | 1.92 | | 0.74 | AItPPLPESTVPFSNGVLK | 3 | 1 | 1 | 0 | T3(Phospho); K19(Label:13C(6)15N(2)) | T(3): 100.0; S(9): 0.0; T(10): 0.0; S(14): 0.0 | SPRDKAItPPLPEST | T3; | 2055.1 | Yes |
| **AKAP1** | NP_001229832.1 | 8165 | A-kinase anchor protein 1, mitochondrial precursor | **T533** | 1.51 | | 0.74 | AItPPLPESTVPFSNGVLK | 5 | 1 | 1 | 0 | T3(Phospho) | T(3): 100.0; S(9): 0.0; T(10): 0.0; S(14): 0.0 | SPRDKAItPPLPEST | T3; | 2047.1 | Yes |
| **AKAP1** | NP_001229832.1 | 8165 | A-kinase anchor protein 1, mitochondrial precursor | **T533** | 0.84 | | 0.74 | DKAItPPLPESTVPFSNGVLK | 3 | 1 | 1 | 1 | T5(Phospho) | T(5): 100.0; S(11): 0.0; T(12): 0.0; S(16): 0.0 | SPRDKAItPPLPEST | T5; | 2290.2 | Yes |
| **AKAP1** | NP_001229832.1 | 8165 | A-kinase anchor protein 1, mitochondrial precursor | **T533** | | 0.98 | 0.74 | DKAItPPLPESTVPFSNGVLK | 4 | 1 | 1 | 1 | K2(Label:13C(6)15N(2)); T5(Phospho); K21(Label:13C(6)15N(2)) | T(5): 100.0; S(11): 0.0; T(12): 0.0; S(16): 0.0 | SPRDKAItPPLPEST | T5; | 2306.2 | Yes |
| **AKAP10** | NP_009133.2 | 11216 | A-kinase anchor protein 10, mitochondrial precursor | **S96** | 1.43 | | #N/A | KQPsHMEAAHFGDLGR | 3 | 1 | 1 | 1 | S4(Phospho) | S(4): 100.0 | ATLKKQPsHMEAAHF | S4; | 1860.8 | Yes |
| **AKAP10** | NP_009133.2 | 11216 | A-kinase anchor protein 10, mitochondrial precursor | **S281** | | **2.39** | #N/A | NSPAsPLKELSGK | 2 | 1 | 1 | 1 | S5(Phospho) | S(2): 0.0; S(5): 100.0; S(11): 0.0 | ASRNSPAsPLKELSG | S5; | 1407.7 | Yes |
| **AKAP11** | NP_057332.1 | 11215 | A-kinase anchor protein 11 | **S448** | **2.73** | **2.24** | 1.44 | ASREDSGLFsPIR | 2 | 1 | 1 | 1 | S10(Phospho) | S(2): 0.0; S(6): 0.0; S(10): 100.0 | REDSGLFsPIRSSAF | S10; | 1514.7 | Yes |
| **AKAP11** | NP_057332.1 | 11215 | A-kinase anchor protein 11 | **S1242** | **2.16** | 1.46 | 1.44 | SVsPTFLNPSDENLK | 7 | 1 | 1 | 0 | S3(Phospho) | S(1): 0.0; S(3): 100.0; T(5): 0.0; S(10): 0.0 | VQSQRSVsPTFLNPS | S3; | 1727.8 | Yes |
| **AKAP11** | NP_057332.1 | 11215 | A-kinase anchor protein 11 | **S448** | | **2.38** | 1.44 | EDSGLFsPIR | 2 | 1 | 1 | 0 | S7(Phospho) | S(3): 0.0; S(7): 100.0 | REDSGLFsPIRSSAF | S7; | 1200.5 | Yes |
| **AKAP11** | NP_057332.1 | 11215 | A-kinase anchor protein 11 | **T1100** | | **2.42** | 1.44 | NVIPDtPPSTPLVPSR | 2 | 1 | 1 | 0 | T6(Phospho) | T(6): 100.0; S(9): 0.0; T(10): 0.0; S(15): 0.0 | DRNVIPDtPPSTPLV | T6; | 1769.9 | Yes |
| **AKAP12** | NP_005091.2 | 9590 | A-kinase anchor protein 12 isoform 1 | **S598** | **2.61** | **2.73** | **3.04** | GLAEVQQDGEAEEGATsDGEKKR | 8 | 2 | 1 | 2 | S17(Phospho) | T(16): 1.3; S(17): 98.7 | EAEEGATsDGEKKRE | S17; | 2484.1 | Yes |
| **AKAP12** | NP_005091.2 | 9590 | A-kinase anchor protein 12 isoform 1 | **S1331** | **2.84** | **2.55** | **3.04** | SPPsPVER | 5 | 2 | 1 | 0 | S4(Phospho) | S(1): 0.0; S(4): 100.0 | SHAKSPPsPVEREMV | S4; | 948.4 | Yes |
| **AKAP12** | NP_005091.2 | 9590 | A-kinase anchor protein 12 isoform 1 | **S514** | 1.87 | 1.82 | **3.04** | VQGsPLKK | 3 | 2 | 1 | 1 | S4(Phospho) | S(4): 100.0 | ERMKVQGsPLKKLFT | S4; | 936.5 | Yes |
| **AKAP12** | NP_005091.2 | 9590 | A-kinase anchor protein 12 isoform 1 | **T597** | | | **3.04** | GLAEVQQDGEAEEGAtSDGEKKR | 2 | 2 | 1 | 2 | S17(Phospho); K21(Label:13C(6)15N(2)); K22(Label:13C(6)15N(2)); R23(Label:13C(6)) | T(16): 100.0; S(17): 0.0 | GEAEEGAtSDGEKKR | T16; | 2506.1 | Yes |
| **AKAP12** | NP_005091.2 | 9590 | A-kinase anchor protein 12 isoform 1 | **S627; S629** | **2.48** | | **3.04** | RPsEsDKEDELDKVK | 1 | 2 | 1 | 2 | S3(Phospho); S5(Phospho) | S(3): 100.0; S(5): 100.0 | KKRVRRPsESDKEDE;RVRRPSEsDKEDELD | S3; S5; | 1934.8 | Yes;Yes |

| GeneSymbol | NP_Accession | Gene ID | ProteinDescription | PhosphoSite (Protein) | SCC-R / SCC-S R1 | SCC-R / SCC-S R2 | SCC-R / SCC-S Total Proteome | Sequence | # PSMs | # Proteins | # Protein Groups | # Missed Cleavages | Modifications | phosphoRS Site Probabilities | Phosphowindow | PhosphoSite (Peptide) | MH+ [Da] | PhosphositePlusEvidence |
|---|---|---|---|---|---|---|---|---|---|---|---|---|---|---|---|---|---|---|
| **AKAP12** | NP_005091.2 | 9590 | A-kinase anchor protein 12 isoform 1 | **S612** | 2.82 | 3.44 | 3.04 | EGVTPWAsFK | 3 | 2 | 1 | 0 | S8(Phospho) | T(4): 0.0; S(8): 100.0 | EGVTPWAsFKKMVTP | S8; | 1201.5 | Yes |
| **AKAP12** | NP_005091.2 | 9590 | A-kinase anchor protein 12 isoform 1 | **S612** | | 2.53 | 3.04 | EGVTPWAsFKK | 4 | 2 | 1 | 1 | S8(Phospho) | T(4): 0.0; S(8): 100.0 | EGVTPWAsFKKMVTP | S8; | 1329.6 | Yes |
| **AKAP12** | NP_005091.2 | 9590 | A-kinase anchor protein 12 isoform 1 | **S627** | | 2.41 | 3.04 | RPsESDKEDELDKVK | 3 | 2 | 1 | 2 | S3(Phospho) | S(3): 98.4; S(5): 1.6 | KKRVRRPsESDKEDE | S3; | 1854.8 | Yes |
| **AKAP12** | NP_005091.2 | 9590 | A-kinase anchor protein 12 isoform 1 | **S505** | | | 3.04 | VLSKPPEGVVSEVEMLSsQER | 2 | 2 | 1 | 0 | S18(Phospho) | S(3): 0.0; S(11): 0.0; S(17): 0.0; S(18): 100.0 | SEVEMLSsQERMKVQ | S18; | 2380.1 | Yes |
| **AKAP13** | NP_009131.2 | 11214 | A-kinase anchor protein 13 isoform 2 | **S983** | 0.36 | | 0.37 | ALQLSNsPGASSAFLK | 2 | 2 | 1 | 0 | S7(Phospho); K16(Label:13C(6)15N(2)) | S(5): 0.0; S(7): 100.0; S(11): 0.0; S(12): 0.0 | KALQLSNsPGASSAF | S7; | 1678.8 | Yes |
| **AKAP13** | NP_009131.2 | 11214 | A-kinase anchor protein 13 isoform 2 | **S1559** | 0.34 | 0.36 | 0.37 | SLsPFR | 2 | 3 | 1 | 0 | S3(Phospho) | S(1): 0.0; S(3): 100.0 | RSSMRSLsPFRRHSW | S3; | 786.4 | Yes |
| **AKAP13** | NP_009131.2 | 11214 | A-kinase anchor protein 13 isoform 2 | **S1565** | 0.42 | 0.45 | 0.37 | HsWGPGK | 4 | 3 | 1 | 0 | S2(Phospho); K7(Label:13C(6)15N(2)) | S(2): 100.0 | LSPFRRHsWGPGKNA | S2; | 856.4 | Yes |
| **AKAP13** | NP_009131.2 | 11214 | A-kinase anchor protein 13 isoform 2 | **S1929; S1932** | 0.29 | | 0.37 | FLSHsTDsLNKISK | 1 | 3 | 1 | 1 | S5(Phospho); S8(Phospho); K11(Label:13C(6)15N(2)); K14(Label:13C(6)15N(2)) | S(3): 0.0; S(5): 98.0; T(6): 4.0; S(8): 98.0; S(13): 0.0 | TWKFLSHsTDSLNKI;FLSHSTDsLNKISKV | S5; S8; | 1752.8 | Yes;Yes |
| **AKAP13** | NP_009131.2 | 11214 | A-kinase anchor protein 13 isoform 2 | **S1565** | | | 0.37 | HsWGPGK | 4 | 3 | 1 | 0 | S2(Phospho) | S(2): 100.0 | LSPFRRHsWGPGKNA | S2; | 848.3 | Yes |
| **AKAP13** | NP_009131.2 | 11214 | A-kinase anchor protein 13 isoform 2 | **S1876** | | 0.67 | 0.37 | sAVLLVDETATTPIFANRR | 2 | 3 | 1 | 1 | S1(Phospho) | S(1): 100.0; T(9): 0.0; T(11): 0.0; T(12): 0.0 | QPKERPRsAVLLVDE | S1; | 2154.1 | Yes |
| **AKAP13** | NP_009131.2 | 11214 | A-kinase anchor protein 13 isoform 2 | **S1929; S1932** | | 0.4 | 0.37 | FLSHsTDsLNK | 2 | 3 | 1 | 0 | S5(Phospho); S8(Phospho) | S(3): 0.0; S(5): 100.0; T(6): 0.0; S(8): 100.0 | TWKFLSHsTDSLNKI;FLSHSTDsLNKISKV | S5; S8; | 1408.6 | Yes;Yes |
| **AKAP13** | NP_009131.2 | 11214 | A-kinase anchor protein 13 isoform 2 | **S1876** | | | 0.37 | sAVLLVDETATTPIFANR | 1 | 3 | 1 | 0 | S1(Phospho); R18(Label:13C(6)) | S(1): 100.0; T(9): 0.0; T(11): 0.0; T(12): 0.0 | QPKERPRsAVLLVDE | S1; | 2004.0 | Yes |
| **AKAP13** | NP_009131.2 | 11214 | A-kinase anchor protein 13 isoform 2 | **S2709** | | 2.15 | 0.37 | IPSFFPSPEEPPsPSAPSIAK | 1 | 3 | 1 | 0 | S13(Phospho) | S(3): 0.0; S(7): 0.0; S(13): 100.0; S(15): 0.0; S(18): 0.0 | PSPEEPPsPSAPSIA | S13; | 2262.1 | Yes |
| **AKAP13** | NP_009131.2 | 11214 | A-kinase anchor protein 13 isoform 2 | **S1929** | | 0.58 | 0.37 | FLSHsTDSLNKISK | 1 | 3 | 1 | 1 | S5(Phospho); K11(Label:13C(6)15N(2)); K14(Label:13C(6)15N(2)) | S(3): 1.8; S(5): 96.5; T(6): 1.8; S(8): 0.0; S(13): 0.0 | TWKFLSHsTDSLNKI | S5; | 1672.8 | Yes |
| **AKAP13** | NP_009131.2 | 11214 | A-kinase anchor protein 13 isoform 2 | **S1929** | | 0.41 | 0.37 | FLSHsTDSLNK | 1 | 3 | 1 | 0 | S5(Phospho); S8(Phospho); K11(Label:13C(6)15N(2)) | S(3): 0.0; S(5): 100.0; T(6): 50.0; S(8): 50.0 | TWKFLSHsTDSLNKI | S5; | 1416.6 | Yes |
| **AKAP13** | NP_009131.2 | 11214 | A-kinase anchor protein 13 isoform 2 | **S1876** | | 0.62 | 0.37 | sAVLLVDETATTPIFANRR | 1 | 3 | 1 | 1 | S1(Phospho); R18(Label:13C(6)); R19(Label:13C(6)) | S(1): 100.0; T(9): 0.0; T(11): 0.0; T(12): 0.0 | QPKERPRsAVLLVDE | S1; | 2166.1 | Yes |
| **AKAP17A** | NP_005079.2 | 8227 | A-kinase anchor protein 17A isoform 1 | **S537** | 0.95 | 1.08 | #NQ | VVPEDGsPEKR | 7 | 1 | 1 | 1 | S7(Phospho) | S(7): 100.0 | RVVPEDGsPEKRCPG | S7; | 1292.6 | Yes |
| **AKAP8** | NP_005849.1 | 10270 | A-kinase anchor protein 8 | **S339** | | 0.86 | 0.92 | sGDEEFKGEDELCDSGR | 4 | 1 | 1 | 1 | S1(Phospho); C13(Carbamidomethyl) | S(1): 100.0; S(15): 0.0 | DAAGDFRsGDEEFKG | S1; | 2009.7 | Yes |
| **AKAP8** | NP_005849.1 | 10270 | A-kinase anchor protein 8 | **S323; S328** | | 0.86 | 0.92 | LARVDsEGDFsENDDAAGDFR | 2 | 1 | 1 | 1 | S6(Phospho); S11(Phospho) | S(6): 100.0; S(11): 100.0 | TKLARVDsEGDFSEN;VDSEGDFsENDDAAG | S6; S11; | 2445.9 | Yes;Yes |
| **AKAP8** | NP_005849.1 | 10270 | A-kinase anchor protein 8 | **S323; S328** | | | 0.92 | LARVDsEGDFsENDDAAGDFR | 1 | 1 | 1 | 1 | R3(Label:13C(6)); S6(Phospho); S11(Phospho); R21(Label:13C(6)) | S(6): 100.0; S(11): 100.0 | TKLARVDsEGDFSEN;VDSEGDFsENDDAAG | S6; S11; | 2458.0 | Yes;Yes |
| **AKAP8** | NP_005849.1 | 10270 | A-kinase anchor protein 8 | **S339** | | | 0.92 | sGDEEFKGEDELCDSGR | 1 | 1 | 1 | 1 | S1(Phospho); K7(Label:13C(6)15N(2)); C13(Carbamidomethyl); R17(Label:13C(6)) | S(1): 100.0; S(15): 0.0 | DAAGDFRsGDEEFKG | S1; | 2023.8 | Yes |
| **AKIRIN1** | NP_001129747.1 | 79647 | akirin-1 isoform 2 | **S22** | | 0.97 | #NQ | RPMEFEAALLSPGsPK | 2 | 2 | 1 | 0 | S14(Phospho) | S(11): 0.0; S(14): 100.0 | AALLSPGsPKRRRCA | S14; | 1809.9 | Yes |
| **AKIRIN1** | NP_001129747.1 | 79647 | akirin-1 isoform 2 | **S22** | | 1 | #NQ | RPMEFEAALLSPGsPK | 1 | 2 | 1 | 0 | R1(Label:13C(6)); S14(Phospho); K16(Label:13C(6)15N(2)) | S(11): 0.0; S(14): 100.0 | AALLSPGsPKRRRCA | S14; | 1823.9 | Yes |
| **AKT1** | NP_001014431.1 | 207 | RAC-alpha serine/threonine-protein kinase | **S122; S124** | | | 1.20 | sGsPSDNSGAEEMEVSLAKPK | 2 | 1 | 1 | 0 | S1(Phospho); S8(Phospho) | S(1): 97.3; S(3): 99.9; S(5): 2.7; S(8): 0.1; S(16): 0.0 | EEEMDFRsGSPSDNS;EMDFRSGsPSDNSGA | S1; S3; | 2279.9 | Yes;Yes |
| **AKT1S1** | NP_001265089.1 | 84335 | proline-rich AKT1 substrate 1 isoform b | **T246** | 1.1 | 0.96 | 2.00 | LNtSDFQKLK | 4 | 2 | 1 | 1 | T3(Phospho) | T(3): 100.0; S(4): 0.0 | LPRPRLNtSDFQKLK | T3; | 1273.6 | Yes |
| **AKT1S1** | NP_001265089.1 | 84335 | proline-rich AKT1 substrate 1 isoform b | **S88; S92** | 16.95 | 5.7 | 2.00 | AATAARPPAPPPAPQPPsPTPsPPRPTLAR | 3 | 2 | 1 | 0 | S18(Phospho); S22(Phospho) | T(3): 0.0; S(18): 99.9; T(20): 0.1; S(22): 100.0; T(27): 0.0 | PPAPQPPsPTPSPPR;QPPSPTPsPPRPTLA | S18; S22; | 3124.6 | Yes;Yes |
| **AKT1S1** | NP_001265089.1 | 84335 | proline-rich AKT1 substrate 1 isoform b | **S183** | | 3.17 | 2.00 | sLPVSVPVWGFK | 2 | 2 | 1 | 0 | S1(Phospho) | S(1): 100.0; S(5): 0.0 | PTQQYAKsLPVSVPV | S1; | 1395.7 | Yes |
| **AKT1S1** | NP_001265089.1 | 84335 | proline-rich AKT1 substrate 1 isoform b | **S183** | | | 2.00 | sLPVSVPVWGFKEK | 2 | 2 | 1 | 1 | S1(Phospho) | S(1): 100.0; S(5): 0.0 | PTQQYAKsLPVSVPV | S1; | 1652.8 | Yes |
| **AKT1S1** | NP_001265089.1 | 84335 | proline-rich AKT1 substrate 1 isoform b | **T246** | | | 2.00 | LNtSDFQK | 2 | 2 | 1 | 0 | T3(Phospho); K8(Label:13C(6)15N(2)) | T(3): 99.1; S(4): 0.9 | LPRPRLNtSDFQKLK | T3; | 1040.5 | Yes |
| **ALDOA** | NP_908932.1 | 226 | fructose-bisphosphate aldolase A isoform 1 | **S36** | 2.12 | 2 | 1.19 | GILAADEsTGSIAKR | 11 | 2 | 1 | 1 | S8(Phospho) | S(8): 99.0; T(9): 1.0; S(11): 0.0 | GILAADEsTGSIAKR | S8; | 1568.8 | Yes |
| **ALDOA** | NP_908932.1 | 226 | fructose-bisphosphate aldolase A isoform 1 | **S39** | 2.22 | | 1.19 | GILAADESTGsIAKR | 3 | 2 | 1 | 1 | S11(Phospho); K14(Label:13C(6)15N(2)); R15(Label:13C(6)) | S(8): 0.0; T(9): 0.0; S(11): 100.0 | AADESTGsIAKRLQS | S11; | 1582.8 | Yes |
| **ALDOA** | NP_908932.1 | 226 | fructose-bisphosphate aldolase A isoform 1 | **S39** | | 1.79 | 1.19 | GILAADESTGsIAK | 4 | 2 | 1 | 0 | S11(Phospho) | S(8): 0.0; T(9): 0.0; S(11): 100.0 | AADESTGsIAKRLQS | S11; | 1412.7 | Yes |
| **ALG3** | NP_005778.1 | 10195 | dol-P-Man:Man(5)GlcNAc(2)-PP-Dol alpha-1,3-mannosyltransferase isoform a | **S13** | | 0.16 | #N/A | SGsAAQAEGLCK | 4 | 1 | 1 | 0 | S3(Phospho); C11(Carbamidomethyl); K12(Label:13C(6)15N(2)) | S(1): 0.0; S(3): 100.0 | RKRGRSGsAAQAEGL | S3; | 1266.5 | Yes |
| **ALKBH5** | NP_060228.3 | 54890 | RNA demethylase ALKBH5 | **Y51; S54** | | | 0.58 | EAAAAAAAAVAAAAAAAAAAEPyPVsGAKR | 1 | 1 | 1 | 1 | Y23(Phospho); S26(Phospho); K29(Label:13C(6)15N(2)) | Y(23): 100.0; S(26): 100.0 | AAAAAEPyPVSGAKR;AAEPYPVsGAKRKYQ | Y23; S26; | 2778.3 | Yes;Yes |
| **ALS2** | NP_065970.2 | 57679 | alsin isoform 1 | **S492** | 0.8 | 2.2 | #N/A | LSLPGLLSQVsPR | 4 | 1 | 1 | 0 | S11(Phospho) | S(2): 0.0; S(8): 0.0; S(11): 100.0 | PGLLSQVsPRLLRKA | S11; | 1446.8 | Yes |
| **ALS2** | NP_065970.2 | 57679 | alsin isoform 1 | **S492** | | | #N/A | LSLPGLLSQVsPR | 2 | 1 | 1 | 0 | S11(Phospho); R13(Label:13C(6)) | S(2): 0.0; S(8): 0.0; S(11): 100.0 | PGLLSQVsPRLLRKA | S11; | 1452.8 | Yes |
| **ALS2** | NP_065970.2 | 57679 | alsin isoform 1 | **S1464** | | 0.93 | #N/A | SEsPEPGYVVTSSGLLLPVLLPR | 2 | 1 | 1 | 0 | S3(Phospho) | S(1): 1.3; S(3): 98.7; Y(8): 0.0; T(11): 0.0; S(12): 0.0; S(13): 0.0 | KSDSRSEsPEPGYVV | S3; | 2490.3 | Yes |
| **ALS2** | NP_065970.2 | 57679 | alsin isoform 1 | **S483; S492** | | | #N/A | RLsLPGLLSQVsPR | 2 | 1 | 1 | 1 | S3(Phospho); S12(Phospho) | S(3): 100.0; S(9): 0.0; S(12): 100.0 | EGGSRRLsLPGLLSQ;PGLLSQVsPRLLRKA | S3; S12; | 1682.8 | Yes;Yes |
| **AMMECR1L** | NP_113633.2 | 83607 | AMMECR1-like protein | **S74** | | 1.55 | #N/A | ENVSDLTLGPGNsPITR | 2 | 1 | 1 | 0 | S13(Phospho) | S(4): 0.0; T(7): 0.0; S(13): 98.4; T(16): 1.6 | LTLGPGNsPITRMNP | S13; | 1849.9 | Yes |
| **AMMECR1L** | NP_113633.2 | 83607 | AMMECR1-like protein | **S74** | | | #N/A | ENVSDLTLGPGNsPITR | 1 | 1 | 1 | 0 | S13(Phospho); R17(Label:13C(6)) | S(4): 0.0; T(7): 0.0; S(13): 100.0; T(16): 0.0 | LTLGPGNsPITRMNP | S13; | 1855.9 | Yes |
| **AMOTL1** | NP_570899.1 | 154810 | angiomotin-like protein 1 | **S828** | | | #N/A | GsIGLLLGK | 1 | 1 | 1 | 0 | S2(Phospho); K9(Label:13C(6)15N(2)) | S(2): 100.0 | EEKTWKGsIGLLLGK | S2; | 945.5 | Yes |
| **AMOTL1** | NP_570899.1 | 154810 | angiomotin-like protein 1 | **S793** | | 0.29 | #N/A | sVPSIAAATGTHSR | 2 | 1 | 1 | 0 | S1(Phospho); R14(Label:13C(6)) | S(1): 100.0; S(4): 0.0; T(9): 0.0; T(11): 0.0; S(13): 0.0 | SSLRPARsVPSIAAA | S1; | 1440.7 | Yes |
| **AMOTL2** | NP_057285.3 | 51421 | angiomotin-like protein 2 isoform 2 | **T45** | 3.49 | | 1.70 | GGAGtGGTGSPQASLEILAPEDSQVLQQATR | 2 | 3 | 1 | 0 | T5(Phospho) | T(5): 100.0; T(8): 0.0; S(10): 0.0; S(14): 0.0; S(23): 0.0; T(30): 0.0 | ALRGGAGtGGTGSPQ | T5; | 3076.5 | Yes |
| **AMOTL2** | NP_057285.3 | 51421 | angiomotin-like protein 2 isoform 2 | **S760** | 1.99 | | 1.70 | AAsLDSVATSR | 4 | 3 | 1 | 0 | S3(Phospho) | S(3): 100.0; S(6): 0.0; T(9): 0.0; S(10): 0.0 | SSSQRAAsLDSVATS | S3; | 1157.5 | No |
| **AMOTL2** | NP_057285.3 | 51421 | angiomotin-like protein 2 isoform 2 | **S236** | 2.5 | 2.4 | 1.70 | ARGsPHFQHAEVR | 2 | 3 | 1 | 1 | S4(Phospho) | S(4): 100.0 | PRYRARGsPHFQHAE | S4; | 1571.7 | Yes |
| **AMOTL2** | NP_057285.3 | 51421 | angiomotin-like protein 2 isoform 2 | **S667** | | | 1.70 | sVPSVFAAAAAGTQGWQGLSSSER | 3 | 3 | 1 | 0 | S1(Phospho) | S(1): 99.9; S(4): 0.1; T(13): 0.0; S(20): 0.0; S(21): 0.0; S(22): 0.0 | GSLRPAKsVPSVFAA | S1; | 2444.1 | No |
| **AMOTL2** | NP_057285.3 | 51421 | angiomotin-like protein 2 isoform 2 | **S760** | | 2.82 | 1.70 | AAsLDSVATSR | 2 | 3 | 1 | 0 | S3(Phospho); R11(Label:13C(6)) | S(3): 100.0; S(6): 0.0; T(9): 0.0; S(10): 0.0 | SSSQRAAsLDSVATS | S3; | 1163.5 | No |
| **AMOTL2** | NP_057285.3 | 51421 | angiomotin-like protein 2 isoform 2 | **S540** | | 3.58 | 1.70 | QAGAPGGSSGSGGsPELSALR | 4 | 2 | 1 | 0 | S14(Phospho) | S(8): 0.0; S(9): 0.0; S(11): 0.0; S(14): 100.0; S(18): 0.0 | GSSGSGGsPELSALR | S14; | 1922.9 | Yes |
| **AMOTL2** | NP_057285.3 | 51421 | angiomotin-like protein 2 isoform 2 | **S183** | | | 1.70 | APSHMSSSHsFPQLAR | 2 | 3 | 1 | 0 | S10(Phospho) | S(3): 0.0; S(6): 0.0; S(7): 0.0; S(8): 0.0; S(10): 100.0 | SHMSSSHsFPQLARN | S10; | 1819.8 | Yes |
| **AMPD2** | NP_004028.3 | 271 | AMP deaminase 2 isoform 1 | **S76** | | | 1.86 | RAsLQASTAAPEAR | 2 | 1 | 1 | 1 | S3(Phospho) | S(3): 100.0; S(7): 0.0; T(8): 0.0 | YPFKKRAsLQASTAA | S3; | 1508.7 | Yes |
| **AMPD2** | NP_004028.3 | 271 | AMP deaminase 2 isoform 1 | **S168** | 1.18 | 1.39 | 1.86 | QIsQDVKLEPDILLR | 3 | 4 | 1 | 1 | S3(Phospho) | S(3): 100.0 | QRLERQIsQDVKLEP | S3; | 1847.0 | Yes |
| **AMPD2** | NP_004028.3 | 271 | AMP deaminase 2 isoform 1 | **S168** | | | 1.86 | QIsQDVK | 1 | 4 | 1 | 0 | S3(Phospho); K7(Label:13C(6)15N(2)) | S(3): 100.0 | QRLERQIsQDVKLEP | S3; | 905.4 | Yes |
| **AMPD2** | NP_004028.3 | 271 | AMP deaminase 2 isoform 1 | **S168** | 1.24 | | 1.86 | QIsQDVKLEPDILLR | 2 | 4 | 1 | 1 | S3(Phospho); K7(Label:13C(6)15N(2)); R15(Label:13C(6)) | S(3): 100.0 | QRLERQIsQDVKLEP | S3; | 1861.0 | Yes |
| **AMPD2** | NP_004028.3 | 271 | AMP deaminase 2 isoform 1 | **S100** | | 1.75 | 1.86 | sLPGPAPCLK | 2 | 2 | 1 | 0 | S1(Phospho); C8(Carbamidomethyl) | S(1): 100.0 | PPLQSARsLPGPAPC | S1; | 1119.5 | Yes |
| **ANAPC1** | NP_073153.1 | 64682 | anaphase-promoting complex subunit 1 | **S688** | 1.62 | 1.59 | 0.90 | NFDFEGSLsPVIAPK | 4 | 3 | 1 | 0 | S9(Phospho) | S(7): 0.0; S(9): 100.0 | FDFEGSLsPVIAPKK | S9; | 1700.8 | Yes |
| **ANAPC1** | NP_073153.1 | 64682 | anaphase-promoting complex subunit 1 | **S688** | 1.7 | | 0.90 | NFDFEGSLsPVIAPK | 4 | 3 | 1 | 0 | S9(Phospho); K15(Label:13C(6)15N(2)) | S(7): 0.0; S(9): 100.0 | FDFEGSLsPVIAPKK | S9; | 1708.8 | Yes |
| **ANAPC1** | NP_073153.1 | 64682 | anaphase-promoting complex subunit 1 | **S555** | 2.64 | 1.07 | 0.90 | LLGSLDEVVLLsPVPELR | 2 | 1 | 1 | 0 | S12(Phospho) | S(4): 0.0; S(12): 100.0 | LDEVVLLsPVPELRD | S12; | 2029.1 | Yes |
| **ANAPC1** | NP_073153.1 | 64682 | anaphase-promoting complex subunit 1 | **S355** | 0.75 | 0.64 | 0.90 | AHsPALGVHSFSGVQR | 2 | 1 | 1 | 0 | S3(Phospho); R16(Label:13C(6)) | S(3): 100.0; S(10): 0.0; S(12): 0.0 | AALSRAHsPALGVHS | S3; | 1735.8 | Yes |
| **ANAPC1** | NP_073153.1 | 64682 | anaphase-promoting complex subunit 1 | **S688** | | 1.87 | 0.90 | NFDFEGSLsPVIAPKK | 3 | 3 | 1 | 1 | S9(Phospho) | S(7): 0.0; S(9): 100.0 | FDFEGSLsPVIAPKK | S9; | 1828.9 | Yes |
| **ANAPC1** | NP_073153.1 | 64682 | anaphase-promoting complex subunit 1 | **S688** | | | 0.90 | NFDFEGSLsPVIAPKK | 1 | 3 | 1 | 1 | S9(Phospho); K15(Label:13C(6)15N(2)); K16(Label:13C(6)15N(2)) | S(7): 0.0; S(9): 100.0 | FDFEGSLsPVIAPKK | S9; | 1844.9 | Yes |

| GeneSymbol | NP_Accession | Gene ID | ProteinDescription | PhosphoSite (Protein) | SCC-R / SCC-S R1 | SCC-R / SCC-S R2 | SCC-R / SCC-S Total Proteome | Sequence | # PSMs | # Proteins | # Protein Groups | # Missed Cleavages | Modifications | phosphoRS Site Probabilities | Phosphowindow | PhosphoSite (Peptide) | MH+ [Da] | PhosphositePlusEvidence |
|---|---|---|---|---|---|---|---|---|---|---|---|---|---|---|---|---|---|---|
| **ANAPC2** | NP_037498.1 | 29882 | anaphase-promoting complex subunit 2 | **S534** | | 0.73 | #N/A | LLHQFSFsPER | 1 | 1 | 1 | 0 | S6(Phospho); R11(Label:13C(6)) | S(6): 0.9; S(8): 99.1 | LLHQFSFsPEREIRN | S8; | 1446.7 | Yes |
| **ANKHD1-EIF4EBP3** | NP_065741.3 | 404734 | ANKHD1-EIF4EBP3 protein | **S1679** | 1.06 | | #N/A | LNLTsPK | 3 | 2 | 1 | 0 | S5(Phospho); K7(Label:13C(6)15N(2)) | T(4): 0.0; S(5): 100.0 | NIKLNLTsPKRGQKR | S5; | 860.4 | Yes |
| **ANKHD1-EIF4EBP3** | NP_065741.3 | 404734 | ANKHD1-EIF4EBP3 protein | **S1679** | | 1.13 | #N/A | LNLTsPK | 6 | 2 | 1 | 0 | S5(Phospho) | T(4): 0.0; S(5): 100.0 | NIKLNLTsPKRGQKR | S5; | 852.4 | Yes |
| **ANKHD1-EIF4EBP3** | NP_065741.3 | 404734 | ANKHD1-EIF4EBP3 protein | **S2539** | | | #N/A | VIQNSTECTDAQQASLLPSVPALKGEIPsPQLTRPK | 1 | 1 | 1 | 1 | C8(Carbamidomethyl); S29(Phospho) | S(5): 0.0; T(6): 0.0; T(9): 0.0; S(15): 0.0; S(19): 0.0; S(29): 97.5; T(33): 2.5 | ALKGEIPsPQLTRPK | S29; | 3954.0 | Yes |
| **ANKHD1-EIF4EBP3** | NP_065741.3 | 404734 | ANKHD1-EIF4EBP3 protein | **S2556** | | **2.87** | #N/A | IGRPMVAsPNQR | 1 | 1 | 1 | 0 | S8(Phospho) | S(8): 100.0 | IGRPMVAsPNQRHQD | S8; | 1405.7 | Yes |
| **ANKIB1** | NP_061877.1 | 54467 | ankyrin repeat and IBR domain-containing protein 1 | **S737** | | 1.82 | #N/A | GVAPADsPEAPRR | 3 | 1 | 1 | 1 | S7(Phospho) | S(7): 100.0 | RGVAPADsPEAPRRS | S7; | 1402.6 | Yes |
| **ANKIB1** | NP_061877.1 | 54467 | ankyrin repeat and IBR domain-containing protein 1 | **S737** | | 1.83 | #N/A | GVAPADsPEAPR | 4 | 1 | 1 | 0 | S7(Phospho) | S(7): 100.0 | RGVAPADsPEAPRRS | S7; | 1246.5 | Yes |
| **ANKIB1** | NP_061877.1 | 54467 | ankyrin repeat and IBR domain-containing protein 1 | **S737** | | 1.88 | #N/A | GVAPADsPEAPRR | 2 | 1 | 1 | 1 | S7(Phospho); R12(Label:13C(6)); R13(Label:13C(6)) | S(7): 100.0 | RGVAPADsPEAPRRS | S7; | 1414.7 | Yes |
| **ANKIB1** | NP_061877.1 | 54467 | ankyrin repeat and IBR domain-containing protein 1 | **S737** | | 1.89 | #N/A | GVAPADsPEAPR | 2 | 1 | 1 | 0 | S7(Phospho); R12(Label:13C(6)) | S(7): 100.0 | RGVAPADsPEAPRRS | S7; | 1252.6 | Yes |
| **ANKLE2** | NP_055929.1 | 23141 | ankyrin repeat and LEM domain-containing protein 2 | **S662** | 0.82 | 0.9 | 1.59 | NNsPPTVGAFGHTR | 15 | 1 | 1 | 0 | S3(Phospho) | S(3): 100.0; T(6): 0.0; T(13): 0.0 | QNAARNNsPPTVGAF | S3; | 1534.7 | Yes |
| **ANKLE2** | NP_055929.1 | 23141 | ankyrin repeat and LEM domain-containing protein 2 | **S528** | | | 1.59 | AFAGPLsPAKAEDFR | 2 | 1 | 1 | 1 | S7(Phospho) | S(7): 100.0 | RAFAGPLsPAKAEDF | S7; | 1656.8 | Yes |
| **ANKLE2** | NP_055929.1 | 23141 | ankyrin repeat and LEM domain-containing protein 2 | **S662** | 0.86 | 0.91 | 1.59 | NNsPPTVGAFGHTR | 9 | 1 | 1 | 0 | S3(Phospho); R14(Label:13C(6)) | S(3): 100.0; T(6): 0.0; T(13): 0.0 | QNAARNNsPPTVGAF | S3; | 1540.7 | Yes |
| **ANKRD11** | NP_037407.4 | 29123 | ankyrin repeat domain-containing protein 11 | **S276** | 0.67 | 0.63 | #N/A | VANsPTMVNLLLGK | 4 | 1 | 1 | 0 | S4(Phospho) | S(4): 100.0; T(6): 0.0 | TPLKVANsPTMVNLL | S4; | 1536.8 | Yes |
| **ANKRD11** | NP_037407.4 | 29123 | ankyrin repeat domain-containing protein 11 | **S276** | | | #N/A | VANsPTMVNLLLGK | 1 | 1 | 1 | 0 | S4(Phospho); K14(Label:13C(6)15N(2)) | S(4): 100.0; T(6): 0.0 | TPLKVANsPTMVNLL | S4; | 1544.8 | Yes |
| **ANKRD12** | NP_001077094.1 | 23253 | ankyrin repeat domain-containing protein 12 isoform 2 | **S327** | **0.16** | **0.11** | #NQ | QILPsK | 2 | 2 | 1 | 0 | S5(Phospho); K6(Label:13C(6)15N(2)) | S(5): 100.0 | ESKQILPsKTPLPSA | S5; | 773.4 | No |
| **ANKRD13D** | NP_997237.2 | 338692 | ankyrin repeat domain-containing protein 13D | **S552; T556** | | | #N/A | GPGsPPRtPPAPGPPSFEEQLR | 1 | 1 | 1 | 1 | S4(Phospho); T8(Phospho) | S(4): 100.0; T(8): 100.0; S(16): 0.0 | TEPRGPGsPPRTPPA; GPGSPPRtPPAPGPP | S4; T8; | 2431.1 | No;No |
| **ANKRD17** | NP_115593.3 | 26057 | ankyrin repeat domain-containing protein 17 isoform a | **S1940** | 0.91 | 0.84 | 0.87 | ATNsPKPHMVPR | 3 | 3 | 1 | 0 | S4(Phospho) | T(2): 0.0; S(4): 100.0 | SPARATNsPKPHMVP | S4; | 1414.7 | Yes |
| **ANKRD17** | NP_115593.3 | 26057 | ankyrin repeat domain-containing protein 17 isoform a | **S2401** | **3.4** | **3.24** | 0.87 | APsPAPSSVPLGSEKPSNVSQDR | 6 | 3 | 1 | 0 | S3(Phospho) | S(3): 100.0; S(7): 0.0; S(8): 0.0; S(13): 0.0; S(17): 0.0; S(20): 0.0 | SSGVRAPsPAPSSVP | S3; | 2387.1 | Yes |
| **ANKRD17** | NP_115593.3 | 26057 | ankyrin repeat domain-containing protein 17 isoform a | **S1709** | 0.69 | 0.68 | 0.87 | LTVAsPK | 2 | 3 | 1 | 0 | S5(Phospho); K7(Label:13C(6)15N(2)) | T(2): 0.0; S(5): 100.0 | NGKLTVAsPKRGQKR | S5; | 803.4 | Yes |
| **ANKRD17** | NP_115593.3 | 26057 | ankyrin repeat domain-containing protein 17 isoform a | **Y2038** | | 0.78 | 0.87 | EHyPVSSPSSPSPPAQPGGVSR | 3 | 3 | 1 | 0 | S10(Phospho) | Y(3): 98.1; S(6): 1.9; S(7): 0.0; S(9): 0.0; S(10): 0.0; S(12): 0.0; S(21): 0.0 | MPTAKEHyPVSSPSS | Y3; | 2300.0 | Yes |
| **ANKRD17** | NP_115593.3 | 26057 | ankyrin repeat domain-containing protein 17 isoform a | **S1709** | | | 0.87 | LTVAsPK | 3 | 3 | 1 | 0 | S5(Phospho) | T(2): 0.0; S(5): 100.0 | NGKLTVAsPKRGQKR | S5; | 795.4 | Yes |
| **ANKRD17** | NP_115593.3 | 26057 | ankyrin repeat domain-containing protein 17 isoform a | **S2401** | | | 0.87 | APsPAPSSVPLGSEKPSNVSQDR | 2 | 3 | 1 | 0 | S3(Phospho); K15(Label:13C(6)15N(2)); R23(Label:13C(6)) | S(3): 100.0; S(7): 0.0; S(8): 0.0; S(13): 0.0; S(17): 0.0; S(20): 0.0 | SSGVRAPsPAPSSVP | S3; | 2401.2 | Yes |
| **ANKRD17** | NP_115593.3 | 26057 | ankyrin repeat domain-containing protein 17 isoform a | **T5** | | | 0.87 | AtVPVAAATAAEGEGSPPAVAAVAGPPAAAEVGGGVGGSSR | 2 | 2 | 1 | 0 | T2(Phospho) | T(2): 100.0; T(9): 0.0; S(16): 0.0; S(39): 0.0; S(40): 0.0 | _MEKAtVPVAAAT | T2; | 3564.7 | Yes |
| **ANKRD17** | NP_115593.3 | 26057 | ankyrin repeat domain-containing protein 17 isoform a | **S1940** | | 0.87 | 0.87 | ATNsPKPHMVPR | 1 | 3 | 1 | 0 | S4(Phospho); K6(Label:13C(6)15N(2)); R12(Label:13C(6)) | T(2): 0.0; S(4): 100.0 | SPARATNsPKPHMVP | S4; | 1428.7 | Yes |
| **ANKRD28** | NP_056014.2 | 23243 | serine/threonine-protein phosphatase 6 regulatory ankyrin repeat subunit A isoform a | **S1011** | | **5.36** | **2.84** | TVsFEALPIMR | 2 | 2 | 1 | 0 | S3(Phospho) | T(1): 0.0; S(3): 100.0 | TNTSKTVsFEALPIM | S3; | 1343.6 | Yes |
| **ANKRD28** | NP_056014.2 | 23243 | serine/threonine-protein phosphatase 6 regulatory ankyrin repeat subunit A isoform a | **S1011** | | **5.53** | **2.84** | TVsFEALPIMR | 1 | 2 | 1 | 0 | S3(Phospho); R11(Label:13C(6)) | T(1): 0.0; S(3): 100.0 | TNTSKTVsFEALPIM | S3; | 1349.7 | Yes |
| **ANKRD54** | NP_620152.1 | 129138 | ankyrin repeat domain-containing protein 54 | **S63** | 0.81 | 0.83 | #NQ | ASGGAQsPLR | 2 | 1 | 1 | 0 | S7(Phospho); R10(Label:13C(6)) | S(2): 0.0; S(7): 100.0 | RASGGAQsPLRYLHV | S7; | 1029.5 | Yes |
| **ANKS1A** | NP_056060.2 | 23294 | ankyrin repeat and SAM domain-containing protein 1A | **S624** | | 0.91 | 0.81 | SLsKSDSDLLTCSPTEDATMGSR | 2 | 1 | 1 | 1 | S3(Phospho); C12(Carbamidomethyl); M20(Oxidation) | S(1): 0.0; S(3): 97.8; S(5): 2.1; S(7): 0.0; T(11): 0.0; S(13): 0.0; T(15): 0.0; T(19): 0.0; S(22): 0.0 | LKLSRSLsKSDSDLL | S3; | 2554.1 | No |
| **ANKS1A** | NP_056060.2 | 23294 | ankyrin repeat and SAM domain-containing protein 1A | **S628** | | | 0.81 | SLSKSDsDLLTCSPTEDATMGSR | 1 | 1 | 1 | 1 | S3(Phospho); K4(Label:13C(6)15N(2)); C12(Carbamidomethyl); R23(Label:13C(6)) | S(1): 0.0; S(3): 1.5; S(5): 1.5; S(7): 97.0; T(11): 0.0; S(13): 0.0; T(15): 0.0; T(19): 0.0; S(22): 0.0 | RSLSKSDsDLLTCSP | S7; | 2552.1 | Yes |
| **ANKZF1** | NP_001269721.1 | 55139 | ankyrin repeat and zinc finger domain-containing protein 1 isoform 2 | **S188** | **0.44** | | #NQ | EALGQNEEsPK | 2 | 2 | 1 | 0 | S9(Phospho); K11(Label:13C(6)15N(2)) | S(9): 100.0 | ALGQNEEsPKQGSGS | S9; | 1289.6 | No |
| **ANKZF1** | NP_001269721.1 | 55139 | ankyrin repeat and zinc finger domain-containing protein 1 isoform 2 | **S323** | **0.25** | **0.21** | #NQ | VLSLLSAPLGsGGFTLLHAAAAAGR | 6 | 2 | 1 | 0 | S11(Phospho); R25(Label:13C(6)) | S(3): 0.0; S(6): 0.0; S(11): 100.0; T(15): 0.0 | LLSAPLGsGGFTLLH | S11; | 2436.3 | No |
| **ANLN** | NP_001271231.1 | 54443 | actin-binding protein anillin isoform 3 | **S295** | 1.45 | 1.47 | 1.35 | ATsPVKSTTSITDAK | 12 | 3 | 1 | 1 | S3(Phospho) | T(2): 0.0; S(3): 100.0; S(7): 0.0; T(8): 0.0; T(9): 0.0; S(10): 0.0; T(12): 0.0 | SSSVKATsPVKSTTS | S3; | 1586.8 | Yes |
| **ANLN** | NP_001271231.1 | 54443 | actin-binding protein anillin isoform 3 | **S295** | 1.54 | | 1.35 | ATsPVKSTTSITDAK | 5 | 3 | 1 | 1 | S3(Phospho); K6(Label:13C(6)15N(2)); K15(Label:13C(6)15N(2)) | T(2): 1.1; S(3): 98.9; S(7): 0.0; T(8): 0.0; T(9): 0.0; S(10): 0.0; T(12): 0.0 | SSSVKATsPVKSTTS | S3; | 1602.8 | Yes |
| **ANLN** | NP_001271231.1 | 54443 | actin-binding protein anillin isoform 3 | **S182** | **2.5** | **2.16** | 1.35 | AAsPPRPLLSNASATPVGR | 8 | 3 | 1 | 0 | S3(Phospho) | S(3): 100.0; S(10): 0.0; S(13): 0.0; T(15): 0.0 | PSEEKAAsPPRPLLS | S3; | 1942.0 | Yes |
| **ANLN** | NP_001271231.1 | 54443 | actin-binding protein anillin isoform 3 | **S323** | | 1.73 | 1.35 | TPIsPLK | 1 | 3 | 1 | 0 | S4(Phospho) | T(1): 0.0; S(4): 100.0 | LLPKTPIsPLKTGVS | S4; | 835.4 | Yes |
| **ANLN** | NP_001271231.1 | 54443 | actin-binding protein anillin isoform 3 | **S323** | | | 1.35 | TPIsPLK | 1 | 3 | 1 | 0 | S4(Phospho); K7(Label:13C(6)15N(2)) | T(1): 0.0; S(4): 100.0 | LLPKTPIsPLKTGVS | S4; | 843.4 | Yes |
| **ANLN** | NP_001271231.1 | 54443 | actin-binding protein anillin isoform 3 | **S97** | | **2.39** | 1.35 | SCsPSPVSPQVQPQAADTISDSVAVPASLLGMR | 1 | 3 | 1 | 0 | C2(Carbamidomethyl); S5(Phospho) | S(1): 3.5; S(3): 92.9; S(5): 3.5; S(8): 0.0; T(18): 0.0; S(20): 0.0; S(22): 0.0; S(28): 0.0 | STSAKSCsPSPVSPQ | S3; | 3432.6 | Yes |
| **ANLN** | NP_001271231.1 | 54443 | actin-binding protein anillin isoform 3 | **S182** | | | 1.35 | AAsPPRPLLSNASATPVGR | 1 | 3 | 1 | 0 | S3(Phospho); R6(Label:13C(6)); R19(Label:13C(6)) | S(3): 100.0; S(10): 0.0; S(13): 0.0; T(15): 0.0 | PSEEKAAsPPRPLLS | S3; | 1954.0 | Yes |
| **ANLN** | NP_001271231.1 | 54443 | actin-binding protein anillin isoform 3 | **S323** | | **2.52** | 1.35 | TPIsPLKTGVSKPIVK | 1 | 3 | 1 | 1 | S4(Phospho) | T(1): 0.0; S(4): 100.0; T(8): 0.0; S(11): 0.0 | LLPKTPIsPLKTGVS | S4; | 1745.0 | Yes |
| **ANO6** | NP_001191732.1 | 196527 | anoctamin-6 isoform d | **S930** | | **0.37** | #N/A | MIEAVDNNLRPKsE | 2 | 3 | 1 | 1 | S13(Phospho) | S(13): 100.0 | DNNLRPKsE_ | S13; | 1695.8 | No |
| **ANO6** | NP_001191732.1 | 196527 | anoctamin-6 isoform d | **S930** | | | #N/A | MIEAVDNNLRPKsE | 1 | 3 | 1 | 1 | R10(Label:13C(6)); K12(Label:13C(6)15N(2)); S13(Phospho) | S(13): 100.0 | DNNLRPKsE_ | S13; | 1709.8 | No |
| **ANP32A** | NP_006296.1 | 8125 | acidic leucine-rich nuclear phosphoprotein 32 family member A | **T15** | | **4.46** | 1.73 | NRtPSDVKELVLDNSR | 6 | 1 | 1 | 2 | T3(Phospho) | T(3): 100.0; S(5): 0.0; S(15): 0.0 | HLELRNRtPSDVKEL | T3; | 1922.9 | Yes |
| **ANP32A** | NP_006296.1 | 8125 | acidic leucine-rich nuclear phosphoprotein 32 family member A | **T15** | | | 1.73 | NRtPSDVKELVLDNSR | 1 | 1 | 1 | 2 | R2(Label:13C(6)); T3(Phospho); K8(Label:13C(6)15N(2)); R16(Label:13C(6)) | T(3): 100.0; S(5): 0.0; S(15): 0.0 | HLELRNRtPSDVKEL | T3; | 1943.0 | Yes |
| **ANTXR1** | NP_115584.1 | 84168 | anthrax toxin receptor 1 isoform 1 precursor | **S362** | | | #N/A | EVPPPPAEEsEEEDDDGLPK | 1 | 1 | 1 | 0 | S10(Phospho) | S(10): 100.0 | PPPPAEEsEEEDDDG | S10; | 2258.9 | Yes |
| **ANXA2** | NP_001002857.1 | 302 | annexin A2 isoform 2 | **Y24** | | 1.19 | **1.96** | LSLEGDHSTPPSAyGSVK | 15 | 2 | 1 | 0 | Y14(Phospho) | S(2): 0.0; S(8): 0.0; T(9): 0.0; S(12): 0.0; Y(14): 100.0; S(16): 0.0 | HSTPPSAyGSVKAYT | Y14; | 1924.9 | Yes |
| **ANXA2** | NP_001002857.1 | 302 | annexin A2 isoform 2 | **S127** | | **4.28** | **1.96** | GLGTDEDsLIEIICSR | 2 | 2 | 1 | 0 | S8(Phospho); C14(Carbamidomethyl) | T(4): 1.7; S(8): 98.3; S(15): 0.0 | GLGTDEDsLIEIICS | S8; | 1857.8 | Yes |
| **ANXA2** | NP_001002857.1 | 302 | annexin A2 isoform 2 | **Y24** | | | **1.96** | LSLEGDHSTPPSAyGSVK | 7 | 2 | 1 | 0 | Y14(Phospho); K18(Label:13C(6)15N(2)) | S(2): 0.0; S(8): 0.0; T(9): 0.0; S(12): 1.8; Y(14): 98.2; S(16): 0.0 | HSTPPSAyGSVKAYT | Y14; | 1932.9 | Yes |
| **AP1S3** | NP_001034658.1 | 130340 | AP-1 complex subunit sigma-3 | **S41** | | | #N/A | EIVQIILsRGHR | 1 | 1 | 1 | 1 | S8(Phospho) | S(8): 100.0 | EIVQIILsRGHRTSS | S8; | 1500.8 | No |
| **AP3B1** | NP_001258698.1 | 8546 | AP-3 complex subunit beta-1 isoform 2 | **S227** | | 0.79 | 0.58 | EGDELEDNGKNFYEsDDDQKEK | 2 | 2 | 1 | 2 | K10(Label:13C(6)15N(2)); S15(Phospho); K20(Label:13C(6)15N(2)); K22(Label:13C(6)15N(2)) | Y(13): 0.0; S(15): 100.0 | NGKNFYEsDDDQKEK | S15; | 2708.1 | No |
| **APBB2** | NP_775098.2 | 323 | amyloid beta A4 precursor protein-binding family B member 2 isoform c | **T34** | | **2.31** | #N/A | NSPAtPPNTLNLR | 4 | 3 | 1 | 0 | T5(Phospho) | S(2): 0.0; T(5): 100.0; T(9): 0.0 | TNRNSPAtPPNTLNL | T5; | 1474.7 | Yes |
| **APC** | NP_001120983.2 | 324 | adenomatous polyposis coli protein isoform a | **T488; S503** | | | **0.45** | YAGMALtNLTFGDVANKATLCsMK | 1 | 2 | 1 | 1 | T7(Phospho); C21(Carbamidomethyl); S22(Phospho); M23(Oxidation) | Y(1): 0.0; T(7): 94.7; T(10): 10.2; T(19): 5.3; S(22): 89.8 | RYAGMALtNLTFGDV;ANKATLCsMKGCMRA | T7; S22; | 2753.2 | No;No |
| **APC** | NP_001120983.2 | 324 | adenomatous polyposis coli protein isoform a | **S2754** | 0.68 | | **0.45** | HSsPSGTVAAR | 1 | 2 | 1 | 0 | S3(Phospho); R11(Label:13C(6)) | S(2): 0.0; S(3): 100.0; S(5): 0.0; T(7): 0.0 | SSSSKHSsPSGTVAA | S3; | 1155.5 | No |
| **APC** | NP_001120983.2 | 324 | adenomatous polyposis coli protein isoform a | **S2265** | | 0.65 | **0.45** | SELsPVAR | 1 | 2 | 1 | 0 | S4(Phospho) | S(1): 0.0; S(4): 100.0 | PSVKSELsPVARQTS | S4; | 938.4 | No |
| **APC** | NP_001120983.2 | 324 | adenomatous polyposis coli protein isoform a | **S2817** | | | **0.45** | HsGSYLVTSV | 2 | 2 | 1 | 0 | S2(Phospho) | S(2): 99.1; S(4): 0.9; Y(5): 0.0; T(8): 0.0; S(9): 0.0 | TQSPKRHsGSYLVTS | S2; | 1129.5 | No |
| **API5** | NP_006586.1 | 8539 | apoptosis inhibitor 5 isoform b | **S464** | 1.01 | 0.94 | 0.83 | ASEDTTSGsPPKK | 8 | 4 | 1 | 1 | S9(Phospho); K12(Label:13C(6)15N(2)); K13(Label:13C(6)15N(2)) | S(2): 0.0; T(5): 0.0; T(6): 0.0; S(7): 0.0; S(9): 100.0 | SEDTTSGsPPKKSSA | S9; | 1400.6 | Yes |

| GeneSymbol | NP_Accession | Gene ID | ProteinDescription | PhosphoSite (Protein) | SCC-R / SCC-S R1 | SCC-R / SCC-S R2 | SCC-R / SCC-S Total Proteome | Sequence | # PSMs | # Proteins | # Protein Groups | # Missed Cleavages | Modifications | phosphoRS Site Probabilities | Phosphowindow | PhosphoSite (Peptide) | MH+ [Da] | PhosphositePlusEvidence |
|---|---|---|---|---|---|---|---|---|---|---|---|---|---|---|---|---|---|---|
| **API5** | NP_006586.1 | 8539 | apoptosis inhibitor 5 isoform b | **S464** | 1.09 | 0.93 | 0.83 | ASEDTTSGsPPKK | 7 | 4 | 1 | 1 | S9(Phospho) | S(2): 0.0; T(5): 0.0; T(6): 0.0; S(7): 0.0; S(9): 100.0 | SEDTTSGsPPKKSSA | S9; | 1384.6 | Yes |
| **API5** | NP_006586.1 | 8539 | apoptosis inhibitor 5 isoform b | **S464** | 1.07 | | 0.83 | RASEDTTSGsPPKK | 2 | 4 | 1 | 2 | S10(Phospho) | S(3): 0.0; T(6): 0.0; T(7): 0.0; S(8): 0.0; S(10): 100.0 | SEDTTSGsPPKKSSA | S10; | 1540.7 | Yes |
| **API5** | NP_006586.1 | 8539 | apoptosis inhibitor 5 isoform b | **S464** | 1.01 | | 0.83 | ASEDTTSGsPPK | 2 | 4 | 1 | 0 | S9(Phospho); K12(Label:13C(6)15N(2)) | S(2): 0.0; T(5): 0.0; T(6): 0.0; S(7): 0.0; S(9): 100.0 | SEDTTSGsPPKKSSA | S9; | 1264.5 | Yes |
| **APLP2** | NP_001135749.1 | 334 | amyloid-like protein 2 isoform 3 precursor | **S213** | **2.79** | | **2.17** | IIGSVsK | 1 | 4 | 1 | 0 | S6(Phospho); K7(Label:13C(6)15N(2)) | S(4): 0.0; S(6): 100.0 | TKIIGSVsKEEEEED | S6; | 791.4 | Yes |
| **APPL1** | NP_036228.1 | 26060 | DCC-interacting protein 13-alpha | **S401** | | | 0.67 | VNQSALEAVTPsPSFQQR | 3 | 1 | 1 | 0 | S12(Phospho); R18(Label:13C(6)) | S(4): 0.0; T(10): 0.0; S(12): 100.0; S(14): 0.0 | ALEAVTPsPSFQQRH | S12; | 2045.0 | Yes |
| **ARAF\|\|RAF1** | NP_001645.1\|\|NP_002871.1 | 369\|\|5894 | serine/threonine-protein kinase A-Raf isoform 1\|\|RAF proto-oncogene serine/threonine-protein kinase | **S582\|\|S621** | | | #N/A | SAsEPSLHR | 2 | 3 | 2 | 0 | S3(Phospho); R9(Label:13C(6)) | S(1): 0.0; S(3): 100.0; S(6): 0.0 | PKIERSAsEPSLHRT\|\|PKINRSAsEPSLHRA | S3; | 1069.5 | Yes\|\|Yes |
| **ARAF\|\|RAF1** | NP_001645.1\|\|NP_002871.1 | 369\|\|5894 | serine/threonine-protein kinase A-Raf isoform 1\|\|RAF proto-oncogene serine/threonine-protein kinase | **S582\|\|S621** | | | #N/A | SAsEPSLHR | 2 | 3 | 2 | 0 | S3(Phospho) | S(1): 0.0; S(3): 100.0; S(6): 0.0 | PKIERSAsEPSLHRT\|\|PKINRSAsEPSLHRA | S3; | 1063.5 | Yes\|\|Yes |
| **ARAP3** | NP_071926.4 | 64411 | arf-GAP with Rho-GAP domain, ANK repeat and PH domain-containing protein 3 | **S1444** | | **2.01** | #N/A | sLDQPFLSK | 2 | 1 | 1 | 0 | S1(Phospho) | S(1): 100.0; S(8): 0.0 | NPLTSQKsLDQPFLS | S1; | 1114.5 | Yes |
| **ARFGAP2** | NP_115765.2 | 84364 | ADP-ribosylation factor GTPase-activating protein 2 isoform 1 | **S432** | **0.3** | **0.29** | **0.39** | AIsSDMFFGR | 6 | 2 | 1 | 0 | S3(Phospho) | S(3): 100.0; S(4): 0.0 | FAGAKAIsSDMFFGR | S3; | 1210.5 | Yes |
| **ARFGAP2** | NP_115765.2 | 84364 | ADP-ribosylation factor GTPase-activating protein 2 isoform 1 | **S146** | **0.46** | 0.54 | **0.39** | HGTDLWIDNMSSAVPNHsPEKK | 8 | 1 | 1 | 1 | S18(Phospho); K21(Label:13C(6)15N(2)); K22(Label:13C(6)15N(2)) | T(3): 0.0; S(11): 0.0; S(12): 0.0; S(18): 100.0 | SSAVPNHsPEKKDSD | S18; | 2559.2 | Yes |
| **ARFGAP2** | NP_115765.2 | 84364 | ADP-ribosylation factor GTPase-activating protein 2 isoform 1 | **S432** | **0.31** | **0.29** | **0.39** | AIsSDMFFGR | 4 | 2 | 1 | 0 | S3(Phospho); R10(Label:13C(6)) | S(3): 100.0; S(4): 0.0 | FAGAKAIsSDMFFGR | S3; | 1216.5 | Yes |
| **ARFGEF1** | NP_006412.2 | 10565 | brefeldin A-inhibited guanine nucleotide-exchange protein 1 | **S1569** | 1 | 0.95 | 0.90 | sVDIHDSIQPR | 11 | 1 | 1 | 0 | S1(Phospho) | S(1): 100.0; S(7): 0.0 | LDTISQKsVDIHDSI | S1; | 1346.6 | Yes |
| **ARFGEF1** | NP_006412.2 | 10565 | brefeldin A-inhibited guanine nucleotide-exchange protein 1 | **S1569** | 0.99 | 1.02 | 0.90 | sVDIHDSIQPR | 2 | 1 | 1 | 0 | S1(Phospho); R11(Label:13C(6)) | S(1): 100.0; S(7): 0.0 | LDTISQKsVDIHDSI | S1; | 1352.6 | Yes |
| **ARFGEF2** | NP_006411.2 | 10564 | brefeldin A-inhibited guanine nucleotide-exchange protein 2 | **S227** | 1.24 | 1.39 | 1.41 | ELEKPIQSKPQSPVIQAAAVsPK | 6 | 1 | 1 | 0 | K4(Label:13C(6)15N(2)); K9(Label:13C(6)15N(2)); S21(Phospho); K23(Label:13C(6)15N(2)) | S(8): 0.0; S(12): 0.0; S(21): 100.0 | VIQAAAVsPKFVRLK | S21; | 2549.4 | Yes |
| **ARFGEF2** | NP_006411.2 | 10564 | brefeldin A-inhibited guanine nucleotide-exchange protein 2 | **S227** | 1.3 | 1.36 | 1.41 | ELEKPIQSKPQSPVIQAAAVsPK | 10 | 1 | 1 | 0 | S21(Phospho) | S(8): 0.0; S(12): 0.0; S(21): 100.0 | VIQAAAVsPKFVRLK | S21; | 2525.3 | Yes |
| **ARFGEF2** | NP_006411.2 | 10564 | brefeldin A-inhibited guanine nucleotide-exchange protein 2 | **S218; S227** | | **4.21** | 1.41 | ELEKPIQSKPQsPVIQAAAVsPK | 1 | 1 | 1 | 0 | K4(Label:13C(6)15N(2)); K9(Label:13C(6)15N(2)); S12(Phospho); S21(Phospho); K23(Label:13C(6)15N(2)) | S(8): 0.0; S(12): 100.0; S(21): 100.0 | PIQSKPQsPVIQAAA;VIQAAAVsPKFVRLK | S12; S21; | 2629.3 | Yes;Yes |
| **ARFGEF2** | NP_006411.2 | 10564 | brefeldin A-inhibited guanine nucleotide-exchange protein 2 | **S218; S227** | | **4.35** | 1.41 | ELEKPIQSKPQsPVIQAAAVsPK | 2 | 1 | 1 | 0 | S12(Phospho); S21(Phospho) | S(8): 0.0; S(12): 100.0; S(21): 100.0 | PIQSKPQsPVIQAAA;VIQAAAVsPKFVRLK | S12; S21; | 2605.3 | Yes;Yes |
| **ARGLU1** | NP_060481.3 | 55082 | arginine and glutamate-rich protein 1 | **S77** | 1.66 | 1.45 | 1.75 | ASsPPDRIDIFGR | 7 | 1 | 1 | 1 | S3(Phospho) | S(2): 0.0; S(3): 100.0 | RDRERASsPPDRIDI | S3; | 1510.7 | Yes |
| **ARGLU1** | NP_060481.3 | 55082 | arginine and glutamate-rich protein 1 | **S77** | | | 1.75 | ASsPPDRIDIFGR | 1 | 1 | 1 | 1 | S3(Phospho); R7(Label:13C(6)); R13(Label:13C(6)) | S(2): 1.4; S(3): 98.6 | RDRERASsPPDRIDI | S3; | 1522.7 | Yes |
| **ARHGAP11A** | NP_001273408.1 | 9824 | rho GTPase-activating protein 11A isoform 3 | **T134** | 0.66 | 0.74 | #N/A | IAQLSESPVILtPNAKR | 2 | 3 | 1 | 1 | T12(Phospho); K16(Label:13C(6)15N(2)); R17(Label:13C(6)) | S(5): 0.0; S(7): 0.0; T(12): 100.0 | SESPVILtPNAKRTL | T12; | 1931.1 | No |
| **ARHGAP11A** | NP_001273408.1 | 9824 | rho GTPase-activating protein 11A isoform 3 | **T134** | | | #N/A | IAQLSESPVILtPNAKR | 1 | 3 | 1 | 1 | T12(Phospho) | S(5): 0.0; S(7): 0.0; T(12): 100.0 | SESPVILtPNAKRTL | T12; | 1917.0 | No |
| **ARHGAP12** | NP_001257627.1 | 94134 | rho GTPase-activating protein 12 isoform 5 | **T230; S240** | **2.34** | | #N/A | AtTPPNQGRPDsPVYANLQELK | 1 | 6 | 1 | 0 | T2(Phospho); R9(Label:13C(6)); S12(Phospho); K22(Label:13C(6)15N(2)) | T(2): 96.7; T(3): 3.3; S(12): 99.9; Y(15): 0.1 | STEQIRAtTPPNQGR;PNQGRPDsPVYANLQ | T2; S12; | 2570.2 | Yes;Yes |
| **ARHGAP12** | NP_001257627.1 | 94134 | rho GTPase-activating protein 12 isoform 5 | **S240** | | | #N/A | ATTPPNQGRPDsPVYANLQELK | 1 | 6 | 1 | 0 | T2(Phospho); S12(Phospho) | T(2): 50.0; T(3): 50.0; S(12): 99.8; Y(15): 0.2 | PNQGRPDsPVYANLQ | S12; | 2556.2 | Yes |
| **ARHGAP12** | NP_001257627.1 | 94134 | rho GTPase-activating protein 12 isoform 5 | **S240** | | **3.72** | #N/A | ATTPPNQGRPDsPVYANLQELK | 1 | 6 | 1 | 0 | R9(Label:13C(6)); S12(Phospho); K22(Label:13C(6)15N(2)) | T(2): 0.0; T(3): 0.0; S(12): 100.0; Y(15): 0.0 | PNQGRPDsPVYANLQ | S12; | 2490.2 | Yes |
| **ARHGAP12** | NP_001257627.1 | 94134 | rho GTPase-activating protein 12 isoform 5 | **S240** | | | #N/A | ATTPPNQGRPDsPVYANLQELK | 1 | 6 | 1 | 0 | S12(Phospho) | T(2): 2.4; T(3): 0.1; S(12): 97.4; Y(15): 0.1 | PNQGRPDsPVYANLQ | S12; | 2476.2 | Yes |
| **ARHGAP17** | NP_001006635.1 | 55114 | rho GTPase-activating protein 17 isoform 1 | **S625** | | | 0.95 | NNSQIASGQNQPQAAAGSHQLSMGQPHNAAGPsPHTLRR | 2 | 2 | 1 | 1 | S33(Phospho) | S(3): 0.0; S(7): 0.0; S(18): 0.0; S(22): 0.0; S(33): 99.9; T(36): 0.1 | PHNAAGPsPHTLRRA | S33; | 4095.9 | Yes |
| **ARHGAP17** | NP_001006635.1 | 55114 | rho GTPase-activating protein 17 isoform 1 | **S625** | | **3.06** | 0.95 | NNSQIASGQNQPQAAAGSHQLSMGQPHNAAGPsPHTLR | 2 | 2 | 1 | 0 | T36(Phospho) | S(3): 0.0; S(7): 0.0; S(18): 0.0; S(22): 0.0; S(33): 97.0; T(36): 3.0 | PHNAAGPsPHTLRRA | S33; | 3939.8 | Yes |
| **ARHGAP21** | NP_065875.3 | 57584 | rho GTPase-activating protein 21 | **S1861** | 1.46 | 1.48 | #N/A | LRTsTSDLSR | 2 | 1 | 1 | 1 | S4(Phospho) | T(3): 1.1; S(4): 97.7; T(5): 0.0; S(6): 1.1; S(9): 0.0 | ARERLRTsTSDLSRG | S4; | 1215.6 | No |
| **ARHGAP21** | NP_065875.3 | 57584 | rho GTPase-activating protein 21 | **S616** | **2.65** | | #N/A | GISQDRsPLVK | 1 | 1 | 1 | 1 | S7(Phospho) | S(3): 0.0; S(7): 100.0 | RGISQDRsPLVKVRS | S7; | 1279.6 | No |
| **ARHGAP21** | NP_065875.3 | 57584 | rho GTPase-activating protein 21 | **S1669** | | | #N/A | RNsEGSELSCTEGSLTSSLDSR | 2 | 1 | 1 | 1 | S3(Phospho); C10(Carbamidomethyl) | S(3): 99.9; S(6): 0.1; S(9): 0.0; T(11): 0.0; S(14): 0.0; T(16): 0.0; S(17): 0.0; S(18): 0.0; S(21): 0.0 | TKSSRRNsEGSELSC | S3; | 2452.0 | No |
| **ARHGAP21** | NP_065875.3 | 57584 | rho GTPase-activating protein 21 | **S1669** | | | #N/A | RNsEGSELSCTEGSLTSSLDSR | 1 | 1 | 1 | 1 | R1(Label:13C(6)); S3(Phospho); C10(Carbamidomethyl); R22(Label:13C(6)) | S(3): 100.0; S(6): 0.0; S(9): 0.0; T(11): 0.0; S(14): 0.0; T(16): 0.0; S(17): 0.0; S(18): 0.0; S(21): 0.0 | TKSSRRNsEGSELSC | S3; | 2464.1 | No |
| **ARHGAP23** | NP_001186346.1 | 57636 | rho GTPase-activating protein 23 | **S647** | 0.85 | 1.03 | 0.90 | LPNRIPsLR | 3 | 1 | 1 | 1 | S7(Phospho) | S(7): 100.0 | RLPNRIPsLRMLRSF | S7; | 1145.6 | Yes |
| **ARHGAP23** | NP_001186346.1 | 57636 | rho GTPase-activating protein 23 | **S361** | 0.66 | | 0.90 | sAEALGPGALVSPR | 1 | 1 | 1 | 0 | S1(Phospho); R14(Label:13C(6)) | S(1): 100.0; S(12): 0.0 | YLSRATRsAEALGPG | S1; | 1410.7 | Yes |
| **ARHGAP23** | NP_001186346.1 | 57636 | rho GTPase-activating protein 23 | **S554** | | | 0.90 | KVAPLATTEDSLASIPFIDEPTsPSIDLQAK | 2 | 1 | 1 | 1 | S23(Phospho) | T(7): 0.0; T(8): 0.0; S(11): 0.0; S(14): 0.0; T(22): 0.0; S(23): 100.0; S(25): 0.0 | PFIDEPTsPSIDLQA | S23; | 3334.7 | Yes |
| **ARHGAP23** | NP_001186346.1 | 57636 | rho GTPase-activating protein 23 | **S372** | | 1.65 | 0.90 | SAEALGPGALVsPR | 2 | 1 | 1 | 0 | S12(Phospho) | S(1): 0.0; S(12): 100.0 | LGPGALVsPRFERCG | S12; | 1404.7 | Yes |
| **ARHGAP23** | NP_001186346.1 | 57636 | rho GTPase-activating protein 23 | **S1393** | | 0.81 | 0.90 | RPLsPETR | 1 | 1 | 1 | 0 | R1(Label:13C(6)); S4(Phospho); R8(Label:13C(6)) | S(4): 100.0; T(7): 0.0 | VRLRRPLsPETRRRR | S4; | 1047.5 | Yes |
| **ARHGAP26** | NP_001129080.1 | 23092 | rho GTPase-activating protein 26 isoform b | **T229; S231; T235; T242** | | | #NQ | TQLtIsIQNtRNRFEGtR | 1 | 2 | 1 | 2 | T4(Phospho); S6(Phospho); T10(Phospho); T17(Phospho) | T(1): 0.8; T(4): 99.2; S(6): 100.0; T(10): 100.0; T(17): 100.0 | GDFKTQLtISIQNTR;FKTQLTIsIQNTRNR;LTISIQNtRNRFEGT;TRNRFEGtRSEVESL | T4; S6; T10; T17; | 2455.0 | No;No;No;No |
| **ARHGAP29** | NP_004806.3 | 9411 | rho GTPase-activating protein 29 | **S552** | **2.98** | 1.7 | 1.15 | sLDSESISPGDFHR | 6 | 1 | 1 | 0 | S1(Phospho) | S(1): 100.0; S(4): 0.0; S(6): 0.0; S(8): 0.0 | GGSSESRsLDSESIS | S1; | 1626.7 | Yes |
| **ARHGAP29** | NP_004806.3 | 9411 | rho GTPase-activating protein 29 | **S1029** | **5.68** | **6.55** | 1.15 | LLLAsPPNER | 14 | 1 | 1 | 0 | S5(Phospho) | S(5): 100.0 | VDRLLLAsPPNERNG | S5; | 1189.6 | Yes |
| **ARHGAP29** | NP_004806.3 | 9411 | rho GTPase-activating protein 29 | **S1029** | | | 1.15 | LLLAsPPNER | 2 | 1 | 1 | 0 | S5(Phospho); R10(Label:13C(6)) | S(5): 100.0 | VDRLLLAsPPNERNG | S5; | 1195.6 | Yes |
| **ARHGAP29** | NP_004806.3 | 9411 | rho GTPase-activating protein 29 | **S589** | | **3.88** | 1.15 | EPPsPSETGPNSLGTFKK | 1 | 1 | 1 | 1 | S4(Phospho); K17(Label:13C(6)15N(2)); K18(Label:13C(6)15N(2)) | S(4): 100.0; S(6): 0.0; T(8): 0.0; S(12): 0.0; T(15): 0.0 | LDEREPPsPSETGPN | S4; | 1968.9 | Yes |
| **ARHGAP29** | NP_004806.3 | 9411 | rho GTPase-activating protein 29 | **S552** | | **1.98** | 1.15 | sLDSESISPGDFHR | 1 | 1 | 1 | 0 | S1(Phospho); R14(Label:13C(6)) | S(1): 100.0; S(4): 0.0; S(6): 0.0; S(8): 0.0 | GGSSESRsLDSESIS | S1; | 1632.7 | Yes |
| **ARHGAP29** | NP_004806.3 | 9411 | rho GTPase-activating protein 29 | **S949** | | 1.41 | 1.15 | ATsFEESER | 1 | 1 | 1 | 0 | S3(Phospho); R9(Label:13C(6)) | T(2): 0.0; S(3): 100.0; S(7): 0.0 | KIFERATsFEESERK | S3; | 1141.5 | Yes |
| **ARHGAP31** | NP_065805.2 | 57514 | rho GTPase-activating protein 31 | **S1432** | | | #NQ | sVILDGR | 1 | 1 | 1 | 0 | S1(Phospho) | S(1): 100.0 | FYQPQRRsVILDGRS | S1; | 839.4 | No |
| **ARHGAP31** | NP_065805.2 | 57514 | rho GTPase-activating protein 31 | **S1374** | | | #NQ | VLLsPIRSPTQTVSPGLLCGELAENTWVTPEGVTLR | 1 | 1 | 1 | 1 | S14(Phospho); C19(Carbamidomethyl) | S(4): 86.0; S(8): 3.5; T(10): 3.5; T(12): 3.5; S(14): 3.5; T(26): 0.2; T(29): 0.0; T(34): 0.0 | TDSKVLLsPIRSPTQ | S4; | 3971.0 | No |
| **ARHGAP31** | NP_065805.2 | 57514 | rho GTPase-activating protein 31 | **S346** | | | #NQ | sMDSLCSVPVEGK | 2 | 1 | 1 | 0 | S1(Phospho); C6(Carbamidomethyl) | S(1): 98.9; S(4): 1.1; S(7): 0.0 | ATIRPAKsMDSLCSV | S1; | 1488.6 | Yes |
| **ARHGAP32** | NP_055530.2 | 9743 | rho GTPase-activating protein 32 isoform 2 | **S543** | 0.53 | | #N/A | VVYAFsPK | 2 | 2 | 1 | 0 | S6(Phospho); K8(Label:13C(6)15N(2)) | Y(3): 0.0; S(6): 100.0 | EKVVYAFsPKIGRKL | S6; | 998.5 | No |
| **ARHGAP35** | NP_004482.4 | 2909 | rho GTPase-activating protein 35 | **S1127** | **0.47** | | #N/A | AQsNGSGNGSDSEMDTSSLER | 1 | 1 | 1 | 0 | S10(Phospho); R21(Label:13C(6)) | S(3): 98.1; S(6): 0.0; S(10): 1.8; S(12): 0.0; T(16): 0.0; S(17): 0.0; S(18): 0.0 | RNINKAQsNGSGNGS | S3; | 2214.9 | Yes |
| **ARHGAP35** | NP_004482.4 | 2909 | rho GTPase-activating protein 35 | **S1179** | | 0.79 | #N/A | TSFSVGsDDELGPIR | 2 | 1 | 1 | 0 | S7(Phospho) | T(1): 0.0; S(2): 0.0; S(4): 0.0; S(7): 100.0 | RTSFSVGsDDELGPI | S7; | 1659.7 | Yes |
| **ARHGAP35** | NP_004482.4 | 2909 | rho GTPase-activating protein 35 | **Y1105** | | 0.57 | #N/A | NEEENIySVPHDSTQGK | 1 | 1 | 1 | 0 | Y7(Phospho); K17(Label:13C(6)15N(2)) | Y(7): 100.0; S(8): 0.0; S(13): 0.0; T(14): 0.0 | RNEEENIySVPHDST | Y7; | 2034.9 | Yes |
| **ARHGAP35** | NP_004482.4 | 2909 | rho GTPase-activating protein 35 | **Y1105** | | | #N/A | NEEENIySVPHDSTQGK | 1 | 1 | 1 | 0 | Y7(Phospho) | Y(7): 98.3; S(8): 1.7; S(13): 0.0; T(14): 0.0 | RNEEENIySVPHDST | Y7; | 2026.8 | Yes |

**Supplementary Table S5:List of phosphopeptides identified in SCC-S and SCC-R cells using SequestHT and Mascot search algorithms**

| GeneSymbol | NP_Accession | Gene ID | ProteinDescription | PhosphoSite (Protein) | SCC-R / SCC-S R1 | SCC-R / SCC-S R2 | SCC-R / SCC-S Total Proteome | Sequence | # PSMs | # Proteins | # Protein Groups | # Missed Cleavages | Modifications | phosphoRS Site Probabilities | Phosphowindow | PhosphoSite (Peptide) | MH+ [Da] | PhosphositePlusEvidence |
|---|---|---|---|---|---|---|---|---|---|---|---|---|---|---|---|---|---|---|
| **ARHGAP40** | NP_001157903.1 | 343578 | rho GTPase-activating protein 40 | **S203; S206** | | | #N/A | GAQLSsGAsKFPPAAEPGGLQEQAGR | 1 | 1 | 1 | 1 | S6(Phospho); S9(Phospho); K10(Label:13C(6)15N(2)) | S(5): 4.5; S(6): 95.7; S(9): 99.8 | MKGAQLSsGASKFPP; AQLSSGAsKFPPAAE | S6; S9; | 2679.2 | No;No |
| **ARHGEF1** | NP_945328.1 | 9138 | rho guanine nucleotide exchange factor 1 isoform 3 | **S10** | | | **3.57** | GAAsPGPSRPGLVPVSIIGAEDEDFENELETNSEEQNSQFQSLEQVK | 1 | 3 | 1 | 0 | S8(Phospho) | S(4): 99.9; S(8): 0.1; S(16): 0.0; T(31): 0.0; S(33): 0.0; S(38): 0.0; S(42): 0.0 | DFARGAAsPGPSRPG | S4; | 5109.4 | Yes |
| **ARHGEF1** | NP_945328.1 | 9138 | rho guanine nucleotide exchange factor 1 isoform 3 | **T682; T687** | | | **3.57** | TMLRPVLRLTSAMtREVAtDHK | 1 | 3 | 1 | 2 | R8(Label:13C(6)); M13(Oxidation); T14(Phospho); T19(Phospho) | T(1): 0.0; T(10): 0.5; S(11): 7.2; T(14): 96.2; T(19): 96.2 | LRLTSAMtREVATDH;AMTREVAtDHKAFYV | T14; T19; | 2708.3 | No;No |
| **ARHGEF10** | NP_055444.2 | 9639 | rho guanine nucleotide exchange factor 10 | **S354** | **5.54** | **8.19** | 1.57 | sLIAQDHR | 4 | 1 | 1 | 0 | S1(Phospho) | S(1): 100.0 | RSFIRTKsLIAQDHR | S1; | 1019.5 | No |
| **ARHGEF10** | NP_055444.2 | 9639 | rho guanine nucleotide exchange factor 10 | **S354** | **5.81** | | 1.57 | sLIAQDHR | 1 | 1 | 1 | 0 | S1(Phospho); R8(Label:13C(6)) | S(1): 100.0 | RSFIRTKsLIAQDHR | S1; | 1025.5 | No |
| **ARHGEF11** | NP_055599.1 | 9826 | rho guanine nucleotide exchange factor 11 isoform 1 | **S1480** | 1.46 | 1.72 | #N/A | WTDGSLsPPAKEPLASDSR | 11 | 2 | 1 | 1 | S7(Phospho) | T(2): 0.0; S(5): 0.0; S(7): 100.0; S(16): 0.0; S(18): 0.0 | RWTDGSLsPPAKEPL | S7; | 2094.0 | Yes |
| **ARHGEF11** | NP_055599.1 | 9826 | rho guanine nucleotide exchange factor 11 isoform 1 | **S1480** | | 1.44 | #N/A | WTDGSLsPPAKEPLASDSR | 4 | 2 | 1 | 1 | S7(Phospho); K11(Label:13C(6)15N(2)); R19(Label:13C(6)) | T(2): 0.0; S(5): 0.0; S(7): 100.0; S(16): 0.0; S(18): 0.0 | RWTDGSLsPPAKEPL | S7; | 2108.0 | Yes |
| **ARHGEF11** | NP_055599.1 | 9826 | rho guanine nucleotide exchange factor 11 isoform 1 | **S1155** | | **1.95** | #N/A | HQVLLEDPEQEGsAEEEELGVLPCPSTSLDGENR | 2 | 2 | 1 | 0 | S13(Phospho); C24(Carbamidomethyl) | S(13): 100.0; S(26): 0.0; T(27): 0.0; S(28): 0.0 | EDPEQEGsAEEEELG | S13; | 3843.7 | Yes |
| **ARHGEF11** | NP_055599.1 | 9826 | rho guanine nucleotide exchange factor 11 isoform 1 | **S251; S255** | | 1.43 | #N/A | NSVLSDPGLDsPRTsPVIMAR | 2 | 2 | 1 | 1 | S11(Phospho); S15(Phospho) | S(2): 0.0; S(5): 0.0; S(11): 100.0; T(14): 0.0; S(15): 100.0 | LSDPGLDsPRTSPVI;GLDSPRTsPVIMARV | S11; S15; | 2372.1 | Yes;Yes |
| **ARHGEF11** | NP_055599.1 | 9826 | rho guanine nucleotide exchange factor 11 isoform 1 | **S251; S255** | | 1.41 | #N/A | NSVLSDPGLDsPRTsPVIMAR | 2 | 2 | 1 | 1 | S11(Phospho); R13(Label:13C(6)); S15(Phospho); R21(Label:13C(6)) | S(2): 0.0; S(5): 0.0; S(11): 100.0; T(14): 1.7; S(15): 98.3 | LSDPGLDsPRTSPVI;GLDSPRTsPVIMARV | S11; S15; | 2384.1 | Yes;Yes |
| **ARHGEF11** | NP_055599.1 | 9826 | rho guanine nucleotide exchange factor 11 isoform 1 | **Y1320** | | | #N/A | NLAEDASSTEAAGGyK | 1 | 2 | 1 | 0 | Y15(Phospho); K16(Label:13C(6)15N(2)) | S(7): 0.0; S(8): 0.1; T(9): 2.7; Y(15): 97.2 | STEAAGGyKVVRKAE | Y15; | 1671.7 | No |
| **ARHGEF11** | NP_055599.1 | 9826 | rho guanine nucleotide exchange factor 11 isoform 1 | **T668** | | 1.11 | #N/A | SLENPtPPFTPK | 1 | 2 | 1 | 0 | T6(Phospho); K12(Label:13C(6)15N(2)) | S(1): 0.0; T(6): 100.0; T(10): 0.0 | TRSLENPtPPFTPKM | T6; | 1415.7 | Yes |
| **ARHGEF12** | NP_001185594.1 | 23365 | rho guanine nucleotide exchange factor 12 isoform 2 | **T717** | 1.08 | | #NQ | VTEHGtPKPFR | 1 | 2 | 1 | 0 | T6(Phospho) | T(2): 0.0; T(6): 100.0 | ERVTEHGtPKPFRKF | T6; | 1348.6 | No |
| **ARHGEF12** | NP_001185594.1 | 23365 | rho guanine nucleotide exchange factor 12 isoform 2 | **S322** | | 1.34 | #NQ | IYLEENPEKSETIQDTDTQSLVGsPSTR | 1 | 2 | 1 | 1 | S24(Phospho) | Y(2): 0.0; S(10): 0.0; T(12): 0.0; T(16): 0.0; T(18): 0.0; S(20): 0.0; S(24): 96.3; S(26): 1.8; T(27): 1.8 | DTQSLVGsPSTRIAP | S24; | 3217.5 | No |
| **ARHGEF16** | NP_055263.2 | 27237 | rho guanine nucleotide exchange factor 16 | **S174** | **0.33** | | **0.41** | GLGKPGGQGDAIQLsPK | 2 | 1 | 1 | 0 | S15(Phospho) | S(15): 100.0 | QGDAIQLsPKLQALA | S15; | 1702.9 | Yes |
| **ARHGEF16** | NP_055263.2 | 27237 | rho guanine nucleotide exchange factor 16 | **S174** | | 0.52 | **0.41** | GLGKPGGQGDAIQLsPK | 2 | 1 | 1 | 0 | K4(Label:13C(6)15N(2)); S15(Phospho); K17(Label:13C(6)15N(2)) | S(15): 100.0 | QGDAIQLsPKLQALA | S15; | 1718.9 | Yes |
| **ARHGEF17** | NP_055601.2 | 9828 | rho guanine nucleotide exchange factor 17 | **S914** | 1.37 | 1.42 | #N/A | LADILsPR | 9 | 1 | 1 | 0 | S6(Phospho) | S(6): 100.0 | PKLADILsPRLIRRG | S6; | 964.5 | Yes |
| **ARHGEF17** | NP_055601.2 | 9828 | rho guanine nucleotide exchange factor 17 | **S914** | 1.26 | | #N/A | LADILsPR | 3 | 1 | 1 | 0 | S6(Phospho); R8(Label:13C(6)) | S(6): 100.0 | PKLADILsPRLIRRG | S6; | 970.5 | Yes |
| **ARHGEF17** | NP_055601.2 | 9828 | rho guanine nucleotide exchange factor 17 | **S463** | | **2.36** | #N/A | SLsNPDIASETLTLLSFLR | 4 | 1 | 1 | 0 | S3(Phospho) | S(1): 0.0; S(3): 100.0; S(9): 0.0; T(11): 0.0; T(13): 0.0; S(16): 0.0 | SRQRKSLsNPDIASE | S3; | 2157.1 | Yes |
| **ARHGEF17** | NP_055601.2 | 9828 | rho guanine nucleotide exchange factor 17 | **S142** | | 1.32 | #N/A | RPsADSESPGTPSPDGAAWEPPAR | 2 | 1 | 1 | 0 | S3(Phospho) | S(3): 100.0; S(6): 0.0; S(8): 0.0; T(11): 0.0; S(13): 0.0 | GPGSRRPsADSESPG | S3; | 2515.1 | Yes |
| **ARHGEF17** | NP_055601.2 | 9828 | rho guanine nucleotide exchange factor 17 | **S461** | | **1.95** | #N/A | KsLSNPDIASETLTLLSFLR | 2 | 1 | 1 | 1 | S4(Phospho) | S(2): 99.2; S(4): 0.8; S(10): 0.0; T(12): 0.0; T(14): 0.0; S(17): 0.0 | EKSRQRKsLSNPDIA | S2; | 2285.2 | Yes |
| **ARHGEF17** | NP_055601.2 | 9828 | rho guanine nucleotide exchange factor 17 | **S79** | | **2.06** | #N/A | SLsPSVR | 1 | 1 | 1 | 0 | S3(Phospho) | S(1): 0.0; S(3): 100.0; S(5): 0.0 | PRPLRSLsPSVRQLS | S3; | 825.4 | Yes |
| **ARHGEF17** | NP_055601.2 | 9828 | rho guanine nucleotide exchange factor 17 | **S463** | | **2.01** | #N/A | KSLsNPDIASETLTLLSFLR | 1 | 1 | 1 | 1 | K1(Label:13C(6)15N(2)); S4(Phospho); R20(Label:13C(6)) | S(2): 1.4; S(4): 98.6; S(10): 0.0; T(12): 0.0; T(14): 0.0; S(17): 0.0 | SRQRKSLsNPDIASE | S4; | 2299.2 | Yes |
| **ARHGEF17** | NP_055601.2 | 9828 | rho guanine nucleotide exchange factor 17 | **S142** | | | #N/A | RPsADSESPGTPSPDGAAWEPPAR | 1 | 1 | 1 | 0 | R1(Label:13C(6)); S3(Phospho); R24(Label:13C(6)) | S(3): 100.0; S(6): 0.0; S(8): 0.0; T(11): 0.0; S(13): 0.0 | GPGSRRPsADSESPG | S3; | 2527.1 | Yes |
| **ARHGEF18** | NP_056133.2 | 23370 | rho guanine nucleotide exchange factor 18 isoform a | **S966** | | | 0.63 | HSPAPPPDPGFPAPsPPPADSPSEGFSLK | 1 | 2 | 1 | 0 | S15(Phospho) | S(2): 0.0; S(15): 100.0; S(21): 0.0; S(23): 0.0; S(27): 0.0 | DPGFPAPsPPPADSP | S15; | 2960.4 | No |
| **ARHGEF18** | NP_056133.2 | 23370 | rho guanine nucleotide exchange factor 18 isoform a | **S945** | | 0.85 | 0.63 | SLsPILPGR | 1 | 2 | 1 | 0 | S3(Phospho) | S(1): 0.0; S(3): 100.0 | SRNRRSLsPILPGRH | S3; | 1019.5 | No |
| **ARHGEF2** | NP_004714.2 | 9181 | rho guanine nucleotide exchange factor 2 isoform 3 | **S147** | **2.57** | **2.54** | #NQ | ILSQsTDSLNMR | 6 | 3 | 1 | 0 | S5(Phospho) | S(3): 0.0; S(5): 99.2; T(6): 0.8; S(8): 0.0 | LRRILSQsTDSLNMR | S5; | 1444.6 | No |
| **ARHGEF2** | NP_004714.2 | 9181 | rho guanine nucleotide exchange factor 2 isoform 3 | **S124** | | | #NQ | SVsTTNIAGHFNDESPLGLR | 8 | 3 | 1 | 0 | S3(Phospho) | S(1): 0.0; S(3): 98.7; T(4): 1.2; T(5): 0.0; S(15): 0.0 | LSLAKSVsTTNIAGH | S3; | 2195.0 | No |
| **ARHGEF2** | NP_004714.2 | 9181 | rho guanine nucleotide exchange factor 2 isoform 3 | **S124** | **6.24** | **6.05** | #NQ | SVsTTNIAGHFNDESPLGLRR | 4 | 3 | 1 | 1 | S3(Phospho) | S(1): 0.0; S(3): 100.0; T(4): 0.0; T(5): 0.0; S(15): 0.0 | LSLAKSVsTTNIAGH | S3; | 2351.1 | No |
| **ARHGEF2** | NP_004714.2 | 9181 | rho guanine nucleotide exchange factor 2 isoform 3 | **S147** | 1.36 | **4.15** | #NQ | ILSQsTDSLNMR | 3 | 3 | 1 | 0 | S5(Phospho); M11(Oxidation) | S(3): 0.0; S(5): 98.8; T(6): 1.2; S(8): 0.0 | LRRILSQsTDSLNMR | S5; | 1460.6 | No |
| **ARHGEF2** | NP_004714.2 | 9181 | rho guanine nucleotide exchange factor 2 isoform 3 | **S147** | | | #NQ | ILSQsTDSLNMR | 2 | 3 | 1 | 0 | S5(Phospho); R12(Label:13C(6)) | S(3): 0.0; S(5): 99.1; T(6): 0.9; S(8): 0.0 | LRRILSQsTDSLNMR | S5; | 1450.7 | No |
| **ARHGEF2** | NP_004714.2 | 9181 | rho guanine nucleotide exchange factor 2 isoform 3 | **S858** | | **3.05** | #NQ | sLPAGDALYLSFNPPQPSR | 8 | 3 | 1 | 0 | S1(Phospho) | S(1): 100.0; Y(9): 0.0; S(11): 0.0; S(18): 0.0 | PVDPRRRsLPAGDAL | S1; | 2110.0 | No |
| **ARHGEF2** | NP_004714.2 | 9181 | rho guanine nucleotide exchange factor 2 isoform 3 | **S904** | | **2.18** | #NQ | ERQELGsPEER | 1 | 3 | 1 | 1 | S7(Phospho) | S(7): 100.0 | RERQELGsPEERLQD | S7; | 1409.6 | No |
| **ARHGEF40** | NP_060541.3 | 55701 | rho guanine nucleotide exchange factor 40 isoform 1 | **T371** | | **2.5** | #N/A | GGGGGGQGAEGPPGtPR | 2 | 1 | 1 | 0 | T15(Phospho) | T(15): 100.0 | GAEGPPGtPRRTGKG | T15; | 1488.6 | Yes |
| **ARHGEF5** | NP_005426.2 | 7984 | rho guanine nucleotide exchange factor 5 | **S11** | **0.29** | **0.5** | #N/A | GAsPPISAIEEFSIIPEAPMR | 6 | 1 | 1 | 0 | S3(Phospho) | S(3): 100.0; S(7): 0.0; S(13): 0.0 | EEAQRGAsPPISAIE | S3; | 2292.1 | Yes |
| **ARHGEF5** | NP_005426.2 | 7984 | rho guanine nucleotide exchange factor 5 | **S11** | 0.68 | 0.57 | #N/A | GAsPPISAIEEFSIIPEAPMR | 7 | 1 | 1 | 0 | S3(Phospho); R21(Label:13C(6)) | S(3): 100.0; S(7): 0.0; S(13): 0.0 | EEAQRGAsPPISAIE | S3; | 2298.1 | Yes |
| **ARHGEF5** | NP_005426.2 | 7984 | rho guanine nucleotide exchange factor 5 | **S450** | | **0.42** | #N/A | AEELSPAALsPSLEPIR | 2 | 2 | 1 | 0 | S10(Phospho) | S(5): 0.0; S(10): 100.0; S(12): 0.0 | ELSPAALsPSLEPIR | S10; | 1859.9 | Yes |
| **ARHGEF5** | NP_005426.2 | 7984 | rho guanine nucleotide exchange factor 5 | **S450** | | **0.43** | #N/A | AEELSPAALsPSLEPIR | 1 | 2 | 1 | 0 | S10(Phospho); R17(Label:13C(6)) | S(5): 0.0; S(10): 100.0; S(12): 0.0 | ELSPAALsPSLEPIR | S10; | 1865.9 | Yes |
| **ARHGEF7** | NP_003890.1 | 8874 | rho guanine nucleotide exchange factor 7 isoform a | **S340** | | | 0.85 | MsGFIYQGK | 2 | 5 | 1 | 0 | S2(Phospho); K9(Label:13C(6)15N(2)) | S(2): 100.0; Y(6): 0.0 | LSASPRMsGFIYQGK | S2; | 1118.5 | Yes |
| **ARHGEF7** | NP_003890.1 | 8874 | rho guanine nucleotide exchange factor 7 isoform a | **S516** | **2.9** | | 0.85 | KPsDEEFASR | 1 | 4 | 1 | 0 | S3(Phospho) | S(3): 100.0; S(9): 0.0 | RKPERKPsDEEFASR | S3; | 1245.5 | Yes |
| **ARHGEF7** | NP_003890.1 | 8874 | rho guanine nucleotide exchange factor 7 isoform a | **S340** | | 1.14 | 0.85 | MsGFIYQGKLPTTGMTITK | 3 | 4 | 1 | 1 | S2(Phospho) | S(2): 100.0; Y(6): 0.0; T(12): 0.0; T(13): 0.0; T(16): 0.0; T(18): 0.0 | LSASPRMsGFIYQGK | S2; | 2154.0 | Yes |
| **ARHGEF7** | NP_003890.1 | 8874 | rho guanine nucleotide exchange factor 7 isoform a | **S340** | | 1.14 | 0.85 | MsGFIYQGKLPTTGMTITK | 4 | 4 | 1 | 1 | S2(Phospho); K9(Label:13C(6)15N(2)); K19(Label:13C(6)15N(2)) | S(2): 100.0; Y(6): 0.0; T(12): 0.0; T(13): 0.0; T(16): 0.0; T(18): 0.0 | LSASPRMsGFIYQGK | S2; | 2170.1 | Yes |
| **ARHGEF7** | NP_003890.1 | 8874 | rho guanine nucleotide exchange factor 7 isoform a | **S498** | | 1.03 | 0.85 | EDLSKsPK | 2 | 4 | 1 | 1 | S6(Phospho) | S(4): 0.0; S(6): 100.0 | YKEDLSKsPKTMKKL | S6; | 983.4 | No |
| **ARID1A** | NP_006006.3 | 8289 | AT-rich interactive domain-containing protein 1A isoform a | **S772** | | | 0.57 | NPQMPQYSSPQPGSALsPR | 2 | 2 | 1 | 0 | S17(Phospho) | Y(7): 0.0; S(8): 0.0; S(9): 0.0; S(14): 1.3; S(17): 98.7 | PQPGSALsPRQPSGG | S17; | 2121.9 | Yes |
| **ARID1A** | NP_006006.3 | 8289 | AT-rich interactive domain-containing protein 1A isoform a | **S772** | | | 0.57 | NPQMPQYSSPQPGSALsPR | 2 | 2 | 1 | 0 | S17(Phospho); R19(Label:13C(6)) | Y(7): 0.0; S(8): 0.0; S(9): 0.0; S(14): 0.0; S(17): 100.0 | PQPGSALsPRQPSGG | S17; | 2128.0 | Yes |
| **ARID1B** | NP_059989.2 | 57492 | AT-rich interactive domain-containing protein 1B isoform 1 | **S1555** | | 1.73 | #N/A | MsPSKSPFLPSMK | 2 | 2 | 1 | 1 | S2(Phospho) | S(2): 100.0; S(4): 0.0; S(6): 0.0; S(11): 0.0 | RSLENRMsPSKSPFL | S2; | 1516.7 | Yes |
| **ARID1B** | NP_059989.2 | 57492 | AT-rich interactive domain-containing protein 1B isoform 1 | **S1555** | | | #N/A | MsPSKSPFLPSMK | 2 | 2 | 1 | 1 | S2(Phospho); K5(Label:13C(6)15N(2)); K13(Label:13C(6)15N(2)) | S(2): 100.0; S(4): 0.0; S(6): 0.0; S(11): 0.0 | RSLENRMsPSKSPFL | S2; | 1532.7 | Yes |
| **ARID1B** | NP_059989.2 | 57492 | AT-rich interactive domain-containing protein 1B isoform 1 | **S1555** | | 1.27 | #N/A | MsPSKSPFLPSMK | 1 | 2 | 1 | 1 | S2(Phospho); M12(Oxidation) | S(2): 98.1; S(4): 1.9; S(6): 0.0; S(11): 0.0 | RSLENRMsPSKSPFL | S2; | 1532.7 | Yes |
| **ARID4A** | NP_075377.2 | 5926 | AT-rich interactive domain-containing protein 4A isoform III | **S864** | | 0.99 | #N/A | ILGQSsPEKK | 3 | 3 | 1 | 1 | S6(Phospho); K9(Label:13C(6)15N(2)); K10(Label:13C(6)15N(2)) | S(5): 0.0; S(6): 100.0 | RKILGQSsPEKKIRI | S6; | 1182.6 | Yes |
| **ARID4A** | NP_075377.2 | 5926 | AT-rich interactive domain-containing protein 4A isoform III | **S864** | | | #N/A | ILGQSsPEKK | 1 | 3 | 1 | 1 | S6(Phospho) | S(5): 1.2; S(6): 98.8 | RKILGQSsPEKKIRI | S6; | 1166.6 | Yes |
| **ARID5B** | NP_001231567.1 | 84159 | AT-rich interactive domain-containing protein 5B isoform 2 | **S789** | | **0.04** | #N/A | AVsPLDPSKEVSGKEK | 1 | 2 | 1 | 2 | S3(Phospho); K9(Label:13C(6)15N(2)); K14(Label:13C(6)15N(2)); K16(Label:13C(6)15N(2)) | S(3): 100.0; S(8): 0.0; S(12): 0.0 | GKKARAVsPLDPSKE | S3; | 1774.9 | No |
| **ARL2** | NP_001186674.1 | 402 | ADP-ribosylation factor-like protein 2 isoform 2 | **S45** | | **3.53** | **2.44** | KFNGEDIDTIsPTLGFNIK | 2 | 2 | 1 | 1 | S11(Phospho) | T(9): 2.1; S(11): 97.9; T(13): 0.0 | GEDIDTIsPTLGFNI | S11; | 2189.1 | Yes |
| **ARL6IP4** | NP_001265309.1 | 51329 | ADP-ribosylation factor-like protein 6-interacting protein 4 isoform 7 | **S137** | 0.79 | | 0.99 | sAGEEEDGPVLTDEQK | 10 | 6 | 1 | 0 | S1(Phospho) | S(1): 100.0; T(12): 0.0 | SLDQWHRsAGEEEDG | S1; | 1783.7 | No |
| **ARL6IP4** | NP_001265309.1 | 51329 | ADP-ribosylation factor-like protein 6-interacting protein 4 isoform 7 | **S137** | | 0.78 | 0.99 | sAGEEEDGPVLTDEQK | 6 | 6 | 1 | 0 | S1(Phospho); K16(Label:13C(6)15N(2)) | S(1): 100.0; T(12): 0.0 | SLDQWHRsAGEEEDG | S1; | 1791.7 | No |
| **ARL6IP5** | NP_006398.1 | 10550 | PRA1 family protein 3 | **T160** | **0.48** | | **0.37** | tPMGIVLDALEQQEEGINR | 1 | 1 | 1 | 0 | T1(Phospho); M3(Oxidation) | T(1): 100.0 | EGIGLKRtPMGIVLD | T1; | 2209.0 | No |
| **ARMC10** | NP_001154485.1 | 83787 | armadillo repeat-containing protein 10 isoform f | **S45** | | 1.4 | 1.62 | sAEDLTDGSYDDVLNAEQLQK | 4 | 4 | 1 | 0 | S1(Phospho) | S(1): 100.0; T(6): 0.0; S(9): 0.0; Y(10): 0.0 | LGIRSSKsAEDLTDG | S1; | 2391.0 | Yes |
| **ARMC10** | NP_001154485.1 | 83787 | armadillo repeat-containing protein 10 isoform f | **S45** | | | 1.62 | sAEDLTDGSYDDVLNAEQLQK | 4 | 4 | 1 | 0 | S1(Phospho); K21(Label:13C(6)15N(2)) | S(1): 100.0; T(6): 0.0; S(9): 0.0; Y(10): 0.0 | LGIRSSKsAEDLTDG | S1; | 2399.0 | Yes |

| GeneSymbol | NP_Accession | Gene ID | ProteinDescription | PhosphoSite (Protein) | SCC-R / SCC-S R1 | SCC-R / SCC-S R2 | SCC-R / SCC-S Total Proteome | Sequence | # PSMs | # Proteins | # Protein Groups | # Missed Cleavages | Modifications | phosphoRS Site Probabilities | Phosphowindow | PhosphoSite (Peptide) | MH+ [Da] | PhosphositePlusEvidence |
|---|---|---|---|---|---|---|---|---|---|---|---|---|---|---|---|---|---|---|
| **ARMCX3** | NP_808817.1 | 51566 | armadillo repeat-containing X-linked protein 3 | **S110** | | | 0.66 | RAsPNSDDTVLSPQELQK | 1 | 1 | 1 | 1 | R1(Label:13C(6)); S3(Phospho); K18(Label:13C(6)15N(2)) | S(3): 100.0; S(6): 0.0; T(9): 0.0; S(12): 0.0 | RAVQKRAsPNSDDTV | S3; | 2079.0 | Yes |
| **ARMCX3** | NP_808817.1 | 51566 | armadillo repeat-containing X-linked protein 3 | **S61** | | 0.71 | 0.66 | YNDWsDDDDDSNESK | 1 | 1 | 1 | 0 | S5(Phospho); K15(Label:13C(6)15N(2)) | Y(1): 0.0; S(5): 100.0; S(11): 0.0; S(14): 0.0 | GARYNDWsDDDDDSN | S5; | 1892.6 | Yes |
| **ARMCX5** | NP_001161950.1 | 64860 | armadillo repeat-containing X-linked protein 5 | **S117; S122** | | | #N/A | VsTVTKsEVK | 1 | 1 | 1 | 1 | S2(Phospho); S7(Phospho); K10(Label:13C(6)15N(2)) | S(2): 100.0; T(3): 0.0; T(5): 1.7; S(7): 98.3 | AMPMSRVsTVTKSEV; RVSTVTKsEVKVVAV | S2; S7; | 1245.6 | No;No |
| **ARNT** | NP_001272964.1 | 405 | aryl hydrocarbon receptor nuclear translocator isoform 5 | **S68** | | 0.81 | #NQ | FARsDDEQSSADKER | 1 | 3 | 1 | 2 | S4(Phospho) | S(4): 100.0; S(9): 0.0; S(10): 0.0 | DKERFARsDDEQSSA | S4; | 1820.7 | No |
| **ARPC5L** | NP_112240.1 | 81873 | actin-related protein 2/3 complex subunit 5-like protein | **S64** | 0.51 | 0.72 | 0.98 | AFHAALRNsPVNTK | 3 | 1 | 1 | 1 | R7(Label:13C(6)); S9(Phospho); K14(Label:13C(6)15N(2)) | S(9): 100.0; T(13): 0.0 | FHAALRNsPVNTKNQ | S9; | 1619.8 | Yes |
| **ARPP19\|\|ENSA** | NP_006619.1\|\|NP_996927.1 | 10776\|\|2029 | cAMP-regulated phosphoprotein 19\|\|alpha-endosulfine isoform 4 | **S62\|\|S67** | | | #N/A | YFDsGDYNMAK | 4 | 8 | 2 | 0 | S4(Phospho) | Y(1): 0.0; S(4): 100.0; Y(7): 0.0 | KGQKYFDsGDYNMAK\|\|KGQKYFDsGDYNMAK | S4; | 1390.5 | Yes\|\|Yes |
| **ARPP19\|\|ENSA** | NP_006619.1\|\|NP_996927.1 | 10776\|\|2029 | cAMP-regulated phosphoprotein 19\|\|alpha-endosulfine isoform 4 | **S62\|\|S67** | | | #N/A | YFDsGDYNMAK | 4 | 8 | 2 | 0 | S4(Phospho); K11(Label:13C(6)15N(2)) | Y(1): 0.0; S(4): 100.0; Y(7): 0.0 | KGQKYFDsGDYNMAK\|\|KGQKYFDsGDYNMAK | S4; | 1398.5 | Yes\|\|Yes |
| **ARPP19\|\|ENSA** | NP_006619.1\|\|NP_996927.1 | 10776\|\|2029 | cAMP-regulated phosphoprotein 19\|\|alpha-endosulfine isoform 4 | **S62\|\|S67** | | | #N/A | YFDsGDYNMAK | 1 | 8 | 2 | 0 | S4(Phospho); M9(Oxidation); K11(Label:13C(6)15N(2)) | Y(1): 0.0; S(4): 100.0; Y(7): 0.0 | KGQKYFDsGDYNMAK\|\|KGQKYFDsGDYNMAK | S4; | 1414.5 | Yes\|\|Yes |
| **ARPP19\|\|ENSA** | NP_006619.1\|\|NP_996927.1 | 10776\|\|2029 | cAMP-regulated phosphoprotein 19\|\|alpha-endosulfine isoform 4 | **S62\|\|S67** | | | #N/A | YFDsGDYNMAK | 1 | 8 | 2 | 0 | S4(Phospho); M9(Oxidation) | Y(1): 0.0; S(4): 100.0; Y(7): 0.0 | KGQKYFDsGDYNMAK\|\|KGQKYFDsGDYNMAK | S4; | 1406.5 | Yes\|\|Yes |
| **ARRDC3** | NP_065852.1 | 57561 | arrestin domain-containing protein 3 | **T176** | | | #NQ | KEFTVFEHIDINTPSLLSPQAGtKEK | 1 | 1 | 1 | 2 | T23(Phospho); K24(Label:13C(6)15N(2)); K26(Label:13C(6)15N(2)) | T(4): 0.0; T(13): 0.0; S(15): 0.0; S(18): 3.3; T(23): 96.7 | LLSPQAGtKEKTLCC | T23; | 3025.5 | No |
| **ARVCF** | NP_001661.1 | 421 | armadillo repeat protein deleted in velo-cardio-facial syndrome | **T642** | | | #N/A | NFDtLDLPK | 1 | 1 | 1 | 0 | T4(Phospho); K9(Label:13C(6)15N(2)) | T(4): 100.0 | EMDRNFDtLDLPKRT | T4; | 1150.5 | Yes |
| **ASCC2** | NP_001229835.1 | 84164 | activating signal cointegrator 1 complex subunit 2 isoform 2 | **S637** | | 0.38 | 0.14 | HDSSTAVAGsPR | 1 | 2 | 1 | 0 | S10(Phospho); R12(Label:13C(6)) | S(3): 0.0; S(4): 0.0; T(5): 0.0; S(10): 100.0 | SSTAVAGsPRGHGQS | S10; | 1270.6 | No |
| **ASF1B** | NP_060624.1 | 55723 | histone chaperone ASF1B | **S198** | | | #NQ | GLGLPGCIPGLLPENsMDCI | 2 | 1 | 1 | 0 | C7(Carbamidomethyl); S16(Phospho); C19(Carbamidomethyl) | S(16): 100.0 | PGLLPENsMDCI_ | S16; | 2193.0 | Yes |
| **ASH2L** | NP_001269201.1 | 9070 | set1/Ash2 histone methyltransferase complex subunit ASH2 isoform d | **S484** | 0.91 | | 0.91 | RsPPWEP | 6 | 4 | 1 | 1 | S2(Phospho) | S(2): 100.0 | TEVDGRRsPPWEP_ | S2; | 948.4 | No |
| **ASH2L** | NP_001269201.1 | 9070 | set1/Ash2 histone methyltransferase complex subunit ASH2 isoform d | **S484** | 0.78 | | 0.91 | RsPPWEP | 2 | 4 | 1 | 1 | R1(Label:13C(6)); S2(Phospho) | S(2): 100.0 | TEVDGRRsPPWEP_ | S2; | 954.4 | No |
| **ASMTL** | NP_001166945.1 | 8623 | N-acetylserotonin O-methyltransferase-like protein isoform 3 | **S21** | 0.89 | 0.99 | 1.01 | VVLASAsPR | 3 | 2 | 1 | 0 | S7(Phospho) | S(5): 0.0; S(7): 100.0 | RVVLASAsPRRQEIL | S7; | 979.5 | Yes |
| **ASMTL** | NP_001166945.1 | 8623 | N-acetylserotonin O-methyltransferase-like protein isoform 3 | **S21** | 0.93 | 1.02 | 1.01 | VVLASAsPR | 2 | 2 | 1 | 0 | S7(Phospho); R9(Label:13C(6)) | S(5): 0.8; S(7): 99.2 | RVVLASAsPRRQEIL | S7; | 985.5 | Yes |
| **ASPSCR1** | NP_076988.1 | 79058 | tether containing UBX domain for GLUT4 isoform 1 | **S500** | | 1.27 | 1.27 | AAGsPSPLPAPDPAPK | 3 | 2 | 1 | 0 | S4(Phospho) | S(4): 100.0; S(6): 0.0 | YMSRAAGsPSPLPAP | S4; | 1552.7 | Yes |
| **ATAD3A** | NP_001164006.1 | 55210 | ATPase family AAA domain-containing protein 3A isoform 2 | **S321** | 0.75 | | 0.70 | LLSRPQDALEGVVLsPSLEAR | 2 | 3 | 1 | 0 | S15(Phospho) | S(3): 0.0; S(15): 100.0; S(17): 0.0 | ALEGVVLsPSLEARV | S15; | 2330.2 | No |
| **ATAD5** | NP_079133.3 | 79915 | ATPase family AAA domain-containing protein 5 | **S1625; T1632** | | | #N/A | KsIPCPPKtTAGK | 1 | 1 | 1 | 2 | S2(Phospho); C5(Carbamidomethyl); T9(Phospho) | S(2): 97.2; T(9): 97.2; T(10): 5.5 | NNPETKKsIPCPPKT;SIPCPPKtTAGKKCS | S2; T9; | 1544.7 | No;No |
| **ATF2** | NP_001243020.1 | 1386 | cyclic AMP-dependent transcription factor ATF-2 isoform 2 | **S94** | 1.66 | 1.73 | 0.38 | MPLDLsPLATPIIR | 6 | 5 | 1 | 0 | S6(Phospho) | S(6): 100.0; T(10): 0.0 | KKMPLDLsPLATPII | S6; | 1616.8 | No |
| **ATF2** | NP_001243020.1 | 1386 | cyclic AMP-dependent transcription factor ATF-2 isoform 2 | **S94** | 1.67 | 1.8 | 0.38 | KMPLDLsPLATPIIR | 4 | 5 | 1 | 1 | S7(Phospho) | S(7): 100.0; T(11): 0.0 | KKMPLDLsPLATPII | S7; | 1744.9 | No |
| **ATF2** | NP_001243020.1 | 1386 | cyclic AMP-dependent transcription factor ATF-2 isoform 2 | **S94** | 1.76 | 1.86 | 0.38 | KMPLDLsPLATPIIR | 2 | 5 | 1 | 1 | K1(Label:13C(6)15N(2)); S7(Phospho); R15(Label:13C(6)) | S(7): 100.0; T(11): 0.0 | KKMPLDLsPLATPII | S7; | 1759.0 | No |
| **ATF2** | NP_001243020.1 | 1386 | cyclic AMP-dependent transcription factor ATF-2 isoform 2 | **S94** | 1.74 | 1.78 | 0.38 | MPLDLsPLATPIIR | 2 | 5 | 1 | 0 | S6(Phospho); R14(Label:13C(6)) | S(6): 100.0; T(10): 0.0 | KKMPLDLsPLATPII | S6; | 1622.9 | No |
| **ATF2** | NP_001243020.1 | 1386 | cyclic AMP-dependent transcription factor ATF-2 isoform 2 | **T53** | | 4.17 | 0.38 | NDSVIVADQTPtPTR | 2 | 3 | 1 | 0 | T12(Phospho) | S(3): 0.0; T(10): 0.0; T(12): 100.0; T(14): 0.0 | IVADQTPtPTRFLKN | T12; | 1693.8 | No |
| **ATF2** | NP_001243020.1 | 1386 | cyclic AMP-dependent transcription factor ATF-2 isoform 2 | **T53** | | 7.08 | 0.38 | FGPARNDSVIVADQTPtPTR | 2 | 3 | 1 | 1 | T17(Phospho) | S(8): 0.0; T(15): 0.0; T(17): 98.4; T(19): 1.6 | IVADQTPtPTRFLKN | T17; | 2222.1 | No |
| **ATF2** | NP_001243020.1 | 1386 | cyclic AMP-dependent transcription factor ATF-2 isoform 2 | **T51** | | | 0.38 | FGPARNDSVIVADQtPTPTR | 1 | 3 | 1 | 1 | T15(Phospho); T17(Phospho) | S(8): 0.0; T(15): 100.0; T(17): 50.0; T(19): 50.0 | SVIVADQtPTPTRFL | T15; | 2302.0 | No |
| **ATF2** | NP_001243020.1 | 1386 | cyclic AMP-dependent transcription factor ATF-2 isoform 2 | **T51; T53** | | | 0.38 | NDSVIVADQtPtPTR | 1 | 3 | 1 | 0 | T10(Phospho); T12(Phospho) | S(3): 0.0; T(10): 100.0; T(12): 98.5; T(14): 1.5 | SVIVADQtPTPTRFL;IVADQTPtPTRFLKN | T10; T12; | 1773.7 | No;No |
| **ATF7** | NP_006847.1 | 11016 | cyclic AMP-dependent transcription factor ATF-7 isoform 2 | **S171** | 5.3 | | 0.86 | EVTPKPVLISTPTPTIVRPGSLPLHLGYDPLHPTLPsPTSVITQAPPSNR | 2 | 1 | 1 | 0 | Y28(Phospho) | T(3): 0.0; S(10): 0.0; T(11): 0.0; T(13): 0.0; T(15): 0.0; S(21): 0.0; Y(28): 0.0; T(34): 0.0; S(37): 94.7; T(39): 5.0; S(40): 0.3; T(43): 0.0; S(48): 0.0 | PLHPTLPsPTSVITQ | S37; | 5368.9 | No |
| **ATF7IP** | NP_001273443.1 | 55729 | activating transcription factor 7-interacting protein 1 isoform 3 | **S672** | 1.39 | | 0.63 | RHEHPPNPPVsPGK | 2 | 4 | 1 | 1 | S11(Phospho) | S(11): 100.0 | HPPNPPVsPGKTVND | S11; | 1628.8 | No |
| **ATF7IP** | NP_001273443.1 | 55729 | activating transcription factor 7-interacting protein 1 isoform 3 | **S672** | | | 0.63 | RHEHPPNPPVsPGK | 2 | 4 | 1 | 1 | R1(Label:13C(6)); S11(Phospho); K14(Label:13C(6)15N(2)) | S(11): 100.0 | HPPNPPVsPGKTVND | S11; | 1642.8 | No |
| **ATF7IP** | NP_001273443.1 | 55729 | activating transcription factor 7-interacting protein 1 isoform 3 | **S445** | | 0.81 | 0.63 | LAPsEDELTCFSK | 2 | 4 | 1 | 0 | S4(Phospho); C10(Carbamidomethyl); K13(Label:13C(6)15N(2)) | S(4): 100.0; T(9): 0.0; S(12): 0.0 | ILEKLAPsEDELTCF | S4; | 1584.7 | Yes |
| **ATF7IP** | NP_001273443.1 | 55729 | activating transcription factor 7-interacting protein 1 isoform 3 | **S445** | | 0.83 | 0.63 | LAPsEDELTCFSK | 2 | 4 | 1 | 0 | S4(Phospho); C10(Carbamidomethyl) | S(4): 100.0; T(9): 0.0; S(12): 0.0 | ILEKLAPsEDELTCF | S4; | 1576.7 | Yes |
| **ATF7IP** | NP_001273443.1 | 55729 | activating transcription factor 7-interacting protein 1 isoform 3 | **S672** | | 1.05 | 0.63 | HEHPPNPPVsPGKTVNDVNSNNNMSYR | 1 | 4 | 1 | 1 | S10(Phospho) | S(10): 100.0; T(14): 0.0; S(20): 0.0; S(25): 0.0; Y(26): 0.0 | HPPNPPVsPGKTVND | S10; | 3081.4 | No |
| **ATF7IP** | NP_001273443.1 | 55729 | activating transcription factor 7-interacting protein 1 isoform 3 | **S672** | | 1.08 | 0.63 | HEHPPNPPVsPGKTVNDVNSNNNMSYR | 1 | 4 | 1 | 1 | S10(Phospho); K13(Label:13C(6)15N(2)); R27(Label:13C(6)) | S(10): 100.0; T(14): 0.0; S(20): 0.0; S(25): 0.0; Y(26): 0.0 | HPPNPPVsPGKTVND | S10; | 3095.4 | No |
| **ATG2B** | NP_060506.5 | 55102 | autophagy-related protein 2 homolog B | **S255** | | 0.67 | #NQ | LsPSWNPK | 1 | 1 | 1 | 0 | S2(Phospho) | S(2): 100.0; S(4): 0.0 | VETEPKLsPSWNPKI | S2; | 1008.5 | Yes |
| **ATG4C** | NP_116241.2 | 84938 | cysteine protease ATG4C | **S451** | | 0.64 | #NQ | RFsTEEFVLL | 2 | 1 | 1 | 1 | S3(Phospho) | S(3): 99.3; T(4): 0.7 | KKQLKRFsTEEFVLL | S3; | 1320.6 | Yes |
| **ATG4C** | NP_116241.2 | 84938 | cysteine protease ATG4C | **S451** | | | #NQ | RFsTEEFVLL | 1 | 1 | 1 | 1 | R1(Label:13C(6)); S3(Phospho) | S(3): 98.7; T(4): 1.3 | KKQLKRFsTEEFVLL | S3; | 1326.6 | Yes |
| **ATG9A** | NP_076990.4 | 79065 | autophagy-related protein 9A | **S18** | | | #N/A | LEASYSDsPPGEEDLLVHVAEGSK | 3 | 1 | 1 | 0 | S8(Phospho) | S(4): 0.1; Y(5): 0.1; S(6): 0.1; S(8): 99.8; S(23): 0.0 | LEASYSDsPPGEEDL | S8; | 2609.2 | Yes |
| **ATP10A** | NP_077816.1 | 57194 | probable phospholipid-transporting ATPase VA | **S515** | 0.86 | | #N/A | AsMLSK | 1 | 1 | 1 | 0 | S2(Phospho); M3(Oxidation) | S(2): 100.0; S(5): 0.0 | RAEAKRAsMLSKHTA | S2; | 732.3 | Yes |
| **ATP13A3** | NP_078800.3 | 79572 | probable cation-transporting ATPase 13A3 | **S817** | | 0.63 | #N/A | LVHDsLEDLQMTR | 1 | 1 | 1 | 0 | S5(Phospho) | S(5): 100.0; T(12): 0.0 | PVKLVHDsLEDLQMT | S5; | 1636.7 | Yes |
| **ATP13A3** | NP_078800.3 | 79572 | probable cation-transporting ATPase 13A3 | **S817** | | 0.65 | #N/A | LVHDsLEDLQMTR | 1 | 1 | 1 | 0 | S5(Phospho); R13(Label:13C(6)) | S(5): 100.0; T(12): 0.0 | PVKLVHDsLEDLQMT | S5; | 1642.8 | Yes |
| **ATP2A2** | NP_001672.1 | 488 | sarcoplasmic/endoplasmic reticulum calcium ATPase 2 isoform a | **S663** | | | 0.43 | AFTGREFDELNPsAQR | 1 | 2 | 1 | 1 | S13(Phospho) | T(3): 0.0; S(13): 100.0 | EFDELNPsAQRDACL | S13; | 1917.9 | Yes |
| **ATP2B1** | NP_001673.2 | 490 | plasma membrane calcium-transporting ATPase 1 isoform 1b | **S1140** | 1.3 | | 2.39 | SsIHNFMTHPEFR | 2 | 1 | 1 | 0 | S2(Phospho); R13(Label:13C(6)) | S(1): 1.2; S(2): 98.8; T(8): 0.0 | EKPESRSsIHNFMTH | S2; | 1688.7 | No |
| **ATP8** | YP_003024030.1 | 4509 | ATP synthase F0 subunit 8 | **T56** | | | #NQ | NYNKPWEPKWtK | 1 | 1 | 1 | 1 | K4(Label:13C(6)15N(2)); T11(Phospho) | Y(2): 0.0; T(11): 100.0 | KPWEPKWtKICSLHS | T11; | 1678.8 | No |
| **ATP8A2** | NP_057613.4 | 51761 | probable phospholipid-transporting ATPase IB | **S24** | | | #NQ | IRSsVGPVR | 1 | 1 | 1 | 1 | S4(Phospho) | S(3): 1.0; S(4): 99.0 | RRSRIRSsVGPVRSS | S4; | 1050.5 | Yes |
| **ATRIP** | NP_001257951.1 | 84126 | ATR-interacting protein isoform 3 | **S97** | 0.69 | 0.76 | #N/A | LAAPSVSHVsPR | 14 | 4 | 1 | 0 | S10(Phospho) | S(5): 0.0; S(7): 0.0; S(10): 100.0 | APSVSHVsPRKNPSV | S10; | 1300.6 | No |
| **ATRIP** | NP_001257951.1 | 84126 | ATR-interacting protein isoform 3 | **S97** | 0.74 | 0.77 | #N/A | LAAPSVSHVsPR | 6 | 4 | 1 | 0 | S10(Phospho); R12(Label:13C(6)) | S(5): 0.0; S(7): 0.0; S(10): 100.0 | APSVSHVsPRKNPSV | S10; | 1306.7 | No |
| **ATRIP** | NP_001257951.1 | 84126 | ATR-interacting protein isoform 3 | **T589** | | | #N/A | TDQQRRtVR | 3 | 4 | 1 | 2 | R6(Label:13C(6)); T7(Phospho); R9(Label:13C(6)) | T(1): 0.0; T(7): 100.0 | RTDQQRRtVRCLRDT | T7; | 1251.6 | No |
| **ATRX** | NP_612114.2 | 546 | transcriptional regulator ATRX isoform 2 | **S1489** | | 0.94 | 0.69 | EVIEIEDAsPTKCPITTK | 4 | 2 | 1 | 1 | S9(Phospho); K12(Label:13C(6)15N(2)); C13(Carbamidomethyl); K18(Label:13C(6)15N(2)) | S(9): 100.0; T(11): 0.0; T(16): 0.0; T(17): 0.0 | VIEIEDAsPTKCPIT | S9; | 2127.0 | No |
| **ATRX** | NP_612114.2 | 546 | transcriptional regulator ATRX isoform 2 | **T1951; S1953** | | | 0.69 | AtSsSNPSSPAPDWYKDFVTDADAEVLEHSGK | 2 | 2 | 1 | 1 | S3(Phospho); S4(Phospho) | T(2): 96.4; S(3): 6.7; S(4): 86.8; S(5): 3.3; S(8): 3.3; S(9): 3.3; Y(15): 0.1; T(20): 0.0; S(30): 0.0 | KLEESKAtSSSNPSS;EESKATSsSNPSSPA | T2; S4; | 3568.5 | No;No |
| **ATRX** | NP_612114.2 | 546 | transcriptional regulator ATRX isoform 2 | **S1489** | | 0.88 | 0.69 | EVIEIEDAsPTKCPITTK | 4 | 2 | 1 | 1 | S9(Phospho); C13(Carbamidomethyl) | S(9): 98.6; T(11): 1.4; T(16): 0.0; T(17): 0.0 | VIEIEDAsPTKCPIT | S9; | 2111.0 | No |

| GeneSymbol | NP_Accession | Gene ID | ProteinDescription | PhosphoSite (Protein) | SCC-R / SCC-S R1 | SCC-R / SCC-S R2 | SCC-R / SCC-S Total Proteome | Sequence | # PSMs | # Proteins | # Protein Groups | # Missed Cleavages | Modifications | phosphoRS Site Probabilities | Phosphowindow | PhosphoSite (Peptide) | MH+ [Da] | PhosphositePlusEvidence |
|---|---|---|---|---|---|---|---|---|---|---|---|---|---|---|---|---|---|---|
| **ATRX** | NP_612114.2 | 546 | transcriptional regulator ATRX isoform 2 | **T1951** | | | 0.69 | AtSSSNPSSPAPDWYKDFVTDADAEVLEHSGK | 1 | 2 | 1 | 1 | S9(Phospho); Y15(Phospho); K16(Label:13C(6)15N(2)); K32(Label:13C(6)15N(2)) | T(2): 99.9; S(3): 1.8; S(4): 47.5; S(5): 47.5; S(8): 1.7; S(9): 1.7; Y(15): 0.1; T(20): 0.0; S(30): 0.0 | KLEESKAtSSSNPSS | T2; | 3584.5 | No |
| **ATRX** | NP_612114.2 | 546 | transcriptional regulator ATRX isoform 2 | **S1489** | | 1.07 | 0.69 | LREVIEIEDAsPTKCPITTK | 1 | 2 | 1 | 2 | R2(Label:13C(6)); S11(Phospho); K14(Label:13C(6)15N(2)); C15(Carbamidomethyl); K20(Label:13C(6)15N(2)) | S(11): 100.0; T(13): 0.0; T(18): 0.0; T(19): 0.0 | VIEIEDAsPTKCPIT | S11; | 2402.2 | No |
| **ATXN2** | NP_002964.3 | 6311 | ataxin-2 | **S624** | 1.44 | 1.32 | 0.82 | MSSEGPPRMsPK | 4 | 1 | 1 | 1 | S10(Phospho) | S(2): 0.0; S(3): 0.0; S(10): 100.0 | SEGPPRMsPKAQRHP | S10; | 1383.6 | Yes |
| **ATXN2** | NP_002964.3 | 6311 | ataxin-2 | **S728** | 1.43 | **1.95** | 0.82 | QNSIGNTPSGPVLASPQAGIIPTEAVAMPIPAAsPTPASPASNR | 6 | 1 | 1 | 0 | S34(Phospho) | S(3): 0.0; T(7): 0.0; S(9): 0.0; S(15): 0.0; T(23): 0.0; S(34): 100.0; T(36): 0.0; S(39): 0.0; S(42): 0.0 | AMPIPAAsPTPASPA | S34; | 4315.1 | Yes |
| **ATXN2** | NP_002964.3 | 6311 | ataxin-2 | **S624** | | | 0.82 | MSSEGPPRMsPK | 1 | 1 | 1 | 1 | R8(Label:13C(6)); S10(Phospho); K12(Label:13C(6)15N(2)) | S(2): 0.0; S(3): 0.0; S(10): 100.0 | SEGPPRMsPKAQRHP | S10; | 1397.6 | Yes |
| **ATXN2** | NP_002964.3 | 6311 | ataxin-2 | **T741** | | 0.86 | 0.82 | AVtPSSEAKDSR | 4 | 1 | 1 | 1 | T3(Phospho) | T(3): 100.0; S(5): 0.0; S(6): 0.0; S(11): 0.0 | PASNRAVtPSSEAKD | T3; | 1327.6 | Yes |
| **ATXN2** | NP_002964.3 | 6311 | ataxin-2 | **T741** | | 0.89 | 0.82 | AVtPSSEAKDSR | 2 | 1 | 1 | 1 | T3(Phospho); K9(Label:13C(6)15N(2)); R12(Label:13C(6)) | T(3): 100.0; S(5): 0.0; S(6): 0.0; S(11): 0.0 | PASNRAVtPSSEAKD | T3; | 1341.6 | Yes |
| **ATXN2L** | NP_009176.2 | 11273 | ataxin-2-like protein isoform A | **S559** | 1.5 | **2.08** | **2.15** | LQPSSsPENSLDPFPPR | 34 | 6 | 1 | 0 | S6(Phospho) | S(4): 0.0; S(5): 0.0; S(6): 100.0; S(10): 0.0 | FKLQPSSsPENSLDP | S6; | 1947.9 | Yes |
| **ATXN2L** | NP_009176.2 | 11273 | ataxin-2-like protein isoform A | **S409** | | | **2.15** | GINGGPSRMsPK | 2 | 6 | 1 | 1 | S10(Phospho) | S(7): 0.0; S(10): 100.0 | NGGPSRMsPKAQRPL | S10; | 1280.6 | Yes |
| **ATXN2L** | NP_009176.2 | 11273 | ataxin-2-like protein isoform A | **S559** | | | **2.15** | LQPSSsPENSLDPFPPR | 10 | 6 | 1 | 0 | S6(Phospho); R17(Label:13C(6)) | S(4): 0.0; S(5): 0.0; S(6): 100.0; S(10): 0.0 | FKLQPSSsPENSLDP | S6; | 1953.9 | Yes |
| **ATXN2L** | NP_009176.2 | 11273 | ataxin-2-like protein isoform A | **S409** | | | **2.15** | GINGGPSRMsPK | 1 | 6 | 1 | 1 | R8(Label:13C(6)); S10(Phospho); K12(Label:13C(6)15N(2)) | S(7): 1.0; S(10): 99.0 | NGGPSRMsPKAQRPL | S10; | 1294.6 | Yes |
| **ATXN2L** | NP_009176.2 | 11273 | ataxin-2-like protein isoform A | **S111** | | 1.56 | **2.15** | GPPQsPVFEGVYNNSR | 5 | 6 | 1 | 0 | S5(Phospho) | S(5): 100.0; Y(12): 0.0; S(15): 0.0 | TGKGPPQsPVFEGVY | S5; | 1827.8 | Yes |
| **ATXN2L** | NP_009176.2 | 11273 | ataxin-2-like protein isoform A | **S111** | | **4.14** | **2.15** | GQSTGKGPPQsPVFEGVYNNSR | 1 | 6 | 1 | 1 | S11(Phospho) | S(3): 0.0; T(4): 0.0; S(11): 100.0; Y(18): 0.0; S(21): 0.0 | TGKGPPQsPVFEGVY | S11; | 2386.1 | Yes |
| **ATXN2L** | NP_009176.2 | 11273 | ataxin-2-like protein isoform A | **S684** | | 1.66 | **2.15** | STSTPTsPGPR | 4 | 6 | 1 | 0 | S7(Phospho) | S(1): 0.0; T(2): 0.0; S(3): 0.0; T(4): 0.0; T(6): 0.0; S(7): 100.0 | KSTSTPTsPGPRTHS | S7; | 1167.5 | Yes |
| **ATXN2L** | NP_009176.2 | 11273 | ataxin-2-like protein isoform A | **S339** | | 1.34 | **2.15** | QGSGREsPSLASR | 4 | 6 | 1 | 1 | S7(Phospho) | S(3): 0.0; S(7): 100.0; S(9): 0.0; S(12): 0.0 | RQGSGREsPSLASRE | S7; | 1411.6 | Yes |
| **ATXN2L** | NP_009176.2 | 11273 | ataxin-2-like protein isoform A | **S594** | | 1.25 | **2.15** | EVDGLLTSEPMGsPVSSK | 4 | 6 | 1 | 0 | S13(Phospho); K18(Label:13C(6)15N(2)) | T(7): 0.0; S(8): 0.0; S(13): 100.0; S(16): 0.0; S(17): 0.0 | LTSEPMGsPVSSKTE | S13; | 1920.9 | Yes |
| **ATXN2L** | NP_009176.2 | 11273 | ataxin-2-like protein isoform A | **S594** | | 1.32 | **2.15** | EVDGLLTSEPMGsPVSSK | 4 | 6 | 1 | 0 | S13(Phospho) | T(7): 0.0; S(8): 0.0; S(13): 98.8; S(16): 1.1; S(17): 0.0 | LTSEPMGsPVSSKTE | S13; | 1912.9 | Yes |
| **ATXN2L** | NP_009176.2 | 11273 | ataxin-2-like protein isoform A | **S594** | | 1.28 | **2.15** | EKEVDGLLTSEPMGsPVSSK | 2 | 6 | 1 | 1 | S15(Phospho) | T(9): 0.0; S(10): 0.0; S(15): 97.2; S(18): 1.4; S(19): 1.4 | LTSEPMGsPVSSKTE | S15; | 2170.0 | Yes |
| **ATXN2L** | NP_009176.2 | 11273 | ataxin-2-like protein isoform A | **S594** | | | **2.15** | EKEVDGLLTSEPMGsPVSSK | 2 | 6 | 1 | 1 | K2(Label:13C(6)15N(2)); S15(Phospho); K20(Label:13C(6)15N(2)) | T(9): 0.0; S(10): 0.0; S(15): 100.0; S(18): 0.0; S(19): 0.0 | LTSEPMGsPVSSKTE | S15; | 2186.0 | Yes |
| **ATXN2L** | NP_009176.2 | 11273 | ataxin-2-like protein isoform A | **S111** | | 1.61 | **2.15** | GPPQsPVFEGVYNNSR | 3 | 6 | 1 | 0 | S5(Phospho); R16(Label:13C(6)) | S(5): 100.0; Y(12): 0.0; S(15): 0.0 | TGKGPPQsPVFEGVY | S5; | 1833.8 | Yes |
| **ATXN2L** | NP_009176.2 | 11273 | ataxin-2-like protein isoform A | **S684** | | | **2.15** | STSTPTsPGPR | 1 | 6 | 1 | 0 | S7(Phospho); R11(Label:13C(6)) | S(1): 0.0; T(2): 0.0; S(3): 0.0; T(4): 0.0; T(6): 0.0; S(7): 100.0 | KSTSTPTsPGPRTHS | S7; | 1173.5 | Yes |
| **ATXN2L** | NP_009176.2 | 11273 | ataxin-2-like protein isoform A | **S306** | | 0.65 | **2.15** | EIESsPQYR | 1 | 6 | 1 | 0 | S5(Phospho); R9(Label:13C(6)) | S(4): 0.0; S(5): 100.0; Y(8): 0.0 | LAREIESsPQYRLRI | S5; | 1194.5 | Yes |
| **ATXN7L3** | NP_001092303.1 | 56970 | ataxin-7-like protein 3 isoform b | **S281** | | | #N/A | LQWDGSSDLsPSDSGSSK | 2 | 2 | 1 | 0 | S10(Phospho); K18(Label:13C(6)15N(2)) | S(6): 0.0; S(7): 0.0; S(10): 100.0; S(12): 0.0; S(14): 0.0; S(16): 0.0; S(17): 0.0 | WDGSSDLsPSDSGSS | S10; | 1940.8 | Yes |
| **ATXN7L3** | NP_001092303.1 | 56970 | ataxin-7-like protein 3 isoform b | **S281** | | | #N/A | LQWDGSSDLsPSDSGSSK | 1 | 2 | 1 | 0 | S10(Phospho) | S(6): 0.0; S(7): 0.0; S(10): 100.0; S(12): 0.0; S(14): 0.0; S(16): 0.0; S(17): 0.0 | WDGSSDLsPSDSGSS | S10; | 1932.8 | Yes |
| **ATXN7L3B** | NP_001129734.1 | 552889 | putative ataxin-7-like protein 3B | **S92** | 1.94 | | #NQ | GACRLPLCSLPGEPGNGPDQQLQRsPPEFQ | 3 | 1 | 1 | 2 | C3(Carbamidomethyl); C8(Carbamidomethyl); S25(Phospho) | S(9): 0.0; S(25): 100.0 | PDQQLQRsPPEFQ_ | S25; | 3385.5 | Yes |
| **ATXN7L3B** | NP_001129734.1 | 552889 | putative ataxin-7-like protein 3B | **S92** | 1.83 | 1.71 | #NQ | LPLCSLPGEPGNGPDQQLQRsPPEFQ | 3 | 1 | 1 | 1 | C4(Carbamidomethyl); S21(Phospho) | S(5): 0.0; S(21): 100.0 | PDQQLQRsPPEFQ_ | S21; | 2941.4 | Yes |
| **ATXN7L3B** | NP_001129734.1 | 552889 | putative ataxin-7-like protein 3B | **S92** | | | #NQ | GACRLPLCSLPGEPGNGPDQQLQRsPPEFQ | 1 | 1 | 1 | 2 | C3(Carbamidomethyl); R4(Label:13C(6)); C8(Carbamidomethyl); R24(Label:13C(6)); S25(Phospho) | S(9): 0.0; S(25): 100.0 | PDQQLQRsPPEFQ_ | S25; | 3397.6 | Yes |
| **AXL** | NP_001690.2 | 558 | tyrosine-protein kinase receptor UFO isoform 2 precursor | **S603** | | **16.08** | **11.15** | EsFPAPVVILPFMK | 4 | 3 | 1 | 0 | S2(Phospho) | S(2): 100.0 | FQGSEREsFPAPVVI | S2; | 1654.8 | No |
| **B3GNTL1** | NP_001009905.1 | 146712 | UDP-GlcNAc:betaGal beta-1,3-N-acetylglucosaminyltransferase-like protein 1 | **S62** | | | #N/A | DKsGAIIEK | 3 | 1 | 1 | 1 | K2(Label:13C(6)15N(2)); S3(Phospho); K9(Label:13C(6)15N(2)) | S(3): 100.0 | NDASKDKsGAIIEKW | S3; | 1056.5 | No |
| **BABAM1** | NP_001275686.1 | 29086 | BRISC and BRCA1-A complex member 1 isoform 2 | **S29** | 0.68 | | 1.17 | TRsNPEGAEDR | 1 | 2 | 1 | 1 | R2(Label:13C(6)); S3(Phospho); R11(Label:13C(6)) | T(1): 0.0; S(3): 100.0 | EPRPRTRsNPEGAED | S3; | 1323.6 | Yes |
| **BACH1** | NP_996749.1 | 571 | transcription regulator protein BACH1 | **S445** | **8.25** | **9.62** | #NQ | ISEsPEPGQR | 3 | 1 | 1 | 0 | S4(Phospho) | S(2): 0.0; S(4): 100.0 | LGIRISEsPEPGQRT | S4; | 1179.5 | Yes |
| **BAD** | NP_116784.1 | 572 | bcl2 antagonist of cell death | **S134** | 1.8 | 1.67 | **2.54** | sAGTATQMR | 4 | 1 | 1 | 0 | S1(Phospho) | S(1): 100.0; T(4): 0.0; T(6): 0.0 | KGLPRPKsAGTATQM | S1; | 1002.4 | Yes |
| **BAD** | NP_116784.1 | 572 | bcl2 antagonist of cell death | **S134** | | | **2.54** | sAGTATQMR | 1 | 1 | 1 | 0 | S1(Phospho); R9(Label:13C(6)) | S(1): 100.0; T(4): 0.0; T(6): 0.0 | KGLPRPKsAGTATQM | S1; | 1008.4 | Yes |
| **BAD** | NP_116784.1 | 572 | bcl2 antagonist of cell death | **S118** | | 1.09 | **2.54** | RMsDEFVDSFKK | 2 | 1 | 1 | 2 | S3(Phospho) | S(3): 100.0; S(9): 0.0 | GRELRRMsDEFVDSF | S3; | 1568.7 | Yes |
| **BAD** | NP_116784.1 | 572 | bcl2 antagonist of cell death | **S99** | | 0.91 | **2.54** | sAPPNLWAAQR | 2 | 1 | 1 | 0 | S1(Phospho) | S(1): 100.0 | PFRGRSRsAPPNLWA | S1; | 1290.6 | Yes |
| **BAD** | NP_116784.1 | 572 | bcl2 antagonist of cell death | **S118** | | | **2.54** | RMsDEFVDSFKK | 1 | 1 | 1 | 2 | R1(Label:13C(6)); S3(Phospho); K11(Label:13C(6)15N(2)); K12(Label:13C(6)15N(2)) | S(3): 100.0; S(9): 0.0 | GRELRRMsDEFVDSF | S3; | 1590.7 | Yes |
| **BAG3** | NP_004272.2 | 9531 | BAG family molecular chaperone regulator 3 | **S377** | **2.97** | **2.73** | 0.61 | VPPAPVPCPPPsPGPSAVPSSPK | 18 | 1 | 1 | 0 | C8(Carbamidomethyl); S12(Phospho) | S(12): 100.0; S(16): 0.0; S(20): 0.0; S(21): 0.0 | PVPCPPPsPGPSAVP | S12; | 2299.1 | Yes |
| **BAG3** | NP_004272.2 | 9531 | BAG family molecular chaperone regulator 3 | **S264** | 0.68 | 0.68 | 0.61 | AAsPFR | 5 | 1 | 1 | 0 | S3(Phospho) | S(3): 100.0 | PRPLRAAsPFRSSVQ | S3; | 728.3 | Yes |
| **BAG3** | NP_004272.2 | 9531 | BAG family molecular chaperone regulator 3 | **S289** | 0.78 | | 0.61 | SSTPLHsPSPIR | 1 | 1 | 1 | 0 | T3(Phospho) | S(1): 0.0; S(2): 0.0; T(3): 1.5; S(7): 98.5; S(9): 0.0 | RSSTPLHsPSPIRVH | S7; | 1358.6 | Yes |
| **BAG3** | NP_004272.2 | 9531 | BAG family molecular chaperone regulator 3 | **S377** | **3.94** | | 0.61 | VPPAPVPCPPPsPGPSAVPSSPK | 8 | 1 | 1 | 0 | C8(Carbamidomethyl); S12(Phospho); K23(Label:13C(6)15N(2)) | S(12): 100.0; S(16): 0.0; S(20): 0.0; S(21): 0.0 | PVPCPPPsPGPSAVP | S12; | 2307.1 | Yes |
| **BAG3** | NP_004272.2 | 9531 | BAG family molecular chaperone regulator 3 | **T285; S289** | | **2.65** | 0.61 | SStPLHsPSPIR | 2 | 1 | 1 | 0 | S2(Phospho); S7(Phospho) | S(1): 0.0; S(2): 0.0; T(3): 100.0; S(7): 100.0; S(9): 0.0 | GSPARSStPLHSPSP;RSSTPLHsPSPIRVH | T3; S7; | 1438.6 | Yes;Yes |
| **BAG3** | NP_004272.2 | 9531 | BAG family molecular chaperone regulator 3 | **S377; S386** | | 1.17 | 0.61 | VPPAPVPCPPPsPGPSAVPSsPK | 1 | 1 | 1 | 0 | C8(Carbamidomethyl); S12(Phospho); S16(Phospho) | S(12): 100.0; S(16): 0.0; S(20): 2.0; S(21): 98.0 | PVPCPPPsPGPSAVP;GPSAVPSsPKSVATE | S12; S21; | 2379.1 | Yes;Yes |
| **BAG3** | NP_004272.2 | 9531 | BAG family molecular chaperone regulator 3 | **T285; S289** | | | 0.61 | SStPLHsPSPIR | 1 | 1 | 1 | 0 | S2(Phospho); S7(Phospho); R12(Label:13C(6)) | S(1): 0.0; S(2): 0.0; T(3): 100.0; S(7): 100.0; S(9): 0.0 | GSPARSStPLHSPSP;RSSTPLHsPSPIRVH | T3; S7; | 1444.6 | Yes;Yes |
| **BAG6** | NP_001092004.1 | 7917 | large proline-rich protein BAG6 isoform b | **S1075** | | | 1.03 | AAGARPLTsPESLSR | 1 | 2 | 1 | 0 | T8(Phospho) | T(8): 1.3; S(9): 98.7; S(12): 0.0; S(14): 0.0 | AGARPLTsPESLSRD | S9; | 1592.8 | No |
| **BAG6** | NP_001092004.1 | 7917 | large proline-rich protein BAG6 isoform b | **S113** | 1.32 | 1.37 | 1.03 | APPQTHLPSGASSGTGSASATHGGGsPPGTR | 7 | 4 | 1 | 0 | S26(Phospho) | T(5): 0.0; S(9): 0.0; S(12): 0.0; S(13): 0.0; T(15): 0.0; S(17): 0.0; S(19): 0.0; T(21): 0.0; S(26): 100.0; T(30): 0.0 | SATHGGGsPPGTRGP | S26; | 2865.3 | Yes |
| **BAG6** | NP_001092004.1 | 7917 | large proline-rich protein BAG6 isoform b | **S113** | | | 1.03 | APPQTHLPSGASSGTGSASATHGGGsPPGTR | 1 | 4 | 1 | 0 | S26(Phospho); R31(Label:13C(6)) | T(5): 0.0; S(9): 0.0; S(12): 0.0; S(13): 0.0; T(15): 0.0; S(17): 0.0; S(19): 0.0; T(21): 0.0; S(26): 100.0; T(30): 0.0 | SATHGGGsPPGTRGP | S26; | 2871.3 | Yes |
| **BAG6** | NP_001092004.1 | 7917 | large proline-rich protein BAG6 isoform b | **S958** | | 1.87 | 1.03 | VGDPPQPLPEEPMEVQGAERAsPEPQR | 2 | 3 | 1 | 1 | S22(Phospho) | S(22): 100.0 | VQGAERAsPEPQREN | S22; | 3020.4 | No |
| **BAG6** | NP_001092004.1 | 7917 | large proline-rich protein BAG6 isoform b | **S958** | **2.1** | | 1.03 | RVGDPPQPLPEEPMEVQGAERAsPEPQR | 1 | 3 | 1 | 2 | S23(Phospho) | S(23): 100.0 | VQGAERAsPEPQREN | S23; | 3176.5 | No |
| **BAG6** | NP_001092004.1 | 7917 | large proline-rich protein BAG6 isoform b | **S967** | | 1.15 | 1.03 | ENAsPAPGTTAEEAMSR | 2 | 3 | 1 | 0 | S4(Phospho) | S(4): 100.0; T(9): 0.0; T(10): 0.0; S(16): 0.0 | EPQRENAsPAPGTTA | S4; | 1798.7 | No |
| **BAG6** | NP_001092004.1 | 7917 | large proline-rich protein BAG6 isoform b | **S958** | | 1.44 | 1.03 | AsPEPQRENASPAPGTTAEEAMSR | 3 | 3 | 1 | 1 | S2(Phospho) | S(2): 100.0; S(11): 0.0; T(16): 0.0; T(17): 0.0; S(23): 0.0 | VQGAERAsPEPQREN | S2; | 2564.1 | No |
| **BAG6** | NP_001092004.1 | 7917 | large proline-rich protein BAG6 isoform b | **S958** | | | 1.03 | AsPEPQRENASPAPGTTAEEAMSR | 1 | 3 | 1 | 1 | S2(Phospho); R7(Label:13C(6)); R24(Label:13C(6)) | S(2): 100.0; S(11): 0.0; T(16): 0.0; T(17): 0.0; S(23): 0.0 | VQGAERAsPEPQREN | S2; | 2576.2 | No |

| GeneSymbol | NP_Accession | Gene ID | ProteinDescription | PhosphoSite (Protein) | SCC-R / SCC-S R1 | SCC-R / SCC-S R2 | SCC-R / SCC-S Total Proteome | Sequence | # PSMs | # Proteins | # Protein Groups | # Missed Cleavages | Modifications | phosphoRS Site Probabilities | Phosphowindow | PhosphoSite (Peptide) | MH+ [Da] | PhosphositePlusEvidence |
|---|---|---|---|---|---|---|---|---|---|---|---|---|---|---|---|---|---|---|
| **BAG6** | NP_001092004.1 | 7917 | large proline-rich protein BAG6 isoform b | **S1075** | | 0.88 | 1.03 | AAGARPLTsPESLSR | 1 | 2 | 1 | 0 | R5(Label:13C(6)); S9(Phospho); R15(Label:13C(6)) | T(8): 1.7; S(9): 98.2; S(12): 0.0; S(14): 0.0 | AGARPLTsPESLSRD | S9; | 1604.8 | No |
| **BAG6** | NP_001092004.1 | 7917 | large proline-rich protein BAG6 isoform b | **S967** | | | 1.03 | ENAsPAPGTTAEEAMSR | 1 | 3 | 1 | 0 | S4(Phospho); R17(Label:13C(6)) | S(4): 100.0; T(9): 0.0; T(10): 0.0; S(16): 0.0 | EPQRENAsPAPGTTA | S4; | 1804.8 | No |
| **BAIAP2** | NP_006331.1 | 10458 | brain-specific angiogenesis inhibitor 1-associated protein 2 isoform 3 | **S366** | | 0.98 | 0.24 | SSsMAAGLER | 2 | 4 | 1 | 0 | S3(Phospho) | S(1): 0.0; S(2): 0.0; S(3): 100.0 | KTLPRSSsMAAGLER | S3; | 1088.4 | Yes |
| **BAIAP2** | NP_006331.1 | 10458 | brain-specific angiogenesis inhibitor 1-associated protein 2 isoform 3 | **S366** | | | 0.24 | SSsMAAGLER | 1 | 4 | 1 | 0 | S3(Phospho); R10(Label:13C(6)) | S(1): 0.0; S(2): 0.0; S(3): 100.0 | KTLPRSSsMAAGLER | S3; | 1094.5 | Yes |
| **BAIAP2L1** | NP_061330.2 | 55971 | brain-specific angiogenesis inhibitor 1-associated protein 2-like protein 1 | **S261** | | | 0.28 | IMNMIEEIKTPASTPVSGTPQAsPMIER | 1 | 1 | 1 | 1 | K9(Label:13C(6)15N(2)); S23(Phospho); R28(Label:13C(6)) | T(10): 0.0; S(13): 0.0; T(14): 0.0; S(17): 0.0; T(19): 0.0; S(23): 100.0 | VSGTPQAsPMIERSN | S23; | 3122.5 | Yes |
| **BAIAP2L1** | NP_061330.2 | 55971 | brain-specific angiogenesis inhibitor 1-associated protein 2-like protein 1 | **S414** | | 0.85 | 0.28 | LLEENETEAVTVPTPsPTPVR | 12 | 1 | 1 | 0 | S16(Phospho) | T(7): 0.0; T(11): 0.0; T(14): 0.0; S(16): 100.0; T(18): 0.0 | AVTVPTPsPTPVRSI | S16; | 2359.1 | Yes |
| **BAIAP2L1** | NP_061330.2 | 55971 | brain-specific angiogenesis inhibitor 1-associated protein 2-like protein 1 | **S414** | | 1.08 | 0.28 | LLEENETEAVTVPTPsPTPVR | 8 | 1 | 1 | 0 | S16(Phospho); R21(Label:13C(6)) | T(7): 0.0; T(11): 0.0; T(14): 0.0; S(16): 98.3; T(18): 1.7 | AVTVPTPsPTPVRSI | S16; | 2365.2 | Yes |
| **BAIAP2L2** | NP_079321.3 | 80115 | brain-specific angiogenesis inhibitor 1-associated protein 2-like protein 2 | **S465** | | | #N/A | VPSRAPsPAPPPLPSSR | 2 | 1 | 1 | 1 | S7(Phospho) | S(3): 0.0; S(7): 100.0; S(15): 0.0; S(16): 0.0 | RVPSRAPsPAPPPLP | S7; | 1792.9 | Yes |
| **BAIAP2L2** | NP_079321.3 | 80115 | brain-specific angiogenesis inhibitor 1-associated protein 2-like protein 2 | **S465** | | | #N/A | APsPAPPPLPSSR | 2 | 1 | 1 | 0 | S3(Phospho) | S(3): 100.0; S(11): 0.0; S(12): 0.0 | RVPSRAPsPAPPPLP | S3; | 1353.7 | Yes |
| **BAIAP3** | NP_003924.2 | 8938 | BAI1-associated protein 3 isoform 1 | **S31** | | | #N/A | MRPRGAAFAAGPPGDLHLGTAIGFAGAIWRsR | 4 | 1 | 1 | 2 | M1(Oxidation); S31(Phospho) | T(20): 0.0; S(31): 100.0 | FAGAIWRsRSPAMST | S31; | 3373.7 | No |
| **BARD1** | NP_001269472.1 | 580 | BRCA1-associated RING domain protein 1 isoform 2 | **S311** | 0.85 | | #N/A | LSsPISKR | 1 | 2 | 1 | 1 | S3(Phospho) | S(2): 0.0; S(3): 100.0; S(6): 0.0 | GHHNRLSsPISKRCR | S3; | 967.5 | No |
| **BASP1** | NP_006308.3 | 10409 | brain acid soluble protein 1 | **T36** | 3.75 | | 2.12 | AEGAATEEEGtPK | 4 | 1 | 1 | 0 | T11(Phospho) | T(6): 0.0; T(11): 100.0 | AATEEEGtPKESEPQ | T11; | 1369.5 | Yes |
| **BASP1** | NP_006308.3 | 10409 | brain acid soluble protein 1 | **T36** | | 2.5 | 2.12 | KAEGAATEEEGtPKESEPQAAAEPAEAK | 2 | 1 | 1 | 2 | T12(Phospho) | T(7): 0.0; T(12): 100.0; S(16): 0.0 | AATEEEGtPKESEPQ | T12; | 2906.3 | Yes |
| **BASP1** | NP_006308.3 | 10409 | brain acid soluble protein 1 | **T36** | | 2.63 | 2.12 | AEGAATEEEGtPKESEPQAAAEPAEAK | 6 | 1 | 1 | 1 | T11(Phospho) | T(6): 0.0; T(11): 100.0; S(15): 0.0 | AATEEEGtPKESEPQ | T11; | 2778.2 | Yes |
| **BASP1** | NP_006308.3 | 10409 | brain acid soluble protein 1 | **T36** | | | 2.12 | AEGAATEEEGtPKESEPQAAAEPAEAK | 4 | 1 | 1 | 1 | T11(Phospho); K13(Label:13C(6)15N(2)); K27(Label:13C(6)15N(2)) | T(6): 0.1; T(11): 99.9; S(15): 0.0 | AATEEEGtPKESEPQ | T11; | 2794.2 | Yes |
| **BASP1** | NP_006308.3 | 10409 | brain acid soluble protein 1 | **T196** | | | 2.12 | ETPAATEAPSStPK | 2 | 1 | 1 | 0 | T12(Phospho) | T(2): 0.0; T(6): 0.0; S(10): 1.3; S(11): 1.3; T(12): 97.3 | ATEAPSStPKAQGPA | T12; | 1466.6 | Yes |
| **BAZ1A** | NP_872589.1 | 11177 | bromodomain adjacent to zinc finger domain protein 1A isoform b | **S1381** | 0.34 | 0.3 | #NQ | QsPEPSPVTLGR | 4 | 2 | 1 | 0 | S2(Phospho) | S(2): 100.0; S(6): 0.0; T(9): 0.0 | RRCRKRQsPEPSPVT | S2; | 1347.6 | No |
| **BAZ1A** | NP_872589.1 | 11177 | bromodomain adjacent to zinc finger domain protein 1A isoform b | **S1381** | 0.36 | 0.3 | #NQ | QsPEPSPVTLGR | 2 | 2 | 1 | 0 | S2(Phospho); R12(Label:13C(6)) | S(2): 100.0; S(6): 0.0; T(9): 0.0 | RRCRKRQsPEPSPVT | S2; | 1353.7 | No |
| **BAZ1A** | NP_872589.1 | 11177 | bromodomain adjacent to zinc finger domain protein 1A isoform b | **S1381** | | 0.34 | #NQ | RQsPEPSPVTLGR | 2 | 2 | 1 | 1 | S3(Phospho) | S(3): 100.0; S(7): 0.0; T(10): 0.0 | RRCRKRQsPEPSPVT | S3; | 1503.7 | No |
| **BAZ1A** | NP_872589.1 | 11177 | bromodomain adjacent to zinc finger domain protein 1A isoform b | **S1381** | | | #NQ | KRQsPEPSPVTLGR | 1 | 2 | 1 | 2 | K1(Label:13C(6)15N(2)); R2(Label:13C(6)); S4(Phospho); R14(Label:13C(6)) | S(4): 100.0; S(8): 0.0; T(11): 0.0 | RRCRKRQsPEPSPVT | S4; | 1651.9 | No |
| **BAZ1A** | NP_872589.1 | 11177 | bromodomain adjacent to zinc finger domain protein 1A isoform b | **S1381** | | 0.35 | #NQ | RQsPEPSPVTLGR | 1 | 2 | 1 | 1 | R1(Label:13C(6)); S3(Phospho); R13(Label:13C(6)) | S(3): 100.0; S(7): 0.0; T(10): 0.0 | RRCRKRQsPEPSPVT | S3; | 1515.8 | No |
| **BAZ1B** | NP_115784.1 | 9031 | tyrosine-protein kinase BAZ1B | **S1468** | 0.43 | | 0.40 | KFPDRLAEDEGDsEPEAVGQSR | 1 | 1 | 1 | 2 | S13(Phospho) | S(13): 100.0; S(21): 0.0 | LAEDEGDsEPEAVGQ | S13; | 2512.1 | Yes |
| **BAZ1B** | NP_115784.1 | 9031 | tyrosine-protein kinase BAZ1B | **S1468** | | 0.36 | 0.40 | KFPDRLAEDEGDsEPEAVGQSR | 2 | 1 | 1 | 2 | K1(Label:13C(6)15N(2)); R5(Label:13C(6)); S13(Phospho); R22(Label:13C(6)) | S(13): 100.0; S(21): 0.0 | LAEDEGDsEPEAVGQ | S13; | 2532.1 | Yes |
| **BAZ1B** | NP_115784.1 | 9031 | tyrosine-protein kinase BAZ1B | **S1468** | | 0.42 | 0.40 | LAEDEGDsEPEAVGQSR | 2 | 1 | 1 | 0 | S8(Phospho) | S(8): 100.0; S(16): 0.0 | LAEDEGDsEPEAVGQ | S8; | 1868.8 | Yes |
| **BAZ1B** | NP_115784.1 | 9031 | tyrosine-protein kinase BAZ1B | **S1468** | | | 0.40 | LAEDEGDsEPEAVGQSR | 2 | 1 | 1 | 0 | S8(Phospho); R17(Label:13C(6)) | S(8): 100.0; S(16): 0.0 | LAEDEGDsEPEAVGQ | S8; | 1874.8 | Yes |
| **BAZ2A** | NP_038477.2 | 11176 | bromodomain adjacent to zinc finger domain protein 2A | **S1184** | 0.38 | 0.4 | 0.68 | MELAGSNTTASsPAR | 4 | 1 | 1 | 0 | S12(Phospho) | S(6): 0.0; T(8): 0.0; T(9): 0.0; S(11): 1.0; S(12): 99.0 | GSNTTASsPARARGR | S12; | 1572.7 | Yes |
| **BAZ2A** | NP_038477.2 | 11176 | bromodomain adjacent to zinc finger domain protein 2A | **S1397** | 0.82 | | 0.68 | RAGDPGEMPQsPTGLGQPK | 2 | 1 | 1 | 1 | S11(Phospho) | S(11): 100.0; T(13): 0.0 | DPGEMPQsPTGLGQP | S11; | 2002.9 | Yes |
| **BAZ2A** | NP_038477.2 | 11176 | bromodomain adjacent to zinc finger domain protein 2A | **S1184** | 0.39 | 0.41 | 0.68 | MELAGSNTTASsPAR | 3 | 1 | 1 | 0 | S12(Phospho); R15(Label:13C(6)) | S(6): 0.0; T(8): 0.0; T(9): 0.0; S(11): 0.0; S(12): 100.0 | GSNTTASsPARARGR | S12; | 1578.7 | Yes |
| **BAZ2A** | NP_038477.2 | 11176 | bromodomain adjacent to zinc finger domain protein 2A | **S1397** | | | 0.68 | RAGDPGEMPQsPTGLGQPK | 1 | 1 | 1 | 1 | R1(Label:13C(6)); S11(Phospho); K19(Label:13C(6)15N(2)) | S(11): 100.0; T(13): 0.0 | DPGEMPQsPTGLGQP | S11; | 2016.9 | Yes |
| **BAZ2A** | NP_038477.2 | 11176 | bromodomain adjacent to zinc finger domain protein 2A | **S1397** | 1.36 | 1.16 | 0.68 | RRAGDPGEMPQsPTGLGQPK | 2 | 1 | 1 | 2 | R1(Label:13C(6)); R2(Label:13C(6)); S12(Phospho); K20(Label:13C(6)15N(2)) | S(12): 100.0; T(14): 0.0 | DPGEMPQsPTGLGQP | S12; | 2179.1 | Yes |
| **BAZ2A** | NP_038477.2 | 11176 | bromodomain adjacent to zinc finger domain protein 2A | **S135** | | 0.87 | 0.68 | QPSsPSHNTNLR | 2 | 1 | 1 | 0 | S4(Phospho) | S(3): 1.3; S(4): 98.7; S(6): 0.0; T(9): 0.0 | GGSRQPSsPSHNTNL | S4; | 1417.6 | Yes |
| **BAZ2A** | NP_038477.2 | 11176 | bromodomain adjacent to zinc finger domain protein 2A | **S1397** | | 0.9 | 0.68 | AGDPGEMPQsPTGLGQPKR | 1 | 1 | 1 | 1 | S10(Phospho) | S(10): 100.0; T(12): 0.0 | DPGEMPQsPTGLGQP | S10; | 2002.9 | Yes |
| **BAZ2A** | NP_038477.2 | 11176 | bromodomain adjacent to zinc finger domain protein 2A | **S135** | | 0.89 | 0.68 | QPSsPSHNTNLR | 1 | 1 | 1 | 0 | S4(Phospho); R12(Label:13C(6)) | S(3): 1.2; S(4): 98.8; S(6): 0.0; T(9): 0.0 | GGSRQPSsPSHNTNL | S4; | 1423.6 | Yes |
| **BAZ2B** | NP_001276904.1 | 29994 | bromodomain adjacent to zinc finger domain protein 2B isoform b | **T1978** | | | #N/A | KVTLTGDtEDEDSASTSSSLKR | 1 | 2 | 1 | 2 | K1(Label:13C(6)15N(2)); T8(Phospho); K21(Label:13C(6)15N(2)); R22(Label:13C(6)) | T(3): 0.0; T(5): 0.0; T(8): 100.0; S(13): 0.0; S(15): 0.0; T(16): 0.0; S(17): 0.0; S(18): 0.0; S(19): 0.0 | KVTLTGDtEDEDSAS | T8; | 2429.1 | No |
| **BAZ2B** | NP_001276904.1 | 29994 | bromodomain adjacent to zinc finger domain protein 2B isoform b | **T1247** | | | #N/A | DTSGGIDLGEEQHPLGtPTPGR | 2 | 2 | 1 | 0 | T17(Phospho); R22(Label:13C(6)) | T(2): 0.0; S(3): 0.0; T(17): 97.9; T(19): 2.1 | EEQHPLGtPTPGRKR | T17; | 2320.1 | No |
| **BBX** | NP_064620.2 | 56987 | HMG box transcription factor BBX isoform 2 | **S704** | 0.68 | | 0.71 | FNSLPQYsPVTFDRK | 1 | 2 | 1 | 1 | S8(Phospho); R14(Label:13C(6)); K15(Label:13C(6)15N(2)) | S(3): 0.0; Y(7): 0.0; S(8): 100.0; T(11): 0.0 | FNSLPQYsPVTFDRK | S8; | 1892.9 | Yes |
| **BBX** | NP_064620.2 | 56987 | HMG box transcription factor BBX isoform 2 | **S159** | | 1.34 | 0.71 | sPTPTVNPR | 2 | 3 | 1 | 0 | S1(Phospho) | S(1): 100.0; T(3): 0.0; T(5): 0.0 | TTNKPVKsPTPTVNP | S1; | 1048.5 | Yes |
| **BCAR1** | NP_001164190.1 | 9564 | breast cancer anti-estrogen resistance protein 1 isoform 7 | **Y247** | | | 2.45 | HLLAPGPQDIyDVPPVR | 3 | 9 | 1 | 0 | Y11(Phospho) | Y(11): 100.0 | APGPQDIyDVPPVRG | Y11; | 1967.0 | No |
| **BCAR1** | NP_001164190.1 | 9564 | breast cancer anti-estrogen resistance protein 1 isoform 7 | **Y385** | 4.72 | 5.41 | 2.45 | RPGPGTLyDVPR | 2 | 9 | 1 | 0 | Y8(Phospho) | T(6): 1.3; Y(8): 98.7 | RPGPGTLyDVPRERV | Y8; | 1407.7 | No |
| **BCAR1** | NP_001164190.1 | 9564 | breast cancer anti-estrogen resistance protein 1 isoform 7 | **S426** | 0.42 | | 2.45 | LsASSTGSTR | 2 | 9 | 1 | 0 | S2(Phospho) | S(2): 100.0; S(4): 0.0; S(5): 0.0; T(6): 0.0; S(8): 0.0; T(9): 0.0 | PAEGKRLsASSTGST | S2; | 1046.5 | No |
| **BCAR1** | NP_001164190.1 | 9564 | breast cancer anti-estrogen resistance protein 1 isoform 7 | **Y408** | | | 2.45 | VLPPEVADGGVVDSGVyAVPPPAER | 1 | 9 | 1 | 0 | Y17(Phospho) | S(14): 0.0; Y(17): 100.0 | GVVDSGVyAVPPPAE | Y17; | 2570.3 | No |
| **BCAR1** | NP_001164190.1 | 9564 | breast cancer anti-estrogen resistance protein 1 isoform 7 | **S426** | | 0.98 | 2.45 | RLsASSTGSTR | 2 | 9 | 1 | 1 | S3(Phospho) | S(3): 100.0; S(5): 0.0; S(6): 0.0; T(7): 0.0; S(9): 0.0; T(10): 0.0 | PAEGKRLsASSTGST | S3; | 1202.6 | No |
| **BCAR1** | NP_001164190.1 | 9564 | breast cancer anti-estrogen resistance protein 1 isoform 7 | **S426** | | 1.01 | 2.45 | RLsASSTGSTR | 1 | 9 | 1 | 1 | R1(Label:13C(6)); S3(Phospho); R11(Label:13C(6)) | S(3): 100.0; S(5): 0.0; S(6): 0.0; T(7): 0.0; S(9): 0.0; T(10): 0.0 | PAEGKRLsASSTGST | S3; | 1214.6 | No |
| **BCAR3** | NP_001248338.1 | 8412 | breast cancer anti-estrogen resistance protein 3 isoform 1 | **S290** | 6.16 | 5.89 | 8.18 | RLsLTMGGVQAR | 9 | 2 | 1 | 1 | S3(Phospho) | S(3): 100.0; T(5): 0.0 | PDVAKRLsLTMGGVQ | S3; | 1368.7 | Yes |
| **BCAR3** | NP_001248338.1 | 8412 | breast cancer anti-estrogen resistance protein 3 isoform 1 | **S8** | 12.55 | 16.15 | 8.18 | FAsLPR | 7 | 1 | 1 | 0 | S3(Phospho) | S(3): 100.0 | MAAGKFAsLPRNMPV | S3; | 770.4 | Yes |
| **BCAR3** | NP_001248338.1 | 8412 | breast cancer anti-estrogen resistance protein 3 isoform 1 | **S290** | 8.02 | | 8.18 | LsLTMGGVQAR | 2 | 2 | 1 | 0 | S2(Phospho) | S(2): 100.0; T(4): 0.0 | PDVAKRLsLTMGGVQ | S2; | 1212.6 | Yes |
| **BCAR3** | NP_001248338.1 | 8412 | breast cancer anti-estrogen resistance protein 3 isoform 1 | **S375** | | 2.01 | 8.18 | TGSEPALsPAVVR | 6 | 2 | 1 | 0 | S8(Phospho) | T(1): 0.0; S(3): 0.0; S(8): 100.0 | TGSEPALsPAVVRRV | S8; | 1363.7 | Yes |
| **BCAR3** | NP_001248338.1 | 8412 | breast cancer anti-estrogen resistance protein 3 isoform 1 | **S363** | | | 8.18 | LPPQSSGVDTSPCPNsPVFR | 7 | 2 | 1 | 0 | C13(Carbamidomethyl); S16(Phospho) | S(5): 0.0; S(6): 0.0; T(10): 0.0; S(11): 0.0; S(16): 100.0 | DTSPCPNsPVFRTGS | S16; | 2222.0 | Yes |
| **BCAR3** | NP_001248338.1 | 8412 | breast cancer anti-estrogen resistance protein 3 isoform 1 | **S290** | | | 8.18 | RLsLTMGGVQAR | 1 | 2 | 1 | 1 | S3(Phospho); M6(Oxidation) | S(3): 100.0; T(5): 0.0 | PDVAKRLsLTMGGVQ | S3; | 1384.7 | Yes |
| **BCAR3** | NP_001248338.1 | 8412 | breast cancer anti-estrogen resistance protein 3 isoform 1 | **T130** | | | 8.18 | HIMDRtPEK | 1 | 2 | 1 | 1 | T6(Phospho) | T(6): 100.0 | ERHIMDRtPEKLKKE | T6; | 1206.5 | Yes |
| **BCAR3** | NP_001248338.1 | 8412 | breast cancer anti-estrogen resistance protein 3 isoform 1 | **S370** | | | 8.18 | TGsEPALSPAVVR | 1 | 2 | 1 | 0 | S3(Phospho); R13(Label:13C(6)) | T(1): 0.0; S(3): 100.0; S(8): 0.0 | SPVFRTGsEPALSPA | S3; | 1369.7 | Yes |
| **BCAS3** | NP_060149.3 | 54828 | breast carcinoma-amplified sequence 3 isoform 2 | **S856** | | | 0.83 | LADAMAESPsRDVVGSGTELQR | 2 | 2 | 1 | 1 | S8(Phospho) | S(8): 1.6; S(10): 98.4; S(16): 0.0; T(18): 0.0 | DAMAESPsRDVVGSG | S10; | 2369.1 | No |

| GeneSymbol | NP_Accession | Gene ID | ProteinDescription | PhosphoSite (Protein) | SCC-R / SCC-S R1 | SCC-R / SCC-S R2 | SCC-R / SCC-S Total Proteome | Sequence | # PSMs | # Proteins | # Protein Groups | # Missed Cleavages | Modifications | phosphoRS Site Probabilities | Phosphowindow | PhosphoSite (Peptide) | MH+ [Da] | PhosphositePlusEvidence |
|---|---|---|---|---|---|---|---|---|---|---|---|---|---|---|---|---|---|---|
| **BCAS3** | NP_060149.3 | 54828 | breast carcinoma-amplified sequence 3 isoform 2 | **S856** | | | 0.83 | LADAMAESPsRDVVGSGTELQR | 1 | 2 | 1 | 1 | S8(Phospho); R11(Label:13C(6)); R22(Label:13C(6)) | S(8): 1.5; S(10): 98.5; S(16): 0.0; T(18): 0.0 | DAMAESPsRDVVGSG | S10; | 2381.1 | No |
| **BCAT1** | NP_001171564.1 | 586 | branched-chain-amino-acid aminotransferase, cytosolic isoform 4 | **T25; S30** | | | 1.83 | QDCSNGCSAECtGEGGsK | 1 | 1 | 1 | 0 | C3(Carbamidomethyl); C7(Carbamidomethyl); S8(Phospho); C11(Carbamidomethyl); S17(Phospho) | S(4): 0.7; S(8): 0.7; T(12): 98.9; S(17): 99.8 | NGCSAECtGEGGSKE; ECTGEGGsKEVVGTF | T12; S17; | 2063.6 | Yes;Yes |
| **BCKDHA** | NP_000700.1 | 593 | 2-oxoisovalerate dehydrogenase subunit alpha, mitochondrial isoform 1 precursor | **S347** | 1.14 | | 1.62 | sVDEVNYWDKQDHPISR | 1 | 2 | 1 | 1 | S1(Phospho); K10(Label:13C(6)15N(2)); R17(Label:13C(6)) | S(1): 100.0; Y(7): 0.0; S(16): 0.0 | DDSSAYRsVDEVNYW | S1; | 2182.0 | Yes |
| **BCKDHA** | NP_000700.1 | 593 | 2-oxoisovalerate dehydrogenase subunit alpha, mitochondrial isoform 1 precursor | **S337** | | 0.38 | 1.62 | IGHHsTSDDSSAYR | 1 | 2 | 1 | 0 | S5(Phospho); R14(Label:13C(6)) | S(5): 100.0; T(6): 0.0; S(7): 0.0; S(10): 0.0; S(11): 0.0; Y(13): 0.0 | TYRIGHHsTSDDSSA | S5; | 1618.7 | Yes |
| **BCL2L12** | NP_001035758.1 | 83596 | bcl-2-like protein 12 isoform 3 | **S241** | | | #N/A | LVRLsSDSFAR | 1 | 2 | 1 | 1 | R3(Label:13C(6)); S5(Phospho); R11(Label:13C(6)) | S(5): 99.0; S(6): 1.0; S(8): 0.0 | RSKLVRLsSDSFARL | S5; | 1342.7 | No |
| **BCL3** | NP_005169.2 | 602 | B-cell lymphoma 3 protein | **S41** | 1.44 | | #N/A | APsPEPAAPR | 2 | 1 | 1 | 0 | S3(Phospho) | S(3): 100.0 | KRPLRAPsPEPAAPR | S3; | 1072.5 | Yes |
| **BCL3** | NP_005169.2 | 602 | B-cell lymphoma 3 protein | **S41** | 1.51 | | #N/A | APsPEPAAPR | 2 | 1 | 1 | 0 | S3(Phospho); R10(Label:13C(6)) | S(3): 100.0 | KRPLRAPsPEPAAPR | S3; | 1078.5 | Yes |
| **BCL9** | NP_004317.2 | 607 | B-cell CLL/lymphoma 9 protein | **S687** | 0.91 | | #N/A | IPVEGPLsPSRGDFPK | 1 | 1 | 1 | 1 | S8(Phospho) | S(8): 98.5; S(10): 1.5 | IPVEGPLsPSRGDFP | S8; | 1775.9 | Yes |
| **BCL9L** | NP_872363.1 | 283149 | B-cell CLL/lymphoma 9-like protein | **S750** | 1.41 | 1.3 | #NQ | GLLsPPMGQSGLR | 6 | 1 | 1 | 0 | S4(Phospho) | S(4): 100.0; S(10): 0.0 | GGGRGLLsPPMGQSG | S4; | 1392.7 | Yes |
| **BCL9L** | NP_872363.1 | 283149 | B-cell CLL/lymphoma 9-like protein | **S21** | 1.6 | 2 | #NQ | EAPGsPPLSPR | 8 | 1 | 1 | 0 | S5(Phospho) | S(5): 100.0; S(9): 0.0 | RRREAPGsPPLSPRG | S5; | 1187.5 | Yes |
| **BCL9L** | NP_872363.1 | 283149 | B-cell CLL/lymphoma 9-like protein | **S750** | | | #NQ | GLLsPPMGQSGLR | 1 | 1 | 1 | 0 | S4(Phospho); R13(Label:13C(6)) | S(4): 100.0; S(10): 0.0 | GGGRGLLsPPMGQSG | S4; | 1398.7 | Yes |
| **BCL9L** | NP_872363.1 | 283149 | B-cell CLL/lymphoma 9-like protein | **S999** | | 2.84 | #NQ | LKSPsMAVPSPGWVASPK | 2 | 1 | 1 | 1 | S5(Phospho) | S(3): 2.1; S(5): 97.9; S(10): 0.0; S(16): 0.0 | PSRLKSPsMAVPSPG | S5; | 1919.0 | Yes |
| **BCL9L** | NP_872363.1 | 283149 | B-cell CLL/lymphoma 9-like protein | **S915** | | 24.64 | #NQ | RPsDLTISINQMGSPGMGHLK | 2 | 1 | 1 | 0 | S3(Phospho) | S(3): 100.0; T(6): 0.0; S(8): 0.0; S(14): 0.0 | RGLGRRPsDLTISIN | S3; | 2319.1 | Yes |
| **BCL9L** | NP_872363.1 | 283149 | B-cell CLL/lymphoma 9-like protein | **S118** | | 2.62 | #NQ | DRSVsVDSGEQR | 1 | 1 | 1 | 1 | S5(Phospho) | S(3): 0.0; S(5): 100.0; S(8): 0.0 | VKRDRSVsVDSGEQR | S5; | 1414.6 | Yes |
| **BCL9L** | NP_872363.1 | 283149 | B-cell CLL/lymphoma 9-like protein | **S21; S25** | | 2.43 | #NQ | EAPGsPPLsPR | 2 | 1 | 1 | 0 | S5(Phospho); S9(Phospho) | S(5): 100.0; S(9): 100.0 | RRREAPGsPPLSPRG; APGSPPLsPRGHCPP | S5; S9; | 1267.5 | Yes;Yes |
| **BCL9L** | NP_872363.1 | 283149 | B-cell CLL/lymphoma 9-like protein | **S21; S25** | | | #NQ | EAPGsPPLsPR | 1 | 1 | 1 | 0 | S5(Phospho); S9(Phospho); R11(Label:13C(6)) | S(5): 100.0; S(9): 100.0 | RRREAPGsPPLSPRG; APGSPPLsPRGHCPP | S5; S9; | 1273.5 | Yes;Yes |
| **BCL9L** | NP_872363.1 | 283149 | B-cell CLL/lymphoma 9-like protein | **S21** | | 2.43 | #NQ | EAPGsPPLSPR | 1 | 1 | 1 | 0 | S5(Phospho); R11(Label:13C(6)) | S(5): 100.0; S(9): 0.0 | RRREAPGsPPLSPRG | S5; | 1193.6 | Yes |
| **BCLAF1** | NP_001070908.1 | 9774 | bcl-2-associated transcription factor 1 isoform 2 | **S494** | 1.11 | 1.08 | 1.07 | ETQsPEQVKSEK | 7 | 2 | 1 | 1 | S4(Phospho); K9(Label:13C(6)15N(2)); K12(Label:13C(6)15N(2)) | T(2): 0.0; S(4): 100.0; S(10): 0.0 | TVKKETQsPEQVKSE | S4; | 1485.7 | No |
| **BCLAF1** | NP_001070908.1 | 9774 | bcl-2-associated transcription factor 1 isoform 2 | **S494** | 1.09 | 1.07 | 1.07 | ETQsPEQVK | 2 | 2 | 1 | 0 | S4(Phospho); K9(Label:13C(6)15N(2)) | T(2): 0.0; S(4): 100.0 | TVKKETQsPEQVKSE | S4; | 1133.5 | No |
| **BCLAF1** | NP_001070908.1 | 9774 | bcl-2-associated transcription factor 1 isoform 2 | **S494** | 1.16 | 1.15 | 1.07 | KETQsPEQVKSEK | 4 | 2 | 1 | 2 | S5(Phospho) | T(3): 0.0; S(5): 100.0; S(11): 0.0 | TVKKETQsPEQVKSE | S5; | 1597.7 | No |
| **BCLAF1** | NP_001070908.1 | 9774 | bcl-2-associated transcription factor 1 isoform 2 | **S510** | 1.21 | 1.18 | 1.07 | LKDLFDYsPPLHK | 16 | 2 | 1 | 1 | K2(Label:13C(6)15N(2)); S8(Phospho); K13(Label:13C(6)15N(2)) | Y(7): 0.0; S(8): 100.0 | LKDLFDYsPPLHKNL | S8; | 1668.8 | No |
| **BCLAF1** | NP_001070908.1 | 9774 | bcl-2-associated transcription factor 1 isoform 2 | **S494** | | | 1.07 | ETQsPEQVKSEK | 6 | 2 | 1 | 1 | S4(Phospho) | T(2): 0.0; S(4): 100.0; S(10): 0.0 | TVKKETQsPEQVKSE | S4; | 1469.7 | No |
| **BCLAF1** | NP_001070908.1 | 9774 | bcl-2-associated transcription factor 1 isoform 2 | **S510** | 1.43 | 1.29 | 1.07 | LKDLFDYsPPLHK | 24 | 2 | 1 | 1 | S8(Phospho) | Y(7): 0.0; S(8): 100.0 | LKDLFDYsPPLHKNL | S8; | 1652.8 | No |
| **BCLAF1** | NP_001070908.1 | 9774 | bcl-2-associated transcription factor 1 isoform 2 | **S494** | 1.29 | 1.21 | 1.07 | KETQsPEQVK | 4 | 2 | 1 | 1 | S5(Phospho) | T(3): 0.0; S(5): 100.0 | TVKKETQsPEQVKSE | S5; | 1253.6 | No |
| **BCLAF1** | NP_001070908.1 | 9774 | bcl-2-associated transcription factor 1 isoform 2 | **S510** | 1.57 | | 1.07 | DLFDYsPPLHK | 3 | 2 | 1 | 0 | S6(Phospho) | Y(5): 0.0; S(6): 100.0 | LKDLFDYsPPLHKNL | S6; | 1411.6 | No |
| **BCLAF1** | NP_001070908.1 | 9774 | bcl-2-associated transcription factor 1 isoform 2 | **S510** | 1.05 | | 1.07 | DLFDYsPPLHK | 5 | 2 | 1 | 0 | S6(Phospho); K11(Label:13C(6)15N(2)) | Y(5): 0.0; S(6): 100.0 | LKDLFDYsPPLHKNL | S6; | 1419.6 | No |
| **BCLAF1** | NP_001070908.1 | 9774 | bcl-2-associated transcription factor 1 isoform 2 | **S494** | 1.14 | | 1.07 | ETQsPEQVK | 3 | 2 | 1 | 0 | S4(Phospho) | T(2): 0.0; S(4): 100.0 | TVKKETQsPEQVKSE | S4; | 1125.5 | No |
| **BCLAF1** | NP_001070908.1 | 9774 | bcl-2-associated transcription factor 1 isoform 2 | **S494** | | | 1.07 | KETQsPEQVK | 3 | 2 | 1 | 1 | K1(Label:13C(6)15N(2)); S5(Phospho); K10(Label:13C(6)15N(2)) | T(3): 0.0; S(5): 100.0 | TVKKETQsPEQVKSE | S5; | 1269.6 | No |
| **BCLAF1** | NP_001070908.1 | 9774 | bcl-2-associated transcription factor 1 isoform 2 | **S395** | | 0.96 | 1.07 | FNDsEGDDTEETEDYR | 6 | 2 | 1 | 0 | S4(Phospho) | S(4): 100.0; T(9): 0.0; T(12): 0.0; Y(15): 0.0 | GKQKFNDsEGDDTEE | S4; | 2001.7 | No |
| **BCLAF1** | NP_001070908.1 | 9774 | bcl-2-associated transcription factor 1 isoform 2 | **T339** | | 1.76 | 1.07 | RFtDEESR | 2 | 2 | 1 | 1 | T3(Phospho) | T(3): 100.0; S(7): 0.0 | GKFLKRFtDEESRVF | T3; | 1119.4 | No |
| **BCLAF1** | NP_001070908.1 | 9774 | bcl-2-associated transcription factor 1 isoform 2 | **S395; T400** | | 1.42 | 1.07 | FNDsEGDDtEETEDYR | 1 | 2 | 1 | 0 | S4(Phospho); T9(Phospho) | S(4): 100.0; T(9): 100.0; T(12): 0.0; Y(15): 0.0 | GKQKFNDsEGDDTEE; NDSEGDDtEETEDYR | S4; T9; | 2081.7 | No;No |
| **BCLAF1** | NP_001070908.1 | 9774 | bcl-2-associated transcription factor 1 isoform 2 | **S494** | | 1.15 | 1.07 | KETQsPEQVKSEK | 2 | 2 | 1 | 2 | K1(Label:13C(6)15N(2)); S5(Phospho); K10(Label:13C(6)15N(2)); K13(Label:13C(6)15N(2)) | T(3): 0.0; S(5): 100.0; S(11): 0.0 | TVKKETQsPEQVKSE | S5; | 1621.8 | No |
| **BCLAF1** | NP_001070909.1 | 9774 | bcl-2-associated transcription factor 1 isoform 3 | **S339** | | 0.52 | 1.07 | FLKsPPLHK | 1 | 1 | 1 | 1 | S4(Phospho) | S(4): 100.0 | KTGKFLKsPPLHKNL | S4; | 1146.6 | Yes |
| **BCLAF1** | NP_001070908.1 | 9774 | bcl-2-associated transcription factor 1 isoform 2 | **S395** | | | 1.07 | FNDsEGDDTEETEDYR | 2 | 2 | 1 | 0 | S4(Phospho); R16(Label:13C(6)) | S(4): 100.0; T(9): 0.0; T(12): 0.0; Y(15): 0.0 | GKQKFNDsEGDDTEE | S4; | 2007.7 | No |
| **BCLAF1\|\|BCLAF1** | NP_001070908.1\|\| NP_001070909.1 | 9774\|\|9774 | bcl-2-associated transcription factor 1 isoform 2\|\|bcl-2-associated transcription factor 1 isoform 3 | **S266\|\|S268** | | | #N/A | NTPSQHSHSIQHsPER | 10 | 3 | 2 | 0 | S13(Phospho) | T(2): 0.0; S(4): 0.0; S(7): 0.0; S(9): 0.0; S(13): 100.0 | HSHSIQHsPERSGSG\|\| HSHSIQHsPERSGSG | S13; | 1921.8 | No\|\|Yes |
| **BCLAF1\|\|BCLAF1** | NP_001070908.1\|\| NP_001070909.1 | 9774\|\|9774 | bcl-2-associated transcription factor 1 isoform 2\|\|bcl-2-associated transcription factor 1 isoform 3 | **S175\|\|S177** | | | #N/A | KAEGEPQEEsPLKSK | 9 | 3 | 2 | 2 | K1(Label:13C(6)15N(2)); S10(Phospho); K13(Label:13C(6)15N(2)); K15(Label:13C(6)15N(2)) | S(10): 100.0; S(14): 0.0 | EGEPQEEsPLKSKSQ\|\| EGEPQEEsPLKSKSQ | S10; | 1760.9 | No\|\|Yes |
| **BCLAF1\|\|BCLAF1** | NP_001070908.1\|\| NP_001070909.1 | 9774\|\|9774 | bcl-2-associated transcription factor 1 isoform 2\|\|bcl-2-associated transcription factor 1 isoform 3 | **S656\|\|S485** | | | #N/A | IDIsPSTLR | 46 | 3 | 2 | 0 | S4(Phospho) | S(4): 100.0; S(6): 0.0; T(7): 0.0 | IHRRIDIsPSTLRKH\|\|IH RRIDIsPSTLRKH | S4; | 1081.5 | No\|\|No |
| **BCLAF1\|\|BCLAF1** | NP_001070908.1\|\| NP_001070909.1 | 9774\|\|9774 | bcl-2-associated transcription factor 1 isoform 2\|\|bcl-2-associated transcription factor 1 isoform 3 | **S175\|\|S177** | | | #N/A | KAEGEPQEEsPLKSK | 9 | 3 | 2 | 2 | S10(Phospho) | S(10): 100.0; S(14): 0.0 | EGEPQEEsPLKSKSQ\|\| EGEPQEEsPLKSKSQ | S10; | 1736.8 | No\|\|Yes |
| **BCLAF1\|\|BCLAF1** | NP_001070908.1\|\| NP_001070909.1 | 9774\|\|9774 | bcl-2-associated transcription factor 1 isoform 2\|\|bcl-2-associated transcription factor 1 isoform 3 | **S298\|\|S300** | | | #N/A | sPAKTIAPQNAPR | 4 | 3 | 2 | 1 | S1(Phospho) | S(1): 100.0; T(5): 0.0 | HHIPSRRsPAKTIAP\|\|H HIPSRRsPAKTIAP | S1; | 1430.7 | No\|\|Yes |
| **BCLAF1\|\|BCLAF1** | NP_001070908.1\|\| NP_001070909.1 | 9774\|\|9774 | bcl-2-associated transcription factor 1 isoform 2\|\|bcl-2-associated transcription factor 1 isoform 3 | **S646\|\|S475** | | | #N/A | QKsPEIHR | 5 | 3 | 2 | 1 | S3(Phospho) | S(3): 100.0 | EHSTRQKsPEIHRRI\|\|E HSTRQKsPEIHRRI | S3; | 1074.5 | No\|\|No |
| **BCLAF1\|\|BCLAF1** | NP_001070908.1\|\| NP_001070909.1 | 9774\|\|9774 | bcl-2-associated transcription factor 1 isoform 2\|\|bcl-2-associated transcription factor 1 isoform 3 | **T255; S266\|\|T257** | | | #N/A | NtPSQHSHSIQHsPER | 3 | 3 | 2 | 0 | T2(Phospho); S13(Phospho) | T(2): 100.0; S(4): 0.0; S(7): 0.0; S(9): 0.0; S(13): 100.0 | TVHSAKNtPSQHSHS; HSHSIQHsPERSGSG\|\| TVHSAKNtPSQHSHS | T2; S13; | 2001.8 | Yes |
| **BCLAF1\|\|BCLAF1** | NP_001070908.1\|\| NP_001070909.1 | 9774\|\|9774 | bcl-2-associated transcription factor 1 isoform 2\|\|bcl-2-associated transcription factor 1 isoform 3 | **S656\|\|S485** | | | #N/A | RIDIsPSTLR | 18 | 3 | 2 | 1 | S5(Phospho) | S(5): 100.0; S(7): 0.0; T(8): 0.0 | IHRRIDIsPSTLRKH\|\|IH RRIDIsPSTLRKH | S5; | 1237.6 | No\|\|No |
| **BCLAF1\|\|BCLAF1** | NP_001070908.1\|\| NP_001070909.1 | 9774\|\|9774 | bcl-2-associated transcription factor 1 isoform 2\|\|bcl-2-associated transcription factor 1 isoform 3 | **S175\|\|S177** | | | #N/A | AEGEPQEEsPLKSK | 3 | 3 | 2 | 1 | S9(Phospho) | S(9): 100.0; S(13): 0.0 | EGEPQEEsPLKSKSQ\|\| EGEPQEEsPLKSKSQ | S9; | 1608.7 | No\|\|Yes |
| **BCLAF1\|\|BCLAF1** | NP_001070908.1\|\| NP_001070909.1 | 9774\|\|9774 | bcl-2-associated transcription factor 1 isoform 2\|\|bcl-2-associated transcription factor 1 isoform 3 | **S656\|\|S485** | | | #N/A | IDIsPSTLRK | 8 | 3 | 2 | 1 | S4(Phospho) | S(4): 100.0; S(6): 0.0; T(7): 0.0 | IHRRIDIsPSTLRKH\|\|IH RRIDIsPSTLRKH | S4; | 1209.6 | No\|\|No |
| **BCLAF1\|\|BCLAF1** | NP_001070908.1\|\| NP_001070909.1 | 9774\|\|9774 | bcl-2-associated transcription factor 1 isoform 2\|\|bcl-2-associated transcription factor 1 isoform 3 | **S175\|\|S177** | | | #N/A | AEGEPQEEsPLKSK | 6 | 3 | 2 | 1 | S9(Phospho); K12(Label:13C(6)15N(2)); K14(Label:13C(6)15N(2)) | S(9): 98.9; S(13): 1.1 | EGEPQEEsPLKSKSQ\|\| EGEPQEEsPLKSKSQ | S9; | 1624.7 | No\|\|Yes |
| **BCLAF1\|\|BCLAF1** | NP_001070908.1\|\| NP_001070909.1 | 9774\|\|9774 | bcl-2-associated transcription factor 1 isoform 2\|\|bcl-2-associated transcription factor 1 isoform 3 | **S656\|\|S485** | | | #N/A | RIDIsPSTLRK | 2 | 3 | 2 | 2 | S5(Phospho) | S(5): 100.0; S(7): 0.0; T(8): 0.0 | IHRRIDIsPSTLRKH\|\|IH RRIDIsPSTLRKH | S5; | 1365.7 | No\|\|No |
| **BCLAF1\|\|BCLAF1** | NP_001070908.1\|\| NP_001070909.1 | 9774\|\|9774 | bcl-2-associated transcription factor 1 isoform 2\|\|bcl-2-associated transcription factor 1 isoform 3 | **S529\|\|S358** | | | #N/A | EEsPLRIK | 4 | 3 | 2 | 1 | S3(Phospho) | S(3): 100.0 | KSTFREEsPLRIKMI\|\|K STFREEsPLRIKMI | S3; | 1051.5 | No\|\|No |
| **BCLAF1\|\|BCLAF1** | NP_001070908.1\|\| NP_001070909.1 | 9774\|\|9774 | bcl-2-associated transcription factor 1 isoform 2\|\|bcl-2-associated transcription factor 1 isoform 3 | **S266\|\|S268** | | | #N/A | NTPSQHSHSIQHsPER | 5 | 3 | 2 | 0 | S13(Phospho); R16(Label:13C(6)) | T(2): 0.0; S(4): 0.0; S(7): 0.0; S(9): 0.0; S(13): 100.0 | HSHSIQHsPERSGSG\|\| HSHSIQHsPERSGSG | S13; | 1927.9 | No\|\|Yes |
| **BCLAF1\|\|BCLAF1** | NP_001070908.1\|\| NP_001070909.1 | 9774\|\|9774 | bcl-2-associated transcription factor 1 isoform 2\|\|bcl-2-associated transcription factor 1 isoform 3 | **T255; S266\|\|T257** | | | #N/A | NtPSQHSHSIQHsPER | 1 | 3 | 2 | 0 | T2(Phospho); S13(Phospho); R16(Label:13C(6)) | T(2): 100.0; S(4): 0.0; S(7): 0.0; S(9): 0.0; S(13): 100.0 | TVHSAKNtPSQHSHS; HSHSIQHsPERSGSG\|\| TVHSAKNtPSQHSHS | T2; S13; | 2007.8 | Yes |
| **BCLAF1\|\|BCLAF1** | NP_001070908.1\|\| NP_001070909.1 | 9774\|\|9774 | bcl-2-associated transcription factor 1 isoform 2\|\|bcl-2-associated transcription factor 1 isoform 3 | **S298\|\|S300** | | | #N/A | sPAKTIAPQNAPR | 3 | 3 | 2 | 1 | S1(Phospho); K4(Label:13C(6)15N(2)); R13(Label:13C(6)) | S(1): 100.0; T(5): 0.0 | HHIPSRRsPAKTIAP\|\|H HIPSRRsPAKTIAP | S1; | 1444.8 | No\|\|Yes |
| **BCLAF1\|\|BCLAF1** | NP_001070908.1\|\| NP_001070909.1 | 9774\|\|9774 | bcl-2-associated transcription factor 1 isoform 2\|\|bcl-2-associated transcription factor 1 isoform 3 | **S656\|\|S485** | | | #N/A | IDIsPSTLR | 18 | 3 | 2 | 0 | S4(Phospho); R9(Label:13C(6)) | S(4): 100.0; S(6): 0.0; T(7): 0.0 | IHRRIDIsPSTLRKH\|\|IH RRIDIsPSTLRKH | S4; | 1087.5 | No\|\|No |
| **BCLAF1\|\|BCLAF1** | NP_001070908.1\|\| NP_001070909.1 | 9774\|\|9774 | bcl-2-associated transcription factor 1 isoform 2\|\|bcl-2-associated transcription factor 1 isoform 3 | **S656\|\|S485** | | | #N/A | RIDIsPSTLR | 8 | 3 | 2 | 1 | R1(Label:13C(6)); S5(Phospho); R10(Label:13C(6)) | S(5): 100.0; S(7): 0.0; T(8): 0.0 | IHRRIDIsPSTLRKH\|\|IH RRIDIsPSTLRKH | S5; | 1249.7 | No\|\|No |

| GeneSymbol | NP_Accession | Gene ID | ProteinDescription | PhosphoSite (Protein) | SCC-R / SCC-S R1 | SCC-R / SCC-S R2 | SCC-R / SCC-S Total Proteome | Sequence | # PSMs | # Proteins | # Protein Groups | # Missed Cleavages | Modifications | phosphoRS Site Probabilities | Phosphowindow | PhosphoSite (Peptide) | MH+ [Da] | PhosphositePlusEvidence |
|---|---|---|---|---|---|---|---|---|---|---|---|---|---|---|---|---|---|---|
| **BCLAF1\|\|BCLAF1** | NP_001070908.1\|\| NP_001070909.1 | 9774\|\|9774 | bcl-2-associated transcription factor 1 isoform 2\|\|bcl-2-associated transcription factor 1 isoform 3 | **S656\|\|S485** | | | #N/A | IDIsPSTLRK | 2 | 3 | 2 | 1 | S4(Phospho); R9(Label:13C(6)); K10(Label:13C(6)15N(2)) | S(4): 100.0; S(6): 0.0; T(7): 0.0 | IHRRIDIsPSTLRKH\|\|IHRRIDIsPSTLRKH | S4; | 1223.7 | No\|\|No |
| **BCLAF1\|\|BCLAF1** | NP_001070908.1\|\| NP_001070909.1 | 9774\|\|9774 | bcl-2-associated transcription factor 1 isoform 2\|\|bcl-2-associated transcription factor 1 isoform 3 | **S175\|\|S177** | | | #N/A | KAEGEPQEEsPLK | 3 | 3 | 2 | 1 | K1(Label:13C(6)15N(2)); S10(Phospho); K13(Label:13C(6)15N(2)) | S(10): 100.0 | EGEPQEEsPLKSKSQ\|\|EGEPQEEsPLKSKSQ | S10; | 1537.7 | No\|\|Yes |
| **BCLAF1\|\|BCLAF1** | NP_001070908.1\|\| NP_001070909.1 | 9774\|\|9774 | bcl-2-associated transcription factor 1 isoform 2\|\|bcl-2-associated transcription factor 1 isoform 3 | **S175\|\|S177** | | | #N/A | KAEGEPQEEsPLK | 2 | 3 | 2 | 1 | S10(Phospho) | S(10): 100.0 | EGEPQEEsPLKSKSQ\|\|EGEPQEEsPLKSKSQ | S10; | 1521.7 | No\|\|Yes |
| **BCLAF1\|\|BCLAF1** | NP_001070908.1\|\| NP_001070909.1 | 9774\|\|9774 | bcl-2-associated transcription factor 1 isoform 2\|\|bcl-2-associated transcription factor 1 isoform 3 | **S529\|\|S358** | | | #N/A | EEsPLRIK | 1 | 3 | 2 | 1 | S3(Phospho); R6(Label:13C(6)); K8(Label:13C(6)15N(2)) | S(3): 100.0 | KSTFREEsPLRIKMI\|\|KSTFREEsPLRIKMI | S3; | 1065.6 | No\|\|No |
| **BCLAF1\|\|BCLAF1\|\|THRAP3** | NP_001070908.1\|\| NP_001070909.1\|\| NP_005110.2 | 9774\|\|9774\|\|9967 | bcl-2-associated transcription factor 1 isoform 2\|\|bcl-2-associated transcription factor 1 isoform 3\|\|thyroid hormone receptor-associated protein 3 | **S646\|\|S475\|\|S672** | | | #N/A | sPEIHR | 3 | 4 | 3 | 0 | S1(Phospho) | S(1): 100.0 | EHSTRQKsPEIHRRI\|\|EHSTRQKsPEIHRRI\|\|EAAKNKKsPEIHRRI | S1; | 818.4 | No\|\|No\|\|Yes |
| **BCOR** | NP_001116856.1 | 54880 | BCL-6 corepressor isoform b | **S173** | | | #N/A | SAVATAEALGLDRPAsDKQSPLNINGASYLR | 1 | 3 | 1 | 1 | T5(Phospho); K18(Label:13C(6)15N(2)); S28(Phospho) | S(1): 0.6; T(5): 0.6; S(16): 91.6; S(20): 7.1; S(28): 50.0; Y(29): 50.0 | LGLDRPAsDKQSPLN | S16; | 3353.6 | No |
| **BET1L** | NP_057610.2 | 51272 | BET1-like protein isoform 2 | **S9** | | 0.77 | 0.83 | AQsPGAVEEILDRENKR | 2 | 2 | 1 | 2 | S3(Phospho) | S(3): 100.0 | ADWARAQsPGAVEEI | S3; | 1992.0 | Yes |
| **BICD2** | NP_056065.1 | 23299 | protein bicaudal D homolog 2 isoform 2 | **S582** | 1.03 | 1.07 | 0.87 | sPILLPK | 6 | 2 | 1 | 0 | S1(Phospho) | S(1): 100.0 | PEARGRRsPILLPKG | S1; | 847.5 | Yes |
| **BICD2** | NP_056065.1 | 23299 | protein bicaudal D homolog 2 isoform 2 | **S582** | | | 0.87 | sPILLPK | 4 | 2 | 1 | 0 | S1(Phospho); K7(Label:13C(6)15N(2)) | S(1): 100.0 | PEARGRRsPILLPKG | S1; | 855.5 | Yes |
| **BIN1** | NP_647598.1 | 274 | myc box-dependent-interacting protein 1 isoform 6 | **S300** | | 1.6 | #N/A | VNHEPEPAGGATPGATLPKsPSQLR | 8 | 4 | 1 | 1 | S20(Phospho) | T(12): 0.0; T(16): 0.0; S(20): 100.0; S(22): 0.0 | PGATLPKsPSQLRKG | S20; | 2591.3 | Yes |
| **BIN1** | NP_647598.1 | 274 | myc box-dependent-interacting protein 1 isoform 6 | **S300** | | 1.8 | #N/A | VNHEPEPAGGATPGATLPKsPSQLR | 5 | 4 | 1 | 1 | K19(Label:13C(6)15N(2)); S20(Phospho); R25(Label:13C(6)) | T(12): 0.0; T(16): 0.0; S(20): 98.5; S(22): 1.5 | PGATLPKsPSQLRKG | S20; | 2605.3 | Yes |
| **BLNK** | NP_001245369.1 | 29760 | B-cell linker protein isoform 3 | **S214; S215** | | | #N/A | APMVNRSTKPNssTPASPPGTASGR | 1 | 2 | 1 | 1 | S12(Phospho); S13(Phospho) | S(7): 12.8; T(8): 12.8; S(12): 86.9; S(13): 86.9; T(14): 0.6; S(17): 0.0; T(21): 0.0; S(23): 0.0 | NRSTKPNsSTPASPP; RSTKPNSsTPASPPG | S12; S13; | 2628.2 | No;No |
| **BLOC1S3** | NP_997715.1 | 388552 | biogenesis of lysosome-related organelles complex 1 subunit 3 | **T63; S65** | | 1.78 | 1.55 | VAGEAAEtDsEPEPEPEPTAAPR | 6 | 1 | 1 | 0 | T8(Phospho); S10(Phospho) | T(8): 100.0; S(10): 100.0; T(19): 0.0 | VAGEAAEtDSEPEPE; GEAAETDsEPEPEPE | T8; S10; | 2510.0 | Yes;Yes |
| **BLOC1S3** | NP_997715.1 | 388552 | biogenesis of lysosome-related organelles complex 1 subunit 3 | **T63; S65** | | | 1.55 | VAGEAAEtDsEPEPEPEPTAAPR | 1 | 1 | 1 | 0 | T8(Phospho); S10(Phospho); R23(Label:13C(6)) | T(8): 100.0; S(10): 100.0; T(19): 0.0 | VAGEAAEtDSEPEPE; GEAAETDsEPEPEPE | T8; S10; | 2516.0 | Yes;Yes |
| **BMS1** | NP_055568.3 | 9790 | ribosome biogenesis protein BMS1 homolog | **S552** | 0.57 | 0.55 | 0.61 | AGLsPANCQSDRVNLEK | 4 | 1 | 1 | 1 | S4(Phospho); C8(Carbamidomethyl) | S(4): 100.0; S(10): 0.0 | EGSKAGLsPANCQSD | S4; | 1938.9 | Yes |
| **BMS1** | NP_055568.3 | 9790 | ribosome biogenesis protein BMS1 homolog | **S552** | 0.63 | 0.63 | 0.61 | AGLsPANCQSDRVNLEK | 4 | 1 | 1 | 1 | S4(Phospho); C8(Carbamidomethyl); R12(Label:13C(6)); K17(Label:13C(6)15N(2)) | S(4): 100.0; S(10): 0.0 | EGSKAGLsPANCQSD | S4; | 1952.9 | Yes |
| **BOD1L1** | NP_683692.2 | 259282 | biorientation of chromosomes in cell division protein 1-like 1 | **T779** | | | 1.36 | VEENIQKQSQQtKLSSDDK | 1 | 1 | 1 | 2 | K7(Label:13C(6)15N(2)); T12(Phospho) | S(9): 0.2; T(12): 99.4; S(15): 0.2; S(16): 0.2 | IQKQSQQtKLSSDDK | T12; | 2293.1 | No |
| **BOD1L1** | NP_683692.2 | 259282 | biorientation of chromosomes in cell division protein 1-like 1 | **S3029** | | 1.13 | 1.36 | REVsPPGAR | 4 | 1 | 1 | 1 | S4(Phospho) | S(4): 100.0 | IKRKREVsPPGARTR | S4; | 1048.5 | Yes |
| **BOD1L1** | NP_683692.2 | 259282 | biorientation of chromosomes in cell division protein 1-like 1 | **S3029** | | 1.24 | 1.36 | EVsPPGAR | 1 | 1 | 1 | 0 | S3(Phospho) | S(3): 100.0 | IKRKREVsPPGARTR | S3; | 892.4 | Yes |
| **BOLA1** | NP_057158.1 | 51027 | bolA-like protein 1 precursor | **S81** | | 0.57 | 1.15 | FEGLsPLQR | 1 | 1 | 1 | 0 | S5(Phospho) | S(5): 100.0 | SSRFEGLsPLQRHRL | S5; | 1126.5 | Yes |
| **BOLA1** | NP_057158.1 | 51027 | bolA-like protein 1 precursor | **S81** | | 0.59 | 1.15 | FEGLsPLQR | 1 | 1 | 1 | 0 | S5(Phospho); R9(Label:13C(6)) | S(5): 100.0 | SSRFEGLsPLQRHRL | S5; | 1132.5 | Yes |
| **BOP1** | NP_056016.1 | 23246 | ribosome biogenesis protein BOP1 | **S126; S127** | | 0.88 | 1.15 | IGDEYAEDssDEEDIR | 2 | 1 | 1 | 0 | S9(Phospho); S10(Phospho) | Y(5): 0.0; S(9): 100.0; S(10): 100.0 | GDEYAEDsSDEEDIR; DEYAEDSsDEEDIRN | S9; S10; | 2002.7 | Yes;Yes |
| **BOP1** | NP_056016.1 | 23246 | ribosome biogenesis protein BOP1 | **S126; S127** | | 0.91 | 1.15 | IGDEYAEDssDEEDIR | 1 | 1 | 1 | 0 | S9(Phospho); S10(Phospho); R16(Label:13C(6)) | Y(5): 0.0; S(9): 100.0; S(10): 100.0 | GDEYAEDsSDEEDIR; DEYAEDSsDEEDIRN | S9; S10; | 2008.7 | Yes;Yes |
| **BPTF** | NP_004450.3 | 2186 | nucleosome-remodeling factor subunit BPTF isoform 2 | **S263** | 0.32 | 0.26 | 0.45 | LsPFR | 2 | 2 | 1 | 0 | S2(Phospho) | S(2): 100.0 | FGTVLRLsPFRFEDF | S2; | 699.3 | No |
| **BPTF** | NP_004450.3 | 2186 | nucleosome-remodeling factor subunit BPTF isoform 2 | **S1297** | | | 0.45 | DRVLDDVsIRSPETK | 2 | 2 | 1 | 2 | R2(Label:13C(6)); S8(Phospho); R10(Label:13C(6)); K15(Label:13C(6)15N(2)) | S(8): 97.6; S(11): 2.4; T(14): 0.0 | DRVLDDVsIRSPETK | S8; | 1829.9 | Yes |
| **BPTF** | NP_004450.3 | 2186 | nucleosome-remodeling factor subunit BPTF isoform 2 | **S216** | | 0.62 | 0.45 | sPILEEKDIPPLEFPK | 2 | 2 | 1 | 1 | S1(Phospho) | S(1): 100.0 | PRVHRPRsPILEEKD | S1; | 1932.0 | Yes |
| **BPTF** | NP_004450.3 | 2186 | nucleosome-remodeling factor subunit BPTF isoform 2 | **S216** | | 0.52 | 0.45 | sPILEEK | 3 | 2 | 1 | 0 | S1(Phospho); K7(Label:13C(6)15N(2)) | S(1): 100.0 | PRVHRPRsPILEEKD | S1; | 903.4 | Yes |
| **BPTF** | NP_004450.3 | 2186 | nucleosome-remodeling factor subunit BPTF isoform 2 | **S216** | | 0.64 | 0.45 | sPILEEKDIPPLEFPK | 2 | 2 | 1 | 1 | S1(Phospho); K7(Label:13C(6)15N(2)); K16(Label:13C(6)15N(2)) | S(1): 100.0 | PRVHRPRsPILEEKD | S1; | 1948.0 | Yes |
| **BPTF** | NP_004450.3 | 2186 | nucleosome-remodeling factor subunit BPTF isoform 2 | **S216** | | | 0.45 | sPILEEK | 2 | 2 | 1 | 0 | S1(Phospho) | S(1): 100.0 | PRVHRPRsPILEEKD | S1; | 895.4 | Yes |
| **BPTF** | NP_004450.3 | 2186 | nucleosome-remodeling factor subunit BPTF isoform 2 | **T1303** | | 0.5 | 0.45 | VLDDVSIRSPEtK | 3 | 2 | 1 | 1 | S9(Phospho) | S(6): 0.0; S(9): 1.0; T(12): 99.0 | VSIRSPEtKCPKQNS | T12; | 1538.7 | Yes |
| **BPTF** | NP_004450.3 | 2186 | nucleosome-remodeling factor subunit BPTF isoform 2 | **S1300** | | | 0.45 | DRVLDDVSIRsPETK | 1 | 2 | 1 | 2 | S8(Phospho) | S(8): 2.1; S(11): 97.9; T(14): 0.0 | LDDVSIRsPETKCPK | S11; | 1809.9 | Yes |
| **BPTF** | NP_004450.3 | 2186 | nucleosome-remodeling factor subunit BPTF isoform 2 | **S1300** | | | 0.45 | VLDDVSIRsPETK | 1 | 2 | 1 | 1 | R8(Label:13C(6)); S9(Phospho); K13(Label:13C(6)15N(2)) | S(6): 0.0; S(9): 100.0; T(12): 0.0 | LDDVSIRsPETKCPK | S9; | 1552.8 | Yes |
| **BRAF** | NP_004324.2 | 673 | serine/threonine-protein kinase B-raf | **S419** | 1.68 | | #N/A | ALQKsPGPQR | 1 | 1 | 1 | 1 | S5(Phospho) | S(5): 100.0 | NVKALQKsPGPQRER | S5; | 1161.6 | Yes |
| **BRAF** | NP_004324.2 | 673 | serine/threonine-protein kinase B-raf | **S446** | | 1.08 | #N/A | RDsSDDWEIPDGQITVGQR | 2 | 1 | 1 | 1 | S3(Phospho) | S(3): 98.7; S(4): 1.3; T(15): 0.0 | KTLGRRDsSDDWEIP | S3; | 2254.0 | Yes |
| **BRAF** | NP_004324.2 | 673 | serine/threonine-protein kinase B-raf | **S729** | | 1.42 | #N/A | SAsEPSLNR | 1 | 1 | 1 | 0 | S3(Phospho) | S(1): 0.0; S(3): 100.0; S(6): 0.0 | PKIHRSAsEPSLNRA | S3; | 1040.4 | Yes |
| **BRAF** | NP_004324.2 | 673 | serine/threonine-protein kinase B-raf | **S729** | | 1.47 | #N/A | SAsEPSLNR | 1 | 1 | 1 | 0 | S3(Phospho); R9(Label:13C(6)) | S(1): 0.0; S(3): 100.0; S(6): 0.0 | PKIHRSAsEPSLNRA | S3; | 1046.5 | Yes |
| **BRD1** | NP_055392.1 | 23774 | bromodomain-containing protein 1 | **S128** | 1.16 | 1.22 | #N/A | IVEYsPPSAPR | 3 | 1 | 1 | 0 | S5(Phospho); R11(Label:13C(6)) | Y(4): 0.0; S(5): 100.0; S(8): 0.0 | KVRIVEYsPPSAPRR | S5; | 1301.6 | Yes |
| **BRD1** | NP_055392.1 | 23774 | bromodomain-containing protein 1 | **S128** | | | #N/A | IVEYsPPSAPR | 3 | 1 | 1 | 0 | S5(Phospho) | Y(4): 0.0; S(5): 100.0; S(8): 0.0 | KVRIVEYsPPSAPRR | S5; | 1295.6 | Yes |
| **BRD1** | NP_055392.1 | 23774 | bromodomain-containing protein 1 | **S1052; S1055** | | | #N/A | VHGEPTsDLsDID | 2 | 1 | 1 | 0 | S7(Phospho); S10(Phospho) | T(6): 1.7; S(7): 98.3; S(10): 100.0 | RVHGEPTsDLSDID_;GEPTSDLsDID_ | S7; S10; | 1544.6 | Yes;Yes |
| **BRD2** | NP_001278915.1 | 6046 | bromodomain-containing protein 2 isoform 4 | **S178; S185** | | | 1.54 | ADTTTPTPTAILAPGsPASPPGsLEPK | 1 | 4 | 1 | 0 | S16(Phospho); S19(Phospho) | T(3): 0.0; T(4): 0.0; T(5): 0.0; T(7): 0.0; T(9): 0.0; S(16): 100.0; S(19): 4.9; S(23): 95.1 | TAILAPGsPASPPGS;SPASPPGsLEPKAAR | S16; S23; | 2734.3 | No;No |
| **BRD2** | NP_001278915.1 | 6046 | bromodomain-containing protein 2 isoform 4 | **S178** | | | 1.54 | KADTTTPTPTAILAPGsPASPPGSLEPK | 1 | 4 | 1 | 1 | S17(Phospho); S20(Phospho) | T(4): 0.0; T(5): 0.0; T(6): 0.0; T(8): 0.0; T(10): 0.0; S(17): 100.0; S(20): 50.0; S(24): 50.0 | TAILAPGsPASPPGS | S17; | 2862.4 | No |
| **BRD2** | NP_001278915.1 | 6046 | bromodomain-containing protein 2 isoform 4 | **S178; S185** | | | 1.54 | RKADTTTPTPTAILAPGsPASPPGsLEPK | 1 | 4 | 1 | 2 | S18(Phospho); S21(Phospho) | T(5): 0.0; T(6): 0.0; T(7): 0.0; T(9): 0.0; T(11): 0.0; S(18): 100.0; S(21): 2.8; S(25): 97.2 | TAILAPGsPASPPGS;SPASPPGsLEPKAAR | S18; S25; | 3018.5 | No;No |
| **BRD3** | NP_031397.1 | 8019 | bromodomain-containing protein 3 | **S263** | | 0.91 | 0.56 | SEsPPPLSDPK | 3 | 1 | 1 | 0 | S3(Phospho); K11(Label:13C(6)15N(2)) | S(1): 0.0; S(3): 100.0; S(8): 0.0 | ITASRSEsPPPLSDP | S3; | 1241.6 | Yes |
| **BRD3** | NP_031397.1 | 8019 | bromodomain-containing protein 3 | **S263** | | 0.94 | 0.56 | SEsPPPLSDPK | 5 | 1 | 1 | 0 | S3(Phospho) | S(1): 0.0; S(3): 100.0; S(8): 0.0 | ITASRSEsPPPLSDP | S3; | 1233.5 | Yes |
| **BRD4** | NP_490597.1 | 23476 | bromodomain-containing protein 4 isoform long | **S470** | 2.15 | 2.15 | 1.56 | FAKMPDEPEEPVVAVSsPAVPPPTK | 7 | 2 | 1 | 1 | S17(Phospho) | S(16): 0.0; S(17): 100.0; T(24): 0.0 | EPVVAVSsPAVPPPT | S17; | 2699.3 | Yes |
| **BRD4** | NP_490597.1 | 23476 | bromodomain-containing protein 4 isoform long | **S470** | 2.2 | 3.45 | 1.56 | MPDEPEEPVVAVSsPAVPPPTK | 10 | 2 | 1 | 0 | S14(Phospho) | S(13): 1.7; S(14): 98.3; T(21): 0.0 | EPVVAVSsPAVPPPT | S14; | 2353.1 | Yes |
| **BRD4** | NP_490597.1 | 23476 | bromodomain-containing protein 4 isoform long | **S1083** | | | 1.56 | EAPSPLMIHSPQMSQFQSLTHQsPPQQNVQPK | 4 | 1 | 1 | 0 | S23(Phospho) | S(4): 0.0; S(10): 0.0; S(14): 0.0; S(18): 0.0; T(20): 0.1; S(23): 99.9 | FQSLTHQsPPQQNVQ | S23; | 3677.7 | Yes |
| **BRD4** | NP_490597.1 | 23476 | bromodomain-containing protein 4 isoform long | **S1117** | 0.59 | | 1.56 | IHsPIIR | 1 | 1 | 1 | 0 | S3(Phospho); R7(Label:13C(6)) | S(3): 100.0 | VKEEKIHsPIIRSEP | S3; | 921.5 | Yes |
| **BRD4** | NP_490597.1 | 23476 | bromodomain-containing protein 4 isoform long | **S1117** | | 0.87 | 1.56 | IHsPIIR | 2 | 1 | 1 | 0 | S3(Phospho) | S(3): 100.0 | VKEEKIHsPIIRSEP | S3; | 915.5 | Yes |
| **BRD8** | NP_001157798.1 | 10902 | bromodomain-containing protein 8 isoform 4 | **T264** | 0.84 | | #NQ | AtPPPSPLLSELLKK | 4 | 2 | 1 | 1 | T2(Phospho) | T(2): 100.0; S(6): 0.0; S(10): 0.0 | KMLGQKAtPPPSPLL | T2; | 1670.9 | Yes |
| **BRD8** | NP_001157798.1 | 10902 | bromodomain-containing protein 8 isoform 4 | **T264** | 0.88 | | #NQ | AtPPPSPLLSELLKK | 4 | 2 | 1 | 1 | T2(Phospho); K14(Label:13C(6)15N(2)); K15(Label:13C(6)15N(2)) | T(2): 100.0; S(6): 0.0; S(10): 0.0 | KMLGQKAtPPPSPLL | T2; | 1686.9 | Yes |
| **BRD8** | NP_001157798.1 | 10902 | bromodomain-containing protein 8 isoform 4 | **T264** | | 0.92 | #NQ | AtPPPSPLLSELLK | 2 | 2 | 1 | 0 | T2(Phospho); K14(Label:13C(6)15N(2)) | T(2): 100.0; S(6): 0.0; S(10): 0.0 | KMLGQKAtPPPSPLL | T2; | 1550.8 | Yes |
| **BRD8** | NP_001157798.1 | 10902 | bromodomain-containing protein 8 isoform 4 | **T264** | | 3.7 | #NQ | AtPPPSPLLSELLK | 2 | 2 | 1 | 0 | T2(Phospho) | T(2): 100.0; S(6): 0.0; S(10): 0.0 | KMLGQKAtPPPSPLL | T2; | 1542.8 | Yes |
| **BRD8** | NP_001157798.1 | 10902 | bromodomain-containing protein 8 isoform 4 | **T264; S268** | | 2.29 | #NQ | AtPPPsPLLSELLKK | 1 | 2 | 1 | 1 | T2(Phospho); S6(Phospho) | T(2): 100.0; S(6): 100.0; S(10): 0.0 | KMLGQKAtPPPSPLL;QKATPPPsPLLSELL | T2; S6; | 1750.9 | Yes;Yes |
| **BRD8** | NP_001157798.1 | 10902 | bromodomain-containing protein 8 isoform 4 | **T264; S268** | | | #NQ | MLGQKAtPPPsPLLSELLK | 1 | 2 | 1 | 1 | K5(Label:13C(6)15N(2)); T7(Phospho); S11(Phospho); K19(Label:13C(6)15N(2)) | T(7): 100.0; S(11): 100.0; S(15): 0.0 | KMLGQKAtPPPSPLL;QKATPPPsPLLSELL | T7; S11; | 2196.1 | Yes;Yes |
| **BRD9** | NP_001009877.2 | 65980 | bromodomain-containing protein 9 isoform 2 | **S513** | 0.81 | 0.93 | #N/A | DQHHLGsPSR | 2 | 2 | 1 | 0 | S7(Phospho) | S(7): 100.0; S(9): 0.0 | RDQHHLGsPSRLSVG | S7; | 1213.5 | No |
| **BRF2** | NP_060780.2 | 55290 | transcription factor IIIB 50 kDa subunit | **S353** | | 1.01 | #N/A | RPAsPALLLPPCMLK | 2 | 1 | 1 | 0 | S4(Phospho); C12(Carbamidomethyl) | S(4): 100.0 | PQGKRPAsPALLLPP | S4; | 1743.9 | Yes |

Supplementary Table S5:List of phosphopeptides identified in SCC-S and SCC-R cells using SequestHT and Mascot search algorithms

| GeneSymbol | NP_Accession | Gene ID | ProteinDescription | PhosphoSite (Protein) | SCC-R / SCC-S R1 | SCC-R / SCC-S R2 | SCC-R / SCC-S Total Proteome | Sequence | # PSMs | # Proteins | # Protein Groups | # Missed Cleavages | Modifications | phosphoRS Site Probabilities | Phosphowindow | PhosphoSite (Peptide) | MH+ [Da] | PhosphositePlusEvidence |
|---|---|---|---|---|---|---|---|---|---|---|---|---|---|---|---|---|---|---|
| **BRF2** | NP_060780.2 | 55290 | transcription factor IIIB 50 kDa subunit | **S353** | | | #N/A | RPAsPALLLPPCMLK | 1 | 1 | 1 | 0 | R1(Label:13C(6)); S4(Phospho); C12(Carbamidomethyl); K15(Label:13C(6)15N(2)) | S(4): 100.0 | PQGKRPAsPALLLPP | S4; | 1757.9 | Yes |
| **BRIP1** | NP_114432.2 | 83990 | Fanconi anemia group J protein | **T1020** | | | #N/A | IPKAtPELGSSENSASSPPR | 2 | 1 | 1 | 1 | T5(Phospho) | T(5): 100.0; S(10): 0.0; S(11): 0.0; S(14): 0.0; S(16): 0.0; S(17): 0.0 | TGKIPKAtPELGSSE | T5; | 2105.0 | Yes |
| **BRPF1** | NP_004625.2 | 7862 | peregrin isoform 2 | **S1076** | | 2.07 | #N/A | GAGWLsEDEDSPLDALDLVWAK | 1 | 2 | 1 | 0 | S11(Phospho) | S(6): 97.1; S(11): 2.9 | GRGAGWLsEDEDSPL | S6; | 2467.1 | Yes |
| **BRPF1** | NP_004625.2 | 7862 | peregrin isoform 2 | **S1076** | | 0.99 | #N/A | GAGWLsEDEDSPLDALDLVWAK | 1 | 2 | 1 | 0 | S11(Phospho); K22(Label:13C(6)15N(2)) | S(6): 99.9; S(11): 0.1 | GRGAGWLsEDEDSPL | S6; | 2475.1 | Yes |
| **BRSK2** | NP_001269147.1 | 9024 | serine/threonine-protein kinase BRSK2 isoform 5 | **S379** | | | #N/A | VTPHPSPRGsPLPTPKGTPVHTPK | 1 | 5 | 1 | 2 | R8(Label:13C(6)); K16(Label:13C(6)15N(2)); T18(Phospho) | T(2): 0.0; S(6): 4.7; S(10): 85.4; T(14): 4.7; T(18): 4.7; T(22): 0.3 | PHPSPRGsPLPTPKG | S10; | 2579.4 | No |
| **BRWD1** | NP_387505.1 | 54014 | bromodomain and WD repeat-containing protein 1 isoform B | **S696** | 1.5 | | #N/A | RLsLDIQSPPNIGLR | 2 | 2 | 1 | 1 | S3(Phospho) | S(3): 100.0; S(8): 0.0 | RRGFRRLsLDIQSPP | S3; | 1758.9 | Yes |
| **BRWD1** | NP_387505.1 | 54014 | bromodomain and WD repeat-containing protein 1 isoform B | **S1475** | | | #N/A | IIPELVGsPTQSTSSR | 1 | 2 | 1 | 0 | S8(Phospho); R16(Label:13C(6)) | S(8): 100.0; T(10): 0.0; S(12): 0.0; T(13): 0.0; S(14): 0.0; S(15): 0.0 | IIPELVGsPTQSTSS | S8; | 1757.9 | Yes |
| **BTF3** | NP_001198.2 | 689 | transcription factor BTF3 isoform B | **S114** | 1.09 | 1.01 | **0.39** | QLTEMLPSILNQLGADsLTSLR | 2 | 2 | 1 | 0 | S17(Phospho); R22(Label:13C(6)) | T(3): 0.0; S(8): 0.0; S(17): 100.0; T(19): 0.0; S(20): 0.0 | LNQLGADsLTSLRRL | S17; | 2486.3 | No |
| **BTF3** | NP_001198.2 | 689 | transcription factor BTF3 isoform B | **S114** | | | **0.39** | QLTEMLPSILNQLGADsLTSLRR | 1 | 2 | 1 | 1 | S17(Phospho); R22(Label:13C(6)); R23(Label:13C(6)) | T(3): 0.0; S(8): 0.0; S(17): 97.7; T(19): 2.3; S(20): 0.1 | LNQLGADsLTSLRRL | S17; | 2648.4 | No |
| **BUB1B** | NP_001202.4 | 701 | mitotic checkpoint serine/threonine-protein kinase BUB1 beta | **S670** | | **0.41** | #N/A | KLsPIIEDSR | 2 | 1 | 1 | 1 | S3(Phospho) | S(3): 100.0; S(9): 0.0 | TLSIKKLsPIIEDSR | S3; | 1237.6 | Yes |
| **BUB1B** | NP_001202.4 | 701 | mitotic checkpoint serine/threonine-protein kinase BUB1 beta | **S670** | | | #N/A | KLsPIIEDSR | 1 | 1 | 1 | 1 | K1(Label:13C(6)15N(2)); S3(Phospho); R10(Label:13C(6)) | S(3): 100.0; S(9): 0.0 | TLSIKKLsPIIEDSR | S3; | 1251.7 | Yes |
| **BUD13** | NP_116114.1 | 84811 | BUD13 homolog isoform 1 | **S358** | 0.85 | | #N/A | ATDSDLSsPR | 2 | 2 | 1 | 0 | S8(Phospho) | T(2): 0.0; S(4): 0.0; S(7): 0.7; S(8): 99.3 | ATDSDLSsPRHKQSP | S8; | 1128.5 | Yes |
| **BUD13** | NP_116114.1 | 84811 | BUD13 homolog isoform 1 | **S271** | 1.09 | | #N/A | ARHDsPDLAPNVTYSLPR | 4 | 1 | 1 | 1 | S5(Phospho) | S(5): 100.0; T(13): 0.0; Y(14): 0.0; S(15): 0.0 | LRRARHDsPDLAPNV | S5; | 2089.0 | Yes |
| **BUD13** | NP_116114.1 | 84811 | BUD13 homolog isoform 1 | **S197; S201** | | | #N/A | HDsPDPsPPR | 1 | 2 | 1 | 0 | S3(Phospho); S7(Phospho) | S(3): 100.0; S(7): 100.0 | PRRARHDsPDPSPPR; RHDSPDPsPPRRPQH | S3; S7; | 1264.4 | Yes;Yes |
| **BUD13** | NP_116114.1 | 84811 | BUD13 homolog isoform 1 | **S240** | 0.76 | | #N/A | HGSSDISsPR | 2 | 1 | 1 | 0 | S8(Phospho) | S(3): 0.0; S(4): 0.0; S(7): 1.0; S(8): 99.0 | HGSSDISsPRRVHNN | S8; | 1122.5 | Yes |
| **BUD13** | NP_116114.1 | 84811 | BUD13 homolog isoform 1 | **S271** | | | #N/A | ARHDsPDLAPNVTYSLPR | 3 | 1 | 1 | 1 | R2(Label:13C(6)); S5(Phospho); R18(Label:13C(6)) | S(5): 100.0; T(13): 0.0; Y(14): 0.0; S(15): 0.0 | LRRARHDsPDLAPNV | S5; | 2101.0 | Yes |
| **BUD13** | NP_116114.1 | 84811 | BUD13 homolog isoform 1 | **T123** | 0.9 | | #N/A | HFRHDtPDSSPR | 1 | 2 | 1 | 1 | T6(Phospho); S10(Phospho) | T(6): 100.0; S(9): 50.0; S(10): 50.0 | NRHFRHDtPDSSPRR | T6; | 1611.6 | Yes |
| **BUD13** | NP_116114.1 | 84811 | BUD13 homolog isoform 1 | **S271** | | 1.19 | #N/A | HDsPDLAPNVTYSLPR | 2 | 1 | 1 | 0 | S3(Phospho) | S(3): 100.0; T(11): 0.0; Y(12): 0.0; S(13): 0.0 | LRRARHDsPDLAPNV | S3; | 1861.8 | Yes |
| **BUD13** | NP_116114.1 | 84811 | BUD13 homolog isoform 1 | **T135** | | 1.3 | #N/A | VRHGtPDPSPR | 1 | 2 | 1 | 1 | T5(Phospho) | T(5): 100.0; S(9): 0.0 | PRRVRHGtPDPSPRK | T5; | 1298.6 | Yes |
| **BUD13** | NP_116114.1 | 84811 | BUD13 homolog isoform 1 | **T147; S151** | | 1.05 | #N/A | KDRHDtPDPsPR | 2 | 2 | 1 | 2 | T6(Phospho); S10(Phospho) | T(6): 100.0; S(10): 100.0 | PRKDRHDtPDPSPRR; RHDTPDPsPRRARHD | T6; S10; | 1580.6 | Yes;Yes |
| **BUD13** | NP_116114.1 | 84811 | BUD13 homolog isoform 1 | **S271** | | | #N/A | HDsPDLAPNVTYSLPR | 1 | 1 | 1 | 0 | S3(Phospho); R16(Label:13C(6)) | S(3): 100.0; T(11): 0.0; Y(12): 0.0; S(13): 0.0 | LRRARHDsPDLAPNV | S3; | 1867.9 | Yes |
| **BUD13** | NP_116114.1 | 84811 | BUD13 homolog isoform 1 | **T159; S163** | | 1.17 | #N/A | HDtPDPsPLR | 1 | 2 | 1 | 0 | T3(Phospho); S7(Phospho) | T(3): 100.0; S(7): 100.0 | PRRARHDtPDPSPLR; RHDTPDPsPLRGARH | T3; S7; | 1294.5 | Yes;Yes |
| **BYSL** | NP_004044.3 | 705 | bystin | **S98** | | 0.71 | 0.55 | MPQDGsDDEDEEWPTLEK | 1 | 1 | 1 | 0 | S6(Phospho); K18(Label:13C(6)15N(2)) | S(6): 100.0; T(15): 0.0 | PRMPQDGsDDEDEEW | S6; | 2208.8 | Yes |
| **BZW1** | NP_055485.2 | 9689 | basic leucine zipper and W2 domain-containing protein 1 isoform 4 | **S345** | | | 1.19 | NAEEEsESEAEEGD | 2 | 4 | 1 | 0 | S6(Phospho) | S(6): 100.0; S(8): 0.0 | LKNAEEEsESEAEEG | S6; | 1604.5 | No |
| **C10orf11** | NP_114413.1 | 83938 | leucine-rich repeat-containing protein C10orf11 | **S153** | 6.16 | | #NQ | ASSEDVASsPER | 2 | 1 | 1 | 0 | S9(Phospho) | S(2): 0.0; S(3): 0.0; S(8): 0.9; S(9): 99.1 | SSEDVASsPERHYTP | S9; | 1314.5 | Yes |
| **C10orf12** | NP_056467.2 | 26148 | uncharacterized protein C10orf12 | **S273** | | 0.58 | #N/A | HTVDVQLPREDNPEEPSKEITSHEEGGGDVsPR | 1 | 1 | 1 | 2 | R9(Label:13C(6)); K18(Label:13C(6)15N(2)); S31(Phospho); R33(Label:13C(6)) | T(2): 0.0; S(17): 0.0; T(21): 0.0; S(22): 0.0; S(31): 100.0 | EEGGGDVsPRKEPQE | S31; | 3740.7 | Yes |
| **C10orf12** | NP_056467.2 | 26148 | uncharacterized protein C10orf12 | **S294** | | **0.44** | #N/A | IKPNLSsSPR | 1 | 1 | 1 | 0 | K2(Label:13C(6)15N(2)); S8(Phospho); R10(Label:13C(6)) | S(6): 0.7; S(7): 98.5; S(8): 0.7 | KIKPNLSsSPRSEET | S7; | 1192.6 | Yes |
| **C10orf54** | NP_071436.1 | 64115 | platelet receptor Gi24 precursor | **S305** | | | #N/A | HLLSEPSTPLSPPGPGDVFFPSLDPVPDsPNFEVI | 1 | 1 | 1 | 0 | S29(Phospho) | S(4): 0.0; S(7): 0.0; T(8): 0.0; S(11): 0.0; S(22): 0.0; S(29): 100.0 | SLDPVPDsPNFEVI_ | S29; | 3780.8 | Yes |
| **C10orf90** | NP_001004298.2 | 118611 | centrosomal protein C10orf90 | **T571** | | | #N/A | SLtLQEALEVR | 8 | 1 | 1 | 0 | T3(Phospho) | S(1): 0.0; T(3): 100.0 | QTPARSLtLQEALEV | T3; | 1338.7 | No |
| **C11orf30** | NP_064578.2 | 56946 | protein EMSY | **S1213** | 0.84 | 0.83 | #N/A | ESCSsPSTVGSSLTTR | 4 | 1 | 1 | 0 | C3(Carbamidomethyl); S5(Phospho) | S(2): 0.0; S(4): 0.0; S(5): 100.0; S(7): 0.0; T(8): 0.0; S(11): 0.0; S(12): 0.0; T(14): 0.0; T(15): 0.0 | KCRESCSsPSTVGSS | S5; | 1735.7 | No |
| **C11orf30** | NP_064578.2 | 56946 | protein EMSY | **S1213** | | | #N/A | ESCSsPSTVGSSLTTR | 1 | 1 | 1 | 0 | C3(Carbamidomethyl); S5(Phospho); R16(Label:13C(6)) | S(2): 1.2; S(4): 0.0; S(5): 98.7; S(7): 0.0; T(8): 0.0; S(11): 0.0; S(12): 0.0; T(14): 0.0; T(15): 0.0 | KCRESCSsPSTVGSS | S5; | 1741.7 | No |
| **C11orf30** | NP_064578.2 | 56946 | protein EMSY | **S238** | | **0.49** | #N/A | TITVPVSGsPK | 1 | 1 | 1 | 0 | S9(Phospho) | T(1): 0.0; T(3): 0.0; S(7): 1.3; S(9): 98.7 | ITVPVSGsPKMSNIM | S9; | 1165.6 | No |
| **C11orf58** | NP_055082.1 | 10944 | small acidic protein | **S17** | 1.85 | 2.06 | 1.51 | SAsPDDDLGSSNWEAADLGNEER | 10 | 1 | 1 | 0 | S3(Phospho) | S(1): 1.4; S(3): 98.6; S(10): 0.0; S(11): 0.0 | HGVKRSAsPDDDLGS | S3; | 2515.0 | Yes |
| **C11orf58** | NP_055082.1 | 10944 | small acidic protein | **S17** | 2.38 | 1.78 | 1.51 | SAsPDDDLGSSNWEAADLGNEERK | 4 | 1 | 1 | 1 | S3(Phospho) | S(1): 1.4; S(3): 98.6; S(10): 0.0; S(11): 0.0 | HGVKRSAsPDDDLGS | S3; | 2643.1 | Yes |
| **C11orf58** | NP_055082.1 | 10944 | small acidic protein | **S15** | 1.94 | 2.12 | 1.51 | sASPDDDLGSSNWEAADLGNEER | 4 | 1 | 1 | 0 | S3(Phospho); R23(Label:13C(6)) | S(1): 98.4; S(3): 1.6; S(10): 0.0; S(11): 0.0 | HPHGVKRsASPDDDL | S1; | 2521.0 | Yes |
| **C11orf58** | NP_055082.1 | 10944 | small acidic protein | **S17** | | | 1.51 | SAsPDDDLGSSNWEAADLGNEERK | 1 | 1 | 1 | 1 | S3(Phospho); R23(Label:13C(6)); K24(Label:13C(6)15N(2)) | S(1): 1.6; S(3): 98.4; S(10): 0.0; S(11): 0.0 | HGVKRSAsPDDDLGS | S3; | 2657.1 | Yes |
| **C11orf84** | NP_612480.1 | 144097 | uncharacterized protein C11orf84 | **S308** | 2.06 | 2.06 | #N/A | AEsPSPAPPPGLR | 6 | 1 | 1 | 0 | S3(Phospho) | S(3): 100.0; S(5): 0.0 | GGLPRAEsPSPAPPP | S3; | 1355.6 | Yes |
| **C11orf84** | NP_612480.1 | 144097 | uncharacterized protein C11orf84 | **S308** | | 2.13 | #N/A | AEsPSPAPPPGLR | 3 | 1 | 1 | 0 | S3(Phospho); R13(Label:13C(6)) | S(3): 100.0; S(5): 0.0 | GGLPRAEsPSPAPPP | S3; | 1361.7 | Yes |
| **C11orf84** | NP_612480.1 | 144097 | uncharacterized protein C11orf84 | **S310** | | 4.11 | #N/A | DREVAEGGLPRAESPsPAPPPGLR | 1 | 1 | 1 | 2 | S14(Phospho) | S(14): 1.6; S(16): 98.4 | LPRAESPsPAPPPGL | S16; | 2535.2 | Yes |
| **C12orf43** | NP_001273127.1 | 64897 | uncharacterized protein C12orf43 isoform g | **S138** | | | 0.59 | EAAVSASDILQESAIHsPGTVEKEAK | 2 | 7 | 1 | 1 | S17(Phospho); K23(Label:13C(6)15N(2)); K26(Label:13C(6)15N(2)) | S(5): 0.0; S(7): 0.0; S(13): 0.0; S(17): 100.0; T(20): 0.0 | LQESAIHsPGTVEKE | S17; | 2763.3 | No |
| **C12orf43** | NP_001273127.1 | 64897 | uncharacterized protein C12orf43 isoform g | **S138** | | 0.77 | 0.59 | EAAVSASDILQESAIHsPGTVEK | 2 | 7 | 1 | 0 | S17(Phospho); K23(Label:13C(6)15N(2)) | S(5): 0.0; S(7): 0.0; S(13): 0.0; S(17): 100.0; T(20): 0.0 | LQESAIHsPGTVEKE | S17; | 2427.2 | No |
| **C12orf45** | NP_689531.2 | 121053 | uncharacterized protein C12orf45 | **S178** | 0.55 | | #N/A | IEVLDsPASKK | 2 | 1 | 1 | 1 | S6(Phospho) | S(6): 100.0; S(9): 0.0 | GKIEVLDsPASKKKK | S6; | 1266.6 | Yes |
| **C12orf45** | NP_689531.2 | 121053 | uncharacterized protein C12orf45 | **S66** | | | #N/A | IERsPLLDQVQTFLPQMAR | 2 | 1 | 1 | 1 | S4(Phospho) | S(4): 100.0; T(12): 0.0 | QTVRIERsPLLDQVQ | S4; | 2322.2 | Yes |
| **C14orf93** | NP_001269898.1 | 60686 | uncharacterized protein C14orf93 isoform b | **S44** | | **0.24** | #NQ | QLsPATQLAIQR | 1 | 3 | 1 | 0 | S3(Phospho); R12(Label:13C(6)) | S(3): 100.0; T(6): 0.0 | PAYAKQLsPATQLAI | S3; | 1411.7 | No |
| **C15orf52** | NP_997263.2 | 388115 | uncharacterized protein C15orf52 | **S201** | 11.31 | | #N/A | VTRsPPTQVAISSDSAR | 10 | 1 | 1 | 1 | S4(Phospho) | T(2): 0.0; S(4): 100.0; T(7): 0.0; S(12): 0.0; S(13): 0.0; S(15): 0.0 | PGGRVTRsPPTQVAI | S4; | 1851.9 | Yes |
| **C15orf52** | NP_997263.2 | 388115 | uncharacterized protein C15orf52 | **S193** | | | #N/A | NQGIEGsPGGR | 2 | 1 | 1 | 0 | S7(Phospho) | S(7): 100.0 | RNQGIEGsPGGRVTR | S7; | 1151.5 | Yes |
| **C16orf62** | NP_064710.4 | 57020 | UPF0505 protein C16orf62 | **T249; S261** | | | 1.38 | tRLEELDDFEEGsQK | 1 | 1 | 1 | 1 | T1(Phospho); R2(Label:13C(6)); S13(Phospho) | T(1): 100.0; S(13): 100.0 | AMSEKVRtRLEELDD;LDDFEEGsQKELLNL | T1; S13; | 1961.8 | No;No |
| **C16orf71** | NP_631909.2 | 146562 | uncharacterized protein C16orf71 | **S454** | | | #N/A | GTAQPELPAsKGPAGGRAQAPEDTAGSR | 1 | 1 | 1 | 2 | S10(Phospho); K11(Label:13C(6)15N(2)); R28(Label:13C(6)) | T(2): 0.0; S(10): 100.0; T(24): 0.0; S(27): 0.0 | AQPELPAsKGPAGGR | S10; | 2771.3 | No |
| **C16orf74** | NP_996850.1 | 404550 | uncharacterized protein C16orf74 | **T44** | 1.47 | 1.48 | #N/A | HLDVPDIIITPPtPTGMMLPR | 9 | 1 | 1 | 0 | T13(Phospho) | T(10): 0.0; T(13): 100.0; T(15): 0.0 | DIIITPPtPTGMMLP | T13; | 2394.2 | Yes |
| **C16orf74** | NP_996850.1 | 404550 | uncharacterized protein C16orf74 | **T44** | | | #N/A | HLDVPDIIITPPtPTGMMLPR | 7 | 1 | 1 | 0 | T13(Phospho); M17(Oxidation) | T(10): 0.0; T(13): 98.7; T(15): 1.3 | DIIITPPtPTGMMLP | T13; | 2410.2 | Yes |
| **C16orf74** | NP_996850.1 | 404550 | uncharacterized protein C16orf74 | **T44** | | 1.29 | #N/A | HLDVPDIIITPPtPTGMMLPR | 3 | 1 | 1 | 0 | T13(Phospho); R21(Label:13C(6)) | T(10): 0.0; T(13): 100.0; T(15): 0.0 | DIIITPPtPTGMMLP | T13; | 2400.2 | Yes |
| **C16orf74** | NP_996850.1 | 404550 | uncharacterized protein C16orf74 | **T44** | | | #N/A | HLDVPDIIITPPtPTGMMLPR | 2 | 1 | 1 | 0 | T13(Phospho); M18(Oxidation); R21(Label:13C(6)) | T(10): 0.0; T(13): 100.0; T(15): 0.0 | DIIITPPtPTGMMLP | T13; | 2416.2 | Yes |
| **C17orf49** | NP_001136271.1 | 124944 | chromatin complexes subunit BAP18 isoform 3 | **S36** | | 0.83 | **0.34** | LGELTMQLHPVADSsPAGAQIK | 4 | 1 | 1 | 0 | S15(Phospho) | T(5): 0.0; S(14): 2.0; S(15): 98.0 | LHPVADSsPAGAQIK | S15; | 2343.1 | Yes |
| **C17orf49\|\|C17orf49** | NP_777553.1\|\|NP_001136271.1 | 124944\|\|124944 | chromatin complexes subunit BAP18 isoform 2\|\|chromatin complexes subunit BAP18 isoform 3 | **S96\|\|S62** | | | #N/A | VYEDSGIPLPAEsPKKGPK | 4 | 3 | 2 | 2 | S13(Phospho); K15(Label:13C(6)15N(2)); K16(Label:13C(6)15N(2)); K19(Label:13C(6)15N(2)) | Y(2): 0.0; S(5): 0.0; S(13): 100.0 | GIPLPAEsPKKGPKK\|\|GIPLPAEsPKKGPKK | S13; | 2116.1 | Yes\|\|No |

| GeneSymbol | NP_Accession | Gene ID | ProteinDescription | PhosphoSite (Protein) | SCC-R / SCC-S R1 | SCC-R / SCC-S R2 | SCC-R / SCC-S Total Proteome | Sequence | # PSMs | # Proteins | # Protein Groups | # Missed Cleavages | Modifications | phosphoRS Site Probabilities | Phosphowindow | PhosphoSite (Peptide) | MH+ [Da] | PhosphositePlusEvidence |
|---|---|---|---|---|---|---|---|---|---|---|---|---|---|---|---|---|---|---|
| **C17orf49\|\|C17orf49** | NP_777553.1\|\|NP_001136271.1 | 124944\|\|124944 | chromatin complexes subunit BAP18 isoform 2\|\|chromatin complexes subunit BAP18 isoform 3 | **S96\|\|S62** | | | #N/A | VYEDSGIPLPAEsPKKGPK | 2 | 3 | 2 | 2 | S13(Phospho) | Y(2): 0.0; S(5): 0.0; S(13): 100.0 | GIPLPAEsPKKGPKK\|\|GIPLPAEsPKKGPKK | S13; | 2092.0 | Yes\|\|No |
| **C17orf49\|\|C17orf49** | NP_777553.1\|\|NP_001136271.1 | 124944\|\|124944 | chromatin complexes subunit BAP18 isoform 2\|\|chromatin complexes subunit BAP18 isoform 3 | **S96\|\|S62** | | | #N/A | KVYEDSGIPLPAEsPK | 1 | 3 | 2 | 1 | S14(Phospho) | Y(3): 0.0; S(6): 0.0; S(14): 100.0 | GIPLPAEsPKKGPKK\|\|GIPLPAEsPKKGPKK | S14; | 1809.9 | Yes\|\|No |
| **C17orf49\|\|C17orf49** | NP_777553.1\|\|NP_001136271.1 | 124944\|\|124944 | chromatin complexes subunit BAP18 isoform 2\|\|chromatin complexes subunit BAP18 isoform 3 | **S96\|\|S62** | | | #N/A | KVYEDSGIPLPAEsPK | 2 | 3 | 2 | 1 | K1(Label:13C(6)15N(2)); S14(Phospho); K16(Label:13C(6)15N(2)) | Y(3): 0.0; S(6): 0.0; S(14): 100.0 | GIPLPAEsPKKGPKK\|\|GIPLPAEsPKKGPKK | S14; | 1825.9 | Yes\|\|No |
| **C17orf70** | NP_079437.5 | 80233 | Fanconi anemia-associated protein of 100 kDa isoform b | **S667** | **0.51** | | #N/A | APsPLGPTRDPVATFLETCREPGSQPAGPASLR | 1 | 1 | 1 | 2 | S3(Phospho); C19(Carbamidomethyl) | S(3): 96.9; T(8): 3.1; T(14): 0.0; T(18): 0.0; S(24): 0.0; S(31): 0.0 | PPHTRAPsPLGPTRD | S3; | 3512.7 | No |
| **C17orf85** | NP_001107590.1 | 55421 | uncharacterized protein C17orf85 | **S415** | 0.73 | 0.73 | 0.79 | MISTPsPK | 5 | 1 | 1 | 0 | S6(Phospho) | S(3): 0.0; T(4): 0.0; S(6): 100.0 | LKMISTPsPKKSMKM | S6; | 940.4 | Yes |
| **C17orf85** | NP_001107590.1 | 55421 | uncharacterized protein C17orf85 | **S415** | | 0.7 | 0.79 | MISTPsPK | 4 | 1 | 1 | 0 | S6(Phospho); K8(Label:13C(6)15N(2)) | S(3): 0.0; T(4): 0.0; S(6): 100.0 | LKMISTPsPKKSMKM | S6; | 948.4 | Yes |
| **C17orf85** | NP_001107590.1 | 55421 | uncharacterized protein C17orf85 | **S415** | | 1 | 0.79 | MISTPsPKK | 2 | 1 | 1 | 1 | S6(Phospho) | S(3): 0.0; T(4): 0.0; S(6): 100.0 | LKMISTPsPKKSMKM | S6; | 1068.5 | Yes |
| **C17orf85** | NP_001107590.1 | 55421 | uncharacterized protein C17orf85 | **S30** | | 1.4 | 0.79 | AEAPAGPALGLPSPEAEsGVDRGEPEPMEVEEGELEIVPVRR | 1 | 1 | 1 | 2 | S13(Phospho) | S(13): 0.0; S(18): 100.0 | LPSPEAEsGVDRGEP | S18; | 4446.1 | Yes |
| **C18orf25** | NP_001008240.1 | 147339 | uncharacterized protein C18orf25 isoform b | **S66** | | 1.16 | **0.13** | RDsSESQLASTESDKPTTGR | 2 | 2 | 1 | 1 | S3(Phospho) | S(3): 100.0; S(4): 0.0; S(6): 0.0; S(10): 0.0; T(11): 0.0; S(13): 0.0; T(17): 0.0; T(18): 0.0 | LMELRRDsSESQLAS | S3; | 2232.0 | Yes |
| **C18orf25** | NP_001008240.1 | 147339 | uncharacterized protein C18orf25 isoform b | **S66** | | | **0.13** | RDsSESQLASTESDKPTTGR | 1 | 2 | 1 | 1 | R1(Label:13C(6)); S3(Phospho); K15(Label:13C(6)15N(2)); R20(Label:13C(6)) | S(3): 98.7; S(4): 1.3; S(6): 0.0; S(10): 0.0; T(11): 0.0; S(13): 0.0; T(17): 0.0; T(18): 0.0 | LMELRRDsSESQLAS | S3; | 2252.0 | Yes |
| **C19orf24** | NP_060384.3 | 55009 | uncharacterized membrane protein C19orf24 precursor | **S117; S120** | | | #N/A | RYGLLANTEDPTEMAsLDsDEETVFESR | 1 | 1 | 1 | 1 | S16(Phospho); S19(Phospho) | Y(2): 0.0; T(8): 0.0; T(12): 3.3; S(16): 96.7; S(19): 100.0; T(23): 0.0; S(27): 0.0 | EDPTEMAsLDSDEET; TEMASLDsDEETVFE | S16; S19; | 3335.4 | Yes;Yes |
| **C19orf24** | NP_060384.3 | 55009 | uncharacterized membrane protein C19orf24 precursor | **S117; S120** | | 0.62 | #N/A | RYGLLANTEDPTEMAsLDsDEETVFESR | 1 | 1 | 1 | 1 | R1(Label:13C(6)); S16(Phospho); S19(Phospho); R28(Label:13C(6)) | Y(2): 0.0; T(8): 0.0; T(12): 0.0; S(16): 100.0; S(19): 100.0; T(23): 0.0; S(27): 0.0 | EDPTEMAsLDSDEET; TEMASLDsDEETVFE | S16; S19; | 3347.4 | Yes;Yes |
| **C19orf33** | NP_277055.1 | 64073 | immortalization up-regulated protein | **S29** | **6.27** | **6.18** | #NQ | LsPHKVQGR | 4 | 1 | 1 | 1 | S2(Phospho) | S(2): 100.0 | HGGDPKLsPHKVQGR | S2; | 1101.6 | No |
| **C19orf43** | NP_076943.1 | 79002 | uncharacterized protein C19orf43 | **S33** | | 0.64 | 1.35 | WAESGsGTSPESGDEEVSGAGSSPVSGGVNLFANDGSFLELFKR | 2 | 1 | 1 | 1 | S12(Phospho) | S(4): 0.0; S(6): 96.9; T(8): 2.9; S(9): 0.1; S(12): 0.1; S(18): 0.0; S(22): 0.0; S(23): 0.0; S(26): 0.0; S(37): 0.0 | SRWAESGsGTSPESG | S6; | 4498.0 | No |
| **C19orf43** | NP_076943.1 | 79002 | uncharacterized protein C19orf43 | **S33** | | 0.67 | 1.35 | WAESGsGTSPESGDEEVSGAGSSPVSGGVNLFANDGSFLELFKR | 2 | 1 | 1 | 1 | S12(Phospho); K43(Label:13C(6)15N(2)); R44(Label:13C(6)) | S(4): 0.1; S(6): 96.6; T(8): 3.1; S(9): 0.1; S(12): 0.1; S(18): 0.0; S(22): 0.0; S(23): 0.0; S(26): 0.0; S(37): 0.0 | SRWAESGsGTSPESG | S6; | 4512.0 | No |
| **C1orf116** | NP_001077393.1 | 79098 | specifically androgen-regulated gene protein isoform 2 | **S216** | | | #NQ | ANSALTPPKPEsGLTLQESNTPGLR | 1 | 2 | 1 | 0 | K9(Label:13C(6)15N(2)); S12(Phospho); R25(Label:13C(6)) | S(3): 2.4; T(6): 2.4; S(12): 92.8; T(15): 2.4; S(19): 0.0; T(21): 0.0 | LTPPKPEsGLTLQES | S12; | 2672.4 | No |
| **C1orf173** | XP_006710412.1 | 127254 | uncharacterized protein C1orf173 isoform X4 | **T1309** | | | #N/A | ELSPESLQAMATLPVKPDFTEtR | 1 | 6 | 1 | 0 | M10(Oxidation); T12(Phospho); K16(Label:13C(6)15N(2)) | S(3): 0.0; S(6): 0.0; T(12): 0.0; T(20): 4.7; T(22): 95.3 | VKPDFTEtREKQQHM | T22; | 2664.3 | No |
| **C1orf35** | NP_077295.1 | 79169 | multiple myeloma tumor-associated protein 2 | **T215; S220** | 0.92 | | #N/A | RPAEAtSSPTsPERPR | 1 | 1 | 1 | 0 | S8(Phospho); S11(Phospho) | T(6): 95.9; S(7): 0.0; S(8): 2.0; T(10): 2.1; S(11): 100.0 | HRRPAEAtSSPTSPE;EATSSPTsPERPRHH | T6; S11; | 1898.8 | Yes;Yes |
| **C1orf35** | NP_077295.1 | 79169 | multiple myeloma tumor-associated protein 2 | **S220** | | 0.89 | #N/A | RPAEATSSPTsPERPR | 1 | 1 | 1 | 0 | R1(Label:13C(6)); S11(Phospho); R14(Label:13C(6)); R16(Label:13C(6)) | T(6): 0.0; S(7): 0.0; S(8): 0.0; T(10): 0.0; S(11): 100.0 | EATSSPTsPERPRHH | S11; | 1836.9 | Yes |
| **C1orf52** | NP_932343.1 | 148423 | UPF0690 protein C1orf52 | **S158** | | 1.68 | #N/A | LLPEGEETLEsDDEKDEHTSK | 2 | 1 | 1 | 1 | S11(Phospho); K15(Label:13C(6)15N(2)); K21(Label:13C(6)15N(2)) | T(8): 0.0; S(11): 100.0; T(19): 0.0; S(20): 0.0 | EGEETLEsDDEKDEH | S11; | 2497.1 | Yes |
| **C2CD4D** | NP_001129475.1 | 1E+08 | C2 calcium-dependent domain-containing protein 4D | **S164** | | | #N/A | VsRPHSLSPEKASSADTSPHSPR | 1 | 1 | 1 | 1 | S2(Phospho); R3(Label:13C(6)); K11(Label:13C(6)15N(2)); R23(Label:13C(6)) | S(2): 99.8; S(6): 0.2; S(8): 0.0; S(13): 0.0; S(14): 0.0; T(17): 0.0; S(18): 0.0; S(21): 0.0 | GLGRRRVsRPHSLSP | S2; | 2530.2 | No |
| **C2CD5** | NP_055617.1 | 9847 | C2 domain-containing protein 5 isoform e | **S852** | | | #NQ | AMTVEKAsPVGDGNFR | 1 | 1 | 1 | 1 | M2(Oxidation); S8(Phospho) | T(3): 0.0; S(8): 100.0 | AMTVEKAsPVGDGNF | S8; | 1774.8 | Yes |
| **C2CD5** | NP_055617.1 | 9847 | C2 domain-containing protein 5 isoform e | **S852** | | 0.54 | #NQ | AMTVEKAsPVGDGNFR | 3 | 1 | 1 | 1 | S8(Phospho) | T(3): 0.0; S(8): 100.0 | AMTVEKAsPVGDGNF | S8; | 1758.8 | Yes |
| **C2CD5** | NP_055617.1 | 9847 | C2 domain-containing protein 5 isoform e | **S852** | | 0.6 | #NQ | AMTVEKAsPVGDGNFR | 3 | 1 | 1 | 1 | K6(Label:13C(6)15N(2)); S8(Phospho); R16(Label:13C(6)) | T(3): 0.0; S(8): 100.0 | AMTVEKAsPVGDGNF | S8; | 1772.8 | Yes |
| **C2CD5** | NP_001273106.1 | 9847 | C2 domain-containing protein 5 isoform d | **S855** | | 1.74 | #NQ | ATsVDYSSFADR | 1 | 3 | 1 | 0 | S3(Phospho); R12(Label:13C(6)) | T(2): 0.0; S(3): 100.0; Y(6): 0.0; S(7): 0.0; S(8): 0.0 | PAAQRATsVDYSSFA | S3; | 1404.6 | No |
| **C2CD5\|\|C2CD5** | NP_001273106.1\|\|NP_055617.1 | 9847\|\|9847 | C2 domain-containing protein 5 isoform d\|\|C2 domain-containing protein 5 isoform e | **T330\|\|T339** | | | #N/A | QQtQSALEQR | 2 | 5 | 2 | 0 | T3(Phospho) | T(3): 100.0; S(5): 0.0 | KALLRQQtQSALEQR\|\|KALLRQQtQSALEQR | T3; | 1268.6 | No\|\|Yes |
| **C2orf44** | NP_079479.1 | 80304 | WD repeat-containing protein C2orf44 isoform 1 | **S690** | 0.91 | | #N/A | DSFSHsPGAVSSLK | 4 | 1 | 1 | 0 | S6(Phospho); K14(Label:13C(6)15N(2)) | S(2): 0.0; S(4): 0.0; S(6): 100.0; S(11): 0.0; S(12): 0.0 | FRDSFSHsPGAVSSL | S6; | 1506.7 | Yes |
| **C2orf49** | NP_076998.1 | 79074 | ashwin isoform 1 | **S182; S189** | | **4.08** | 1.44 | sPSGPVKsPPLSPVGTTPVK | 2 | 2 | 1 | 1 | S3(Phospho); S8(Phospho) | S(1): 97.6; S(3): 2.4; S(8): 100.0; S(12): 0.0; T(16): 0.0; T(17): 0.0 | HDLTHRKsPSGPVKS; SPSGPVKsPPLSPVG | S1; S8; | 2092.0 | Yes;Yes |
| **C6orf106** | NP_073595.2 | 64771 | uncharacterized protein C6orf106 isoform b | **S149** | 0.81 | 0.79 | 0.55 | KVEGNFNPFAsPQK | 6 | 2 | 1 | 1 | K1(Label:13C(6)15N(2)); S11(Phospho); K14(Label:13C(6)15N(2)) | S(11): 100.0 | GNFNPFAsPQKNRQS | S11; | 1658.8 | No |
| **C6orf106** | NP_073595.2 | 64771 | uncharacterized protein C6orf106 isoform b | **S149** | | 0.76 | 0.55 | KVEGNFNPFAsPQK | 2 | 2 | 1 | 1 | S11(Phospho) | S(11): 100.0 | GNFNPFAsPQKNRQS | S11; | 1642.8 | No |
| **C6orf106** | NP_073595.2 | 64771 | uncharacterized protein C6orf106 isoform b | **S206** | | **0.43** | 0.55 | LSQNSVNLsPSSHANNLSVVTYSK | 1 | 2 | 1 | 0 | S9(Phospho); K24(Label:13C(6)15N(2)) | S(2): 0.0; S(5): 0.1; S(9): 99.9; S(11): 0.0; S(12): 0.0; S(18): 0.0; T(21): 0.0; Y(22): 0.0; S(23): 0.0 | SQNSVNLsPSSHANN | S9; | 2634.3 | No |
| **C7orf43** | NP_060745.3 | 55262 | uncharacterized protein C7orf43 | **S517** | | | #N/A | SQsFSHQQPSR | 2 | 1 | 1 | 0 | S3(Phospho) | S(1): 0.0; S(3): 100.0; S(5): 0.0; S(10): 0.0 | ASLGRSQsFSHQQPS | S3; | 1368.6 | Yes |
| **C7orf50** | NP_001127867.1 | 84310 | uncharacterized protein C7orf50 | **S175** | | 0.9 | 1.41 | ELDEEGsDPPLPGR | 4 | 1 | 1 | 0 | S7(Phospho) | S(7): 100.0 | RELDEEGsDPPLPGR | S7; | 1590.7 | Yes |
| **C7orf50** | NP_001127867.1 | 84310 | uncharacterized protein C7orf50 | **S175** | | | 1.41 | ELDEEGsDPPLPGR | 2 | 1 | 1 | 0 | S7(Phospho); R14(Label:13C(6)) | S(7): 100.0 | RELDEEGsDPPLPGR | S7; | 1596.7 | Yes |
| **C9orf142** | NP_899064.1 | 286257 | uncharacterized protein C9orf142 | **S148** | **0.32** | **0.31** | **0.38** | LAAAEETAVsPR | 16 | 1 | 1 | 0 | S10(Phospho) | T(7): 0.0; S(10): 100.0 | AAEETAVsPRKSPRP | S10; | 1294.6 | Yes |
| **C9orf142** | NP_899064.1 | 286257 | uncharacterized protein C9orf142 | **S148** | **0.36** | **0.36** | **0.38** | RLAAAEETAVsPR | 2 | 1 | 1 | 1 | S11(Phospho) | T(8): 0.0; S(11): 100.0 | AAEETAVsPRKSPRP | S11; | 1450.7 | Yes |
| **C9orf142** | NP_899064.1 | 286257 | uncharacterized protein C9orf142 | **S148** | **0.34** | **0.32** | **0.38** | LAAAEETAVsPR | 15 | 1 | 1 | 0 | S10(Phospho); R12(Label:13C(6)) | T(7): 0.0; S(10): 100.0 | AAEETAVsPRKSPRP | S10; | 1300.6 | Yes |
| **C9orf142** | NP_899064.1 | 286257 | uncharacterized protein C9orf142 | **S148** | **0.36** | **0.3** | **0.38** | RLAAAEETAVsPR | 8 | 1 | 1 | 1 | R1(Label:13C(6)); S11(Phospho); R13(Label:13C(6)) | T(8): 0.0; S(11): 100.0 | AAEETAVsPRKSPRP | S11; | 1462.7 | Yes |
| **C9orf142** | NP_899064.1 | 286257 | uncharacterized protein C9orf142 | **S152** | | 0.7 | **0.38** | KsPRPAGPQLFLPDPDPQR | 2 | 1 | 1 | 1 | S2(Phospho) | S(2): 100.0 | TAVSPRKsPRPAGPQ | S2; | 2196.1 | Yes |
| **C9orf142** | NP_899064.1 | 286257 | uncharacterized protein C9orf142 | **S152** | | 0.72 | **0.38** | KsPRPAGPQLFLPDPDPQR | 1 | 1 | 1 | 1 | K1(Label:13C(6)15N(2)); S2(Phospho); R4(Label:13C(6)); R19(Label:13C(6)) | S(2): 100.0 | TAVSPRKsPRPAGPQ | S2; | 2216.2 | Yes |
| **C9orf16** | NP_077017.1 | 79095 | UPF0184 protein C9orf16 | **S82** | | | #N/A | LEFQQQLGEAPSDAsP | 2 | 1 | 1 | 0 | S15(Phospho) | S(12): 0.0; S(15): 100.0 | GEAPSDAsP_ | S15; | 1796.8 | Yes |
| **C9orf40** | NP_060468.2 | 55071 | uncharacterized protein C9orf40 | **S69** | **3.41** | | #N/A | KIDAGTMAEPSAsPSKR | 2 | 1 | 1 | 2 | S13(Phospho) | T(6): 0.0; S(11): 0.0; S(13): 100.0; S(15): 0.0 | TMAEPSAsPSKRRDS | S13; | 1825.8 | Yes |
| **C9orf40** | NP_060468.2 | 55071 | uncharacterized protein C9orf40 | **S69** | **5.05** | | #N/A | IDAGTMAEPSAsPSKR | 2 | 1 | 1 | 1 | S12(Phospho) | T(5): 0.0; S(10): 1.0; S(12): 98.1; S(14): 1.0 | TMAEPSAsPSKRRDS | S12; | 1697.8 | Yes |
| **C9orf40** | NP_060468.2 | 55071 | uncharacterized protein C9orf40 | **S76** | **2.4** | | #N/A | RRDsGDNSAPSGQER | 1 | 1 | 1 | 2 | S4(Phospho) | S(4): 100.0; S(8): 0.0; S(11): 0.0 | SPSKRRDsGDNSAPS | S4; | 1711.7 | Yes |
| **C9orf40** | NP_060468.2 | 55071 | uncharacterized protein C9orf40 | **S69** | | | #N/A | KIDAGTMAEPSAsPSKR | 1 | 1 | 1 | 2 | K1(Label:13C(6)15N(2)); S13(Phospho); K16(Label:13C(6)15N(2)); R17(Label:13C(6)) | T(6): 0.0; S(11): 0.0; S(13): 100.0; S(15): 0.0 | TMAEPSAsPSKRRDS | S13; | 1847.9 | Yes |
| **C9orf78** | NP_057604.1 | 51759 | uncharacterized protein C9orf78 | **S15; S17** | 0.92 | 0.91 | 0.84 | RRGDsEsEEDEQDSEEVR | 4 | 1 | 1 | 2 | S5(Phospho); S7(Phospho) | S(5): 100.0; S(7): 100.0; S(14): 0.0 | FRRRRGDsESEEDEQ; RRRGDSEsEEDEQDS | S5; S7; | 2311.8 | Yes;Yes |
| **C9orf78** | NP_057604.1 | 51759 | uncharacterized protein C9orf78 | **S261** | 1.18 | 1.18 | 0.84 | VGDTEKPEPERsPPNR | 4 | 1 | 1 | 1 | S12(Phospho) | T(4): 0.0; S(12): 100.0 | EKPEPERsPPNRKRP | S12; | 1887.9 | Yes |
| **C9orf78** | NP_057604.1 | 51759 | uncharacterized protein C9orf78 | **S261** | | 1.22 | 0.84 | VGDTEKPEPERsPPNR | 2 | 1 | 1 | 1 | K6(Label:13C(6)15N(2)); R11(Label:13C(6)); S12(Phospho); R16(Label:13C(6)) | T(4): 0.0; S(12): 100.0 | EKPEPERsPPNRKRP | S12; | 1907.9 | Yes |
| **CACTIN** | NP_067054.1 | 58509 | cactin | **S489** | **2.3** | | #N/A | LKQEQGVESEPLFPILKQEPQsPSR | 3 | 1 | 1 | 2 | S22(Phospho) | S(9): 0.0; S(22): 98.8; S(24): 1.2 | ILKQEPQsPSRSLEP | S22; | 2944.5 | Yes |
| **CAD** | NP_004332.2 | 790 | CAD protein | **S1859** | **0.45** | **0.45** | **0.38** | IHRAsDPGLPAEEPKEK | 13 | 1 | 1 | 2 | S5(Phospho) | S(5): 100.0 | PPRIHRAsDPGLPAE | S5; | 1953.9 | Yes |
| **CAD** | NP_004332.2 | 790 | CAD protein | **S1900** | | 0.7 | **0.38** | QAsPQNLGTPGLLHPQTSPLLHSLVGQHILSVQQFTK | 11 | 1 | 1 | 0 | S3(Phospho); K37(Label:13C(6)15N(2)) | S(3): 100.0; T(9): 0.0; T(17): 0.0; S(18): 0.0; S(23): 0.0; S(31): 0.0; T(36): 0.0 | PPVPRQAsPQNLGTP | S3; | 4060.1 | Yes |

Supplementary Table S5:List of phosphopeptides identified in SCC-S and SCC-R cells using SequestHT and Mascot search algorithms

| GeneSymbol | NP_Accession | Gene ID | ProteinDescription | PhosphoSite (Protein) | SCC-R / SCC-S R1 | SCC-R / SCC-S R2 | SCC-R / SCC-S Total Proteome | Sequence | # PSMs | # Proteins | # Protein Groups | # Missed Cleavages | Modifications | phosphoRS Site Probabilities | Phosphowindow | PhosphoSite (Peptide) | MH+ [Da] | PhosphositePlusEvidence |
|---|---|---|---|---|---|---|---|---|---|---|---|---|---|---|---|---|---|---|
| **CAD** | NP_004332.2 | 790 | CAD protein | **S1900** | 0.68 | | **0.38** | QAsPQNLGTPGLLHPQTSPLLHSLVGQHILSVQQFTK | 2 | 1 | 1 | 0 | S3(Phospho) | S(3): 100.0; T(9): 0.0; T(17): 0.0; S(18): 0.0; S(23): 0.0; S(31): 0.0; T(36): 0.0 | PPVPRQAsPQNLGTP | S3; | 4052.1 | Yes |
| **CAD** | NP_004332.2 | 790 | CAD protein | **S1859** | **0.41** | | **0.38** | IHRAsDPGLPAEEPK | 3 | 1 | 1 | 1 | S5(Phospho) | S(5): 100.0 | PPRIHRAsDPGLPAE | S5; | 1696.8 | Yes |
| **CAD** | NP_004332.2 | 790 | CAD protein | **S1406** | **0.39** | | **0.38** | RLsSFVTK | 2 | 1 | 1 | 1 | S3(Phospho) | S(3): 99.4; S(4): 0.6; T(7): 0.0 | GAGGRRLsSFVTKGY | S3; | 1017.5 | Yes |
| **CAD** | NP_004332.2 | 790 | CAD protein | **S1859** | **0.45** | **0.38** | **0.38** | IHRAsDPGLPAEEPKEK | 6 | 1 | 1 | 2 | R3(Label:13C(6)); S5(Phospho); K15(Label:13C(6)15N(2)); K17(Label:13C(6)15N(2)) | S(5): 100.0 | PPRIHRAsDPGLPAE | S5; | 1976.0 | Yes |
| **CAD** | NP_004332.2 | 790 | CAD protein | **S1859** | | | **0.38** | IHRAsDPGLPAEEPKEK | 1 | 1 | 1 | 2 | R3(Label:13C(6)); S5(Phospho); K15(Label:13C(6)15N(2)) | S(5): 100.0 | PPRIHRAsDPGLPAE | S5; | 1968.0 | Yes |
| **CAD** | NP_004332.2 | 790 | CAD protein | **S1859** | **0.39** | | **0.38** | IHRAsDPGLPAEEPK | 1 | 1 | 1 | 1 | R3(Label:13C(6)); S5(Phospho); K15(Label:13C(6)15N(2)) | S(5): 100.0 | PPRIHRAsDPGLPAE | S5; | 1710.8 | Yes |
| **CAD** | NP_004332.2 | 790 | CAD protein | **S1406** | **0.41** | | **0.38** | RLsSFVTK | 1 | 1 | 1 | 1 | R1(Label:13C(6)); S3(Phospho); K8(Label:13C(6)15N(2)) | S(3): 100.0; S(4): 0.0; T(7): 0.0 | GAGGRRLsSFVTKGY | S3; | 1031.5 | Yes |
| **CAD** | NP_004332.2 | 790 | CAD protein | **S1038** | | **0.16** | **0.38** | VLGTsPEAIDSAENRFK | 1 | 1 | 1 | 1 | S5(Phospho); R15(Label:13C(6)); K17(Label:13C(6)15N(2)) | T(4): 1.5; S(5): 98.5; S(11): 0.0 | QCRVLGTsPEAIDSA | S5; | 1927.9 | Yes |
| **CALD1\|\|CALD1** | NP_149131.1\|\|NP_004333.1 | 800\|\|800 | caldesmon isoform 5\|\|caldesmon isoform 2 | **S528\|\|S534** | | | #N/A | QSVDKVTsPTKV | 2 | 5 | 2 | 2 | S8(Phospho) | S(2): 0.0; T(7): 1.1; S(8): 97.9; T(10): 1.1 | QSVDKVTsPTKV_\|\|QSVDKVTsPTKV_ | S8; | 1368.7 | No\|\|Yes |
| **CALD1\|\|CALD1** | NP_149131.1\|\|NP_004333.1 | 800\|\|800 | caldesmon isoform 5\|\|caldesmon isoform 2 | **S196\|\|S202** | | | #N/A | RGsIGENQIKDEK | 4 | 2 | 2 | 2 | S3(Phospho) | S(3): 100.0 | EEKPKRGsIGENQIK\|\|EEKPKRGsIGENQIK | S3; | 1553.7 | No\|\|Yes |
| **CALD1\|\|CALD1** | NP_149131.1\|\|NP_004333.1 | 800\|\|800 | caldesmon isoform 5\|\|caldesmon isoform 2 | **S528\|\|S534** | | | #N/A | QSVDKVTsPTKV | 2 | 5 | 2 | 2 | K5(Label:13C(6)15N(2)); S8(Phospho); K11(Label:13C(6)15N(2)) | S(2): 0.0; T(7): 1.1; S(8): 98.9; T(10): 0.0 | QSVDKVTsPTKV_\|\|QSVDKVTsPTKV_ | S8; | 1384.7 | No\|\|Yes |
| **CALD1\|\|CALD1** | NP_149131.1\|\|NP_004333.1 | 800\|\|800 | caldesmon isoform 5\|\|caldesmon isoform 2 | **S196\|\|S202** | | | #N/A | RGsIGENQIKDEK | 1 | 2 | 2 | 2 | R1(Label:13C(6)); S3(Phospho); K10(Label:13C(6)15N(2)); K13(Label:13C(6)15N(2)) | S(3): 100.0 | EEKPKRGsIGENQIK\|\|EEKPKRGsIGENQIK | S3; | 1575.8 | No\|\|Yes |
| **CAMK2G** | NP_751911.1 | 818 | calcium/calmodulin-dependent protein kinase type II subunit gamma isoform 1 | **S417** | | | 0.64 | DRTAPSAGMQPQPsLCSSAMR | 1 | 1 | 1 | 1 | S14(Phospho); C16(Carbamidomethyl) | T(3): 0.0; S(6): 0.0; S(14): 100.0; S(17): 0.0; S(18): 0.0 | AGMQPQPsLCSSAMR | S14; | 2328.0 | No |
| **CAMK2G\|\|CAMK2G** | NP_751909.1\|\|NP_751911.1 | 818\|\|818 | calcium/calmodulin-dependent protein kinase type II subunit gamma isoform 2\|\|calcium/calmodulin-dependent protein kinase type II subunit gamma isoform 1 | **T287\|\|T287** | | | #N/A | QEtVECLR | 1 | 6 | 2 | 0 | T3(Phospho); C6(Carbamidomethyl) | T(3): 100.0 | SMMHRQEtVECLRKF\|\|SMMHRQEtVECLRKF | T3; | 1114.5 | Yes\|\|Yes |
| **CAMSAP1** | NP_056262.3 | 157922 | calmodulin-regulated spectrin-associated protein 1 | **S1080** | 0.76 | 0.76 | #N/A | APVHFVEPLsPTGVAGHR | 6 | 1 | 1 | 0 | S10(Phospho) | S(10): 100.0; T(12): 0.0 | VHFVEPLsPTGVAGH | S10; | 1951.0 | Yes |
| **CAMSAP1** | NP_056262.3 | 157922 | calmodulin-regulated spectrin-associated protein 1 | **S1080** | 0.72 | 0.75 | #N/A | APVHFVEPLsPTGVAGHR | 4 | 1 | 1 | 0 | S10(Phospho); R18(Label:13C(6)) | S(10): 100.0; T(12): 0.0 | VHFVEPLsPTGVAGH | S10; | 1957.0 | Yes |
| **CAMSAP1** | NP_056262.3 | 157922 | calmodulin-regulated spectrin-associated protein 1 | **S563** | 0.67 | 0.64 | #N/A | ADVVPQQADPEFPRAsPR | 2 | 1 | 1 | 1 | R14(Label:13C(6)); S16(Phospho); R18(Label:13C(6)) | S(16): 100.0 | DPEFPRAsPRALGLT | S16; | 2072.0 | Yes |
| **CAMSAP1** | NP_056262.3 | 157922 | calmodulin-regulated spectrin-associated protein 1 | **S862** | | **0.28** | #N/A | EQsPSQHGKDPASLLASELVQLHMQLEEK | 1 | 1 | 1 | 1 | K9(Label:13C(6)15N(2)); S13(Phospho); K29(Label:13C(6)15N(2)) | S(3): 98.2; S(5): 1.8; S(13): 0.0; S(17): 0.0 | WRQKREQsPSQHGKD | S3; | 3325.6 | Yes |
| **CAMSAP1** | NP_056262.3 | 157922 | calmodulin-regulated spectrin-associated protein 1 | **S563** | | 0.62 | #N/A | ADVVPQQADPEFPRAsPR | 2 | 1 | 1 | 1 | S16(Phospho) | S(16): 100.0 | DPEFPRAsPRALGLT | S16; | 2060.0 | Yes |
| **CAMSAP1** | NP_056262.3 | 157922 | calmodulin-regulated spectrin-associated protein 1 | **S1080** | | 0.83 | #N/A | VKAPVHFVEPLsPTGVAGHR | 1 | 1 | 1 | 1 | K2(Label:13C(6)15N(2)); S12(Phospho); R20(Label:13C(6)) | S(12): 100.0; T(14): 0.0 | VHFVEPLsPTGVAGH | S12; | 2192.2 | Yes |
| **CAMSAP2** | NP_982284.1 | 23271 | calmodulin-regulated spectrin-associated protein 2 | **S588** | **0.25** | **0.28** | **0.49** | LNQSsPDNVTDTK | 6 | 1 | 1 | 0 | S5(Phospho); K13(Label:13C(6)15N(2)) | S(4): 0.0; S(5): 100.0; T(10): 0.0; T(12): 0.0 | NIKLNQSsPDNVTDT | S5; | 1506.7 | No |
| **CAMSAP2** | NP_982284.1 | 23271 | calmodulin-regulated spectrin-associated protein 2 | **S588** | | **0.28** | **0.49** | LNQSsPDNVTDTK | 3 | 1 | 1 | 0 | S5(Phospho) | S(4): 0.0; S(5): 100.0; T(10): 0.0; T(12): 0.0 | NIKLNQSsPDNVTDT | S5; | 1498.6 | No |
| **CAMSAP2** | NP_982284.1 | 23271 | calmodulin-regulated spectrin-associated protein 2 | **S1008** | **0.42** | | **0.49** | RFsPSQVPIQTR | 2 | 1 | 1 | 1 | S3(Phospho) | S(3): 100.0; S(5): 0.0; T(11): 0.0 | LPRLRRFsPSQVPIQ | S3; | 1495.7 | No |
| **CAMSAP2** | NP_982284.1 | 23271 | calmodulin-regulated spectrin-associated protein 2 | **S453** | **0.44** | **0.5** | **0.49** | SIsNEGLTLNNSHVSK | 5 | 1 | 1 | 0 | S3(Phospho); K16(Label:13C(6)15N(2)) | S(1): 0.0; S(3): 100.0; T(8): 0.0; S(12): 0.0; S(15): 0.0 | RGITRSIsNEGLTLN | S3; | 1787.8 | No |
| **CAMSAP2** | NP_982284.1 | 23271 | calmodulin-regulated spectrin-associated protein 2 | **S453; T458** | | | **0.49** | GITRSIsNEGLtLNNSHVSK | 1 | 1 | 1 | 1 | R4(Label:13C(6)); S7(Phospho); S16(Phospho) | T(3): 0.0; S(5): 0.2; S(7): 99.8; T(12): 92.3; S(16): 3.9; S(19): 3.9 | RGITRSIsNEGLTLN;SISNEGLtLNNSHVS | S7; T12; | 2293.1 | No;No |
| **CAMSAP2** | NP_982284.1 | 23271 | calmodulin-regulated spectrin-associated protein 2 | **S453** | | | **0.49** | SIsNEGLTLNNSHVSK | 3 | 1 | 1 | 0 | S3(Phospho) | S(1): 0.0; S(3): 100.0; T(8): 0.0; S(12): 0.0; S(15): 0.0 | RGITRSIsNEGLTLN | S3; | 1779.8 | No |
| **CAMSAP3** | NP_065953.1 | 57662 | calmodulin-regulated spectrin-associated protein 3 isoform 2 | **S334** | **0.31** | **0.35** | **0.39** | DLPDGHAAsPR | 4 | 2 | 1 | 0 | S9(Phospho) | S(9): 100.0 | LPDGHAAsPRGTEAS | S9; | 1215.5 | Yes |
| **CAMSAP3** | NP_065953.1 | 57662 | calmodulin-regulated spectrin-associated protein 3 isoform 2 | **S334** | **0.32** | | **0.39** | DLPDGHAAsPR | 1 | 2 | 1 | 0 | S9(Phospho); R11(Label:13C(6)) | S(9): 100.0 | LPDGHAAsPRGTEAS | S9; | 1221.5 | Yes |
| **CAMSAP3** | NP_065953.1 | 57662 | calmodulin-regulated spectrin-associated protein 3 isoform 2 | **S1074** | | **0.18** | **0.39** | APsPSGLMSPSR | 2 | 2 | 1 | 0 | S3(Phospho); R12(Label:13C(6)) | S(3): 100.0; S(5): 0.0; S(9): 0.0; S(11): 0.0 | RPTSRAPsPSGLMSP | S3; | 1272.6 | Yes |
| **CAMSAP3** | NP_065953.1 | 57662 | calmodulin-regulated spectrin-associated protein 3 isoform 2 | **S1020** | | **0.26** | **0.39** | sGCCDDSALAR | 1 | 2 | 1 | 0 | S1(Phospho); C3(Carbamidomethyl); C4(Carbamidomethyl); R11(Label:13C(6)) | S(1): 100.0; S(7): 0.0 | RRATRPRsGCCDDSA | S1; | 1297.5 | Yes |
| **CANX** | NP_001737.1 | 821 | calnexin precursor | **S554** | 1.21 | 1.09 | 1.12 | QKsDAEEDGGTVSQEEEDRKPK | 15 | 1 | 1 | 2 | S3(Phospho) | S(3): 100.0; T(11): 0.0; S(13): 0.0 | KLEEKQKsDAEEDGG | S3; | 2542.1 | Yes |
| **CANX** | NP_001737.1 | 821 | calnexin precursor | **S554** | 0.92 | 1.45 | 1.12 | QKsDAEEDGGTVSQEEEDR | 3 | 1 | 1 | 1 | S3(Phospho) | S(3): 99.9; T(11): 0.0; S(13): 0.0 | KLEEKQKsDAEEDGG | S3; | 2188.9 | Yes |
| **CANX** | NP_001737.1 | 821 | calnexin precursor | **S554; S564** | 0.7 | | 1.12 | QKsDAEEDGGTVsQEEEDR | 1 | 1 | 1 | 1 | S3(Phospho); S13(Phospho) | S(3): 100.0; T(11): 0.0; S(13): 100.0 | KLEEKQKsDAEEDGG;EEDGGTVsQEEEDRK | S3; S13; | 2268.8 | Yes;Yes |
| **CANX** | NP_001737.1 | 821 | calnexin precursor | **S554; S564** | 0.7 | 0.7 | 1.12 | QKsDAEEDGGTVsQEEEDRKPK | 8 | 1 | 1 | 2 | S3(Phospho); S13(Phospho) | S(3): 100.0; T(11): 0.0; S(13): 100.0 | KLEEKQKsDAEEDGG;EEDGGTVsQEEEDRK | S3; S13; | 2622.1 | Yes;Yes |
| **CANX** | NP_001737.1 | 821 | calnexin precursor | **S583** | 0.87 | 0.86 | 1.12 | AEEDEILNRsPR | 52 | 1 | 1 | 1 | S10(Phospho) | S(10): 100.0 | EDEILNRsPRNRKPR | S10; | 1508.7 | Yes |
| **CANX** | NP_001737.1 | 821 | calnexin precursor | **S583** | | | 1.12 | AEEDEILNRsPR | 2 | 1 | 1 | 1 | R9(Label:13C(6)); S10(Phospho) | S(10): 100.0 | EDEILNRsPRNRKPR | S10; | 1514.7 | Yes |
| **CANX** | NP_001737.1 | 821 | calnexin precursor | **S583** | 0.92 | 0.92 | 1.12 | AEEDEILNRsPR | 23 | 1 | 1 | 1 | R9(Label:13C(6)); S10(Phospho); R12(Label:13C(6)) | S(10): 100.0 | EDEILNRsPRNRKPR | S10; | 1520.7 | Yes |
| **CAP1** | NP_001099000.1 | 10487 | adenylyl cyclase-associated protein 1 | **Y31** | | 0.72 | 1.53 | GyADSPSKAGAAPYVQAFDSLLAGPVAEYLK | 2 | 1 | 1 | 1 | Y2(Phospho); K8(Label:13C(6)15N(2)); K31(Label:13C(6)15N(2)) | Y(2): 99.9; S(5): 0.1; S(7): 0.1; Y(14): 0.0; S(20): 0.0; Y(29): 0.0 | TSDMHRGyADSPSKA | Y2; | 3252.6 | Yes |
| **CAP2** | NP_006357.1 | 10486 | adenylyl cyclase-associated protein 2 | **S312** | | **2.47** | **2.23** | SHTPSPTsPK | 1 | 1 | 1 | 0 | S8(Phospho) | S(1): 0.0; T(3): 0.0; S(5): 0.0; T(7): 1.3; S(8): 98.7 | SHTPSPTsPKSYPSQ | S8; | 1118.5 | Yes |
| **CARD11** | NP_115791.3 | 84433 | caspase recruitment domain-containing protein 11 | **S925** | 1.14 | | 1.58 | IISGsPLGSLAR | 2 | 1 | 1 | 0 | S5(Phospho) | S(3): 0.0; S(5): 100.0; S(9): 0.0 | RVRIISGsPLGSLAR | S5; | 1250.7 | Yes |
| **CARD11** | NP_115791.3 | 84433 | caspase recruitment domain-containing protein 11 | **S886** | | **3.14** | 1.58 | NTLQPEEALSTSDPRVsPR | 1 | 1 | 1 | 1 | S17(Phospho) | T(2): 0.0; S(10): 0.0; T(11): 0.0; S(12): 0.0; S(17): 100.0 | STSDPRVsPRLSRAS | S17; | 2177.0 | Yes |
| **CARD11** | NP_115791.3 | 84433 | caspase recruitment domain-containing protein 11 | **S886** | | | 1.58 | NTLQPEEALSTSDPRVsPR | 1 | 1 | 1 | 1 | R15(Label:13C(6)); S17(Phospho); R19(Label:13C(6)) | T(2): 0.0; S(10): 0.0; T(11): 0.0; S(12): 0.0; S(17): 100.0 | STSDPRVsPRLSRAS | S17; | 2189.1 | Yes |
| **CARHSP1** | NP_055131.2 | 23589 | calcium-regulated heat stable protein 1 | **S52** | 1.28 | 1.29 | 1.43 | TFsATVR | 11 | 1 | 1 | 0 | S3(Phospho) | T(1): 0.0; S(3): 100.0; T(5): 0.0 | TRRTRTFsATVRASQ | S3; | 861.4 | Yes |
| **CARHSP1** | NP_055131.2 | 23589 | calcium-regulated heat stable protein 1 | **S30; S32; T45** | **2.23** | | 1.43 | ERsPsPLRGNVVPSPLPtR | 1 | 1 | 1 | 2 | S3(Phospho); S5(Phospho); S14(Phospho) | S(3): 100.0; S(5): 99.9; S(14): 2.6; T(18): 97.4 | TPRSRERsPSPLRGN;RSRERSPsPLRGNVV;VVPSPLPtRRTRTFS | S3; S5; T18; | 2299.0 | Yes;Yes;Yes |
| **CARHSP1** | NP_055131.2 | 23589 | calcium-regulated heat stable protein 1 | **S30** | 1.07 | 1.06 | 1.43 | ERsPSPLR | 2 | 1 | 1 | 1 | R2(Label:13C(6)); S3(Phospho); R8(Label:13C(6)) | S(3): 100.0; S(5): 0.0 | TPRSRERsPSPLRGN | S3; | 1033.5 | Yes |
| **CARHSP1** | NP_055131.2 | 23589 | calcium-regulated heat stable protein 1 | **S52** | 1.34 | | 1.43 | TFsATVR | 1 | 1 | 1 | 0 | S3(Phospho); R7(Label:13C(6)) | T(1): 0.0; S(3): 99.1; T(5): 0.9 | TRRTRTFsATVRASQ | S3; | 867.4 | Yes |
| **CARHSP1** | NP_055131.2 | 23589 | calcium-regulated heat stable protein 1 | **S30** | | | 1.43 | ERsPSPLR | 2 | 1 | 1 | 1 | S3(Phospho) | S(3): 100.0; S(5): 0.0 | TPRSRERsPSPLRGN | S3; | 1021.5 | Yes |
| **CARHSP1** | NP_055131.2 | 23589 | calcium-regulated heat stable protein 1 | **S41** | | | 1.43 | GNVVPsPLPTR | 2 | 1 | 1 | 0 | S6(Phospho) | S(6): 100.0; T(10): 0.0 | LRGNVVPsPLPTRRT | S6; | 1216.6 | Yes |
| **CARHSP1** | NP_055131.2 | 23589 | calcium-regulated heat stable protein 1 | **S30; S32** | | 1.26 | 1.43 | ERsPsPLRGNVVPSPLPTR | 1 | 1 | 1 | 2 | S3(Phospho); S5(Phospho) | S(3): 100.0; S(5): 100.0; S(14): 0.0; T(18): 0.0 | TPRSRERsPSPLRGN;RSRERSPsPLRGNVV | S3; S5; | 2219.1 | Yes;Yes |
| **CARHSP1** | NP_055131.2 | 23589 | calcium-regulated heat stable protein 1 | **S30; S32; S41** | | **3.77** | 1.43 | sPsPLRGNVVPsPLPTRR | 2 | 1 | 1 | 2 | S1(Phospho); S3(Phospho); S12(Phospho) | S(1): 100.0; S(3): 100.0; S(12): 100.0; T(16): 0.0 | TPRSRERsPSPLRGN;RSRERSPsPLRGNVV;LRGNVVPsPLPTRRT | S1; S3; S12; | 2170.0 | Yes;Yes;Yes |
| **CARHSP1** | NP_055131.2 | 23589 | calcium-regulated heat stable protein 1 | **S41** | | | 1.43 | GNVVPsPLPTR | 1 | 1 | 1 | 0 | S6(Phospho); R11(Label:13C(6)) | S(6): 100.0; T(10): 0.0 | LRGNVVPsPLPTRRT | S6; | 1222.6 | Yes |
| **CARHSP1** | NP_055131.2 | 23589 | calcium-regulated heat stable protein 1 | **S41** | | | 1.43 | GNVVPsPLPTRR | 1 | 1 | 1 | 1 | S6(Phospho); R11(Label:13C(6)); R12(Label:13C(6)) | S(6): 100.0; T(10): 0.0 | LRGNVVPsPLPTRRT | S6; | 1384.7 | Yes |
| **CARHSP1** | NP_055131.2 | 23589 | calcium-regulated heat stable protein 1 | **S41** | | | 1.43 | GNVVPsPLPTRR | 1 | 1 | 1 | 1 | S6(Phospho) | S(6): 100.0; T(10): 0.0 | LRGNVVPsPLPTRRT | S6; | 1372.7 | Yes |

| GeneSymbol | NP_Accession | Gene ID | ProteinDescription | PhosphoSite (Protein) | SCC-R / SCC-S R1 | SCC-R / SCC-S R2 | SCC-R / SCC-S Total Proteome | Sequence | # PSMs | # Proteins | # Protein Groups | # Missed Cleavages | Modifications | phosphoRS Site Probabilities | Phosphowindow | PhosphoSite (Peptide) | MH+ [Da] | PhosphositePlusEvidence |
|---|---|---|---|---|---|---|---|---|---|---|---|---|---|---|---|---|---|---|
| **CASC3** | NP_031385.2 | 22794 | protein CASC3 | **S148** | 1.3 | | #N/A | STVTGERQsGDGQESTEPVENKVGK | 1 | 1 | 1 | 2 | T2(Phospho) | S(1): 0.1; T(2): 0.1; T(4): 0.1; S(9): 99.8; S(15): 0.0; T(16): 0.0 | TVTGERQsGDGQEST | S9; | 2699.2 | Yes |
| **CASC3** | NP_031385.2 | 22794 | protein CASC3 | **T443** | **2.98** | | #N/A | LAEEVPPPPEGLIPAPPVPETTPtPPTK | 2 | 1 | 1 | 0 | T24(Phospho) | T(21): 0.0; T(22): 0.1; T(24): 97.6; T(27): 2.3 | PVPETTPtPPTKTGT | T24; | 2951.5 | Yes |
| **CASC3** | NP_031385.2 | 22794 | protein CASC3 | **S148** | 1.07 | | #N/A | STVTGERQsGDGQESTEPVENK | 1 | 1 | 1 | 1 | R7(Label:13C(6)); S9(Phospho); K22(Label:13C(6)15N(2)) | S(1): 0.0; T(2): 0.0; T(4): 0.0; S(9): 100.0; S(15): 0.0; T(16): 0.0 | TVTGERQsGDGQEST | S9; | 2429.1 | Yes |
| **CASC4** | NP_816929.1 | 113201 | protein CASC4 isoform b | **S328** | | 1.13 | #NQ | LIPGsNLDSEPR | 1 | 2 | 1 | 0 | S5(Phospho) | S(5): 100.0; S(9): 0.0 | LQRLIPGsNLDSEPR | S5; | 1377.6 | Yes |
| **CASC5** | NP_653091.3 | 57082 | protein CASC5 isoform 2 | **S60** | | | #N/A | RVsFADTIK | 2 | 2 | 1 | 1 | S3(Phospho) | S(3): 100.0; T(7): 0.0 | KKNSRRVsFADTIKV | S3; | 1116.5 | Yes |
| **CASK** | NP_001119527.1 | 8573 | peripheral plasma membrane protein CASK isoform 3 | **S571** | | | 0.79 | TQSSsCEDLPSTTQPK | 2 | 2 | 1 | 0 | S5(Phospho); C6(Carbamidomethyl) | T(1): 0.0; S(3): 0.0; S(4): 1.4; S(5): 98.6; S(11): 0.0; T(12): 0.0; T(13): 0.0 | SYRTQSSsCEDLPST | S5; | 1845.8 | Yes |
| **CASKIN2** | NP_001136115.1 | 57513 | caskin-2 isoform b | **S321** | 1.19 | | #N/A | VGLSPDSPAGDRNsVGSEGSVGSIR | 1 | 2 | 1 | 1 | R12(Label:13C(6)); S14(Phospho); R25(Label:13C(6)) | S(4): 0.0; S(7): 0.0; S(14): 97.7; S(17): 2.3; S(20): 0.0; S(23): 0.0 | SPAGDRNsVGSEGSV | S14; | 2492.2 | No |
| **CASKIN2** | NP_001136115.1 | 57513 | caskin-2 isoform b | **S321** | | | #N/A | VGLSPDSPAGDRNsVGSEGSVGSIR | 1 | 2 | 1 | 1 | S14(Phospho) | S(4): 0.0; S(7): 0.0; S(14): 99.8; S(17): 0.1; S(20): 0.0; S(23): 0.1 | SPAGDRNsVGSEGSV | S14; | 2480.1 | No |
| **CASKIN2** | NP_001136115.1 | 57513 | caskin-2 isoform b | **S613** | | **2.64** | #N/A | LLTFQGSELSPELQAAMAGGGPEPLPLPPARsPSQESIGAR | 2 | 2 | 1 | 1 | S32(Phospho) | T(3): 0.0; S(7): 0.0; S(10): 0.0; S(32): 96.7; S(34): 3.2; S(37): 0.1 | LPLPPARsPSQESIG | S32; | 4237.1 | No |
| **CASKIN2** | NP_001136115.1 | 57513 | caskin-2 isoform b | **S613** | | **2.72** | #N/A | LLTFQGSELSPELQAAMAGGGPEPLPLPPARsPSQESIGAR | 1 | 2 | 1 | 1 | R31(Label:13C(6)); S32(Phospho); R41(Label:13C(6)) | T(3): 0.0; S(7): 0.0; S(10): 0.0; S(32): 99.9; S(34): 0.1; S(37): 0.0 | LPLPPARsPSQESIG | S32; | 4249.1 | No |
| **CASP4** | NP_001216.1 | 837 | caspase-4 isoform alpha precursor | **S83** | **3.98** | **2.48** | 1.87 | MAGQMLLQTFFNIDQIsPNKK | 33 | 2 | 1 | 1 | S17(Phospho) | T(9): 0.0; S(17): 100.0 | FFNIDQIsPNKKAHP | S17; | 2504.2 | Yes |
| **CASP4** | NP_001216.1 | 837 | caspase-4 isoform alpha precursor | **S83** | 1.06 | | 1.87 | MAGQMLLQTFFNIDQIsPNKK | 18 | 2 | 1 | 1 | M1(Oxidation); S17(Phospho) | T(9): 0.0; S(17): 100.0 | FFNIDQIsPNKKAHP | S17; | 2520.2 | Yes |
| **CASP4** | NP_001216.1 | 837 | caspase-4 isoform alpha precursor | **S83** | | | 1.87 | MAGQMLLQTFFNIDQIsPNKK | 15 | 2 | 1 | 1 | S17(Phospho); K20(Label:13C(6)15N(2)); K21(Label:13C(6)15N(2)) | T(9): 0.0; S(17): 100.0 | FFNIDQIsPNKKAHP | S17; | 2520.2 | Yes |
| **CASP4** | NP_001216.1 | 837 | caspase-4 isoform alpha precursor | **S83** | | **3.27** | 1.87 | MAGQMLLQTFFNIDQIsPNK | 1 | 2 | 1 | 0 | S17(Phospho) | T(9): 0.0; S(17): 100.0 | FFNIDQIsPNKKAHP | S17; | 2376.1 | Yes |
| **CAST** | NP_001271141.1 | 831 | calpastatin isoform n | **S202** | | 1.04 | 1.39 | KEGITGPPADSSKPIGPDDAIDALSSDFTCGsPTAAGK | 2 | 4 | 1 | 1 | C30(Carbamidomethyl); S32(Phospho) | T(5): 0.0; S(11): 0.0; S(12): 0.0; S(25): 0.0; S(26): 0.0; T(29): 0.0; S(32): 97.7; T(34): 2.3 | SSDFTCGsPTAAGKK | S32; | 3810.7 | No |
| **CAV1** | NP_001744.2 | 857 | caveolin-1 isoform alpha | **S37** | 1.7 | 1.68 | 1.20 | AMADELsEK | 6 | 1 | 1 | 0 | M2(Oxidation); S7(Phospho) | S(7): 100.0 | KAMADELsEKQVYDA | S7; | 1089.4 | Yes |
| **CAV1** | NP_001744.2 | 857 | caveolin-1 isoform alpha | **S37** | | 1.48 | 1.20 | AMADELsEK | 4 | 1 | 1 | 0 | M2(Oxidation); S7(Phospho); K9(Label:13C(6)15N(2)) | S(7): 100.0 | KAMADELsEKQVYDA | S7; | 1097.4 | Yes |
| **CAV1** | NP_001744.2 | 857 | caveolin-1 isoform alpha | **S37** | 1.84 | 1.59 | 1.20 | AMADELsEKQVYDAHTK | 3 | 1 | 1 | 1 | S7(Phospho); K9(Label:13C(6)15N(2)); K17(Label:13C(6)15N(2)) | S(7): 100.0; Y(12): 0.0; T(16): 0.0 | KAMADELsEKQVYDA | S7; | 2031.9 | Yes |
| **CAV1** | NP_001744.2 | 857 | caveolin-1 isoform alpha | **S37** | 1.94 | 1.54 | 1.20 | AMADELsEKQVYDAHTK | 2 | 1 | 1 | 1 | S7(Phospho) | S(7): 100.0; Y(12): 0.0; T(16): 0.0 | KAMADELsEKQVYDA | S7; | 2015.9 | Yes |
| **CAV1** | NP_001744.2 | 857 | caveolin-1 isoform alpha | **S37** | | 1.28 | 1.20 | AMADELsEK | 3 | 1 | 1 | 0 | S7(Phospho); K9(Label:13C(6)15N(2)) | S(7): 100.0 | KAMADELsEKQVYDA | S7; | 1081.4 | Yes |
| **CAV1** | NP_001744.2 | 857 | caveolin-1 isoform alpha | **S37** | | 1.32 | 1.20 | AMADELsEK | 3 | 1 | 1 | 0 | S7(Phospho) | S(7): 100.0 | KAMADELsEKQVYDA | S7; | 1073.4 | Yes |
| **CAV1** | NP_001166367.1 | 857 | caveolin-1 isoform beta | **S6** | | **3.38** | 1.20 | MADELsEK | 1 | 1 | 1 | 0 | S6(Phospho); K8(Label:13C(6)15N(2)) | S(6): 100.0 | _MADELsEKQVYDA | S6; | 1010.4 | No |
| **CAV2** | NP_937855.1 | 858 | caveolin-2 isoform c | **S20; S23** | | 0.65 | #NQ | ADVQLFMDDDSYsHHsGLEYADPEK | 2 | 2 | 1 | 0 | S13(Phospho); S16(Phospho) | S(11): 2.5; Y(12): 2.5; S(13): 95.0; S(16): 100.0; Y(20): 0.0 | FMDDDSYsHHSGLEY; DDSYSHHsGLEYADP | S13; S16; | 3029.2 | Yes;Yes |
| **CAV2** | NP_937855.1 | 858 | caveolin-2 isoform c | **S23** | | | #NQ | ADVQLFMDDDSYSHHsGLEYADPEK | 2 | 2 | 1 | 0 | S11(Phospho); S16(Phospho); K25(Label:13C(6)15N(2)) | S(11): 33.3; Y(12): 33.3; S(13): 33.4; S(16): 100.0; Y(20): 0.0 | DDSYSHHsGLEYADP | S16; | 3037.2 | Yes |
| **CBFA2T2** | NP_001034798.1 | 9139 | protein CBFA2T2 isoform MTGR1a | **S235** | | 1.43 | #N/A | VCTIsPAPR | 2 | 3 | 1 | 0 | C2(Carbamidomethyl); S5(Phospho) | T(3): 0.0; S(5): 100.0 | AKRVCTIsPAPRHSP | S5; | 1080.5 | No |
| **CBFB** | NP_074036.1 | 865 | core-binding factor subunit beta isoform 1 | **S173** | | 1.89 | 1.33 | RQQDPsPGSNLGGGDDLKLR | 3 | 1 | 1 | 2 | S6(Phospho) | S(6): 100.0; S(9): 0.0 | ARRQQDPsPGSNLGG | S6; | 2190.0 | Yes |
| **CBFB** | NP_074036.1 | 865 | core-binding factor subunit beta isoform 1 | **S173** | | **2.38** | 1.33 | QQDPsPGSNLGGGDDLKLR | 1 | 1 | 1 | 1 | S8(Phospho) | S(5): 100.0; S(8): 0.0 | ARRQQDPsPGSNLGG | S5; | 2033.9 | Yes |
| **CBFB** | NP_074036.1 | 865 | core-binding factor subunit beta isoform 1 | **S173** | | | 1.33 | RQQDPsPGSNLGGGDDLKLR | 1 | 1 | 1 | 2 | R1(Label:13C(6)); S6(Phospho); K18(Label:13C(6)15N(2)); R20(Label:13C(6)) | S(6): 100.0; S(9): 0.0 | ARRQQDPsPGSNLGG | S6; | 2210.1 | Yes |
| **CBL** | NP_005179.2 | 867 | E3 ubiquitin-protein ligase CBL | **S483** | | 1.61 | 0.71 | VERPPsPFSMAPQASLPPVPPR | 6 | 1 | 1 | 0 | S6(Phospho) | S(6): 98.3; S(9): 1.7; S(15): 0.0 | AKVERPPsPFSMAPQ | S6; | 2437.2 | Yes |
| **CBL** | NP_005179.2 | 867 | E3 ubiquitin-protein ligase CBL | **S483** | **6.75** | **3.11** | 0.71 | VERPPsPFSMAPQASLPPVPPRLDLLPQR | 4 | 1 | 1 | 1 | S6(Phospho) | S(6): 97.3; S(9): 2.7; S(15): 0.0 | AKVERPPsPFSMAPQ | S6; | 3272.7 | Yes |
| **CBX1** | NP_006798.1 | 10951 | chromobox protein homolog 1 | **S89** | 1.14 | 1.14 | 1.30 | KADsDSEDKGEESKPK | 2 | 1 | 1 | 2 | S4(Phospho) | S(4): 100.0; S(6): 0.0; S(13): 0.0 | GGKRKADsDSEDKGE | S4; | 1829.8 | Yes |
| **CBX3** | NP_009207.2 | 11335 | chromobox protein homolog 3 | **S95** | 0.79 | | 0.97 | KSLsDSESDDSK | 2 | 1 | 1 | 1 | K1(Label:13C(6)15N(2)); S4(Phospho); K12(Label:13C(6)15N(2)) | S(2): 0.0; S(4): 100.0; S(6): 0.0; S(8): 0.0; S(11): 0.0 | GTKRKSLsDSESDDS | S4; | 1393.6 | Yes |
| **CBX3** | NP_009207.2 | 11335 | chromobox protein homolog 3 | **S95** | 0.92 | | 0.97 | SLsDSESDDSK | 2 | 1 | 1 | 0 | S3(Phospho) | S(1): 0.0; S(3): 100.0; S(5): 0.0; S(7): 0.0; S(10): 0.0 | GTKRKSLsDSESDDS | S3; | 1249.4 | Yes |
| **CBX3** | NP_009207.2 | 11335 | chromobox protein homolog 3 | **S95** | | | 0.97 | KSLsDSESDDSK | 2 | 1 | 1 | 1 | S4(Phospho) | S(2): 0.0; S(4): 100.0; S(6): 0.0; S(8): 0.0; S(11): 0.0 | GTKRKSLsDSESDDS | S4; | 1377.5 | Yes |
| **CBX3** | NP_009207.2 | 11335 | chromobox protein homolog 3 | **S95** | | | 0.97 | SLsDSESDDSK | 2 | 1 | 1 | 0 | S3(Phospho); K11(Label:13C(6)15N(2)) | S(1): 0.0; S(3): 100.0; S(5): 0.0; S(7): 0.0; S(10): 0.0 | GTKRKSLsDSESDDS | S3; | 1257.5 | Yes |
| **CBX3** | NP_009207.2 | 11335 | chromobox protein homolog 3 | **S95** | 1.16 | | 0.97 | SLsDSESDDSKSK | 2 | 1 | 1 | 1 | S3(Phospho); K11(Label:13C(6)15N(2)); K13(Label:13C(6)15N(2)) | S(1): 0.0; S(3): 100.0; S(5): 0.0; S(7): 0.0; S(10): 0.0; S(12): 0.0 | GTKRKSLsDSESDDS | S3; | 1480.6 | Yes |
| **CBX3** | NP_009207.2 | 11335 | chromobox protein homolog 3 | **S93** | 0.92 | | 0.97 | RKsLSDSESDDSK | 2 | 1 | 1 | 2 | S3(Phospho) | S(3): 100.0; S(5): 0.0; S(7): 0.0; S(9): 0.0; S(12): 0.0 | KDGTKRKsLSDSESD | S3; | 1533.6 | Yes |
| **CBX3** | NP_009207.2 | 11335 | chromobox protein homolog 3 | **S95** | 0.95 | | 0.97 | KSLsDSESDDSKSK | 2 | 1 | 1 | 2 | S4(Phospho) | S(2): 0.0; S(4): 100.0; S(6): 0.0; S(8): 0.0; S(11): 0.0; S(13): 0.0 | GTKRKSLsDSESDDS | S4; | 1592.7 | Yes |
| **CBX3** | NP_009207.2 | 11335 | chromobox protein homolog 3 | **S176** | | | 0.97 | LTWHsCPEDEAQ | 6 | 1 | 1 | 0 | S5(Phospho); C6(Carbamidomethyl) | T(2): 0.8; S(5): 99.2 | EERLTWHsCPEDEAQ | S5; | 1552.6 | Yes |
| **CBX8** | NP_065700.1 | 57332 | chromobox protein homolog 8 | **S191** | **0.43** | | #N/A | VDDKPSsPGDSSK | 2 | 1 | 1 | 0 | K4(Label:13C(6)15N(2)); S7(Phospho); K13(Label:13C(6)15N(2)) | S(6): 0.0; S(7): 100.0; S(11): 0.0; S(12): 0.0 | RVDDKPSsPGDSSKK | S7; | 1414.6 | Yes |
| **CBY1** | NP_056188.1 | 25776 | protein chibby homolog 1 isoform a | **S20** | 1.56 | 1.5 | #N/A | SAsLSNLHSLDR | 4 | 2 | 1 | 0 | S3(Phospho) | S(1): 0.0; S(3): 100.0; S(5): 0.0; S(9): 0.0 | TPPRKSAsLSNLHSL | S3; | 1379.6 | Yes |
| **CBY1** | NP_056188.1 | 25776 | protein chibby homolog 1 isoform a | **S9** | | **2.53** | #N/A | PFFGNTFsPK | 2 | 1 | 1 | 0 | S8(Phospho) | T(6): 0.0; S(8): 100.0 | PFFGNTFsPKKTPPR | S8; | 1221.5 | Yes |
| **CBY1** | NP_056188.1 | 25776 | protein chibby homolog 1 isoform a | **S9** | | | #N/A | PFFGNTFsPK | 1 | 1 | 1 | 0 | S8(Phospho); K10(Label:13C(6)15N(2)) | T(6): 0.0; S(8): 100.0 | PFFGNTFsPKKTPPR | S8; | 1229.5 | Yes |
| **CBY1** | NP_056188.1 | 25776 | protein chibby homolog 1 isoform a | **S20** | | | #N/A | SAsLSNLHSLDR | 1 | 2 | 1 | 0 | S3(Phospho); R12(Label:13C(6)) | S(1): 0.0; S(3): 100.0; S(5): 0.0; S(9): 0.0 | TPPRKSAsLSNLHSL | S3; | 1385.7 | Yes |
| **CC2D1A** | NP_060191.3 | 54862 | coiled-coil and C2 domain-containing protein 1A | **S118** | | 1.07 | 1.50 | AsETPPPVAQPKPEAPHPGLETTLQER | 1 | 1 | 1 | 0 | T4(Phospho) | S(2): 97.8; T(4): 2.2; T(22): 0.0; T(23): 0.0 | LGEEQKAsETPPPVA | S2; | 2957.4 | No |
| **CCAR2** | NP_066997.3 | 57805 | cell cycle and apoptosis regulator protein 2 | **S675; S678** | | 0.55 | 0.82 | sVAsNQSEMEFSSLQDMPK | 7 | 1 | 1 | 0 | S1(Phospho); S4(Phospho); K19(Label:13C(6)15N(2)) | S(1): 100.0; S(4): 100.0; S(7): 0.0; S(12): 0.0; S(13): 0.0 | LEDSEVRsVASNQSE; SEVRSVAsNQSEMEF | S1; S4; | 2282.9 | Yes;Yes |
| **CCAR2** | NP_066997.3 | 57805 | cell cycle and apoptosis regulator protein 2 | **T35** | 0.74 | | 0.82 | NFSGTASTSLLGPPPGLLtPPVATELSQNAR | 1 | 1 | 1 | 0 | T19(Phospho); R31(Label:13C(6)) | S(3): 0.0; T(5): 0.0; S(7): 0.0; T(8): 0.0; S(9): 0.0; T(19): 100.0; T(24): 0.0; S(27): 0.0 | GPPPGLLtPPVATEL | T19; | 3179.6 | Yes |
| **CCAR2** | NP_066997.3 | 57805 | cell cycle and apoptosis regulator protein 2 | **S678; S681** | | 0.82 | 0.82 | SVAsNQsEMEFSSLQDMPK | 2 | 1 | 1 | 0 | S4(Phospho); S7(Phospho) | S(1): 2.1; S(4): 97.9; S(7): 100.0; S(12): 0.0; S(13): 0.0 | SEVRSVAsNQSEMEF; RSVASNQsEMEFSSL | S4; S7; | 2274.9 | Yes;Yes |
| **CCAR2** | NP_066997.3 | 57805 | cell cycle and apoptosis regulator protein 2 | **S124** | | 0.83 | 0.82 | VQTLSNQPLLKsPAPPLLHVAALGQK | 1 | 1 | 1 | 1 | K11(Label:13C(6)15N(2)); S12(Phospho); K26(Label:13C(6)15N(2)) | T(3): 1.8; S(5): 0.0; S(12): 98.2 | SNQPLLKsPAPPLLH | S12; | 2816.6 | Yes |
| **CCDC12** | NP_001264003.1 | 151903 | coiled-coil domain-containing protein 12 2 | **S165** | 0.84 | | 1.17 | LKGQEDSLASAVDAATEQKTCDsD | 6 | 2 | 1 | 2 | C21(Carbamidomethyl); S23(Phospho) | S(7): 0.0; S(10): 0.0; T(16): 0.0; T(20): 0.0; S(23): 100.0 | TEQKTCDsD_ | S23; | 2619.1 | Yes |
| **CCDC12** | NP_001264003.1 | 151903 | coiled-coil domain-containing protein 12 2 | **S165** | | | 1.17 | LKGQEDSLASAVDAATEQKTCDsD | 3 | 2 | 1 | 2 | K2(Label:13C(6)15N(2)); K19(Label:13C(6)15N(2)); C21(Carbamidomethyl); S23(Phospho) | S(7): 0.0; S(10): 0.0; T(16): 0.0; T(20): 0.0; S(23): 100.0 | TEQKTCDsD_ | S23; | 2635.1 | Yes |
| **CCDC120** | NP_001156795.1 | 90060 | coiled-coil domain-containing protein 120 isoform 4 | **S348** | | | #N/A | RSNsSEALLVDR | 1 | 4 | 1 | 1 | R1(Label:13C(6)); S4(Phospho); R12(Label:13C(6)) | S(2): 0.0; S(4): 100.0; S(5): 0.0 | ARTRRSNsSEALLVD | S4; | 1438.7 | No |
| **CCDC120** | NP_001156795.1 | 90060 | coiled-coil domain-containing protein 120 isoform 4 | **T276** | **0.08** | **0.11** | #N/A | NSVASPTSPtR | 2 | 4 | 1 | 0 | S8(Phospho); R11(Label:13C(6)) | S(2): 0.0; S(5): 0.0; T(7): 0.0; S(8): 0.0; T(10): 100.0 | VASPTSPtRSLPRSA | T10; | 1202.6 | No |

| GeneSymbol | NP_Accession | Gene ID | ProteinDescription | PhosphoSite (Protein) | SCC-R / SCC-S R1 | SCC-R / SCC-S R2 | SCC-R / SCC-S Total Proteome | Sequence | # PSMs | # Proteins | # Protein Groups | # Missed Cleavages | Modifications | phosphoRS Site Probabilities | Phosphowindow | PhosphoSite (Peptide) | MH+ [Da] | PhosphositePlusEvidence |
|---|---|---|---|---|---|---|---|---|---|---|---|---|---|---|---|---|---|---|
| **CCDC120** | NP_001156795.1 | 90060 | coiled-coil domain-containing protein 120 isoform 4 | **S364** | | | #N/A | AAGGGAGsPPAPLAPSASGPPVCK | 2 | 4 | 1 | 0 | S8(Phospho); C23(Carbamidomethyl); K24(Label:13C(6)15N(2)) | S(8): 100.0; S(16): 0.0; S(18): 0.0 | AAGGGAGsPPAPLAP | S8; | 2162.0 | No |
| **CCDC120** | NP_001156795.1 | 90060 | coiled-coil domain-containing protein 120 isoform 4 | **S290** | | 0.14 | #N/A | sVPATPVLTR | 1 | 4 | 1 | 0 | S1(Phospho); R10(Label:13C(6)) | S(1): 100.0; T(5): 0.0; T(9): 0.0 | ASSFEGRsVPATPVL | S1; | 1126.6 | No |
| **CCDC137** | NP_954981.1 | 339230 | coiled-coil domain-containing protein 137 | **S19** | 0.83 | 0.84 | 0.84 | VQAGPGsPR | 7 | 1 | 1 | 0 | S7(Phospho) | S(7): 100.0 | RVQAGPGsPRRARGR | S7; | 948.4 | Yes |
| **CCDC137** | NP_954981.1 | 339230 | coiled-coil domain-containing protein 137 | **S19** | 0.78 | | 0.84 | VQAGPGsPR | 4 | 1 | 1 | 0 | S7(Phospho); R9(Label:13C(6)) | S(7): 100.0 | RVQAGPGsPRRARGR | S7; | 954.4 | Yes |
| **CCDC137** | NP_954981.1 | 339230 | coiled-coil domain-containing protein 137 | **S233** | | 0.5 | 0.84 | MLLsPGGVSQPLTASLAR | 1 | 1 | 1 | 0 | S4(Phospho); R18(Label:13C(6)) | S(4): 100.0; S(9): 0.0; T(13): 0.0; S(15): 0.0 | QMLRMLLsPGGVSQP | S4; | 1884.0 | Yes |
| **CCDC137** | NP_954981.1 | 339230 | coiled-coil domain-containing protein 137 | **S19** | | | 0.84 | VQAGPGsPRR | 1 | 1 | 1 | 1 | S7(Phospho); R9(Label:13C(6)); R10(Label:13C(6)) | S(7): 100.0 | RVQAGPGsPRRARGR | S7; | 1116.6 | Yes |
| **CCDC158** | NP_001036249.1 | 339965 | coiled-coil domain-containing protein 158 | **T467** | | | #N/A | NESLEKVSSLtAQLESTKEMLR | 2 | 1 | 1 | 2 | K6(Label:13C(6)15N(2)); S9(Phospho); S16(Phospho); R22(Label:13C(6)) | S(3): 0.3; S(8): 0.3; S(9): 0.3; T(11): 99.1; S(16): 50.0; T(17): 50.0 | LEKVSSLtAQLESTK | T11; | 2667.2 | No |
| **CCDC22** | NP_054727.1 | 28952 | coiled-coil domain-containing protein 22 | **T285** | | | 1.35 | tGAPKGSRFTHSEK | 1 | 1 | 1 | 2 | S7(Phospho) | T(1): 96.2; S(7): 3.5; T(10): 0.2; S(12): 0.2 | AWGAGAKtGAPKGSR | T1; | 1582.7 | No |
| **CCDC42B** | NP_001138344.1 | 387885 | coiled-coil domain-containing protein 42B | **T60** | | | #N/A | TKtAALK | 1 | 1 | 1 | 1 | T3(Phospho) | T(1): 1.4; T(3): 98.6 | KEVFRTKtAALKQRW | T3; | 812.4 | No |
| **CCDC50** | NP_848018.1 | 152137 | coiled-coil domain-containing protein 50 long isoform | **T310** | | 0.45 | 1.47 | tPPFSESEEQLHLHDAGMKPR | 1 | 1 | 1 | 0 | T1(Phospho) | T(1): 100.0; S(5): 0.0; S(7): 0.0 | KRRHRPRtPPFSESE | T1; | 2486.1 | Yes |
| **CCDC50** | NP_848018.1 | 152137 | coiled-coil domain-containing protein 50 long isoform | **T310** | | 0.46 | 1.47 | tPPFSESEEQLHLHDAGMKPR | 1 | 1 | 1 | 0 | T1(Phospho); K19(Label:13C(6)15N(2)); R21(Label:13C(6)) | T(1): 100.0; S(5): 0.0; S(7): 0.0 | KRRHRPRtPPFSESE | T1; | 2500.1 | Yes |
| **CCDC6** | NP_005427.2 | 8030 | coiled-coil domain-containing protein 6 | **S244** | 0.7 | 0.69 | 0.68 | ILQEKLDQPVSAPPsPR | 9 | 1 | 1 | 1 | S15(Phospho) | S(11): 0.0; S(15): 100.0 | QPVSAPPsPRDISME | S15; | 1955.0 | Yes |
| **CCDC6** | NP_005427.2 | 8030 | coiled-coil domain-containing protein 6 | **S52** | 0.34 | 0.49 | 0.68 | SGGIVIsPFRLEELTNR | 3 | 1 | 1 | 1 | S7(Phospho) | S(1): 0.0; S(7): 100.0; T(15): 0.0 | KSGGIVIsPFRLEEL | S7; | 1968.0 | Yes |
| **CCDC6** | NP_005427.2 | 8030 | coiled-coil domain-containing protein 6 | **S244** | 0.74 | 0.72 | 0.68 | ILQEKLDQPVSAPPsPR | 5 | 1 | 1 | 1 | K5(Label:13C(6)15N(2)); S15(Phospho); R17(Label:13C(6)) | S(11): 0.0; S(15): 100.0 | QPVSAPPsPRDISME | S15; | 1969.0 | Yes |
| **CCDC6** | NP_005427.2 | 8030 | coiled-coil domain-containing protein 6 | **S52** | 0.35 | 0.5 | 0.68 | SGGIVIsPFRLEELTNR | 2 | 1 | 1 | 1 | S7(Phospho); R10(Label:13C(6)); R17(Label:13C(6)) | S(1): 0.0; S(7): 100.0; T(15): 0.0 | KSGGIVIsPFRLEEL | S7; | 1980.0 | Yes |
| **CCDC6** | NP_005427.2 | 8030 | coiled-coil domain-containing protein 6 | **S240; S244** | | 1.6 | 0.68 | LDQPVsAPPsPR | 5 | 1 | 1 | 0 | S6(Phospho); S10(Phospho) | S(6): 100.0; S(10): 100.0 | EKLDQPVsAPPSPRD; QPVSAPPsPRDISME | S6; S10; | 1423.6 | Yes;Yes |
| **CCDC6** | NP_005427.2 | 8030 | coiled-coil domain-containing protein 6 | **S254** | 0.43 | | 0.68 | DISMEIDsPENMMR | 1 | 1 | 1 | 0 | S8(Phospho); R14(Label:13C(6)) | S(3): 0.0; S(8): 100.0 | DISMEIDsPENMMRH | S8; | 1753.7 | Yes |
| **CCDC6** | NP_005427.2 | 8030 | coiled-coil domain-containing protein 6 | **S240; S244** | | 1.79 | 0.68 | ILQEKLDQPVsAPPsPR | 12 | 1 | 1 | 1 | S11(Phospho); S15(Phospho) | S(11): 100.0; S(15): 100.0 | EKLDQPVsAPPSPRD; QPVSAPPsPRDISME | S11; S15; | 2035.0 | Yes;Yes |
| **CCDC6** | NP_005427.2 | 8030 | coiled-coil domain-containing protein 6 | **S240; S244** | | 2.51 | 0.68 | ILQEKLDQPVsAPPsPR | 3 | 1 | 1 | 1 | K5(Label:13C(6)15N(2)); S11(Phospho); S15(Phospho); R17(Label:13C(6)) | S(11): 100.0; S(15): 100.0 | EKLDQPVsAPPSPRD; QPVSAPPsPRDISME | S11; S15; | 2049.0 | Yes;Yes |
| **CCDC6** | NP_005427.2 | 8030 | coiled-coil domain-containing protein 6 | **S240; S244** | | 1.57 | 0.68 | RILQEKLDQPVsAPPsPR | 1 | 1 | 1 | 2 | S12(Phospho); S16(Phospho) | S(12): 100.0; S(16): 100.0 | EKLDQPVsAPPSPRD; QPVSAPPsPRDISME | S12; S16; | 2191.1 | Yes;Yes |
| **CCDC6** | NP_005427.2 | 8030 | coiled-coil domain-containing protein 6 | **S240; S244** | | | 0.68 | LDQPVsAPPsPR | 2 | 1 | 1 | 0 | S6(Phospho); S10(Phospho); R12(Label:13C(6)) | S(6): 100.0; S(10): 100.0 | EKLDQPVsAPPSPRD; QPVSAPPsPRDISME | S6; S10; | 1429.6 | Yes;Yes |
| **CCDC82** | NP_079001.2 | 79780 | coiled-coil domain-containing protein 82 | **S219** | | 3.88 | 3.16 | RVVEDEGsSVEMEQKTPEK | 2 | 1 | 1 | 2 | S8(Phospho) | S(8): 98.6; S(9): 1.4; T(16): 0.0 | RVVEDEGsSVEMEQK | S8; | 2257.0 | Yes |
| **CCDC82** | NP_079001.2 | 79780 | coiled-coil domain-containing protein 82 | **S131** | | 5 | 3.16 | HLsQEDNDLNK | 2 | 1 | 1 | 0 | S3(Phospho) | S(3): 100.0 | QDQEKHLsQEDNDLN | S3; | 1392.6 | Yes |
| **CCDC82** | NP_079001.2 | 79780 | coiled-coil domain-containing protein 82 | **S88** | | | 3.16 | ELNLsKIQSEGNDSK | 1 | 1 | 1 | 1 | S5(Phospho) | S(5): 99.9; S(9): 0.1; S(14): 0.0 | SERELNLsKIQSEGN | S5; | 1741.8 | Yes |
| **CCDC85C** | NP_001138467.1 | 317762 | coiled-coil domain-containing protein 85C | **S246** | 0.16 | | #NQ | sLDDLSAPPHHR | 2 | 1 | 1 | 0 | S1(Phospho); R12(Label:13C(6)) | S(1): 100.0; S(6): 0.0 | KAGATRRsLDDLSAP | S1; | 1430.7 | Yes |
| **CCDC86** | NP_077003.1 | 79080 | coiled-coil domain-containing protein 86 | **S58** | 0.82 | 0.83 | 0.92 | AGLGsPERPPK | 5 | 1 | 1 | 0 | S5(Phospho) | S(5): 100.0 | VQRAGLGsPERPPKT | S5; | 1188.6 | Yes |
| **CCDC86** | NP_077003.1 | 79080 | coiled-coil domain-containing protein 86 | **S47** | 0.91 | 0.91 | 0.92 | ALVEFESNPEETREPGsPPSVQR | 7 | 1 | 1 | 1 | S17(Phospho) | S(7): 0.0; T(12): 0.0; S(17): 100.0; S(20): 0.0 | EETREPGsPPSVQRA | S17; | 2635.2 | Yes |
| **CCDC86** | NP_077003.1 | 79080 | coiled-coil domain-containing protein 86 | **S91** | 1.2 | 1.06 | 0.92 | LQQGAGLESPQGQPEPGAAsPQR | 11 | 1 | 1 | 0 | S20(Phospho) | S(9): 0.0; S(20): 100.0 | QPEPGAAsPQRQQDL | S20; | 2383.1 | Yes |
| **CCDC86** | NP_077003.1 | 79080 | coiled-coil domain-containing protein 86 | **S18** | 0.88 | 0.88 | 0.92 | LGGLRPEsPESLTSVSR | 15 | 1 | 1 | 0 | S8(Phospho) | S(8): 100.0; S(11): 0.0; T(13): 0.0; S(14): 0.0; S(16): 0.0 | LGGLRPEsPESLTSV | S8; | 1864.9 | Yes |
| **CCDC86** | NP_077003.1 | 79080 | coiled-coil domain-containing protein 86 | **S91** | 1.1 | | 0.92 | LQQGAGLESPQGQPEPGAAsPQRQQDLHLESPQR | 1 | 1 | 1 | 1 | S20(Phospho) | S(9): 0.0; S(20): 100.0; S(31): 0.0 | QPEPGAAsPQRQQDL | S20; | 3714.8 | Yes |
| **CCDC86** | NP_077003.1 | 79080 | coiled-coil domain-containing protein 86 | **S91** | | | 0.92 | LQQGAGLESPQGQPEPGAAsPQRQQDLHLESPQR | 1 | 1 | 1 | 1 | S20(Phospho); R23(Label:13C(6)); R34(Label:13C(6)) | S(9): 0.0; S(20): 100.0; S(31): 0.0 | QPEPGAAsPQRQQDL | S20; | 3726.8 | Yes |
| **CCDC86** | NP_077003.1 | 79080 | coiled-coil domain-containing protein 86 | **S47** | 0.53 | | 0.92 | ALVEFESNPEETREPGsPPSVQR | 5 | 1 | 1 | 1 | R13(Label:13C(6)); S17(Phospho); R23(Label:13C(6)) | S(7): 0.0; T(12): 0.0; S(17): 100.0; S(20): 0.0 | EETREPGsPPSVQRA | S17; | 2647.2 | Yes |
| **CCDC86** | NP_077003.1 | 79080 | coiled-coil domain-containing protein 86 | **S91** | 1.11 | 1.13 | 0.92 | LQQGAGLESPQGQPEPGAAsPQR | 9 | 1 | 1 | 0 | S20(Phospho); R23(Label:13C(6)) | S(9): 0.0; S(20): 100.0 | QPEPGAAsPQRQQDL | S20; | 2389.1 | Yes |
| **CCDC86** | NP_077003.1 | 79080 | coiled-coil domain-containing protein 86 | **S18** | 1.05 | 0.9 | 0.92 | LGGLRPEsPESLTSVSR | 9 | 1 | 1 | 0 | R5(Label:13C(6)); S8(Phospho); R17(Label:13C(6)) | S(8): 100.0; S(11): 0.0; T(13): 0.0; S(14): 0.0; S(16): 0.0 | LGGLRPEsPESLTSV | S8; | 1877.0 | Yes |
| **CCDC86** | NP_077003.1 | 79080 | coiled-coil domain-containing protein 86 | **S66** | 1.6 | | 0.92 | AGLGSPERPPKTsPGSPR | 1 | 1 | 1 | 1 | S16(Phospho) | S(5): 0.0; T(12): 1.6; S(13): 98.3; S(16): 0.0 | PERPPKTsPGSPRLQ | S13; | 1870.9 | Yes |
| **CCDC86** | NP_077003.1 | 79080 | coiled-coil domain-containing protein 86 | **S58** | | 0.91 | 0.92 | AGLGsPERPPK | 2 | 1 | 1 | 0 | S5(Phospho); R8(Label:13C(6)); K11(Label:13C(6)15N(2)) | S(5): 100.0 | VQRAGLGsPERPPKT | S5; | 1202.6 | Yes |
| **CCDC86** | NP_077003.1 | 79080 | coiled-coil domain-containing protein 86 | **S47** | | 0.9 | 0.92 | RALVEFESNPEETREPGsPPSVQR | 3 | 1 | 1 | 2 | S18(Phospho) | S(8): 0.0; T(13): 0.0; S(18): 100.0; S(21): 0.0 | EETREPGsPPSVQRA | S18; | 2791.3 | Yes |
| **CCDC86** | NP_077003.1 | 79080 | coiled-coil domain-containing protein 86 | **S91; S113** | | 1.55 | 0.92 | LQQGAGLESPQGQPEPGAAsPQRQQDLHLESPQRQPEYSPEsPR | 1 | 1 | 1 | 2 | S20(Phospho); S31(Phospho) | S(9): 0.0; S(20): 99.4; S(31): 0.5; Y(38): 1.0; S(39): 6.5; S(42): 92.6 | QPEPGAAsPQRQQDL; QPEYSPEsPRCQPKP | S20; S42; | 4965.3 | Yes;Yes |
| **CCDC86** | NP_077003.1 | 79080 | coiled-coil domain-containing protein 86 | **S47** | | 0.93 | 0.92 | RALVEFESNPEETREPGsPPSVQR | 2 | 1 | 1 | 2 | R1(Label:13C(6)); R14(Label:13C(6)); S18(Phospho); R24(Label:13C(6)) | S(8): 0.0; T(13): 0.0; S(18): 100.0; S(21): 0.0 | EETREPGsPPSVQRA | S18; | 2809.4 | Yes |
| **CCDC86** | NP_077003.1 | 79080 | coiled-coil domain-containing protein 86 | **S58; S69** | | 0.82 | 0.92 | AGLGsPERPPKTSPGsPR | 1 | 1 | 1 | 1 | S5(Phospho); S13(Phospho); S16(Phospho) | S(5): 100.0; T(12): 50.0; S(13): 50.0; S(16): 100.0 | VQRAGLGsPERPPKT; PPKTSPGsPRLQQGA | S5; S16; | 2030.8 | Yes;Yes |
| **CCDC88A** | NP_001241872.1 | 55704 | girdin isoform 3 | **S1386** | | 1.55 | #NQ | FYDPsPPR | 1 | 3 | 1 | 0 | S5(Phospho) | Y(2): 0.0; S(5): 100.0 | QYKFYDPsPPRRRGN | S5; | 1058.4 | No |
| **CCDC88B** | NP_115627.6 | 283234 | coiled-coil domain-containing protein 88B precursor | **T1387** | | | #N/A | RAQSSLCLRDEtLAGGQR | 1 | 1 | 1 | 2 | R1(Label:13C(6)); S4(Phospho); C7(Carbamidomethyl) | S(4): 3.2; S(5): 3.2; T(12): 93.5 | SLCLRDEtLAGGQRR | T12; | 2104.0 | No |
| **CCDC9** | NP_056418.1 | 26093 | coiled-coil domain-containing protein 9 | **S376** | 1.63 | 1.42 | #NQ | EGAAsPAPETPQPTSPETSPK | 4 | 1 | 1 | 0 | S5(Phospho); K21(Label:13C(6)15N(2)) | S(5): 100.0; T(10): 0.0; T(14): 0.0; S(15): 0.0; T(18): 0.0; S(19): 0.0 | ETKEGAAsPAPETPQ | S5; | 2167.0 | Yes |
| **CCDC9** | NP_056418.1 | 26093 | coiled-coil domain-containing protein 9 | **S376** | | | #NQ | EGAAsPAPETPQPTSPETSPK | 2 | 1 | 1 | 0 | S5(Phospho) | S(5): 100.0; T(10): 0.0; T(14): 0.0; S(15): 0.0; T(18): 0.0; S(19): 0.0 | ETKEGAAsPAPETPQ | S5; | 2159.0 | Yes |
| **CCDC9** | NP_056418.1 | 26093 | coiled-coil domain-containing protein 9 | **S80** | | 1.64 | #NQ | sPGTPRPPGASK | 1 | 1 | 1 | 0 | S1(Phospho) | S(1): 100.0; T(4): 0.0; S(11): 0.0 | NLGPSRRsPGTPRPP | S1; | 1231.6 | Yes |
| **CCDC94** | NP_060544.2 | 55702 | coiled-coil domain-containing protein 94 | **S211; S213** | | | #NQ | RLLEDsDsEDEAAPSPLQPALRPNPTAILDEAPKPK | 1 | 1 | 1 | 1 | S6(Phospho); S8(Phospho) | S(6): 100.0; S(8): 100.0; S(15): 0.0; T(26): 0.0 | KRRLLEDsDSEDEAA; RLLEDSDsEDEAAPS | S6; S8; | 4040.9 | Yes;Yes |
| **CCM2** | NP_001161407.1 | 83605 | malcavernin isoform 4 | **S293** | 1.78 | 1.85 | 1.70 | GIITDsFGR | 8 | 4 | 1 | 0 | S6(Phospho) | T(4): 0.0; S(6): 100.0 | GRGIITDsFGRHRRA | S6; | 1045.5 | No |
| **CCM2** | NP_001161407.1 | 83605 | malcavernin isoform 4 | **S306** | | 1.83 | 1.70 | ALSTTSsSTTNGNR | 1 | 4 | 1 | 0 | S6(Phospho) | S(3): 0.0; T(4): 0.0; T(5): 0.0; S(6): 0.9; S(7): 98.1; S(8): 0.9; T(9): 0.0; T(10): 0.0 | RALSTTSsSTTNGNR | S7; | 1476.6 | No |
| **CCND2** | NP_001750.1 | 894 | G1/S-specific cyclin-D2 | **S271** | | | #N/A | DGSKsEDELDQASTPTDVR | 1 | 1 | 1 | 1 | K4(Label:13C(6)15N(2)); S5(Phospho); R19(Label:13C(6)) | S(3): 1.5; S(5): 98.5; S(13): 0.0; T(14): 0.0; T(16): 0.0 | DQRDGSKsEDELDQA | S5; | 2143.9 | Yes |
| **CCNK** | NP_001092872.1 | 8812 | cyclin-K | **S340** | 0.82 | 0.97 | 2.04 | AVVVsPKEENK | 13 | 1 | 1 | 1 | S5(Phospho) | S(5): 100.0 | VKRAVVVsPKEENKA | S5; | 1279.6 | Yes |
| **CCNK** | NP_001092872.1 | 8812 | cyclin-K | **S340** | 1.04 | 0.92 | 2.04 | AVVVsPKEENK | 13 | 1 | 1 | 1 | S5(Phospho); K7(Label:13C(6)15N(2)); K11(Label:13C(6)15N(2)) | S(5): 100.0 | VKRAVVVsPKEENKA | S5; | 1295.7 | Yes |
| **CCNK** | NP_001092872.1 | 8812 | cyclin-K | **S340** | 0.94 | | 2.04 | AVVVsPK | 3 | 1 | 1 | 0 | S5(Phospho); K7(Label:13C(6)15N(2)) | S(5): 100.0 | VKRAVVVsPKEENKA | S5; | 787.4 | Yes |
| **CCNK** | NP_001092872.1 | 8812 | cyclin-K | **S340** | 0.96 | 0.93 | 2.04 | AVVVsPK | 2 | 1 | 1 | 0 | S5(Phospho) | S(5): 100.0 | VKRAVVVsPKEENKA | S5; | 779.4 | Yes |
| **CCNL1** | NP_064703.1 | 57018 | cyclin-L1 | **S352** | 1.45 | 1.54 | #N/A | AEEKsPISINVK | 11 | 1 | 1 | 1 | K4(Label:13C(6)15N(2)); S5(Phospho); K12(Label:13C(6)15N(2)) | S(5): 100.0; S(8): 0.0 | EVKAEEKsPISINVK | S5; | 1410.7 | Yes |
| **CCNL1** | NP_064703.1 | 57018 | cyclin-L1 | **S342** | 1.75 | 1.75 | #N/A | GLNPDGTPALSTLGGFSPASKPSsPR | 4 | 1 | 1 | 0 | S24(Phospho) | T(7): 0.0; S(11): 0.0; T(12): 0.0; S(17): 0.0; S(20): 0.0; S(23): 1.3; S(24): 98.7 | SPASKPSsPREVKAE | S24; | 2591.3 | Yes |
| **CCNL1** | NP_064703.1 | 57018 | cyclin-L1 | **S352** | | 1.51 | #N/A | AEEKsPISINVK | 10 | 1 | 1 | 1 | S5(Phospho) | S(5): 100.0; S(8): 0.0 | EVKAEEKsPISINVK | S5; | 1394.7 | Yes |

| GeneSymbol | NP_Accession | Gene ID | ProteinDescription | PhosphoSite (Protein) | SCC-R / SCC-S R1 | SCC-R / SCC-S R2 | SCC-R / SCC-S Total Proteome | Sequence | # PSMs | # Proteins | # Protein Groups | # Missed Cleavages | Modifications | phosphoRS Site Probabilities | Phosphowindow | PhosphoSite (Peptide) | MH+ [Da] | PhosphositePlusEvidence |
|---|---|---|---|---|---|---|---|---|---|---|---|---|---|---|---|---|---|---|
| **CCNL1** | NP_064703.1 | 57018 | cyclin-L1 | **S445** | 1.41 | | #N/A | HHNHGsPHLK | 1 | 1 | 1 | 0 | S6(Phospho); K10(Label:13C(6)15N(2)) | S(6): 100.0 | RRHHNHGsPHLKAKH | S6; | 1251.6 | Yes |
| **CCNL1** | NP_064703.1 | 57018 | cyclin-L1 | **S445** | 1.48 | 1.28 | #N/A | HHNHGsPHLK | 3 | 1 | 1 | 0 | S6(Phospho) | S(6): 100.0 | RRHHNHGsPHLKAKH | S6; | 1243.5 | Yes |
| **CCNL1** | NP_064703.1 | 57018 | cyclin-L1 | **T67** | | **2.06** | #N/A | LSPtPSMQDGLDLPSETDLR | 4 | 1 | 1 | 0 | T4(Phospho) | S(2): 1.3; T(4): 97.4; S(6): 1.3; S(15): 0.0; T(17): 0.0 | PEERLSPtPSMQDGL | T4; | 2252.0 | Yes |
| **CCNL1** | NP_064703.1 | 57018 | cyclin-L1 | **S352** | | 1.3 | #N/A | EVKAEEKsPISINVK | 3 | 1 | 1 | 2 | S8(Phospho) | S(8): 100.0; S(11): 0.0 | EVKAEEKsPISINVK | S8; | 1750.9 | Yes |
| **CCNL1** | NP_064703.1 | 57018 | cyclin-L1 | **S352** | | 1.35 | #N/A | EVKAEEKsPISINVK | 2 | 1 | 1 | 2 | K3(Label:13C(6)15N(2)); K7(Label:13C(6)15N(2)); S8(Phospho); K15(Label:13C(6)15N(2)) | S(8): 100.0; S(11): 0.0 | EVKAEEKsPISINVK | S8; | 1774.9 | Yes |
| **CCNL2** | NP_112199.2 | 81669 | cyclin-L2 isoform A | **S330** | 0.52 | 0.59 | #N/A | GLLPGGTQVLDGTSGFsPAPK | 9 | 1 | 1 | 0 | S17(Phospho); K21(Label:13C(6)15N(2)) | T(7): 0.0; T(13): 0.0; S(14): 0.0; S(17): 100.0 | LDGTSGFsPAPKLVE | S17; | 2087.0 | Yes |
| **CCNL2** | NP_112199.2 | 81669 | cyclin-L2 isoform A | **S369** | **0.51** | **0.33** | #N/A | ADsPVNGLPK | 3 | 1 | 1 | 0 | S3(Phospho); K10(Label:13C(6)15N(2)) | S(3): 100.0 | AKKAKADsPVNGLPK | S3; | 1085.5 | Yes |
| **CCNL2** | NP_112199.2 | 81669 | cyclin-L2 isoform A | **S330** | 0.57 | | #N/A | GLLPGGTQVLDGTSGFsPAPK | 7 | 1 | 1 | 0 | S17(Phospho) | T(7): 0.0; T(13): 0.0; S(14): 0.0; S(17): 100.0 | LDGTSGFsPAPKLVE | S17; | 2079.0 | Yes |
| **CCNL2** | NP_112199.2 | 81669 | cyclin-L2 isoform A | **S338** | | **0.46** | #N/A | LVEsPKEGK | 2 | 1 | 1 | 1 | S4(Phospho) | S(4): 100.0 | PAPKLVEsPKEGKGS | S4; | 1066.5 | Yes |
| **CCNL2** | NP_112199.2 | 81669 | cyclin-L2 isoform A | **S338** | | **0.44** | #N/A | LVEsPK | 1 | 1 | 1 | 0 | S4(Phospho) | S(4): 100.0 | PAPKLVEsPKEGKGS | S4; | 752.4 | Yes |
| **CCNL2** | NP_112199.2 | 81669 | cyclin-L2 isoform A | **S338** | | | #N/A | LVEsPK | 1 | 1 | 1 | 0 | S4(Phospho); K6(Label:13C(6)15N(2)) | S(4): 100.0 | PAPKLVEsPKEGKGS | S4; | 760.4 | Yes |
| **CCNL2** | NP_112199.2 | 81669 | cyclin-L2 isoform A | **S369** | | **0.37** | #N/A | AKADsPVNGLPK | 1 | 1 | 1 | 1 | K2(Label:13C(6)15N(2)); S5(Phospho); K12(Label:13C(6)15N(2)) | S(5): 100.0 | AKKAKADsPVNGLPK | S5; | 1292.7 | Yes |
| **CCNY** | NP_001269782.1 | 219771 | cyclin-Y isoform 2 | **S272** | 1.16 | 1 | #N/A | SAsADNLTLPR | 7 | 4 | 1 | 0 | S3(Phospho) | S(1): 0.0; S(3): 100.0; T(8): 0.0 | SARKRSAsADNLTLP | S3; | 1224.6 | No |
| **CCNY** | NP_001269782.1 | 219771 | cyclin-Y isoform 2 | **S29** | 1.92 | | #N/A | sLFINHHPPGQIAR | 1 | 3 | 1 | 0 | S1(Phospho) | S(1): 100.0 | DVREKRKsLFINHHP | S1; | 1666.8 | No |
| **CCNY** | NP_001269782.1 | 219771 | cyclin-Y isoform 2 | **S272** | 0.98 | | #N/A | SAsADNLTLPR | 3 | 4 | 1 | 0 | S3(Phospho); R11(Label:13C(6)) | S(1): 0.0; S(3): 100.0; T(8): 0.0 | SARKRSAsADNLTLP | S3; | 1230.6 | No |
| **CCNY** | NP_001269782.1 | 219771 | cyclin-Y isoform 2 | **S29** | | 1.69 | #N/A | sLFINHHPPGQIAR | 1 | 3 | 1 | 0 | S1(Phospho); R14(Label:13C(6)) | S(1): 100.0 | DVREKRKsLFINHHP | S1; | 1672.8 | No |
| **CCNYL1** | NP_689736.1 | 151195 | cyclin-Y-like protein 1 isoform 2 | **S35** | **0.34** | | #N/A | sNHLNHVSPGQLTK | 1 | 1 | 1 | 0 | S1(Phospho); K14(Label:13C(6)15N(2)) | S(1): 100.0; S(8): 0.0; T(13): 0.0 | DVREKRKsNHLNHVS | S1; | 1619.8 | No |
| **CCP110** | NP_055526.3 | 9738 | centriolar coiled-coil protein of 110 kDa isoform 2 | **S372** | 1.7 | 1.73 | #N/A | LPSPEPSMsPK | 7 | 2 | 1 | 0 | S9(Phospho) | S(3): 0.0; S(7): 0.0; S(9): 100.0 | PSPEPSMsPKMHRRR | S9; | 1249.6 | Yes |
| **CCP110** | NP_055526.3 | 9738 | centriolar coiled-coil protein of 110 kDa isoform 2 | **S372** | | | #N/A | LPSPEPSMsPK | 6 | 2 | 1 | 0 | S9(Phospho); K11(Label:13C(6)15N(2)) | S(3): 0.0; S(7): 0.0; S(9): 100.0 | PSPEPSMsPKMHRRR | S9; | 1257.6 | Yes |
| **CD2AP** | NP_036252.1 | 23607 | CD2-associated protein | **S458** | **0.41** | **0.43** | 1.09 | sVDFDSLTVR | 10 | 1 | 1 | 0 | S1(Phospho) | S(1): 100.0; S(6): 0.0; T(8): 0.0 | QLPLRPKsVDFDSLT | S1; | 1218.5 | Yes |
| **CD2AP** | NP_036252.1 | 23607 | CD2-associated protein | **S224** | 1.27 | 1.01 | 1.09 | GIGFGDIFKEGsVK | 2 | 1 | 1 | 1 | S12(Phospho) | S(12): 100.0 | GDIFKEGsVKLRTRT | S12; | 1533.7 | Yes |
| **CD2AP** | NP_036252.1 | 23607 | CD2-associated protein | **S224** | | | 1.09 | GIGFGDIFKEGsVK | 4 | 1 | 1 | 1 | K9(Label:13C(6)15N(2)); S12(Phospho); K14(Label:13C(6)15N(2)) | S(12): 100.0 | GDIFKEGsVKLRTRT | S12; | 1549.8 | Yes |
| **CD2AP** | NP_036252.1 | 23607 | CD2-associated protein | **S510** | 0.97 | 1.18 | 1.09 | FNGGHsPTHSPEK | 8 | 1 | 1 | 0 | S6(Phospho); K13(Label:13C(6)15N(2)) | S(6): 100.0; T(8): 0.0; S(10): 0.0 | GRFNGGHsPTHSPEK | S6; | 1482.6 | Yes |
| **CD2AP** | NP_036252.1 | 23607 | CD2-associated protein | **S458** | **0.43** | **0.45** | 1.09 | sVDFDSLTVR | 5 | 1 | 1 | 0 | S1(Phospho); R10(Label:13C(6)) | S(1): 100.0; S(6): 0.0; T(8): 0.0 | QLPLRPKsVDFDSLT | S1; | 1224.6 | Yes |
| **CD2AP** | NP_036252.1 | 23607 | CD2-associated protein | **S510; S514** | | 0.83 | 1.09 | FNGGHsPTHsPEK | 1 | 1 | 1 | 0 | S6(Phospho); S10(Phospho); K13(Label:13C(6)15N(2)) | S(6): 100.0; T(8): 1.5; S(10): 98.5 | GRFNGGHsPTHSPEK; GGHSPTHsPEKILKL | S6; S10; | 1562.6 | Yes;Yes |
| **CD300LG** | NP_660316.2 | 146894 | CMRF35-like molecule 9 isoform 1 precursor | **S313; S327; S331** | **6.22** | | #N/A | EAPSQAPEGDVIsMPPLHTSEEELGFsKFVsA | 1 | 1 | 1 | 1 | S20(Phospho); S27(Phospho); S31(Phospho) | S(4): 0.0; S(13): 93.7; T(19): 0.4; S(20): 5.9; S(27): 100.0; S(31): 100.0 | APEGDVIsMPPLHTS;SEEELGFsKFVSA_;LGFSKFVsA_ | S13; S27; S31; | 3626.5 | No;No;No |
| **CD3EAP** | NP_036231.1 | 10849 | DNA-directed RNA polymerase I subunit RPA34 | **S490** | 0.84 | 0.97 | 0.82 | MPETVPQEEMPGPPLNSEsGEEAPTGR | 6 | 1 | 1 | 0 | S19(Phospho) | T(4): 0.0; S(17): 0.0; S(19): 100.0; T(25): 0.0 | GPPLNSEsGEEAPTG | S19; | 2946.3 | Yes |
| **CD3EAP** | NP_036231.1 | 10849 | DNA-directed RNA polymerase I subunit RPA34 | **S128** | 0.83 | **4.17** | 0.82 | ILEGPQQSLSGsPLQPIPASPPPQIPPGLRPR | 4 | 1 | 1 | 0 | S12(Phospho) | S(8): 0.0; S(10): 2.7; S(12): 97.3; S(20): 0.0 | PQQSLSGsPLQPIPA | S12; | 3404.8 | Yes |
| **CD3EAP** | NP_036231.1 | 10849 | DNA-directed RNA polymerase I subunit RPA34 | **S490** | 0.88 | | 0.82 | MPETVPQEEMPGPPLNSEsGEEAPTGR | 3 | 1 | 1 | 0 | S19(Phospho); R27(Label:13C(6)) | T(4): 0.0; S(17): 0.0; S(19): 100.0; T(25): 0.0 | GPPLNSEsGEEAPTG | S19; | 2952.3 | Yes |
| **CD3EAP** | NP_036231.1 | 10849 | DNA-directed RNA polymerase I subunit RPA34 | **S128** | 0.88 | | 0.82 | ILEGPQQSLSGsPLQPIPASPPPQIPPGLRPR | 1 | 1 | 1 | 0 | S12(Phospho); R30(Label:13C(6)); R32(Label:13C(6)) | S(8): 3.2; S(10): 3.2; S(12): 93.6; S(20): 0.0 | PQQSLSGsPLQPIPA | S12; | 3416.8 | Yes |
| **CD3EAP** | NP_036231.1 | 10849 | DNA-directed RNA polymerase I subunit RPA34 | **S285** | | 0.7 | 0.82 | QEQINTEPLEDTVLsPTKKR | 1 | 1 | 1 | 2 | S15(Phospho) | T(6): 0.0; T(12): 0.0; S(15): 98.4; T(17): 1.6 | PLEDTVLsPTKKRKR | S15; | 2406.2 | Yes |
| **CD44** | NP_001189485.1 | 960 | CD44 antigen isoform 7 precursor | **S304** | 1.6 | 1.64 | **2.71** | sQEMVHLVNKESSETPDQFMTADETR | 4 | 6 | 1 | 1 | S1(Phospho) | S(1): 100.0; S(12): 0.0; S(13): 0.0; T(15): 0.0; T(21): 0.0; T(25): 0.0 | LNGEASKsQEMVHLV | S1; | 3089.3 | No |
| **CD44** | NP_001189485.1 | 960 | CD44 antigen isoform 7 precursor | **S304** | **1.98** | **2.07** | **2.71** | sQEMVHLVNK | 8 | 6 | 1 | 0 | S1(Phospho) | S(1): 100.0 | LNGEASKsQEMVHLV | S1; | 1264.6 | No |
| **CD44** | NP_001189485.1 | 960 | CD44 antigen isoform 7 precursor | **S295** | | **3.6** | **2.71** | LVINSGNGAVEDRKPsGLNGEASK | 2 | 6 | 1 | 1 | S16(Phospho) | S(5): 0.0; S(16): 100.0; S(23): 0.0 | AVEDRKPsGLNGEAS | S16; | 2492.2 | No |
| **CD44** | NP_001189485.1 | 960 | CD44 antigen isoform 7 precursor | **S304** | | | **2.71** | sQEMVHLVNK | 4 | 6 | 1 | 0 | S1(Phospho); K10(Label:13C(6)15N(2)) | S(1): 100.0 | LNGEASKsQEMVHLV | S1; | 1272.6 | No |
| **CD44** | NP_001189485.1 | 960 | CD44 antigen isoform 7 precursor | **S304** | 1.59 | | **2.71** | LVINSGNGAVEDRKPSGLNGEASKsQEMVHLVNK | 2 | 6 | 1 | 2 | S25(Phospho) | S(5): 0.0; S(16): 0.0; S(23): 0.0; S(25): 100.0 | LNGEASKsQEMVHLV | S25; | 3657.8 | No |
| **CD44** | NP_001189485.1 | 960 | CD44 antigen isoform 7 precursor | **S284** | | | **2.71** | LVINsGNGAVEDR | 1 | 6 | 1 | 0 | S5(Phospho) | S(5): 100.0 | KKKLVINsGNGAVED | S5; | 1423.7 | No |
| **CD44** | NP_001189485.1 | 960 | CD44 antigen isoform 7 precursor | **S304** | 1.67 | | **2.71** | sQEMVHLVNKESSETPDQFMTADETR | 1 | 6 | 1 | 1 | S1(Phospho); K10(Label:13C(6)15N(2)); R26(Label:13C(6)) | S(1): 100.0; S(12): 0.0; S(13): 0.0; T(15): 0.0; T(21): 0.0; T(25): 0.0 | LNGEASKsQEMVHLV | S1; | 3103.4 | No |
| **CD44** | NP_001189485.1 | 960 | CD44 antigen isoform 7 precursor | **S304** | | 1.51 | **2.71** | KPSGLNGEASKsQEMVHLVNK | 8 | 6 | 1 | 1 | K1(Label:13C(6)15N(2)); K11(Label:13C(6)15N(2)); S12(Phospho); K21(Label:13C(6)15N(2)) | S(3): 0.0; S(10): 0.0; S(12): 100.0 | LNGEASKsQEMVHLV | S12; | 2357.2 | No |
| **CD44** | NP_001189485.1 | 960 | CD44 antigen isoform 7 precursor | **S304** | | 1.59 | **2.71** | KPSGLNGEASKsQEMVHLVNK | 8 | 6 | 1 | 1 | S12(Phospho) | S(3): 0.0; S(10): 0.0; S(12): 100.0 | LNGEASKsQEMVHLV | S12; | 2333.1 | No |
| **CD44** | NP_001189485.1 | 960 | CD44 antigen isoform 7 precursor | **S304** | | 1.41 | **2.71** | KPSGLNGEASKsQEMVHLVNKESSETPDQFMTADETR | 3 | 6 | 1 | 2 | S12(Phospho) | S(3): 0.0; S(10): 0.1; S(12): 99.8; S(23): 0.0; S(24): 0.0; T(26): 0.0; T(32): 0.0; T(36): 0.0 | LNGEASKsQEMVHLV | S12; | 4157.9 | No |
| **CD44** | NP_001189485.1 | 960 | CD44 antigen isoform 7 precursor | **S304** | | | **2.71** | KPSGLNGEASKsQEMVHLVNK | 1 | 6 | 1 | 1 | S12(Phospho); M15(Oxidation) | S(3): 0.0; S(10): 0.0; S(12): 100.0 | LNGEASKsQEMVHLV | S12; | 2349.1 | No |
| **CD97** | NP_001775.2 | 976 | CD97 antigen isoform 2 preproprotein | **S738** | **3.48** | | **9.77** | ALRAsESGI | 5 | 3 | 1 | 1 | S5(Phospho) | S(5): 100.0; S(7): 0.0 | QTRALRAsESGI_ | S5; | 983.5 | No |
| **CDAN1** | NP_612486.2 | 146059 | codanin-1 | **T71** | | 0.74 | #N/A | VLPQGPPtPAK | 6 | 1 | 1 | 0 | T8(Phospho); K11(Label:13C(6)15N(2)) | T(8): 100.0 | VLPQGPPtPAKTPGA | T8; | 1192.6 | Yes |
| **CDAN1** | NP_612486.2 | 146059 | codanin-1 | **T71** | | 0.74 | #N/A | VLPQGPPtPAK | 2 | 1 | 1 | 0 | T8(Phospho) | T(8): 100.0 | VLPQGPPtPAKTPGA | T8; | 1184.6 | Yes |
| **CDC16** | NP_003894.3 | 8881 | cell division cycle protein 16 homolog | **S560** | | | 0.99 | NIIsPPWDFR | 2 | 1 | 1 | 0 | S4(Phospho) | S(4): 100.0 | KTLKNIIsPPWDFRE | S4; | 1324.6 | Yes |
| **CDC20** | NP_001246.2 | 991 | cell division cycle protein 20 homolog | **S41** | 1.86 | | #N/A | AKEAAGPAPsPMR | 2 | 1 | 1 | 1 | S10(Phospho) | S(10): 100.0 | EAAGPAPsPMRAANR | S10; | 1362.6 | Yes |
| **CDC20** | NP_001246.2 | 991 | cell division cycle protein 20 homolog | **S41** | **2.23** | **2.23** | #N/A | EAAGPAPsPMR | 8 | 1 | 1 | 0 | S8(Phospho) | S(8): 100.0 | EAAGPAPsPMRAANR | S8; | 1163.5 | Yes |
| **CDC20** | NP_001246.2 | 991 | cell division cycle protein 20 homolog | **S41** | | | #N/A | EAAGPAPsPMR | 3 | 1 | 1 | 0 | S8(Phospho); R11(Label:13C(6)) | S(8): 100.0 | EAAGPAPsPMRAANR | S8; | 1169.5 | Yes |
| **CDC20** | NP_001246.2 | 991 | cell division cycle protein 20 homolog | **T70** | | **2.36** | #N/A | VQTtPSKPGGDR | 2 | 1 | 1 | 0 | T4(Phospho) | T(3): 0.0; T(4): 100.0; S(6): 0.0 | SSSKVQTtPSKPGGD | T4; | 1322.6 | Yes |
| **CDC20** | NP_001246.2 | 991 | cell division cycle protein 20 homolog | **S41** | | 1.89 | #N/A | EAAGPAPsPMR | 2 | 1 | 1 | 0 | S8(Phospho); M10(Oxidation) | S(8): 100.0 | EAAGPAPsPMRAANR | S8; | 1179.5 | Yes |
| **CDC20** | NP_001246.2 | 991 | cell division cycle protein 20 homolog | **S41** | | | #N/A | EAAGPAPsPMR | 1 | 1 | 1 | 0 | S8(Phospho); M10(Oxidation); R11(Label:13C(6)) | S(8): 100.0 | EAAGPAPsPMRAANR | S8; | 1185.5 | Yes |
| **CDC23** | NP_004652.2 | 8697 | cell division cycle protein 23 homolog | **T596** | | | 1.04 | VSPLNLSSVtP | 5 | 1 | 1 | 0 | T10(Phospho) | S(2): 0.0; S(7): 0.0; S(8): 0.0; T(10): 100.0 | PLNLSSVtP_ | T10; | 1193.6 | Yes |
| **CDC23** | NP_004652.2 | 8697 | cell division cycle protein 23 homolog | **S588** | | | 1.04 | RVsPLNLSSVTP | 2 | 1 | 1 | 1 | S3(Phospho) | S(3): 100.0; S(8): 0.0; S(9): 0.0; T(11): 0.0 | NTPTRRVsPLNLSSV | S3; | 1349.7 | Yes |
| **CDC23** | NP_004652.2 | 8697 | cell division cycle protein 23 homolog | **S588** | | 1.18 | 1.04 | RVsPLNLSSVTP | 5 | 1 | 1 | 1 | R1(Label:13C(6)); S3(Phospho) | S(3): 100.0; S(8): 0.0; S(9): 0.0; T(11): 0.0 | NTPTRRVsPLNLSSV | S3; | 1355.7 | Yes |
| **CDC23** | NP_004652.2 | 8697 | cell division cycle protein 23 homolog | **T562** | | **3.12** | 1.04 | NQGEtPTTEVPAPFFLPASLSANNTPTR | 2 | 1 | 1 | 0 | T5(Phospho) | T(5): 95.2; T(7): 2.4; T(8): 2.4; S(19): 0.0; S(21): 0.0; T(25): 0.0; T(27): 0.0 | QLRNQGEtPTTEVPA | T5; | 3037.4 | Yes |
| **CDC23** | NP_004652.2 | 8697 | cell division cycle protein 23 homolog | **S588; T596** | | 1.02 | 1.04 | RVsPLNLSSVtP | 2 | 1 | 1 | 1 | S3(Phospho); T11(Phospho) | S(3): 100.0; S(8): 1.2; S(9): 1.2; T(11): 97.5 | NTPTRRVsPLNLSSV;PLNLSSVtP_ | S3; T11; | 1429.6 | Yes;Yes |
| **CDC23** | NP_004652.2 | 8697 | cell division cycle protein 23 homolog | **S588; S593** | | | 1.04 | RVsPLNLsSVTP | 1 | 1 | 1 | 1 | R1(Label:13C(6)); S3(Phospho); S8(Phospho) | S(3): 100.0; S(8): 100.0; S(9): 0.0; T(11): 0.0 | NTPTRRVsPLNLSSV; RVSPLNLsSVTP_ | S3; S8; | 1435.7 | Yes;Yes |
| **CDC25B** | NP_001274449.1 | 994 | M-phase inducer phosphatase 2 isoform 7 | **S39** | | **3.26** | #N/A | FQsMPVR | 1 | 6 | 1 | 0 | S3(Phospho) | S(3): 100.0 | FAIRRFQsMPVRLLG | S3; | 944.4 | No |

| GeneSymbol | NP_Accession | Gene ID | ProteinDescription | PhosphoSite (Protein) | SCC-R / SCC-S R1 | SCC-R / SCC-S R2 | SCC-R / SCC-S Total Proteome | Sequence | # PSMs | # Proteins | # Protein Groups | # Missed Cleavages | Modifications | phosphoRS Site Probabilities | Phosphowindow | PhosphoSite (Peptide) | MH+ [Da] | PhosphositePlusEvidence |
|---|---|---|---|---|---|---|---|---|---|---|---|---|---|---|---|---|---|---|
| CDC42BPG | NP_059995.2 | 55561 | serine/threonine-protein kinase MRCK gamma | S1492 | | | #NQ | RPAsMGSEGLGGDADPMKR | 2 | 1 | 1 | 1 | R1(Label:13C(6)); S4(Phospho); K18(Label:13C(6)15N(2)); R19(Label:13C(6)) | S(4): 100.0; S(7): 0.0 | EALRRPAsMGSEGLG | S4; | 2031.9 | Yes |
| CDC42EP1 | NP_689449.1 | 11135 | cdc42 effector protein 1 | S121 | 0.96 | 1.21 | 0.80 | NAIsLPQLNQAAYDSLVVGK | 10 | 1 | 1 | 0 | S4(Phospho); K20(Label:13C(6)15N(2)) | S(4): 100.0; Y(13): 0.0; S(15): 0.0 | PIIKNAIsLPQLNQA | S4; | 2189.1 | Yes |
| CDC42EP1 | NP_689449.1 | 11135 | cdc42 effector protein 1 | S121 | 1.01 | 1.1 | 0.80 | NAIsLPQLNQAAYDSLVVGK | 20 | 1 | 1 | 0 | S4(Phospho) | S(4): 100.0; Y(13): 0.0; S(15): 0.0 | PIIKNAIsLPQLNQA | S4; | 2181.1 | Yes |
| CDC42EP1 | NP_689449.1 | 11135 | cdc42 effector protein 1 | S101 | 1.55 | | 0.80 | MAsPPAPSPAPPAISPIIK | 3 | 1 | 1 | 0 | S3(Phospho) | S(3): 100.0; S(8): 0.0; S(15): 0.0 | APPRRMAsPPAPSPA | S3; | 1922.0 | Yes |
| CDC42EP1 | NP_689449.1 | 11135 | cdc42 effector protein 1 | S77 | | 0.95 | 0.80 | GGDVFGDTSFLSNHGGSSGSTHRsPR | 1 | 1 | 1 | 1 | R23(Label:13C(6)); S24(Phospho); R26(Label:13C(6)) | T(8): 0.0; S(9): 0.0; S(12): 0.0; S(17): 2.3; S(18): 2.3; S(20): 2.3; T(21): 2.3; S(24): 91.0 | SSGSTHRsPRSFLAK | S24; | 2711.2 | Yes |
| CDC42EP1 | NP_689449.1 | 11135 | cdc42 effector protein 1 | S192 | | | 0.80 | RSDsLLSFR | 1 | 1 | 1 | 1 | R1(Label:13C(6)); S4(Phospho); R9(Label:13C(6)) | S(2): 0.0; S(4): 100.0; S(7): 0.0 | PGLRRSDsLLSFRLD | S4; | 1172.6 | Yes |
| CDC42EP3 | NP_001257365.1 | 10602 | cdc42 effector protein 3 | S89 | | 4.49 | 3.62 | ANsTSDSVFTETPSPVLK | 4 | 1 | 1 | 0 | S3(Phospho) | S(3): 98.9; T(4): 1.1; S(5): 0.0; S(7): 0.0; T(10): 0.0; T(12): 0.0; S(14): 0.0 | NEFFRANsTSDSVFT | S3; | 1959.9 | Yes |
| CDC42EP3 | NP_001257365.1 | 10602 | cdc42 effector protein 3 | S108 | | | 3.62 | NAIsLPTIGGSQALMLPLLSPVTFNSK | 4 | 1 | 1 | 0 | S4(Phospho) | S(4): 98.6; T(7): 1.4; S(11): 0.0; S(20): 0.0; T(23): 0.0; S(26): 0.0 | PVLKNAIsLPTIGGS | S4; | 2849.5 | Yes |
| CDC42EP3 | NP_001257365.1 | 10602 | cdc42 effector protein 3 | S108 | 7.25 | | 3.62 | NAIsLPTIGGSQALMLPLLSPVTFNSK | 2 | 1 | 1 | 0 | S4(Phospho); K27(Label:13C(6)15N(2)) | S(4): 100.0; T(7): 0.0; S(11): 0.0; S(20): 0.0; T(23): 0.0; S(26): 0.0 | PVLKNAIsLPTIGGS | S4; | 2857.5 | Yes |
| CDC42EP4 | NP_036253.2 | 23580 | cdc42 effector protein 4 | S174 | | | #N/A | RNGAAGPHsPDPLLDEQAFGDLTDLPVVPK | 4 | 1 | 1 | 1 | S9(Phospho) | S(9): 100.0; T(23): 0.0 | NGAAGPHsPDPLLDE | S9; | 3206.6 | Yes |
| CDC42EP4 | NP_036253.2 | 23580 | cdc42 effector protein 4 | S18 | 5.84 | 3.33 | #N/A | sRADLTAEMISAPLGDFR | 12 | 1 | 1 | 1 | S1(Phospho) | S(1): 100.0; T(6): 0.0; S(11): 0.0 | SVHSKRRsRADLTAE | S1; | 2029.9 | Yes |
| CDC42EP4 | NP_036253.2 | 23580 | cdc42 effector protein 4 | S11 | 13.47 | 3.15 | #N/A | QLVSSsVHSK | 3 | 1 | 1 | 0 | S6(Phospho) | S(4): 0.0; S(5): 0.0; S(6): 100.0; S(9): 0.0 | LKQLVSSsVHSKRRS | S6; | 1151.5 | Yes |
| CDC42EP4 | NP_036253.2 | 23580 | cdc42 effector protein 4 | S138; S142 | | 1.65 | #N/A | sLSSsPVKKANDGEGGDEEAGTEEAVPR | 2 | 1 | 1 | 2 | S3(Phospho); S4(Phospho) | S(1): 96.7; S(3): 3.3; S(4): 3.3; S(5): 96.7; T(22): 0.0 | GTSKLPKsLSSSPVK;LPKSLSSsPVKKAND | S1; S5; | 2976.3 | Yes;Yes |
| CDC42EP4 | NP_036253.2 | 23580 | cdc42 effector protein 4 | S64 | | 1.46 | #N/A | AGEPDGEsLDEQPSSSSSKR | 2 | 1 | 1 | 1 | S8(Phospho) | S(8): 100.0; S(14): 0.0; S(15): 0.0; S(16): 0.0; S(17): 0.0; S(18): 0.0 | AGEPDGEsLDEQPSS | S8; | 2142.9 | Yes |
| CDC42EP4 | NP_036253.2 | 23580 | cdc42 effector protein 4 | S118 | | 1.63 | #N/A | NAMsLPQLNEKEAAEK | 1 | 1 | 1 | 1 | S4(Phospho); K11(Label:13C(6)15N(2)); K16(Label:13C(6)15N(2)) | S(4): 100.0 | LFVKNAMsLPQLNEK | S4; | 1868.9 | Yes |
| CDC42EP4 | NP_036253.2 | 23580 | cdc42 effector protein 4 | S105 | | 1.75 | #N/A | DMLGsLR | 1 | 1 | 1 | 0 | S5(Phospho) | S(5): 100.0 | EQRDMLGsLRDSALF | S5; | 871.4 | Yes |
| CDC42EP4 | NP_036253.2 | 23580 | cdc42 effector protein 4 | S292; S295 | | | #N/A | AGPDLPSLPSHALEDEGWAAAAPsPGsAR | 1 | 1 | 1 | 0 | S24(Phospho); S27(Phospho) | S(7): 0.0; S(10): 0.0; S(24): 100.0; S(27): 100.0 | GWAAAAPsPGSARSM;AAAPSPGsARSMGSH | S24; S27; | 2987.3 | Yes;Yes |
| CDC42EP4 | NP_036253.2 | 23580 | cdc42 effector protein 4 | S64 | | 1.51 | #N/A | AGEPDGEsLDEQPSSSSSKR | 1 | 1 | 1 | 1 | S8(Phospho); K19(Label:13C(6)15N(2)); R20(Label:13C(6)) | S(8): 100.0; S(14): 0.0; S(15): 0.0; S(16): 0.0; S(17): 0.0; S(18): 0.0 | AGEPDGEsLDEQPSS | S8; | 2156.9 | Yes |
| CDC42EP4 | NP_036253.2 | 23580 | cdc42 effector protein 4 | S18 | | 3.44 | #N/A | sRADLTAEMISAPLGDFR | 1 | 1 | 1 | 1 | S1(Phospho); R2(Label:13C(6)); R18(Label:13C(6)) | S(1): 100.0; T(6): 0.0; S(11): 0.0 | SVHSKRRsRADLTAE | S1; | 2042.0 | Yes |
| CDC6 | NP_001245.1 | 990 | cell division control protein 6 homolog | S45 | 0.33 | | #N/A | LEPTNVQTVTCsPR | 1 | 1 | 1 | 0 | C11(Carbamidomethyl); S12(Phospho); R14(Label:13C(6)) | T(4): 0.0; T(8): 0.0; T(10): 1.2; S(12): 98.8 | NVQTVTCsPRVKALP | S12; | 1687.8 | Yes |
| CDC6 | NP_001245.1 | 990 | cell division control protein 6 homolog | S54 | | | #N/A | ALPLsPR | 1 | 1 | 1 | 0 | S5(Phospho) | S(5): 100.0 | RVKALPLsPRKRLGD | S5; | 833.4 | Yes |
| CDC6 | NP_001245.1 | 990 | cell division control protein 6 homolog | S45 | | 0.38 | #N/A | NSSDAKLEPTNVQTVTCsPR | 1 | 1 | 1 | 1 | K6(Label:13C(6)15N(2)); C17(Carbamidomethyl); S18(Phospho); R20(Label:13C(6)) | S(2): 0.0; S(3): 0.0; T(10): 0.0; T(14): 0.0; T(16): 0.0; S(18): 100.0 | NVQTVTCsPRVKALP | S18; | 2298.1 | Yes |
| CDCA3 | NP_112589.1 | 83461 | cell division cycle-associated protein 3 | S29 | | 1.91 | 1.29 | sPSAGILR | 1 | 1 | 1 | 0 | S1(Phospho) | S(1): 100.0; S(3): 0.0 | ARVADPRsPSAGILR | S1; | 880.4 | Yes |
| CDCA5 | NP_542399.1 | 113130 | sororin | S75 | 1.06 | | #N/A | IVAHAVEVPAVQsPR | 3 | 1 | 1 | 0 | S13(Phospho) | S(13): 100.0 | VEVPAVQsPRRSPRI | S13; | 1652.9 | Yes |
| CDCA5 | NP_542399.1 | 113130 | sororin | S75 | | 1.03 | #N/A | IVAHAVEVPAVQsPR | 4 | 1 | 1 | 0 | S13(Phospho); R15(Label:13C(6)) | S(13): 100.0 | VEVPAVQsPRRSPRI | S13; | 1658.9 | Yes |
| CDCA5 | NP_542399.1 | 113130 | sororin | S75 | | | #N/A | RIVAHAVEVPAVQsPR | 2 | 1 | 1 | 1 | S14(Phospho) | S(14): 100.0 | VEVPAVQsPRRSPRI | S14; | 1809.0 | Yes |
| CDCA8 | NP_060571.1 | 55143 | borealin | S219 | | | #N/A | IYNISGNGsPLADSKEIFLTVPVGGESLR | 1 | 1 | 1 | 1 | S9(Phospho); K15(Label:13C(6)15N(2)); R30(Label:13C(6)) | Y(2): 0.0; S(5): 0.0; S(9): 97.8; S(14): 2.2; T(20): 0.0; S(28): 0.0 | YNISGNGsPLADSKE | S9; | 3184.6 | Yes |
| CDK1 | NP_001777.1 | 983 | cyclin-dependent kinase 1 isoform 1 | T14; Y15 | 4.74 | | 0.87 | IGEGtyGVVYKGR | 2 | 3 | 1 | 1 | T5(Phospho); Y6(Phospho) | T(5): 100.0; Y(6): 100.0; Y(10): 0.0 | IEKIGEGtYGVVYKG;EKIGEGTyGVVYKGR | T5; Y6; | 1558.7 | Yes;Yes |
| CDK1 | NP_001777.1 | 983 | cyclin-dependent kinase 1 isoform 1 | T14; Y15 | | | 0.87 | IEKIGEGtyGVVYK | 2 | 3 | 1 | 1 | T8(Phospho); Y9(Phospho) | T(8): 100.0; Y(9): 100.0; Y(13): 0.0 | IEKIGEGtYGVVYKG;EKIGEGTyGVVYKGR | T8; Y9; | 1715.8 | Yes;Yes |
| CDK1 | NP_001777.1 | 983 | cyclin-dependent kinase 1 isoform 1 | Y15 | | 0.59 | 0.87 | IEKIGEGTyGVVYK | 7 | 3 | 1 | 1 | K3(Label:13C(6)15N(2)); Y9(Phospho); K14(Label:13C(6)15N(2)) | T(8): 1.3; Y(9): 98.7; Y(13): 0.0 | EKIGEGTyGVVYKGR | Y9; | 1651.8 | Yes |
[truncated: 1,759,808 more chars]
